# Bibliometric analysis for the identification of main limitations and future directions of vaccines for the control of ticks and tick-borne pathogens in Uganda

**DOI:** 10.1016/j.crpvbd.2024.100175

**Published:** 2024-04-18

**Authors:** José de la Fuente, Justus Rutaisire

**Affiliations:** a SaBio, Instituto de Investigación en Recursos Cinegéticos (IREC), Consejo Superior de Investigaciones Científicas (CSIC), Universidad de Castilla-La Mancha (UCLM)-Junta de Comunidades de Castilla-La Mancha (JCCM), Ronda de Toledo 12, 13005 Ciudad Real, Spain; b Department of Veterinary Pathobiology, Center for Veterinary Health Sciences, Oklahoma State University, Stillwater, OK 74078, USA; c National Livestock Resources Research Institute (NaLIRRI), National Agricultural Research Organization, Kampala P.O. Box 5704, Uganda

**Keywords:** Africa, Bibliometric, Pathogen, Tick, Tick-borne diseases, Uganda, Vaccine

## Abstract

Ticks and tick-borne diseases (TBD) are a growing threat for human and animal health worldwide with high incidence in African countries such as Uganda where it affects cattle health and production. Considering recent advances in bibliometric analysis, in this review we used a bibliometric descriptive approach for the analysis of publications and patents in the fields of ticks, TBD, and vaccines in Uganda. The results showed that major gaps and limitations are associated with (i) low contributions from Ugandan institutions, (ii) limited international collaborations, (iii) poor impact of translational research, and (iv) little research on tick control vaccines. The results were then used to propose future directions to approach these limitations in Uganda. Although ongoing initiatives and international collaborations are contributing to address major gaps and limitations, future directions should advance in these collaborative projects together with new initiatives addressing (i) basic and translational research on TBD such as CCHF and ASF, (ii) participation of Ugandan institutions in new international consortia in this area, (iii) promoting communication of these initiatives to Ugandan cattle holders and general population to attract support from public and private sectors, (iv) stimulate and support scientific publications and patents with participation of Ugandan scientists, and (v) build and implement production capacity for vaccines in Uganda. These results contribute to guiding Ugandan scientists and national authorities to face challenges posed by ticks and TBD with implications for other African countries.

## Highlights

• Bibliometric analysis identified major gaps and limitations in tick vaccines in Uganda.
• Limitations are associated with research, contributions and international collaborations of Ugandan institutions.
• Future directions include basic and translational research, social communication, publications and vaccine production.
• Study results contribute guiding African scientists and national authorities.

## Graphical abstract

**Figure:**
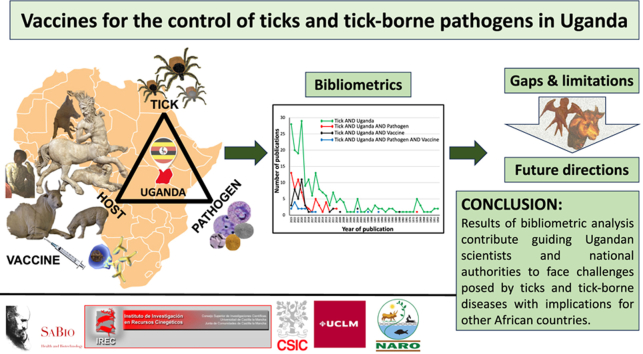

## 1 Introduction

Ticks and tick-borne diseases (TBD) are a growing threat for human and animal health worldwide with high incidence in Uganda as in other African countries (Baneth, 2014; de la Fuente et al., 2023; Lilak et al., 2024). Ticks and TBD affect cattle health and production in Uganda with increasing incidence and economic impact (Kasaija et al., 2021). Tick-transmitted zoonotic pathogens such as Crimean-Congo hemorrhagic fever (CCHF) affect human health with high incidence in eastern Africa (Rodarte et al., 2023).

Within a One Health perspective, vaccines are the most effective, environmentally friendly and sustainable alternative to chemical acaricides for the control of ticks and TBD (Kasaija et al., 2022a). Anti-tick vaccines progressed from the only registered and commercialized TickGard and Gavac formulations (Gavac is still commercially available in some Latin American countries) based on *Rhipicephalus microplus* BM86 recombinant antigen to the identification and characterization of new tick protective antigens such as Subolesin (SUB) to “personalized medicine” approach and innovative formulations for the control of cattle tick infestations (Kasaija et al., 2022a, 2022b; Muhanguzi et al., 2022).

Bibliometrics is a statistical method to analyze scientific literature and patents for evaluation of progression, identification of major gaps and challenges and prediction of future directions in different fields (Miao et al., 2022; Mukhopadhyay et al., 2024). In this way, bibliometric quantitative analysis uses mathematical and statistical techniques to measure and evaluate the quantity, quality, structure, and impact of scientific publications and patents in a specific field or subject (Kokol et al., 2021; Miao et al., 2022). A recent study on targeted literature search provided information on ticks species and tick-borne pathogens (TBP) in sub-Saharan African countries including Uganda (Lilak et al., 2024).

In this review, we used a bibliometric descriptive approach for the analysis of publications and patents in the fields of ticks, TBD, and vaccines in Uganda. The results were used to identify major gaps and limitations in vaccines for the control of TBP and propose future directions to approach these limitations in Uganda and in other African countries.

## 2 Datasets and analysis

Publications were retrieved from PubMed, National Institutes of Health (NIH), (https://pubmed.ncbi.nlm.nih.gov) and Web of Science Citation Index-Expanded (https://clarivate.com/products/scientific-and-academic-research/research-discovery-and-workflow-solutions/webofscience-platform/web-of-science-core-collection/science-citation-index-expanded/), accessed on February 7, 2024, and including all articles published during 1950–2023. Terms for search as disclosed for each set included “Tick AND Uganda” (T&U), “Tick AND Uganda AND Pathogen” (T&U&P), “Tick AND Uganda AND Vaccine” (T&U&V), “Tick AND Uganda AND Pathogen AND Vaccine” (T&U&P&V), and “Vaccine AND Uganda” (V&U).

Patents filed in Uganda through the African Regional Intellectual Property Organization (ARIPO) for the period 2020–2023 were retrieved from ARIPO Journal, the Official Industrial Property Journal of ARIPO (http://eservice.aripo.org/ppb/pjd/PPBJournalViewList.do), accessed on February 7, 2024. Terms for search included “Vaccine” (Vaccine(s) and/or immunostimulant), and “Tick”. Only “Vaccine” (Vaccine(s) and/or immunostimulant) patents were found.

The number of publications and patents were recorded per year of publication or file/registration, respectively (Supplementary Data S1). Then, data analysis was focused on T&U&P&V term in publications and for V&U term in both publications and patents with emphasis on those co-authored or filed from Uganda. The analysis of most cited articles was conducted for T&V&U and T&V&P&U terms through Scopus (https://www.scopus.com/) with export date February 19, 2024 and used for calculating average citations per year as Number of citations/(2023 – Year of publication) (Supplementary Data S2).

## 3 Bibliometric analysis of scientific publications

**Fig. 1:**
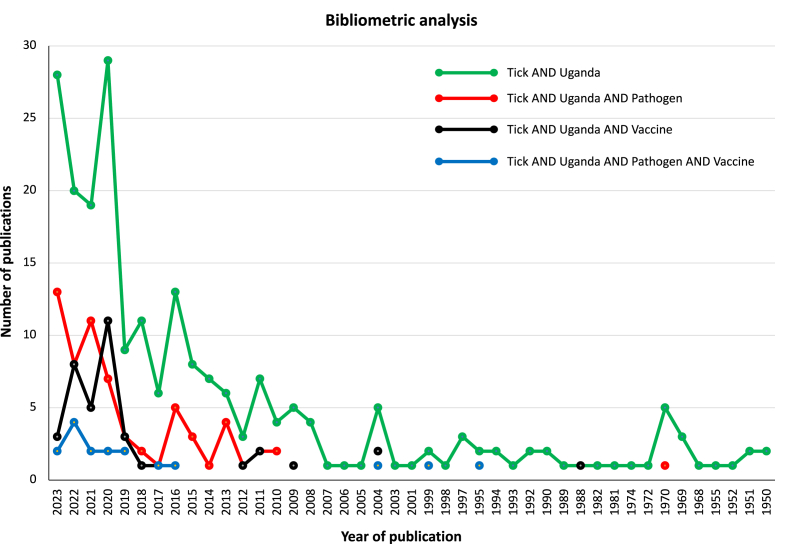
Bibliometric analysis. Publication datasets. Source: PubMed, NIH. Terms for search for each set: “Tick AND Uganda”; “Tick AND Uganda AND Pathogen”; “Tick AND Uganda AND Vaccine”; and “Tick AND Uganda AND Pathogen AND Vaccine”.

**Fig. 2:**
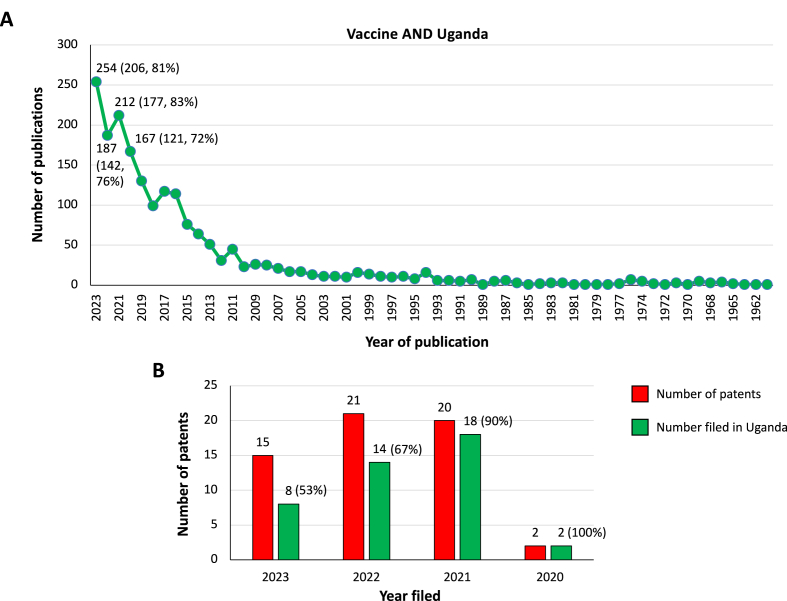
Bibliometric analysis. Publication and patent datasets for “Vaccine AND Uganda”. **A** Source for publications: PubMed, NIH. Term for search: “Vaccine AND Uganda”. Total number of publications, and those co-authored by scientists from Ugandan institutions (*n*, %) during 2020–2023 are indicated. **B** Source for patents: Patents filed in Uganda through ARIPO, 2020–2023. Source for patents ARIPO Journal, the official industrial property journal of ARIPO. Accessed on February 7, 2024. Terms for search: “Vaccine (Vaccine(s) and/or immunostimulant)”; and “Tick”. Only “Vaccine (Vaccine(s) and/or immunostimulant)” patents were found. Total number of patents, and thus filed in Uganda (*n*, %) during 2020–2023 are indicated.

The number of publications per year for each term were as follows: (i) T&U, 225 publications, 1950–2023; (ii) T&U&P, 66 publications, 1970–2023; (iii) T&U&V, 42 publications, 1988–2023; (iv) T&U&P&V, 17 publications, 1995–2023; and (v) V&U, 1897 publications, 1956–2023 (Fig. 1, Fig. 2A; Supplementary Data S1). Although the number of publications tended to increase in recent years (Fig. 1, Fig. 2A), only between 72% and 83% publications in V&U were co-authored by scientists from Ugandan institutions (Fig. 2A).

Focusing on the T&U&P&V term, the 17 publications included research on vaccines for the control of pathogens and tick vectors. Research addressed live vaccines for *Theileria parva* (Morzaria et al., 1999), recombinant vaccines against hemoparasites (Oura et al., 2004), oral vaccine formulation with recombinant *R. microplus* or *Rhipicephalus decoloratus* SUB against multi-species tick infestations (Contreras et al., 2019; Kasaija et al., 2022b), identification of candidate tick-specific Aquaporin-1 vaccine antigen (Ndekezi et al., 2019), modeling structure and characterization of vaccine efficacy with Crimean-Congo hemorrhagic fever virus (CCHFV) secreted glycoprotein (GP38) (Mishra et al., 2020), characterization of *Rhipicephalus appendiculatus* as tick vaccine protective antigen against multiple tick species (Kasaija et al., 2020), methodology for the evaluation of anti-tick vaccine efficacy and effectiveness (Ndawula, 2021), identification of candidate *Theileria parva*, the causative pathogen of East Cost fever, genotypes for cattle vaccine formulations (Muleya et al., 2022), and effectiveness and limitations of “Muguga cocktail” live vaccine, delivered by an infection and treatment method (ITM) for the control of East Coast fever in Uganda (Oligo et al., 2023).

**Fig. 3:**
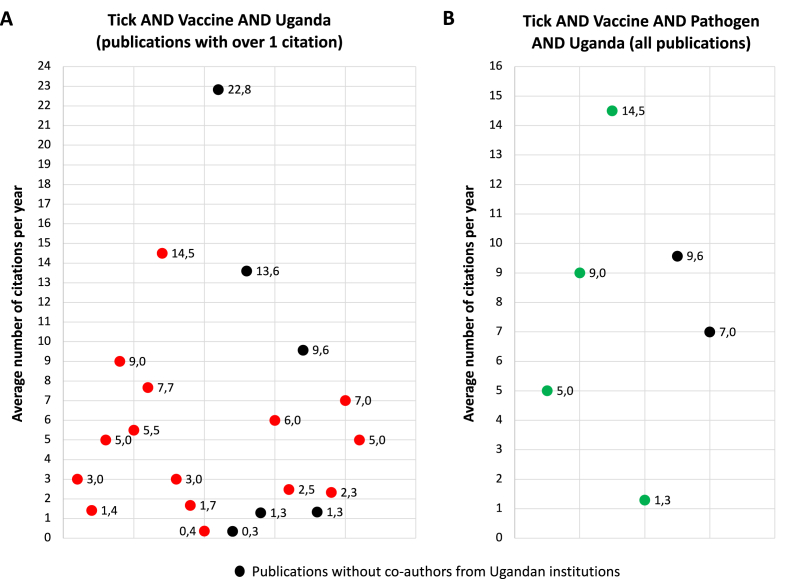
Analysis of most cited publications. Data were collected from Scopus for the terms “Tick AND Vaccine AND Uganda” (T&V&U) (**A**) and “Tick AND Vaccine AND Pathogen AND Uganda” (T&V&P&U) (**B**) for publications with over one citation or all publications, respectively. Average number of citations (Number of citations/(2023 – Year of publication) is shown for each publication with black dots for publications without co-authors from Ugandan institutions.

The analysis of most cited publications was conducted in Scopus for T&V&U (publications with over one citation, *n* = 21; mean ± standard deviation, SD: 5.9 ± 5.6 citations) (Fig. 3A) and T&V&P&U (all publications, n = 6; mean ± SD: 7.7 ± 4.5 citations) (Fig. 3B). Of these, most publications were co-authored by Ugandan institutions with a similar number of citations for both terms, T&V&U (*n* = 15, mean ± SD: 4.9 ± 3.6 citations) (Fig. 3A) and T&V&P&U (*n* = 4, mean ± SD: 8.9 ± 4.1 citations) (Fig. 3B). The most cited publications for each term were Heinz and Stiasny (2017) (22.8 citations/year with a total of 137 citations) and Kasaija et al. (2021) (14.5 citations/year with a total of 29 citations) for T&V&U, and Kasaija et al. (2021) and Pavela et al. (2016) (9.6 citations/year with a total of 67 citations) for T&V&P&U. These publications highlight international collaborations with Ugandan institutions such as the National Livestock Resources Research Institute (NaLIRRI), National Agricultural Research Organization (NARO) and Makerere University.

## 4 Bibliometric analysis of patents

The patents were related to vaccines against pathogens and formulations but not to the control of tick infestations with 8–18 patents, representing 53–100% of the total number of patents filed in Uganda during 2020–2023 (Fig. 2B). None of the patents were filed by Ugandan institutions and no correlation was found between trends in the number of publications and patents (Fig. 2B).

The patents filed in Uganda included: “Vaccine against african swine fever virus (ASFV) infection” (AP/P/2022/014,350, WO2021176236A1, https://patents.google.com/patent/WO2021176236A1/en) filed in 2022; “A multi-epitome DNA vaccine for heartwater” (AP/P/2019/011,737, filed in 2022); “Recombinant vaccine against helminths in *Pichia pastoris* and methods for producing and purifying proteins for use as vaccines against helminths” (AP/P/2019/011,766, filed in 2022); “Vaccine immunogens” (AP/P/2021/013,615, filed in 2022); “Methods for improving the adsorption of polysaccharide-protein conjugates and multivalent vaccine formulation obtained thereof” (AP/P/2018/010,428, filed in 2021); “Immunostimulant for use against pathogens” (AP/P/2021/013,316, filed in 2021); “Preparation including vaccine adjuvant” (AP/P/2021/013,339, filed in 2021 in Uganda), “Mycoplasma vaccines and uses thereof” (AP/P/2018/010,530, filed in 2021); “Ice-lined vaccine refrigerator” (AP/P/2021/012,955, filed in 2021); and “Methods and compositions for enhancing immune responses” (AP/P/2020/012,788, filed in 2020).

## 5 Limitations in vaccines for the control of ticks and TBD in Uganda

The criteria used to identify the main gaps and limitations and future directions in vaccines for the control of ticks and TBD in Uganda were based on authorsʼ experience in this area together with the bibliometric analysis using scientific publications and patents focused on the terms T&U&P&V and V&U with emphasis on those co-authored or filed from Uganda and the analysis of most cited articles for the terms T&V&U and T&V&P&U.

The results of the bibliometric analysis showed that major gaps and limitations in developing vaccines for the control of ticks and tick-borne pathogens in Uganda are associated with (i) low contributions from Ugandan institutions, (ii) limited international collaborations, (iii) poor impact of translational research, and (iv) little research on tick control vaccines.

## 6 Future directions

Agriculture is a key sector in Ugandaʼs economy accounting according to (NARO (https://naro.go.ug) for about 25% of the gross domestic product (GDP), 65% of total export earnings and employing about 70% of the population. NARO is an agency of the Ministry of Agriculture, Animal Industry and Fisheries (MAAIF) in charge of coordinating all agricultural research in Uganda with 16 institutes across the country. NARO research covers different areas of agriculture including livestock, fisheries, crops, forestry, agro-machinery, natural resources, and socioeconomics.

To address major gaps and limitations in developing vaccines against ticks and TBD, ongoing research projects at NARO include “Feed the Future Initiative” (https://naro.go.ug/research/research-projects/) in collaboration with the United States Agency for International Development (USAID) and “Technology and Innovation” (https://naro.go.ug/research/technology-innovation/) for implementation of new innovative technologies including integrated pest and disease management for both crops and livestock to improve nutrition in Uganda. A project on “Deciphering the virome of tick along the wildlife-livestock interface” is ongoing at Makerere University, Kampala (https://rif.mak.ac.ug/deciphering-the-virome-of-tick-along-the-wildlife-livestock-interface/). Within livestock research, NARO constituted at NaLIRRI a vaccinology research programme for vaccine development for the control of tick infestations, food and mouth disease (FMD), African swine fever (ASF), and other livestock and poultry vaccines.

Research on anti-tick vaccine has been implemented in collaboration between NaLIRRI and the group of Health and Biotechnology, SaBio at the Instituto de Investigación en Recursos Cinegéticos (IREC; Spanish National Research Council CSIC, University of Castilla-La Mancha UCLM, Junta de Comunidades de Castilla-La Mancha JCCM) in Spain. The project applied a “personalized medicine” approach to identify the *R. decoloratus* SUB tick protective antigen for the control of multi-species cattle tick infestations in Uganda (Kasaija et al., 2020). Currently, vaccine field trial has been completed for approval by national regulatory authorities of vaccine registration and commercialization (Kabi et al., 2022). Building of production facility and personnel capacity are currently ongoing at NaLIRRI for production of recombinant SUB and vaccine formulation in Uganda. Research is also ongoing with an oral vaccine formulation combining recombinant SUB tick protective antigen with adjuvant (patent “Immunostimulant for use against pathogens” AP/P/2021/013,316; Kasaija et al., 2022b).

Ongoing initiatives at NARO with international collaborations are contributing to addressing major gaps and limitations in developing vaccines for the control of ticks and TBP in Uganda. However, future directions should advance in these collaborative projects together with new initiatives addressing (i) basic and translational research on TBD such as CCHF and ASF, (ii) participation of Ugandan institutions in new international consortia in this area, (iii) promoting communication of these initiatives to Ugandan cattle holders and general population to attract support from public and private sectors, (iv) stimulate and support scientific publications and patents with participation of Ugandan scientists, and (v) build and implement production capacity for vaccines in Uganda. Proposed future directions will also positively impact other African countries.

## Funding

The authors would like to acknowledge the Government of Uganda, National Agricultural Research Organisation, Ministry of Agriculture, Animal Industry and Fisheries, Spanish National Research Council (CSIC), and University of Castilla-La Mancha (UCLM), for their support to the tick vaccine project.

## CRediT authorship contribution statement

**José de la Fuente:** Conceptualization, Methodology, Validation, Investigation, Writing - original draft, Writing - review & editing. **Justus Rutaisire:** Investigation, Writing - original draft, Writing - review & editing. All authors read and approved the final manuscript.

## Declaration of competing interests

The authors declare that they have no known competing interests or personal relationships that could have appeared to influence the work reported in this paper.

## Data Availability

The data supporting the conclusions of this article are included within the article and its supplementary file.
