# Supplementary material for: Bibliometric analysis for the identification of main limitations and future directions of vaccines for the control of ticks and tick-borne pathogens in Uganda
Source: Curr Res Parasitol Vector Borne Dis. 2024 Apr 18;5:100175. doi: 10.1016/j.crpvbd.2024.100175 (PMC11058720; doi:10.1016/j.crpvbd.2024.100175)
Supplement: Multimedia component 1 [file mmc1.pdf]

Supplementary material

Supplementary Data S1. Datasets (publications and patents) for bibliometric analysis.

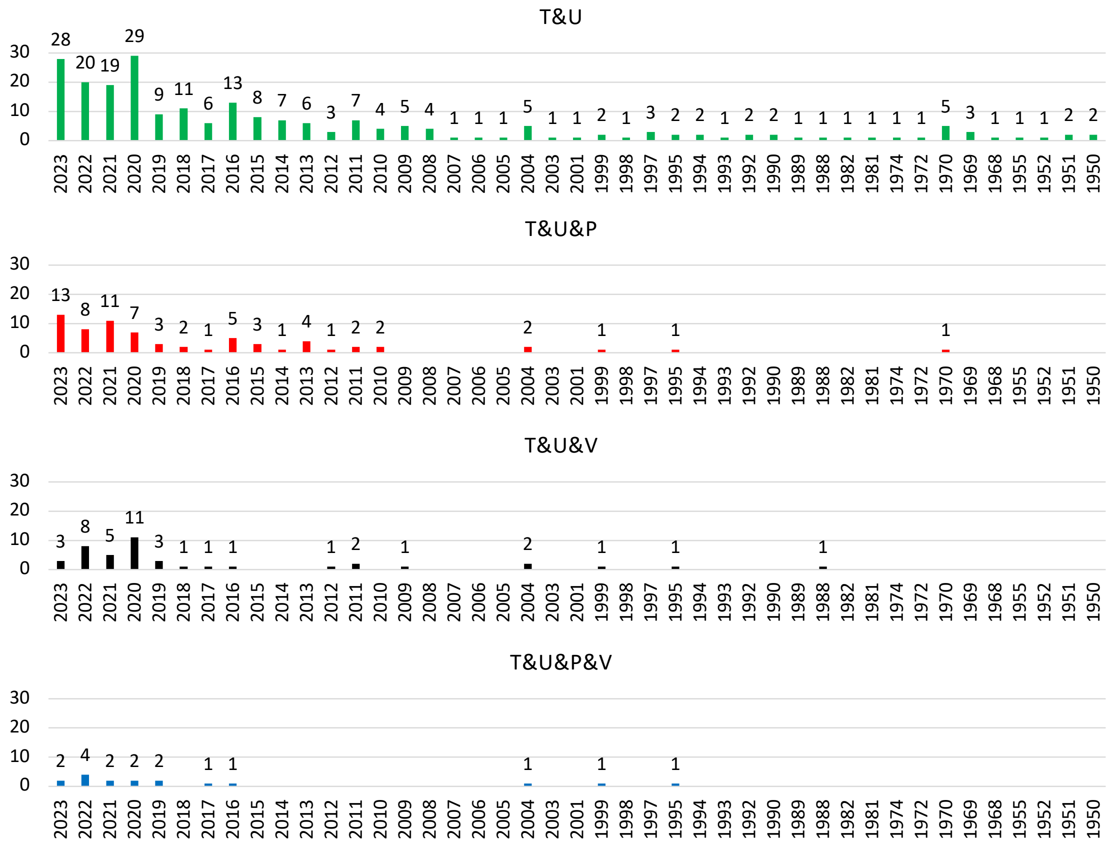

Publication datasets. SOURCE: PUBMED, NIH, https://pubmed.ncbi.nlm.nih.gov and Science Citation Index-Expanded, https://clarivate.com/products/scientific-and-academic-research/research-discovery-and-workflow-solutions/webofscience-platform/web-of-science-core-collection/science-citation-index-expanded/. Accessed on February 7, 2024. **Terms for search:** As disclosed for each set, Tick AND Uganda (T&U), Tick AND Uganda AND Pathogen (T&U&P), Tick AND Uganda AND Vaccine (T&U&V), Tick AND Uganda AND Pathogen AND Vaccine (T&U&P&V).

tickANDUganda-set

1. Parasitology. 1950 Oct;40(3-4):195-209. doi: 10.1017/s0031182000018059.
An experimental study of east coast fever in Uganda. I. A study of the type of east coast fever reactions produced when the number of infected ticks is controlled.
WILSON SG.

2. Parasitology. 1950 Oct;40(3-4):210-4. doi: 10.1017/s0031182000018060.
An experimental study of east coast fever in Uganda. II. The durability of immunity in east coast fever.
WILSON SG.

3. Parasitology. 1951 Jul;41(1-2):23-35. doi: 10.1017/s0031182000016541.
An experimental study of East Coast fever in Uganda. III. A study of the East Coast fever reactions produced when infected ticks 31 days old are fed on susceptible calves in limited numbers over a period of 3 weeks.
WILSON SG.

4. Parasitology. 1951 Jul;41(1-2):36-45. doi: 10.1017/s0031182000016553.
An experimental study of East Coast fever in Uganda. IV. A study of the East Coast fever reactions produced when infected ticks 171 days old were fed on susceptible calves in limited numbers over a period of 3 weeks.
WILSON SG.

5. East Afr Med J. 1952 Nov;29(11):477-9.
First record of Ornithodoros erraticus (Lucas) from Uganda, with some speculations on the origin of Spirochaeta duttoni Novy and Knapp.
HEISCH RB.

6. East Afr Med J. 1955 Sep;32(9):357-60.
A second locality record in Uganda for the tick parasite, Hunterellus hookeri Howard, and a few discussions on other tick parasites.
STEYN JJ.

7. Bull Epizoot Dis Afr. 1968 Dec;16(4):507-12.
The investigation of suspected ixodicide resistance on small dairy farms in Uganda.
Smith MW.

8. Trans R Soc Trop Med Hyg. 1969;63(1):22.
The ecology of cattle ticks in Bugisu, Uganda.
Smith MW.

9. Bull Epizoot Dis Afr. 1969 Mar;17(1):55-75.
Variations in tick species and populations in the Bugisu district of Uganda. I. The tick survey.
Smith MW.

10. Bull Epizoot Dis Afr. 1969 Mar;17(1):77-106.
Variations in tick species and populations in the Bugisu district of Uganda. II. The effects of altitude, climate, vegetation and husbandry on tick species and populations.
Smith MW.

11. Arch Gesamte Virusforsch. 1970;31(1):33-50. doi: 10.1007/BF01241664.
Experimental infection of the argasid tick, Ornithodoros moubata porcinus, with African swine fever virus.
Plowright W, Perry CT, Peirce MA, Parker J.

12. East Afr Med J. 1970 May;47(5):265-72.
Virus isolations from Ixodid ticks in Uganda. I. Isolation and characterisation of ten strains of a virus not previously described from Eastern Africa.
Tukei PM, Williams MC, Mukwaya LG, Henderson BE, Kafuko GW, McCrae AW.

13. East Afr Med J. 1970 May;47(5):273-6.
Virus isolations from Ixodid ticks in Uganda. II. Kadam virus--a new member of arbovirus group B isolated from Rhipicephalus pravus Dontiz.
Henderson BE, Tukei PM, McCrae AW, Ssenkubuge Y, Mugo WN.

14. Bull Epizoot Dis Afr. 1970 Jun;18(2):137-42.
Toxaphene-resistant ticks occurring on cattle in Uganda: Boophilus decolaratus, Rhipicephalus evertsi and Rhipicephalus appendiculatus.
Kitaka FX, Oteng AK, Kamya EP.

15. Am J Trop Med Hyg. 1970 Nov;19(6):Suppl:1141-3.
Congo (CON) strain. 3011.
[No authors listed]

16. J Parasitol. 1972 Oct;58(5):960-78.
Notes on African Haemaphysalis ticks. X. H. (Kaiseriana) aciculifer Warburton and H. (K.) rugosa Santos Dias, the African representatives of the Spinigera sugroup (Ixodoidea: Ixodidae).
Hoogstraal H, el-Kammah KM.

17. Tropenmed Parasitol. 1974 Jun;25(2):207-16.
Some experiments on the transmission of Theileria mutans (Theiler, 1906) and Theileria parva (Theiler, 1904) by the ticks Amblyomma variegatum (Fabricius, 1794) and Rhipicephalus appendiculatus Neumann, 1901, in Uganda.
Uilenberg G, Robson J, Pedersen V.

18. Trop Anim Health Prod. 1981 Feb;13(1):1-11. doi: 10.1007/BF02237878.
Theileriosis in Uganda. Parasitological and serological responses in cattle continually exposed to natural infection.
Robson J, Pedersen V, Uilenberg G, Odeke GM.
Cattle living in a circumscribed area in Uganda, where both Theileria parva and T. mutans occurred naturally, were monitored for periods of up to 1 year by examination of blood and lymph node smears for parasites and the indirect fluorescent antibody test for antibodies to Theileria. Uninfected control cattle were added at intervals to ensure continuous challenge and to monitor the challenge. Although all 29 control animals became infected with T. parva,

challenge was light at first as mortality from theileriosis only commenced in the 18 introduced after day 138 of the study; 14 of these died of the disease. Most of the 34 animals in the original group appeared to be susceptible to T. parva at the start of the study and 6 died of theileriosis. All animals were, or became, infected with T. mutans. Several animals exhibited patent T. parva infections on more than 1 occasion and 1 died from the second infection indicating the existence of immunologically different strains. Recovered animals appeared to acquire eventually a solid immunity to the local Theileria strains but 9 of the original animals and 7 of the controls died exhibiting massive tick and helminth burdens, sometimes aggravated by the effects of previous T. parva and/or T. mutans infections.

19. Trop Anim Health Prod. 1982 May;14(2):63-74. doi: 10.1007/BF02282583.
Relationship between ticks and Zebu cattle in southern Uganda.
Kaiser MN, Sutherst RW, Bourne AS.
Tick populations were observed on zebu (Bos indicus) cattle over a period of 2 years at Entebbe, Uganda where the climate was thought to be highly favourable for the free-living stages of ticks. collections of all instars of ticks were made from the body surfaces of the cattle at intervals of between 1 and 5 weeks. The species recorded in order of decreasing abundance were Rhipicephalus appendiculatus, Amblyomma variegatum, Boophilus decoloratus, Rhipicephalus evertsi evertsi, Rhipicephalus simus, Rhipicephalus compositus and Hyalomma marginatum rufipes. The rankings of the cattle based on burdens of any particular species of tick were always correlated with their rankings for other species; animals that carried more adult stages of a species also carried more of its immature stages. There were more adult males than females of R. appendiculatus, A. variegatum and R. e. evertsi even when the cattle had had all ticks removed 1 week previously; several possible mechanisms are suggested to explain the biased sex ratio. It is concluded that there is promise for improvement in control of 3-host ticks by increasing the resistance of herds of zebu cattle by culling or selective breeding.

20. Vopr Virusol. 1988 Jul-Aug;33(4):461-5.
[Monoclonal antibodies to flavivirus antigens induced by a vaccinal strain of yellow fever].[Article in Russian]
Gaĭdamovich SIa, Mel'nikova EE, Mikheeva TG, Shutkova TM, Sveshnikova NA.
Monoclonal antibodies (MABs) YEL-2 induced by the vaccine FNS Dakar yellow fever (YF) virus were characterized for their capacity to enter into serological reactions and to react with heterologous flaviviruses. YEL-2 MABs belong to the IgG2a class of immunoglobulins, possess the antihemagglutination properties, are active in indirect IF test but do not activate complement and have no neutralizing properties. The inability to enter into CFT in the presence of antihemagglutinating properties suggests that YEL-2 MABs are directed for the structural E glycoprotein. YEL-2 MABs reacted similarly with the vaccine 17D strain and the FNS Dakar strain by which they had been induced. In addition to YF virus, YEL-2 MABs reacted with Tyuleniy, Rosio, Ilhéus, Uganda S, Karshi, and Sokuluk viruses, the reaction with Tyuleniy virus reaching the same titer as

with the homologous virus but was of one-way nature. No reaction of YEL-2 MABs was observed with the viruses of the tick-borne encephalitis complex, Japanese encephalitis, West Nile, Dengue 2 and 4 viruses. These results specify the antigenic classification of flaviviruses.

21. J Gen Virol. 1989 Jan;70 (Pt 1):37-43. doi: 10.1099/0022-1317-70-1-37.
Antigenic relationships between flaviviruses as determined by cross-neutralization tests with polyclonal antisera.
Calisher CH(1), Karabatsos N, Dalrymple JM, Shope RE, Porterfield JS, Westaway EG, Brandt WE.
Author information:
(1)Division of Vector-Borne Viral Diseases, Center for Disease Control, U.S. Department of Health and Human Services, Fort Collins, Colorado 80522-2087.
The recently established virus family Flaviviridae contains at least 68 recognized members. Sixty-six of these viruses were tested by cross-neutralization in cell cultures. Flaviviruses were separated into eight complexes [tick-borne encephalitis (12 viruses), Rio Bravo (six), Japanese encephalitis (10), Tyuleniy (three), Ntaya (five), Uganda S (four), dengue (four) and Modoc (five)] containing 49 viruses; 17 other viruses were not sufficiently related to warrant inclusion in any of these complexes.

22. Vet Parasitol. 1990 Jan;34(4):273-88. doi: 10.1016/0304-4017(90)90074-l.
The effects of bovine tick resistance on the susceptibility of Hyalomma anatolicum anatolicum to infection with Theileria annulata (Ankara).
Rubaire-Akiiki CM(1).
Author information:
(1)Department of Veterinary Parasitology and Microbiology, Faculty of Veterinary Medicine, Makerere University, Kampala, Uganda.
Two Bos taurus calves were made resistant to tick infestation by exposing them to approximately 500 rabbit-reared nymphs of Hyalomma anatolicum anatolicum twice at a 2-week interval. These two calves, together with a tick-susceptible control calf, were inoculated with a stabilate of Theileria annulata (Ankara). Patent infection resulted in all three calves. Seven-hundred and fifty gerbil-reared nymphs were then applied on each of these calves as well as another tick-susceptible calf that was Theileria free. This infestation was carried out on Day 8 post-inoculation. Ticks that dropped on Day 13 post-inoculation were examined to note the development of T. annulata in them and the histological changes that occurred in the gut and salivary glands. During the second phase of feeding, the gut epithelia of the ticks from the tick-resistant calves were less active. There were no notable differences in the characteristics of the developmental stages of T. annulata between the ticks from the tick-resistant calves and those from the susceptible calf. However, ticks from one calf that acquired a higher level of tick resistance were significantly less susceptible to infection by T. annulata. Bovine tick resistance therefore compromises the vector capacity of H. a. anatolicum and this may be of epidemiological significance in the endemic areas of tropical theileriosis.

23. Mol Biochem Parasitol. 1990 May;40(2):203-11. doi: 10.1016/0166-6851(90)90042-k.
SfiI and NotI polymorphisms in Theileria stocks detected by pulsed field gel electrophoresis.
Morzaria SP(1), Spooner PR, Bishop RP, Musoke AJ, Young JR.
Author information:
(1)International Laboratory for Research on Animal Diseases, Nairobi, Kenya.
DNAs of Theileria parva parva, T. p. lawrencei, T. p. bovis and Theileria mutans stocks, from Kenya, Uganda, Zanzibar and Zimbabwe were digested with either SfiI or NotI and analysed using contour-clamped homogeneous electric field (CHEF) and field-inversion gel electrophoresis (FIGE). The SfiI-digested T. parva genomic DNA resolved into approximately 30 fragments while the NotI digestion produced between 4-7 bands. The summation of the sizes of SfiI fragments gave an estimate of 9-10 X 10(6) base pairs for the size of the T. parva genome. Heterogeneity within T. p. parva Muguga, Pemba/Mnarani and Mariakani stocks was detected. All the T. parva stocks analysed showed SfiI and NotI restriction fragment length polymorphisms (RFLP). Hybridisation of 5 SfiI-digested T. parva DNAs with a Plasmodium berghei telomeric repeat probe suggested that most of the polymorphisms and heterogeneity occurred in the telomeric or sub-telomeric regions of the genome. The recognition by the Plasmodium telomeric probe of 7-8 strongly hybridising SfiI bands indicates that the T. parva genome may possess at least 4 chromosomes. The T. mutans genome was cut frequently with the above enzymes resulting in large numbers of fragments predominantly below 50 kb, thus suggesting either a much higher G + C content than T. parva or the presence of highly reiterated G + C-rich regions.

24. Scand J Immunol Suppl. 1992;11:107-9. doi: 10.1111/j.1365-3083.1992.tb01631.x.
Anaplasmosis in Uganda. II. Prevalence of bovine anaplasmosis.
Ssenyonga GS(1), Kakoma I, Montenegro-James S, Nyeko PJ, Nanteza A, Buga R.
Author information:
(1)Makerere University, Faculty of Veterinary Medicine, Kampala, Uganda.
The prevalence of bovine anaplasmosis was studied in 320 Zebu cattle randomly selected from three regions of Uganda (central, south-western and north-western) using DOT-ELISA, Western immunoblotting, Rapid Card Agglutination Test (RCAT), Capillary Tube Agglutination Test (CAT), Complement Fixation Test (CFT), and parasitological techniques. Dried blood on Whatman filter paper no. 1 was eluated in PBS 0.05% Tween 20 prior to testing at an initial dilution of 1:25. The incidence of parasitaemia ranged from 25% in the central region to 35% in the north-western region and the serological prevalence was lower in the central region and highest in the north-west. Prevalence rates assayed by DOT-ELISA and Western immunoblotting were 1.5-fold greater than those tested with RCAT and 3-fold greater than in CAT. The overall prevalence rates by DOT-ELISA and Western immunoblotting compared favourably with CFT data. The present data utilizing dried blood on filter papers indicate that there is a high prevalence of anaplasmosis in those regions of Uganda surveyed and it confirms our observations and those of others that collecting blood on filter papers is a suitable technique for large-scale screening and for seroepidemiological

studies.

25. Onderstepoort J Vet Res. 1992 Jun;59(2):161-2.
Anaplasmosis in Uganda. III. Parasitological and serological evidence of Anaplasma infection in Ugandan goats.
Ssenyonga GS(1), Kakoma I, Nyeko JP, Buga R, Hansen R.
Author information:
(1)Makerere University, Faculty of Veterinary Medicine, Kampala, Uganda.
Randomly selected goat sera from north-western, central, and south-western regions of Uganda were analyzed parasitologically and serologically for evidence of anaplasmosis. Prevalence rates of 3.2% by parasitemia, 4.8% by card-agglutination test, and 12.9% by DOT-ELISA combined with western blotting were established. Parasitologically positive samples were consistently serologically positive. Positive samples were all from either the north-western or south-western regions of the country. Goats in these regions graze with cattle and are presumable exposed to the same tick species. There was no evidence of clinical caprine anaplasmosis, whereas bovine anaplasmosis cases are very common. Rhipicephalus evertsi was frequently observed on goats which cograze with cattle.

26. Onderstepoort J Vet Res. 1993 Sep;60(3):205-10.
Rhipicephalus aquatilis sp. nov. (Acari: Ixodidae), a new tick species parasitic mainly on the sitatunga, Tragelaphus spekei, in east and central Africa.
Walker JB(1), Keirans JE, Pegram RG.
Author information:
(1)Onderstepoort Veterinary Institute, South Africa.
This tick, originally referred to as Rhipicephalus species III by Yeoman & Walker (1967), was first collected from a sitatunga, Tragelaphus spekei, and then occasionally from cattle, in Tanzania. Further collections, mostly from sitatunga, have since been seen from Uganda and Zambia. Descriptions of the adults of this species are given, together with information on their hosts and their distribution.

27. Trop Anim Health Prod. 1994 Feb;26(1):21-7. doi: 10.1007/BF02241128.
Control of tsetse flies in Uganda by dipping cattle in deltamethrin.
Okello-Onen J(1), Heinonen R, Ssekitto CM, Mwayi WT, Kakaire D, Kabarema M.
Author information:
(1)Animal Health Research Centre, Entebbe, Uganda.
The effect of treating cattle with deltamethrin to control tsetse flies and ticks was investigated on 2 ranches 8 km apart in central Uganda where there is a high risk of trypanosomosis. This area had a moderate challenge of Glossina pallidipes. The cattle had very low challenge of Rhipicephalus appendiculatus, Amblyomma variegatum, Boophilus decolaratus and Rhipicephalus evertsi evertsi, due to regular treatment with dioxathion. On one ranch a dip was charged with deltamethrin to treat cattle regularly for 3 months. The other ranch was used as a control for the tsetse population, but the animals continued to be treated regularly with dioxathion using hand sprays. On the ranch using deltamethrin

treatment a reduction of 96.9% in the tsetse population was recorded after 2 treatments at 2 week intervals. Total (100%) control of tsetse was achieved from the fourth treatment up to the end of the trial period. The ranch with dioxathion treatment experienced an overall tsetse reduction of 19.15% during the thirteenth to fifteenth weeks and the factors contributing to this are discussed. However, the mean apparent tsetse density of 4.83 flies/trap/day recorded at the control ranch was significantly different from the mean of 0.81 flies/trap/day ($P < 0.001$) at the ranch using deltamethrin treatment. It was difficult to assess the effect of this product on ticks because of lack of controls. However, there are indications that deltamethrin can also reduce tick populations and the incidence of tick-borne diseases. The possible limitations and practical implications associated with large scale use of this product in the country are discussed.

28. Med Vet Entomol. 1994 Oct;8(4):351-68. doi: 10.1111/j.1365-2915.1994.tb00099.x.
Population dynamics and density-dependent seasonal mortality indices of the tick Rhipicephalus appendiculatus in eastern and southern Africa.
Randolph SE(1).
Author information:
(1)Department of Zoology, University of Oxford, U.K.
Eight sets of previously published data on the seasonal abundance of the tick Rhipicephalus appendiculatus in Burundi, Uganda, Tanzania and Zimbabwe are analysed to yield seasonal mortality indices. Correlations between these indices and abiotic (climatic) and biotic (tick density) variables suggest that it is the stage from females to larvae that is most sensitive to adverse abiotic conditions, specifically low moisture availability. Mortality at the other stages of the tick's life cycle is strongly density-dependent. The precise nature of this density-dependence suggests that it may be caused by acquired resistance to ticks by cattle. Robust correlations between satellite-derived vegetation indices, climatic factors and mortality indices suggest that detailed climatic data, often unavailable, may be replaced by satellite data, now widely available, for use in modelling tick populations.

29. Parasitol Res. 1995;81(4):334-42. doi: 10.1007/BF00931541.
Humoral immune responses to Theileria parva in cattle as measured by two-dimensional western blotting.
Kishima M(1), Dolan TT, Njamunggeh RE, Nkonge CG, Spooner PR.
Author information:
(1)International Laboratory for Research on Animal Diseases, Nairobi, Kenya.
Humoral immune responses to schizont antigens from six stocks of Theileria parva were compared by two-dimensional Western blotting using sera from cattle that had been infected with a T. parva stock or a clone. Isoelectric points of a polymorphic immunodominant molecule (PIM) of schizonts that induces strong antibody responses in cattle ranged from acidic to basic. Molecular masses (Mr) of the PIM of the respective T. parva stocks were as follows: T. parva Muguga, 86 kDa; Mariakani, 83 kDa; Marikebuni, 83 kDa; Uganda, 83 kDa; T. parva Boleni, 83 kDa; and T. parva 7014, 100 kDa. Among nine cattle infected with T. parva

Muguga, four produced antibodies to a basic antigen having an Mr of 32 kDa. The PIM of T. parva Muguga, T. parva Boleni, and T. parva 7014 reacted strongly with serum obtained from an animal that had been infected with T. parva Muguga. Two-dimensional Western blotting using antischizont monoclonal antibodies enabled us to differentiate between stocks of T. parva.

30. Parasitology. 1995 Jul;111 (Pt 1):39-49. doi: 10.1017/s0031182000064581.
Generation and characterization of cloned Theileria parva parasites.
Morzaria SP(1), Dolan TT, Norval RA, Bishop RP, Spooner PR.
Author information:
(1)International Livestock Research Institute, Nairobi, Kenya.
A 3-step procedure for cloning Theileria parva parasites was developed. The first step involved the in vitro infection of a fixed number of bovine lymphocytes with titrated sporozoites. The cell lines obtained from infections initiated using sporozoite/lymphocyte ratios below 1:100 were then selected for cloning as these contained schizont-infected cells, each of which was derived from infection with a single sporozoite. In the second step, these cell lines were cloned by limiting dilution. As sporozoites infect lymphocytes and transform to induce clonal multiplication, this step produced infected cell lines containing both cloned parasites and cloned lymphocytes. In the third step, the cloned cell lines were used to infect cattle and isolation of the parasite in ticks was made during piroplasm parasitaemia. Finally, sporozoites were harvested from infected ticks and used for further characterization. Sporozoites derived from cloned cell lines of T. parva Muguga, Marikebuni, Boleni, Uganda and buffalo-derived 7014 were characterized using monoclonal antibody profiles, DNA restriction fragment length polymorphism detected using repetitive and telomeric probes, in vivo infectivity and, in one case, cross-immunity studies. Additionally, several distinct schizont-infected lymphocyte clones were isolated from the Muguga, Mariakani and buffalo-derived 7014 stocks. The combined results of the characterization revealed that the cloning procedure selected clones of T. parva from the parental stocks which were known to contain a mixture of genetically different parasite populations. The cloning method and the clones generated will be of value in studies of the biology of the parasite and in elucidating the strain specificity of immune responses in cattle.

31. Trop Anim Health Prod. 1997 May;29(2):102-4. doi: 10.1007/BF02632328.
Dermatophilosis and abscessation of lymph nodes in a group of tick-infested horses in Uganda.
Shannon D.

32. Parassitologia. 1997 Jun;39(2):143-4.
Major tick-borne diseases of sheep and goats in the Sudan.
Osman AM(1).
Author information:
(1)Ministry of Animal Resources, Khartoum, Sudan.
Tick-borne diseases of sheep and goats have not been thoroughly investigated in

the Sudan. Heartwater, the most important of the group, was reported only in the early '60s and malignant theileriosis of sheep in the mid '80s. Other tick-borne diseases of sheep and goats like anaplasmosis, Q fever, Nairobi sheep disease and babesiosis are expected to be present although the vector which transmit Nairobi sheep disease, Rhipicephalus appendiculatus is confined to a very narrow stretch on the Southern border with Zaire, Uganda and Kenya.

33. Parasitology. 1997 Sep;115 (Pt 3):265-79. doi: 10.1017/s0031182097001315.
A generic population model for the African tick Rhipicephalus appendiculatus.
Randolph SE(1), Rogers DJ.
Author information:
(1)Department of Zoology, University of Oxford. sarah.randolph@zoology.ox.ac.uk
We present a simulation population model for the African tick Rhipicephalus appendiculatus, based on previous analyses of the mortality factors most closely correlated with observed population changes at 11 sites in equatorial and South Africa. The model incorporates temperature-dependent rates of egg production and development, climate-driven density-independent mortality rates, particularly during the adult-larval stage, and density-dependent regulation of both nymphs and adults. Diapause is also included for tick populations in southern Africa. The model successfully describes both the seasonality and annual range of variation in numbers of each tick stage observed at each of 4 test sites in Uganda, Burundi and South Africa. Sensitivity analysis showed that the final version of the model is robust to 4-fold variation in most parameter values (that were per force based on informed guesses), but is more sensitive to the regression coefficients determining density-dependent interstadial mortality (that were derived from analysis of field data). The model is able to predict the seasonality of ticks from a site in Kenya where a full prior population analysis was not possible because only adults and nymphs had been counted. The model is potentially applicable to other species of ticks, both tropical and temperate, to predict tick abundance and seasonality as risk factors for tick-borne diseases.

34. Prev Vet Med. 1998 Jan;33(1-4):241-50. doi: 10.1016/s0167-5877(97)00035-4.
Financial analysis of dipping strategies for indigenous cattle under ranch conditions in Uganda.
Okello-Onen J(1), Mukhebi AW, Tukahirwa EM, Musisi G, Bode E, Heinonen R, Perry BD, Opuda-Asibo J.
Auhor information:
(1)Livestock Health Research Institute, Tororo, Uganda.
A financial analysis was performed to assess the performance of three acaricide-treatment groups of indigenous breeds (Zebu and Nganda) of cattle on a ranch in Luwero District, Uganda. The treatments were based on different frequencies: twice-a-week dipping, once-a-month dipping and no tick control. The objective was to evaluate the economic justification for intensive acaricide application for tick and tick-borne disease control in Uganda. Data were collected by monitoring cattle performance over a period of 34 months. Biological data collected included the number of cows at the beginning and end

of the study, net starting liveweight, number of calves born, number of animals dying due to tick-borne diseases and other causes, number of animals sold or slaughtered and milk yield. Records of variable costs (acaricides, drugs, labour, etc.) and output prices were assembled and calculated by treatment group. Gross margin and marginal analysis were used in the financial analysis. The exchange rate in 1990-1993 was one US$ to 1200 Uganda shillings. The gross benefits obtained from animal sales, herd value and milk yield were Uganda shillings 1175, 1389 and 1311 per kg of net starting liveweight for animals dipped twice-a-week, once-a-month and not dipped, respectively. The variable costs were Ug. shs. 424, 390 and 360 per kg of net starting liveweight, respectively. Consequently, the gross margins were Ug. shs. 751, 999 and 951 per kg of net starting liveweight. Furthermore, the marginal rate of return (MRR) in changing from no tick control to once-a-month dipping was 160%, while changing from no tick control to twice-a-week dipping was negative (-313%). The above results showed that the value of increased gains in production obtained from twice-a-week dipping strategy does not offset the costs of inputs for intensive dipping. Once-a-month dipping (strategic) therefore appears to be the most-profitable tick-control strategy for the farmer. The need to conduct further studies in different livestock- production systems and to rationalise future tick control policies is discussed.

35. Exp Appl Acarol. 1999 Jan;23(1):79-88. doi: 10.1023/a:1006058317111.
Population dynamics of ticks on indigenous cattle in a pastoral dry to semi-arid rangeland zone of Uganda.
Okello-Onen J(1), Tukahirwa EM, Perry BD, Rowlands GJ, Nagda SM, Musisi G, Bode E, Heinonen R, Mwayi W, Opuda-Asibo J.
Author information:
(1)Livestock Health Research Institute, Tororo, Uganda.
Studies on seasonality and population dynamics of ticks on indigenous cattle and their crosses (calves) were carried out in Buruli Ranching Scheme, Nabiswera, Luwero district of Uganda on three treatment groups of animals: group 1 (twice a week dipping), group 2 (once a month dipping) and group 3 (no tick control). During this study, four major species of ticks of economic importance were recorded in decreasing order of abundance: Rhipicephalus appendiculatus, Rhipicephalus evertsi evertsi, Amblyomma variegatum and Boophilus decoloratus. Of these ticks, a seasonal pattern of activity was only observed in R. appendiculatus ticks, with peak activities occurring during rainy seasons. Greater numbers of ticks were recorded on cows than calves in the three treatment groups, with the exception of A. variegatum where the reverse occurred. The mean numbers of ticks per animal were highly significantly different ($p < 0.01$) when group 1 animals were compared with group 2 and 3 animals. However, no significant differences ($p > 0.05$) were observed in mean tick numbers between group 2 and 3 animals. Highly significant differences ($p < 0.01$) were observed in mean tick numbers on cows and calves (more than 12 months old) in different calving seasons. The state of lactation only affected tick counts on cows in group 1; significantly more ticks ($p < 0.01$) were observed in lactating than non-lactating cows. Furthermore, significantly greater ($p < 0.05$)

numbers of ticks (with the exception of B. decoloratus) were recorded during the second year of study (March 1992-May 1993) than the first year (January 1991-February 1992), despite lower rainfall during the former period.

36. Parassitologia. 1999 Sep;41 Suppl 1:73-80.
Development of sero-diagnostic and molecular tools for the control of important tick-borne pathogens of cattle in Africa.
Morzaria SP(1), Katende J, Musoke A, Nene V, Skilton R, Bishop R.
Author information:
(1)International Livestock Research Institute (ILRI), Nairobi, Kenya.
s.morzaria@cgiar.org
Tick-borne diseases (TBDs) are a major economic constraint to livestock production in sub-Saharan Africa. ILRI is focussing on developing a range of products, such as vaccines, diagnostics and decision support services to underpin improved control programmes against these diseases. We have developed three highly sensitive and specific enzyme linked immuno-assays (ELISAs), which allow precise diagnosis of Theileria parva, Babesia bigemina and Anaplasma marginale. These tests have been standardised and validated using defined experimental and field infection sera. Parasite specific recombinant antigens and monoclonal antibodies against bovine immunoglobulins as secondary antibodies have played an important role in in enhancing the sensitivity and specificity of the assays. They have been further evaluated in on-farm longitudinal sero-epidemiological studies to define infection dynamics and disease risks in various farming systems in Kenya and Uganda. In addition, DNA-based tests for differentiation of Theileria species and characterisation of Theileria parva stocks have been developed. These tests have been derived through physical mapping and sequencing of key elements of the T. parva genome, which include repetitive and telomeric regions, minisatellite sequences, antigen genes and a number of random DNA sequences. These tools are currently being deployed in conjunction with field immunisation programmes to determine the biological impact of introducing live vaccines of T. parva on population dynamics.

37. Vet Rec. 2001 Jun 9;148(23):725. doi: 10.1136/vr.148.23.725.
Cowdriosis (heartwater) in a Ugandan goat: a case report.
Saimo MK(1), Bizimenyera ES, Mugisha A, Okuni JB, Nsamba P.
Author information:
(1)Department of Veterinary Medicine, Makerere University, Kampala, Uganda.

38. Trop Anim Health Prod. 2003 Jun;35(3):237-47. doi: 10.1023/a:1023395413568.

The impact of tick control on the productivity of indigenous cattle under ranch conditions in Uganda.
Okello-Onen J(1), Tukahirwa EM, Perry BD, Rowlands GJ, Nagda SN, Musisi G, Bode E, Heinonen R, Mwayi W, Opuda-Asibo J.
Author information:
(1)Livestock Health Research Institute, PO Box 96, Tororo, Uganda.
The impact of tick control on the productivity of indigenous cattle was

investigated in three herds of East African shorthorn Zebu and Zebu x Ankole (Nganda) breeds, maintained under three tick management systems practised under ranch conditions: twice-a-week dipping, once-a-month dipping, and no tick control. The milk production and the growth rates of pre-weaned and post-weaned calves were monitored once a month over a period of 34 months. Milk offtake was 23% higher during the heavy rainy seasons than in the long dry seasons. Twice-a-week dipping increased the milk offtake by 21% in the second year of study and prolonged the duration of lactation in cows. Similarly, twice-a-week dipping increased the pre-weaning growth rate by 39% in the second year of study, but had no significant effect on the post-weaning growth rate. Generally, the growth rate of calves were greatest during the heavy rainy seasons and least during the long dry seasons. These results provide a basis for assessing the losses under different tick management systems in the various production systems.

39. Int J Parasitol. 2004 Apr;34(5):603-13. doi: 10.1016/j.ijpara.2003.12.012.
Application of a reverse line blot assay to the study of haemoparasites in cattle in Uganda.
Oura CA(1), Bishop RP, Wampande EM, Lubega GW, Tait A.
Author information:
(1)Department of Microbiology and Parasitology, Faculty of Vet Medicine, Makerere University, Kampala, Uganda. chrisoura@hotmail.com
Recent advances in genomic technology have focused many veterinary researchers on the possibility of producing one multivalent recombinant vaccine against all the haemoparasites that infect cattle in the tropics. Before such a vaccine is developed it is essential to define target cattle populations as well as the range of anti-pathogen vaccines required in order to control disease. To further this objective, we have evaluated a reverse line blot (RLB) assay, which simultaneously detects the principal tick transmitted protozoan and rickettsial cattle pathogens, in different epidemiological scenarios in Uganda. A critical question is the sensitivity, particularly in relation to detecting carrier animals. As Theileria parva is considered to be the most important pathogen in the region, we assessed the sensitivity of the RLB assay for T. parva and showed that 1-2 x 10(3) parasites per ml of blood could be detected-a level comparable with previously developed PCR methods and well below conventional microscopic detection. We applied the RLB assay to evaluate the differences in pathogen profiles between crossbred and indigenous cattle and show that there were different profiles, with a low prevalence of T. parva and Theileria taurotragi in the indigenous cattle compared to a high prevalence in the crossbred cattle. In contrast, we show higher prevalences of Theileria mutans and Theileria velifera in the indigenous compared to the crossbred cattle. Interestingly Anaplasma marginale, Babesia bovis and Babesia bigemina were of low prevalence but a high prevalence of Ehrlichia bovis was seen, raising the question of whether this rickettsial species could be pathogenic in cattle. Analysis of animals with clinical symptoms of East Coast Fever showed that, while T. parva is a major cause of these symptoms, T. mutans and possibly T. taurotragi and T. velifera, may also cause clinical disease. Overall, the results presented here

highlight the complexity of tick-borne pathogen infections in cattle in Uganda.

40. Parasitology. 2004 Jul;129(Pt 1):27-42. doi: 10.1017/s003118200400513x.
The persistence of component Theileria parva stocks in cattle immunized with the 'Muguga cocktail' live vaccine against East Coast fever in Uganda.
Oura CA(1), Bishop R, Wampande EM, Lubega GW, Tait A.
Author information:
(1)Department of Microbiology and Parasitology, Faculty of Veterinary Medicine, Makerere University, P.O. Box 7062, Kampala, Uganda. chrisoura@hotmail.com
The 'Muguga cocktail' live vaccine comprises three Theileria parva stocks (Muguga, Kiambu 5 and the buffalo-derived Serengeti-transformed) and has been used extensively in Eastern, Central and Southern Africa with an infection and treatment protocol to protect cattle against East Coast fever. We report the characterization of the three component vaccine stocks using a panel of polymorphic micro-satellite and mini-satellite markers and the development of a stock-derived PCR method that distinguishes two of the vaccine stocks. These markers, with the use of a recently developed Reverse Line Blot assay, have enabled us to address four important questions in relation to vaccination.
First, how closely related are the vaccine stocks, secondly do all three stocks persist post-vaccination and induce a carrier state, thirdly is there evidence for the transmission of the vaccine stocks and fourthly does vaccination prevent infection with local genotypes? The results show that Muguga and Serengeti-transformed stocks are highly related but very distinct from Kiambu 5 that persists in vaccinated cattle establishing a carrier state. No evidence was obtained for the transmission of vaccine stocks to co-grazed animals, although these animals were infected with up to 8 different T. parva genotypes showing there was a significant level of tick challenge. Some of the vaccinated animals become infected with a subset of local genotypes providing evidence for limited vaccine 'breakthrough'.

41. Onderstepoort J Vet Res. 2004 Sep;71(3):189-95. doi: 10.4102/ojvr.v71i3.259.
Endemic stability for Theileria parva infections in Ankole calves of the Ankole ranching scheme, Uganda.
Kivaria FM(1), Heuer C, Jongejan F, Okello-Onen J, Rutagwenda T, Unger F, Boehle W.
Author information:
(1)Population Studies, Animal Disease Research Institute, Dar es Salaam, Tanzania. fredkiv@yahoo.com
A population-based study was carried out on the Ankole ranching scheme in south-west Uganda with the aim of determining the endemic status of Theileria parva infections. For this purpose, the age-related sero-prevalence of T. parva and the specific calf mortality associated with the parasite were assessed.
Blood samples were collected from 931 Ankole calves of up to 12 months of age from 81 randomly selected herds. The relationship between rainfall pattern and whole-body Rhipicephalus appendiculatus counts was determined. The influence of tick control practices on East Coast fever-related calf mortality, and sero-positivity were also determined. A significant ($r2 = 0.76$, $P = 0.000$)

association between R. appendiculatus counts and rainfall was observed. There was no significant (P > 0.05) association between theileriosis-related calf mortality, sero-positivity and the different tick control practices. Antibody prevalence based on the PIM ELISA was above 70% among calves of 6 months of age in 96% in all the herds. Theileria parva-related calf mortality determined by repeated herd visits and farm records ranged between 0 % and 5.4 %. It was concluded that endemic stability for theileriosis, caused by T. parva, existed in the study area, and that the risk of the occurrence of economically important outbreaks of East Coast fever in indigenous cattle was regarded as minimal under the prevailing conditions.

42. J Insect Sci. 2004;4:8. doi: 10.1093/jis/4.1.8. Epub 2004 Mar 25.
The prevalence of serum antibodies to tick-borne infections in Mbale District, Uganda: the effect of agro-ecological zone, grazing management and age of cattle.
Rubaire-Akiiki C(1), Okello-Onen J, Nasinyama GW, Vaarst M, Kabagambe EK, Mwayi W, Musunga D, Wandukwa W.
Author information:
(1)Faculty of Veterinary Medicine, Makerere University, P.O. Box 7062, Kampala, Uganda. rubaire@vetmed.mak.ac.ug
Between August and October 2000, a cross-sectional study was conducted in smallholder dairy farms in Mbale District, Uganda to assess the prevalence of ticks and tick-borne diseases under different grazing systems and agro-ecological zones and understand the circumstances under which farmers operated. A questionnaire was administered to obtain information on dairy farm circumstances and practices. A total of 102 farms were visited and sera and ticks were collected from 478 animals. Sero-prevalence of tick-borne diseases was determined using an enzyme-linked immunoassay. Acaricides were used indiscriminately but the intensity of their use varied with the grazing system and zone. Cattle from different farms mixed for various reasons. During the dry seasons farmers have to get additional fodder from outside their farms that can result in importation of ticks. The prevalence of ticks and serum antibodies to tick-borne infections differed across the grazing systems and zones. The highest serum antibody prevalence (>60%) was recorded in the lowland zone under the free range and tethering grazing systems. The lowest tick challenge and serum antibody levels (<50%) were recorded in the midland and upland zones under a zero-grazing system. These findings suggest that endemic stability to East Coast Fever, babesiosis and anaplasmosis is most likely to have existed in the lowland zone, particularly, under the tethering and free-range grazing systems. Also, endemic stability for babesiosis existed in the upland zones. Endemic instability for East Coast Fever existed in the midland and upland zones. These structured observational studies are instrumental in planning of control strategies for ticks and tick borne diseases since production systems and the cattle population at high risk of the diseases in the district have been identified.

43. Trop Anim Health Prod. 2005 Aug;37(6):479-89. doi: 10.1007/s11250-005-2174-1.

Strategies, effectiveness and rationale of vector-borne disease control in the pastoralist system of south-western Uganda.
Mugisha A(1), McLeod A, Percy R, Kyewalabye E.
Author information:
(1)Veterinary Epidemiology and Economics Research Unit, School of Agriculture, Policy and Development, Reading University, Reading, UK. mugishaa@yahoo.com
In Uganda, control of vector-borne diseases is mainly in form of vector control, and chemotherapy. There have been reports that acaricides are being misused in the pastoralist systems in Uganda. This is because of the belief by scientists that intensive application of acaricide is uneconomical and unsustainable particularly in the indigenous cattle. The objective of this study was to investigate the strategies, rationale and effectiveness of vector-borne disease control by pastoralists. To systematically carry out these investigations, a combination of qualitative and quantitative research methods was used, in both the collection and the analysis of data. Cattle keepers were found to control tick-borne diseases (TBDs) mainly through spraying, in contrast with the control of trypanosomosis for which the main method of control was by chemotherapy. The majority of herders applied acaricides weekly and used an acaricide of lower strength than recommended by the manufacturers. They used very little acaricide wash, and spraying was preferred to dipping. Furthermore, pastoralists either treated sick animals themselves or did nothing at all, rather than using veterinary personnel. Oxytetracycline (OTC) was the drug commonly used in the treatment of TBDs. Nevertheless, although pastoralists may not have been following recommended practices in their control of ticks and tick-borne diseases, they were neither wasteful nor uneconomical and their methods appeared to be effective. Trypanosomosis was not a problem either in Sembabule or Mbarara district. Those who used trypanocides were found to use more drugs than were necessary.

44. Prev Vet Med. 2006 Aug 17;75(3-4):251-66. doi: 10.1016/j.prevetmed.2006.04.015.
Effect of agro-ecological zone and grazing system on incidence of East Coast Fever in calves in Mbale and Sironko Districts of Eastern Uganda.
Rubaire-Akiiki CM(1), Okello-Onen J, Musunga D, Kabagambe EK, Vaarst M, Okello D, Opolot C, Bisagaya A, Okori C, Bisagati C, Ongyera S, Mwayi MT.
Author information:
(1)Makerere University, Department of Veterinary Parasitology and Microbiology, P.O. Box 7062, Kampala, Uganda. rubaire@vetmed.mak.ac.ug
Between May 2002 and February 2003 a longitudinal survey was carried out in Mbale and Sironko Districts of Eastern Uganda to determine the influence of agro-ecological zones (AEZ) and grazing systems on tick infestation patterns and incidence of East Coast Fever (ECF) in bovine calves. The study area was stratified into AEZ (lowland, midland and upland) and grazing systems {zero grazing (ZG), restricted-outdoor grazing (ROG) and communal grazing (CG)}, whose strata had previously been shown to influence the prevalence of ECF, babesiosis and anaplasmosis. One hundred and eighty-five smallholder dairy farms with a total of 198 calves of both sexes, between the ages of 1 day and 6 weeks, were purposively selected from the AEZ-grazing system strata. Nine dynamic cohorts

(11-51 calves in each) of these calves were examined and sampled monthly. Ticks infesting the calves were counted from one side of the animal body and categorized into the different species, sex and feeding status. Sera were collected at recruitment and monthly thereafter and antibodies against Theileria parva, T. mutans, Babesia bigemina, B. bovis and Anaplasma marginale were measured using ELISA. Tick challenge (total and specific) varied with AEZ and grazing system. The risk of infection with T. parva was higher in the lowland zone compared to the upland zone (hazard ratio (HR)=2.59; 95% CI: 1.00-6.34). The risk of infection with T. parva was higher in the CG system than the ZG system (HR=10.00; 95% CI: 3.61-27.92). The incidence risk for sero-conversion, over the 10 months study period, was 62, 16 and 9% in the lowland, midland and upland zones, respectively. Ninety-eight percent of the calves in lowland-CG stratum sero-converted by the age of 6 months, while 56 and 8% did so in the lowland-ROG and the lowland-ZG stratum, respectively. The results of this study show the need to consider farm circumstances and the variation in ECF risk, both spatially and temporally when designing control strategies for ECF.

45. Arch Inst Pasteur Tunis. 2004;81(1-4):47-50.
Reverse line blotting: a new technique for the sensitive detection of tick borne pathogens.
Tait A(1), Oura Cl.
Auhor information:
(1)Department of Veterinary Parasitology, Institute of Comparative Medicine, University of Glasgow, Bearsden Rd, Glasgow G61 1QH, UK.
Recently, a new molecular diagnostic technique for tick borne pathogens has been developed and is called Reverse Line Blotting. This paper outlines the basis of this method and then illustrates its use for the analysis of blood samples obtained from Ugandan cattle and buffalo. The sensitivity and the specificity are discussed in relation to the use of this technique in the Maghreb

46. Emerg Infect Dis. 2007 Oct;13(10):1508-10. doi: 10.3201/eid1310.070078.
Identification of Rickettsiae, Uganda and Djibouti.
Socolovschi C, Matsumoto K, Marie JL, Davoust B, Raoult D, Parola P.

47. Trop Anim Health Prod. 2008 May;40(4):287-97. doi: 10.1007/s11250-007-9093-2.
Socio-economic factors influencing control of vector-borne diseases in the pastoralist system of south western Uganda.
Mugisha A(1), McLeod A, Percy R, Kyewalabye E.
Author information:
(1)Faculty of Veterinary Medicine, Makerere University, Box 7062, Kampala, Uganda. amugisha@vetmed.mak.ac.ug
Research in control of tick-borne diseases and trypanosomosis, and their vectors, namely, ticks and tsetse flies respectively, has been on going for decades. However, very little attention has been paid to the socio-economic factors that are likely to influence the outcome of the interventions in the control of these diseases. Thus, this study was designed to investigate these factors, mainly the intra-household factors influencing decision-making in the

control of Vector-borne diseases in the pastoralist areas of Uganda. These factors included: indigenous technical knowledge, household economic factors, and gender. Both qualitative and quantitative methods were used in the collection and analysis of data. The tools used for data collection included among others, participatory learning and action (PLA), and Case studies. The findings included the following: In pastoralist households, a big proportion of the household budget was allocated to vector-borne diseases control. In the male-headed households, men dominated decision-making on vector-borne diseases control, although the goals and priorities of men and women in these households were not the same. Also, vector-borne disease control was predominantly by use of modern veterinary drugs, and pastoralists treated sick cattle by themselves even in situations where there were veterinary personnel.

48. Vet Parasitol. 2008 Aug 17;155(3-4):273-80. doi: 10.1016/j.vetpar.2008.05.022.
Clinical features associated with seroconversion to Anaplasma marginale, Babesia bigemina and Theileria parva infections in African cattle under natural tick challenge.
Magona JW(1), Walubengo J, Olaho-Mukani W, Jonsson NN, Welburn SC, Eisler MC.
Author information:
(1)National Livestock Resources Research Institute (NaLIRRI), P.O. Box 96, Tororo, Uganda. Magonaw@live.com
A longitudinal study was conducted in Southeast Uganda for 14 months on 640 Zebu cattle kept under natural tick challenge, with a view to identifying clinical features for prediction of seroconversion to Anaplasma marginale, Babesia bigemina and Theileria parva infections. Physical examination, condition scoring and tick counts were undertaken on all cattle every 4 weeks. In addition, 5300 sera were collected and analysed for antibodies against A. marginale, B. bigemina and T. parva infections using the enzyme-linked immunosorbent assay (ELISA). The major clinical features compiled included weight loss, fever (rectal temperature), anaemia (packed cell volume), pallor of mucous membranes, lymph node enlargement, staring coat, diarrhoea and lacrymation. The risk factors included tick challenge at village level, sex, age, Rhipicephalus spp. density and Boophilus spp. density on individual animals. Using a binary logistic regression model, the clinical features and risk factors were analysed. The results suggest that increasing rectal temperature was associated with increased probability for seroconversion to A. marginale, while high level of Rhipicephalus spp. density and increasing packed cell volume (PCV) were significantly associated with reduced probability of seroconversion. Although statistically significant, none of the factors had large effects, with odds ratios (OR) of 0.87, 1.15 and 0.98 for Rhipicephalus spp. density, rectal temperature and PCV, respectively. For B. bigemina infection, a high level of Boophilus spp. density, anaemia and staring coat were significantly associated with increased probability of seroconversion (OR 1.50, 1.78, 1.37, respectively). Presence of lacrymation and old age were associated with reduced probability of seroconversion (OR 0.52, 0.86 respectively). For T. parva infection, lymph node enlargement (OR 1.30) was associated with increased probability of seroconversion, while high Rhipicephalus spp. density and

increasing packed cell volume (PCV) were associated with reduced probability of seroconversion (OR 0.68 and 0.98, respectively). In conclusion, presence and intensity of the respective tick vectors for tick-borne diseases, age and clinical features such as anaemia, fever, staring coat, lymph node enlargement and lacrymation are indicators for seroconversion to A. marginale, B. bigemina and T. parva infections in cattle. These indicators for seroconversion could be exploited in the development of decision support tools for clinical diagnosis of tick-borne diseases.

49. Trop Anim Health Prod. 2009 Jun;41(5):731-9. doi: 10.1007/s11250-008-9245-z.
Economic impact of ticks and tick-borne diseases on cattle production systems around Lake Mburo National Park in South Western Uganda.
Ocaido M(1), Muwazi RT, Opuda JA.
Author information:
(1)Department of Wildlife and Animal Resource Management, Makerere University, Kampala, Uganda. mocaido@vetmed.mak.ac.ug
A longitudinal economic impact study of Ticks and Tick-Borne Diseases (TTBDs) in cattle around Lake Mburo National Park (LMNP) was done. Impact was valued using Uganda Shilling (Ug. Shs) (exchange rate of 1USD to Ug. Shs 1,420). The costs for controlling TTBDs was constituting 85.6 +/- 3.2% (pastoral) and 73.8 +/- 4.2% (ranches) to total disease control costs. The main costs were on tick control, constituting 83.1% (ranches) and 87.9% (pastoral). In pastoral herds, the costs were negatively correlated to herd size (r = -0.99). The mean annual cost per cattle for controlling TTBDs for ranch and pastoral herds was similar Ug. Shs 5,900 +/- 545. The mean annual Economic Cost (EC) of TTBDs per cattle was not significantly different (p > 0.05) between pastoral (Ug. Shs 6,700 +/- 580) and ranch herds (Ug. Shs 7,600 +/- 970). The mean annual EC per cattle was negatively correlated (r = -0.99) with herd size in pastoral systems contrary to positive correlation (r = 0.99) observed among the ranches. The major component of EC of TTBDs of 88.2% (pastoral) and 78.6% (ranches) was due to their control. The other component was owed to mortality, which was positively correlated (p < 0.01) to the ranch herd size. The total annual EC of TTBDs around LMNP was Ug. Shs 437,754,600 (USD 308,144).

50. Vet Parasitol. 2009 Mar 23;160(3-4):301-5. doi: 10.1016/j.vetpar.2008.11.020.
Diagnostic value of rectal temperature of African cattle of variable coat colour infected with trypanosomes and tick-borne infections.
Magona JW(1), Walubengo J, Olaho-Mukani W, Jonsson NN, Eisler MC.

Author information:
(1)National Livestock Resources Research Institute, Tororo, Uganda.
Magonaw@live.com
Diagnosis of major endemic bovine parasitic diseases in sub-Saharan Africa such as trypanosomosis, theileriosis, anaplasmosis, babesiosis and cowdriosis is increasingly relying on clinical diagnosis due to deterioration of veterinary services and laboratory facilities. Pyrexia is a common clinical feature of aforementioned diseases whose detection relies on measurement of rectal

temperature. The research undertaken in this study was aimed at assessing the effects of diurnal changes and variable coat colour of indigenous Nkedi Zebu cattle on the diagnostic value of rectal temperature under tropical conditions. The results revealed that variation in rectal temperature was significantly influenced by time of day it was taken and by the coat colour of the Nkedi Zebu cattle (P<0.001). Rectal temperature experienced diurnal changes: steadily rising to reach a peak at 17.00h before declining. The mean rectal temperature of unhealthy cattle was significantly higher (P<0.05) than that of the healthy ones only between 13.00 and 17.00h of the day. During which period the proportion of unhealthy cattle having a rectal temperature of 39.4 degrees C or higher was significantly higher than that of healthy ones (P<0.001). Regarding the variable coat colour of indigenous breeds, rectal temperature among cattle of different coat colours was significantly different (P<0.05). In conclusion it is important to consider diurnal changes in rectal temperature and differences due to variable coat colour of indigenous African breeds when measuring rectal temperature for assessing pyrexia, during clinical diagnosis of bovine trypanosomosis and tick-borne diseases that are endemic in many countries in sub-Saharan Africa.

51. Trop Anim Health Prod. 2009 Oct;41(7):1299-308. doi: 10.1007/s11250-009-9315-x.
Disease incidence in ranch and pastoral livestock herds around Lake Mburo National Park, in South Western Uganda.
Ocaido M(1), Muwazi RT, Opuda JA.
Author information:
(1)Department of Wildlife and Animal Resource Management, Faculty of Veterinary Medicine, Makerere University, P.O. Box 7062, Kampala, Uganda.
mocaido@vetmed.mak.ac.ug
A longitudinal study was done to monitor incidence of livestock diseases in ranch and pastoral herds around Lake Mburo National Park, in South-western Uganda with a high level of wildlife (impala and zebra) and livestock (cattle and goats) interactions. East Coast Fever (ECF), abortions, helminthosis and starvation due to drought were major livestock disease problems encountered. ECF was a major disease affecting mainly calves, causing mortality both in ranch (8.5%) and pastoral (8.2%) herds. Meanwhile, drought was a more serious in pastoral than ranch herds (p < 0.05), killing 28.5% and 5.8% of adult female cattle in respective production systems. Other endemic diseases found and controlled by vaccination were FMD, CBPP and blackquarter. In both cattle and goats helminthosis was causing mortalities, 3.3% and 0.8% among calves in pastoral and ranches respectively; and 4.8% and 6.9% among kids and sub-adult goats respectively. Many cases of abortions occurred among pastoral herds. In conclusion, the disease incidence among livestock was the same or relatively low as compared to other areas without wildlife.

52. Trop Anim Health Prod. 2010 Jan;42(1):131-6. doi: 10.1007/s11250-009-9396-6.
Socio-economic factors influencing the use of acaricides on livestock: a case study of the pastoralist communities of Nakasongola District, Central Uganda.
Mugabi KN(1), Mugisha A, Ocaido M.

Author information:
(1)Department of Livestock Health and Entomology, Ministry of Agriculture, Animal Industry and Fisheries, P.O Box 513, Entebbe, Uganda. kmugabi@gmail.com
A study was conducted in Nakasongola district to determine socioeconomic factors that influence the use of acaricides on livestock. The information was got through focus group discussions (FGDs) and use of a questionnaire. Questionnaire was administered to one hundred households. Acaricides were used to kill ticks and biting flies which transmit diseases and cause discomfort to livestock. But to a less extent was also done for cosmetic purposes. Most of the farmers were aware of the correct acaricide dilutions as recommended by the manufacturers but they ignored them. But through trial and error came up with their own dilutions, which they said were very cost-effective. Further, they experimented on concoctions of different acaricide mixes and came up with acaricide combinations which were more effective in killing ticks and flies. Veterinarians and acaricide manufacturing companies had called this a malpractice. On the contrary, this should be treated as an innovation by farmers in their endeavour to find a cheaper sustainable method of controlling ticks and flies. Further research should therefore be done on these working "malpractices".

53. J Gen Virol. 2009 Nov;90(Pt 11):2669-2678. doi: 10.1099/vir.0.014183-0. Epub
Isolation of a novel species of flavivirus and a new strain of Culex flavivirus (Flaviviridae) from a natural mosquito population in Uganda.
Cook S(1), Moureau G(2), Harbach RE(1), Mukwaya L(3), Goodger K(1), Ssenfuka F(3), Gould E(4)(2), Holmes EC(5), de Lamballerie X(2).
Author information:
(1)Natural History Museum, Cromwell Road, London SW7 5BD, UK.
(2)Unité des Virus Emergents UMR190 'Emergence des Pathologies Virales', Université de la Méditerranée et Institut de Recherche pour le Développement, Marseille, France.
(3)Mosquito Research Programme, Uganda Virus Research Institute, PO Box 49, Entebbe, Uganda.
(4)Centre for Ecology and Hydrology Oxford, Mansfield Road, Oxford OX1 3SR, UK.
(5)Department of Biology, The Pennsylvania State University, University Park, PA 16802, USA.
The genus Flavivirus, which contains approximately 70 single-stranded, positive-sense RNA viruses, represents a unique model for studying the evolution of vector-borne disease, as it includes viruses that are mosquito-borne, tick-borne or have no known vector. Both theoretical work and field studies suggest the existence of a large number of undiscovered flaviviruses. Recently, the first isolation of cell fusing agent virus (CFAV) was reported from a natural mosquito population in Puerto Rico, and sequences related to CFAV have been discovered in mosquitoes from Thailand. CFAV had previously been isolated from a mosquito cell line in 1975 and represented the only known 'insect-only' flavivirus, appearing to replicate in insect cells alone. A second member of the 'insect-only' group, Kamiti River virus (KRV), was isolated from Kenyan mosquitoes in 2003. A third tentative member of the 'insect-only' group, Culex flavivirus (CxFV), was first isolated in 2007 from Japan and further strains

have subsequently been reported from the Americas. We report the discovery, isolation and characterization of two novel 'insect-only' flaviviruses from Entebbe, Uganda: a novel lineage tentatively designated Nakiwogo virus (NAKV) and a new strain of CxFV. The individual mosquitoes from which these strains were isolated, identified retrospectively by using a reference molecular phylogeny generated using voucher specimens from the region, were Mansonia africana nigerrima and Culex quinquefasciatus, respectively. This represents the first isolation, to our knowledge, of a novel insect-only flavivirus from a Mansonia species and the first isolation of a strain of CxFV from Africa.

54. Parasitol Res. 2011 Jun;108(6):1365-74. doi: 10.1007/s00436-010-2030-8. Epub
Theileria parva genetic diversity and haemoparasite prevalence in cattle and wildlife in and around Lake Mburo National Park in Uganda.
Oura CA(1), Tait A, Asiimwe B, Lubega GW, Weir W.
Author information:
(1)Department of Microbiology and Parasitology, Faculty of Vet. Medicine, University of Makerere, P.O. Box 7062, Kampala, Uganda. chris.oura@bbsrc.ac.uk
Wildlife, especially Cape buffalo (Syncerus caffer), are thought to act as a reservoir for many of the important tick-borne pathogens of cattle. In this study, we have determined the prevalence of the most significant tick-borne haemoparasites in wildlife (buffalo, impala, eland and bushbuck) as well as in cattle grazing inside and neighbouring Lake Mburo National Park (LMNP) in Uganda. A high percentage of buffalo were carriers of Theileria parva, Theileria mutans, Theileria velifera, Theileria buffeli and Theileria sp. (buffalo) as well as Anaplasma marginale and Anaplasma centrale. The majority of impala sampled were carriers of A. centrale, and all were carriers of an unidentified Babesia/Theileria species. The eland and bushbuck sampled were all carriers of Theileria taurotragi and Theileria buffeli, and the majority were carriers of T. mutans. The bushbuck sampled were also carriers for Erhlichia bovis. There were some differences in the prevalence of haemoparasites between the calves sampled inside and neighbouring LMNP. In order to address the question of whether there is evidence for interbreeding between buffalo-associated and cattle-associated T. parva populations, multi-locus genotypes (MLGs) of T. parva (based on micro-satellite markers) from buffalo and from calves grazing inside and outside LMNP were compared, and the results revealed that buffalo and cattle gene pools were distinct, showing no evidence for transmission of buffalo-derived T. parva genotypes to the cattle population.

55. J Gen Virol. 2011 Feb;92(Pt 2):432-44. doi: 10.1099/vir.0.025874-0. Epub 2010
African swine fever viruses with two different genotypes, both of which occur in domestic pigs, are associated with ticks and adult warthogs, respectively, at a single geographical site.
Gallardo C(1), Okoth E, Pelayo V, Anchuelo R, Martín E, Simón A, Llorente A, Nieto R, Soler A, Martín R, Arias M, Bishop RP.
Author information:
(1)Centro de Investigación en Sanidad Animal, INIA, Valdeolmos, Madrid 28130, Spain. gallardo@inia.es

The role of the ancestral sylvatic cycle of the African swine fever virus (ASFV) is not well understood in the endemic areas of eastern Africa. We therefore analysed the ASF infection status on samples collected from 51 free-ranging warthogs (Phacocherus africanus) and 1576 Ornithodorus porcinus ticks from 26 independent warthog burrows at a single ranch in Kenya. Abattoir samples from 83 domestic pigs without clinical symptoms, originating from specific locations with no recent reported ASF outbreaks were included in this study. All samples were derived from areas of central Kenya, where ASF outbreaks have been reported in the past. Infection with ASFV was confirmed in 22 % of O. porcinus pools, 3.22 % of adult warthog serum samples and 49 % of domestic pig serum samples by using p72-based PCR. All of the warthog sera were positive for anti-ASFV antibodies, investigated by using ELISA, but none of the domestic pig sera were positive. Twenty O. porcinus-, 12 domestic pig- and three warthog-derived viruses were genotyped at four polymorphic loci. The ASFV isolates from ticks and domestic pigs clustered within p72 genotype X. By contrast, ASF viruses genotyped directly from warthog sera, at same locality as the tick isolates, were within p72 genotype IX and genetically similar to viruses causing recent ASF outbreaks in Kenya and Uganda. This represents the first report of the co-existence of different ASFV genotypes in warthog burrow-associated ticks and adult wild warthogs. The data from this and earlier studies suggest transfer of viruses of at least two different p72 genotypes, from wild to domestic pigs in East Africa.

56. Vet Parasitol. 2011 Feb 10;175(3-4):212-9. doi: 10.1016/j.vetpar.2010.10.032.
Haemoparasite prevalence and Theileria parva strain diversity in Cape buffalo (Syncerus caffer) in Uganda.
Oura CA(1), Tait A, Asiimwe B, Lubega GW, Weir W.
Author information:
(1)Department of Microbiology and Parasitology, Faculty of Veterinary Medicine, University of Makerere, P.O. Box 7062, Kampala, Uganda. chris.oura@bbsrc.ac.uk
Cape buffalo (Syncerus caffer) are considered to be an important reservoir for various tick-borne haemoparasites of veterinary importance. In this study we have compared the haemoparasite carrier prevalence in buffalo from four geographically isolated national parks in Uganda [Lake Mburo National Park (LMNP), Queen Elizabeth National Park (QENP), Murchison Falls National Park (MFNP) and Kidepo Valley National Park (KVNP)]. Differences were seen in haemoparasite prevalence in buffalo from the four national parks. All the buffalo sampled in LMNP were carriers of Theileria parva however, buffalo from MFNP and KVNP, which are both located in the north of Uganda, were negative for T. parva. Interestingly, 95% of buffalo in the northern part of QENP were T. parva positive, however all buffalo sampled in the south of the park were negative. A high multiplicity of infection was recorded in all the buffalo found to be carrying T. parva, with evidence of at least nine parasite genotypes in some animals. Most of the buffalo sampled in all four national parks were carriers of T. mutans and T. velifera, however none were carriers of T. taurotragi, Babesia bovis, Babesia bigemina, Ehrlichia bovis or Ehrlichia ruminantium. All the buffalo sampled from LMNP were positive for T. buffeli and

T. sp. (buffalo) however, buffalo from the parks in the north of the country (KVNP and MFNP) were negative for these haemoparasites. Anaplasma centrale and Anaplasma marginale were circulating in buffalo from all four national parks. T. parva gene pools from two geographically separated populations of buffalo in two of the national parks in Uganda (LMNP and QENP) were compared. The T. parva populations in the two national parks were distinct, indicating that there was limited gene flow between the populations. The results presented highlight the complexity of tick-borne pathogen infections in buffalo and the significant role that buffalo may play as reservoir hosts for veterinary haemoparasites that have the potential to cause severe disease in domestic cattle.

57. Trop Anim Health Prod. 2011 Jun;43(5):1019-33. doi: 10.1007/s11250-011-9801-9.
Response of Nkedi Zebu and Ankole cattle to tick infestation and natural tick-borne, helminth and trypanosome infections in Uganda.
Magona JW(1), Walubengo J, Kabi F.
Author information:
(1)National Livestock Resources Research Institute, Tororo, Uganda.
magona.joseph@gmail.com
A cross-sectional study was conducted in Soroti district of Uganda to establish important traits of Nkedi Zebu and Ankole cattle regarding their production performance responses to natural infections of trypanosomes, gastrointestinal nematodes, Theileria parva, Babesia bigemina, Anaplasma marginale and tick infestations. Over four visits between October 2006 to August 2007, tick counts were performed and blood, faecal samples and sera were collected from the Nkedi Zebu (295) and Ankole (165) cattle from 86 herds in six locations per visit. Low parasitological prevalence of trypanosome infection (<6%) and high prevalence of gastrointestinal nematode infections (>30%) with low faecal egg counts (110-300 eggs per gramme (EPG)) were observed in the Nkedi Zebu and Ankole cattle. Both breeds had high, moderate and low mean counts of Rhipicephalus appendiculatus (18.0-24.0), Rhipecephalus (Boophilus) decoloratus (3.6-10.3) and Amblyomma variegatum ticks (1.7-4.3), respectively. In addition, both breeds had similar mean packed cell volumes (26.4-31.2) and a similar percentage of animals were anaemic (14.5-36.6%). The Nkedi Zebu cattle further had higher mean optical density (OD) values for antibodies against T. parva (1.093-1.445) and A. marginale infections (0.573-0.583), and significantly ($P < 0.001$) higher mean OD values of antibodies against B. bigemina infections (1.07-2.175) than the Ankole cattle: T. parva (1.030-1.302); A. marginale (0.442-0.603) and B. bigemina infections (0.863-2.154). The Ankole cows produced significantly more ($P < 0.001$) milk per day (2.68 L) than the Nkedi Zebu cows (1.98 L), and the Ankole oxen had significantly higher ($P < 0.05$) draught power output (2.57 days/acre) than the Nkedi Zebu oxen (2.93 days/acre). Liveweights of calves aged 0-12 months of both breeds were comparable, suggesting that the Nkedi Zebu and Ankole cattle under similar disease challenge exhibited similar growth rates. In conclusion, the Nkedi Zebu cattle seem to possess a higher degree of disease resistance against endemic parasitic diseases, while the Ankole cattle seem to possess a moderate degree of disease resistance coupled with a moderate production potential.

58. Exp Appl Acarol. 2011 Oct;55(2):203-13. doi: 10.1007/s10493-011-9456-2. Epub
Spatial variation of tick abundance and seroconversion rates of indigenous cattle to Anaplasma marginale, Babesia bigemina and Theileria parva infections in Uganda.
Magona JW(1), Walubengo J, Olaho-Mukani W, Jonsson NN, Welburn SW, Eisler MC.
Author information:
(1)National Livestock Resources Research Institute (NaLIRRI), P.O. Box 96, Tororo, Uganda. magona.joseph@gmail.com
Tick abundance and seroconversion rates of 640 indigenous cattle in a mixed crop-livestock system in Uganda were investigated in a 14 months longitudinal study. Up to 100% of the cattle in Buyimini, Kubo, Nanjeho, Ojilai and Sitengo villages (high tick challenge zone) were consistently infested with Rhipicephalus appendiculatus, whereas on average 50% of the cattle in Bunghaji, Hitunga and Magoje villages (low tick challenge zone) were inconsistently infested. Likewise, up to 50% of the cattle in Buyimini, Kubo, Nanjeho, Ojilai and Sitengo villages were consistently infested with R. (Boophilus) decoloratus ticks, while on average 30% of the cattle in Bunghaji, Hitunga and Magoje were inconsistently infested. Seroconversion rates of cattle to Anaplasma marginale infection under low tick challenge were higher than those under high tick challenge, but the reverse was true for Babesia bigemina infection. For Theileria parva infection, seroconversion rates of cattle older than 6 months under low tick challenge were significantly higher than those under high tick challenge ($P < 0.05$). However, the likelihood of occurrence of theileriosis cases among calves (0-6 m) under high tick challenge was 6 times (Odds ratio = 5.82 [1.30-36.37]) higher than under low tick challenge. The high density of anti-tick plants Lantana camara and Ocimum suave that were widespread in villages with low tick challenge, among other factors, was probably the cause for unfavourable tick survival.

59. Parasit Vectors. 2011 Jul 15;4:137. doi: 10.1186/1756-3305-4-137.
Multi-locus sequence typing of Ehrlichia ruminantium strains from geographically diverse origins and collected in Amblyomma variegatum from Uganda.
Nakao R(1), Magona JW, Zhou L, Jongejan F, Sugimoto C.
Author information:
(1)Department of Collaboration and Education, Research Center for Zoonosis Control, Hokkaido University, Kita 20, Nishi 10, Kita-ku, Sapporo, Hokkaido 001-0020, Japan.

BACKGROUND: The rickettsial bacterium Ehrlichia ruminantium is the causative agent of heartwater in ruminants. A better understanding of the population genetics of its different strains is, however, needed for the development of novel diagnostic tools, therapeutics and prevention strategies. Specifically, the development of effective vaccination policies relies on the proper genotyping and characterisation of field isolates. Although multi-locus sequence typing (MLST) has been recently developed, only strains from geographically restricted collections have been analysed so far. The expansion of the MLST

database to include global strains with different geographic origins is therefore essential. In this study, we used a panel of reference strains from geographically diverse origins and field samples of E. ruminantium detected from its vector, Amblyomma variegatum, in heartwater-endemic areas in Uganda.
RESULTS: A total of 31 novel alleles (six, four, six, three, two, five, three, and two for gltA, groEL, lepA, lipA, lipB, secY, sodB, and sucA loci, respectively) and 19 novel sequence types (STs) were identified. Both neighbour-joining and minimum spanning tree analyses indicated a high degree of genetic heterogeneity among these strains. No association was observed between genotypes and geographic origins, except for four STs from West African countries. When we performed six different tests for recombination (GeneConv, Bootscan, MaxChi, Chimaera, SiScan, and 3Seq) on concatenated sequences, four possible recombination events were identified in six different STs. All the recombination breakpoints were located near gene borders, indicating the occurrence of intergenic recombination. All four STs that localized to a distinct group in clustering analysis showed evidence of identical recombination events, suggesting that recombination may play a significant role in the diversification of E. ruminantium.
CONCLUSIONS: The compilation of MLST data set across the African continent will be particularly valuable for the understanding of the existing genetic diversity of field isolates in African countries. Comprehensive information on the degree of cross-protection between strains and further understanding of possible relationships between genotypes and phenotypes such as vaccine efficacy are expected to lead to the development of region-specific vaccination strategies.

60. Parasitology. 2012 Jan;139(1):69-82. doi: 10.1017/S003118201100165X. Epub 2011
Development of multiple-locus variable-number tandem-repeat analysis for rapid genotyping of Ehrlichia ruminantium and its application to infected Amblyomma variegatum collected in heartwater endemic areas in Uganda.
Nakao R(1), Morrison LJ, Zhou L, Magona JW, Jongejan F, Sugimoto C.
Author information:
(1)Department of Collaboration and Education, Research Center for Zoonosis Control, Hokkaido University, Kita 20, Nishi 10, Kita-ku, Sapporo, Hokkaido 001-0020, Japan.
The rickettsial bacterium Ehrlichia ruminantium is the causative agent of heartwater, a serious tick-borne disease in ruminants. The genetic diversity of organisms in the field will have implications for cross-protective capacities of any vaccine developed, and for an effective vaccine design strategy proper genotyping and understanding of existing genetic diversity in the field is necessary. We searched for variable-number tandem-repeat (VNTR) loci for use in a multi-locus VNTR analysis (MLVA). Sequencing analysis of 30 potential VNTRs using a panel of 17 reference strains from geographically diverse origins identified 12 VNTRs with allelic profiles differing between strains. Application of MLVA to 38 E. ruminantium-infected Amblyomma variegatum collected from indigenous cattle in 6 different districts of Uganda identified 21 MLVA types. The discriminatory power of MLVA was greater than that of map1 PCR-restriction fragment length polymorphism analysis, with which only 6 genotypes were

obtained. The high discriminatory power as well as cost- effective performance of MLVA provide the potential for this technique to be applied in the future with respect to optimizing vaccine trials by identifying local strain diversity, and also raise the possibility of exploring the association between E. ruminantium genotypes and phenotypes such as pathological outcome in the ruminant host.

61. Trop Anim Health Prod. 2012 Oct;44(7):1593-8. doi: 10.1007/s11250-012-0112-6.
African swine fever among slaughter pigs in Mubende district, Uganda.
Muwonge A(1), Munang'andu HM, Kankya C, Biffa D, Oura C, Skjerve E, Oloya J.
Author information:
(1)Department of Food Safety and Infection Biology, Norwegian School of Veterinary Science, P.O. Box 8146 Dep., 0033, Oslo, Norway.
adrian.muwonge@veths.no
Owing to frequent reports of suspected outbreaks and the presence of reservoir hosts and vectors (warthogs, bushpigs and O. moubata ticks), African swine fever (ASF) is believed to be an endemic disease in Uganda. There have, however, been very few studies carried out to confirm its existence in Uganda. This study was carried out to describe the prevalence of ASF based on pathologic lesions and analysis of serum samples from slaughtered pigs during a suspected outbreak in the Mubende district of Uganda. The study was based on visits to 22 slaughterhouses where individual pigs were randomly selected for a detailed ante-mortem and post-mortem inspections. Sera were also collected for laboratory analysis. A total of 997 pigs (53.7% male and 46.3% female) were examined for lesions suggestive of ASF and sero-positivity of sera for ASF antibodies. The sera were tested using enzyme-linked immunosorbent assay (ELISA) and positive samples were further confirmed with an immunoblot assay. The results showed that 3.8% (38/997) of the pigs examined had clinical signs and post-mortem lesions suggestive of ASF. Two of 997 (0.2%) sera analysed were positive for ASF antibodies. Of the sub-counties investigated, Bagezza (12%) and Kiyuni (11%) had the highest prevalence of lesions suggestive of ASF based on ante- and post-mortem examination results, while Mubende town council (1.7%) had the lowest. This study found a low number of pigs (3.8%) with lesions suggestive of ASF at slaughter and an even lower number of pigs (0.2%) that were seropositive at slaughter, however a significantly higher number of pigs were slaughtered during the outbreak as a strategy for farmers to avoid losses associated with mortality.

62. Vet Parasitol. 2013 Mar 31;193(1-3):8-14. doi: 10.1016/j.vetpar.2012.12.017.
Haemoparasite infection kinetics and the population structure of Theileria parva on a single farm in Uganda.
Asiimwe BB(1), Weir W, Tait A, Lubega GW, Oura CA.
Author information:
(1)Department of Microbiology and Parasitology, Faculty of Veterinary Medicine, University of Makerere, P.O. Box 7062, Kampala, Uganda.
The development of sensitive PCR-based species-specific diagnostics and parasite genotyping methods offer the opportunity to provide important and detailed

information on the infection dynamics of tick-borne disease. In this study we have exploited such tools to investigate the infection kinetics and parasite diversity within Theileria parva in a single farm in Uganda. Initial analysis of a sample of cattle showed high levels of infection with three Theileria species and Ehrlichia bovis, with most animals being infected with more than one pathogen. To study the infection dynamics, newborn calves were sampled longitudinally and it was shown that all animals became infected with T. parva, T. mutans, T. velifera and E. bovis with the average time to first infection being 53, 74, 116 and 109 days, respectively. However, the majority of these calves cleared the infections with T. parva and E. bovis but remained infected with the other two species of Theileria. In order to investigate the diversity of infecting genotypes of T. parva, samples from six calves were genotyped with a single mini-satellite marker at time points over a nine-month period. Each animal was infected with multiple different sets of genotypes and these were lost over different periods of time, implying that immunity is induced against particular infecting strains. To undertake a higher resolution analysis of parasite genotypes, samples from 30 calves were genotyped with a full panel of 12 micro- and mini-satellite markers but, due to the presence of mixed infections, only 16 samples could be used to generate parasite multi-locus genotypes (MLGs). A high degree of diversity of T. parva was seen on the farm, although some MLGs occurred more than once. Similarity analysis demonstrated a level of sub-structuring and the T. parva population was found to be in linkage disequilibrium. The basis for this high diversity coupled with apparent sub-structuring is discussed in relation to the possible causes.

63. J Egypt Soc Parasitol. 2013 Apr;43(1):87-102. doi: 10.12816/0006369.
The mosquito borne West Nile virus infection: is it threating to Egypt or a neglected endemic disease?
El-Bahnasawy MM(1), Khater MK, Morsy TA.
Author information:
(1)The Military Medical Services for Preventive Medicine, Ain Shams University, Cairo 11566&, Egypt. Mamdouh25@hotmail.com
West Nile virus (WNV) is a mosquito-borne zoonotic arbovirus belonging to the genus Flavivirus in the family Flaviviridae. The virus is found in temperate and tropical regions worldwide, but first identified in the West Nile sub-region in the East African nation of Uganda in 1937. Prior to the mid-1990s WNV infection was sporadically and considered a minor risk for humans, until an outbreak in Algeria in 1994, with cases of WNV-caused encephalitis, and the first large outbreak in Romania in 1996; with a high number of cases with neuroinvasive disease. WNV has now spread globally to Europe beyond the Mediterranean Basin and the United States, is now considered to be an endemic pathogen in worldwide especially in Africa The WNV transmission is mainly b y v a rious mosquitoes species, also tickswere incriminated The birds especially passerines are the most commonly infected animal and serving as the prime reservoir host In Egypt more than 110 mosquito species and subspecies and more than 32 genera of ticks were identified. Besides, not less than 150 species of migratory birds visit Egypt annually in addition to 350 resident ones. This review provided an

overview of the current understanding flaviviruses mainly WNFV. Primary care physician and senior nurse should be able to include the disaster diseases in differential diagnosis of various clinical conditions. They should take a thorough history to request specific dependable laboratory test(s) as soon as possible, and positive patient should be transferred to the fever hospital.

64. Parasit Vectors. 2013 Jul 10;6:204. doi: 10.1186/1756-3305-6-204.
Conflict of interest: use of pyrethroids and amidines against tsetse and ticks in zoonotic sleeping sickness endemic areas of Uganda.
Bardosh K(1), Waiswa C, Welburn SC.
Author information:
(1)Division of Pathway Medicine and Centre for Infectious Diseases, School of Biomedical Sciences, College of Medicine and Veterinary Medicine, The University of Edinburgh, Chancellor's Building, 49 Little France Crescent, Edinburgh EH16 4SB, UK. sue.welburn@ed.ac.uk.
BACKGROUND: Caused by trypanosomes and transmitted by tsetse flies, Human African Trypanosomiasis and bovine trypanosomiasis remain endemic across much of rural Uganda where the major reservoir of acute human infection is cattle. Following elimination of trypanosomes by mass trypanocidal treatment, it is crucial that farmers regularly apply pyrethroid-based insecticides to cattle to sustain parasite reductions, which also protect against tick-borne diseases. The private veterinary market is divided between products only effective against ticks (amidines) and those effective against both ticks and tsetse (pyrethroids). This study explored insecticide sales, demand and use in four districts of Uganda where mass cattle treatments have been undertaken by the 'Stamp Out Sleeping Sickness' programme.
METHODS: A mixed-methods study was undertaken in Dokolo, Kaberamaido, Serere and Soroti districts of Uganda between September 2011 and February 2012. This included: focus groups in 40 villages, a livestock keeper survey (n = 495), a veterinary drug shop questionnaire (n = 74), participatory methods in six villages and numerous semi-structured interviews.
RESULTS: Although 70.5% of livestock keepers reportedly used insecticide each month during the rainy season, due to a variety of perceptions and practices nearly half used products only effective against ticks and not tsetse. Between 640 and 740 litres of insecticide were being sold monthly, covering an average of 53.7 cattle/km(2). Sales were roughly divided between seven pyrethroid-based products and five products only effective against ticks. In the high-risk HAT district of Kaberamaido, almost double the volume of non-tsetse effective insecticide was being sold. Factors influencing insecticide choice included: disease knowledge, brand recognition, product price, half-life and mode of product action, product availability, and dissemination of information. Stakeholders considered market restriction of non-tsetse effective products the most effective way to increase pyrethroid use.
CONCLUSIONS: Conflicts of interest between veterinary business and vector control were found to constrain sleeping sickness control. While a variety of strategies could increase pyrethroid use, regulation of the insecticide market could effectively double the number of treated cattle with little cost to

government, donors or farmers. Such regulation is entirely consistent with the role of the state in a privatised veterinary system and should include a mitigation strategy against the potential development of tick resistance.

65. Emerg Infect Dis. 2013 Oct;19(10):1705-7. doi: 10.3201/eid1910.130389.
Rickettsia africae in Amblyomma variegatum ticks, Uganda and Nigeria.
Lorusso V, Gruszka KA, Majekodunmi A, Igweh A, Welburn SC, Picozzi K.

66. Am J Trop Med Hyg. 2013 Nov;89(5):924-7. doi: 10.4269/ajtmh.13-0081. Epub 2013
Coincident tick infestations in the nostrils of wild chimpanzees and a human in Uganda.
Hamer SA(1), Bernard AB, Donovan RM, Hartel JA, Wrangham RW, Otali E, Goldberg TL.
Author information:
(1)Department of Veterinary Integrative Biosciences, Texas A&M University, College Station, Texas; Freelance Nature Photographer, Merchantville, New Jersey; Freelance Nature Photographer, Helena Montana; Department of Biological Sciences, University of Southern California, Los Angeles, California; Department of Human Evolutionary Biology, Harvard University, Cambridge Massachusetts; Makerere University Biological Field Station, Fort Portal, Uganda; Department of Pathobiological Sciences, University of Wisconsin, Madison, Wisconsin.
Ticks in the nostrils of humans visiting equatorial African forests have been reported sporadically for decades, but their taxonomy and natural history have remained obscure. We report human infestation with a nostril tick in Kibale National Park, Uganda, coincident with infestation of chimpanzees in the same location with nostril ticks, as shown by high-resolution digital photography. The human-derived nostril tick was identified morphologically and genetically as a nymph of the genus Amblyomma, but the mitochondrial 12S ribosomal RNA or the nuclear intergenic transcribed spacer 2 DNA sequences of the specimen were not represented in GenBank. These ticks may represent a previously uncharacterized species that is adapted to infesting chimpanzee nostrils as a defense against grooming. Ticks that feed upon apes and humans may facilitate cross-species transmission of pathogens, and the risk of exposure is likely elevated for persons who frequent ape habitats.

67. Ticks Tick Borne Dis. 2013 Dec;4(6):506-12. doi: 10.1016/j.ttbdis.2013.07.001.
High prevalence of spotted fever group rickettsiae in Amblyomma variegatum from Uganda and their identification using sizes of intergenic spacers.

Nakao R(1), Qiu Y(2), Igarashi M(3), Magona JW(4), Zhou L(2), Ito K(3), Sugimoto C(5).
Author information:
(1)Division of Collaboration and Education, Hokkaido University Research Center for Zoonosis Control, Kita 20, Nishi 10, Kita-ku, Sapporo, Hokkaido 001-0020, Japan; Division of Bioinformatics, Hokkaido University Research Center for Zoonosis Control, Kita 20, Nishi 10, Kita-ku, Sapporo, Hokkaido 001-0020, Japan.
(2)Division of Collaboration and Education, Hokkaido University Research Center

for Zoonosis Control, Kita 20, Nishi 10, Kita-ku, Sapporo, Hokkaido 001-0020, Japan.
(3)Division of Bioinformatics, Hokkaido University Research Center for Zoonosis Control, Kita 20, Nishi 10, Kita-ku, Sapporo, Hokkaido 001-0020, Japan.
(4)Division of Collaboration and Education, Hokkaido University Research Center for Zoonosis Control, Kita 20, Nishi 10, Kita-ku, Sapporo, Hokkaido 001-0020, Japan; National Livestock Resources Research Institute (NaLIRRI), Tororo, Uganda.
(5)Division of Collaboration and Education, Hokkaido University Research Center for Zoonosis Control, Kita 20, Nishi 10, Kita-ku, Sapporo, Hokkaido 001-0020, Japan. Electronic address: sugimoto@czc.hokudai.ac.jp.
The spotted fever group (SFG) rickettsiae are obligate intracellular bacteria transmitted by ticks that cause several tick-borne rickettsioses in humans worldwide. This study was intended to determine the prevalence of SFG rickettsiae in Amblyomma variegatum from 7 districts across Uganda. In addition to sequencing of gltA and ompA genes, identification of Rickettsia species based on the sizes of highly variable intergenic spacers, namely, dksA-xerC, mppA-purC, and rpmE-tRNA(fMet) was carried out. Application of multiplex PCR for simultaneous amplification of 3 spacers combined with capillary electrophoresis separation allowed simple, accurate, and high-throughput fragment sizing with considerable time and cost savings. Rickettsia genus-specific real-time PCR detected 136 positives out of 140 samples, giving an overall prevalence of 97.1%. Most samples (n=113) had a size combination of 225, 195, and 341 bp for dksA-xerC, mppA-purC, and rpmE-tRNA(fMet), respectively, which was identical to that of R. africae, a causative agent of African tick bite fever. In addition, several samples had size variants in either dksA-xerC or rpmE-tRNA(fMet). Nonetheless, the partial sequences of gltA and ompA genes of samples of all size combinations showed the greatest similarity to R. africae (99.3-100% for gltA and 98.1-100% for ompA). Given these results, it is highly possible that the tested ticks were infected with R. africae or closely related species. This is a first report on molecular genetic detection of R. africae and its high endemicity in Uganda. Clinicians in this country should be aware of this pathogen as a cause of non-malarial febrile illness. This study provided a starting point for the development of Rickettsia species identification based on the sizes of intergenic spacers. The procedure is simple, rapid, and cost-effective to perform; hence it might be particularly well suited for preliminary species identification in epidemiological investigations. The results may be more detailed and reliable when simultaneous sequencing analysis is performed.
68. Vector Borne Zoonotic Dis. 2014 Mar;14(3):182-8. doi: 10.1089/vbz.2013.1375.
Bartonella species in invasive rats and indigenous rodents from Uganda.
Billeter SA(1), Borchert JN, Atiku LA, Mpanga JT, Gage KL, Kosoy MY.
Author information:
(1)1 Centers for Disease Control and Prevention , Division of Vector Borne Diseases, Fort Collins, Colorado.
The presence of bartonellae in invasive rats (Rattus rattus) and indigenous rodents (Arvicanthis niloticus and Cricetomys gambianus) from two districts in

Uganda, Arua and Zombo, was examined by PCR detection and culture. Blood from a total of 228 R. rattus, 31 A. niloticus, and 5 C. gambianus was screened using genus-specific primers targeting the 16S-23S intergenic spacer region. Furthermore, rodent blood was plated on brain heart infusion blood agar, and isolates were verified as Bartonella species using citrate synthase gene- (gltA) specific primers. One hundred and four fleas recovered from R. rattus were also tested for the presence of Bartonella species using the same gltA primer set. An overall prevalence of 1.3% (three of 228) was obtained in R. rattus, whereas 61.3% of 31 A. niloticus and 60% of five C. gambianus were positive for the presence of Bartonella species. Genotypes related to Bartonella elizabethae, a known zoonotic pathogen, were detected in three R. rattus and one C. gambianus. Bartonella strains, similar to bacteria detected in indigenous rodents from other African countries, were isolated from the blood of A. niloticus. Bartonellae, similar to bacteria initially cultured from Ornithodorus sonrai (soft tick) from Senegal, were found in two C. gambianus. Interestingly, bartonellae detected in fleas from invasive rats were similar to bacteria identified in indigenous rodents and not their rat hosts, with an overall prevalence of 6.7%. These results suggest that if fleas are competent vectors of these bartonellae, humans residing in these two districts of Uganda are potentially at greater risk for exposure to Bartonella species from native rodents than from invasive rats. The low prevalence of bartonellae in R. rattus was quite surprising, in contrast, to the detection of these organisms in a large percentage of Rattus species from other geographical areas. A possible reason for this disparity is discussed.

69. Parasit Vectors. 2014 Mar 3;7:91. doi: 10.1186/1756-3305-7-91.
Prevalence and spatial distribution of Theileria parva in cattle under crop-livestock farming systems in Tororo District, Eastern Uganda.
Muhanguzi D(1), Picozzi K, Hatendorf J, Thrusfield M, Welburn SC, Kabasa JD, Waiswa C.
Author information:
(1)College of Veterinary Medicine Animal Resources and Biosecurity, Makerere University, P,O, Box 7062, Kampala, Uganda. luckydenno@gmail.com.
BACKGROUND: Tick-borne diseases (TBDs) present a major economic burden to communities across East Africa. Farmers in East Africa must use acaracides to target ticks and prevent transmission of tick-borne diseases such as anaplasmosis, babesiosis, cowdriosis and theileriosis; the major causes of cattle mortality and morbidity. The costs of controlling East Coast Fever (ECF), caused by Theileria parva, in Uganda are significant and measures taken to control ticks, to be cost-effective, should take into account the burden of disease. The aim of the present work was to estimate the burden presented by T. parva and its spatial distribution in a crop-livestock production system in Eastern Uganda.
METHODS: A cross sectional study was carried out to determine the prevalence and spatial distribution of T. parva in Tororo District, Uganda. Blood samples were taken from all cattle (n: 2,658) in 22 randomly selected villages across Tororo District from September to December 2011. Samples were analysed by PCR and T.

parva prevalence and spatial distribution determined.
RESULTS: The overall prevalence of T. parva was found to be 5.3%. Herd level prevalence ranged from 0% to 21% with majority of the infections located in the North, North-Eastern and South-Eastern parts of Tororo District. No statistically significant differences in risk of infection were found between age classes, sex and cattle breed.
CONCLUSIONS: T. parva infection is widely distributed in Tororo District, Uganda. The prevalence and distribution of T. parva is most likely determined by spatial distribution of R. appendiculatus, restricted grazing of calves and preferential tick control targeting draft animals.

70. Res Vet Sci. 2014 Oct;97(2):218-25. doi: 10.1016/j.rvsc.2014.07.001. Epub 2014
Drivers and risk factors for circulating African swine fever virus in Uganda, 2012-2013.
Kabuuka T(1), Kasaija PD(2), Mulindwa H(2), Shittu A(3), Bastos AD(4), Fasina FO(5).
Author information:
(1)Department of Production Animal Studies (DPAS), Faculty of Veterinary Science, University of Pretoria, Private Bag X04, Onderstepoort 0110, South Africa; National Livestock Resources Research Institute (NaLIRRI), National Agricultural Research Organisation (NARO), P.O. Box 96, Tororo, Uganda. Electronic address: tkabuuka@naro.go.ug.
(2)National Livestock Resources Research Institute (NaLIRRI), National Agricultural Research Organisation (NARO), P.O. Box 96, Tororo, Uganda.
(3)Department of Epidemiology and Population Health, Institute of Infection and Global Health, University of Liverpool, Leahurst Campus, Cheshire CH64 7TE, UK; Department of Theriogenology and Animal Production, Faculty of Veterinary Medicine, Usmanu Danfodiyo University, P.M.B 2254, Sokoto, Nigeria.
(4)Mammal Research Institute, Department of Zoology and Entomology, Faculty of Natural and Agricultural Science, University of Pretoria, Hatfield, South Africa.
(5)Department of Production Animal Studies (DPAS), Faculty of Veterinary Science, University of Pretoria, Private Bag X04, Onderstepoort 0110, South Africa.
We explored observed risk factors and drivers of infection possibly associated with African swine fever (ASF) epidemiology in Uganda. Representative sub-populations of pig farms and statistics were used in a case-control model. Indiscriminate disposal of pig viscera and waste materials after slaughter, including on open refuse dumps, farm-gate buyers collecting pigs and pig products from within a farm, and retention of survivor pigs were plausible risk factors. Wire mesh-protected windows in pig houses were found to be protective against ASF infection. Sighting engorged ticks on pigs, the presence of a lock for each pig pen and/or a gate at the farm entrance were significantly associated with infection/non-infection; possible explanations were offered. Strict adherence to planned within-farm and community-based biosecurity, and avoidance of identified risk factors is recommended to reduce infection. Training for small-scale and emerging farmers should involve multidimensional

and multidisciplinary approaches to reduce human-related risky behaviours driving infection.

71. Parasit Vectors. 2014 Sep 1;7:414. doi: 10.1186/1756-3305-7-414.
Geographic distribution of non-clinical Theileria parva infection among indigenous cattle populations in contrasting agro-ecological zones of Uganda: implications for control strategies.
Kabi F(1), Masembe C, Muwanika V, Kirunda H, Negrini R.
Author information:
(1)Department of Environmental Management, Molecular Genetics Laboratory, College of Agricultural and Environmental Sciences, Makerere University, P,O, Box 7062/7298 Kampala, Uganda. freddykabi@gmail.com.
BACKGROUND: Non-clinical Theileria parva infection among indigenous cattle occurs upon recovery from primary disease during the first year of life. Continuous exposure to infection through contaminated tick infestations with absence of clinical disease gives rise to endemic stability. Endemic stable populations may become sources of infection if contaminated tick vectors are shared with susceptible exotic cattle. This study aimed at establishing a nationwide distribution of non-clinical T. parva infection among indigenous cattle populations to inform novel control strategies.
METHODS: The occurrence of non-clinical T. parva infection among apparently healthy 925 indigenous cattle from 209 herds spread out in 10 agro-ecological zones (AEZs) was determined using a nested PCR assay. The influence of AEZ, breed, sex, age and farmers' ranking of ECF importance were interrogated for influence of non-clinical parasite occurrence.
RESULTS: The overall prevalence of non-clinical T. parva infection was 30% (278/925). A gradual increase of non-clinical T. parva infection was observed ranging from 17% (95% CI: 0.03-0.23) to 43% (95% CI: 0.3-0.55) in the North Eastern Savannah Grasslands (NESG) to the Western Highland Ranges (WHR) respectively. A similarly associated 18% (95% CI: 0.07-0.28) and 35% (95% CI: 0.3-0.39) non-clinical parasite prevalence was observed among the East African shorthorn Zebu (EASZ) and Ankole cattle respectively. Average herd level non-clinical T. parva prevalence was 28%, ranging from zero to 100%. The likelihood of non-clinical T. parva infection was 35.5% greater in the western highlands compared to the northeastern semi-arid AEZs.
CONCLUSIONS: Non-clinical T. parva occurs countrywide, structured along patterns of AEZ and breed gradients. These findings may guide policy formulation, deployment of integrated control strategies and local cattle improvement programs.
72. Parasit Vectors. 2014 Sep 8;7:432. doi: 10.1186/1756-3305-7-432.
Collateral benefits of restricted insecticide application for control of African trypanosomiasis on Theileria parva in cattle: a randomized controlled trial.
Muhanguzi D(1), Picozzi K, Hatendorf J, Thrusfield M, Welburn SC, Kabasa JD, Waiswa C.
Author information:
(1)Department of Biomolecular and Biolaboratory Sciences, College of Veterinary Medicine Animal Resources and Biosecurity, Makerere University, P,O, Box 7062,

Kampala, Uganda. luckydenno@covab.mak.ac.ug.
BACKGROUND: Tick and tsetse-borne diseases (TTBDs) constrain livestock production in tropical and subtropical regions of the world. Of this community of endemic diseases, East coast fever (T. parva) is the most important tick-borne disease (TBD) accounting for 70% of all losses due to TBDS in this region where control efforts target either tsetse or TBDs and seldom both. In those instances where simultaneous pyrethroid insecticide TTBD control is implemented, collateral benefits of tsetse control on TBD control have not been quantified. In the interest of guiding future TTBD control efforts, the effect of restricting pyrethroid insecticides to the legs, belly and ears (RAP) of cattle for tsetse and trypanosomiasis control on T. parva prevalence in crop-livestock production systems in Tororo district, south-eastern Uganda was determined.
METHODS: We randomly allocated 16 villages to diminazene diaceturate (DA) and 3 graded RAP (25%, 50% and 75% of village herd sprayed respectively) treatment regimens. All cattle were ear-tagged, treated with diminazene diaceturate (DA) and those in regimens 2-4 received monthly graded RAP. Blood samples taken fourteen days post DA treatment and once three monthly were analysed by molecular techniques for T. parva.
RESULTS: In total, 8,975 samples from 3,084 animals were analysed. Prevalence of T. parva varied between 1-3% in different treatment regimens. RAP regimens were associated with slightly lower average risk of infection compared to DA. However, the confidence interval was broad and the result was not statistically significant. There was no evidence of a dose response relationship between graded RAP and T. parva prevalence. These findings are discussed herein with regard to endemic stability development to different TBDs.
CONCLUSIONS: We found only a slight effect of RAP on T. parva infection. Since sample size determination was based on trypanosomes incidence, the study was underpowered given the low T. parva prevalence. While the findings need to be confirmed in future studies, the observed slight reduction in the risk of infection with T. parva might not compromise endemic stability.

73. Parasit Vectors. 2014 Dec 23;7:603. doi: 10.1186/s13071-014-0603-6.
The burden and spatial distribution of bovine African trypanosomes in small holder crop-livestock production systems in Tororo District, south-eastern Uganda.
Muhanguzi D(1)(2), Picozzi K(3), Hattendorf J(4)(5), Thrusfield M(6), Kabasa JD(7), Waiswa C(8), Welburn SC(9).
Author information:
(1)Department of Biomolecular and Biolaboratory Sciences, School of Biosecurity, Biotechnical and Laboratory Sciences, College of Veterinary Medicine Animal Resources and Biosecurity, Makerere University, P.O. Box 7062, Kampala, Uganda. luckydenno@covab.mak.ac.ug.
(2)Division of Infection & Pathway Medicine, Centre for Infectious Diseases, School of Biomedical Sciences, College of Medicine and Veterinary Medicine, The University of Edinburgh, Chancellor's Building, 49 Little France Crescent, Edinburgh, EH16 4SB, UK. luckydenno@covab.mak.ac.ug.

(3)Division of Infection & Pathway Medicine, Centre for Infectious Diseases, School of Biomedical Sciences, College of Medicine and Veterinary Medicine, The University of Edinburgh, Chancellor's Building, 49 Little France Crescent, Edinburgh, EH16 4SB, UK. kim.picozzi@ed.ac.uk.
(4)Department of Public Health and Epidemiology, Swiss Tropical Institute, Socinstrasse 57, CH-4002, Basel, Switzerland. Jan.Hattendorf@unibas.ch.
(5)University of Basel, Petersplatz 1, 4003, Basel, Switzerland. Jan.Hattendorf@unibas.ch.
(6)Royal (Dick) School of Veterinary Studies, The University of Edinburgh, Edinburgh, EH25 9RG, UK. M.Thrusfield@ed.ac.uk.
(7)Department of Biosecurity, Ecosystems & Veterinary Public Health, School of Biosecurity, Biotechnical and Laboratory Sciences, College of Veterinary Medicine Animal Resources and Biosecurity, Makerere University, P.O. Box 7062, Kampala, Uganda. kabasajd@covab.mak.ac.ug.
(8)Department of Pharmacy, Clinical and Comparative Medicine, School of Veterinary Medicine and Animal Resources, College of Veterinary Medicine Animal Resources and Biosecurity, Makerere University, P.O. Box 7062, Kampala, Uganda. cwaiswa@covab.mak.ac.ug.
(9)Division of Infection & Pathway Medicine, Centre for Infectious Diseases, School of Biomedical Sciences, College of Medicine and Veterinary Medicine, The University of Edinburgh, Chancellor's Building, 49 Little France Crescent, Edinburgh, EH16 4SB, UK. sue.welburn@ed.ac.uk.
BACKGROUND: African animal trypanosomiasis (AAT) is considered to be one of the greatest constraints to livestock production and livestock-crop integration in most African countries. South-eastern Uganda has suffered for more than two decades from outbreaks of zoonotic Human African Trypanosomiasis (HAT), adding to the burden faced by communities from AAT. There is insufficient AAT and HAT data available (in the animal reservoir) to guide and prioritize AAT control programs that has been generated using contemporary, sensitive and specific molecular techniques. This study was undertaken to evaluate the burden that AAT presents to the small-scale cattle production systems in south-eastern Uganda.
METHODS: Randomised cluster sampling was used to select 14% (57/401) of all cattle containing villages across Tororo District. Blood samples were taken from all cattle in the selected villages between September-December 2011; preserved on FTA cards and analysed for different trypanosomes using a suite of molecular techniques. Generalized estimating equation and Rogen-Gladen estimator models were used to calculate apparent and true prevalences of different trypanosomes while intra cluster correlations were estimated using a 1-way mixed effect analysis of variance (ANOVA) in R statistical software version 3.0.2.
RESULTS: The prevalence of all trypanosome species in cattle was 15.3% (95% CI; 12.2-19.1) while herd level trypanosome species prevalence varied greatly between 0-43%. Trypanosoma vivax (17.4%, 95% CI; 10.6-16.8) and Trypanosoma brucei rhodesiense (0.03%) were respectively, the most, and least prevalent trypanosome species identified.
CONCLUSIONS: The prevalence of bovine trypanosomes in this study indicates that AAT remains a significant constraint to livestock health and livestock production. There is need to implement tsetse and trypanosomiasis control

efforts across Tororo District by employing effective, cheap and sustainable tsetse and trypanosomiasis control methods that could be integrated in the control of other endemic vector borne diseases like tick-borne diseases.

74. J S Afr Vet Assoc. 2014 Oct 28;85(1):1094. doi: 10.4102/jsava.v85i1.1094.
Bovine trypanosome species prevalence and farmers' trypanosomiasis control methods in south-western Uganda.
Alingu RA, Muhanguzi D(1), MacLeod E, Waiswa C, Fyfe J.
Author information:
(1)College of Veterinary Medicine Animal Resources and Biosecurity, Makerere University. luckydenno@gmail.com.
A cross-sectional study was conducted in Mbarara district, south-western Uganda in May 2012 to determine the burden of African animal trypanosomosis (AAT) in the semi-intensive dairy production systems where pyrethroid acaricides are frequently used in the control of tick-borne diseases (TBDs). A total of 295 cattle blood samples were taken and analysed using a single pair of primers previously designed to amplify internal transcribed spacer (ITS1) of trypanosome ribosomal deoxyribonucleic acid (rDNA). A structured questionnaire was administered to 55 participating livestock farmers to generate data on acaricide and trypanocidal drug usage. The overall prevalence of trypanosome species was 2.4% (95% CI; 1.0% - 4.8%); Trypanosoma vivax was the most predominant species (2.0%; 95% CI; 0.7% - 4.4%). A single mixed infection of T. vivax and Trypanosoma brucei s.l. was detected. All the participating farmers used acaricides for tsetse and TBD control; 89.1% of the acaricides used were pyrethroids. About half of the farmers used trypanocidal drugs, mainly diminazene formulations (Berenil®). Low prevalence of trypanosomes in examined samples is most likely related to the frequent use of pyrethroid insecticides, trypanocides and restricted grazing (paddocking and tethering). These rigorous management practices are geared towards optimising production of exotic dairy breeds kept in this region that are highly susceptible to TBDs and AAT.

75. Parasit Vectors. 2015 Jun 5;8:306. doi: 10.1186/s13071-015-0919-x.
Infection and exposure to vector-borne pathogens in rural dogs and their ticks, Uganda.
Proboste T(1), Kalema-Zikusoka G(2), Altet L(3), Solano-Gallego L(4), Fernández de Mera IG(5), Chirife AD(6), Muro J(7), Bach E(8), Piazza A(9), Cevidanes A(10), Blanda V(11), Mugisha L(12)(13), de la Fuente J(14)(15), Caracappa S(16), Millán J(17).
Author information:
(1)Facultad de Ciencias Silvoagropecuarias, Universidad Mayor, Santiago, Chile. tatianaproboste@gmail.com.
(2)Conservation Through Public Health, Plot 3 Mapera Lane, Uringi Crescent, Entebbe, Uganda. gladys@ctph.org.
(3)VetGenomics, Autonomous University of Barcelona, Cerdanyola, 08193, Barcelona, Spain. laura.altet@vetgenomics.com.
(4)Departament de Medicina i Cirurgia Animals, Universitat Autònoma de Barcelona, Cerdanyola, 08193, Barcelona, Spain. lsolano@quiro.uab.es.

(5)Health & Biotechnology (SaBio) Group, IREC (CSIC-UCLM- JCCM), Ronda de Toledo s/n, Ciudad Real, 13005, Spain. mariaisabel.garcia@uclm.es.
(6)Camino del Rosario P5, Peñaflor, Santiago, Chile. andrea.chirife@gmail.com.
(7)Andorra Veterinary Services, Government of Andorra, Andorra La Vella, Andorra. jesusmuro@andorra.ad.
(8)Servei d'Hematologia Clinica Veterinaria (SHCV), Departament de Medicina i Cirurgia Animals, Facultat de Veterinaria, Universitat Autònoma de Barcelona, Cerdanyola, 08193, Barcelona, Spain. ester.bach@gmail.com.
(9)Istituto Zooprofilattico Sperimentale della Sicilia, via Gino Marinuzzi 3, 90129, Palermo, Italy. ninipiazza@hotmail.it.
(10)Facultad de Ecología y Recursos Naturales, Universidad Andres Bello, Republica 252, Santiago, Chile. aitorcevi@gmail.com.
(11)Istituto Zooprofilattico Sperimentale della Sicilia, via Gino Marinuzzi 3, 90129, Palermo, Italy. valeria.blanda@gmail.com.
(12)College of Veterinary Medicine, Animal Resources and Biosecurity, Makerere University, Kampala, 7062, Uganda. mugishalaw@gmail.com.
(13)Conservation & Ecosystem Health Alliance (CEHA), Kampala, 34153, Uganda. mugishalaw@gmail.com.
(14)Departament de Medicina i Cirurgia Animals, Universitat Autònoma de Barcelona, Cerdanyola, 08193, Barcelona, Spain. josedelafuente@yahoo.com.
(15)Department of Veterinary Pathobiology, Center for Veterinary Health Sciences, Oklahoma State University, Stillwater, 74078, OK, USA. josedelafuente@yahoo.com.
(16)Istituto Zooprofilattico Sperimentale della Sicilia, via Gino Marinuzzi 3, 90129, Palermo, Italy. santo.caracappa@gmail.com.
(17)Facultad de Ecología y Recursos Naturales, Universidad Andres Bello, Republica 252, Santiago, Chile. syngamustrachea@hotmail.com.
BACKGROUND: In rural parts of Africa, dogs live in close association with humans and livestock, roam freely, and usually do not receive prophylactic measures. Thus, they are a source of infectious disease for humans and for wildlife such as protected carnivores. In 2011, an epidemiological study was carried out around three conservation areas in Uganda to detect the presence and determine the prevalence of vector-borne pathogens in rural dogs and associated ticks to evaluate the risk that these pathogens pose to humans and wildlife.
METHODS: Serum samples (n = 105), blood smears (n = 43) and blood preserved on FTA cards (n = 38) and ticks (58 monospecific pools of Haemaphysalis leachi and Rhipicephalus praetextatus including 312 ticks from 52 dogs) were collected from dogs. Dog sera were tested by indirect immunofluorescence to detect the presence of antibodies against Rickettsia conorii and Ehrlichia canis. Antibodies against R. conorii were also examined by indirect enzyme immunoassay. Real time PCR for the detection of Rickettsia spp., Anaplasmataceae, Bartonella spp. and Babesia spp. was performed in DNA extracted from FTA cards and ticks.
RESULTS: 99% of the dogs were seropositive to Rickettsia spp. and 29.5% to Ehrlichia spp. Molecular analyses revealed that 7.8% of the blood samples were infected with Babesia rossi, and all were negative for Rickettsia spp. and Ehrlichia spp. Ticks were infected with Rickettsia sp. (18.9%), including R. conorii and R. massiliae; Ehrlichia sp. (18.9%), including E. chaffeensis and

Anaplasma platys; and B. rossi (1.7%). Bartonella spp. was not detected in any of the blood or tick samples.
CONCLUSIONS: This study confirms the presence of previously undetected vector-borne pathogens of humans and animals in East Africa. We recommend that dog owners in rural Uganda be advised to protect their animals against ectoparasites to prevent the transmission of pathogens to humans and wildlife.

76. Parasit Vectors. 2015 Jul 22;8:387. doi: 10.1186/s13071-015-0998-8.
Cost analysis of options for management of African Animal Trypanosomiasis using interventions targeted at cattle in Tororo District; south-eastern Uganda.
Muhanguzi D(1)(2), Okello WO(3), Kabasa JD(4), Waiswa C(5), Welburn SC(6), Shaw AP(7)(8).
Author information:
(1)Department of Biomolecular and Biolaboratory Sciences, School of Biosecurity, Biotechnical and Laboratory Sciences, College of Veterinary Medicine Animal Resources and Biosecurity, Makerere University, P.O. Box 7062, Kampala, Uganda. luckydenno@covab.mak.ac.ug.
(2)Division of Infection and Pathway Medicine, Centre for Infectious Diseases, School of Biomedical Sciences, College of Medicine and Veterinary Medicine, The University of Edinburgh, Chancellor's Building, 49 Little France Crescent, Edinburgh, EH16 4SB, UK. luckydenno@covab.mak.ac.ug.
(3)Division of Infection and Pathway Medicine, Centre for Infectious Diseases, School of Biomedical Sciences, College of Medicine and Veterinary Medicine, The University of Edinburgh, Chancellor's Building, 49 Little France Crescent, Edinburgh, EH16 4SB, UK. waltokello@yahoo.com.
(4)Department of Biosecurity, Ecosystems & Veterinary Public Health, School of Biosecurity, Biotechnical and Laboratory Sciences, College of Veterinary Medicine Animal Resources and Biosecurity, Makerere University, P.O. Box 7062, Kampala, Uganda. kabasajd@covab.mak.ac.ug.
(5)Department of Pharmacy, Clinical and Comparative Medicine, School of Veterinary Medicine and Animal Resources, College of Veterinary Medicine Animal Resources and Biosecurity, Makerere University, P.O. Box 7062, Kampala, Uganda. cwaiswa@covab.mak.ac.ug.
(6)Division of Infection and Pathway Medicine, Centre for Infectious Diseases, School of Biomedical Sciences, College of Medicine and Veterinary Medicine, The University of Edinburgh, Chancellor's Building, 49 Little France Crescent, Edinburgh, EH16 4SB, UK. sue.welburn@ed.ac.uk.
(7)Division of Infection and Pathway Medicine, Centre for Infectious Diseases, School of Biomedical Sciences, College of Medicine and Veterinary Medicine, The University of Edinburgh, Chancellor's Building, 49 Little France Crescent, Edinburgh, EH16 4SB, UK. alex@apconsultants.co.uk.
(8)Avia-GIS, Risschotlei 33, B-2980, Zoersel, Belgium. alex@apconsultants.co.uk.
BACKGROUND: Tsetse-transmitted African trypanosomes cause both nagana (African animal Trypanosomiasis-AAT) and sleeping sickness (human African Trypanosomiasis - HAT) across Sub-Saharan Africa. Vector control and chemotherapy are the contemporary methods of tsetse and trypanosomiasis control in this region. In most African countries, including Uganda, veterinary services have been

decentralised and privatised. As a result, livestock keepers meet the costs of most of these services. To be sustainable, AAT control programs need to tailor tsetse control to the inelastic budgets of resource-poor small scale farmers. To guide the process of tsetse and AAT control toolkit selection, that now, more than ever before, needs to optimise resources, the costs of different tsetse and trypanosomiasis control options need to be determined.
METHODS: A detailed costing of the restricted application protocol (RAP) for African trypanosomiasis control in Tororo District was undertaken between June 2012 and December 2013. A full cost calculation approach was used; including all overheads, delivery costs, depreciation and netting out transfer payments to calculate the economic (societal) cost of the intervention. Calculations were undertaken in Microsoft Excel without incorporating probabilistic elements.
RESULTS: The cost of delivering RAP to the project was US$ 6.89 per animal per year while that of 4 doses of a curative trypanocide per animal per year was US$ 5.69. However, effective tsetse control does not require the application of RAP to all animals. Protecting cattle from trypanosome infections by spraying 25%, 50% or 75% of all cattle in a village costs US$ 1.72, 3.45 and 5.17 per animal per year respectively. Alternatively, a year of a single dose of curative or prophylactic trypanocide treatment plus 50% RAP would cost US$ 4.87 and US$ 5.23 per animal per year. Pyrethroid insecticides and trypanocides cost 22.4 and 39.1% of the cost of RAP and chemotherapy respectively.
CONCLUSIONS: Cost analyses of low cost tsetse control options should include full delivery costs since they constitute 77.6% of all project costs. The relatively low cost of RAP for AAT control and its collateral impact on tick control make it an attractive option for livestock management by smallholder livestock keepers.

77. Am J Trop Med Hyg. 2015 Nov;93(5):1041-51. doi: 10.4269/ajtmh.15-0344. Epub 2015
Genomic Characterization of Yogue, Kasokero, Issyk-Kul, Keterah, Gossas, and Thiafora Viruses: Nairoviruses Naturally Infecting Bats, Shrews, and Ticks.
Walker PJ(1), Widen SG(2), Firth C(2), Blasdell KR(2), Wood TG(2), Travassos da Rosa AP(2), Guzman H(2), Tesh RB(2), Vasilakis N(2).
Author information:
(1)CSIRO Biosecurity, Australian Animal Health Laboratory, Geelong, Victoria, Australia; Department of Biochemistry and Molecular Biology, The University of Texas Medical Branch, Galveston, Texas; Center for Biodefense and Emerging Infectious Diseases, Department of Pathology, The University of Texas Medical Branch, Galveston, Texas; Center for Tropical Diseases, Institute for Human Infections and Immunity, The University of Texas Medical Branch, Galveston, Texas peter.walker@csiro.au.
(2)CSIRO Biosecurity, Australian Animal Health Laboratory, Geelong, Victoria, Australia; Department of Biochemistry and Molecular Biology, The University of Texas Medical Branch, Galveston, Texas; Center for Biodefense and Emerging Infectious Diseases, Department of Pathology, The University of Texas Medical Branch, Galveston, Texas; Center for Tropical Diseases, Institute for Human Infections and Immunity, The University of Texas Medical Branch, Galveston, Texas.

The genus Nairovirus of arthropod-borne bunyaviruses includes the important emerging human pathogen, Crimean-Congo hemorrhagic fever virus (CCHFV), as well as Nairobi sheep disease virus and many other poorly described viruses isolated from mammals, birds, and ticks. Here, we report genome sequence analysis of six nairoviruses: Thiafora virus (TFAV) that was isolated from a shrew in Senegal; Yogue (YOGV), Kasokero (KKOV), and Gossas (GOSV) viruses isolated from bats in Senegal and Uganda; Issyk-Kul virus (IKV) isolated from bats in Kyrgyzstan; and Keterah virus (KTRV) isolated from ticks infesting a bat in Malaysia. The S, M, and L genome segments of each virus were found to encode proteins corresponding to the nucleoprotein, polyglycoprotein, and polymerase protein of CCHFV. However, as observed in Leopards Hill virus (LPHV) and Erve virus (ERVV), polyglycoproteins encoded in the M segment lack sequences encoding the double-membrane-spanning CCHFV NSm protein. Amino acid sequence identities, complement-fixation tests, and phylogenetic analysis indicated that these viruses cluster into three groups comprising KKOV, YOGV, and LPHV from bats of the suborder Yingochiroptera; KTRV, IKV, and GOSV from bats of the suborder Yangochiroptera; and TFAV and ERVV from shrews (Soricomorpha: Soricidae). This reflects clade-specific host and vector associations that extend across the genus.

78. Prev Vet Med. 2015 Dec 1;122(3):287-97. doi: 10.1016/j.prevetmed.2015.10.011. Epub 2015 Oct 26.
Using participatory epidemiology to investigate management options and relative importance of tick-borne diseases amongst transhumant zebu cattle in Karamoja Region, Uganda.
Byaruhanga C(1), Oosthuizen MC(2), Collins NE(2), Knobel D(2).
Author information:
(1)Department of Veterinary Tropical Diseases, Faculty of Veterinary Science, University of Pretoria, P/Bag X04, Onderstepoort, 0110, South Africa; National Agricultural Research Organisation, P.O. Box 259, Entebbe, Uganda. Electronic address: cbyaruhanga27@yahoo.com.
(2)Department of Veterinary Tropical Diseases, Faculty of Veterinary Science, University of Pretoria, P/Bag X04, Onderstepoort, 0110, South Africa.
A participatory epidemiological (PE) study was conducted with livestock keepers in Moroto and Kotido districts, Karamoja Region, Uganda, between October and December 2013 to determine the management options and relative importance of tick-borne diseases (TBDs) amongst transhumant zebu cattle. Data collection involved 24 focus group discussions (each comprising 8-12 people) in 24 settlement areas (manyattas), key informant interviews (30), direct observation, a review of surveillance data, clinical examination, and laboratory confirmation of cases of TBDs. Methods used in group discussions included semi-structured interviews, simple ranking, pairwise ranking, matrix scoring, proportional piling and participatory mapping. The results of pairwise comparison showed the Ngakarimojong-named diseases, lokit (East Coast fever, ECF), lopid (anaplasmosis), loukoi (contagious bovine pleuropneumonia, CBPP), lokou (heartwater) and lokulam (babesiosis), were considered the most important cattle diseases in Moroto in that order, while ECF, anaplasmosis, trypanosomosis

(ediit), CBPP and nonspecific diarrhoea (loleo) were most important in Kotido. Strong agreement between informant groups (Kendall's coefficient of concordance W=0.568 and 0.682; p<0.001) in pairwise ranking indicated that the diseases were a common problem in selected districts. East Coast fever had the highest median score for incidence (18% [range: 2, 33]) in Moroto, followed by anaplasmosis (17.5% [8,32]) and CBPP (9% [1,21]). Most animals that suffered from ECF, anaplasmosis, heartwater and babesiosis died, as the respective median scores for case fatality rates (CFR) were 89.5% (42, 100), 82.8% (63, 100), 66.7% (20, 100) and 85.7% (0, 100). In Kotido, diseases with high incidence scores were ECF (21% [6,32]), anaplasmosis (17% [10,33]) and trypanosomosis (8% [2,18]). The CFRs for ECF and anaplasmosis were 81.7% (44, 100) and 70.7% (48, 100), respectively. Matrix scoring revealed that disease indicators showed strong agreement (W=0.382-0.659, p<0.05-p<0.001) between informant groups. Inadequate knowledge, poor veterinary services and limited availability of drugs were the main constraints that hindered the control of TBDs. Hand picking of ticks was done by all pastoralists while hand spraying with acaricides was irregular, often determined by availability of drug supplies and money. It was concluded that TBDs, particularly ECF and anaplasmosis were important diseases in this pastoral region. Results from this study may assist in the design of feasible control strategies.

79. Parasit Vectors. 2015 Nov 5;8:571. doi: 10.1186/s13071-015-1191-9.
Contribution of draft cattle to rural livelihoods in a district of southeastern Uganda endemic for bovine parasitic diseases: an economic evaluation.
Okello WO(1), Muhanguzi D(2), MacLeod ET(3), Welburn SC(4), Waiswa C(5), Shaw AP(6).
Author information:
(1)Division of Infection and Pathway Medicine, School of Biomedical Sciences, College of Medicine and Veterinary Medicine, University of Edinburgh, Edinburgh, United Kingdom. W.O.Okello@sms.ed.ac.uk.
(2)Department of Biomolecular and Biolaboratory Sciences, School of Biosecurity, Biotechnical and Laboratory Sciences, College of Veterinary Medicine Animal Resources and Biosecurity, Makerere University, P.O. Box 7062, Kampala, Uganda. luckydenno@covab.mak.ac.ug.
(3)Division of Infection and Pathway Medicine, School of Biomedical Sciences, College of Medicine and Veterinary Medicine, University of Edinburgh, Edinburgh, United Kingdom. Ewan.MacLeod@ed.ac.uk.
(4)Division of Infection and Pathway Medicine, School of Biomedical Sciences, College of Medicine and Veterinary Medicine, University of Edinburgh, Edinburgh, United Kingdom. Sue.Welburn@ed.ac.uk.
(5)Department of Biomolecular and Biolaboratory Sciences, School of Biosecurity, Biotechnical and Laboratory Sciences, College of Veterinary Medicine Animal Resources and Biosecurity, Makerere University, P.O. Box 7062, Kampala, Uganda. cwaiswa@covab.mak.ac.ug.
(6)Avia-GIS, Risschotlei 33, B-2980, Zoersel, Belgium. alex@apconsultants.co.uk.
BACKGROUND: A study was conducted in Tororo District in eastern Uganda to assess the socio-economic contribution of draft cattle to rural livelihoods. The aim of

the study was to empirically quantify the economic value of draft cattle thus contributing to understanding the impact of endemic parasitic diseases of cattle on livestock productivity and subsequently household income, labor and food security.
METHOD: A total of 205 draft cattle keeping households (n = 205) were randomly selected and structured household questionnaires were administered, focusing on work oxen use, productivity, inputs and outputs. The data obtained was analyzed using standard statistical methods and used to calculate the gross margin from the draft cattle enterprise. Secondary data were obtained from focus group discussions and key informant interviews and these were analyzed using Bayesian methods.
RESULTS: The study showed that, apart from being labor saving, the use of animal traction is highly profitable with the gross margin per year from the use of draft cattle amounting to 245 United States dollars per work oxen owning household. The cash obtained from hiring out draft animals was equivalent to nearly a quarter of the average local household's monetary receipts. It also revealed that endemic bovine parasitic diseases such as trypanosomiasis and tick-borne diseases reduced draft cattle output by 20.9 % and potential household income from the use of draft oxen by 32.2 %.
CONCLUSION: The presence of endemic cattle diseases in rural Uganda is adversely affecting the productivity of draft cattle, which in turn affects household income, labor and ultimately food security. This study highlights the contribution of draft cattle to rural livelihoods, thus increasing the expected impact of cost-effective control strategies of endemic production limiting livestock diseases in Uganda.

80. J Biol Dyn. 2016;10:59-70. doi: 10.1080/17513758.2015.1102976.
A mathematical model for Crimean-Congo haemorrhagic fever: tick-borne dynamics with conferred host immunity.
Switkes J(1), Nannyonga B(2), Mugisha JY(2), Nakakawa J(2).
Author information:
(1)a Department of Mathematics and Statistics , California State Polytechnic University , Pomona , CA , USA.
(2)b Department of Mathematics , School of Physical Sciences, College of Natural Sciences, Makerere University , Kampala , Uganda.
Crimean-Congo haemorrhagic fever (CCHF) is a highly contagious tick-borne disease that impacts many countries in parts of Africa, Europe, Asia, and the Middle East. Outbreaks are episodic, but deadly. Due to the highly contagious nature of this disease, suspected cases are taken extremely serious, with very strong control measures implemented almost immediately. It is primarily those living on farms, livestock workers, and medical workers who are at risk. The virus responsible for CCHF is transmitted asymptomatically and transiently to livestock, and symptomatically to humans. The fatality rate in human cases can be very high. The number of methods and directions of viral transmission is large, including tick-to-tick, tick-to-livestock, tick-to-human, livestock-to-tick, livestock-to-human, and human-to-human. We model CCHF using a deterministic system of nonlinear differential equations. This compartment model

allows us to analyse threshold parameters and equilibria describing the magnitude and progression of cases of the disease in a hypothetical outbreak.

81. Ticks Tick Borne Dis. 2016 Mar;7(2):291-7. doi: 10.1016/j.ttbdis.2015.11.004.
Population genetic structure of Theileria parva field isolates from indigenous cattle populations of Uganda.
Muwanika V(1), Kabi F(2), Masembe C(3).
Author information:
(1)Department of Environmental Management, Molecular Genetics Laboratory, College of Agricultural and Environmental Sciences, Makerere University, P.O. Box 7062/7298, Kampala, Uganda.
(2)Department of Environmental Management, Molecular Genetics Laboratory, College of Agricultural and Environmental Sciences, Makerere University, P.O. Box 7062/7298, Kampala, Uganda; National Livestock Resources Research Institute (NaLIRRI), P.O. Box 96, Tororo, Uganda. Electronic address: freddykabi@gmail.com.
(3)Department of Biological Sciences, College of Natural Sciences, Makerere University, Box 7062, Kampala, Uganda.
Theileria parva causes East Coast Fever (ECF) a protozoan infection which manifests as a non-symptomatic syndrome among endemically stable indigenous cattle populations. Knowledge of the current genetic diversity and population structure of T. parva is critical for predicting pathogen evolutionary trends to inform development of effective control strategies. In this study the population genetic structure of 78 field isolates of T. parva from indigenous cattle (Ankole, n=41 and East African shorthorn Zebu (EASZ), n=37) sampled from the different agro ecological zones (AEZs) of Uganda was investigated. A total of eight mini- and micro-satellite markers encompassing the four chromosomes of T. parva were used to genotype the study field isolates. The genetic diversity of the surveyed T. parva populations was observed to range from 0.643±0.55 to 0.663±0.41 among the Central and Western AEZs respectively. The overall Wright's F index showed significant genetic variation between the surveyed T. parva populations based on the different AEZs and indigenous cattle breeds (FST=0.133, p<0.01) and (FST=0.101, p<0.01) respectively. Significant pairwise population genetic differentiations (p<0.05) were observed with FST values ranging from 0.048 to 0.173 between the eastern and northern, eastern and western populations respectively. The principal component analysis (PCA) showed a high level of genetic and geographic sub-structuring among populations. Linkage disequilibrium was observed when populations from all the study AEZs were treated as a single population and when analysed separately. On the overall, the significant genetic diversity and geographic sub-structuring exhibited among the study T. parva isolates has critical implications for ECF control.

82. Vet Parasitol Reg Stud Reports. 2015 Dec;1-2:21-30. doi: 10.1016/j.vprsr.2015.11.001. Epub 2016 Jan 30.
Endemic status of tick-borne infections and tick species diversity among transhumant zebu cattle in Karamoja Region, Uganda: Support for control approaches.

Byaruhanga C(1), Collins NE(2), Knobel D(2), Kabasa W(3), Oosthuizen MC(2).
Author information:
(1)Department of Veterinary Tropical Diseases, Faculty of Veterinary Science, University of Pretoria, Private Bag X04, Onderstepoort 0110, South Africa; National Agricultural Research Organisation, P.O. Box 259, Entebbe, Uganda. Electronic address: cbyaruhanga27@yahoo.com.
(2)Department of Veterinary Tropical Diseases, Faculty of Veterinary Science, University of Pretoria, Private Bag X04, Onderstepoort 0110, South Africa.
(3)College of Veterinary Medicine, Animal Resources and Bio-security, Makerere University, P.O. Box 7062, Kampala, Uganda.
We conducted a study to investigate tick species diversity, seroprevalence of antibodies to Anaplasma marginale and Theileria parva, and the risk factors for these infections among cattle under a transhumant production system in Karamoja Region, Uganda, from November 2013 through January 2014. Twenty herds were randomly selected from 20 purposively-selected superherds. Semi-structured interviews and piling for annual proportion of tick-borne disease (TBD) cases in different age groups, with pastoralist groups, clinical examinations and field observations were employed to obtain information related to the epidemiology of TBDs. Ticks were collected and identified from whole body inspections of at least seven systematically selected cattle in each herd. Concurrently, serum was collected from 397 cattle. Antibodies to A. marginale were detected by MSP-5 competitive inhibition enzyme-linked immunosorbent assay, and to T. parva by indirect fluorescent antibody test. Clinical examinations and informant interviews showed that TBDs affected all age groups of cattle. Tick species that have not been reported in recent studies from other parts of Uganda were collected, namely Amblyomma lepidum, Hyalomma truncatum, Amblyomma gemma, and Rhipicephalus pulchellus. Out of the 10,923 ticks collected, Rhipicephalus appendiculatus (54.4%) was the most abundant species followed by Rhipicephalus (Boophilus) decoloratus (17.7%), Amblyomma variegatum (12%) and A. lepidum (11.6%). Two-thirds of the sampled cattle had moderate (37.3%, 11-50 ticks) to abundant (28.6%, >50 ticks) numbers of ticks. Seroprevalence of A. marginale was high (86.6%, 95% confidence interval [CI] 80.8%-91.8%), while that of T. parva was low (14.6%, 95% CI 7.1%-22.4%). Cattle of 5-12months (18.3%, Odds ratio [OR]=4.1) and 13-24months (30.3%, OR=8.0) were more likely to be seropositive for T. parva than those >24months. For A. marginale, cattle of 13-24months (92.4%, OR=2.7) and >24months (89.7%, OR=2.0) were more likely to be seropositive than those 5-12months. There was a significant difference (p<0.001, OR=6.5) in the proportion of T. parva seropositive animals between Moroto (24.5%) and Kotido districts (4.8%), but not for A. marginale. In conclusion, the low seroprevalence for T. parva, possibly due to limited exposure in calves, may suggest a high likelihood of ECF in cattle. High seroprevalence for A. marginale suggests that a high proportion of cattle were exposed to infection. The findings provide knowledge of epidemiology of TBDs in Karamoja cattle and support for strategic control and improvement of cattle productivity.

83. Parasit Vectors. 2016 Jan 4;9:4. doi: 10.1186/s13071-015-1278-3.
Emergence of multi-acaricide resistant Rhipicephalus ticks and its implication

on chemical tick control in Uganda.
Vudriko P(1)(2)(3), Okwee-Acai J(4), Tayebwa DS(5), Byaruhanga J(6), Kakooza S(7), Wampande E(8)(9), Omara R(10), Muhindo JB(11)(12), Tweyongyere R(13)(14), Owiny DO(15)(16), Hatta T(17)(18), Tsuji N(19)(20), Umemiya-Shirafuji R(21), Xuan X(22), Kanameda M(23)(24), Fujisaki K(25), Suzuki H(26).
Author information:
(1)Department of Veterinary Pharmacy, Clinics and Comparative Medicine, School of Veterinary Medicine and Animal Resources, College of Veterinary Medicine, Animal Resources and Biosecurity, Makerere University, P. O Box 7062, Kampala, Uganda. vpato@covab.mak.ac.ug.
(2)Central Diagnostic Laboratory, College of Veterinary Medicine, Animal Resources and Biosecurity, Makerere University, P. O Box 7062, Kampala, Uganda. vpato@covab.mak.ac.ug.
(3)National Research Center for Protozoan Diseases, Obihiro University of Agriculture and Veterinary Medicine, Inada-Cho, Obihiro, Hokkaido, 080-8555, Japan. vpato@covab.mak.ac.ug.
(4)Department of Veterinary Pharmacy, Clinics and Comparative Medicine, School of Veterinary Medicine and Animal Resources, College of Veterinary Medicine, Animal Resources and Biosecurity, Makerere University, P. O Box 7062, Kampala, Uganda. jokwee@yahoo.co.uk.
(5)Central Diagnostic Laboratory, College of Veterinary Medicine, Animal Resources and Biosecurity, Makerere University, P. O Box 7062, Kampala, Uganda. tayebwa.dickson@gmail.com.
(6)Central Diagnostic Laboratory, College of Veterinary Medicine, Animal Resources and Biosecurity, Makerere University, P. O Box 7062, Kampala, Uganda. josephjbvincent@gmail.com.
(7)Central Diagnostic Laboratory, College of Veterinary Medicine, Animal Resources and Biosecurity, Makerere University, P. O Box 7062, Kampala, Uganda. ksteven310@gmail.com.
(8)Department of Veterinary Pharmacy, Clinics and Comparative Medicine, School of Veterinary Medicine and Animal Resources, College of Veterinary Medicine, Animal Resources and Biosecurity, Makerere University, P. O Box 7062, Kampala, Uganda. ewampande@yahoo.co.uk.
(9)Central Diagnostic Laboratory, College of Veterinary Medicine, Animal Resources and Biosecurity, Makerere University, P. O Box 7062, Kampala, Uganda. ewampande@yahoo.co.uk.
(10)Department of Veterinary Pharmacy, Clinics and Comparative Medicine, School of Veterinary Medicine and Animal Resources, College of Veterinary Medicine, Animal Resources and Biosecurity, Makerere University, P. O Box 7062, Kampala, Uganda. omararoberto@gmail.com.
(11)Department of Veterinary Pharmacy, Clinics and Comparative Medicine, School of Veterinary Medicine and Animal Resources, College of Veterinary Medicine, Animal Resources and Biosecurity, Makerere University, P. O Box 7062, Kampala, Uganda. mjeannebukeka@gmail.com.
(12)Drug Information Department, National Drug Authority, P. O. Box 23096, Kampala, Uganda. mjeannebukeka@gmail.com.
(13)Department of Veterinary Pharmacy, Clinics and Comparative Medicine, School

of Veterinary Medicine and Animal Resources, College of Veterinary Medicine, Animal Resources and Biosecurity, Makerere University, P. O Box 7062, Kampala, Uganda. rtweyongyere@covab.mak.ac.ug.
(14)Central Diagnostic Laboratory, College of Veterinary Medicine, Animal Resources and Biosecurity, Makerere University, P. O Box 7062, Kampala, Uganda. rtweyongyere@covab.mak.ac.ug.
(15)Department of Veterinary Pharmacy, Clinics and Comparative Medicine, School of Veterinary Medicine and Animal Resources, College of Veterinary Medicine, Animal Resources and Biosecurity, Makerere University, P. O Box 7062, Kampala, Uganda. dowiny@covab.mak.ac.ug.
(16)Directorate of Research and Graduate Training, Makerere University, P.O Box 7062, Kampala, Uganda. dowiny@covab.mak.ac.ug.
(17)Laboratory of Parasitic Diseases, National Institute of Animal Health, National Agricultural and Food Research Organization, 3-1-5 Kannondai, Tsukuba, Ibaraki, 305-0856, Japan. htakeshi@med.kitasato-u.ac.jp.
(18)Present Address: Department of Parasitology, Kitasato University School of Medicine, Kitasato, Minami-ku, Sagamihara, Kanagawa, 252-0374, Japan. htakeshi@med.kitasato-u.ac.jp.
(19)Laboratory of Parasitic Diseases, National Institute of Animal Health, National Agricultural and Food Research Organization, 3-1-5 Kannondai, Tsukuba, Ibaraki, 305-0856, Japan. tsujin@med.kitasato-u.ac.jp.
(20)Present Address: Department of Parasitology, Kitasato University School of Medicine, Kitasato, Minami-ku, Sagamihara, Kanagawa, 252-0374, Japan. tsujin@med.kitasato-u.ac.jp.
(21)National Research Center for Protozoan Diseases, Obihiro University of Agriculture and Veterinary Medicine, Inada-Cho, Obihiro, Hokkaido, 080-8555, Japan. umemiya@obihiro.ac.jp.
(22)National Research Center for Protozoan Diseases, Obihiro University of Agriculture and Veterinary Medicine, Inada-Cho, Obihiro, Hokkaido, 080-8555, Japan. gen@obihiro.ac.jp.
(23)Central Diagnostic Laboratory, College of Veterinary Medicine, Animal Resources and Biosecurity, Makerere University, P. O Box 7062, Kampala, Uganda. harukana@koalanet.ne.jp.
(24)Present Address: Japan International Cooperation Agency, Nibancho Center Building, 5-25, Nibancho, Chiyoda-ku, Tokyo, 108-8012, Japan. harukana@koalanet.ne.jp.
(25)Laboratory of Parasitic Diseases, National Institute of Animal Health, National Agricultural and Food Research Organization, 3-1-5 Kannondai, Tsukuba, Ibaraki, 305-0856, Japan. acarikf@nifty.com.
(26)National Research Center for Protozoan Diseases, Obihiro University of Agriculture and Veterinary Medicine, Inada-Cho, Obihiro, Hokkaido, 080-8555, Japan. hisuzuki@obihiro.ac.jp.
BACKGROUND: Acaricide failure has been on the rise in the western and central cattle corridor of Uganda. In this study, we identified the tick species associated with acaricide failure and determined their susceptibility to various acaricide molecules used for tick control in Uganda.
METHODS: In this cross sectional study, tick samples were collected and

identified to species level from 54 purposively selected farms (from 17 districts) that mostly had a history of acaricide failure. Larval packet test was used to screen 31 tick populations from 30 farms for susceptibility at discriminating dose (DD) and 2 × DD of five panels of commercial acaricide molecules belonging to the following classes; amidine, synthetic pyrethroid (SP), organophosphate (OP) and OP-SP co-formulations (COF). Resistance was assessed based on World Health Organization criteria for screening insecticide resistance.
RESULTS: Of the 1357 ticks identified, Rhipicephalus (Rhipicephalus) appendiculatus and Rhipicephalus (Boophilus) decoloratus were the major (95.6%) tick species in farms sampled. Resistance against SP was detected in 90.0% (27/30) of the tick populations tested. Worryingly, 60.0% (18/30) and 63.0% (19/30) of the above ticks were super resistant (0% mortality) against 2 × DD cypermethrin and deltamethrin, respectively. Resistance was also detected against COF (43.3%), OP chlorfenvinphos (13.3%) and amitraz (12.9%). In two years, 74.1% (20/27) of the farms had used two to three acaricide molecules, and 55.6% (15/27) rotated the molecules wrongly. Multi-acaricide resistance (at least 2 molecules) was detected in 55.2% (16/29) of the resistant Rhipicephalus ticks and significantly associated with R. decoloratus ($p = 0.0133$), use of both SP and COF in the last 2 years ($p < 0.001$) and Kiruhura district ($p = 0.0339$). Despite emergence of amitraz resistance in the greater Bushenyi area, it was the most efficacious molecule against SP and COF resistant ticks.
CONCLUSION: This study is the first to report emergence of super SP resistant and multi-acaricide resistant Rhipicephalus ticks in Uganda. Amitraz was the best acaricide against SP and COF resistant ticks. However, in the absence of technical interventions, farmer-led solutions aimed at troubleshooting for efficacy of multitude of acaricides at their disposal are expected to potentially cause negative collateral effects on future chemical tick control options, animal welfare and public health.

84. Parasit Vectors. 2016 Mar 5;9:128. doi: 10.1186/s13071-016-1390-z.
No evidence for the involvement of the argasid tick Ornithodoros faini in the enzootic maintenance of marburgvirus within Egyptian rousette bats Rousettus aegyptiacus.
Schuh AJ(1), Amman BR(2), Apanaskevich DA(3), Sealy TK(4), Nichol ST(5), Towner JS(6).
Author information:
(1)Viral Special Pathogens Branch, Division of High-Consequence Pathogens and Pathology, Centers for Disease Control and Prevention, Atlanta, GA, USA. wuc2@cdc.gov.
(2)Viral Special Pathogens Branch, Division of High-Consequence Pathogens and Pathology, Centers for Disease Control and Prevention, Atlanta, GA, USA. cxx1@cdc.gov.
(3)United States National Tick Collection, the James H. Oliver, Jr. Institute for Coastal Plain Science, Georgia Southern University, Statesboro, GA, USA. dapanaskevich@georgiasouthern.edu.
(4)Viral Special Pathogens Branch, Division of High-Consequence Pathogens and

Pathology, Centers for Disease Control and Prevention, Atlanta, GA, USA.
tss3@cdc.gov.
(5)Viral Special Pathogens Branch, Division of High-Consequence Pathogens and
Pathology, Centers for Disease Control and Prevention, Atlanta, GA, USA.
stn1@cdc.gov.
(6)Viral Special Pathogens Branch, Division of High-Consequence Pathogens and
Pathology, Centers for Disease Control and Prevention, Atlanta, GA, USA.
jit8@cdc.gov.
BACKGROUND: The cave-dwelling Egyptian rousette bat (ERB; Rousettus aegyptiacus) was recently identified as a natural reservoir host of marburgviruses. However, the mechanisms of transmission for the enzootic maintenance of marburgviruses within ERBs are unclear. Previous ecological investigations of large ERB colonies inhabiting Python Cave and Kitaka Mine, Uganda revealed that argasid ticks (Ornithodoros faini) are hematophagous ectoparasites of ERBs. Yet, their potential role as transmission vectors for marburgvirus has not been sufficiently assessed.
FINDINGS: In the present study, 3,125 O. faini were collected during April 2013 from the rock crevices of Python Cave, Uganda. None of the ticks tested positive for marburgvirus-specific RNA by Q-RT-PCR. The probability of failure to detect marburgvirus at a conservative prevalence of 0.1 % was 0.05.
CONCLUSIONS: The absence of marburgvirus RNA in O. faini suggests they do not play a significant role in the transmission and enzootic maintenance of marburgvirus within their natural reservoir host.

85. Vet Parasitol Reg Stud Reports. 2016 Jun;3-4:27-35. doi:
10.1016/j.vprsr.2016.06.004. Epub 2016 Jun 29.
Molecular investigation of tick-borne haemoparasite infections among transhumant zebu cattle in Karamoja Region, Uganda.
Byaruhanga C(1), Collins NE(2), Knobel D(2), Chaisi ME(2), Vorster I(2), Steyn HC(3), Oosthuizen MC(2).
Author information:
(1)Department of Veterinary Tropical Diseases, Faculty of Veterinary Science,
University of Pretoria, Private Bag X04, Onderstepoort, 0110, South Africa;
National Agricultural Research Organisation, P.O. Box 259, Entebbe, Uganda.
Electronic address: cbyaruhanga27@yahoo.com.
(2)Department of Veterinary Tropical Diseases, Faculty of Veterinary Science,
University of Pretoria, Private Bag X04, Onderstepoort, 0110, South Africa.
(3)Onderstepoort Veterinary Institute, Private Bag X05, Onderstepoort, 0110,
South Africa.
Tick-borne diseases (TBDs) are a major constraint to cattle production in pastoral areas in Africa. Although information on tick-borne infections is important to prioritise control approaches, it is limited for transhumant zebu cattle in Karamoja, Uganda. We conducted a study to determine the occurrence and level of tick-borne infections among cattle in Karamoja Region. A total of 240 cattle were selected for blood collection using systematic sampling in 20 randomly-selected herds in two districts. The hypervariable V4 region of the 18S rRNA gene for Theileria/Babesia and the V1 region of the 16S rRNA gene for

Ehrlichia/Anaplasma were amplified and hybridised to genus- and species-specific oligonucleotide probes on a reverse line blot (RLB) membrane. A duplex quantitative real-time polymerase chain reaction (qPCR) assay based on msp1β and groEL genes was used for the detection of Anaplasma marginale and A. centrale, while monoplex qPCR assays were used for the detection of Ehrlichia ruminantium (226bp fragment of the pCS20 region) and Theileria parva (18S rRNA gene). The RLB hybridisation assay demonstrated the presence of tick-borne haemoparasites in all but one sample (99.6%), mostly as mixed infections (97.5%). The most frequently detected species were Theileria mutans (88.3%, 95% confidence interval: 84.6-91.7%), A. marginale (73.8%: 68.3-78.8%), Theileria velifera (71.3%: 65.8-76.7%) and Anaplasma sp. Omatjenne (63.3%: 57.5-68.8%). Other virulent pathogens, namely Babesia bigemina (5.0%) and T. parva (2.9%), were also detected with RLB, but not E. ruminantium. The proportions of qPCR positive samples were 82.9% (A. marginale), 12.1% (A. centrale), 3.3% (T. parva), and 1.7% (E. ruminantium). The full-length 18S rRNA genes from 6 out of 47 samples that were positive on RLB for the Babesia genus-specific probe and not for any of the Babesia species-specific probes were amplified, cloned and sequenced. The sequences were used to construct phylogenetic trees. Variations (5 to 9 nucleotides) in the 18S rRNA gene sequences of B. bigemina were identified, when compared with B. bigemina sequences from other parts of the world. Three nucleotide differences in the B. bigemina probe region may explain the failure of the RLB hybridisation assay to detect B. bigemina in some samples. T. mutans and B. bigemina sequences grouped in separate clades from previously published sequences. In conclusion, this study demonstrated high and widespread occurrence, and sequence variation of tick-borne haemoparasites among cattle in the pastoral area of Karamoja, which is useful for diagnosis and control of TBDs.

86. Virol J. 2016 Jun 8;13:95. doi: 10.1186/s12985-016-0551-1.
Nucleotide composition of the Zika virus RNA genome and its codon usage.
van Hemert F(1), Berkhout B(2).
Author information:
(1)Laboratory of Experimental Virology, Department of Medical Microbiology, Center for Infection and Immunity Amsterdam (CINIMA), Academic Medical Center, University of Amsterdam, Meibergdreef 15, 1105 AZ, Amsterdam, The Netherlands.
(2)Laboratory of Experimental Virology, Department of Medical Microbiology, Center for Infection and Immunity Amsterdam (CINIMA), Academic Medical Center, University of Amsterdam, Meibergdreef 15, 1105 AZ, Amsterdam, The Netherlands. b.berkhout@amc.uva.nl.
BACKGROUND: RNA viruses have genomes with a distinct nucleotide composition and codon usage. We present the global characteristics of the RNA genome of Zika virus (ZIKV), an emerging pathogen within the Flavivirus genus. ZIKV was first isolated in 1947 in Uganda, caused a widespread epidemic in South and Central America and the Caribbean in 2015 and has recently been associated with microcephaly in newborns.
METHODS: The nearly 11 kb positive-stranded RNA genome of ZIKV was analyzed for its nucleotide composition, also in the context of the folded RNA molecule.

Nucleotide trends were investigated along the genome length by skew analyses and we analyzed the codons used for translation of the ZIKV proteins.
RESULTS: ZIKV RNA has a biased nucleotide composition in being purine-rich and pyrimidine-poor. This preference for purines is a general characteristic of the mosquito-borne and tick-borne flaviviruses. The virus-specific nucleotide bias is further enriched in the unpaired, single-stranded regions of the structured ZIKV RNA genome, thus further imposing this ZIKV-specific signature. The codons used for translation of the ZIKV proteins is also unusual, but we show that it is the underlying bias in nucleotide composition of the viral RNA that largely dictates these codon preferences.
CONCLUSIONS: The ZIKV RNA genome has a biased nucleotide composition that dictates the codon usage of this flavivirus. We discuss the evolutionary scenarios and molecular mechanisms that may be responsible for these distinctive ZIKV RNA genome features.

87. Front Public Health. 2016 Jun 28;4:137. doi: 10.3389/fpubh.2016.00137.
Awareness and Need for Knowledge of Health and Safety among Dairy Farmers Interviewed in Uganda.
Lunner-Kolstrup C(1), Ssali TK(2).
Author information:
(1)Department of Work Science, Business Economics and Environmental Psychology (AEM), Swedish University of Agricultural Sciences (SLU) , Alnarp , Sweden.
(2)Faculty of Agriculture, Uganda Martyrs University (UMU) , Kampala , Uganda.
INTRODUCTION: Safe working conditions are essential for healthy living and for ensuring food security among farmers and farm communities in developing countries. There is limited research on this topic, and documentation is essential to understand and change patterns of human health and safety.
METHODS: In May 2014, six male and female farmers on four dairy farms in Uganda and a female veterinarian were interviewed about their awareness and attitudes to agricultural risk factors, health, and safety. In addition, transect walks were conducted on the four dairy farms.
RESULTS: The dairy farmers reported health and safety concerns, e.g., diarrhea, coughs, fever, cuts while using machetes in plantations, bruises when handling animals, and dizziness and poisoning symptoms from using different agrochemicals, and considered these an occupational hazard. The most important topic mentioned was the use of agrochemicals and drugs on livestock. The farmers spray their animals with insecticides to prevent ticks, lice, tsetse flies, and other biting nuisance flies, using a backpack or hand sprayer. Spraying is conducted without personal protection equipment, which is considered too expensive and difficult to obtain. The farmers reported that they usually feel dizzy, vomit, and have pain and a burning feeling in their face and eyes after spraying. The symptoms are sometimes so severe that they require treatment. In such cases, the farmers buy medication without a prescription at the local drugstore, where the storekeeper often has limited or no knowledge of agrochemicals or drugs except for dosage. Agricultural health and safety training in the region is non-existent, and the farmers expressed a need and desire for improvements in this area.

CONCLUSION: The level of knowledge and awareness of agricultural health and safety risks, disease, and injury prevention among the Ugandan dairy farmers interviewed was low. The farmers mentioned few agriculture-related complaints, injuries, or diseases except poisoning from using agrochemicals. Training on health and safety in Ugandan agriculture is urgently needed.

88. Trop Anim Health Prod. 2016 Dec;48(8):1525-1532. doi: 10.1007/s11250-016-1123-5. Epub 2016 Aug 18.
Cattle management practices and milk production on mixed smallholder organic pineapple farms in Central Uganda.
Nalubwama S(1)(2), Kabi F(3), Vaarst M(4), Smolders G(5), Kiggundu M(3).
Author information:
(1)Department of Livestock and Industrial Resources, Makerere University, P.O. Box 7062, Kampala, Uganda. drnalubwama@gmail.com.
(2)Department of Agricultural Production, Makerere University, P.O. Box 7062, Kampala, Uganda. drnalubwama@gmail.com.
(3)Department of Agricultural Production, Makerere University, P.O. Box 7062, Kampala, Uganda.
(4)Department of Animal Science, Aarhus University, P.O. Box 50, DK-8830, Tjele, Denmark.
(5)OrgANIMprove, Karveel 13-03, 8231 AT, Lelystad, The Netherlands.
A longitudinal study to assess animal management practices and milk production was conducted for a period of 12 months on 30 smallholder farms keeping dairy cattle and certified organic pineapple production in Luwero and Kayunga districts, based on questionnaire and on-farm collected data. Farm sizes were 9.3 ± 6.7 acres in tethering system and 4.3 ± 2.6 acres in zero-grazing. Fifty-four percent of the zero-grazing herds had animal housing facilities. All farmers in tethering system kept cows on earthen floors and calves without bedding. Hygiene level in existing farms was low. Majority of calves were fed once a day by restricted suckling (77 %). Seventy-four percent of tethered cows were only fed on natural grass, while cows under zero-grazing system had a more diversified diet but with 82 % feeding mainly Napier grass. Most farms (87 %) used bulls for breeding. Milk production was higher ($P < 0.05$) in zero-grazing (6.5 L/cow/day) than tethering system, and higher ($P < 0.05$) for Holstein-Friesian crossbred cows (5.2 L/cow/day) than local breed cows (2.6 L/cow/day). Less than 1 L of milk per farm per day on average was sold. Disease treatments were exclusively for helminths, East Coast fever, and trypanasomiasis. Spraying of ticks and deworming were important control measures of vector-borne diseases. There is potential to develop alternative feed resources for dairy cattle and biorational pesticides for control and treatment of vector-borne diseases.

89. Cell Host Microbe. 2016 Sep 14;20(3):357-367. doi: 10.1016/j.chom.2016.07.011.
A Multicomponent Animal Virus Isolated from Mosquitoes.
Ladner JT(1), Wiley MR(2), Beitzel B(2), Auguste AJ(3), Dupuis AP 2nd(4), Lindquist ME(5), Sibley SD(6), Kota KP(7), Fetterer D(8), Eastwood G(4), Kimmel D(2), Prieto K(2), Guzman H(3), Aliota MT(4), Reyes D(2), Brueggemann EE(7), St

John L(2), Hyeroba D(9), Lauck M(10), Friedrich TC(11), O'Connor DH(10), Gestole MC(2), Cazares LH(12), Popov VL(3), Castro-Llanos F(13), Kochel TJ(13), Kenny T(7), White B(2), Ward MD(7), Loaiza JR(14), Goldberg TL(15), Weaver SC(3), Kramer LD(16), Tesh RB(3), Palacios G(17).
Author information:
(1)Center for Genome Sciences, U.S. Army Medical Research Institute of Infectious Diseases, Fort Detrick, MD 21702, USA. Electronic address: jason.t.ladner.ctr@mail.mil.
(2)Center for Genome Sciences, U.S. Army Medical Research Institute of Infectious Diseases, Fort Detrick, MD 21702, USA.
(3)Institute for Human Infections and Immunity, Departments of Pathology, Microbiology & Immunology, and Center for Biodefense and Emerging Infectious Diseases, University of Texas Medical Branch, Galveston, TX 77555, USA.
(4)Arbovirus Laboratory, Wadsworth Center, New York State Department of Health, Albany, NY 12159, USA.
(5)Virology Division, U.S. Army Medical Research Institute of Infectious Diseases, Fort Detrick, MD 21702, USA.
(6)Department of Pathobiological Sciences, University of Wisconsin-Madison, Madison, WI 53706, USA.
(7)Molecular and Translational Sciences Division, U.S. Army Medical Research Institute of Infectious Diseases, Fort Detrick, MD 21702, USA.
(8)Research Support Division, U.S. Army Medical Research Institute of Infectious Diseases, Fort Detrick, MD 21702, USA.
(9)Makerere University, Kampala, Uganda.
(10)Wisconsin National Primate Research Center, Madison, WI 53715, USA; Department of Pathology and Laboratory Medicine, University of Wisconsin School of Medicine and Public Health, Madison, WI 53706, USA.
(11)Department of Pathobiological Sciences, University of Wisconsin-Madison, Madison, WI 53706, USA; Wisconsin National Primate Research Center, Madison, WI 53715, USA.
(12)Molecular and Translational Sciences Division, U.S. Army Medical Research Institute of Infectious Diseases, Fort Detrick, MD 21702, USA; Henry M. Jackson Foundation, Bethesda, MD 20817, USA; DoD Biotechnology High Performance Computing Software Applications Institute, Frederick, MD 21702, USA; Telemedicine and Advanced Technology Research Center, U.S. Army Medical Research and Materiel Command, Fort Detrick, MD 21702, USA.
(13)U.S. Naval Medical Research Unit No. 6, Lima, Peru.
(14)Centro de Biodiversidad y Descubrimiento de Drogas, Instituto de Investigaciones Científicas y Servicios de Alta Tecnología, Ciudad de Panamá, Panamá.
(15)Department of Pathobiological Sciences, University of Wisconsin-Madison, Madison, WI 53706, USA; Makerere University, Kampala, Uganda; Wisconsin National Primate Research Center, Madison, WI 53715, USA.
(16)Arbovirus Laboratory, Wadsworth Center, New York State Department of Health, Albany, NY 12159, USA; School of Public Health, State University of New York at Albany, One University Place Rensselaer, East Greenbush, NY 12144, USA.
(17)Center for Genome Sciences, U.S. Army Medical Research Institute of

Infectious Diseases, Fort Detrick, MD 21702, USA. Electronic address:
gustavo.f.palacios.ctr@mail.mil.
Comment in
Nat Rev Microbiol. 2016 Oct;14(10):606-7.
Cell Host Microbe. 2016 Sep 14;20(3):279-280.
RNA viruses exhibit a variety of genome organization strategies, including multicomponent genomes in which each segment is packaged separately. Although multicomponent genomes are common among viruses infecting plants and fungi, their prevalence among those infecting animals remains unclear. We characterize a multicomponent RNA virus isolated from mosquitoes, designated Guaico Culex virus (GCXV). GCXV belongs to a diverse clade of segmented viruses (Jingmenvirus) related to the prototypically unsegmented Flaviviridae. The GCXV genome comprises five segments, each of which appears to be separately packaged. The smallest segment is not required for replication, and its presence is variable in natural infections. We also describe a variant of Jingmen tick virus, another Jingmenvirus, sequenced from a Ugandan red colobus monkey, thus expanding the host range of this segmented and likely multicomponent virus group. Collectively, this study provides evidence for the existence of multicomponent animal viruses and their potential relevance for animal and human health.

90. Emerg Infect Dis. 2016 Oct;22(10):1848-9. doi: 10.3201/eid2210.160650.
African Tick-Bite Fever in Traveler Returning to Slovenia from Uganda.
Bogovic P, Lotric-Furlan S, Korva M, Avsic-Zupanc T.

91. PLoS Negl Trop Dis. 2016 Oct 11;10(10):e0005056. doi:
10.1371/journal.pntd.0005056. eCollection 2016 Oct.
Successful Treatment of Severe Tungiasis in Pigs Using a Topical Aerosol Containing Chlorfenvinphos, Dichlorphos and Gentian Violet.
Mutebi F(1), von Samson-Himmelstjerna G(2), Feldmeier H(3), Waiswa C(1), Bukeka Muhindo J(4), Krücken J(2).
Author information:
(1)School of Veterinary Medicine and Animal Resources, College of Veterinary Medicine, Animal Resources and Bio-security, Makerere University, Kampala, Uganda.
(2)Institute for Parasitology and Tropical Veterinary Medicine, Freie Universität Berlin, Berlin, Germany.
(3)Institute of Microbiology and Hygiene, Charité University Medicine, Berlin, Campus Benjamin Franklin, Berlin, Germany.
(4)National Drug Authority, Kampala, Uganda.
BACKGROUND: In endemic communities, zoonotic tungiasis, a severe skin disease caused by penetrating female sand fleas, is a public health hazard causing significant human and animal morbidity. No validated drugs are currently available for treatment of animal tungiasis. Due to the reservoir in domestic animals, integrated management of human and animal tungiasis is required to avert its negative effects.
METHODS AND PRINCIPAL FINDINGS: A topical aerosol containing chlorfenvinphos

4.8%, dichlorphos 0.75% and gentian violet 0.145% licensed to treat tick infestations, myiasis and wound sepsis in animals in the study area, was tested for its potential tungicidal effects in a randomized controlled field trial against pig tungiasis in rural Uganda. Animals with at least one embedded flea were randomized in a treatment (n = 29) and a control (n = 26) group. One week after treatment, 58.6% of the treated pigs did not show any viable flea lesion whereas all control pigs had at least one viable lesion. After treatment the number of viable lesions (treated median = 0, overall range = 0-18 vs. control median = 11.5, range = 1-180) and the severity score for estimating acute pathology in pig tungiasis (treated median = 1, range = 0-3.5 vs. control median = 7, range = 0-25) were significantly lower in treated than in control pigs (p < 0.001). In the treatment group the median number of viable flea lesions decreased from 8.5 to 0 (p < 0.001). Similarly, the median acute severity score dropped from 6 to 1 (p < 0.001). Every pig in the treatment group showed a decrease in the number of viable fleas and tungiasis-associated acute morbidity while medians for both increased in the control group.
CONCLUSIONS: The study demonstrates that a topical treatment based on chlorfenvinphos, dichlorphos and gentian violet is highly effective against pig tungiasis. Due to its simplicity, the new approach can be used for the treatment of individual animals as well as in mass campaigns.

92. Transbound Emerg Dis. 2016 Dec;63(6):e197-e210. doi: 10.1111/tbed.12332. Epub
The Prevalence of Brucellosis in Cattle, Goats and Humans in Rural Uganda: A Comparative Study.
Miller R(1), Nakavuma JL(2), Ssajjakambwe P(2), Vudriko P(2), Musisi N(2), Kaneene JB(3).
Author information:
(1)Center for Comparative Epidemiology, East Lansing, MI, 48824, USA.
(2)College of Veterinary Medicine, Animal Resources and Biosecurity, Makerere University, Kampala, Uganda.
(3)Center for Comparative Epidemiology, East Lansing, MI, 48824, USA. kaneenej@cvm.msu.edu.
A cross-sectional study was conducted to determine the presence of brucellosis in cattle, goats and humans in farms from south-western Uganda and identify risk factors associated with brucellosis in these three host groups. Data and serum samples were collected from 768 cattle, 315 goats and 236 humans, with 635 samples of bovine milk, from 70 farms in two different study areas in south-western Uganda. Sera from livestock were tested with the Rose Bengal Plate test, using B. abortus and B. melitensis antigens, and human sera were tested with a commercial IgG/IgM lateral flow assay. Milk samples were tested using the OIE-approved milk ring test. Screening tests for brucellosis were positive in 14% of cattle serum, 29% of bovine milk, 17% of goat serum and 11% of human serum samples. There were significant differences in the test prevalence of brucellosis by study site, with levels higher in the study area near Lake Mburo National Park than in the study area near Queen Elizabeth National Park. Multivariable regression models identified risk factors associated with increasing test positivity at the individual and farm levels for cattle, goats

and humans. Positive associations were seen between increasing seropositivity of brucellosis in goats, cattle and humans. Results of multivariable analyses suggest that improvements in farm biosecurity and hygiene may reduce the risk of brucellosis on the farm and suggest a role for ticks in bovine brucellosis. Although cattle are the focus of brucellosis control in Uganda, the significant associations between seropositivity in humans and seropositivity in goats suggest that brucellosis in goats may be an important contributor to the epidemiology of the disease on the farm.

93. Res Vet Sci. 2016 Dec;109:1-9. doi: 10.1016/j.rvsc.2016.09.001. Epub 2016 Sep 9.
Application of ethnobotanical repellents and acaricides in prevention, control and management of livestock ticks: A review.
Pavela R(1), Canale A(2), Mehlhorn H(3), Benelli G(4).
Author information:
(1)Crop Research Institute, Drnovska 507, 161 06 Prague 6, Czech Republic.
(2)Insect Behaviour Group, Department of Agriculture, Food and Environment, University of Pisa, via del Borghetto 80, 56124 Pisa, Italy.
(3)Department of Parasitology, Heinrich Heine University, Düsseldorf, Germany.
(4)Insect Behaviour Group, Department of Agriculture, Food and Environment, University of Pisa, via del Borghetto 80, 56124 Pisa, Italy. Electronic address: benelli.giovanni@gmail.com.
Ticks transmit at least the same number or even more pathogens than any other group of blood-feeding arthropods worldwide affecting humans and animals. The eco-friendly control and management of tick vectors in a constantly changing environment is a crucial challenge. Besides the development of vaccines against ticks, IPM practices aimed at reducing tick interactions with livestock, emerging pheromone-based control tools, and few biological control agents, the extensive employment of acaricides and tick repellents still remain the most effective and ready-to-use strategies. However, the former is limited by the development of growing resistances as well as environmental concerns. Exploiting plants and plant products as sources of effective tick repellents and acaricides represents a promising strategy. In this scenario, the preservation of ethnobotanical information on repellent and acaricidal potential of plants is crucial. Here, we evaluated relevant information published in recent years, focused on plants used as repellents and acaricides against tick vectors in different regions worldwide. We selected a total of 238 plant species, which are traditionally used against ticks by native and local communities of Africa (Kenya, Uganda, Zimbabwe, South Africa), Europe (Serbia, Macedonia, Romania), Asia (Pakistan, India) and America (Brazil, Canada), from 56 families. However, only 7 families (i.e. Asteraceae, Euphorbiaceae, Fabaceae, Lamiaceae, Meliaceae, Apocynaceae and Solanaceae) represent the major quote (46%) of all plant species. We evaluated the differences in acaricidal and repellent efficacy of different formulations used. In the final section, implications arising from the surveyed anti-tick ethnobotanical knowledge and challenges for its future are discussed.

94. Microbiol Mol Biol Rev. 2017 Feb 8;81(1):e00055-16. doi: 10.1128/MMBR.00055-16.

The Antigenic Structure of Zika Virus and Its Relation to Other Flaviviruses: Implications for Infection and Immunoprophylaxis.
Heinz FX(1), Stiasny K(2).
Author information:
(1)Department of Virology, Medical University of Vienna, Vienna, Austria franz.x.heinz@meduniwien.ac.at.
(2)Department of Virology, Medical University of Vienna, Vienna, Austria.
Zika virus was discovered ~70 years ago in Uganda and maintained a low profile as a human disease agent in Africa and Asia. Only recently has it caused explosive outbreaks in previously unaffected regions, first in Oceania and then in the Americas since 2015. Of special concern is the newly identified link between congenital malformations (especially microcephaly) and Zika virus infections during pregnancy. At present, it is unclear whether Zika virus changed its pathogenicity or whether the huge number of infections allowed the recognition of a previously cryptic pathogenic property. The purpose of this review is to discuss recent data on the molecular antigenic structure of Zika virus in the context of antibody-mediated neutralization and antibody-dependent enhancement (ADE) of infection, a phenomenon that has been implicated in the development of severe disease caused by the related dengue viruses. Emphasis is given to epitopes of antibodies that potently neutralize Zika virus and also to epitopes that provide antigenic links to other important human-pathogenic flaviviruses such as dengue, yellow fever, West Nile, Japanese encephalitis, and tick-borne encephalitis viruses. The antigenic cross talk between Zika and dengue viruses appears to be of special importance, since they cocirculate in many regions of endemicity and sequential infections are likely to occur frequently. New insights into the molecular antigenic structure of Zika virus and flaviviruses in general have provided the foundation for great progress made in developing Zika virus vaccines and antibodies for passive immunization.

95. Ticks Tick Borne Dis. 2017 Jun;8(4):537-539. doi: 10.1016/j.ttbdis.2017.02.016.
Splenic dysfunction from celiac disease resulting in severe babesiosis.
O'Connell S(1), Lyons C(2), Abdou M(3), Patowary R(3), Aslam S(3), Kinsella N(4), Zintl A(5), Hunfeld KP(6), Wormser GP(7), Gray J(8), Merry C(9), Alizadeh H(10).
Author information:
(1)Department of Infectious Diseases, St. James's Hospital, Dublin 8, Ireland. Electronic address: soconnell@stjames.ie.
(2)Department of Surgery, Letterkenny University Hospital, Letterkenny, Ireland.
(3)Department of Haematology, Letterkenny University Hospital, Letterkenny, Ireland.
(4)Department of Haematology, St. James's Hospital, Dublin, Ireland.
(5)School of Veterinary Medicine, University College Dublin, Dublin, Ireland.
(6)Institute for Laboratory Medicine, Microbiology & Infection Control, Northwest Medical Centre, Academic Teaching Hospital, Goethe-University, Frankfurt, Germany.
(7)Division of Infectious Diseases, Department of Medicine, New York Medical College, Valhalla, NY, USA.

(8)UCD School of Biology and Environmental Science, University College Dublin, Dublin 4, Ireland.
(9)Department of Infectious Diseases, St. James's Hospital, Dublin 8, Ireland; Northwestern Memorial University Chicago, USA; Makerere University Uganda, Uganda.
(10)Department of Haematology, Letterkenny University Hospital, Letterkenny, Ireland; Pecs University, Faculty of General Medicine, Pecs, Hungary.
We describe a 79-year-old Irish man who, because he had hyposplenism and splenic atrophy due to adult celiac disease, became critically ill from a severe Babesia divergens infection. Greater awareness of the possible consequences of splenic dysfunction from adult celiac disease, such as serious pneumococcal infections and babesiosis, is warranted.

96. Ticks Tick Borne Dis. 2018 Feb;9(2):254-265. doi: 10.1016/j.ttbdis.2017.09.011.
Evidence-based tick acaricide resistance intervention strategy in Uganda: Concept and feedback of farmers and stakeholders.
Vudriko P(1), Okwee-Acai J(2), Byaruhanga J(3), Tayebwa DS(4), Omara R(5), Muhindo JB(6), Lagu C(7), Umemiya-Shirafuji R(8), Xuan X(8), Suzuki H(9).
Author information:
(1)Research Center for Ticks and Tick-borne Diseases Control (RTC), Department of Veterinary Pharmacy, Clinical and Comparative Medicine, College of Veterinary Medicine, Animal Resources and Biosecurity (COVAB), Makerere University, P. O Box 7062, Kampala, Uganda; College of Veterinary Medicine, Animal Resources and Biosecurity (COVAB), Makerere University, P. O Box 7062, Kampala, Uganda; National Research Center for Protozoan Diseases (NRCPD), Obihiro University of Agriculture and Veterinary Medicine, Inada-Cho, Obihiro 080-8555, Hokkaido, Japan.
(2)Research Center for Ticks and Tick-borne Diseases Control (RTC), Department of Veterinary Pharmacy, Clinical and Comparative Medicine, College of Veterinary Medicine, Animal Resources and Biosecurity (COVAB), Makerere University, P. O Box 7062, Kampala, Uganda; College of Veterinary Medicine, Animal Resources and Biosecurity (COVAB), Makerere University, P. O Box 7062, Kampala, Uganda.
(3)Research Center for Ticks and Tick-borne Diseases Control (RTC), Department of Veterinary Pharmacy, Clinical and Comparative Medicine, College of Veterinary Medicine, Animal Resources and Biosecurity (COVAB), Makerere University, P. O Box 7062, Kampala, Uganda.
(4)Research Center for Ticks and Tick-borne Diseases Control (RTC), Department of Veterinary Pharmacy, Clinical and Comparative Medicine, College of Veterinary Medicine, Animal Resources and Biosecurity (COVAB), Makerere University, P. O Box 7062, Kampala, Uganda; National Research Center for Protozoan Diseases (NRCPD), Obihiro University of Agriculture and Veterinary Medicine, Inada-Cho, Obihiro 080-8555, Hokkaido, Japan.
(5)College of Veterinary Medicine, Animal Resources and Biosecurity (COVAB), Makerere University, P. O Box 7062, Kampala, Uganda; Veterinary Department, State House, P. O Box 25497, Kampala, Uganda.
(6)College of Veterinary Medicine, Animal Resources and Biosecurity (COVAB), Makerere University, P. O Box 7062, Kampala, Uganda; Directorate of Product

Safety, National Drug Authority, P. O. Box 23096, Kampala, Uganda.
(7)National Agricultural Advisory Services (NAADS), P O Box 389, Mbarara ZARDI, Uganda.
(8)National Research Center for Protozoan Diseases (NRCPD), Obihiro University of Agriculture and Veterinary Medicine, Inada-Cho, Obihiro 080-8555, Hokkaido, Japan; Research Center for Global Agromedicine, Obihiro University of Agriculture and Veterinary Medicine, Inada-Cho, Obihiro, Hokkaido 080-8555, Japan.
(9)National Research Center for Protozoan Diseases (NRCPD), Obihiro University of Agriculture and Veterinary Medicine, Inada-Cho, Obihiro 080-8555, Hokkaido, Japan; Research Center for Global Agromedicine, Obihiro University of Agriculture and Veterinary Medicine, Inada-Cho, Obihiro, Hokkaido 080-8555, Japan. Electronic address: hisuzuki@obihiro.ac.jp.
The emergence of multi-acaricide resistant ticks has led to unprecedented level of acaricide failure in central and western Uganda. In the absence of a national acaricide resistance management strategy, the country's dairy sector is threatened by upsurge of ticks and tick-borne diseases. In this study, we developed a short-to-medium-term intervention approach called Evidence-Based Acaricide Tick Control (EBATIC): Identify, Test, Intervene and Eradicate (IT-IE). Furthermore, the perception of 199 farmers and extension workers, 12 key informants in four districts and 47 stakeholders in the animal industry in Uganda were assessed using semi-structured questionnaires. We report that the establishment of a specialized laboratory is pivotal in identifying and testing (IT) acaricide resistant ticks for prompt intervention and eradication (IE). The laboratory test results and the farm tick control gaps identified are very important in guiding acaricide resistance management strategies such as evidence-based acaricide rotation, development and dissemination of extension materials, training of farmers and extension workers, and stakeholders' engagement towards finding sustainable solutions. All the 47 stakeholders and 91.0% (181/199) of the farmers and extension workers reported that the EBATIC approach will help in solving the tick acaricide resistance crisis in Uganda. Similarly, all the 12 key informants and 92.5% (184/199) of the farmers and extension workers suggested that the EBATIC approach should be sustained and rolled out to other districts. The EBATIC stakeholders' dialogue generated both short-to-medium and long-term strategies for sustainable management of tick acaricide resistance in the country. Overall, the positive feedback from farmers, district veterinarians and stakeholders in the animal industry suggest that the EBATIC approach is a useful proof-of-concept on scalable intervention pathway against tick acaricide resistance in Uganda with possibility of adoption in other African countries.

97. Pediatr Emerg Care. 2020 Mar;36(3):e160-e162. doi: 10.1097/PEC.0000000000001265.
Pediatric Poisonings in a Rural Ugandan Emergency Department.
Boyle KL(1)(2), Periyanayagam U(2)(3)(4), Babu KM(5), Rice BT(4)(6), Bisanzo M(4)(7).
Author information:
(1)From the Beth Israel Deaconess Medical Center.

(2)Harvard Medical School.
(3)Brigham and Women's Hospital, Boston.
(4)Global Emergency Care Collaborative, Shrewsbury.
(5)University of Massachusetts Medical School, Worcester, MA.
(6)New York University School of Medicine, New York, NY.
(7)University of Vermont College of Medicine, Burlington, VT.
OBJECTIVE: This study aims to describe pediatric poisonings presenting to a rural Ugandan emergency department (ED), identifying demographic factors and causative agents.
METHODS: This retrospective study was conducted in the ED of a rural hospital in the Rukungiri District of Uganda. A prospectively collected quality assurance database of ED visits was queried for poisonings in patients under the age of 5 who were admitted to the hospital. Cases were included if the chief complaint or final diagnosis included anything referable to poisoning, ingestion, or intoxication, or if a toxicologic antidote was administered. The database was coded by a blinded investigator, and descriptive statistics were performed.
RESULTS: From November 9, 2009, to July 11, 2014, 3428 patients under the age of 5 were admitted to the hospital. A total of 123 cases (3.6%) met the inclusion criteria. Seventy-two patients were male (58.5%). The average age was 2.3 (SD, 0.97) years with 45 children (36.6%) under the age of 2 years. There were 19 cases (15.4%) lost to 3-day follow-up. The top 3 documented exposures responsible for pediatric poisonings were cow tick or organophosphates (36 cases, 29.2%), general poison or drug overdose (26 cases, 21.1%), and paraffin or hydrocarbon (24 cases, 19.5%).Of the admitted patients, 1 died in the ED and 2 died at 72-hour follow-up, for an overall 72-hour mortality of 2.4%. Patients who died were exposed to iron, cow tick, and rat poison.
CONCLUSIONS: Pediatric poisoning affects patients in rural sub-Saharan Africa. The mortality rate at one rural Ugandan hospital was greater than 2%.

98. BMC Vet Res. 2017 Nov 25;13(1):355. doi: 10.1186/s12917-017-1285-z.
African animal trypanosomiasis as a constraint to livestock health and production in Karamoja region: a detailed qualitative and quantitative assessment.
Muhanguzi D(1), Mugenyi A(2), Bigirwa G(3), Kamusiime M(4), Kitibwa A(3), Akurut GG(3), Ochwo S(3), Amanyire W(3), Okech SG(3), Hattendorf J(5)(6), Tweyongyere R(3).
Author information:
(1)College of Veterinary Medicine Animal Resources and Biosecurity, Makerere University, P.O. Box 7062, Kampala, Uganda. luckydenno@covab.mak.ac.ug.
(2)Coordinating Office for Control of Trypanosomiasis in Uganda, Ministry of Agriculture, Animal Industry and Fisheries, Plot 78, Buganda Road, P. O. Box: 16345 Wandegeya, Kampala, Uganda.
(3)College of Veterinary Medicine Animal Resources and Biosecurity, Makerere University, P.O. Box 7062, Kampala, Uganda.
(4)Mercy Corps Uganda, PO Box 32021, Clock Tower, Kampala, Uganda.
(5)Swiss Tropical Institute, Socinstrasse 57, -4002, Basel, CH, Switzerland.
(6)University of Basel, Petersplatz 1, 4003, Basel, Switzerland.

BACKGROUND: Nagana (African Animal Trypanosomiasis-AAT) and tick-borne diseases (TBDs) constrain livestock production in most parts of sub-Saharan Africa. To this realisation, Uganda government set up an African trypanosomiasis (AT) control unit, which among other activities generates national tsetse control priority maps using apparent tsetse density data. Such maps underestimate mechanically transmitted AAT and thus ought to be refined using actual AT prevalence data. We therefore set out to generate up-to-date cattle and donkey trypanosomiasis prevalence data as well as find out the constraints to livestock production in Karamoja region in a bid to re-define AT control priority in this region.
RESULTS: Livestock keepers and animal health workers indicated that TBDs and AAT were the most important livestock diseases in Karamoja region. The prevalence of Trypanosoma spp. in cattle and donkeys was 16.3% (95% CI: 12.4-21.1%) and 32.4% (95% CI; 20.2-47.6%) respectively. Trypanosoma vivax (12.1%) and Trypanosoma congolense savannah (29.6%) were the most prevalent Trypanosoma spp. in cattle and donkeys respectively. Majority of the cattle (85.7%) and more than half of the donkey (57.1%) herds were positive for Trypanosoma spp.
CONCLUSIONS: African animal trypanosomiasis and TBDs are the most important constraints to livestock production in Karamoja region. In order to improve livestock production and hence Karamajong livelihoods, government of Uganda and her development partners will need to invest in livestock health programs particularly targeting tsetse and TBD control.

99. Pestic Biochem Physiol. 2017 Nov;143:181-190. doi: 10.1016/j.pestbp.2017.07.009.
Genetic mutations in sodium channel domain II and carboxylesterase genes associated with phenotypic resistance against synthetic pyrethroids by Rhipicephalus (Boophilus) decoloratus ticks in Uganda.
Vudriko P(1), Umemiya-Shirafuji R(2), Okwee-Acai J(3), Tayebwa DS(4), Byaruhanga J(4), Jirapattharasate C(5), Liu M(5), Adjou Moumouni PF(5), Fujisaki K(6), Xuan X(7), Suzuki H(8).
Author information:
(1)Department of Veterinary Pharmacy, Clinics and Comparative Medicine, School of Veterinary Medicine and Animal Resources, College of Veterinary Medicine, Animal Resources and Biosecurity, Makerere University, P. O. Box 7062, Kampala, Uganda; Research Center for Ticks and Tick-borne Disease Control (RTC), College of Veterinary Medicine, Animal Resources and Biosecurity, Makerere University, P. O. Box 7062, Kampala, Uganda; National Research Center for Protozoan Diseases, Obihiro University of Agriculture and Veterinary Medicine, Inada-eCho, Obihiro 080-8555, Hokkaido, Japan.
(2)National Research Center for Protozoan Diseases, Obihiro University of Agriculture and Veterinary Medicine, Inada-eCho, Obihiro 080-8555, Hokkaido, Japan; United Graduate School of Veterinary Sciences, Gifu University, 1-1 Yanagido, Gifu 501-1193, Japan; Research Center for Global Agromedicine, Obihiro University of Agriculture and Veterinary Medicine, Inada-Cho, Obihiro, Hokkaido 080-8555, Japan.
(3)Department of Veterinary Pharmacy, Clinics and Comparative Medicine, School of Veterinary Medicine and Animal Resources, College of Veterinary Medicine,

Animal Resources and Biosecurity, Makerere University, P. O. Box 7062, Kampala, Uganda; Research Center for Ticks and Tick-borne Disease Control (RTC), College of Veterinary Medicine, Animal Resources and Biosecurity, Makerere University, P. O. Box 7062, Kampala, Uganda.
(4)Research Center for Ticks and Tick-borne Disease Control (RTC), College of Veterinary Medicine, Animal Resources and Biosecurity, Makerere University, P. O. Box 7062, Kampala, Uganda.
(5)National Research Center for Protozoan Diseases, Obihiro University of Agriculture and Veterinary Medicine, Inada-eCho, Obihiro 080-8555, Hokkaido, Japan.
(6)National Research Center for Protozoan Diseases, Obihiro University of Agriculture and Veterinary Medicine, Inada-eCho, Obihiro 080-8555, Hokkaido, Japan; National Agricultural and Food Research Organization, 3-1-5 Kannondai, Tsukuba, Ibaraki 305-0856, Japan.
(7)National Research Center for Protozoan Diseases, Obihiro University of Agriculture and Veterinary Medicine, Inada-eCho, Obihiro 080-8555, Hokkaido, Japan; Research Center for Global Agromedicine, Obihiro University of Agriculture and Veterinary Medicine, Inada-Cho, Obihiro, Hokkaido 080-8555, Japan.
(8)National Research Center for Protozoan Diseases, Obihiro University of Agriculture and Veterinary Medicine, Inada-eCho, Obihiro 080-8555, Hokkaido, Japan; United Graduate School of Veterinary Sciences, Gifu University, 1-1 Yanagido, Gifu 501-1193, Japan; Research Center for Global Agromedicine, Obihiro University of Agriculture and Veterinary Medicine, Inada-Cho, Obihiro, Hokkaido 080-8555, Japan. Electronic address: hisuzuki@obihiro.ac.jp.
We previously reported emergence of super synthetic pyrethroid (SP) resistant Rhipicephalus (Boophilus) decoloratus ticks in Uganda. This study investigated the genetic basis of phenotypic resistance against SP in R. (B.) decoloratus and sought to identify novel diagnostic mutations for rapid detection of SP resistance in the above tick species. Genomic DNA was extracted from pooled larvae of 20 tick populations (19 of known SP susceptibility and 1 unknown susceptibility). The voltage sensitive sodium channel (VSSC) domain II S4-5 linker (SP target) and partial carboxylesterase (SP metabolizing enzyme) genes were amplified by PCR, cloned and sequenced. The resultant sequences were analyzed to determine single nucleotide polymorphisms (SNPs) associated with phenotypic resistance in the various tick populations investigated. Novel SNPs that introduced Eco RI and Eco RII restriction sites in carboxylesterase gene were identified in silco and validated with restriction fragment length polymorphism (RFLP) against 18 tick populations of known SP susceptibility. The study identified a super knock down resistance (kdr) mutation T58C in R. (B.) decoloratus VSSC associated with stable SP resistance. We further identified multiple nonsynonymous mutations in carboxylesterase of SP resistant ticks; one of which conferred novel EcoRII (G195C) restriction site for PCR-RFLP detection of SP resistance. In conclusion, this study is the first to report super kdr mutation in sodium channel domain II and multiple mutations in carboxylesterase genes that may concurrently mediate stable resistance against synthetic pyrethroids in R. (B.) decoloratus ticks from Uganda. The Eco RII based PCR-RFLP

is a useful tool for rapid detection of stable SP resistant R. (B.) decoloratus ticks.

100. Int J Infect Dis. 2018 Mar;68:88-93. doi: 10.1016/j.ijid.2018.01.013. Epub 2018
Investigation of an isolated case of human Crimean-Congo hemorrhagic fever in Central Uganda, 2015.
Balinandi S(1), Patel K(2), Ojwang J(3), Kyondo J(4), Mulei S(4), Tumusiime A(1), Lubwama B(5), Nyakarahuka L(4), Klena JD(2), Lutwama J(4), Ströher U(2), Nichol ST(2), Shoemaker TR(6).
Author information:
(1)Viral Special Pathogens Branch, Centers for Disease Control and Prevention-Uganda, U.S. Embassy, Plot 1577 Ggaba Road, P.O. Box 7007, Kampala, Uganda.
(2)Viral Special Pathogens Branch, Centers for Disease Control and Prevention, 1600 Clifton Rd., Atlanta, GA, USA.
(3)Global Health Security Unit, Centers for Disease Control and Prevention-Uganda, U.S. Embassy, Plot 1577 Ggaba Road, P.O. Box 7007, Kampala, Uganda.
(4)Department of Emerging, Reemerging and Arbovirus Infections, Uganda Virus Research Institute, Plot 51-57 Nakiwogo Road, P.O. Box 49, Entebbe, Uganda.
(5)Epidemiological Surveillance Division, Ministry of Health, Plot 6, Lourdel Road, P.O. Box 7272, Kampala, Uganda.
(6)Viral Special Pathogens Branch, Centers for Disease Control and Prevention-Uganda, U.S. Embassy, Plot 1577 Ggaba Road, P.O. Box 7007, Kampala, Uganda; Viral Special Pathogens Branch, Centers for Disease Control and Prevention, 1600 Clifton Rd., Atlanta, GA, USA. Electronic address: tis8@cdc.gov.
BACKGROUND: Crimean-Congo hemorrhagic fever (CCHF) is the most geographically widespread tick-borne viral infection. Outbreaks of CCHF in sub-Saharan Africa are largely undetected and thus under-reported. On November 9, 2015, the National Viral Hemorrhagic Fever Laboratory at the Uganda Virus Research Institute received an alert for a suspect VHF case in a 33-year-old male who presented with VHF compatible signs and symptoms at Mengo Hospital in Kampala.
METHODS: A blood sample from the suspect patient was tested by RT-PCR for CCHF and found positive. Serological testing on sequential blood specimens collected from this patient showed increasing anti-CCHFV IgM antibody titers, confirming recent infection. Repeat sampling of the confirmed case post recovery showed high titers for anti-CCHFV-specific IgG. An epidemiological outbreak investigation was initiated following the initial RT-PCR positive detection to identify any additional suspect cases.
RESULTS: Only a single acute case of CCHF was detected from this outbreak. No additional acute CCHF cases were identified following field investigations. Environmental investigations collected 53 tick samples, with only 1, a Boophilus decoloratus, having detectable CCHFV RNA by RT-PCR. Full-length genomic sequencing on a viral isolate from the index human case showed the virus to be related to the DRC (Africa 2) lineage.
CONCLUSIONS: This is the fourth confirmed CCHF outbreak in Uganda within 2 years

after more than 50 years of no reported human CCHF cases in this country. Our investigations reaffirm the endemicity of CCHFV in Uganda, and show that exposure to ticks poses a significant risk for human infection. These findings also reflect the importance of having an established national VHF surveillance system and diagnostic capacity in a developing country like Uganda, in order to identify the first cases of VHF outbreaks and rapidly respond to reduce secondary cases. Additional efforts should focus on implementing effective tick control methods and investigating the circulation of CCHFV throughout the country.

101. Ticks Tick Borne Dis. 2018 Mar;9(3):580-588. doi: 10.1016/j.ttbdis.2018.01.012.
Molecular detection and phylogenetic analysis of Anaplasma marginale and Anaplasma centrale amongst transhumant cattle in north-eastern Uganda.
Byaruhanga C(1), Collins NE(2), Knobel DL(2), Khumalo ZTH(2), Chaisi ME(2), Oosthuizen MC(2).
Author information:
(1)Vectors and Vector-Borne Diseases Research Programme, Department of Veterinary Tropical Diseases, Faculty of Veterinary Science, University of Pretoria, Private Bag X04, Onderstepoort, 0110, South Africa; National Agricultural Research Organisation, P.O. Box 259, Entebbe, Uganda. Electronic address: cbyaruhanga27@yahoo.com.
(2)Vectors and Vector-Borne Diseases Research Programme, Department of Veterinary Tropical Diseases, Faculty of Veterinary Science, University of Pretoria, Private Bag X04, Onderstepoort, 0110, South Africa.
There is little molecular data from Anaplasma marginale and Anaplasma centrale isolates from cattle in Uganda. Between November 2013 and January 2014, blood was collected from 240 cattle in 20 randomly-selected herds in two districts of the Karamoja Region in north-eastern Uganda. A duplex quantitative real-time polymerase chain reaction (qPCR) assay was used to detect and determine the prevalence of A. marginale (targeting the msp1β gene) and A. centrale (targeting the groEL gene). The qPCR assay revealed that most cattle (82.9%; 95% confidence interval [CI] 78.2-87.7%) were positive for A. marginale DNA, while fewer cattle (12.1%; 95% CI 7.9-16.2%) were positive for A. centrale DNA. A mixed effects logistic regression model showed that the age of cattle was significantly associated with A. centrale infection, while the prevalence of A. marginale varied significantly according to locality. The near full-length 16S ribosomal RNA (16S rRNA) gene and the heat shock protein gene, groEL, for both Anaplasma species were amplified from a selection of samples. The amplicons were cloned and the resulting recombinants sequenced. We found three novel A. marginale 16S rRNA variants, seven A. marginale groEL gene sequence variants and two A. centrale groEL gene sequence variants. Phylogenetic trees were inferred from sequence alignments of the 16S rRNA gene and GroEL amino acid sequences determined here and published sequences using maximum likelihood, Bayesian inference and parsimony methods Phylogenetic analyses classified the 16S rRNA gene and GroEL amino acid sequences into one clade for A. marginale and a separate clade for A. centrale. This study reveals a high prevalence and sequence variability of A. marginale and A. centrale, and is the first report on

the phylogenetic characterisation of A. marginale and A. centrale from cattle in Uganda using molecular markers. Sequence variation can be attributed to mobile pastoralism, communal grazing and grazing with wildlife. These data support future epidemiological investigations for bovine anaplasmosis in Uganda.

102. Ticks Tick Borne Dis. 2018 Mar;9(3):707-717. doi: 10.1016/j.ttbdis.2018.02.004.
Occurrence of tick-borne haemoparasites in cattle in the Mungwi District, Northern Province, Zambia.
Tembo S(1), Collins NE(2), Sibeko-Matjila KP(2), Troskie M(2), Vorster I(2), Byaruhanga C(3), Oosthuizen MC(4).
Author information:
(1)Department of Veterinary Services, Box 19, Mungwi, Zambia; Vectors and Vector-borne Diseases Research Programme, Department of Veterinary Tropical Diseases, Faculty of Veterinary Science, University of Pretoria, Private Bag X04, Onderstepoort 0110, South Africa.
(2)Vectors and Vector-borne Diseases Research Programme, Department of Veterinary Tropical Diseases, Faculty of Veterinary Science, University of Pretoria, Private Bag X04, Onderstepoort 0110, South Africa.
(3)Vectors and Vector-borne Diseases Research Programme, Department of Veterinary Tropical Diseases, Faculty of Veterinary Science, University of Pretoria, Private Bag X04, Onderstepoort 0110, South Africa; National Agricultural Research Organization, P.O. Box 259, Entebbe, Uganda.
(4)Vectors and Vector-borne Diseases Research Programme, Department of Veterinary Tropical Diseases, Faculty of Veterinary Science, University of Pretoria, Private Bag X04, Onderstepoort 0110, South Africa. Electronic address: marinda.oosthuizen@up.ac.za.
Little is known about the occurrence of haemoparasites in cattle in communal grazing areas of Mungwi District of Northern Province, Zambia. Clinical signs and post mortem lesions are pathognomonic of mixed tick-borne infections especially babesiosis, anaplasmosis and East Coast fever. The main objective of this study was to screen selected communal herds of cattle for tick-borne haemoparasites, and identify the tick vectors associated with the high cattle mortalities due to suspected tick-borne diseases in the local breeds of cattle grazing along the banks of the Chambeshi River in Mungwi District, Northern Province, Zambia. A total of 299 cattle blood samples were collected from July to September 2010 from Kapamba (n = 50), Chifulo (n = 102), Chisanga (n = 38), Kowa (n = 95) and Mungwi central (n = 14) in the Mungwi District. A total of 5288 ticks were also collected from the sampled cattle from April to July 2011. DNA was extracted from the cattle blood and the hypervariable region of the parasite small subunit rRNA gene was amplified and subjected to the reverse line blot (RLB) hybridization assay. The results of the RLB assay revealed the presence of tick-borne haemoparasites in 259 (86.6%) cattle blood samples occurring either as single (11.0%) or mixed (75.6%) infections. The most prevalent species present were the benign Theileria mutans (54.5%) and T. velifera (51.5%). Anaplasma marginale (25.7%), Babesia bovis (7.7%) and B. bigemina (3.3%) DNA were also detected in the samples. Only one sample (from Kapamba) tested positive for the presence of T. parva. This was an unexpected

finding; also because the tick vector, Rhipicephalus appendiculatus, was identified on animals from Kowa (14.0%), Chisanga (8.5%), Chifulo (6.0%) and Kapamba (1.4%). One sample (from Kapamba) tested positive for the presence of Ehrlichia ruminantium even though Amblyomma variegatum ticks were identified from 52.9% of the sampled animals from all study areas. There was significant positive association between T. mutans and T. velifera ($p < 0.001$) infections, and between A. marginale and B. bovis ($p = 0.005$). The presence of R. microplus tick vectors on cattle was significantly associated with B. bovis (odds ratio, OR = 28.4, $p < 0.001$) and A. marginale (OR = 42.0, $p < 0.001$) infections, while A. variegatum presence was significantly associated with T. mutans (OR = 213.0, $p < 0.001$) and T. velifera (OR = 459.0, $p < 0.001$) infections. Rhipicephalus decoloratus was significantly associated with B. bigemina (OR = 21.6, $p = 0.004$) and A. marginale (OR = 28.5, $p < 0.001$). Multivariable analysis showed a significant association between location and tick-borne pathogen status for A. marginale ($p < 0.001$), T. mutans ($p = 0.004$), T. velifera ($p = 0.003$) and T. taurotragi ($p = 0.005$). The results of our study suggest that the cause of cattle mortalities in Mungwi during the winter outbreaks is mainly due to A. marginale, B. bovis and B. bigemina infections. This was confirmed by the clinical manifestation of the disease in the affected cattle and the tick species identified on the animals. The relatively low prevalence of T. parva, B. bigemina, B. bovis and E. ruminantium could indicate the existence of endemic instability with a pool of susceptible cattle and the occurrence of disease outbreaks.

103. Ticks Tick Borne Dis. 2018 May;9(4):945-955. doi: 10.1016/j.ttbdis.2018.03.009. Epub 2018 Mar 15.

Chemical tick control practices in southwestern and northwestern Uganda.

Vudriko P(1), Okwee-Acai J(2), Byaruhanga J(3), Tayebwa DS(4), Okech SG(5), Tweyongyere R(5), Wampande EM(5), Okurut ARA(6), Mugabi K(6), Muhindo JB(7), Nakavuma JL(5), Umemiya-Shirafuji R(8), Xuan X(8), Suzuki H(9).
Author information:
(1)Research Center for Ticks and Tick-borne Diseases Control, Department of Veterinary Pharmacy, Clinical and Comparative Medicine, College of Veterinary Medicine, Animal Resources and Biosecurity (COVAB), Makerere University, P. O Box 7062, Kampala, Uganda; College of Veterinary Medicine, Animal Resources and Biosecurity (COVAB), Makerere University, P. O Box 7062, Kampala, Uganda; National Research Center for Protozoan Diseases (NRCPD), Obihiro University of Agriculture and Veterinary Medicine, Inada-Cho, Obihiro 080-8555, Hokkaido, Japan.
(2)Research Center for Ticks and Tick-borne Diseases Control, Department of Veterinary Pharmacy, Clinical and Comparative Medicine, College of Veterinary Medicine, Animal Resources and Biosecurity (COVAB), Makerere University, P. O Box 7062, Kampala, Uganda; College of Veterinary Medicine, Animal Resources and Biosecurity (COVAB), Makerere University, P. O Box 7062, Kampala, Uganda.
(3)Research Center for Ticks and Tick-borne Diseases Control, Department of Veterinary Pharmacy, Clinical and Comparative Medicine, College of Veterinary Medicine, Animal Resources and Biosecurity (COVAB), Makerere University, P. O

Box 7062, Kampala, Uganda.
(4)Research Center for Ticks and Tick-borne Diseases Control, Department of Veterinary Pharmacy, Clinical and Comparative Medicine, College of Veterinary Medicine, Animal Resources and Biosecurity (COVAB), Makerere University, P. O Box 7062, Kampala, Uganda; National Research Center for Protozoan Diseases (NRCPD), Obihiro University of Agriculture and Veterinary Medicine, Inada-Cho, Obihiro 080-8555, Hokkaido, Japan.
(5)College of Veterinary Medicine, Animal Resources and Biosecurity (COVAB), Makerere University, P. O Box 7062, Kampala, Uganda.
(6)Ministry of Agriculture, Animal Industry and Fisheries, National Animal Disease Diagnostics and Epidemiology Centre, P.O. Box 513, Entebbe, Uganda.
(7)College of Veterinary Medicine, Animal Resources and Biosecurity (COVAB), Makerere University, P. O Box 7062, Kampala, Uganda; Directorate of Product Safety, National Drug Authority, P. O. Box 23096, Kampala, Uganda.
(8)National Research Center for Protozoan Diseases (NRCPD), Obihiro University of Agriculture and Veterinary Medicine, Inada-Cho, Obihiro 080-8555, Hokkaido, Japan; Research Center for Global Agromedicine, Obihiro University of Agriculture and Veterinary Medicine, Inada-Cho, Obihiro, Hokkaido 080-8555, Japan.
(9)National Research Center for Protozoan Diseases (NRCPD), Obihiro University of Agriculture and Veterinary Medicine, Inada-Cho, Obihiro 080-8555, Hokkaido, Japan; Research Center for Global Agromedicine, Obihiro University of Agriculture and Veterinary Medicine, Inada-Cho, Obihiro, Hokkaido 080-8555, Japan. Electronic address: hisuzuki@obihiro.ac.jp.
Tick acaricide failure is one of the leading challenges to cattle production in Uganda. To gain an understanding into the possible drivers of acaricide failure, this study characterized the current chemical tick control practices in the southwestern (Mbarara, Mitooma and Rukungiri districts) and northwestern (Adjumani district) regions of Uganda. A total of 85 farms participated in a survey that utilized a semi-structured questionnaire. Moreover, ticks were collected to determine the most common species on the farms. Tick acaricide failure was mainly encountered in the districts where 95% (60/63) of the farms reared exotic cattle (dairy cross-breeds) under a paddocking (fenced) system. In the northwestern region, local cattle were reared in communal grazing areas. All farms used chemical acaricides for tick control, predominantly amidine (amitraz) (48%, 41/85) and co-formulated organophosphates and pyrethroids (38%, 32/85). The spraying method was the most common (91%, 77/85) acaricide application technique, with cattle crush (81%, 69/85) as a common means of physical restraint. Less than optimal tick control practices encountered included use of substandard equipment for spraying, inappropriate dilutions, frequent interaction between animals in neighboring farms despite lack of synchronized chemical tick control and malpractices in acaricide rotation. Only Rhipicephalus appendiculatus and R. (Boophilus) decoloratus ticks were found in the southwestern region, where 51% (32/63) of the farmers used high acaricide concentrations above the manufacturers' recommendation. Farmers in the northwestern region used 2.2 times less acaricide volume per cattle than those in the southwestern region, and more diverse tick species were encountered.

Toxic effects of acaricide to cattle and workers were reported by 13% (11/85) and 32% (27/85) of the respondents, respectively. All 27 cases of human acaricide toxicity reported were from the southwestern region. Overall, our findings may inform strategies for more prudent chemical tick control and safe acaricide handling to benefit animal welfare, food safety and public health.

104. Ticks Tick Borne Dis. 2018 Jul;9(5):1192-1201. doi: 10.1016/j.ttbdis.2018.04.019. Epub 2018 Apr 27.
The effects of nitidine chloride and camptothecin on the growth of Babesia and Theileria parasites.
Tayebwa DS(1), Tuvshintulga B(2), Guswanto A(3), Nugraha AB(4), Batiha GE(5), Gantuya S(6), Rizk MA(7), Vudriko P(8), Sivakumar T(9), Yokoyama N(10), Igarashi I(11).
Author information:
(1)National Research Center for Protozoan Diseases, Obihiro University of Agriculture Veterinary Medicine, Nishi 2 Sen-11, Inada-cho, Obihiro, Hokkaido 080-8555, Japan; Central Diagnostic Laboratory, College of Veterinary Medicine, Animal Resources and Biosecurity, Makerere University, 7062, Kampala, Uganda; Research Center for Tick and Tick-borne Diseases Control, College of Veterinary Medicine, Animal Resources and Biosecurity, Makerere University, 7062, Kampala, Uganda. Electronic address: tayebwa.dickson@gmail.com.
(2)National Research Center for Protozoan Diseases, Obihiro University of Agriculture Veterinary Medicine, Nishi 2 Sen-11, Inada-cho, Obihiro, Hokkaido 080-8555, Japan. Electronic address: tuvshintulga@gmail.com.
(3)National Research Center for Protozoan Diseases, Obihiro University of Agriculture Veterinary Medicine, Nishi 2 Sen-11, Inada-cho, Obihiro, Hokkaido 080-8555, Japan. Electronic address: guswanto7@gmail.com.
(4)National Research Center for Protozoan Diseases, Obihiro University of Agriculture Veterinary Medicine, Nishi 2 Sen-11, Inada-cho, Obihiro, Hokkaido 080-8555, Japan. Electronic address: arifin.budiman.fkh@gmail.com.
(5)National Research Center for Protozoan Diseases, Obihiro University of Agriculture Veterinary Medicine, Nishi 2 Sen-11, Inada-cho, Obihiro, Hokkaido 080-8555, Japan; Department of Pharmacology and Therapeutics, Faculty of Veterinary Medicine, Damanhour University, 22511, Albeheira, Egypt. Electronic address: gaberbatiha@gmail.com.
(6)National Research Center for Protozoan Diseases, Obihiro University of Agriculture Veterinary Medicine, Nishi 2 Sen-11, Inada-cho, Obihiro, Hokkaido 080-8555, Japan. Electronic address: sgantuya@gmail.com.
(7)National Research Center for Protozoan Diseases, Obihiro University of Agriculture Veterinary Medicine, Nishi 2 Sen-11, Inada-cho, Obihiro, Hokkaido 080-8555, Japan; Department of Internal Medicine and Infectious Diseases, Faculty of Veterinary Medicine, Mansoura University, 35516, Egypt. Electronic address: Dr_moh_abdo2008@mans.edu.eg.
(8)National Research Center for Protozoan Diseases, Obihiro University of Agriculture Veterinary Medicine, Nishi 2 Sen-11, Inada-cho, Obihiro, Hokkaido 080-8555, Japan; Research Center for Tick and Tick-borne Diseases Control, College of Veterinary Medicine, Animal Resources and Biosecurity, Makerere

University, 7062, Kampala, Uganda. Electronic address: vpato2009@gmail.com.
(9)National Research Center for Protozoan Diseases, Obihiro University of Agriculture Veterinary Medicine, Nishi 2 Sen-11, Inada-cho, Obihiro, Hokkaido 080-8555, Japan. Electronic address: Siva@obihiro.ac.jp.
(10)National Research Center for Protozoan Diseases, Obihiro University of Agriculture Veterinary Medicine, Nishi 2 Sen-11, Inada-cho, Obihiro, Hokkaido 080-8555, Japan. Electronic address: yokoyama@obihiro.ac.jp.
(11)National Research Center for Protozoan Diseases, Obihiro University of Agriculture Veterinary Medicine, Nishi 2 Sen-11, Inada-cho, Obihiro, Hokkaido 080-8555, Japan. Electronic address: igarcpmi@obihiro.ac.jp.
The treatment of bovine and equine piroplasmosis is limited to diminazene aceturate (DA) and imidocarb dipropionate. To address this challenge, we need to explore novel drug compounds and targets. Topoisomerases are potential drug targets because they play a vital role in solving topological errors of DNA strands during replication. This study documented the effectiveness of topoisomerase inhibitors, nitidine chloride (NC) and camptothecin (Cpt), on the growth of Babesia and Theileria parasites. The half maximal inhibitory concentrations (IC50s) against B. bovis, B. bigemina, B. caballi, and T. equi were 1.01 ± 0.2, 5.34 ± 1.0, 0.11 ± 0.03, and 2.05 ± 0.4 µM for NC and 11.67 ± 1.6, 4.00 ± 1.0, 2.07 ± 0.6, and 0.33 ± 0.02 µM for Cpt, respectively. The viability experiment revealed that 4, 10, and 4 µM treatments of NC or 48, 8, and 8 µM treatments of Cpt were sufficient to stop the in vitro regrowth of B. bovis, B. bigemina, and B. caballi, respectively. However, T. equi regrew in all of the concentrations used. Moreover, increasing the concentration of NC and Cpt to 16 µM and 1.2 µM (8 × IC50) did not eliminate T. equi. The micrographs of B. bigemina and B. caballi taken at 24 h and 72 h showed deformed merozoites and remnants of parasites within the red blood cell (RBC), respectively. The treatments of 25 mg/kg DA and 20 mg/kg NC administered intraperitoneally and 20 mg/kg NC given orally showed 93.7, 90.7, and 83.6% inhibition against Babesia microti (B. microti), respectively, compared to the untreated group on day 8. In summary, NC and Cpt were effective against Babesia and Theileria parasites in vitro. Moreover, 20 mg/kg NC administered intraperitoneally was as effective as 25 mg/kg DA against B. microti in mice and showed no toxic symptoms in mice. The results indicate that NC may, after further evaluations, prove to be an alternative drug against bovine and equine piroplasmoses.

105. Cochrane Database Syst Rev. 2018 Jun 5;6(6):CD012713. doi: 10.1002/14651858.CD012713.pub2.

Ribavirin for treating Crimean Congo haemorrhagic fever.
Johnson S(1), Henschke N, Maayan N, Mills I, Buckley BS, Kakourou A, Marshall R.
Author information:
(1)Department of Clinical Sciences, Liverpool School of Tropical Medicine, Pembroke Place, Liverpool, UK, L3 5QA.
BACKGROUND: Crimean Congo haemorrhagic fever (CCHF) is a tick-borne disease that occurs in parts of Asia, Europe and Africa. Since 2000 the infection has caused epidemics in Turkey, Iran, Russia, Uganda and Pakistan. Good-quality general

supportive medical care helps reduce mortality. There is uncertainty and controversy about treating CCHF with the antiviral drug ribavirin.
OBJECTIVES: To assess the effects of ribavirin for treating people with Crimean Congo haemorrhagic fever.
SEARCH METHODS: We searched the Cochrane Infectious Diseases Group Specialized Register; the Central Register of Controlled Trials (CENTRAL); MEDLINE (PubMed); Embase (OVID); Science Citation Index-Expanded, Social Sciences Citation index, conference proceedings (Web of Science); and CINAHL (EBSCOHost). We also searched the WHO International Clinical Trials Registry Platform (ICTRP) and ClinicalTrials.gov for trials in progress. We conducted all searches up to 16 October 2017. We also contacted experts in the field and obtained further studies from these sources.
SELECTION CRITERIA: We evaluated studies assessing the use of ribavirin in people with suspected or confirmed Crimean Congo haemorrhagic fever. We included randomised control trials (RCTs); non-randomised studies (NRSs) that included more than 10 participants designed as cohort studies with comparators; and case-control studies.
DATA COLLECTION AND ANALYSIS: Two review authors assessed eligibility, risk of bias, and extracted data. For non-randomized studies we used the ROBINS-I tool to assess risk of bias. The main effects analysis included all studies where we judged the risk of bias to be low, moderate or high. We summarized dichotomous outcomes using risk ratios (RRs) and continuous outcomes using mean differences (MDs), and used meta-analyses where appropriate. We carried out a subsidiary appraisal and analysis of studies with critical risk of bias for the primary outcome, as these are often cited to support using ribavirin.
MAIN RESULTS: For the main effects analysis, five studies met our inclusion criteria: one RCT with 136 participants and four non-randomized studies with 612 participants. We excluded 18 non-randomized studies with critical risk of bias, where none had attempted to control for confounding.We do not know if ribavirin reduces mortality (1 RCT; RR 1.13, 95% confidence interval (CI) 0.29 to 4.32; 136 participants; very low-certainty evidence; 3 non-randomized studies; RR 0.72, 95% CI 0.41 to 1.28; 549 participants; very low-certainty evidence). We do not know if ribavirin reduces the length of stay in hospital (1 RCT: mean difference (MD) 0.70 days, 95% CI -0.39 to 1.79; 136 participants; and 1 non-randomized study: MD -0.80, 95% CI -2.70 to 1.10; 50 participants; very low-certainty evidence). We do not know if it reduces the risk of patients needing platelet transfusions (1 RCT: RR 1.23, 95% CI 0.77 to 1.96; 136 participants; very low-certainty evidence). For adverse effects (including haemolytic anaemia and a need to discontinue treatment), we do not know whether there is an increased risk with ribavirin in people with CCHF as data are insufficient.We do not know if adding ribavirin to early supportive care improves outcomes. One non-randomized study assessed mortality in people receiving ribavirin and supportive care within four days or less from symptom onset compared to after four days since symptom onset: mortality was lower in the group receiving early supportive care and ribavirin, but it is not possible to distinguish between the effects of ribavirin and early supportive medical care alone.In the subsidiary analysis, 18 studies compared people receiving

ribavirin with those not receiving ribavirin. All had a critical risk of bias due to confounding, reflected in the mortality point estimates favouring ribavirin.
AUTHORS' CONCLUSIONS: We do not know if ribavirin is effective for treating Crimean Congo haemorrhagic fever. Non-randomized studies are often cited as evidence of an effect, but the risk of bias in these studies is high.

106. NPJ Vaccines. 2018 Jun 11;3:24. doi: 10.1038/s41541-018-0061-9. eCollection
Current status of Zika vaccine development: Zika vaccines advance into clinical evaluation.
Barrett ADT(1).
Author information:
(1)Sealy Institute for Vaccine Sciences and Department of Pathology, University of Texas Medical Branch, Galveston, TX 77555-0436 USA.
Zika virus (ZIKV), a mosquito-borne flavivirus, was first identified in the 1940s in Uganda in Africa and emerged in the Americas in Brazil in May 2015. In the 30 months since ZIKV emerged as a major public health problem, spectacular progress has been made with vaccine development cumulating with the publication of three reports of phase 1 clinical trials in the 4th quarter of 2017. Clinical trials involving candidate DNA and purified inactivated virus vaccines showed all were safe and well-tolerated in the small number of volunteers and all induced neutralizing antibodies, although these varied by vaccine candidate and dosing regimen. These results suggest that a Zika vaccine can be developed and that phase 2 clinical trials are warranted. However, it is difficult to compare the results from the different phase 1 studies or with neutralizing antibodies induced by licensed flavivirus vaccines (Japanese encephalitis, tick-borne encephalitis, and yellow fever) as neutralizing antibody assays vary and, unfortunately, there are no standards for Zika virus neutralizing antibodies. In addition to clinical studies, substantial progress continues to be made in nonclinical development, particularly in terms of the ability of candidate vaccines to protect reproductive tissues, and the potential use of monoclonal antibodies for passive prophylaxis.

107. Ticks Tick Borne Dis. 2018 Sep;9(6):1475-1483. doi: 10.1016/j.ttbdis.2018.06.012. Epub 2018 Jun 28.
Molecular epidemiology of Babesia species, Theileria parva, and Anaplasma marginale infecting cattle and the tick control malpractices in Central and Eastern Uganda.

Tayebwa DS(1), Vudriko P(2), Tuvshintulga B(3), Guswanto A(4), Nugraha AB(5), Gantuya S(6), Batiha GE(7), Musinguzi SP(8), Komugisha M(9), Bbira JS(10), Okwee-Acai J(11), Tweyongyere R(12), Wampande EM(13), Byaruhanga J(14), Adjou Moumouni PF(15), Sivakumar T(16), Yokoyama N(17), Igarashi I(18).
Author information:
(1)National Research Center for Protozoan Diseases, Obihiro University of Agriculture and Veterinary Medicine, Nishi 2 Sen-11, Inada-cho, 080-8555, Obihiro, Hokkaido, Japan; Central Diagnostic Laboratory, Makerere University,

College of Veterinary Medicine, Animal Resources and Biosecurity, 7062, Kampala, Uganda; Research Center for Ticks and Tick-borne Diseases, Makerere University, College of Veterinary Medicine, Animal Resources and Biosecurity, 7062, Kampala, Uganda. Electronic address: tayebwa.dickson@gmail.com.
(2)National Research Center for Protozoan Diseases, Obihiro University of Agriculture and Veterinary Medicine, Nishi 2 Sen-11, Inada-cho, 080-8555, Obihiro, Hokkaido, Japan; Research Center for Ticks and Tick-borne Diseases, Makerere University, College of Veterinary Medicine, Animal Resources and Biosecurity, 7062, Kampala, Uganda; School of Veterinary Medicine and Animal Resources, Makerere University, College of Veterinary Medicine, Animal Resources and Biosecurity, 7062, Kampala, Uganda. Electronic address: vpato2009@gmail.com.
(3)National Research Center for Protozoan Diseases, Obihiro University of Agriculture and Veterinary Medicine, Nishi 2 Sen-11, Inada-cho, 080-8555, Obihiro, Hokkaido, Japan. Electronic address: tuvshintulga@gmail.com.
(4)National Research Center for Protozoan Diseases, Obihiro University of Agriculture and Veterinary Medicine, Nishi 2 Sen-11, Inada-cho, 080-8555, Obihiro, Hokkaido, Japan. Electronic address: guswanto7@gmail.com.
(5)National Research Center for Protozoan Diseases, Obihiro University of Agriculture and Veterinary Medicine, Nishi 2 Sen-11, Inada-cho, 080-8555, Obihiro, Hokkaido, Japan. Electronic address: arifin.budiman.fkh@gmail.com.
(6)National Research Center for Protozoan Diseases, Obihiro University of Agriculture and Veterinary Medicine, Nishi 2 Sen-11, Inada-cho, 080-8555, Obihiro, Hokkaido, Japan. Electronic address: sgantuya@gmail.com.
(7)National Research Center for Protozoan Diseases, Obihiro University of Agriculture and Veterinary Medicine, Nishi 2 Sen-11, Inada-cho, 080-8555, Obihiro, Hokkaido, Japan; Department of Pharmacology and Therapeutics, Faculty of Veterinary Medicine, Damanhour University, 22511, Albeheira, Egypt. Electronic address: dr_gaber_batiha@yahoo.com.
(8)National Research Center for Protozoan Diseases, Obihiro University of Agriculture and Veterinary Medicine, Nishi 2 Sen-11, Inada-cho, 080-8555, Obihiro, Hokkaido, Japan. Electronic address: musinguzisimonpeter@gmail.com.
(9)Research Center for Ticks and Tick-borne Diseases, Makerere University, College of Veterinary Medicine, Animal Resources and Biosecurity, 7062, Kampala, Uganda. Electronic address: mariamkomugisha@gmail.com.
(10)Research Center for Ticks and Tick-borne Diseases, Makerere University, College of Veterinary Medicine, Animal Resources and Biosecurity, 7062, Kampala, Uganda. Electronic address: bbirajohnson@gmail.com.
(11)Research Center for Ticks and Tick-borne Diseases, Makerere University, College of Veterinary Medicine, Animal Resources and Biosecurity, 7062, Kampala, Uganda; School of Veterinary Medicine and Animal Resources, Makerere University, College of Veterinary Medicine, Animal Resources and Biosecurity, 7062, Kampala, Uganda. Electronic address: jokwee@yahoo.co.uk.
(12)School of Veterinary Medicine and Animal Resources, Makerere University, College of Veterinary Medicine, Animal Resources and Biosecurity, 7062, Kampala, Uganda. Electronic address: tmrobert966@gmail.com.
(13)School of Veterinary Medicine and Animal Resources, Makerere University, College of Veterinary Medicine, Animal Resources and Biosecurity, 7062, Kampala,

Uganda. Electronic address: ewampande@yahoo.co.uk.
(14)Research Center for Ticks and Tick-borne Diseases, Makerere University, College of Veterinary Medicine, Animal Resources and Biosecurity, 7062, Kampala, Uganda. Electronic address: josephjbvincent@gmail.com.
(15)National Research Center for Protozoan Diseases, Obihiro University of Agriculture and Veterinary Medicine, Nishi 2 Sen-11, Inada-cho, 080-8555, Obihiro, Hokkaido, Japan. Electronic address: chakirou82@yahoo.fr.
(16)National Research Center for Protozoan Diseases, Obihiro University of Agriculture and Veterinary Medicine, Nishi 2 Sen-11, Inada-cho, 080-8555, Obihiro, Hokkaido, Japan. Electronic address: sivavets@gmail.com.
(17)National Research Center for Protozoan Diseases, Obihiro University of Agriculture and Veterinary Medicine, Nishi 2 Sen-11, Inada-cho, 080-8555, Obihiro, Hokkaido, Japan. Electronic address: yokoyama@obihiro.ac.jp.
(18)National Research Center for Protozoan Diseases, Obihiro University of Agriculture and Veterinary Medicine, Nishi 2 Sen-11, Inada-cho, 080-8555, Obihiro, Hokkaido, Japan. Electronic address: igarcpmi@obihiro.ac.jp.
East Coast fever, babesiosis, and anaplasmosis are the major tick-borne diseases affecting cattle productivity in Uganda. The emergence of acaricide-resistant ticks is suspected to have caused a rise in hemoparasites. This study sought to detect and characterize hemoparasites among farms in acaricide-failure hotspots of central as compared to the acaricide-failure naïve areas in Eastern Uganda. Nested PCR assays were performed to determine the prevalences of Babesia bovis, Babesia bigemina, Theileria parva, and Anaplasma marginale in cattle blood samples sourced from randomly selected farms. Randomly selected isolates were sequenced to determine the genetic diversity of the parasites using the following marker genes: B. bovis spherical body protein 4, B. bigemina rhoptry-associated protein 1a, T. parva 104 kDa microneme-rhoptry antigen, and A. marginale major surface protein 5. Furthermore, partially and fully engorged adult ticks were collected for taxonomy, and tick-control practices were assessed using a semi-structured questionnaire. The prevalences of B. bigemina, T. parva, and A. marginale in cattle were 17.2, 65.1, and 22.0%, and 10.0, 26.5, and 3% in the central and eastern region, respectively. Whilst, B. bovis was not detected in the farms involved. The sequences for B. bigemina, T. parva, and A. marginale from the central region showed 99% identity with those from the eastern region. Of the 548 ticks collected, 319, 147, 76, and 6 were Rhipicephalus (Boophilus) decoloratus, Rhipicephalus appendiculatus, Amblyomma variegatum, and Rhipicephalus evertsi evertsi, respectively. The Rhipicephalus ticks were more abundant in the central region, whereas A. variegatum ticks were more abundant in the eastern region. Tick control malpractices were found in both Central and Eastern Uganda, and 42 of the 56 surveyed farms lacked appropriate restraining facilities and so they utilized either ropes or a 'boma' (enclosure). In summary, B. bigemina, T. parva, A. marginale and their co-infections were more prevalent in the central than eastern region; even though, tick control malpractices were observed in both regions. Therefore, an urgent tick and TBD control strategy is needed.

108. Ticks Tick Borne Dis. 2018 Sep;9(6):1590-1593. doi:

10.1016/j.ttbdis.2018.08.007. Epub 2018 Aug 8.
C190A knockdown mutation in sodium channel domain II of pyrethroid-resistant Rhipicephalus appendiculatus.
Vudriko P(1), Umemiya-Shirafuji R(2), Okwee-Acai J(3), Tayebwa DS(4), Byaruhanga J(4), Bbira JS(4), Fujisaki K(5), Xuan X(6), Suzuki H(7).
Author information:
(1)Department of Veterinary Pharmacy, Clinics and Comparative Medicine, School of Veterinary Medicine and Animal Resources, College of Veterinary Medicine, Animal Resources and Biosecurity, Makerere University, P. O Box 7062, Kampala, Uganda; Research Center for Ticks and Tick-borne Disease Control (RTC), College of Veterinary Medicine, Animal Resources and Biosecurity, Makerere University, P. O Box 7062, Kampala, Uganda; National Research Center for Protozoan Diseases, Obihiro University of Agriculture and Veterinary Medicine, Inada-Cho, Obihiro 080-8555, Hokkaido, Japan.
(2)National Research Center for Protozoan Diseases, Obihiro University of Agriculture and Veterinary Medicine, Inada-Cho, Obihiro 080-8555, Hokkaido, Japan; United Graduate School of Veterinary Sciences, Gifu University, 1-1 Yanagido, Gifu 501-1193, Japan; Research Center for Global Agromedicine, Obihiro University of Agriculture and Veterinary Medicine, Inada-Cho, Obihiro, Hokkaido 080-8555, Japan.
(3)Department of Veterinary Pharmacy, Clinics and Comparative Medicine, School of Veterinary Medicine and Animal Resources, College of Veterinary Medicine, Animal Resources and Biosecurity, Makerere University, P. O Box 7062, Kampala, Uganda; Research Center for Ticks and Tick-borne Disease Control (RTC), College of Veterinary Medicine, Animal Resources and Biosecurity, Makerere University, P. O Box 7062, Kampala, Uganda.
(4)Research Center for Ticks and Tick-borne Disease Control (RTC), College of Veterinary Medicine, Animal Resources and Biosecurity, Makerere University, P. O Box 7062, Kampala, Uganda.
(5)National Research Center for Protozoan Diseases, Obihiro University of Agriculture and Veterinary Medicine, Inada-Cho, Obihiro 080-8555, Hokkaido, Japan; National Agricultural and Food Research Organization, 3-1-5 Kannondai, Tsukuba, Ibaraki 305-0856, Japan.
(6)National Research Center for Protozoan Diseases, Obihiro University of Agriculture and Veterinary Medicine, Inada-Cho, Obihiro 080-8555, Hokkaido, Japan; Research Center for Global Agromedicine, Obihiro University of Agriculture and Veterinary Medicine, Inada-Cho, Obihiro, Hokkaido 080-8555, Japan.
(7)National Research Center for Protozoan Diseases, Obihiro University of Agriculture and Veterinary Medicine, Inada-Cho, Obihiro 080-8555, Hokkaido, Japan; United Graduate School of Veterinary Sciences, Gifu University, 1-1 Yanagido, Gifu 501-1193, Japan; Research Center for Global Agromedicine, Obihiro University of Agriculture and Veterinary Medicine, Inada-Cho, Obihiro, Hokkaido 080-8555, Japan. Electronic address: hisuzuki@obihiro.ac.jp.
This study investigated the target site mutations in the partial sequence of voltage-sensitive sodium channel (VSSC) domain II of synthetic pyrethroid (SP)-resistant Rhipicephalus appendiculatus. Genomic DNA was extracted from

seven tick populations (two susceptible and five resistant) collected from central, eastern and southwestern Uganda. The PCR amplicons of the VSSC domain II were cloned and sequenced to determine novel single nucleotide polymorphisms (SNP). A non-synonymous mutation C78 A corresponding to C190 A was found in all the five SP-resistant ticks. The C78 A mutation led to amino acid substitution from leucine to isoleucine (L21I) which was previously reported to confer knockdown (kdr) mutation in R. (Boophilus) microplus. The genetic confirmation of SP-resistant R. appendiculatus in central and southwestern Uganda calls for an urgent strategy for controlling the ticks.

109. Int J Vet Sci Med. 2017 Aug 7;5(2):168-174. doi: 10.1016/j.ijvsm.2017.07.001. eCollection 2017 Dec.
Retrospective study on cattle and poultry diseases in Uganda.
Byaruhanga J(1), Tayebwa DS(1)(2), Eneku W(2)(3), Afayoa M(2)(3), Mutebi F(2)(3), Ndyanabo S(2)(3), Kakooza S(2), Okwee-Acai J(1)(3), Tweyongyere R(3), Wampande EM(2)(3), Vudriko P(1)(3).
Author information:
(1)Research Center for Ticks and Tick-borne Diseases Control (RTC), College of Veterinary Medicine, Animal Resources and Biosecurity, Makerere University, P.O. Box 7062, Kampala, Uganda.
(2)Central Diagnostic Laboratory, College of Veterinary Medicine, Animal Resources and Biosecurity, Makerere University, P.O. Box 7062, Kampala, Uganda.
(3)School of Veterinary Medicine and Animal Resources, College of Veterinary Medicine, Animal Resources and Biosecurity, Makerere University, P.O. Box 7062, Kampala, Uganda.
Cattle and poultry enterprises are among the major contributors to food security and socioeconomic empowerment of households in Uganda. However, various diseases constrain their productivity. A two-year retrospective study between April 2012 and March 2014 was conducted using records for cattle and poultry diseases diagnosed at the Central Diagnostic Laboratory (CDL) to determine prevalent diseases in Uganda. The laboratory received 836 samples from poultry (36.3%) and cattle (63.7%). Of the 836 samples, 47.5% had a definitive diagnosis of disease causation. Most of the cattle and poultry diseases diagnosed were protozoan diseases (39.3%) followed by bacterial (21.4%), viral (17.1%), helminthiasis (11.1%), nutritional diseases (4%) and others (7.1%). For poultry, viral diseases (29.5%) and protozoan diseases (27.1%) especially newcastle disease (44.3%) and coccidiosis (100%) respectively, were the most diagnosed. While for cattle, hemo-protozoan parasites (52.1%) were the most prevalent, of which 92.9% were east coast fever infection. Bacterial infection (20.5%) in cattle were the second most diagnosed diseases and mastitis was the most diagnosed (46.2%). In summary, coccidioisis, collibacillosis, newcastle disease, gumboro disease, and avian helminthiasis were the most prevalent poultry diseases while in cattle, east coast fever, helminthiasis, mastitis, brucellosis and rabies were the most frequently diagnosed diseases. This study has identified the major diseases that hinder poultry and cattle production in Uganda. The data generated by CDL could be used for surveillance, monitoring and designing strategic interventions for control of poultry and cattle diseases in Uganda.

110. Front Genet. 2018 Oct 3;9:385. doi: 10.3389/fgene.2018.00385. eCollection 2018.
Combining Landscape Genomics and Ecological Modelling to Investigate Local Adaptation of Indigenous Ugandan Cattle to East Coast Fever.
Vajana E(1)(2), Barbato M(1), Colli L(1), Milanesi M(1)(3)(4), Rochat E(2), Fabrizi E(5), Mukasa C(6), Del Corvo M(1), Masembe C(7), Muwanika VB(8), Kabi F(9), Sonstegard TS(10), Huson HJ(11), Negrini R(1)(12); NextGen Consortium; Joost S(2), Ajmone-Marsan P(1).
Author information:
(1)Department of Animal Science, Food and Nutrition (DIANA), Biodiversity and Ancient DNA Research Centre (BioDNA), and Proteomics and Nutrigenomics Research Centre (PRONUTRIGEN), Università Cattolica del Sacro Cuore, Piacenza, Italy.
(2)Laboratory of Geographic Information Systems (LASIG), School of Architecture, Civil and Environmental Engineering (ENAC), École Polytechnique Fédérale de Lausanne (EPFL), Lausanne, Switzerland.
(3)Department of Support, Production and Animal Health, School of Veterinary Medicine, São Paulo State University, Araçatuba, Brazil.
(4)International Atomic Energy Agency (IAEA), Collaborating Centre on Animal Genomics and Bioinformatics, Araçatuba, Brazil.
(5)Department of Economics and Social Sciences, Università Cattolica del Sacro Cuore, Piacenza, Italy.
(6)National Animal Genetic Resource Centre and Data Bank, Entebbe, Uganda.
(7)Department of Zoology, Entomology and Fisheries, Makerere University, Kampala, Uganda.
(8)Department of Environmental Management, Makerere University, Kampala, Uganda.
(9)National Livestock Resources Research Institute (NaLIRRI), National Agricultural Research Organisation, Tororo, Uganda.
(10)Recombinetics, Inc., St. Paul, MN, United States.
(11)Department of Animal Science, Cornell University, Ithaca, NY, United States.
(12)Associazione Italiana Allevatori (AIA), Rome, Italy.
East Coast fever (ECF) is a fatal sickness affecting cattle populations of eastern, central, and southern Africa. The disease is transmitted by the tick Rhipicephalus appendiculatus, and caused by the protozoan Theileria parva parva, which invades host lymphocytes and promotes their clonal expansion. Importantly, indigenous cattle show tolerance to infection in ECF-endemically stable areas. Here, the putative genetic bases underlying ECF-tolerance were investigated using molecular data and epidemiological information from 823 indigenous cattle from Uganda. Vector distribution and host infection risk were estimated over the study area and subsequently tested as triggers of local adaptation by means of landscape genomics analysis. We identified 41 and seven candidate adaptive loci for tick resistance and infection tolerance, respectively. Among the genes associated with the candidate adaptive loci are PRKG1 and SLA2. PRKG1 was already described as associated with tick resistance in indigenous South African cattle, due to its role into inflammatory response. SLA2 is part of the regulatory pathways involved into lymphocytes' proliferation. Additionally, local ancestry analysis suggested the zebuine origin of the genomic region candidate for tick resistance.

111. J Med Entomol. 2019 Apr 16;56(3):873-877. doi: 10.1093/jme/tjy221.
General and Local Morphological Anomalies in Amblyomma lepidum (Acari: Ixodidae) and Rhipicephalus decoloratus Infesting Cattle in Uganda.
Balinandi S(1)(2), Mugisha L(2)(3), Bbira J(2), Kabasa W(2), Nakayiki T(1), Bakkes DK(4), Lutwama JJ(1), Chitimia-Dobler L(5)(6), Malmberg M(7)(8).
Author information:
(1)Department of Arbovirology, Emerging and Reemrging Infections, Uganda Virus Research Institute, Entebbe, Uganda.
(2)College of Veterinary Medicine, Animal Resources & Biosecurity (COVAB), Makerere University, Kampala, Uganda.
(3)Ecohealth Research Group, Conservation & Ecosystem Health Alliance (CEHA), Kampala, Uganda.
(4)Gertrud Theiler Tick Museum, Epidemiology, Parasites and Vectors, Agricultural Research Council - Onderstepoort Veterinary Research, Pretoria, South Africa.
(5)Department of Virology-Rickettsiology, Bundeswehr Institute of Microbiology, Munich, Germany.
(6)Parasitology Unit, University of Hohenheim, Stuttgart, Germany.
(7)Section of Virology, Department of Biomedical Sciences and Veterinary Public Health, Swedish University of Agricultural Sciences, Uppsala, Sweden.
(8)SLU Global Bioinformatics Centre, Department of Animal Breeding and Genetics, Swedish University of Agricultural Sciences, Uppsala, Sweden.
Morphological abnormalities in ticks seem to be rare phenomena in nature, and are underreported in Africa. In this article, we describe general and local anomalies in two Amblyomma lepidum females and one Rhipicephalus decoloratus female collected from cattle in Moroto and Kasese districts, Uganda. One A. lepidum specimen displayed metagynander gynandromorphism with the presence of both male and female features in the same organism. The second A. lepidum female showed slight asymmetry and lacked a genital aperture. The R. decoloratus displayed multiple anomalies that included asymmetry on the right side in association with ectromely, chitinous formations and constrictions on the left side. This article presents the first report of metagynander gynandromorphism, as well as genital aperture absence which is not linked to gynandromorphism, in A. lepidum collected from cattle.

112. Viruses. 2019 Mar 2;11(3):209. doi: 10.3390/v11030209.
Discovery and Characterization of Bukakata orbivirus (Reoviridae:Orbivirus), a Novel Virus from a Ugandan Bat.
Fagre AC(1), Lee JS(2), Kityo RM(3), Bergren NA(4), Mossel EC(5), Nakayiki T(6), Nalikka B(7), Nyakarahuka L(8)(9), Gilbert AT(10), Peterhans JK(11), Crabtree MB(12), Towner JS(13), Amman BR(14), Sealy TK(15), Schuh AJ(16)(17), Nichol ST(18), Lutwama JJ(19), Miller BR(20), Kading RC(21)(22).
Author information:
(1)Department of Microbiology, Immunology, and Pathology, Colorado State University, Fort Collins, CO 80523, USA. anna.fagre@colostate.edu.
(2)Department of Microbiology, Immunology, and Pathology, Colorado State

University, Fort Collins, CO 80523, USA. justin.lee@colostate.edu.
(3)Department of Zoology, Entomology and Fisheries Sciences, Makerere University, Kampala, Uganda. kityrob@gmail.com.
(4)Department of Microbiology, Immunology, and Pathology, Colorado State University, Fort Collins, CO 80523, USA. nicholas.bergren@colostate.edu.
(5)Arboviral Diseases Branch, Division of Vector-borne Diseases, Centers for Disease Control and Prevention, Fort Collins, CO 80523, USA. ilv8@cdc.gov.
(6)Department of Arbovirology, Emerging, and Re-emerging Viral Infections, Uganda Virus Research Institute, Entebbe, Uganda. nakayikiteddie@yahoo.com.
(7)Department of Zoology, Entomology and Fisheries Sciences, Makerere University, Kampala, Uganda. bnalikka@gmail.com.
(8)Department of Arbovirology, Emerging, and Re-emerging Viral Infections, Uganda Virus Research Institute, Entebbe, Uganda. nyakarahuka@gmail.com.
(9)Department of Biosecurity, Ecosystems and Veterinary Public Health, Makerere University, Kampala, Uganda. nyakarahuka@gmail.com.
(10)National Wildlife Research Center, US Department of Agriculture, Animal and Plant Health Inspection Service, Wildlife Services, Fort Collins, CO 80521, USA. Amy.T.Gilbert@aphis.usda.gov.
(11)College of Arts and Sciences, Roosevelt University, Collections & Research, The Field Museum of Natural History, Chicago, IL 60605, USA. jkerbis@fieldmuseum.org.
(12)Arboviral Diseases Branch, Division of Vector-borne Diseases, Centers for Disease Control and Prevention, Fort Collins, CO 80523, USA. mbcrab@gmail.com.
(13)Viral Special Pathogens Branch, Division of High Consequence Pathogens and Pathology, Centers for Disease Control and Prevention, Atlanta, GA 30333, USA. jit8@cdc.gov.
(14)Viral Special Pathogens Branch, Division of High Consequence Pathogens and Pathology, Centers for Disease Control and Prevention, Atlanta, GA 30333, USA. cxx1@cdc.gov.
(15)Viral Special Pathogens Branch, Division of High Consequence Pathogens and Pathology, Centers for Disease Control and Prevention, Atlanta, GA 30333, USA. tss3@cdc.gov.
(16)Viral Special Pathogens Branch, Division of High Consequence Pathogens and Pathology, Centers for Disease Control and Prevention, Atlanta, GA 30333, USA. wuc2@cdc.gov.
(17)United States Public Health Service, Commissioned Corps, Rockville, MD 20852, USA. wuc2@cdc.gov.
(18)Viral Special Pathogens Branch, Division of High Consequence Pathogens and Pathology, Centers for Disease Control and Prevention, Atlanta, GA 30333, USA. Stn1@cdc.gov.
(19)Department of Arbovirology, Emerging, and Re-emerging Viral Infections, Uganda Virus Research Institute, Entebbe, Uganda. jjlutwama03@yahoo.com.
(20)Arboviral Diseases Branch, Division of Vector-borne Diseases, Centers for Disease Control and Prevention, Fort Collins, CO 80523, USA. millerbr1@msn.com.
(21)Department of Microbiology, Immunology, and Pathology, Colorado State University, Fort Collins, CO 80523, USA. rebekah.kading@colostate.edu.
(22)Arboviral Diseases Branch, Division of Vector-borne Diseases, Centers for

Disease Control and Prevention, Fort Collins, CO 80523, USA.
rebekah.kading@colostate.edu.
While serological and virological evidence documents the exposure of bats to medically-important arboviruses, their role as reservoirs or amplifying hosts is less well-characterized. We describe a novel orbivirus (Reoviridae:Orbivirus) isolated from an Egyptian fruit bat (Rousettus aegyptiacus leachii) trapped in 2013 in Uganda and named Bukakata orbivirus. This is the fifth orbivirus isolated from a bat, however genetic information had previously only been available for one bat-associated orbivirus. We performed whole-genome sequencing on Bukakata orbivirus and three other bat-associated orbiviruses (Fomede, Ife, and Japanaut) to assess their phylogenetic relationship within the genus Orbivirus and develop hypotheses regarding potential arthropod vectors. Replication kinetics were assessed for Bukakata orbivirus in three different vertebrate cell lines. Lastly, qRT-PCR and nested PCR were used to determine the prevalence of Bukakata orbivirus RNA in archived samples from three populations of Egyptian fruit bats and one population of cave-associated soft ticks in Uganda. Complete coding sequences were obtained for all ten segments of Fomede, Ife, and Japanaut orbiviruses and for nine of the ten segments for Bukakata orbivirus. Phylogenetic analysis placed Bukakata and Fomede in the tick-borne orbivirus clade and Ife and Japanaut within the Culicoides/phlebotomine sandfly orbivirus clade. Further, Bukakata and Fomede appear to be serotypes of the Chobar Gorge virus species. Bukakata orbivirus replicated to high titers (10^{6}–10^{7} PFU/mL) in Vero, BHK-21 [C-13], and R06E (Egyptian fruit bat) cells. Preliminary screening of archived bat and tick samples do not support Bukakata orbivirus presence in these collections, however additional testing is warranted given the phylogenetic associations observed. This study provided complete coding sequence for several bat-associated orbiviruses and in vitro characterization of a bat-associated orbivirus. Our results indicate that bats may play an important role in the epidemiology of viruses in the genus Orbivirus and further investigation is warranted into vector-host associations and ongoing surveillance efforts.

113. Front Cell Infect Microbiol. 2019 Mar 1;9:45. doi: 10.3389/fcimb.2019.00045.
Oral Vaccination With a Formulation Combining Rhipicephalus microplus Subolesin With Heat Inactivated Mycobacterium bovis Reduces Tick Infestations in Cattle.
Contreras M(1), Kasaija PD(1)(2), Merino O(3), de la Cruz-Hernandez NI(3), Gortazar C(1), de la Fuente J(1)(4).
Author information:
(1)SaBio, Instituto de Investigación en Recursos Cinegéticos IREC-CSIC-UCLM-JCCM, Ciudad Real, Spain.
(2)National Livestock Resources Research Institute (NaLIRRI/NARO), Tororo, Uganda.
(3)Facultad de Medicina Veterinaria y Zootecnia, Universidad Autónoma de Tamaulipas, Ciudad Victoria, Mexico.
(4)Department of Veterinary Pathobiology, Center for Veterinary Health Sciences, Oklahoma State University, Stillwater, OK, United States.
Vaccines are an environmentally friendly alternative to acaracides for the

control of tick infestations, to reduce the risk for tick-borne diseases affecting human and animal health worldwide, and to improve animal welfare and production. Subolesin (SUB, also known as 4D8) is the functional homolog of Akirin2 involved in the regulation of development and innate immune response, and a proven protective antigen for the control of ectoparasite infestations and pathogen infection. Oral vaccination combining protein antigens with immunostimulants has proven efficacy with increased host welfare and safety, but has not been used for the control of tick infestations. Here we describe the efficacy of oral vaccination with a formulation combining Rhipicephalus microplus SUB and heat inactivated Mycobacterium bovis (IV) on cattle tick infestations and fertility. The levels of IgG antibody titers against SUB and M. bovis P22, and the expression of selected immune response genes were determined and analyzed as possible correlates of protection. We demonstrated that oral immunization with the SUB+IV formulation resulted in 51% reduction in the number of female ticks and 30% reduction in fertility with an overall efficacy of 65% in the control of R. microplus infestations by considering the cumulative effect on reducing tick survival and fertility in cattle. The akr2, IL-1β, and C3 mRNA levels together with antibody levels against SUB correlated with vaccine efficacy. The effect of the oral immunization with SUB+IV in cattle on tick survival and fertility is essential to reduce tick infestations, and extended previous results on the effect of R. microplus SUB for the control of cattle tick infestations. These results support the development of oral vaccines formulations for the control of tick infestations and the incidence of tick-borne diseases.

114. Ticks Tick Borne Dis. 2019 Aug;10(5):949-958. doi: 10.1016/j.ttbdis.2019.04.016.
Inhibitory effects of Syzygium aromaticum and Camellia sinensis methanolic extracts on the growth of Babesia and Theileria parasites.
Batiha GE(1), Beshbishy AM(2), Tayebwa DS(3), Shaheen HM(4), Yokoyama N(2), Igarashi I(5).
Author information:
(1)National Research Center for Protozoan Diseases, Obihiro University of Agriculture and Veterinary Medicine, Nishi 2 Sen-11, Inada-cho, Obihiro, Hokkaido, 080-8555, Japan; Department of Pharmacology and Therapeutics, Faculty of Veterinary Medicine, Damanhour University, Al Beheira, Damanhour, 22511, Egypt.
(2)National Research Center for Protozoan Diseases, Obihiro University of Agriculture and Veterinary Medicine, Nishi 2 Sen-11, Inada-cho, Obihiro, Hokkaido, 080-8555, Japan.
(3)National Research Center for Protozoan Diseases, Obihiro University of Agriculture and Veterinary Medicine, Nishi 2 Sen-11, Inada-cho, Obihiro, Hokkaido, 080-8555, Japan; Research Center for Tick and Tick-borne diseases, College of Veterinary Medicine, Animal Resources and Biosecurity, Makerere University, PO BOX 7062, Kampala, Uganda.
(4)Department of Pharmacology and Therapeutics, Faculty of Veterinary Medicine, Damanhour University, Al Beheira, Damanhour, 22511, Egypt.
(5)National Research Center for Protozoan Diseases, Obihiro University of

Agriculture and Veterinary Medicine, Nishi 2 Sen-11, Inada-cho, Obihiro,
Hokkaido, 080-8555, Japan. Electronic address: igarcpmi@obihiro.ac.jp.
Currently, chemotherapeutics against piroplasmosis are also associated with toxicity and the emergence of drug-resistant parasites. Therefore, the discovery of new drug compounds is necessary for the effective control of bovine and equine piroplasms. Syzygium aromaticum (clove) and Camellia sinensis (green tea) have several documented medicinal properties. In the present study, the growth-inhibiting effects of S. aromaticum and C. sinensis methanolic extracts were evaluated in vitro and in vivo. The half-maximal inhibitory concentration (IC50) values for methanolic S. aromaticum against Babesia bovis, B. bigemina, B. divergens, B. caballi, and Theileria equi were 109.8 ± 3.8, 8.7 ± 0.09, 76.4 ± 4.5, 19.6 ± 2.2, and 60 ± 7.3 µg/ml, respectively. Methanolic C. sinensis exhibited IC50 values of 114 ± 6.1, 71.3 ± 3.7, 35.9 ± 6.8, 32.7 ± 20.3, and 60.8 ± 7.9 µg/ml against B. bovis, B. bigemina, B. divergens, B. caballi, and T. equi, respectively. The toxicity assay on Madin-Darby bovine kidney (MDBK), mouse embryonic fibroblast (NIH/3T3), and human foreskin fibroblast (HFF) cell lines showed that methanolic S. aromaticum and methanolic C. sinensis affected only the viability of the MDBK cell line with half-maximal effective concentrations (EC50) of 894.7 ± 4.9 and 473.7 ± 7.4 µg/ml, respectively, while the viability of NIH/3T3 and HFF cell lines was not affected even at 1000 µg/ml. In the in vivo experiment, methanolic S. aromaticum and methanolic C. sinensis oral treatments at 150 mg/kg inhibited the growth of Babesia microti in mice by 69.2% and 42.4%, respectively. These findings suggest that methanolic S. aromaticum and methanolic C. sinensis extracts have the potential as alternative remedies for treating piroplasmosis.

115. PLoS Negl Trop Dis. 2019 May 24;13(5):e0007030. doi:
10.1371/journal.pntd.0007030. eCollection 2019 May.
The effects of trans-chalcone and chalcone 4 hydrate on the growth of Babesia and Theileria.
Batiha GE(1)(2), Beshbishy AM(1), Tayebwa DS(1)(3), Adeyemi OS(4), Shaheen H(2), Yokoyama N(1), Igarashi I(1).
Author information:
(1)National Research Center for Protozoan Diseases, Obihiro University of Agriculture and Veterinary Medicine, Obihiro, Hokkaido, Japan.
(2)Department of Pharmacology and Therapeutics, Faculty of Veterinary Medicine, Damanhour University, Damanhour, AlBeheira, Egypt.
(3)Research Center for Tick and Tick-Borne Diseases, College of Veterinary Medicine, Animal Resources and Biosecurity, Makerere University, Kampala, Uganda.
(4)Medicinal Biochemistry, Nanomedicine and Toxicology Laboratory, Department of Biological Sciences Landmark University, Kwara State, Nigeria.
BACKGROUND: Chemotherapy is a principle tool for the control and prevention of piroplasmosis. The search for a new chemotherapy against Babesia and Theileria parasites has become increasingly urgent due to the toxic side effects of and developed resistance to the current drugs. Chalcones have attracted much attention due to their diverse biological activities. With the aim to discover

new drugs and drug targets, in vitro and in vivo antibabesial activity of trans-chalcone (TC) and chalcone 4 hydrate (CH) alone and combined with diminazene aceturate (DA), clofazimine (CF) and atovaquone (AQ) were investigated.
METHODOLOGY/PRINCIPAL FINDINGS: The fluorescence-based assay was used for evaluating the inhibitory effect of TC and CH on four Babesia species, including B. bovis, B. bigemina, B. divergens, B. caballi, and T. equi, the combination with DA, CF, and AQ on in vitro cultures, and on the multiplication of a B. microti-infected mouse model. The cytotoxicity of compounds was tested on Madin-Darby bovine kidney (MDBK), mouse embryonic fibroblast (NIH/3T3), and human foreskin fibroblast (HFF) cell lines. The half maximal inhibitory concentration (IC50) values of TC and CH against B. bovis, B. bigemina, B. divergens, B. caballi, and T. equi were 69.6 ± 2.3, 33.3 ± 1.2, 64.8 ± 2.5, 18.9 ± 1.7, and 14.3 ± 1.6 μM and 138.4 ± 4.4, 60.9 ± 1.1, 82.3 ± 2.3, 27.9 ± 1.2, and 19.2 ± 1.5 μM, respectively. In toxicity assays, TC and CH affected the viability of MDBK, NIH/3T3, and HFF cell lines the with half maximum effective concentration (EC50) values of 293.9 ± 2.9, 434.4 ± 2.7, and 498 ± 3.1 μM and 252.7 ± 1.7, 406.3 ± 9.7, and 466 ± 5.7 μM, respectively. In the mouse experiment, TC reduced the peak parasitemia of B. microti by 71.8% when administered intraperitoneally at 25 mg/kg. Combination therapies of TC-DA and TC-CF were more potent against B. microti infection in mice than their monotherapies.
CONCLUSIONS/SIGNIFICANCE: In conclusion, both TC and CH inhibited the growth of Babesia and Theileria in vitro, and TC inhibited the growth of B. microti in vivo. Therefore, TC and CH could be candidates for the treatment of piroplasmosis after further studies.

116. Trop Med Health. 2019 Jul 11;47:42. doi: 10.1186/s41182-019-0171-8. eCollection
Evaluation of the inhibitory effect of ivermectin on the growth of Babesia and Theileria parasites in vitro and in vivo.
Batiha GE(#)(1)(2), Beshbishy AM(#)(1), Tayebwa DS(1)(3), Adeyemi OS(4), Yokoyama N(1), Igarashi I(1).
Author information:
(1)1National Research Center for Protozoan Diseases, Obihiro University of Agriculture and Veterinary Medicine, Nishi 2-13, Inada-cho, Obihiro, Hokkaido 080-8555 Japan.
(2)2Department of Pharmacology and Therapeutics, Faculty of Veterinary Medicine, Damanhour University, Damanhour, El Beheira 22511 Egypt.
(3)3Research Center for Tick and Tick-Borne Diseases, College of Veterinary Medicine, Animal Resources and Biosecurity, Makerere University, PO Box 7062, Kampala, Uganda.
(4)4Medicinal Biochemistry, Nanomedicine and Toxicology Laboratory, Department of Biological Sciences, Landmark University, Omu-Aran, Kwara 251101 Nigeria.
(#)Contributed equally
BACKGROUND: Treatment is the principle way to control and eliminate piroplasmosis. The search for new chemotherapy against Babesia and Theileria has become increasingly urgent due to parasite resistance to current drugs.

Ivermectin (IVM) was the world's first endectocide, capable of killing a wide variety of parasites and vectors, both inside and outside the body. It is currently authorized to treat onchocerciasis, lymphatic filariasis, strongyloidiasis, and scabies. The current study documented the efficacy of IVM on the growth of Babesia and Theileria in vitro and in vivo.
METHODS: The fluorescence-based assay was used for evaluating the inhibitory effect of IVM on four Babesia species, including B. bovis, B. bigemina, B. divergens, B. caballi, and Theileria equi, the combination with diminazene aceturate (DA), clofazimine (CF), and atovaquone (AQ) on in vitro cultures, and on the multiplication of a B. microti-infected mouse model. The cytotoxicity of compounds was tested on Madin-Darby bovine kidney (MDBK), mouse embryonic fibroblast (NIH/3 T3), and human foreskin fibroblast (HFF) cell lines.
RESULTS: The half-maximal inhibitory concentration (IC50) values determined for IVM against B. bovis, B. bigemina, B. divergens, B. caballi, and T. equi were 53.3 ± 4.8, 98.6 ± 5.7, 30.1 ± 2.2, 43.7 ± 3.7, and 90.1 ± 8.1 µM, respectively. Toxicity assays on MDBK, NIH/3 T3, and HFF cell lines showed that IVM affected the viability of cells with a half-maximal effective concentration (EC50) of 138.9 ± 4.9, 283.8 ± 3.6, and 287.5 ± 7.6 µM, respectively. In the in vivo experiment, IVM, when administered intraperitoneally at 4 mg/kg, significantly ($p < 0.05$) inhibited the growth of B. microti in mice by 63%. Furthermore, combination therapies of IVM-DA, IVM-AQ, and IVM-CF at a half dose reduced the peak parasitemia of B. microti by 83.7%, 76.5%, and 74.4%, respectively. Moreover, this study confirmed the absence of B. microti DNA in groups treated with combination chemotherapy of IVM + DA and IVM + AQ 49 days after infection.
CONCLUSIONS: These findings suggest that IVM has the potential to be an alternative remedy for treating piroplasmosis.

117. Front Bioeng Biotechnol. 2019 Sep 26;7:236. doi: 10.3389/fbioe.2019.00236.
Identification of Ixodid Tick-Specific Aquaporin-1 Potential Anti-tick Vaccine Epitopes: An in-silico Analysis.
Ndekezi C(1), Nkamwesiga J(1), Ochwo S(1), Kimuda MP(1)(2), Mwiine FN(1), Tweyongyere R(3), Amanyire W(1), Muhanguzi D(1)(4).
Author information:
(1)School of Biosecurity, Biotechnical and Laboratory Science, College of Veterinary Medicine Animal Resources and Biosecurity, Makerere University, Kampala, Uganda.
(2)Research Unit in Bioinformatics, Department of Biochemistry and Microbiology, Rhodes University, Grahamstown, South Africa.
(3)School of Veterinary Medicine and Animal Resources, College of Veterinary Medicine Animal Resources and Biosecurity, Makerere University, Kampala, Uganda.
(4)Makerere University/Uganda Virus Research Institute Centre of Excellence in Infection and Immunity Research and Training, Entebbe, Uganda.
Ticks are arthropod vectors of pathogens of both Veterinary and Public health importance. Acaricide application, which is currently the mainstay of tick control, is hampered by high cost, the need for regular application and a selection of multi-acaricide resistant tick populations. In light of this, future tick control approaches are poised to rely on the integration of rational

acaricide application and other methods, such as vaccination. To contribute to systematic research-guided efforts to produce anti-tick vaccines, we carried out an in-silico analysis of tick aquaporin-1 (AQP1) protein in order to identify tick-specific AQP1 peptide motifs that can be used in future peptide anti-tick vaccine development. We carried out multiple sequence alignment (MSA), motif analysis, homology modeling, and structural analysis to identify tick-specific AQP1 peptide motifs. BepiPred, Chou and Fasman-Turn, Karplus and Schulz Flexibility, and Parker-Hydrophilicity prediction models were used to predict these motifs' potential to induce B cell mediated immune responses. The tick AQP1 (GenBankID: QDO67142.1) protein was largely similar to the bovine AQP1 (PDB:1J4N) (23 % sequence similarity; Structural superimposition of the homology model and 14JN homotetramers gave an RMSD = 3.269 while superimposition of a single chain gave an RMSD = 1.475). Tick and bovine AQP1 transmembrane domains were largely similar while their extracellular and cytoplasmic domain loops showed variation. Two tick-specific AQP1 peptide motifs; M7 (residues 106-125, p = 5.4e-25), and M8 (residues 85-104, p = 3.3e-24) were identified. These two motifs are located on the extra-cellular AQP1 domain. Motifs; M7 and M8 showed the highest Parker-Hydrophilicity prediction immunogenicity scores of 1.784 and 1.536, respectively. These two motifs can be a good starting point for the development of potential tick AQP1 peptide-based anti-tick vaccines. Further analyses such as molecular dynamics, in vitro assays, and in vivo immunization assays are required to validate these findings.

118. Vaccines (Basel). 2019 Oct 31;7(4):165. doi: 10.3390/vaccines7040165.
Towards a Multidisciplinary Approach to Improve Cattle Health and Production in Uganda.
de la Fuente J(1)(2), Contreras M(3), Kasaija PD(4)(5), Gortazar C(6), Ruiz-Fons JF(7), Mateo R(8), Kabi F(9).
Author information:
(1)SaBio, Instituto de Investigación en Recursos Cinegéticos (IREC), Consejo Superior de Investigaciones Científicas (CSIC), Universidad de Castilla-La Mancha (UCLM)-Junta de Comunidades de Castilla-La Mancha (JCCM), Ronda de Toledo s/n, 13005 Ciudad Real, Spain. jose_delafuente@yahoo.com.
(2)Department of Veterinary Pathobiology, Center for Veterinary Health Sciences, Oklahoma State University, Stillwater, OK 74078, USA. jose_delafuente@yahoo.com.
(3)SaBio, Instituto de Investigación en Recursos Cinegéticos (IREC), Consejo Superior de Investigaciones Científicas (CSIC), Universidad de Castilla-La Mancha (UCLM)-Junta de Comunidades de Castilla-La Mancha (JCCM), Ronda de Toledo s/n, 13005 Ciudad Real, Spain. marinelacr@hotmail.com.
(4)SaBio, Instituto de Investigación en Recursos Cinegéticos (IREC), Consejo Superior de Investigaciones Científicas (CSIC), Universidad de Castilla-La Mancha (UCLM)-Junta de Comunidades de Castilla-La Mancha (JCCM), Ronda de Toledo s/n, 13005 Ciudad Real, Spain. kpauldavis@gmail.com.
(5)National Livestock Resources Research Institute (NaLIRRI/NARO), Kampala 5704, Uganda. kpauldavis@gmail.com.
(6)SaBio, Instituto de Investigación en Recursos Cinegéticos (IREC), Consejo Superior de Investigaciones Científicas (CSIC), Universidad de Castilla-La

Mancha (UCLM)-Junta de Comunidades de Castilla-La Mancha (JCCM), Ronda de Toledo s/n, 13005 Ciudad Real, Spain. christian.gortazar@uclm.es.
(7)SaBio, Instituto de Investigación en Recursos Cinegéticos (IREC), Consejo Superior de Investigaciones Científicas (CSIC), Universidad de Castilla-La Mancha (UCLM)-Junta de Comunidades de Castilla-La Mancha (JCCM), Ronda de Toledo s/n, 13005 Ciudad Real, Spain. josefrancisco.ruiz@uclm.es.
(8)SaBio, Instituto de Investigación en Recursos Cinegéticos (IREC), Consejo Superior de Investigaciones Científicas (CSIC), Universidad de Castilla-La Mancha (UCLM)-Junta de Comunidades de Castilla-La Mancha (JCCM), Ronda de Toledo s/n, 13005 Ciudad Real, Spain. rafael.mateo@uclm.es.
(9)National Livestock Resources Research Institute (NaLIRRI/NARO), Kampala 5704, Uganda. freddykabi@gmail.com.
A meeting and course supported by the Vice-Presidency for International Affairs of the Spanish National Research Council (CSIC) and the National Agricultural Research Organization of Uganda (NARO) were held at the National Livestock Resources Research Institute (NaLIRRI) in Nakyesasa, Wakiso, Uganda on September 2-9, 2019. The activities were conducted within the collaboration program between the Institute of Game and Wildlife Research (IREC, CSIC-UCLM-JCCM, Spain) and NARO for the development of vaccines and other interventions for the control of cattle ticks in Uganda.

119. Viruses. 2019 Dec 31;12(1):48. doi: 10.3390/v12010048.
Novel Viruses Found in Antricola Ticks Collected in Bat Caves in the Western Amazonia of Brazil.
Blomström AL(1), Luz HR(2), Öhlund P(1), Lukenge M(1)(3), Brandão PE(2), Labruna MB(2), Berg M(1).
Author information:
(1)Department of Biomedical Sciences and Veterinary Public Health, Swedish University of Agricultural Sciences, Box 7028, 75007 Uppsala, Sweden.
(2)Departments of Preventive Veterinary Medicine and Animal Health, School of Veterinary Medicine and Animal Science, University of São Paulo, São Paulo P.O. 05508-270, Brazil.
(3)Vector Biology Unit, Division of Entomology, Uganda Virus Research Institute, Entebbe P.O. Box 49, Uganda.
In this study, we describe the viral composition of adult Antricoladelacruzi ticks collected in a hot bat cave in the state of Rondônia, Western Amazonia, Brazil. A.delacruzi ticks, are special, compared to many other ticks, in that they feed on both bats (larval blood feeding) and bat guano (nymphal and adult feeding) instead of feeding exclusively on vertebrate hosts (blood feeding). Considering this unique life-cycle it is potentially possible that these ticks can pick up/be infected by viruses not only present in the blood of viremic bats but also by virus shed through the bat guano. The viral metagenomic investigation of adult ticks showed that single-stranded negative-sense RNA viruses were the dominant group of viruses identified in the investigated ticks. Out of these, members of the Nairoviridae family were in clear majority constituting 88% of all viral reads in the data set. Genetic and phylogenetic analyses indicate the presence of several different orthonairoviruses in the

investigated ticks with only distant relationship to previously described ones. In addition, identification of viral sequences belonging to Orthomyxoviridae, Iflaviridae, Dicistroviridae, Polycipiviridae, Reoviridae and different unclassified RNA viruses showed the presence of viruses with low sequence similarity to previously described viruses.

120. J Virol. 2020 Mar 31;94(8):e02005-19. doi: 10.1128/JVI.02005-19. Print 2020 Mar
Structure and Characterization of Crimean-Congo Hemorrhagic Fever Virus GP38.
Mishra AK(1), Moyer CL(2), Abelson DM(2), Deer DJ(3), El Omari K(4), Duman R(4), Lobel L(5), Lutwama JJ(6), Dye JM(7), Wagner A(4), Chandran K(8), Cross RW(3), Geisbert TW(3), Zeitlin L(2), Bornholdt ZA(2), McLellan JS(9).
Author information:
(1)Department of Molecular Biosciences, University of Texas at Austin, Austin, Texas, USA.
(2)Mapp Biopharmaceutical, San Diego, California, USA.
(3)Department of Microbiology and Immunology, University of Texas Medical Branch, Galveston, Texas, USA.
(4)Diamond Light Source, Harwell Science and Innovation Campus, Didcot, Oxfordshire, United Kingdom.
(5)Department of Microbiology, Immunology and Genetics, Faculty of Health Sciences, Ben-Gurion University of the Negev, Beer-Sheva, Israel.
(6)Department of Arbovirology, Emerging and Re-emerging Infection, Uganda Virus Research Institute, Entebbe, Uganda.
(7)US Army Medical Research Institute of Infectious Diseases, Fort Detrick, Maryland, USA.
(8)Department of Microbiology and Immunology, Albert Einstein College of Medicine, Bronx, New York, USA.
(9)Department of Molecular Biosciences, University of Texas at Austin, Austin, Texas, USA jmclellan@austin.utexas.edu.
Crimean-Congo hemorrhagic fever virus (CCHFV) is the causative agent of the most widespread tick-borne viral infection in humans. CCHFV encodes a secreted glycoprotein (GP38) of unknown function that is the target of a protective antibody. Here, we present the crystal structure of GP38 at a resolution of 2.5 Å, which revealed a novel fold primarily consisting of a 3-helix bundle and a β-sandwich. Sequence alignment and homology modeling showed distant homology between GP38 and the ectodomain of Gn (a structural glycoprotein in CCHFV), suggestive of a gene duplication event. Analysis of convalescent-phase sera showed high titers of GP38 antibodies indicating immunogenicity in humans during natural CCHFV infection. The only protective antibody for CCHFV in an adult mouse model reported to date, 13G8, bound GP38 with subnanomolar affinity and protected against heterologous CCHFV challenge in a STAT1-knockout mouse model. Our data strongly suggest that GP38 should be evaluated as a vaccine antigen and that its structure provides a foundation to investigate functions of this protein in the viral life cycle.IMPORTANCE Crimean-Congo hemorrhagic fever virus (CCHFV) is a priority pathogen that poses a high risk to public health. Due to the high morbidity and mortality rates associated with CCHFV infection, there is an urgent need to develop medical countermeasures for disease prevention and

treatment. CCHFV GP38, a secreted glycoprotein of unknown function unique to the Nairoviridae family, was recently shown to be the target of a protective antibody against CCHFV. Here, we present the crystal structure of GP38, which revealed a novel fold with distant homology to another CCHFV glycoprotein that is suggestive of a gene duplication event. We also demonstrate that antibody 13G8 protects STAT1-knockout mice against heterologous CCHFV challenge using a clinical isolate from regions where CCHFV is endemic. Collectively, these data advance our understanding of GP38 structure and antigenicity and should facilitate future studies investigating its function.

121. Trop Med Infect Dis. 2020 Feb 7;5(1):24. doi: 10.3390/tropicalmed5010024.
Haemoparasitic Infections in Cattle from a Trypanosoma brucei Rhodesiense Sleeping Sickness Endemic District of Eastern Uganda.
Matovu E(1), Mugasa CM(1), Waiswa P(1), Kitibwa A(1), Boobo A(1), Ndung'u JM(2).
Author information:
(1)College of Veterinary Medicine, Animal Resources and Biosecurity, Makerere University Kampala, P. O. Box 7062 Kampala, Uganda.
(2)Foundation for Innovative New Diagnostics, Campus Biotech, Chemin des Mines 9, CH 1202 Geneva, Switzerland.
We carried out a baseline survey of cattle in Kaberamaido district, in the context of controlling the domestic animal reservoir of Trypanosoma brucei rhodesiense human African trypanosomiasis (rHAT) towards elimination. Cattle blood was subjected to capillary tube centrifugation followed by measurement of the packed cell volume (PCV) and examination of the buffy coat area for motile trypanosomes. Trypanosomes were detected in 561 (21.4%) out of 2621 cattle screened by microscopy. These 561 in addition to 724 apparently trypanosome negative samples with low PCVs (≤25%) were transported to the laboratory and tested by PCR targeting the trypanosomal Internal Transcribed Spacer (ITS-1) as well as suspect Tick-Borne Diseases (TBDs) including Anaplasmamosis, Babesiosis, and Theileriosis. PCR for Anaplasma sp yielded the highest number of positive animals (45.2%), followed by Trypanosoma sp (44%), Theileria sp (42.4%) and Babesia (26.3%); multiple infections were a common occurrence. Interestingly, 373 (29%) of these cattle with low PCVs were negative by PCR, pointing to other possible causes of aneamia, such as helminthiasis. Among the trypanosome infections classified as T. brucei by ITS-PCR, 5.5% were positive by SRA PCR, and were, therefore, confirmed as T. b. rhodesiense. Efforts against HAT should therefore consider packages that address a range of conditions. This may enhance acceptability and participation of livestock keepers in programs to eliminate this important but neglected tropical disease. In addition, we demonstrated that cattle remain an eminent reservoir for T. b. rhodesiense in eastern Uganda, which must be addressed to sustain HAT elimination.

122. Transbound Emerg Dis. 2020 Sep;67(5):2233-2239. doi: 10.1111/tbed.13546. Epub
Host range and geographical distribution of Babesia sp. Mymensingh.
Sivakumar T(1), Tuvshintulga B(1), Kothalawala H(2), Silva SSP(2), Lan DTB(3), Long PT(3), Ybañez AP(4), Ybañez RHD(1), Benitez DF(5), Tayebwa DS(6), De Macedo ACC(7), Schnittger L(8)(9), Yokoyama N(1)(10).

Author information:
(1)National Research Center for Protozoan Diseases, Obihiro University of Agriculture and Veterinary Medicine, Hokkaido, Japan.
(2)Veterinary Research Institute, Peradeniya, Sri Lanka.
(3)University of Agriculture and Forestry, Hue University, Hue, Vietnam.
(4)College of Veterinary Medicine and Institute of Molecular Parasitology and Vector-borne Diseases, Cebu Technological University, Cebu City, Philippines.
(5)Estacion Experimental Agropecuaria (EEA)-Mercedes, Instituto Nacional de Tecnologia Agropecuaria (INTA), Mercedes, Argentina.
(6)Research Center for Tick and Tick-borne Diseases, College of Veterinary Medicine, Animal Resources and Biosecurity, Makerere University, Kampala, Uganda.
(7)Conselho Regional de Medicina Veterinária da Bahia, Bahia, Brazil.
(8)Instituto de Patobiología Veterinaria, (CICVyA), Instituto Nacional de Tecnología Agropecuaria (INTA), Hurlingham, Argentina.
(9)Consejo Nacional de Investigaciones Científicas y Técnicas (CONICET), Buenos Aires, Argentina.
(10)OIE Reference Laboratory for Bovine Babesiosis and Equine Piroplasmosis, National Research Center for Protozoan Diseases, Obihiro University of Agriculture and Veterinary Medicine, Hokkaido, Japan.
Bovine babesiosis represents a serious threat to the cattle industry in the tropics and subtropics. Although several Babesia species infect cattle, only B. bovis, B. bigemina and B. divergens are known to cause clinical babesiosis. However, our recent study demonstrated that the newly discovered Babesia sp. Mymensingh might be a virulent species capable of causing clinical babesiosis in cattle. The objective of this study was to determine the host range and geographical distribution of Babesia sp. Mymensingh on a global scale. A total of 2,860 archived DNA samples from 2,263 cattle in Sri Lanka (n = 672), the Philippines (n = 408), Vietnam (n = 460), Uganda (n = 409), Brazil (n = 164) and Argentina (n = 150); 419 buffalo in Sri Lanka (n = 327) and Vietnam (n = 92); and 127 goats and 51 sheep in Vietnam were screened using a Babesia sp. Mymensingh-specific PCR assay. Babesia sp. Mymensingh infection was detected in cattle, buffalo, sheep and goats. Cattle of all countries surveyed in this study except Brazil were found to be infected with Babesia sp. Mymensingh. The highest positive rates were recorded in cattle from the Philippines (11.3%) and Vietnam (9.6%), followed by Argentina (4.7%), Sri Lanka (1.5%) and Uganda (1.0%). Buffalo were found to be infected with this parasite in Sri Lanka (1.2%) and Vietnam (10.9%). Unexpectedly, Babesia sp. Mymensingh was also detected in sheep (2.0%) and goats (1.3%) from Vietnam. These findings were confirmed by PCR amplicon sequencing. In conclusion, our present findings indicate that Babesia sp. Mymensingh, which infects cattle, buffalo, sheep and goats, is endemic in Asia, Africa and South America.

123. Transbound Emerg Dis. 2020 Mar;67 Suppl 1:99-107. doi: 10.1111/tbed.13311.
Antigen gene and variable number tandem repeat (VNTR) diversity in Theileria parva parasites from Ankole cattle in south-western Uganda: Evidence for conservation in antigen gene sequences combined with extensive polymorphism at

VNTR loci.

Nanteza A(1), Obara I(2), Kasaija P(1), Mwega E(3), Kabi F(4), Salih DA(5), Njahira M(6), Njuguna J(6), Odongo D(7), Bishop RP(8), Skilton RA(6), Ahmed J(1), Clausen PH(2), Lubega GW(1).
Author information:
(1)College of Veterinary Medicine, Animal Resources and Biosecurity, Makerere University, Kampala, Uganda.
(2)Institute for Parasitology and Tropical Veterinary Medicine, Freie Universität Berlin, Berlin, Germany.
(3)Faculty of Veterinary Medicine, Sokoine University of Agriculture, Morogoro, Tanzania.
(4)College of Natural Sciences, Makerere University, Kampala, Uganda.
(5)Central Veterinary Research Laboratories, Khartoum, Sudan.
(6)Biosciences East and Central Africa (BecA), Nairobi, Kenya.
(7)School of Biological Sciences, University of Nairobi, Nairobi, Kenya.
(8)Department of Veterinary Microbiology and Pathology, Washington State University, Pullman, WA, USA.

Theileria parva is a tick-transmitted apicomplexan protozoan parasite that infects lymphocytes of cattle and African Cape buffalo (Syncerus caffer), causing a frequently fatal disease of cattle in eastern, central and southern Africa. A live vaccination procedure, known as infection and treatment method (ITM), the most frequently used version of which comprises the Muguga, Serengeti-transformed and Kiambu 5 stocks of T. parva, delivered as a trivalent cocktail, is generally effective. However, it does not always induce 100% protection against heterologous parasite challenge. Knowledge of the genetic diversity of T. parva in target cattle populations is therefore important prior to extensive vaccine deployment. This study investigated the extent of genetic diversity within T. parva field isolates derived from Ankole (Bos taurus) cattle in south-western Uganda using 14 variable number tandem repeat (VNTR) satellite loci and the sequences of two antigen-encoding genes that are targets of CD8+T-cell responses induced by ITM, designated Tp1 and Tp2. The findings revealed a T. parva prevalence of 51% confirming endemicity of the parasite in south-western Uganda. Cattle-derived T. parva VNTR genotypes revealed a high degree of polymorphism. However, all of the T. parva Tp1 and Tp2 alleles identified in this study have been reported previously, indicating that they are widespread geographically in East Africa and highly conserved.

124. Transbound Emerg Dis. 2020 Mar;67 Suppl 1:88-98. doi: 10.1111/tbed.13417.
Multilocus genotyping of Theileria parva isolates associated with a live vaccination trial in Kenya provides evidence for transmission of immunizing parasites into local tick and cattle populations.

Bishop RP(1)(2), Odongo DO(1)(3), Spooner PR(1), Morzaria SP(1), Oura CAL(4), Skilton RA(1).
Author information:
(1)International Livestock Research Institute (ILRI), Nairobi, Kenya.
(2)Department of Veterinary Microbiology and Pathology, Washington State University, Pullman, WA, USA.

(3)School of Biological Sciences, University of Nairobi, Nairobi, Kenya.
(4)Faculty of Medical Sciences, School of Veterinary Medicine, University of the West Indies, St Augustine, Trinidad and Tobago.
The live infection and treatment (ITM) vaccination procedure using the trivalent Muguga cocktail is increasingly being used to control East Coast fever, with potential implications for Theileria parva population genetic structure in the field. Transmission of the Kiambu V T. parva component to unvaccinated cattle has previously been described in Uganda. We monitored the T. parva carrier state in vaccinated and control animals on a farm in West Kenya where an ITM stabilate derived from the Kenyan T. parva Marikebuni stock was evaluated for field efficacy. A nested PCR-based Marikebuni-specific marker identified a carrier state in nine of ten vaccinated animals, detectable for a period of two years. We used 22 variable number tandem repeat (VNTR) markers to determine multilocus genotypes (MLGs) of 19 T. parva schizont-infected lymphocyte isolates derived from cattle and field ticks. Two isolates from unimmunized cattle were identical to the Marikebuni vaccination stock. Two cattle isolates were identical to a Muguga cocktail component Kiambu V. Seven isolates from ticks exhibited MLGs that were identical to the Serengeti/Muguga vaccine stocks. Six cattle and two tick-derived stocks exhibited unique MLGs. The data strongly suggest transmission of immunizing genotypes, from Marikebuni vaccine-induced carrier cattle to unimmunized cattle. It is possible that genotypes similar to those in the Muguga cocktail are present in the field in Western Kenya. An alternative hypothesis is that these parasites may have originated from vaccine trial sites in Eastern Uganda. If correct, this suggests that T. parva stocks used for immunization can potentially be disseminated 125 km beyond the immediate vaccination site. Regardless of their origin, the data provide evidence that genotypes similar to those in the Muguga cocktail are circulating in the field in East Africa, alleviating concerns about dissemination of 'alien' T. parva germplasm through live vaccination.

125. Transbound Emerg Dis. 2020 Mar;67 Suppl 1:56-67. doi: 10.1111/tbed.13325.
A review of recent research on Theileria parva: Implications for the infection and treatment vaccination method for control of East Coast fever.
Bishop RP(1), Odongo D(2), Ahmed J(3), Mwamuye M(3), Fry LM(1)(4), Knowles DP(1), Nanteza A(5), Lubega G(5), Gwakisa P(6), Clausen PH(3), Obara I(3).
Author information:
(1)Department of Veterinary Microbiology & Pathology, Washington State University, Pullman, WA, USA.
(2)School of Biological Sciences, University of Nairobi, Nairobi, Kenya.
(3)Institute for Parasitology and Tropical Veterinary Medicine, Freie Universität Berlin, Berlin, Germany.
(4)Animal Disease Research Unit, Agricultural Research Service, US Department of Agriculture, Pullman, WA, USA.
(5)College of Veterinary Medicine, Animal Resources and Biosecurity, Makerere University, Kampala, Uganda.
(6)Genome Science Laboratory, College of Veterinary Medicine and Biomedical Sciences, Sokoine University of Agriculture, Morogoro, Tanzania.

The infection and treatment (ITM) live vaccination method for control of Theileria parva infection in cattle is increasingly being adopted, particularly in Maasai pastoralist systems. Several studies indicate positive impacts on human livelihoods. Importantly, the first detailed protocol for live vaccine production at scale has recently been published. However, quality control and delivery issues constrain vaccination sustainability and deployment. There is evidence that the distribution of T. parva is spreading from endemic areas in East Africa, North into Southern Sudan and West into Cameroon, probably as a result of anthropogenic movement of cattle. It has also recently been demonstrated that in Kenya, T. parva derived from cape buffalo can 'breakthrough' the immunity induced by ITM. However, in Tanzania, breakthrough has not been reported in areas where cattle co-graze with buffalo. It has been confirmed that buffalo in northern Uganda national parks are not infected with T. parva and R. appendiculatus appears to be absent, raising issues regarding vector distribution. Recently, there have been multiple field population genetic studies using variable number tandem repeat (VNTR) sequences and sequencing of antigen genes encoding targets of CD8+ T-cell responses. The VNTR markers generally reveal high levels of diversity. The antigen gene sequences present within the trivalent Muguga cocktail are relatively conserved among cattle transmissible T. parva populations. By contrast, greater genetic diversity is present in antigen genes from T. parva of buffalo origin. There is also evidence from several studies for transmission of components of stocks present within the Muguga cocktail, into field ticks and cattle following induction of a carrier state by immunization. In the short term, this may increase live vaccine effectiveness, through a more homogeneous challenge, but the long-term consequences are unknown.

126. Ticks Tick Borne Dis. 2020 Jul;11(4):101415. doi: 10.1016/j.ttbdis.2020.101415.
Improved detection of Babesia bigemina from various geographical areas in Africa using quantitative PCR and reverse line blot hybridisation.
Stoltsz H(1), Byaruhanga C(2), Troskie M(1), Makgabo M(1), Oosthuizen MC(1), Collins NE(1), Neves L(3).
Author information:
(1)Vectors and Vector-Borne Diseases Research Programme, Department of Veterinary Tropical Diseases, Faculty of Veterinary Science, University of Pretoria, Private Bag X04, Onderstepoort, 0110, South Africa.
(2)Vectors and Vector-Borne Diseases Research Programme, Department of Veterinary Tropical Diseases, Faculty of Veterinary Science, University of Pretoria, Private Bag X04, Onderstepoort, 0110, South Africa; National Agricultural Research Organisation, P.O. Box 259, Entebbe, Uganda. Electronic address: cbyaruhanga27@yahoo.com.
(3)Vectors and Vector-Borne Diseases Research Programme, Department of Veterinary Tropical Diseases, Faculty of Veterinary Science, University of Pretoria, Private Bag X04, Onderstepoort, 0110, South Africa; Biotechnology Center, Eduardo Mondlane University, Maputo, Mozambique.
Babesia bigemina is one of the aetiological agents of bovine babesiosis, which causes economic losses through mortality, loss of production and control costs.

Effective means of detecting and quantifying B. bigemina in cattle populations is therefore important to inform control approaches. In order to examine the parasite genetic diversity in African countries, B. bigemina 18S rRNA genes from cattle from South Africa, Uganda and Angola were sequenced. The 25 distinct B. bigemina 18S rRNA gene sequences obtained in this study showed 99 to 100% identity with previously published sequences of strains from African and other continents. The sequences of the previously published B. bigemina 18S rRNA gene-specific quantitative PCR (qPCR) primers and probe, developed based on American and Asian strains, were conserved in the African B. bigemina sequences. The qPCR assay was evaluated using 10-fold and 2-fold serial dilutions of B. bigemina-infected erythrocytes to determine the efficiency and analytical sensitivity. The qPCR assay had an efficiency of 98.14 ± 1.71%, and the limit of detection was approximately 1.5 infected red blood cells (iRBCs) per microlitre (µl) of blood. The detection rate of B. bigemina from duplicates of field-collected blood samples from cattle from South Africa, Mozambique and Angola was 37% (30/81), 12% (6/49) and 50% (38/76), respectively. Reverse line blot hybridisation (RLB) results obtained from the same samples in previous studies, using a previously published B. bigemina-specific probe, detected the parasite DNA in only 1.5% (3/206) of the samples. A new B. bigemina-specific RLB oligonucleotide probe was designed in the hypervariable V4 region of the 18S rRNA gene. Screening of field blood samples from cattle showed that the new probe was specific, and its frequency of detection of B. bigemina was three times higher than the previously published probe. The qPCR assay and the newly developed B. bigemina-specific RLB probe provide good tools for epidemiological studies, which are essential in the control of bovine babesiosis.

127. Parasit Vectors. 2020 Apr 3;13(1):165. doi: 10.1186/s13071-020-04043-z.
Invasive cattle ticks in East Africa: morphological and molecular confirmation of the presence of Rhipicephalus microplus in south-eastern Uganda.
Muhanguzi D(1), Byaruhanga J(1), Amanyire W(1), Ndekezi C(1), Ochwo S(1), Nkamwesiga J(1), Mwiine FN(1), Tweyongyere R(1), Fourie J(2), Madder M(3)(4), Schetters T(5)(6), Horak I(6), Juleff N(7), Jongejan F(6)(8).
Author information:
(1)College of Veterinary Medicine Animal Resources and Biosecurity, Makerere University, P.O. Box 7062, Kampala, Uganda.
(2)ClinVet International (Pty) Ltd, P.O. Box 11186, Universitas, 9321, Bloemfontein, South Africa.
(3)Clinglobal, B03/04, The Tamarin Commercial Hub, Jacaranda Avenue, Tamarin, 90903, Mauritius. Maxime.Madder@clinglobal.com.
(4)Department of Veterinary Tropical Diseases, Faculty of Veterinary Science, University of Pretoria, Private Bag X04, Onderstepoort, 0110, South Africa. Maxime.Madder@clinglobal.com.
(5)Clinglobal, B03/04, The Tamarin Commercial Hub, Jacaranda Avenue, Tamarin, 90903, Mauritius.
(6)Department of Veterinary Tropical Diseases, Faculty of Veterinary Science, University of Pretoria, Private Bag X04, Onderstepoort, 0110, South Africa.
(7)Bill & Melinda Gates Foundation, Seattle, WA, USA.

(8)Utrecht Centre for Tick-borne Diseases (UCTD), Faculty of Veterinary
Medicine, Utrecht University, Yalelaan 1, 3584 CL, Utrecht, The Netherlands.
BACKGROUND: Rhipicephalus microplus, an invasive tick species of Asian origin and the main vector of Babesia species, is considered one of the most widespread ectoparasites of livestock. The tick has spread from its native habitats on translocated livestock to large parts of the tropical world, where it has replaced some of the local populations of Rhipicephalus decoloratus ticks. Although the tick was reported in Uganda 70 years ago, it has not been found in any subsequent surveys. This study was carried out to update the national tick species distribution on livestock in Uganda as a basis for tick and tick-borne disease control, with particular reference to R. microplus.
METHODS: The study was carried out in Kadungulu, Serere district, south-eastern Uganda, which is dominated by small scale livestock producers. All the ticks collected from 240 cattle from six villages were identified microscopically.
Five R. microplus specimens were further processed for phylogenetic analysis and species confirmation.
RESULTS: The predominant tick species found on cattle was Rhipicephalus appendiculatus (86.9 %; n = 16,509). Other species found were Amblyomma variegatum (7.2 %; n = 1377), Rhipicephalus evertsi (2.3 %; n = 434) and R. microplus (3.6 %; n = 687). Phylogenetic analysis of the 12S rRNA, 16S rRNA and ITS2 gene sequences of R. microplus confirmed the morphological identification.
CONCLUSIONS: It is concluded that R. microplus has replaced R. decoloratus in the sampled villages in Kadungulu sub-county, since the latter was not any longer found in this area. There is currently no livestock movement policy in force in Uganda, which could possibly limit the further spread of R. microplus ticks. Future surveys, but also retrospective surveys of museum specimens, will reveal the extent of distribution of R. microplus in Uganda and also for how long this tick has been present on livestock without being noticed.

128. Ticks Tick Borne Dis. 2020 Jul;11(4):101438. doi: 10.1016/j.ttbdis.2020.101438.
Effect of chemical tick control practices on tick infestation and Theileria parva infection in an intensive dairy production region of Uganda.
Miyama T(1), Byaruhanga J(2), Okamura I(3), Uchida L(4), Muramatsu Y(5), Mwebembezi W(6), Vudriko P(7), Makita K(8).
Author information:
(1)Graduate School of Veterinary Medicine, Rakuno Gakuen University, 582 Bunkyodai Midorimachi, Ebetsu, Hokkaido, 069-8501, Japan. Electronic address: s21641006@g.rakuno.ac.jp.
(2)Research Center for Tropical Diseases and Vector Control, Department of Veterinary Pharmacy, Clinical and Comparative Medicine, College of Veterinary Medicine, Animal Resources and Biosecurity, Makerere University, Kampala, Uganda; Rakuno Gakuen University, 582 Bunkyodai Midorimachi, Ebetsu, Hokkaido 069-8501, Japan. Electronic address: josephjbvincent@gmail.com.
(3)Rakuno Gakuen University, 582 Bunkyodai Midorimachi, Ebetsu, Hokkaido 069-8501, Japan. Electronic address: m.hana-0702@bf6.so-net.ne.jp.
(4)Zoonotic Diseases Unit, School of Veterinary Medicine, Rakuno Gakuen University, 582 Bunkyodai Midorimachi, Ebetsu, 069-8501, Japan. Electronic

address: uchidaleo@rakuno.ac.jp.
(5)Zoonotic Diseases Unit, School of Veterinary Medicine, Rakuno Gakuen University, 582 Bunkyodai Midorimachi, Ebetsu, 069-8501, Japan. Electronic address: y-mrmt@rakuno.ac.jp.
(6)Mbarara District Veterinary Office, Mbarara District Local Government, Galt Road plot 5 Boma Hill, P.O. Box 1, Mbarara, Uganda. Electronic address: mwebembeziwilly@yahoo.co.uk.
(7)Research Center for Tropical Diseases and Vector Control, Department of Veterinary Pharmacy, Clinical and Comparative Medicine, College of Veterinary Medicine, Animal Resources and Biosecurity, Makerere University, Kampala, Uganda. Electronic address: vpato2009@gmail.com.
(8)Graduate School of Veterinary Medicine, Rakuno Gakuen University, 582 Bunkyodai Midorimachi, Ebetsu, Hokkaido, 069-8501, Japan. Electronic address: kmakita@rakuno.ac.jp.
Chemical tick control is a major means of preventing East Coast fever (ECF), especially in sub-Saharan Africa. However, in southwestern Uganda, improper tick control practices have led to severe acaricide resistance. The objectives of this study were to determine the risk factors associated with tick infestation in dairy cattle and Theileria parva infection, and to generate evidence for the prioritization of effective countermeasures for tick control. A cross-sectional study was conducted in 30 farms in Mbarara District, and information on tick control practices and tick infestation were collected. Tick samples were collected from 13 farms to test tick acaricide efficacy. A total of 420 blood samples from calves to adults of exotic- and cross-breed dairy cattle were collected, and T. parva diagnosis via polymerase chain reaction was performed. All the 13 tick populations tested were resistant to deltamethrin (synthetic pyrethroid). Resistance to single-formulation organophosphate-chlorfenvinphos was 39 % (5/13); co-formulations (chlorpyrifos + cypermethrin), 69 % (9/13); and amitraz (amidine), 85 % (11/13). The overall prevalence of T. parva infection at the individual-level was 45.2 % (190/420, 95 % confidence interval (CI): 40.4-50.1), and that at the farm-level was 83 % (25/30, 95 %CI: 65-94). A good quality cattle crush was a preventive factor for tick infestation (odds ratio (OR): 0.32, 95 %CI: 0.15-0.63, p = 0.001). Well-managed acaricide storage (OR: 0.36, 95 %CI: 0.17-0.76, p = 0.008), and a good quality measuring cylinder for acaricide were preventive factors (OR: 0.32, 95 %CI: 0.11-0.93, p = 0.036) for T. parva infection. The risk factors for T. parva infection were a longer period of acaricide use of the same brand (OR: 1.06, 95 %CI: 1.01-1.10, p = 0.012), and a higher frequency (twice a week) of acaricide use rather than once a week (OR: 11.70, 95 %CI: 1.95-70.13, p = 0.007). These risk factors should be given high intervention priority in order to effectively control ticks and prevent T. parva infections in dairy farms. Teaching proper practices to dairy farmers and to technical staff should be used to overcome the severe challenge of acaricide resistance in Mbarara District.

129. Parasitol Res. 2020 Jul;119(7):2363-2367. doi: 10.1007/s00436-020-06728-x. Epub
The Rhipicephalus appendiculatus tick vector of Theileria parva is absent from cape buffalo (Syncerus caffer) populations and associated ecosystems in northern

Uganda.
Obara I(1), Githaka N(2), Nijhof A(3), Krücken J(3), Nanteza A(4), Odongo D(5), Lubembe D(5), Atimnedi P(6), Mijele D(7), Njeri A(2), Mwaura S(2), Owido G(2), Ahmed J(3), Clausen PH(3), Bishop RP(8).
Author information:
(1)Institute for Parasitology and Tropical Veterinary Medicine, Freie Universität Berlin, Robert-von-Ostertag-Str. 7-13, 14163, Berlin, Germany. i.b.obara@gmail.com.
(2)International Livestock Research Institute (ILRI), Nairobi, Kenya.
(3)Institute for Parasitology and Tropical Veterinary Medicine, Freie Universität Berlin, Robert-von-Ostertag-Str. 7-13, 14163, Berlin, Germany.
(4)College of Veterinary Medicine, Animal Resources and Biosecurity, Makerere University, Kampala, Uganda.
(5)School of Biological Sciences, University of Nairobi, Nairobi, Kenya.
(6)Uganda Wildlife Authority, Kampala, Uganda.
(7)Kenya Wildlife Service, Nairobi, Kenya.
(8)Department of Veterinary Microbiology & Pathology, Washington State University, Pullman, WA, USA.
Rhipicephalus appendiculatus is the major tick vector of Theileria parva, an apicomplexan protozoan parasite that causes the most economically important and lethal disease of cattle in East and central Africa. The African cape buffalo (Syncerus caffer) is the major wildlife host of T. parva from southern Uganda and Kenya to southern Africa. We show herein that R. appendiculatus appears to be absent from the two largest national parks in northern Uganda. Syncerus caffer is common in both of these national parks, specifically Murchison falls (MFNP) and Kidepo Valley (KVNP). We re-confirmed the previously reported absence of T. parva in buffalo sampled in the two northern parks based on RLB data using a nested PCR based on the T. parva p104 gene. By contrast, T. parva-infected R. appendiculatus ticks and parasite-infected buffalo were present in Lake Mburo (LMNP) in South central Uganda. This suggests that the distribution of R. appendiculatus, which is predicted to include the higher rainfall regions of northern Uganda, may be limited by additional, as yet unknown factors.

130. Parasitol Res. 2020 Aug;119(8):2411-2420. doi: 10.1007/s00436-020-06742-z. Epub
Morphological and molecular identification of ixodid tick species (Acari: Ixodidae) infesting cattle in Uganda.
Balinandi S(1)(2), Chitimia-Dobler L(3), Grandi G(4), Nakayiki T(1), Kabasa W(2), Bbira J(2), Lutwama JJ(1), Bakkes DK(5)(6), Malmberg M(7)(8), Mugisha L(2)(9).
Author information:
(1)Uganda Virus Research Institute, P.O. Box 49, Entebbe, Uganda.
(2)College of Veterinary Medicine, Animal Resources and Biosecurity, Makerere University, P.O. Box 7062, Kampala, Uganda.
(3)Bundeswehr Institute of Microbiology, Neuherbergstrasse 11, 80937, Munich, Germany.
(4)Section of Virology, Department of Biomedical Sciences and Veterinary Public Health, Swedish University of Agricultural Sciences, Box 7028, 750 07, Uppsala,

Sweden.
(5)Gertrud Theiler Tick Museum, Epidemiology, Parasites and Vectors, Agricultural Research Council - Onderstepoort Veterinary Research, Pretoria, 0110, South Africa.
(6)Evolutionary Genomics Group, Department of Botany and Zoology, Stellenbosch University, Merriman Street, Private Bag X1, Stellenbosch, 7602, South Africa.
(7)Section of Virology, Department of Biomedical Sciences and Veterinary Public Health, Swedish University of Agricultural Sciences, Box 7028, 750 07, Uppsala, Sweden. maja.malmberg@slu.se.
(8)SLU Global Bioinformatics Centre, Department of Animal Breeding and Genetics, Swedish University of Agricultural Sciences, Box 7023, 750 07, Uppsala, Sweden. maja.malmberg@slu.se.
(9)Ecohealth Research Group, Conservation & Ecosystem Health Alliance, P.O. Box 34153, Kampala, Uganda.
In Uganda, the role of ticks in zoonotic disease transmission is not well described, partly, due to limited available information on tick diversity. This study aimed to identify the tick species that infest cattle. Between September and November 2017, ticks (n = 4362) were collected from 5 districts across Uganda (Kasese, Hoima, Gulu, Soroti, and Moroto) and identified morphologically at Uganda Virus Research Institute. Morphological and genetic validation was performed in Germany on representative identified specimens and on all unidentified ticks. Ticks were belonging to 15 species: 8 Rhipicephalus species (Rhipicephalus appendiculatus, Rhipicephalus evertsi evertsi, Rhipicephalus microplus, Rhipicephalus decoloratus, Rhipicephalus afranicus, Rhipicephalus pulchellus, Rhipicephalus simus, and Rhipicephalus sanguineus tropical lineage); 5 Amblyomma species (Amblyomma lepidum, Amblyomma variegatum, Amblyomma cohaerens, Amblyomma gemma, and Amblyomma paulopunctatum); and 2 Hyalomma species (Hyalomma rufipes and Hyalomma truncatum). The most common species were R. appendiculatus (51.8%), A. lepidum (21.0%), A. variegatum (14.3%), R. evertsi evertsi (8.2%), and R. decoloratus (2.4%). R. afranicus is a new species recently described in South Africa and we report its presence in Uganda for the first time. The sequences of R. afranicus were 2.4% divergent from those obtained in Southern Africa. We confirm the presence of the invasive R. microplus in two districts (Soroti and Gulu). Species diversity was highest in Moroto district (p = 0.004) and geographical predominance by specific ticks was observed (p = 0.001). The study expands the knowledge on tick fauna in Uganda and demonstrates that multiple tick species with potential to transmit several tick-borne diseases including zoonotic pathogens are infesting cattle.

131. Vaccines (Basel). 2020 Jun 18;8(2):319. doi: 10.3390/vaccines8020319.
Vaccination with Recombinant Subolesin Antigens Provides Cross-Tick Species Protection in Bos indicus and Crossbred Cattle in Uganda.
Kasaija PD(1)(2), Contreras M(1)(3), Kabi F(2), Mugerwa S(2), de la Fuente J(1)(4).
Author information:
(1)SaBio, Instituto de Investigación en Recursos Cinegéticos (IREC), Consejo Superior de Investigaciones Científicas (CSIC), Universidad de Castilla-La

Mancha (UCLM)-Junta de Comunidades de Castilla-La Mancha (JCCM), Ronda de Toledo s/n, 13005 Ciudad Real, Spain.
(2)National Livestock Resources Research Institute (NaLIRRI/NARO), P.O. Box 5704 Kampala, Uganda.
(3)Interdisciplinary Laboratory of Clinical Analysis, Interlab-UMU, Regional Campus of International Excellence Campus Mare Nostrum, University of Murcia, Espinardo, 30100 Murcia, Spain.
(4)Department of Veterinary Pathobiology, Center for Veterinary Health Sciences, Oklahoma State University, Stillwater, OK 74078, USA.
Cattle tick infestations and transmitted pathogens affect animal health, production and welfare with an impact on cattle industry in tropical and subtropical countries. Anti-tick vaccines constitute an effective and sustainable alternative to the traditional methods for the control of tick infestations. Subolesin (SUB)-based vaccines have shown efficacy for the control of multiple tick species, but several factors affect the development of new and more effective vaccines for the control of tick infestations. To address this challenge, herein we used a regional and host/tick species driven approach for vaccine design and implementation. The objective of the study was to develop SUB-based vaccines for the control of the most important tick species (Rhipicephalus appendiculatus, R. decoloratus and Amblyomma variegatum) affecting production of common cattle breeds (Bos indicus and B. indicus x B. taurus crossbred) in Uganda. In this way, we addressed the development of anti-tick vaccines as an intervention to prevent the economic losses caused by ticks and tick-borne diseases in the cattle industry in Uganda. The results showed the possibility of using SUB antigens for the control of multiple tick species in B. indicus and crossbred cattle and suggested the use of R. appendiculatus SUB to continue research on vaccine design and formulation for the control of cattle ticks in Uganda. Future directions would include quantum vaccinology approaches based on the characterization of the SUB protective epitopes, modeling of the vaccine E under Ugandan ecological and epidemiological conditions and optimization of vaccine formulation including the possibility of oral administration.

132. PLoS One. 2020 Jun 29;15(6):e0231434. doi: 10.1371/journal.pone.0231434.
Analysis of p67 allelic sequences reveals a subtype of allele type 1 unique to buffalo-derived Theileria parva parasites from southern Africa.
Mukolwe LD(1)(2), Odongo DO(3), Byaruhanga C(1)(4), Snyman LP(1)(5), Sibeko-Matjila KP(1).
Author information:
(1)Vectors and Vector-borne Diseases Research Programme, Department of Veterinary Tropical Diseases, Faculty of Veterinary Science, University of Pretoria, Onderstepoort, South Africa.
(2)Department of Veterinary Pathology, Microbiology & Parasitology, Faculty of Veterinary Medicine and Surgery, Egerton University, Egerton, Kenya.
(3)School of Biological Sciences, University of Nairobi, Nairobi, Kenya.
(4)National Agricultural Research Organization, Entebbe, Uganda.
(5)Durban Natural Science Museum, Durban, South Africa.

East Coast fever (ECF) and Corridor disease (CD) caused by cattle- and buffalo-derived T. parva respectively are the most economically important tick-borne diseases of cattle in the affected African countries. The p67 gene has been evaluated as a recombinant subunit vaccine against ECF, and for discrimination of T. parva parasites causing ECF and Corridor disease. The p67 allele type 1 was first identified in cattle-derived T. parva parasites from East Africa, where parasites possessing this allele type have been associated with ECF. Subsequent characterization of buffalo-derived T. parva parasites from South Africa where ECF was eradicated, revealed the presence of a similar allele type, raising concerns as to whether or not allele type 1 from parasites from the two regions is identical. A 900 bp central fragment of the gene encoding p67 was PCR amplified from T. parva DNA extracted from blood collected from cattle and buffalo in South Africa, Mozambique, Kenya, Tanzania and Uganda, followed by DNA sequence analysis. Four p67 allele types previously described were identified. A subtype of p67 allele type 1 was identified in parasites from clinical cases of CD and buffalo from southern Africa. Notably, p67 allele type 1 sequences from parasites associated with ECF in East Africa and CD in Kenya were identical. Analysis of two p67 B-cell epitopes (TpM12 and AR22.7) revealed amino acid substitutions in allele type 1 from buffalo-derived T. parva parasites from southern Africa. However, both epitopes were conserved in allele type 1 from cattle- and buffalo-derived T. parva parasites from East Africa. These findings reveal detection of a subtype of p67 allele type 1 associated with T. parva parasites transmissible from buffalo to cattle in southern Africa.

133. Parasitol Res. 2020 Sep;119(9):3061-3073. doi: 10.1007/s00436-020-06796-z. Epub
Inhibitory effects of novel ciprofloxacin derivatives on the growth of four Babesia species and Theileria equi.
Batiha GE(1)(2), Tayebwa DS(1)(3), Beshbishy AM(1), N'Da DD(4), Yokoyama N(1), Igarashi I(5).
Author information:
(1)National Research Center for Protozoan Diseases, Obihiro University of Agriculture and Veterinary Medicine, Nishi 2-13, Inada-cho, Obihiro, Hokkaido, 080-8555, Japan.
(2)Department of Pharmacology and Therapeutics, Faculty of Veterinary Medicine, Damanhour University, Damanhour, Al-Beheira, 22511, Egypt.
(3)Research Center for Tick and Tick-borne diseases, College of Veterinary Medicine, Animal Resources and Biosecurity, Makerere University, PO Box 7062, Kampala, Uganda.
(4)Centre of Excellence for Pharmaceutical Sciences (PHARMACEN), North-West University, Potchefstroom, South Africa.
(5)National Research Center for Protozoan Diseases, Obihiro University of Agriculture and Veterinary Medicine, Nishi 2-13, Inada-cho, Obihiro, Hokkaido, 080-8555, Japan. igarcpmi@obihiro.ac.jp.
The problems of parasite resistance, as well as the toxic residues to most of the commercially available antipiroplasmic drugs severely weaken their effective, curative, and environmental safe employment. Therefore, it is clear that the development of treatment options for piroplasmosis is vital for

improving disease treatment and control. Ciprofloxacin is a broad-spectrum antibiotic that targets mainly the DNA replication machinery by inhibiting DNA gyrase and topoisomerase enzymes. As a result, ciprofloxacin is used for treating several bacterial and parasitic infections. In this study, the efficacy of 15 novel ciprofloxacin derivatives (NCD) that had been developed against drug-resistant Mycobacterium tuberculosis was evaluated against piroplasm parasite multiplication in vitro. The half-maximal inhibitory concentration (IC50) values of the most effective five compounds of NCD (No. 3, 5, 10, 14, 15) on Babesia bovis, Babesia bigemina, Babesia caballi, and Theileria equi were 32.9, 13.7, 14.9, and 30.9; 14.9, 25.8, 13.6, and 27.5; 34.9, 33.9, 21.1, and 22.3; 26.7, 28.3, 34.5, and 29.1; and 4.7, 26.6, 33.9, and 29.1 µM, respectively. Possible detrimental effects of tested NCD on host cells were assessed using mouse embryonic fibroblast (NIH/3T3) and Madin-Darby bovine kidney (MDBK) cell lines. Tested NCD did not suppress NIH/3T3 and MDBK cell viability, even at the highest concentration used (500 µM). Combination treatments of the identified most effective compounds of NCD/diminazene aceturate (DA), /atovaquone (AQ), and /clofazimine (CF) showed mainly synergistic and additive effects. The IC50 values of NCD showed that they are promising future candidates against piroplasmosis. Further in vivo trials are required to evaluate the therapeutic potential of NCD.

134. Front Vet Sci. 2020 Jul 22;7:419. doi: 10.3389/fvets.2020.00419. eCollection
Nairobi Sheep Disease Virus: A Historical and Epidemiological Perspective.
Krasteva S(1), Jara M(1), Frias-De-Diego A(1), Machado G(1).
Author information:
(1)Department of Population Health and Pathobiology, College of Veterinary Medicine, North Carolina State University, Raleigh, NC, United States.
Nairobi Sheep Disease virus (NSDv) is a zoonotic and tick-borne disease that can cause over 90% mortality in small ruminants. NSDv has historically circulated in East Africa and has recently emerged in the Asian continent. Despite efforts to control the disease, some regions, mostly in warmer climates, persistently report disease outbreaks. Consequently, it is necessary to understand how environmental tolerances and factors that influence transmission may shed light on its possible emergence in other regions. In this study, we quantified the available literature of NSDv from which occurrence data was extracted. In total, 308 locations from Uganda, Kenya, Tanzania, Somalia, India, Sri Lanka and China were coupled with landscape conditions to reconstruct the ecological conditions for NSDv circulation and identify areas of potential disease transmission risk. Our results identified areas suitable for NSDv in Ethiopia, Malawi, Zimbabwe, Southeastern China, Taiwan, and Vietnam. Unsuitable areas included Democratic Republic of Congo, Zambia, and Southern Somalia. In summary, soil moisture, livestock density, and precipitation predispose certain areas to NSDv circulation. It is critical to investigate the epidemiology of NSDv in order to promote better allocation of resources to control its spread in regions that are more at risk. This will help reduce disease impact worldwide as climate change will favor emergence of such vector-borne diseases in areas with dense small ruminant populations.

135. Vaccines (Basel). 2020 Aug 19;8(3):457. doi: 10.3390/vaccines8030457.
Cocktail Anti-Tick Vaccines: The Unforeseen Constraints and Approaches toward Enhanced Efficacies.
Ndawula C Jr(1), Tabor AE(2)(3).
Author information:
(1)Vaccinology Research Program, National Livestock Resources Research Institute, P O. Box 5746, Nakyesasa 256, Uganda.
(2)Centre for Animal Science, Queensland Alliance for Agriculture & Food Innovation, The University of Queensland Australia, St Lucia 4072, Queensland, Australia.
(3)School of Chemistry & Molecular Biosciences, The University of Queensland, St Lucia 4072, Queensland, Australia.
Ticks are second to mosquitoes as vectors of disease. Ticks affect livestock industries in Asia, Africa and Australia at ~$1.13 billion USD per annum. For instance, 80% of the global cattle population is at risk of infestation by the Rhipicephalus microplus species-complex, which in 2016 was estimated to cause $22-30 billion USD annual losses. Although the management of tick populations mainly relies on the application of acaricides, this raises concerns due to tick resistance and accumulation of chemical residues in milk, meat, and the environment. To counteract acaricide-resistant tick populations, immunological tick control is regarded among the most promising sustainable strategies. Indeed, immense efforts have been devoted toward identifying tick vaccine antigens. Until now, Bm86-based vaccines have been the most effective under field conditions, but they have shown mixed success worldwide. Currently, of the two Bm86 vaccines commercialized in the 1990s (GavacTM in Cuba and TickGARDPLUSTM in Australia), only GavacTM is available. There is thus growing consensus that combining antigens could broaden the protection range and enhance the efficacies of tick vaccines. Yet, the anticipated outcomes have not been achieved under field conditions. Therefore, this review demystifies the potential limitations and proposes ways of sustaining enhanced cocktail tick vaccine efficacy.

136. Parasit Vectors. 2020 Sep 7;13(1):452. doi: 10.1186/s13071-020-04322-9.
Sequence diversity of cytotoxic T cell antigens and satellite marker analysis of Theileria parva informs the immunization against East Coast fever in Rwanda.
Atuhaire DK(1), Muleya W(2), Mbao V(3), Bazarusanga T(4), Gafarasi I(5), Salt J(6), Namangala B(7), Musoke AJ(8).
Author information:
(1)Centre for Ticks and Tick-Borne Diseases, Private Bag A130, Lilongwe, Malawi.
(2)Department of Biomedical Sciences, School of Veterinary Medicine, University of Zambia, P.O. Box 32379, Lusaka, 10101, Zambia. muleyawalter@gmail.com.
(3)International Development Research Centre, Eastern and Southern Africa Regional Office, Nairobi, Kenya.
(4)Acre Africa, P.O. Box 6782, Kigali, Rwanda.
(5)Rwanda Agricultural Board, P.O. Box 5016, Kigali, Rwanda.
(6)Global Alliance for Livestock Veterinary Medicines, Doherty Building,

Pentlands Science Park, Bush Loan, Penicuik, Edinburgh, EH26 0PZ, Scotland, UK.
(7)Department of Paraclinical Studies, School of Veterinary Medicine, University of Zambia, P.O. Box 32379, Lusaka, 10101, Zambia.
(8)LMK Medical laboratories and consultancies, P.O. Box 33686, Kampala, Uganda.
BACKGROUND: East Coast fever (ECF) caused by Theileria parva is endemic in Rwanda. In this study, the antigenic and genetic diversity of T. parva coupled with immunization and field challenge were undertaken to provide evidence for the introduction of ECF immunization in Rwanda.
METHODS: Blood collected from cattle in the field was screened for T. parva using ELISA and PCR targeting the p104 gene. Tp1 and Tp2 gene sequences were generated from field samples and from Gikongoro and Nyakizu isolates. Furthermore, multilocus genotype data was generated using 5 satellite markers and an immunization challenge trial under field conditions using Muguga cocktail vaccine undertaken.
RESULTS: Out of 120 samples, 44 and 20 were positive on ELISA and PCR, respectively. Antigenic diversity of the Tp1 and Tp2 gene sequences revealed an abundance of Muguga, Kiambu and Serengeti epitopes in the samples. A further three clusters were observed on both Tp1 and Tp2 phylogenetic trees; two clusters comprising of field samples and vaccine isolates and the third cluster comprising exclusively of Rwanda samples. Both antigens exhibited purifying selection with no positive selection sites. In addition, satellite marker analysis revealed that field samples possessed both shared alleles with Muguga cocktail on all loci and also a higher proportion of unique alleles. The Muguga cocktail (Muguga, Kiambu and Serengeti) genotype compared to other vaccine isolates, was the most represented in the field samples. Further low genetic sub-structuring (FST = 0.037) coupled with linkage disequilibrium between Muguga cocktail and the field samples was observed. Using the above data to guide a field immunization challenge trial comprising 41 immunized and 40 control animals resulted in 85% seroconversion in the immunized animals and an efficacy of vaccination of 81.7%, implying high protection against ECF.
CONCLUSIONS: Antigenic and genetic diversity analysis of T. parva facilitated the use of Muguga cocktail vaccine in field conditions. A protection level of 81.7% was achieved, demonstrating the importance of combining molecular tools with field trials to establish the suitability of implementation of immunization campaigns. Based on the information in this study, Muguga cocktail immunization in Rwanda has a potential to produce desirable results.

137. Vet Med Int. 2020 Aug 27;2020:4606059. doi: 10.1155/2020/4606059. eCollection 2020.

Comparison of Tick Control and Antibiotic Use Practices at Farm Level in Regions of High and Low Acaricide Resistance in Uganda.
Byaruhanga J(1), Odua F(1), Ssebunya Y(2), Aketch O(1), Tayebwa DS(1), Rwego IB(3)(4), Vudriko P(1).
Author information:
(1)Research Center for Tropical Diseases and Vector Control, Department of Veterinary Pharmacy, Clinics and Comparative Medicine, School of Veterinary

Medicine and Animal Resources, College of Veterinary Medicine, Animal Resources
and Biosecurity, Makerere University, Kampala, Uganda.
(2)Division of Veterinary Regulation and Inspection, Department of Animal
Health, Ministry of Agriculture, Animal Industry and Fisheries, Entebbe, Uganda.
(3)One Health Division, Department of Veterinary Population Medicine, College of
Veterinary Medicine, St. Paul, Falcon Heights, MN 55108, USA.
(4)Department of Biosecurity, Ecosystems and Veterinary Public Health, College
of Veterinary Medicine, Animal Resources and Biosecurity (COVAB), Makerere
University, Kampala, Uganda.
Uganda has experienced tick acaricide resistance in the livestock sector. With
increase in incidence of tick-borne diseases (TBDs), use of antibiotics for
control of TBDs and other opportunistic diseases has raisedserious concerns. The
purpose of this study was to compare the farmers' tick control and antibiotic
use practices on farms in regions of low (LARA) and high (HARA) tick acaricide
resistance in Uganda, determine the prevalence of antibiotic residues in milk
from both regions, and identify factors associated with antibiotic residues in
milk. One representative district was selected from each region from which 10
farms were randomly selected. Delvotest SP-NT® test kit was used to detect
antibiotic residues in milk. Half-body tick counts and acaricide efficacy tests
were performed. Majority (70%) of HARA's respondents reported a corresponding
increase in a monthly incidence of TBDs with an average of 3.2 cases of TBDs
treated per farm compared to 0.2 cases in LARA. East Coast fever (ECF) was
identified as the most common TBD in both regions, though cases of coinfection
were more common in HARA. Half of HARA's respondents reported a corresponding
increase in the use of antibiotics on their farms due to tick resistance
compared to LARA. Antibiotics were the most used drugs on farms in both regions
with oxytetracycline being the commonly used antibiotic. Ticks from HARA were
resistant to deltamethrin, amitraz, and coformulation (chlorpyriphos and
cypermethrin) while resistance against deltamethrin was confirmed in LARA. HARA
farms had a significantly higher prevalence of antibiotic residues (21.25%) in
raw milk than in LARA (4%) farms ($p < 0.05$). Acaricide resistance and practice
of reading drug use instructions were significantly associated with antibiotic
residues in milk at farm level. Overall, the study provides vital information
linking acaricide resistance to antibiotic use practices, consequently leading
to antibiotic residues in milk.

138. Ticks Tick Borne Dis. 2020 Nov;11(6):101539. doi: 10.1016/j.ttbdis.2020.101539.
Microsatellite and minisatellite genotyping of Theileria parva population from
southern Africa reveals possible discriminatory allele profiles with parasites
from eastern Africa.
Lubembe DM(1), Odongo DO(2), Salih DA(3), Sibeko-Matjila KP(4).
Author information:
(1)Vectors and Vector-borne Diseases Research Programme, Department of
Veterinary Tropical Diseases, Faculty of Veterinary Science, University of
Pretoria, Private Bag X04, Onderstepoort, 0110, South Africa. Electronic
address: dlubembe@gmail.com.
(2)School of Biological Sciences, University of Nairobi, P.O. Box 30197,

Nairobi, 00100, Kenya.
(3)Central Veterinary Research Laboratory, P.O. Box 8067, Khartoum, Sudan.
(4)Vectors and Vector-borne Diseases Research Programme, Department of Veterinary Tropical Diseases, Faculty of Veterinary Science, University of Pretoria, Private Bag X04, Onderstepoort, 0110, South Africa.
The control of Theileria parva, a protozoan parasite that threatens almost 50% of the cattle population in Africa, is still a challenge in many affected countries. Theileria parva field parasites from eastern Africa, and parasites comprising the current live T. parva vaccine widely deployed in the same region have been reported to be genotypically diverse. However, similar reports on T. parva parasites from southern Africa are limited, especially in Corridor disease designated areas. Establishing the extent of genetic exchange in T. parva populations is necessary for effective control of the parasite infection. Twelve polymorphic microsatellite and minisatellite loci were targeted for genotypic and population genetics analysis of T. parva parasites from South Africa, Mozambique, Kenya and Uganda using genomic DNA prepared from cattle and buffalo blood samples. The results revealed genotypic similarities among parasites from the two regions of Africa, with possible distinguishing allelic profiles on three loci (MS8, MS19 and MS33) for parasites associated with Corridor disease in South Africa, and East Coast fever in eastern Africa. Individual populations were in linkage equilibrium (VD<L), but when considered as one combined population, linkage disequilibrium (VD>L) was observed. Genetic divergence was observed to be more within (AMOVA = 74%) than between (AMOVA = 26%) populations. Principal coordinate analysis showed clustering that separated buffalo-derived from cattle-derived T. parva parasites, although parasites from cattle showed a close genetic relationship. The results also demonstrated geographic sub-structuring of T. parva parasites based on the disease syndromes caused in cattle in the two regions of Africa. These findings provide additional information on the genotypic diversity of T. parva parasites from South Africa, and reveal possible differences based on three loci (MS8, MS19 and MS33) and similarities between buffalo-derived T. parva parasites from southern and eastern Africa.

139. BMC Vet Res. 2020 Oct 8;16(1):380. doi: 10.1186/s12917-020-02585-z.
Molecular detection and genetic characterization of Anaplasma marginale and Anaplasma platys-like (Rickettsiales: Anaplasmataceae) in water buffalo from eight provinces of Thailand.
Nguyen AHL(1)(2), Tiawsirisup S(3), Kaewthamasorn M(4)(5).
Author information:
(1)The international graduate course of Veterinary Science and Technology (VST), Faculty of Veterinary Science, Chulalongkorn University, Bangkok, 10330, Thailand.
(2)Veterinary Parasitology Research Group, Faculty of Veterinary Science, Chulalongkorn University, Bangkok, 10330, Thailand.
(3)Animal Vector-Borne Disease Research Unit, The Veterinary Parasitology Unit, Department of Veterinary Pathology, Faculty of Veterinary Science, Chulalongkorn University, Bangkok, 10330, Thailand.

(4)Veterinary Parasitology Research Group, Faculty of Veterinary Science, Chulalongkorn University, Bangkok, 10330, Thailand. morakot.k@chula.ac.th.
(5)Animal Vector-Borne Disease Research Unit, The Veterinary Parasitology Unit, Department of Veterinary Pathology, Faculty of Veterinary Science, Chulalongkorn University, Bangkok, 10330, Thailand. morakot.k@chula.ac.th.
BACKGROUND: Anaplasmosis, an animal disease caused by rickettsial bacteria in the genus Anaplasma, is of considerable economic importance in livestock animals in many countries worldwide. The objectives of this study were to determine the identity, prevalence, and geographic distribution of Ehrlichia and Anaplasma in naturally infected water buffalo in Thailand using PCR amplification and sequencing of the 16S ribosomal RNA and heat shock protein groEL genes. A total of 456 buffalo blood samples from Thailand were investigated. Species identification and genetic differentiation of intra-population and inter-population with the global isolates were conducted based on nucleotide sequences. Interplay between the infection and host factors was also assessed.
RESULTS: Overall, 41% of water buffalo were found to be infected with rickettsial organisms in the family Anaplasmataceae, but Ehrlichia spp., Neorickettsia spp., and Wolbachia spp. were not found in any of the sequenced samples in this study. Female buffalo were more frequently infected with bacteria in the family Anaplasmataceae than males [71 out of 176 females (40.3%) versus 11 out of 47 males (23.4%)]. The Odds Ratio value indicated that the risk of infection for female buffalo was 2.2-fold higher than that for males ($p < 0.05$). We detected three haplotypes of A. marginale 16S rRNA gene and they were placed in a clade that was closely related to the A. marginale in buffalo in China; and cattle in Thailand, Uganda, and China. Homology searching of groEL sequences against the GenBank™ database using the BLASTn algorithm revealed that the obtained sequences had a high percentage similarity (98.36-99.62%) to A. platys sequences. The groEL sequences of three A. platys-like isolates were clustered in the same clade as the A. platys from the tick Rhipicephalus microplus in China.
CONCLUSIONS: Our data showed that the apparently healthy buffalo were naturally infected by bacteria in the family Anaplasmataceae at a relatively high prevalence. We also report the finding of A. platys-like infections in water buffalo in Thailand for the first time. Water buffalo serving as the reservoir host of anaplasmosis is of concern for managing the disease control and prevention in ruminants.

140. Pathogens. 2020 Oct 27;9(11):895. doi: 10.3390/pathogens9110895.
First Molecular Detection of Babesia ovis, Theileria spp., Anaplasma spp., and Ehrlichia ruminantium in Goats from Western Uganda.
Tumwebaze MA(1)(2), Byamukama B(1)(2), Tayebwa DS(2)(3), Byaruhanga J(2), Angwe MK(2), Galon EM(1), Liu M(1), Lee SH(1)(4), Ringo AE(1)(5), Adjou Moumouni PF(1), Li J(1), Li Y(1), Ji S(1), Vudriko P(2)(3), Xuan X(1).
Author information:
(1)National Research Center for Protozoan Diseases, Obihiro University of Agriculture and Veterinary Medicine, Obihiro, Hokkaido 080-8555, Japan.
(2)Research Center for Tropical Diseases and Vector Control, College of

Veterinary Medicine, Animal Resources and Biosecurity, Makerere University,
Kampala 7062, Uganda.
(3)Department of Veterinary Pharmacy, Clinical & Comparative Medicine, School of
Veterinary Medicine and Animal Resources, College of Veterinary Medicine, Animal
Resources and Biosecurity, Makerere University, Kampala 7062, Uganda.
(4)College of Veterinary Medicine, Chungbuk National University, Cheongju 28644,
Korea.
(5)Zanzibar Livestock Research Institute, Ministry of Agriculture, Natural
Resources, Livestock and Fisheries, P.O. Box 159 Zanzibar, Tanzania.
Ticks and tick-borne diseases are major impediments to livestock production. To date, there have been several studies on the prevalence of tick-borne pathogens (TBPs) in cattle, but very few studies have documented TBPs in goats in Uganda. In this study, polymerase chain reaction assays and sequence analysis of different molecular markers were used to assess the presence and genetic characteristics of TBPs in 201 goats from Kasese district in western Uganda. The risk factors associated with TBP infections were also analyzed. We detected Theileria spp. (13.4%), Anaplasma phagocytophilum (10.9%), Anaplasma ovis (5.5%), Babesia ovis (5.5%), and Ehrlichia ruminantium (0.5%). The sequences of B. ovis ssu rRNA and A. ovismsp4 genes showed some degree of diversity among the parasite isolates in this study. The E. ruminantium pCS20 sequence formed a well-supported clade with isolates from Amblyomma variegatum ticks from Uganda. Wildlife interaction, sampling location, low body condition score, tick infestation, and herd size were significantly associated with TBP infections in the goats. The findings in this study provide important information on the epidemiology of tick-borne pathogens in Uganda, and show that goats could be potential reservoirs for tick-borne pathogens.

141. Vet World. 2020 Sep;13(9):1910-1921. doi: 10.14202/vetworld.2020.1910-1921. Epub
Development of a practical framework for sustainable surveillance and control of
ticks and tick-borne diseases in Africa.
Nchu F(1), Nyangiwe N(2)(3), Muhanguzi D(4), Nzalawahe J(5), Nagagi YP(6),
Msalya G(7)(8), Joseph NA(9), Kimaro EG(6), Mollel M(6), Temba V(6), Harouna
DV(10).
Author information:
(1)Department of Horticultural Sciences, Cape Peninsula University of
Technology, Bellville, Symphony Way, Bellville, Cape Town, 7535, South Africa.
(2)Department of Rural Development and Agrarian Reform, Döhne Agricultural
Development Institute, Private Bag X15, Stutterheim 4930, South Africa.
(3)Department of Conservation Ecology and Entomology, University of
Stellenbosch, Stellenbosch 7602, South Africa.
(4)Department of Biomolecular and Biolaboratory Sciences, College of Veterinary
Medicine Animal Resources and Biosecurity, Makerere University, P.O. Box 7062,
Kampala, Uganda.
(5)Department of Veterinary Microbiology, Parasitology and Biotechnology,
College of Veterinary Medicine and Biomedical Sciences, Sokoine University of
Agriculture, P. O. Box 3019, Chuo Kikuu, Morogoro, Tanzania.
(6)Division of Livestock and Human Diseases Vector Control, Tropical Pesticides

Research Institute, P.O. Box 3024, Arusha, Tanzania.
(7)Department of Animal, Aquaculture and Range Sciences, College of Agriculture, Sokoine University of Agriculture, P. O. Box 3004, Chuo Kikuu, Morogoro, Tanzania.
(8)Laboratory of Animal Breeding and Genetics, Kagoshima University, 1-21-24 Korimoto, Kagoshima 890-0065, Japan.
(9)Department of Veterinary Parasitology, Faculty of Veterinary Medicine, Ahmadu Bello University, Zaria, Nigeria.
(10)Department of Food Biotechnology and Nutritional Sciences, Nelson Mandela African Institution of Science and Technology, P. O. Box 447, Arusha, Tanzania.
A workshop on ticks and tick-borne diseases (T&TBDs) was held on June 25 and 26, 2019, at the Tropical Pesticides Research Institute, Division of Livestock and Human Diseases Vector Control, Arusha, Tanzania. The objectives of the workshop were to discuss the current situation and to formulate actionable strategies to improve surveillance and control of T&TBDs in Africa. The workshop was funded by the National Research Foundation and the Cape Peninsula University of Technology and attended by livestock health providers, farmers, and researchers from East, West, and Southern African countries. During the workshop, experts presented recent surveillance data focused on T&TBDs; participants discussed research opportunities and community engagement. The primary outcome of the workshop was the creation of a new research consortium known as The African Consortium for T&TBDs. The consortium is intended to function as a community for researchers, students, farmers, policymakers, extension workers, and community members who are interested in the advancement of T&TBD control. The consortium will engage in research activities that focus on comprehensive surveillance of T&TBDs, developing tick acaricide resistance, alternative tick control programs, and policy development and education. These areas were identified as top priorities to be developed to improve T&TBD control on the continent.

142. Transbound Emerg Dis. 2021 Jul;68(4):2429-2445. doi: 10.1111/tbed.13911. Epub
Tick-borne pathogens, including Crimean-Congo haemorrhagic fever virus, at livestock markets and slaughterhouses in western Kenya.
Chiuya T(1)(2), Masiga DK(1), Falzon LC(3)(4), Bastos ADS(2), Fèvre EM(3)(4), Villinger J(1).
Author information:
(1)International Centre of Insect Physiology and Ecology (icipe), Nairobi, Kenya.
(2)Department of Zoology and Entomology, University of Pretoria, Pretoria, South Africa.
(3)Institute of Infection, Veterinary and Ecological Sciences, University of Liverpool, Neston, UK.
(4)International Livestock Research Institute, Nairobi, Kenya.
Vectors of emerging infectious diseases have expanded their distributional ranges in recent decades due to increased global travel, trade connectivity and climate change. Transboundary range shifts, arising from the continuous movement of humans and livestock across borders, are of particular disease control concern. Several tick-borne diseases are known to circulate between eastern

Uganda and the western counties of Kenya, with one fatal case of Crimean-Congo haemorrhagic fever (CCHF) reported in 2000 in western Kenya. Recent reports of CCHF in Uganda have highlighted the risk of cross-border disease translocation and the importance of establishing inter-epidemic, early warning systems to detect possible outbreaks. We therefore carried out surveillance of tick-borne zoonotic pathogens at livestock markets and slaughterhouses in three counties of western Kenya that neighbour Uganda. Ticks and other ectoparasites were collected from livestock and identified using morphological keys. The two most frequently sampled tick species were Rhipicephalus decoloratus (35%) and Amblyomma variegatum (30%); Ctenocephalides felis fleas and Haematopinus suis lice were also present. In total, 486 ticks, lice and fleas were screened for pathogen presence using established molecular workflows incorporating high-resolution melting analysis and identified through sequencing of PCR products. We detected CCHF virus in Rh. decoloratus and Rhipicephalus sp. cattle ticks, and 82 of 96 pools of Am. variegatum were positive for Rickettsia africae. Apicomplexan protozoa and bacteria of veterinary importance, such as Theileria parva, Babesia bigemina and Anaplasma marginale, were primarily detected in rhipicephaline ticks. Our findings show the presence of several pathogens of public health and veterinary importance in ticks from livestock at livestock markets and slaughterhouses in western Kenya. Confirmation of CCHF virus, a Nairovirus that causes haemorrhagic fever with a high case fatality rate in humans, highlights the risk of under-diagnosed zoonotic diseases and calls for continuous surveillance and the development of preventative measures.

143. Vet Parasitol. 2020 Dec;288:109304. doi: 10.1016/j.vetpar.2020.109304. Epub 2020
An alternative cold chain for storing and transporting East Coast fever vaccine.
Atuhaire DK(1), Lieberman D(2), Marcotty T(3), Musoke AJ(4), Madan D(5).
Author information:
(1)Centre for Ticks and Tick-Borne Diseases, Lilongwe, Malawi.
(2)Global Health Labs, Bellevue, Washington, United States; Intellectual Ventures' Global Good Fund, Bellevue, Washington, United States.
(3)University of Namur, Belgium.
(4)LMK Medical Laboratories and Consultancies, Kampala, Uganda.
(5)Global Health Labs, Bellevue, Washington, United States; Intellectual Ventures' Global Good Fund, Bellevue, Washington, United States. Electronic address: damian.madan@ghlabs.org.
East Coast fever (ECF) is an often fatal, economically important cattle disease that predominantly affects eastern, central, and southern Africa. ECF is controlled through vaccination by means of simultaneous injection of oxytetracycline and cryogenically preserved stabilate containing live, disease-causing parasites. Storage and transportation of the stabilate requires liquid nitrogen, a commodity that is commonly unreliable in low-resource settings. Here we show that storage of conventionally prepared stabilate at -80 °C for up to 30 days does not significantly affect its ability to infect cultured peripheral blood mononucleated cells or live cattle, suggesting an alternative cold chain that maintains these temperatures could be used to effectively manage ECF.

144. Syst Parasitol. 2020 Dec;97(6):601-621. doi: 10.1007/s11230-020-09954-0. Epub
Description of two new species of Haemaphysalis Koch, 1844 (Acari: Ixodidae) from the H. (Rhipistoma) spinulosa subgroup, parasites of carnivores and other mammals in Africa.
Apanaskevich DA(1)(2)(3), Tomlinson JA(4)(5).
Author information:
(1)United States National Tick Collection, The James H. Oliver, Jr. Institute for Coastal Plain Science, Georgia Southern University, Statesboro, GA, 30460-8042, USA. dapanaskevich@georgiasouthern.edu.
(2)Biology Department, Georgia Southern University, Statesboro, GA, 30460, USA. dapanaskevich@georgiasouthern.edu.
(3)Zoological Institute of Russian Academy of Sciences, Saint Petersburg, Russia, 199034. dapanaskevich@georgiasouthern.edu.
(4)United States National Tick Collection, The James H. Oliver, Jr. Institute for Coastal Plain Science, Georgia Southern University, Statesboro, GA, 30460-8042, USA.
(5)Biology Department, Georgia Southern University, Statesboro, GA, 30460, USA.
Haemaphysalis (Rhipistoma) latitudinis n. sp. (Acari: Ixodidae) and H. (R.) setosa n. sp. are described based on adults. Adults of H. (R.) latitudinis n. sp. were mostly collected from carnivores (Carnivora: Canidae, Felidae, Herpestidae, Mustelidae, Viverridae), few specimens were found on rodents (Rodentia: Muridae), bat (Chiroptera: Molossidae) and domestic cattle (Artiodactyla: Bovidae) in Benin, Central African Republic, Côte d'Ivoire, Democratic Republic of the Congo, Ethiopia, Ghana, Kenya, Nigeria, South Sudan and Uganda. Adults of H. (R.) setosa n. sp. were mostly found on carnivores (Carnivora: Felidae, Herpestidae, Viverridae), few specimens were found on rodents (Rodentia: Muridae), hare (Lagomorpha: Leporidae), hyrax (Hyracoidea: Procaviidae) and human (Primates: Hominidae) in Kenya. Males and females of both species can be differentiated from each other and other H. spinulosa-like ticks by their size, pattern of punctations on conscutum/scutum, size of setae, shape of genital structures, size and shape of spiracular plates, dorsal cornua, posterodorsal and posteroventral spurs on palpal segment II and spur on coxae and dentition on hypostome.

145. Emerg Infect Dis. 2020 Dec;26(12):2944-2950. doi: 10.3201/eid2612.202411.
Human-Pathogenic Kasokero Virus in Field-Collected Ticks.
Schuh AJ, Amman BR, Patel K, Sealy TK, Swanepoel R, Towner JS.
Kasokero virus (KASV; genus Orthonairovirus) was first isolated in 1977 at Uganda Virus Research Institute from serum collected from Rousettus aegyptiacus bats captured at Kasokero Cave, Uganda. During virus characterization studies at the institute, 4 laboratory-associated infections resulted in mild to severe disease. Although orthonairoviruses are typically associated with vertebrate and tick hosts, a tick vector of KASV never has been reported. We tested 786 Ornithodoros (Reticulinasus) faini tick pools (3,930 ticks) for KASV. The ticks were collected from a large R. aegyptiacus bat roosting site in western Uganda. We detected KASV RNA in 43 tick pools and recovered 2 infectious isolates, 1 of

which was derived from host blood-depleted ticks. Our findings suggest that KASV is maintained in an enzootic transmission cycle involving O. (R.) faini ticks and R. aegyptiacus bats and has the potential for incidental virus spillover to humans.

146. Afr Health Sci. 2020 Sep;20(3):1168-1178. doi: 10.4314/ahs.v20i3.20.
Rodents as potential hosts and reservoirs of parasites along the edge of a Central African forest: Bwindi impenetrable national park, South Western Uganda.
Mawanda P(1), Rwego I(2)(3), Kisakye JJ(2), Sheil D(4).
Author information:
(1)Makerere University, College of Education and External Studies, School of Distance and Lifelong studies, Department of distance education. P. O. Box 7062, Kampala, Uganda.
(2)Makerere University, College of Natural Sciences, Department of Biological Sciences, P. O. Box 7062, Kampala, Uganda.
(3)College of Veterinary Medicine, One Health Division, University of Minnesota, St. Paul, 1954 Buford Avenue, MN 55108, USA.
(4)Department of Ecology and Natural Resource Management, Norwegian University of Life Sciences, P. O. Box 5003 NO-1432 Ås, Norway. Center for International Forestry Research (CIFOR), GPO Box 113 BOCBD, Bogor 1600, Indonesia. Institute of Tropical Forest Conservation, P. O. Box 44, Kabale, Uganda.
BACKGROUND: Rodents which constitute 42% of the world's mammalian population are major reservoirs of pathogens that cause zoonoses. Currently we know little about rodents' potential zoonotic transfer from human settlements into protected areas and how any such threats might be reduced.
OBJECTIVE: To investigate the role of rodents as reservoirs of zoonotic pathogens along the boundary of Bwindi.
METHODS: A rodent inventory in three villages along the edge of Bwindi, was carried using live trapping techniques and the local rodents' ecto and endoparasite fauna investigated.
RESULTS: Two hundred eighty eight rodents captured belonged to 24 species, 17 genera and 4 families with Lophuromys aquilus being most abundant (30.2%). 240 ectoparasites which included mites, fleas and ticks were collected from 88 rodents out of 249. Proamys jacksoni rodents were most infested. Although the mites represented the largest proportion (84.6%), the highest species diversity was shown among the fleas (9 species). Some 36.9% of the rodents were infected with endoparasites of which L. aquilus haboured most. Endoparasitic genera identified included Nippostrongylus, Ascaris, Strongyloides, Trichuris, Hymenolepis, Taenia and Cryptosporidium.
CONCLUSION: Rodents have a zoonotic potentiality. There is need for developing effective integrated rodent management programs against rodent to reduce chances of parasite transmission within the protected areas.

147. Parasitol Res. 2021 Mar;120(3):1097-1102. doi: 10.1007/s00436-020-07009-3. Epub
Ticks and tick-borne pathogens infecting livestock and dogs in Tchicala-Tcholoanga, Huambo Province, Angola.
Sili G(1)(2), Byaruhanga C(3)(4), Horak I(1), Steyn H(5), Chaisi M(1)(6),

Oosthuizen MC(1), Neves L(1)(7).
Author information:
(1)Vectors and Vector-Borne Diseases Research Programme, Department of Veterinary Tropical Diseases, Faculty of Veterinary Science, University of Pretoria, Private Bag X04, Onderstepoort, 0110, South Africa.
(2)Department of Basic Science, Faculty of Veterinary Medicine, University Jose Eduardo dos Santos, Huambo, Angola.
(3)Vectors and Vector-Borne Diseases Research Programme, Department of Veterinary Tropical Diseases, Faculty of Veterinary Science, University of Pretoria, Private Bag X04, Onderstepoort, 0110, South Africa.
cbyaruhanga27@yahoo.com.
(4)National Agricultural Research Organisation, P.O. Box 259, Entebbe, Uganda.
cbyaruhanga27@yahoo.com.
(5)Onderstepoort Veterinary Institute, Private Bag X05, Onderstepoort, 0110, South Africa.
(6)Foundation Research and Services, South African National Biodiversity Institute, 232 Boom Street, Pretoria, 0001, South Africa.
(7)Centro de Biotecnologia, Universidade Eduardo Mondlane, Av. de Moçambique Km 1.5, Maputo, Mozambique.
The diversity of ticks and tick-borne pathogens (TBPs) infesting domestic animals in Tchicala-Tcholoanga, Angola, in 2016 was investigated. Seventeen tick species were recorded, Amblyomma pomposum being the most abundant on cattle (40%), goats (38%) and sheep (35%); Rhipicephalus turanicus was the most abundant on dogs (46%). This study presents new records of Haemaphysalis paraleachi, R. compositus, R. kochi and R. sulcatus in Angola, the first georeferenced population of Ha. leachi in southern Africa and the second record of R. microplus in Angola. Using the reverse line blot (RLB) hybridisation assay, fifteen TBP species were detected in blood samples from cattle (n = 88), goats (n = 82), sheep (n = 85) and dogs (n = 85). F The most frequently detected species were Theileria velifera in cattle (78%), Theileria ovis in sheep (80%) and Babesia vogeli in dogs (35%). Species-specific quantitative PCR assays detected Babesia bigemina in 43% (35/80) of blood samples of cattle, while E. ruminantium was detected in 4% (3/70) of blood samples and in 7% of A. pomposum ticks. Anaplasma platys was detected from cattle (18%) and sheep (6%) during RLB analysis. These findings constitute pioneering research in Angola.

148. Heliyon. 2020 Dec 31;7(1):e05688. doi: 10.1016/j.heliyon.2020.e05688.
Molecular epidemiology of anaplasmosis in small ruminants along a human-livestock-wildlife interface in Uganda.
Kasozi KI(1)(2)(3), Welburn SC(1)(4), Batiha GE(5), Marraiki N(6), Nalumenya DP(7), Namayanja M(7), Matama K(8), Zalwango KK(7), Matovu W(7), Zirintunda G(2), Ekou J(2), Kembabazi S(8), Mugasa CM(7), Kitibwa A(7), Tayebwa DS(7), Musinguzi SP(9), Mahero M(10), Ssengendo I(8), Nanteza A(7), Matovu E(7), MacLeod ET(1).
Author information:
(1)Infection Medicine, Deanery of Biomedical Sciences, College of Medicine and Veterinary Medicine, The University of Edinburgh, 1 George Square, Edinburgh EH8

9JZ, United Kingdom.
(2)Department of Animal Production and Management, Faculty of Agriculture and Agricultural Sciences, Busitema University Arapai Campus, Box 203 Soroti, Uganda.
(3)School of Medicine, Kabale University, Box 317 Kabale, Uganda.
(4)Zhejiang University-University of Edinburgh Institute, Zhejiang University School of Medicine, International Campus, 718 East Haizhou Road, Haining 314400, China.
(5)Department of Pharmacology and Therapeutics, Faculty of Veterinary Medicine, Damanhour University, Damanhour 22511, AlBeheira, Egypt.
(6)Department of Botany and Microbiology, College of Science, King Saud University, Riyadh 11451, Saudi Arabia.
(7)College of Veterinary Medicine Animal Resources and Biosecurity, Makerere University, Box 7062 Kampala, Uganda.
(8)Kampala International University Western Campus, Box 71 Bushenyi, Uganda.
(9)Faculty of Agriculture and Environmental Sciences, Kabale University, Box 315 Kabale, Uganda.
(10)Department of Veterinary Population Medicine, College of Veterinary Medicine, University of Minnesota, USA.
BACKGROUND: Information as regards the epidemiology of the Anaplasmataceae in small ruminants in several low- and middle-income countries is scarce.
METHODS: In this study a total of 712 DNA samples collected from small ruminants were analyzed for Anaplasmataceae and Anaplasma ovis using the 16S rRNA and MSP4 genes respectively. Infection risk was assessed by location, sex and age of the animals and qGIS® was used to construct spatial maps.
RESULTS: The prevalence of Anaplasmataceae spp was 89.1% (95% CI: 77.5-95.9) and 79.1% (95% CI: 75.9-82.1) in ovines and caprines respectively (RR = 1.1, 95% CI: 1.0-1.3); higher than those previously reported in other eastern African countries. The prevalence of A. ovis was 26.1% and 25.4% for both ovines and caprines respectively with ovines showing significantly higher levels of infection than caprines (P < 0.05). The risk of Anaplasma ovis infections was not affected by age (OR = 1.2, 95% CI: 0.9-1.7) or sex (OR = 1.1, 95% CI: 0.6-2.0). Small ruminants located at the forest edge (<0.3 km) showed higher A. ovis prevalence than those found inland with infections present in the midland regions associated with increased agricultural activity.
CONCLUSION: Anaplasma ovis remains a major challenge for small ruminant husbandry in Uganda and infections are under-reported. Policy efforts to prioritize management of Anaplasmataceae for small ruminant health would promote livestock productivity in vulnerable communities, improving livelihoods and ecosystem health.

149. J Virol Methods. 2021 Apr;290:114075. doi: 10.1016/j.jviromet.2021.114075. Epub
Serological and molecular study of Crimean-Congo Hemorrhagic Fever Virus in cattle from selected districts in Uganda.
Balinandi S(1), von Brömssen C(2), Tumusiime A(3), Kyondo J(4), Kwon H(5), Monteil VM(6), Mirazimi A(7), Lutwama J(8), Mugisha L(9), Malmberg M(10).
Author information:

(1)Uganda Virus Research Institute, P.O. Box 49, Entebbe, Uganda; College of Veterinary Medicine, Animal Resources and Biosecurity, Makerere University, P.O. Box 7062, Kampala, Uganda. Electronic address: sbalinandi@uvri.go.ug.
(2)Division of Applied Statistics and Mathematics, Department of Engergy and Technology, Swedish University of Agricultural Sciences, Box 7013, Uppsala, 750 07, Sweden. Electronic address: claudia.von.bromssen@slu.se.
(3)Uganda Virus Research Institute, P.O. Box 49, Entebbe, Uganda. Electronic address: tumusiimeug@yahoo.co.uk.
(4)Uganda Virus Research Institute, P.O. Box 49, Entebbe, Uganda. Electronic address: jacksonkyondo@gmail.com.
(5)National Veterinary Institute, Uppsala, 751 89, Sweden. Electronic address: hyesoo.kwon@sva.se.
(6)Department of Laboratory Medicine, Karolinska Institute and Karolinska Hospital University, Stockholm, 171 77, Sweden. Electronic address: vanessa.monteil@ki.se.
(7)National Veterinary Institute, Uppsala, 751 89, Sweden; Department of Laboratory Medicine, Karolinska Institute and Karolinska Hospital University, Stockholm, 171 77, Sweden; Public Health Agency of Sweden, Stockholm, 171 82, Sweden. Electronic address: ali.mirazimi@folkhalsomyndigheten.se.
(8)Uganda Virus Research Institute, P.O. Box 49, Entebbe, Uganda. Electronic address: jlutwama@uvri.go.ug.
(9)College of Veterinary Medicine, Animal Resources and Biosecurity, Makerere University, P.O. Box 7062, Kampala, Uganda; Ecohealth Research Group, Conservation & Ecosystem Health Alliance (CEHA), Box 34153, Kampala, Uganda. Electronic address: lmugisha@covab.mak.ac.ug.
(10)Section of Virology, Department of Biomedical Sciences and Veterinary Public Health, Swedish University of Agricultural Sciences, Box 7028, Uppsala, 750 07, Sweden; SLU Global Bioinformatics Centre, Department of Animal Breeding and Genetics, Swedish University of Agricultural Sciences, Box 7023, Uppsala, 750 07, Sweden. Electronic address: maja.malmberg@slu.se.
BACKGROUND: Crimean-Congo Hemorrhagic Fever (CCHF) is a severe tick-borne viral hemorrhagic disease caused by Crimean-Congo Hemorrhagic Fever Virus (CCHFV) that poses serious public health challenges in many parts of Africa, Europe and Asia.
METHODS: We examined 500 cattle sera samples from five districts for CCHFV antibodies using in-house and commercially available (IDVet) ELISA, Immunofluorescent assay (IFA) and Real-time polymerase chain reaction (RT-PCR).
RESULTS: 500 cattle (73.8 % females) were analyzed; CCHFV seropositivity was 12.6 % (n = 63) and 75.0 % (n = 375) with the in-house and IDVet ELISAs, respectively. Seropositivity was associated with geographical location, increasing age, being female, and having a higher tick burden. Twenty four out of the 37 (64.8 %) were seropositive for CCHFV using IFA and all were negative for virus on RT-PCR. The IFA results were more comparable to IDVet (κcoefficient = 0.88, p = <0.01) than to in-house (κcoefficient = 0.32, p = 0.02).
CONCLUSIONS: Our study confirmed the presence and high prevalence of anti-CCHF antibodies in cattle based on three methods from all the five study districts, confirming presence and exposure of CCHFV. Given the zoonotic potential for

CCHFV, we recommend a multidisciplinary public health surveillance and epidemiology of CCHFV in both animals and humans throughout the country.

150. Microorganisms. 2021 Feb 20;9(2):438. doi: 10.3390/microorganisms9020438.
Phylogenetic Characterization of Crimean-Congo Hemorrhagic Fever Virus Detected in African Blue Ticks Feeding on Cattle in a Ugandan Abattoir.
Wampande EM(1), Waiswa P(1), Allen DJ(2), Hewson R(2)(3), Frost SDW(4)(5), Stubbs SCB(4)(6).
Author information:
(1)College of Veterinary Medicine, Animal Resources and Biosecurity, Makerere University, Kampala, Uganda.
(2)Department of Infection Biology, London School of Hygiene and Tropical Medicine, London WC1E 7HT, UK.
(3)Virology and Pathogenesis Group, Public Health England, Porton Down, Salisbury SP4 0JG, UK.
(4)Department of Infectious Disease Epidemiology, London School of Hygiene and Tropical Medicine, London WC1E 7HT, UK.
(5)Microsoft Research, Redmond, Washington, DC 98052, USA.
(6)Department of Veterinary Medicine, University of Cambridge, Cambridge CB3 0ES, UK.
Crimean-Congo hemorrhagic fever virus (CCHFV) is the most geographically widespread of the tick-borne viruses. However, African strains of CCHFV are poorly represented in sequence databases. In addition, almost all sequence data collected to date have been obtained from cases of human disease, while information regarding the circulation of the virus in tick and animal reservoirs is severely lacking. Here, we characterize the complete coding region of a novel CCHFV strain, detected in African blue ticks (Rhipicephalus (Boophilus) decoloratus) feeding on cattle in an abattoir in Kampala, Uganda. These cattle originated from a farm in Mbarara, a major cattle-trading hub for much of Uganda. Phylogenetic analysis indicates that the newly sequenced strain belongs to the African genotype II clade, which predominantly contains the sequences of strains isolated from West Africa in the 1950s, and South Africa in the 1980s. Whilst the viral S (nucleoprotein) and L (RNA polymerase) genome segments shared >90% nucleotide similarity with previously reported genotype II strains, the glycoprotein-coding M segment shared only 80% nucleotide similarity with the next most closely related strains, which were derived from ticks in Western India and Northern China. This genome segment also displayed a large number of non-synonymous mutations previously unreported in the genotype II strains. Characterization of this novel strain adds to our limited understanding of the natural diversity of CCHFV circulating in both ticks and in Africa. Such data can be used to inform the design of vaccines and diagnostics, as well as studies exploring the epidemiology and evolution of the virus for the establishment of future CCHFV control strategies.

151. PLoS Negl Trop Dis. 2021 Mar 8;15(3):e0009213. doi: 10.1371/journal.pntd.0009213. eCollection 2021 Mar.
Sporadic outbreaks of crimean-congo haemorrhagic fever in Uganda, July

2018-January 2019.
Mirembe BB(1), Musewa A(1), Kadobera D(1), Kisaakye E(1), Birungi D(1), Eurien D(1), Nyakarahuka L(2)(3), Balinandi S(2)(3), Tumusiime A(2), Kyondo J(2), Mulei SM(2), Baluku J(2), Kwesiga B(1), Kabwama SN(1), Zhu BP(4)(5), Harris JR(4)(5), Lutwama JJ(2), Ario AR(1)(6).
Author information:
(1)Uganda Public Health Fellowship Program, Ministry of Health, Kampala, Uganda.
(2)Uganda Virus Research Institute, Entebbe, Uganda.
(3)School of Biosecurity, Biotechnical and Laboratory Sciences, College of Veterinary Medicine, Animal Resources & Biosecurity, Makerere University, Kampala, Uganda.
(4)US Centers for Disease Control and Prevention, Kampala, Uganda.
(5)Division of Global Health Protection, Center for Global Health, Atlanta, GA, United States of America.
(6)Ministry of Health, Kampala, Uganda.
INTRODUCTION: Crimean-Congo haemorrhagic fever (CCHF) is a tick-borne, zoonotic viral disease that causes haemorrhagic symptoms. Despite having eight confirmed outbreaks between 2013 and 2017, all within Uganda's 'cattle corridor', no targeted tick control programs exist in Uganda to prevent disease. During a seven-month-period from July 2018-January 2019, the Ministry of Health confirmed multiple independent CCHF outbreaks. We investigated to identify risk factors and recommend interventions to prevent future outbreaks.
METHODS: We defined a confirmed case as sudden onset of fever (≥37.5°C) with ≥4 of the following signs and symptoms: anorexia, vomiting, diarrhoea, headache, abdominal pain, joint pain, or sudden unexplained bleeding in a resident of the affected districts who tested positive for Crimean-Congo haemorrhagic fever virus (CCHFv) by RT-PCR from 1 July 2018-30 January 2019. We reviewed medical records and performed active case-finding. We conducted a case-control study and compared exposures of case-patients with age-, sex-, and sub-county-matched control-persons (1:4).
RESULTS: We identified 14 confirmed cases (64% males) with five deaths (case-fatality rate: 36%) from 11 districts in western and central region. Of these, eight (73%) case-patients resided in Uganda's 'cattle corridor'. One outbreak involved two case-patients and the remainder involved one. All case-patients had fever and 93% had unexplained bleeding. Case-patients were aged 6-36 years, with persons aged 20-44 years more affected (AR: 7.2/1,000,000) than persons ≤19 years (2.0/1,000,000), p = 0.015. Most (93%) case-patients had contact with livestock ≤2 weeks before symptom onset. Twelve (86%) lived <1 km from grazing fields compared with 27 (48%) controls (ORM-H = 18, 95% CI = 3.2-∞) and 10 (71%) of 14 case-patients found ticks attached to their bodies ≤2 weeks before symptom onset, compared to 15 (27%) of 56 control-persons (ORM-H = 9.3, 95%CI = 1.9-46).
CONCLUSIONS: CCHF outbreaks occurred sporadically during 2018-2019, both within and outside 'cattle corridor' districts of Uganda. Most cases were associated with tick exposure. The Ministry of Health should partner with the Ministry of Agriculture, Animal Industry and Fisheries to develop joint nationwide tick control programs and strategies with shared responsibilities through a One

Health approach.

152. Ticks Tick Borne Dis. 2021 Jul;12(4):101709. doi: 10.1016/j.ttbdis.2021.101709.
Molecular detection and characterisation of protozoan and rickettsial pathogens in ticks from cattle in the pastoral area of Karamoja, Uganda.
Byaruhanga C(1), Akure PC(2), Lubembe DM(3), Sibeko-Matjila K(2), Troskie M(2), Oosthuizen MC(2), Stoltsz H(2).
Author information:
(1)Vectors and Vector-Borne Diseases Research Programme, Department of Veterinary Tropical Diseases, Faculty of Veterinary Science, University of Pretoria, Private Bag X04, Onderstepoort, 0110, South Africa; National Agricultural Research Organisation, P.O. Box 259, Entebbe, Uganda. Electronic address: cbyaruhanga27@yahoo.com.
(2)Vectors and Vector-Borne Diseases Research Programme, Department of Veterinary Tropical Diseases, Faculty of Veterinary Science, University of Pretoria, Private Bag X04, Onderstepoort, 0110, South Africa.
(3)Vectors and Vector-Borne Diseases Research Programme, Department of Veterinary Tropical Diseases, Faculty of Veterinary Science, University of Pretoria, Private Bag X04, Onderstepoort, 0110, South Africa; Department of Veterinary Pathology, Microbiology and Parasitology, Faculty of Veterinary Medicine and Surgery, Egerton University, P.O. Box 536, Egerton, 20115, Kenya.
Ticks and tick-borne diseases (TBDs) significantly affect cattle production and the livelihoods of communities in pastoralist areas. Data on protozoan and rickettsial pathogens in ticks infesting cattle in Uganda is scanty; while it is an indicator of the likelihood of disease transmission and occurrence. A cross-sectional study was conducted amongst cattle in the Karamoja Region, northeastern Uganda, from July through September 2017, to determine the tick species diversity, identify protozoan and rickettsial pathogens in the ticks, and characterise pathogenic species by sequence and phylogenetic analyses. About 50 % of the ticks detected from each predilection site on each animal were collected from 100 purposively-selected cattle from 20 randomly-selected herds. Twelve tick species belonging to the genera Amblyomma, Rhipicephalus and Hyalomma were identified, the most abundant being Amblyomma lepidum (93.9 %), followed by Amblyomma variegatum (2.0 %) and Rhipicephalus evertsi evertsi (1.0 %). Tick species that have not been reported in recent studies amongst cattle in Uganda were found, namely Rhipicephalus pravus, Rhipicephalus praetextatus and Rhipicephalus turanicus. The ticks were grouped into 40 pools, by species and location, and the reverse line blot (RLB) hybridisation assay was used to detect pathogens from the ticks. The most frequently detected tick-borne parasites were Theileria mutans, Theileria velifera and Theileria parva, each observed in 25 % (10/40) of the tick pools. Tick-borne pathogens, namely Babesia rossi, Babesia microti and Theileria sp. (sable) that are not common to, or not known to infect, cattle were identified from ticks. The gene encoding Ehrlichia ruminantium pCS20 region, the Ehrlichia and Anaplasma 16S rRNA gene, and T. parva p67 sporozoite antigen gene were amplified, cloned and sequenced. Seven novel E. ruminantium pCS20 variants were identified, and these grouped into two separate clusters with sequences from other parts of Africa and Asia. The T.

parva p67 sequences were of the allele type 1, and parasites possessing this allele type are commonly associated with East Coast fever in eastern Africa. Analysis of the Ehrlichia and Anaplasma 16S rRNA gene sequences showed that they were closely related to Rickettsia africae and to a new Ehrlichia species variant recently found in China. Our R. africae 16S rRNA sequences grouped with R. africae isolates from Nigeria, Egypt and Benin. The information on tick species diversity and pathogens in the various tick species provides an indicator of potential transmission amongst cattle populations, and to humans, and can be useful to estimate disease risk and in control strategies.

153. Front Vet Sci. 2021 Mar 22;8:616865. doi: 10.3389/fvets.2021.616865. eCollection
Controlling Tsetse Flies and Ticks Using Insecticide Treatment of Cattle in Tororo District Uganda: Cost Benefit Analysis.
Okello WO(1)(2), MacLeod ET(1), Muhanguzi D(1)(3), Waiswa C(1)(3)(4), Welburn SC(1)(5).
Author information:
(1)Infection Medicine, Deanery of Biomedical Sciences, College of Medicine and Veterinary Medicine, The University of Edinburgh, Edinburgh, United Kingdom.
(2)Commonwealth Scientific and Industrial Research Organisation, Black Mountain Science and Innovation Park, Canberra, ACT, Australia.
(3)Department of Biomolecular and Biolaboratory Sciences, School of Biosecurity, Biotechnical and Laboratory Sciences, College of Veterinary Medicine Animal Resources and Biosecurity, Makerere University, Kampala, Uganda.
(4)The Coordinating Office for Control of Trypanosomiasis in Uganda, Kampala, Uganda.
(5)Zhejiang University-University of Edinburgh Institute, Zhejiang University, International Campus, Haining, China.
Background: The endemic vector-borne diseases transmitted by tsetse and ticks impose heavy burdens on the livestock keepers in Africa. Applying deltamethrin to the belly, legs, and ears of cattle offers a possibility of mitigating these losses at a cost affordable to livestock keepers. Although studies have quantified the impacts of individual diseases on livestock productivity, little is known about the dual economic benefits of controlling both tsetse and ticks, nor about the number of cattle that need to be treated to confer these benefits. Alongside an epidemiological study in south-east Uganda, a farm level assessment was done to investigate the benefits and costs of spraying different proportions of the village cattle population using this restricted application protocol.
Methods: A study comprising 1,902 semi-structured interviews was undertaken over a period of 18 months. Financial data on household income and expenditure on cattle was collected, and cost-benefit analysis was done pre- and post-intervention and for different spraying regimes. The total cost of the intervention was obtained from the implementation costs of the epidemiological study and from expenses incurred by participating farmers enabling examination of benefit-cost ratios and incremental benefit-cost ratios for each treatment regime. Results: The benefit-cost analysis of spraying 25%, 50%, and 75% of the cattle population yielded average benefit-cost ratios of 3.85, 4.51, and 4.46. The incremental benefit-cost ratios from spraying each additional 25% of the

cattle population were 11.38, 3.89, and 0.79, showing a very high return on investment for spraying 50% of the population, with returns reducing thereafter.
Conclusion: Comparing the gross margins per bovine, the study found that increasing the proportion of cattle sprayed yielded increasing benefits to the farmers, but that these benefits were subject to diminishing returns. From a practical viewpoint, this study recommends spraying only draft cattle to control trypanosomiasis and tick-borne diseases in this area as they make 38.62% of the cattle population, approaching the 50% threshold. In areas with a lower proportion of draft males, farmers could be advised to also include cows.

154. PLoS One. 2021 May 17;16(5):e0251500. doi: 10.1371/journal.pone.0251500.
Molecular characterization and population genetics of Theileria parva in Burundi's unvaccinated cattle: Towards the introduction of East Coast fever vaccine.
Atuhaire DK(1), Muleya W(2), Mbao V(3), Niyongabo J(4), Nyabongo L(4), Nsanganiyumwami D(4), Salt J(5), Namangala B(6), Musoke AJ(7).
Author information:
(1)Centre for Ticks and Tick-Borne Diseases, Lilongwe, Malawi.
(2)Department of Biomedical Sciences, School of Veterinary Medicine, University of Zambia, Lusaka, Zambia.
(3)International Development Research Centre, Eastern and Southern Africa Regional Office, Nairobi, Kenya.
(4)National Veterinary Research Laboratory, Directorate of Animal Health, Bujumbura, Burundi.
(5)Global Alliance for Livestock Veterinary Medicines, Pentlands Science Park, Bush Loan, Penicuik Edinburgh, Scotland.
(6)Department of Paraclinical Studies, School of Veterinary Medicine, University of Zambia, Lusaka, Zambia.
(7)LMK Medical laboratories and Consultancies, Kampala, Uganda.
Theileria parva (T. parva) is a protozoan parasite that causes East Coast fever (ECF). The disease is endemic in Burundi and is a major constraint to livestock development. In this study, the parasite prevalence in cattle in six regions namely; Northern, Southern, Eastern, Western, Central and North Eastern was estimated. Furthermore, the sequence diversity of p67, Tp1 and Tp2 genes was assessed coupled with the population genetic structure of T. parva using five satellite markers. The prevalence of ECF was 30% (332/1109) on microscopy, 60% (860/1431) on ELISA and 79% (158/200) on p104 gene PCR. Phylogenetic analysis of p67 gene revealed that only allele 1 was present in the field samples. Furthermore, phylogenetic analysis of Tp1 and Tp2 showed that the majority of samples clustered with Muguga, Kiambu and Serengeti and shared similar epitopes. On the other hand, genetic analysis revealed that field samples shared only two alleles with Muguga Cocktail. The populations from the different regions indicated low genetic differentiation (FST = 0.047) coupled with linkage disequilibrium and non-panmixia. A low to moderate genetic differentiation (FST = 0.065) was also observed between samples and Muguga cocktail. In conclusion, the data presented revealed the presence of a parasite population that shared similar epitopes with Muguga Cocktail and was moderately genetically

differentiated from it. Thus, use of Muguga Cocktail vaccine in Burundi is likely to confer protection against T. parva in field challenge trials.

155. Glob Heart. 2021 Apr 27;16(1):28. doi: 10.5334/gh.996.
The RHD Action Small Grants Programme: Small Investment, Big Return!
Abdullahi L(1), Albertus C(2), Perkins S(2), Ralston K(3), White A(3), Mwangi J(4), Zühlke LJ(2)(4)(5)(6).
Author information:
(1)African Institute for Development Policy (AFIDEP). Nairobi, KE.
(2)Reach, Cape Town, ZA.
(3)World Heart Federation, Geneva, CH.
(4)Reach, Geneva, CH.
(5)Division of Pediatric Cardiology, Department of Pediatrics, Red Cross War Memorial Children's Hospital, University of Cape Town, Cape Town, ZA.
(6)Division of Cardiology, Department of Medicine, Groote Schuur Hospital, University of Cape Town, Cape Town, ZA.
Rheumatic Heart Disease (RHD) remains endemic in low- and middle-income countries (LMICs) despite its virtual elimination in high-income countries. RHD Action was launched to amplify global efforts to control RHD in 2015 by World Heart Federation and Reach, with demonstration projects in Uganda and Tanzania, and support from Medtronic Foundation. The Small Grants Programme focuses on three domains: People and Communities, Medicines and Technologies, and Systems and Services. It is designed to support patient and community groups in promoting awareness, advocacy, and to build health workers' capacity to prevent and treat RHD in LMICs. Our study evaluates the impact and effectiveness of the RHD Action Small Grants Programme. Methods: We conducted a mixed method study that involved both quantitative and qualitative surveys, through phone interviews and online surveys amongst the grant beneficiaries, to assess the impact and effectiveness of the small grant programme. An invitation to complete an online survey, using a Google Forms format, was issued to Small Grant Project Directors and Co-Directors that received funding for projects between 2017 and 2019. The online survey requested basic project information using tick boxes, Likert scales, and short answer open-ended questions about successes and challenges faced by recipients. The questionnaire also addressed recipients' experience with the RHD Action Small Grants process - applying for the grant, nature and quality of support received to carry out project, the reporting process, and any media coverage provided. For the phone interviews, responses to the short-answer questions were used as the basis for follow up phone interviews. The discussions were recorded, transcribed and thematically analysed for new and recurring themes emerging from the in-depth discussions. Initiated in 2017, RHD Action has funded 21 proposals from a pool of 60 submissions. Recipient countries include Zambia, Uganda (2), Namibia, Kenya, Malawi (2), Egypt, Ethiopia, Nigeria (3), Rwanda (2), Mozambique, and Cameroon (2) as well as Fiji (2), the Philippines and Nepal. Five recipients were funded in 2017, eight in 2018 and eight in 2019. Project directors are primarily junior doctors and project managers supervised by senior mentors. In most cases, this is their first funding award. These projects have demonstrated tangible impact and have

provided content for first manuscript and abstract submissions and presentations at professional conferences. Grant reports are presented as website stories showcasing the achievements of small local efforts with meaningful impact. For RHD Action, there is large return on a modest monetary investment resulting in a very visible, viable global RHD networking platform for enthusiastic community and provider activists.

156. Viruses. 2021 May 8;13(5):860. doi: 10.3390/v13050860.
Bat Flies of the Family Streblidae (Diptera: Hippoboscoidea) Host Relatives of Medically and Agriculturally Important "Bat-Associated" Viruses.
Ramírez-Martínez MM(1), Bennett AJ(2)(3), Dunn CD(2), Yuill TM(2), Goldberg TL(2).
Author information:
(1)Departamento de Ciencias de la Salud y Ecología Humana, Universidad de Guadalajara, Guadalajara, Autlán CP 48900, Mexico.
(2)Department of Pathobiological Sciences, School of Veterinary Medicine, University of Wisconsin-Madison, Madison, WI 53706, USA.
(3)Genomics and Bioinformatics Department, Biological Defense Research Directorate, Naval Medical Research Center-Frederick, Fort Detrick, Frederick, MD 21702, USA.
Bat flies (Hippoboscoidea: Nycteribiidae and Streblidae) are obligate hematophagous ectoparasites of bats. We collected streblid bat flies from the New World (México) and the Old World (Uganda), and used metagenomics to identify their viruses. In México, we found méjal virus (Rhabdoviridae; Vesiculovirus), Amate virus (Reoviridae: Orbivirus), and two unclassified viruses of invertebrates. Méjal virus is related to emerging zoonotic encephalitis viruses and to the agriculturally important vesicular stomatitis viruses (VSV). Amate virus and its sister taxon from a bat are most closely related to mosquito- and tick-borne orbiviruses, suggesting a previously unrecognized orbivirus transmission cycle involving bats and bat flies. In Uganda, we found mamucuso virus (Peribunyaviridae: Orthobunyavirus) and two unclassified viruses (a rhabdovirus and an invertebrate virus). Mamucuso virus is related to encephalitic viruses of mammals and to viruses from nycteribiid bat flies and louse flies, suggesting a previously unrecognized orthobunyavirus transmission cycle involving hippoboscoid insects. Bat fly virus transmission may be neither strictly vector-borne nor strictly vertical, with opportunistic feeding by bat flies occasionally leading to zoonotic transmission. Many "bat-associated" viruses, which are ecologically and epidemiologically associated with bats but rarely or never found in bats themselves, may actually be viruses of bat flies or other bat ectoparasites.

157. Ticks Tick Borne Dis. 2021 Sep;12(5):101756. doi: 10.1016/j.ttbdis.2021.101756.
Cattle ticks and tick-borne diseases: a review of Uganda's situation.
Kasaija PD(1), Estrada-Peña A(2), Contreras M(3), Kirunda H(4), de la Fuente J(5).
Author information:
(1)SaBio, Instituto de Investigación en Recursos Cinegéticos (IREC), Consejo

Superior de Investigaciones Científicas (CSIC), Universidad de Castilla-La Mancha (UCLM)-Junta de Comunidades de Castilla-La Mancha (JCCM), Ronda de Toledo s/n, 13005 Ciudad Real, Spain; National Livestock Resources Research Institute (NaLIRRI/NARO), P.O. Box 5704, Kampala, Uganda.
(2)Department of Animal Pathology, Faculty of Veterinary Medicine, 50013 Zaragoza, Spain.
(3)SaBio, Instituto de Investigación en Recursos Cinegéticos (IREC), Consejo Superior de Investigaciones Científicas (CSIC), Universidad de Castilla-La Mancha (UCLM)-Junta de Comunidades de Castilla-La Mancha (JCCM), Ronda de Toledo s/n, 13005 Ciudad Real, Spain; Interdisciplinary Laboratory of Clinical Analysis, Interlab-UMU, Regional Campus of International Excellence Campus Mare Nostrum, University of Murcia, Espinardo, 30100 Murcia, Spain.
(4)National Livestock Resources Research Institute (NaLIRRI/NARO), P.O. Box 5704, Kampala, Uganda.
(5)SaBio, Instituto de Investigación en Recursos Cinegéticos (IREC), Consejo Superior de Investigaciones Científicas (CSIC), Universidad de Castilla-La Mancha (UCLM)-Junta de Comunidades de Castilla-La Mancha (JCCM), Ronda de Toledo s/n, 13005 Ciudad Real, Spain; Department of Veterinary Pathobiology, Center for Veterinary Health Sciences, Oklahoma State University, Stillwater, OK 74078, USA. Electronic address: josedejesus.fuente@uclm.es.
Herein we review the epidemiology of ticks and tick-borne diseases (TTBDs), their impact on livestock health and on the economy, control and associated challenges in Uganda. Ticks are leading vectors of economically important pathogens and are widespread in Uganda due to suitable climatic conditions. Besides the physical injury inflicted on the animal host, ticks transmit a number of pathogens that can cause morbidity and mortality of livestock if untreated, resulting in economic losses. Uganda suffers an aggregated annual loss (direct and indirect) of over USD 1.1 billion in the TTBDs complex. East Coast fever (ECF) caused by a protozoan haemoparasite, Theileria parva, is the most prevalent and economically important tick-borne disease (TBD) in Uganda and its vector, the brown ear tick (Rhipicephalus appendiculatus) widely distributed. Other prevalent TBDs in Uganda include anaplasmosis, babesiosis and heartwater. We highlight the role of agro-ecological zones (AEZs) and livestock management system in the distribution of TTBDs, citing warm and humid lowlands as being ideal habitats for ticks and endemic for TBDs. Control of TTBDs is a matter of great importance as far as animal health is concerned in Uganda. Indigenous cattle, which make up over 90% of the national herd are known to be more tolerant to TTBDs and most farms rely on endemic stability to TBDs for control. However, exotic cattle breeds are more capital intensive than indigenous breeds, but the increasing adoption of tick-susceptible exotic cattle breeds (especially dairy) in western and central Uganda demands intensive use of acaricides for tick control and prevention of TBDs. Such acaricide pressure has unfortunately led to selection of acaricide-resistant tick populations and the consequent acaricide resistance observed in the field. Vaccination against ECF, selective breeding for tick resistance and integrated tick control approaches that limit tick exposure, could be adopted to interrupt spread of acaricide resistance. We recommend increasing monitoring and surveillance for TTBDs and

for emerging acaricide resistance, improved extension services and sensitization of farmers on tick control measures, appropriate acaricide use and the development and implementation of vaccines for the control of TTBDs as more sustainable and effective interventions. A tick control policy should be developed, taking into account variations of agro-ecological zones, farm circumstances and indigenous technical knowledge, and this should be incorporated into the overall animal health program.

158. Acta Parasitol. 2022 Mar;67(1):72-78. doi: 10.1007/s11686-021-00433-0. Epub 2021
Molecular Evidence and Hematological Profile of Bovines Naturally Infected with Ehrlichiosis in Southern Punjab, Pakistan.
Basit MA(1), Ijaz M(2), Khan JA(1), Ashraf K(3), Abbas RZ(4).
Author information:
(1)Department of Veterinary Medicine, University of Veterinary and Animal Sciences, Lahore, 54600, Pakistan.
(2)Department of Veterinary Medicine, University of Veterinary and Animal Sciences, Lahore, 54600, Pakistan. mijaz@uvas.edu.pk.
(3)Department of Parasitology, University of Veterinary and Animal Sciences, Lahore, 54600, Pakistan.
(4)Department of Parasitology, University of Agriculture, Faisalabad, 38040, Pakistan.
PURPOSE: Ehrlichiosis is one of the tick-borne diseases, which is not only neglected in Pakistan but only a few reports have been documented throughout the globe. The purpose of this study is to highlight and report the neglected pathogen from bovines in Pakistan.
METHODS: In this study, the pathogen was detected initially based on microscopy, followed by the molecular confirmation and phylogenetic analysis of the pathogen from bovines from south Punjab, Pakistan. The hematological parameters were also assessed in Ehrlichia positive and negative animals. The information of different disease determinants was analyzed by a logistic regression model on SPSS.
RESULTS: The study has reported an 11.98% (23/192) prevalence of Ehrlichiosis from bovines. The prevalence was slightly more in cattle (13.5%) as compared to the buffaloes (10.4%). The previous tick's history and tick control methods were proved to be the key factors with the occurrence of the disease. The isolates from Pakistan Ehrlichia spp. Pakistan/31, 36, and 8 clustered with the isolates from the USA, Nicaraguan, France, South Africa, and Uganda. Platelet count, hemoglobin level, and hematocrit were found significantly decreased in Ehrlichia affected animals when compared to the healthy bovines.
CONCLUSION: This is the first report of ehrlichiosis from bovines in Pakistan and will provide the roadmap for future research.

159. Ticks Tick Borne Dis. 2021 Sep;12(5):101772. doi: 10.1016/j.ttbdis.2021.101772.
Molecular detection of selected tick-borne pathogens infecting cattle at the wildlife-livestock interface of Queen Elizabeth National Park in Kasese District, Uganda.
Byamukama B(1), Vudriko P(2), Tumwebaze MA(1), Tayebwa DS(3), Byaruhanga J(4),

Angwe MK(4), Li J(5), Galon EM(5), Ringo A(5), Liu M(5), Li Y(5), Ji S(5), Rizk MA(6), Moumouni PFA(5), Lee SH(7), Sevinc F(8), Xuan X(9).
Author information:
(1)National Research Center for Protozoan Diseases, Obihiro University of Agriculture and Veterinary Medicine, Obihiro, Hokkaido 080-8555, Japan; Research Center for Tropical Diseases and Vector Control, School of Veterinary Medicine and Animal Resources, Makerere University, 7062, Kampala, Uganda.
(2)Research Center for Tropical Diseases and Vector Control, School of Veterinary Medicine and Animal Resources, Makerere University, 7062, Kampala, Uganda; Department of Veterinary Pharmacy, Clinics & Comparative Medicine, School of Veterinary Medicine and Animal Resources, College of Veterinary Medicine, Animal Resources and Biosecurity, Makerere University, 7062, Kampala, Uganda. Electronic address: vpato2009@gmail.com.
(3)Research Center for Tropical Diseases and Vector Control, School of Veterinary Medicine and Animal Resources, Makerere University, 7062, Kampala, Uganda; Department of Veterinary Pharmacy, Clinics & Comparative Medicine, School of Veterinary Medicine and Animal Resources, College of Veterinary Medicine, Animal Resources and Biosecurity, Makerere University, 7062, Kampala, Uganda.
(4)Research Center for Tropical Diseases and Vector Control, School of Veterinary Medicine and Animal Resources, Makerere University, 7062, Kampala, Uganda.
(5)National Research Center for Protozoan Diseases, Obihiro University of Agriculture and Veterinary Medicine, Obihiro, Hokkaido 080-8555, Japan.
(6)National Research Center for Protozoan Diseases, Obihiro University of Agriculture and Veterinary Medicine, Obihiro, Hokkaido 080-8555, Japan; Department of Internal Medicine and Infectious Diseases, Faculty of Veterinary Medicine, Mansoura University, Mansoura 35516, Egypt.
(7)College of Veterinary Medicine, Chungbuk National University, Cheongju 28644, Korea.
(8)Faculty of Veterinary Medicine, University of Selcuk, Konya, Turkey.
(9)National Research Center for Protozoan Diseases, Obihiro University of Agriculture and Veterinary Medicine, Obihiro, Hokkaido 080-8555, Japan. Electronic address: gen@obihiro.ac.jp.
In Uganda, ticks and tick-borne diseases (TBDs) pose a big challenge to farmers. They reduce cattle productivity and cause severe economic damage. Several studies have documented the prevalence of tick-borne pathogens in cattle; however, their genetic characteristics and the role of wildlife-livestock interaction in the epidemiology of the TBDs are not well documented. This study assessed the prevalence and genetic diversity of various tick-borne pathogens (TBPs) as well as the risk factors associated with the occurrence of TBPs in blood samples of 208 randomly selected cattle from 16 farms located around Queen Elizabeth National Park (QENP) in Kasese District in western Uganda. Farming practices, disease challenges, and the level of wildlife-livestock interactions were assessed by a questionnaire survey amongst farm owners. Polymerase chain reaction (PCR) assays revealed that 62.9% (131/208) cattle samples were positive for one or more pathogens. Using specific PCR assays, we detected Theileria

spp., Theileria parva, Anaplasma marginale, Anaplasma platys-like, and Babesia bigemina at 50.5%, 27.9%, 19.2%, 11.5% and 8.7%, respectively. We also confirmed the infection of samples by Theileria velifera and Theileria mutans after sequencing the Theileria spp. 18S rRNA gene. The risk factors associated with the occurrence of TBPs included communal grazing, herd size, age, and proximity to QENP. Phylogenetic analysis of the T. parva p104 gene showed a high identity to the previous isolates from Uganda and other East African countries and clustered closer to the buffalo (Syncerus caffer) isolates, suggesting a possible cross-species transmission. The sequences of A. marginale groEL and B. bigemina RAP-1a formed well-supported clades with high identities to the previous isolates identified from central and eastern Uganda. The isolates obtained from A. phagocytophilum 16S rRNA gene sequences showed relationship with A. platys-like, Anaplasma sp., uncultured Anaplasma species and A. phagocytophilum isolates from Africa, Asia, Europe, and the USA. The findings of the present study showed that TBDs are still a burden to farmers and that management practices in this area may increase the transmission of pathogens between livestock and wildlife.

160. Pathogens. 2021 Jul 15;10(7):899. doi: 10.3390/pathogens10070899.
A Scoping Review of West Nile Virus Seroprevalence Studies among African Equids.
Olufemi OT(1)(2), Barba M(3)(4), Daly JM(1).
Author information:
(1)One Virology-Wolfson Centre for Global Virus Research, School of Veterinary Medicine and Science, University of Nottingham, Loughborough LE12 5RD, UK.
(2)Department of Veterinary Public Health and Preventive Medicine, Faculty of Veterinary Medicine, University of Jos, Jos 930003, Nigeria.
(3)Microbiological Agents Associated with Animal Reproduction (ProVaginBio), Veterinary Faculty, University Cardenal Herrera-CEU, CEU Universities, 46115 Alfara del Patriarca, Spain.
(4)Innovation Centre in Rural Bioeconomy, Agrifood Research and Technology Centre of Aragon (CITA), Av. Montañana, 930, 50059 Zaragoza, Spain.
West Nile virus (WNV) is an emerging and re-emerging zoonotic flavivirus first identified in and endemic to Africa. The virus is transmitted between birds by biting mosquitoes, with equids and humans being incidental hosts. The majority of infected incidental hosts display no or only mild clinical signs, but a fraction develop encephalitis. The aim of this scoping review was to identify and evaluate primary research on the presence of antibodies to WNV among African equids. Three bibliographic databases and the grey literature were searched. Of 283 articles identified, only 16 satisfied all the inclusion criteria. Data were collated on study design and outcomes. The overall seroprevalence reported ranged from 17.4 to 90.3%, with 1998 (35%) of the 5746 horses, donkeys and mules having screened positive for WNV antibodies. Several articles determined that seroprevalence increased significantly with age. Due to co-circulation of other flaviviruses in Africa, in the majority of studies that screened samples by ELISA, positive results were confirmed using a more specific neutralization test. However, only eight studies tested against other flaviviruses, including Potiskum, Uganda S, Wesselsbron and yellow fever virus in one, Japanese

encephalitis and Usutu virus (USUV) in one, tick-borne encephalitis and USUV in one and USUV only in three. Equids are regarded as useful sentinel animals for WNV, but variation in study design poses challenges when trying to determine risk factors for, and trends in, WNV seroprevalence.

161. Am J Trop Med Hyg. 2021 Oct 18;106(1):88-98. doi: 10.4269/ajtmh.21-0685.
Clinical and Molecular Epidemiology of Crimean-Congo Hemorrhagic Fever in Humans in Uganda, 2013-2019.
Balinandi S(1)(2), Whitmer S(3), Mulei S(1), Nyakarahuka L(1)(2), Tumusiime A(1), Kyondo J(1), Baluku J(1), Mutyaba J(1), Mugisha L(4)(5), Malmberg M(6)(7), Lutwama J(1), Shoemaker TR(3), Klena JD(3).
Author information:
(1)Department of Arbovirology, Emerging and Re-emerging Infectious Diseases, Uganda Virus Research Institute, Entebbe, Uganda.
(2)School of Biosecurity, Biotechnical and Laboratory Sciences, College of Veterinary Medicine, Animal Resources and Biosecurity, Makerere University, Kampala, Uganda.
(3)Viral Special Pathogens Branch, Centers for Disease Control and Prevention, Atlanta, Georgia, USA.
(4)School of Veterinary Medicine and Animal Resources, College of Veterinary Medicine, Animal Resources and Biosecurity, Makerere University, Kampala, Uganda.
(5)EcoHealth Research Group, Conservation & Ecosystem Health Alliance (CEHA), Kampala, Uganda.
(6)Section of Virology, Department of Biomedical Sciences and Veterinary Public Health, Swedish University of Agricultural Sciences, Uppsala, Sweden.
(7)SLU Global Bioinformatics Centre, Department of Animal Breeding and Genetics, Swedish University of Agricultural Sciences, Uppsala, Sweden.
Crimean-Congo Hemorrhagic Fever (CCHF) is endemic in Uganda, yet its epidemiology remains largely uncharacterized. To better understand its occurrence within Uganda, case reports of patients hospitalized with CCHF between 2013 and 2019 were reviewed. Further, genome sequences of CCHF-positive RNA obtained during this period were determined for phylogenetic comparisons. We found that a total of 32 cases (75% males; CFR, 31.2%), aged between 9 to 68 years, were reported during the study period. Most cases were detected during July to December of each outbreak year (81.2%; $P < 0.01$) and were located along the "cattle corridor" (68.7%, $P = 0.03$). The most common presenting symptoms were fever (93.8%), hemorrhage (81.3%), headache (78.1%), fatigue (68.8%), vomiting (68.8%), and myalgia (65.6%). In five patients for whom hematological data were available, varied abnormalities were observed including thrombocytopenia, leukopenia, anemia, lymphopenia, lymphocytosis, polycythemia, and microcytosis. About 56.3% ($P = 0.47$) of patients reported tick bites or exposure to livestock as their potential source of infection. Person-to-person transmission was suspected for two cases. Using unbiased metagenomics, we found that the viral S- and L- segments have remained conserved in Africa 2 clade since the 1950s. In contrast, the M segment split into two geographically interspersed clades; one that belongs to Africa 2 and another that is ancestral

to Africa 1 and 2. Overall, this data summarizes information on the history and clinical presentation of human CCHF in Uganda. Importantly, it identifies vulnerable populations as well as temporal and geographic regions in Uganda where surveillance and control interventions could be focused.

162. Vaccines (Basel). 2021 Oct 15;9(10):1185. doi: 10.3390/vaccines9101185.
From Bench to Field: A Guide to Formulating and Evaluating Anti-Tick Vaccines Delving beyond Efficacy to Effectiveness.
Ndawula C Jr(1)(2).
Author information:
(1)National Agricultural Research Organization, P.O. Box 295, Entebbe, Wakiso 256, Uganda.
(2)National Livestock Resources Research Institute, Vaccinology Research Programme, P.O. Box 5704, Nakyesasa, Wakiso 256, Uganda.
Ticks are ubiquitous blood-sucking ectoparasites capable of transmitting a wide range of pathogens such as bacteria, viruses, protozoa, and fungi to animals and humans. Although the use of chemicals (acaricides) is the predominant method of tick-control, there are increasing incidents of acaricide tick resistance. Furthermore, there are concerns over accumulation of acaricide residues in meat, milk and in the environment. Therefore, alternative methods of tick-control have been proposed, of which anti-tick cattle vaccination is regarded as sustainable and user-friendly. Over the years, tremendous progress has been made in identifying and evaluating novel candidate tick vaccines, yet none of them have reached the global market. Until now, Bm86-based vaccines (Gavac™ in Cuba and TickGARDPLUS™ Australia-ceased in 2010) are still the only globally commercialized anti-tick vaccines. In contrast to Bm86, often, the novel candidate anti-tick vaccines show a lower protection efficacy. Why is this so? In response, herein, the potential bottlenecks to formulating efficacious anti-tick vaccines are examined. Aside from Bm86, the effectiveness of other anti-tick vaccines is rarely assessed. So, how can the researchers assess anti-tick vaccine effectiveness before field application? The approaches that are currently used to determine anti-tick vaccine efficacy are re-examined in this review. In addition, a model is proposed to aid in assessing anti-tick vaccine effectiveness. Finally, based on the principles for the development of general veterinary vaccines, a pipeline is proposed to guide in the development of anti-tick vaccines.

163. Parasit Vectors. 2021 Nov 16;14(1):576. doi: 10.1186/s13071-021-05014-8.
A community approach of pathogens and their arthropod vectors (ticks and fleas) in dogs of African Sub-Sahara.
Heylen D(1)(2)(3), Day M(4), Schunack B(5), Fourie J(6)(7), Labuschange M(8), Johnson S(9), Githigia SM(10), Akande FA(11), Nzalawahe JS(12), Tayebwa DS(13), Aschenborn O(14), Marcondes M(15), Madder M(16)(17).
Author information:
(1)Eco-Epidemiology Group, Department of Biomedical Sciences, Institute of Tropical Medicine, Antwerp, Belgium. dheylen@itg.be.
(2)Interuniversity Institute for Biostatistics and Statistical Bioinformatics,

Hasselt University, Diepenbeek, Belgium. dheylen@itg.be.
(3)Department of Ecology and Evolutionary Biology, Princeton University, Princeton, NJ, USA. dheylen@itg.be.
(4)School of Veterinary and Life Sciences, Murdoch University, Murdoch, WA, Australia.
(5)Bayer Animal Health, Elanco Animal Health Inc., Leverkusen, Germany.
(6)Clinvet LLC, Waverly, USA.
(7)ClinVet International (Pty) Ltd., Bloemfontein, South Africa.
(8)Clinomics, Bloemfontein, South Africa.
(9)School of Veterinary Medicine, College of Basic and Applied Sciences (CBAS), University of Ghana, Accra, Ghana.
(10)Department of Veterinary Pathology, Microbiology and Parasitology, University of Nairobi, Nairobi, Kenya.
(11)Department of Veterinary Parasitology and Entomology, College of Veterinary Medicine, Federal University of Agriculture, Abeokuta, Nigeria.
(12)Sokoine University of Agriculture, Morogoro, Tanzania.
(13)Research Center for Tropical Diseases and Vector Control, College of Veterinary Medicine, Animal Resources and Biosecurity, Makerere University, Kampala, Uganda.
(14)School of Veterinary Medicine, University of Namibia, Neudamm, Namibia, South Africa.
(15)São Paulo State University, São Paulo, Brazil.
(16)University of Pretoria, Pretoria, South Africa.
(17)Clinglobal, Tamarin, Mauritius.
BACKGROUND: Arthropod-borne pathogens and their vectors are present throughout Africa. They have been well-studied in livestock of sub-Saharan Africa, but poorly in companion animals. Given the socio-economic importance of companion animals, the African Small Companion Animal Network (AFSCAN), as part of the WSAVA Foundation, initiated a standardized multi-country surveillance study.
METHODS: Macro-geographic variation in ectoparasite (ticks and fleas) and pathogen communities in dogs was assessed through molecular screening of approximately 100 infested dogs in each of six countries (Ghana, Kenya, Nigeria, Tanzania, Uganda and Namibia), both in rural and urban settings. The most important intrinsic and extrinsic risk factors within the subpopulation of infested dogs were evaluated.
RESULTS: Despite the large macro-geographic variation in the dogs screened, there was no consistent difference between East and West Africa in terms of the diversity and numbers of ticks. The highest and lowest numbers of ticks were found in Nigeria and Namibia, respectively. Most often, there was a higher diversity of ticks in rural habitats than in urban habitats, although the highest diversity was observed in an urban Uganda setting. With the exception of Namibia, more fleas were collected in rural areas. We identified tick species (including Haemaphysalis spinulosa) as well as zoonotic pathogens (Coxiella burnetti, Trypanosoma spp.) that are not classically associated with companion animals. Rhipicephalus sanguineus was the most abundant tick, with a preference for urban areas. Exophilic ticks, such as Haemaphysalis spp., were more often found in rural areas. Several multi-host ticks occurred in urban areas. For R.

sanguineus, housing conditions and additional pets were relevant factors in terms of infestation, while for a rural tick species (Haemaphysalis elliptica), free-roaming dogs were more often infested. Tick occurrence was associated to the use of endoparasiticide, but not to the use of ectoparasiticide. The most prevalent tick-borne pathogen was Hepatozoon canis followed by Ehrlichia canis. High levels of co-parasitism were observed in all countries and habitats.
CONCLUSIONS: As dogs share a common environment with people, they have the potential to extend the network of pathogen transmission to humans. Our study will help epidemiologists to provide recommendations for surveillance and prevention of pathogens in dogs and humans.

164. Methods Mol Biol. 2022;2411:253-267. doi: 10.1007/978-1-0716-1888-2_15.
Anti-Tick Vaccines: Current Advances and Future Prospects.
Muhanguzi D(1), Ndekezi C(2), Nkamwesiga J(2), Kalayou S(3), Ochwo S(2), Vuyani M(4), Kimuda MP(2).
Author information:
(1)School of Biosecurity, Biotechnical and Laboratory Science, College of Veterinary Medicine Animal Resources and Biosecurity, Makerere University, Kampala, Uganda. dennis.muhanguzi@mak.ac.ug.
(2)School of Biosecurity, Biotechnical and Laboratory Science, College of Veterinary Medicine Animal Resources and Biosecurity, Makerere University, Kampala, Uganda.
(3)International Centre of Insect Physiology and Ecology (ICIPE), Nairobi, Kenya.
(4)Research Unit in Bioinformatics (RUBi), Department of Biochemistry and Microbiology, Rhodes University, Grahamstown, South Africa.
Ticks are increasingly a global public health and veterinary concern. They transmit numerous pathogens that are of veterinary and public health importance. Acaricides, livestock breeding for tick resistance, tick handpicking, pasture spelling, and anti-tick vaccines (ATVs) are in use for the control of ticks and tick-borne diseases (TTBDs); acaricides and ATVs being the most and least used TTBD control methods respectively. The overuse and misuse of acaricides has inadvertently selected for tick strains that are resistant to acaricides. Furthermore, vaccines are rare and not commercially available in sub-Saharan Africa (SSA). It doesn't help that many of the other methods are labor-intensive and found impractical especially for larger farm operations. The success of TTBD control is therefore dependent on integrating all the currently available methods. Vaccines have been shown to be cheap and effective. However, their large-scale deployment for TTBD control in SSA is hindered by commercial unavailability of efficacious anti-tick vaccines against sub-Saharan African tick strains. Thanks to advances in genomics, transcriptomics, and proteomics technologies, many promising anti-tick vaccine antigens (ATVA) have been identified. However, few of them have been investigated for their potential as ATV candidates. Reverse vaccinology (RV) can be leveraged to accelerate ATV discovery. It is cheap and shortens the lead time from ATVA discovery to vaccine production. This chapter provides a brief overview of recent advances in ATV development, ATVs, ATV effector mechanisms, and anti-tick RV. Additionally, it

provides a detailed outline of vaccine antigen selection and analysis using computational methods.

165. PLoS One. 2021 Dec 10;16(12):e0261218. doi: 10.1371/journal.pone.0261218.
Molecular survey of cattle ticks in Burundi: First report on the presence of the invasive Rhipicephalus microplus tick.
Nyabongo L(1)(2)(3), Odongo DO(1), Milton G(4), Machuka E(2), Vudriko P(5), Pelle R(2), Kanduma EG(6).
Author information:
(1)Faculty of Science and Technology, Department of Biology, University of Nairobi (UoN), Nairobi, Kenya.
(2)Biosciences eastern and central Africa-International Livestock Research Institute (BecA-ILRI) Hub, Nairobi, Kenya.
(3)National Veterinary Laboratory of Bujumbura, Bujumbura, Burundi.
(4)Tick Unit, International Livestock Research Institute, Nairobi, Kenya.
(5)College of Veterinary Medicine, Animal Resources and Biosecurity, Makerere University, Kampala, Uganda.
(6)Faculty of Science and Technology, Department of Biochemistry, University of Nairobi, Nairobi, Kenya.
A recent research study on prevalence of tick-borne pathogens in Burundi reported high prevalence and endemicity of Theileria parva, Anaplasma marginale and Babesia bigemina infections in cattle. Detailed information about tick species infesting animals, their distribution and genetic diversity in Burundi is outdated and limited. This study therefore assessed the prevalence and genetic diversity of tick species infesting cattle across agroecological zones (AEZs) in Burundi. A cross-sectional study on the occurrence of tick species was conducted in 24 districts of Burundi between October and December 2017. Differential identification and characterization of ticks collected was conducted using tick morphological keys and molecular tools (cox1 and 12S rRNA gene). Chi-square test was used to test for association between agroecological zones and the prevalence of tick species. Phylogenetic relationships were inferred using bayesian and maximum likelihood algorithms. A total of 483 ticks were collected from the five AEZs sampled. Six tick species comprising of Rhipicephalus appendiculatus, R. sanguineus, R. evertsi evertsi, R. microplus, R. decoloratus and Amblyomma variegatum were observed. Rhipicephalus appendiculatus were the most prevalent ticks (~45%). A total of 138 specimens (28%) were found to be Rhipicephalus microplus, suggesting an emerging threat for cattle farmers. Twelve R. appendiculatus cox1 haplotypes were obtained from 106 specimens that were sequenced. Two cox1 haplotypes of R. microplus which clustered into previously reported Clade A were observed. Rhipicephalus sanguineus and R. evertsi evertsi ticks, the vectors of numerous zoonotic pathogens, were collected from cattle, which constitute a high risk for public health. These findings reveal an overlapping distribution of tick vectors in Burundi. The design of ticks and tick-borne diseases control strategies should consider the distribution of different vectors across the AEZs particularly the presence of the highly invasive R. microplus tick in Burundi and the potential risk of introducing the pathogenic Babesia bovis.

166. Heliyon. 2022 Jan 7;8(1):e08718. doi: 10.1016/j.heliyon.2022.e08718. eCollection
Development of acaricide resistance in tick populations of cattle: A systematic review and meta-analysis.
Dzemo WD(1)(2), Thekisoe O(2), Vudriko P(3).
Author information:
(1)Department of Biological and Environmental Sciences, Faculty of Natural Sciences, Walter Sisulu University, Private Bag X1, Mthatha 5117, South Africa.
(2)Unit for Environmental Sciences and Management, North West University, Potchefstroom Campus, Private Bag X6001, Potchefstroom 2520, South Africa.
(3)Research Center for Tropical Diseases and Vector Control, Department of Veterinary Pharmacy, Clinics and Comparative Medicine, College of Veterinary Medicine, Animal Resources and Biosecurity, Makerere University, P.O Box 7062, Kampala, Uganda.
The development of acaricide resistance in ticks infesting cattle is a major problem in the livestock industry in tropical and subtropical regions worldwide. To determine the current global trends and prevalence of acaricide resistance development (ARD) in tick populations of cattle, a systematic review and meta-analysis with an emphasis on Rhipicephalus (Boophilus) microplus was conducted. Data searches from five English electronic databases yielded 88 journal articles published between 1992 and 2020. In total, 218 in - vitro bioassays were used to investigate 3939 tick populations of cattle; of these, the 57.6% that exhibited ARD were largely limited to South America (Brazil), Central America (Mexico), and Asia (India). A total of 3391 of these tick populations were R. (B.) microplus, of which 2013 exhibited ARD. Random effects meta-analyses indicated that the exhibition of ARD was higher in R. (B.) microplus (66.2%) than in other tick species. Global prevalence estimates of ARD in R. (B.) microplus vary as a function of geography, detection methods, and acaricide compounds. In general, high heterogeneity was noted among the studies. However, homogeneity was observed among studies from India, suggesting the establishment of acaricide resistance in Indian R. (B.) microplus populations. Current tick control interventions are urgently required to limit the evolution and implications of resistance development.

167. Pathogens. 2022 Jan 19;11(2):114. doi: 10.3390/pathogens11020114.
Sequence Diversity of Tp1 and Tp2 Antigens and Population Genetic Analysis of Theileria parva in Unvaccinated Cattle in Zambia's Chongwe and Chisamba Districts.
Muleya W(1), Atuhaire DK(2), Mupila Z(1), Mbao V(3), Mayembe P(4), Kalenga S(4), Fandamu P(4), Namangala B(5), Salt J(6), Musoke AJ(7).
Author information:
(1)Department of Biomedical Sciences, School of Veterinary Medicine, University of Zambia, Lusaka 10101, Zambia.
(2)Centre for Ticks and Tick-Borne Diseases, Lilongwe, Malawi.
(3)Eastern and Southern Africa Regional Office, International Development Research Centre, Nairobi 00200, Kenya.
(4)Department of Veterinary Services, Ministry of Fisheries and Livestock,

Lusaka 50060, Zambia.
(5)Department of Paraclinical Studies, School of Veterinary Medicine, University of Zambia, Lusaka 10101, Zambia.
(6)Global Alliance for Livestock Veterinary Medicines, Pentlands Science Park, Bush Loan, Penicuik EH26 0PZ, UK.
(7)LMK Medical Laboratories and Consultancies, Kampala P.O. Box 33686, Uganda.
East Coast Fever (ECF), caused by Theileria parva, is a major constraint to improved livestock keeping in east and central Africa, including Zambia. To understand the dynamics and determine the candidates for immunization in Zambia's Chongwe and Chisamba districts, a combination of Tp1 and Tp2 gene sequencing and microsatellite analysis using nine markers was conducted from which an abundance of Muguga, Kiambu, Serengeti and Katete epitopes in the field samples was obtained. Phylogenetic analysis showed six (Tp1) and three (Tp2) clusters with an absence of geographical origin clustering. The majority of haplotypes were related to Muguga, Kiambu, Serengeti and Katete, and only a few were related to Chitongo. Both antigens showed purifying selection with an absence of positive selection sites. Furthermore, low to moderate genetic differentiation was observed among and within the populations, and when vaccine stocks were compared with field samples, Chongwe samples showed more similarity to Katete and less to Chitongo, while Chisamba samples showed similarity to both Katete and Chitongo and not to Muguga, Kiambu or Serengeti. We conclude that the use of Katete stock for immunization trials in both Chongwe and Chisamba districts might produce desirable protection against ECF.

168. Viruses. 2022 May 13;14(5):1041. doi: 10.3390/v14051041.
Jingmen Tick Virus in Ticks from Kenya.
Ogola EO(1)(2), Kopp A(3)(4), Bastos ADS(2), Slothouwer I(3)(4), Marklewitz M(3)(4), Omoga D(1), Rotich G(1), Getugi C(1), Sang R(1)(5), Torto B(1)(2), Junglen S(3)(4), Tchouassi DP(1).
Author information:
(1)International Centre of Insect Physiology and Ecology, Nairobi P.O. Box 30772-00100, Kenya.
(2)Department of Zoology and Entomology, University of Pretoria, Private Bag 20, Pretoria 0028, South Africa.
(3)Berlin Institute of Health, Institute of Virology, Charité-Universitätsmedizin Berlin, Corporate Member of Free University Berlin, Humboldt-University Berlin, Chariteplatz 1, 10117 Berlin, Germany.
(4)German Centre for Infection Research (DZIF), Associated Partner Site Charité, 10117 Berlin, Germany.
(5)Kenya Medical Research Institute (KEMRI), Off Raila Odinga Way, Nairobi P.O. Box 54840-00200, Kenya.
Jingmen tick virus (JMTV) is an arbovirus with a multisegmented genome related to those of unsegmented flaviviruses. The virus first described in Rhipicephalus microplus ticks collected in Jingmen city (Hubei Province, China) in 2010 is associated with febrile illness in humans. Since then, the geographic range has expanded to include Trinidad and Tobago, Brazil, and Uganda. However, the ecology of JMTV remains poorly described in Africa. We screened adult ticks (n =

4550, 718 pools) for JMTV infection by reverse transcription polymerase chain reaction (RT-PCR). Ticks were collected from cattle (n = 859, 18.88%), goats (n = 2070, 45.49%), sheep (n = 1574, 34.59%), and free-ranging tortoises (Leopard tortoise, Stigmochelys pardalis) (n = 47, 1.03%) in two Kenyan pastoralist-dominated areas (Baringo and Kajiado counties) with a history of undiagnosed febrile human illness. Surprisingly, ticks collected from goats (0.3%, 95% confidence interval (CI) 0.1-0.5), sheep (1.8%, 95% CI 1.2-2.5), and tortoise (74.5%, 95% CI 60.9-85.4, were found infected with JMTV, but ticks collected from cattle were all negative. JMTV ribonucleic acid (RNA) was also detected in blood from tortoises (66.7%, 95% CI 16.1-97.7). Intragenetic distance of JMTV sequences originating from tortoise-associated ticks was greater than that of sheep-associated ticks. Phylogenetic analyses of seven complete-coding genome sequences generated from tortoise-associated ticks formed a monophyletic clade within JMTV strains from other countries. In summary, our findings confirm the circulation of JMTV in ticks in Kenya. Further epidemiological surveys are needed to assess the potential public health impact of JMTV in Kenya.

169. Curr Res Parasitol Vector Borne Dis. 2022 May 14;2:100090. doi: 10.1016/j.crpvbd.2022.100090. eCollection 2022.
Acaricide resistance in livestock ticks infesting cattle in Africa: Current status and potential mitigation strategies.
Githaka NW(1), Kanduma EG(2), Wieland B(1), Darghouth MA(3), Bishop RP(4).
Author information:
(1)International Livestock Research Institute (ILRI), P.O. Box 30709-00100, Nairobi, Kenya.
(2)Department of Biochemistry, Faculty of Science and Technology, University of Nairobi, P.O. Box 30197-00100, Nairobi, Kenya.
(3)Laboratoire de Parasitologie, Ecole Nationale de Médecine Vétérinaire, Institution de la Recherche et de l'Enseignement Supérieur Agricoles and La Manouba University, 2020, Sidi Thabet, Tunisia.
(4)Department of Veterinary Microbiology and Pathology, Washington State University, Pullman, WA, USA.
In many African countries, tick control has recently been the responsibility of resource-poor farmers rather than central government veterinary departments. This has led to an increase in acaricide resistance, threatening the welfare of livestock farmers in sub-Saharan Africa. Resistance has evolved to the three classes of acaricides used most extensively in the continent, namely fourth-generation synthetic pyrethroids (SP), organophosphates (OP) and amidines (AM), in virtually all countries in which they have been deployed across the globe. Most current data are derived from research in Australia and Latin America, with the majority of studies on acaricide resistance in Africa performed in South Africa. There is also limited recent research from West Africa and Uganda. These studies confirm that acaricide resistance in cattle ticks is a major problem in Africa. Resistance is most frequently directly assayed in ticks using the larval packet test (LPT) that is endorsed by FAO, but such tests require a specialist tick-rearing laboratory and are relatively time

consuming. To date they have only been used on a limited scale in Africa and resistance is often still inferred from tick numbers on animals. Rapid tests for resistance in ticks, would be better than the LPT and are theoretically possible to develop. However, these are not yet available. Resistance can be mitigated through integrated control strategies, comprising a combination of methods, including acaricide class rotation or co-formulations, ethnoveterinary practices, vaccination against ticks and modified land management use by cattle, with the goal of minimising the number of acaricide applications required per year. There are data suggesting that small-scale farmers in Africa are often unaware of the chemical differences between different acaricide brands and use these products at concentrations other than those recommended by the manufacturers, or in incorrect rotations or combinations of the different classes of chemicals on the market. There is an urgent need for a more evidence-based approach to acaricide usage in small-scale livestock systems in Africa, including direct measurements of resistance levels, combined with better education of farmers regarding acaricide products and how they should be deployed for control of livestock ticks.

170. Vaccine. 2022 Jul 30;40(32):4564-4573. doi: 10.1016/j.vaccine.2022.06.036. Epub
Oral vaccine formulation combining tick Subolesin with heat inactivated mycobacteria provides control of cross-species cattle tick infestations.
Kasaija PD(1), Contreras M(2), Kabi F(3), Mugerwa S(3), Garrido JM(4), Gortazar C(2), de la Fuente J(5).
Author information:
(1)SaBio, Instituto de Investigación en Recursos Cinegéticos IREC-CSIC-UCLM-JCCM, Ronda de Toledo s/n, 13005 Ciudad Real, Spain; National Livestock Resources Research Institute (NaLIRRI/NARO), P.O. Box 5704, Wakiso District, Uganda.
(2)SaBio, Instituto de Investigación en Recursos Cinegéticos IREC-CSIC-UCLM-JCCM, Ronda de Toledo s/n, 13005 Ciudad Real, Spain.
(3)National Livestock Resources Research Institute (NaLIRRI/NARO), P.O. Box 5704, Wakiso District, Uganda.
(4)NEIKER-Instituto Vasco de Investigación y Desarrollo Agrario, Animal Health Department, Bizkaia Science and Technology Park 812L, 48160 Derio (Bizkaia), Spain.
(5)SaBio, Instituto de Investigación en Recursos Cinegéticos IREC-CSIC-UCLM-JCCM, Ronda de Toledo s/n, 13005 Ciudad Real, Spain; Department of Veterinary Pathobiology, Center for Veterinary Health Sciences, Oklahoma State University, Stillwater, OK 74078, USA. Electronic address: jose_delafuente@yahoo.com.
Tick vaccines are necessary as part of a One Health approach for the control of tick infestations and tick-borne diseases. Subolesin (SUB, also known as 4D8) is a tick protective antigen that has shown efficacy in vaccine formulations for the control of ectoparasite infestations and pathogen infection/transmission. A recent proof-of-concept study reported oral vaccination combining Rhipicephalus microplus SUB with heat inactivated Mycobacterium bovis (IV) as an immunostimulant for the control of cattle tick infestations. Based on the

efficacy of Rhipicephalus decoloratus SUB for the control of multiple cattle tick species in Uganda, herein we design a controlled pen trial using an oral formulation combining R. decoloratus SUB with IV for the control of R. decoloratus and Rhipicephalus appendiculatus cattle tick infestations. Vaccine efficacy (E) of SUB + IV on tick life cycle was compared with IV and SUB alone and with PBS as control. The IgG antibody titers against SUB and M. bovis P22 and the serum levels of selected protein immune biomarkers (IL-1beta, TNF-alpha, C3) were determined and analyzed as possible correlates of protection. Oral immunization with IV and SUB alone and in SUB + IV combination were effective for the control of tick infestations (E = 71-96% for R. decoloratus and 87-99% for R. appendiculatus) with highest E (higher than 95%) for SUB + IV. The results demonstrated that oral immunization with the SUB + IV formulation resulted in effective control of cattle tick infestations through the activation of multiple immune mechanisms. These results support the application of oral vaccine formulations with SUB + IV for the control of cattle infestations with Rhipicephalus species towards improving animal health.

171. Front Vet Sci. 2022 Jun 13;9:863526. doi: 10.3389/fvets.2022.863526. eCollection
Awareness, Knowledge, and Perceptions Regarding Rabies Prevention Among Rural Communities in Masaka District, Central Uganda: A Qualitative Study.
Kankya C(1), Dürr S(2), Hartnack S(3), Warembourg C(2), Okello J(1), Muleme J(1)(4), Okello W(1), Methodius T(1), Alobo G(1), Odoch T(1).
Author information:
(1)Department of Biosecurity, Ecosystems and Veterinary Public Health, School of Biosecurity, Biotechnical and Laboratory Sciences, College of Veterinary Medicine, Animal Resources and Biosecurity, Makerere University, Kampala, Uganda.
(2)Veterinary Public Health Institute, Vetsuisse Faculty, University of Bern, Bern, Switzerland.
(3)Section of Epidemiology, Vetsuisse Faculty, University of Zurich, Zurich, Switzerland.
(4)Department of Disease Control and Environmental Health, School of Public Health, College of Health Sciences, Makerere University, Kampala, Uganda.
Rabies is a zoonotic disease that is mainly transmitted to humans through dog bites. It remains a major public health threat in many Asian and African countries, including Uganda. The main objective of this study was to investigate awareness, knowledge, and perceptions of communities toward human and dog health related to rabies prevention, as well as dog management practices within Masaka district, central Uganda. Data collection involved nine key informant interviews (KIIs) and six focus group discussions (FGDs). Methods used during focus group discussions included qualitative interviews (using open-ended questions), simple ranking, and proportional piling. Data from KIIs and FGDs were analyzed using content analysis in NVivo (version 12.0). This study reveals that community members in the rural settings uses herbal concoctions in replacement or as an alternative to dog vaccination. Furthermore, the study reveals that dogs play the vital roles in the households like as they offer protection to people and household properties, despite being ranked second least among the household

animals. The commonest livelihood activity was a small-scale mixed farming. Most of the households kept dogs, but they are ranked at second lowest in terms of economic value among all domestic animals. Free roaming and tethering were the common dog-keeping systems, and home-based feed (food leftovers, bones) was provided mainly to the tethered dogs. Rabies, also locally known as "Eddalu Lyembwa" (that can be translated as "madness of the dogs"), was ranked as the disease of most important among dogs, besides other common diseases such as skin diseases, venereal diseases, worm infestations, and tick infestations.
Inadequate vaccination services for both humans and dogs were reported, and dog bite victims traveled a long distance to seek for post-exposure prophylaxis after dog bites. It can be concluded that there is a clear request for periodic mass vaccination campaigns against rabies among dogs, and access to vaccines within reasonable distances by humans after a rabies exposure, but also pre-emptive vaccination for those at high risk, such as veterinarians, needs to be improved.

172. Animals (Basel). 2022 Jul 1;12(13):1708. doi: 10.3390/ani12131708.
Molecular Survey and Genetic Characterization of Anaplasma marginale in Ticks Collected from Livestock Hosts in Pakistan.
Khan Z(1), Shehla S(1), Alouffi A(2), Kashif Obaid M(1), Zeb Khan A(3), Almutairi MM(4), Numan M(1), Aiman O(1), Alam S(1), Ullah S(1), Zaman Safi S(5)(6), Tanaka T(7), Ali A(1).
Author information:
(1)Department of Zoology, Abdul Wali Khan University Mardan, Mardan 23200, Pakistan.
(2)King Abdulaziz City for Science and Technology, Riyadh 12354, Saudi Arabia.
(3)Department of Pediatrics, Yale School of Medicine, Yale University, New Haven, CT 06510, USA.
(4)Department of Pharmacology and Toxicology, College of Pharmacy, King Saud University, Riyadh 11451, Saudi Arabia.
(5)Faculty of Medicine, Bioscience and Nursing, MAHSA University, Jenjarom 42610, Selangor, Malaysia.
(6)Interdisciplinary Research Centre in Biomedical Materials, Lahore Campus, COMSATS University Islamabad, Lahore 54000, Pakistan.
(7)Laboratory of Infectious Diseases, Joint Faculty of Veterinary Medicine, Kagoshima University, 1-21-24 Korimoto, Kagoshima 890-0065, Japan.
Ticks transmit pathogens to animals and humans more often than any other arthropod vector. The rural economy of Pakistan mainly depends on livestock farming, and tick infestations cause severe problems in this sector. The present study aimed to molecularly characterize the Anaplasma spp. in hard ticks collected from six districts of Khyber Pakhtunkhwa, Pakistan. Ticks were collected from various livestock hosts, including cattle breeds (Holstein-Friesian, Jersey, Sahiwal, and Achai), Asian water buffaloes, sheep, and goats from March 2018 to February 2019. Collected ticks were morphologically identified and subjected to molecular screening of Anaplasma spp. by amplifying 16S rDNA sequences. Six hundred seventy-six ticks were collected from infested hosts (224/350, 64%). Among the nine morphologically identified tick species,

the highest occurrence was noted for Rhipicephalus microplus (254, 37.6%), followed by Hyalomma anatolicum (136, 20.1%), Rhipicephalus haemaphysaloides (119, 17.6%), Rhipicephalus turanicus (116, 17.1%), Haemaphysalis montgomeryi (14, 2.1%), Hyalomma dromedarii (11, 1.6%), Haemaphysalis bispinosa (10, 1.5%), Hyalomma scupense (8, 1.2%), and Haemaphysalis kashmirensis (8, 1.2%). The occurrence of tick females was highest (260, 38.5%), followed by nymphs (246, 36.4%) and males (170, 25.1%). Overall, the highest occurrence of ticks was recorded in the Peshawar district (239, 35.3%), followed by Mardan (183, 27.1%), Charsadda (110, 16.3%), Swat (52, 7.7%), Shangla (48, 7.1%), and Chitral (44, 6.5%). Among these ticks, Anaplasma marginale was detected in R. microplus, R. turanicus, and R. haemaphysaloides. The 16S rDNA sequences showed high identity (98-100%) with A. marginale reported from Australia, China, Japan, Pakistan, Thailand, Uganda, and the USA. In phylogenetic analysis, the sequence of A. marginale clustered with the same species reported from Australia, China, Pakistan, Thailand, Uruguay, and the USA. Further molecular work regarding the diversity of tick species and associated pathogens is essential across the country.

173. Vaccines (Basel). 2022 Aug 16;10(8):1327. doi: 10.3390/vaccines10081327.
The Correlation between Subolesin-Reactive Epitopes and Vaccine Efficacy.
Contreras M(1), Kasaija PD(1)(2), Kabi F(2), Mugerwa S(2), De la Fuente J(1)(3).
Author information:
(1)SaBio, Instituto de Investigación en Recursos Cinegéticos (IREC), Consejo Superior de Investigaciones Científicas (CSIC), Universidad de Castilla-La Mancha (UCLM)-Junta de Comunidades de Castilla-La Mancha (JCCM), Ronda de Toledo s/n, 13005 Ciudad Real, Spain.
(2)National Livestock Resources Research Institute (NaLIRRI/NARO), Kampala P.O. Box 5704, Uganda.
(3)Center for Veterinary Health Sciences, Department of Veterinary Pathobiology, Oklahoma State University, Stillwater, OK 74078, USA.
Vaccination is an environmentally-friendly alternative for tick control. The tick antigen Subolesin (SUB) has shown protection in vaccines for the control of multiple tick species in cattle. Additionally, recent approaches in quantum vaccinomics have predicted SUB-protective epitopes and the peptide sequences involved in protein−protein interactions in this tick antigen. Therefore, the identification of B-cell−reactive epitopes by epitope mapping using a SUB peptide array could be essential as a novel strategy for vaccine development. Subolesin can be used as a model to evaluate the effectiveness of these approaches for the identification of protective epitopes related to vaccine protection and efficacy. In this study, the mapping of B-cell linear epitopes of SUB from three different tick species common in Uganda (Rhipicephalus appendiculatus, R. decoloratus, and Amblyomma variegatum) was conducted using serum samples from two cattle breeds immunized with SUB-based vaccines. The results showed that in cattle immunized with SUB from R. appendiculatus (SUBra) all the reactive peptides (Z-score > 2) recognized by IgG were also significant (Z-ratio > 1.96) when compared to the control group. Additionally, some of the reactive peptides recognized by IgG from the control group were also recognized

in SUB cocktail–immunized groups. As a significant result, cattle groups that showed the highest vaccine efficacy were Bos indicus immunized with a SUB cocktail (92%), and crossbred cattle were immunized with SUBra (90%) against R. appendiculatus ticks; the IgG from these groups recognized overlapping epitopes from the peptide
SPTGLSPGLSPVRDQPLFTFRQVGLICERMMKERESQIRDEYDHVLSAKLAEQYDTFVKFTYDQKRFEGAT
PSYLS
(Z-ratio > 1.96), which partially corresponded to a Q38 peptide and the SUB protein interaction domain. These identified epitopes could be related to the protection and efficacy of the SUB-based vaccines, and new chimeras containing these protective epitopes could be designed using this new approach.

174. Parasit Vectors. 2022 Sep 9;15(1):321. doi: 10.1186/s13071-022-05436-y.
A community approach for pathogens and their arthropod vectors (ticks and fleas) in cats of sub-Saharan Africa.
Madder M(1), Day M(2), Schunack B(3), Fourie J(4)(5), Labuschange M(6), van der Westhuizen W(6), Johnson S(7), Githigia SM(8), Akande FA(9), Nzalawahe JS(10), Tayebwa DS(11), Aschenborn O(12), Marcondes M(13), Heylen D(14)(15)(16).
Author information:
(1)Clinglobal, Tamarin, Mauritius. maxime.madder@clinglobal.com.
(2)School of Veterinary and Life Sciences, Murdoch University, Murdoch, WA, Australia.
(3)Elanco Animal Health, Leverkusen, Germany.
(4)Clinvet, Waverly, USA.
(5)Clinvet, Bloemfontein, South Africa.
(6)Clinomics, Bloemfontein, South Africa.
(7)School of Veterinary Medicine, CBAS, University of Ghana, Accra, Ghana.
(8)Department of Veterinary Pathology, Microbiology & Parasitology, University of Nairobi, Nairobi, Kenya.
(9)Department of Veterinary Parasitology and Entomology, College of Veterinary Medicine, Federal University of Agriculture, Abeokuta, Nigeria.
(10)Sokoine University of Agriculture, Morogoro, Tanzania.
(11)Research Center for Tropical Diseases and Vector Control, College of Veterinary Medicine, Animal Resources and Biosecurity, Makerere University, Kampala, Uganda.
(12)School of Veterinary Medicine, University of Namibia, Ondekaremba, Namibia.
(13)São Paulo State University, São Paulo, Brazil.
(14)Eco-Epidemiology Group, Department of Biomedical Sciences, Institute of Tropical Medicine, Antwerp, Belgium.
(15)Interuniversity Institute for Biostatistics and Statistical Bioinformatics, Hasselt University, Diepenbeek, Belgium.
(16)Department of Ecology and Evolutionary Biology, Princeton University, Princeton, NJ, USA.
BACKGROUND: Arthropod-borne pathogens and their vectors are present throughout Africa. They have been well studied in livestock of sub-Saharan Africa, but poorly studied in companion animals. Given their socioeconomic importance, the African Small Companion Animal Network (AFSCAN), as part of the WSAVA

Foundation, initiated a standardized multi-country surveillance study.
METHODS: In six countries (Ghana, Kenya, Nigeria, Tanzania, Uganda, and Namibia) in both rural and urban settings, 160 infested cats were sampled to assess their ectoparasite community (ticks and fleas), as well as the micro-parasite prevalence within those ectoparasites (60 and 118 pools of ticks and fleas, respectively) and blood (276 cats, including 116 non-infested).
RESULTS: Almost two thirds of all infested cats originated from Tanzania and Kenya. Despite the large macro-geographical variation, no consistent difference was found in ectoparasite diversity and numbers between East and West Africa. Far more flea-infested than tick-infested cats were found. The most dominant ectoparasite was Ctenocephalides felis. Among the ticks, the exophilic Haemaphysalis spp. were the commonest, including species that are not typically linked with companion animals (Haemaphysalis spinulosa and Haemaphysalis elliptica). The most prevalent pathogens found in the blood and fleas were Bartonella henselae and Mycoplasma haemofelis. In the ticks, the dog-associated Hepatozoon canis was most commonly found. A high degree of co-parasitism was found in all countries and habitats.
CONCLUSIONS: Our continent-wide standardized field study highlights the cat's potential to serve as a reservoir of pathogens that can be transmitted to humans or livestock, especially when cats are expected to become more commonly kept in African villages and towns.

175. J Infect. 2022 Dec;85(6):693-701. doi: 10.1016/j.jinf.2022.09.007. Epub 2022 Sep
Risk factors for Crimean-Congo Haemorrhagic Fever (CCHF) virus exposure in farming communities in Uganda.
Atim SA(1), Ashraf S(2), Belij-Rammerstorfer S(3), Ademun AR(4), Vudriko P(5), Nakayiki T(6), Niebel M(2), Shepherd J(2), Balinandi S(6), Nakanjako G(7), Abaasa A(8), Johnson PCD(9), Odongo S(5), Esau M(4), Bahati M(4), Kaleebu P(10), Lutwama JJ(6), Masembe C(11), Lambe T(3), Thomson EC(12), Tweyongyere R(13).
Author information:
(1)College of Veterinary Medicine, Animal Resources and Biosecurity (CoVAB), Makerere University, Kampala, Uganda; MRC-University of Glasgow Centre for Virus Research (CVR), Glasgow, United Kingdom; Ministry of Agriculture, Animal Industry and Fisheries, Entebbe, Uganda.
(2)MRC-University of Glasgow Centre for Virus Research (CVR), Glasgow, United Kingdom.
(3)The Jenner Institute, Nuffield Department of Medicine, University of Oxford, Oxford, United Kingdom.
(4)Ministry of Agriculture, Animal Industry and Fisheries, Entebbe, Uganda.
(5)College of Veterinary Medicine, Animal Resources and Biosecurity (CoVAB), Makerere University, Kampala, Uganda.
(6)Department of Arbovirology, Emerging and Re-emerging Infections, Uganda Virus Research Institute, Entebbe, Uganda.
(7)Ministry of Agriculture, Animal Industry and Fisheries, Entebbe, Uganda; Department of Arbovirology, Emerging and Re-emerging Infections, Uganda Virus Research Institute, Entebbe, Uganda.
(8)MRC/UVRI and LSHTM Uganda Research Unit, Entebbe Uganda.

(9)Institute of Biodiversity Animal Health and Comparative Medicine, University of Glasgow, Glasgow, United Kingdom.
(10)Department of Arbovirology, Emerging and Re-emerging Infections, Uganda Virus Research Institute, Entebbe, Uganda; MRC/UVRI and LSHTM Uganda Research Unit, Entebbe Uganda.
(11)College of Natural Resources (CoNAS), Makerere University, Kampala, Uganda.
(12)MRC-University of Glasgow Centre for Virus Research (CVR), Glasgow, United Kingdom; London School of Hygiene and Tropical Medicine, London, UK.
(13)College of Veterinary Medicine, Animal Resources and Biosecurity (CoVAB), Makerere University, Kampala, Uganda. Electronic address: robert.tweyongyere@mak.ac.ug.
BACKGROUND: Crimean-Congo Haemorrhagic Fever (CCHF) is an emerging human-health threat causing sporadic outbreaks in livestock farming communities. However, the full extent and the risks associated with exposure of such communities has not previously been well-described.
METHODS: We collected blood samples from 800 humans, 666 cattle, 549 goats and 32 dogs in districts within and outside Ugandan cattle corridor in a cross-sectional survey, and tested for CCHFV-specific IgG antibodies using Enzyme-Linked Immunosorbent Assays. Sociodemographic and epidemiological data were recorded using structured questionnaire. Ticks were collected to identify circulating nairoviruses by metagenomic sequencing.
RESULTS: CCHFV seropositivity was in 221/800 (27·6%) in humans, 612/666 (91·8%) in cattle, 413/549 (75·2%) in goats and 18/32 (56·2%) in dogs. Human seropositivity was associated with livestock farming (AOR=5·68, p<0·0001), age (AOR=2·99, p=0·002) and collecting/eating engorged ticks (AOR=2·13, p=0·004). In animals, seropositivity was higher in cattle versus goats (AOR=2·58, p<0·0001), female sex (AOR=2·13, p=0·002) and heavy tick infestation (>50 ticks: AOR=3·52, p=0·004). CCHFV was identified in multiple tick pools of Rhipicephalus appendiculatus.
INTERPRETATION: The very high CCHF seropositivity especially among livestock farmers and multiple regional risk factors associated exposures, including collecting/eating engorged ticks previously unrecognised, highlights need for further surveillance and sensitisation and control policies against the disease.

176. J Infect. 2022 Dec;85(6):683-692. doi: 10.1016/j.jinf.2022.09.016. Epub 2022 Sep
Widespread exposure to Crimean-Congo haemorrhagic fever in Uganda might be driven by transmission from Rhipicephalus ticks: Evidence from cross-sectional and modelling studies.
Lule SA(1), Gibb R(2), Kizito D(3), Nakanjako G(3), Mutyaba J(3), Balinandi S(3), Owen L(1), Jones KE(2), Abubakar I(1), Lutwama JJ(3), Field N(4).
Author information:
(1)Institute for Global Health (IGH), University College London, London, UK.
(2)Centre for Biodiversity and Environment Research, Department of Genetics, Evolution and Environment, University College London, London, UK.
(3)Department of Arbovirology, Emerging and Re-Emerging Infectious Diseases (DAERID), Uganda Virus Research Institute (UVRI), Entebbe, Uganda.
(4)Institute for Global Health (IGH), University College London, London, UK.

Electronic address: nigel.field@ucl.ac.uk.
BACKGROUND: Crimean-Congo haemorrhagic fever (CCHF) is a widespread tick-borne viral infection, present across Africa and Eurasia, which might pose a cryptic public health problem in Uganda. We aimed to understand the magnitude and distribution of CCHF risk in humans, livestock and ticks across Uganda by synthesising epidemiological (cross-sectional) and ecological (modelling) studies.
METHODS: We conducted a cross-sectional study at three urban abattoirs receiving cattle from across Uganda. We sampled humans (n = 478), livestock (n = 419) and ticks (n = 1065) and used commercially-available kits to detect human and livestock CCHF virus (CCHFV) antibodies and antigen in tick pools. We developed boosted regression tree models to evaluate the correlates and geographical distribution of expected tick and wildlife hosts, and of human CCHF exposures, drawing on continent-wide data.
FINDINGS: The cross-sectional study found CCHFV IgG/IgM seroprevalence in humans of 10·3% (7·8-13·3), with antibody detection positively associated with reported history of tick bite (age-adjusted odds ratio = 2·09 (1·09-3·98)). Cattle had a seroprevalence of 69·7% (65·1-73·4). Only one Hyalomma tick (CCHFV-negative) was found. However, CCHFV antigen was detected in Rhipicephalus (5·9% of 304 pools) and Amblyomma (2·9% of 34 pools) species. Modelling predicted high human CCHF risk across much of Uganda, low environmental suitability for Hyalomma, and high suitability for Rhipicephalus and Amblyomma.
INTERPRETATION: Our epidemiological and ecological studies provide complementary evidence that CCHF exposure risk is widespread across Uganda. We challenge the idea that Hyalomma ticks are consistently the principal reservoir and vector for CCHFV, and postulate that Rhipicephalus might be important for CCHFV transmission in Uganda, due to high frequency of infected ticks and predicted environmental suitability.
FUNDING: UCL Global Challenges Research Fund (GCRF) and Pan-African Network on Emerging and Re-Emerging Infections (PANDORA-ID-NET) funded by the European and Developing Countries Clinical Trials Partnership (EDCTP) under the EU Horizon 2020 Framework Programme for Research and Innovation.

177. Emerg Microbes Infect. 2022 Dec;11(1):2711-2714. doi: 10.1080/22221751.2022.2136537.
Multiple genotypes of Crimean-Congo hemorrhagic fever virus detected in ticks during a one health survey in Agnam, Northeastern Senegal.
Mhamadi M(1)(2), Badji A(3), Dieng I(1), Gaye A(3), Ndiaye EH(3), Ndiaye M(1), Mhamadi M(4), Toure CT(1), Barry A(5), Ndiaye O(1)(4), Faye B(2), Ba FA(6), Diop B(7), Ndiaye M(7), Sagne SN(5), Fall G(1), Loucoubar C(5), Bovendo HF(8), Sall AA(1), Kobinger G(9), Faye O(1), Diallo M(3), Faye O(1).
Author information:
(1)Virology Department, Institut Pasteur de Dakar, Dakar, Senegal.
(2)Parasitology Department, Université Cheikh Anta Diop de Dakar, Dakar, Senegal.
(3)Medical Zoology Department, Institut Pasteur de Dakar, Dakar, Senegal.
(4)Institut Pasteur de Dakar, DIATROPIX, Dakar, Senegal.

(5)Epidemiology, Clinical Research and Data Science Department, Institut Pasteur
de Dakar, Dakar, Senegal.
(6)Microbiology, Immunology and Infectious Pathology Service, Department of
Public Health and Environment, EISMV of Dakar, Dakar, Senegal.
(7)Ministry of Health and Social Action, Dakar, Senegal.
(8)Global Urgent and Advanced Research and Development, Batiscan, QC, Canada.
(9)University of Texas Medical Branch, Galveston, TX, USA.
A Crimean-Congo Hemorrhagic Fever Virus (CCHFV) survey in Agnam (North Senegal)
permits the detection of three isolates in ticks. These isolates belong
genetically to multiple genotypes (I, II, III) and clustered with strains from
Uganda, Sudan, Mauritania, and Senegal. The role of ticks in CCHF emergence and
widespread is highlighted.

178. Vaccines (Basel). 2022 Sep 22;10(10):1594. doi: 10.3390/vaccines10101594.
Monitoring the Subolesin Vaccine Field Trial for Safer Control of Cattle Ticks
Amidst Increasing Acaricide Resistance in Uganda.
Kabi F(1), Dhikusooka M(1), Matovu M(1), Mugerwa S(1), Kasaija P(1)(2), Emudong
P(1), Kirunda H(3), Contreras M(2), Gortazar C(2), De la Fuente J(2)(4).
Author information:
(1)National Livestock Resources Research Institute (NaLIRRI/NARO), Kampala P.O.
Box 5704, Uganda.
(2)SaBio, Instituto de Investigación en Recursos Cinegéticos (IREC), Consejo
Superior de Investigaciones Cientí ficas (CSIC), Universidad de Castilla-La
Mancha (UCLM)-Junta de Comunidades de Castilla-La Mancha (JCCM), Ronda de Toledo
12, 13005 Ciudad Real, Spain.
(3)Mbarara Zonal Agricultural Research and Development Institute (MbaZARDI),
Mbarara City P.O. Box 389, Uganda.
(4)Department of Veterinary Pathobiology, Center for Veterinary Health Sciences,
Oklahoma State University, Stillwater, OK 74078, USA.
A collaboration program was established between the group of Health and
Biotechnology (SaBio) of the IREC Institute of Game and Wildlife Research
(CSIC-UCLM-JCCM, Spain) and the National Agricultural Research Organization of
Uganda (NARO) for the development of vaccines for the control of cattle ticks in
Uganda. Controlled pen trials identified a tick protective antigen,
Rhipicephalus appendiculatus Subolesin, and a cross-species-effective vaccine
formulation. As the next step, a controlled vaccine field trial has been
approved by Ugandan state regulatory authorities, the Uganda National Council
for Science and Technology (UNCST) and the National Drug Authority (NDA), to
evaluate the efficacy and effectiveness of the vaccine formulation for the
control of cattle tick infestations under field conditions. The results of this
trial may lead to the approval of the vaccine for application in Uganda to
improve cattle health and production while reducing the use of acaricides.

179. J Med Entomol. 2023 Jan 12;60(1):185-192. doi: 10.1093/jme/tjac166.
Minimal Rickettsial Infection Rates and Distribution of Ticks in Uganda: An
Assessment of the Seasonal Effects and Relevance to Tick-Borne Disease Risk in
East Africa.

Corrigan J(1)(2), Marion B(1)(2), English J(1), Eneku W(3), Weng JL(1), Rugg M(1), Dotrang T(1), Dunford J(1), Byaruhanga AM(3), Byarugaba DK(3), Ramalho-Ortigao M(1), Wanja EW(1).
Author information:
(1)Division of Global Public Health, Department of Preventive Medicine and Biostatistics, Uniformed Services University, 4301 Jones Bridge Road, Bethesda, MD 20814, USA.
(2)MPH Program, Department of Preventive Medicine and Biostatistics, Uniformed Services University, 4301 Jones Bridge Road, Bethesda, MD 20814, USA.
(3)Makerere University, Kampala, Uganda.
Tick-borne diseases (TBDs) pose a significant risk to humans and represent one of the major factors influencing readiness within the United States' military worldwide. Additionally, ticks and TBDs constitute major animal health problems leading to economic losses at multiple levels affecting low- and middle-income countries the hardest. Tick control is frequently hampered by issues ranging from acaricide resistance to lack of data on tick distribution and infection rates. We conducted a cross-sectional study to assess tick species distribution, host use, and rickettsial pathogen infection rate of ticks in different areas of the Uganda Cattle Corridor. We identified 4,425 hard ticks (Ixodida: Ixodidae) comprised of seven species by morphological characters with 3,315 ticks collected from four locations during the dry season and 1,110 ticks from one location during the wet season. Rickettsial pathogen prevalence was assessed in ticks collected from two districts to determine the minimum infection rate compared across seasons, village location, and tick species. We found statistically significant differences in the abundance and distribution of tick species among districts in the dry season, host animal species, and the proportion of rickettsial positive pools between villages. Seasonality, village location, and tick species do not affect the minimum infection rate of rickettsial pathogens of ticks in Uganda, but village location affects the proportion of positive tick pools. These results indicate geographical and seasonal differences among pathogen-harboring ticks contributing to our understanding of the current distribution of ticks and TBDs in Uganda.

180. Ticks Tick Borne Dis. 2023 Mar;14(2):102084. doi: 10.1016/j.ttbdis.2022.102084.
Genetic diversity in Babesia bovis from southern Africa and estimation of B. bovis infection levels in cattle using an optimised quantitative PCR assay.
Byaruhanga C(1), Makgabo SM(2), Choopa CN(3), Mulandane FC(2), Vorster I(2), Troskie M(2), Chaisi ME(4), Collins NE(2).
Author information:
(1)Vectors and Vector-Borne Diseases Research Programme, Department of Veterinary Tropical Diseases, Faculty of Veterinary Science, University of Pretoria, Private Bag X04, Onderstepoort 0110, South Africa; National Agricultural Research Organisation, P.O. Box 259, Entebbe, Uganda. Electronic address: cbyaruhanga27@yahoo.com.
(2)Vectors and Vector-Borne Diseases Research Programme, Department of Veterinary Tropical Diseases, Faculty of Veterinary Science, University of Pretoria, Private Bag X04, Onderstepoort 0110, South Africa.

(3)Vectors and Vector-Borne Diseases Research Programme, Department of Veterinary Tropical Diseases, Faculty of Veterinary Science, University of Pretoria, Private Bag X04, Onderstepoort 0110, South Africa; Central Veterinary Research Institute, Ministry of Fisheries and Livestock, P.O Box 33980, Lusaka, Zambia.
(4)Vectors and Vector-Borne Diseases Research Programme, Department of Veterinary Tropical Diseases, Faculty of Veterinary Science, University of Pretoria, Private Bag X04, Onderstepoort 0110, South Africa; Foundational Biodiversity Science, South African National Biodiversity Institute, 232 Boom Street, Pretoria 0001, South Africa.
Babesia bovis is a causal agent of bovine babesiosis, a disease which leads to mortality and morbidity and impacts the cattle industry worldwide. We amplified, cloned and sequenced the B. bovis merozoite surface antigen-2b (msa-2b) gene (~940 bp) and the near full-length 18S rRNA gene (~1600 bp) from cattle samples from South Africa and Mozambique to determine sequence variation between B. bovis parasites in the region. A TaqMan quantitative real-time PCR (qPCR) assay (18S rRNA gene) was optimised for the detection of B. bovis and estimation of parasitaemia in field samples from cattle from southern Africa. Phylogenetic analysis grouped the Msa-2b sequences in six clades and these were 59.7 to 99.6% identical to reference sequences. Sequence variation amongst B. bovis 18S rRNA sequences was found at 2 to 36 positions, and the sequences were 97 to 99% identical to published sequences. Mismatches between the B. bovis 18S rRNA sequences and a previously published qPCR forward primer (BoF) were observed; therefore, we developed a new forward primer (BoF2), and optimised the qPCR assay. Six 10-fold dilution series of B. bovis infected erythrocytes (2 × 108 to 2 × 103 infected red blood cells [iRBC]/ml) were analysed in triplicate in each of six separate qPCR runs, to determine the efficiency of the assay. The qPCR assay amplified the B. bovis 18S rRNA gene with 92.0 to 94.9% efficiency. The detection limit of the qPCR assay was approximately 6 iRBCs/µl. The performance of the optimised assay to diagnose B. bovis in field samples was assessed by testing DNA from 222 field samples of cattle from South Africa and Mozambique using three methods: the optimised qPCR assay, the reverse line blot (RLB) hybridisation assay, and the previously published qPCR assay. The detection rate of B. bovis using the optimised qPCR assay (31.1%, 69/222) was significantly higher ($p<0.001$) than both that using RLB (20.7%, 46/222) and the previously published qPCR assay (5.4%; 12/222). The B. bovis parasitaemia in samples from infected cattle ranged from 6 iRBCs/µl to 101,852 iRBCs/µl of blood. Our study revealed marked sequence variation between B. bovis parasites from southern Africa. The optimised qPCR assay will be useful in epidemiological studies and clinical diagnosis of B. bovis in southern Africa, and can be used to determine parasitaemia and potential carrier status in cattle populations, which is essential in the control of babesiosis.

181. Sci Rep. 2022 Dec 3;12(1):20936. doi: 10.1038/s41598-022-24673-w.
Natural reservoir Rousettus aegyptiacus bat host model of orthonairovirus infection identifies potential zoonotic spillover mechanisms.
Schuh AJ(1)(2), Amman BR(3), Guito JC(3), Graziano JC(3), Sealy TK(3), Kirejczyk

SGM(3), Towner JS(4).
Author information:
(1)Viral Special Pathogens Branch, Division of High-Consequence Pathogens and Pathology, United States Centers for Disease Control and Prevention, Atlanta, GA, USA. wuc2@cdc.gov.
(2)United States Public Health Service Commissioned Corps, Rockville, MD, USA. wuc2@cdc.gov.
(3)Viral Special Pathogens Branch, Division of High-Consequence Pathogens and Pathology, United States Centers for Disease Control and Prevention, Atlanta, GA, USA.
(4)Viral Special Pathogens Branch, Division of High-Consequence Pathogens and Pathology, United States Centers for Disease Control and Prevention, Atlanta, GA, USA. jit8@cdc.gov.
The human-pathogenic Kasokero virus (KASV; genus Orthonairovirus) has been isolated from the sera of Egyptian rousette bats (ERBs; Rousettus aegyptiacus) captured in Uganda and unengorged Ornithodoros (Reticulinasus) faini ticks collected from the rock crevices of ERB colonies in South Africa and Uganda. Although evidence suggests that KASV is maintained in an enzootic transmission cycle between O. (R.) faini ticks and ERBs with potential for incidental virus spillover to humans through the bite of an infected tick, the vertebrate reservoir status of ERBs for KASV has never been experimentally evaluated. Furthermore, the potential for bat-to-bat and bat-to-human transmission of KASV is unknown. Herein, we inoculate two groups of ERBs with KASV; one group of bats is serially sampled to assess viremia, oral, fecal, and urinary shedding and the second group of bats is serially euthanized to assess virus-tissue tropism. Throughout the study, none of the bats exhibit overt signs of clinical disease. Following the detection of high KASV loads of long duration in blood, oral, fecal, and urine specimens collected from ERBs in the serial sampling group, all bats seroconvert to KASV. ERBs from the serial euthanasia group exhibit high KASV loads indicative of virus replication in the skin at the inoculation site, spleen, and inguinal lymph node tissue, and histopathology and in situ hybridization reveal virus replication in the liver and self-limiting, KASV-induced lymphohistiocytic hepatitis. The results of this study suggest that ERBs are competent, natural vertebrate reservoir hosts for KASV that can sustain viremias of appropriate magnitude and duration to support virus maintenance through bat-tick-bat transmission cycles. Viral shedding data suggests that KASV might also be transmitted bat-to-bat and highlights the potential for KASV spillover to humans through contact with infectious oral secretions, feces, or urine.

182. Acta Trop. 2023 Feb;238:106800. doi: 10.1016/j.actatropica.2022.106800. Epub
Linking spatial distribution of Rhipicephalus appendiculatus to climatic variables important for the successful biocontrol by Metarhizium anisopliae in Eastern Africa.
Guimapi RA(1), Klingen I(2), Tonnang HEZ(3), Nana P(4).
Author information:
(1)Norwegian Institute of Bioeconomy Research (NIBIO), P.O. Box, Ås 1433,

Norway. Electronic address: ritter.guimapi@nibio.no.
(2)Norwegian Institute of Bioeconomy Research (NIBIO), P.O. Box, Ås 1433, Norway.
(3)International Centre of Insect Physiology and Ecology (icipe), P.O. Box 30772-00100, Nairobi, Kenya.
(4)Advanced School of Agriculture, Forestry, Water Resources and Environment, University of Ebolowa, P.O. Box 786, Ebolowa, Cameroon.
Cattle production is constantly threatened by diseases like East Coast fever, also known as theileriosis, caused by the protozoan parasite Theileria parva which is transmitted by ticks such as the brown ear tick, Rhipicephalus appendiculatus. To reduce the extensive use of chemical acaricides, fungal-based microbial control agents such as Metarhizium anisopliae have been tested and show promising results against R. appendiculatus both in field and in semi-field experiments in Africa. However, no known endeavors to link the spatial distribution of R. appendiculatus to climatic variables important for the successful application of M. anisopliae in selected East African countries exists. This work therefore aims to improve the successful application of M. anisopliae against R. appendiculatus by designing a temperature-dependent model for the efficacy of M. anisopliae against three developmental stages (larvae, nymphs, adults) of R. appendiculatus. Afterward a spatial prediction of potential areas where this entomopathogenic fungus might cause a significant epizootic in R. appendiculatus population in three selected countries (Kenya, Tanzania, Uganda) in Eastern Africa were generated. This can help to determine whether the temperature and rainfall at a local or regional scale might give good conditions for application of M. anisopliae and successful microbial control of R. appendiculatus.

183. Microorganisms. 2022 Nov 30;10(12):2384. doi: 10.3390/microorganisms10122384.
Molecular Characterization of Octopamine/Tyramine Receptor Gene of Amitraz-Resistant Rhipicephalus (Boophilus) decoloratus Ticks from Uganda.
Vudriko P(1)(2), Umemiya-Shirafuji R(3), Tayebwa DS(1), Byaruhanga J(1), Byamukama B(1), Tumwebaze M(1), Xuan X(3), Suzuki H(2)(3).
Author information:
(1)Research Center for Tropical Diseases and Vector Control, Department of Veterinary Pharmacy, Clinics and Comparative Medicine, School of Veterinary Medicine and Animal Resources, College of Veterinary Medicine, Animal Resources and Biosecurity, Makerere University, Kampala P.O. Box 7062, Uganda.
(2)National Research Center for Protozoan Diseases, Obihiro University of Agriculture and Veterinary Medicine, Inada-Cho, Obihiro 080-8555, Japan.
(3)United Graduate School of Veterinary Sciences, Gifu University, 1-1 Yanagido, Gifu 501-1193, Japan.
We previously reported the emergence of amitraz-resistant Rhipicephalus (Boophilus) decoloratus ticks in the western region of Uganda. This study characterized the octopamine/tyramine receptor gene (OCT/Tyr) of amitraz-resistant and -susceptible R. (B.) decoloratus ticks from four regions of Uganda. The OCT/Tyr gene was amplified from genomic DNA of 17 R. (B.) decoloratus larval populations of known susceptibility to amitraz. The amplicons

were purified, cloned and sequenced to determine mutations in the partial coding region of the OCT/Tyr gene. The amplified R. (B.) decoloratus OCT/Tyr gene was 91-100% identical to the R. (B.) microplus OCT/Tyr gene. Up to 24 single nucleotide polymorphisms (SNPs) were found in the OCT/Tyr gene from ticks obtained from high acaricide pressure areas, compared to 8 from the low acaricide pressure areas. A total of eight amino acid mutations were recorded in the partial OCT/Tyr gene from ticks from the western region, and four of them were associated with amitraz-resistant tick populations. The amino acid mutations M1G, L16F, D41G and V72A were associated with phenotypic resistance to amitraz with no specific pattern. Phylogenetic analysis revealed that the OCT/Tyr gene sequence from this study clustered into two distinct groups that separated the genotype from high acaricide pressure areas from the susceptible populations. In conclusion, this study is the first to characterize the R. (B.) decoloratus OCT/Tyr receptor gene and reports four novel amino acid mutations associated with phenotypic amitraz resistance in Uganda. However, lack of mutations in the ORF of the OCT/Tyr gene fragment for some of the amitraz-resistant R. (B.) decoloratus ticks could suggest that other mechanisms of resistance may be responsible for amitraz resistance, hence the need for further investigation.

184. Parasit Vectors. 2023 Jan 7;16(1):7. doi: 10.1186/s13071-022-05588-x.
Prevalence of Crimean-Congo haemorrhagic fever in livestock following a confirmed human case in Lyantonde district, Uganda.
Atim SA(1)(2)(3), Niebel M(2), Ashraf S(2), Vudriko P(1), Odongo S(1), Balinandi S(4), Aber P(5), Bameka R(6), Ademun AR(3), Masembe C(7), Tweyongyere R(#)(1), Thomson EC(#)(8)(9).
Author information:
(1)College of Veterinary Medicine, Animal Resources and Biosecurity (CoVAB), Makerere University, Kampala, Uganda.
(2)Centre of Virus Research (CVR), University of Glasgow, Glasgow, UK.
(3)Ministry of Agriculture, Animal Industry and Fisheries, Entebbe, Uganda.
(4)Department of Emerging, Re-Emerging and Arbovirus Infections, Uganda Virus Research Institute, Entebbe, Uganda.
(5)Case Western Research Collaboration, Makerere University, Kampala, Uganda.
(6)Lyantonde District Local Government, Lyantonde, Uganda.
(7)College of Natural Resources (CoNAS), Makerere University, Kampala, Uganda.
(8)Centre of Virus Research (CVR), University of Glasgow, Glasgow, UK. emma.thomson@glasgow.ac.uk.
(9)MRC-University of Glasgow Centre for Virus Research, Stoker Building, 464 Bearsden Road, Glasgow, G61 1QH, UK. emma.thomson@glasgow.ac.uk.
(#)Contributed equally
BACKGROUND: Crimean-Congo haemorrhagic fever (CCHF) is a tick-borne viral infection, characterized by haemorrhagic fever in humans and transient asymptomatic infection in animals. It is an emerging human health threat causing sporadic outbreaks in Uganda. We conducted a detailed outbreak investigation in the animal population following the death from CCHF of a 42-year-old male cattle trader in Lyantonde district, Uganda. This was to ascertain the extent of CCHF

virus (CCHFV) circulation among cattle and goats and to identify affected farms
and ongoing increased environmental risk for future human infections.
METHODS: We collected blood and tick samples from 117 cattle and 93 goats, and tested these for anti-CCHFV antibodies and antigen using an enzyme-linked immunosorbent assay (ELISA), quantitative reverse transcriptase polymerase chain reaction (qRT-PCR) and target enrichment next generation sequencing.
RESULTS: CCHFV-specific IgG antibodies were detected in 110/117 (94.0%) cattle and 83/93 (89.3%) goats. Animal seropositivity was independently associated with female animals (AOR = 9.42, P = 0.002), and animals reared under a pastoral animal production system (AOR = 6.02, P = 0.019] were more likely to be seropositive than tethered or communally grazed animals. CCHFV was detected by sequencing in Rhipicephalus appendiculatus ticks but not in domestic animals.
CONCLUSION: This investigation demonstrated very high seroprevalence of CCHFV antibodies in both cattle and goats in farms associated with a human case of CCHF in Lyantonde. Therefore, building surveillance programs for CCHF around farms in this area and the Ugandan cattle corridor is indicated, in order to identify opportunities for case prevention and control.

185. Pathogens. 2023 Jan 10;12(1):115. doi: 10.3390/pathogens12010115.
Assessment of the Impact of Early Diagnosis and Early Treatment in the Integrated Control of East Coast Fever (ECF) Involving Acquired Immunity Induced by Natural Infection in Ankole Cattle.
Nanteza A(1), Nsadha Z(1), Nsubuga J(1), Oligo S(1), Kazibwe A(1), Terundaja C(1), Matovu E(1), Lubega GW(1).
Author information:
(1)College of Veterinary Medicine, Animal Resources and Biosecurity, Makerere University, Kampala P.O. Box 7062, Uganda.
The integrated control of East Coast fever (ECF) by early diagnosis and treatment involving acquired immunity induced by natural infection in Ankole cattle was assessed. A longitudinal study was carried out in Kiruhura district, southwestern Uganda for six months on 244 Ankole breed of cattle from 18 herds under natural tick challenge and relaxed tick control measures. Calves aged three to six months old were recruited and monitored daily by farmers for detection of ECF clinical symptoms. The reported sick animals were treated using Buparvaquone and treatment outcome determined. Monthly follow-ups and blood collections were done to monitor ECF status. Blood was analyzed for Theileria parva parasites by microscopy, DNA by polymerase chain reaction (PCR) and antibodies by enzyme-linked immunosorbent assay (ELISA). The overall prevalence of ECF clinical disease within six months period was 30.3% (74). The major symptoms of early clinical ECF disease were fever and enlarged parotid or prescapular lymph nodes. Clinical cases were categorized as mild, 24% (18) or moderate, 76% (56). There was an overall recovery rate of 100% (74) of the ECF cases whereby 94.6% (70) recovered promptly and 5.4% (4) recovered slowly. Based on blood analysis, prevalence of ECF at baseline was 3.7% (9) by microscopy, 31.1% (76) by PCR and 38.1% (93) by ELISA. A significant increase (p < 0.05) was shown by the increased number of calves with T. parva specific antibodies in the sera from 38.1% at baseline to 68.8% after six months. High antibody levels

(positive percentage ≥ 50%) were detected in all ECF-treated and recovered calves at the end of six months. The acquired immunity to ECF was high in treated and recovered cattle, indicating that natural exposure to infection, accurate early diagnosis and effective treatment enhance development of immune-protection in indigenous cattle in an endemic area. The prominent early clinical symptoms for ECF could be exploited in the development of decision support tools for chemotherapy and other integrated control measures.

186. Vaccines (Basel). 2022 Dec 31;11(1):99. doi: 10.3390/vaccines11010099.
Inspiring Anti-Tick Vaccine Research, Development and Deployment in Tropical Africa for the Control of Cattle Ticks: Review and Insights.
Kasaija PD(1)(2), Contreras M(1), Kirunda H(3), Nanteza A(4), Kabi F(2), Mugerwa S(2), de la Fuente J(1)(5).
Author information:
(1)SaBio, Instituto de Investigación en Recursos Cinegéticos (IREC), Consejo Superior de Investigaciones Científicas (CSIC), Universidad de Castilla-La Mancha (UCLM)-Junta de Comunidades de Castilla-La Mancha (JCCM), Ronda de Toledo s/n, 13005 Ciudad Real, Spain.
(2)National Livestock Resources Research Institute (NaLIRRI), Kampala P.O. Box 5704, Uganda.
(3)National Agricultural Research Organisation (NARO), Mbarara ZARDI, Mbarara P.O. Box 389, Uganda.
(4)College of Veterinary Medicine, Animal Resources and Biosecurity, Makerere University, Kampala P.O. Box 7062, Uganda.
(5)Department of Veterinary Pathobiology, Center for Veterinary Health Sciences, Oklahoma State University, Stillwater, OK 74078, USA.
Ticks are worldwide ectoparasites to humans and animals, and are associated with numerous health and economic effects. Threatening over 80% of the global cattle population, tick and tick-borne diseases (TTBDs) particularly constrain livestock production in the East, Central and Southern Africa. This, therefore, makes their control critical to the sustainability of the animal industry in the region. Since ticks are developing resistance against acaricides, anti-tick vaccines (ATVs) have been proposed as an environmentally friendly control alternative. Whereas they have been used in Latin America and Australia to reduce tick populations, pathogenic infections and number of acaricide treatments, commercially registered ATVs have not been adopted in tropical Africa for tick control. This is majorly due to their limited protection against economically important tick species of Africa and lack of research. Recent advances in various omics technologies and reverse vaccinology have enabled the identification of many candidate anti-tick antigens (ATAs), and are likely to usher in the next generation of vaccines, for which Africa should prepare to embrace. Herein, we highlight some scientific principles and approaches that have been used to identify ATAs, outline characteristics of a desirable ATA for vaccine design and propose the need for African governments to investment in ATV research to develop vaccines relevant to local tick species (personalized vaccines). We have also discussed the prospect of incorporating anti-tick vaccines into the integrated TTBDs control strategies in the sub-Saharan Africa,

citing the case of Uganda.

187. Parasit Vectors. 2023 Jan 22;16(1):22. doi: 10.1186/s13071-022-05632-w.
Survey of ticks and tick-borne pathogens in wild chimpanzee habitat in Western Uganda.
Lacroux C(1)(2)(3)(4), Bonnet S(5)(6), Pouydebat E(7), Buysse M(8)(9), Rahola N(8), Rakotobe S(10), Okimat JP(11), Koual R(8), Asalu E(12), Krief S(#)(13)(14), Duron O(#)(15).
Author information:
(1)UMR 7206 CNRS/MNHN/P7, Eco-anthropologie, Museum National d'Histoire Naturelle, Musée de l'Homme, 17 Place du Trocadéro, 75116, Paris, France. camille.lacroux@mnhn.fr.
(2)Sebitoli Chimpanzee Project, Great Ape Conservation Project, Kibale National Park, Fort Portal, Uganda. camille.lacroux@mnhn.fr.
(3)UMR 7179 CNRS/MNHN, Mécanismes Adaptatifs et Evolution, Museum National d'Histoire Naturelle, 57 Rue Cuvier, 75231, Paris, France. camille.lacroux@mnhn.fr.
(4)La Phocéenne de Cosmétique, ZA Les Roquassiers, 174 Rue de la Forge, 13300, Salon-de-Provence, France. camille.lacroux@mnhn.fr.
(5)UMR 2000, Ecology and Emergence of Arthropod-Borne Pathogens, Institut Pasteur/CNRS/Université Paris-Cité, 75015, Paris, France.
(6)Animal Health Department, INRAE, 37380, Nouzilly, France.
(7)UMR 7179 CNRS/MNHN, Mécanismes Adaptatifs et Evolution, Museum National d'Histoire Naturelle, 57 Rue Cuvier, 75231, Paris, France.
(8)UMR 5290 MIVEGEC (Maladies Infectieuses et Vecteurs : Ecologie, Génétique, Evolution et Contrôle), CNRS/IRD/Université de Montpellier, 911 Avenue Agropolis, 34394, Montpellier, France.
(9)MEEDiN (Montpellier Ecology and Evolution of Disease Network), Montpellier, France.
(10)UMR BIPAR ANSES-INRAE-EnvA, Laboratoire Santé Animale, 94701, Maisons-Alfort, France.
(11)Sebitoli Chimpanzee Project, Great Ape Conservation Project, Kibale National Park, Fort Portal, Uganda.
(12)Uganda Wildlife Authority, Plot 7 Kira Road, Kamwokya, Kampala City, Uganda.
(13)UMR 7206 CNRS/MNHN/P7, Eco-anthropologie, Museum National d'Histoire Naturelle, Musée de l'Homme, 17 Place du Trocadéro, 75116, Paris, France. sabrina.krief@mnhn.fr.
(14)Sebitoli Chimpanzee Project, Great Ape Conservation Project, Kibale National Park, Fort Portal, Uganda. sabrina.krief@mnhn.fr.
(15)UMR 5290 MIVEGEC (Maladies Infectieuses et Vecteurs : Ecologie, Génétique, Evolution et Contrôle), CNRS/IRD/Université de Montpellier, 911 Avenue Agropolis, 34394, Montpellier, France. olivier.duron@ird.fr.
(#)Contributed equally
BACKGROUND: Ticks and tick-borne pathogens significantly impact both human and animal health and therefore are of major concern to the scientific community. Knowledge of tick-borne pathogens is crucial for prescription of mitigation measures. In Africa, much research on ticks has focused on domestic animals.

Little is known about ticks and their pathogens in wild habitats and wild animals like the endangered chimpanzee, our closest relative.
METHODS: In this study, we collected ticks in the forested habitat of a community of 100 chimpanzees living in Kibale National Park, Western Uganda, and assessed how their presence and abundance are influenced by environmental factors. We used non-invasive methods of flagging the vegetation and visual search of ticks both on human team members and in chimpanzee nests. We identified adult and nymph ticks through morphological features. Molecular techniques were used to detect and identify tick-borne piroplasmids and bacterial pathogens.
RESULTS: A total of 470 ticks were collected, which led to the identification of seven tick species: Haemaphysalis parmata (68.77%), Amblyomma tholloni (20.70%), Ixodes rasus sensu lato (7.37%), Rhipicephalus dux (1.40%), Haemaphysalis punctaleachi (0.70%), Ixodes muniensis (0.70%) and Amblyomma paulopunctatum (0.35%). The presence of ticks, irrespective of species, was influenced by temperature and type of vegetation but not by relative humidity. Molecular detection revealed the presence of at least six genera of tick-borne pathogens (Babesia, Theileria, Borrelia, Cryptoplasma, Ehrlichia and Rickettsia). The Afrotopical tick Amblyomma tholloni found in one chimpanzee nest was infected by Rickettsia sp.
CONCLUSIONS: In conclusion, this study presented ticks and tick-borne pathogens in a Ugandan wildlife habitat whose potential effects on animal health remain to be elucidated.

188. PLoS Negl Trop Dis. 2023 Feb 2;17(2):e0011107. doi:
10.1371/journal.pntd.0011107. eCollection 2023 Feb.
Knowledge, attitudes, and practices of Crimean Congo hemorrhagic fever among livestock value chain actors in Kagadi district, Uganda.
Ayebare D(1)(2), Menya M(3), Mulyowa A(3), Muhwezi A(3), Tweyongyere R(3), Atim SA(2).
Author information:
(1)Royal (Dick) School of Veterinary Studies, University of Edinburgh, Edinburgh, United Kingdom.
(2)Ministry of Agriculture, Animal Industry and Fisheries, Entebbe, Uganda.
(3)Makerere University, Kampala, Uganda.
BACKGROUND: Crimean Congo hemorrhagic fever (CCHF) is a zoonotic tick-borne disease with an increasing number of outbreaks among communities in Uganda. Following the disease outbreak in the western district of Kagadi on 20th February 2020, a KAP survey was conducted to identify knowledge gaps and at-risk behaviors related to the disease among livestock value chain actors.
METHODS: A household survey using a semi-structured questionnaire was conducted in 399 households in the two sub counties of Bwikara and Ruteete, Kagadi district. A focus group discussion with members of the community was conducted as well as key informant interviews with at-risk individuals. Descriptive and inferential analysis was performed using STATA version 13 (Statacorp Texas; USA). Comparative analysis of the data from the two sub counties was also performed using cross tabulations in STATA, between each independent variable

and the subcounty variable. The descriptive and comparative statistics used were minimum, mean and maximum values, standard deviations, frequencies, percentages, chi square values and t-statistics. A chi-square test was then employed on each tabulation, to determine whether there was an association between the two categorical variables or not. The test was set at an alpha level of 0.05, and where the p-value was less than or equal to the alpha value, we concluded that the 2 variables were associated.
RESULTS: Although majority of the respondents believed in the existence of the disease, only 12.8% had knowledge of prevention measures against CCHF. 67.2% of the respondents reported regular interaction with ticks during routine farm operations and they employed tick control measures on their farms. Although the respondents believe the disease is fatal, almost all of them (99%) would welcome a CCHF survivor back into the community. 95.2% of the respondents actively attended to animals but only 25.8% participated in slaughtering animals. Qualitatively, the technical informants had knowledge about CCHF but non technical informants hardly knew about the disease. Limited funding appropriated for local governments, as well as limited engagement in One health activities were some of the barriers highlighted towards the infection prevention and control activities. Most of the focus group discussion participants knew about the disease, but lacked knowledge on its transmission and prevention. Limited access to personal protective equipment and high exposure to tick-prevalent areas when slaughtering and grazing animals respectively, were the major challenges highlighted.
CONCLUSION: Knowledge on CCHF among majority of the respondents was poor. There is a need for educational programs to increase awareness of CCHF in communities. This awareness should be done by both the community leaders and technical people to ensure the community receives enough knowledge on how to prevent and control the disease. To ensure effectiveness of these programs a One health approach should be adopted to implement prevention and control strategies.

189. Clin Infect Dis. 2023 Jun 8;76(11):2018-2024. doi: 10.1093/cid/ciad066.
Guidance on the Use of Convalescent Plasma to Treat Immunocompromised Patients With Coronavirus Disease 2019.
Bloch EM(1), Focosi D(2), Shoham S(3), Senefeld J(4), Tobian AAR(1), Baden LR(5), Tiberghien P(6), Sullivan DJ(7), Cohn C(8), Dioverti V(3), Henderson JP(9), So-Osman C(10)(11), Juskewitch JE(12), Razonable RR(13), Franchini M(14), Goel R(15), Grossman BJ(16), Casadevall A(7), Joyner MJ(4), Avery RK(3), Pirofski LA(17), Gebo KA(3).
Author information:
(1)Department of Pathology, Johns Hopkins University School of Medicine, Baltimore, Maryland, USA.
(2)North-Western Tuscany Blood Bank, Pisa University Hospital, Pisa, Italy.
(3)Department of Medicine, Division of Infectious Diseases, Johns Hopkins University School of Medicine, Baltimore, Maryland, USA.
(4)Department of Anesthesiology and Perioperative Medicine, Mayo Clinic, Rochester, Minnesota, USA.
(5)Department of Infectious Diseases, Brigham and Women's Hospital, Boston,

Massachusetts, USA.
(6)Etablissement Français du Sang, La Plaine-St-Denis and Université de
Franche-Comté, Besançon, France.
(7)Department of Molecular Microbiology and Immunology, Johns Hopkins Bloomberg
School of Public Health, Baltimore, MD, USA.
(8)Department of Laboratory Medicine and Pathology, University of Minnesota,
Minneapolis, Minnesota, USA.
(9)Departments of Internal Medicine (Division of Infectious Diseases) and
Molecular Microbiology, Washington University School of Medicine, St. Louis, MO,
USA.
(10)Department Transfusion Medicine, Division Blood Bank, Sanquin Blood Supply
Foundation, Amsterdam, The Netherlands.
(11)Department Haematology, Erasmus Medical Centre, Rotterdam, The Netherlands.
(12)Department of Laboratory Medicine and Pathology, Mayo Clinic, Rochester
campus, Minnesota, USA.
(13)Department of Internal Medicine, Division of Infectious Diseases, Mayo
Clinic, Rochester, Minnesota, USA.
(14)Department of Hematology and Transfusion Medicine, Carlo Poma Hospital,
Mantua, Italy.
(15)Division of Hematology/Oncology, Simmons Cancer Institute at SIU School of
Medicine and Mississippi Valley Regional Blood Center, Springfield, Illinois,
USA.
(16)Department of Pathology & Immunology, Washington University School of
Medicine, St. Louis, Missouri, USA.
(17)Department of Medicine, Infectious Diseases, Albert Einstein College of
Medicine, Bronx, New York, USA.
Coronavirus disease 2019 (COVID-19) convalescent plasma (CCP) is a safe and effective treatment for COVID-19 in immunocompromised (IC) patients. IC patients have a higher risk of persistent infection, severe disease, and death from COVID-19. Despite the continued clinical use of CCP to treat IC patients, the optimal dose, frequency/schedule, and duration of CCP treatment has yet to be determined, and related best practices guidelines are lacking. A group of individuals with expertise spanning infectious diseases, virology and transfusion medicine was assembled to render an expert opinion statement pertaining to the use of CCP for IC patients. For optimal effect, CCP should be recently and locally collected to match circulating variant. CCP should be considered for the treatment of IC patients with acute and protracted COVID-19; dosage depends on clinical setting (acute vs protracted COVID-19). CCP containing high-titer severe acute respiratory syndrome coronavirus 2 (SARS-CoV-2) antibodies, retains activity against circulating SARS-CoV-2 variants, which have otherwise rendered monoclonal antibodies ineffective.

190. Pathogens. 2023 Feb 10;12(2):295. doi: 10.3390/pathogens12020295.
East Coast Fever Carrier Status and Theileria parva Breakthrough Strains in Recently ITM Vaccinated and Non-Vaccinated Cattle in Iganga District, Eastern Uganda.
Oligo S(1), Nanteza A(1), Nsubuga J(1), Musoba A(1), Kazibwe A(1), Lubega GW(1).

Author information:
(1)College of Veterinary Medicine, Animal Resources and Biosecurity (COVAB), Makerere University, Kampala P.O. Box 7062, Uganda.
East Coast fever (ECF) is a tick-borne disease of cattle that hinders the development of the livestock industry in eastern, central and southern Africa. The 'Muguga cocktail' live vaccine, delivered by an infection and treatment method (ITM), remains the only immunisation strategy of controlling ECF. However, there are challenges of the live vaccine inducing ECF carrier status in immunised animals and the possibility of lack of protection from parasite strains that are antigenically different from the vaccine strains. In Uganda, there are insufficient data regarding the ECF carrier status and T. parva genetic diversity in vaccinated and associated non-vaccinated cattle to assess the effectiveness of ITM vaccination. Blood was collected from recently ECF vaccinated (98) and non-vaccinated (73) cattle from Iganga district in Eastern Uganda at 120 days post-vaccination. The p104 gene nested PCR was used to screen for T. parva DNA, 11 minisatellite and 3 microsatellite markers (SSR) were used for genotyping. Two minisatellite markers (MS7 and MS19) were used to determine whether ECF carrier status was due to the T. parva vaccine or local strains. The prevalence of T. parva based on p104 nPCR was 61.2% (60/98) (RR 2.234, 95% CI 1.49-3.35, p-value < 0.001) among recently vaccinated cattle and 27.4% (20/73) (RR 1.00) among associated non-vaccinated cattle. The Muguga cocktail vaccine strains were responsible for carrier status in 10 (58.8%) by MS7 and 11 (64.7%) by MS19 in vaccinated cattle. Genotypes of T. parva with different-sized alleles to the vaccine strains that could be potential 'breakthroughs' were detected in 2 (11.8%)) and 4 (23.5%) isolates from vaccinated cattle based on MS7 and MS19 minisatellite markers, respectively. Using 14 SSR markers, T. parva diversity was higher in vaccinated (Na = 2.214, Ne = 1.978, He = 0.465) than associated non-vaccinated (Na = 1.071, Ne = 1.048, He = 0.259) cattle. The principal component analysis (PCA) showed isolates from vaccinated cattle were closely related to those from non-vaccinated cattle. The analysis of molecular variance (AMOVA) revealed high genetic variation (96%) within T. parva isolates from vaccinated and non-vaccinated cattle but low variation (4%) between vaccinated and non-vaccinated cattle. This study reveals the role of ITM in inducing the carrier status and higher T. parva genetic diversity in vaccinated cattle. The low genetic variation between T. parva isolates in both vaccinated and non-vaccinated cattle may be suggestive of the protective role of vaccine strains against genetically related local strains in the study area.

191. Insects. 2023 Feb 23;14(3):221. doi: 10.3390/insects14030221.
Mosquito-Borne Diseases and Their Control Strategies: An Overview Focused on Green Synthesized Plant-Based Metallic Nanoparticles.
Onen H(1), Luzala MM(2)(3), Kigozi S(4), Sikumbili RM(3)(5), Muanga CK(2)(3), Zola EN(2)(3), Wendji SN(2)(3), Buya AB(2)(3), Balciunaitiene A(6), Viškelis J(6), Kaddumukasa MA(4), Memvanga PB(2)(3).
Author information:
(1)Department of Entomology, Uganda Virus Research Institute, Plot 51/59 Nakiwogo Road, Entebbe P.O. Box 49, Uganda.

(2)Laboratory of Pharmaceutics and Phytopharmaceutical Drug Development, Faculty of Pharmaceutical Sciences, University of Kinshasa, Kinshasa B.P. 212, Democratic Republic of the Congo.
(3)Centre de Recherche et d'Innovation Technologique en Environnement et en Sciences de la Santé (CRITESS), University of Kinshasa, Kinshasa B.P. 212, Democratic Republic of the Congo.
(4)Department of Biological Sciences, Faculty of Science, Kyambogo University, Kampala P.O. Box 1, Uganda.
(5)Department of Chemistry, Faculty of Science, University of Kinshasa, Kinshasa B.P. 190, Democratic Republic of the Congo.
(6)Institute of Horticulture, Lithuanian Research Centre for Agriculture and Forestry, 54333 Babtai, Lithuania.
Mosquitoes act as vectors of pathogens that cause most life-threatening diseases, such as malaria, Dengue, Chikungunya, Yellow fever, Zika, West Nile, Lymphatic filariasis, etc. To reduce the transmission of these mosquito-borne diseases in humans, several chemical, biological, mechanical, and pharmaceutical methods of control are used. However, these different strategies are facing important and timely challenges that include the rapid spread of highly invasive mosquitoes worldwide, the development of resistance in several mosquito species, and the recent outbreaks of novel arthropod-borne viruses (e.g., Dengue, Rift Valley fever, tick-borne encephalitis, West Nile, yellow fever, etc.).
Therefore, the development of novel and effective methods of control is urgently needed to manage mosquito vectors. Adapting the principles of nanobiotechnology to mosquito vector control is one of the current approaches. As a single-step, eco-friendly, and biodegradable method that does not require the use of toxic chemicals, the green synthesis of nanoparticles using active toxic agents from plant extracts available since ancient times exhibits antagonistic responses and broad-spectrum target-specific activities against different species of vector mosquitoes. In this article, the current state of knowledge on the different mosquito control strategies in general, and on repellent and mosquitocidal plant-mediated synthesis of nanoparticles in particular, has been reviewed. By doing so, this review may open new doors for research on mosquito-borne diseases.

192. Clin Infect Dis. 2023 Jul 26;77(2):237-241. doi: 10.1093/cid/ciad185.
Coronavirus Disease 2019 Convalescent Plasma Utilization in the United States: Data From the National Inpatient Sample.
Bloch EM(1), Goel R(1)(2), Zhu X(1), Patel EU(1), Shoham S(3), Sullivan DJ(4), Gebo KA(3), Casadevall A(4), Tobian AAR(1).
Author information:
(1)Division of Transfusion Medicine, Department of Pathology, Johns Hopkins University, Baltimore, Maryland, USA.
(2)Simmons Cancer Institute at Southern Illinois University, Springfield, Illinois, USA.
(3)Department of Medicine, Division of Infectious Diseases, Johns Hopkins University School of Medicine, Baltimore, Maryland, USA.
(4)Department of Molecular Microbiology and Immunology, Johns Hopkins Bloomberg

School of Public Health, Baltimore, Maryland, USA.
Coronavirus disease 2019 (COVID-19) convalescent plasma (CCP) use between October and December 2020 was characterized using the National Inpatient Sample database. CCP was administered in 18.0% of COVID-19-associated hospitalizations and was strongly associated with older age and increased disease severity. There were disparities in the receipt of CCP by race and ethnicity, geography, and insurance.

193. Parasit Vectors. 2023 Mar 30;16(1):117. doi: 10.1186/s13071-023-05709-0.
Tick-borne pathogens and body condition of cattle in smallholder rural livestock production systems in East and West Africa.
Heylen DJA(1)(2)(3), Kumsa B(4), Kimbita E(5), Frank MN(6), Muhanguzi D(6), Jongejan F(7), Adehan SB(8), Toure A(9), Aboagye-Antwi F(10), Ogo NI(11), Juleff N(12), Crafford D(13), Fourie J(13), Labuchange M(14), Madder M(15).
Author information:
(1)Evolutionary Ecology Group, Department of Biology, University of Antwerp, Wilrijk, Belgium. dieter.heylen@uantwerpen.be.
(2)Eco-Epidemiology Group, Department of Biomedical Sciences, Institute of Tropical Medicine, Antwerp, Belgium. dieter.heylen@uantwerpen.be.
(3)Interuniversity Institute for Biostatistics and Statistical Bioinformatics, Hasselt University, Diepenbeek, Belgium. dieter.heylen@uantwerpen.be.
(4)Department of Parasitology, College of Veterinary Medicine and Agriculture, Addis Ababa University, Bishoftu, Ethiopia.
(5)Department of Veterinary Microbiology and Parasitology, College of Veterinary Medicine and Biomedical Sciences, Sokoine University of Agriculture, PO Box 3019, Morogoro, Tanzania.
(6)Department of Bio-Molecular Resources and Bio-Laboratory Sciences (BBS), College of Veterinary Medicine, Makerere University, Kampala, Uganda.
(7)Department of Veterinary Tropical Diseases, Faculty of Veterinary Science, University of Pretoria, Onderstepoort, South Africa.
(8)Zootechnical, Veterinary and Halieutic Research Laboratory (LRZVH), National Institute of Agricultural Research (INRAB), 01 BP 884, Cotonou, Benin.
(9)Université Nangui Abrogoua, UFR Sciences de La Nature, 02 Bp 801, Abidjan 02, Côte d'Ivoire.
(10)Department of Animal Biology and Conservation Science, School of Biological Sciences, College of Basic and Applied Sciences, University of Ghana, Legon-Accra, Ghana.
(11)National Veterinary Research Institute, Vom, Plateau State, Nigeria.
(12)Bill & Melinda Gates Foundation, Seattle, WA, USA.
(13)Clinvet International Pty (Ltd), 1479 Talmadge Hill South, Waverly, NY, 14892, USA.
(14), Clinomics, Bloemfontein, South Africa.
(15)Clinglobal, B03/04, The Tamarin Commercial Hub, Jacaranda Avenue, Tamarin, 90903, Mauritius.
BACKGROUND: The majority of the African population lives in rural areas where they heavily depend on crop and livestock production for their livelihoods.
Given their socio-economic importance, we initiated a standardized multi-country

(Benin, Burkina Faso, Ghana, Nigeria, Ethiopia Tanzania and Uganda) surveillance study to assess the current status of important tick-borne haemoparasites (TBHPs) of cattle.
METHODS: We assessed pathogen prevalences (Anaplasma marginale, Anaplasma centrale, Babesia bigemina, Babesia bovis, Ehrlichia ruminantium, and Theileria parva) in the blood of 6447 animals spread over fourteen districts (two districts per country). In addition, we screened for intrinsic (sex, weight, body condition) and extrinsic (husbandry, tick exposure) risk factors as predictors of infections with TBHPs.
RESULTS: There was a large macro-geographic variation observed in A. marginale, B. bigemina, B. bovis and E. ruminantium prevalences. Most correlated with the co-occurrence of their specific sets of vector-competent ticks. Highest numbers of infected cattle were found in Ghana and Benin, and lowest in Burkina Faso. While T. parva was seldomly found (Uganda only: 3.0%), A. marginale was found in each country with a prevalence of at least 40%. Babesia bovis infected individuals had lower body condition scores. Age (as estimated via body weight) was higher in A. marginale infected cattle, but was negatively correlated with B. bigemina and E. ruminantium prevalences. Ehrlichia ruminantium infection was more often found in males, and A. marginale more often in transhumance farming. High levels of co-infection, especially the combination A. marginale × B. bigemina, were observed in all countries, except for Uganda and Burkina Faso. Babesia bigemina was more or less often observed than expected by chance, when cattle were also co-infected with E. ruminantium or A. marginale, respectively.
CONCLUSIONS: Tick-borne pathogens of cattle are ubiquitous in African's smallholder cattle production systems. Our standardized study will help a wide range of stakeholders to provide recommendations for TBHP surveillance and prevention in cattle, especially for B. bovis which heavily impacts production and continues its spread over the African continent via the invasive Rhipicephalus microplus tick.

194. Parasitol Res. 2023 Jun;122(6):1381-1390. doi: 10.1007/s00436-023-07838-y. Epub
Conservation and variation in the region of the Theileria parva p104 antigen coding gene used for PCR surveillance of the parasite.
Obara I(1)(2), Makori P(3), Sibeko KP(4), Bishop RP(5), Nijhof AM(6)(7), Mwamuye M(6)(8).
Author information:
(1)Department of Veterinary Medicine, Institute for Parasitology and Tropical Veterinary Medicine, Freie Universität Berlin, Berlin, Germany. i.b.obara@gmail.com.
(2)Department of Veterinary Medicine, Veterinary Centre for Resistance Research, Freie Universität Berlin, Berlin, Germany. i.b.obara@gmail.com.
(3)University of Eldoret, Eldoret, Kenya.
(4)Vector and Vector-Borne Disease Research Programme, Department of Veterinary Tropical Diseases, Faculty of Veterinary Science, University of Pretoria, Pretoria, South Africa.
(5)Department of Veterinary Microbiology and Pathology, Washington State University, Pullman, WA, USA.

(6)Department of Veterinary Medicine, Institute for Parasitology and Tropical Veterinary Medicine, Freie Universität Berlin, Berlin, Germany.
(7)Department of Veterinary Medicine, Veterinary Centre for Resistance Research, Freie Universität Berlin, Berlin, Germany.
(8)Department of Environment and Natural Resource Management, Africa Nazarene University, Kajiado, Kenya.
The range of the protozoan parasite Theileria parva, which causes East Coast fever in cattle, has been expanding to countries where it has not previously been detected, as a result of cross-border domestic cattle movement. Countries where T. parva has not previously been observed until recently include Cameroon and South Sudan. This raises the issue of the conservation of the p104 antigen gene, on which the nested PCR assay that is widely used for T. parva surveillance in the blood of infected cattle is based. We sampled 40 isolates from six countries widely distributed across the geographical range of the parasite, including eastern, central and southern Africa, for p104 sequence polymorphism. These included parasites from both domestic cattle and the Cape buffalo (Syncerus caffer) wildlife reservoir. The most frequent allelic variants were present in cattle transmissible isolates from multiple widely separated geographical regions in Zambia, Uganda, Kenya, Tanzania, Rwanda and South Africa. These frequent p104 variants were also present in the three component stocks of the Muguga cocktail used for the infection and treatment live immunisation procedure to control T. parva in the field. Other isolates exhibited unique alleles. This includes some of the p104 sequences from Cameroon, which is outside the known range of the Rhipicephalus tick vector and whose origin is therefore unclear. The nested primer oligonucleotides used to generate the amplicons were universally conserved in cattle-derived parasites and a majority of buffalo-derived isolates across the geographical range of the parasite. However, some rare South African buffalo-derived isolates exhibited one or two mismatches with the primer sequences. It therefore remains possible that some p104 alleles may be so divergent that they do not amplify with the current diagnostic primers and are not detectable in surveys, hence the need for increasing knowledge of genetic heterogeneity of diagnostic targets. There was no evidence for positive selection among those p104 mutations that resulted in residue changes. Importantly, the data indicate that the p104-based PCR detection assay should be effective across the majority of the range of T. parva, and if the one or two mismatches are shown in future to result in the primers annealing less efficiently, then the assay can be further improved by introduction of degenerate bases to enable amplification of the less frequent South African buffalo-derived variant p104 genes.

195. Parasite. 2023;30:13. doi: 10.1051/parasite/2023014. Epub 2023 May 10.
Diversity and phylogeny of the tick-borne bacterial genus Candidatus Allocryptoplasma (Anaplasmataceae).
Ouass S(1), Boulanger N(2), Lelouvier B(3), Insonere JL(3), Lacroux C(4), Krief S(5), Asalu E(6), Rahola N(1), Duron O(1).
Author information:
(1)MIVEGEC, University of Montpellier (UM), Centre National de la Recherche

Scientifique (CNRS), Institut pour la Recherche de la Développement (IRD), 34394 Montpellier, France.
(2)University of Strasbourg, French National Reference Center for Borrelia, Hôpitaux Universitaires de Strasbourg, Strasbourg, France.
(3)Vaiomer, 516 rue Pierre et Marie Curie, 31670 Labège, France.
(4)UMR 7206 CNRS/MNHN/P7, Eco-anthropologie, Muséum National d'Histoire Naturelle, Musée de l'Homme, 17 place du Trocadéro, 75116, Paris, France - La Phocéenne de Cosmétique, ZA Les Roquassiers, 174 Rue de la Forge, 13300 Salon-de-Provence, France - Sebitoli Chimpanzee Project, Great Ape Conservation Project, Kibale National Park, Fort Portal, Uganda.
(5)UMR 7206 CNRS/MNHN/P7, Eco-anthropologie, Muséum National d'Histoire Naturelle, Musée de l'Homme, 17 place du Trocadéro, 75116, Paris, France - Sebitoli Chimpanzee Project, Great Ape Conservation Project, Kibale National Park, Fort Portal, Uganda.
(6)Sebitoli Chimpanzee Project, Great Ape Conservation Project, Kibale National Park, Fort Portal, Uganda - Uganda Wildlife Authority, Kibale National Park, Uganda.
The family Anaplasmataceae includes tick-borne bacteria of major public and veterinary health interest, as best illustrated by members of the genera Anaplasma and Ehrlichia. Recent epidemiological surveys have also reported on the presence of a novel putative genus in the Anaplasmataceae, Candidatus Allocryptoplasma, previously described as Candidatus Cryptoplasma in the western black-legged tick, Ixodes pacificus. However, the genetic diversity of Ca. Allocryptoplasma and its phylogenetic relationship with other Anaplasmataceae remain unclear. In this study, we developed a multi-locus sequence typing approach, examining the DNA sequence variation at five genes of Ca. Allocryptoplasma found in ticks. Combining this multi-locus sequence typing and genetic data available on public databases, we found that substantial genetic diversity of Ca. Allocryptoplasma is present in Ixodes, Amblyomma and Haemaphysalis spp. ticks on most continents. Further analyses confirmed that the Ca. Allocryptoplasma of ticks, the Ca. Allocryptoplasma of lizards and some Anaplasma-like bacteria of wild mice cluster into a monophyletic genus, divergent from all other genera of the family Anaplasmataceae. Candidatus Allocryptoplasma appears as a sister genus of Anaplasma and, with the genera Ehrlichia and Neoehrlichia, they form a monophyletic subgroup of Anaplasmataceae associated with tick-borne diseases. The detection of genetically distinct Ca. Allocryptoplasma in ticks of significant medical or veterinary interest supports the hypothesis that it is an emergent genus of tick-borne pathogens of general concern.

196. Microb Pathog. 2023 Aug;181:106179. doi: 10.1016/j.micpath.2023.106179. Epub
Global haplotype distribution of Babesia ovis inferred by 18S rRNA sequences; a phylogeographical systematic review.
Spotin A(1), Dalir F(2), Hazratian T(3), Shekarchi AA(4), Mahami-Oskouei M(2), Farmani M(3), Dolatkhah A(3), Ahmadpour E(2).
Author information:
(1)Immunology Research Center, Tabriz University of Medical Sciences, Tabriz,

Iran; Department of Parasitology and Mycology, School of Medicine, Tabriz
University of Medical, Sciences, Tabriz, Iran. Electronic address:
Adelespotin@gmail.com.
(2)Immunology Research Center, Tabriz University of Medical Sciences, Tabriz,
Iran; Department of Parasitology and Mycology, School of Medicine, Tabriz
University of Medical, Sciences, Tabriz, Iran.
(3)Department of Parasitology and Mycology, School of Medicine, Tabriz
University of Medical, Sciences, Tabriz, Iran.
(4)Department of Pathology, Tabriz University of Medical Sciences, Tabriz, Iran.
The genetic variability of apicomplexan parasite Babesia species is a principal strategy used by piroplasma to evade their hosts' immune responses. The purpose of this review was to evaluate our current knowledge on global haplotype distribution and phylogeography of Babesia ovis derived from sheep, goat, horse and ixodid (hard) ticks. Bibliographic English databases were searched from 2017 to 2023, identifying a total of 11 publications. The 18S ribosomal RNA (18S rRNA) sequences of B. ovis from Asia, Europe, and Africa were retrieved and subjected to estimate the genetic diversity and phylogenetic assessment. A haplotype network indicated a total of 29 haplotypes being classified into two distinct geographical haplogroups I and II including Nigeria and Uganda-derived B. ovis isolates. A moderately high level of genetic diversity was characterized in sheep/tick-derived B. ovis isolates originating from Iraq (Haplotype diversity: 0.781) and Turkey (Hd: 0.841). Based on the cladistic phylogenetic tree, two geographically different lineages of A and B were genetically differentiated except for Turkish isolates, indicating haplotype migration occurred between various geographical clades. In addition, the topology of UPGMA tree indicated that B. ovis population has a distinct clade compared to the rest clades of ovine babesiosis (B. crassa and B. motasi). The present results strengthen our knowledge to evaluate the evolutionary paradigms and transmission dynamics of B. ovis in different regions of the world; also it will provide groundwork for public health policy to control ovine babesiosis.

197. Exp Appl Acarol. 2023 Jun;90(1-2):83-98. doi: 10.1007/s10493-023-00804-4. Epub
Natural Clerodendrum-derived tick repellent: learning from Nepali culture.
Mazuecos L(#)(1), Contreras M(#)(2), Kasaija PD(2)(3), Manandhar P(4),
Grąźlewska W(2)(5), Guisantes-Batan E(6), Gomez-Alonso S(6), Deulofeu K(7),
Fernandez-Moratalla I(8), Rajbhandari RM(4), Sojka D(9), Grubhoffer L(9),
Karmacharya D(4), Gortazar C(2), de la Fuente J(2)(10).
Author information:
(1)Instituto de Investigación en Recursos Cinegéticos IREC-CSIC-UCLM-JCCM, Ronda
de Toledo s/n, Ciudad Real, 13005, Spain. Lorena.Mazuecos@uclm.es.
(2)Instituto de Investigación en Recursos Cinegéticos IREC-CSIC-UCLM-JCCM, Ronda
de Toledo s/n, Ciudad Real, 13005, Spain.
(3)National Livestock Resources Research Institute (NaLIRRI/NARO), Wakiso
District, P.O. Box 5704, Wakiso, Uganda.
(4)Center for Molecular Dynamics Nepal (CMDN), Thapathali Road 11, Kathmandu,
44600, Nepal.
(5)Department of Molecular Biotechnology and Microbiology, Faculty of Chemistry,

Gdańsk University of Technology, Gdańsk, 80-233, Poland.
(6)Instituto Regional de Investigación Científica Aplicada (IRICA), Universidad de Castilla-La Mancha, Ciudad Real, 13005, Spain.
(7)KGJ Collection, Adelfa 75, Ciudad Real, 13005, Spain.
(8), 7 Manantiales 2, Villanueva de los Infantes, 13320, Spain.
(9)Institute of Parasitology, Biology Centre, Academy of Sciences of the Czech Republic, Branišovská 1160/31, České Budějovice, 37005, Czech Republic.
(10)Department of Veterinary Pathobiology, Center for Veterinary Health Sciences, Oklahoma State University, Stillwater, OK, 74078, USA.
(#)Contributed equally
Ticks attaching to ear canals of humans and animals are the cause of otoacariasis, common in rural areas of Nepal. The plant Clerodendrum viscosum is used in multiple indigenous systems of medicine by ethnic communities in the Indo-Nepali-Malaysian region. Visiting the Chitwan National Park, we learned that in indigenous medicine, flower extract of C. viscosum is utilized to treat digestive disorders and extracts from leaves as tick repellent to prevent ticks from invading or to remove them from the ear canal. The objective of our study was to provide support to indigenous medicine by characterizing the in vivo effect of leave extracts on ticks under laboratory conditions and its phytochemical composition. We collected plant parts of C. viscosum (leaves and flowers) and mango (Mangifera indica) leaves at the Chitwan National Park, previously associated with repellent activity to characterize their effect on Ixodes ricinus ticks by in vivo bioassays. A Q-ToF high-resolution analysis (HPLC-ESI-QToF) was conducted to elucidate phenolic compounds with potential repellent activity. Clerodendrum viscosum and M. indica leaf extracts had the highest tick repellent efficacy (%E = 80-100%) with significant differences when compared to C. viscosum flowers extracts (%E = 20-60%) and phosphate-buffered saline. Phytochemicals with tick repellent function as caffeic acid, fumaric acid and p-coumaric acid glucoside were identified in C. viscosum leaf extracts by HPLC-ESI-QToF, but not in non-repellent flower extracts. These results support the Nepali indigenous medicine application of C. viscosum leaf extracts to repel ticks. Additional research is needed for the development of natural and green repellent formulations to reduce the risks associated with ticks resistant to acaricides.

198. Parasit Vectors. 2023 Jun 19;16(1):206. doi: 10.1186/s13071-023-05801-5.
Tick communities of cattle in smallholder rural livestock production systems in sub-Saharan Africa.
Heylen DJA(1)(2), Kumsa B(3), Kimbita E(4), Frank MN(5), Muhanguzi D(5), Jongejan F(6), Adehan SB(7), Toure A(8), Aboagye-Antwi F(9), Ogo NI(10), Juleff N(11), Fourie J(12), Evans A(13), Byaruhanga J(14), Madder M(13).
Author information:
(1)Evolutionary Ecology Group, Department of Biology, University of Antwerp, Wilrijk, Belgium. dieter.heylen@uantwerpen.be.
(2)Interuniversity Institute for Biostatistics and Statistical Bioinformatics, Hasselt University, Diepenbeek, Belgium. dieter.heylen@uantwerpen.be.
(3)Department of Parasitology, College of Veterinary Medicine and Agriculture,

Addis Ababa University, Bishoftu, Ethiopia.
(4)Department of Veterinary Microbiology and Parasitology, College of Veterinary Medicine and Biomedical Sciences, Sokoine University of Agriculture, 3019, Morogoro, Tanzania.
(5)Department of Bio-molecular Resources and Bio-Laboratory Sciences (BBS), College of Veterinary Medicine, Makerere University, Kampala, Uganda.
(6)Department of Veterinary Tropical Diseases, Faculty of Veterinary Science, University of Pretoria, Onderstepoort, South Africa.
(7)National Institute of Agricultural Research (INRAB), Zootechnical, Veterinary and Halieutic Research Laboratory (LRZVH), 01 BP 884, Cotonou, Benin.
(8)Université Nangui Abrogoua, UFR Sciences de la Nature, 02 Bp 801, Abidjan 02, Côte d'Ivoire.
(9)Department of Animal Biology and Conservation Science, School of Biological Sciences, College of Basic and Applied Sciences, University of Ghana, Legon-Accra, Ghana.
(10)National Veterinary Research Institute, Vom, Plateau State, Nigeria.
(11)Bill & Melinda Gates Foundation, Seattle, WA, USA.
(12)Clinvet International Pty (Ltd), 1479 Talmadge Hill South, Waverly, NY, 14892, USA.
(13)Clinglobal, B03/04, The Tamarin Commercial Hub, Jacaranda Avenue, Tamarin, 90903, Mauritius.
(14)Research Center for Tropical Diseases and Vector Control (RTC), Department of Veterinary Pharmacy, Clinics and Comparative Medicine, School of Veterinary Medicine and Animal Resources, College of Veterinary Medicine, Animal Resources and Biosecurity, Makerere University, Kampala, Uganda.
Erratum in
Parasit Vectors. 2023 Aug 9;16(1):272.
Parasit Vectors. 2023 Nov 16;16(1):423.
BACKGROUND: The majority of the African population lives in rural areas and depends on agriculture for their livelihoods. To increase the productivity and sustainability of their farms, they need access to affordable yield-enhancing inputs of which parasite control is of paramount importance. We therefore determined the status of current tick species with the highest economic impact on cattle by sampling representative numbers of animals in each of seven sub-Saharan countries.
METHODS: Data included tick species' half-body counts from approximately 120 cattle at each of two districts per country, collected four times in approximately 1 year (to include seasonality). Study sites were chosen in each country to include high cattle density and tick burden.
RESULTS: East Africa (Ethiopia, Uganda and Tanzania) showed overall a higher diversity and prevalence in tick infestations compared to West African countries (Benin, Burkina Faso, Ghana and Nigeria). In East Africa, Amblyomma variegatum (vector of Ehrlichia ruminantium), Rhipicephalus microplus (Babesia bovis, B. bigemina, Anaplasma marginale), R. evertsi evertsi (A. marginale) and R. appendiculatus (Theileria parva) were the most prevalent tick species of economic importance. While the latter species was absent in West Africa, here both A. variegatum and R. microplus occurred in high numbers. Rhipicephalus

microplus had spread to Uganda, infesting half of the cattle sampled. Rhipicephalus appendiculatus is known for its invasive behaviour and displacement of other blue tick species, as observed in other East and West African countries. Individual cattle with higher body weights, as well as males, were more likely to be infested. For six tick species, we found reduced infestation levels when hosts were treated with anti-parasiticides.
CONCLUSIONS: These baseline data allow the determination of possible changes in presence and prevalence of ticks in each of the countries targeted, which is of importance in the light of human-caused climate and habitat alterations or anthropogenic activities. As many of the ticks in this study are vectors of important pathogens, but also, as cattle may act as end hosts for ticks of importance to human health, our study will help a wide range of stakeholders to provide recommendations for tick infestation surveillance and prevention.

199. Parasit Vectors. 2023 Jul 6;16(1):224. doi: 10.1186/s13071-023-05803-3.
Control of ticks and tick-borne diseases in Africa through improved diagnosis and utilisation of data on acaricide resistance.
Bishop RP(1), Githaka NW(2), Bazarusanga T(3), Bhushan C(4), Biguezoton A(5), Vudriko P(6), Muhanguzi D(7), Tumwebaze M(6), Bosco TJ(8), Shacklock C(9), Kiama J(10), Madder M(11), Maritz-Olivier C(12), Zhao W(13), Maree F(14), Majekodunmi AO(15), Halos L(16), Jongejan F(13), Evans A(11).
Author information:
(1)Washington State University, Pullman, WA, USA. bishop5030@gmail.com.
(2)International Livestock Research Institute (ILRI), Nairobi, Kenya.
(3)Farmers Solutions Ltd., Kigali, Rwanda.
(4)Elanco Animal Health, Monheim am Rhein, Germany.
(5)CIRDES, Bobo Dioulasso, Burkina Faso.
(6)Research Centre for Tropical Diseases and Vector Control (RTC) Makerere University, Kampala, Uganda.
(7)Molecular and Computational Biology Research Group, Makerere University, Kampala, Uganda.
(8)Veterinary Office Kiboga District, Kiboga, Uganda.
(9)Afrivet Tick Unit, Howick, South Africa.
(10)Department of Veterinary Services, Kabete, Kenya.
(11)Clinglobal, Tamarin, Mauritius.
(12)Department of Biochemistry, Genetics and Microbiology, University of Pretoria, Pretoria, South Africa.
(13)United Nations Food and Agriculture Organisation, Rome, Italy.
(14)Clinomics, Bloemfontein, South Africa.
(15)Emergency Centre for Transboundary Diseases (ECTAD), Food and Agriculture Organisation of the United Nations, Rome, Italy.
(16)Bill and Melinda Gates Foundation, Seattle, WA, USA.
A meeting, sponsored by the Bill and Melinda Gates Foundation (BMGF) and organised by Clinglobal, was held at The International Livestock Research Institute (ILRI) in Nairobi, Kenya, from 19th - to 21st October 2022. The meeting assembled a unique group of experts on tick control in Africa. Academia, international agencies (FAO and ILRI), the private Animal Health sector and

government veterinary services were represented. The significant outcomes included: (i) a shared commitment to standardisation and improvement of acaricide resistance bioassay protocols, particularly the widely used larval packet test (LPT); (ii) development of novel molecular assays for detecting acaricide resistance; (3) creation of platforms for disseminating acaricide resistance data to farmers, veterinary service providers and veterinary authorities to enable more rational evidence-based control of livestock ticks. Implementation of enhanced control will be facilitated by several recently established networks focused on control of parasites in Africa and globally, whose activities were presented at the meeting. These include a newly launched community of practice on management of livestock ticks, coordinated by FAO, an African module of the World Association for the Advancement of Veterinary Parasitology (WAAVP-AN) and the MAHABA (Managing Animal Health and Acaricides for a Better Africa) initiative of Elanco Animal Health.

200. PLoS Negl Trop Dis. 2023 Jul 27;17(7):e0011273. doi:
10.1371/journal.pntd.0011273. eCollection 2023 Jul.
Wide distribution of Mediterranean and African spotted fever agents and the first identification of Israeli spotted fever agent in ticks in Uganda.
Eneku W(1)(2), Erima B(2), Byaruhanga AM(2), Atim G(2), Tugume T(2), Ukuli QA(2), Kibuuka H(2), Mworozi E(3), Douglas C(4), Koehler JW(4), Cleary NG(5), von Fricken ME(5), Tweyongyere R(1), Wabwire-Mangen F(2)(6), Byarugaba DK(1)(2).
Author information:
(1)Makerere University, College of Veterinary Medicine, Kampala, Uganda.
(2)Makerere University Walter Reed Project, Kampala, Uganda.
(3)Makerere University, College of Health Sciences, Kampala, Uganda.
(4)Diagnostic Systems Division, USAMRIID, Fort Detrick, Maryland, United States of America.
(5)Global and Community Health, George Mason University, Fairfax, Virginia, United States of America.
(6)Makerere University, School of Public Health, Kampala, Uganda.
Rickettsia microorganisms are causative agents of several neglected emerging infectious diseases in humans transmitted by arthropods including ticks. In this study, ticks were collected from four geographical regions of Uganda and pooled in sizes of 1-179 ticks based on location, tick species, life stage, host, and time of collection. Then, they were tested by real-time PCR for Rickettsia species with primers targeting gltA, 17kDa and ompA genes, followed by Sanger sequencing of the 17kDa and ompA genes. Of the 471 tick pools tested, 116 (24.6%) were positive for Rickettsia spp. by the gltA primers. The prevalence of Rickettsia varied by district with Gulu recording the highest (30.1%) followed by Luwero (28.1%) and Kasese had the lowest (14%). Tick pools from livestock (cattle, goats, sheep, and pigs) had the highest positivity rate, 26.9%, followed by vegetation, 23.1%, and pets (dogs and cats), 19.7%. Of 116 gltA-positive tick pools, 86 pools were positive using 17kDa primers of which 48 purified PCR products were successfully sequenced. The predominant Rickettsia spp. identified was R. africae (n = 15) in four tick species, followed by R. conorii (n = 5) in three tick species (Haemaphysalis elliptica, Rhipicephalus

appendiculatus, and Rh. decoloratus). Rickettsia conorii subsp. israelensis was detected in one tick pool. These findings indicate that multiple Rickettsia spp. capable of causing human illness are circulating in the four diverse geographical regions of Uganda including new strains previously known to occur in the Mediterranean region. Physicians should be informed about Rickettsia spp. as potential causes of acute febrile illnesses in these regions. Continued and expanded surveillance is essential to further identify and locate potential hotspots with Rickettsia spp. of concern.

201. Pathogens. 2023 Jun 26;12(7):875. doi: 10.3390/pathogens12070875.
Acaricide Resistance Development in Rhipicephalus (Boophilus) microplus (Acari: Ixodidae) Populations against Amitraz and Deltamethrin on Communal Farms of the King Sabata Dalindyebo Municipality, South Africa.
Dzemo WD(1)(2), Vudriko P(3), Ramatla T(2), Thekisoe O(2).
Author information:
(1)Department of Biological and Environmental Sciences, Faculty of Natural Sciences, Walter Sisulu University, Mthatha 5117, South Africa.
(2)Unit for Environmental Sciences and Management, North-West University, Potchefstroom 2531, South Africa.
(3)Research Center for Tropical Diseases and Vector Control, Department of Veterinary Pharmacy, Clinics and Comparative Medicine, College of Veterinary Medicine, Animal Resources and Biosecurity, Makerere University, Kampala P.O. Box 7062, Uganda.
Chemical acaricides are widely used to control ticks and tick-borne pathogens in cattle. However, prolonged and indiscriminate use of these chemicals inevitably leads to the selection of resistant ticks. In-vitro bioassays (adult and larval immersion tests) were conducted to assess amitraz and deltamethrin resistance in Rhipicephalus (Boophilus) microplus populations from communal farms of the King Sabata Dalindyebo municipality of South Africa. Data generated on percentage inhibition of oviposition (%IO) revealed that all the tick populations assessed showed resistance (%IO ≤ 95%) to at least one of the acaricides. All six tick populations assessed for efficacy (%IO ≥ 95%) at the DD) with deltamethrin were resistant (%IO ≤ 95%) and only one of the six tick populations assessed for efficacy with amitraz was susceptible. Based on the resistance ratios (RR), the adult immersion test detected amitraz and deltamethrin resistance in three (RR ranging from 2.30 to 3.21) and five (RR ranging from 4.10 to 14.59) of the six tick populations, respectively. With the larval immersion test, deltamethrin and amitraz resistance (larval mortality < 90% at the DD) was detected in all four and three of four R. (B.) microplus populations assessed, respectively. These data are critical for the design of an effective and sustainable tick control strategy on the communal farms.

202. Parasit Vectors. 2023 Aug 9;16(1):272. doi: 10.1186/s13071-023-05895-x.
Correction: Tick communities of cattle in smallholder rural livestock production systems in sub-Saharan Africa.
Heylen DJA(1)(2), Kumsa B(3), Kimbita E(4), Frank MN(5), Muhanguzi D(5), Jongejan F(6), Adehan SB(7), Toure A(8), Aboagye-Antwi F(9), Ogo NI(10), Julef

N(11), Fourie J(12), Evans A(13), Byaruhanga J(14), Madder M(13).
Author information:
(1)Evolutionary Ecology Group, Department of Biology, University of Antwerp, Wilrijk, Belgium. dieter.heylen@uantwerpen.be.
(2)Interuniversity Institute for Biostatistics and Statistical Bioinformatics, Hasselt University, Diepenbeek, Belgium. dieter.heylen@uantwerpen.be.
(3)Department of Parasitology, College of Veterinary Medicine and Agriculture, Addis Ababa University, Bishoftu, Ethiopia.
(4)Department of Veterinary Microbiology and Parasitology, College of Veterinary Medicine and Biomedical Sciences, Sokoine University of Agriculture, 3019, Morogoro, Tanzania.
(5)Department of Bio-molecular Resources and Bio-Laboratory Sciences (BBS), College of Veterinary Medicine, Makerere University, Kampala, Uganda.
(6)Department of Veterinary Tropical Diseases, Faculty of Veterinary Science, University of Pretoria, Onderstepoort, South Africa.
(7)Zootechnical, Veterinary and Halieutic Research Laboratory (LRZVH), National Institute of Agricultural Research (INRAB), 01 BP 884, Cotonou, Benin.
(8)Université Nangui Abrogoua, UFR Sciences de la Nature, 02 BP 801, Abidjan 02, Côte d'Ivoire.
(9)Department of Animal Biology and Conservation Science, School of Biological Sciences, College of Basic and Applied Sciences, University of Ghana, Legon-Accra, Ghana.
(10)National Veterinary Research Institute, Vom, Plateau State, Nigeria.
(11)Bill and Melinda Gates Foundation, Seattle, WA, USA.
(12)Clinvet International Pty (Ltd), 1479 Talmadge Hill South, Waverly, NY, 14892, USA.
(13)Clinglobal, B03/04, The Tamarin Commercial Hub, Jacaranda Avenue, Tamarin, 90903, Mauritius.
(14)Department of Veterinary Pharmacy, Clinics and Comparative Medicine, School of Veterinary Medicine and Animal Resources, Research Center for Tropical Diseases and Vector Control (RTC), College of Veterinary Medicine, Animal Resources and Biosecurity, Makerere University, Kampala, Uganda.
Erratum for
Parasit Vectors. 2023 Jun 19;16(1):206.

203. Pathogens. 2023 Oct 19;12(10):1258. doi: 10.3390/pathogens12101258.
Perception of Ticks and Tick-Borne Diseases Worldwide.

de la Fuente J(1)(2), Estrada-Peña A(3)(4), Rafael M(1), Almazán C(5), Bermúdez S(6), Abdelbaset AE(7), Kasaija PD(8), Kabi F(8), Akande FA(9), Ajagbe DO(10), Bamgbose T(11), Ghosh S(12)(13), Palavesam A(14), Hamid PH(15), Oskam CL(16)(17), Egan SL(16)(17), Duarte-Barbosa A(17)(18), Hekimoğlu O(19), Szabó MPJ(20), Labruna MB(21), Dahal A(22).
Author information:
(1)SaBio, Instituto de Investigación en Recursos Cinegéticos, IREC-CSIC-UCLM-JCCM, Ronda de Toledo 12, 13005 Ciudad Real, Spain.
(2)Department of Veterinary Pathobiology, Center for Veterinary Health Sciences,

Oklahoma State University, Stillwater, OK 74078, USA.
(3)Department of Animal Health, Faculty of Veterinary Medicine, University of Zaragoza, 50013 Zaragoza, Spain.
(4)Research Group in Emerging Zoonoses, Instituto Agroalimentario de Aragón-IA2, Universidad de Zaragoza-CITA, 50013 Zaragoza, Spain.
(5)Facultad de Ciencias Naturales, Universidad Autonóma de Querétaro, Avenida de las Ciencias S/N Juriquilla, Querétaro 76230, Mexico.
(6)Medical Entomology Research Department, Gorgas Memorial Institute for Health Research, Panama City 0816-02593, Panama.
(7)Laboratory of Parasitology, Graduate School of Infectious Diseases, Faculty of Veterinary Medicine, Hokkaido University, Kita-18, Nishi-9, Sapporo 060-0818, Hokkaido, Japan.
(8)National Livestock Resources Research Institute (NaLIRRI/NARO), Kampala P.O. Box 5704, Uganda.
(9)Department of Veterinary Parasitology and Entomology, College of Veterinary Medicine, Federal University of Agriculture, Abeokuta 111101, Ogun State, Nigeria.
(10)Department of Pure and Applied Zoology, College of Biological Sciences, Federal University of Agriculture, Abeokuta 111101, Ogun State, Nigeria.
(11)Department of Biological Sciences, Microbiology Unit, Faculty of Science, Kings University, Ode-Omu City 221102, Osun State, Nigeria.
(12)Entomology Laboratory, Indian Veterinary Research Institute, Izatnagar, Bareilly 243122, Uttar Pradesh, India.
(13)IVRI-Eastern Regional Station, 37, Belgachia Road, Kolkata 700037, West Bengal, India.
(14)Translational Research Platform for Veterinary Biologicals, Centre for Animal Health Studies, Tamil Nadu Veterinary and Animal Sciences University, Chennai 600051, Tamil Nadu, India.
(15)Department of Animal Science, Universitas Sebelas Maret, Surakarta 57126, Indonesia.
(16)School of Medical, Molecular and Forensic Sciences, Murdoch University, Perth, WA 6150, Australia.
(17)Centre for One Health and Biosecurity, Harry Butler Institute, Murdoch University, Perth, WA 6150, Australia.
(18)School of Veterinary Medicine, Murdoch University, Perth, WA 6150, Australia.
(19)Division of Ecology, Faculty of Science, Hacettepe University, Beytepe, Ankara 06800, Turkey.
(20)Laboratório de Ixodologia, Faculdade de Medicina Veterinária, Universidade Federal de Uberlândia, Av. Pará, 1720/Campus Umuarama-Bloco 2T, Uberlândia 38400-902, Brazil.
(21)Faculty of Veterinary Medicine and Animal Science, University of São Paulo, Sao Paulo 05508-220, Brazil.
(22)Department of Microbiology and Parasitology, Faculty of Animal Science, Veterinary Science and Fisheries, Agriculture and Forestry University, Chitwan 44200, Nepal.
In this comprehensive review study, we addressed the challenge posed by ticks

and tick-borne diseases (TBDs) with growing incidence affecting human and animal health worldwide. Data and perspectives were collected from different countries and regions worldwide, including America, Europe, Africa, Asia, and Oceania. The results updated the current situation with ticks and TBD and how it is perceived by society with information bias and gaps. The study reinforces the importance of multidisciplinary and international collaborations to advance in the surveillance, communication and proposed future directions to address these challenges.

204. J Parasitol Res. 2023 Oct 28;2023:7074041. doi: 10.1155/2023/7074041.
Ectoparasite Fauna of Rodents and Shrews with Their Spatial, Temporal, and Dispersal along a Degradation Gradient in Mabira Central Forest Reserve.
Babyesiza WS(1)(2)(3), Mpagi J(4), Ssuuna J(1)(2), Akoth S(1)(2)(3), Katakweba A(1)(5).
Author information:
(1)Africa Centre of Excellence for Innovative Rodent Pest Management and Biosensor Technology Development (ACE IRPM&BTD), Uganda.
(2)Department of Wildlife Management, Sokoine University of Agriculture, Morogoro, Tanzania.
(3)Department of Zoology, Entomology and Fisheries Science, Makerere University, Uganda.
(4)Department of Microbiology and Immunology, Busitema University, Uganda.
(5)Institute of Pest Management, Sokoine University of Agriculture, Morogoro, Tanzania.
Ectoparasites like fleas, mites, and ticks that are key carriers of harmful pathogens such as viruses, bacteria, cestodes, and nematodes live on rodents and shrews. It should be noted that rodents' ecological adaptability makes them suitable as parasite hosts. The main objective of the study was to determine the ectoparasite assemblages in rodents and shrews along a degradation gradient, while comparing infestation levels in different habitats with varying levels of degradation. The study was conducted in Mabira Central Forest Reserve. Ectoparasites were collected following rodent and shrew removal trapping which was done using Sherman's traps set along transects of 200 meters in three habitat strata that included adjacent forest habitats, degraded forest edge, and regenerating forest interior. Data was collected intermittently with a break every two months for one year from November 2018 to December 2019. A total of 1411 rodents and shrews were collected, yielding a total of 5692 ectoparasites from 22 host species (17 rodents and 5 shrews). The most prevalent group of ectoparasites was mites followed by fleas, lice, ticks, and earwig. Ectoparasite prevalence significantly differed depending on hosts species ($P = 0.001$) and host age ($P = 0.022$), but not host sex ($P = 0.78$), while mean infestation significantly varied basing on host species ($P = 0.001$), host sex ($P = 0.001$), season ($P = 0.001$), and habitat ($P = 0.001$). Prevalence ($P = 0.001$) and mean infestation ($P = 0.001$) significantly varied across studied habitats. The study has emphasized the significance of Praomys jacksoni and Hylomyscus stella as significant hosts for mites and S. congicus as a significant host for fleas. Additionally, environment and host characteristics have a bearing on prevalence

and infestation of ectoparasites with habitat degradation playing a significant role in the occurrence of ectoparasites, thereby emphasizing its contribution to zoonotic outbreaks.

205. PLoS One. 2023 Nov 9;18(11):e0288587. doi: 10.1371/journal.pone.0288587.
Seroepidemiological investigation of Crimean Congo hemorrhagic fever virus in livestock in Uganda, 2017.
Nyakarahuka L(1)(2), Kyondo J(1), Telford C(3), Whitesell A(3), Tumusiime A(1), Mulei S(1), Baluku J(1), Cossaboom CM(3), Cannon DL(3), Montgomery JM(3), Lutwama JJ(1), Nichol ST(3), Balinandi SK(1), Klena JD(3), Shoemaker TR(3).
Author information:
(1)Department of Arbovirology, Emerging and Reemerging Infectious Diseases, Uganda Virus Research Institute, Entebbe, Uganda.
(2)Department of Biosecurity, Ecosystems and Veterinary Public Health, College of Veterinary Medicine, Animal Resources and Biosecurity, Makerere University, Kampala, Uganda.
(3)Viral Special Pathogens Branch, Division of High-Consequence Pathogens and Pathology, United States Centers for Disease Control and Prevention, Atlanta, Georgia, United States of America.
Crimean-Congo Hemorrhagic fever (CCHF) is an important zoonotic disease transmitted to humans both by tick vectors and contact with fluids from an infected animal or human. Although animals are not symptomatic when infected, they are the main source of human infection. Uganda has reported sporadic human outbreaks of CCHF in various parts of the country since 2013. We designed a nationwide epidemiological study to investigate the burden of CCHF in livestock. A total of 3181 animals were sampled; 1732 cattle (54.4%), 1091 goats (34.3%), and 358 sheep (11.3%) resulting in overall livestock seropositivity of IgG antibodies against CCHF virus (CCHFV) of 31.4% (999/3181). Seropositivity in cattle was 16.9% and in sheep and goats was 48.8%. Adult and juvenile animals had higher seropositivity compared to recently born animals, and seropositivity was higher in female animals (33.5%) compared to male animals (24.1%). Local breeds had higher (36.8%) compared to exotic (2.8%) and cross breeds (19.3%). Animals that had a history of abortion or stillbirth had higher seropositivity compared to those without a history of abortion or stillbirth. CCHFV seropositivity appeared to be generally higher in northern districts of the country, though spatial trends among sampled districts were not examined. A multivariate regression analysis using a generalized linear mixed model showed that animal species, age, sex, region, and elevation were all significantly associated with CCHFV seropositivity after adjusting for the effects of other model predictors. This study shows that CCHFV is actively circulating in Uganda, posing a serious risk for human infection. The results from this study can be used to help target surveillance efforts for early case detection in animals and limit subsequent spillover into humans.

206. Parasit Vectors. 2023 Nov 16;16(1):423. doi: 10.1186/s13071-023-06034-2.
Publisher Correction: Tick communities of cattle in smallholder rural livestock production systems in sub-Saharan Africa.

Heylen DJA(1)(2), Kumsa B(3), Kimbita E(4), Frank MN(5), Muhanguzi D(5), Jongejan F(6), Adehan SB(7), Toure A(8), Aboagye-Antwi F(9), Ogo NI(10), Juleff N(11), Fourie J(12), Evans A(13), Byaruhanga J(14), Madder M(13).
Author information:
(1)Evolutionary Ecology Group, Department of Biology, University of Antwerp, Wilrijk, Belgium. dieter.heylen@uantwerpen.be.
(2)Interuniversity Institute for Biostatistics and Statistical Bioinformatics, Hasselt University, Diepenbeek, Belgium. dieter.heylen@uantwerpen.be.
(3)Department of Parasitology, College of Veterinary Medicine and Agriculture, Addis Ababa University, Bishoftu, Ethiopia.
(4)Department of Veterinary Microbiology and Parasitology, College of Veterinary Medicine and Biomedical Sciences, Sokoine University of Agriculture, 3019, Morogoro, Tanzania.
(5)Department of Bio-molecular Resources and Bio-Laboratory Sciences (BBS), College of Veterinary Medicine, Makerere University, Kampala, Uganda.
(6)Department of Veterinary Tropical Diseases, Faculty of Veterinary Science, University of Pretoria, Onderstepoort, South Africa.
(7)National Institute of Agricultural Research (INRAB), Zootechnical, Veterinary and Halieutic Research Laboratory (LRZVH), 01 BP 884, Cotonou, Benin.
(8)Universite Nangui Abrogoua, UFR Sciences de la Nature, 02 Bp 801, Abidjan 02, Côte d'Ivoire.
(9)Department of Animal Biology and Conservation Science, School of Biological Sciences, College of Basic and Applied Sciences, University of Ghana, Legon-Accra, Ghana.
(10)National Veterinary Research Institute, Vom, Plateau State, Nigeria.
(11)Bill & Melinda Gates Foundation, Seattle, WA, USA.
(12)Clinvet International Pty (Ltd), 1479 Talmadge Hill South, Waverly, NY, 14892, USA.
(13)Clinglobal, B03/04, The Tamarin Commercial Hub, Jacaranda Avenue, Tamarin, 90903, Mauritius.
(14)Research Center for Tropical Diseases and Vector Control (RTC), Department of Veterinary Pharmacy, Clinics and Comparative Medicine, School of Veterinary Medicine and Animal Resources, College of Veterinary Medicine, Animal Resources and Biosecurity, Makerere University, Kampala, Uganda.
Erratum for
Parasit Vectors. 2023 Jun 19;16(1):206.
DOI: 10.1186/s13071-023-06034-2

207. One Health. 2023 Jun 12;17:100576. doi: 10.1016/j.onehlt.2023.100576.
Spatial prediction of Crimean Congo hemorrhagic fever virus seroprevalence among livestock in Uganda.
Telford C(1)(2), Nyakarahuka L(3)(4), Waller L(5), Kitron U(5), Shoemaker T(1).
Author information:
(1)Viral Special Pathogens Branch, Centers for Disease Control and Prevention, 1600 Clifton Rd, Atlanta, GA 30333, USA.
(2)Gillings School of Global Public Health, University of North Carolina, 135 Dauer Dr, Chapel Hill, NC 27599, USA.

(3)Uganda Virus Research Institute, 51-59 Nakiwogo Road, Entebbe, Uganda.
(4)Department of Biosecurity, Ecosystems and Veterinary Public Health, Makerere University, Kampala Uganda, 7062 University Rd, Kampala, Uganda.
(5)Rollins School of Public Health, Emory University, 1518 Clifton Rd, Atlanta, GA 30322, USA.
Crimean-Congo Hemorrhagic Fever (CCHF) is a viral disease that can infect humans via contact with tick vectors or livestock reservoirs and can cause moderate to severe disease. The first human case of CCHF in Uganda was identified in 2013. To determine the geographic distribution of the CCHF virus (CCHFV), serosampling among herds of livestock was conducted in 28 Uganda districts in 2017. A geostatistical model of CCHF seroprevalence among livestock was developed to incorporate environmental and anthropogenic variables associated with elevated CCHF seroprevalence to predict CCHF seroprevalence on a map of Uganda and estimate the probability that CCHF seroprevalence exceeded 30% at each prediction location. Environmental and anthropogenic variables were also analyzed in separate models to determine the spatially varying drivers of prediction and determine which covariate class resulted in best prediction certainty. Covariates used in the full model included distance to the nearest croplands, average annual change in night-time light index, percent sand soil content, land surface temperature, and enhanced vegetation index. Elevated CCHF seroprevalence occurred in patches throughout the country, being highest in northern Uganda. Environmental covariates drove predicted seroprevalence in the full model more than anthropogenic covariates. Combination of environmental and anthropogenic variables resulted in the best prediction certainty. An understanding of the spatial distribution of CCHF across Uganda and the variables that drove predictions can be used to prioritize specific locations and activities to reduce the risk of future CCHF transmission.

208. Semin Nephrol. 2023 Dec 28:151466. doi: 10.1016/j.semnephrol.2023.151466. Online
Infections and Acute Kidney Injury: A Global Perspective.
Batte A(1), Shahrin L(2), Claure-Del Granado R(3), Luyckx VA(4), Conroy AL(5).
Author information:
(1)Child Health and Development Centre, Makerere University College of Health Sciences, Kampala, Uganda; Global Health Uganda, Kampala, Uganda. Electronic address: ttbatte@gmail.com.
(2)Clinical and Diagnostic Services, International Centre for Diarrhoeal Disease Research, Dhaka, Bangladesh.
(3)Division of Nephrology, Hospital Obrero No 2, Caja Nacional de Salud, Cochabamba, Bolivia; Instituto de Investigaciones Biomédicas e Investigación Social (IIBISMED), Facultad de Medicina, Universidad Mayor de San Simon, Cochabamba, Bolivia.
(4)Department of Public and Global Health, Epidemiology, Biostatistics and Prevention Institute, University of Zurich, Zurich, Switzerland; Renal Division, Brigham and Women's Hospital, Harvard Medical School, Boston, MA; Department of Paediatrics and Child Health, University of Cape Town, Cape Town, South Africa.
(5)Ryan White Center for Pediatric Infectious Diseases, Department of Pediatrics, Indiana University School of Medicine, Indianapolis, IN; Center for

Global Health, Indiana University School of Medicine, Indianapolis, IN.
Globally, there are an estimated 13.3 million cases of acute kidney injury (AKI) annually. Although infections are a common cause of AKI globally, most infection-associated AKI occurs in low- and lower-middle-income countries. There are marked differences in the etiology of infection-associated AKI across age groups, populations at risk, and geographic location. This article provides a global overview of different infections that are associated commonly with AKI, including severe acute respiratory syndrome coronavirus 2 (SARS-CoV-2), human immunodeficiency virus, malaria, dengue, leptospirosis, tick-borne illnesses, and viral hemorrhagic fevers. Further discussion focuses on infectious conditions associated with AKI including sepsis, diarrheal diseases and pregnancy, peripartum and neonatal AKI. This article also discusses the future of infection-associated AKI in the framework of climate change. It explores how increased investment in achieving the sustainable development goals may contribute to the International Society of Nephrology's 0 by 25 objective to curtail avoidable AKI-related fatalities by 2025.

209. Front Microbiol. 2023 Dec 13;14:1296522. doi: 10.3389/fmicb.2023.1296522.
Trypanocide usage in the cattle belt of southwestern Uganda.
Kasozi KI(1)(2), MacLeod ET(1), Sones KR(1)(3), Welburn SC(1)(4).
Author information:
(1)Infection Medicine, College of Medicine and Veterinary Medicine, Biomedical Sciences: Edinburgh Medical School, College of Medicine and Veterinary Medicine, The University of Edinburgh, Edinburgh, Scotland, United Kingdom.
(2)School of Medicine, Kabale University, Kabale, Uganda.
(3)Keith Sones Associates, Warkworth House, Warkworth, Banbury, United Kingdom.
(4)Zhejiang University - University of Edinburgh Institute, Zhejiang University School of Medicine, Haining, China.
BACKGROUND: Systematic infrastructure and regulatory weaknesses over many decades, in communities struggling with animal African trypanosomiasis (AAT) would be expected to create an environment that would promote drug misuse and risk development of drug resistance. Here, we explore rural community practices of livestock keepers, livestock extension officers and drug shop attendants to determine whether appropriate practice was being followed in administration of trypanocides and other drugs.
METHODS: A questionnaire-based survey was undertaken in southwestern Uganda in 2022 involving 451 farmers who kept cattle, sheep or goats and 79 "professionals" who were either livestock extension officers or drug shop attendants.
RESULTS: Respondents reported using one or more type of trypanocidal drug on 80.1% of the 451 farms in the last 30 days. Diminazene aceturate was used on around three-quarters of farms, while isometamidium chloride was used on around one-fifth. Homidium bromide was used on less than 1% of farms. Cattle were significantly more likely to be treated with trypanocides than sheep or goats.
On around two-thirds of farms, trypanocides were prepared and injected by farmers, with extension officers administering these drugs on most of the other third, especially on cattle farms. Almost all drugs were obtained from

privately-owned drug shops. For treatment of AAT with trypanocides, prescription-only medicines were routinely used by farmers without professional supervision and in the absence of a definitive diagnosis. While a far greater proportion of professionals had a better education and had received training on the use of trypanocides than farmers, there was relatively little difference in their ability to use these drugs correctly. Farmers were more likely than professionals to use only DA to treat trypanosomiasis and were more likely to use antibiotics as well as trypanocidal drugs to treat the animal. Furthermore, they estimated, on average, that twice the recommended dose of either diminazene aceturate or isometamidium chloride was needed to treat a hypothetical 400 kg bovine. A minority of both farmers and professionals reported that they observed the recommended withdrawal times following injection of trypanocidal drugs and very few of either group knew the recommended withdrawal times for milk or meat. Only one in six farmers reported using the sanative pair (alternating use of diminazene aceturate and isometamidium chloride), to reduce the risk of drug resistant trypanosome strains emerging, while this approach was more widely used by professionals. Farmers reported using antibiotics more commonly than the professionals, especially in sheep and goats, raising concerns as to overuse and misuse of this critical class of drugs. In addition to using trypanocides, most farmers also reported using a topical veterinary pesticide for the control of ticks and tsetse. On average, farmers spent 12.2% of their income from livestock sales on trypanocides.

CONCLUSION: This study highlights the complexity of issues involved in the fight against AAT using drug treatment. A multistakeholder campaign to increase awareness among farmers, drug shop attendants, and extension workers of the importance of adherence to recommended drug dosing, using the sanative pair and following recommended drug withdrawal guidance would promote best practice, reduce the risk of emergence of resistant strains of trypanosomes, and support enhanced food safety.

tickANDUgandaANDpathogenANDvaccine-set

1. Parasitology. 1995 Jul;111 (Pt 1):39-49. doi: 10.1017/s0031182000064581.
Generation and characterization of cloned Theileria parva parasites.
Morzaria SP(1), Dolan TT, Norval RA, Bishop RP, Spooner PR.
Author information:
(1)International Livestock Research Institute, Nairobi, Kenya.
A 3-step procedure for cloning Theileria parva parasites was developed. The first step involved the in vitro infection of a fixed number of bovine lymphocytes with titrated sporozoites. The cell lines obtained from infections initiated using sporozoite/lymphocyte ratios below 1:100 were then selected for cloning as these contained schizont-infected cells, each of which was derived from infection with a single sporozoite. In the second step, these cell lines were cloned by limiting dilution. As sporozoites infect lymphocytes and transform to induce clonal multiplication, this step produced infected cell lines containing both cloned parasites and cloned lymphocytes. In the third step, the cloned cell lines were used to infect cattle and isolation of the parasite in ticks was made during piroplasm parasitaemia. Finally, sporozoites were harvested from infected ticks and used for further characterization. Sporozoites derived from cloned cell lines of T. parva Muguga, Marikebuni, Boleni, Uganda and buffalo-derived 7014 were characterized using monoclonal antibody profiles, DNA restriction fragment length polymorphism detected using repetitive and telomeric probes, in vivo infectivity and, in one case, cross-immunity studies. Additionally, several distinct schizont-infected lymphocyte clones were isolated from the Muguga, Mariakani and buffalo-derived 7014 stocks. The combined results of the characterization revealed that the cloning procedure selected clones of T. parva from the parental stocks which were known to contain a mixture of genetically different parasite populations. The cloning method and the clones generated will be of value in studies of the biology of the parasite and in elucidating the strain specificity of immune responses in cattle.

2. Parassitologia. 1999 Sep;41 Suppl 1:73-80.
Development of sero-diagnostic and molecular tools for the control of important tick-borne pathogens of cattle in Africa.
Morzaria SP(1), Katende J, Musoke A, Nene V, Skilton R, Bishop R.
Author information:
(1)International Livestock Research Institute (ILRI), Nairobi, Kenya.
s.morzaria@cgiar.org
Tick-borne diseases (TBDs) are a major economic constraint to livestock production in sub-Saharan Africa. ILRI is focussing on developing a range of products, such as vaccines, diagnostics and decision support services to underpin improved control programmes against these diseases. We have developed three highly sensitive and specific enzyme linked immuno-assays (ELISAs), which allow precise diagnosis of Theileria parva, Babesia bigemina and Anaplasma marginale. These tests have been standardised and validated using defined experimental and field infection sera. Parasite specific recombinant antigens

and monoclonal antibodies against bovine immunoglobulins as secondary antibodies have played an important role in in enhancing the sensitivity and specificity of the assays. They have been further evaluated in on-farm longitudinal sero-epidemiological studies to define infection dynamics and disease risks in various farming systems in Kenya and Uganda. In addition, DNA-based tests for differentiation of Theileria species and characterisation of Theileria parva stocks have been developed. These tests have been derived through physical mapping and sequencing of key elements of the T. parva genome, which include repetitive and telomeric regions, minisatellite sequences, antigen genes and a number of random DNA sequences. These tools are currently being deployed in conjunction with field immunisation programmes to determine the biological impact of introducing live vaccines of T. parva on population dynamics.

3. Int J Parasitol. 2004 Apr;34(5):603-13. doi: 10.1016/j.ijpara.2003.12.012.
Application of a reverse line blot assay to the study of haemoparasites in cattle in Uganda.
Oura CA(1), Bishop RP, Wampande EM, Lubega GW, Tait A.
Author information:
(1)Department of Microbiology and Parasitology, Faculty of Vet Medicine, Makerere University, Kampala, Uganda. chrisoura@hotmail.com
Recent advances in genomic technology have focused many veterinary researchers on the possibility of producing one multivalent recombinant vaccine against all the haemoparasites that infect cattle in the tropics. Before such a vaccine is developed it is essential to define target cattle populations as well as the range of anti-pathogen vaccines required in order to control disease. To further this objective, we have evaluated a reverse line blot (RLB) assay, which simultaneously detects the principal tick transmitted protozoan and rickettsial cattle pathogens, in different epidemiological scenarios in Uganda. A critical question is the sensitivity, particularly in relation to detecting carrier animals. As Theileria parva is considered to be the most important pathogen in the region, we assessed the sensitivity of the RLB assay for T. parva and showed that 1-2 x 10(3) parasites per ml of blood could be detected-a level comparable with previously developed PCR methods and well below conventional microscopic detection. We applied the RLB assay to evaluate the differences in pathogen profiles between crossbred and indigenous cattle and show that there were different profiles, with a low prevalence of T. parva and Theileria taurotragi in the indigenous cattle compared to a high prevalence in the crossbred cattle. In contrast, we show higher prevalences of Theileria mutans and Theileria velifera in the indigenous compared to the crossbred cattle. Interestingly Anaplasma marginale, Babesia bovis and Babesia bigemina were of low prevalence but a high prevalence of Ehrlichia bovis was seen, raising the question of whether this rickettsial species could be pathogenic in cattle. Analysis of animals with clinical symptoms of East Coast Fever showed that, while T. parva is a major cause of these symptoms, T. mutans and possibly T. taurotragi and T. velifera, may also cause clinical disease. Overall, the results presented here highlight the complexity of tick-borne pathogen infections in cattle in Uganda.

4. Res Vet Sci. 2016 Dec;109:1-9. doi: 10.1016/j.rvsc.2016.09.001. Epub 2016 Sep 9.
Application of ethnobotanical repellents and acaricides in prevention, control and management of livestock ticks: A review.
Pavela R(1), Canale A(2), Mehlhorn H(3), Benelli G(4).
Author information:
(1)Crop Research Institute, Drnovska 507, 161 06 Prague 6, Czech Republic.
(2)Insect Behaviour Group, Department of Agriculture, Food and Environment, University of Pisa, via del Borghetto 80, 56124 Pisa, Italy.
(3)Department of Parasitology, Heinrich Heine University, Düsseldorf, Germany.
(4)Insect Behaviour Group, Department of Agriculture, Food and Environment, University of Pisa, via del Borghetto 80, 56124 Pisa, Italy. Electronic address: benelli.giovanni@gmail.com.
Ticks transmit at least the same number or even more pathogens than any other group of blood-feeding arthropods worldwide affecting humans and animals. The eco-friendly control and management of tick vectors in a constantly changing environment is a crucial challenge. Besides the development of vaccines against ticks, IPM practices aimed at reducing tick interactions with livestock, emerging pheromone-based control tools, and few biological control agents, the extensive employment of acaricides and tick repellents still remain the most effective and ready-to-use strategies. However, the former is limited by the development of growing resistances as well as environmental concerns. Exploiting plants and plant products as sources of effective tick repellents and acaricides represents a promising strategy. In this scenario, the preservation of ethnobotanical information on repellent and acaricidal potential of plants is crucial. Here, we evaluated relevant information published in recent years, focused on plants used as repellents and acaricides against tick vectors in different regions worldwide. We selected a total of 238 plant species, which are traditionally used against ticks by native and local communities of Africa (Kenya, Uganda, Zimbabwe, South Africa), Europe (Serbia, Macedonia, Romania), Asia (Pakistan, India) and America (Brazil, Canada), from 56 families. However, only 7 families (i.e. Asteraceae, Euphorbiaceae, Fabaceae, Lamiaceae, Meliaceae, Apocynaceae and Solanaceae) represent the major quote (46%) of all plant species. We evaluated the differences in acaricidal and repellent efficacy of different formulations used. In the final section, implications arising from the surveyed anti-tick ethnobotanical knowledge and challenges for its future are discussed.

5. Microbiol Mol Biol Rev. 2017 Feb 8;81(1):e00055-16. doi: 10.1128/MMBR.00055-16.
The Antigenic Structure of Zika Virus and Its Relation to Other Flaviviruses: Implications for Infection and Immunoprophylaxis.
Heinz FX(1), Stiasny K(2).
Author information:
(1)Department of Virology, Medical University of Vienna, Vienna, Austria franz.x.heinz@meduniwien.ac.at.
(2)Department of Virology, Medical University of Vienna, Vienna, Austria.
Zika virus was discovered ~70 years ago in Uganda and maintained a low profile as a human disease agent in Africa and Asia. Only recently has it caused

explosive outbreaks in previously unaffected regions, first in Oceania and then in the Americas since 2015. Of special concern is the newly identified link between congenital malformations (especially microcephaly) and Zika virus infections during pregnancy. At present, it is unclear whether Zika virus changed its pathogenicity or whether the huge number of infections allowed the recognition of a previously cryptic pathogenic property. The purpose of this review is to discuss recent data on the molecular antigenic structure of Zika virus in the context of antibody-mediated neutralization and antibody-dependent enhancement (ADE) of infection, a phenomenon that has been implicated in the development of severe disease caused by the related dengue viruses. Emphasis is given to epitopes of antibodies that potently neutralize Zika virus and also to epitopes that provide antigenic links to other important human-pathogenic flaviviruses such as dengue, yellow fever, West Nile, Japanese encephalitis, and tick-borne encephalitis viruses. The antigenic cross talk between Zika and dengue viruses appears to be of special importance, since they cocirculate in many regions of endemicity and sequential infections are likely to occur frequently. New insights into the molecular antigenic structure of Zika virus and flaviviruses in general have provided the foundation for great progress made in developing Zika virus vaccines and antibodies for passive immunization.

6. Front Cell Infect Microbiol. 2019 Mar 1;9:45. doi: 10.3389/fcimb.2019.00045.
Oral Vaccination With a Formulation Combining Rhipicephalus microplus Subolesin With Heat Inactivated Mycobacterium bovis Reduces Tick Infestations in Cattle.
Contreras M(1), Kasaija PD(1)(2), Merino O(3), de la Cruz-Hernandez NI(3), Gortazar C(1), de la Fuente J(1)(4).
Author information:
(1)SaBio, Instituto de Investigación en Recursos Cinegéticos IREC-CSIC-UCLM-JCCM, Ciudad Real, Spain.
(2)National Livestock Resources Research Institute (NaLIRRI/NARO), Tororo, Uganda.
(3)Facultad de Medicina Veterinaria y Zootecnia, Universidad Autónoma de Tamaulipas, Ciudad Victoria, Mexico.
(4)Department of Veterinary Pathobiology, Center for Veterinary Health Sciences, Oklahoma State University, Stillwater, OK, United States.
Vaccines are an environmentally friendly alternative to acaracides for the control of tick infestations, to reduce the risk for tick-borne diseases affecting human and animal health worldwide, and to improve animal welfare and production. Subolesin (SUB, also known as 4D8) is the functional homolog of Akirin2 involved in the regulation of development and innate immune response, and a proven protective antigen for the control of ectoparasite infestations and pathogen infection. Oral vaccination combining protein antigens with immunostimulants has proven efficacy with increased host welfare and safety, but has not been used for the control of tick infestations. Here we describe the efficacy of oral vaccination with a formulation combining Rhipicephalus microplus SUB and heat inactivated Mycobacterium bovis (IV) on cattle tick infestations and fertility. The levels of IgG antibody titers against SUB and M. bovis P22, and the expression of selected immune response genes were determined

and analyzed as possible correlates of protection. We demonstrated that oral immunization with the SUB+IV formulation resulted in 51% reduction in the number of female ticks and 30% reduction in fertility with an overall efficacy of 65% in the control of R. microplus infestations by considering the cumulative effect on reducing tick survival and fertility in cattle. The akr2, IL-1β, and C3 mRNA levels together with antibody levels against SUB correlated with vaccine efficacy. The effect of the oral immunization with SUB+IV in cattle on tick survival and fertility is essential to reduce tick infestations, and extended previous results on the effect of R. microplus SUB for the control of cattle tick infestations. These results support the development of oral vaccines formulations for the control of tick infestations and the incidence of tick-borne diseases.

7. Front Bioeng Biotechnol. 2019 Sep 26;7:236. doi: 10.3389/fbioe.2019.00236.
Identification of Ixodid Tick-Specific Aquaporin-1 Potential Anti-tick Vaccine Epitopes: An in-silico Analysis.
Ndekezi C(1), Nkamwesiga J(1), Ochwo S(1), Kimuda MP(1)(2), Mwiine FN(1), Tweyongyere R(3), Amanyire W(1), Muhanguzi D(1)(4).
Author information:
(1)School of Biosecurity, Biotechnical and Laboratory Science, College of Veterinary Medicine Animal Resources and Biosecurity, Makerere University, Kampala, Uganda.
(2)Research Unit in Bioinformatics, Department of Biochemistry and Microbiology, Rhodes University, Grahamstown, South Africa.
(3)School of Veterinary Medicine and Animal Resources, College of Veterinary Medicine Animal Resources and Biosecurity, Makerere University, Kampala, Uganda.
(4)Makerere University/Uganda Virus Research Institute Centre of Excellence in Infection and Immunity Research and Training, Entebbe, Uganda.
Ticks are arthropod vectors of pathogens of both Veterinary and Public health importance. Acaricide application, which is currently the mainstay of tick control, is hampered by high cost, the need for regular application and a selection of multi-acaricide resistant tick populations. In light of this, future tick control approaches are poised to rely on the integration of rational acaricide application and other methods, such as vaccination. To contribute to systematic research-guided efforts to produce anti-tick vaccines, we carried out an in-silico analysis of tick aquaporin-1 (AQP1) protein in order to identify tick-specific AQP1 peptide motifs that can be used in future peptide anti-tick vaccine development. We carried out multiple sequence alignment (MSA), motif analysis, homology modeling, and structural analysis to identify tick-specific AQP1 peptide motifs. BepiPred, Chou and Fasman-Turn, Karplus and Schulz Flexibility, and Parker-Hydrophilicity prediction models were used to predict these motifs' potential to induce B cell mediated immune responses. The tick AQP1 (GenBankID: QDO67142.1) protein was largely similar to the bovine AQP1 (PDB:1J4N) (23 % sequence similarity; Structural superimposition of the homology model and 14JN homotetramers gave an RMSD = 3.269 while superimposition of a single chain gave an RMSD = 1.475). Tick and bovine AQP1 transmembrane domains were largely similar while their extracellular and cytoplasmic domain loops

showed variation. Two tick-specific AQP1 peptide motifs; M7 (residues 106-125, p = 5.4e-25), and M8 (residues 85-104, p = 3.3e-24) were identified. These two motifs are located on the extra-cellular AQP1 domain. Motifs; M7 and M8 showed the highest Parker-Hydrophilicity prediction immunogenicity scores of 1.784 and 1.536, respectively. These two motifs can be a good starting point for the development of potential tick AQP1 peptide-based anti-tick vaccines. Further analyses such as molecular dynamics, in vitro assays, and in vivo immunization assays are required to validate these findings.

8. J Virol. 2020 Mar 31;94(8):e02005-19. doi: 10.1128/JVI.02005-19. Print 2020 Mar
Structure and Characterization of Crimean-Congo Hemorrhagic Fever Virus GP38.
Mishra AK(1), Moyer CL(2), Abelson DM(2), Deer DJ(3), El Omari K(4), Duman R(4), Lobel L(5), Lutwama JJ(6), Dye JM(7), Wagner A(4), Chandran K(8), Cross RW(3), Geisbert TW(3), Zeitlin L(2), Bornholdt ZA(2), McLellan JS(9).
Author information:
(1)Department of Molecular Biosciences, University of Texas at Austin, Austin, Texas, USA.
(2)Mapp Biopharmaceutical, San Diego, California, USA.
(3)Department of Microbiology and Immunology, University of Texas Medical Branch, Galveston, Texas, USA.
(4)Diamond Light Source, Harwell Science and Innovation Campus, Didcot, Oxfordshire, United Kingdom.
(5)Department of Microbiology, Immunology and Genetics, Faculty of Health Sciences, Ben-Gurion University of the Negev, Beer-Sheva, Israel.
(6)Department of Arbovirology, Emerging and Re-emerging Infection, Uganda Virus Research Institute, Entebbe, Uganda.
(7)US Army Medical Research Institute of Infectious Diseases, Fort Detrick, Maryland, USA.
(8)Department of Microbiology and Immunology, Albert Einstein College of Medicine, Bronx, New York, USA.
(9)Department of Molecular Biosciences, University of Texas at Austin, Austin, Texas, USA jmclellan@austin.utexas.edu.
Crimean-Congo hemorrhagic fever virus (CCHFV) is the causative agent of the most widespread tick-borne viral infection in humans. CCHFV encodes a secreted glycoprotein (GP38) of unknown function that is the target of a protective antibody. Here, we present the crystal structure of GP38 at a resolution of 2.5 Å, which revealed a novel fold primarily consisting of a 3-helix bundle and a β-sandwich. Sequence alignment and homology modeling showed distant homology between GP38 and the ectodomain of Gn (a structural glycoprotein in CCHFV), suggestive of a gene duplication event. Analysis of convalescent-phase sera showed high titers of GP38 antibodies indicating immunogenicity in humans during natural CCHFV infection. The only protective antibody for CCHFV in an adult mouse model reported to date, 13G8, bound GP38 with subnanomolar affinity and protected against heterologous CCHFV challenge in a STAT1-knockout mouse model. Our data strongly suggest that GP38 should be evaluated as a vaccine antigen and that its structure provides a foundation to investigate functions of this protein in the viral life cycle.IMPORTANCE Crimean-Congo hemorrhagic fever virus

(CCHFV) is a priority pathogen that poses a high risk to public health. Due to the high morbidity and mortality rates associated with CCHFV infection, there is an urgent need to develop medical countermeasures for disease prevention and treatment. CCHFV GP38, a secreted glycoprotein of unknown function unique to the Nairoviridae family, was recently shown to be the target of a protective antibody against CCHFV. Here, we present the crystal structure of GP38, which revealed a novel fold with distant homology to another CCHFV glycoprotein that is suggestive of a gene duplication event. We also demonstrate that antibody 13G8 protects STAT1-knockout mice against heterologous CCHFV challenge using a clinical isolate from regions where CCHFV is endemic. Collectively, these data advance our understanding of GP38 structure and antigenicity and should facilitate future studies investigating its function.

9. Vaccines (Basel). 2020 Jun 18;8(2):319. doi: 10.3390/vaccines8020319.
Vaccination with Recombinant Subolesin Antigens Provides Cross-Tick Species Protection in Bos indicus and Crossbred Cattle in Uganda.
Kasaija PD(1)(2), Contreras M(1)(3), Kabi F(2), Mugerwa S(2), de la Fuente J(1)(4).
Author information:
(1)SaBio, Instituto de Investigación en Recursos Cinegéticos (IREC), Consejo Superior de Investigaciones Científicas (CSIC), Universidad de Castilla-La Mancha (UCLM)-Junta de Comunidades de Castilla-La Mancha (JCCM), Ronda de Toledo s/n, 13005 Ciudad Real, Spain.
(2)National Livestock Resources Research Institute (NaLIRRI/NARO), P.O. Box 5704 Kampala, Uganda.
(3)Interdisciplinary Laboratory of Clinical Analysis, Interlab-UMU, Regional Campus of International Excellence Campus Mare Nostrum, University of Murcia, Espinardo, 30100 Murcia, Spain.
(4)Department of Veterinary Pathobiology, Center for Veterinary Health Sciences, Oklahoma State University, Stillwater, OK 74078, USA.
Cattle tick infestations and transmitted pathogens affect animal health, production and welfare with an impact on cattle industry in tropical and subtropical countries. Anti-tick vaccines constitute an effective and sustainable alternative to the traditional methods for the control of tick infestations. Subolesin (SUB)-based vaccines have shown efficacy for the control of multiple tick species, but several factors affect the development of new and more effective vaccines for the control of tick infestations. To address this challenge, herein we used a regional and host/tick species driven approach for vaccine design and implementation. The objective of the study was to develop SUB-based vaccines for the control of the most important tick species (Rhipicephalus appendiculatus, R. decoloratus and Amblyomma variegatum) affecting production of common cattle breeds (Bos indicus and B. indicus x B. taurus crossbred) in Uganda. In this way, we addressed the development of anti-tick vaccines as an intervention to prevent the economic losses caused by ticks and tick-borne diseases in the cattle industry in Uganda. The results showed the possibility of using SUB antigens for the control of multiple tick species in B. indicus and crossbred cattle and suggested the use of R.

appendiculatus SUB to continue research on vaccine design and formulation for the control of cattle ticks in Uganda. Future directions would include quantum vaccinology approaches based on the characterization of the SUB protective epitopes, modeling of the vaccine E under Ugandan ecological and epidemiological conditions and optimization of vaccine formulation including the possibility of oral administration.

10. Ticks Tick Borne Dis. 2021 Sep;12(5):101756. doi: 10.1016/j.ttbdis.2021.101756.
Cattle ticks and tick-borne diseases: a review of Uganda's situation.
Kasaija PD(1), Estrada-Peña A(2), Contreras M(3), Kirunda H(4), de la Fuente J(5).
Author information:
(1)SaBio, Instituto de Investigación en Recursos Cinegéticos (IREC), Consejo Superior de Investigaciones Científicas (CSIC), Universidad de Castilla-La Mancha (UCLM)-Junta de Comunidades de Castilla-La Mancha (JCCM), Ronda de Toledo s/n, 13005 Ciudad Real, Spain; National Livestock Resources Research Institute (NaLIRRI/NARO), P.O. Box 5704, Kampala, Uganda.
(2)Department of Animal Pathology, Faculty of Veterinary Medicine, 50013 Zaragoza, Spain.
(3)SaBio, Instituto de Investigación en Recursos Cinegéticos (IREC), Consejo Superior de Investigaciones Científicas (CSIC), Universidad de Castilla-La Mancha (UCLM)-Junta de Comunidades de Castilla-La Mancha (JCCM), Ronda de Toledo s/n, 13005 Ciudad Real, Spain; Interdisciplinary Laboratory of Clinical Analysis, Interlab-UMU, Regional Campus of International Excellence Campus Mare Nostrum, University of Murcia, Espinardo, 30100 Murcia, Spain.
(4)National Livestock Resources Research Institute (NaLIRRI/NARO), P.O. Box 5704, Kampala, Uganda.
(5)SaBio, Instituto de Investigación en Recursos Cinegéticos (IREC), Consejo Superior de Investigaciones Científicas (CSIC), Universidad de Castilla-La Mancha (UCLM)-Junta de Comunidades de Castilla-La Mancha (JCCM), Ronda de Toledo s/n, 13005 Ciudad Real, Spain; Department of Veterinary Pathobiology, Center for Veterinary Health Sciences, Oklahoma State University, Stillwater, OK 74078, USA. Electronic address: josedejesus.fuente@uclm.es.
Herein we review the epidemiology of ticks and tick-borne diseases (TTBDs), their impact on livestock health and on the economy, control and associated challenges in Uganda. Ticks are leading vectors of economically important pathogens and are widespread in Uganda due to suitable climatic conditions. Besides the physical injury inflicted on the animal host, ticks transmit a number of pathogens that can cause morbidity and mortality of livestock if untreated, resulting in economic losses. Uganda suffers an aggregated annual loss (direct and indirect) of over USD 1.1 billion in the TTBDs complex. East Coast fever (ECF) caused by a protozoan haemoparasite, Theileria parva, is the most prevalent and economically important tick-borne disease (TBD) in Uganda and its vector, the brown ear tick (Rhipicephalus appendiculatus) widely distributed. Other prevalent TBDs in Uganda include anaplasmosis, babesiosis and heartwater. We highlight the role of agro-ecological zones (AEZs) and livestock management system in the distribution of TTBDs, citing warm and humid lowlands

as being ideal habitats for ticks and endemic for TBDs. Control of TTBDs is a matter of great importance as far as animal health is concerned in Uganda. Indigenous cattle, which make up over 90% of the national herd are known to be more tolerant to TTBDs and most farms rely on endemic stability to TBDs for control. However, exotic cattle breeds are more capital intensive than indigenous breeds, but the increasing adoption of tick-susceptible exotic cattle breeds (especially dairy) in western and central Uganda demands intensive use of acaricides for tick control and prevention of TBDs. Such acaricide pressure has unfortunately led to selection of acaricide-resistant tick populations and the consequent acaricide resistance observed in the field. Vaccination against ECF, selective breeding for tick resistance and integrated tick control approaches that limit tick exposure, could be adopted to interrupt spread of acaricide resistance. We recommend increasing monitoring and surveillance for TTBDs and for emerging acaricide resistance, improved extension services and sensitization of farmers on tick control measures, appropriate acaricide use and the development and implementation of vaccines for the control of TTBDs as more sustainable and effective interventions. A tick control policy should be developed, taking into account variations of agro-ecological zones, farm circumstances and indigenous technical knowledge, and this should be incorporated into the overall animal health program.

11. Vaccines (Basel). 2021 Oct 15;9(10):1185. doi: 10.3390/vaccines9101185.
From Bench to Field: A Guide to Formulating and Evaluating Anti-Tick Vaccines Delving beyond Efficacy to Effectiveness.
Ndawula C Jr(1)(2).
Author information:
(1)National Agricultural Research Organization, P.O. Box 295, Entebbe, Wakiso 256, Uganda.
(2)National Livestock Resources Research Institute, Vaccinology Research Programme, P.O. Box 5704, Nakyesasa, Wakiso 256, Uganda.
Ticks are ubiquitous blood-sucking ectoparasites capable of transmitting a wide range of pathogens such as bacteria, viruses, protozoa, and fungi to animals and humans. Although the use of chemicals (acaricides) is the predominant method of tick-control, there are increasing incidents of acaricide tick resistance. Furthermore, there are concerns over accumulation of acaricide residues in meat, milk and in the environment. Therefore, alternative methods of tick-control have been proposed, of which anti-tick cattle vaccination is regarded as sustainable and user-friendly. Over the years, tremendous progress has been made in identifying and evaluating novel candidate tick vaccines, yet none of them have reached the global market. Until now, Bm86-based vaccines (Gavac™ in Cuba and TickGARDPLUS™ Australia-ceased in 2010) are still the only globally commercialized anti-tick vaccines. In contrast to Bm86, often, the novel candidate anti-tick vaccines show a lower protection efficacy. Why is this so? In response, herein, the potential bottlenecks to formulating efficacious anti-tick vaccines are examined. Aside from Bm86, the effectiveness of other anti-tick vaccines is rarely assessed. So, how can the researchers assess anti-tick vaccine effectiveness before field application? The approaches that

are currently used to determine anti-tick vaccine efficacy are re-examined in this review. In addition, a model is proposed to aid in assessing anti-tick vaccine effectiveness. Finally, based on the principles for the development of general veterinary vaccines, a pipeline is proposed to guide in the development of anti-tick vaccines.

12. Methods Mol Biol. 2022;2411:253-267. doi: 10.1007/978-1-0716-1888-2_15.

Anti-Tick Vaccines: Current Advances and Future Prospects.
Muhanguzi D(1), Ndekezi C(2), Nkamwesiga J(2), Kalayou S(3), Ochwo S(2), Vuyani M(4), Kimuda MP(2).
Author information:
(1)School of Biosecurity, Biotechnical and Laboratory Science, College of Veterinary Medicine Animal Resources and Biosecurity, Makerere University, Kampala, Uganda. dennis.muhanguzi@mak.ac.ug.
(2)School of Biosecurity, Biotechnical and Laboratory Science, College of Veterinary Medicine Animal Resources and Biosecurity, Makerere University, Kampala, Uganda.
(3)International Centre of Insect Physiology and Ecology (ICIPE), Nairobi, Kenya.
(4)Research Unit in Bioinformatics (RUBi), Department of Biochemistry and Microbiology, Rhodes University, Grahamstown, South Africa.
Ticks are increasingly a global public health and veterinary concern. They transmit numerous pathogens that are of veterinary and public health importance. Acaricides, livestock breeding for tick resistance, tick handpicking, pasture spelling, and anti-tick vaccines (ATVs) are in use for the control of ticks and tick-borne diseases (TTBDs); acaricides and ATVs being the most and least used TTBD control methods respectively. The overuse and misuse of acaricides has inadvertently selected for tick strains that are resistant to acaricides. Furthermore, vaccines are rare and not commercially available in sub-Saharan Africa (SSA). It doesn't help that many of the other methods are labor-intensive and found impractical especially for larger farm operations. The success of TTBD control is therefore dependent on integrating all the currently available methods. Vaccines have been shown to be cheap and effective. However, their large-scale deployment for TTBD control in SSA is hindered by commercial unavailability of efficacious anti-tick vaccines against sub-Saharan African tick strains. Thanks to advances in genomics, transcriptomics, and proteomics technologies, many promising anti-tick vaccine antigens (ATVA) have been identified. However, few of them have been investigated for their potential as ATV candidates. Reverse vaccinology (RV) can be leveraged to accelerate ATV discovery. It is cheap and shortens the lead time from ATVA discovery to vaccine production. This chapter provides a brief overview of recent advances in ATV development, ATVs, ATV effector mechanisms, and anti-tick RV. Additionally, it provides a detailed outline of vaccine antigen selection and analysis using computational methods.

13. Pathogens. 2022 Jan 19;11(2):114. doi: 10.3390/pathogens11020114.

Sequence Diversity of Tp1 and Tp2 Antigens and Population Genetic Analysis of Theileria parva in Unvaccinated Cattle in Zambia's Chongwe and Chisamba Districts.

Muleya W(1), Atuhaire DK(2), Mupila Z(1), Mbao V(3), Mayembe P(4), Kalenga S(4), Fandamu P(4), Namangala B(5), Salt J(6), Musoke AJ(7).
Author information:
(1)Department of Biomedical Sciences, School of Veterinary Medicine, University of Zambia, Lusaka 10101, Zambia.
(2)Centre for Ticks and Tick-Borne Diseases, Lilongwe, Malawi.
(3)Eastern and Southern Africa Regional Office, International Development Research Centre, Nairobi 00200, Kenya.
(4)Department of Veterinary Services, Ministry of Fisheries and Livestock, Lusaka 50060, Zambia.
(5)Department of Paraclinical Studies, School of Veterinary Medicine, University of Zambia, Lusaka 10101, Zambia.
(6)Global Alliance for Livestock Veterinary Medicines, Pentlands Science Park, Bush Loan, Penicuik EH26 0PZ, UK.
(7)LMK Medical Laboratories and Consultancies, Kampala P.O. Box 33686, Uganda.

East Coast Fever (ECF), caused by Theileria parva, is a major constraint to improved livestock keeping in east and central Africa, including Zambia. To understand the dynamics and determine the candidates for immunization in Zambia's Chongwe and Chisamba districts, a combination of Tp1 and Tp2 gene sequencing and microsatellite analysis using nine markers was conducted from which an abundance of Muguga, Kiambu, Serengeti and Katete epitopes in the field samples was obtained. Phylogenetic analysis showed six (Tp1) and three (Tp2) clusters with an absence of geographical origin clustering. The majority of haplotypes were related to Muguga, Kiambu, Serengeti and Katete, and only a few were related to Chitongo. Both antigens showed purifying selection with an absence of positive selection sites. Furthermore, low to moderate genetic differentiation was observed among and within the populations, and when vaccine stocks were compared with field samples, Chongwe samples showed more similarity to Katete and less to Chitongo, while Chisamba samples showed similarity to both Katete and Chitongo and not to Muguga, Kiambu or Serengeti. We conclude that the use of Katete stock for immunization trials in both Chongwe and Chisamba districts might produce desirable protection against ECF.

14. Vaccine. 2022 Jul 30;40(32):4564-4573. doi: 10.1016/j.vaccine.2022.06.036. Epub
Oral vaccine formulation combining tick Subolesin with heat inactivated mycobacteria provides control of cross-species cattle tick infestations.

Kasaija PD(1), Contreras M(2), Kabi F(3), Mugerwa S(3), Garrido JM(4), Gortazar C(2), de la Fuente J(5).
Author information:
(1)SaBio, Instituto de Investigación en Recursos Cinegéticos IREC-CSIC-UCLM-JCCM, Ronda de Toledo s/n, 13005 Ciudad Real, Spain; National Livestock Resources Research Institute (NaLIRRI/NARO), P.O. Box 5704, Wakiso District, Uganda.
(2)SaBio, Instituto de Investigación en Recursos Cinegéticos

IREC-CSIC-UCLM-JCCM, Ronda de Toledo s/n, 13005 Ciudad Real, Spain.
(3)National Livestock Resources Research Institute (NaLIRRI/NARO), P.O. Box 5704, Wakiso District, Uganda.
(4)NEIKER-Instituto Vasco de Investigación y Desarrollo Agrario, Animal Health Department, Bizkaia Science and Technology Park 812L, 48160 Derio (Bizkaia), Spain.
(5)SaBio, Instituto de Investigación en Recursos Cinegéticos IREC-CSIC-UCLM-JCCM, Ronda de Toledo s/n, 13005 Ciudad Real, Spain; Department of Veterinary Pathobiology, Center for Veterinary Health Sciences, Oklahoma State University, Stillwater, OK 74078, USA. Electronic address: jose_delafuente@yahoo.com.
Tick vaccines are necessary as part of a One Health approach for the control of tick infestations and tick-borne diseases. Subolesin (SUB, also known as 4D8) is a tick protective antigen that has shown efficacy in vaccine formulations for the control of ectoparasite infestations and pathogen infection/transmission. A recent proof-of-concept study reported oral vaccination combining Rhipicephalus microplus SUB with heat inactivated Mycobacterium bovis (IV) as an immunostimulant for the control of cattle tick infestations. Based on the efficacy of Rhipicephalus decoloratus SUB for the control of multiple cattle tick species in Uganda, herein we design a controlled pen trial using an oral formulation combining R. decoloratus SUB with IV for the control of R. decoloratus and Rhipicephalus appendiculatus cattle tick infestations. Vaccine efficacy (E) of SUB + IV on tick life cycle was compared with IV and SUB alone and with PBS as control. The IgG antibody titers against SUB and M. bovis P22 and the serum levels of selected protein immune biomarkers (IL-1beta, TNF-alpha, C3) were determined and analyzed as possible correlates of protection. Oral immunization with IV and SUB alone and in SUB + IV combination were effective for the control of tick infestations (E = 71-96% for R. decoloratus and 87-99% for R. appendiculatus) with highest E (higher than 95%) for SUB + IV. The results demonstrated that oral immunization with the SUB + IV formulation resulted in effective control of cattle tick infestations through the activation of multiple immune mechanisms. These results support the application of oral vaccine formulations with SUB + IV for the control of cattle infestations with Rhipicephalus species towards improving animal health.

15. Vaccines (Basel). 2022 Dec 31;11(1):99. doi: 10.3390/vaccines11010099.
Inspiring Anti-Tick Vaccine Research, Development and Deployment in Tropical Africa for the Control of Cattle Ticks: Review and Insights.
Kasaija PD(1)(2), Contreras M(1), Kirunda H(3), Nanteza A(4), Kabi F(2), Mugerwa S(2), de la Fuente J(1)(5).
Author information:
(1)SaBio, Instituto de Investigación en Recursos Cinegéticos (IREC), Consejo Superior de Investigaciones Científicas (CSIC), Universidad de Castilla-La Mancha (UCLM)-Junta de Comunidades de Castilla-La Mancha (JCCM), Ronda de Toledo s/n, 13005 Ciudad Real, Spain.
(2)National Livestock Resources Research Institute (NaLIRRI), Kampala P.O. Box 5704, Uganda.

(3)National Agricultural Research Organisation (NARO), Mbarara ZARDI, Mbarara P.O. Box 389, Uganda.
(4)College of Veterinary Medicine, Animal Resources and Biosecurity, Makerere University, Kampala P.O. Box 7062, Uganda.
(5)Department of Veterinary Pathobiology, Center for Veterinary Health Sciences, Oklahoma State University, Stillwater, OK 74078, USA.
Ticks are worldwide ectoparasites to humans and animals, and are associated with numerous health and economic effects. Threatening over 80% of the global cattle population, tick and tick-borne diseases (TTBDs) particularly constrain livestock production in the East, Central and Southern Africa. This, therefore, makes their control critical to the sustainability of the animal industry in the region. Since ticks are developing resistance against acaricides, anti-tick vaccines (ATVs) have been proposed as an environmentally friendly control alternative. Whereas they have been used in Latin America and Australia to reduce tick populations, pathogenic infections and number of acaricide treatments, commercially registered ATVs have not been adopted in tropical Africa for tick control. This is majorly due to their limited protection against economically important tick species of Africa and lack of research. Recent advances in various omics technologies and reverse vaccinology have enabled the identification of many candidate anti-tick antigens (ATAs), and are likely to usher in the next generation of vaccines, for which Africa should prepare to embrace. Herein, we highlight some scientific principles and approaches that have been used to identify ATAs, outline characteristics of a desirable ATA for vaccine design and propose the need for African governments to investment in ATV research to develop vaccines relevant to local tick species (personalized vaccines). We have also discussed the prospect of incorporating anti-tick vaccines into the integrated TTBDs control strategies in the sub-Saharan Africa, citing the case of Uganda.

16. Pathogens. 2023 Feb 10;12(2):295. doi: 10.3390/pathogens12020295.
East Coast Fever Carrier Status and Theileria parva Breakthrough Strains in Recently ITM Vaccinated and Non-Vaccinated Cattle in Iganga District, Eastern Uganda.
Oligo S(1), Nanteza A(1), Nsubuga J(1), Musoba A(1), Kazibwe A(1), Lubega GW(1).
Author information:
(1)College of Veterinary Medicine, Animal Resources and Biosecurity (COVAB), Makerere University, Kampala P.O. Box 7062, Uganda.
East Coast fever (ECF) is a tick-borne disease of cattle that hinders the development of the livestock industry in eastern, central and southern Africa. The 'Muguga cocktail' live vaccine, delivered by an infection and treatment method (ITM), remains the only immunisation strategy of controlling ECF. However, there are challenges of the live vaccine inducing ECF carrier status in immunised animals and the possibility of lack of protection from parasite strains that are antigenically different from the vaccine strains. In Uganda, there are insufficient data regarding the ECF carrier status and T. parva genetic diversity in vaccinated and associated non-vaccinated cattle to assess the effectiveness of ITM vaccination. Blood was collected from recently ECF

vaccinated (98) and non-vaccinated (73) cattle from Iganga district in Eastern Uganda at 120 days post-vaccination. The p104 gene nested PCR was used to screen for T. parva DNA, 11 minisatellite and 3 microsatellite markers (SSR) were used for genotyping. Two minisatellite markers (MS7 and MS19) were used to determine whether ECF carrier status was due to the T. parva vaccine or local strains. The prevalence of T. parva based on p104 nPCR was 61.2% (60/98) (RR 2.234, 95% CI 1.49-3.35, p-value < 0.001) among recently vaccinated cattle and 27.4% (20/73) (RR 1.00) among associated non-vaccinated cattle. The Muguga cocktail vaccine strains were responsible for carrier status in 10 (58.8%) by MS7 and 11 (64.7%) by MS19 in vaccinated cattle. Genotypes of T. parva with different-sized alleles to the vaccine strains that could be potential 'breakthroughs' were detected in 2 (11.8%)) and 4 (23.5%) isolates from vaccinated cattle based on MS7 and MS19 minisatellite markers, respectively. Using 14 SSR markers, T. parva diversity was higher in vaccinated (Na = 2.214, Ne = 1.978, He = 0.465) than associated non-vaccinated (Na = 1.071, Ne = 1.048, He = 0.259) cattle. The principal component analysis (PCA) showed isolates from vaccinated cattle were closely related to those from non-vaccinated cattle. The analysis of molecular variance (AMOVA) revealed high genetic variation (96%) within T. parva isolates from vaccinated and non-vaccinated cattle but low variation (4%) between vaccinated and non-vaccinated cattle. This study reveals the role of ITM in inducing the carrier status and higher T. parva genetic diversity in vaccinated cattle. The low genetic variation between T. parva isolates in both vaccinated and non-vaccinated cattle may be suggestive of the protective role of vaccine strains against genetically related local strains in the study area.

17. Pathogens. 2023 Oct 19;12(10):1258. doi: 10.3390/pathogens12101258.
Perception of Ticks and Tick-Borne Diseases Worldwide.
de la Fuente J(1)(2), Estrada-Peña A(3)(4), Rafael M(1), Almazán C(5), Bermúdez S(6), Abdelbaset AE(7), Kasaija PD(8), Kabi F(8), Akande FA(9), Ajagbe DO(10), Bamgbose T(11), Ghosh S(12)(13), Palavesam A(14), Hamid PH(15), Oskam CL(16)(17), Egan SL(16)(17), Duarte-Barbosa A(17)(18), Hekimoğlu O(19), Szabó MPJ(20), Labruna MB(21), Dahal A(22).
Author information:
(1)SaBio, Instituto de Investigación en Recursos Cinegéticos, IREC-CSIC-UCLM-JCCM, Ronda de Toledo 12, 13005 Ciudad Real, Spain.
(2)Department of Veterinary Pathobiology, Center for Veterinary Health Sciences, Oklahoma State University, Stillwater, OK 74078, USA.
(3)Department of Animal Health, Faculty of Veterinary Medicine, University of Zaragoza, 50013 Zaragoza, Spain.
(4)Research Group in Emerging Zoonoses, Instituto Agroalimentario de Aragón-IA2, Universidad de Zaragoza-CITA, 50013 Zaragoza, Spain.
(5)Facultad de Ciencias Naturales, Universidad Autonóma de Querétaro, Avenida de las Ciencias S/N Juriquilla, Querétaro 76230, Mexico.
(6)Medical Entomology Research Department, Gorgas Memorial Institute for Health Research, Panama City 0816-02593, Panama.
(7)Laboratory of Parasitology, Graduate School of Infectious Diseases, Faculty of Veterinary Medicine, Hokkaido University, Kita-18, Nishi-9, Sapporo 060-0818,

Hokkaido, Japan.
(8)National Livestock Resources Research Institute (NaLIRRI/NARO), Kampala P.O. Box 5704, Uganda.
(9)Department of Veterinary Parasitology and Entomology, College of Veterinary Medicine, Federal University of Agriculture, Abeokuta 111101, Ogun State, Nigeria.
(10)Department of Pure and Applied Zoology, College of Biological Sciences, Federal University of Agriculture, Abeokuta 111101, Ogun State, Nigeria.
(11)Department of Biological Sciences, Microbiology Unit, Faculty of Science, Kings University, Ode-Omu City 221102, Osun State, Nigeria.
(12)Entomology Laboratory, Indian Veterinary Research Institute, Izatnagar, Bareilly 243122, Uttar Pradesh, India.
(13)IVRI-Eastern Regional Station, 37, Belgachia Road, Kolkata 700037, West Bengal, India.
(14)Translational Research Platform for Veterinary Biologicals, Centre for Animal Health Studies, Tamil Nadu Veterinary and Animal Sciences University, Chennai 600051, Tamil Nadu, India.
(15)Department of Animal Science, Universitas Sebelas Maret, Surakarta 57126, Indonesia.
(16)School of Medical, Molecular and Forensic Sciences, Murdoch University, Perth, WA 6150, Australia.
(17)Centre for One Health and Biosecurity, Harry Butler Institute, Murdoch University, Perth, WA 6150, Australia.
(18)School of Veterinary Medicine, Murdoch University, Perth, WA 6150, Australia.
(19)Division of Ecology, Faculty of Science, Hacettepe University, Beytepe, Ankara 06800, Turkey.
(20)Laboratório de Ixodologia, Faculdade de Medicina Veterinária, Universidade Federal de Uberlândia, Av. Pará, 1720/Campus Umuarama-Bloco 2T, Uberlândia 38400-902, Brazil.
(21)Faculty of Veterinary Medicine and Animal Science, University of São Paulo, Sao Paulo 05508-220, Brazil.
(22)Department of Microbiology and Parasitology, Faculty of Animal Science, Veterinary Science and Fisheries, Agriculture and Forestry University, Chitwan 44200, Nepal.
In this comprehensive review study, we addressed the challenge posed by ticks and tick-borne diseases (TBDs) with growing incidence affecting human and animal health worldwide. Data and perspectives were collected from different countries and regions worldwide, including America, Europe, Africa, Asia, and Oceania. The results updated the current situation with ticks and TBD and how it is perceived by society with information bias and gaps. The study reinforces the importance of multidisciplinary and international collaborations to advance in the surveillance, communication and proposed future directions to address these challenges.

tickANDUgandaANDpathogen-set

1. Am J Trop Med Hyg. 1970 Nov;19(6):Suppl:1141-3.
Congo (CON) strain. 3011.
[No authors listed]

2. Parasitology. 1995 Jul;111 (Pt 1):39-49. doi: 10.1017/s0031182000064581.
Generation and characterization of cloned Theileria parva parasites.
Morzaria SP(1), Dolan TT, Norval RA, Bishop RP, Spooner PR.
Author information:
(1)International Livestock Research Institute, Nairobi, Kenya.
A 3-step procedure for cloning Theileria parva parasites was developed. The first step involved the in vitro infection of a fixed number of bovine lymphocytes with titrated sporozoites. The cell lines obtained from infections initiated using sporozoite/lymphocyte ratios below 1:100 were then selected for cloning as these contained schizont-infected cells, each of which was derived from infection with a single sporozoite. In the second step, these cell lines were cloned by limiting dilution. As sporozoites infect lymphocytes and transform to induce clonal multiplication, this step produced infected cell lines containing both cloned parasites and cloned lymphocytes. In the third step, the cloned cell lines were used to infect cattle and isolation of the parasite in ticks was made during piroplasm parasitaemia. Finally, sporozoites were harvested from infected ticks and used for further characterization. Sporozoites derived from cloned cell lines of T. parva Muguga, Marikebuni, Boleni, Uganda and buffalo-derived 7014 were characterized using monoclonal antibody profiles, DNA restriction fragment length polymorphism detected using repetitive and telomeric probes, in vivo infectivity and, in one case, cross-immunity studies. Additionally, several distinct schizont-infected lymphocyte clones were isolated from the Muguga, Mariakani and buffalo-derived 7014 stocks. The combined results of the characterization revealed that the cloning procedure selected clones of T. parva from the parental stocks which were known to contain a mixture of genetically different parasite populations. The cloning method and the clones generated will be of value in studies of the biology of the parasite and in elucidating the strain specificity of immune responses in cattle.

3. Parassitologia. 1999 Sep;41 Suppl 1:73-80.
Development of sero-diagnostic and molecular tools for the control of important tick-borne pathogens of cattle in Africa.
Morzaria SP(1), Katende J, Musoke A, Nene V, Skilton R, Bishop R.
Author information:
(1)International Livestock Research Institute (ILRI), Nairobi, Kenya.
s.morzaria@cgiar.org
Tick-borne diseases (TBDs) are a major economic constraint to livestock production in sub-Saharan Africa. ILRI is focussing on developing a range of products, such as vaccines, diagnostics and decision support services to underpin improved control programmes against these diseases. We have developed

three highly sensitive and specific enzyme linked immuno-assays (ELISAs), which allow precise diagnosis of Theileria parva, Babesia bigemina and Anaplasma marginale. These tests have been standardised and validated using defined experimental and field infection sera. Parasite specific recombinant antigens and monoclonal antibodies against bovine immunoglobulins as secondary antibodies have played an important role in in enhancing the sensitivity and specificity of the assays. They have been further evaluated in on-farm longitudinal sero-epidemiological studies to define infection dynamics and disease risks in various farming systems in Kenya and Uganda. In addition, DNA-based tests for differentiation of Theileria species and characterisation of Theileria parva stocks have been developed. These tests have been derived through physical mapping and sequencing of key elements of the T. parva genome, which include repetitive and telomeric regions, minisatellite sequences, antigen genes and a number of random DNA sequences. These tools are currently being deployed in conjunction with field immunisation programmes to determine the biological impact of introducing live vaccines of T. parva on population dynamics.

4. Int J Parasitol. 2004 Apr;34(5):603-13. doi: 10.1016/j.ijpara.2003.12.012.
Application of a reverse line blot assay to the study of haemoparasites in cattle in Uganda.
Oura CA(1), Bishop RP, Wampande EM, Lubega GW, Tait A.
Author information:
(1)Department of Microbiology and Parasitology, Faculty of Vet Medicine, Makerere University, Kampala, Uganda. chrisoura@hotmail.com
Recent advances in genomic technology have focused many veterinary researchers on the possibility of producing one multivalent recombinant vaccine against all the haemoparasites that infect cattle in the tropics. Before such a vaccine is developed it is essential to define target cattle populations as well as the range of anti-pathogen vaccines required in order to control disease. To further this objective, we have evaluated a reverse line blot (RLB) assay, which simultaneously detects the principal tick transmitted protozoan and rickettsial cattle pathogens, in different epidemiological scenarios in Uganda. A critical question is the sensitivity, particularly in relation to detecting carrier animals. As Theileria parva is considered to be the most important pathogen in the region, we assessed the sensitivity of the RLB assay for T. parva and showed that 1-2 x 10(3) parasites per ml of blood could be detected-a level comparable with previously developed PCR methods and well below conventional microscopic detection. We applied the RLB assay to evaluate the differences in pathogen profiles between crossbred and indigenous cattle and show that there were different profiles, with a low prevalence of T. parva and Theileria taurotragi in the indigenous cattle compared to a high prevalence in the crossbred cattle. In contrast, we show higher prevalences of Theileria mutans and Theileria velifera in the indigenous compared to the crossbred cattle. Interestingly Anaplasma marginale, Babesia bovis and Babesia bigemina were of low prevalence but a high prevalence of Ehrlichia bovis was seen, raising the question of whether this rickettsial species could be pathogenic in cattle. Analysis of animals with clinical symptoms of East Coast Fever showed that, while T. parva

is a major cause of these symptoms, T. mutans and possibly T. taurotragi and T. velifera, may also cause clinical disease. Overall, the results presented here highlight the complexity of tick-borne pathogen infections in cattle in Uganda.

5. Arch Inst Pasteur Tunis. 2004;81(1-4):47-50.
Reverse line blotting: a new technique for the sensitive detection of tick borne pathogens.
Tait A(1), Oura Cl.
Author information:
(1)Department of Veterinary Parasitology, Institute of Comparative Medicine, University of Glasgow, Bearsden Rd, Glasgow G61 1QH, UK.
Recently, a new molecular diagnostic technique for tick borne pathogens has been developed and is called Reverse Line Blotting. This paper outlines the basis of this method and then illustrates its use for the analysis of blood samples obtained from Ugandan cattle and buffalo. The sensitivity and the specificity are discussed in relation to the use of this technique in the Maghreb

6. Parasitol Res. 2011 Jun;108(6):1365-74. doi: 10.1007/s00436-010-2030-8. Epub 2010 Sep 9.
Theileria parva genetic diversity and haemoparasite prevalence in cattle and wildlife in and around Lake Mburo National Park in Uganda.
Oura CA(1), Tait A, Asiimwe B, Lubega GW, Weir W.
Author information:
(1)Department of Microbiology and Parasitology, Faculty of Vet. Medicine, University of Makerere, P.O. Box 7062, Kampala, Uganda. chris.oura@bbsrc.ac.uk
Wildlife, especially Cape buffalo (Syncerus caffer), are thought to act as a reservoir for many of the important tick-borne pathogens of cattle. In this study, we have determined the prevalence of the most significant tick-borne haemoparasites in wildlife (buffalo, impala, eland and bushbuck) as well as in cattle grazing inside and neighbouring Lake Mburo National Park (LMNP) in Uganda. A high percentage of buffalo were carriers of Theileria parva, Theileria mutans, Theileria velifera, Theileria buffeli and Theileria sp. (buffalo) as well as Anaplasma marginale and Anaplasma centrale. The majority of impala sampled were carriers of A. centrale, and all were carriers of an unidentified Babesia/Theileria species. The eland and bushbuck sampled were all carriers of Theileria taurotragi and Theileria buffeli, and the majority were carriers of T. mutans. The bushbuck sampled were also carriers for Erhlichia bovis. There were some differences in the prevalence of haemoparasites between the calves sampled inside and neighbouring LMNP. In order to address the question of whether there is evidence for interbreeding between buffalo-associated and cattle-associated T. parva populations, multi-locus genotypes (MLGs) of T. parva (based on micro-satellite markers) from buffalo and from calves grazing inside and outside LMNP were compared, and the results revealed that buffalo and cattle gene pools were distinct, showing no evidence for transmission of buffalo-derived T. parva genotypes to the cattle population.

7. Vet Parasitol. 2011 Feb 10;175(3-4):212-9. doi: 10.1016/j.vetpar.2010.10.032.
Haemoparasite prevalence and Theileria parva strain diversity in Cape buffalo (Syncerus caffer) in Uganda.
Oura CA(1), Tait A, Asiimwe B, Lubega GW, Weir W.
Author information:
(1)Department of Microbiology and Parasitology, Faculty of Veterinary Medicine, University of Makerere, P.O. Box 7062, Kampala, Uganda. chris.oura@bbsrc.ac.uk
Cape buffalo (Syncerus caffer) are considered to be an important reservoir for various tick-borne haemoparasites of veterinary importance. In this study we have compared the haemoparasite carrier prevalence in buffalo from four geographically isolated national parks in Uganda [Lake Mburo National Park (LMNP), Queen Elizabeth National Park (QENP), Murchison Falls National Park (MFNP) and Kidepo Valley National Park (KVNP)]. Differences were seen in haemoparasite prevalence in buffalo from the four national parks. All the buffalo sampled in LMNP were carriers of Theileria parva however, buffalo from MFNP and KVNP, which are both located in the north of Uganda, were negative for T. parva. Interestingly, 95% of buffalo in the northern part of QENP were T. parva positive, however all buffalo sampled in the south of the park were negative. A high multiplicity of infection was recorded in all the buffalo found to be carrying T. parva, with evidence of at least nine parasite genotypes in some animals. Most of the buffalo sampled in all four national parks were carriers of T. mutans and T. velifera, however none were carriers of T. taurotragi, Babesia bovis, Babesia bigemina, Ehrlichia bovis or Ehrlichia ruminantium. All the buffalo sampled from LMNP were positive for T. buffeli and T. sp. (buffalo) however, buffalo from the parks in the north of the country (KVNP and MFNP) were negative for these haemoparasites. Anaplasma centrale and Anaplasma marginale were circulating in buffalo from all four national parks. T. parva gene pools from two geographically separated populations of buffalo in two of the national parks in Uganda (LMNP and QENP) were compared. The T. parva populations in the two national parks were distinct, indicating that there was limited gene flow between the populations. The results presented highlight the complexity of tick-borne pathogen infections in buffalo and the significant role that buffalo may play as reservoir hosts for veterinary haemoparasites that have the potential to cause severe disease in domestic cattle.

8. Vet Parasitol. 2013 Mar 31;193(1-3):8-14. doi: 10.1016/j.vetpar.2012.12.017.
Haemoparasite infection kinetics and the population structure of Theileria parva on a single farm in Uganda.
Asiimwe BB(1), Weir W, Tait A, Lubega GW, Oura CA.
Author information:
(1)Department of Microbiology and Parasitology, Faculty of Veterinary Medicine, University of Makerere, P.O. Box 7062, Kampala, Uganda.
The development of sensitive PCR-based species-specific diagnostics and parasite genotyping methods offer the opportunity to provide important and detailed information on the infection dynamics of tick-borne disease. In this study we have exploited such tools to investigate the infection kinetics and parasite diversity within Theileria parva in a single farm in Uganda. Initial analysis of

a sample of cattle showed high levels of infection with three Theileria species and Ehrlichia bovis, with most animals being infected with more than one pathogen. To study the infection dynamics, newborn calves were sampled longitudinally and it was shown that all animals became infected with T. parva, T. mutans, T. velifera and E. bovis with the average time to first infection being 53, 74, 116 and 109 days, respectively. However, the majority of these calves cleared the infections with T. parva and E. bovis but remained infected with the other two species of Theileria. In order to investigate the diversity of infecting genotypes of T. parva, samples from six calves were genotyped with a single mini-satellite marker at time points over a nine-month period. Each animal was infected with multiple different sets of genotypes and these were lost over different periods of time, implying that immunity is induced against particular infecting strains. To undertake a higher resolution analysis of parasite genotypes, samples from 30 calves were genotyped with a full panel of 12 micro- and mini-satellite markers but, due to the presence of mixed infections, only 16 samples could be used to generate parasite multi-locus genotypes (MLGs). A high degree of diversity of T. parva was seen on the farm, although some MLGs occurred more than once. Similarity analysis demonstrated a level of sub-structuring and the T. parva population was found to be in linkage disequilibrium. The basis for this high diversity coupled with apparent sub-structuring is discussed in relation to the possible causes.

9. J Egypt Soc Parasitol. 2013 Apr;43(1):87-102. doi: 10.12816/0006369.
The mosquito borne West Nile virus infection: is it threating to Egypt or a neglected endemic disease?
El-Bahnasawy MM(1), Khater MK, Morsy TA.
Author information:
(1)The Military Medical Services for Preventive Medicine, Ain Shams University, Cairo 11566&, Egypt. Mamdouh25@hotmail.com
West Nile virus (WNV) is a mosquito-borne zoonotic arbovirus belonging to the genus Flavivirus in the family Flaviviridae. The virus is found in temperate and tropical regions worldwide, but first identified in the West Nile sub-region in the East African nation of Uganda in 1937. Prior to the mid-1990s WNV infection was sporadically and considered a minor risk for humans, until an outbreak in Algeria in 1994, with cases of WNV-caused encephalitis, and the first large outbreak in Romania in 1996; with a high number of cases with neuroinvasive disease. WNV has now spread globally to Europe beyond the Mediterranean Basin and the United States, is now considered to be an endemic pathogen in worldwide especially in Africa The WNV transmission is mainly b y v a rious mosquitoes species, also tickswere incriminated The birds especially passerines are the most commonly infected animal and serving as the prime reservoir host In Egypt more than 110 mosquito species and subspecies and more than 32 genera of ticks were identified. Besides, not less than 150 species of migratory birds visit Egypt annually in addition to 350 resident ones. This review provided an overview of the current understanding flaviviruses mainly WNFV. Primary care physician and senior nurse should be able to include the disaster diseases in differential diagnosis of various clinical conditions. They should take a

thorough history to request specific dependable laboratory test(s) as soon as possible, and positive patient should be transferred to the fever hospital.

10. Am J Trop Med Hyg. 2013 Nov;89(5):924-7. doi: 10.4269/ajtmh.13-0081. Epub 2013
Coincident tick infestations in the nostrils of wild chimpanzees and a human in Uganda.
Hamer SA(1), Bernard AB, Donovan RM, Hartel JA, Wrangham RW, Otali E, Goldberg TL.
Author information:
(1)Department of Veterinary Integrative Biosciences, Texas A&M University, College Station, Texas; Freelance Nature Photographer, Merchantville, New Jersey; Freelance Nature Photographer, Helena Montana; Department of Biological Sciences, University of Southern California, Los Angeles, California; Department of Human Evolutionary Biology, Harvard University, Cambridge Massachusetts; Makerere University Biological Field Station, Fort Portal, Uganda; Department of Pathobiological Sciences, University of Wisconsin, Madison, Wisconsin.
Ticks in the nostrils of humans visiting equatorial African forests have been reported sporadically for decades, but their taxonomy and natural history have remained obscure. We report human infestation with a nostril tick in Kibale National Park, Uganda, coincident with infestation of chimpanzees in the same location with nostril ticks, as shown by high-resolution digital photography. The human-derived nostril tick was identified morphologically and genetically as a nymph of the genus Amblyomma, but the mitochondrial 12S ribosomal RNA or the nuclear intergenic transcribed spacer 2 DNA sequences of the specimen were not represented in GenBank. These ticks may represent a previously uncharacterized species that is adapted to infesting chimpanzee nostrils as a defense against grooming. Ticks that feed upon apes and humans may facilitate cross-species transmission of pathogens, and the risk of exposure is likely elevated for persons who frequent ape habitats.

11. Ticks Tick Borne Dis. 2013 Dec;4(6):506-12. doi: 10.1016/j.ttbdis.2013.07.001.
High prevalence of spotted fever group rickettsiae in Amblyomma variegatum from Uganda and their identification using sizes of intergenic spacers.
Nakao R(1), Qiu Y(2), Igarashi M(3), Magona JW(4), Zhou L(2), Ito K(3), Sugimoto C(5).
Author information:
(1)Division of Collaboration and Education, Hokkaido University Research Center for Zoonosis Control, Kita 20, Nishi 10, Kita-ku, Sapporo, Hokkaido 001-0020, Japan; Division of Bioinformatics, Hokkaido University Research Center for Zoonosis Control, Kita 20, Nishi 10, Kita-ku, Sapporo, Hokkaido 001-0020, Japan.
(2)Division of Collaboration and Education, Hokkaido University Research Center for Zoonosis Control, Kita 20, Nishi 10, Kita-ku, Sapporo, Hokkaido 001-0020, Japan.
(3)Division of Bioinformatics, Hokkaido University Research Center for Zoonosis Control, Kita 20, Nishi 10, Kita-ku, Sapporo, Hokkaido 001-0020, Japan.
(4)Division of Collaboration and Education, Hokkaido University Research Center for Zoonosis Control, Kita 20, Nishi 10, Kita-ku, Sapporo, Hokkaido 001-0020,

Japan; National Livestock Resources Research Institute (NaLIRRI), Tororo, Uganda.
(5)Division of Collaboration and Education, Hokkaido University Research Center for Zoonosis Control, Kita 20, Nishi 10, Kita-ku, Sapporo, Hokkaido 001-0020, Japan. Electronic address: sugimoto@czc.hokudai.ac.jp.
The spotted fever group (SFG) rickettsiae are obligate intracellular bacteria transmitted by ticks that cause several tick-borne rickettsioses in humans worldwide. This study was intended to determine the prevalence of SFG rickettsiae in Amblyomma variegatum from 7 districts across Uganda. In addition to sequencing of gltA and ompA genes, identification of Rickettsia species based on the sizes of highly variable intergenic spacers, namely, dksA-xerC, mppA-purC, and rpmE-tRNA(fMet) was carried out. Application of multiplex PCR for simultaneous amplification of 3 spacers combined with capillary electrophoresis separation allowed simple, accurate, and high-throughput fragment sizing with considerable time and cost savings. Rickettsia genus-specific real-time PCR detected 136 positives out of 140 samples, giving an overall prevalence of 97.1%. Most samples (n=113) had a size combination of 225, 195, and 341 bp for dksA-xerC, mppA-purC, and rpmE-tRNA(fMet), respectively, which was identical to that of R. africae, a causative agent of African tick bite fever. In addition, several samples had size variants in either dksA-xerC or rpmE-tRNA(fMet). Nonetheless, the partial sequences of gltA and ompA genes of samples of all size combinations showed the greatest similarity to R. africae (99.3-100% for gltA and 98.1-100% for ompA). Given these results, it is highly possible that the tested ticks were infected with R. africae or closely related species. This is a first report on molecular genetic detection of R. africae and its high endemicity in Uganda. Clinicians in this country should be aware of this pathogen as a cause of non-malarial febrile illness. This study provided a starting point for the development of Rickettsia species identification based on the sizes of intergenic spacers. The procedure is simple, rapid, and cost-effective to perform; hence it might be particularly well suited for preliminary species identification in epidemiological investigations. The results may be more detailed and reliable when simultaneous sequencing analysis is performed.

12. Vector Borne Zoonotic Dis. 2014 Mar;14(3):182-8. doi: 10.1089/vbz.2013.1375.
Bartonella species in invasive rats and indigenous rodents from Uganda.
Billeter SA(1), Borchert JN, Atiku LA, Mpanga JT, Gage KL, Kosoy MY.
Author information:
(1)1 Centers for Disease Control and Prevention , Division of Vector Borne Diseases, Fort Collins, Colorado.
The presence of bartonellae in invasive rats (Rattus rattus) and indigenous rodents (Arvicanthis niloticus and Cricetomys gambianus) from two districts in Uganda, Arua and Zombo, was examined by PCR detection and culture. Blood from a total of 228 R. rattus, 31 A. niloticus, and 5 C. gambianus was screened using genus-specific primers targeting the 16S-23S intergenic spacer region. Furthermore, rodent blood was plated on brain heart infusion blood agar, and isolates were verified as Bartonella species using citrate synthase gene- (gltA)

specific primers. One hundred and four fleas recovered from R. rattus were also tested for the presence of Bartonella species using the same gltA primer set. An overall prevalence of 1.3% (three of 228) was obtained in R. rattus, whereas 61.3% of 31 A. niloticus and 60% of five C. gambianus were positive for the presence of Bartonella species. Genotypes related to Bartonella elizabethae, a known zoonotic pathogen, were detected in three R. rattus and one C. gambianus. Bartonella strains, similar to bacteria detected in indigenous rodents from other African countries, were isolated from the blood of A. niloticus. Bartonellae, similar to bacteria initially cultured from Ornithodorus sonrai (soft tick) from Senegal, were found in two C. gambianus. Interestingly, bartonellae detected in fleas from invasive rats were similar to bacteria identified in indigenous rodents and not their rat hosts, with an overall prevalence of 6.7%. These results suggest that if fleas are competent vectors of these bartonellae, humans residing in these two districts of Uganda are potentially at greater risk for exposure to Bartonella species from native rodents than from invasive rats. The low prevalence of bartonellae in R. rattus was quite surprising, in contrast, to the detection of these organisms in a large percentage of Rattus species from other geographical areas. A possible reason for this disparity is discussed.

13. Parasit Vectors. 2015 Jun 5;8:306. doi: 10.1186/s13071-015-0919-x.
Infection and exposure to vector-borne pathogens in rural dogs and their ticks, Uganda.
Proboste T(1), Kalema-Zikusoka G(2), Altet L(3), Solano-Gallego L(4), Fernández de Mera IG(5), Chirife AD(6), Muro J(7), Bach E(8), Piazza A(9), Cevidanes A(10), Blanda V(11), Mugisha L(12)(13), de la Fuente J(14)(15), Caracappa S(16), Millán J(17).
Author information:
(1)Facultad de Ciencias Silvoagropecuarias, Universidad Mayor, Santiago, Chile. tatianaproboste@gmail.com.
(2)Conservation Through Public Health, Plot 3 Mapera Lane, Uringi Crescent, Entebbe, Uganda. gladys@ctph.org.
(3)VetGenomics, Autonomous University of Barcelona, Cerdanyola, 08193, Barcelona, Spain. laura.altet@vetgenomics.com.
(4)Departament de Medicina i Cirurgia Animals, Universitat Autònoma de Barcelona, Cerdanyola, 08193, Barcelona, Spain. lsolano@quiro.uab.es.
(5)Health & Biotechnology (SaBio) Group, IREC (CSIC-UCLM- JCCM), Ronda de Toledo s/n, Ciudad Real, 13005, Spain. mariaisabel.garcia@uclm.es.
(6)Camino del Rosario P5, Peñaflor, Santiago, Chile. andrea.chirife@gmail.com.
(7)Andorra Veterinary Services, Government of Andorra, Andorra La Vella, Andorra. jesusmuro@andorra.ad.
(8)Servei d'Hematologia Clinica Veterinaria (SHCV), Departament de Medicina i Cirurgia Animals, Facultat de Veterinaria, Universitat Autònoma de Barcelona, Cerdanyola, 08193, Barcelona, Spain. ester.bach@gmail.com.
(9)Istituto Zooprofilattico Sperimentale della Sicilia, via Gino Marinuzzi 3, 90129, Palermo, Italy. ninipiazza@hotmail.it.
(10)Facultad de Ecología y Recursos Naturales, Universidad Andres Bello,

Republica 252, Santiago, Chile. aitorcevi@gmail.com.
(11)Istituto Zooprofilattico Sperimentale della Sicilia, via Gino Marinuzzi 3, 90129, Palermo, Italy. valeria.blanda@gmail.com.
(12)College of Veterinary Medicine, Animal Resources and Biosecurity, Makerere University, Kampala, 7062, Uganda. mugishalaw@gmail.com.
(13)Conservation & Ecosystem Health Alliance (CEHA), Kampala, 34153, Uganda. mugishalaw@gmail.com.
(14)Departament de Medicina i Cirurgia Animals, Universitat Autònoma de Barcelona, Cerdanyola, 08193, Barcelona, Spain. josedelafuente@yahoo.com.
(15)Department of Veterinary Pathobiology, Center for Veterinary Health Sciences, Oklahoma State University, Stillwater, 74078, OK, USA. josedelafuente@yahoo.com.
(16)Istituto Zooprofilattico Sperimentale della Sicilia, via Gino Marinuzzi 3, 90129, Palermo, Italy. santo.caracappa@gmail.com.
(17)Facultad de Ecología y Recursos Naturales, Universidad Andres Bello, Republica 252, Santiago, Chile. syngamustrachea@hotmail.com.
BACKGROUND: In rural parts of Africa, dogs live in close association with humans and livestock, roam freely, and usually do not receive prophylactic measures. Thus, they are a source of infectious disease for humans and for wildlife such as protected carnivores. In 2011, an epidemiological study was carried out around three conservation areas in Uganda to detect the presence and determine the prevalence of vector-borne pathogens in rural dogs and associated ticks to evaluate the risk that these pathogens pose to humans and wildlife.
METHODS: Serum samples (n = 105), blood smears (n = 43) and blood preserved on FTA cards (n = 38) and ticks (58 monospecific pools of Haemaphysalis leachi and Rhipicephalus praetextatus including 312 ticks from 52 dogs) were collected from dogs. Dog sera were tested by indirect immunofluorescence to detect the presence of antibodies against Rickettsia conorii and Ehrlichia canis. Antibodies against R. conorii were also examined by indirect enzyme immunoassay. Real time PCR for the detection of Rickettsia spp., Anaplasmataceae, Bartonella spp. and Babesia spp. was performed in DNA extracted from FTA cards and ticks.
RESULTS: 99% of the dogs were seropositive to Rickettsia spp. and 29.5% to Ehrlichia spp. Molecular analyses revealed that 7.8% of the blood samples were infected with Babesia rossi, and all were negative for Rickettsia spp. and Ehrlichia spp. Ticks were infected with Rickettsia sp. (18.9%), including R. conorii and R. massiliae; Ehrlichia sp. (18.9%), including E. chaffeensis and Anaplasma platys; and B. rossi (1.7%). Bartonella spp. was not detected in any of the blood or tick samples.
CONCLUSIONS: This study confirms the presence of previously undetected vector-borne pathogens of humans and animals in East Africa. We recommend that dog owners in rural Uganda be advised to protect their animals against ectoparasites to prevent the transmission of pathogens to humans and wildlife.

14. Am J Trop Med Hyg. 2015 Nov;93(5):1041-51. doi: 10.4269/ajtmh.15-0344. Epub 2015
Genomic Characterization of Yogue, Kasokero, Issyk-Kul, Keterah, Gossas, and Thiafora Viruses: Nairoviruses Naturally Infecting Bats, Shrews, and Ticks.
Walker PJ(1), Widen SG(2), Firth C(2), Blasdell KR(2), Wood TG(2), Travassos da

Rosa AP(2), Guzman H(2), Tesh RB(2), Vasilakis N(2).
Author information:
(1)CSIRO Biosecurity, Australian Animal Health Laboratory, Geelong, Victoria, Australia; Department of Biochemistry and Molecular Biology, The University of Texas Medical Branch, Galveston, Texas; Center for Biodefense and Emerging Infectious Diseases, Department of Pathology, The University of Texas Medical Branch, Galveston, Texas; Center for Tropical Diseases, Institute for Human Infections and Immunity, The University of Texas Medical Branch, Galveston, Texas peter.walker@csiro.au.
(2)CSIRO Biosecurity, Australian Animal Health Laboratory, Geelong, Victoria, Australia; Department of Biochemistry and Molecular Biology, The University of Texas Medical Branch, Galveston, Texas; Center for Biodefense and Emerging Infectious Diseases, Department of Pathology, The University of Texas Medical Branch, Galveston, Texas; Center for Tropical Diseases, Institute for Human Infections and Immunity, The University of Texas Medical Branch, Galveston, Texas.
The genus Nairovirus of arthropod-borne bunyaviruses includes the important emerging human pathogen, Crimean-Congo hemorrhagic fever virus (CCHFV), as well as Nairobi sheep disease virus and many other poorly described viruses isolated from mammals, birds, and ticks. Here, we report genome sequence analysis of six nairoviruses: Thiafora virus (TFAV) that was isolated from a shrew in Senegal; Yogue (YOGV), Kasokero (KKOV), and Gossas (GOSV) viruses isolated from bats in Senegal and Uganda; Issyk-Kul virus (IKV) isolated from bats in Kyrgyzstan; and Keterah virus (KTRV) isolated from ticks infesting a bat in Malaysia. The S, M, and L genome segments of each virus were found to encode proteins corresponding to the nucleoprotein, polyglycoprotein, and polymerase protein of CCHFV. However, as observed in Leopards Hill virus (LPHV) and Erve virus (ERVV), polyglycoproteins encoded in the M segment lack sequences encoding the double-membrane-spanning CCHFV NSm protein. Amino acid sequence identities, complement-fixation tests, and phylogenetic analysis indicated that these viruses cluster into three groups comprising KKOV, YOGV, and LPHV from bats of the suborder Yingochiroptera; KTRV, IKV, and GOSV from bats of the suborder Yangochiroptera; and TFAV and ERVV from shrews (Soricomorpha: Soricidae). This reflects clade-specific host and vector associations that extend across the genus.

15. Ticks Tick Borne Dis. 2016 Mar;7(2):291-7. doi: 10.1016/j.ttbdis.2015.11.004.
Population genetic structure of Theileria parva field isolates from indigenous cattle populations of Uganda.
Muwanika V(1), Kabi F(2), Masembe C(3).
Author information:
(1)Department of Environmental Management, Molecular Genetics Laboratory, College of Agricultural and Environmental Sciences, Makerere University, P.O. Box 7062/7298, Kampala, Uganda.
(2)Department of Environmental Management, Molecular Genetics Laboratory, College of Agricultural and Environmental Sciences, Makerere University, P.O. Box 7062/7298, Kampala, Uganda; National Livestock Resources Research Institute

(NaLIRRI), P.O. Box 96, Tororo, Uganda. Electronic address:
freddykabi@gmail.com.
(3)Department of Biological Sciences, College of Natural Sciences, Makerere University, Box 7062, Kampala, Uganda.
Theileria parva causes East Coast Fever (ECF) a protozoan infection which manifests as a non-symptomatic syndrome among endemically stable indigenous cattle populations. Knowledge of the current genetic diversity and population structure of T. parva is critical for predicting pathogen evolutionary trends to inform development of effective control strategies. In this study the population genetic structure of 78 field isolates of T. parva from indigenous cattle (Ankole, n=41 and East African shorthorn Zebu (EASZ), n=37) sampled from the different agro ecological zones (AEZs) of Uganda was investigated. A total of eight mini- and micro-satellite markers encompassing the four chromosomes of T. parva were used to genotype the study field isolates. The genetic diversity of the surveyed T. parva populations was observed to range from 0.643±0.55 to 0.663±0.41 among the Central and Western AEZs respectively. The overall Wright's F index showed significant genetic variation between the surveyed T. parva populations based on the different AEZs and indigenous cattle breeds (FST=0.133, p<0.01) and (FST=0.101, p<0.01) respectively. Significant pairwise population genetic differentiations (p<0.05) were observed with FST values ranging from 0.048 to 0.173 between the eastern and northern, eastern and western populations respectively. The principal component analysis (PCA) showed a high level of genetic and geographic sub-structuring among populations. Linkage disequilibrium was observed when populations from all the study AEZs were treated as a single population and when analysed separately. On the overall, the significant genetic diversity and geographic sub-structuring exhibited among the study T. parva isolates has critical implications for ECF control.

16. Parasit Vectors. 2016 Mar 5;9:128. doi: 10.1186/s13071-016-1390-z.
No evidence for the involvement of the argasid tick Ornithodoros faini in the enzootic maintenance of marburgvirus within Egyptian rousette bats Rousettus aegyptiacus.
Schuh AJ(1), Amman BR(2), Apanaskevich DA(3), Sealy TK(4), Nichol ST(5), Towner JS(6).
Author information:
(1)Viral Special Pathogens Branch, Division of High-Consequence Pathogens and Pathology, Centers for Disease Control and Prevention, Atlanta, GA, USA.
wuc2@cdc.gov.
(2)Viral Special Pathogens Branch, Division of High-Consequence Pathogens and Pathology, Centers for Disease Control and Prevention, Atlanta, GA, USA.
cxx1@cdc.gov.
(3)United States National Tick Collection, the James H. Oliver, Jr. Institute for Coastal Plain Science, Georgia Southern University, Statesboro, GA, USA.
dapanaskevich@georgiasouthern.edu.
(4)Viral Special Pathogens Branch, Division of High-Consequence Pathogens and Pathology, Centers for Disease Control and Prevention, Atlanta, GA, USA.
tss3@cdc.gov.

(5)Viral Special Pathogens Branch, Division of High-Consequence Pathogens and
Pathology, Centers for Disease Control and Prevention, Atlanta, GA, USA.
stn1@cdc.gov.
(6)Viral Special Pathogens Branch, Division of High-Consequence Pathogens and
Pathology, Centers for Disease Control and Prevention, Atlanta, GA, USA.
jit8@cdc.gov.
BACKGROUND: The cave-dwelling Egyptian rousette bat (ERB; Rousettus aegyptiacus) was recently identified as a natural reservoir host of marburgviruses. However, the mechanisms of transmission for the enzootic maintenance of marburgviruses within ERBs are unclear. Previous ecological investigations of large ERB colonies inhabiting Python Cave and Kitaka Mine, Uganda revealed that argasid ticks (Ornithodoros faini) are hematophagous ectoparasites of ERBs. Yet, their potential role as transmission vectors for marburgvirus has not been sufficiently assessed.
FINDINGS: In the present study, 3,125 O. faini were collected during April 2013 from the rock crevices of Python Cave, Uganda. None of the ticks tested positive for marburgvirus-specific RNA by Q-RT-PCR. The probability of failure to detect marburgvirus at a conservative prevalence of 0.1 % was 0.05.
CONCLUSIONS: The absence of marburgvirus RNA in O. faini suggests they do not play a significant role in the transmission and enzootic maintenance of marburgvirus within their natural reservoir host.

17. Vet Parasitol Reg Stud Reports. 2016 Jun;3-4:27-35. doi:
10.1016/j.vprsr.2016.06.004. Epub 2016 Jun 29.
Molecular investigation of tick-borne haemoparasite infections among transhumant zebu cattle in Karamoja Region, Uganda.
Byaruhanga C(1), Collins NE(2), Knobel D(2), Chaisi ME(2), Vorster I(2), Steyn HC(3), Oosthuizen MC(2).
Author information:
(1)Department of Veterinary Tropical Diseases, Faculty of Veterinary Science,
University of Pretoria, Private Bag X04, Onderstepoort, 0110, South Africa;
National Agricultural Research Organisation, P.O. Box 259, Entebbe, Uganda.
Electronic address: cbyaruhanga27@yahoo.com.
(2)Department of Veterinary Tropical Diseases, Faculty of Veterinary Science,
University of Pretoria, Private Bag X04, Onderstepoort, 0110, South Africa.
(3)Onderstepoort Veterinary Institute, Private Bag X05, Onderstepoort, 0110,
South Africa.
Tick-borne diseases (TBDs) are a major constraint to cattle production in pastoral areas in Africa. Although information on tick-borne infections is important to prioritise control approaches, it is limited for transhumant zebu cattle in Karamoja, Uganda. We conducted a study to determine the occurrence and level of tick-borne infections among cattle in Karamoja Region. A total of 240 cattle were selected for blood collection using systematic sampling in 20 randomly-selected herds in two districts. The hypervariable V4 region of the 18S rRNA gene for Theileria/Babesia and the V1 region of the 16S rRNA gene for Ehrlichia/Anaplasma were amplified and hybridised to genus- and species-specific oligonucleotide probes on a reverse line blot (RLB) membrane. A duplex

quantitative real-time polymerase chain reaction (qPCR) assay based on msp1β and groEL genes was used for the detection of Anaplasma marginale and A. centrale, while monoplex qPCR assays were used for the detection of Ehrlichia ruminantium (226bp fragment of the pCS20 region) and Theileria parva (18S rRNA gene). The RLB hybridisation assay demonstrated the presence of tick-borne haemoparasites in all but one sample (99.6%), mostly as mixed infections (97.5%). The most frequently detected species were Theileria mutans (88.3%, 95% confidence interval: 84.6-91.7%), A. marginale (73.8%: 68.3-78.8%), Theileria velifera (71.3%: 65.8-76.7%) and Anaplasma sp. Omatjenne (63.3%: 57.5-68.8%). Other virulent pathogens, namely Babesia bigemina (5.0%) and T. parva (2.9%), were also detected with RLB, but not E. ruminantium. The proportions of qPCR positive samples were 82.9% (A. marginale), 12.1% (A. centrale), 3.3% (T. parva), and 1.7% (E. ruminantium). The full-length 18S rRNA genes from 6 out of 47 samples that were positive on RLB for the Babesia genus-specific probe and not for any of the Babesia species-specific probes were amplified, cloned and sequenced. The sequences were used to construct phylogenetic trees. Variations (5 to 9 nucleotides) in the 18S rRNA gene sequences of B. bigemina were identified, when compared with B. bigemina sequences from other parts of the world. Three nucleotide differences in the B. bigemina probe region may explain the failure of the RLB hybridisation assay to detect B. bigemina in some samples. T. mutans and B. bigemina sequences grouped in separate clades from previously published sequences. In conclusion, this study demonstrated high and widespread occurrence, and sequence variation of tick-borne haemoparasites among cattle in the pastoral area of Karamoja, which is useful for diagnosis and control of TBDs.

18. Virol J. 2016 Jun 8;13:95. doi: 10.1186/s12985-016-0551-1.
Nucleotide composition of the Zika virus RNA genome and its codon usage.
van Hemert F(1), Berkhout B(2).
Author information:
(1)Laboratory of Experimental Virology, Department of Medical Microbiology, Center for Infection and Immunity Amsterdam (CINIMA), Academic Medical Center, University of Amsterdam, Meibergdreef 15, 1105 AZ, Amsterdam, The Netherlands.
(2)Laboratory of Experimental Virology, Department of Medical Microbiology, Center for Infection and Immunity Amsterdam (CINIMA), Academic Medical Center, University of Amsterdam, Meibergdreef 15, 1105 AZ, Amsterdam, The Netherlands. b.berkhout@amc.uva.nl.
BACKGROUND: RNA viruses have genomes with a distinct nucleotide composition and codon usage. We present the global characteristics of the RNA genome of Zika virus (ZIKV), an emerging pathogen within the Flavivirus genus. ZIKV was first isolated in 1947 in Uganda, caused a widespread epidemic in South and Central America and the Caribbean in 2015 and has recently been associated with microcephaly in newborns.
METHODS: The nearly 11 kb positive-stranded RNA genome of ZIKV was analyzed for its nucleotide composition, also in the context of the folded RNA molecule. Nucleotide trends were investigated along the genome length by skew analyses and we analyzed the codons used for translation of the ZIKV proteins.

RESULTS: ZIKV RNA has a biased nucleotide composition in being purine-rich and pyrimidine-poor. This preference for purines is a general characteristic of the mosquito-borne and tick-borne flaviviruses. The virus-specific nucleotide bias is further enriched in the unpaired, single-stranded regions of the structured ZIKV RNA genome, thus further imposing this ZIKV-specific signature. The codons used for translation of the ZIKV proteins is also unusual, but we show that it is the underlying bias in nucleotide composition of the viral RNA that largely dictates these codon preferences.
CONCLUSIONS: The ZIKV RNA genome has a biased nucleotide composition that dictates the codon usage of this flavivirus. We discuss the evolutionary scenarios and molecular mechanisms that may be responsible for these distinctive ZIKV RNA genome features.

19. Res Vet Sci. 2016 Dec;109:1-9. doi: 10.1016/j.rvsc.2016.09.001. Epub 2016 Sep 9.
Application of ethnobotanical repellents and acaricides in prevention, control and management of livestock ticks: A review.
Pavela R(1), Canale A(2), Mehlhorn H(3), Benelli G(4).
Author information:
(1)Crop Research Institute, Drnovska 507, 161 06 Prague 6, Czech Republic.
(2)Insect Behaviour Group, Department of Agriculture, Food and Environment, University of Pisa, via del Borghetto 80, 56124 Pisa, Italy.
(3)Department of Parasitology, Heinrich Heine University, Düsseldorf, Germany.
(4)Insect Behaviour Group, Department of Agriculture, Food and Environment, University of Pisa, via del Borghetto 80, 56124 Pisa, Italy. Electronic address: benelli.giovanni@gmail.com.
Ticks transmit at least the same number or even more pathogens than any other group of blood-feeding arthropods worldwide affecting humans and animals. The eco-friendly control and management of tick vectors in a constantly changing environment is a crucial challenge. Besides the development of vaccines against ticks, IPM practices aimed at reducing tick interactions with livestock, emerging pheromone-based control tools, and few biological control agents, the extensive employment of acaricides and tick repellents still remain the most effective and ready-to-use strategies. However, the former is limited by the development of growing resistances as well as environmental concerns. Exploiting plants and plant products as sources of effective tick repellents and acaricides represents a promising strategy. In this scenario, the preservation of ethnobotanical information on repellent and acaricidal potential of plants is crucial. Here, we evaluated relevant information published in recent years, focused on plants used as repellents and acaricides against tick vectors in different regions worldwide. We selected a total of 238 plant species, which are traditionally used against ticks by native and local communities of Africa (Kenya, Uganda, Zimbabwe, South Africa), Europe (Serbia, Macedonia, Romania), Asia (Pakistan, India) and America (Brazil, Canada), from 56 families. However, only 7 families (i.e. Asteraceae, Euphorbiaceae, Fabaceae, Lamiaceae, Meliaceae, Apocynaceae and Solanaceae) represent the major quote (46%) of all plant species. We evaluated the differences in acaricidal and repellent efficacy of different formulations used. In the final section, implications arising from the

surveyed anti-tick ethnobotanical knowledge and challenges for its future are discussed.

20. Microbiol Mol Biol Rev. 2017 Feb 8;81(1):e00055-16. doi: 10.1128/MMBR.00055-16.
The Antigenic Structure of Zika Virus and Its Relation to Other Flaviviruses: Implications for Infection and Immunoprophylaxis.
Heinz FX(1), Stiasny K(2).
Author information:
(1)Department of Virology, Medical University of Vienna, Vienna, Austria franz.x.heinz@meduniwien.ac.at.
(2)Department of Virology, Medical University of Vienna, Vienna, Austria.
Zika virus was discovered ∼70 years ago in Uganda and maintained a low profile as a human disease agent in Africa and Asia. Only recently has it caused explosive outbreaks in previously unaffected regions, first in Oceania and then in the Americas since 2015. Of special concern is the newly identified link between congenital malformations (especially microcephaly) and Zika virus infections during pregnancy. At present, it is unclear whether Zika virus changed its pathogenicity or whether the huge number of infections allowed the recognition of a previously cryptic pathogenic property. The purpose of this review is to discuss recent data on the molecular antigenic structure of Zika virus in the context of antibody-mediated neutralization and antibody-dependent enhancement (ADE) of infection, a phenomenon that has been implicated in the development of severe disease caused by the related dengue viruses. Emphasis is given to epitopes of antibodies that potently neutralize Zika virus and also to epitopes that provide antigenic links to other important human-pathogenic flaviviruses such as dengue, yellow fever, West Nile, Japanese encephalitis, and tick-borne encephalitis viruses. The antigenic cross talk between Zika and dengue viruses appears to be of special importance, since they cocirculate in many regions of endemicity and sequential infections are likely to occur frequently. New insights into the molecular antigenic structure of Zika virus and flaviviruses in general have provided the foundation for great progress made in developing Zika virus vaccines and antibodies for passive immunization.

21. Int J Infect Dis. 2018 Mar;68:88-93. doi: 10.1016/j.ijid.2018.01.013. Epub 2018
Investigation of an isolated case of human Crimean-Congo hemorrhagic fever in Central Uganda, 2015.
Balinandi S(1), Patel K(2), Ojwang J(3), Kyondo J(4), Mulei S(4), Tumusiime A(1), Lubwama B(5), Nyakarahuka L(4), Klena JD(2), Lutwama J(4), Ströher U(2), Nichol ST(2), Shoemaker TR(6).
Author information:
(1)Viral Special Pathogens Branch, Centers for Disease Control and Prevention-Uganda, U.S. Embassy, Plot 1577 Ggaba Road, P.O. Box 7007, Kampala, Uganda.
(2)Viral Special Pathogens Branch, Centers for Disease Control and Prevention, 1600 Clifton Rd., Atlanta, GA, USA.
(3)Global Health Security Unit, Centers for Disease Control and Prevention-Uganda, U.S. Embassy, Plot 1577 Ggaba Road, P.O. Box 7007, Kampala,

Uganda.
(4)Department of Emerging, Reemerging and Arbovirus Infections, Uganda Virus Research Institute, Plot 51-57 Nakiwogo Road, P.O. Box 49, Entebbe, Uganda.
(5)Epidemiological Surveillance Division, Ministry of Health, Plot 6, Lourdel Road, P.O. Box 7272, Kampala, Uganda.
(6)Viral Special Pathogens Branch, Centers for Disease Control and Prevention-Uganda, U.S. Embassy, Plot 1577 Ggaba Road, P.O. Box 7007, Kampala, Uganda; Viral Special Pathogens Branch, Centers for Disease Control and Prevention, 1600 Clifton Rd., Atlanta, GA, USA. Electronic address: tis8@cdc.gov.
BACKGROUND: Crimean-Congo hemorrhagic fever (CCHF) is the most geographically widespread tick-borne viral infection. Outbreaks of CCHF in sub-Saharan Africa are largely undetected and thus under-reported. On November 9, 2015, the National Viral Hemorrhagic Fever Laboratory at the Uganda Virus Research Institute received an alert for a suspect VHF case in a 33-year-old male who presented with VHF compatible signs and symptoms at Mengo Hospital in Kampala.
METHODS: A blood sample from the suspect patient was tested by RT-PCR for CCHF and found positive. Serological testing on sequential blood specimens collected from this patient showed increasing anti-CCHFV IgM antibody titers, confirming recent infection. Repeat sampling of the confirmed case post recovery showed high titers for anti-CCHFV-specific IgG. An epidemiological outbreak investigation was initiated following the initial RT-PCR positive detection to identify any additional suspect cases.
RESULTS: Only a single acute case of CCHF was detected from this outbreak. No additional acute CCHF cases were identified following field investigations. Environmental investigations collected 53 tick samples, with only 1, a Boophilus decoloratus, having detectable CCHFV RNA by RT-PCR. Full-length genomic sequencing on a viral isolate from the index human case showed the virus to be related to the DRC (Africa 2) lineage.
CONCLUSIONS: This is the fourth confirmed CCHF outbreak in Uganda within 2 years after more than 50 years of no reported human CCHF cases in this country. Our investigations reaffirm the endemicity of CCHFV in Uganda, and show that exposure to ticks poses a significant risk for human infection. These findings also reflect the importance of having an established national VHF surveillance system and diagnostic capacity in a developing country like Uganda, in order to identify the first cases of VHF outbreaks and rapidly respond to reduce secondary cases. Additional efforts should focus on implementing effective tick control methods and investigating the circulation of CCHFV throughout the country.

22. Ticks Tick Borne Dis. 2018 Mar;9(3):707-717. doi: 10.1016/j.ttbdis.2018.02.004.
Occurrence of tick-borne haemoparasites in cattle in the Mungwi District, Northern Province, Zambia.
Tembo S(1), Collins NE(2), Sibeko-Matjila KP(2), Troskie M(2), Vorster I(2), Byaruhanga C(3), Oosthuizen MC(4).
Author information:
(1)Department of Veterinary Services, Box 19, Mungwi, Zambia; Vectors and

Vector-borne Diseases Research Programme, Department of Veterinary Tropical Diseases, Faculty of Veterinary Science, University of Pretoria, Private Bag X04, Onderstepoort 0110, South Africa.
(2)Vectors and Vector-borne Diseases Research Programme, Department of Veterinary Tropical Diseases, Faculty of Veterinary Science, University of Pretoria, Private Bag X04, Onderstepoort 0110, South Africa.
(3)Vectors and Vector-borne Diseases Research Programme, Department of Veterinary Tropical Diseases, Faculty of Veterinary Science, University of Pretoria, Private Bag X04, Onderstepoort 0110, South Africa; National Agricultural Research Organization, P.O. Box 259, Entebbe, Uganda.
(4)Vectors and Vector-borne Diseases Research Programme, Department of Veterinary Tropical Diseases, Faculty of Veterinary Science, University of Pretoria, Private Bag X04, Onderstepoort 0110, South Africa. Electronic address: marinda.oosthuizen@up.ac.za.
Little is known about the occurrence of haemoparasites in cattle in communal grazing areas of Mungwi District of Northern Province, Zambia. Clinical signs and post mortem lesions are pathognomonic of mixed tick-borne infections especially babesiosis, anaplasmosis and East Coast fever. The main objective of this study was to screen selected communal herds of cattle for tick-borne haemoparasites, and identify the tick vectors associated with the high cattle mortalities due to suspected tick-borne diseases in the local breeds of cattle grazing along the banks of the Chambeshi River in Mungwi District, Northern Province, Zambia. A total of 299 cattle blood samples were collected from July to September 2010 from Kapamba (n = 50), Chifulo (n = 102), Chisanga (n = 38), Kowa (n = 95) and Mungwi central (n = 14) in the Mungwi District. A total of 5288 ticks were also collected from the sampled cattle from April to July 2011. DNA was extracted from the cattle blood and the hypervariable region of the parasite small subunit rRNA gene was amplified and subjected to the reverse line blot (RLB) hybridization assay. The results of the RLB assay revealed the presence of tick-borne haemoparasites in 259 (86.6%) cattle blood samples occurring either as single (11.0%) or mixed (75.6%) infections. The most prevalent species present were the benign Theileria mutans (54.5%) and T. velifera (51.5%). Anaplasma marginale (25.7%), Babesia bovis (7.7%) and B. bigemina (3.3%) DNA were also detected in the samples. Only one sample (from Kapamba) tested positive for the presence of T. parva. This was an unexpected finding; also because the tick vector, Rhipicephalus appendiculatus, was identified on animals from Kowa (14.0%), Chisanga (8.5%), Chifulo (6.0%) and Kapamba (1.4%). One sample (from Kapamba) tested positive for the presence of Ehrlichia ruminantium even though Amblyomma variegatum ticks were identified from 52.9% of the sampled animals from all study areas. There was significant positive association between T. mutans and T. velifera ($p < 0.001$) infections, and between A. marginale and B. bovis ($p = 0.005$). The presence of R. microplus tick vectors on cattle was significantly associated with B. bovis (odds ratio, OR = 28.4, $p < 0.001$) and A. marginale (OR = 42.0, $p < 0.001$) infections, while A. variegatum presence was significantly associated with T. mutans (OR = 213.0, $p < 0.001$) and T. velifera (OR = 459.0, $p < 0.001$) infections. Rhipicephalus decoloratus was significantly associated with B. bigemina (OR = 21.6, $p = 0.004$)

and A. marginale (OR = 28.5, $p < 0.001$). Multivariable analysis showed a significant association between location and tick-borne pathogen status for A. marginale ($p < 0.001$), T. mutans ($p = 0.004$), T. velifera ($p = 0.003$) and T. taurotragi ($p = 0.005$). The results of our study suggest that the cause of cattle mortalities in Mungwi during the winter outbreaks is mainly due to A. marginale, B. bovis and B. bigemina infections. This was confirmed by the clinical manifestation of the disease in the affected cattle and the tick species identified on the animals. The relatively low prevalence of T. parva, B. bigemina, B. bovis and E. ruminantium could indicate the existence of endemic instability with a pool of susceptible cattle and the occurrence of disease outbreaks.

23. Viruses. 2019 Mar 2;11(3):209. doi: 10.3390/v11030209.
Discovery and Characterization of Bukakata orbivirus (Reoviridae:Orbivirus), a Novel Virus from a Ugandan Bat.
Fagre AC(1), Lee JS(2), Kityo RM(3), Bergren NA(4), Mossel EC(5), Nakayiki T(6), Nalikka B(7), Nyakarahuka L(8)(9), Gilbert AT(10), Peterhans JK(11), Crabtree MB(12), Towner JS(13), Amman BR(14), Sealy TK(15), Schuh AJ(16)(17), Nichol ST(18), Lutwama JJ(19), Miller BR(20), Kading RC(21)(22).
Author information:
(1)Department of Microbiology, Immunology, and Pathology, Colorado State University, Fort Collins, CO 80523, USA. anna.fagre@colostate.edu.
(2)Department of Microbiology, Immunology, and Pathology, Colorado State University, Fort Collins, CO 80523, USA. justin.lee@colostate.edu.
(3)Department of Zoology, Entomology and Fisheries Sciences, Makerere University, Kampala, Uganda. kityrob@gmail.com.
(4)Department of Microbiology, Immunology, and Pathology, Colorado State University, Fort Collins, CO 80523, USA. nicholas.bergren@colostate.edu.
(5)Arboviral Diseases Branch, Division of Vector-borne Diseases, Centers for Disease Control and Prevention, Fort Collins, CO 80523, USA. ilv8@cdc.gov.
(6)Department of Arbovirology, Emerging, and Re-emerging Viral Infections, Uganda Virus Research Institute, Entebbe, Uganda. nakayikiteddie@yahoo.com.
(7)Department of Zoology, Entomology and Fisheries Sciences, Makerere University, Kampala, Uganda. bnalikka@gmail.com.
(8)Department of Arbovirology, Emerging, and Re-emerging Viral Infections, Uganda Virus Research Institute, Entebbe, Uganda. nyakarahuka@gmail.com.
(9)Department of Biosecurity, Ecosystems and Veterinary Public Health, Makerere University, Kampala, Uganda. nyakarahuka@gmail.com.
(10)National Wildlife Research Center, US Department of Agriculture, Animal and Plant Health Inspection Service, Wildlife Services, Fort Collins, CO 80521, USA. Amy.T.Gilbert@aphis.usda.gov.
(11)College of Arts and Sciences, Roosevelt University, Collections & Research, The Field Museum of Natural History, Chicago, IL 60605, USA. jkerbis@fieldmuseum.org.
(12)Arboviral Diseases Branch, Division of Vector-borne Diseases, Centers for Disease Control and Prevention, Fort Collins, CO 80523, USA. mbcrab@gmail.com.
(13)Viral Special Pathogens Branch, Division of High Consequence Pathogens and

Pathology, Centers for Disease Control and Prevention, Atlanta, GA 30333, USA. jit8@cdc.gov.
(14)Viral Special Pathogens Branch, Division of High Consequence Pathogens and Pathology, Centers for Disease Control and Prevention, Atlanta, GA 30333, USA. cxx1@cdc.gov.
(15)Viral Special Pathogens Branch, Division of High Consequence Pathogens and Pathology, Centers for Disease Control and Prevention, Atlanta, GA 30333, USA. tss3@cdc.gov.
(16)Viral Special Pathogens Branch, Division of High Consequence Pathogens and Pathology, Centers for Disease Control and Prevention, Atlanta, GA 30333, USA. wuc2@cdc.gov.
(17)United States Public Health Service, Commissioned Corps, Rockville, MD 20852, USA. wuc2@cdc.gov.
(18)Viral Special Pathogens Branch, Division of High Consequence Pathogens and Pathology, Centers for Disease Control and Prevention, Atlanta, GA 30333, USA. Stn1@cdc.gov.
(19)Department of Arbovirology, Emerging, and Re-emerging Viral Infections, Uganda Virus Research Institute, Entebbe, Uganda. jjlutwama03@yahoo.com.
(20)Arboviral Diseases Branch, Division of Vector-borne Diseases, Centers for Disease Control and Prevention, Fort Collins, CO 80523, USA. millerbr1@msn.com.
(21)Department of Microbiology, Immunology, and Pathology, Colorado State University, Fort Collins, CO 80523, USA. rebekah.kading@colostate.edu.
(22)Arboviral Diseases Branch, Division of Vector-borne Diseases, Centers for Disease Control and Prevention, Fort Collins, CO 80523, USA. rebekah.kading@colostate.edu.
While serological and virological evidence documents the exposure of bats to medically-important arboviruses, their role as reservoirs or amplifying hosts is less well-characterized. We describe a novel orbivirus (Reoviridae:Orbivirus) isolated from an Egyptian fruit bat (Rousettus aegyptiacus leachii) trapped in 2013 in Uganda and named Bukakata orbivirus. This is the fifth orbivirus isolated from a bat, however genetic information had previously only been available for one bat-associated orbivirus. We performed whole-genome sequencing on Bukakata orbivirus and three other bat-associated orbiviruses (Fomede, Ife, and Japanaut) to assess their phylogenetic relationship within the genus Orbivirus and develop hypotheses regarding potential arthropod vectors. Replication kinetics were assessed for Bukakata orbivirus in three different vertebrate cell lines. Lastly, qRT-PCR and nested PCR were used to determine the prevalence of Bukakata orbivirus RNA in archived samples from three populations of Egyptian fruit bats and one population of cave-associated soft ticks in Uganda. Complete coding sequences were obtained for all ten segments of Fomede, Ife, and Japanaut orbiviruses and for nine of the ten segments for Bukakata orbivirus. Phylogenetic analysis placed Bukakata and Fomede in the tick-borne orbivirus clade and Ife and Japanaut within the Culicoides/phlebotomine sandfly orbivirus clade. Further, Bukakata and Fomede appear to be serotypes of the Chobar Gorge virus species. Bukakata orbivirus replicated to high titers (10^6–10^7 PFU/mL) in Vero, BHK-21 [C-13], and R06E (Egyptian fruit bat) cells. Preliminary screening of archived bat and tick samples do not support Bukakata

orbivirus presence in these collections, however additional testing is warranted given the phylogenetic associations observed. This study provided complete coding sequence for several bat-associated orbiviruses and in vitro characterization of a bat-associated orbivirus. Our results indicate that bats may play an important role in the epidemiology of viruses in the genus Orbivirus and further investigation is warranted into vector-host associations and ongoing surveillance efforts.

24. Front Cell Infect Microbiol. 2019 Mar 1;9:45. doi: 10.3389/fcimb.2019.00045.
Oral Vaccination With a Formulation Combining Rhipicephalus microplus Subolesin With Heat Inactivated Mycobacterium bovis Reduces Tick Infestations in Cattle.
Contreras M(1), Kasaija PD(1)(2), Merino O(3), de la Cruz-Hernandez NI(3), Gortazar C(1), de la Fuente J(1)(4).
Author information:
(1)SaBio, Instituto de Investigación en Recursos Cinegéticos IREC-CSIC-UCLM-JCCM, Ciudad Real, Spain.
(2)National Livestock Resources Research Institute (NaLIRRI/NARO), Tororo, Uganda.
(3)Facultad de Medicina Veterinaria y Zootecnia, Universidad Autónoma de Tamaulipas, Ciudad Victoria, Mexico.
(4)Department of Veterinary Pathobiology, Center for Veterinary Health Sciences, Oklahoma State University, Stillwater, OK, United States.
Vaccines are an environmentally friendly alternative to acaracides for the control of tick infestations, to reduce the risk for tick-borne diseases affecting human and animal health worldwide, and to improve animal welfare and production. Subolesin (SUB, also known as 4D8) is the functional homolog of Akirin2 involved in the regulation of development and innate immune response, and a proven protective antigen for the control of ectoparasite infestations and pathogen infection. Oral vaccination combining protein antigens with immunostimulants has proven efficacy with increased host welfare and safety, but has not been used for the control of tick infestations. Here we describe the efficacy of oral vaccination with a formulation combining Rhipicephalus microplus SUB and heat inactivated Mycobacterium bovis (IV) on cattle tick infestations and fertility. The levels of IgG antibody titers against SUB and M. bovis P22, and the expression of selected immune response genes were determined and analyzed as possible correlates of protection. We demonstrated that oral immunization with the SUB+IV formulation resulted in 51% reduction in the number of female ticks and 30% reduction in fertility with an overall efficacy of 65% in the control of R. microplus infestations by considering the cumulative effect on reducing tick survival and fertility in cattle. The akr2, IL-1β, and C3 mRNA levels together with antibody levels against SUB correlated with vaccine efficacy. The effect of the oral immunization with SUB+IV in cattle on tick survival and fertility is essential to reduce tick infestations, and extended previous results on the effect of R. microplus SUB for the control of cattle tick infestations. These results support the development of oral vaccines formulations for the control of tick infestations and the incidence of tick-borne diseases.

25. Front Bioeng Biotechnol. 2019 Sep 26;7:236. doi: 10.3389/fbioe.2019.00236.
Identification of Ixodid Tick-Specific Aquaporin-1 Potential Anti-tick Vaccine Epitopes: An in-silico Analysis.
Ndekezi C(1), Nkamwesiga J(1), Ochwo S(1), Kimuda MP(1)(2), Mwiine FN(1), Tweyongyere R(3), Amanyire W(1), Muhanguzi D(1)(4).
Author information:
(1)School of Biosecurity, Biotechnical and Laboratory Science, College of Veterinary Medicine Animal Resources and Biosecurity, Makerere University, Kampala, Uganda.
(2)Research Unit in Bioinformatics, Department of Biochemistry and Microbiology, Rhodes University, Grahamstown, South Africa.
(3)School of Veterinary Medicine and Animal Resources, College of Veterinary Medicine Animal Resources and Biosecurity, Makerere University, Kampala, Uganda.
(4)Makerere University/Uganda Virus Research Institute Centre of Excellence in Infection and Immunity Research and Training, Entebbe, Uganda.
Ticks are arthropod vectors of pathogens of both Veterinary and Public health importance. Acaricide application, which is currently the mainstay of tick control, is hampered by high cost, the need for regular application and a selection of multi-acaricide resistant tick populations. In light of this, future tick control approaches are poised to rely on the integration of rational acaricide application and other methods, such as vaccination. To contribute to systematic research-guided efforts to produce anti-tick vaccines, we carried out an in-silico analysis of tick aquaporin-1 (AQP1) protein in order to identify tick-specific AQP1 peptide motifs that can be used in future peptide anti-tick vaccine development. We carried out multiple sequence alignment (MSA), motif analysis, homology modeling, and structural analysis to identify tick-specific AQP1 peptide motifs. BepiPred, Chou and Fasman-Turn, Karplus and Schulz Flexibility, and Parker-Hydrophilicity prediction models were used to predict these motifs' potential to induce B cell mediated immune responses. The tick AQP1 (GenBankID: QDO67142.1) protein was largely similar to the bovine AQP1 (PDB:1J4N) (23 % sequence similarity; Structural superimposition of the homology model and 14JN homotetramers gave an RMSD = 3.269 while superimposition of a single chain gave an RMSD = 1.475). Tick and bovine AQP1 transmembrane domains were largely similar while their extracellular and cytoplasmic domain loops showed variation. Two tick-specific AQP1 peptide motifs; M7 (residues 106-125, p = 5.4e-25), and M8 (residues 85-104, p = 3.3e-24) were identified. These two motifs are located on the extra-cellular AQP1 domain. Motifs; M7 and M8 showed the highest Parker-Hydrophilicity prediction immunogenicity scores of 1.784 and 1.536, respectively. These two motifs can be a good starting point for the development of potential tick AQP1 peptide-based anti-tick vaccines. Further analyses such as molecular dynamics, in vitro assays, and in vivo immunization assays are required to validate these findings.

26. J Virol. 2020 Mar 31;94(8):e02005-19. doi: 10.1128/JVI.02005-19. Print 2020 Mar
Structure and Characterization of Crimean-Congo Hemorrhagic Fever Virus GP38.
Mishra AK(1), Moyer CL(2), Abelson DM(2), Deer DJ(3), El Omari K(4), Duman R(4), Lobel L(5), Lutwama JJ(6), Dye JM(7), Wagner A(4), Chandran K(8), Cross RW(3),

Geisbert TW(3), Zeitlin L(2), Bornholdt ZA(2), McLellan JS(9).
Author information:
(1)Department of Molecular Biosciences, University of Texas at Austin, Austin, Texas, USA.
(2)Mapp Biopharmaceutical, San Diego, California, USA.
(3)Department of Microbiology and Immunology, University of Texas Medical Branch, Galveston, Texas, USA.
(4)Diamond Light Source, Harwell Science and Innovation Campus, Didcot, Oxfordshire, United Kingdom.
(5)Department of Microbiology, Immunology and Genetics, Faculty of Health Sciences, Ben-Gurion University of the Negev, Beer-Sheva, Israel.
(6)Department of Arbovirology, Emerging and Re-emerging Infection, Uganda Virus Research Institute, Entebbe, Uganda.
(7)US Army Medical Research Institute of Infectious Diseases, Fort Detrick, Maryland, USA.
(8)Department of Microbiology and Immunology, Albert Einstein College of Medicine, Bronx, New York, USA.
(9)Department of Molecular Biosciences, University of Texas at Austin, Austin, Texas, USA jmclellan@austin.utexas.edu.
Crimean-Congo hemorrhagic fever virus (CCHFV) is the causative agent of the most widespread tick-borne viral infection in humans. CCHFV encodes a secreted glycoprotein (GP38) of unknown function that is the target of a protective antibody. Here, we present the crystal structure of GP38 at a resolution of 2.5 Å, which revealed a novel fold primarily consisting of a 3-helix bundle and a β-sandwich. Sequence alignment and homology modeling showed distant homology between GP38 and the ectodomain of Gn (a structural glycoprotein in CCHFV), suggestive of a gene duplication event. Analysis of convalescent-phase sera showed high titers of GP38 antibodies indicating immunogenicity in humans during natural CCHFV infection. The only protective antibody for CCHFV in an adult mouse model reported to date, 13G8, bound GP38 with subnanomolar affinity and protected against heterologous CCHFV challenge in a STAT1-knockout mouse model. Our data strongly suggest that GP38 should be evaluated as a vaccine antigen and that its structure provides a foundation to investigate functions of this protein in the viral life cycle.IMPORTANCE Crimean-Congo hemorrhagic fever virus (CCHFV) is a priority pathogen that poses a high risk to public health. Due to the high morbidity and mortality rates associated with CCHFV infection, there is an urgent need to develop medical countermeasures for disease prevention and treatment. CCHFV GP38, a secreted glycoprotein of unknown function unique to the Nairoviridae family, was recently shown to be the target of a protective antibody against CCHFV. Here, we present the crystal structure of GP38, which revealed a novel fold with distant homology to another CCHFV glycoprotein that is suggestive of a gene duplication event. We also demonstrate that antibody 13G8 protects STAT1-knockout mice against heterologous CCHFV challenge using a clinical isolate from regions where CCHFV is endemic. Collectively, these data advance our understanding of GP38 structure and antigenicity and should facilitate future studies investigating its function.

27. Parasitol Res. 2020 Aug;119(8):2411-2420. doi: 10.1007/s00436-020-06742-z. Epub
Morphological and molecular identification of ixodid tick species (Acari: Ixodidae) infesting cattle in Uganda.
Balinandi S(1)(2), Chitimia-Dobler L(3), Grandi G(4), Nakayiki T(1), Kabasa W(2), Bbira J(2), Lutwama JJ(1), Bakkes DK(5)(6), Malmberg M(7)(8), Mugisha L(2)(9).
Author information:
(1)Uganda Virus Research Institute, P.O. Box 49, Entebbe, Uganda.
(2)College of Veterinary Medicine, Animal Resources and Biosecurity, Makerere University, P.O. Box 7062, Kampala, Uganda.
(3)Bundeswehr Institute of Microbiology, Neuherbergstrasse 11, 80937, Munich, Germany.
(4)Section of Virology, Department of Biomedical Sciences and Veterinary Public Health, Swedish University of Agricultural Sciences, Box 7028, 750 07, Uppsala, Sweden.
(5)Gertrud Theiler Tick Museum, Epidemiology, Parasites and Vectors, Agricultural Research Council - Onderstepoort Veterinary Research, Pretoria, 0110, South Africa.
(6)Evolutionary Genomics Group, Department of Botany and Zoology, Stellenbosch University, Merriman Street, Private Bag X1, Stellenbosch, 7602, South Africa.
(7)Section of Virology, Department of Biomedical Sciences and Veterinary Public Health, Swedish University of Agricultural Sciences, Box 7028, 750 07, Uppsala, Sweden. maja.malmberg@slu.se.
(8)SLU Global Bioinformatics Centre, Department of Animal Breeding and Genetics, Swedish University of Agricultural Sciences, Box 7023, 750 07, Uppsala, Sweden. maja.malmberg@slu.se.
(9)Ecohealth Research Group, Conservation & Ecosystem Health Alliance, P.O. Box 34153, Kampala, Uganda.
In Uganda, the role of ticks in zoonotic disease transmission is not well described, partly, due to limited available information on tick diversity. This study aimed to identify the tick species that infest cattle. Between September and November 2017, ticks (n = 4362) were collected from 5 districts across Uganda (Kasese, Hoima, Gulu, Soroti, and Moroto) and identified morphologically at Uganda Virus Research Institute. Morphological and genetic validation was performed in Germany on representative identified specimens and on all unidentified ticks. Ticks were belonging to 15 species: 8 Rhipicephalus species (Rhipicephalus appendiculatus, Rhipicephalus evertsi evertsi, Rhipicephalus microplus, Rhipicephalus decoloratus, Rhipicephalus afranicus, Rhipicephalus pulchellus, Rhipicephalus simus, and Rhipicephalus sanguineus tropical lineage); 5 Amblyomma species (Amblyomma lepidum, Amblyomma variegatum, Amblyomma cohaerens, Amblyomma gemma, and Amblyomma paulopunctatum); and 2 Hyalomma species (Hyalomma rufipes and Hyalomma truncatum). The most common species were R. appendiculatus (51.8%), A. lepidum (21.0%), A. variegatum (14.3%), R. evertsi evertsi (8.2%), and R. decoloratus (2.4%). R. afranicus is a new species recently described in South Africa and we report its presence in Uganda for the first time. The sequences of R. afranicus were 2.4% divergent from those obtained in Southern Africa. We confirm the presence of the invasive R.

microplus in two districts (Soroti and Gulu). Species diversity was highest in Moroto district (p = 0.004) and geographical predominance by specific ticks was observed (p = 0.001). The study expands the knowledge on tick fauna in Uganda and demonstrates that multiple tick species with potential to transmit several tick-borne diseases including zoonotic pathogens are infesting cattle.

28. Vaccines (Basel). 2020 Jun 18;8(2):319. doi: 10.3390/vaccines8020319.
Vaccination with Recombinant Subolesin Antigens Provides Cross-Tick Species Protection in Bos indicus and Crossbred Cattle in Uganda.
Kasaija PD(1)(2), Contreras M(1)(3), Kabi F(2), Mugerwa S(2), de la Fuente J(1)(4).
Author information:
(1)SaBio, Instituto de Investigación en Recursos Cinegéticos (IREC), Consejo Superior de Investigaciones Científicas (CSIC), Universidad de Castilla-La Mancha (UCLM)-Junta de Comunidades de Castilla-La Mancha (JCCM), Ronda de Toledo s/n, 13005 Ciudad Real, Spain.
(2)National Livestock Resources Research Institute (NaLIRRI/NARO), P.O. Box 5704 Kampala, Uganda.
(3)Interdisciplinary Laboratory of Clinical Analysis, Interlab-UMU, Regional Campus of International Excellence Campus Mare Nostrum, University of Murcia, Espinardo, 30100 Murcia, Spain.
(4)Department of Veterinary Pathobiology, Center for Veterinary Health Sciences, Oklahoma State University, Stillwater, OK 74078, USA.
Cattle tick infestations and transmitted pathogens affect animal health, production and welfare with an impact on cattle industry in tropical and subtropical countries. Anti-tick vaccines constitute an effective and sustainable alternative to the traditional methods for the control of tick infestations. Subolesin (SUB)-based vaccines have shown efficacy for the control of multiple tick species, but several factors affect the development of new and more effective vaccines for the control of tick infestations. To address this challenge, herein we used a regional and host/tick species driven approach for vaccine design and implementation. The objective of the study was to develop SUB-based vaccines for the control of the most important tick species (Rhipicephalus appendiculatus, R. decoloratus and Amblyomma variegatum) affecting production of common cattle breeds (Bos indicus and B. indicus x B. taurus crossbred) in Uganda. In this way, we addressed the development of anti-tick vaccines as an intervention to prevent the economic losses caused by ticks and tick-borne diseases in the cattle industry in Uganda. The results showed the possibility of using SUB antigens for the control of multiple tick species in B. indicus and crossbred cattle and suggested the use of R. appendiculatus SUB to continue research on vaccine design and formulation for the control of cattle ticks in Uganda. Future directions would include quantum vaccinology approaches based on the characterization of the SUB protective epitopes, modeling of the vaccine E under Ugandan ecological and epidemiological conditions and optimization of vaccine formulation including the possibility of oral administration.

29. Pathogens. 2020 Oct 27;9(11):895. doi: 10.3390/pathogens9110895.
First Molecular Detection of Babesia ovis, Theileria spp., Anaplasma spp., and Ehrlichia ruminantium in Goats from Western Uganda.
Tumwebaze MA(1)(2), Byamukama B(1)(2), Tayebwa DS(2)(3), Byaruhanga J(2), Angwe MK(2), Galon EM(1), Liu M(1), Lee SH(1)(4), Ringo AE(1)(5), Adjou Moumouni PF(1), Li J(1), Li Y(1), Ji S(1), Vudriko P(2)(3), Xuan X(1).
Author information:
(1)National Research Center for Protozoan Diseases, Obihiro University of Agriculture and Veterinary Medicine, Obihiro, Hokkaido 080-8555, Japan.
(2)Research Center for Tropical Diseases and Vector Control, College of Veterinary Medicine, Animal Resources and Biosecurity, Makerere University, Kampala 7062, Uganda.
(3)Department of Veterinary Pharmacy, Clinical & Comparative Medicine, School of Veterinary Medicine and Animal Resources, College of Veterinary Medicine, Animal Resources and Biosecurity, Makerere University, Kampala 7062, Uganda.
(4)College of Veterinary Medicine, Chungbuk National University, Cheongju 28644, Korea.
(5)Zanzibar Livestock Research Institute, Ministry of Agriculture, Natural Resources, Livestock and Fisheries, P.O. Box 159 Zanzibar, Tanzania.
Ticks and tick-borne diseases are major impediments to livestock production. To date, there have been several studies on the prevalence of tick-borne pathogens (TBPs) in cattle, but very few studies have documented TBPs in goats in Uganda. In this study, polymerase chain reaction assays and sequence analysis of different molecular markers were used to assess the presence and genetic characteristics of TBPs in 201 goats from Kasese district in western Uganda. The risk factors associated with TBP infections were also analyzed. We detected Theileria spp. (13.4%), Anaplasma phagocytophilum (10.9%), Anaplasma ovis (5.5%), Babesia ovis (5.5%), and Ehrlichia ruminantium (0.5%). The sequences of B. ovis ssu rRNA and A. ovismsp4 genes showed some degree of diversity among the parasite isolates in this study. The E. ruminantium pCS20 sequence formed a well-supported clade with isolates from Amblyomma variegatum ticks from Uganda. Wildlife interaction, sampling location, low body condition score, tick infestation, and herd size were significantly associated with TBP infections in the goats. The findings in this study provide important information on the epidemiology of tick-borne pathogens in Uganda, and show that goats could be potential reservoirs for tick-borne pathogens.

30. Transbound Emerg Dis. 2021 Jul;68(4):2429-2445. doi: 10.1111/tbed.13911. Epub
Tick-borne pathogens, including Crimean-Congo haemorrhagic fever virus, at livestock markets and slaughterhouses in western Kenya.
Chiuya T(1)(2), Masiga DK(1), Falzon LC(3)(4), Bastos ADS(2), Fèvre EM(3)(4), Villinger J(1).
Author information:
(1)International Centre of Insect Physiology and Ecology (icipe), Nairobi, Kenya.
(2)Department of Zoology and Entomology, University of Pretoria, Pretoria, South Africa.

(3)Institute of Infection, Veterinary and Ecological Sciences, University of Liverpool, Neston, UK.
(4)International Livestock Research Institute, Nairobi, Kenya.
Vectors of emerging infectious diseases have expanded their distributional ranges in recent decades due to increased global travel, trade connectivity and climate change. Transboundary range shifts, arising from the continuous movement of humans and livestock across borders, are of particular disease control concern. Several tick-borne diseases are known to circulate between eastern Uganda and the western counties of Kenya, with one fatal case of Crimean-Congo haemorrhagic fever (CCHF) reported in 2000 in western Kenya. Recent reports of CCHF in Uganda have highlighted the risk of cross-border disease translocation and the importance of establishing inter-epidemic, early warning systems to detect possible outbreaks. We therefore carried out surveillance of tick-borne zoonotic pathogens at livestock markets and slaughterhouses in three counties of western Kenya that neighbour Uganda. Ticks and other ectoparasites were collected from livestock and identified using morphological keys. The two most frequently sampled tick species were Rhipicephalus decoloratus (35%) and Amblyomma variegatum (30%); Ctenocephalides felis fleas and Haematopinus suis lice were also present. In total, 486 ticks, lice and fleas were screened for pathogen presence using established molecular workflows incorporating high-resolution melting analysis and identified through sequencing of PCR products. We detected CCHF virus in Rh. decoloratus and Rhipicephalus sp. cattle ticks, and 82 of 96 pools of Am. variegatum were positive for Rickettsia africae. Apicomplexan protozoa and bacteria of veterinary importance, such as Theileria parva, Babesia bigemina and Anaplasma marginale, were primarily detected in rhipicephaline ticks. Our findings show the presence of several pathogens of public health and veterinary importance in ticks from livestock at livestock markets and slaughterhouses in western Kenya. Confirmation of CCHF virus, a Nairovirus that causes haemorrhagic fever with a high case fatality rate in humans, highlights the risk of under-diagnosed zoonotic diseases and calls for continuous surveillance and the development of preventative measures.

31. Emerg Infect Dis. 2020 Dec;26(12):2944-2950. doi: 10.3201/eid2612.202411.
Human-Pathogenic Kasokero Virus in Field-Collected Ticks.
Schuh AJ, Amman BR, Patel K, Sealy TK, Swanepoel R, Towner JS.
Kasokero virus (KASV; genus Orthonairovirus) was first isolated in 1977 at Uganda Virus Research Institute from serum collected from Rousettus aegyptiacus bats captured at Kasokero Cave, Uganda. During virus characterization studies at the institute, 4 laboratory-associated infections resulted in mild to severe disease. Although orthonairoviruses are typically associated with vertebrate and tick hosts, a tick vector of KASV never has been reported. We tested 786 Ornithodoros (Reticulinasus) faini tick pools (3,930 ticks) for KASV. The ticks were collected from a large R. aegyptiacus bat roosting site in western Uganda. We detected KASV RNA in 43 tick pools and recovered 2 infectious isolates, 1 of which was derived from host blood-depleted ticks. Our findings suggest that KASV is maintained in an enzootic transmission cycle involving O. (R.) faini ticks and R. aegyptiacus bats and has the potential for incidental virus spillover to

humans.

32. Afr Health Sci. 2020 Sep;20(3):1168-1178. doi: 10.4314/ahs.v20i3.20.
Rodents as potential hosts and reservoirs of parasites along the edge of a Central African forest: Bwindi impenetrable national park, South Western Uganda.
Mawanda P(1), Rwego I(2)(3), Kisakye JJ(2), Sheil D(4).
Autor information:
(1)Makerere University, College of Education and External Studies, School of Distance and Lifelong studies, Department of distance education. P. O. Box 7062, Kampala, Uganda.
(2)Makerere University, College of Natural Sciences, Department of Biological Sciences, P. O. Box 7062, Kampala, Uganda.
(3)College of Veterinary Medicine, One Health Division, University of Minnesota, St. Paul, 1954 Buford Avenue, MN 55108, USA.
(4)Department of Ecology and Natural Resource Management, Norwegian University of Life Sciences, P. O. Box 5003 NO-1432 Ås, Norway. Center for International Forestry Research (CIFOR), GPO Box 113 BOCBD, Bogor 1600, Indonesia. Institute of Tropical Forest Conservation, P. O. Box 44, Kabale, Uganda.
BACKGROUND: Rodents which constitute 42% of the world's mammalian population are major reservoirs of pathogens that cause zoonoses. Currently we know little about rodents' potential zoonotic transfer from human settlements into protected areas and how any such threats might be reduced.
OBJECTIVE: To investigate the role of rodents as reservoirs of zoonotic pathogens along the boundary of Bwindi.
METHODS: A rodent inventory in three villages along the edge of Bwindi, was carried using live trapping techniques and the local rodents' ecto and endoparasite fauna investigated.
RESULTS: Two hundred eighty eight rodents captured belonged to 24 species, 17 genera and 4 families with Lophuromys aquilus being most abundant (30.2%). 240 ectoparasites which included mites, fleas and ticks were collected from 88 rodents out of 249. Proamys jacksoni rodents were most infested. Although the mites represented the largest proportion (84.6%), the highest species diversity was shown among the fleas (9 species). Some 36.9% of the rodents were infected with endoparasites of which L. aquilus haboured most. Endoparasitic genera identified included Nippostrongylus, Ascaris, Strongyloides, Trichuris, Hymenolepis, Taenia and Cryptosporidium.
CONCLUSION: Rodents have a zoonotic potentiality. There is need for developing effective integrated rodent management programs against rodent to reduce chances of parasite transmission within the protected areas.

33. Parasitol Res. 2021 Mar;120(3):1097-1102. doi: 10.1007/s00436-020-07009-3. Epub
Ticks and tick-borne pathogens infecting livestock and dogs in Tchicala-Tcholoanga, Huambo Province, Angola.
Sili G(1)(2), Byaruhanga C(3)(4), Horak I(1), Steyn H(5), Chaisi M(1)(6), Oosthuizen MC(1), Neves L(1)(7).
Author information:
(1)Vectors and Vector-Borne Diseases Research Programme, Department of

Veterinary Tropical Diseases, Faculty of Veterinary Science, University of
Pretoria, Private Bag X04, Onderstepoort, 0110, South Africa.
(2)Department of Basic Science, Faculty of Veterinary Medicine, University Jose
Eduardo dos Santos, Huambo, Angola.
(3)Vectors and Vector-Borne Diseases Research Programme, Department of
Veterinary Tropical Diseases, Faculty of Veterinary Science, University of
Pretoria, Private Bag X04, Onderstepoort, 0110, South Africa.
cbyaruhanga27@yahoo.com.
(4)National Agricultural Research Organisation, P.O. Box 259, Entebbe, Uganda.
cbyaruhanga27@yahoo.com.
(5)Onderstepoort Veterinary Institute, Private Bag X05, Onderstepoort, 0110,
South Africa.
(6)Foundation Research and Services, South African National Biodiversity
Institute, 232 Boom Street, Pretoria, 0001, South Africa.
(7)Centro de Biotecnologia, Universidade Eduardo Mondlane, Av. de Moçambique Km
1.5, Maputo, Mozambique.
The diversity of ticks and tick-borne pathogens (TBPs) infesting domestic animals in Tchicala-Tcholoanga, Angola, in 2016 was investigated. Seventeen tick species were recorded, Amblyomma pomposum being the most abundant on cattle (40%), goats (38%) and sheep (35%); Rhipicephalus turanicus was the most abundant on dogs (46%). This study presents new records of Haemaphysalis paraleachi, R. compositus, R. kochi and R. sulcatus in Angola, the first georeferenced population of Ha. leachi in southern Africa and the second record of R. microplus in Angola. Using the reverse line blot (RLB) hybridisation assay, fifteen TBP species were detected in blood samples from cattle (n = 88), goats (n = 82), sheep (n = 85) and dogs (n = 85). F The most frequently detected species were Theileria velifera in cattle (78%), Theileria ovis in sheep (80%) and Babesia vogeli in dogs (35%). Species-specific quantitative PCR assays detected Babesia bigemina in 43% (35/80) of blood samples of cattle, while E. ruminantium was detected in 4% (3/70) of blood samples and in 7% of A. pomposum ticks. Anaplasma platys was detected from cattle (18%) and sheep (6%) during RLB analysis. These findings constitute pioneering research in Angola.

34. Ticks Tick Borne Dis. 2021 Jul;12(4):101709. doi: 10.1016/j.ttbdis.2021.101709.
Molecular detection and characterisation of protozoan and rickettsial pathogens in ticks from cattle in the pastoral area of Karamoja, Uganda.
Byaruhanga C(1), Akure PC(2), Lubembe DM(3), Sibeko-Matjila K(2), Troskie M(2), Oosthuizen MC(2), Stoltsz H(2).
Author information:
(1)Vectors and Vector-Borne Diseases Research Programme, Department of
Veterinary Tropical Diseases, Faculty of Veterinary Science, University of
Pretoria, Private Bag X04, Onderstepoort, 0110, South Africa; National
Agricultural Research Organisation, P.O. Box 259, Entebbe, Uganda. Electronic
address: cbyaruhanga27@yahoo.com.
(2)Vectors and Vector-Borne Diseases Research Programme, Department of
Veterinary Tropical Diseases, Faculty of Veterinary Science, University of
Pretoria, Private Bag X04, Onderstepoort, 0110, South Africa.

(3)Vectors and Vector-Borne Diseases Research Programme, Department of
Veterinary Tropical Diseases, Faculty of Veterinary Science, University of
Pretoria, Private Bag X04, Onderstepoort, 0110, South Africa; Department of
Veterinary Pathology, Microbiology and Parasitology, Faculty of Veterinary
Medicine and Surgery, Egerton University, P.O. Box 536, Egerton, 20115, Kenya.
Ticks and tick-borne diseases (TBDs) significantly affect cattle production and
the livelihoods of communities in pastoralist areas. Data on protozoan and
rickettsial pathogens in ticks infesting cattle in Uganda is scanty; while it is
an indicator of the likelihood of disease transmission and occurrence. A
cross-sectional study was conducted amongst cattle in the Karamoja Region,
northeastern Uganda, from July through September 2017, to determine the tick
species diversity, identify protozoan and rickettsial pathogens in the ticks,
and characterise pathogenic species by sequence and phylogenetic analyses. About
50 % of the ticks detected from each predilection site on each animal were
collected from 100 purposively-selected cattle from 20 randomly-selected herds.
Twelve tick species belonging to the genera Amblyomma, Rhipicephalus and
Hyalomma were identified, the most abundant being Amblyomma lepidum (93.9 %),
followed by Amblyomma variegatum (2.0 %) and Rhipicephalus evertsi evertsi (1.0
%). Tick species that have not been reported in recent studies amongst cattle in
Uganda were found, namely Rhipicephalus pravus, Rhipicephalus praetextatus and
Rhipicephalus turanicus. The ticks were grouped into 40 pools, by species and
location, and the reverse line blot (RLB) hybridisation assay was used to detect
pathogens from the ticks. The most frequently detected tick-borne parasites were
Theileria mutans, Theileria velifera and Theileria parva, each observed in 25 %
(10/40) of the tick pools. Tick-borne pathogens, namely Babesia rossi, Babesia
microti and Theileria sp. (sable) that are not common to, or not known to
infect, cattle were identified from ticks. The gene encoding Ehrlichia
ruminantium pCS20 region, the Ehrlichia and Anaplasma 16S rRNA gene, and T.
parva p67 sporozoite antigen gene were amplified, cloned and sequenced. Seven
novel E. ruminantium pCS20 variants were identified, and these grouped into two
separate clusters with sequences from other parts of Africa and Asia. The T.
parva p67 sequences were of the allele type 1, and parasites possessing this
allele type are commonly associated with East Coast fever in eastern Africa.
Analysis of the Ehrlichia and Anaplasma 16S rRNA gene sequences showed that they
were closely related to Rickettsia africae and to a new Ehrlichia species
variant recently found in China. Our R. africae 16S rRNA sequences grouped with
R. africae isolates from Nigeria, Egypt and Benin. The information on tick
species diversity and pathogens in the various tick species provides an
indicator of potential transmission amongst cattle populations, and to humans,
and can be useful to estimate disease risk and in control strategies.

35. Ticks Tick Borne Dis. 2021 Sep;12(5):101756. doi: 10.1016/j.ttbdis.2021.101756.
Cattle ticks and tick-borne diseases: a review of Uganda's situation.
Kasaija PD(1), Estrada-Peña A(2), Contreras M(3), Kirunda H(4), de la Fuente
J(5).
Author information:
(1)SaBio, Instituto de Investigación en Recursos Cinegéticos (IREC), Consejo

Superior de Investigaciones Científicas (CSIC), Universidad de Castilla-La Mancha (UCLM)-Junta de Comunidades de Castilla-La Mancha (JCCM), Ronda de Toledo s/n, 13005 Ciudad Real, Spain; National Livestock Resources Research Institute (NaLIRRI/NARO), P.O. Box 5704, Kampala, Uganda.
(2)Department of Animal Pathology, Faculty of Veterinary Medicine, 50013 Zaragoza, Spain.
(3)SaBio, Instituto de Investigación en Recursos Cinegéticos (IREC), Consejo Superior de Investigaciones Científicas (CSIC), Universidad de Castilla-La Mancha (UCLM)-Junta de Comunidades de Castilla-La Mancha (JCCM), Ronda de Toledo s/n, 13005 Ciudad Real, Spain; Interdisciplinary Laboratory of Clinical Analysis, Interlab-UMU, Regional Campus of International Excellence Campus Mare Nostrum, University of Murcia, Espinardo, 30100 Murcia, Spain.
(4)National Livestock Resources Research Institute (NaLIRRI/NARO), P.O. Box 5704, Kampala, Uganda.
(5)SaBio, Instituto de Investigación en Recursos Cinegéticos (IREC), Consejo Superior de Investigaciones Científicas (CSIC), Universidad de Castilla-La Mancha (UCLM)-Junta de Comunidades de Castilla-La Mancha (JCCM), Ronda de Toledo s/n, 13005 Ciudad Real, Spain; Department of Veterinary Pathobiology, Center for Veterinary Health Sciences, Oklahoma State University, Stillwater, OK 74078, USA. Electronic address: josedejesus.fuente@uclm.es.
Herein we review the epidemiology of ticks and tick-borne diseases (TTBDs), their impact on livestock health and on the economy, control and associated challenges in Uganda. Ticks are leading vectors of economically important pathogens and are widespread in Uganda due to suitable climatic conditions. Besides the physical injury inflicted on the animal host, ticks transmit a number of pathogens that can cause morbidity and mortality of livestock if untreated, resulting in economic losses. Uganda suffers an aggregated annual loss (direct and indirect) of over USD 1.1 billion in the TTBDs complex. East Coast fever (ECF) caused by a protozoan haemoparasite, Theileria parva, is the most prevalent and economically important tick-borne disease (TBD) in Uganda and its vector, the brown ear tick (Rhipicephalus appendiculatus) widely distributed. Other prevalent TBDs in Uganda include anaplasmosis, babesiosis and heartwater. We highlight the role of agro-ecological zones (AEZs) and livestock management system in the distribution of TTBDs, citing warm and humid lowlands as being ideal habitats for ticks and endemic for TBDs. Control of TTBDs is a matter of great importance as far as animal health is concerned in Uganda. Indigenous cattle, which make up over 90% of the national herd are known to be more tolerant to TTBDs and most farms rely on endemic stability to TBDs for control. However, exotic cattle breeds are more capital intensive than indigenous breeds, but the increasing adoption of tick-susceptible exotic cattle breeds (especially dairy) in western and central Uganda demands intensive use of acaricides for tick control and prevention of TBDs. Such acaricide pressure has unfortunately led to selection of acaricide-resistant tick populations and the consequent acaricide resistance observed in the field. Vaccination against ECF, selective breeding for tick resistance and integrated tick control approaches that limit tick exposure, could be adopted to interrupt spread of acaricide resistance. We recommend increasing monitoring and surveillance for TTBDs and

for emerging acaricide resistance, improved extension services and sensitization of farmers on tick control measures, appropriate acaricide use and the development and implementation of vaccines for the control of TTBDs as more sustainable and effective interventions. A tick control policy should be developed, taking into account variations of agro-ecological zones, farm circumstances and indigenous technical knowledge, and this should be incorporated into the overall animal health program.

36. Acta Parasitol. 2022 Mar;67(1):72-78. doi: 10.1007/s11686-021-00433-0. Epub 2021
Molecular Evidence and Hematological Profile of Bovines Naturally Infected with Ehrlichiosis in Southern Punjab, Pakistan.
Basit MA(1), Ijaz M(2), Khan JA(1), Ashraf K(3), Abbas RZ(4).
Author information:
(1)Department of Veterinary Medicine, University of Veterinary and Animal Sciences, Lahore, 54600, Pakistan.
(2)Department of Veterinary Medicine, University of Veterinary and Animal Sciences, Lahore, 54600, Pakistan. mijaz@uvas.edu.pk.
(3)Department of Parasitology, University of Veterinary and Animal Sciences, Lahore, 54600, Pakistan.
(4)Department of Parasitology, University of Agriculture, Faisalabad, 38040, Pakistan.
PURPOSE: Ehrlichiosis is one of the tick-borne diseases, which is not only neglected in Pakistan but only a few reports have been documented throughout the globe. The purpose of this study is to highlight and report the neglected pathogen from bovines in Pakistan.
METHODS: In this study, the pathogen was detected initially based on microscopy, followed by the molecular confirmation and phylogenetic analysis of the pathogen from bovines from south Punjab, Pakistan. The hematological parameters were also assessed in Ehrlichia positive and negative animals. The information of different disease determinants was analyzed by a logistic regression model on SPSS.
RESULTS: The study has reported an 11.98% (23/192) prevalence of Ehrlichiosis from bovines. The prevalence was slightly more in cattle (13.5%) as compared to the buffaloes (10.4%). The previous tick's history and tick control methods were proved to be the key factors with the occurrence of the disease. The isolates from Pakistan Ehrlichia spp. Pakistan/31, 36, and 8 clustered with the isolates from the USA, Nicaraguan, France, South Africa, and Uganda. Platelet count, hemoglobin level, and hematocrit were found significantly decreased in Ehrlichia affected animals when compared to the healthy bovines.
CONCLUSION: This is the first report of ehrlichiosis from bovines in Pakistan and will provide the roadmap for future research.

37. Ticks Tick Borne Dis. 2021 Sep;12(5):101772. doi: 10.1016/j.ttbdis.2021.101772.
Molecular detection of selected tick-borne pathogens infecting cattle at the wildlife-livestock interface of Queen Elizabeth National Park in Kasese District, Uganda.
Byamukama B(1), Vudriko P(2), Tumwebaze MA(1), Tayebwa DS(3), Byaruhanga J(4),

Angwe MK(4), Li J(5), Galon EM(5), Ringo A(5), Liu M(5), Li Y(5), Ji S(5), Rizk MA(6), Moumouni PFA(5), Lee SH(7), Sevinc F(8), Xuan X(9).
Author information:
(1)National Research Center for Protozoan Diseases, Obihiro University of Agriculture and Veterinary Medicine, Obihiro, Hokkaido 080-8555, Japan; Research Center for Tropical Diseases and Vector Control, School of Veterinary Medicine and Animal Resources, Makerere University, 7062, Kampala, Uganda.
(2)Research Center for Tropical Diseases and Vector Control, School of Veterinary Medicine and Animal Resources, Makerere University, 7062, Kampala, Uganda; Department of Veterinary Pharmacy, Clinics & Comparative Medicine, School of Veterinary Medicine and Animal Resources, College of Veterinary Medicine, Animal Resources and Biosecurity, Makerere University, 7062, Kampala, Uganda. Electronic address: vpato2009@gmail.com.
(3)Research Center for Tropical Diseases and Vector Control, School of Veterinary Medicine and Animal Resources, Makerere University, 7062, Kampala, Uganda; Department of Veterinary Pharmacy, Clinics & Comparative Medicine, School of Veterinary Medicine and Animal Resources, College of Veterinary Medicine, Animal Resources and Biosecurity, Makerere University, 7062, Kampala, Uganda.
(4)Research Center for Tropical Diseases and Vector Control, School of Veterinary Medicine and Animal Resources, Makerere University, 7062, Kampala, Uganda.
(5)National Research Center for Protozoan Diseases, Obihiro University of Agriculture and Veterinary Medicine, Obihiro, Hokkaido 080-8555, Japan.
(6)National Research Center for Protozoan Diseases, Obihiro University of Agriculture and Veterinary Medicine, Obihiro, Hokkaido 080-8555, Japan; Department of Internal Medicine and Infectious Diseases, Faculty of Veterinary Medicine, Mansoura University, Mansoura 35516, Egypt.
(7)College of Veterinary Medicine, Chungbuk National University, Cheongju 28644, Korea.
(8)Faculty of Veterinary Medicine, University of Selcuk, Konya, Turkey.
(9)National Research Center for Protozoan Diseases, Obihiro University of Agriculture and Veterinary Medicine, Obihiro, Hokkaido 080-8555, Japan. Electronic address: gen@obihiro.ac.jp.
In Uganda, ticks and tick-borne diseases (TBDs) pose a big challenge to farmers. They reduce cattle productivity and cause severe economic damage. Several studies have documented the prevalence of tick-borne pathogens in cattle; however, their genetic characteristics and the role of wildlife-livestock interaction in the epidemiology of the TBDs are not well documented. This study assessed the prevalence and genetic diversity of various tick-borne pathogens (TBPs) as well as the risk factors associated with the occurrence of TBPs in blood samples of 208 randomly selected cattle from 16 farms located around Queen Elizabeth National Park (QENP) in Kasese District in western Uganda. Farming practices, disease challenges, and the level of wildlife-livestock interactions were assessed by a questionnaire survey amongst farm owners. Polymerase chain reaction (PCR) assays revealed that 62.9% (131/208) cattle samples were positive for one or more pathogens. Using specific PCR assays, we detected Theileria

spp., Theileria parva, Anaplasma marginale, Anaplasma platys-like, and Babesia bigemina at 50.5%, 27.9%, 19.2%, 11.5% and 8.7%, respectively. We also confirmed the infection of samples by Theileria velifera and Theileria mutans after sequencing the Theileria spp. 18S rRNA gene. The risk factors associated with the occurrence of TBPs included communal grazing, herd size, age, and proximity to QENP. Phylogenetic analysis of the T. parva p104 gene showed a high identity to the previous isolates from Uganda and other East African countries and clustered closer to the buffalo (Syncerus caffer) isolates, suggesting a possible cross-species transmission. The sequences of A. marginale groEL and B. bigemina RAP-1a formed well-supported clades with high identities to the previous isolates identified from central and eastern Uganda. The isolates obtained from A. phagocytophilum 16S rRNA gene sequences showed relationship with A. platys-like, Anaplasma sp., uncultured Anaplasma species and A. phagocytophilum isolates from Africa, Asia, Europe, and the USA. The findings of the present study showed that TBDs are still a burden to farmers and that management practices in this area may increase the transmission of pathogens between livestock and wildlife.

38. Pathogens. 2021 Jul 15;10(7):899. doi: 10.3390/pathogens10070899.
A Scoping Review of West Nile Virus Seroprevalence Studies among African Equids.
Olufemi OT(1)(2), Barba M(3)(4), Daly JM(1).
Author information:
(1)One Virology-Wolfson Centre for Global Virus Research, School of Veterinary Medicine and Science, University of Nottingham, Loughborough LE12 5RD, UK.
(2)Department of Veterinary Public Health and Preventive Medicine, Faculty of Veterinary Medicine, University of Jos, Jos 930003, Nigeria.
(3)Microbiological Agents Associated with Animal Reproduction (ProVaginBio), Veterinary Faculty, University Cardenal Herrera-CEU, CEU Universities, 46115 Alfara del Patriarca, Spain.
(4)Innovation Centre in Rural Bioeconomy, Agrifood Research and Technology Centre of Aragon (CITA), Av. Montañana, 930, 50059 Zaragoza, Spain.
West Nile virus (WNV) is an emerging and re-emerging zoonotic flavivirus first identified in and endemic to Africa. The virus is transmitted between birds by biting mosquitoes, with equids and humans being incidental hosts. The majority of infected incidental hosts display no or only mild clinical signs, but a fraction develop encephalitis. The aim of this scoping review was to identify and evaluate primary research on the presence of antibodies to WNV among African equids. Three bibliographic databases and the grey literature were searched. Of 283 articles identified, only 16 satisfied all the inclusion criteria. Data were collated on study design and outcomes. The overall seroprevalence reported ranged from 17.4 to 90.3%, with 1998 (35%) of the 5746 horses, donkeys and mules having screened positive for WNV antibodies. Several articles determined that seroprevalence increased significantly with age. Due to co-circulation of other flaviviruses in Africa, in the majority of studies that screened samples by ELISA, positive results were confirmed using a more specific neutralization test. However, only eight studies tested against other flaviviruses, including Potiskum, Uganda S, Wesselsbron and yellow fever virus in one, Japanese

encephalitis and Usutu virus (USUV) in one, tick-borne encephalitis and USUV in one and USUV only in three. Equids are regarded as useful sentinel animals for WNV, but variation in study design poses challenges when trying to determine risk factors for, and trends in, WNV seroprevalence.

39. Am J Trop Med Hyg. 2021 Oct 18;106(1):88-98. doi: 10.4269/ajtmh.21-0685.
Clinical and Molecular Epidemiology of Crimean-Congo Hemorrhagic Fever in Humans in Uganda, 2013-2019.
Balinandi S(1)(2), Whitmer S(3), Mulei S(1), Nyakarahuka L(1)(2), Tumusiime A(1), Kyondo J(1), Baluku J(1), Mutyaba J(1), Mugisha L(4)(5), Malmberg M(6)(7), Lutwama J(1), Shoemaker TR(3), Klena JD(3).
Author information:
(1)Department of Arbovirology, Emerging and Re-emerging Infectious Diseases, Uganda Virus Research Institute, Entebbe, Uganda.
(2)School of Biosecurity, Biotechnical and Laboratory Sciences, College of Veterinary Medicine, Animal Resources and Biosecurity, Makerere University, Kampala, Uganda.
(3)Viral Special Pathogens Branch, Centers for Disease Control and Prevention, Atlanta, Georgia, USA.
(4)School of Veterinary Medicine and Animal Resources, College of Veterinary Medicine, Animal Resources and Biosecurity, Makerere University, Kampala, Uganda.
(5)EcoHealth Research Group, Conservation & Ecosystem Health Alliance (CEHA), Kampala, Uganda.
(6)Section of Virology, Department of Biomedical Sciences and Veterinary Public Health, Swedish University of Agricultural Sciences, Uppsala, Sweden.
(7)SLU Global Bioinformatics Centre, Department of Animal Breeding and Genetics, Swedish University of Agricultural Sciences, Uppsala, Sweden.
Crimean-Congo Hemorrhagic Fever (CCHF) is endemic in Uganda, yet its epidemiology remains largely uncharacterized. To better understand its occurrence within Uganda, case reports of patients hospitalized with CCHF between 2013 and 2019 were reviewed. Further, genome sequences of CCHF-positive RNA obtained during this period were determined for phylogenetic comparisons. We found that a total of 32 cases (75% males; CFR, 31.2%), aged between 9 to 68 years, were reported during the study period. Most cases were detected during July to December of each outbreak year (81.2%; $P < 0.01$) and were located along the "cattle corridor" (68.7%, $P = 0.03$). The most common presenting symptoms were fever (93.8%), hemorrhage (81.3%), headache (78.1%), fatigue (68.8%), vomiting (68.8%), and myalgia (65.6%). In five patients for whom hematological data were available, varied abnormalities were observed including thrombocytopenia, leukopenia, anemia, lymphopenia, lymphocytosis, polycythemia, and microcytosis. About 56.3% ($P = 0.47$) of patients reported tick bites or exposure to livestock as their potential source of infection. Person-to-person transmission was suspected for two cases. Using unbiased metagenomics, we found that the viral S- and L- segments have remained conserved in Africa 2 clade since the 1950s. In contrast, the M segment split into two geographically interspersed clades; one that belongs to Africa 2 and another that is ancestral

to Africa 1 and 2. Overall, this data summarizes information on the history and clinical presentation of human CCHF in Uganda. Importantly, it identifies vulnerable populations as well as temporal and geographic regions in Uganda where surveillance and control interventions could be focused.

40. Vaccines (Basel). 2021 Oct 15;9(10):1185. doi: 10.3390/vaccines9101185.
From Bench to Field: A Guide to Formulating and Evaluating Anti-Tick Vaccines Delving beyond Efficacy to Effectiveness.
Ndawula C Jr(1)(2).
Author information:
(1)National Agricultural Research Organization, P.O. Box 295, Entebbe, Wakiso 256, Uganda.
(2)National Livestock Resources Research Institute, Vaccinology Research Programme, P.O. Box 5704, Nakyesasa, Wakiso 256, Uganda.
Ticks are ubiquitous blood-sucking ectoparasites capable of transmitting a wide range of pathogens such as bacteria, viruses, protozoa, and fungi to animals and humans. Although the use of chemicals (acaricides) is the predominant method of tick-control, there are increasing incidents of acaricide tick resistance. Furthermore, there are concerns over accumulation of acaricide residues in meat, milk and in the environment. Therefore, alternative methods of tick-control have been proposed, of which anti-tick cattle vaccination is regarded as sustainable and user-friendly. Over the years, tremendous progress has been made in identifying and evaluating novel candidate tick vaccines, yet none of them have reached the global market. Until now, Bm86-based vaccines (Gavac™ in Cuba and TickGARDPLUS™ Australia-ceased in 2010) are still the only globally commercialized anti-tick vaccines. In contrast to Bm86, often, the novel candidate anti-tick vaccines show a lower protection efficacy. Why is this so? In response, herein, the potential bottlenecks to formulating efficacious anti-tick vaccines are examined. Aside from Bm86, the effectiveness of other anti-tick vaccines is rarely assessed. So, how can the researchers assess anti-tick vaccine effectiveness before field application? The approaches that are currently used to determine anti-tick vaccine efficacy are re-examined in this review. In addition, a model is proposed to aid in assessing anti-tick vaccine effectiveness. Finally, based on the principles for the development of general veterinary vaccines, a pipeline is proposed to guide in the development of anti-tick vaccines.

41. Parasit Vectors. 2021 Nov 16;14(1):576. doi: 10.1186/s13071-021-05014-8.
A community approach of pathogens and their arthropod vectors (ticks and fleas) in dogs of African Sub-Sahara.
Heylen D(1)(2)(3), Day M(4), Schunack B(5), Fourie J(6)(7), Labuschange M(8), Johnson S(9), Githigia SM(10), Akande FA(11), Nzalawahe JS(12), Tayebwa DS(13), Aschenborn O(14), Marcondes M(15), Madder M(16)(17).
Author information:
(1)Eco-Epidemiology Group, Department of Biomedical Sciences, Institute of Tropical Medicine, Antwerp, Belgium. dheylen@itg.be.
(2)Interuniversity Institute for Biostatistics and Statistical Bioinformatics,

Hasselt University, Diepenbeek, Belgium. dheylen@itg.be.
(3)Department of Ecology and Evolutionary Biology, Princeton University, Princeton, NJ, USA. dheylen@itg.be.
(4)School of Veterinary and Life Sciences, Murdoch University, Murdoch, WA, Australia.
(5)Bayer Animal Health, Elanco Animal Health Inc., Leverkusen, Germany.
(6)Clinvet LLC, Waverly, USA.
(7)ClinVet International (Pty) Ltd., Bloemfontein, South Africa.
(8)Clinomics, Bloemfontein, South Africa.
(9)School of Veterinary Medicine, College of Basic and Applied Sciences (CBAS), University of Ghana, Accra, Ghana.
(10)Department of Veterinary Pathology, Microbiology and Parasitology, University of Nairobi, Nairobi, Kenya.
(11)Department of Veterinary Parasitology and Entomology, College of Veterinary Medicine, Federal University of Agriculture, Abeokuta, Nigeria.
(12)Sokoine University of Agriculture, Morogoro, Tanzania.
(13)Research Center for Tropical Diseases and Vector Control, College of Veterinary Medicine, Animal Resources and Biosecurity, Makerere University, Kampala, Uganda.
(14)School of Veterinary Medicine, University of Namibia, Neudamm, Namibia, South Africa.
(15)São Paulo State University, São Paulo, Brazil.
(16)University of Pretoria, Pretoria, South Africa.
(17)Clinglobal, Tamarin, Mauritius.
BACKGROUND: Arthropod-borne pathogens and their vectors are present throughout Africa. They have been well-studied in livestock of sub-Saharan Africa, but poorly in companion animals. Given the socio-economic importance of companion animals, the African Small Companion Animal Network (AFSCAN), as part of the WSAVA Foundation, initiated a standardized multi-country surveillance study.
METHODS: Macro-geographic variation in ectoparasite (ticks and fleas) and pathogen communities in dogs was assessed through molecular screening of approximately 100 infested dogs in each of six countries (Ghana, Kenya, Nigeria, Tanzania, Uganda and Namibia), both in rural and urban settings. The most important intrinsic and extrinsic risk factors within the subpopulation of infested dogs were evaluated.
RESULTS: Despite the large macro-geographic variation in the dogs screened, there was no consistent difference between East and West Africa in terms of the diversity and numbers of ticks. The highest and lowest numbers of ticks were found in Nigeria and Namibia, respectively. Most often, there was a higher diversity of ticks in rural habitats than in urban habitats, although the highest diversity was observed in an urban Uganda setting. With the exception of Namibia, more fleas were collected in rural areas. We identified tick species (including Haemaphysalis spinulosa) as well as zoonotic pathogens (Coxiella burnetti, Trypanosoma spp.) that are not classically associated with companion animals. Rhipicephalus sanguineus was the most abundant tick, with a preference for urban areas. Exophilic ticks, such as Haemaphysalis spp., were more often found in rural areas. Several multi-host ticks occurred in urban areas. For R.

sanguineus, housing conditions and additional pets were relevant factors in terms of infestation, while for a rural tick species (Haemaphysalis elliptica), free-roaming dogs were more often infested. Tick occurrence was associated to the use of endoparasiticide, but not to the use of ectoparasiticide. The most prevalent tick-borne pathogen was Hepatozoon canis followed by Ehrlichia canis. High levels of co-parasitism were observed in all countries and habitats. CONCLUSIONS: As dogs share a common environment with people, they have the potential to extend the network of pathogen transmission to humans. Our study will help epidemiologists to provide recommendations for surveillance and prevention of pathogens in dogs and humans.

42. Methods Mol Biol. 2022;2411:253-267. doi: 10.1007/978-1-0716-1888-2_15.
Anti-Tick Vaccines: Current Advances and Future Prospects.
Muhanguzi D(1), Ndekezi C(2), Nkamwesiga J(2), Kalayou S(3), Ochwo S(2), Vuyani M(4), Kimuda MP(2).
Author information:
(1)School of Biosecurity, Biotechnical and Laboratory Science, College of Veterinary Medicine Animal Resources and Biosecurity, Makerere University, Kampala, Uganda. dennis.muhanguzi@mak.ac.ug.
(2)School of Biosecurity, Biotechnical and Laboratory Science, College of Veterinary Medicine Animal Resources and Biosecurity, Makerere University, Kampala, Uganda.
(3)International Centre of Insect Physiology and Ecology (ICIPE), Nairobi, Kenya.
(4)Research Unit in Bioinformatics (RUBi), Department of Biochemistry and Microbiology, Rhodes University, Grahamstown, South Africa.
Ticks are increasingly a global public health and veterinary concern. They transmit numerous pathogens that are of veterinary and public health importance. Acaricides, livestock breeding for tick resistance, tick handpicking, pasture spelling, and anti-tick vaccines (ATVs) are in use for the control of ticks and tick-borne diseases (TTBDs); acaricides and ATVs being the most and least used TTBD control methods respectively. The overuse and misuse of acaricides has inadvertently selected for tick strains that are resistant to acaricides. Furthermore, vaccines are rare and not commercially available in sub-Saharan Africa (SSA). It doesn't help that many of the other methods are labor-intensive and found impractical especially for larger farm operations. The success of TTBD control is therefore dependent on integrating all the currently available methods. Vaccines have been shown to be cheap and effective. However, their large-scale deployment for TTBD control in SSA is hindered by commercial unavailability of efficacious anti-tick vaccines against sub-Saharan African tick strains. Thanks to advances in genomics, transcriptomics, and proteomics technologies, many promising anti-tick vaccine antigens (ATVA) have been identified. However, few of them have been investigated for their potential as ATV candidates. Reverse vaccinology (RV) can be leveraged to accelerate ATV discovery. It is cheap and shortens the lead time from ATVA discovery to vaccine production. This chapter provides a brief overview of recent advances in ATV development, ATVs, ATV effector mechanisms, and anti-tick RV. Additionally, it

provides a detailed outline of vaccine antigen selection and analysis using computational methods.

43. PLoS One. 2021 Dec 10;16(12):e0261218. doi: 10.1371/journal.pone.0261218.
Molecular survey of cattle ticks in Burundi: First report on the presence of the invasive Rhipicephalus microplus tick.
Nyabongo L(1)(2)(3), Odongo DO(1), Milton G(4), Machuka E(2), Vudriko P(5), Pelle R(2), Kanduma EG(6).
Author information:
(1)Faculty of Science and Technology, Department of Biology, University of Nairobi (UoN), Nairobi, Kenya.
(2)Biosciences eastern and central Africa-International Livestock Research Institute (BecA-ILRI) Hub, Nairobi, Kenya.
(3)National Veterinary Laboratory of Bujumbura, Bujumbura, Burundi.
(4)Tick Unit, International Livestock Research Institute, Nairobi, Kenya.
(5)College of Veterinary Medicine, Animal Resources and Biosecurity, Makerere University, Kampala, Uganda.
(6)Faculty of Science and Technology, Department of Biochemistry, University of Nairobi, Nairobi, Kenya.
A recent research study on prevalence of tick-borne pathogens in Burundi reported high prevalence and endemicity of Theileria parva, Anaplasma marginale and Babesia bigemina infections in cattle. Detailed information about tick species infesting animals, their distribution and genetic diversity in Burundi is outdated and limited. This study therefore assessed the prevalence and genetic diversity of tick species infesting cattle across agroecological zones (AEZs) in Burundi. A cross-sectional study on the occurrence of tick species was conducted in 24 districts of Burundi between October and December 2017. Differential identification and characterization of ticks collected was conducted using tick morphological keys and molecular tools (cox1 and 12S rRNA gene). Chi-square test was used to test for association between agroecological zones and the prevalence of tick species. Phylogenetic relationships were inferred using bayesian and maximum likelihood algorithms. A total of 483 ticks were collected from the five AEZs sampled. Six tick species comprising of Rhipicephalus appendiculatus, R. sanguineus, R. evertsi evertsi, R. microplus, R. decoloratus and Amblyomma variegatum were observed. Rhipicephalus appendiculatus were the most prevalent ticks (~45%). A total of 138 specimens (28%) were found to be Rhipicephalus microplus, suggesting an emerging threat for cattle farmers. Twelve R. appendiculatus cox1 haplotypes were obtained from 106 specimens that were sequenced. Two cox1 haplotypes of R. microplus which clustered into previously reported Clade A were observed. Rhipicephalus sanguineus and R. evertsi evertsi ticks, the vectors of numerous zoonotic pathogens, were collected from cattle, which constitute a high risk for public health. These findings reveal an overlapping distribution of tick vectors in Burundi. The design of ticks and tick-borne diseases control strategies should consider the distribution of different vectors across the AEZs particularly the presence of the highly invasive R. microplus tick in Burundi and the potential risk of introducing the pathogenic Babesia bovis.

44. Pathogens. 2022 Jan 19;11(2):114. doi: 10.3390/pathogens11020114.
Sequence Diversity of Tp1 and Tp2 Antigens and Population Genetic Analysis of Theileria parva in Unvaccinated Cattle in Zambia's Chongwe and Chisamba Districts.
Muleya W(1), Atuhaire DK(2), Mupila Z(1), Mbao V(3), Mayembe P(4), Kalenga S(4), Fandamu P(4), Namangala B(5), Salt J(6), Musoke AJ(7).
Author information:
(1)Department of Biomedical Sciences, School of Veterinary Medicine, University of Zambia, Lusaka 10101, Zambia.
(2)Centre for Ticks and Tick-Borne Diseases, Lilongwe, Malawi.
(3)Eastern and Southern Africa Regional Office, International Development Research Centre, Nairobi 00200, Kenya.
(4)Department of Veterinary Services, Ministry of Fisheries and Livestock, Lusaka 50060, Zambia.
(5)Department of Paraclinical Studies, School of Veterinary Medicine, University of Zambia, Lusaka 10101, Zambia.
(6)Global Alliance for Livestock Veterinary Medicines, Pentlands Science Park, Bush Loan, Penicuik EH26 0PZ, UK.
(7)LMK Medical Laboratories and Consultancies, Kampala P.O. Box 33686, Uganda.
East Coast Fever (ECF), caused by Theileria parva, is a major constraint to improved livestock keeping in east and central Africa, including Zambia. To understand the dynamics and determine the candidates for immunization in Zambia's Chongwe and Chisamba districts, a combination of Tp1 and Tp2 gene sequencing and microsatellite analysis using nine markers was conducted from which an abundance of Muguga, Kiambu, Serengeti and Katete epitopes in the field samples was obtained. Phylogenetic analysis showed six (Tp1) and three (Tp2) clusters with an absence of geographical origin clustering. The majority of haplotypes were related to Muguga, Kiambu, Serengeti and Katete, and only a few were related to Chitongo. Both antigens showed purifying selection with an absence of positive selection sites. Furthermore, low to moderate genetic differentiation was observed among and within the populations, and when vaccine stocks were compared with field samples, Chongwe samples showed more similarity to Katete and less to Chitongo, while Chisamba samples showed similarity to both Katete and Chitongo and not to Muguga, Kiambu or Serengeti. We conclude that the use of Katete stock for immunization trials in both Chongwe and Chisamba districts might produce desirable protection against ECF.

45. Vaccine. 2022 Jul 30;40(32):4564-4573. doi: 10.1016/j.vaccine.2022.06.036. Epub
Oral vaccine formulation combining tick Subolesin with heat inactivated mycobacteria provides control of cross-species cattle tick infestations.
Kasaija PD(1), Contreras M(2), Kabi F(3), Mugerwa S(3), Garrido JM(4), Gortazar C(2), de la Fuente J(5).
Author information:
(1)SaBio, Instituto de Investigación en Recursos Cinegéticos IREC-CSIC-UCLM-JCCM, Ronda de Toledo s/n, 13005 Ciudad Real, Spain; National Livestock Resources Research Institute (NaLIRRI/NARO), P.O. Box 5704, Wakiso

District, Uganda.
(2)SaBio, Instituto de Investigación en Recursos Cinegéticos
IREC-CSIC-UCLM-JCCM, Ronda de Toledo s/n, 13005 Ciudad Real, Spain.
(3)National Livestock Resources Research Institute (NaLIRRI/NARO), P.O. Box
5704, Wakiso District, Uganda.
(4)NEIKER-Instituto Vasco de Investigación y Desarrollo Agrario, Animal Health
Department, Bizkaia Science and Technology Park 812L, 48160 Derio (Bizkaia),
Spain.
(5)SaBio, Instituto de Investigación en Recursos Cinegéticos
IREC-CSIC-UCLM-JCCM, Ronda de Toledo s/n, 13005 Ciudad Real, Spain; Department
of Veterinary Pathobiology, Center for Veterinary Health Sciences, Oklahoma
State University, Stillwater, OK 74078, USA. Electronic address:
jose_delafuente@yahoo.com.
Tick vaccines are necessary as part of a One Health approach for the control of tick infestations and tick-borne diseases. Subolesin (SUB, also known as 4D8) is a tick protective antigen that has shown efficacy in vaccine formulations for the control of ectoparasite infestations and pathogen infection/transmission. A recent proof-of-concept study reported oral vaccination combining Rhipicephalus microplus SUB with heat inactivated Mycobacterium bovis (IV) as an immunostimulant for the control of cattle tick infestations. Based on the efficacy of Rhipicephalus decoloratus SUB for the control of multiple cattle tick species in Uganda, herein we design a controlled pen trial using an oral formulation combining R. decoloratus SUB with IV for the control of R. decoloratus and Rhipicephalus appendiculatus cattle tick infestations. Vaccine efficacy (E) of SUB + IV on tick life cycle was compared with IV and SUB alone and with PBS as control. The IgG antibody titers against SUB and M. bovis P22 and the serum levels of selected protein immune biomarkers (IL-1beta, TNF-alpha, C3) were determined and analyzed as possible correlates of protection. Oral immunization with IV and SUB alone and in SUB + IV combination were effective for the control of tick infestations (E = 71-96% for R. decoloratus and 87-99% for R. appendiculatus) with highest E (higher than 95%) for SUB + IV. The results demonstrated that oral immunization with the SUB + IV formulation resulted in effective control of cattle tick infestations through the activation of multiple immune mechanisms. These results support the application of oral vaccine formulations with SUB + IV for the control of cattle infestations with Rhipicephalus species towards improving animal health.

46. Animals (Basel). 2022 Jul 1;12(13):1708. doi: 10.3390/ani12131708.
Molecular Survey and Genetic Characterization of Anaplasma marginale in Ticks Collected from Livestock Hosts in Pakistan.
Khan Z(1), Shehla S(1), Alouffi A(2), Kashif Obaid M(1), Zeb Khan A(3), Almutairi MM(4), Numan M(1), Aiman O(1), Alam S(1), Ullah S(1), Zaman Safi S(5)(6), Tanaka T(7), Ali A(1).
Author information:
(1)Department of Zoology, Abdul Wali Khan University Mardan, Mardan 23200, Pakistan.
(2)King Abdulaziz City for Science and Technology, Riyadh 12354, Saudi Arabia.

(3)Department of Pediatrics, Yale School of Medicine, Yale University, New Haven, CT 06510, USA.
(4)Department of Pharmacology and Toxicology, College of Pharmacy, King Saud University, Riyadh 11451, Saudi Arabia.
(5)Faculty of Medicine, Bioscience and Nursing, MAHSA University, Jenjarom 42610, Selangor, Malaysia.
(6)Interdisciplinary Research Centre in Biomedical Materials, Lahore Campus, COMSATS University Islamabad, Lahore 54000, Pakistan.
(7)Laboratory of Infectious Diseases, Joint Faculty of Veterinary Medicine, Kagoshima University, 1-21-24 Korimoto, Kagoshima 890-0065, Japan.
Ticks transmit pathogens to animals and humans more often than any other arthropod vector. The rural economy of Pakistan mainly depends on livestock farming, and tick infestations cause severe problems in this sector. The present study aimed to molecularly characterize the Anaplasma spp. in hard ticks collected from six districts of Khyber Pakhtunkhwa, Pakistan. Ticks were collected from various livestock hosts, including cattle breeds (Holstein-Friesian, Jersey, Sahiwal, and Achai), Asian water buffaloes, sheep, and goats from March 2018 to February 2019. Collected ticks were morphologically identified and subjected to molecular screening of Anaplasma spp. by amplifying 16S rDNA sequences. Six hundred seventy-six ticks were collected from infested hosts (224/350, 64%). Among the nine morphologically identified tick species, the highest occurrence was noted for Rhipicephalus microplus (254, 37.6%), followed by Hyalomma anatolicum (136, 20.1%), Rhipicephalus haemaphysaloides (119, 17.6%), Rhipicephalus turanicus (116, 17.1%), Haemaphysalis montgomeryi (14, 2.1%), Hyalomma dromedarii (11, 1.6%), Haemaphysalis bispinosa (10, 1.5%), Hyalomma scupense (8, 1.2%), and Haemaphysalis kashmirensis (8, 1.2%). The occurrence of tick females was highest (260, 38.5%), followed by nymphs (246, 36.4%) and males (170, 25.1%). Overall, the highest occurrence of ticks was recorded in the Peshawar district (239, 35.3%), followed by Mardan (183, 27.1%), Charsadda (110, 16.3%), Swat (52, 7.7%), Shangla (48, 7.1%), and Chitral (44, 6.5%). Among these ticks, Anaplasma marginale was detected in R. microplus, R. turanicus, and R. haemaphysaloides. The 16S rDNA sequences showed high identity (98-100%) with A. marginale reported from Australia, China, Japan, Pakistan, Thailand, Uganda, and the USA. In phylogenetic analysis, the sequence of A. marginale clustered with the same species reported from Australia, China, Pakistan, Thailand, Uruguay, and the USA. Further molecular work regarding the diversity of tick species and associated pathogens is essential across the country.

47. Parasit Vectors. 2022 Sep 9;15(1):321. doi: 10.1186/s13071-022-05436-y.
A community approach for pathogens and their arthropod vectors (ticks and fleas) in cats of sub-Saharan Africa.
Madder M(1), Day M(2), Schunack B(3), Fourie J(4)(5), Labuschange M(6), van der Westhuizen W(6), Johnson S(7), Githigia SM(8), Akande FA(9), Nzalawahe JS(10), Tayebwa DS(11), Aschenborn O(12), Marcondes M(13), Heylen D(14)(15)(16).
Author information:
(1)Clinglobal, Tamarin, Mauritius. maxime.madder@clinglobal.com.

(2)School of Veterinary and Life Sciences, Murdoch University, Murdoch, WA, Australia.
(3)Elanco Animal Health, Leverkusen, Germany.
(4)Clinvet, Waverly, USA.
(5)Clinvet, Bloemfontein, South Africa.
(6)Clinomics, Bloemfontein, South Africa.
(7)School of Veterinary Medicine, CBAS, University of Ghana, Accra, Ghana.
(8)Department of Veterinary Pathology, Microbiology & Parasitology, University of Nairobi, Nairobi, Kenya.
(9)Department of Veterinary Parasitology and Entomology, College of Veterinary Medicine, Federal University of Agriculture, Abeokuta, Nigeria.
(10)Sokoine University of Agriculture, Morogoro, Tanzania.
(11)Research Center for Tropical Diseases and Vector Control, College of Veterinary Medicine, Animal Resources and Biosecurity, Makerere University, Kampala, Uganda.
(12)School of Veterinary Medicine, University of Namibia, Ondekaremba, Namibia.
(13)São Paulo State University, São Paulo, Brazil.
(14)Eco-Epidemiology Group, Department of Biomedical Sciences, Institute of Tropical Medicine, Antwerp, Belgium.
(15)Interuniversity Institute for Biostatistics and Statistical Bioinformatics, Hasselt University, Diepenbeek, Belgium.
(16)Department of Ecology and Evolutionary Biology, Princeton University, Princeton, NJ, USA.
BACKGROUND: Arthropod-borne pathogens and their vectors are present throughout Africa. They have been well studied in livestock of sub-Saharan Africa, but poorly studied in companion animals. Given their socioeconomic importance, the African Small Companion Animal Network (AFSCAN), as part of the WSAVA Foundation, initiated a standardized multi-country surveillance study.
METHODS: In six countries (Ghana, Kenya, Nigeria, Tanzania, Uganda, and Namibia) in both rural and urban settings, 160 infested cats were sampled to assess their ectoparasite community (ticks and fleas), as well as the micro-parasite prevalence within those ectoparasites (60 and 118 pools of ticks and fleas, respectively) and blood (276 cats, including 116 non-infested).
RESULTS: Almost two thirds of all infested cats originated from Tanzania and Kenya. Despite the large macro-geographical variation, no consistent difference was found in ectoparasite diversity and numbers between East and West Africa. Far more flea-infested than tick-infested cats were found. The most dominant ectoparasite was Ctenocephalides felis. Among the ticks, the exophilic Haemaphysalis spp. were the commonest, including species that are not typically linked with companion animals (Haemaphysalis spinulosa and Haemaphysalis elliptica). The most prevalent pathogens found in the blood and fleas were Bartonella henselae and Mycoplasma haemofelis. In the ticks, the dog-associated Hepatozoon canis was most commonly found. A high degree of co-parasitism was found in all countries and habitats.
CONCLUSIONS: Our continent-wide standardized field study highlights the cat's potential to serve as a reservoir of pathogens that can be transmitted to humans or livestock, especially when cats are expected to become more commonly kept in

African villages and towns.

48. J Med Entomol. 2023 Jan 12;60(1):185-192. doi: 10.1093/jme/tjac166.
Minimal Rickettsial Infection Rates and Distribution of Ticks in Uganda: An Assessment of the Seasonal Effects and Relevance to Tick-Borne Disease Risk in East Africa.
Corrigan J(1)(2), Marion B(1)(2), English J(1), Eneku W(3), Weng JL(1), Rugg M(1), Dotrang T(1), Dunford J(1), Byaruhanga AM(3), Byarugaba DK(3), Ramalho-Ortigao M(1), Wanja EW(1).
Author information:
(1)Division of Global Public Health, Department of Preventive Medicine and Biostatistics, Uniformed Services University, 4301 Jones Bridge Road, Bethesda, MD 20814, USA.
(2)MPH Program, Department of Preventive Medicine and Biostatistics, Uniformed Services University, 4301 Jones Bridge Road, Bethesda, MD 20814, USA.
(3)Makerere University, Kampala, Uganda.
Tick-borne diseases (TBDs) pose a significant risk to humans and represent one of the major factors influencing readiness within the United States' military worldwide. Additionally, ticks and TBDs constitute major animal health problems leading to economic losses at multiple levels affecting low- and middle-income countries the hardest. Tick control is frequently hampered by issues ranging from acaricide resistance to lack of data on tick distribution and infection rates. We conducted a cross-sectional study to assess tick species distribution, host use, and rickettsial pathogen infection rate of ticks in different areas of the Uganda Cattle Corridor. We identified 4,425 hard ticks (Ixodida: Ixodidae) comprised of seven species by morphological characters with 3,315 ticks collected from four locations during the dry season and 1,110 ticks from one location during the wet season. Rickettsial pathogen prevalence was assessed in ticks collected from two districts to determine the minimum infection rate compared across seasons, village location, and tick species. We found statistically significant differences in the abundance and distribution of tick species among districts in the dry season, host animal species, and the proportion of rickettsial positive pools between villages. Seasonality, village location, and tick species do not affect the minimum infection rate of rickettsial pathogens of ticks in Uganda, but village location affects the proportion of positive tick pools. These results indicate geographical and seasonal differences among pathogen-harboring ticks contributing to our understanding of the current distribution of ticks and TBDs in Uganda.

49. Sci Rep. 2022 Dec 3;12(1):20936. doi: 10.1038/s41598-022-24673-w.
Natural reservoir Rousettus aegyptiacus bat host model of orthonairovirus infection identifies potential zoonotic spillover mechanisms.
Schuh AJ(1)(2), Amman BR(3), Guito JC(3), Graziano JC(3), Sealy TK(3), Kirejczyk SGM(3), Towner JS(4).
Author information:
(1)Viral Special Pathogens Branch, Division of High-Consequence Pathogens and Pathology, United States Centers for Disease Control and Prevention, Atlanta,

GA, USA. wuc2@cdc.gov.
(2)United States Public Health Service Commissioned Corps, Rockville, MD, USA. wuc2@cdc.gov.
(3)Viral Special Pathogens Branch, Division of High-Consequence Pathogens and Pathology, United States Centers for Disease Control and Prevention, Atlanta, GA, USA.
(4)Viral Special Pathogens Branch, Division of High-Consequence Pathogens and Pathology, United States Centers for Disease Control and Prevention, Atlanta, GA, USA. jit8@cdc.gov.
The human-pathogenic Kasokero virus (KASV; genus Orthonairovirus) has been isolated from the sera of Egyptian rousette bats (ERBs; Rousettus aegyptiacus) captured in Uganda and unengorged Ornithodoros (Reticulinasus) faini ticks collected from the rock crevices of ERB colonies in South Africa and Uganda. Although evidence suggests that KASV is maintained in an enzootic transmission cycle between O. (R.) faini ticks and ERBs with potential for incidental virus spillover to humans through the bite of an infected tick, the vertebrate reservoir status of ERBs for KASV has never been experimentally evaluated. Furthermore, the potential for bat-to-bat and bat-to-human transmission of KASV is unknown. Herein, we inoculate two groups of ERBs with KASV; one group of bats is serially sampled to assess viremia, oral, fecal, and urinary shedding and the second group of bats is serially euthanized to assess virus-tissue tropism. Throughout the study, none of the bats exhibit overt signs of clinical disease. Following the detection of high KASV loads of long duration in blood, oral, fecal, and urine specimens collected from ERBs in the serial sampling group, all bats seroconvert to KASV. ERBs from the serial euthanasia group exhibit high KASV loads indicative of virus replication in the skin at the inoculation site, spleen, and inguinal lymph node tissue, and histopathology and in situ hybridization reveal virus replication in the liver and self-limiting, KASV-induced lymphohistiocytic hepatitis. The results of this study suggest that ERBs are competent, natural vertebrate reservoir hosts for KASV that can sustain viremias of appropriate magnitude and duration to support virus maintenance through bat-tick-bat transmission cycles. Viral shedding data suggests that KASV might also be transmitted bat-to-bat and highlights the potential for KASV spillover to humans through contact with infectious oral secretions, feces, or urine.

50. Pathogens. 2023 Jan 10;12(1):115. doi: 10.3390/pathogens12010115.
Assessment of the Impact of Early Diagnosis and Early Treatment in the Integrated Control of East Coast Fever (ECF) Involving Acquired Immunity Induced by Natural Infection in Ankole Cattle.
Nanteza A(1), Nsadha Z(1), Nsubuga J(1), Oligo S(1), Kazibwe A(1), Terundaja C(1), Matovu E(1), Lubega GW(1).
Author information:
(1)College of Veterinary Medicine, Animal Resources and Biosecurity, Makerere University, Kampala P.O. Box 7062, Uganda.
The integrated control of East Coast fever (ECF) by early diagnosis and treatment involving acquired immunity induced by natural infection in Ankole

cattle was assessed. A longitudinal study was carried out in Kiruhura district, southwestern Uganda for six months on 244 Ankole breed of cattle from 18 herds under natural tick challenge and relaxed tick control measures. Calves aged three to six months old were recruited and monitored daily by farmers for detection of ECF clinical symptoms. The reported sick animals were treated using Buparvaquone and treatment outcome determined. Monthly follow-ups and blood collections were done to monitor ECF status. Blood was analyzed for Theileria parva parasites by microscopy, DNA by polymerase chain reaction (PCR) and antibodies by enzyme-linked immunosorbent assay (ELISA). The overall prevalence of ECF clinical disease within six months period was 30.3% (74). The major symptoms of early clinical ECF disease were fever and enlarged parotid or prescapular lymph nodes. Clinical cases were categorized as mild, 24% (18) or moderate, 76% (56). There was an overall recovery rate of 100% (74) of the ECF cases whereby 94.6% (70) recovered promptly and 5.4% (4) recovered slowly. Based on blood analysis, prevalence of ECF at baseline was 3.7% (9) by microscopy, 31.1% (76) by PCR and 38.1% (93) by ELISA. A significant increase ($p < 0.05$) was shown by the increased number of calves with T. parva specific antibodies in the sera from 38.1% at baseline to 68.8% after six months. High antibody levels (positive percentage ≥ 50%) were detected in all ECF-treated and recovered calves at the end of six months. The acquired immunity to ECF was high in treated and recovered cattle, indicating that natural exposure to infection, accurate early diagnosis and effective treatment enhance development of immune-protection in indigenous cattle in an endemic area. The prominent early clinical symptoms for ECF could be exploited in the development of decision support tools for chemotherapy and other integrated control measures.

51. Vaccines (Basel). 2022 Dec 31;11(1):99. doi: 10.3390/vaccines11010099.
Inspiring Anti-Tick Vaccine Research, Development and Deployment in Tropical Africa for the Control of Cattle Ticks: Review and Insights.
Kasaija PD(1)(2), Contreras M(1), Kirunda H(3), Nanteza A(4), Kabi F(2), Mugerwa S(2), de la Fuente J(1)(5).
Author information:
(1)SaBio, Instituto de Investigación en Recursos Cinegéticos (IREC), Consejo Superior de Investigaciones Científicas (CSIC), Universidad de Castilla-La Mancha (UCLM)-Junta de Comunidades de Castilla-La Mancha (JCCM), Ronda de Toledo s/n, 13005 Ciudad Real, Spain.
(2)National Livestock Resources Research Institute (NaLIRRI), Kampala P.O. Box 5704, Uganda.
(3)National Agricultural Research Organisation (NARO), Mbarara ZARDI, Mbarara P.O. Box 389, Uganda.
(4)College of Veterinary Medicine, Animal Resources and Biosecurity, Makerere University, Kampala P.O. Box 7062, Uganda.
(5)Department of Veterinary Pathobiology, Center for Veterinary Health Sciences, Oklahoma State University, Stillwater, OK 74078, USA.
Ticks are worldwide ectoparasites to humans and animals, and are associated with numerous health and economic effects. Threatening over 80% of the global cattle population, tick and tick-borne diseases (TTBDs) particularly constrain

livestock production in the East, Central and Southern Africa. This, therefore, makes their control critical to the sustainability of the animal industry in the region. Since ticks are developing resistance against acaricides, anti-tick vaccines (ATVs) have been proposed as an environmentally friendly control alternative. Whereas they have been used in Latin America and Australia to reduce tick populations, pathogenic infections and number of acaricide treatments, commercially registered ATVs have not been adopted in tropical Africa for tick control. This is majorly due to their limited protection against economically important tick species of Africa and lack of research. Recent advances in various omics technologies and reverse vaccinology have enabled the identification of many candidate anti-tick antigens (ATAs), and are likely to usher in the next generation of vaccines, for which Africa should prepare to embrace. Herein, we highlight some scientific principles and approaches that have been used to identify ATAs, outline characteristics of a desirable ATA for vaccine design and propose the need for African governments to investment in ATV research to develop vaccines relevant to local tick species (personalized vaccines). We have also discussed the prospect of incorporating anti-tick vaccines into the integrated TTBDs control strategies in the sub-Saharan Africa, citing the case of Uganda.

52. Parasit Vectors. 2023 Jan 22;16(1):22. doi: 10.1186/s13071-022-05632-w.
Survey of ticks and tick-borne pathogens in wild chimpanzee habitat in Western Uganda.
Lacroux C(1)(2)(3)(4), Bonnet S(5)(6), Pouydebat E(7), Buysse M(8)(9), Rahola N(8), Rakotobe S(10), Okimat JP(11), Koual R(8), Asalu E(12), Krief S(#)(13)(14), Duron O(#)(15).
Author information:
(1)UMR 7206 CNRS/MNHN/P7, Eco-anthropologie, Museum National d'Histoire Naturelle, Musée de l'Homme, 17 Place du Trocadéro, 75116, Paris, France. camille.lacroux@mnhn.fr.
(2)Sebitoli Chimpanzee Project, Great Ape Conservation Project, Kibale National Park, Fort Portal, Uganda. camille.lacroux@mnhn.fr.
(3)UMR 7179 CNRS/MNHN, Mécanismes Adaptatifs et Evolution, Museum National d'Histoire Naturelle, 57 Rue Cuvier, 75231, Paris, France. camille.lacroux@mnhn.fr.
(4)La Phocéenne de Cosmétique, ZA Les Roquassiers, 174 Rue de la Forge, 13300, Salon-de-Provence, France. camille.lacroux@mnhn.fr.
(5)UMR 2000, Ecology and Emergence of Arthropod-Borne Pathogens, Institut Pasteur/CNRS/Université Paris-Cité, 75015, Paris, France.
(6)Animal Health Department, INRAE, 37380, Nouzilly, France.
(7)UMR 7179 CNRS/MNHN, Mécanismes Adaptatifs et Evolution, Museum National d'Histoire Naturelle, 57 Rue Cuvier, 75231, Paris, France.
(8)UMR 5290 MIVEGEC (Maladies Infectieuses et Vecteurs : Ecologie, Génétique, Evolution et Contrôle), CNRS/IRD/Université de Montpellier, 911 Avenue Agropolis, 34394, Montpellier, France.
(9)MEEDiN (Montpellier Ecology and Evolution of Disease Network), Montpellier, France.

(10)UMR BIPAR ANSES-INRAE-EnvA, Laboratoire Santé Animale, 94701, Maisons-Alfort, France.
(11)Sebitoli Chimpanzee Project, Great Ape Conservation Project, Kibale National Park, Fort Portal, Uganda.
(12)Uganda Wildlife Authority, Plot 7 Kira Road, Kamwokya, Kampala City, Uganda.
(13)UMR 7206 CNRS/MNHN/P7, Eco-anthropologie, Museum National d'Histoire Naturelle, Musée de l'Homme, 17 Place du Trocadéro, 75116, Paris, France. sabrina.krief@mnhn.fr.
(14)Sebitoli Chimpanzee Project, Great Ape Conservation Project, Kibale National Park, Fort Portal, Uganda. sabrina.krief@mnhn.fr.
(15)UMR 5290 MIVEGEC (Maladies Infectieuses et Vecteurs : Ecologie, Génétique, Evolution et Contrôle), CNRS/IRD/Université de Montpellier, 911 Avenue Agropolis, 34394, Montpellier, France. olivier.duron@ird.fr.
BACKGROUND: Ticks and tick-borne pathogens significantly impact both human and animal health and therefore are of major concern to the scientific community. Knowledge of tick-borne pathogens is crucial for prescription of mitigation measures. In Africa, much research on ticks has focused on domestic animals. Little is known about ticks and their pathogens in wild habitats and wild animals like the endangered chimpanzee, our closest relative.
METHODS: In this study, we collected ticks in the forested habitat of a community of 100 chimpanzees living in Kibale National Park, Western Uganda, and assessed how their presence and abundance are influenced by environmental factors. We used non-invasive methods of flagging the vegetation and visual search of ticks both on human team members and in chimpanzee nests. We identified adult and nymph ticks through morphological features. Molecular techniques were used to detect and identify tick-borne piroplasmids and bacterial pathogens.
RESULTS: A total of 470 ticks were collected, which led to the identification of seven tick species: Haemaphysalis parmata (68.77%), Amblyomma tholloni (20.70%), Ixodes rasus sensu lato (7.37%), Rhipicephalus dux (1.40%), Haemaphysalis punctaleachi (0.70%), Ixodes muniensis (0.70%) and Amblyomma paulopunctatum (0.35%). The presence of ticks, irrespective of species, was influenced by temperature and type of vegetation but not by relative humidity. Molecular detection revealed the presence of at least six genera of tick-borne pathogens (Babesia, Theileria, Borrelia, Cryptoplasma, Ehrlichia and Rickettsia). The Afrotopical tick Amblyomma tholloni found in one chimpanzee nest was infected by Rickettsia sp.
CONCLUSIONS: In conclusion, this study presented ticks and tick-borne pathogens in a Ugandan wildlife habitat whose potential effects on animal health remain to be elucidated.

53. Pathogens. 2023 Feb 10;12(2):295. doi: 10.3390/pathogens12020295.
East Coast Fever Carrier Status and Theileria parva Breakthrough Strains in Recently ITM Vaccinated and Non-Vaccinated Cattle in Iganga District, Eastern Uganda.
Oligo S(1), Nanteza A(1), Nsubuga J(1), Musoba A(1), Kazibwe A(1), Lubega GW(1).

Author information:
(1)College of Veterinary Medicine, Animal Resources and Biosecurity (COVAB), Makerere University, Kampala P.O. Box 7062, Uganda.
East Coast fever (ECF) is a tick-borne disease of cattle that hinders the development of the livestock industry in eastern, central and southern Africa. The 'Muguga cocktail' live vaccine, delivered by an infection and treatment method (ITM), remains the only immunisation strategy of controlling ECF. However, there are challenges of the live vaccine inducing ECF carrier status in immunised animals and the possibility of lack of protection from parasite strains that are antigenically different from the vaccine strains. In Uganda, there are insufficient data regarding the ECF carrier status and T. parva genetic diversity in vaccinated and associated non-vaccinated cattle to assess the effectiveness of ITM vaccination. Blood was collected from recently ECF vaccinated (98) and non-vaccinated (73) cattle from Iganga district in Eastern Uganda at 120 days post-vaccination. The p104 gene nested PCR was used to screen for T. parva DNA, 11 minisatellite and 3 microsatellite markers (SSR) were used for genotyping. Two minisatellite markers (MS7 and MS19) were used to determine whether ECF carrier status was due to the T. parva vaccine or local strains. The prevalence of T. parva based on p104 nPCR was 61.2% (60/98) (RR 2.234, 95% CI 1.49-3.35, p-value < 0.001) among recently vaccinated cattle and 27.4% (20/73) (RR 1.00) among associated non-vaccinated cattle. The Muguga cocktail vaccine strains were responsible for carrier status in 10 (58.8%) by MS7 and 11 (64.7%) by MS19 in vaccinated cattle. Genotypes of T. parva with different-sized alleles to the vaccine strains that could be potential 'breakthroughs' were detected in 2 (11.8%)) and 4 (23.5%) isolates from vaccinated cattle based on MS7 and MS19 minisatellite markers, respectively. Using 14 SSR markers, T. parva diversity was higher in vaccinated (Na = 2.214, Ne = 1.978, He = 0.465) than associated non-vaccinated (Na = 1.071, Ne = 1.048, He = 0.259) cattle. The principal component analysis (PCA) showed isolates from vaccinated cattle were closely related to those from non-vaccinated cattle. The analysis of molecular variance (AMOVA) revealed high genetic variation (96%) within T. parva isolates from vaccinated and non-vaccinated cattle but low variation (4%) between vaccinated and non-vaccinated cattle. This study reveals the role of ITM in inducing the carrier status and higher T. parva genetic diversity in vaccinated cattle. The low genetic variation between T. parva isolates in both vaccinated and non-vaccinated cattle may be suggestive of the protective role of vaccine strains against genetically related local strains in the study area.

54. Insects. 2023 Feb 23;14(3):221. doi: 10.3390/insects14030221.
Mosquito-Borne Diseases and Their Control Strategies: An Overview Focused on Green Synthesized Plant-Based Metallic Nanoparticles.
Onen H(1), Luzala MM(2)(3), Kigozi S(4), Sikumbili RM(3)(5), Muanga CK(2)(3), Zola EN(2)(3), Wendji SN(2)(3), Buya AB(2)(3), Balciunaitiene A(6), Viškelis J(6), Kaddumukasa MA(4), Memvanga PB(2)(3).
Author information:
(1)Department of Entomology, Uganda Virus Research Institute, Plot 51/59 Nakiwogo Road, Entebbe P.O. Box 49, Uganda.

(2)Laboratory of Pharmaceutics and Phytopharmaceutical Drug Development, Faculty of Pharmaceutical Sciences, University of Kinshasa, Kinshasa B.P. 212, Democratic Republic of the Congo.
(3)Centre de Recherche et d'Innovation Technologique en Environnement et en Sciences de la Santé (CRITESS), University of Kinshasa, Kinshasa B.P. 212, Democratic Republic of the Congo.
(4)Department of Biological Sciences, Faculty of Science, Kyambogo University, Kampala P.O. Box 1, Uganda.
(5)Department of Chemistry, Faculty of Science, University of Kinshasa, Kinshasa B.P. 190, Democratic Republic of the Congo.
(6)Institute of Horticulture, Lithuanian Research Centre for Agriculture and Forestry, 54333 Babtai, Lithuania.
Mosquitoes act as vectors of pathogens that cause most life-threatening diseases, such as malaria, Dengue, Chikungunya, Yellow fever, Zika, West Nile, Lymphatic filariasis, etc. To reduce the transmission of these mosquito-borne diseases in humans, several chemical, biological, mechanical, and pharmaceutical methods of control are used. However, these different strategies are facing important and timely challenges that include the rapid spread of highly invasive mosquitoes worldwide, the development of resistance in several mosquito species, and the recent outbreaks of novel arthropod-borne viruses (e.g., Dengue, Rift Valley fever, tick-borne encephalitis, West Nile, yellow fever, etc.).
Therefore, the development of novel and effective methods of control is urgently needed to manage mosquito vectors. Adapting the principles of nanobiotechnology to mosquito vector control is one of the current approaches. As a single-step, eco-friendly, and biodegradable method that does not require the use of toxic chemicals, the green synthesis of nanoparticles using active toxic agents from plant extracts available since ancient times exhibits antagonistic responses and broad-spectrum target-specific activities against different species of vector mosquitoes. In this article, the current state of knowledge on the different mosquito control strategies in general, and on repellent and mosquitocidal plant-mediated synthesis of nanoparticles in particular, has been reviewed. By doing so, this review may open new doors for research on mosquito-borne diseases.

55. Parasit Vectors. 2023 Mar 30;16(1):117. doi: 10.1186/s13071-023-05709-0.
Tick-borne pathogens and body condition of cattle in smallholder rural livestock production systems in East and West Africa.
Heylen DJA(1)(2)(3), Kumsa B(4), Kimbita E(5), Frank MN(6), Muhanguzi D(6), Jongejan F(7), Adehan SB(8), Toure A(9), Aboagye-Antwi F(10), Ogo NI(11), Juleff N(12), Crafford D(13), Fourie J(13), Labuchange M(14), Madder M(15).
Author information:
(1)Evolutionary Ecology Group, Department of Biology, University of Antwerp, Wilrijk, Belgium. dieter.heylen@uantwerpen.be.
(2)Eco-Epidemiology Group, Department of Biomedical Sciences, Institute of Tropical Medicine, Antwerp, Belgium. dieter.heylen@uantwerpen.be.
(3)Interuniversity Institute for Biostatistics and Statistical Bioinformatics, Hasselt University, Diepenbeek, Belgium. dieter.heylen@uantwerpen.be.

(4)Department of Parasitology, College of Veterinary Medicine and Agriculture, Addis Ababa University, Bishoftu, Ethiopia.
(5)Department of Veterinary Microbiology and Parasitology, College of Veterinary Medicine and Biomedical Sciences, Sokoine University of Agriculture, PO Box 3019, Morogoro, Tanzania.
(6)Department of Bio-Molecular Resources and Bio-Laboratory Sciences (BBS), College of Veterinary Medicine, Makerere University, Kampala, Uganda.
(7)Department of Veterinary Tropical Diseases, Faculty of Veterinary Science, University of Pretoria, Onderstepoort, South Africa.
(8)Zootechnical, Veterinary and Halieutic Research Laboratory (LRZVH), National Institute of Agricultural Research (INRAB), 01 BP 884, Cotonou, Benin.
(9)Université Nangui Abrogoua, UFR Sciences de La Nature, 02 Bp 801, Abidjan 02, Côte d'Ivoire.
(10)Department of Animal Biology and Conservation Science, School of Biological Sciences, College of Basic and Applied Sciences, University of Ghana, Legon-Accra, Ghana.
(11)National Veterinary Research Institute, Vom, Plateau State, Nigeria.
(12)Bill & Melinda Gates Foundation, Seattle, WA, USA.
(13)Clinvet International Pty (Ltd), 1479 Talmadge Hill South, Waverly, NY, 14892, USA.
(14), Clinomics, Bloemfontein, South Africa.
(15)Clinglobal, B03/04, The Tamarin Commercial Hub, Jacaranda Avenue, Tamarin, 90903, Mauritius.
BACKGROUND: The majority of the African population lives in rural areas where they heavily depend on crop and livestock production for their livelihoods. Given their socio-economic importance, we initiated a standardized multi-country (Benin, Burkina Faso, Ghana, Nigeria, Ethiopia Tanzania and Uganda) surveillance study to assess the current status of important tick-borne haemoparasites (TBHPs) of cattle.
METHODS: We assessed pathogen prevalences (Anaplasma marginale, Anaplasma centrale, Babesia bigemina, Babesia bovis, Ehrlichia ruminantium, and Theileria parva) in the blood of 6447 animals spread over fourteen districts (two districts per country). In addition, we screened for intrinsic (sex, weight, body condition) and extrinsic (husbandry, tick exposure) risk factors as predictors of infections with TBHPs.
RESULTS: There was a large macro-geographic variation observed in A. marginale, B. bigemina, B. bovis and E. ruminantium prevalences. Most correlated with the co-occurrence of their specific sets of vector-competent ticks. Highest numbers of infected cattle were found in Ghana and Benin, and lowest in Burkina Faso. While T. parva was seldomly found (Uganda only: 3.0%), A. marginale was found in each country with a prevalence of at least 40%. Babesia bovis infected individuals had lower body condition scores. Age (as estimated via body weight) was higher in A. marginale infected cattle, but was negatively correlated with B. bigemina and E. ruminantium prevalences. Ehrlichia ruminantium infection was more often found in males, and A. marginale more often in transhumance farming. High levels of co-infection, especially the combination A. marginale × B. bigemina, were observed in all countries, except for Uganda and Burkina Faso.

Babesia bigemina was more or less often observed than expected by chance, when cattle were also co-infected with E. ruminantium or A. marginale, respectively. CONCLUSIONS: Tick-borne pathogens of cattle are ubiquitous in African's smallholder cattle production systems. Our standardized study will help a wide range of stakeholders to provide recommendations for TBHP surveillance and prevention in cattle, especially for B. bovis which heavily impacts production and continues its spread over the African continent via the invasive Rhipicephalus microplus tick.

56. Parasite. 2023;30:13. doi: 10.1051/parasite/2023014. Epub 2023 May 10.
Diversity and phylogeny of the tick-borne bacterial genus Candidatus Allocryptoplasma (Anaplasmataceae).
Ouass S(1), Boulanger N(2), Lelouvier B(3), Insonere JL(3), Lacroux C(4), Krief S(5), Asalu E(6), Rahola N(1), Duron O(1).
Author information:
(1)MIVEGEC, University of Montpellier (UM), Centre National de la Recherche Scientifique (CNRS), Institut pour la Recherche de la Développement (IRD), 34394 Montpellier, France.
(2)University of Strasbourg, French National Reference Center for Borrelia, Hôpitaux Universitaires de Strasbourg, Strasbourg, France.
(3)Vaiomer, 516 rue Pierre et Marie Curie, 31670 Labège, France.
(4)UMR 7206 CNRS/MNHN/P7, Eco-anthropologie, Muséum National d'Histoire Naturelle, Musée de l'Homme, 17 place du Trocadéro, 75116, Paris, France - La Phocéenne de Cosmétique, ZA Les Roquassiers, 174 Rue de la Forge, 13300 Salon-de-Provence, France - Sebitoli Chimpanzee Project, Great Ape Conservation Project, Kibale National Park, Fort Portal, Uganda.
(5)UMR 7206 CNRS/MNHN/P7, Eco-anthropologie, Muséum National d'Histoire Naturelle, Musée de l'Homme, 17 place du Trocadéro, 75116, Paris, France - Sebitoli Chimpanzee Project, Great Ape Conservation Project, Kibale National Park, Fort Portal, Uganda.
(6)Sebitoli Chimpanzee Project, Great Ape Conservation Project, Kibale National Park, Fort Portal, Uganda - Uganda Wildlife Authority, Kibale National Park, Uganda.
The family Anaplasmataceae includes tick-borne bacteria of major public and veterinary health interest, as best illustrated by members of the genera Anaplasma and Ehrlichia. Recent epidemiological surveys have also reported on the presence of a novel putative genus in the Anaplasmataceae, Candidatus Allocryptoplasma, previously described as Candidatus Cryptoplasma in the western black-legged tick, Ixodes pacificus. However, the genetic diversity of Ca. Allocryptoplasma and its phylogenetic relationship with other Anaplasmataceae remain unclear. In this study, we developed a multi-locus sequence typing approach, examining the DNA sequence variation at five genes of Ca. Allocryptoplasma found in ticks. Combining this multi-locus sequence typing and genetic data available on public databases, we found that substantial genetic diversity of Ca. Allocryptoplasma is present in Ixodes, Amblyomma and Haemaphysalis spp. ticks on most continents. Further analyses confirmed that the Ca. Allocryptoplasma of ticks, the Ca. Allocryptoplasma of lizards and some

Anaplasma-like bacteria of wild mice cluster into a monophyletic genus, divergent from all other genera of the family Anaplasmataceae. Candidatus Allocryptoplasma appears as a sister genus of Anaplasma and, with the genera Ehrlichia and Neoehrlichia, they form a monophyletic subgroup of Anaplasmataceae associated with tick-borne diseases. The detection of genetically distinct Ca. Allocryptoplasma in ticks of significant medical or veterinary interest supports the hypothesis that it is an emergent genus of tick-borne pathogens of general concern.

57. Parasit Vectors. 2023 Jun 19;16(1):206. doi: 10.1186/s13071-023-05801-5.
Tick communities of cattle in smallholder rural livestock production systems in sub-Saharan Africa.
Heylen DJA(1)(2), Kumsa B(3), Kimbita E(4), Frank MN(5), Muhanguzi D(5), Jongejan F(6), Adehan SB(7), Toure A(8), Aboagye-Antwi F(9), Ogo NI(10), Juleff N(11), Fourie J(12), Evans A(13), Byaruhanga J(14), Madder M(13).
Author information:
(1)Evolutionary Ecology Group, Department of Biology, University of Antwerp, Wilrijk, Belgium. dieter.heylen@uantwerpen.be.
(2)Interuniversity Institute for Biostatistics and Statistical Bioinformatics, Hasselt University, Diepenbeek, Belgium. dieter.heylen@uantwerpen.be.
(3)Department of Parasitology, College of Veterinary Medicine and Agriculture, Addis Ababa University, Bishoftu, Ethiopia.
(4)Department of Veterinary Microbiology and Parasitology, College of Veterinary Medicine and Biomedical Sciences, Sokoine University of Agriculture, 3019, Morogoro, Tanzania.
(5)Department of Bio-molecular Resources and Bio-Laboratory Sciences (BBS), College of Veterinary Medicine, Makerere University, Kampala, Uganda.
(6)Department of Veterinary Tropical Diseases, Faculty of Veterinary Science, University of Pretoria, Onderstepoort, South Africa.
(7)National Institute of Agricultural Research (INRAB), Zootechnical, Veterinary and Halieutic Research Laboratory (LRZVH), 01 BP 884, Cotonou, Benin.
(8)Université Nangui Abrogoua, UFR Sciences de la Nature, 02 Bp 801, Abidjan 02, Côte d'Ivoire.
(9)Department of Animal Biology and Conservation Science, School of Biological Sciences, College of Basic and Applied Sciences, University of Ghana, Legon-Accra, Ghana.
(10)National Veterinary Research Institute, Vom, Plateau State, Nigeria.
(11)Bill & Melinda Gates Foundation, Seattle, WA, USA.
(12)Clinvet International Pty (Ltd), 1479 Talmadge Hill South, Waverly, NY, 14892, USA.
(13)Clinglobal, B03/04, The Tamarin Commercial Hub, Jacaranda Avenue, Tamarin, 90903, Mauritius.
(14)Research Center for Tropical Diseases and Vector Control (RTC), Department of Veterinary Pharmacy, Clinics and Comparative Medicine, School of Veterinary Medicine and Animal Resources, College of Veterinary Medicine, Animal Resources and Biosecurity, Makerere University, Kampala, Uganda.

Erratum in
Parasit Vectors. 2023 Aug 9;16(1):272.
Parasit Vectors. 2023 Nov 16;16(1):423.
BACKGROUND: The majority of the African population lives in rural areas and depends on agriculture for their livelihoods. To increase the productivity and sustainability of their farms, they need access to affordable yield-enhancing inputs of which parasite control is of paramount importance. We therefore determined the status of current tick species with the highest economic impact on cattle by sampling representative numbers of animals in each of seven sub-Saharan countries.
METHODS: Data included tick species' half-body counts from approximately 120 cattle at each of two districts per country, collected four times in approximately 1 year (to include seasonality). Study sites were chosen in each country to include high cattle density and tick burden.
RESULTS: East Africa (Ethiopia, Uganda and Tanzania) showed overall a higher diversity and prevalence in tick infestations compared to West African countries (Benin, Burkina Faso, Ghana and Nigeria). In East Africa, Amblyomma variegatum (vector of Ehrlichia ruminantium), Rhipicephalus microplus (Babesia bovis, B. bigemina, Anaplasma marginale), R. evertsi evertsi (A. marginale) and R. appendiculatus (Theileria parva) were the most prevalent tick species of economic importance. While the latter species was absent in West Africa, here both A. variegatum and R. microplus occurred in high numbers. Rhipicephalus microplus had spread to Uganda, infesting half of the cattle sampled. Rhipicephalus appendiculatus is known for its invasive behaviour and displacement of other blue tick species, as observed in other East and West African countries. Individual cattle with higher body weights, as well as males, were more likely to be infested. For six tick species, we found reduced infestation levels when hosts were treated with anti-parasiticides.
CONCLUSIONS: These baseline data allow the determination of possible changes in presence and prevalence of ticks in each of the countries targeted, which is of importance in the light of human-caused climate and habitat alterations or anthropogenic activities. As many of the ticks in this study are vectors of important pathogens, but also, as cattle may act as end hosts for ticks of importance to human health, our study will help a wide range of stakeholders to provide recommendations for tick infestation surveillance and prevention.

58. Pathogens. 2023 Jun 26;12(7):875. doi: 10.3390/pathogens12070875.
Acaricide Resistance Development in Rhipicephalus (Boophilus) microplus (Acari: Ixodidae) Populations against Amitraz and Deltamethrin on Communal Farms of the King Sabata Dalindyebo Municipality, South Africa.
Dzemo WD(1)(2), Vudriko P(3), Ramatla T(2), Thekisoe O(2).
Author information:
(1)Department of Biological and Environmental Sciences, Faculty of Natural Sciences, Walter Sisulu University, Mthatha 5117, South Africa.
(2)Unit for Environmental Sciences and Management, North-West University, Potchefstroom 2531, South Africa.
(3)Research Center for Tropical Diseases and Vector Control, Department of

Veterinary Pharmacy, Clinics and Comparative Medicine, College of Veterinary Medicine, Animal Resources and Biosecurity, Makerere University, Kampala P.O. Box 7062, Uganda.
Chemical acaricides are widely used to control ticks and tick-borne pathogens in cattle. However, prolonged and indiscriminate use of these chemicals inevitably leads to the selection of resistant ticks. In-vitro bioassays (adult and larval immersion tests) were conducted to assess amitraz and deltamethrin resistance in Rhipicephalus (Boophilus) microplus populations from communal farms of the King Sabata Dalindyebo municipality of South Africa. Data generated on percentage inhibition of oviposition (%IO) revealed that all the tick populations assessed showed resistance (%IO ≤ 95%) to at least one of the acaricides. All six tick populations assessed for efficacy (%IO ≥ 95%) at the DD) with deltamethrin were resistant (%IO ≤ 95%) and only one of the six tick populations assessed for efficacy with amitraz was susceptible. Based on the resistance ratios (RR), the adult immersion test detected amitraz and deltamethrin resistance in three (RR ranging from 2.30 to 3.21) and five (RR ranging from 4.10 to 14.59) of the six tick populations, respectively. With the larval immersion test, deltamethrin and amitraz resistance (larval mortality < 90% at the DD) was detected in all four and three of four R. (B.) microplus populations assessed, respectively. These data are critical for the design of an effective and sustainable tick control strategy on the communal farms.

59. Pathogens. 2023 Oct 19;12(10):1258. doi: 10.3390/pathogens12101258.
Perception of Ticks and Tick-Borne Diseases Worldwide.
de la Fuente J(1)(2), Estrada-Peña A(3)(4), Rafael M(1), Almazán C(5), Bermúdez S(6), Abdelbaset AE(7), Kasaija PD(8), Kabi F(8), Akande FA(9), Ajagbe DO(10), Bamgbose T(11), Ghosh S(12)(13), Palavesam A(14), Hamid PH(15), Oskam CL(16)(17), Egan SL(16)(17), Duarte-Barbosa A(17)(18), Hekimoğlu O(19), Szabó MPJ(20), Labruna MB(21), Dahal A(22).
Author information:
(1)SaBio, Instituto de Investigación en Recursos Cinegéticos, IREC-CSIC-UCLM-JCCM, Ronda de Toledo 12, 13005 Ciudad Real, Spain.
(2)Department of Veterinary Pathobiology, Center for Veterinary Health Sciences, Oklahoma State University, Stillwater, OK 74078, USA.
(3)Department of Animal Health, Faculty of Veterinary Medicine, University of Zaragoza, 50013 Zaragoza, Spain.
(4)Research Group in Emerging Zoonoses, Instituto Agroalimentario de Aragón-IA2, Universidad de Zaragoza-CITA, 50013 Zaragoza, Spain.
(5)Facultad de Ciencias Naturales, Universidad Autonóma de Querétaro, Avenida de las Ciencias S/N Juriquilla, Querétaro 76230, Mexico.
(6)Medical Entomology Research Department, Gorgas Memorial Institute for Health Research, Panama City 0816-02593, Panama.
(7)Laboratory of Parasitology, Graduate School of Infectious Diseases, Faculty of Veterinary Medicine, Hokkaido University, Kita-18, Nishi-9, Sapporo 060-0818, Hokkaido, Japan.
(8)National Livestock Resources Research Institute (NaLIRRI/NARO), Kampala P.O. Box 5704, Uganda.

(9)Department of Veterinary Parasitology and Entomology, College of Veterinary Medicine, Federal University of Agriculture, Abeokuta 111101, Ogun State, Nigeria.
(10)Department of Pure and Applied Zoology, College of Biological Sciences, Federal University of Agriculture, Abeokuta 111101, Ogun State, Nigeria.
(11)Department of Biological Sciences, Microbiology Unit, Faculty of Science, Kings University, Ode-Omu City 221102, Osun State, Nigeria.
(12)Entomology Laboratory, Indian Veterinary Research Institute, Izatnagar, Bareilly 243122, Uttar Pradesh, India.
(13)IVRI-Eastern Regional Station, 37, Belgachia Road, Kolkata 700037, West Bengal, India.
(14)Translational Research Platform for Veterinary Biologicals, Centre for Animal Health Studies, Tamil Nadu Veterinary and Animal Sciences University, Chennai 600051, Tamil Nadu, India.
(15)Department of Animal Science, Universitas Sebelas Maret, Surakarta 57126, Indonesia.
(16)School of Medical, Molecular and Forensic Sciences, Murdoch University, Perth, WA 6150, Australia.
(17)Centre for One Health and Biosecurity, Harry Butler Institute, Murdoch University, Perth, WA 6150, Australia.
(18)School of Veterinary Medicine, Murdoch University, Perth, WA 6150, Australia.
(19)Division of Ecology, Faculty of Science, Hacettepe University, Beytepe, Ankara 06800, Turkey.
(20)Laboratório de Ixodologia, Faculdade de Medicina Veterinária, Universidade Federal de Uberlândia, Av. Pará, 1720/Campus Umuarama-Bloco 2T, Uberlândia 38400-902, Brazil.
(21)Faculty of Veterinary Medicine and Animal Science, University of São Paulo, Sao Paulo 05508-220, Brazil.
(22)Department of Microbiology and Parasitology, Faculty of Animal Science, Veterinary Science and Fisheries, Agriculture and Forestry University, Chitwan 44200, Nepal.
In this comprehensive review study, we addressed the challenge posed by ticks and tick-borne diseases (TBDs) with growing incidence affecting human and animal health worldwide. Data and perspectives were collected from different countries and regions worldwide, including America, Europe, Africa, Asia, and Oceania. The results updated the current situation with ticks and TBD and how it is perceived by society with information bias and gaps. The study reinforces the importance of multidisciplinary and international collaborations to advance in the surveillance, communication and proposed future directions to address these challenges.

60. J Parasitol Res. 2023 Oct 28;2023:7074041. doi: 10.1155/2023/7074041.
Ectoparasite Fauna of Rodents and Shrews with Their Spatial, Temporal, and Dispersal along a Degradation Gradient in Mabira Central Forest Reserve.
Babyesiza WS(1)(2)(3), Mpagi J(4), Ssuuna J(1)(2), Akoth S(1)(2)(3), Katakweba A(1)(5).

Author information:
(1)Africa Centre of Excellence for Innovative Rodent Pest Management and Biosensor Technology Development (ACE IRPM&BTD), Uganda.
(2)Department of Wildlife Management, Sokoine University of Agriculture, Morogoro, Tanzania.
(3)Department of Zoology, Entomology and Fisheries Science, Makerere University, Uganda.
(4)Department of Microbiology and Immunology, Busitema University, Uganda.
(5)Institute of Pest Management, Sokoine University of Agriculture, Morogoro, Tanzania.
Ectoparasites like fleas, mites, and ticks that are key carriers of harmful pathogens such as viruses, bacteria, cestodes, and nematodes live on rodents and shrews. It should be noted that rodents' ecological adaptability makes them suitable as parasite hosts. The main objective of the study was to determine the ectoparasite assemblages in rodents and shrews along a degradation gradient, while comparing infestation levels in different habitats with varying levels of degradation. The study was conducted in Mabira Central Forest Reserve. Ectoparasites were collected following rodent and shrew removal trapping which was done using Sherman's traps set along transects of 200 meters in three habitat strata that included adjacent forest habitats, degraded forest edge, and regenerating forest interior. Data was collected intermittently with a break every two months for one year from November 2018 to December 2019. A total of 1411 rodents and shrews were collected, yielding a total of 5692 ectoparasites from 22 host species (17 rodents and 5 shrews). The most prevalent group of ectoparasites was mites followed by fleas, lice, ticks, and earwig. Ectoparasite prevalence significantly differed depending on hosts species (P = 0.001) and host age (P = 0.022), but not host sex (P = 0.78), while mean infestation significantly varied basing on host species (P = 0.001), host sex (P = 0.001), season (P = 0.001), and habitat (P = 0.001). Prevalence (P = 0.001) and mean infestation (P = 0.001) significantly varied across studied habitats. The study has emphasized the significance of Praomys jacksoni and Hylomyscus stella as significant hosts for mites and S. congicus as a significant host for fleas. Additionally, environment and host characteristics have a bearing on prevalence and infestation of ectoparasites with habitat degradation playing a significant role in the occurrence of ectoparasites, thereby emphasizing its contribution to zoonotic outbreaks.

61. PLoS One. 2023 Nov 9;18(11):e0288587. doi: 10.1371/journal.pone.0288587.
Seroepidemiological investigation of Crimean Congo hemorrhagic fever virus in livestock in Uganda, 2017.
Nyakarahuka L(1)(2), Kyondo J(1), Telford C(3), Whitesell A(3), Tumusiime A(1), Mulei S(1), Baluku J(1), Cossaboom CM(3), Cannon DL(3), Montgomery JM(3), Lutwama JJ(1), Nichol ST(3), Balinandi SK(1), Klena JD(3), Shoemaker TR(3).
Author information:
(1)Department of Arbovirology, Emerging and Reemerging Infectious Diseases, Uganda Virus Research Institute, Entebbe, Uganda.
(2)Department of Biosecurity, Ecosystems and Veterinary Public Health, College

of Veterinary Medicine, Animal Resources and Biosecurity, Makerere University, Kampala, Uganda.
(3)Viral Special Pathogens Branch, Division of High-Consequence Pathogens and Pathology, United States Centers for Disease Control and Prevention, Atlanta, Georgia, United States of America.
Crimean-Congo Hemorrhagic fever (CCHF) is an important zoonotic disease transmitted to humans both by tick vectors and contact with fluids from an infected animal or human. Although animals are not symptomatic when infected, they are the main source of human infection. Uganda has reported sporadic human outbreaks of CCHF in various parts of the country since 2013. We designed a nationwide epidemiological study to investigate the burden of CCHF in livestock. A total of 3181 animals were sampled; 1732 cattle (54.4%), 1091 goats (34.3%), and 358 sheep (11.3%) resulting in overall livestock seropositivity of IgG antibodies against CCHF virus (CCHFV) of 31.4% (999/3181). Seropositivity in cattle was 16.9% and in sheep and goats was 48.8%. Adult and juvenile animals had higher seropositivity compared to recently born animals, and seropositivity was higher in female animals (33.5%) compared to male animals (24.1%). Local breeds had higher (36.8%) compared to exotic (2.8%) and cross breeds (19.3%). Animals that had a history of abortion or stillbirth had higher seropositivity compared to those without a history of abortion or stillbirth. CCHFV seropositivity appeared to be generally higher in northern districts of the country, though spatial trends among sampled districts were not examined. A multivariate regression analysis using a generalized linear mixed model showed that animal species, age, sex, region, and elevation were all significantly associated with CCHFV seropositivity after adjusting for the effects of other model predictors. This study shows that CCHFV is actively circulating in Uganda, posing a serious risk for human infection. The results from this study can be used to help target surveillance efforts for early case detection in animals and limit subsequent spillover into humans.

62. One Health. 2023 Jun 12;17:100576. doi: 10.1016/j.onehlt.2023.100576.
Spatial prediction of Crimean Congo hemorrhagic fever virus seroprevalence among livestock in Uganda.
Telford C(1)(2), Nyakarahuka L(3)(4), Waller L(5), Kitron U(5), Shoemaker T(1).
Author information:
(1)Viral Special Pathogens Branch, Centers for Disease Control and Prevention, 1600 Clifton Rd, Atlanta, GA 30333, USA.
(2)Gillings School of Global Public Health, University of North Carolina, 135 Dauer Dr, Chapel Hill, NC 27599, USA.
(3)Uganda Virus Research Institute, 51-59 Nakiwogo Road, Entebbe, Uganda.
(4)Department of Biosecurity, Ecosystems and Veterinary Public Health, Makerere University, Kampala Uganda, 7062 University Rd, Kampala, Uganda.
(5)Rollins School of Public Health, Emory University, 1518 Clifton Rd, Atlanta, GA 30322, USA.
Crimean-Congo Hemorrhagic Fever (CCHF) is a viral disease that can infect humans via contact with tick vectors or livestock reservoirs and can cause moderate to severe disease. The first human case of CCHF in Uganda was identified in 2013.

To determine the geographic distribution of the CCHF virus (CCHFV), serosampling among herds of livestock was conducted in 28 Uganda districts in 2017. A geostatistical model of CCHF seroprevalence among livestock was developed to incorporate environmental and anthropogenic variables associated with elevated CCHF seroprevalence to predict CCHF seroprevalence on a map of Uganda and estimate the probability that CCHF seroprevalence exceeded 30% at each prediction location. Environmental and anthropogenic variables were also analyzed in separate models to determine the spatially varying drivers of prediction and determine which covariate class resulted in best prediction certainty. Covariates used in the full model included distance to the nearest croplands, average annual change in night-time light index, percent sand soil content, land surface temperature, and enhanced vegetation index. Elevated CCHF seroprevalence occurred in patches throughout the country, being highest in northern Uganda. Environmental covariates drove predicted seroprevalence in the full model more than anthropogenic covariates. Combination of environmental and anthropogenic variables resulted in the best prediction certainty. An understanding of the spatial distribution of CCHF across Uganda and the variables that drove predictions can be used to prioritize specific locations and activities to reduce the risk of future CCHF transmission.

tickANDUgandaANDvaccine-set

1. Vopr Virusol. 1988 Jul-Aug;33(4):461-5.
[Monoclonal antibodies to flavivirus antigens induced by a vaccinal strain of yellow fever].[Article in Russian]
Gaĭdamovich SIa, Mel'nikova EE, Mikheeva TG, Shutkova TM, Sveshnikova NA.
Monoclonal antibodies (MABs) YEL-2 induced by the vaccine FNS Dakar yellow fever (YF) virus were characterized for their capacity to enter into serological reactions and to react with heterologous flaviviruses. YEL-2 MABs belong to the IgG2a class of immunoglobulins, possess the antihemagglutination properties, are active in indirect IF test but do not activate complement and have no neutralizing properties. The inability to enter into CFT in the presence of antihemagglutinating properties suggests that YEL-2 MABs are directed for the structural E glycoprotein. YEL-2 MABs reacted similarly with the vaccine 17D strain and the FNS Dakar strain by which they had been induced. In addition to YF virus, YEL-2 MABs reacted with Tyuleniy, Rosio, Ilhéus, Uganda S, Karshi, and Sokuluk viruses, the reaction with Tyuleniy virus reaching the same titer as with the homologous virus but was of one-way nature. No reaction of YEL-2 MABs was observed with the viruses of the tick-borne encephalitis complex, Japanese encephalitis, West Nile, Dengue 2 and 4 viruses. These results specify the antigenic classification of flaviviruses.

2. Parasitology. 1995 Jul;111 (Pt 1):39-49. doi: 10.1017/s0031182000064581.
Generation and characterization of cloned Theileria parva parasites.
Morzaria SP(1), Dolan TT, Norval RA, Bishop RP, Spooner PR.
Author information:
(1)International Livestock Research Institute, Nairobi, Kenya.
A 3-step procedure for cloning Theileria parva parasites was developed. The first step involved the in vitro infection of a fixed number of bovine lymphocytes with titrated sporozoites. The cell lines obtained from infections initiated using sporozoite/lymphocyte ratios below 1:100 were then selected for cloning as these contained schizont-infected cells, each of which was derived from infection with a single sporozoite. In the second step, these cell lines were cloned by limiting dilution. As sporozoites infect lymphocytes and transform to induce clonal multiplication, this step produced infected cell lines containing both cloned parasites and cloned lymphocytes. In the third step, the cloned cell lines were used to infect cattle and isolation of the parasite in ticks was made during piroplasm parasitaemia. Finally, sporozoites were harvested from infected ticks and used for further characterization. Sporozoites derived from cloned cell lines of T. parva Muguga, Marikebuni, Boleni, Uganda and buffalo-derived 7014 were characterized using monoclonal antibody profiles, DNA restriction fragment length polymorphism detected using repetitive and telomeric probes, in vivo infectivity and, in one case, cross-immunity studies. Additionally, several distinct schizont-infected lymphocyte clones were isolated from the Muguga, Mariakani and buffalo-derived 7014 stocks. The combined results of the characterization revealed that the cloning procedure selected clones of T. parva from the parental stocks which

were known to contain a mixture of genetically different parasite populations. The cloning method and the clones generated will be of value in studies of the biology of the parasite and in elucidating the strain specificity of immune responses in cattle.

3. Parassitologia. 1999 Sep;41 Suppl 1:73-80.
Development of sero-diagnostic and molecular tools for the control of important tick-borne pathogens of cattle in Africa.
Morzaria SP(1), Katende J, Musoke A, Nene V, Skilton R, Bishop R.
Author information:
(1)International Livestock Research Institute (ILRI), Nairobi, Kenya.
s.morzaria@cgiar.org
Tick-borne diseases (TBDs) are a major economic constraint to livestock production in sub-Saharan Africa. ILRI is focussing on developing a range of products, such as vaccines, diagnostics and decision support services to underpin improved control programmes against these diseases. We have developed three highly sensitive and specific enzyme linked immuno-assays (ELISAs), which allow precise diagnosis of Theileria parva, Babesia bigemina and Anaplasma marginale. These tests have been standardised and validated using defined experimental and field infection sera. Parasite specific recombinant antigens and monoclonal antibodies against bovine immunoglobulins as secondary antibodies have played an important role in in enhancing the sensitivity and specificity of the assays. They have been further evaluated in on-farm longitudinal sero-epidemiological studies to define infection dynamics and disease risks in various farming systems in Kenya and Uganda. In addition, DNA-based tests for differentiation of Theileria species and characterisation of Theileria parva stocks have been developed. These tests have been derived through physical mapping and sequencing of key elements of the T. parva genome, which include repetitive and telomeric regions, minisatellite sequences, antigen genes and a number of random DNA sequences. These tools are currently being deployed in conjunction with field immunisation programmes to determine the biological impact of introducing live vaccines of T. parva on population dynamics.

4. Int J Parasitol. 2004 Apr;34(5):603-13. doi: 10.1016/j.ijpara.2003.12.012.
Application of a reverse line blot assay to the study of haemoparasites in cattle in Uganda.
Oura CA(1), Bishop RP, Wampande EM, Lubega GW, Tait A.
Author information:
(1)Department of Microbiology and Parasitology, Faculty of Vet Medicine, Makerere University, Kampala, Uganda. chrisoura@hotmail.com
Recent advances in genomic technology have focused many veterinary researchers on the possibility of producing one multivalent recombinant vaccine against all the haemoparasites that infect cattle in the tropics. Before such a vaccine is developed it is essential to define target cattle populations as well as the range of anti-pathogen vaccines required in order to control disease. To further this objective, we have evaluated a reverse line blot (RLB) assay, which simultaneously detects the principal tick transmitted protozoan and rickettsial

cattle pathogens, in different epidemiological scenarios in Uganda. A critical question is the sensitivity, particularly in relation to detecting carrier animals. As Theileria parva is considered to be the most important pathogen in the region, we assessed the sensitivity of the RLB assay for T. parva and showed that 1-2 x 10(3) parasites per ml of blood could be detected-a level comparable with previously developed PCR methods and well below conventional microscopic detection. We applied the RLB assay to evaluate the differences in pathogen profiles between crossbred and indigenous cattle and show that there were different profiles, with a low prevalence of T. parva and Theileria taurotragi in the indigenous cattle compared to a high prevalence in the crossbred cattle. In contrast, we show higher prevalences of Theileria mutans and Theileria velifera in the indigenous compared to the crossbred cattle. Interestingly Anaplasma marginale, Babesia bovis and Babesia bigemina were of low prevalence but a high prevalence of Ehrlichia bovis was seen, raising the question of whether this rickettsial species could be pathogenic in cattle. Analysis of animals with clinical symptoms of East Coast Fever showed that, while T. parva is a major cause of these symptoms, T. mutans and possibly T. taurotragi and T. velifera, may also cause clinical disease. Overall, the results presented here highlight the complexity of tick-borne pathogen infections in cattle in Uganda.

5. Parasitology. 2004 Jul;129(Pt 1):27-42. doi: 10.1017/s003118200400513x.
The persistence of component Theileria parva stocks in cattle immunized with the 'Muguga cocktail' live vaccine against East Coast fever in Uganda.
Oura CA(1), Bishop R, Wampande EM, Lubega GW, Tait A.
Author information:
(1)Department of Microbiology and Parasitology, Faculty of Veterinary Medicine, Makerere University, P.O. Box 7062, Kampala, Uganda. chrisoura@hotmail.com
The 'Muguga cocktail' live vaccine comprises three Theileria parva stocks (Muguga, Kiambu 5 and the buffalo-derived Serengeti-transformed) and has been used extensively in Eastern, Central and Southern Africa with an infection and treatment protocol to protect cattle against East Coast fever. We report the characterization of the three component vaccine stocks using a panel of polymorphic micro-satellite and mini-satellite markers and the development of a stock-derived PCR method that distinguishes two of the vaccine stocks. These markers, with the use of a recently developed Reverse Line Blot assay, have enabled us to address four important questions in relation to vaccination. First, how closely related are the vaccine stocks, secondly do all three stocks persist post-vaccination and induce a carrier state, thirdly is there evidence for the transmission of the vaccine stocks and fourthly does vaccination prevent infection with local genotypes? The results show that Muguga and Serengeti-transformed stocks are highly related but very distinct from Kiambu 5 that persists in vaccinated cattle establishing a carrier state. No evidence was obtained for the transmission of vaccine stocks to co-grazed animals, although these animals were infected with up to 8 different T. parva genotypes showing there was a significant level of tick challenge. Some of the vaccinated animals become infected with a subset of local genotypes providing evidence for limited vaccine 'breakthrough'.

6. Trop Anim Health Prod. 2009 Oct;41(7):1299-308. doi: 10.1007/s11250-009-9315-x.
Disease incidence in ranch and pastoral livestock herds around Lake Mburo National Park, in South Western Uganda.
Ocaido M(1), Muwazi RT, Opuda JA.
Author information:
(1)Department of Wildlife and Animal Resource Management, Faculty of Veterinary Medicine, Makerere University, P.O. Box 7062, Kampala, Uganda.
mocaido@vetmed.mak.ac.ug
A longitudinal study was done to monitor incidence of livestock diseases in ranch and pastoral herds around Lake Mburo National Park, in South-western Uganda with a high level of wildlife (impala and zebra) and livestock (cattle and goats) interactions. East Coast Fever (ECF), abortions, helminthosis and starvation due to drought were major livestock disease problems encountered. ECF was a major disease affecting mainly calves, causing mortality both in ranch (8.5%) and pastoral (8.2%) herds. Meanwhile, drought was a more serious in pastoral than ranch herds ($p < 0.05$), killing 28.5% and 5.8% of adult female cattle in respective production systems. Other endemic diseases found and controlled by vaccination were FMD, CBPP and blackquarter. In both cattle and goats helminthosis was causing mortalities, 3.3% and 0.8% among calves in pastoral and ranches respectively; and 4.8% and 6.9% among kids and sub-adult goats respectively. Many cases of abortions occurred among pastoral herds. In conclusion, the disease incidence among livestock was the same or relatively low as compared to other areas without wildlife.

7. Parasit Vectors. 2011 Jul 15;4:137. doi: 10.1186/1756-3305-4-137.
Multi-locus sequence typing of Ehrlichia ruminantium strains from geographically diverse origins and collected in Amblyomma variegatum from Uganda.
Nakao R(1), Magona JW, Zhou L, Jongejan F, Sugimoto C.
Author information:
(1)Department of Collaboration and Education, Research Center for Zoonosis Control, Hokkaido University, Kita 20, Nishi 10, Kita-ku, Sapporo, Hokkaido 001-0020, Japan.
BACKGROUND: The rickettsial bacterium Ehrlichia ruminantium is the causative agent of heartwater in ruminants. A better understanding of the population genetics of its different strains is, however, needed for the development of novel diagnostic tools, therapeutics and prevention strategies. Specifically, the development of effective vaccination policies relies on the proper genotyping and characterisation of field isolates. Although multi-locus sequence typing (MLST) has been recently developed, only strains from geographically restricted collections have been analysed so far. The expansion of the MLST database to include global strains with different geographic origins is therefore essential. In this study, we used a panel of reference strains from geographically diverse origins and field samples of E. ruminantium detected from its vector, Amblyomma variegatum, in heartwater-endemic areas in Uganda.
RESULTS: A total of 31 novel alleles (six, four, six, three, two, five, three, and two for gltA, groEL, lepA, lipA, lipB, secY, sodB, and sucA loci,

respectively) and 19 novel sequence types (STs) were identified. Both neighbour-joining and minimum spanning tree analyses indicated a high degree of genetic heterogeneity among these strains. No association was observed between genotypes and geographic origins, except for four STs from West African countries. When we performed six different tests for recombination (GeneConv, Bootscan, MaxChi, Chimaera, SiScan, and 3Seq) on concatenated sequences, four possible recombination events were identified in six different STs. All the recombination breakpoints were located near gene borders, indicating the occurrence of intergenic recombination. All four STs that localized to a distinct group in clustering analysis showed evidence of identical recombination events, suggesting that recombination may play a significant role in the diversification of E. ruminantium.
CONCLUSIONS: The compilation of MLST data set across the African continent will be particularly valuable for the understanding of the existing genetic diversity of field isolates in African countries. Comprehensive information on the degree of cross-protection between strains and further understanding of possible relationships between genotypes and phenotypes such as vaccine efficacy are expected to lead to the development of region-specific vaccination strategies.

8. Parasitology. 2012 Jan;139(1):69-82. doi: 10.1017/S003118201100165X. Epub 2011
Development of multiple-locus variable-number tandem-repeat analysis for rapid genotyping of Ehrlichia ruminantium and its application to infected Amblyomma variegatum collected in heartwater endemic areas in Uganda.
Nakao R(1), Morrison LJ, Zhou L, Magona JW, Jongejan F, Sugimoto C.
Author information:
(1)Department of Collaboration and Education, Research Center for Zoonosis Control, Hokkaido University, Kita 20, Nishi 10, Kita-ku, Sapporo, Hokkaido 001-0020, Japan.

The rickettsial bacterium Ehrlichia ruminantium is the causative agent of heartwater, a serious tick-borne disease in ruminants. The genetic diversity of organisms in the field will have implications for cross-protective capacities of any vaccine developed, and for an effective vaccine design strategy proper genotyping and understanding of existing genetic diversity in the field is necessary. We searched for variable-number tandem-repeat (VNTR) loci for use in a multi-locus VNTR analysis (MLVA). Sequencing analysis of 30 potential VNTRs using a panel of 17 reference strains from geographically diverse origins identified 12 VNTRs with allelic profiles differing between strains. Application of MLVA to 38 E. ruminantium-infected Amblyomma variegatum collected from indigenous cattle in 6 different districts of Uganda identified 21 MLVA types. The discriminatory power of MLVA was greater than that of map1 PCR-restriction fragment length polymorphism analysis, with which only 6 genotypes were obtained. The high discriminatory power as well as cost- effective performance of MLVA provide the potential for this technique to be applied in the future with respect to optimizing vaccine trials by identifying local strain diversity, and also raise the possibility of exploring the association between E. ruminantium genotypes and phenotypes such as pathological outcome in the

ruminant host.

9. Res Vet Sci. 2016 Dec;109:1-9. doi: 10.1016/j.rvsc.2016.09.001. Epub 2016 Sep 9.
Application of ethnobotanical repellents and acaricides in prevention, control and management of livestock ticks: A review.
Pavela R(1), Canale A(2), Mehlhorn H(3), Benelli G(4).
Author information:
(1)Crop Research Institute, Drnovska 507, 161 06 Prague 6, Czech Republic.
(2)Insect Behaviour Group, Department of Agriculture, Food and Environment, University of Pisa, via del Borghetto 80, 56124 Pisa, Italy.
(3)Department of Parasitology, Heinrich Heine University, Düsseldorf, Germany.
(4)Insect Behaviour Group, Department of Agriculture, Food and Environment, University of Pisa, via del Borghetto 80, 56124 Pisa, Italy. Electronic address: benelli.giovanni@gmail.com.
Ticks transmit at least the same number or even more pathogens than any other group of blood-feeding arthropods worldwide affecting humans and animals. The eco-friendly control and management of tick vectors in a constantly changing environment is a crucial challenge. Besides the development of vaccines against ticks, IPM practices aimed at reducing tick interactions with livestock, emerging pheromone-based control tools, and few biological control agents, the extensive employment of acaricides and tick repellents still remain the most effective and ready-to-use strategies. However, the former is limited by the development of growing resistances as well as environmental concerns. Exploiting plants and plant products as sources of effective tick repellents and acaricides represents a promising strategy. In this scenario, the preservation of ethnobotanical information on repellent and acaricidal potential of plants is crucial. Here, we evaluated relevant information published in recent years, focused on plants used as repellents and acaricides against tick vectors in different regions worldwide. We selected a total of 238 plant species, which are traditionally used against ticks by native and local communities of Africa (Kenya, Uganda, Zimbabwe, South Africa), Europe (Serbia, Macedonia, Romania), Asia (Pakistan, India) and America (Brazil, Canada), from 56 families. However, only 7 families (i.e. Asteraceae, Euphorbiaceae, Fabaceae, Lamiaceae, Meliaceae, Apocynaceae and Solanaceae) represent the major quote (46%) of all plant species. We evaluated the differences in acaricidal and repellent efficacy of different formulations used. In the final section, implications arising from the surveyed anti-tick ethnobotanical knowledge and challenges for its future are discussed.

10. Microbiol Mol Biol Rev. 2017 Feb 8;81(1):e00055-16. doi: 10.1128/MMBR.00055-16.
The Antigenic Structure of Zika Virus and Its Relation to Other Flaviviruses: Implications for Infection and Immunoprophylaxis.
Heinz FX(1), Stiasny K(2).
Author information:
(1)Department of Virology, Medical University of Vienna, Vienna, Austria franz.x.heinz@meduniwien.ac.at.
(2)Department of Virology, Medical University of Vienna, Vienna, Austria.

Zika virus was discovered ~70 years ago in Uganda and maintained a low profile as a human disease agent in Africa and Asia. Only recently has it caused explosive outbreaks in previously unaffected regions, first in Oceania and then in the Americas since 2015. Of special concern is the newly identified link between congenital malformations (especially microcephaly) and Zika virus infections during pregnancy. At present, it is unclear whether Zika virus changed its pathogenicity or whether the huge number of infections allowed the recognition of a previously cryptic pathogenic property. The purpose of this review is to discuss recent data on the molecular antigenic structure of Zika virus in the context of antibody-mediated neutralization and antibody-dependent enhancement (ADE) of infection, a phenomenon that has been implicated in the development of severe disease caused by the related dengue viruses. Emphasis is given to epitopes of antibodies that potently neutralize Zika virus and also to epitopes that provide antigenic links to other important human-pathogenic flaviviruses such as dengue, yellow fever, West Nile, Japanese encephalitis, and tick-borne encephalitis viruses. The antigenic cross talk between Zika and dengue viruses appears to be of special importance, since they cocirculate in many regions of endemicity and sequential infections are likely to occur frequently. New insights into the molecular antigenic structure of Zika virus and flaviviruses in general have provided the foundation for great progress made in developing Zika virus vaccines and antibodies for passive immunization.

11. NPJ Vaccines. 2018 Jun 11;3:24. doi: 10.1038/s41541-018-0061-9. eCollection
Current status of Zika vaccine development: Zika vaccines advance into clinical evaluation.
Barrett ADT(1).
Author information:
(1)Sealy Institute for Vaccine Sciences and Department of Pathology, University of Texas Medical Branch, Galveston, TX 77555-0436 USA.
Zika virus (ZIKV), a mosquito-borne flavivirus, was first identified in the 1940s in Uganda in Africa and emerged in the Americas in Brazil in May 2015. In the 30 months since ZIKV emerged as a major public health problem, spectacular progress has been made with vaccine development cumulating with the publication of three reports of phase 1 clinical trials in the 4th quarter of 2017. Clinical trials involving candidate DNA and purified inactivated virus vaccines showed all were safe and well-tolerated in the small number of volunteers and all induced neutralizing antibodies, although these varied by vaccine candidate and dosing regimen. These results suggest that a Zika vaccine can be developed and that phase 2 clinical trials are warranted. However, it is difficult to compare the results from the different phase 1 studies or with neutralizing antibodies induced by licensed flavivirus vaccines (Japanese encephalitis, tick-borne encephalitis, and yellow fever) as neutralizing antibody assays vary and, unfortunately, there are no standards for Zika virus neutralizing antibodies. In addition to clinical studies, substantial progress continues to be made in nonclinical development, particularly in terms of the ability of candidate vaccines to protect reproductive tissues, and the potential use of monoclonal antibodies for passive prophylaxis.

12. Front Cell Infect Microbiol. 2019 Mar 1;9:45. doi: 10.3389/fcimb.2019.00045.
Oral Vaccination With a Formulation Combining Rhipicephalus microplus Subolesin With Heat Inactivated Mycobacterium bovis Reduces Tick Infestations in Cattle.
Contreras M(1), Kasaija PD(1)(2), Merino O(3), de la Cruz-Hernandez NI(3), Gortazar C(1), de la Fuente J(1)(4).
Author information:
(1)SaBio, Instituto de Investigación en Recursos Cinegéticos IREC-CSIC-UCLM-JCCM, Ciudad Real, Spain.
(2)National Livestock Resources Research Institute (NaLIRRI/NARO), Tororo, Uganda.
(3)Facultad de Medicina Veterinaria y Zootecnia, Universidad Autónoma de Tamaulipas, Ciudad Victoria, Mexico.
(4)Department of Veterinary Pathobiology, Center for Veterinary Health Sciences, Oklahoma State University, Stillwater, OK, United States.
Vaccines are an environmentally friendly alternative to acaracides for the control of tick infestations, to reduce the risk for tick-borne diseases affecting human and animal health worldwide, and to improve animal welfare and production. Subolesin (SUB, also known as 4D8) is the functional homolog of Akirin2 involved in the regulation of development and innate immune response, and a proven protective antigen for the control of ectoparasite infestations and pathogen infection. Oral vaccination combining protein antigens with immunostimulants has proven efficacy with increased host welfare and safety, but has not been used for the control of tick infestations. Here we describe the efficacy of oral vaccination with a formulation combining Rhipicephalus microplus SUB and heat inactivated Mycobacterium bovis (IV) on cattle tick infestations and fertility. The levels of IgG antibody titers against SUB and M. bovis P22, and the expression of selected immune response genes were determined and analyzed as possible correlates of protection. We demonstrated that oral immunization with the SUB+IV formulation resulted in 51% reduction in the number of female ticks and 30% reduction in fertility with an overall efficacy of 65% in the control of R. microplus infestations by considering the cumulative effect on reducing tick survival and fertility in cattle. The akr2, IL-1β, and C3 mRNA levels together with antibody levels against SUB correlated with vaccine efficacy. The effect of the oral immunization with SUB+IV in cattle on tick survival and fertility is essential to reduce tick infestations, and extended previous results on the effect of R. microplus SUB for the control of cattle tick infestations. These results support the development of oral vaccines formulations for the control of tick infestations and the incidence of tick-borne diseases.

13. Front Bioeng Biotechnol. 2019 Sep 26;7:236. doi: 10.3389/fbioe.2019.00236.
Identification of Ixodid Tick-Specific Aquaporin-1 Potential Anti-tick Vaccine Epitopes: An in-silico Analysis.
Ndekezi C(1), Nkamwesiga J(1), Ochwo S(1), Kimuda MP(1)(2), Mwiine FN(1), Tweyongyere R(3), Amanyire W(1), Muhanguzi D(1)(4).
Author information:

(1)School of Biosecurity, Biotechnical and Laboratory Science, College of Veterinary Medicine Animal Resources and Biosecurity, Makerere University, Kampala, Uganda.
(2)Research Unit in Bioinformatics, Department of Biochemistry and Microbiology, Rhodes University, Grahamstown, South Africa.
(3)School of Veterinary Medicine and Animal Resources, College of Veterinary Medicine Animal Resources and Biosecurity, Makerere University, Kampala, Uganda.
(4)Makerere University/Uganda Virus Research Institute Centre of Excellence in Infection and Immunity Research and Training, Entebbe, Uganda.
Ticks are arthropod vectors of pathogens of both Veterinary and Public health importance. Acaricide application, which is currently the mainstay of tick control, is hampered by high cost, the need for regular application and a selection of multi-acaricide resistant tick populations. In light of this, future tick control approaches are poised to rely on the integration of rational acaricide application and other methods, such as vaccination. To contribute to systematic research-guided efforts to produce anti-tick vaccines, we carried out an in-silico analysis of tick aquaporin-1 (AQP1) protein in order to identify tick-specific AQP1 peptide motifs that can be used in future peptide anti-tick vaccine development. We carried out multiple sequence alignment (MSA), motif analysis, homology modeling, and structural analysis to identify tick-specific AQP1 peptide motifs. BepiPred, Chou and Fasman-Turn, Karplus and Schulz Flexibility, and Parker-Hydrophilicity prediction models were used to predict these motifs' potential to induce B cell mediated immune responses. The tick AQP1 (GenBankID: QDO67142.1) protein was largely similar to the bovine AQP1 (PDB:1J4N) (23 % sequence similarity; Structural superimposition of the homology model and 14JN homotetramers gave an RMSD = 3.269 while superimposition of a single chain gave an RMSD = 1.475). Tick and bovine AQP1 transmembrane domains were largely similar while their extracellular and cytoplasmic domain loops showed variation. Two tick-specific AQP1 peptide motifs; M7 (residues 106-125, p = 5.4e-25), and M8 (residues 85-104, p = 3.3e-24) were identified. These two motifs are located on the extra-cellular AQP1 domain. Motifs; M7 and M8 showed the highest Parker-Hydrophilicity prediction immunogenicity scores of 1.784 and 1.536, respectively. These two motifs can be a good starting point for the development of potential tick AQP1 peptide-based anti-tick vaccines. Further analyses such as molecular dynamics, in vitro assays, and in vivo immunization assays are required to validate these findings.

14. Vaccines (Basel). 2019 Oct 31;7(4):165. doi: 10.3390/vaccines7040165.
Towards a Multidisciplinary Approach to Improve Cattle Health and Production in Uganda.
de la Fuente J(1)(2), Contreras M(3), Kasaija PD(4)(5), Gortazar C(6), Ruiz-Fons JF(7), Mateo R(8), Kabi F(9).
Author information:
(1)SaBio, Instituto de Investigación en Recursos Cinegéticos (IREC), Consejo Superior de Investigaciones Científicas (CSIC), Universidad de Castilla-La Mancha (UCLM)-Junta de Comunidades de Castilla-La Mancha (JCCM), Ronda de Toledo s/n, 13005 Ciudad Real, Spain. jose_delafuente@yahoo.com.

(2)Department of Veterinary Pathobiology, Center for Veterinary Health Sciences, Oklahoma State University, Stillwater, OK 74078, USA. jose_delafuente@yahoo.com.
(3)SaBio, Instituto de Investigación en Recursos Cinegéticos (IREC), Consejo Superior de Investigaciones Científicas (CSIC), Universidad de Castilla-La Mancha (UCLM)-Junta de Comunidades de Castilla-La Mancha (JCCM), Ronda de Toledo s/n, 13005 Ciudad Real, Spain. marinelacr@hotmail.com.
(4)SaBio, Instituto de Investigación en Recursos Cinegéticos (IREC), Consejo Superior de Investigaciones Científicas (CSIC), Universidad de Castilla-La Mancha (UCLM)-Junta de Comunidades de Castilla-La Mancha (JCCM), Ronda de Toledo s/n, 13005 Ciudad Real, Spain. kpauldavis@gmail.com.
(5)National Livestock Resources Research Institute (NaLIRRI/NARO), Kampala 5704, Uganda. kpauldavis@gmail.com.
(6)SaBio, Instituto de Investigación en Recursos Cinegéticos (IREC), Consejo Superior de Investigaciones Científicas (CSIC), Universidad de Castilla-La Mancha (UCLM)-Junta de Comunidades de Castilla-La Mancha (JCCM), Ronda de Toledo s/n, 13005 Ciudad Real, Spain. christian.gortazar@uclm.es.
(7)SaBio, Instituto de Investigación en Recursos Cinegéticos (IREC), Consejo Superior de Investigaciones Científicas (CSIC), Universidad de Castilla-La Mancha (UCLM)-Junta de Comunidades de Castilla-La Mancha (JCCM), Ronda de Toledo s/n, 13005 Ciudad Real, Spain. josefrancisco.ruiz@uclm.es.
(8)SaBio, Instituto de Investigación en Recursos Cinegéticos (IREC), Consejo Superior de Investigaciones Científicas (CSIC), Universidad de Castilla-La Mancha (UCLM)-Junta de Comunidades de Castilla-La Mancha (JCCM), Ronda de Toledo s/n, 13005 Ciudad Real, Spain. rafael.mateo@uclm.es.
(9)National Livestock Resources Research Institute (NaLIRRI/NARO), Kampala 5704, Uganda. freddykabi@gmail.com.
A meeting and course supported by the Vice-Presidency for International Affairs of the Spanish National Research Council (CSIC) and the National Agricultural Research Organization of Uganda (NARO) were held at the National Livestock Resources Research Institute (NaLIRRI) in Nakyesasa, Wakiso, Uganda on September 2-9, 2019. The activities were conducted within the collaboration program between the Institute of Game and Wildlife Research (IREC, CSIC-UCLM-JCCM, Spain) and NARO for the development of vaccines and other interventions for the control of cattle ticks in Uganda.

15. J Virol. 2020 Mar 31;94(8):e02005-19. doi: 10.1128/JVI.02005-19. Print 2020 Mar
Structure and Characterization of Crimean-Congo Hemorrhagic Fever Virus GP38.
Mishra AK(1), Moyer CL(2), Abelson DM(2), Deer DJ(3), El Omari K(4), Duman R(4), Lobel L(5), Lutwama JJ(6), Dye JM(7), Wagner A(4), Chandran K(8), Cross RW(3), Geisbert TW(3), Zeitlin L(2), Bornholdt ZA(2), McLellan JS(9).
Author information:
(1)Department of Molecular Biosciences, University of Texas at Austin, Austin, Texas, USA.
(2)Mapp Biopharmaceutical, San Diego, California, USA.
(3)Department of Microbiology and Immunology, University of Texas Medical Branch, Galveston, Texas, USA.
(4)Diamond Light Source, Harwell Science and Innovation Campus, Didcot,

Oxfordshire, United Kingdom.
(5)Department of Microbiology, Immunology and Genetics, Faculty of Health Sciences, Ben-Gurion University of the Negev, Beer-Sheva, Israel.
(6)Department of Arbovirology, Emerging and Re-emerging Infection, Uganda Virus Research Institute, Entebbe, Uganda.
(7)US Army Medical Research Institute of Infectious Diseases, Fort Detrick, Maryland, USA.
(8)Department of Microbiology and Immunology, Albert Einstein College of Medicine, Bronx, New York, USA.
(9)Department of Molecular Biosciences, University of Texas at Austin, Austin, Texas, USA jmclellan@austin.utexas.edu.
Crimean-Congo hemorrhagic fever virus (CCHFV) is the causative agent of the most widespread tick-borne viral infection in humans. CCHFV encodes a secreted glycoprotein (GP38) of unknown function that is the target of a protective antibody. Here, we present the crystal structure of GP38 at a resolution of 2.5 Å, which revealed a novel fold primarily consisting of a 3-helix bundle and a β-sandwich. Sequence alignment and homology modeling showed distant homology between GP38 and the ectodomain of Gn (a structural glycoprotein in CCHFV), suggestive of a gene duplication event. Analysis of convalescent-phase sera showed high titers of GP38 antibodies indicating immunogenicity in humans during natural CCHFV infection. The only protective antibody for CCHFV in an adult mouse model reported to date, 13G8, bound GP38 with subnanomolar affinity and protected against heterologous CCHFV challenge in a STAT1-knockout mouse model. Our data strongly suggest that GP38 should be evaluated as a vaccine antigen and that its structure provides a foundation to investigate functions of this protein in the viral life cycle.IMPORTANCE Crimean-Congo hemorrhagic fever virus (CCHFV) is a priority pathogen that poses a high risk to public health. Due to the high morbidity and mortality rates associated with CCHFV infection, there is an urgent need to develop medical countermeasures for disease prevention and treatment. CCHFV GP38, a secreted glycoprotein of unknown function unique to the Nairoviridae family, was recently shown to be the target of a protective antibody against CCHFV. Here, we present the crystal structure of GP38, which revealed a novel fold with distant homology to another CCHFV glycoprotein that is suggestive of a gene duplication event. We also demonstrate that antibody 13G8 protects STAT1-knockout mice against heterologous CCHFV challenge using a clinical isolate from regions where CCHFV is endemic. Collectively, these data advance our understanding of GP38 structure and antigenicity and should facilitate future studies investigating its function.

16. Transbound Emerg Dis. 2020 Mar;67 Suppl 1:99-107. doi: 10.1111/tbed.13311.
Antigen gene and variable number tandem repeat (VNTR) diversity in Theileria parva parasites from Ankole cattle in south-western Uganda: Evidence for conservation in antigen gene sequences combined with extensive polymorphism at VNTR loci.
Nanteza A(1), Obara I(2), Kasaija P(1), Mwega E(3), Kabi F(4), Salih DA(5), Njahira M(6), Njuguna J(6), Odongo D(7), Bishop RP(8), Skilton RA(6), Ahmed J(1), Clausen PH(2), Lubega GW(1).

Author information:
(1)College of Veterinary Medicine, Animal Resources and Biosecurity, Makerere University, Kampala, Uganda.
(2)Institute for Parasitology and Tropical Veterinary Medicine, Freie Universität Berlin, Berlin, Germany.
(3)Faculty of Veterinary Medicine, Sokoine University of Agriculture, Morogoro, Tanzania.
(4)College of Natural Sciences, Makerere University, Kampala, Uganda.
(5)Central Veterinary Research Laboratories, Khartoum, Sudan.
(6)Biosciences East and Central Africa (BecA), Nairobi, Kenya.
(7)School of Biological Sciences, University of Nairobi, Nairobi, Kenya.
(8)Department of Veterinary Microbiology and Pathology, Washington State University, Pullman, WA, USA.
Theileria parva is a tick-transmitted apicomplexan protozoan parasite that infects lymphocytes of cattle and African Cape buffalo (Syncerus caffer), causing a frequently fatal disease of cattle in eastern, central and southern Africa. A live vaccination procedure, known as infection and treatment method (ITM), the most frequently used version of which comprises the Muguga, Serengeti-transformed and Kiambu 5 stocks of T. parva, delivered as a trivalent cocktail, is generally effective. However, it does not always induce 100% protection against heterologous parasite challenge. Knowledge of the genetic diversity of T. parva in target cattle populations is therefore important prior to extensive vaccine deployment. This study investigated the extent of genetic diversity within T. parva field isolates derived from Ankole (Bos taurus) cattle in south-western Uganda using 14 variable number tandem repeat (VNTR) satellite loci and the sequences of two antigen-encoding genes that are targets of CD8+T-cell responses induced by ITM, designated Tp1 and Tp2. The findings revealed a T. parva prevalence of 51% confirming endemicity of the parasite in south-western Uganda. Cattle-derived T. parva VNTR genotypes revealed a high degree of polymorphism. However, all of the T. parva Tp1 and Tp2 alleles identified in this study have been reported previously, indicating that they are widespread geographically in East Africa and highly conserved.

17. Transbound Emerg Dis. 2020 Mar;67 Suppl 1:88-98. doi: 10.1111/tbed.13417.
Multilocus genotyping of Theileria parva isolates associated with a live vaccination trial in Kenya provides evidence for transmission of immunizing parasites into local tick and cattle populations.
Bishop RP(1)(2), Odongo DO(1)(3), Spooner PR(1), Morzaria SP(1), Oura CAL(4), Skilton RA(1).
Author information:
(1)International Livestock Research Institute (ILRI), Nairobi, Kenya.
(2)Department of Veterinary Microbiology and Pathology, Washington State University, Pullman, WA, USA.
(3)School of Biological Sciences, University of Nairobi, Nairobi, Kenya.
(4)Faculty of Medical Sciences, School of Veterinary Medicine, University of the West Indies, St Augustine, Trinidad and Tobago.
The live infection and treatment (ITM) vaccination procedure using the trivalent

Muguga cocktail is increasingly being used to control East Coast fever, with potential implications for Theileria parva population genetic structure in the field. Transmission of the Kiambu V T. parva component to unvaccinated cattle has previously been described in Uganda. We monitored the T. parva carrier state in vaccinated and control animals on a farm in West Kenya where an ITM stabilate derived from the Kenyan T. parva Marikebuni stock was evaluated for field efficacy. A nested PCR-based Marikebuni-specific marker identified a carrier state in nine of ten vaccinated animals, detectable for a period of two years. We used 22 variable number tandem repeat (VNTR) markers to determine multilocus genotypes (MLGs) of 19 T. parva schizont-infected lymphocyte isolates derived from cattle and field ticks. Two isolates from unimmunized cattle were identical to the Marikebuni vaccination stock. Two cattle isolates were identical to a Muguga cocktail component Kiambu V. Seven isolates from ticks exhibited MLGs that were identical to the Serengeti/Muguga vaccine stocks. Six cattle and two tick-derived stocks exhibited unique MLGs. The data strongly suggest transmission of immunizing genotypes, from Marikebuni vaccine-induced carrier cattle to unimmunized cattle. It is possible that genotypes similar to those in the Muguga cocktail are present in the field in Western Kenya. An alternative hypothesis is that these parasites may have originated from vaccine trial sites in Eastern Uganda. If correct, this suggests that T. parva stocks used for immunization can potentially be disseminated 125 km beyond the immediate vaccination site. Regardless of their origin, the data provide evidence that genotypes similar to those in the Muguga cocktail are circulating in the field in East Africa, alleviating concerns about dissemination of 'alien' T. parva germplasm through live vaccination.

18. Transbound Emerg Dis. 2020 Mar;67 Suppl 1:56-67. doi: 10.1111/tbed.13325.
A review of recent research on Theileria parva: Implications for the infection and treatment vaccination method for control of East Coast fever.
Bishop RP(1), Odongo D(2), Ahmed J(3), Mwamuye M(3), Fry LM(1)(4), Knowles DP(1), Nanteza A(5), Lubega G(5), Gwakisa P(6), Clausen PH(3), Obara I(3).
Author information:
(1)Department of Veterinary Microbiology & Pathology, Washington State University, Pullman, WA, USA.
(2)School of Biological Sciences, University of Nairobi, Nairobi, Kenya.
(3)Institute for Parasitology and Tropical Veterinary Medicine, Freie Universität Berlin, Berlin, Germany.
(4)Animal Disease Research Unit, Agricultural Research Service, US Department of Agriculture, Pullman, WA, USA.
(5)College of Veterinary Medicine, Animal Resources and Biosecurity, Makerere University, Kampala, Uganda.
(6)Genome Science Laboratory, College of Veterinary Medicine and Biomedical Sciences, Sokoine University of Agriculture, Morogoro, Tanzania.
The infection and treatment (ITM) live vaccination method for control of Theileria parva infection in cattle is increasingly being adopted, particularly in Maasai pastoralist systems. Several studies indicate positive impacts on human livelihoods. Importantly, the first detailed protocol for live vaccine

production at scale has recently been published. However, quality control and delivery issues constrain vaccination sustainability and deployment. There is evidence that the distribution of T. parva is spreading from endemic areas in East Africa, North into Southern Sudan and West into Cameroon, probably as a result of anthropogenic movement of cattle. It has also recently been demonstrated that in Kenya, T. parva derived from cape buffalo can 'breakthrough' the immunity induced by ITM. However, in Tanzania, breakthrough has not been reported in areas where cattle co-graze with buffalo. It has been confirmed that buffalo in northern Uganda national parks are not infected with T. parva and R. appendiculatus appears to be absent, raising issues regarding vector distribution. Recently, there have been multiple field population genetic studies using variable number tandem repeat (VNTR) sequences and sequencing of antigen genes encoding targets of CD8+ T-cell responses. The VNTR markers generally reveal high levels of diversity. The antigen gene sequences present within the trivalent Muguga cocktail are relatively conserved among cattle transmissible T. parva populations. By contrast, greater genetic diversity is present in antigen genes from T. parva of buffalo origin. There is also evidence from several studies for transmission of components of stocks present within the Muguga cocktail, into field ticks and cattle following induction of a carrier state by immunization. In the short term, this may increase live vaccine effectiveness, through a more homogeneous challenge, but the long-term consequences are unknown.

19. Vaccines (Basel). 2020 Jun 18;8(2):319. doi: 10.3390/vaccines8020319.
Vaccination with Recombinant Subolesin Antigens Provides Cross-Tick Species Protection in Bos indicus and Crossbred Cattle in Uganda.
Kasaija PD(1)(2), Contreras M(1)(3), Kabi F(2), Mugerwa S(2), de la Fuente J(1)(4).
Author information:
(1)SaBio, Instituto de Investigación en Recursos Cinegéticos (IREC), Consejo Superior de Investigaciones Científicas (CSIC), Universidad de Castilla-La Mancha (UCLM)-Junta de Comunidades de Castilla-La Mancha (JCCM), Ronda de Toledo s/n, 13005 Ciudad Real, Spain.
(2)National Livestock Resources Research Institute (NaLIRRI/NARO), P.O. Box 5704 Kampala, Uganda.
(3)Interdisciplinary Laboratory of Clinical Analysis, Interlab-UMU, Regional Campus of International Excellence Campus Mare Nostrum, University of Murcia, Espinardo, 30100 Murcia, Spain.
(4)Department of Veterinary Pathobiology, Center for Veterinary Health Sciences, Oklahoma State University, Stillwater, OK 74078, USA.
Cattle tick infestations and transmitted pathogens affect animal health, production and welfare with an impact on cattle industry in tropical and subtropical countries. Anti-tick vaccines constitute an effective and sustainable alternative to the traditional methods for the control of tick infestations. Subolesin (SUB)-based vaccines have shown efficacy for the control of multiple tick species, but several factors affect the development of new and more effective vaccines for the control of tick infestations. To address this

challenge, herein we used a regional and host/tick species driven approach for vaccine design and implementation. The objective of the study was to develop SUB-based vaccines for the control of the most important tick species (Rhipicephalus appendiculatus, R. decoloratus and Amblyomma variegatum) affecting production of common cattle breeds (Bos indicus and B. indicus x B. taurus crossbred) in Uganda. In this way, we addressed the development of anti-tick vaccines as an intervention to prevent the economic losses caused by ticks and tick-borne diseases in the cattle industry in Uganda. The results showed the possibility of using SUB antigens for the control of multiple tick species in B. indicus and crossbred cattle and suggested the use of R. appendiculatus SUB to continue research on vaccine design and formulation for the control of cattle ticks in Uganda. Future directions would include quantum vaccinology approaches based on the characterization of the SUB protective epitopes, modeling of the vaccine E under Ugandan ecological and epidemiological conditions and optimization of vaccine formulation including the possibility of oral administration.

20. PLoS One. 2020 Jun 29;15(6):e0231434. doi: 10.1371/journal.pone.0231434.
Analysis of p67 allelic sequences reveals a subtype of allele type 1 unique to buffalo-derived Theileria parva parasites from southern Africa.
Mukolwe LD(1)(2), Odongo DO(3), Byaruhanga C(1)(4), Snyman LP(1)(5), Sibeko-Matjila KP(1).
Author information:
(1)Vectors and Vector-borne Diseases Research Programme, Department of Veterinary Tropical Diseases, Faculty of Veterinary Science, University of Pretoria, Onderstepoort, South Africa.
(2)Department of Veterinary Pathology, Microbiology & Parasitology, Faculty of Veterinary Medicine and Surgery, Egerton University, Egerton, Kenya.
(3)School of Biological Sciences, University of Nairobi, Nairobi, Kenya.
(4)National Agricultural Research Organization, Entebbe, Uganda.
(5)Durban Natural Science Museum, Durban, South Africa.
East Coast fever (ECF) and Corridor disease (CD) caused by cattle- and buffalo-derived T. parva respectively are the most economically important tick-borne diseases of cattle in the affected African countries. The p67 gene has been evaluated as a recombinant subunit vaccine against ECF, and for discrimination of T. parva parasites causing ECF and Corridor disease. The p67 allele type 1 was first identified in cattle-derived T. parva parasites from East Africa, where parasites possessing this allele type have been associated with ECF. Subsequent characterization of buffalo-derived T. parva parasites from South Africa where ECF was eradicated, revealed the presence of a similar allele type, raising concerns as to whether or not allele type 1 from parasites from the two regions is identical. A 900 bp central fragment of the gene encoding p67 was PCR amplified from T. parva DNA extracted from blood collected from cattle and buffalo in South Africa, Mozambique, Kenya, Tanzania and Uganda, followed by DNA sequence analysis. Four p67 allele types previously described were identified. A subtype of p67 allele type 1 was identified in parasites from clinical cases of CD and buffalo from southern Africa. Notably, p67 allele type

1 sequences from parasites associated with ECF in East Africa and CD in Kenya were identical. Analysis of two p67 B-cell epitopes (TpM12 and AR22.7) revealed amino acid substitutions in allele type 1 from buffalo-derived T. parva parasites from southern Africa. However, both epitopes were conserved in allele type 1 from cattle- and buffalo-derived T. parva parasites from East Africa. These findings reveal detection of a subtype of p67 allele type 1 associated with T. parva parasites transmissible from buffalo to cattle in southern Africa.

21. Vaccines (Basel). 2020 Aug 19;8(3):457. doi: 10.3390/vaccines8030457.
Cocktail Anti-Tick Vaccines: The Unforeseen Constraints and Approaches toward Enhanced Efficacies.
Ndawula C Jr(1), Tabor AE(2)(3).
Author information:
(1)Vaccinology Research Program, National Livestock Resources Research Institute, P O. Box 5746, Nakyesasa 256, Uganda.
(2)Centre for Animal Science, Queensland Alliance for Agriculture & Food Innovation, The University of Queensland Australia, St Lucia 4072, Queensland, Australia.
(3)School of Chemistry & Molecular Biosciences, The University of Queensland, St Lucia 4072, Queensland, Australia.
Ticks are second to mosquitoes as vectors of disease. Ticks affect livestock industries in Asia, Africa and Australia at ~$1.13 billion USD per annum. For instance, 80% of the global cattle population is at risk of infestation by the Rhipicephalus microplus species-complex, which in 2016 was estimated to cause $22-30 billion USD annual losses. Although the management of tick populations mainly relies on the application of acaricides, this raises concerns due to tick resistance and accumulation of chemical residues in milk, meat, and the environment. To counteract acaricide-resistant tick populations, immunological tick control is regarded among the most promising sustainable strategies. Indeed, immense efforts have been devoted toward identifying tick vaccine antigens. Until now, Bm86-based vaccines have been the most effective under field conditions, but they have shown mixed success worldwide. Currently, of the two Bm86 vaccines commercialized in the 1990s (GavacTM in Cuba and TickGARDPLUSTM in Australia), only GavacTM is available. There is thus growing consensus that combining antigens could broaden the protection range and enhance the efficacies of tick vaccines. Yet, the anticipated outcomes have not been achieved under field conditions. Therefore, this review demystifies the potential limitations and proposes ways of sustaining enhanced cocktail tick vaccine efficacy.

22. Parasit Vectors. 2020 Sep 7;13(1):452. doi: 10.1186/s13071-020-04322-9.
Sequence diversity of cytotoxic T cell antigens and satellite marker analysis of Theileria parva informs the immunization against East Coast fever in Rwanda.
Atuhaire DK(1), Muleya W(2), Mbao V(3), Bazarusanga T(4), Gafarasi I(5), Salt J(6), Namangala B(7), Musoke AJ(8).
Author information:
(1)Centre for Ticks and Tick-Borne Diseases, Private Bag A130, Lilongwe, Malawi.

(2)Department of Biomedical Sciences, School of Veterinary Medicine, University of Zambia, P.O. Box 32379, Lusaka, 10101, Zambia. muleyawalter@gmail.com.
(3)International Development Research Centre, Eastern and Southern Africa Regional Office, Nairobi, Kenya.
(4)Acre Africa, P.O. Box 6782, Kigali, Rwanda.
(5)Rwanda Agricultural Board, P.O. Box 5016, Kigali, Rwanda.
(6)Global Alliance for Livestock Veterinary Medicines, Doherty Building, Pentlands Science Park, Bush Loan, Penicuik, Edinburgh, EH26 0PZ, Scotland, UK.
(7)Department of Paraclinical Studies, School of Veterinary Medicine, University of Zambia, P.O. Box 32379, Lusaka, 10101, Zambia.
(8)LMK Medical laboratories and consultancies, P.O. Box 33686, Kampala, Uganda.
BACKGROUND: East Coast fever (ECF) caused by Theileria parva is endemic in Rwanda. In this study, the antigenic and genetic diversity of T. parva coupled with immunization and field challenge were undertaken to provide evidence for the introduction of ECF immunization in Rwanda.
METHODS: Blood collected from cattle in the field was screened for T. parva using ELISA and PCR targeting the p104 gene. Tp1 and Tp2 gene sequences were generated from field samples and from Gikongoro and Nyakizu isolates. Furthermore, multilocus genotype data was generated using 5 satellite markers and an immunization challenge trial under field conditions using Muguga cocktail vaccine undertaken.
RESULTS: Out of 120 samples, 44 and 20 were positive on ELISA and PCR, respectively. Antigenic diversity of the Tp1 and Tp2 gene sequences revealed an abundance of Muguga, Kiambu and Serengeti epitopes in the samples. A further three clusters were observed on both Tp1 and Tp2 phylogenetic trees; two clusters comprising of field samples and vaccine isolates and the third cluster comprising exclusively of Rwanda samples. Both antigens exhibited purifying selection with no positive selection sites. In addition, satellite marker analysis revealed that field samples possessed both shared alleles with Muguga cocktail on all loci and also a higher proportion of unique alleles. The Muguga cocktail (Muguga, Kiambu and Serengeti) genotype compared to other vaccine isolates, was the most represented in the field samples. Further low genetic sub-structuring (FST = 0.037) coupled with linkage disequilibrium between Muguga cocktail and the field samples was observed. Using the above data to guide a field immunization challenge trial comprising 41 immunized and 40 control animals resulted in 85% seroconversion in the immunized animals and an efficacy of vaccination of 81.7%, implying high protection against ECF.
CONCLUSIONS: Antigenic and genetic diversity analysis of T. parva facilitated the use of Muguga cocktail vaccine in field conditions. A protection level of 81.7% was achieved, demonstrating the importance of combining molecular tools with field trials to establish the suitability of implementation of immunization campaigns. Based on the information in this study, Muguga cocktail immunization in Rwanda has a potential to produce desirable results.

23. Ticks Tick Borne Dis. 2020 Nov;11(6):101539. doi: 10.1016/j.ttbdis.2020.101539.
Microsatellite and minisatellite genotyping of Theileria parva population from southern Africa reveals possible discriminatory allele profiles with parasites

from eastern Africa.
Lubembe DM(1), Odongo DO(2), Salih DA(3), Sibeko-Matjila KP(4).
Author information:
(1)Vectors and Vector-borne Diseases Research Programme, Department of Veterinary Tropical Diseases, Faculty of Veterinary Science, University of Pretoria, Private Bag X04, Onderstepoort, 0110, South Africa. Electronic address: dlubembe@gmail.com.
(2)School of Biological Sciences, University of Nairobi, P.O. Box 30197, Nairobi, 00100, Kenya.
(3)Central Veterinary Research Laboratory, P.O. Box 8067, Khartoum, Sudan.
(4)Vectors and Vector-borne Diseases Research Programme, Department of Veterinary Tropical Diseases, Faculty of Veterinary Science, University of Pretoria, Private Bag X04, Onderstepoort, 0110, South Africa.
The control of Theileria parva, a protozoan parasite that threatens almost 50% of the cattle population in Africa, is still a challenge in many affected countries. Theileria parva field parasites from eastern Africa, and parasites comprising the current live T. parva vaccine widely deployed in the same region have been reported to be genotypically diverse. However, similar reports on T. parva parasites from southern Africa are limited, especially in Corridor disease designated areas. Establishing the extent of genetic exchange in T. parva populations is necessary for effective control of the parasite infection. Twelve polymorphic microsatellite and minisatellite loci were targeted for genotypic and population genetics analysis of T. parva parasites from South Africa, Mozambique, Kenya and Uganda using genomic DNA prepared from cattle and buffalo blood samples. The results revealed genotypic similarities among parasites from the two regions of Africa, with possible distinguishing allelic profiles on three loci (MS8, MS19 and MS33) for parasites associated with Corridor disease in South Africa, and East Coast fever in eastern Africa. Individual populations were in linkage equilibrium (VD<L), but when considered as one combined population, linkage disequilibrium (VD>L) was observed. Genetic divergence was observed to be more within (AMOVA = 74%) than between (AMOVA = 26%) populations. Principal coordinate analysis showed clustering that separated buffalo-derived from cattle-derived T. parva parasites, although parasites from cattle showed a close genetic relationship. The results also demonstrated geographic sub-structuring of T. parva parasites based on the disease syndromes caused in cattle in the two regions of Africa. These findings provide additional information on the genotypic diversity of T. parva parasites from South Africa, and reveal possible differences based on three loci (MS8, MS19 and MS33) and similarities between buffalo-derived T. parva parasites from southern and eastern Africa.

24. Vet Parasitol. 2020 Dec;288:109304. doi: 10.1016/j.vetpar.2020.109304. Epub 2020
An alternative cold chain for storing and transporting East Coast fever vaccine.
Atuhaire DK(1), Lieberman D(2), Marcotty T(3), Musoke AJ(4), Madan D(5).
Author information:
(1)Centre for Ticks and Tick-Borne Diseases, Lilongwe, Malawi.
(2)Global Health Labs, Bellevue, Washington, United States; Intellectual

Ventures' Global Good Fund, Bellevue, Washington, United States.
(3)University of Namur, Belgium.
(4)LMK Medical Laboratories and Consultancies, Kampala, Uganda.
(5)Global Health Labs, Bellevue, Washington, United States; Intellectual
Ventures' Global Good Fund, Bellevue, Washington, United States. Electronic
address: damian.madan@ghlabs.org.
East Coast fever (ECF) is an often fatal, economically important cattle disease that predominantly affects eastern, central, and southern Africa. ECF is controlled through vaccination by means of simultaneous injection of oxytetracycline and cryogenically preserved stabilate containing live, disease-causing parasites. Storage and transportation of the stabilate requires liquid nitrogen, a commodity that is commonly unreliable in low-resource settings. Here we show that storage of conventionally prepared stabilate at -80 °C for up to 30 days does not significantly affect its ability to infect cultured peripheral blood mononucleated cells or live cattle, suggesting an alternative cold chain that maintains these temperatures could be used to effectively manage ECF.

25. Heliyon. 2020 Dec 31;7(1):e05688. doi: 10.1016/j.heliyon.2020.e05688.
Molecular epidemiology of anaplasmosis in small ruminants along a human-livestock-wildlife interface in Uganda.
Kasozi KI(1)(2)(3), Welburn SC(1)(4), Batiha GE(5), Marraiki N(6), Nalumenya DP(7), Namayanja M(7), Matama K(8), Zalwango KK(7), Matovu W(7), Zirintunda G(2), Ekou J(2), Kembabazi S(8), Mugasa CM(7), Kitibwa A(7), Tayebwa DS(7), Musinguzi SP(9), Mahero M(10), Ssengendo I(8), Nanteza A(7), Matovu E(7), MacLeod ET(1).
Author information:
(1)Infection Medicine, Deanery of Biomedical Sciences, College of Medicine and Veterinary Medicine, The University of Edinburgh, 1 George Square, Edinburgh EH8 9JZ, United Kingdom.
(2)Department of Animal Production and Management, Faculty of Agriculture and Agricultural Sciences, Busitema University Arapai Campus, Box 203 Soroti, Uganda.
(3)School of Medicine, Kabale University, Box 317 Kabale, Uganda.
(4)Zhejiang University-University of Edinburgh Institute, Zhejiang University School of Medicine, International Campus, 718 East Haizhou Road, Haining 314400, China.
(5)Department of Pharmacology and Therapeutics, Faculty of Veterinary Medicine, Damanhour University, Damanhour 22511, AlBeheira, Egypt.
(6)Department of Botany and Microbiology, College of Science, King Saud University, Riyadh 11451, Saudi Arabia.
(7)College of Veterinary Medicine Animal Resources and Biosecurity, Makerere University, Box 7062 Kampala, Uganda.
(8)Kampala International University Western Campus, Box 71 Bushenyi, Uganda.
(9)Faculty of Agriculture and Environmental Sciences, Kabale University, Box 315 Kabale, Uganda.
(10)Department of Veterinary Population Medicine, College of Veterinary

Medicine, University of Minnesota, USA.
BACKGROUND: Information as regards the epidemiology of the Anaplasmataceae in small ruminants in several low- and middle-income countries is scarce.
METHODS: In this study a total of 712 DNA samples collected from small ruminants were analyzed for Anaplasmataceae and Anaplasma ovis using the 16S rRNA and MSP4 genes respectively. Infection risk was assessed by location, sex and age of the animals and qGIS® was used to construct spatial maps.
RESULTS: The prevalence of Anaplasmataceae spp was 89.1% (95% CI: 77.5-95.9) and 79.1% (95% CI: 75.9-82.1) in ovines and caprines respectively (RR = 1.1, 95% CI: 1.0-1.3); higher than those previously reported in other eastern African countries. The prevalence of A. ovis was 26.1% and 25.4% for both ovines and caprines respectively with ovines showing significantly higher levels of infection than caprines ($P < 0.05$). The risk of Anaplasma ovis infections was not affected by age (OR = 1.2, 95% CI: 0.9-1.7) or sex (OR = 1.1, 95% CI: 0.6-2.0). Small ruminants located at the forest edge (<0.3 km) showed higher A. ovis prevalence than those found inland with infections present in the midland regions associated with increased agricultural activity.
CONCLUSION: Anaplasma ovis remains a major challenge for small ruminant husbandry in Uganda and infections are under-reported. Policy efforts to prioritize management of Anaplasmataceae for small ruminant health would promote livestock productivity in vulnerable communities, improving livelihoods and ecosystem health.

26. Microorganisms. 2021 Feb 20;9(2):438. doi: 10.3390/microorganisms9020438.
Phylogenetic Characterization of Crimean-Congo Hemorrhagic Fever Virus Detected in African Blue Ticks Feeding on Cattle in a Ugandan Abattoir.
Wampande EM(1), Waiswa P(1), Allen DJ(2), Hewson R(2)(3), Frost SDW(4)(5), Stubbs SCB(4)(6).
Author information:
(1)College of Veterinary Medicine, Animal Resources and Biosecurity, Makerere University, Kampala, Uganda.
(2)Department of Infection Biology, London School of Hygiene and Tropical Medicine, London WC1E 7HT, UK.
(3)Virology and Pathogenesis Group, Public Health England, Porton Down, Salisbury SP4 0JG, UK.
(4)Department of Infectious Disease Epidemiology, London School of Hygiene and Tropical Medicine, London WC1E 7HT, UK.
(5)Microsoft Research, Redmond, Washington, DC 98052, USA.
(6)Department of Veterinary Medicine, University of Cambridge, Cambridge CB3 0ES, UK.
Crimean-Congo hemorrhagic fever virus (CCHFV) is the most geographically widespread of the tick-borne viruses. However, African strains of CCHFV are poorly represented in sequence databases. In addition, almost all sequence data collected to date have been obtained from cases of human disease, while information regarding the circulation of the virus in tick and animal reservoirs is severely lacking. Here, we characterize the complete coding region of a novel CCHFV strain, detected in African blue ticks (Rhipicephalus (Boophilus)

decoloratus) feeding on cattle in an abattoir in Kampala, Uganda. These cattle originated from a farm in Mbarara, a major cattle-trading hub for much of Uganda. Phylogenetic analysis indicates that the newly sequenced strain belongs to the African genotype II clade, which predominantly contains the sequences of strains isolated from West Africa in the 1950s, and South Africa in the 1980s. Whilst the viral S (nucleoprotein) and L (RNA polymerase) genome segments shared >90% nucleotide similarity with previously reported genotype II strains, the glycoprotein-coding M segment shared only 80% nucleotide similarity with the next most closely related strains, which were derived from ticks in Western India and Northern China. This genome segment also displayed a large number of non-synonymous mutations previously unreported in the genotype II strains. Characterization of this novel strain adds to our limited understanding of the natural diversity of CCHFV circulating in both ticks and in Africa. Such data can be used to inform the design of vaccines and diagnostics, as well as studies exploring the epidemiology and evolution of the virus for the establishment of future CCHFV control strategies.

27. PLoS One. 2021 May 17;16(5):e0251500. doi: 10.1371/journal.pone.0251500.
Molecular characterization and population genetics of Theileria parva in Burundi's unvaccinated cattle: Towards the introduction of East Coast fever vaccine.
Atuhaire DK(1), Muleya W(2), Mbao V(3), Niyongabo J(4), Nyabongo L(4), Nsanganiyumwami D(4), Salt J(5), Namangala B(6), Musoke AJ(7).
Author information:
(1)Centre for Ticks and Tick-Borne Diseases, Lilongwe, Malawi.
(2)Department of Biomedical Sciences, School of Veterinary Medicine, University of Zambia, Lusaka, Zambia.
(3)International Development Research Centre, Eastern and Southern Africa Regional Office, Nairobi, Kenya.
(4)National Veterinary Research Laboratory, Directorate of Animal Health, Bujumbura, Burundi.
(5)Global Alliance for Livestock Veterinary Medicines, Pentlands Science Park, Bush Loan, Penicuik Edinburgh, Scotland.
(6)Department of Paraclinical Studies, School of Veterinary Medicine, University of Zambia, Lusaka, Zambia.
(7)LMK Medical laboratories and Consultancies, Kampala, Uganda.
Theileria parva (T. parva) is a protozoan parasite that causes East Coast fever (ECF). The disease is endemic in Burundi and is a major constraint to livestock development. In this study, the parasite prevalence in cattle in six regions namely; Northern, Southern, Eastern, Western, Central and North Eastern was estimated. Furthermore, the sequence diversity of p67, Tp1 and Tp2 genes was assessed coupled with the population genetic structure of T. parva using five satellite markers. The prevalence of ECF was 30% (332/1109) on microscopy, 60% (860/1431) on ELISA and 79% (158/200) on p104 gene PCR. Phylogenetic analysis of p67 gene revealed that only allele 1 was present in the field samples. Furthermore, phylogenetic analysis of Tp1 and Tp2 showed that the majority of samples clustered with Muguga, Kiambu and Serengeti and shared similar epitopes.

On the other hand, genetic analysis revealed that field samples shared only two alleles with Muguga Cocktail. The populations from the different regions indicated low genetic differentiation (FST = 0.047) coupled with linkage disequilibrium and non-panmixia. A low to moderate genetic differentiation (FST = 0.065) was also observed between samples and Muguga cocktail. In conclusion, the data presented revealed the presence of a parasite population that shared similar epitopes with Muguga Cocktail and was moderately genetically differentiated from it. Thus, use of Muguga Cocktail vaccine in Burundi is likely to confer protection against T. parva in field challenge trials.

28. Ticks Tick Borne Dis. 2021 Sep;12(5):101756. doi: 10.1016/j.ttbdis.2021.101756.
Cattle ticks and tick-borne diseases: a review of Uganda's situation.
Kasaija PD(1), Estrada-Peña A(2), Contreras M(3), Kirunda H(4), de la Fuente J(5).
Author information:
(1)SaBio, Instituto de Investigación en Recursos Cinegéticos (IREC), Consejo Superior de Investigaciones Científicas (CSIC), Universidad de Castilla-La Mancha (UCLM)-Junta de Comunidades de Castilla-La Mancha (JCCM), Ronda de Toledo s/n, 13005 Ciudad Real, Spain; National Livestock Resources Research Institute (NaLIRRI/NARO), P.O. Box 5704, Kampala, Uganda.
(2)Department of Animal Pathology, Faculty of Veterinary Medicine, 50013 Zaragoza, Spain.
(3)SaBio, Instituto de Investigación en Recursos Cinegéticos (IREC), Consejo Superior de Investigaciones Científicas (CSIC), Universidad de Castilla-La Mancha (UCLM)-Junta de Comunidades de Castilla-La Mancha (JCCM), Ronda de Toledo s/n, 13005 Ciudad Real, Spain; Interdisciplinary Laboratory of Clinical Analysis, Interlab-UMU, Regional Campus of International Excellence Campus Mare Nostrum, University of Murcia, Espinardo, 30100 Murcia, Spain.
(4)National Livestock Resources Research Institute (NaLIRRI/NARO), P.O. Box 5704, Kampala, Uganda.
(5)SaBio, Instituto de Investigación en Recursos Cinegéticos (IREC), Consejo Superior de Investigaciones Científicas (CSIC), Universidad de Castilla-La Mancha (UCLM)-Junta de Comunidades de Castilla-La Mancha (JCCM), Ronda de Toledo s/n, 13005 Ciudad Real, Spain; Department of Veterinary Pathobiology, Center for Veterinary Health Sciences, Oklahoma State University, Stillwater, OK 74078, USA. Electronic address: josedejesus.fuente@uclm.es.
Herein we review the epidemiology of ticks and tick-borne diseases (TTBDs), their impact on livestock health and on the economy, control and associated challenges in Uganda. Ticks are leading vectors of economically important pathogens and are widespread in Uganda due to suitable climatic conditions. Besides the physical injury inflicted on the animal host, ticks transmit a number of pathogens that can cause morbidity and mortality of livestock if untreated, resulting in economic losses. Uganda suffers an aggregated annual loss (direct and indirect) of over USD 1.1 billion in the TTBDs complex. East Coast fever (ECF) caused by a protozoan haemoparasite, Theileria parva, is the most prevalent and economically important tick-borne disease (TBD) in Uganda and its vector, the brown ear tick (Rhipicephalus appendiculatus) widely

distributed. Other prevalent TBDs in Uganda include anaplasmosis, babesiosis and heartwater. We highlight the role of agro-ecological zones (AEZs) and livestock management system in the distribution of TTBDs, citing warm and humid lowlands as being ideal habitats for ticks and endemic for TBDs. Control of TTBDs is a matter of great importance as far as animal health is concerned in Uganda. Indigenous cattle, which make up over 90% of the national herd are known to be more tolerant to TTBDs and most farms rely on endemic stability to TBDs for control. However, exotic cattle breeds are more capital intensive than indigenous breeds, but the increasing adoption of tick-susceptible exotic cattle breeds (especially dairy) in western and central Uganda demands intensive use of acaricides for tick control and prevention of TBDs. Such acaricide pressure has unfortunately led to selection of acaricide-resistant tick populations and the consequent acaricide resistance observed in the field. Vaccination against ECF, selective breeding for tick resistance and integrated tick control approaches that limit tick exposure, could be adopted to interrupt spread of acaricide resistance. We recommend increasing monitoring and surveillance for TTBDs and for emerging acaricide resistance, improved extension services and sensitization of farmers on tick control measures, appropriate acaricide use and the development and implementation of vaccines for the control of TTBDs as more sustainable and effective interventions. A tick control policy should be developed, taking into account variations of agro-ecological zones, farm circumstances and indigenous technical knowledge, and this should be incorporated into the overall animal health program.

29. Vaccines (Basel). 2021 Oct 15;9(10):1185. doi: 10.3390/vaccines9101185.
From Bench to Field: A Guide to Formulating and Evaluating Anti-Tick Vaccines Delving beyond Efficacy to Effectiveness.
Ndawula C Jr(1)(2).
Author information:
(1)National Agricultural Research Organization, P.O. Box 295, Entebbe, Wakiso 256, Uganda.
(2)National Livestock Resources Research Institute, Vaccinology Research Programme, P.O. Box 5704, Nakyesasa, Wakiso 256, Uganda.
Ticks are ubiquitous blood-sucking ectoparasites capable of transmitting a wide range of pathogens such as bacteria, viruses, protozoa, and fungi to animals and humans. Although the use of chemicals (acaricides) is the predominant method of tick-control, there are increasing incidents of acaricide tick resistance. Furthermore, there are concerns over accumulation of acaricide residues in meat, milk and in the environment. Therefore, alternative methods of tick-control have been proposed, of which anti-tick cattle vaccination is regarded as sustainable and user-friendly. Over the years, tremendous progress has been made in identifying and evaluating novel candidate tick vaccines, yet none of them have reached the global market. Until now, Bm86-based vaccines (Gavac™ in Cuba and TickGARDPLUS™ Australia-ceased in 2010) are still the only globally commercialized anti-tick vaccines. In contrast to Bm86, often, the novel candidate anti-tick vaccines show a lower protection efficacy. Why is this so? In response, herein, the potential bottlenecks to formulating efficacious

anti-tick vaccines are examined. Aside from Bm86, the effectiveness of other anti-tick vaccines is rarely assessed. So, how can the researchers assess anti-tick vaccine effectiveness before field application? The approaches that are currently used to determine anti-tick vaccine efficacy are re-examined in this review. In addition, a model is proposed to aid in assessing anti-tick vaccine effectiveness. Finally, based on the principles for the development of general veterinary vaccines, a pipeline is proposed to guide in the development of anti-tick vaccines.

30. Methods Mol Biol. 2022;2411:253-267. doi: 10.1007/978-1-0716-1888-2_15.
Anti-Tick Vaccines: Current Advances and Future Prospects.
Muhanguzi D(1), Ndekezi C(2), Nkamwesiga J(2), Kalayou S(3), Ochwo S(2), Vuyani M(4), Kimuda MP(2).
Author information:
(1)School of Biosecurity, Biotechnical and Laboratory Science, College of Veterinary Medicine Animal Resources and Biosecurity, Makerere University, Kampala, Uganda. dennis.muhanguzi@mak.ac.ug.
(2)School of Biosecurity, Biotechnical and Laboratory Science, College of Veterinary Medicine Animal Resources and Biosecurity, Makerere University, Kampala, Uganda.
(3)International Centre of Insect Physiology and Ecology (ICIPE), Nairobi, Kenya.
(4)Research Unit in Bioinformatics (RUBi), Department of Biochemistry and Microbiology, Rhodes University, Grahamstown, South Africa.
Ticks are increasingly a global public health and veterinary concern. They transmit numerous pathogens that are of veterinary and public health importance. Acaricides, livestock breeding for tick resistance, tick handpicking, pasture spelling, and anti-tick vaccines (ATVs) are in use for the control of ticks and tick-borne diseases (TTBDs); acaricides and ATVs being the most and least used TTBD control methods respectively. The overuse and misuse of acaricides has inadvertently selected for tick strains that are resistant to acaricides. Furthermore, vaccines are rare and not commercially available in sub-Saharan Africa (SSA). It doesn't help that many of the other methods are labor-intensive and found impractical especially for larger farm operations. The success of TTBD control is therefore dependent on integrating all the currently available methods. Vaccines have been shown to be cheap and effective. However, their large-scale deployment for TTBD control in SSA is hindered by commercial unavailability of efficacious anti-tick vaccines against sub-Saharan African tick strains. Thanks to advances in genomics, transcriptomics, and proteomics technologies, many promising anti-tick vaccine antigens (ATVA) have been identified. However, few of them have been investigated for their potential as ATV candidates. Reverse vaccinology (RV) can be leveraged to accelerate ATV discovery. It is cheap and shortens the lead time from ATVA discovery to vaccine production. This chapter provides a brief overview of recent advances in ATV development, ATVs, ATV effector mechanisms, and anti-tick RV. Additionally, it provides a detailed outline of vaccine antigen selection and analysis using computational methods.

31. Pathogens. 2022 Jan 19;11(2):114. doi: 10.3390/pathogens11020114.
Sequence Diversity of Tp1 and Tp2 Antigens and Population Genetic Analysis of Theileria parva in Unvaccinated Cattle in Zambia's Chongwe and Chisamba Districts.
Muleya W(1), Atuhaire DK(2), Mupila Z(1), Mbao V(3), Mayembe P(4), Kalenga S(4), Fandamu P(4), Namangala B(5), Salt J(6), Musoke AJ(7).
Author information:
(1)Department of Biomedical Sciences, School of Veterinary Medicine, University of Zambia, Lusaka 10101, Zambia.
(2)Centre for Ticks and Tick-Borne Diseases, Lilongwe, Malawi.
(3)Eastern and Southern Africa Regional Office, International Development Research Centre, Nairobi 00200, Kenya.
(4)Department of Veterinary Services, Ministry of Fisheries and Livestock, Lusaka 50060, Zambia.
(5)Department of Paraclinical Studies, School of Veterinary Medicine, University of Zambia, Lusaka 10101, Zambia.
(6)Global Alliance for Livestock Veterinary Medicines, Pentlands Science Park, Bush Loan, Penicuik EH26 0PZ, UK.
(7)LMK Medical Laboratories and Consultancies, Kampala P.O. Box 33686, Uganda.
East Coast Fever (ECF), caused by Theileria parva, is a major constraint to improved livestock keeping in east and central Africa, including Zambia. To understand the dynamics and determine the candidates for immunization in Zambia's Chongwe and Chisamba districts, a combination of Tp1 and Tp2 gene sequencing and microsatellite analysis using nine markers was conducted from which an abundance of Muguga, Kiambu, Serengeti and Katete epitopes in the field samples was obtained. Phylogenetic analysis showed six (Tp1) and three (Tp2) clusters with an absence of geographical origin clustering. The majority of haplotypes were related to Muguga, Kiambu, Serengeti and Katete, and only a few were related to Chitongo. Both antigens showed purifying selection with an absence of positive selection sites. Furthermore, low to moderate genetic differentiation was observed among and within the populations, and when vaccine stocks were compared with field samples, Chongwe samples showed more similarity to Katete and less to Chitongo, while Chisamba samples showed similarity to both Katete and Chitongo and not to Muguga, Kiambu or Serengeti. We conclude that the use of Katete stock for immunization trials in both Chongwe and Chisamba districts might produce desirable protection against ECF.

32. Curr Res Parasitol Vector Borne Dis. 2022 May 14;2:100090. doi: 10.1016/j.crpvbd.2022.100090. eCollection 2022.
Acaricide resistance in livestock ticks infesting cattle in Africa: Current status and potential mitigation strategies.
Githaka NW(1), Kanduma EG(2), Wieland B(1), Darghouth MA(3), Bishop RP(4).
Author information:
(1)International Livestock Research Institute (ILRI), P.O. Box 30709-00100, Nairobi, Kenya.
(2)Department of Biochemistry, Faculty of Science and Technology, University of

Nairobi, P.O. Box 30197-00100, Nairobi, Kenya.
(3)Laboratoire de Parasitologie, Ecole Nationale de Médecine Vétérinaire, Institution de la Recherche et de l'Enseignement Supérieur Agricoles and La Manouba University, 2020, Sidi Thabet, Tunisia.
(4)Department of Veterinary Microbiology and Pathology, Washington State University, Pullman, WA, USA.
In many African countries, tick control has recently been the responsibility of resource-poor farmers rather than central government veterinary departments. This has led to an increase in acaricide resistance, threatening the welfare of livestock farmers in sub-Saharan Africa. Resistance has evolved to the three classes of acaricides used most extensively in the continent, namely fourth-generation synthetic pyrethroids (SP), organophosphates (OP) and amidines (AM), in virtually all countries in which they have been deployed across the globe. Most current data are derived from research in Australia and Latin America, with the majority of studies on acaricide resistance in Africa performed in South Africa. There is also limited recent research from West Africa and Uganda. These studies confirm that acaricide resistance in cattle ticks is a major problem in Africa. Resistance is most frequently directly assayed in ticks using the larval packet test (LPT) that is endorsed by FAO, but such tests require a specialist tick-rearing laboratory and are relatively time consuming. To date they have only been used on a limited scale in Africa and resistance is often still inferred from tick numbers on animals. Rapid tests for resistance in ticks, would be better than the LPT and are theoretically possible to develop. However, these are not yet available. Resistance can be mitigated through integrated control strategies, comprising a combination of methods, including acaricide class rotation or co-formulations, ethnoveterinary practices, vaccination against ticks and modified land management use by cattle, with the goal of minimising the number of acaricide applications required per year. There are data suggesting that small-scale farmers in Africa are often unaware of the chemical differences between different acaricide brands and use these products at concentrations other than those recommended by the manufacturers, or in incorrect rotations or combinations of the different classes of chemicals on the market. There is an urgent need for a more evidence-based approach to acaricide usage in small-scale livestock systems in Africa, including direct measurements of resistance levels, combined with better education of farmers regarding acaricide products and how they should be deployed for control of livestock ticks.

33. Vaccine. 2022 Jul 30;40(32):4564-4573. doi: 10.1016/j.vaccine.2022.06.036. Epub
Oral vaccine formulation combining tick Subolesin with heat inactivated mycobacteria provides control of cross-species cattle tick infestations.
Kasaija PD(1), Contreras M(2), Kabi F(3), Mugerwa S(3), Garrido JM(4), Gortazar C(2), de la Fuente J(5).
Author information:
(1)SaBio, Instituto de Investigación en Recursos Cinegéticos IREC-CSIC-UCLM-JCCM, Ronda de Toledo s/n, 13005 Ciudad Real, Spain; National Livestock Resources Research Institute (NaLIRRI/NARO), P.O. Box 5704, Wakiso

District, Uganda.
(2)SaBio, Instituto de Investigación en Recursos Cinegéticos
IREC-CSIC-UCLM-JCCM, Ronda de Toledo s/n, 13005 Ciudad Real, Spain.
(3)National Livestock Resources Research Institute (NaLIRRI/NARO), P.O. Box
5704, Wakiso District, Uganda.
(4)NEIKER-Instituto Vasco de Investigación y Desarrollo Agrario, Animal Health
Department, Bizkaia Science and Technology Park 812L, 48160 Derio (Bizkaia),
Spain.
(5)SaBio, Instituto de Investigación en Recursos Cinegéticos
IREC-CSIC-UCLM-JCCM, Ronda de Toledo s/n, 13005 Ciudad Real, Spain; Department
of Veterinary Pathobiology, Center for Veterinary Health Sciences, Oklahoma
State University, Stillwater, OK 74078, USA. Electronic address:
jose_delafuente@yahoo.com.
Tick vaccines are necessary as part of a One Health approach for the control of tick infestations and tick-borne diseases. Subolesin (SUB, also known as 4D8) is a tick protective antigen that has shown efficacy in vaccine formulations for the control of ectoparasite infestations and pathogen infection/transmission. A recent proof-of-concept study reported oral vaccination combining Rhipicephalus microplus SUB with heat inactivated Mycobacterium bovis (IV) as an immunostimulant for the control of cattle tick infestations. Based on the efficacy of Rhipicephalus decoloratus SUB for the control of multiple cattle tick species in Uganda, herein we design a controlled pen trial using an oral formulation combining R. decoloratus SUB with IV for the control of R. decoloratus and Rhipicephalus appendiculatus cattle tick infestations. Vaccine efficacy (E) of SUB + IV on tick life cycle was compared with IV and SUB alone and with PBS as control. The IgG antibody titers against SUB and M. bovis P22 and the serum levels of selected protein immune biomarkers (IL-1beta, TNF-alpha, C3) were determined and analyzed as possible correlates of protection. Oral immunization with IV and SUB alone and in SUB + IV combination were effective for the control of tick infestations (E = 71-96% for R. decoloratus and 87-99% for R. appendiculatus) with highest E (higher than 95%) for SUB + IV. The results demonstrated that oral immunization with the SUB + IV formulation resulted in effective control of cattle tick infestations through the activation of multiple immune mechanisms. These results support the application of oral vaccine formulations with SUB + IV for the control of cattle infestations with Rhipicephalus species towards improving animal health.

34. Front Vet Sci. 2022 Jun 13;9:863526. doi: 10.3389/fvets.2022.863526. eCollection
Awareness, Knowledge, and Perceptions Regarding Rabies Prevention Among Rural Communities in Masaka District, Central Uganda: A Qualitative Study.
Kankya C(1), Dürr S(2), Hartnack S(3), Warembourg C(2), Okello J(1), Muleme J(1)(4), Okello W(1), Methodius T(1), Alobo G(1), Odoch T(1).
Author information:
(1)Department of Biosecurity, Ecosystems and Veterinary Public Health, School of Biosecurity, Biotechnical and Laboratory Sciences, College of Veterinary Medicine, Animal Resources and Biosecurity, Makerere University, Kampala, Uganda.

(2)Veterinary Public Health Institute, Vetsuisse Faculty, University of Bern, Bern, Switzerland.
(3)Section of Epidemiology, Vetsuisse Faculty, University of Zurich, Zurich, Switzerland.
(4)Department of Disease Control and Environmental Health, School of Public Health, College of Health Sciences, Makerere University, Kampala, Uganda.
Rabies is a zoonotic disease that is mainly transmitted to humans through dog bites. It remains a major public health threat in many Asian and African countries, including Uganda. The main objective of this study was to investigate awareness, knowledge, and perceptions of communities toward human and dog health related to rabies prevention, as well as dog management practices within Masaka district, central Uganda. Data collection involved nine key informant interviews (KIIs) and six focus group discussions (FGDs). Methods used during focus group discussions included qualitative interviews (using open-ended questions), simple ranking, and proportional piling. Data from KIIs and FGDs were analyzed using content analysis in NVivo (version 12.0). This study reveals that community members in the rural settings uses herbal concoctions in replacement or as an alternative to dog vaccination. Furthermore, the study reveals that dogs play the vital roles in the households like as they offer protection to people and household properties, despite being ranked second least among the household animals. The commonest livelihood activity was a small-scale mixed farming. Most of the households kept dogs, but they are ranked at second lowest in terms of economic value among all domestic animals. Free roaming and tethering were the common dog-keeping systems, and home-based feed (food leftovers, bones) was provided mainly to the tethered dogs. Rabies, also locally known as "Eddalu Lyembwa" (that can be translated as "madness of the dogs"), was ranked as the disease of most important among dogs, besides other common diseases such as skin diseases, venereal diseases, worm infestations, and tick infestations.
Inadequate vaccination services for both humans and dogs were reported, and dog bite victims traveled a long distance to seek for post-exposure prophylaxis after dog bites. It can be concluded that there is a clear request for periodic mass vaccination campaigns against rabies among dogs, and access to vaccines within reasonable distances by humans after a rabies exposure, but also pre-emptive vaccination for those at high risk, such as veterinarians, needs to be improved.

35. Vaccines (Basel). 2022 Aug 16;10(8):1327. doi: 10.3390/vaccines10081327.
The Correlation between Subolesin-Reactive Epitopes and Vaccine Efficacy.
Contreras M(1), Kasaija PD(1)(2), Kabi F(2), Mugerwa S(2), De la Fuente J(1)(3).
Author information:
(1)SaBio, Instituto de Investigación en Recursos Cinegéticos (IREC), Consejo Superior de Investigaciones Científicas (CSIC), Universidad de Castilla-La Mancha (UCLM)-Junta de Comunidades de Castilla-La Mancha (JCCM), Ronda de Toledo s/n, 13005 Ciudad Real, Spain.
(2)National Livestock Resources Research Institute (NaLIRRI/NARO), Kampala P.O. Box 5704, Uganda.
(3)Center for Veterinary Health Sciences, Department of Veterinary Pathobiology,

Oklahoma State University, Stillwater, OK 74078, USA.
Vaccination is an environmentally-friendly alternative for tick control. The
tick antigen Subolesin (SUB) has shown protection in vaccines for the control of
multiple tick species in cattle. Additionally, recent approaches in quantum
vaccinomics have predicted SUB-protective epitopes and the peptide sequences
involved in protein−protein interactions in this tick antigen. Therefore, the
identification of B-cell−reactive epitopes by epitope mapping using a SUB
peptide array could be essential as a novel strategy for vaccine development.
Subolesin can be used as a model to evaluate the effectiveness of these
approaches for the identification of protective epitopes related to vaccine
protection and efficacy. In this study, the mapping of B-cell linear epitopes of
SUB from three different tick species common in Uganda (Rhipicephalus
appendiculatus, R. decoloratus, and Amblyomma variegatum) was conducted using
serum samples from two cattle breeds immunized with SUB-based vaccines. The
results showed that in cattle immunized with SUB from R. appendiculatus (SUBra)
all the reactive peptides (Z-score > 2) recognized by IgG were also significant
(Z-ratio > 1.96) when compared to the control group. Additionally, some of the
reactive peptides recognized by IgG from the control group were also recognized
in SUB cocktail−immunized groups. As a significant result, cattle groups that
showed the highest vaccine efficacy were Bos indicus immunized with a SUB
cocktail (92%), and crossbred cattle were immunized with SUBra (90%) against R.
appendiculatus ticks; the IgG from these groups recognized overlapping epitopes
from the peptide
SPTGLSPGLSPVRDQPLFTFRQVGLICERMMKERESQIRDEYDHVLSAKLAEQYDTFVKFTYDQKRFEGAT
PSYLS
(Z-ratio > 1.96), which partially corresponded to a Q38 peptide and the SUB
protein interaction domain. These identified epitopes could be related to the
protection and efficacy of the SUB-based vaccines, and new chimeras containing
these protective epitopes could be designed using this new approach.

36. Vaccines (Basel). 2022 Sep 22;10(10):1594. doi: 10.3390/vaccines10101594.
Monitoring the Subolesin Vaccine Field Trial for Safer Control of Cattle Ticks
Amidst Increasing Acaricide Resistance in Uganda.
Kabi F(1), Dhikusooka M(1), Matovu M(1), Mugerwa S(1), Kasaija P(1)(2), Emudong
P(1), Kirunda H(3), Contreras M(2), Gortazar C(2), De la Fuente J(2)(4).
Author information:
(1)National Livestock Resources Research Institute (NaLIRRI/NARO), Kampala P.O.
Box 5704, Uganda.
(2)SaBio, Instituto de Investigación en Recursos Cinegéticos (IREC), Consejo
Superior de Investigaciones Cientí ficas (CSIC), Universidad de Castilla-La
Mancha (UCLM)-Junta de Comunidades de Castilla-La Mancha (JCCM), Ronda de Toledo
12, 13005 Ciudad Real, Spain.
(3)Mbarara Zonal Agricultural Research and Development Institute (MbaZARDI),
Mbarara City P.O. Box 389, Uganda.
(4)Department of Veterinary Pathobiology, Center for Veterinary Health Sciences,
Oklahoma State University, Stillwater, OK 74078, USA.
A collaboration program was established between the group of Health and

Biotechnology (SaBio) of the IREC Institute of Game and Wildlife Research (CSIC-UCLM-JCCM, Spain) and the National Agricultural Research Organization of Uganda (NARO) for the development of vaccines for the control of cattle ticks in Uganda. Controlled pen trials identified a tick protective antigen, Rhipicephalus appendiculatus Subolesin, and a cross-species-effective vaccine formulation. As the next step, a controlled vaccine field trial has been approved by Ugandan state regulatory authorities, the Uganda National Council for Science and Technology (UNCST) and the National Drug Authority (NDA), to evaluate the efficacy and effectiveness of the vaccine formulation for the control of cattle tick infestations under field conditions. The results of this trial may lead to the approval of the vaccine for application in Uganda to improve cattle health and production while reducing the use of acaricides.

37. Vaccines (Basel). 2022 Dec 31;11(1):99. doi: 10.3390/vaccines11010099.
Inspiring Anti-Tick Vaccine Research, Development and Deployment in Tropical Africa for the Control of Cattle Ticks: Review and Insights.
Kasaija PD(1)(2), Contreras M(1), Kirunda H(3), Nanteza A(4), Kabi F(2), Mugerwa S(2), de la Fuente J(1)(5).
Author information:
(1)SaBio, Instituto de Investigación en Recursos Cinegéticos (IREC), Consejo Superior de Investigaciones Científicas (CSIC), Universidad de Castilla-La Mancha (UCLM)-Junta de Comunidades de Castilla-La Mancha (JCCM), Ronda de Toledo s/n, 13005 Ciudad Real, Spain.
(2)National Livestock Resources Research Institute (NaLIRRI), Kampala P.O. Box 5704, Uganda.
(3)National Agricultural Research Organisation (NARO), Mbarara ZARDI, Mbarara P.O. Box 389, Uganda.
(4)College of Veterinary Medicine, Animal Resources and Biosecurity, Makerere University, Kampala P.O. Box 7062, Uganda.
(5)Department of Veterinary Pathobiology, Center for Veterinary Health Sciences, Oklahoma State University, Stillwater, OK 74078, USA.
Ticks are worldwide ectoparasites to humans and animals, and are associated with numerous health and economic effects. Threatening over 80% of the global cattle population, tick and tick-borne diseases (TTBDs) particularly constrain livestock production in the East, Central and Southern Africa. This, therefore, makes their control critical to the sustainability of the animal industry in the region. Since ticks are developing resistance against acaricides, anti-tick vaccines (ATVs) have been proposed as an environmentally friendly control alternative. Whereas they have been used in Latin America and Australia to reduce tick populations, pathogenic infections and number of acaricide treatments, commercially registered ATVs have not been adopted in tropical Africa for tick control. This is majorly due to their limited protection against economically important tick species of Africa and lack of research. Recent advances in various omics technologies and reverse vaccinology have enabled the identification of many candidate anti-tick antigens (ATAs), and are likely to usher in the next generation of vaccines, for which Africa should prepare to embrace. Herein, we highlight some scientific principles and approaches that

have been used to identify ATAs, outline characteristics of a desirable ATA for vaccine design and propose the need for African governments to investment in ATV research to develop vaccines relevant to local tick species (personalized vaccines). We have also discussed the prospect of incorporating anti-tick vaccines into the integrated TTBDs control strategies in the sub-Saharan Africa, citing the case of Uganda.

38. Clin Infect Dis. 2023 Jun 8;76(11):2018-2024. doi: 10.1093/cid/ciad066.
Guidance on the Use of Convalescent Plasma to Treat Immunocompromised Patients With Coronavirus Disease 2019.
Bloch EM(1), Focosi D(2), Shoham S(3), Senefeld J(4), Tobian AAR(1), Baden LR(5), Tiberghien P(6), Sullivan DJ(7), Cohn C(8), Dioverti V(3), Henderson JP(9), So-Osman C(10)(11), Juskewitch JE(12), Razonable RR(13), Franchini M(14), Goel R(15), Grossman BJ(16), Casadevall A(7), Joyner MJ(4), Avery RK(3), Pirofski LA(17), Gebo KA(3).
Author information:
(1)Department of Pathology, Johns Hopkins University School of Medicine, Baltimore, Maryland, USA.
(2)North-Western Tuscany Blood Bank, Pisa University Hospital, Pisa, Italy.
(3)Department of Medicine, Division of Infectious Diseases, Johns Hopkins University School of Medicine, Baltimore, Maryland, USA.
(4)Department of Anesthesiology and Perioperative Medicine, Mayo Clinic, Rochester, Minnesota, USA.
(5)Department of Infectious Diseases, Brigham and Women's Hospital, Boston, Massachusetts, USA.
(6)Etablissement Français du Sang, La Plaine-St-Denis and Université de Franche-Comté, Besançon, France.
(7)Department of Molecular Microbiology and Immunology, Johns Hopkins Bloomberg School of Public Health, Baltimore, MD, USA.
(8)Department of Laboratory Medicine and Pathology, University of Minnesota, Minneapolis, Minnesota, USA.
(9)Departments of Internal Medicine (Division of Infectious Diseases) and Molecular Microbiology, Washington University School of Medicine, St. Louis, MO, USA.
(10)Department Transfusion Medicine, Division Blood Bank, Sanquin Blood Supply Foundation, Amsterdam, The Netherlands.
(11)Department Haematology, Erasmus Medical Centre, Rotterdam, The Netherlands.
(12)Department of Laboratory Medicine and Pathology, Mayo Clinic, Rochester campus, Minnesota, USA.
(13)Department of Internal Medicine, Division of Infectious Diseases, Mayo Clinic, Rochester, Minnesota, USA.
(14)Department of Hematology and Transfusion Medicine, Carlo Poma Hospital, Mantua, Italy.
(15)Division of Hematology/Oncology, Simmons Cancer Institute at SIU School of Medicine and Mississippi Valley Regional Blood Center, Springfield, Illinois, USA.
(16)Department of Pathology & Immunology, Washington University School of

Medicine, St. Louis, Missouri, USA.
(17)Department of Medicine, Infectious Diseases, Albert Einstein College of
Medicine, Bronx, New York, USA.
Coronavirus disease 2019 (COVID-19) convalescent plasma (CCP) is a safe and effective treatment for COVID-19 in immunocompromised (IC) patients. IC patients have a higher risk of persistent infection, severe disease, and death from COVID-19. Despite the continued clinical use of CCP to treat IC patients, the optimal dose, frequency/schedule, and duration of CCP treatment has yet to be determined, and related best practices guidelines are lacking. A group of individuals with expertise spanning infectious diseases, virology and transfusion medicine was assembled to render an expert opinion statement pertaining to the use of CCP for IC patients. For optimal effect, CCP should be recently and locally collected to match circulating variant. CCP should be considered for the treatment of IC patients with acute and protracted COVID-19; dosage depends on clinical setting (acute vs protracted COVID-19). CCP containing high-titer severe acute respiratory syndrome coronavirus 2 (SARS-CoV-2) antibodies, retains activity against circulating SARS-CoV-2 variants, which have otherwise rendered monoclonal antibodies ineffective.

39. Pathogens. 2023 Feb 10;12(2):295. doi: 10.3390/pathogens12020295.
East Coast Fever Carrier Status and Theileria parva Breakthrough Strains in Recently ITM Vaccinated and Non-Vaccinated Cattle in Iganga District, Eastern Uganda.
Oligo S(1), Nanteza A(1), Nsubuga J(1), Musoba A(1), Kazibwe A(1), Lubega GW(1).
Author information:
(1)College of Veterinary Medicine, Animal Resources and Biosecurity (COVAB), Makerere University, Kampala P.O. Box 7062, Uganda.
East Coast fever (ECF) is a tick-borne disease of cattle that hinders the development of the livestock industry in eastern, central and southern Africa. The 'Muguga cocktail' live vaccine, delivered by an infection and treatment method (ITM), remains the only immunisation strategy of controlling ECF. However, there are challenges of the live vaccine inducing ECF carrier status in immunised animals and the possibility of lack of protection from parasite strains that are antigenically different from the vaccine strains. In Uganda, there are insufficient data regarding the ECF carrier status and T. parva genetic diversity in vaccinated and associated non-vaccinated cattle to assess the effectiveness of ITM vaccination. Blood was collected from recently ECF vaccinated (98) and non-vaccinated (73) cattle from Iganga district in Eastern Uganda at 120 days post-vaccination. The p104 gene nested PCR was used to screen for T. parva DNA, 11 minisatellite and 3 microsatellite markers (SSR) were used for genotyping. Two minisatellite markers (MS7 and MS19) were used to determine whether ECF carrier status was due to the T. parva vaccine or local strains. The prevalence of T. parva based on p104 nPCR was 61.2% (60/98) (RR 2.234, 95% CI 1.49-3.35, p-value < 0.001) among recently vaccinated cattle and 27.4% (20/73) (RR 1.00) among associated non-vaccinated cattle. The Muguga cocktail vaccine strains were responsible for carrier status in 10 (58.8%) by MS7 and 11 (64.7%) by MS19 in vaccinated cattle. Genotypes of T. parva with different-sized alleles

to the vaccine strains that could be potential 'breakthroughs' were detected in 2 (11.8%)) and 4 (23.5%) isolates from vaccinated cattle based on MS7 and MS19 minisatellite markers, respectively. Using 14 SSR markers, T. parva diversity was higher in vaccinated (Na = 2.214, Ne = 1.978, He = 0.465) than associated non-vaccinated (Na = 1.071, Ne = 1.048, He = 0.259) cattle. The principal component analysis (PCA) showed isolates from vaccinated cattle were closely related to those from non-vaccinated cattle. The analysis of molecular variance (AMOVA) revealed high genetic variation (96%) within T. parva isolates from vaccinated and non-vaccinated cattle but low variation (4%) between vaccinated and non-vaccinated cattle. This study reveals the role of ITM in inducing the carrier status and higher T. parva genetic diversity in vaccinated cattle. The low genetic variation between T. parva isolates in both vaccinated and non-vaccinated cattle may be suggestive of the protective role of vaccine strains against genetically related local strains in the study area.

40. Pathogens. 2023 Oct 19;12(10):1258. doi: 10.3390/pathogens12101258.
Perception of Ticks and Tick-Borne Diseases Worldwide.
de la Fuente J(1)(2), Estrada-Peña A(3)(4), Rafael M(1), Almazán C(5), Bermúdez S(6), Abdelbaset AE(7), Kasaija PD(8), Kabi F(8), Akande FA(9), Ajagbe DO(10), Bamgbose T(11), Ghosh S(12)(13), Palavesam A(14), Hamid PH(15), Oskam CL(16)(17), Egan SL(16)(17), Duarte-Barbosa A(17)(18), Hekimoğlu O(19), Szabó MPJ(20), Labruna MB(21), Dahal A(22).
Author information:
(1)SaBio, Instituto de Investigación en Recursos Cinegéticos, IREC-CSIC-UCLM-JCCM, Ronda de Toledo 12, 13005 Ciudad Real, Spain.
(2)Department of Veterinary Pathobiology, Center for Veterinary Health Sciences, Oklahoma State University, Stillwater, OK 74078, USA.
(3)Department of Animal Health, Faculty of Veterinary Medicine, University of Zaragoza, 50013 Zaragoza, Spain.
(4)Research Group in Emerging Zoonoses, Instituto Agroalimentario de Aragón-IA2, Universidad de Zaragoza-CITA, 50013 Zaragoza, Spain.
(5)Facultad de Ciencias Naturales, Universidad Autonóma de Querétaro, Avenida de las Ciencias S/N Juriquilla, Querétaro 76230, Mexico.
(6)Medical Entomology Research Department, Gorgas Memorial Institute for Health Research, Panama City 0816-02593, Panama.
(7)Laboratory of Parasitology, Graduate School of Infectious Diseases, Faculty of Veterinary Medicine, Hokkaido University, Kita-18, Nishi-9, Sapporo 060-0818, Hokkaido, Japan.
(8)National Livestock Resources Research Institute (NaLIRRI/NARO), Kampala P.O. Box 5704, Uganda.
(9)Department of Veterinary Parasitology and Entomology, College of Veterinary Medicine, Federal University of Agriculture, Abeokuta 111101, Ogun State, Nigeria.
(10)Department of Pure and Applied Zoology, College of Biological Sciences, Federal University of Agriculture, Abeokuta 111101, Ogun State, Nigeria.
(11)Department of Biological Sciences, Microbiology Unit, Faculty of Science, Kings University, Ode-Omu City 221102, Osun State, Nigeria.

(12)Entomology Laboratory, Indian Veterinary Research Institute, Izatnagar, Bareilly 243122, Uttar Pradesh, India.
(13)IVRI-Eastern Regional Station, 37, Belgachia Road, Kolkata 700037, West Bengal, India.
(14)Translational Research Platform for Veterinary Biologicals, Centre for Animal Health Studies, Tamil Nadu Veterinary and Animal Sciences University, Chennai 600051, Tamil Nadu, India.
(15)Department of Animal Science, Universitas Sebelas Maret, Surakarta 57126, Indonesia.
(16)School of Medical, Molecular and Forensic Sciences, Murdoch University, Perth, WA 6150, Australia.
(17)Centre for One Health and Biosecurity, Harry Butler Institute, Murdoch University, Perth, WA 6150, Australia.
(18)School of Veterinary Medicine, Murdoch University, Perth, WA 6150, Australia.
(19)Division of Ecology, Faculty of Science, Hacettepe University, Beytepe, Ankara 06800, Turkey.
(20)Laboratório de Ixodologia, Faculdade de Medicina Veterinária, Universidade Federal de Uberlândia, Av. Pará, 1720/Campus Umuarama-Bloco 2T, Uberlândia 38400-902, Brazil.
(21)Faculty of Veterinary Medicine and Animal Science, University of São Paulo, Sao Paulo 05508-220, Brazil.
(22)Department of Microbiology and Parasitology, Faculty of Animal Science, Veterinary Science and Fisheries, Agriculture and Forestry University, Chitwan 44200, Nepal.
In this comprehensive review study, we addressed the challenge posed by ticks and tick-borne diseases (TBDs) with growing incidence affecting human and animal health worldwide. Data and perspectives were collected from different countries and regions worldwide, including America, Europe, Africa, Asia, and Oceania. The results updated the current situation with ticks and TBD and how it is perceived by society with information bias and gaps. The study reinforces the importance of multidisciplinary and international collaborations to advance in the surveillance, communication and proposed future directions to address these challenges.

vaccineANDUganda-set

Summary:

abstract-vaccineANDUganda-set (2020 – 2023)		
**Year**	**Number of publications**	**Number with coauthors from Uganda (%)**
2020	167	121 (72%)
2021	212	177 (83%)
2022	187	142 (76%)
2023	254	206 (81%)

1. Lancet Infect Dis. 2024 Feb;24(2):e93-e105. doi: 10.1016/S1473-3099(23)00376-6. Epub 2023
Prevention and post-exposure management of occupational exposure to Ebola virus.
Moso MA(1), Lim CK(2), Williams E(2), Marshall C(3), McCarthy J(3), Williamson DA(4).
Author information:
(1)Victorian Infectious Diseases Reference Laboratory, The Peter Doherty Institute for Infection and Immunity, Melbourne, VIC, Australia; Department of Infectious Diseases, The University of Melbourne, The Peter Doherty Institute for Infection and Immunity, Melbourne, VIC, Australia; Victorian Infectious Diseases Service, The Royal Melbourne Hospital, The Peter Doherty Institute for Infection and Immunity, Melbourne, VIC, Australia. Electronic address: michael.moso@mh.org.au.
(2)Victorian Infectious Diseases Reference Laboratory, The Peter Doherty Institute for Infection and Immunity, Melbourne, VIC, Australia.
(3)Department of Infectious Diseases, The University of Melbourne, The Peter Doherty Institute for Infection and Immunity, Melbourne, VIC, Australia; Victorian Infectious Diseases Service, The Royal Melbourne Hospital, The Peter Doherty Institute for Infection and Immunity, Melbourne, VIC, Australia.
(4)Victorian Infectious Diseases Reference Laboratory, The Peter Doherty Institute for Infection and Immunity, Melbourne, VIC, Australia; Department of Infectious Diseases, The University of Melbourne, The Peter Doherty Institute for Infection and Immunity, Melbourne, VIC, Australia.
There have been significant advances in the prevention and management of Ebola virus disease (EVD) caused by Zaire Ebola virus (ZEBOV), including the development of two effective vaccines, rVSV-ZEBOV and Ad26.ZEBOV/MVA-BN-Filo. In addition, ZEBOV monoclonal antibodies have become first-line therapy for EVD. However, the 2022-23 outbreak of Sudan Ebola virus (SUDV) in Uganda has highlighted the gap in current therapies and vaccines, whose efficacy is uncertain against non-ZEBOV species. Health-care and laboratory staff working in EVD treatment centres or Ebola virus diagnostic and research laboratories face unique risks relating to potential occupational exposure to Ebola viruses. Given the substantial morbidity and mortality associated with EVD, facilities should have strategies in place to manage occupational exposures, including consideration of post-exposure therapies. In this Review, we discuss currently

available evidence for prevention and post-exposure prophylaxis of EVD, including therapies currently under evaluation for SUDV.

2. Vaccine. 2024 Jan 12;42(2):84-98. doi: 10.1016/j.vaccine.2023.11.056. Epub 2023
The immunogenicity and safety of Group B Streptococcal maternal vaccines: A systematic review.
Bjerkhaug AU(1), Ramalingham S(2), Mboizi R(3), Le Doare K(4), Klingenberg C(5).
Author information:
(1)Paediatric Research Group, Faculty of Health Sciences, UiT-The Arctic University of Norway, Tromsø, Norway; Department of Paediatrics and Adolescence Medicine, University Hospital of North Norway, Tromsø, Norway. Electronic address: aline.bjerkhaug@uit.no.
(2)Paediatric Research Group, Faculty of Health Sciences, UiT-The Arctic University of Norway, Tromsø, Norway.
(3)Makerere University Johns Hopkins University (MU-JHU) Research Collaboration (MUJHU CARE LTD), Kampala, Uganda.
(4)Makerere University Johns Hopkins University (MU-JHU) Research Collaboration (MUJHU CARE LTD), Kampala, Uganda; Centre for Neonatal and Paediatric Infection, Maternal and Neonatal Vaccine Immunology Research Group, St Georgés University of London, United Kingdom.
(5)Paediatric Research Group, Faculty of Health Sciences, UiT-The Arctic University of Norway, Tromsø, Norway; Department of Paediatrics and Adolescence Medicine, University Hospital of North Norway, Tromsø, Norway.
PURPOSE: To systematically review immunogenicity and safety data of maternal group B streptococcal (GBS) vaccines in published clinical trials until July 2023.
METHODS: EMBASE, MEDLINE, Cochrane Library and clinicaltrial.gov. databases were searched for clinical studies that reported immunogenicity and/or safety of GBS vaccine in non-pregnant adults, pregnant women and infants between 1st of January 1996 to 31st of July 2023. Pairs of reviewers independently selected, data extracted, and assessed the risk of bias of the studies. Discrepancies were resolved by consensus. (PROSPERO CRD42020185213).
RESULTS: We retrieved 1472 records from the literature search; 20 studies and 6 sub-studies were included, involving 4440 non-pregnant participants and 1325 pregnant women with their newborns. There was a significantly higher IgG Geometric Mean Concentration (GMC) and IgG placental transfer ratios in vaccinated compared to placebo groups, with peak response 4-8 weeks after vaccination. Placental transfer ratio varied from 0.4 to 1.4 across five studies. The different clinical trials used different assays that limited direct comparison. There were no significant differences in the risk of serious adverse events (adjusted OR 0.73; 95 % CI 0.49-1.07), serious adverse events leading to withdrawal (adjusted OR 0.44; 95 % CI 0.13-1.51), and systemic illness or fever (adjusted OR 1.05; 95 % CI 0.26-4.19) between the vaccine and placebo groups.
CONCLUSIONS: The published clinical trials show significant IgG GMC response in subjects receiving the conjugated capsular polysaccharide and surface subunit protein vaccines compared to placebo. In current clinical trials of experimental GBS maternal vaccines, there have been no observed serious adverse events of

special interest directly linked to vaccination.

3. J Pharm Policy Pract. 2023 Dec 27;17(1):232-248. doi:
10.1080/20523211.2023.2292717. eCollection 2024.
A cross-sectional assessment of the effects of select training modalities on vaccine cold chain management.
Daniel A(1)(2), Oloro J(3), Hahirwa I(1), Rizinde T(1), Mukanyangezi MF(1).
Author information:
(1)EAC Regional Centre of Excellence for Vaccines, Immunization and Health Supply Chain Management, College of Medicine and Health Sciences, University of Rwanda, Kigali, Rwanda.
(2)Ministry of Health UgandaLira Regional Referral Hospital Aguma Daniel (A.D), Mbarara, Uganda.
(3)Department of Pharmacology & Therapeutics (O.J), Mbarara University of Science & Technology, Lira, Uganda.
BACKGROUND: Vaccines offer arguably the most cost-effective public health intervention. Vaccine supply chain management which is a critical building block faces many Human resources challenges mainly due to the special attributes of vaccines.
OBJECTIVE: This study attempted to measure the effect of training on vaccine cold chain handler knowledge and practices.
METHODS: A cross-sectional research design, using predominantly quantitative data collection techniques, was used. Facilities that have offered vaccination services for more than a year and report through the HMIS system were eligible for selection. Observation checklists and structured questionnaires were used. SPSS was used to analyse data.
RESULTS: Vaccine cold chain management among the study group had an average score of 65.33% range (31-85%). The average knowledge score among the study respondents was 62.42% with a range (45-95%). The knowledge of respondents generally increases with an additional increase in the number of training modalities.
CONCLUSIONS: The status of VCCM is at about 65.33% below the target of 80% set by the EVM. The trainings have an effect on both knowledge of handlers and their practice especially when deployed in a multi-pronged design and thus these trainings need to be aligned to achieve synergy.
ABBREVIATIONS: CCE, Cold Chain Equipment; DHIS2, District Health Information Systems 2; DHO, District Health Officer; DPT, Diphtheria, Pertussis, Tetanus; DVS, District Vaccine Stores; EPI, Expanded Program for Immunisation; EVM, Effective Vaccine Management; FEFO, First Expiry First Out; GAVI, Global Alliance for Vaccines and Immunisation; HMIS, Health Information Management Systems; IRC, International Rescue Committee; KII, Key Informant Interview; LIAT, logistics indicator assessment tool; PATH, Program for Appropriate Technology in Health; PHC, Primary Health Care; QPPU, Quantification and Planning and Procurement Unit; SOPs, Standard Operating Procedures; SPSS, Statistical Package for Social Sciences; UNEPI, Uganda National Expanded Program for Immunisation; UNICEF, United Nations Children's Fund; VPD, Vaccine Preventable Diseases; VVM, Vaccine Vial Monitors; WHO, World Health

Organisation.

4. J Formos Med Assoc. 2024 Jan;123(1):16-22. doi: 10.1016/j.jfma.2023.06.001. Epub 2023 Jun 22.
Sudan virus disease - A quick review.
Tsou TP(1).
Author information:
(1)Division of Emerging Infectious Diseases and Pandemic Preparedness, Taiwan Centers for Disease Control, 3F, No 6, Linsen S. Road, Taipei, Taiwan.
Electronic address: tsungpei@cdc.gov.tw.

The recent Sudan virus disease (SVD) outbreak in Uganda is a reminder of threat from Filovirus diseases. Unlike Ebola virus disease, no effective antiviral and vaccine is available for SVD. The outbreak was declared over after 115 days, with 142 confirmed cases and case fatality rate of 39%, before any dose of candidate vaccine could be used on contacts. We provide a quick review of up-to-date information on the Uganda outbreak, summary of previous outbreaks, and detail the existing SVD treatment and vaccine candidates. Evolution of disease attributes and the impact on public health were also discussed. For high consequence infectious disease like SVD, it takes international collaboration to be better prepared for the next outbreak.

5. Glob Health Action. 2023 Dec 31;16(1):2272390. doi: 10.1080/16549716.2023.2272390. Epub 2023 Nov 9.
The influence of internship training experience on Kenyan and Ugandan doctors' career intentions and decisions: a qualitative study.
Zhao Y(1), Mbuthia D(2), Ankomisyani DS(3), Blacklock C(1), Gathara D(2)(4), Molyneux S(1)(5), Nicodemo C(6)(7), Okello TR(8), Rutebemberwa E(9), Tweheyo R(9)(10), English M(1)(2).
Author information:
(1)NDM Centre for Global Health Research, Nuffield Department of Medicine, University of Oxford, Oxford, UK.
(2)KEMRI-Wellcome Trust Research Programme, Nairobi, Kenya.
(3)Uganda Virus Research Institute - International AIDS Vaccine Initiative HIV Vaccine Program (UVRI-IAVI), Entebbe, Uganda.
(4)MARCH Centre, London School of Hygiene and Tropical Medicine, London, UK.
(5)KEMRI-Wellcome Trust Research Programme, Kilifi, Kenya.
(6)Nuffield Department of Primary Care Health Sciences, University of Oxford, Oxford, UK.
(7)Department of Economics, Verona University, Verona, Italy.
(8)Department of Surgery, Faculty of Medicine, Lira University, Lira, Uganda.
(9)Department of Health Policy, Planning and Management, Makerere University School of Public Health, Kampala, Uganda.
(10)Centre for Health Systems Research and Development (CHSRD), The University of Free State, Bloemfontein, South Africa.
BACKGROUND: Medical internship is a key period for doctors' individual career planning and also a transition period for the broader labour market.

OBJECTIVES: We aimed to understand the complex set of factors influencing the career intentions and decisions of junior doctors, post-internship in Kenya and Uganda.
METHODS: We conducted semi-structured interviews with 54 junior medical officers and 14 consultants to understand doctors' internship experiences and subsequent employment experiences. We analysed the data using a mix of a direct content approach, informed by an internship experience and career intentions framework developed primarily from high-income country literature, alongside a more inductive thematic analysis.
RESULTS: Echoing the internship experience and career intentions framework, we found that clinical exposure during internship, work-life balance, aspects of workplace culture such as relationships with consultants and other team members, and concerns over future job security and professional development all influenced Kenyan and Ugandan doctors' career preferences. Additionally, we added a new category to the framework to reflect our finding that interns might want to 'fill a health system gap' when they choose their future careers, based on what they witness as interns. However, often career intentions did not match career and employment decisions due to specific contextual factors, most importantly a shortage of job opportunities.
CONCLUSION: We have shown how internship experiences shape medical doctors' career intentions in Kenya and Uganda and highlighted the importance of job availability and context in influencing doctors' career choices.

6. Glob Health Action. 2023 Dec 31;16(1):2242672. doi: 10.1080/16549716.2023.2242672.

Changes in self-reported risky sexual behaviour indicators among adults receiving regular risk reduction counselling and optional initiation of pre-exposure prophylaxis in an HIV vaccine preparedness study in Masaka, Uganda.

Kitonsa J(1), Kansiime S(1), Kusemererwa S(1), Onyango M(1), Nayiga B(1), Kabarambi A(2), Mugisha JO(1), Kaleebu P(1)(3), Ruzagira E(1)(4); PrEPVacc Study Group.
Author information:
(1)Medical Research Council/Uganda Virus Research Institute and London School of Hygiene and Tropical Medicine, Uganda Research Unit, Entebbe, Uganda.
(2)Research, International Centre for Child Health and Development (ICHAD), Masaka, Uganda.
(3)Infection Biology, London School of Hygiene and Tropical Medicine, London, UK.
(4)Epidemiology, London School of Hygiene and Tropical Medicine, London, UK.
BACKGROUND: HIV risk reduction counselling may reduce risk-taking behaviours. Yet, concerns remain about risk compensation among individuals initiating pre-exposure prophylaxis (PrEP).
OBJECTIVE: We assessed changes in risky sexual behaviour indicators among HIV vaccine preparedness study participants who received regular risk reduction counselling and referral for PrEP in Masaka, Uganda.
METHODS: Adults (18-39 years) at high risk of HIV infection were enrolled in the study between July 2018 and December 2021. Data were collected on

socio-demographic factors (baseline) and self-reported sexual risk behaviours (baseline, six monthly). HIV testing and risk-reduction counselling and referral for PrEP were done quarterly. Participants who had completed at least 1 year of follow-up were included in the analysis. Proportional differences and McNemar chi-square tests were used to assess changes in the prevalence of self-reported risky sexual behaviour indicators between baseline and 1 year. Logistic regression was used to assess the predictors of unchanged/increased HIV risk at 1 year.
RESULTS: Three hundred participants [132 (44%) females, 152 (51%) aged ≤24 years] were included in this analysis. Eighty-one (27%) participants initiated PrEP at 1 year. Compared to baseline, there were significant reductions in the prevalence of the following self-reported HIV risk indicators at 1 year (overall, among non-PrEP initiators, and among PrEP initiators): transactional sex, ≥6 sexual partners, unprotected sex with ≥3 partners, sex while drunk, and sexually transmitted infection diagnosis/treatment. Percentage differences ranged from 10% for individuals reporting at least six sexual partners to 30% for those reporting unprotected sex with three or fewer sexual partners. There was weak evidence of association between female gender and unchanged/increased HIV risk at 1 year (adjusted OR: 1.35, 95% CI (0.84-2.17)). No other indicators, including PrEP use, were associated with unchanged/increased HIV risk at 1 year.
CONCLUSION: Regular risk-reduction counselling may reduce risky sexual behaviour, while PrEP initiation may not lead to risk compensation.

7. Vaccines (Basel). 2023 Dec 28;12(1):34. doi: 10.3390/vaccines12010034.
Quantitative Synthesis of Factors Associated with COVID-19 Vaccine Acceptance and Vaccine Hesitancy in 185 Countries.
Dinga JN(1)(2), Kabakama S(3), Njimoh DL(4), Chia JE(5), Morhason-Bello I(6), Lumu I(7).
Author information:
(1)Michael Gahnyam Gbeugvat Foundation, Buea P.O. Box 63, Cameroon.
(2)Biotechnology Unit, University of Buea, Buea P.O. Box 63, Cameroon.
(3)Humanitarian and Public Health Consultant, Mwanza P.O. Box 511, Tanzania.
(4)Department of Biochemistry and Molecular Biology, University of Buea, Buea P.O. Box 63, Cameroon.
(5)World Health Organization-Regional Office for Africa, Brazaville P.O. Box 06, Congo.
(6)College of Medicine, University of Ibadan, Ibadan 200284, Nigeria.
(7)Infectious Diseases Institute, Makerere University College of Health Sciences, Kampala P.O. Box 7072, Uganda.
Mass vaccination against COVID-19 is the best method to ensure herd immunity in order to curb the effect of the pandemic on the global economy. It is therefore important to assess the determinants of COVID-19 vaccine acceptance and hesitancy on a global scale. Factors were recorded from cross-sectional studies analyzed with t-Test, ANOVA, correlation, and meta-regression analyses and synthesized to identify global trends in order to inform policy. We registered the protocol (ID: CRD42022350418) and used standard Cochrane methods and PRISMA guidelines to collect and synthesize cross-sectional articles published between

January 2020 and August 2023. A total of 67 articles with 576 studies from 185 countries involving 3081,766 participants were included in this synthesis.
Global COVID-19 vaccine acceptance was 65.27% (95% CI; 62.72-67.84%), while global vaccine hesitancy stood at 32.1% (95% CI; 29.05-35.17%). One-Way ANOVA showed that there was no significant difference in the percentage Gross Domestic Product spent on vaccine procurement across the World Bank income levels (p < 0.187). There was a significant difference of vaccine acceptance (p < 0.001) and vaccine hesitancy (p < 0.005) across the different World Bank Income levels.
World Bank income level had a strong influence on COVID-19 vaccine acceptance (p < 0.0004) and hesitancy (p < 0.003) but percentage Gross Domestic Product spent on vaccine procurement did not. There was no correlation between percentage Gross Domestic Product spent on vaccine procurement and COVID-19 vaccine acceptance (r = -0.11, p < 0.164) or vaccine hesitancy (r = -0.09, p < 0.234).
Meta-regression analysis showed that living in an urban setting (OR = 4.83, 95% CI; 0.67-212.8), rural setting (OR = 2.53, 95% CI; 0.29-119.33), older (OR = 1.98, 95% CI; 0.99-4.07), higher education (OR = 1.76, 95% CI; 0.85-3.81), and being a low income earner (OR = 2.85, 95% CI; 0.45-30.63) increased the odds of high COVID-19 vaccine acceptance. Factors that increased the odds of high COVID-19 vaccine hesitancy were no influenza vaccine (OR = 33.06, 95% CI; 5.03-1395.01), mistrust for vaccines (OR = 3.91, 95% CI; 1.92-8.24), complacency (OR = 2.86, 95% CI; 1.02-8.83), pregnancy (OR = 2.3, 95% CI; 0.12-141.76), taking traditional herbs (OR = 2.15, 95% CI; 0.52-10.42), being female (OR = 1.53, 95% CI; 0.78-3.01), and safety concerns (OR = 1.29, 95% CI; 0.67-2.51). We proposed a number of recommendations to increase vaccine acceptance and ensure global herd immunity against COVID-19.

8. Cell Rep. 2023 Dec 26;42(12):113450. doi: 10.1016/j.celrep.2023.113450. Epub 2023 Nov 28.

High frequency of HIV precursor-target-specific B cells in sub-Saharan populations.

Matassoli F(1), Cagigi A(2), Shen CH(2), Henry AR(2), Johnston TS(2), Schramm CA(2), Cottrell CA(3), Kalyuzhniy O(3), Spangler A(2), Eller L(4), Robb M(4), Eller M(5), Naluyima P(6), Kwong PD(7), Douek DC(2), Schief WR(3), Andrews SF(8), McDermott AB(2).
Author information:
(1)Vaccine Research Center, National Institute of Allergy and Infectious Diseases, National Institutes of Health, Bethesda, MD, USA. Electronic address: flavio.matassoli@nih.gov.
(2)Vaccine Research Center, National Institute of Allergy and Infectious Diseases, National Institutes of Health, Bethesda, MD, USA.
(3)Department of Immunology and Microbial Science, The Scripps Research Institute, La Jolla, CA, USA.
(4)U.S. Military HIV Research Program, Walter Reed Army Institute of Research, Silver Spring, MD, USA; Henry M. Jackson Foundation for the Advancement of Military Medicine, Bethesda, MD, USA.
(5)U.S. Military HIV Research Program, Walter Reed Army Institute of Research, Silver Spring, MD, USA.

(6)Makerere University Walter Reed Project, Kampala, Uganda.
(7)Vaccine Research Center, National Institute of Allergy and Infectious Diseases, National Institutes of Health, Bethesda, MD, USA; Department of Biochemistry and Molecular Biophysics, Columbia University, New York, NY, USA.
(8)Vaccine Research Center, National Institute of Allergy and Infectious Diseases, National Institutes of Health, Bethesda, MD, USA. Electronic address: sarah.andrews2@nih.gov.
HIV gp120 engineered outer domain germline-targeting version 8 (eOD-GT8) was designed specifically to engage naive B cell precursors of VRC01-class antibodies. However, the frequency and affinity of naive B cell precursors able to recognize eOD-GT8 have been evaluated only in U.S. populations. HIV infection is disproportionally concentrated in sub-Saharan Africa, so we seek to characterize naive B cells able to recognize eOD-GT8 in sub-Saharan cohorts. We demonstrate that people from sub-Saharan Africa have a higher or equivalent frequency of naive B cells able to engage eOD-GT8 compared with people from the U.S. Genetically, the higher frequency of eOD-GT8-positive cells is accompanied by a higher level of naive B cells with gene signatures characteristic of the VRC01 class, as well as other CD4bs-directed antibodies. Our study demonstrates that vaccination with eOD-GT8 in sub-Saharan Africa could be successful at expanding and establishing a pool of CD4bs-directed memory B cells from naive precursors.

9. Ther Adv Infect Dis. 2023 Dec 25;10:20499361231220152. doi: 10.1177/20499361231220152. eCollection 2023 Jan-Dec.
Respiratory morbidity in Schistosoma mansoni infection: a rapid review of literature.
Baluku JB(1)(2), Olum R(3), Sanya RE(1)(4), Ocama P(1)(5).
Author information:
(1)Immunomodulation and Vaccines Programme, MRC/UVRI and LSHTM Uganda Research Unit, Entebbe, Uganda.
(2)Department of Internal Medicine, Makerere University College of Health Sciences, PO Box 26343, Kampala, Uganda.
(3)St. Francis Hospital, Nsambya, Kampala, Uganda.
(4)Chronic Diseases Management Unit, African Population and Health Research Center, Nairobi, Uganda.
(5)Department of Internal Medicine, Makerere University College of Health Sciences, Kampala, Uganda.
BACKGROUND: Schistosomiasis contributes to 2.5 million disability-adjusted life years globally. Acute and chronic respiratory morbidity of Schistosoma mansoni (S. mansoni) is poorly documented in the literature. We conducted a rapid literature review of the burden of respiratory symptoms and lung function abnormalities among patients with S. mansoni. We also report the immunologic and lung imaging findings from the studies reviewed.
METHODS: We carried out a comprehensive literature search in Embase and MEDLINE from the inception of the databases to 13th March 2023.
RESULTS: A total of 2243 patients with S. mansoni were reported from 24 case reports, 11 cross-sectional studies, 7 case series, 2 cohort studies and 2

randomized controlled trials. The prevalence of any respiratory symptom was 13.3-63.3% (total number of patients studied, n = 149). The prevalence of the individual symptoms among patients with S. mansoni in whom respiratory symptoms were sought for was as follows: cough (8.3-80.6%, n = 338), dyspnea (1.7-100.0%, n = 200), chest pain (9.0-57.1%, n = 86), sputum production (20.0-23.3%, n = 30) and wheezing (0.0 - 20.0%, n = 1396). The frequency of the symptoms tended to be higher in acute schistosomiasis. Restrictive lung disease was prevalent in 29.0% (9/31). The commonest chest imaging findings reported were nodules (20-90%, n = 103) and interstitial infiltrates (12.5-23.0%, n = 89). Peripheral blood eosinophilia was prevalent in 72.0-100.0% of patients (n = 130) with acute schistosomiasis and correlated with symptoms and imaging abnormalities. Three case reports in chronic S. mansoni reported elevated C-reactive protein, leucocyte, neutrophil and absolute eosinophil counts, eosinophil percentage, IgE and IgG4.
CONCLUSION: There is a high prevalence of respiratory morbidity among patients with S. mansoni, particularly in the acute stage of the infection, although the studies are relatively small. Larger studies are needed to characterize respiratory morbidity in chronic schistosomiasis and determine the underlying clinical and immunological mechanisms.

10. AIDS. 2023 Dec 22. doi: 10.1097/QAD.0000000000003824. Online ahead of print.
PrEP Availability Among Health Facilities Participating in the Global IeDEA Consortium.
Kebede S(1), Brazier E(2)(3), Freeman AM(4), Muwonge TR(5), Choi JY(6), de Waal R(7), Poda A(8), Cesar C(9), Munyaneza A(10), Kasozi C(11), Pasayan MKU(12), Althoff KN(13), Shongo A(14), Low N(15), Ekouevi D(16), Veloso VG(17), Ross J(18); International Epidemiology Databases to Evaluate AIDS (IeDEA).
Author information:
(1)Department of Medicine, New York University Grossman School of Medicine.
(2)Institute for Implementation Science in Population Health, City University of New York.
(3)Graduate School of Public Health and Health Policy, City University of New York, New York, NY.
(4)Johns Hopkins University, Baltimore, MD, USA.
(5)Infectious Diseases Institute, Makerere University, Kampala Uganda.
(6)Division of Infectious Diseases, Department of Internal Medicine, Yonsei University College of Medicine, Seoul, South Korea.
(7)Centre for Infectious Disease Epidemiology and Research, University of Cape Town, Cape Town, South Africa.
(8)Hôpital de Jour, Service des Maladies Infectieuses, CHU Sourô Sanou, Bobo-Dioulasso, Burkina Faso.
(9)Fundación Huésped, Buenos Aires, Argentina.
(10)Rwanda Military Hospital, Kigali, Rwanda.
(11)Masaka Regional Referral Hospital, Uganda.
(12)Research Institute for Tropical Medicine, Muntinlupa City, Philippines.
(13)Department of Epidemiology, Johns Hopkins Bloomberg School of Public Health, Baltimore, MD, USA.

(14)School of Public Health, University of Kinshasa, Kinshasa, Democratic Republic of Congo.
(15)Institute of Social and Preventive Medicine, Bern, Switzerland.
(16)Université de Lomé, Lomé, Togo.
(17)Instituto Nacional de Infectologia Evandro Chagas, Rio de Janerio, Brazil.
(18)Department of Medicine, Albert Einstein College of Medicine, Bronx, NY, USA.
BACKGROUND: While recognized as a key HIV prevention strategy, preexposure prophylaxis (PrEP) availability and accessibility are not well documented globally. We aimed to describe PrEP drug registration status and the availability of PrEP services across HIV care sites participating in the International epidemiology Databases to Evaluate AIDS (IeDEA) research consortium.
METHODS: We used country-level PrEP drug registration status from the AIDS Vaccine Advocacy Coalition and data from IeDEA surveys conducted in 2014, 2017 and 2020 among participating HIV clinics in seven global regions. We used descriptive statistics to assess PrEP availability across IeDEA sites serving adult patients in 2020 and examined trends in PrEP availability among sites that responded to all three surveys.
RESULTS: Of 199 sites that completed the 2020 survey, PrEP was available in 161 (81%). PrEP availability was highest at sites in North America (29/30; 97%) and East Africa (70/74; 95%) and lowest at sites in Central (10/20; 50%) and West Africa (1/6; 17%). PrEP availability was higher among sites in countries where PrEP was officially registered (146/161; 91%) than where it was not (14/32; 44%). Availability was higher at health centers (109/120; 90%) and district hospitals (14/16; 88%) compared to regional/teaching hospitals (36/63). Among the 94 sites that responded to all three surveys, PrEP availability increased from 47% in 2014 to 60% in 2017 and 76% in 2020.
CONCLUSION: PrEP availability has substantially increased since 2014 and is now available at most IeDEA sites. However, PrEP service provision varies markedly across global regions.

11. Bio Protoc. 2023 Dec 20;13(24):e4905. doi: 10.21769/BioProtoc.4905. eCollection 2023 Dec 20.
An Optimised Indirect ELISA Protocol for Detection and Quantification of Anti-viral Antibodies in Human Plasma or Serum: A Case Study Using SARS-CoV-2.
Baine C(1), Sembera J(1)(2), Kevin Oluka G(1)(2), Katende JS(1)(2), Ankunda V(1)(2), Serwanga J(1)(2).
Author information:
(1)Department of Immunology, Uganda Virus Research Institute, Entebbe, Uganda.
(2)Medical Research Council, Uganda Virus Research Institute and London School of Hygiene and Tropical Medicine, Uganda Research Unit, Entebbe, Uganda.
Advanced immunoassays are crucial in assessing antibody responses, serving immune surveillance goals, characterising immunological responses to evolving viral variants, and guiding subsequent vaccination initiatives. This protocol outlines an indirect ELISA protocol to detect and quantify virus-specific antibodies in plasma or serum after exposure to viral antigens. The assay enables the measurement of IgG, IgA, and IgM antibodies specific to the virus of

interest, providing qualitative and quantitative optical densities and concentration data. Although this protocol refers to SARS-CoV-2, its methodology is versatile and can be modified to assess antibody responses for various viral infections and to evaluate vaccine trial outcomes. Key features • This protocol builds upon previously described methodology [1] explicitly tailored for SARS-CoV-2 and broadens its applicability to other viral infections. • The protocol outlines establishing antibody responses to SARS-CoV-2 infections by determining optical densities and concentrations from blood plasma or serum.

12. BMJ Open. 2023 Dec 20;13(12):e076184. doi: 10.1136/bmjopen-2023-076184.

Health service factors affecting the COVID-19 vaccination campaign in a Ghanaian metropolis: A qualitative exploratory study.

Abraham SAA(1), Amoah JO(2), Agyare DF(3), Sekimpi DK(4), Bosomtwe-Duker D(5), Druye AA(3), Osei Berchie G(6), Obiri-Yeboah D(7)(8)(9).
Author information:
(1)Department of Public Health, School of Nursing and Midwifery, College of Health and Allied Sciences, University of Cape Coast, Cape Coast, Ghana.
(2)Centre for Gender Research, Advocacy and Documentation (CEGRAD), University of Cape Coast College of Humanities and Legal Studies, Cape Coast, Ghana.
(3)Department of Adult Health, School of Nursing and Midwifery, College of Health and Allied Sciences, University of Cape Coast, Cape Coast, Ghana.
(4)Uganda National Association of Community Occupational Health, Kampala, Uganda.
(5)Nursing and Midwifery Training College, 37 Military Hospital, Accra, Ghana.
(6)Department of Maternal and Child Health, School of Nursing and Midwifery, College of Health and Allied Sciences, University of Cape Coast, Cape Coast, Ghana gberchie@ucc.edu.gh.
(7)Directorate of Research, Innovation and Consultancy, University of Cape Coast, Cape Coast, Ghana.
(8)Microbiology and Immunology Department, School of Medical Sciences, College of Health and Allied Sciences, University of Cape Coast, Cape Coast, Ghana.
(9)Clinical Microbiology/Public Health Unit, Cape Coast Teaching Hospital, Cape Coast, Ghana.
OBJECTIVE: The study sought to explore the perspectives of vaccinators on the health system factors that impacted the COVID-19 vaccination campaign.
DESIGN: The study employed an exploratory-descriptive qualitative design. Key-informants' interviews were conducted using semi-structured guide to gather the data. Thematic analysis following the steps of Braun and Clark was conducted using ATLAS.ti software.
SETTING: The study setting was the Cape Coast Metropolis where the Central Regional Health Directorate is located. The Directorate initiates and implements policy decisions across the region. It is also the only metropolis in the region that recorded about 5970 of the total COVID-19 cases recorded in Ghana.
PARTICIPANTS: Eleven vaccinators who had been trained for the COVID-19 vaccination and had participated in the campaign for at least 6 months were purposively sampled through the Regional Public Health Unit.

RESULTS: Four themes were derived from the data after analysis; 'vaccine-related issues'; 'staffing issues'; 'organising and planning the campaign' and 'surveillance and response systems'. Subthemes were generated under each major theme. Our results revealed the health service promoted the COVID-19 vaccination campaign through public education and ensured access to COVID-19 vaccines through the use of community outreaches. Also, the health service ensured adequate logistics supply for carrying out the campaign as well as ensured vaccinators were adequately equipped for adverse incidence reporting and management. Dissatisfaction among COVID-19 vaccinators attributed to low remuneration and delays in receiving allowances as well as shortfalls in efforts at securing transportation and a conducive venue for the vaccination exercise also emerged. Other challenges in the vaccination campaign were attributed to poor data entry platforms and limited access to internet facilities.
CONCLUSION: This study highlights the health system's strategies and challenges during the COVID-19 vaccination campaign, emphasising the need for critical interventions to prevent low vaccination rates.

13. PLOS Glob Public Health. 2023 Dec 19;3(12):e0001884. doi: 10.1371/journal.pgph.0001884. eCollection 2023.
Public perceptions of Ebola vaccines and confidence in health services to treat Ebola, malaria, and tuberculosis: Findings from a cross-sectional household survey in Uganda, 2020.
Koyuncu A(1), Carter RJ(2), Musaazi J(3), Namageyo-Funa A(1), Carter VM(1), Lamorde M(3), Prybylski D(1), Apondi R(1), Bakyaita T(4), Boore AL(1), Homsy J(1)(5), Brown VR(1), Kigozi J(3), Nabaggala MS(3), Nakate V(3), Nkurunziza E(3), Stowell DF(1), Walwema R(3), Olowo A(3), Jalloh MF(1).
Author information:
(1)Center for Global Health, U.S. Centers for Disease Control and Prevention, Atlanta, Georgia, United States of America.
(2)National Center for Immunization and Respiratory Diseases, U.S. Centers for Disease Control and Prevention, Atlanta, Georgia, United States of America.
(3)Infectious Diseases Institute, College of Health Sciences, Makerere University, Kampala, Uganda.
(4)Uganda Ministry of Health, Kampala, Uganda.
(5)Institute for Global Health Sciences, University of California San Francisco, San Francisco, California, United States of America.
Uganda used Ebola vaccines as part of its preparedness and response during the 2018-2020 10th Ebola virus disease (EVD) outbreak in neighboring Democratic Republic of the Congo (DRC). We evaluated the public's perceptions of Ebola vaccines and compared their confidence in health services to treat Ebola versus malaria and tuberculosis as part of a survey on Ebola knowledge, attitudes, and practices (KAP) conducted in March 2020. A cross-sectional household survey was implemented in six districts in Uganda using multi-stage cluster sampling to randomly select participants. The districts were purposively selected from districts classified by the government as at high- or low-risk for an EVD outbreak. We describe perceptions of Ebola vaccines and confidence in health services to treat Ebola, tuberculosis, and malaria. Modified Poisson regression

modeling was used to identify the demographic correlates of these outcomes. Among 3,485 respondents, 18% were aware of Ebola vaccines. Of those, 92% agreed that the vaccines were needed to prevent Ebola. Participants aged 15-24 years were 4% more likely to perceive such need compared to those 60 years and older (adjusted prevalence ratio [aPR] 1.04, 95% confidence interval [CI] 1.0-1.08). The perceived need was 5% lower among participants with at least some secondary education compared to uneducated participants (aPR 0.95; 0.92-0.99). Overall, 81% of those aware of the vaccines believed that everyone or most people in their community would get vaccinated if offered, and 94% said they would likely get vaccinated if offered. Confidence in health services to treat Ebola was lower compared to treating malaria or tuberculosis (55% versus 93% and 77%, respectively). However, participants from the EVD high-risk districts were 22% more likely to be confident in health services to treat Ebola compared to those in low-risk districts (aPR: 1.22; 95% CI: 1.08, 1.38). Our findings suggest that intent to take an Ebola vaccine during an outbreak was strong, but more work needs to be done to increase public awareness of these vaccines. The public's high confidence in health services to treat other health threats, such as malaria and tuberculosis, offer building blocks for strengthening their confidence in health services to treat EVD in the event of an outbreak.

14. iScience. 2023 Nov 20;26(12):108496. doi: 10.1016/j.isci.2023.108496. eCollection 2023 Dec 15.
Atypical B cells consist of subsets with distinct functional profiles.
Reyes RA(1), Batugedara G(1), Dutta P(2), Reers AB(1), Garza R(1), Ssewanyana I(3)(4), Jagannathan P(5)(6), Feeney ME(7)(8), Greenhouse B(7), Bol S(1), Ay F(2)(9), Bunnik EM(1).
Author information:
(1)Department of Microbiology, Immunology and Molecular Genetics, Long School of Medicine, The University of Texas Health Science Center at San Antonio, San Antonio, TX 78229, USA.
(2)Centers for Cancer Immunotherapy and Autoimmunity, La Jolla Institute for Immunology, La Jolla, CA 92037, USA.
(3)Infectious Disease Research Collaboration, Kampala, Uganda.
(4)Department of Infection Biology, London School of Hygiene and Tropical Medicine, London WC1E 7HT, UK.
(5)Department of Medicine, Division of Infectious Diseases, Stanford University, Stanford, CA 94305, USA.
(6)Department of Microbiology and Immunology, Stanford University, Stanford, CA 94305, USA.
(7)Department of Medicine, University of California, San Francisco, San Francisco, CA 94110, USA.
(8)Department of Pediatrics, University of California, San Francisco, San Francisco, CA 94110, USA.
(9)Department of Pediatrics, University of California San Diego, La Jolla, CA 92093, USA.
Atypical B cells are a population of activated B cells that are commonly enriched in individuals with chronic immune activation but are also part of a

normal immune response to infection or vaccination. To better define the role of atypical B cells in the human adaptive immune response, we performed single-cell sequencing of transcriptomes, cell surface markers, and B cell receptors in individuals with chronic exposure to the malaria parasite Plasmodium falciparum, a condition known to lead to accumulation of circulating atypical B cells. We identified three previously uncharacterized populations of atypical B cells with distinct transcriptional and functional profiles and observed marked differences among these three subsets in their ability to produce immunoglobulin G upon T-cell-dependent activation. Our findings help explain the conflicting observations in prior studies regarding the function of atypical B cells and highlight their different roles in the adaptive immune response in chronic inflammatory conditions.

15. J Mol Biol. 2023 Dec 15;435(24):168344. doi: 10.1016/j.jmb.2023.168344. Epub 2023 Nov 4.

Antibody and B-cell Immune Responses Against Bordetella Pertussis Following Infection and Immunization.

Abu-Raya B(1), Esser MJ(2), Nakabembe E(3), Reiné J(4), Amaral K(5), Diks AM(6), Imede E(7), Way SS(8), Harandi AM(9), Gorringe A(10), Le Doare K(11), Halperin SA(12), Berkowska MA(13), Sadarangani M(5).

Author information:

(1)Vaccine Evaluation Center, BC Children's Hospital Research Institute, Vancouver, BC, Canada; Department of Pediatrics, University of British Columbia, Vancouver, BC, Canada. Electronic address: baburaya@bcchr.ubc.ca.

(2)Department of Pediatrics, Maastricht University Medical Center, Maastricht, the Netherlands.

(3)Centre for Neonatal and Paediatric Infectious Diseases Research, St George's, University of London, Cranmer Terrace, London SW17 0RE, UK; Department of Obstetrics and Gynaecology, Makerere University College of Health Sciences, Upper Mulago Hill Road, Kampala, P.O. Box 7072, Uganda.

(4)Clinical Sciences, Liverpool School of Tropical Medicine, Liverpool, United Kingdom; Oxford Vaccine Group, University of Oxford, Oxford, United Kingdom.

(5)Vaccine Evaluation Center, BC Children's Hospital Research Institute, Vancouver, BC, Canada; Department of Pediatrics, University of British Columbia, Vancouver, BC, Canada.

(6)Department of Immunology, Leiden University Medical Center, Albinusdreef 2, Leiden ZA 2333, the Netherlands.

(7)MRC/UVRI and LSHTM Uganda Research Unit, Entebbe, Uganda.

(8)Department of Pediatrics, Division of Infectious Diseases, Center for Inflammation and Tolerance, Cincinnati Children's Hospital Medical Center, University of Cincinnati School of Medicine, Cincinnati, OH, USA.

(9)Vaccine Evaluation Center, BC Children's Hospital Research Institute, Vancouver, BC, Canada; Department of Microbiology and Immunology, University of Gothenburg, Gothenburg, Sweden.

(10)UK Health Security Agency, Porton Down, Salisbury SP4 0JG, UK.

(11)Centre for Neonatal and Paediatric Infectious Diseases Research, St George's, University of London, Cranmer Terrace, London SW17 0RE, UK; Makerere

University-Johns Hopkins University Research Collaboration, MU-JHU, Upper Mulago Hill, Kampala, P.O. Box 23491, Uganda.
(12)Canadian Center for Vaccinology, Departments of Pediatrics and Microbiology and Immunology, Dalhousie University, Izaak Walton Killam Health Centre, and Nova Scotia Health Authority, Halifax, NS, Canada.
(13)Medical Microbiology, University Medical Center Utrecht, Utrecht University, Utrecht, the Netherlands.
Neither immunization nor recovery from natural infection provides life-long protection against Bordetella pertussis. Replacement of a whole-cell pertussis (wP) vaccine with an acellular pertussis (aP) vaccine, mutations in B. pertussis strains, and better diagnostic techniques, contribute to resurgence of number of cases especially in young infants. Development of new immunization strategies relies on a comprehensive understanding of immune system responses to infection and immunization and how triggering these immune components would ensure protective immunity. In this review, we assess how B cells, and their secretory products, antibodies, respond to B. pertussis infection, current and novel vaccines and highlight similarities and differences in these responses. We first focus on antibody-mediated immunity. We discuss antibody (sub)classes, elaborate on antibody avidity, ability to neutralize pertussis toxin, and summarize different effector functions, i.e. ability to activate complement, promote phagocytosis and activate NK cells. We then discuss challenges and opportunities in studying B-cell immunity. We highlight shared and unique aspects of B-cell and plasma cell responses to infection and immunization, and discuss how responses to novel immunization strategies better resemble those triggered by a natural infection (i.e., by triggering responses in mucosa and production of IgA). With this comprehensive review, we aim to shed some new light on the role of B cells and antibodies in the pertussis immunity to guide new vaccine development.

16. Clin Infect Dis. 2023 Dec 15;77(12):1659-1667. doi: 10.1093/cid/ciad440.
Oral Lipid Nanocrystal Amphotericin B for Cryptococcal Meningitis: A Randomized Clinical Trial.
Boulware DR(1), Atukunda M(2), Kagimu E(2), Musubire AK(2), Akampurira A(2), Tugume L(2), Ssebambulidde K(2)(3), Kasibante J(2), Nsangi L(2), Mugabi T(2), Gakuru J(2), Kimuda S(2), Kasozi D(2), Namombwe S(2), Turyasingura I(2), Rutakingirwa MK(2), Mpoza E(2), Kigozi E(4), Muzoora C(4), Ellis J(2), Skipper CP(1), Matkovits T(5), Williamson PR(3), Williams DA(1), Fieberg A(6), Hullsiek KH(6), Abassi M(1), Dai B(6), Meya DB(1)(2).
Author information:
(1)Department of Medicine, University of Minnesota, Minneapolis, Minnesota, USA.
(2)Infectious Diseases Institute, Makerere University, Kampala, Uganda.
(3)Laboratory of Clinical Immunology and Microbiology, National Institute of Allergy and Infectious Diseases, National Institutes of Health, Bethesda, Maryland, USA.
(4)Department of Medicine, Mbarara University of Science and Technology, Mbarara, Uganda.
(5)Matinas Biopharma Nanotechnologies, Bedminster, New Jersey, USA.

(6)Division of Biostatistics, School of Public Health, University of Minnesota, Minneapolis, Minnesota, USA.
BACKGROUND: Amphotericin B is the gold standard treatment for severe mycoses. A new orally delivered, less-toxic formulation of amphotericin has been developed.
METHODS: In our randomized clinical trial, we tested oral lipid nanocrystal (LNC) amphotericin B (MAT2203, Matinas Biopharma) vs intravenous (IV) amphotericin for human immunodeficiency virus-associated cryptococcal meningitis in 4 sequential cohorts. Two pilot cohorts assessed safety and tolerability (n = 10 each), and 2 cohorts assessed efficacy with/without 2 IV loading doses (n = 40 each). The experimental arm received 1.8 g/d oral LNC amphotericin through 2 weeks with 100 mg/kg/d flucytosine, then 1.2 g/d LNC amphotericin through 6 weeks. The randomized control arm (n = 41) received 7 days of IV amphotericin with flucytosine, then 7 days of fluconazole 1200 mg/d. The primary end point was cerebrospinal fluid (CSF) early fungicidal activity (EFA).
RESULTS: We randomized 80 participants to oral LNC amphotericin + flucytosine with (n = 40) and without (n = 40) 2 IV loading doses and 41 control participants to IV amphotericin + flucytosine. Mean EFA was 0.40 log10 colony-forming units (CFU)/mL/d for all-oral LNC amphotericin, 0.42 log10 Cryptococcus CFU/mL/d for oral LNC amphotericin with IV loading doses, and 0.46 log10 CFU/mL/d for IV amphotericin controls. LNC amphotericin groups achieved 2-week CSF sterility in 63% (44 of 70) vs 68% (23 of 34) of controls. The 18-week survival was 85% (34 of 40) with all-oral LNC amphotericin, 90% (36 of 40) with oral LNC amphotericin given IV loading doses, and 85% (35 of 41) with IV amphotericin.Grade 3-4 laboratory adverse events occurred less frequently in LNC amphotericin groups (41%) than the IV amphotericin group (61%, P = .05), particularly for anemia (21% vs 44%; P = .01) and potassium (5% vs 17%; P = .04).
CONCLUSIONS: This new oral amphotericin B LNC formulation appears promising for cryptococcal meningitis with antifungal activity, similar survival, and less toxicity than IV amphotericin.
CLINICAL TRIALS REGISTRATION: NCT04031833.

17. J Infect Dis. 2023 Dec 14:jiad550. doi: 10.1093/infdis/jiad550. Online ahead of
Assessing Per-Sex-Act HIV-1 Risk Reduction Among Women using the Dapivirine Vaginal Ring.
Stalter RM(1), Dong TQ(2), Hendrix CW(3), Palanee-Phillips T(4), van der Straten A(5)(6), Hillier SL(7), Kiweewa FM(8), Mgodi NM(9), Marzinke MA(3), Bekker LG(10), Soto-Torres L(11), Baeten JM(1)(12)(13), Brown ER(2)(14); MTN-020/ASPIRE Study Team.
Collaborators: Baeten J, Palanee-Phillips T, Brown E, Soto-Torres L, Schwartz K, Makanani B, Martinson F, Bekker LG, Govender V, Siva S, Gaffoor Z, Naidoo L, Pather A, Jeenarain N, Nair G, Palanee-Phillips T, Kiweewa FM, Mgodi N, Mhlanga F.
Author information:
(1)Department of Epidemiology, University of Washington, Seattle, WA, USA.
(2)Vaccine and Infectious Disease and Public Health Sciences Divisions, Fred Hutchinson Cancer Research Center, Seattle WA, USA.

(3)Department of Medicine, Johns Hopkins University, Baltimore MD, USA.
(4)Wits Reproductive Health and HIV Research Institute, Faculty of Health Sciences, University of the Witwatersrand, Johannesburg South Africa.
(5)University of California San Francisco, Center for AIDS Prevention Studies, Dept of medicine, San Francisco, CA, USA.
(6)ASTRA consulting, Kensington CA, USA.
(7)Department of Obstetrics, Gynecology and Reproductive Sciences, University of Pittsburgh School of Medicine and Magee-Womens Research Institute, Pittsburgh PA, USA.
(8)Makerere University-Johns Hopkins University Research Collaboration, Kampala Uganda.
(9)University of Zimbabwe Clinical Trials Research Centre, Harare Zimbabwe.
(10)Desmond Tutu HIV Foundation Clinical Research Site, Cape Town South Africa.
(11)Division of AIDS, National Institute of Allergy and Infectious Diseases, National Institutes of Health, Bethesda MD, USA.
(12)Departments of Medicine and Global Health, University of Washington, Seattle, WA, USA.
(13)Gilead Sciences, Foster City, CA, USA.
(14)Department of Biostatistics, University of Washington, Seattle WA, USA.
BACKGROUND: Confounding introduced by individuals' sexual risk behavior is potentially a significant source of bias in HIV-1 prevention intervention studies. To more completely account for sexual behaviors when assessing the efficacy of the monthly dapivirine ring, a new longer-acting HIV-1 prevention option for women, we estimated per-sex-act risk reduction associated with product use.
METHODS: We conducted a secondary analysis of data from MTN-020/ASPIRE, a Phase III, randomized, placebo-controlled efficacy trial of the dapivirine ring that recruited HIV-uninfected, African women between ages 18-45. With cumulative sex acts as the time scale, we used multivariable Cox regression with inverse probability of censoring weights to estimate HIV-1 risk reduction associated with a rate of dapivirine release indicative of consistent product use.
RESULTS: Women in the dapivirine ring group (n = 1187) had an estimated incidence rate of 2.3 (95% CI: 1.8-3.1) HIV-1 acquisition events per 10,000 sex acts versus 3.6 (2.9-4.4) per 10,000 acts in the placebo group (n = 1187). Dapivirine release indicative of consistent ring use was associated with a 63% (95% CI: 33-80%) per-sex-act HIV-1 risk reduction.
CONCLUSIONS: These results support the efficacy of the dapivirine vaginal ring for HIV-1 prevention and help to inform decision-making for women, providers, and policymakers regarding product use.

18. Front Immunol. 2023 Dec 13;14:1275443. doi: 10.3389/fimmu.2023.1275443. eCollection 2023.
Divergent neuroimmune signatures in the cerebrospinal fluid predict differential gender-specific survival among patients with HIV-associated cryptococcal meningitis.
Okurut S(1)(2), Boulware DR(3), Okafor E(3), Rhein J(3), Kajumbula H(2), Bagaya BS(4), Bwanga F(2), Olobo JO(4), Manabe YC(1)(5), Meya DB(1)(3)(6), Janoff

EN(7)(8).
Author information:
(1)Translation Sciences Laboratory, Research Department, Infectious Diseases Institute, Makerere University, Kampala, Uganda.
(2)Department of Medical Microbiology, School of Biomedical Sciences, College of Health Sciences, Makerere University, Kampala, Uganda.
(3)Division of Infectious Diseases and International Medicine, Department of Medicine, University of Minnesota, Minneapolis, MN, United States.
(4)Department of Immunology and Molecular Biology, School of Biomedical Sciences, College of Health Sciences, Makerere University, Kampala, Uganda.
(5)Division of Infectious Diseases, Department of Medicine, John Hopkins University School of Medicine, Baltimore, MD, United States.
(6)Department of Medicine, School of Medicine, College of Health Sciences, Makerere University, Kampala, Uganda.
(7)Mucosal and Vaccine Research Program Colorado, Department of Medicine, Division of Infectious Diseases, University of Colorado Denver, Aurora, CO, United States.
(8)Department of Medicine and Infectious Disease, Denver Veterans Affairs Medical Center, Denver, CO, United States.
INTRODUCTION: Survival among people with HIV-associated cryptococcal meningitis (CM) remains low, particularly among women, despite the currently optimal use of antifungal drugs. Cryptococcus dissemination into the central nervous system [brain, spinal cord, and cerebrospinal fluid (CSF)] elicits the local production of cytokines, chemokines, and other biomarkers. However, no consistent diagnostic or prognostic neuroimmune signature is reported to underpin the risk of death or to identify mechanisms to improve treatment and survival. We hypothesized that distinct neuroimmune signatures in the CSF would distinguish survivors from people who died on antifungal treatment and who may benefit from tailored therapy.
METHODS: We considered baseline clinical features, CSF cryptococcal fungal burden, and CSF neuroimmune signatures with survival at 18 weeks among 419 consenting adults by "gender" (168 women and 251 men by biological sex defined at birth).
RESULTS: Survival at 18 weeks was significantly lower among women than among men {47% vs. 59%, respectively; hazard ratio (HR) = 1.4 [95% confidence interval (CI), 1.0 to 1.9; $p = 0.023$]}. Unsupervised principal component analysis (PCA) demonstrated divergent neuroimmune signatures by gender, survival, and intragender-specific survival. Overall, women had lower levels of programmed death ligand 1, Interleukin (IL) (IL-11RA/IL-1F30, and IL-15 (IL-15) than men (all $p < 0.028$). Female survivors compared with those who died expressed significant elevations in levels of CCL11 and CXCL10 chemokines (both $p = 0.001$), as well as increased T helper 1, regulatory, and T helper 17 cytokines (all $p < 0.041$). In contrast, male survivors expressed lower levels of IL-15 and IL-8 compared with men who died ($p < 0.044$).
CONCLUSIONS: Survivors of both genders demonstrated a significant increase in the levels of immune regulatory IL-10. In conclusion, the lower survival among women with CM was accompanied by distinct differential gender-specific

neuroimmune signatures. These female and male intragender-specific survival-associated neuroimmune signatures provide potential targets for interventions to advance therapy to improve the low survival among people with HIV-associated CM.

19. Sci Rep. 2023 Dec 13;13(1):22182. doi: 10.1038/s41598-023-49674-1.
A systematic review reveals that African children of 15-17 years demonstrate low hepatitis B vaccine seroprotection rates.
Muwanda F(1)(2), Sendagire H(3), Mboowa G(4)(5), Kateete DP(4), Achan B(3), Mupere E(6), Kafeero HM(7)(3), Bagaya BS(4).

Author information:
(1)Department of Medical Microbiology, Faculty of Health Sciences, Habib Medical School, Islamic University in Uganda, P.O. Box 7689, Kampala, Uganda. muwandafahad@gmail.com.
(2)Department of Immunology and Molecular Biology, School of Biomedical Sciences, College of Health Sciences, Makerere University, P.O. Box 7072, Kampala, Uganda. muwandafahad@gmail.com.
(3)Department of Medical Microbiology, School of Biomedical Sciences, College of Health Sciences, Makerere University, P.O. Box 7072, Kampala, Uganda.
(4)Department of Immunology and Molecular Biology, School of Biomedical Sciences, College of Health Sciences, Makerere University, P.O. Box 7072, Kampala, Uganda.
(5)The African Center of Excellence in Bioinformatics and Data-Intensive Sciences, Infectious Diseases Institute, College of Health Sciences, Makerere University, P.O. Box 22418, Kampala, Uganda.
(6)Department of Paediatrics and Child Health, School of Medicine, College of Health Sciences, Makerere University, P.O. Box 7072, Kampala, Uganda.
(7)Department of Medical Microbiology, Faculty of Health Sciences, Habib Medical School, Islamic University in Uganda, P.O. Box 7689, Kampala, Uganda.
Childhood HBV immunization remains globally fundamental to the elimination of hepatitis B virus (HBV). However, monitoring proportions of HBV vaccine seroprotection and their determinants among African Pediatric recipients is crucial. This study sought to verify extent of immune protection accorded by the HBV vaccine in African children of up to 17 years of age by pooling the prevalence of seroprotection reported by primary studies conducted in the Northern, Western, and Southern African regions. We included 19 eligible articles out of the 197 initially downloaded, published from 1999 to 2021 from African Journals Online (AJOL), EMBASE, Scopus, and PubMed. The study protocol was registered with the International Prospective Register of Systematic Reviews (PROSPERO), University of York Centre for Reviews and Dissemination, under the registration number CRD42022361277. Significantly higher ($p < 0.0001$) proportion of HBV vaccine seroprotection (69.07%) was found among children under 15 years of age than children 15-17 years (32.368%), 95% CI [34.2454-39.0847%]. Whereas successful integration of the HBV vaccine on the extended programs on immunizations (EPI) has been a major achievement in the reduction of HBV infection in Africa, markedly reduced HBV vaccine seroprotection is persistently

demonstrated among adolescent children 15-17 years of age. Future studies are required to clarify the need for booster dose vaccination in most at risk populations and age groups.

20. Int J Cancer. 2023 Dec 11. doi: 10.1002/ijc.34798. Online ahead of print.
Risk of COVID-19 death for people with a pre-existing cancer diagnosis prior to COVID-19-vaccination: A systematic review and meta-analysis.
Steinberg J(1), Hughes S(1), Hui H(1), Allsop MJ(2), Egger S(1), David M(1)(3), Caruana M(1), Coxeter P(1), Carle C(1), Onyeka T(4)(5), Rewais I(1), Monroy Iglesias MJ(6), Vives N(7)(8), Wei F(9), Abila DB(10), Carreras G(11), Santero M(12), O'Dowd EL(13), Lui G(1), Tolani MA(14), Mullooly M(15), Lee SF(16)(17), Landy R(18), Hanley SJB(19)(20), Binefa G(21)(22), McShane CM(23), Gizaw M(24)(25)(26), Selvamuthu P(27), Boukheris H(28)(29), Nakaganda A(30), Ergin I(31), Moraes FY(32), Timilshina N(33), Kumar A(34), Vale DB(35), Molina-Barceló A(36), Force LM(37)(38), Campbell DJ(1), Wang Y(39), Wan F(39), Baker AL(39), Singh R(39), Salam RA(1), Yuill S(1)(39), Shah R(40), Lansdorp-Vogelaar I(41), Yusuf A(42), Aggarwal A(43)(44), Murillo R(45)(46), Torode JS(47)(48), Kliewer EV(49), Bray F(40), Chan KKW(50)(51), Peacock S(49)(51)(52), Hanna TP(53)(54), Ginsburg O(55), Van Hemelrijck M(6), Sullivan R(47), Roitberg F(56)(57)(58), Ilbawi AM(57), Soerjomataram I(40), Canfell K(1).
Author information:
(1)The Daffodil Centre, The University of Sydney, a joint venture with Cancer Council NSW, Sydney, Australia.
(2)Leeds Institute of Health Sciences, University of Leeds, Leeds, UK.
(3)School of Medicine and Dentistry, Griffith University, Gold Coast, Australia.
(4)Department of Anaesthesia/Pain & Palliative Care Unit, College of Medicine, University of Nigeria, Ituku-Ozalla Campus, Enugu, Nigeria.
(5)IVAN Research Institute, Enugu, Nigeria.
(6)Translational Oncology and Urology Research (TOUR), Centre for Cancer, Society, and Public Health, School of Cancer and Pharmaceutical Sciences, King's College London, London, UK.
(7)Cancer Screening Unit, Institut Català d'Oncologia (ICO), Early Detection of Cancer Group, Epidemiology, Public Health, Cancer Prevention and Palliative Care Program, Institut d'Investigació Biomèdica de Bellvitge (IDIBELL), Barcelona, Spain.
(8)Ciber Salud Pública (CIBERESP), Instituto Salud Carlos III, Madrid, Spain.
(9)Early Detection, Prevention and Infections Branch, International Agency for Research on Cancer, Lyon, France.
(10)Makerere University College of Health Sciences, Kampala, Uganda.
(11)Oncologic Network, Prevention and Research Institute (ISPRO), Florence, Italy.
(12)Iberoamerican Cochrane Centre, IIB Sant Pau-Servei d'Epidemiologia Clínica i Salut Pública, Hospital de la Santa Creu i Sant Pau, Barcelona, Spain.
(13)Department of Respiratory Medicine, Nottingham University Hospitals NHS Trust, Nottingham, UK.
(14)Department of Surgery, Ahmadu Bello University, Zaria, Nigeria.
(15)School of Population Health, RCSI University of Medicine and Health

Sciences, Dublin, Ireland.
(16)Department of Radiation Oncology, National University Cancer Institute, National University Hospital, Singapore.
(17)Department of Clinical Oncology, Tuen Mun Hospital, New Territories West Cluster, Hospital Authority, Hong Kong, China.
(18)Division of Cancer Epidemiology and Genetics, National Cancer Institute, Rockville, Maryland, USA.
(19)Department of Academic Primary Care, Institute of Applied Health Sciences, University of Aberdeen, Aberdeen, Scotland.
(20)Center for Environmental and Health Sciences, Hokkaido University, Sapporo, Japan.
(21)Cancer Screening Unit, Cancer Prevention and Control Program, Catalan Institute of Oncology, Hospitalet de Llobregat, Barcelona, Spain.
(22)Early Detection of Cancer Research Group, EPIBELL Programme, Bellvitge Biomedical Research Institute, Hospitalet de Llobregat, Barcelona, Spain.
(23)Centre for Public Health, Queen's University Belfast, Institute of Clinical Sciences Block B, Royal Victoria Hospital, Belfast, Northern Ireland.
(24)Department of Preventive Medicine, School of Public Health, Addis Ababa University, Addis Ababa, Ethiopia.
(25)Institute for Medical Epidemiology, Biometrics and Informatics, Martin Luther University of Halle-Wittenberg, Halle, Germany.
(26)NCD Working Group, School of Public Health, Addis Ababa University, Addis Ababa, Ethiopia.
(27)Chennai Antiviral Research and Treatment Center and Clinical Research Site (CART CRS), Infectious Diseases Medical Center, Voluntary Health Services, Chennai, India.
(28)University Abderrahmane Mira of Bejaia, School of Medicine, Bejaia, Algeria.
(29)Department of Epidemiology and Preventive Medicine, University Hospital of Bejaia, Bejaia, Algeria.
(30)Department of Cancer Epidemiology and Clinical Trials, Uganda Cancer Institute, Kampala, Uganda.
(31)Department of Public Health, Faculty of Medicine, Ege University, Bornova, Turkey.
(32)Department of Oncology, Queen's University, Kingston, Ontario, Canada.
(33)Institute of Health Policy, Management and Evaluation, University of Toronto, Toronto, Ontario, Canada.
(34)Department of Anatomy, All India Institute of Medical Sciences-Patna, Patna, India.
(35)Department of Obstetrics and Gynecology, University of Campinas (UNICAMP), Campinas, Brazil.
(36)Cancer and Public Health Research Unit, Biomedical Research Foundation FISABIO, Valencia, Spain.
(37)Department of Health Metrics Sciences, Division of Hematology/Oncology, University of Washington, Seattle, Washington, USA.
(38)Department of Pediatrics, Division of Hematology/Oncology, University of Washington, Seattle, Washington, USA.
(39)School of Public Health, University of Sydney, Sydney, New South Wales,

Australia.
(40)Cancer Surveillance Branch, International Agency for Research on Cancer, Lyon, France.
(41)Department of Public Health, Erasmus MC, University Medical Center Rotterdam, Rotterdam, The Netherlands.
(42)Shaukat Khanum Memorial Cancer Hospital & Research Centre, Lahore & Peshawar, Lahore, Pakistan.
(43)Department of Health Services Research and Policy, School of Hygiene and Tropical Medicine, King's College London, London, UK.
(44)Department of Oncology, Guy's & St Thomas NHS Trust, London, UK.
(45)Centro Javeriano De Oncologia - Hospital Universitario San Ignacio, Bogotá, Colombia.
(46)Pontificia Universidad Javeriana, Bogotá, Colombia.
(47)Institute of Cancer Policy, King's College London, London, UK.
(48)Research Oncology, Bermondsey Wing, Guy's Hospital, London, UK.
(49)Department of Cancer Control Research, BC Cancer Research Institute, Vancouver, British Columbia, Canada.
(50)Odette Cancer Centre, Sunnybrook Health Sciences Centre, Toronto, Ontario, Canada.
(51)Canadian Centre for Applied Research in Cancer Control, Vancouver, British Columbia, Canada.
(52)Faculty of Health Sciences, Simon Fraser University, Burnaby, British Columbia, Canada.
(53)Division of Cancer Care and Epidemiology, Cancer Research Institute at Queen's University, Kingston, Ontario, Canada.
(54)Department of Oncology and Department of Public Health Sciences, Queen's University, Kingston, Ontario, Canada.
(55)Center for Global Health, National Cancer Institute, Rockville, Maryland, USA.
(56)Department of Non-Communicable Diseases, World Health Organisation, Geneva, Switzerland.
(57)Hospital Sírio Libanês, São Paulo, Brazil.
(58)Rede Ebserh, Rede Brasileira de Serviços Hospitalares, Brasília, Brazil.
While previous reviews found a positive association between pre-existing cancer diagnosis and COVID-19-related death, most early studies did not distinguish long-term cancer survivors from those recently diagnosed/treated, nor adjust for important confounders including age. We aimed to consolidate higher-quality evidence on risk of COVID-19-related death for people with recent/active cancer (compared to people without) in the pre-COVID-19-vaccination period. We searched the WHO COVID-19 Global Research Database (20 December 2021), and Medline and Embase (10 May 2023). We included studies adjusting for age and sex, and providing details of cancer status. Risk-of-bias assessment was based on the Newcastle-Ottawa Scale. Pooled adjusted odds or risk ratios (aORs, aRRs) or hazard ratios (aHRs) and 95% confidence intervals (95% CIs) were calculated using generic inverse-variance random-effects models. Random-effects meta-regressions were used to assess associations between effect estimates and time since cancer diagnosis/treatment. Of 23 773 unique title/abstract records,

39 studies were eligible for inclusion (2 low, 17 moderate, 20 high risk of bias). Risk of COVID-19-related death was higher for people with active or recently diagnosed/treated cancer (general population: aOR = 1.48, 95% CI: 1.36-1.61, I2 = 0; people with COVID-19: aOR = 1.58, 95% CI: 1.41-1.77, I2 = 0.58; inpatients with COVID-19: aOR = 1.66, 95% CI: 1.34-2.06, I2 = 0.98). Risks were more elevated for lung (general population: aOR = 3.4, 95% CI: 2.4-4.7) and hematological cancers (general population: aOR = 2.13, 95% CI: 1.68-2.68, I2 = 0.43), and for metastatic cancers. Meta-regression suggested risk of COVID-19-related death decreased with time since diagnosis/treatment, for example, for any/solid cancers, fitted aOR = 1.55 (95% CI: 1.37-1.75) at 1 year and aOR = 0.98 (95% CI: 0.80-1.20) at 5 years post-cancer diagnosis/treatment. In conclusion, before COVID-19-vaccination, risk of COVID-19-related death was higher for people with recent cancer, with risk depending on cancer type and time since diagnosis/treatment.

21. Health Sci Rep. 2023 Dec 8;6(12):e1748. doi: 10.1002/hsr2.1748. eCollection 2023
Hepatitis B immunization strategies and its associated factors: A narrative review.
Obeagu EI(1).
Author information:
(1)Department of Medical Laboratory Science Kampala International University Kampala Uganda.
BACKGROUND: Hepatitis B is a major public health concern as one third of the world's population is infected with hepatitis B virus (HBV). The risk of HBV infection remains primarily through percutaneous or mucosal contact with infected blood or body fluids.
AIM: To update the society on hepatitis B immunization strategies and its associated factors.
MATERIALS AND METHODS: The review paper utilized different search engines such as Pubmed Central, Scopus, Web of Science, Google Scholar, and so on to conduct this review paper.
RESULTS: Improving immunity through general vaccination recommendations has reduced the number of chronic hepatitis B cases among children and adolescents, but an influx of infections from endemic countries has increased the number of chronically infected adults. This is due to immigration from endemic countries to Hepatitis less burden areas like developed countries. Introduction of infant immunization programs in some countries has reportedly reduced the prevalence of HBV infection and reduced the incidence of liver cancer in children and young adults.
CONCLUSION: While hepatitis B vaccination coverage is low, a significant number of people are not adhering to full hepatitis B vaccination posing a potential hazard to health.

22. J Infect Dis. 2023 Dec 8:jiad555. doi: 10.1093/infdis/jiad555.
Long-term Sudan virus Ebola survivors maintain multiple antiviral defense mechanisms.
Sobarzo A(1)(2), Mone Y(3), Lang S(3), Gelkop S(1), Brangel P(4), Kuehne AI(5),

McKendry RA(4), Mell JC(3), Ahmed A(3), Davis C(1), Dye JM(5), Lutwama JJ(6), Lobel L(1), Veas F(7)(8), Ehrlich GD(3).
Author information:
(1)The Shraga Segal Department of Microbiology, Immunology, and Genetics, Faculty of Health Sciences, Ben-Gurion University of the Negev, Beer-Sheva 8410501, Israel.
(2)The Pre-Clinical Research Center, Faculty of Health Sciences, Ben-Gurion University of the Negev, Beer-Sheva 8410501, Israel.
(3)Department of Microbiology & Immunology, Center for Genomic Sciences and Center for Advanced Microbial Processing, Institute for Molecular Medicine and Infectious Disease, Drexel University College of Medicine, 245 N 15th Street, Philadelphia, PA 19102, USA.
(4)London Centre for Nanotechnology and Division of Medicine, University College London, London WC1E 6B, UK.
(5)U.S. Army Medical Research Institute of Infectious Diseases, 1425 Porter St, Fort Detrick, MD 21702-5011, USA.
(6)Department of Arbovirology, Emerging and Re-emerging Infection, Uganda Virus Research Institute, Entebbe P.O Box 49, Uganda.
(7)Molecular Comparative Immuno-Physiopathology Lab, French Institute of Research for Development (IRD) Health Branch of UMR5151/University of Montpellier & UMR Research Unit-Ministry of Defense, Faculty of Pharmacy, 34093 Montpellier, France.
(8)Copernicus Integrated Solutions for Biosafety Risks (CISBR), Faculty of Pharmacy, Montpellier University, 34093 Montpellier, France.
BACKGROUND: The critical issues of sustained memory immunity following ebolavirus disease among long-term survivors (EVD) are still unclear.
METHODS: Here, we examine virus-specific immune and inflammatory responses in 12 Sudan virus (SUDV) long-term survivors from Uganda's 2000-1 Gulu outbreak, 15 years after recovery following in vitro challenge. Total RNA from isolated SUDV-stimulated and unstimulated PBMCs was extracted and analyzed. Matched serum samples were also collected to determine SUDV IgG levels and functionality.
RESULTS: We detected persistent humoral (58%, 7 of 12) and cellular (33%, 4 of 12) immune responses in SUDV long-term survivors and identified critical molecular mechanisms of innate and adaptive immunity. Gene expression in immune pathways, the IFN signaling system, antiviral defense response, and activation and regulation of T- and B-cell responses were observed. SUDV long-term survivors also maintained robust virus-specific IgG antibodies capable of polyfunctional responses, including neutralizing and innate Fc effector functions.
CONCLUSIONS: Data integration identified significant correlations among humoral and cellular immune responses and pinpointed a specific innate and adaptive gene expression signature associated with long-lasting immunity. This could help identify natural and vaccine correlates of protection against ebolavirus disease.

23. Vaccine. 2023 Dec 7;41(50):7573-7580. doi: 10.1016/j.vaccine.2023.10.055.
Safety and immunogenicity of an Ad26.ZEBOV booster vaccine in Human

Immunodeficiency Virus positive (HIV+) adults previously vaccinated with the Ad26.ZEBOV, MVA-BN-Filo vaccine regimen against Ebola: A single-arm, open-label Phase II clinical trial in Kenya and Uganda.

Man-Lik Choi E(1), Abu-Baker Mustapher G(2), Omosa-Manyonyi G(3), Foster J(4), Anywaine Z(5), Musila Mutua M(6), Ayieko P(7), Vudriko T(8), Ann Mwangi I(9), Njie Y(10), Ayoub K(11), Mundia Muriuki M(12), Kasonia K(13), Edward Connor N(14), Florence N(15), Manno D(16), Katwere M(17), McLean C(18), Gaddah A(19), Luhn K(20), Lowe B(21), Greenwood B(22), Robinson C(23), Anzala O(24), Kaleebu P(25), Watson-Jones D(26); EBL2010 Study Team.
Author information:
(1)London School of Hygiene & Tropical Medicine, London, United Kingdom. Electronic address: edward.choi@lshtm.ac.uk.
(2)MRC/UVRI & LSHTM Uganda Research Unit, Entebbe, Uganda. Electronic address: Ggayi.Abu-baker@mrcuganda.org.
(3)KAVI - Institute of Clinical Research, University of Nairobi, Nairobi, Kenya. Electronic address: GOmosa@kaviuon.org.
(4)London School of Hygiene & Tropical Medicine, London, United Kingdom.
(5)MRC/UVRI & LSHTM Uganda Research Unit, Entebbe, Uganda. Electronic address: Zacchaeus.Anywaine@mrcuganda.org.
(6)KAVI - Institute of Clinical Research, University of Nairobi, Nairobi, Kenya. Electronic address: MMutua@kaviuon.org.
(7)London School of Hygiene & Tropical Medicine, London, United Kingdom. Electronic address: Philip.Ayieko@lshtm.ac.uk.
(8)MRC/UVRI & LSHTM Uganda Research Unit, Entebbe, Uganda. Electronic address: tobias.vudriko@yahoo.com.au.
(9)KAVI - Institute of Clinical Research, University of Nairobi, Nairobi, Kenya. Electronic address: IMwangi@kaviuon.org.
(10)London School of Hygiene & Tropical Medicine, London, United Kingdom. Electronic address: Yusupha.Njie@lshtm.ac.uk.
(11)MRC/UVRI & LSHTM Uganda Research Unit, Entebbe, Uganda. Electronic address: Ayoub.Kakande@mrcuganda.org.
(12)KAVI - Institute of Clinical Research, University of Nairobi, Nairobi, Kenya. Electronic address: MMuriuki@kaviuon.org.
(13)London School of Hygiene & Tropical Medicine, London, United Kingdom. Electronic address: Kambale.Kasonia@lshtm.ac.uk.
(14)London School of Hygiene & Tropical Medicine, London, United Kingdom. Electronic address: Nicholas.Connor@lshtm.ac.uk.
(15)London School of Hygiene & Tropical Medicine, London, United Kingdom; MRC/UVRI & LSHTM Uganda Research Unit, Entebbe, Uganda; Uganda Virus Research Institute, Entebbe, Uganda. Electronic address: Florence.Nambaziira@mrcuganda.org.
(16)London School of Hygiene & Tropical Medicine, London, United Kingdom. Electronic address: daniela.manno@lshtm.ac.uk.
(17)Janssen Vaccines and Prevention, Leiden, The Netherlands. Electronic address: mkatwere@its.jnj.com.
(18)Janssen Vaccines and Prevention, Leiden, The Netherlands. Electronic address: CMcLean@ITS.JNJ.com.

(19)Janssen Research and Development, Beerse, Belgium. Electronic address: agaddah@its.jnj.com.
(20)Janssen Vaccines and Prevention, Leiden, The Netherlands. Electronic address: kluhn@its.jnj.com.
(21)London School of Hygiene & Tropical Medicine, London, United Kingdom. Electronic address: Brett.Lowe@lshtm.ac.uk.
(22)London School of Hygiene & Tropical Medicine, London, United Kingdom. Electronic address: Brian.Greenwood@lshtm.ac.uk.
(23)Janssen Vaccines and Prevention, Leiden, The Netherlands. Electronic address: CRobin29@ITS.JNJ.com.
(24)KAVI - Institute of Clinical Research, University of Nairobi, Nairobi, Kenya. Electronic address: OAnzala@kaviuon.org.
(25)London School of Hygiene & Tropical Medicine, London, United Kingdom; MRC/UVRI & LSHTM Uganda Research Unit, Entebbe, Uganda; Uganda Virus Research Institute, Entebbe, Uganda. Electronic address: pontiano.kaleebu@mrcuganda.org.
(26)London School of Hygiene & Tropical Medicine, London, United Kingdom; Mwanza Intervention Trials Unit, National Institute for Medical Research, Mwanza, Tanzania. Electronic address: Deborah.Watson-Jones@lshtm.ac.uk.
BACKGROUND: People living with HIV constitute an important part of the population in regions at risk of Ebola virus disease outbreaks. The two-dose Ad26.ZEBOV, MVA-BN-Filo Ebola vaccine regimen induces strong immune responses in HIV-positive (HIV+) adults but the durability of this response is unknown. It is also unclear whether this regimen can establish immune memory to enable an anamnestic response upon re-exposure to antigen.
METHODS: This paper describes an open-label, phase 2 trial, conducted in Kenya and Uganda, of Ad26.ZEBOV booster vaccination in HIV+ participants who had previously received the Ad26.ZEBOV, MVA-BN-Filo primary regimen. HIV+ adults with well-controlled infection and on highly active antiretroviral therapy were enrolled, vaccinated with booster, and followed for 28 days. The primary objectives were to assess Ad26.ZEBOV booster safety and antibody responses against the Ebola virus glycoprotein using the Filovirus Animal Non-Clinical Group ELISA.
RESULTS: The Ad26.ZEBOV booster was well-tolerated in HIV+ adults with mostly mild to moderate symptoms. No major safety concerns or serious adverse events were reported. Four and a half years after the primary regimen, 24/26 (92 %) participants were still classified as responders, with a pre-booster antibody geometric mean concentration (GMC) of 726 ELISA units (EU)/mL (95 %CI 447-1179). Seven days after the booster, the GMC increased 54-fold to 38,965 EU/mL (95 %CI 23532-64522). Twenty-one days after the booster, the GMC increased 176-fold to 127,959 EU/mL (95 %CI 93872-174422). The responder rate at both post-booster time points was 100 %.
CONCLUSIONS: The Ad26.ZEBOV booster is safe and highly immunogenic in HIV+ adults with well-controlled infection. The Ad26.ZEBOV, MVA-BN-Filo regimen can generate long-term immune memory persisting for at least 4·5 years, resulting in a robust anamnestic response.
TRIAL REGISTRATION: Pan African Clinical Trial Registry (PACTR202102747294430).
CLINICALTRIALS: gov (NCT05064956).

24. Front Immunol. 2023 Dec 6;14:1264351. doi: 10.3389/fimmu.2023.1264351.
Feasibility and acceptability of undertaking postmortem studies for tuberculosis medical research in a low income country.

Ahimbisibwe G(1), Nakibuule M(1), Martin Ssejoba M(1), Oyamo D(2), Mulwana R(2), Nabulime J(2), Babirye F(2), Kizito MA(3), Lekuya HM(4), Adakun AS(2), Nalumansi D(2), Muryasingura S(2), Lukande R(3), Kyazze A(5), Baluku JB(5), Biraro IA(5)(6), Cose S(1)(7).
Author information:
(1)Medical Research Council (MRC)/Uganda Virus Research Institute (UVRI) and London School of Hygiene and Tropical Medicine (LSHTM) Uganda Research Unit, Entebbe, Uganda.
(2)Mulago National Referral Hospital, Kampala, Uganda.
(3)Department of Pathology, Makerere University, Kampala, Uganda.
(4)Department of Surgery, Makerere University, Kampala, Uganda.
(5)Division of Pulmonology, Kiruddu National Referral Hospital, Kampala, Uganda.
(6)Department of Internal Medicine, Makerere University, Kampala, Uganda.
(7)Clinical Research Department, London School of Hygiene and Tropical Medicine, London, United Kingdom.

INTRODUCTION: If we are to break new ground in difficult-to-treat or difficult-to-vaccinate diseases (such as HIV, malaria, or tuberculosis), we must have a better understanding of the immune system at the site of infection in humans. For tuberculosis (TB), the initial site of infection is the lungs, but obtaining lung tissues from subjects suffering from TB has been limited to bronchoalveolar lavage (BAL) or sputum sampling, or surgical resection of diseased lung tissue.
METHODS: We examined the feasibility of undertaking a postmortem study for human tuberculosis research at Mulago National Referral Hospital in Kampala, Uganda.
RESULTS: Postmortem studies give us an opportunity to compare TB-involved and -uninvolved sites, for both diseased and non-diseased individuals. We report good acceptability of the next-of-kin to consent for their relative's tissue to be used for medical research; that postmortem and tissue processing can be undertaken within 8 hours following death; and that immune cells remain viable and functional up to 14 hours after death.
DISCUSSION: Postmortem procedures remain a valuable and essential tool both to establish cause of death, and to advance our medical and scientific understanding of infectious diseases.

25. Wellcome Open Res. 2023 Dec 6;8:120. doi: 10.12688/wellcomeopenres.18915.2. eCollection 2023.
Towards achieving transnational research partnership equity: lessons from implementing adaptive platform trials in low- and middle-income countries.

Modlin C(1)(2), Sugarman J(1)(3)(4), Chongwe G(5), Kass N(1)(4), Nazziwa W(6), Tegli J(7), Shrestha P(8), Ali J(1)(8).
Author information:
(1)Berman Institute for Bioethics, Johns Hopkins University, Baltimore, MD, 21205, USA.

(2)Division of Infectious Diseases, Johns Hopkins Medicine, Baltimore, Maryland, 21205, USA.
(3)Division of General Internal Medicine, Johns Hopkins Medicine, Baltimore, Maryland, 21205, USA.
(4)Department of Health Policy and Management, Johns Hopkins Bloomberg School of Public Health, Baltimore, Maryland, 21205, USA.
(5)School of Public Health, University of Zambia, Lusaka, Zambia.
(6)Uganda National Council for Science and Technology, Kampala, Uganda.
(7)Family Health International Clinical/Partnership for Research on Vaccines and Infectious Diseases in Liberia, Monrovia, Liberia.
(8)Department of International Health, Johns Hopkins Bloomberg School of Public Health, Baltimore, Maryland, 21205, USA.
BACKGROUND: Use of adaptive clinical trials, particularly adaptive platform trials, has grown exponentially in response to the coronavirus disease (COVID-19) pandemic. Implementation of these trials in low- and middle-income countries (LMICs) has been fostered through the formation or modification of transnational research partnerships, typically between research groups from LMICs and high-income countries (HICs). While these partnerships are important to promote collaboration and overcome the structural and economic disadvantages faced by LMIC health researchers, it is critical to focus attention on the multiple dimensions of partnership equity.
METHODS: Based on informal literature reviews and a meeting with leaders of one of the multinational COVID-19 adaptive platform trials, we describe some important considerations about research partnership equity in this context.
RESULTS: We organize these considerations into eight thematic categories: 1) epistemic structures, 2) funding, 3) ethics oversight, 4) regulatory oversight, 5) leadership, 6) post-trial access to interventions, data, and specimens, 7) knowledge translation and dissemination, and 8) research capacity strengthening and maintenance. Within each category we review normative claims that support its relevance to research partnership equity followed by discussion of how adaptive platform trials highlight new dimensions, considerations, or challenges.
CONCLUSION: In aggregate, these observations provide insight into procedural and substantive equity-building measures within transnational global health research partnerships more broadly.

26. Am J Trop Med Hyg. 2023 Nov 6;109(6):1363-1367. doi: 10.4269/ajtmh.23-0464.
COVID Perceptions among Pregnant Women Living in a Malaria Hyperendemic Rural Region in Uganda: A Cross-Sectional Study.
Segala FV(1), Patti G(1), Olal L(2), De Vita E(1), Olung N(3), Papagni R(1), Amone J(3), Totaro V(1), Onapa E(3), Novara R(1), Ngole B(2), L'Episcopia M(4), Okori S(3), Dall'Oglio G(5), Ictho J(5), Severini C(4), Putoto G(6), Lochoro P(5), Di Gennaro F(1), Saracino A(1).
Author information:
(1)Clinic of Infectious Diseases, Department of Precision and Regenerative Medicine and Ionian Area, University of Bari "Aldo Moro," Bari, Italy.
(2)African Network for Change, Kampala, Uganda.

(3)St. John's XXIII Hospital Aber, Jaber, Uganda.
(4)Department of Infectious Diseases, Istituto Superiore di Sanità, Rome, Italy.
(5)Doctors with Africa, CUAMM, Kampala, Uganda.
(6)Operational Research Unit, Doctors with Africa CUAMM, Padua, Italy.
Both SARS-CoV2 and Plasmodium falciparum infection during pregnancy increases the risk for adverse maternal and fetal outcomes, including abortion, severe disease, and death. Indeed, although malaria and COVID-19 show an overlapping clinical presentation, they require a profoundly different approach. The aim of this study was to explore COVID-19 awareness among pregnant women living in a P. falciparum hyperendemic region in rural Uganda. This cross-sectional, prospective study was conducted in one Hospital and two Health Centers (HC) in Lango region, Uganda, from July 14, 2022, to March 14, 2023. Data about demographics, COVID-19 history, and COVID-19 and malaria perceptions were collected using RedCap mobile app platform. Study endpoint was a context-specific COVID-19 awareness score, accounting for the most common disease misconceptions. Association between study variables and good COVID-19 awareness was assessed by χ^2 and t test, as appropriate, and variables found to be statistically significant were further explored in multivariate logistic regression analysis. A total of 888 pregnant women were recruited. Median age was 24 (interquartile range: 20-29) years, whereas 79% (n = 704) attained only primary education and 66.6% (n = 591) were used in agriculture. SARS-CoV2 vaccination rate was 92%. In multivariate analysis (Table 3), variables associated with high COVID knowledge were presenting at antenatal care visit in Atipe HC (adjusted odds ratio [aOR]: 8.1, 95% CI: 4.1-16.48) having a previous good knowledge about malaria (aOR: 1.76, 95% CI: 1.21-2.56). Among pregnant women living in rural Uganda, COVID-19 awareness relies on the overall educational level, malaria knowledge and reference HC. Among pregnant women living in P. falciparum endemic areas, community-level malaria awareness might guide educational interventions during future pandemics.

27. PLoS One. 2023 Dec 5;18(12):e0295325. doi: 10.1371/journal.pone.0295325.
Protocol for the development of a core outcome set for neonatal sepsis (NESCOS).
Taneri PE(1)(2), Kirkham JJ(3), Molloy EJ(4)(5), Biesty L(2)(6), Polin RA(7), Wynn JL(8), Stoll BJ(9)(10)(11), Kissoon N(12), Kawaza K(13), Daly M(14), Branagan A(4)(5), Bonnard LN(15), Giannoni E(16), Strunk T(17), Ohaja M(2), Mugabe K(18), Suguitani D(19), Quirke F(1)(20), Devane D(1)(2)(6).
Author information:
(1)HRB-Trials Methodology Research Network, University of Galway, Galway, Ireland.
(2)School of Nursing and Midwifery, University of Galway, Galway, Ireland.
(3)Centre for Biostatistics, Manchester Academic Health Science Centre, The University of Manchester, Manchester, United Kingdom.
(4)Department of Neonatology, The Coombe Hospital, Dublin, Ireland.
(5)Department of Paediatrics and Child Health, Trinity College Dublin, Trinity Research in Childhood Centre (TRiCC), Neonatology, Children's Health Ireland, Dublin, Ireland.
(6)Evidence Synthesis Ireland & Cochrane Ireland, University of Galway, Galway,

Ireland.
(7)Department of Paediatrics, College of Physicians and Surgeons, Columbia University, New York, New York, United States of America.
(8)Department of Paediatrics, College of Medicine, University of Florida, Gainesville, Florida, United States of America.
(9)China Medical Board, China.
(10)Emory University School of Medicine, Atlanta, GA, United States of America.
(11)McGovern Medical School of the University of Texas Health Science Center at Houston, Houston, Texas, United States of America.
(12)Department of Paediatrics, College of Medicine, University of British Columbia, Vancouver, Canada.
(13)Department of Paediatrics and Child Health, Kamuzu University of Health Sciences, Blantyre, Malawi.
(14)Education and Research, Irish Neonatal Health Alliance, Bray, Ireland.
(15)Patient Advocacy, Melletted a helyem Egyesület, Budapest, Hungary.
(16)Clinic of Neonatology, Department Mother-Woman-Child, Lausanne University Hospital and University of Lausanne, Lausanne, Switzerland.
(17)Neonatal Directorate, Child and Adolescent Health Service, Wesfarmers' Centre for Vaccines and Infectious Diseases, Telethon Kids Institute, University of Western Australia, Perth, Australia.
(18)Mbale Regional Referral Hospital, Busitema University Faculty of Health Sciences, Mbale, Uganda.
(19)Brazilian Parents of Preemies' Association, Brazil.
(20)School of Medicine, University of Limerick, Limerick, Ireland.
Neonatal sepsis is a serious public health problem; however, there is substantial heterogeneity in the outcomes measured and reported in research evaluating the effectiveness of the treatments. Therefore, we aim to develop a Core Outcome Set (COS) for studies evaluating the effectiveness of treatments for neonatal sepsis. Since a systematic review of key outcomes from randomised trials of therapeutic interventions in neonatal sepsis was published recently, we will complement this with a qualitative systematic review of the key outcomes of neonatal sepsis identified by parents, other family members, parent representatives, healthcare providers, policymakers, and researchers. We will interpret the outcomes of both studies using a previously established framework. Stakeholders across three different groups i.e., (1) researchers, (2) healthcare providers, and (3) patients' parents/family members and parent representatives will rate the importance of the outcomes in an online Real-Time Delphi Survey. Afterwards, consensus meetings will be held to agree on the final COS through online discussions with key stakeholders. This COS is expected to minimize outcome heterogeneity in measurements and publications, improve comparability and synthesis, and decrease research waste.

28. Medicine (Baltimore). 2023 Dec 1;102(48):e36462. doi: 10.1097/MD.0000000000036462.
Nosocomial infections in sickle cell anemia patients: Prevention through multi-disciplinary approach: A review.
Obeagu EI(1), Obeagu GU(2), Akinleye CA(3), Igwe MC(4).

Author information:
(1)Department of Medical Laboratory Science, Kampala International University, Uganda.
(2)School of Nursing Science, Kampala International University, Uganda.
(3)Department of Community Medicine, Osun State University, Osogbo, Nigeria.
(4)Department of Public Health, Kampala International University, Uganda.
Sickle cell anemia (SCA), a hereditary blood disorder characterized by the presence of abnormal hemoglobin, poses a unique set of challenges for both patients and healthcare providers. One of the most pressing issues in the care of these individuals is the persistent threat of nosocomial infections, which are infections acquired during hospitalization. This abstract provides a concise overview of the ongoing challenge of nosocomial infections in SCA patients, highlighting the factors contributing to their vulnerability and the preventive measures in place. SCA patients face increased susceptibility to nosocomial infections due to their compromised immune systems, frequent hospitalizations, prolonged stays, and the need for invasive medical interventions. The emergence of multidrug-resistant pathogens further complicates the management of these infections. To address this challenge, healthcare facilities have implemented infection control protocols, vaccination strategies, and antimicrobial stewardship, emphasizing the importance of patient education. Recognizing the gravity of this issue and adopting comprehensive preventive measures is crucial to improving the quality of care and patient outcomes in this vulnerable population. Further research and ongoing efforts are essential to reducing the burden of nosocomial infections in SCA patients and enhancing their overall healthcare experience.

29. One Health. 2023 Sep 14;17:100627. doi: 10.1016/j.onehlt.2023.100627.
How farm practices and antibiotic use drive disease incidence in smallholder livestock farms: Evidence from a survey in Uganda.
Emes E(1), Wieland B(2)(3), Magnusson U(4), Dione M(5).
Author information:
(1)Centre for the Mathematical Modelling of Infectious Diseases, The London School of Hygiene and Tropical Medicine, London WC1E 7HT, UK.
(2)Institute of Virology and Immunology, Mittelhäusern, Switzerland.
(3)Department of Infectious Diseases and Pathobiology, Vetsuisse Faculty, University of Bern, Switzerland.
(4)Department of Clinical Sciences, Swedish University of Agricultural Sciences (SLU), Uppsala, Sweden.
(5)International Livestock Research Institute, Rue 18 Cité Mamelles, BP 24265, Ouakam, Dakar, Senegal.
BACKGROUND: Antimicrobial resistance (AMR) is a growing threat to human and animal health, and the growth in AMR prevalence globally is thought to be partially driven by non-therapeutic antibiotic use in livestock production. However, livestock farms may depend on antibiotics as a prophylactic disease management tool, and reducing antibiotic use in isolation may harm farmers' economic security. In order to help farmers safely reduce their antibiotic use, we must first determine how necessary non-therapeutic antibiotic use is for

disease management, and how other farm practices can guard against disease and make antibiotic use reduction safe and feasible.
METHODS: Using the Antimicrobial Use in Livestock Production Settings (AMUSE) tool, a standardised survey tool for investigating attitudes and practices relating to antibiotic use on farms, we investigated the farming practices and animal disease outcomes of smallholder livestock farms in Uganda. We used logistic regression to investigate the effect of prophylactic antibiotic use; as well as of prophylactic vaccination, non-antimicrobial medicines, and on-farm biosecurity measures; on the likelihood of disease outbreaks.
FINDINGS: We found that prophylactic antibiotic use did indeed seem to guard against disease outbreaks, underlining the rationality of non-therapeutic antibiotic use in smallholder livestock farms and the need to pair antibiotic use reduction with other interventions in order to mitigate risk. The most effective intervention pairing varied by species, with expanded access to animal health services and the use of prophylactic vaccination demonstrating the greatest potential overall.
IMPLICATIONS: These findings echo earlier results generated using the AMUSE survey tool. They should be followed by participatory research in which farmers are consulted to explore intervention options, and subsequently by farm-level intervention trials of combined antimicrobial stewardship interventions to verify their effectiveness.

30. Public Health. 2023 Dec;225:229-236. doi: 10.1016/j.puhe.2023.09.026.
'Unable to have a proper conversation over the phone about my concerns': a multimethods evaluation of the impact of COVID-19 on routine childhood vaccination services in London, UK.
Buck E(1), Burt J(2), Karampatsas K(3), Hsia Y(4), Whyte G(5), Amirthalingam G(6), Skirrow H(7), Le Doare K(8).
Author information:
(1)St George's Hospital Medical School, St. George's, University of London, London, United Kingdom. Electronic address: eleanor.buck3@nhs.net.
(2)Ashford and St Peter's Hospital NHS Foundation Trust, United Kingdom.
(3)Centre for Neonatal and Paediatric Infection, Institute of Infection and Immunity, St. George's, University of London, London, United Kingdom.
(4)Centre for Neonatal and Paediatric Infection, Institute of Infection and Immunity, St. George's, University of London, London, United Kingdom; School of Pharmacy, Queen's University Belfast, Belfast, United Kingdom.
(5)North Croydon Medical Centre, United Kingdom.
(6)Immunisation and Vaccine Preventable Diseases Division, UK Health Security Agency, United Kingdom.
(7)School of Public Health, Imperial College London, United Kingdom.
(8)Centre for Neonatal and Paediatric Infection, Institute of Infection and Immunity, St. George's, University of London, London, United Kingdom; MRC/UVRI @LHSTM Uganda Research Unit, Entebbe, Uganda; Pathogen Immunity Group, UK Health Security Agency, Porton Down, United Kingdom.
OBJECTIVES: Investigating the completion rate of 12-month vaccinations and parental perspectives on vaccine services during COVID-19.

STUDY-DESIGN: Service evaluation including parental questionnaire.
METHODS: Uptake of 12-month vaccinations in three London general practices during three periods: pre-COVID (1/3/2018-28/2/2019, n = 826), during COVID (1/3/2019-28/2/2020, n = 775) and post-COVID first wave (1/8/2020-31/1/2021, n = 419). Questionnaire of parents whose children were registered at the practices (1/4/2019-1/22/2021, n = 1350).
RESULTS: Comparing pre-COVID and both COVID cohorts, the completion rates of 12-month vaccines were lower. Haemophilus influenzae type B/meningococcal group C (Hib/MenC) vaccination uptake was 5.6% lower (89.0% vs 83.4%, P=<0.001), meningococcal group B (MenB) booster uptake was 4.4% lower (87.3% vs 82.9%, P = 0.006), pneumococcal conjugate vaccine (PCV) booster uptake was 6% lower (88.0% vs 82.0%, P < 0.001) and measles, mumps and rubella (MMR) vaccine uptake was 5.2% lower (89.1% vs 83.9%, P = 0.003). Black/Black-British ethnicity children had increased odds of missing their 12-month vaccinations compared to White ethnicity children (adjusted odds ratio 0.43 [95% confidence interval 0.24-0.79, P = 0.005; 0.36 [0.20-0.65], P < 0.001; 0.48 [0.27-0.87], P = 0.01; 0.40 [0.22-0.73], P = 0.002; for Hib/MenC, MenB booster, PCV booster and MMR. Comparing pre-COVID and COVID periods, vaccinations coded as not booked increased for MMR (10%), MenB (7%) and PCV booster (8%). Parents reported changes to vaccination services during COVID-19, including difficulties booking and attending appointments and lack of vaccination reminders.
CONCLUSION: A sustained decrease in 12-month childhood vaccination uptake disproportionally affected Black/Black British ethnicity infants during the first wave of the pandemic. Vaccination reminders and availability of healthcare professionals to discuss parental vaccine queries are vital to maintaining uptake.

31. Prev Vet Med. 2023 Dec;221:106051. doi: 10.1016/j.prevetmed.2023.106051. Epub
Seroprevalence and risk factors of Peste des petits ruminants in different production systems in Uganda.
Nkamwesiga J(1), Lumu P(2), Nalumenya DP(3), Korennoy F(4), Roesel K(5), Wieland B(6), Perez A(7), Kiara H(5), Muhanguzi D(3).
Author information:
(1)Dahlem Research School of Biomedical Sciences, Department of Veterinary Medicine, Freie Universität Berlin, Berlin, Germany; International Livestock Research Institute, Nairobi, Kenya. Electronic address: J.Nkamwesiga@cgiar.org.
(2)Ministry of Agriculture Animal Industry and Fisheries, Entebbe, Uganda.
(3)College of Veterinary Medicine, Animal Resources, and Biosecurity, Makerere University, Kampala, Uganda.
(4)Federal Center for Animal Health (FGBI ARRIAH), Vladimir, Russia.
(5)International Livestock Research Institute, Nairobi, Kenya.
(6)Institute of Virology and Immunology (IVI), Switzerland; Department of Infectious Diseases and Pathobiology (DIP), University of Bern, Switzerland.
(7)College of Veterinary Medicine, University of Minnesota, St. Paul, MN, United States.
Peste des petits ruminants (PPR) is a highly contagious and fatal disease of mostly domestic goats and sheep. First reported in Uganda in 2007, the extent of

peste des petits ruminants virus (PPRV) exposure, geographical distribution and risk factors of its transmission and spread are not clearly understood. In this study, we used cluster random sampling methodology to select study villages from three districts representing three different production systems along Uganda's "cattle corridor". Between October and December 2022, 2520 goat and sheep serum samples were collected from 252 households with no history of PPR vaccination in the past one year. The household heads were interviewed to assess possible risk factors of PPRV transmission using a structured questionnaire. The serum samples were screened with a commercial competitive enzyme-linked immunosorbent assay (cELISA) for PPRV antibodies. The determined overall true seroprevalence of PPRV was 27.3% [95% CI: 25.4-29.1]. The seroprevalence of PPRV antibodies in different production systems was 44.1% [95% CI: 40.6-47.7], 31.7% [95% CI: 28.4-35.0] and 6.1% [95% CI: 4.4-7.9] for pastoral, agropastoral and mixed crop-livestock production systems respectively. A mixed-effects multivariable logistic regression model revealed strong statistical evidence of association between female animals and PPRV antibody seropositivity compared to males [OR= 2.45, 95% CI: 1.7-3.5, p < 0.001]. The likelihood of being PPRV antibody seropositive significantly increased with increasing small ruminant age. Animals older than 3 years were more than three times as likely to be PPRV seropositive compared to animals aged under 1 year [OR= 3.41, 95% CI: 2.39-4.85, p < 0.001]. There was no statistical evidence of association between small ruminant species and PPRV antibody seropositivity (p = 0.423). Village flocks that interacted with neighboring flocks daily during grazing (IRR = 1.59, 95% CI: 1.19-2.13) and watering around swamps (IRR = 1.59, 95% CI: 1.19-2.13) were highly correlated with increased number of PPRV seropositive animals as compared to flocks that were more restricted in grazing and watered around other water sources other than swamps. Flocks from pastoral and agropastoral production systems were more than 10 times more likely to have seropositive animals than mixed crop-livestock flocks. Targeting PPR control interventions (vaccination and livestock movement control) to pastoral and agro-pastoral small ruminant production systems that are very prone to PPR incursions is recommended to prevent PPRV spread to low-risk smallholder mixed crop-livestock production systems.

32. Lancet HIV. 2023 Dec;10(12):e779-e789. doi: 10.1016/S2352-3018(23)00227-8.
Adherence, safety, and choice of the monthly dapivirine vaginal ring or oral emtricitabine plus tenofovir disoproxil fumarate for HIV pre-exposure prophylaxis among African adolescent girls and young women: a randomised, open-label, crossover trial.
Nair G(1), Celum C(2), Szydlo D(3), Brown ER(3), Akello CA(4), Nakalega R(4), Macdonald P(5), Milan G(5), Palanee-Phillips T(6), Reddy K(7), Tahuringana E(8), Muhlanga F(8), Nakabiito C(4), Bekker LG(5), Siziba B(8), Hillier SL(9), Baeten JM(10), Garcia M(11), Johnson S(11), McClure T(11), Levy L(11), Livant E(9), Jacobson C(9), Soto-Torres L(12), van der Straten A(13), Hosek S(14), Rooney JF(15), Steytler J(16), Bunge K(9), Parikh U(17), Hendrix C(18), Anderson P(19), Ngure K(20); REACH Protocol Team.
Author information:
(1)Stellenbosch University, Centre for Medical Ethics and Law, Stellenbosch,

South Africa.
(2)Department of Global Health, Department of Medicine, and Department of Epidemiology, University of Washington, Seattle, WA, USA. Electronic address: ccelum@uw.edu.
(3)Vaccine and Infectious Disease Division, Fred Hutchinson Cancer Research Center, Seattle, WA, USA.
(4)Makerere University-Johns Hopkins University Research Collaboration, Kampala, Uganda.
(5)Desmond Tutu HIV Centre, University of Cape Town, Cape Town, South Africa.
(6)Wits Reproductive Health and HIV Institute, Faculty of Health Sciences, University of the Witwatersrand, Johannesburg, South Africa; Department of Epidemiology, University of Washington, Seattle, WA, USA.
(7)Wits Reproductive Health and HIV Institute, Faculty of Health Sciences, University of the Witwatersrand, Johannesburg, South Africa.
(8)University of Zimbabwe Clinical Trials Research Centre, Harare, Zimbabwe.
(9)Magee Womens Research Institute, University of Pittsburgh School of Medicine, Pittsburgh, PA, USA.
(10)Department of Global Health, Department of Medicine, and Department of Epidemiology, University of Washington, Seattle, WA, USA; Gilead Sciences, Foster City, CA, USA.
(11)FHI 360, Durham, NC, USA.
(12)National Institute of Allergy and Infectious Diseases, National Institutes of Health, Bethesda, MD, USA.
(13)ASTRA Consulting, Kensington, CA, USA; Center for AIDS Prevention Studies, University of California San Francisco, San Francisco, CA, USA.
(14)Department of Medicine, University of Illinois, Chicago, IL, USA.
(15)Gilead Sciences, Foster City, CA, USA.
(16)International Partnership for Microbicides, Johannesburg, South Africa.
(17)Magee Womens Research Institute, University of Pittsburgh School of Medicine, Pittsburgh, PA, USA; Department of Medicine, University of Pittsburgh, Pittsburgh, PA, USA.
(18)Department of Medicine, Johns Hopkins University, Baltimore, MD, USA.
(19)Department of Pharmacology, University of Colorado, Denver, CO, USA.
(20)Jomo Kenyatta University of Agriculture and Technology, School of Public Health, Nairobi, Kenya.
BACKGROUND: Half of new HIV acquisitions in Africa occur in adolescent girls and young women. Pre-exposure prophylaxis (PrEP) with oral tenofovir disoproxil fumarate plus emtricitabine or the monthly dapivirine vaginal ring is efficacious but has lower adherence and effectiveness among adolescent girls and young women. We aimed to assess product adherence, safety, and choice of oral PrEP compared with the dapivirine ring among African adolescent girls and young women.
METHODS: MTN-034/REACH was a randomised, open-label, phase 2a crossover trial among HIV-seronegative, non-pregnant adolescent girls and young women aged 16-21 years at four clinical research sites in South Africa, Uganda, and Zimbabwe. Participants were randomly assigned (1:1) to either the dapivirine ring or daily oral PrEP (200 mg of emtricitabine and 300 mg of tenofovir disoproxil fumarate)

for 6 months, then switched to the other product option for 6 months, followed by a third 6-month period in which participants were given a choice of oral PrEP, the dapivirine ring, or neither. Fixed block randomisation was used, stratified by site. The primary adherence endpoint was use of each product during the randomised periods, with high use defined as tenofovir-diphosphate concentrations greater than or equal to 700 fmol/punch (associated with taking an average of four or more tablets per week in the previous month) and greater than or equal to 4 mg dapivirine released from the returned ring (continuous use for 28 days in the previous month) based on residual drug concentrations. The primary safety endpoint was grade 2 or higher adverse events during each randomised period of 24 weeks of ring and oral PrEP. This trial is registered at ClinicalTrials.gov, NCT03593655.
FINDINGS: From Feb 6, 2019 to Sept 9, 2021, 396 adolescent girls and young women were screened, 247 of whom were enrolled and randomly assigned (6 months of the ring followed by 6 months of oral PrEP n=124; 6 months of oral PrEP followed by 6 months of the ring n=123). Median age was 18 years (IQR 17-19). 54 grade 2 or higher product-related adverse events were reported during oral PrEP and five during dapivirine ring use, with no product-related serious adverse events. High adherence was observed in 753 (57%) of the 1316 oral PrEP visits and 806 (57%) of the 1407 dapivirine ring visits. Four women acquired HIV during follow-up.
INTERPRETATION: Adherence was moderately high and similar between oral PrEP and the dapivirine ring with favourable safety and tolerability. Oral PrEP and the dapivirine ring are effective, safe, and well tolerated HIV prevention options for adolescent girls and young women who would benefit from a choice of PrEP formulations to meet their needs and preferences.

33. Vaccine X. 2023 Jul 25;15:100355. doi: 10.1016/j.jvacx.2023.100355.
Vaccination against SARS-CoV-2 in a rural Ugandan population.
Mugisha J(1), Mpairwe B(1), Kimono B(1), Kaleebu P(1), Newton R(1).
Author information:
(1)MRC/UVRI & LSHTM Uganda Research Unit, Entebbe, Uganda.
Working within the context of a longstanding cohort in rural southwestern Uganda (the General Population Cohort), we collect health-related data in successive survey rounds from all residents of 25 adjacent villages on a biannual basis. Between January 2022 and July 2022, 2318 adult participants in the cohort were asked about their SARS-CoV-2 vaccination status; 80% of participants had received at least one dose of vaccine and 51% had received two doses; 2% had received a third dose.

34. Lancet Infect Dis. 2023 Dec;23(12):1408-1417. doi: 10.1016/S1473-3099(23)00344-4. Epub 2023 Aug 3.
Safety, tolerability, and immunogenicity of the Ebola Sudan chimpanzee adenovirus vector vaccine (cAd3-EBO S) in healthy Ugandan adults: a phase 1, open-label, dose-escalation clinical trial.
Mwesigwa B(1), Houser KV(2), Hofstetter AR(3), Ortega-Villa AM(4), Naluyima P(1), Kiweewa F(1), Nakabuye I(1), Yamshchikov GV(3), Andrews C(3), O'Callahan M(3), Strom L(3), Schech S(5), Anne Eller L(5), Sondergaard EL(5), Scott PT(6),

Amare MF(5), Modjarrad K(6), Wamala A(1), Tindikahwa A(1), Musingye E(1), Nanyondo J(1), Gaudinski MR(3), Gordon IJ(3), Holman LA(3), Saunders JG(3), Costner PJM(3), Mendoza FH(3), Happe M(3), Morgan P(3), Plummer SH(3), Hickman SP(3), Vazquez S(3), Murray T(3), Cordon J(3), Dulan CNM(3), Hunegnaw R(3), Basappa M(3), Padilla M(3), Gajjala SR(3), Swanson PA 2nd(3), Lin BC(3), Coates EE(3), Gall JG(3), McDermott AB(3), Koup RA(3), Mascola JR(3), Ploquin A(3), Sullivan NJ(3), Kibuuka H(1), Ake JA(6), Ledgerwood JE(3); RV 508 Study Team.
Collaborators: Namugabo J, Beingana C, Mukyala MG, Wandege J, Nanteza C, Atwijuka B, Kizanye J, Nassanga G, Namuli J, Kityo H, Ssekitoleko M, Nassaka R, Mutebe H, Kabahubya M, Widge AT, O'Connell SE, Amoa-Awua O, Narpala SR, Flach B, Cox J, Beck A, Guech M, Seo E, Stein JA.
Author information:
(1)Makerere University Walter Reed Project, Kampala, Uganda.
(2)Vaccine Research Center, National Institute of Allergy and Infectious Diseases, National Institutes of Health, Bethesda, MD, USA. Electronic address: katherine.houser@nih.gov.
(3)Vaccine Research Center, National Institute of Allergy and Infectious Diseases, National Institutes of Health, Bethesda, MD, USA.
(4)Biostatistics Research Branch, Division of Clinical Research, National Institute of Allergy and Infectious Diseases, National Institutes of Health, Bethesda, MD, USA.
(5)Walter Reed Army Institute of Research, Silver Spring, MD, USA; Henry M Jackson Foundation for the Advancement of Military Medicine, Bethesda, MD, USA.
(6)Walter Reed Army Institute of Research, Silver Spring, MD, USA.
Erratum in
Lancet Infect Dis. 2023 Oct;23(10):e400.
BACKGROUND: Sudan Ebola virus can cause severe viral disease, with an average case fatality rate of 54%. A recent outbreak of Sudan Ebola virus in Uganda caused 55 deaths among 164 confirmed cases in the second half of 2022. Although vaccines and therapeutics specific for Zaire Ebola virus have been approved for use during outbreak situations, Sudan Ebola virus is an antigenically distinct virus with no approved vaccines available.
METHODS: In this phase 1, open-label, dose-escalation trial we evaluated the safety, tolerability, and immunogenicity of a monovalent chimpanzee adenovirus 3 vaccine against Sudan Ebola virus (cAd3-EBO S) at Makerere University Walter Reed Project in Kampala, Uganda. Study participants were recruited from the Kampala metropolitan area using International Review Board-approved written and electronic media explaining the trial intervention. Healthy adults without previous receipt of Ebola, Marburg, or cAd3 vectored-vaccines were enrolled to receive cAd3-EBO S at either 1 × 1010 or 1 × 1011 particle units (PU) in a single intramuscular vaccination and were followed up for 48 weeks. Primary safety and tolerability endpoints were assessed in all vaccine recipients by reactogenicity for the first 7 days, adverse events for the first 28 days, and serious adverse events throughout the study. Secondary immunogenicity endpoints included evaluation of binding antibody and T-cell responses against the Sudan Ebola virus glycoprotein, and neutralising antibody responses against the cAd3 vector at 4 weeks after vaccination. This study is registered with

ClinicalTrials.gov, NCT04041570, and is completed.
FINDINGS: 40 healthy adults were enrolled between July 22 and Oct 1, 2019, with 20 receiving 1 × 1010 PU and 20 receiving 1 × 1011 PU of cAd3-EBO S. 38 (95%) participants completed all follow-up visits. The cAd3-EBO S vaccine was well tolerated with no severe adverse events. The most common reactogenicity symptoms were pain or tenderness at the injection site (34 [85%] of 40), fatigue (29 [73%] of 40), and headache (26 [65%] of 40), and were mild to moderate in severity. Positive responses for glycoprotein-specific binding antibodies were induced by 2 weeks in 31 (78%) participants, increased to 34 (85%) participants by 4 weeks, and persisted to 48 weeks in 31 (82%) participants. Most participants developed glycoprotein-specific T-cell responses (20 [59%, 95% CI 41-75] of 34; six participants were removed from the T cell analysis after failing quality control parameters) by 4 weeks after vaccination, and neutralising titres against the cAd3 vector were also increased from baseline (90% inhibitory concentration of 47, 95% CI 30-73) to 4 weeks after vaccination (196, 125-308).
INTERPRETATION: The cAd3-EBO S vaccine was safe at both doses, rapidly inducing immune responses in most participants after a single injection. The rapid onset and durability of the vaccine-induced antibodies make this vaccine a strong candidate for emergency deployment in Sudan Ebola virus outbreaks.

35. AIDS Behav. 2023 Dec;27(12):4114-4123. doi: 10.1007/s10461-023-04125-w.
Acceptability and Use of the Dapivirine Vaginal Ring and Daily Oral Pre-exposure Prophylaxis (PrEP) During Breastfeeding in South Africa, Malawi, Zimbabwe, and Uganda.
Stoner MCD(1), Hawley I(2), Mathebula F(3), Horne E(3), Etima J(4), Kemigisha D(4), Mutero P(5), Dandadzi A(5), Seyama L(6), Fabiano Z(6), Scheckter R(7), Noguchi L(8), Owor M(4), Balkus JE(9)(10), Montgomery ET(2).
Author information:
(1)Women's Global Health Imperative, RTI International, 2150 Shattuck Avenue, Berkeley, CA, 94104, USA. mcstoner@rti.org.
(2)Women's Global Health Imperative, RTI International, 2150 Shattuck Avenue, Berkeley, CA, 94104, USA.
(3)Wits Reproductive Health and HIV Institute, Faculty of Health Sciences, University of the Witwatersrand, Johannesburg, South Africa.
(4)Makerere University-Johns Hopkins University (MU-JHU) Research Collaboration, Kampala, Uganda.
(5)Clinical Trials Research Centre, University of Zimbabwe, Harare, Zimbabwe.
(6)Johns Hopkins Project, College of Medicine, University of Malawi, Blantyre, Malawi.
(7), FHI 360, Durham, USA.
(8)Division of Reproductive, Maternal, Newborn, Child and Adolescent Health, Jhpiego/Johns Hopkins University, Washington, DC, USA.
(9)Department of Epidemiology, University of Washington School of Public Health, Seattle, USA.
(10)Vaccine and Infectious Disease Division, Fred Hutch Cancer Center, Seattle, USA.

This study examines qualitative acceptability of the dapivirine vaginal ring (DVR) and oral daily pre-exposure prophylaxis (PrEP) among breastfeeding persons participating in Microbicide Trials Network 043/B-PROTECTED, a phase 3B safety and drug detectability study of DVR and oral PrEP in breastfeeding. A subsample of 52 participants were purposively sampled to participate in an in-depth interview (IDI). Breastfeeding participants found both study products to be acceptable, and easy to use. A common motivation for product use was to protect the baby from HIV, although participants' understanding of how the study drug would work to protect their babies was often unclear. While most participants did not report experiencing side effects, fears about side effects were common as both initial worries about how the study products would affect their health and the health of their baby, and increased anxiety that health issues experienced by them, or their baby were from the products.

36. Front Immunol. 2023 Nov 30;14:1255676. doi: 10.3389/fimmu.2023.1255676.
Sustained spike-specific IgG antibodies following CoronaVac (Sinovac) vaccination in sub-Saharan Africa, but increased breakthrough infections in baseline spike-naive individuals.
Sembera J(1), Baine C(1), Ankunda V(1), Katende JS(1)(2), Oluka GK(1)(2), Akoli CH(2), Kato L(2), Odoch G(2), Ejou P(2), Opio S(2), Musenero M(3); COVID-19 Immunoprofiling Team; Kaleebu P(1)(2), Serwanga J(1)(2).
Author information:
(1)Department of Immunology, Uganda Virus Research Institute, Entebbe, Uganda.
(2)Pathogen Genomics, Phenotype, and Immunity Program, Medical Research Council, Uganda Virus Research Institute and London School of Hygiene and Tropical Medicine, Uganda Research Unit, Entebbe, Uganda.
(3)Science, Technology, and Innovation Secretariat, Office of the President, Government of Uganda, Kampala, Uganda.
INTRODUCTION: This study investigated the antibody responses to the inactivated COVID-19 vaccine, CoronaVac (Sinovac Biotech) in the African population to provide valuable insights into long-term immunity and breakthrough infections against SARS-CoV-2 in individuals with varying prior IgG seropositivity.
METHODS: Real-life cohorts were used to longitudinally track antibody levels against the SARS-CoV-2 spike and nucleoprotein in 60 participants over 12 months to examine the levels of multiple antibody isotypes (S-IgG, S-IgM, S-IgA, N-IgG, and N-IgM).
RESULTS: Throughout the 12 months, we observed consistently high and stable seropositivity rates for spike-IgG antibodies, spike-IgM antibodies showed a decline in frequencies over time, and spike-IgA levels remained moderate and stable. Vaccinated individuals previously positive for spike-IgG antibodies demonstrated strong and persistent seropositivity, while those initially negative experienced a gradual and delayed increase in seropositivity rates. The fold change analysis of S- and N- antibody responses demonstrated a consistently stable and comparable profile over time, indicating that vaccine-induced antibody responses remain constant and lack significant fluctuations beyond the initial boost. The study emphasized that individuals lacking previous IgG positivity showed reduced vaccine-induced spike-IgG antibodies and were more

susceptible to breakthrough infections, highlighting their higher vulnerability. All cases of breakthrough infections were asymptomatic, indicating the conferred protection to the vaccinated individuals.
DISCUSSION: The findings corroborated earlier studies on the effectiveness of the CoronaVac vaccine and emphasized the significance of accounting for pre-existing seropositivity in vaccine assessments. This study effectively demonstrated durable antibody responses against SARS-CoV-2 in the African population following the CoronaVac vaccination, providing crucial insights for informing vaccination strategies and safeguarding vulnerable populations. Continuous surveillance is imperative for tracking breakthrough infections and monitoring waning immunity. The insights gained offer crucial direction for public health strategies and enhance comprehension of vaccine effectiveness in sub-Saharan Africa. Further research should explore functional outcomes, cellular immune responses, and the vaccine's effectiveness against different variants to enhance our understanding and optimize vaccine strategies.

37. PLoS One. 2023 Nov 30;18(11):e0291834. doi: 10.1371/journal.pone.0291834.
Monocyte to Lymphocyte ratio is highly specific in diagnosing latent tuberculosis and declines significantly following tuberculosis preventive therapy: A cross-sectional and nested prospective observational study.
Mayito J(1), Meya DB(1), Miriam A(1), Dhikusooka F(1), Rhein J(2), Sekaggya-Wiltshire C(1).
Author information:
(1)Infectious Diseases Institute, Makerere University College of Health Sciences, Kampala, Uganda.
(2)Division of Infectious Diseases and International Medicine, Department of Medicine, University of Minnesota, Minneapolis, Minnesota, United States of America.
BACKGROUND: Interferon-gamma release assay and tuberculin skin test use is limited by costly sundries and cross-reactivity with non-tuberculous mycobacteria and Bacille Calmette-Guérin (BCG) vaccination respectively. We investigated the Monocyte to Lymphocyte ratio (MLR) as a biomarker to overcome these limitations and for use in monitoring response to tuberculosis preventive therapy (TPT).
METHODS: We conducted a cross-sectional and nested prospective observational study among asymptomatic adults living with Human Immuno-deficiency Virus (HIV) in Kampala, Uganda. Complete blood count (CBC) and QuantiFERON-TB® Gold-plus were measured at baseline and CBC repeated at three months. Multivariable logistic regression was performed to identify factors associated with a high MLR and decline in MLR.
RESULTS: We recruited 110 adults living with HIV and on antiretroviral therapy, of which 82.5% (85/110) had suppressed viral loads, 71.8% (79/110) were female, and 73.6% (81/110) had a BCG scar. The derived MLR diagnostic cut-off was 0.35, based on which the MLR sensitivity, specificity, positive predictive value, and negative predictive value were 12.8%, 91.6%, 45.5%, and 65.7% respectively. The average MLR declined from 0.212 (95% CI: 0.190-0.235) at baseline to 0.182 (95% CI: 0.166-0.198) after three months of TPT. A viral load of >50 copies/ml (aOR,

5.67 [1.12-28.60]) was associated with a high MLR while that of <50 copies/ml (aOR, 0.07 [0.007-0.832]) was associated with a decline in MLR.
CONCLUSION: MLR was highly specific in diagnosing latent TB and declined significantly following three months of TPT. Implications of a high MLR and decline in MLR after TPT need further evaluation in a larger cohort.

38. Vaccine. 2023 Nov 30;41(49):7435-7443. doi: 10.1016/j.vaccine.2023.11.008. Epub
Cost and operational context for national human papillomavirus (HPV) vaccine delivery in six low- and middle-income countries.

Mvundura M(1), Slavkovsky R(2), Debellut F(3), Naddumba T(4), Bayeh A(5), Ndiaye C(6), Anena J(4), Vodicka E(2), Diop A(6), Gamage D(7), Musanabaganwa C(8), Tatkan G(9), Driwale A(10), Zelalem M(11), Badiane O(12), Ginige S(7), Hamilton E(9), Sibomana H(8), Lakew Y(11), Uwinkindi F(8), Dhufera A(13), Ampeire I(10), Kumar S(14), Lamontagne DS(15).
Author information:
(1)PATH, Seattle, WA, USA. Electronic address: mmvundura@path.org.
(2)PATH, Seattle, WA, USA.
(3)PATH, Geneva, Switzerland.
(4)PATH, Kampala, Uganda.
(5)PATH, Addis Ababa, Ethiopia.
(6)PATH, Dakar, Senegal.
(7)Ministry of Health, Colombo, Sri Lanka.
(8)Rwanda Biomedical Center, Kigali, Rwanda.
(9)Ministry of Health, Primary Health Care, Georgetown, Guyana.
(10)Uganda National Expanded Programme on Immunization, Kampala, Uganda.
(11)Federal Ministry of Health, Addis Ababa, Ethiopia.
(12)Ministry of Health, Dakar, Senegal.
(13)Ethiopia Public Health Institute, Addis Ababa, Ethiopia.
(14)PATH, New Delhi, India.
(15)JSI Research & Training Institute, Inc., Arlington, TX, USA(1).
INTRODUCTION: There are concerns from immunization program planners about high delivery costs for human papillomavirus (HPV) vaccine. Most prior research evaluated costs of HPV vaccine delivery during demonstration projects or at introduction, showing relatively high costs, which may not reflect the costs beyond the pilot or introduction years. This study sought to understand the operational context and estimate delivery costs for HPV vaccine in six national programs, beyond their introduction years.
METHODS: Operational research and microcosting methods were used to retrospectively collect primary data on HPV vaccination program activities in Ethiopia, Guyana, Rwanda, Senegal, Sri Lanka, and Uganda. Data were collected from the national level and a sample of subnational administrative offices and health facilities. Operational data collected were tabulated as percentages and frequencies. Financial costs (monetary outlays) and economic costs (financial plus opportunity costs) were estimated, as was the cost per HPV vaccine dose delivered. Costing was done from the health system perspective and reported in 2019 United States dollars (US$).

RESULTS: Across the study countries, between 53 % and 99 % of HPV vaccination sessions were conducted in schools. Differences were observed in intensity and frequency with which program activities were conducted and resources used. Mean annual economic costs at health facilities in each country ranged from $1,207 to $3,190, while at the national level these ranged from $7,657 to $304,278. Mean annual HPV vaccine doses delivered per health facility in each country ranged from 162 to 761. Mean financial costs per dose per study country ranged from $0.27 to $3.32, while the economic cost per dose ranged from $3.09 to $17.20.
CONCLUSION: HPV vaccine delivery costs were lower than at introduction in some study countries. There were differences in the activities carried out for HPV vaccine delivery and the number of doses delivered, impacting the cost estimates.

39. Front Immunol. 2023 Nov 28;14:1295543. doi: 10.3389/fimmu.2023.1295543.
Acquisition of complement fixing antibodies targeting Plasmodium falciparum merozoites in infants and their mothers in Uganda.
Mortazavi SE(1)(2), Lugaajju A(3), Nylander M(1), Danielsson L(1)(4), Tijani MK(1)(5), Beeson JG(6)(7)(8), Persson KEM(1)(4).
Author information:
(1)Department of Laboratory Medicine, Lund University, Lund, Sweden.
(2)Department of Infectious Diseases, Skåne University Hospital, Lund, Sweden.
(3)College of Health Sciences, Makerere University, Kampala, Uganda.
(4)Clinical Chemistry and Pharmacology, Laboratory Medicine, Office for Medical Services, Region Skåne, Lund, Sweden.
(5)Cellular Parasitology Program, Cell Biology and Genetics Unit, Department of Zoology, University of Ibadan, Ibadan, Nigeria.
(6)The Burnet Institute, Melbourne, VIC, Australia.
(7)Department of Infectious Diseases, University of Melbourne, Melbourne, VIC, Australia.
(8)Central Clinical School and Department of Microbiology, Monash University, Melbourne, VIC, Australia.
BACKGROUND: Antibody-mediated complement fixation has previously been associated with protection against malaria in naturally acquired immunity. However, the process of early-life development of complement-fixing antibodies in infants, both in comparison to their respective mothers and to other immune parameters, remains less clear.
RESULTS: We measured complement-fixing antibodies in newborns and their mothers in a malaria endemic area over 5 years follow-up and found that infants' complement-fixing antibody levels were highest at birth, decreased until six months, then increased progressively until they were similar to birth at five years. Infants with high levels at birth experienced a faster decay of complement-fixing antibodies but showed similar levels to the low response group of newborns thereafter. No difference was observed in antibody levels between infant cord blood and mothers at delivery. The same result was found when categorized into high and low response groups, indicating placental transfer of antibodies. Complement-fixing antibodies were positively correlated with total schizont-specific IgG and IgM levels in mothers and infants at several time

points. At nine months, complement-fixing antibodies were negatively correlated with total B cell frequency and osteopontin concentrations in the infants, while positively correlated with atypical memory B cells and P. falciparum-positive atypical memory B cells.
CONCLUSION: This study indicates that complement-fixing antibodies against P. falciparum merozoites are produced in the mothers and placentally-transferred, and they are acquired in infants over time during the first years of life. Understanding early life immune responses is crucial for developing a functional, long lasting malaria vaccine.

40. J Infect Dis. 2023 Nov 28;228(11):1583-1591. doi: 10.1093/infdis/jiad327.
Geographic and Population Distributions of Human Immunodeficiency Virus (HIV)-1 and HIV-2 Circulating Subtypes: A Systematic Literature Review and Meta-analysis (2010-2021).
Williams A(1), Menon S(1), Crowe M(1), Agarwal N(1), Biccler J(1), Bbosa N(2), Ssemwanga D(2), Adungo F(3), Moecklinghoff C(4), Macartney M(5), Oriol-Mathieu V(6).
Author information:
(1)P95 Pharmacovigilance and Epidemiological Services, Leuven, Belgium.
(2)Medical Research Council/Uganda Virus Research Institute and London School of Hygiene and Tropical Medicine Uganda Research Unit, Entebbe.
(3)Kenya Medical Research Institute, Nairobi.
(4)Janssen-Cilag GmbH, Neuss, Germany.
(5)Janssen-Cilag Limited, High Wycombe, United Kingdom.
(6)Janssen Vaccines and Prevention, B.V., Leiden, The Netherlands.
BACKGROUND: HIV poses significant challenges for vaccine development due to its high genetic mutation and recombination rates. Understanding the distribution of HIV subtypes (clades) across regions and populations is crucial. In this study, a systematic review of the past decade was conducted to characterize HIV-1/HIV-2 subtypes.
METHODS: A comprehensive search was performed in PubMed, EMBASE, and CABI Global Health, yielding 454 studies from 91 countries.
RESULTS: Globally, circulating recombinant forms (CRFs)/unique recombinant forms (URFs) accounted for 29% of HIV-1 strains, followed by subtype C (23%) and subtype A (17%). Among studies reporting subtype breakdowns in key populations, 62% of HIV infections among men who have sex with men (MSM) and 38% among people who inject drugs (PWIDs) were CRF/URFs. Latin America and the Caribbean exhibited a 25% increase in other CRFs (excluding CRF01_AE or CRF02_AG) prevalence between 2010-2015 and 2016-2021.
CONCLUSIONS: This review underscores the global distribution of HIV subtypes, with an increasing prevalence of CRFs and a lower prevalence of subtype C. Data on HIV-2 were limited. Understanding subtype diversity is crucial for vaccine development, which need to elicit immune responses capable of targeting various subtypes. Further research is needed to enhance our knowledge and address the challenges posed by HIV subtype diversity.

41. One Health Outlook. 2023 Nov 27;5(1):16. doi: 10.1186/s42522-023-00092-3.

Rift valley fever outbreak in Sembabule District, Uganda, December 2020.

Aceng FL(1), Kayiwa J(2)(3), Elyanu P(4), Ojwang J(5), Nyakarahuka L(6), Balinandi S(6), Byakika-Tusiime J(7), Wejuli A(2), Harris JR(5), Opolot J(2).
Author information:
(1)Department of Integrated Epidemiology, Surveillance and Public Health Emergencies, Ministry of Health, Kampala, Uganda. faceng@musph.ac.ug.
(2)Department of Integrated Epidemiology, Surveillance and Public Health Emergencies, Ministry of Health, Kampala, Uganda.
(3)Uganda Public Health Emergency Operations Centre, Ministry of Health, Kampala, Uganda.
(4)Baylor College of Medicine - Children's Foundation, Kampala, Uganda.
(5)Division of Global Health Protection, Centers for Disease Control and Prevention, Kampala, Uganda.
(6)Uganda Virus Research Institute, Entebbe, Uganda.
(7)World Health Organization, Kampala, Uganda.

Erratum in
One Health Outlook. 2024 Jan 2;6(1):1.

BACKGROUND: Rift Valley Fever (RVF) is a viral zoonosis that can cause severe haemorrhagic fevers in humans and high mortality rates and abortions in livestock. On 10 December 2020, the Uganda Ministry of Health was notified of the death of a 25-year-old male who tested RVF-positive by reverse-transcription polymerase chain reaction (RT-PCR) at the Uganda Virus Research Institute. We investigated to determine the scope of the outbreak, identify exposure factors, and institute control measures.
METHODS: A suspected case was acute-onset fever (or axillary temperature > 37.5 °C) and ≥ 2 of: headache, muscle or joint pain, unexpected bleeding, and any gastroenteritis symptom in a resident of Sembabule District from 1 November to 31 December 2020. A confirmed case was the detection of RVF virus nucleic acid by RT-PCR or serum IgM antibodies detected by enzyme-linked immunosorbent assay (ELISA). A suspected animal case was livestock (cattle, sheep, goats) with any history of abortion. A confirmed animal case was the detection of anti-RVF IgM antibodies by ELISA. We took blood samples from herdsmen who worked with the index case for RVF testing and conducted interviews to understand more about exposures and clinical characteristics. We reviewed medical records and conducted an active community search to identify additional suspects. Blood samples from animals on the index case's farm and two neighbouring farms were taken for RVF testing.
RESULTS: The index case regularly drank raw cow milk. None of the seven herdsmen who worked with him nor his brother's wife had symptoms; however, a blood sample from one herdsman was positive for anti-RVF-specific IgM and IgG. Neither the index case nor the additional confirmed case-patient slaughtered or butchered any sick/dead animals nor handled abortus; however, some of the other herdsmen did report high-risk exposures to animal body fluids and drinking raw milk. Among 55 animal samples collected (2 males and 53 females), 29 (53%) were positive for anti-RVF-IgG.
CONCLUSIONS: Two human RVF cases occurred in Sembabule District during December 2020, likely caused by close interaction between infected cattle and humans. A

district-wide animal serosurvey, animal vaccination, and community education on infection prevention practices campaign could inform RVF exposures and reduce disease burden.

42. PLoS One. 2023 Nov 27;18(11):e0294845. doi: 10.1371/journal.pone.0294845.
Using microbiological data to improve the use of antibiotics for respiratory tract infections: A protocol for an individual patient data meta-analysis.
Boateng I(1), Stuart B(1)(2), Becque T(1), Barrett B(3), Bostock J(1), Bruyndonckx R(4), Carr-Knox L(2), Ciccone EJ(5), Coenen S(6)(7), Ebell M(8), Gillespie D(9), Hayward G(10), Hedin K(11)(12), Hood K(9), Lau TMM(9), Little P(1), Merenstein D(13), Mulogo E(14), Ordóñez-Mena J(10), Muir P(15), Samuel K(1), Shaikh N(16), Tonner S(10), van der Velden AW(17), Verheij T(17), Wang K(1), Hay AD(18), Francis N(1).
Author information:
(1)Primary Care Research Centre, Faculty of Medicine, University of Southampton, Southampton, United Kingdom.
(2)Centre for Evaluation and Methods, Wolfson Institute of Population Health, Queen Mary University of London, London, United Kingdom.
(3)Dept of Family Medicine, University of Wisconsin, Madison, WI, United States of America.
(4)Data Science Institute, I-BioStat, Hasselt University, Martelarenlaan, Hasselt, Belgium.
(5)Division of Infectious Diseases, University of North Carolina School of Medicine, Chapel Hill, North Carolina, United States of America.
(6)Centre for General Practice, Department of Family Medicine and Population Health, University of Antwerp, Antwerp, Belgium.
(7)Laboratory of Medical Microbiology, Vaccine & Infectious Disease Institute, University of Antwerp, Antwerp, Belgium.
(8)Department of Epidemiology and Biostatistics, College of Public Health, University of Georgia, Athens, Georgia, United States of America.
(9)Centre for Trials Research, School of Medicine, Cardiff University, Cardiff, Wales, United Kingdom.
(10)Nuffield Department of Primary Care, University of Oxford, Oxford, United Kingdom.
(11)Futurum, Region Jönköping County, Sweden.
(12)Department of Health, Medicine and Caring Sciences, Linköping University, Linköping, Sweden.
(13)Dept of Family Medicine, Georgetown University, Washington DC, United States of America.
(14)Department of Community Health, Faculty of Medicine, Mbarara University of Science and Technology, Mbarara, Uganda.
(15)UK Health Security Agency South West Regional Laboratory, Southmead Hospital, Bristol, United Kingdom.
(16)School of Medicine, University of Pittsburgh, Pittsburgh, Pennsylvania, United States of America.
(17)Julius Center for Health Sciences and Primary Care, University Medical Center Utrecht, Utrecht, the Netherlands.

(18)Centre for Academic Primary Care, Bristol Medical School: Population Health
Sciences, University of Bristol, Bristol, United Kingdom.
BACKGROUND: Resistance to antibiotics is rising and threatens future antibiotic effectiveness. 'Antibiotic targeting' ensures patients who may benefit from antibiotics receive them, while being safely withheld from those who may not. Point-of-care tests may assist with antibiotic targeting by allowing primary care clinicians to establish if symptomatic patients have a viral, bacterial, combined, or no infection. However, because organisms can be harmlessly carried, it is important to know if the presence of the virus/bacteria is related to the illness for which the patient is being assessed. One way to do this is to look for associations with more severe/prolonged symptoms and test results. Previous research to answer this question for acute respiratory tract infections has given conflicting results with studies has not having enough participants to provide statistical confidence.
AIM: To undertake a synthesis of IPD from both randomised controlled trials (RCTs) and observational cohort studies of respiratory tract infections (RTI) in order to investigate the prognostic value of microbiological data in addition to, or instead of, clinical symptoms and signs.
METHODS: A systematic search of Cochrane Central Register of Controlled Trials, Ovid Medline and Ovid Embase will be carried out for studies of acute respiratory infection in primary care settings. The outcomes of interest are duration of disease, severity of disease, repeated consultation with new/worsening illness and complications requiring hospitalisation. Authors of eligible studies will be contacted to provide anonymised individual participant data. The data will be harmonised and aggregated. Multilevel regression analysis will be conducted to determine key outcome measures for different potential pathogens and whether these offer any additional information on prognosis beyond clinical symptoms and signs.
TRIAL REGISTRATION: PROSPERO Registration number: CRD42023376769.

43. Cancers (Basel). 2023 Nov 26;15(23):5595. doi: 10.3390/cancers15235595.
Cervical Cancer Stages, Human Papillomavirus Integration, and Malignant Genetic Mutations: Integrative Analysis of Datasets from Four Different Cohorts.
Mohammed FA(1)(2)(3), Tune KK(1)(2), Jett M(4), Muhie S(5)(6).
Author information:
(1)Department of Software Engineering, College of Engineering, Addis Ababa Science and Technology University, Addis Ababa 16417, Ethiopia.
(2)Center of Excellence for HPC and Big Data Analytics, Addis Ababa Science and Technology University, Addis Ababa 16417, Ethiopia.
(3)Department of Information Technology, College of Computing and Health Informatics, Wolkite University, Wolkite P.O. Box 07, Ethiopia.
(4)US Army Medical Research and Development Command, Head Quarter, Walter Reed Army Institute of Research, Silver Spring, MD 20910, USA.
(5)Medical Readiness Systems Biology, Walter Reed Army Institute of Research, Silver Spring, MD 20910, USA.
(6)The Geneva Foundation, Silver Spring, MD 20910, USA.
Cervical cancer represents a significant global health concern, stemming from

persistent infections with high-risk types of human papillomavirus (HPV). The understanding of cervical cancer's clinical correlates, risk factors, molecular mechanisms, stages, and associated genetic mutations is important for early detection and improved treatment strategies. Through integrated analysis of clinical and molecular datasets, this study aims to identify key factors that are overlapping and distinct across four cohorts of different races and regions. Here, datasets from four distinct cohorts of patients from Uganda (N = 212), the United States of America (USA) (N = 228), China (N = 106), and Venezuela (N = 858) were examined to comprehensively explore the relationships between cervical cancer stages, HPV types (clades), productive HPV integration, and malignant genetic mutations. Cohort-specific findings included the occurrence frequencies of cervical cancer stages and grades. The majority of patients from the USA and China were diagnosed with stages I and II, while those from Uganda were diagnosed with stages II and III, reflecting levels of awareness and the availability of HPV vaccines and screening services. Conversely, cervical cancer and its stages were positively correlated with HPV types (clades), HPV integration, and risk-factor habits across the cohorts. Our findings indicate that the more common squamous cervical cancer can be potentially due to productive HPV16 (clade 9) integration. At the molecular level, pathways related to HPV infection, cancer-related conditions, and viral carcinogenesis were among the most significant pathways associated with mutated genes in cervical cancer (across cohorts). These findings collectively corroborate the prominent role of HPV infection and integration leading to genetic mutation and hence to the development of cervical cancer and its stages across patients of distinct races and regions.

44. Lancet Infect Dis. 2023 Nov 24:S1473-3099(23)00501-7. doi: 10.1016/S1473-3099(23)00501-7. Online ahead of print.

Safety and immunogenicity of ChAdOx1 85A prime followed by MVA85A boost compared with BCG revaccination among Ugandan adolescents who received BCG at birth: a randomised, open-label trial.

Wajja A(1), Nassanga B(2), Natukunda A(3), Serubanja J(3), Tumusiime J(3), Akurut H(3), Oduru G(3), Nassuuna J(3), Kabagenyi J(3), Morrison H(4), Scott H(5), Doherty RP(5), Marshall JL(5), Puig IC(5), Cose S(6), Kaleebu P(6), Webb EL(7), Satti I(5), McShane H(4), Elliott AM(6); TB042 Study Team.
Collaborators: Namutebi M, Nakazibwe E, Onen C, Apuule B, Akello F, Mukasa M, Nnaluwooza M, Sewankambo M, Kiwanuka S, Kiwudhu F, Imede E, Nkurunungi G, Nakawungu PK, Kabami G, Nuwagaba E, Akello M.
Author information:
(1)MRC/UVRI and LSHTM Uganda Research Unit, Entebbe, Uganda; Department of Global Health, Amsterdam University Medical Centers, Amsterdam, Netherlands; Amsterdam Institute for Global Health and Development, Amsterdam University Medical Centers, Amsterdam, Netherlands; Department of Clinical Research, London School of Hygiene & Tropical Medicine, London, UK.
(2)MRC/UVRI and LSHTM Uganda Research Unit, Entebbe, Uganda; The Jenner Institute, Old Road Campus Research Building, University of Oxford, Oxford, UK; Department of Immunology and Molecular Biology, School of Biomedical Sciences,

College of Health Sciences, Makerere University, Kampala, Uganda. Electronic address: beatrice.nassanga@gmail.com.
(3)MRC/UVRI and LSHTM Uganda Research Unit, Entebbe, Uganda.
(4)The Jenner Institute, Old Road Campus Research Building, University of Oxford, Oxford, UK; Centre for Clinical Vaccinology and Tropical Medicine, The Jenner Institute, University of Oxford, Churchill Hospital, Oxford, UK.
(5)The Jenner Institute, Old Road Campus Research Building, University of Oxford, Oxford, UK.
(6)MRC/UVRI and LSHTM Uganda Research Unit, Entebbe, Uganda; Department of Clinical Research, London School of Hygiene & Tropical Medicine, London, UK.
(7)Department of Infectious Disease Epidemiology, London School of Hygiene & Tropical Medicine, London, UK.
BACKGROUND: BCG confers reduced, variable protection against pulmonary tuberculosis. A more effective vaccine is needed. We evaluated the safety and immunogenicity of candidate regimen ChAdOx1 85A-MVA85A compared with BCG revaccination among Ugandan adolescents.
METHODS: After ChAdOx1 85A dose escalation and age de-escalation, we did a randomised open-label phase 2a trial among healthy adolescents aged 12-17 years, who were BCG vaccinated at birth, without evident tuberculosis exposure, in Entebbe, Uganda. Participants were randomly assigned (1:1) using a block size of 6, to ChAdOx1 85A followed by MVA85A (on day 56) or BCG (Moscow strain). Laboratory staff were masked to group assignment. Primary outcomes were solicited and unsolicited adverse events (AEs) up to day 28 and serious adverse events (SAEs) throughout the trial; and IFN-γ ELISpot response to antigen 85A (day 63 [geometric mean] and days 0-224 [area under the curve; AUC).
FINDINGS: Six adults (group 1, n=3; group 2, n=3) and six adolescents (group 3, n=3; group 4, n=3) were enrolled in the ChAdOx1 85A-only dose-escalation and age de-escalation studies (July to August, 2019). In the phase 2a trial, 60 adolescents were randomly assigned to ChAdOx1 85A-MVA85A (group 5, n=30) or BCG (group 6, n=30; December, 2019, to October, 2020). All 60 participants from groups 5 and 6 were included in the safety analysis, with 28 of 30 from group 5 (ChAdOx1 85A-MVA85A) and 29 of 30 from group 6 (BCG revaccination) analysed for immunogenicity outcomes. In the randomised trial, 60 AEs were reported among 23 (77%) of 30 participants following ChAdOx1 85A-MVA85A, 31 were systemic, with one severe event that occurred after the MVA85A boost that was rapidly self-limiting. All 30 participants in the BCG revaccination group reported at least one mild to moderate solicited AE; most were local reactions. There were no SAEs in either group. Ag85A-specific IFN-γ ELISpot responses peaked on day 63 in the ChAdOx1 85A-MVA85A group and were higher in the ChAdOx1 85A-MVA85A group compared with the BCG revaccination group (geometric mean ratio 30·59 [95% CI 17·46-53·59], p<0·0001, day 63; AUC mean difference 57 091 [95% CI 40 524-73 658], p<0·0001, days 0-224).
INTERPRETATION: The ChAdOx1 85A-MVA85A regimen was safe and induced stronger Ag85A-specific responses than BCG revaccination. Our findings support further development of booster tuberculosis vaccines.
FUNDING: UK Research and Innovations and Medical Research Council.
TRANSLATIONS: For the Swahili and Luganda translations of the abstract see

Supplementary Materials section.

45. Front Vet Sci. 2023 Nov 22;10:1271434. doi: 10.3389/fvets.2023.1271434.
Application of the Nagoya Protocol to veterinary pathogens: concerns for the control of foot-and-mouth disease.
Horsington J(1), Abbeloos E(2), Kassimi LB(3)(4), Boonsuya Seeyo K(3)(5), Capozzo AV(6), Chepkwony E(7), Eblé P(3)(8), Galdo-Novo S(3)(9), Gizaw D(10), Gouverneur L(11), Grazioli S(3)(12), Heath L(3)(13), Hudelet P(2), Hyera JMK(3)(14), Ilott M(1), King A(15), Lefebvre DJ(3)(16), Mackay D(1), Metwally S(17), Mwiine FN(6)(18), Nfon CK(3)(19), Park MK(20), Pituco EM(21), Rosso F(1), Simon F(22), Ularamu HG(23), Vermeij P(15), Vosloo W(6)(24), King DP(3)(11).
Author information:
(1)European Commission for the Control of Foot-and-Mouth Disease (EuFMD), Rome, Italy.
(2)Boehringer Ingelheim Animal Health, Lyon, France.
(3)WOAH/FAO FMD Reference Laboratory Network.
(4)Animal Health Laboratory, UMR1161 Virology, INRAE, ANSES, ENVA, Paris-Est University, Maisons-Alfort, France.
(5)Regional Reference Laboratory for FMD in Southeast Asia, Pakchong, Thailand.
(6)Global Foot-and-Mouth Disease Research Alliance (GFRA).
(7)Foot and Mouth Disease National Laboratory, Embakasi, Directorate of Veterinary Services, State Department of Livestock, Nairobi, Kenya.
(8)Wageningen Bioveterinary Research (WBVR), Lelystad, Netherlands.
(9)Servicio Nacional de Sanidad y Calidad Agroalimentaria (SENASA), Buenos Aires, Argentina.
(10)Animal Health Institute (AHI), Sebeta, Ethiopia.
(11)FAO World Reference Laboratory for FMD, The Pirbright Institute, Pirbright, United Kingdom.
(12)Istituto Zooprofilattico Sperimentale della Lombardia e dell'Emilia-Romagna, Brescia, Italy.
(13)Transboundary Animal Disease Laboratory, Onderstepoort Veterinary Institute, Agricultural Research Council, Onderstepoort, South Africa.
(14)WOAH Reference Laboratory for FMD, Botswana Vaccine Institute, Lejara, Gaborone, Botswana.
(15)MSD Animal Health, Boxmeer, Netherlands.
(16)Sciensano, Scientific Direction of Infectious Diseases in Animals, Service for Exotic and Vector-borne Diseases, Brussels, Belgium.
(17)Food and Agriculture Organization of the United Nations (FAO), Rome, Italy.
(18)College of Veterinary Medicine, Animal Resources, and Biosecurity, Makerere University, Kampala, Uganda.
(19)Canadian Food Inspection Agency, National Centre for Foreign Animal Disease, Winnipeg, MB, Canada.
(20)World Organisation for Animal Health (WOAH), Paris, France.
(21)Pan American Health Organization, Regional Office for the Americas of the World Health Organization, Rio de Janeiro, Brazil.
(22)Biogenesis Bago, Buenos Aires, Argentina.
(23)Viral Research Division, National Veterinary Research Institute, Vom,

Nigeria.
(24)Australian Centre for Disease Preparedness, Commonwealth Scientific and Industrial Research Organisation (CSIRO) Health and Biosecurity, Geelong, VIC, Australia.
The Nagoya Protocol is an international agreement adopted in 2010 (and entered into force in 2014) which governs access to genetic resources and the fair and equitable sharing of benefits from their utilisation. The agreement aims to prevent misappropriation of genetic resources and, through benefit sharing, create incentives for the conservation and sustainable use of biological diversity. While the equitable sharing of the benefits arising from the utilisation of genetic resources is a widely accepted concept, the way in which the provisions of the Nagoya Protocol are currently being implemented through national access and benefit-sharing legislation places significant logistical challenges on the control of transboundary livestock diseases such as foot-and-mouth disease (FMD). Delays to access FMD virus isolates from the field disrupt the production of new FMD vaccines and other tailored tools for research, surveillance and outbreak control. These concerns were raised within the FMD Reference Laboratory Network and were explored at a recent multistakeholder meeting hosted by the European Commission for the Control of FMD. The aim of this paper is to promote wider awareness of the Nagoya Protocol, and to highlight its impacts on the regular exchange and utilisation of biological materials collected from clinical cases which underpin FMD research activities, and work to develop new epidemiologically relevant vaccines and other diagnostic tools to control the disease.

46. Sci Rep. 2023 Nov 22;13(1):20507. doi: 10.1038/s41598-023-47379-z.
Genomic characterization of SARS-CoV-2 from Uganda using MinION nanopore sequencing.
Kia P(1), Katagirya E(2), Kakembo FE(3), Adera DA(4), Nsubuga ML(2), Yiga F(2), Aloyo SM(2), Aujat BR(2), Anguyo DF(5), Katabazi FA(2), Kigozi E(2), Joloba ML(2), Kateete DP(6).
Author information:
(1)Department of Immunology and Molecular Biology, School of Biomedical Sciences, College of Health Sciences, Makerere University, Kampala, Uganda. kiapriscilla@gmail.com.
(2)Department of Immunology and Molecular Biology, School of Biomedical Sciences, College of Health Sciences, Makerere University, Kampala, Uganda.
(3)The African Centers of Excellence in Bioinformatics and Date Intensive Sciences, Infectious Disease Institute, College of Health Sciences, Makerere University, Kampala, Uganda.
(4)Multifunctional Research Laboratories, Gulu University, Gulu, Uganda.
(5)Department of Biology, Muni University, Arua, Uganda.
(6)Department of Immunology and Molecular Biology, School of Biomedical Sciences, College of Health Sciences, Makerere University, Kampala, Uganda. david.kateete@mak.ac.ug.
SARS-CoV-2 undergoes frequent mutations, affecting COVID-19 diagnostics, transmission and vaccine efficacy. Here, we describe the genetic diversity of 49

SARS-CoV-2 samples from Uganda, collected during the COVID-19 waves of 2020/2021. Overall, the samples were similar to previously reported SARS-CoV-2 from Uganda and the Democratic Republic of Congo (DRC). The main lineages were AY.46 and A.23, which are considered to be Delta SARS-CoV-2 variants. Further, a total of 268 unique single nucleotide variants and 1456 mutations were found, with more than seventy percent mutations in the ORF1ab and S genes. The most common mutations were 2042C>G (83.4%), 14143C>T (79.5%), 245T>C (65%), and 1129G>T (51%), which occurred in the S, ORF1ab, ORF7a and N genes, respectively. As well, 28 structural variants-21 insertions and 7 deletions, occurred in 16 samples. Our findings point to the possibility that most SARS-CoV-2 infections in Uganda at the time arose from local spread and were not newly imported. Moreover, the relatedness of variants from Uganda and the DRC reflects high human mobility and interaction between the two countries, which is peculiar to this region of the world.

47. Medicine (Baltimore). 2023 Nov 17;102(46):e36166. doi:
10.1097/MD.0000000000036166.

Regulations of malaria in children with human immunodeficiency virus infection: A review.

Opeyemi AA(1), Obeagu EI(2).
Author information:
(1)Department of Medical Laboratory Science, Achievers University, Owo, Nigeria.
(2)Department of Medical Laboratory Science, Kampala International University, Kampala, Uganda.

This comprehensive review explores the intricate relationship between 2 major global health challenges, malaria and HIV, with a specific focus on their impact on children. These diseases, both endemic in sub-Saharan Africa, create a dual burden that significantly elevates the risk of morbidity and mortality, particularly in children with compromised immune systems due to HIV. The review delves into the complex mechanisms by which these infections interact, from heightened clinical malaria frequencies in HIV-infected individuals to the potential impact of antiretroviral therapy on malaria treatment. Different research engines were utilized in writing this paper such as Web of Science, Google Scholar, Pubmed Central, ResearchGate, and Academia Edu. To address this critical health concern, the study identifies and discusses various regulatory and treatment strategies. It emphasizes the importance of daily cotrimoxazole prophylaxis and insecticide-treated nets in preventing malaria in children with HIV. The potential of antiretroviral protease inhibitors and mRNA-based vaccines as innovative solutions is highlighted. Additionally, the study underscores the significance of climate data and artificial intelligence in improving diagnostics and drug development. Furthermore, the review introduces the concept of genetically modified mosquitoes as a novel approach to vector control, offering a promising avenue to protect HIV-positive individuals from mosquito-borne diseases like malaria. Through a comprehensive analysis of these strategies, the study aims to provide a foundation for policymakers, healthcare professionals, and researchers to develop effective regulations and interventions that reduce the dual burden of malaria and HIV in children,

improving public health outcomes in endemic regions.

48. Health Policy Plan. 2023 Nov 16;38(Supplement_2):ii36-ii50. doi:
10.1093/heapol/czad051.
Justice implications of health and food security policies for Indigenous peoples
facing COVID-19: a qualitative study and policy analysis in Peru.
Chicmana-Zapata V(1), Arotoma-Rojas I(2), Anza-Ramírez C(3), Ford J(2),
Galappaththi EK(4), Pickering K(5), Sacks E(6), Togarepi C(7), Perera CD(4), van
Bavel B(8), Hyams K(9), Akugre FA(10), Nkalubo J(11), Dharmasiri I(4), Nakwafila
O(12), Mensah A(10), Miranda JJ(13), Zavaleta-Cortijo C(14).
Author information:
(1)Universidad Peruana Cayetano Heredia, Av. Honorio Delgado 430, San Martín de
Porres, Lima 15102, Perú.
(2)Priestley International Centre for Climate, University of Leeds, Woodhouse,
Leeds LS2 9JT, United Kingdom.
(3)CRONICAS-Universidad Peruana Cayetano Heredia, Av. Armendáriz 445,
Miraflores, Lima, 15074, Perú.
(4)Department of Geography, Virginia Polytechnic Institute and State University,
238 Wallace Hall, Blacksburg, VA 24060, USA.
(5)Environmental Sustainability Research Centre, Brock University, 1812 Sir
Isaac Brock Way, St. Catharines, ON L2S 3A1, Canada.
(6)Johns Hopkins Bloomberg School of Public Health, 615 N Wolfe St., Baltimore,
MD 21205, USA.
(7)University of Namibia, Ogongo Campus, Private Bag X5507, Eliander Mwatale
Street, Oshakati, Namibia.
(8)University of Leeds, Woodhouse, Leeds LS2 9JT, United Kingdom.
(9)University of Warwick, Coventry CV4 7AL, United Kingdom.
(10)Institute for Environment and Sanitation Studies, University of Ghana,
MR39+C4X, Annie Jiagge Rd, Accra, Ghana.
(11)Mulago National Referral Hospital and Uganda National Health Research
Organization, Mulago Hospital Complex, Mulago Hill, P.O. Box 7051, Kampala,
Uganda.
(12)University of Namibia, 340 Mandume Ndemufayo Avenue, Pioneerspark, Namibia.
(13)CRONICAS-Universidad Peruana Cayetano Heredia, Av. Honorio Delgado 430, San
Martín de Porres, Lima 15102, Perú.
(14)Unidad de Ciudadania Intercultural y Salud Indígena (UCISI), Facultad de
Salud Pública y Administración, Universidad Peruana Cayetano Heredia, Av.
Honorio Delgado 430, San Martín de Porres, Lima 15102, Perú.
The spread of COVID-19 in Peru resulted in the declaration of a national health
emergency, in which Indigenous peoples were identified as being particularly
vulnerable due to their pre-existing poor health indicators and disadvantaged
social conditions. The aim of this paper is to examine how the Peruvian
government responded to the health and food needs of the Shawi and Ashaninka
Indigenous peoples of Peru during the first 18 months of the pandemic (March
2020-August 2021). This study uses both official policy documents and real-world
experiences to evaluate policy responses in terms of their immediate impact and
their longer-term sustainability and contribution to the improvement of health,

well-being and justice for Indigenous communities. Four health and food security responses were evaluated: the Amazon Health Plan and Indigenous Command; food aid; cash aid; and COVID-19 vaccination. We employed the Multidimensional Injustice Framework to analyse the justice implications of the design and implementation of responses. Data collection included 71 interviews with government officials (n = 7), Indigenous leaders (n = 31) and community members (n = 33). The results show how national and regional governments released policies to address the health and food needs of Indigenous peoples directly or indirectly, as part of a broader focus on vulnerable people. However, justice implications were not sufficiently addressed in the design or implementation of the responses. On the distributive dimension, Indigenous communities were prioritized to receive health goods and services, nevertheless, the distribution had shortcomings that impeded their collection and Indigenous food systems and livelihoods were largely overlooked. On the procedural dimension, Indigenous representatives were included to provide culturally sensitive feedback on health interventions, but without funding, and furthermore, the community members had only passive participation. This paper points out the importance of considering and addressing justice implications for more effective and fairer health and food policy responses to current and future health crises.

49. PLoS One. 2023 Nov 16;18(11):e0279528. doi: 10.1371/journal.pone.0279528.
COVID-19 outbreak control strategies and their impact on the provision of essential health services in Ghana: An exploratory-sequential study.
Dwomoh D(1), Yeboah I(2), Ndejjo R(3), Kabwama SN(4), Aheto JM(1), Liu A(5), Lazenby S(5), Ohemeng F(6), Takyi SA(7), Issah I(7), Bawuah SA(7), Wanyenze RK(3), Fobil J(7).
Author information:
(1)Department of Biostatistics, School of Public Health, University of Ghana, Accra, Ghana.
(2)Institute of Work, Employment and Society, University of Professional Studies, Accra, Ghana.
(3)Department of Disease Control and Environmental Health, School of Public Health, Makerere University, Kampala, Uganda.
(4)Department of Community Health and Behavioural Sciences, School of Public Health, Makerere University, Kampala, Uganda.
(5)Gates Ventures, Kirkland, Washington, United States of America.
(6)Department of Sociology, School of Humanities, University of Ghana, Accra, Ghana.
(7)Department of Biological, Environmental, and Occupational Health, School of Public Health, University of Ghana, Accra, Ghana.
BACKGROUND: The COVID-19 pandemic has led to substantial interruptions in critical health services, with 90% of countries reporting interruptions in routine vaccinations, maternal health care and chronic disease management. The use of non-pharmaceutical interventions (NPIs) such as lockdowns and self-isolation had implications on the provision of essential health services (EHS). We investigated exemplary COVID-19 outbreak control strategies and explored the extent to which the adoption of these NPIs affected the provision

of EHS including immunization coverage and facility-based deliveries. Finally, we document core health system strategies and practices adopted to maintain EHS during the early phase of the pandemic.
METHODS: This study used an explanatory sequential study design. First, we utilized data from routine health management information systems to quantify the impact of the pandemic on the provision of EHS using interrupted time series models. Second, we explored exemplary strategies and health system initiatives that were adopted to prevent the spread of COVID-19 infections while maintaining the provision of EHS using in-depth interviews with key informants including policymakers and healthcare providers.
RESULTS: The COVID-19 pandemic and the interventions that were implemented disrupted the provision of EHS. In the first month of the COVID-19 pandemic, Oral Polio and pentavalent vaccination coverage reduced by 15.2% [95% CI = -22.61, -7.87, p<0.001] and 12.4% [95% CI = 17.68, -7.13; p<0.001] respectively. The exemplary strategies adopted in maintaining the provision of EHS while also responding to the spread of infections include the development of new policy guidelines that were disseminated with modified service delivery models, new treatment and prevention guidelines, the use of telemedicine and medical drones to provide EHS and facilitate rapid testing of suspected cases.
CONCLUSION: The implementation of different NPIs during the peak phase of the pandemic disrupted the provision of EHS. However, the Ministry of Health leveraged the resilient health system and deployed efficient, all-inclusive, and integrated infectious disease management and infection prevention control strategies to maintain the provision of EHS while responding to the spread of infections.

50. PLoS Negl Trop Dis. 2023 Nov 15;17(11):e0011455. doi: 10.1371/journal.pntd.0011455. eCollection 2023 Nov.
Transcriptome analysis of peripheral blood of Schistosoma mansoni infected children from the Albert Nile region in Uganda reveals genes implicated in fibrosis pathology.
Namulondo J(1), Nyangiri OA(1), Kimuda MP(1), Nambala P(2), Nassuuna J(3), Egesa M(3)(4), Nerima B(2), Biryomumaisho S(1), Mugasa CM(1), Nabukenya I(1), Kato D(1), Elliott A(3)(4), Noyes H(5), Tweyongyere R(1), Matovu E(1), Mulindwa J(2); TrypanoGEN+ research group of the H3Africa consortium.
Author information:
(1)College of Veterinary Medicine, Animal Resources and Biosecurity, Makerere University, Kampala, Uganda.
(2)College of Natural Sciences, Makerere University, Kampala, Uganda.
(3)Vaccine Research Theme, MRC/UVRI and LSHTM Uganda Research Unit, Entebbe, Uganda.
(4)Department of Infection Biology, London School of Hygiene and Tropical Medicine, London, United Kingdom.
(5)Centre for Genomic Research, University of Liverpool, United Kingdom.
Over 290 million people are infected by schistosomes worldwide. Schistosomiasis control efforts focus on mass drug treatment with praziquantel (PZQ), a drug that kills the adult worm of all Schistosoma species. Nonetheless, re-infections

have continued to be detected in endemic areas with individuals living in the same area presenting with varying infection intensities. Our objective was to characterize the transcriptome profiles in peripheral blood of children between 10-15 years with varying intensities of Schistosoma mansoni infection living along the Albert Nile in Uganda. RNA extracted from peripheral blood collected from 44 S. mansoni infected (34 high and 10 low by circulating anodic antigen [CAA] level) and 20 uninfected children was sequenced using Illumina NovaSeq S4 and the reads aligned to the GRCh38 human genome. Differential gene expression analysis was done using DESeq2. Principal component analysis revealed clustering of gene expression by gender when S. mansoni infected children were compared with uninfected children. In addition, we identified 14 DEGs between S. mansoni infected and uninfected individuals, 56 DEGs between children with high infection intensity and uninfected individuals, 33 DEGs between those with high infection intensity and low infection intensity and no DEGs between those with low infection and uninfected individuals. We also observed upregulation and downregulation of some DEGs that are associated with fibrosis and its regulation. These data suggest expression of fibrosis associated genes as well as genes that regulate fibrosis in S. mansoni infection. The relatively few significant DEGS observed in children with schistosomiasis suggests that chronic S. mansoni infection is a stealth infection that does not stimulate a strong immune response.

51. Viruses. 2023 Nov 11;15(11):2246. doi: 10.3390/v15112246.
A Re-Evaluation of African Swine Fever Genotypes Based on p72 Sequences Reveals the Existence of Only Six Distinct p72 Groups.
Spinard E(1)(2)(3), Dinhobl M(1)(2)(3), Tesler N(1)(4), Birtley H(1)(4), Signore AV(3)(5), Ambagala A(3)(5), Masembe C(3)(6), Borca MV(1)(2)(3), Gladue DP(1)(2)(3).
Author information:
(1)Plum Island Animal Disease Center, Agricultural Research Service, U.S. Department of Agriculture, Foreign Animal Disease Research Unit, Orient, NY 11957, USA.
(2)National Bio and Agro-Defense Facility, Agricultural Research Service, U.S. Department of Agriculture, Manhattan, KS 66502, USA.
(3)Center of Excellence for African Swine Fever Genomics, Guilford, CT 06437, USA.
(4)Oak Ridge Institute for Science and Education (ORISE), Oak Ridge, TN 37830, USA.
(5)National Centre for Foreign Animal Disease, Canadian Food Inspection Agency, Winnipeg, MB R3E 3M4, Canada.
(6)College of Natural Resources (CoNAS), Makerere University, Kampala P.O. Box 7062, Uganda.
The African swine fever virus (ASFV) is currently causing a world-wide pandemic of a highly lethal disease in domestic swine and wild boar. Currently, recombinant ASF live-attenuated vaccines based on a genotype II virus strain are commercially available in Vietnam. With 25 reported ASFV genotypes in the literature, it is important to understand the molecular basis and usefulness of

ASFV genotyping, as well as the true significance of genotypes in the epidemiology, transmission, evolution, control, and prevention of ASFV. Historically, genotyping of ASFV was used for the epidemiological tracking of the disease and was based on the analysis of small fragments that represent less than 1% of the viral genome. The predominant method for genotyping ASFV relies on the sequencing of a fragment within the gene encoding the structural p72 protein. Genotype assignment has been accomplished through automated phylogenetic trees or by comparing the target sequence to the most closely related genotyped p72 gene. To evaluate its appropriateness for the classification of genotypes by p72, we reanalyzed all available genomic data for ASFV. We conclude that the majority of p72-based genotypes, when initially created, were neither identified under any specific methodological criteria nor correctly compared with the already existing ASFV genotypes. Based on our analysis of the p72 protein sequences, we propose that the current twenty-five genotypes, created exclusively based on the p72 sequence, should be reduced to only six genotypes. To help differentiate between the new and old genotype classification systems, we propose that Arabic numerals (1, 2, 8, 9, 15, and 23) be used instead of the previously used Roman numerals. Furthermore, we discuss the usefulness of genotyping ASFV isolates based only on the p72 gene sequence.

52. West Afr J Med. 2023 Nov 10;40(11 Suppl 1):S9.
EPIDEMIOLOGIC TREND AND CASE FATALITY OF CHILDHOOD TETANUS IN FEDERAL TEACHING HOSPITAL GOMBE; A 20YEARS REVIEW.
Isaac WE(1), Jalo I(1), Yohanna G(1), Aliu R(1), Ebiseke K(1), Girbo A(1), Olatoke L(1), Raymond PM(1), Iliya A(1), Ndubusi V(1), Rabo Y(1), Daniel GE(1), Apollos D(1), Bakura B(1), Usman MO(1), Kamaludden H(1), Abdulshaheed B(1), Tukur H(1), Joel Z(1), Abdulkarim H(1), Abdulrahman S(1), Adeniran OA(1), Adamu A(1), Ibrahim B(1), Meller BT(1).
Author information:
(1)Department of Paediatrics, Federal Teaching Hospital, Gombe.
INTRODUCTION: Tetanus is a vaccine-preventable disease, it remains a significant cause of morbidity and mortality in both neonatal and post-neonatal periods, especially in developing countries with limited health facilities and inadequate vaccination. The overall case fatality rate (CFR) is 13.2% globally, highest in the neonatal period and in sub-Saharan Africa. CFR is 64%, 47%, and 43% in Nigeria, Uganda, and Tanzania respectively.
OBJECTIVES: To determine the Case Fatality Rate of Childhood tetanus in FTHG from 2000-2019.
METHODOLOGY: All cases and deaths from tetanus amongst children aged 0-18 years in paediatric medical ward of FTHG over the last two decades diagnosed clinically and classified using ICD-10 were analysed.
RESULTS: 95 cases of tetanus out of 26,716 total admissions constituting 0.004%. There were 49 tetanus deaths out of 3956 total childhood deaths (0.012%) over the study period. Males constituted 66% (63/95). 30% (28/95) were aged 0-28 days; 23.1% (22/95) were adolescents. Fulani and Hausa constituted 37% (34/95) and 31% (29/95) respectively. Admission was highest in the dry season 52% (50/95 %). The overall tetanus CFR was 51.6%; 78% of deaths were in males (38/49), 30%

in neonates, and 23% in adolescents. CFR was highest during the dry season (67.3%). Hausa and Fulani had CFR of 51% and 40% respectively. P-value <0.05 The CFR was 88% between 2000-2004, 72% from 2005-2009, 71% between 2010-2014 and 33% from 2015-2019.
CONCLUSION: Tetanus CFR is still high among neonates and adolescents. Maternal tetanus vaccine and booster doses in children need strengthening.

53. Front Public Health. 2023 Nov 9;11:1256007. doi: 10.3389/fpubh.2023.1256007.
Levels of handwashing and vaccine uptake in Kenya, Uganda, and Tanzania to prevent and control COVID-19: a systematic review and meta-analysis.
Muchangi JM(1), Mturi J(2), Mukasa H(3), Kithuki K(1), Kosgei SJ(1), Kanyangi LM(1), Mararo R(1), Nankanja M(2).
Author information:
(1)Amref Health Africa, Nairobi, Kenya.
(2)Amref Health Africa, Dar es Salaam, Tanzania.
(3)Amref Health Africa, Kampala, Uganda.
INTRODUCTION: Coronavirus Disease 2019 (COVID-19) presents a massive challenge in Africa due to overwhelmed and underresourced health systems, as well as the existing burden of communicable and non- communicable diseases. Self-inoculation may occur when an individual touches their mucous membrane following direct contact between their hands and severe acute respiratory syndrome coronavirus-2 (SARS-CoV-2)-contaminated surfaces. Therefore, handwashing can be used along with COVID-19 vaccines to reduce the spread of SARS-CoV-2 and the burden of COVID-19. We were interested in investigating the levels of handwashing and vaccine uptake to control and prevent COVID-19 in Kenya, Uganda and Tanzania using a systematic review and meta-analysis.
METHODS: We searched PubMed, African Index Medicus and African Journals Online for studies published since inception to 31st January 2023. We included all studies that assessed: the levels of COVID-19 vaccine acceptance and hesitance as indicators of vaccine uptake; and studies on the levels of handwashing to prevent and control COVID-19 in Kenya, Uganda and Tanzania. Study findings were synthesized by meta-analysis to get the pooled effect measure. Three studies were synthesized qualitatively due to high level of heterogeneity in effect measure precluding a quantitative meta-analysis.
RESULTS: Our search identified 128 articles of which 17 studies with 18,305 participants and 622 vaccination sites were reviewed with 14 of them being meta-analyzed. This systematic review and meta-analysis reports high levels of COVID-19 vaccine acceptance and handwashing in Kenya, Uganda and Tanzania at 67% (95% CI: 55, 78) and 88% (95% CI: 73, 97), respectively. Vaccine hesitance among the participants was low at 31% (95% CI: 15, 49).
DISCUSSION: Despite their importance in the control and prevention of COVID-19, some countries do not implement handwashing and vaccination effectively. There is a need for public health strategies to raise awareness about the importance of handwashing and the uptake of the COVID-19 vaccine.
SYSTEMATIC REVIEW REGISTRATION: https://www.crd.york.ac.uk/PROSPERO/, PROSPERO ID CRD42023394698.

54. Hepat Med. 2023 Nov 7;15:209-220. doi: 10.2147/HMER.S384172. eCollection 2023.
COVID-19 and the Liver: A Complex and Evolving Picture.
Taylor-Robinson SD(1)(2), Morgan MY(3).
Author information:
(1)Department of Surgery and Cancer, Imperial College London, London, UK.
(2)Department of Public Health, Busitema University and Mbale Clinical Research Institute, Mbale, Uganda.
(3)UCL Institute for Liver & Digestive Health, Division of Medicine, Royal Free Campus, University College London, London, UK.

Although the severe acute respiratory syndrome coronavirus 2 (SARS-CoV-2) primarily attacks the respiratory system, other organs, such as the liver, are also affected. In this overview, the effects of SARS-CoV-2 infection on the liver in both healthy people and in those with pre-existing liver disease are documented; the relationship between coronavirus disease 19 (COVID-19) vaccination and liver injury is examined; the mechanism of SARS-CoV-2-associated liver injury is explored; and the long-term consequences of COVID-19 are delineated, both in people with and without pre-existing liver disease.

55. Vaccine. 2023 Nov 3;41 Suppl 2:S41-S52. doi: 10.1016/j.vaccine.2023.04.024.
Vaccine value profile for Group B streptococcus.
Trotter CL(1), Alderson M(2), Dangor Z(3), Ip M(4), Le Doare K(5), Nakabembe E(6), Procter SR(7), Sekikubo M(8), Lambach P(9).
Author information:
(1)Department of Veterinary Medicine, University of Cambridge, Madingley Road, Cambridge CB3 0ES, UK. Electronic address: clt56@cam.ac.uk.
(2)PATH, 2201 Westlake Avenue, Suite,200, Seattle, WA 98121, USA. Electronic address: malderson@path.org.
(3)WITS VIDA Research Unit, University of the Witwatersrand, Chris Hani Baragwanath Hospital, 30 Chris Hani Road, Diepkloof, Soweto, 1862 Johannesburg, South Africa. Electronic address: Ziyaad.Dangor@wits.ac.za.
(4)The Chinese University of Hong Kong, Sha Tin, New Territories, Hong Kong, China. Electronic address: margaretip@cuhk.edu.hk.
(5)St George's, University of London, Cranmer Terrace, London SW17 0RE, UK. Electronic address: kiledoar@sgul.ac.uk.
(6)Makerere University School of Medicine, P.O. Box 7072, Kampala, Uganda. Electronic address: drevena@yahoo.co.uk.
(7)London School of Hygiene & Tropical Medicine, Keppel Street, London WC1E 7HT, UK. Electronic address: simon.procter@lshtm.ac.uk.
(8)Makerere University School of Medicine, P.O. Box 7072, Kampala, Uganda. Electronic address: msekikubo@yahoo.com.
(9)World Health Organization, Avenue Appia, Geneva CH-1211, Switzerland. Electronic address: lambachp@who.int.
Group B streptococcus (GBS) is a major global cause of neonatal meningitis, sepsis and pneumonia, with an estimated 91,000 infant deaths per year and an additional 46,000 stillbirths. GBS infection in pregnancy is also associated with adverse maternal outcomes and preterm births. As such, the World Health

Organization (WHO) prioritised the development of a GBS vaccine suitable for use in pregnant women and use in LMICs, where the burden of disease is highest. Several GBS vaccines are in clinical development. The WHO Defeating Meningitis by 2030 has set a target of 2026 for vaccine licensure. This 'Vaccine Value Profile' (VVP) for GBS is intended to provide a high-level, holistic assessment of the information and data that are currently available to inform the potential public health, economic and societal value of pipeline vaccines and vaccine-like products. This VVP was developed by a working group of subject matter experts from academia, non-profit organizations, public private partnerships and multi-lateral organizations, and in collaboration with stakeholders from the WHO regions of AFR, AMR, EUR, WPR. All contributors have extensive expertise on various elements of the GBS VVP and collectively aimed to identify current research and knowledge gaps. The VVP was developed using only existing and publicly available information.

56. BMC Infect Dis. 2023 Nov 3;23(1):758. doi: 10.1186/s12879-023-08776-x.
Gaps in vaccine management practices during vaccination outreach sessions in rural settings in southwestern Uganda.
Flavia A(1), Fred B(2), Eleanor T(2).
Author information:
(1)Department of Community Health, Faculty of Medicine, Mbarara University of Science & Technology, Mbarara, P.O. BOX 1410, Mbarara, Uganda. fatwiine@must.ac.ug.
(2)Department of Community Health, Faculty of Medicine, Mbarara University of Science & Technology, Mbarara, P.O. BOX 1410, Mbarara, Uganda.
BACKGROUND: Outreach efforts were developed to bolster people's access to and use of immunization services in underserved populations. However, there have been multiple outbreaks of diseases like measles in Uganda, prompting policy makers and stakeholders to ask many unanswered questions. This research study was created to uncover the discrepancies between vaccine management practices at immunization outreach sessions in rural South Western Uganda compared with existing standards.
METHODS: The observational qualitative study, was done in 16 public health facilities across four districts of Uganda. Data were collected using in-depth interviews, facility record reviews, and observation. We assessed the vaccine management procedures before immunization session, transportation used, set up at the outreach site, management practices during the outreach session and packing of vaccines - according to World Health Organization immunization practice recommendations. The data were transcribed, coded and categories were formed and triangulated. Themes were generated based on a socio-ecologic framework to gain a better understanding of healthcare provider practices during immunization sessions.
RESULTS: Fifty-one individuals were interviewed; four Assistant District Health Officers, four cold chain technicians, 15 focal persons for the Expanded Program on Immunization, and 28 health care providers. The respondents' mean age was 35, 43 (84.3%) were females and 24 (47.1%) had a diploma. 11 (69%) outreaches were conducted at a distance of 5-12 km from the health facility and 7 (44%) were

conducted in a building. For 8 outreaches (50%) health facility staff did not check the vaccine vial monitor status before the outreach while 12(75%) did not keep the vaccine hard lid cover closed during the sessions. The main areas of concern were insufficient vaccine integrity monitoring, improper handling and storage practices, deficient documentation, and inadequate vaccine transportation. These were similar across immunization outreach sites regardless of vaccine preventable disease outbreaks occurrences. The majority of these gaps were located at the individual level but were enabled by policy/environmental factors.
CONCLUSIONS: There are poor vaccine management procedures during outreach sessions contrary to established guidelines. Specific tactics to tackle knowledge deficiencies, health worker attitude, and fewer equipment shortages could improve compliance to guidelines.

57. Nat Rev Cardiol. 2023 Nov 2. doi: 10.1038/s41569-023-00940-9.
2023 World Heart Federation guidelines for the echocardiographic diagnosis of rheumatic heart disease.
Rwebembera J(#)(1), Marangou J(#)(2)(3)(4), Mwita JC(5), Mocumbi AO(6), Mota C(7)(8), Okello E(9), Nascimento B(10)(11), Thorup L(12), Beaton A(13)(14), Kado J(15)(16), Kaethner A(2)(17), Kumar RK(18), Lawrenson J(19)(20), Marijon E(21), Mirabel M(22), Nunes MCP(10)(11), Piñeiro D(23), Pinto F(24), Ralston K(25), Sable C(26), Sanyahumbi A(27), Saxena A(28), Sliwa K(29), Steer A(30)(31)(32), Viali S(33), Wheaton G(34), Wilson N(35), Zühlke L(36)(37), Reményi B(2)(17)(38).
Author information:
(1)Division of Adult Cardiology, Uganda Heart Institute, Kampala, Uganda. joselynr36@gmail.com.
(2)Global and Tropical Health Division, Menzies School of Health Research, Charles Darwin University, Darwin, Northern Territory, Australia.
(3)Department of Cardiology, Royal Perth Hospital, Perth, Western Australia, Australia.
(4)Department of Cardiology, Fiona Stanley Hospital, Perth, Western Australia, Australia.
(5)Department of Internal Medicine, University of Botswana and Princess Marina Hospital, Gaborone, Botswana.
(6)University Eduardo Mondlane, Maputo, Mozambique.
(7)Departamento de Paediatria, Faculdade de Medicina da Universidade Federal de Minas Gerais, Belo, Horizonte, Brazil.
(8)Divisão de Cardiologia Pediátrica e Fetal/Serviço de Cardiologia e Cirurgia Cardiovascular e Serviço de Paediatria, Hospital das Clínicas da Universidade Federal de Minas Gerais, Belo, Horizonte, Brazil.
(9)Division of Adult Cardiology, Uganda Heart Institute, Kampala, Uganda.
(10)Departamento de Clínica Médica, Faculdade de Medicina da Universidade Federal de Minas Gerais, Belo, Horizonte, Brazil.
(11)Serviço de Cardiologia e Cirurgia Cardiovascular, Hospital das Clínicas da Universidade Federal de Minas Gerais, Belo, Horizonte, Brazil.
(12)Department of Cardiothoracic Surgery, Rigshospitalet, Copenhagen University

Hospital, Copenhagen, Denmark.
(13)Department of Paediatrics, School of Medicine, University of Cincinnati, Cincinnati, OH, USA.
(14)Division of Cardiology, The Heart Institute, Cincinnati Children's Medical Center, Cincinnati, OH, USA.
(15)Wesfarmers Centre of Vaccine and Infectious Diseases, Telethon Kids Institute, Perth, Western Australia, Australia.
(16)School of Medicine, University of Western Australia, Perth, Western Australia, Australia.
(17)NT Cardiac, Darwin, Northern Territory, Australia.
(18)Amrita Institute of Medical Sciences and Research Centre, Kochi, India.
(19)Paediatric Cardiology Service of the Western Cape, Red Cross War Memorial Children's Hospital and Tygerberg Hospital, Cape Town, South Africa.
(20)Department of Paediatrics and Child Health, Stellenbosch University, Stellenbosch, South Africa.
(21)Division of Cardiology, European Georges Pompidou Hospital, Paris, France.
(22)Institut Mutualiste Montsouris, Paris, France.
(23)Faculty of Medicine, University of Buenos Aires, Buenos Aires, Argentina.
(24)Cardiology Department, Centro Hospitalar Universitário Lisboa Norte, Centro Académico de Medicina de Lisboa, The Cardiovascular Centre of the University of Lisbon, Faculdade de Medicina, Universidade de Lisboa, Lisbon, Portugal.
(25)World Heart Federation, Geneva, Switzerland.
(26)Division of Cardiology, Children's National Hospital, Washington, DC, USA.
(27)Baylor College of Medicine, Texas Children's Hospital, Houston, TX, USA.
(28)Pt BD Sharma University of Health Sciences, Rohtak, India.
(29)Cape Heart Institute, Department of Medicine and Cardiology, Groote Schuur Hospital, University of Cape Town, Cape Town, South Africa.
(30)Department of Paediatrics, University of Melbourne, Melbourne, Victoria, Australia.
(31)Tropical Diseases Research Group, Murdoch Children's Research Institute, Melbourne, Victoria, Australia.
(32)Department of General Medicine, Royal Children's Hospital, Melbourne, Victoria, Australia.
(33)School of Medicine, National University of Samoa, Apia, Samoa.
(34)Women's and Children's Hospital, Adelaide, South Australia, Australia.
(35)Green Lane Paediatric and Congenital Cardiac Services, Starship Hospital, Te Whatu Ora, Auckland, New Zealand.
(36)South African Medical Research Council, Extramural Research & Internal Portfolio, Cape Town, South Africa.
(37)Division of Paediatric Cardiology, Department of Paediatrics and Child Health, University of Cape Town, Cape Town, South Africa.
(38)Department of Paediatrics, Royal Darwin Hospital, Darwin, Australia.
(#)Contributed equally
Rheumatic heart disease (RHD) is an important and preventable cause of morbidity and mortality among children and young adults in low-income and middle-income countries, as well as among certain at-risk populations living in high-income countries. The 2012 World Heart Federation echocardiographic criteria provided a

standardized approach for the identification of RHD and facilitated an improvement in early case detection. The 2012 criteria were used to define disease burden in numerous epidemiological studies, but researchers and clinicians have since highlighted limitations that have prompted a revision. In this updated version of the guidelines, we incorporate evidence from a scoping review, an expert panel and end-user feedback and present an approach for active case finding for RHD, including the use of screening and confirmatory criteria. These guidelines also introduce a new stage-based classification for RHD to identify the risk of disease progression. They describe the latest evidence and recommendations on population-based echocardiographic active case finding and risk stratification. Secondary antibiotic prophylaxis, echocardiography equipment and task sharing for RHD active case finding are also discussed. These World Heart Federation 2023 guidelines provide a concise and updated resource for clinical and research applications in RHD-endemic regions.

58. J Infect Dis. 2023 Nov 2;228(9):1198-1207. doi: 10.1093/infdis/jiad113.
Age Patterns of HSV-2 Incidence and Prevalence in Two Ugandan Communities: A Catalytic Incidence Model Applied to Population-Based Seroprevalence Data.
Whittles LK(1)(2), Galiwango RM(3), Mpagazi J(3), Tobian AAR(4)(5), Ssekubugu R(3), Jackson J(4), Peer AD(6), Kennedy C(3)(7), Nakalanzi M(3), Ndyanabo A(3), Kigozi G(3), Chang LW(3)(5)(7)(8), Serwadda D(3)(9), Manabe YC(8), Gaydos CA(8), Laeyendecker O(5)(8)(10), Quinn TC(3)(5)(8)(10), Reynolds SJ(3)(5)(8)(10), Kagaayi J(5)(9), Eaton JW(2), Grabowski MK(3)(4)(5).
Author information:
(1)Department of Infectious Disease Epidemiology, School of Public Health, Imperial College London, London, United Kingdom.
(2)Medical Research Council Centre for Global Infectious Disease Analysis, and NIHR Health Protection Research Unit in Modelling and Health Economics, School of Public Health, Imperial College London, London, United Kingdom.
(3)Rakai Health Sciences Program, Kalisizo, Uganda.
(4)Department of Pathology, Johns Hopkins School of Medicine, Baltimore, Maryland, USA.
(5)Department of Epidemiology, Johns Hopkins Bloomberg School of Public Health, Baltimore, Maryland, USA.
(6)Johns Hopkins School of Medicine, Baltimore, Maryland, USA.
(7)Department of International Health, Johns Hopkins Bloomberg School of Public Health, Baltimore, Maryland, USA.
(8)Department of Medicine, Johns Hopkins University, Baltimore, Maryland, USA.
(9)Department of Disease Control and Environmental Health, Makerere University School of Public Health, Kampala, Uganda.
(10)Laboratory of Immunoregulation, Division of Intramural Research, National Institute for Allergy and Infectious Diseases, National Institutes of Health, Bethesda, Maryland, USA.
BACKGROUND: Herpes simplex virus type 2 (HSV-2) is an incurable sexually transmitted infection associated with increased risk of acquiring and transmitting human immunodeficiency virus (HIV). HSV-2 is highly prevalent in sub-Saharan Africa, but population-level estimates of incidence are sparse.

METHODS: We measured HSV-2 prevalence from cross-sectional serological data among adults aged 18-49 years in 2 south-central Uganda communities (fishing, inland). We identified risk factors for seropositivity, then inferred age patterns of HSV-2 with a Bayesian catalytic model.
RESULTS: HSV-2 prevalence was 53.6% (n = 975/1819; 95% confidence interval, 51.3%-55.9%). Prevalence increased with age, was higher in the fishing community, and among women, reaching 93.6% (95% credible interval, 90.2%-96.6%) by age 49 years. Factors associated with HSV-2 seropositivity included more lifetime sexual partners, HIV positive status, and lower education. HSV-2 incidence peakied at age 18 years for women and 19-20 years for men. HIV prevalence was up to 10-fold higher in HSV-2-positive individuals.
CONCLUSIONS: HSV-2 prevalence and incidence were extremely high, with most infections occurring in late adolescence. Interventions against HSV-2, such as future vaccines or therapeutics, must target young populations. Remarkably higher HIV prevalence among HSV-2-positive individuals underscores this population as a priority for HIV prevention.

59. Microb Genom. 2023 Nov;9(11):001140. doi: 10.1099/mgen.0.001140.
Molecular epidemiology of Streptococcus agalactiae in non-pregnant populations: a systematic review.
Founou LL(1)(2)(3), Khan UB(4), Medugu N(5), Pinto TCA(6), Darboe S(7), Chendi Z(8), Founou RC(9)(3)(10), To KN(11), Jamrozy D(4), Karampatsas K(11), Carr VR(12)(4), Pepper K(4), Dangor Z(13), Ip M(8), Le Doare K(14), Bentley SD(15)(4).
Author information:
(1)Reproductive, Maternal, Newborn and Child Health (ReMARCH) Research Unit, Centre of Expertise and Biological Diagnostic of Cameroon Research Institute (CEDBCAM-RI), Yaoundé, Cameroon.
(2)Bioinformatics and Applied Machine Learning Research Unit, EDEN Biosciences Research Institute (EBRI), EDEN Foundation, Yaoundé, Cameroon.
(3)Antimicrobial Research Unit, School of Health Sciences, University of KwaZulu-Natal, Westville Campus, Durban, 4041, South Africa.
(4)Parasites and Microbes, Wellcome Sanger Institute, Wellcome Genome Campus, Hinxton CB10 1SA, UK.
(5)Department of Medical Microbiology and Parasitology, National Hospital Abuja, Abuja, Nigeria.
(6)Instituto de Microbiologia Paulo de Goes, Universidade Federal do Rio de Janeiro, Rio de Janeiro, Brazil.
(7)Medical Research Council Unit at London School of Hygiene and Tropical Medicine, Banjul, Gambia.
(8)Department of Microbiology, the Chinese University of Hong Kong, Hong Kong, PR China.
(9)Antibiotic Resistance Infectious Diseases (ARID) Research Unit, Centre of Expertise and Biological Diagnostic of Cameroon Research Institute (CEDBCAM-RI), Yaoundé, Cameroon.
(10)Department of Microbiology, Hematology and Immunology, Faculty of Medicine and Pharmaceutical Sciences, University of Dschang, Dschang, Cameroon.

(11)Institute of Infection and Immunity, St George's University of London, London, UK.
(12)Centre for Host-Microbiome Interactions, Faculty of Dentistry, Oral and Craniofacial Sciences, King's College London, London, SE1 9RT, UK.
(13)Vaccines and Infectious Diseases Analytics (VIDA) Research Unit, University of the Witwatersrand, Johannesburg, South Africa.
(14)Medical Research Council Uganda Virus Research Institute and London School of Hygiene and Tropical Medicine Uganda Research Unit, Entebbe, Uganda.
(15)Department of Pathology, University of Cambridge, Cambridge, UK.
Streptococcus agalactiae (group B Streptococcus, GBS) has recently emerged as an important pathogen among adults. However, it is overlooked in this population, with all global efforts being directed towards its containment among pregnant women and neonates. This systematic review assessed the molecular epidemiology and compared how the lineages circulating among non-pregnant populations relate to those of pregnant and neonatal populations worldwide. A systematic search was performed across nine databases from 1 January 2000 up to and including 20 September 2021, with no language restrictions. The Joanna Briggs Institute (JBI) Prevalence Critical Appraisal Tool (PCAT) was used to assess the quality of included studies. The global population structure of GBS from the non-pregnant population was analysed using in silico typing and phylogenetic reconstruction tools. Twenty-four articles out of 13 509 retrieved across 9 databases were eligible. Most studies were conducted in the World Health Organization European region (12/24, 50 %), followed by the Western Pacific region (6/24, 25 %) and the Americas region (6/24, 25 %). Serotype V (23%, 2310/10240) and clonal complex (CC) 1 (29 %, 2157/7470) were the most frequent serotype and CC, respectively. The pilus island PI1 : PI2A combination (29 %, 3931/13751) was the most prevalent surface protein gene, while the tetracycline resistance tetM (55 %, 5892/10624) was the leading antibiotic resistance gene. This study highlights that, given the common serotype distribution identified among non-pregnant populations (V, III, Ia, Ib, II and IV), vaccines including these six serotypes will provide broad coverage. The study indicates advanced molecular epidemiology studies, especially in resource-constrained settings for evidence-based decisions. Finally, the study shows that considering all at-risk populations in an inclusive approach is essential to ensure the sustainable containment of GBS.

60. Open Forum Infect Dis. 2023 Oct 12;10(11):ofad508. doi: 10.1093/ofid/ofad508.
Etiology of Fever and Associated Outcomes Among Adults Receiving Chemotherapy for the Treatment of Solid Tumors in Uganda.
Gulleen EA(1)(2), Holte S(1)(3), Zhang Y(1), Mbarusha I(4), Mubiru D(4), Pedun B(4), Keng M(5), Heysell SK(6), Omoding A(4), Moore CC(5), Phipps W(1)(2).
Author information:
(1)Vaccine and Infectious Diseases Division, Fred Hutchinson Cancer Center, Seattle, Washington, USA.
(2)Allergy and Infectious Diseases Division, Department of Medicine, University of Washington, Seattle, Washington, USA.
(3)Department of Global Health, University of Washington, Seattle, Washington,

USA.
(4)Uganda Cancer Institute, Kampala, Uganda.
(5)Division of Oncology, Department of Medicine, University of Virginia, Charlottesville, USA.
(6)Division of Infectious Diseases and International Health, Department of Medicine, University of Virginia, Charlottesville, Virginia, USA.
BACKGROUND: Little is known about the microbiology and outcomes of chemotherapy-associated febrile illness among patients in sub-Saharan Africa. Understanding the microbiology of febrile illness could improve antibiotic selection and infection-related outcomes.
METHODS: From September 2019 through June 2022, we prospectively enrolled adult inpatients at the Uganda Cancer Institute who had solid tumors and developed fever within 30 days of receiving chemotherapy. Evaluation included blood cultures, malaria rapid diagnostic tests, and urinary lipoarabinomannan testing for tuberculosis. Serum cryptococcal antigen was evaluated in participants with human immunodeficiency virus (HIV). The primary outcome was the mortality rate 40 days after fever onset, which we estimated using Cox proportional hazards models.
RESULTS: A total of 104 febrile episodes occurred among 99 participants. Thirty febrile episodes (29%) had ≥1 positive microbiologic result. The most frequently identified causes of infection were tuberculosis (19%) and bacteremia (12%). The prevalence of tuberculosis did not differ by HIV status. The 40-day case fatality ratio was 25%. There was no difference in all-cause mortality based on HIV serostatus, presence of neutropenia, or positive microbiologic results. A universal vital assessment score of >4 was associated with all-cause mortality (hazard ratio, 14.5 [95% confidence interval, 5-42.7]).
CONCLUSIONS: The 40-day mortality rate among Ugandan patients with solid tumors who developed chemotherapy-associated febrile illness was high, and few had an identified source of infection. Tuberculosis and bacterial bloodstream infections were the leading diagnoses associated with fever. Tuberculosis should be included in the differential diagnosis for patients who develop fever after receiving chemotherapy in tuberculosis-endemic settings, regardless of HIV serostatus.

61. Int J Pediatr Otorhinolaryngol. 2023 Nov;174:111741. doi: 10.1016/j.ijporl.2023.111741. Epub 2023 Sep 29.
ISOM 2023 research Panel 4 - Diagnostics and microbiology of otitis media.
Tamir SO(1), Bialasiewicz S(2), Brennan-Jones CG(3), Der C(4), Kariv L(5), Macharia I(6), Marsh RL(7), Seguya A(8), Thornton R(9).
Author information:
(1)Department of Otolaryngology-Head and Neck Surgery, Sasmon Assuta Ashdod University Hospital, Faculty of Health Sciences, Ben Gurion University of the Negev, Israel. Electronic address: sharono@assuta.co.il.
(2)Australian Centre for Ecogenomics, School of Chemistry and Molecular Biosciences, The University of Queensland, St Lucia, QLD, 4072, Australia.
(3)Wesfarmers Centre of Vaccines and Infectious Diseases, Telethon Kids Institute, The University of Western Australia, Perth, Western Australia,

Australia; Faculty of Health Sciences, Curtin University, Perth, Western Australia, Australia.
(4)Facultad de Medicina, Universidad Del Desarrollo, Dr Luis Calvo Mackenna Hospital, Santiago, Chile.
(5)Hearing, Speech and Language Institute, Sasmon Assuta Ashdod University Hospital, Israel.
(6)Kenyatta University Teaching, Referral & Research Hospital, Kenya.
(7)Menzies School of Health Research, Darwin, Australia; School of Health Sciences, University of Tasmania, Launceston, Australia.
(8)Department of Otolaryngology - Head and Neck Surgery, Mulago National Referral Hospital, Kampala, Uganda.
(9)Wesfarmers Centre of Vaccines and Infectious Diseases, Telethon Kids Institute, The University of Western Australia, Perth, Western Australia, Australia; Centre for Child Health Research, University of Western Australia, Perth, Australia.
OBJECTIVES: To identify and review key research advances from the literature published between 2019 and 2023 on the diagnosis and microbiology of otitis media (OM) including acute otitis media (AOM), recurrent AOM (rAOM), otitis media with effusion (OME), chronic suppurative otitis media (CSOM) and AOM complications (mastoiditis).
DATA SOURCES: PubMed database of the National Library of Medicine.
REVIEW METHODS: All relevant original articles published in Medline in English between July 2019 and February 2023 were identified. Studies that were reviews, case studies, relating to OM complications (other than mastoiditis), and studies focusing on guideline adherence, and consensus statements were excluded. Members of the panel drafted the report based on these search results.
MAIN FINDINGS: For the diagnosis section, 2294 unique records screened, 55 were eligible for inclusion. For the microbiology section 705 unique records were screened and 137 articles were eligible for inclusion. The main themes that arose in OM diagnosis were the need to incorporate multiple modalities including video-otoscopy, tympanometry, telemedicine and artificial intelligence for accurate diagnoses in all diagnostic settings. Further to this, was the use of new, cheap, readily available tools which may improve access in rural and lowmiddle income (LMIC) settings. For OM aetiology, PCR remains the most sensitive method for detecting middle ear pathogens with microbiome analysis still largely restricted to research use. The global pandemic response reduced rates of OM in children, but post-pandemic shifts should be monitored.
IMPLICATION FOR PRACTICE AND FUTURE RESEARCH: Cheap, easy to use multi-technique assessments combined with artificial intelligence and/or telemedicine should be integrated into future practice to improve diagnosis and treatment pathways in OM diagnosis. Longitudinal studies investigating the in-vivo process of OM development, timings and in-depth interactions between the triad of bacteria, viruses and the host immune response are still required. Standardized methods of collection and analysis for microbiome studies to enable inter-study comparisons are required. There is a need to target underlying biofilms if going to effectively prevent rAOM and OME and possibly enhance ventilation tube retention.

62. Am J Trop Med Hyg. 2023 Sep 25;109(5):985-988. doi: 10.4269/ajtmh.23-0331. Print 2023 Nov 1.
Plague Gives Surprises in the Second Decade of the Twenty-First Century.
Butler T(1).
Author information:
(1)Ross University School of Medicine, Bridgetown, Barbados, West Indies, Retired.
From 2010 through 2019, the six leading countries by numbers of human plague cases reported to the WHO were, in order from highest to lowest, Madagascar, Congo, Uganda, Peru, Tanzania, and the United States. From these countries, there was a total of 4,547 cases, of whom 786 (17%) died. Top plague events were four outbreaks of primary pneumonic plague in Madagascar that affected 1,936 persons, including index cases, of whom 137 died. One of the outbreaks was caused by a streptomycin-resistant strain of Yersinia pestis. Person-to-person transmission occurred in a taxi, in households with family caregivers, at burial ceremonies and wakes for victims, and at a hospital where cases were treated. Unique clinical presentations in the United States included a dog owner who acquired pneumonic plague from his sick dog, a boy with septicemic plague who developed complications of osteomyelitis and arthritis that required surgery for bone removal and bone grafting, and a prairie dog handler who acquired bubonic plague from a bite by a sick prairie dog. Efficacy of antibiotics in a model of pneumonic plague in African green monkeys for use in bioterrorism revealed the most effective drugs to be gentamicin, ciprofloxacin, and levofloxacin. A recombinant vaccine containing Fraction 1 antigen and V antigen of Y. pestis designed for first responders during a bioterrorism attack and military personnel was tested for safety and immunogenicity but was not licensed for use by the end of the decade.

63. Lancet Respir Med. 2023 Nov;11(11):975-990. doi: 10.1016/S2213-2600(23)00263-1.
Efficacy of a bivalent (D614 + B.1.351) SARS-CoV-2 recombinant protein vaccine with AS03 adjuvant in adults: a phase 3, parallel, randomised, modified double-blind, placebo-controlled trial.
Dayan GH(1), Rouphael N(2), Walsh SR(3), Chen A(4), Grunenberg N(5), Allen M(6), Antony J(7), Asante KP(8), Bhate AS(9), Beresnev T(6), Bonaparte MI(4), Celle M(10), Ceregido MA(11), Corey L(5), Dobrianskyi D(12), Fu B(6), Grillet MH(13), Keshtkar-Jahromi M(14), Juraska M(5), Kee JJ(5), Kibuuka H(15), Koutsoukos M(11), Masotti R(4), Michael NL(16), Neuzil KM(17), Reynales H(18), Robb ML(19), Villagómez Martínez SM(20), Sawe F(21), Schuerman L(11), Tong T(6), Treanor J(22), Wartel TA(23), Diazgranados CA(4), Chicz RM(24), Gurunathan S(4), Savarino S(4), Sridhar S(25); VAT00008 Study Team.
Collaborators: Abalos K, Accini J, Aloysia N, Amuasi JH, Ansah NA, Benkeser D, Berge A, Beyko H, Bilotkach O, Breuer T, Bonfanti AC, Bukusi E, Canter R, Carrillo JA, Chansinghakul D, Coux F, Das C, Das SK, Devlin L, Espinoza L, Fay M, Follmann D, Frago C, Garinga A, Gilbert PB, Gonzalez C, Granados MA, Guillery L, Huang Y, Hudzina K, Jain M, Kanodia P, Khandelwal N, Mutuluuza CK, Kiweewa F, Kiwanuka N, Kosolsak C, Kukian D, Kushwaha JS, Laot T, Lopez-Medina E, Macareno

Arroyo H, Mandaliya K, Mamod S, Mangarule S, Martínez J, McClelland S, Menard L, Mendoza S, Mohapatra S, Moreau C, Mugo N, Nduba V, Noriega F, Ntege PN, Okech B, Otero M, Ouma SG, Oyieko J, Paredes M, Pardo E, Postol S, Pekala D, Peng P, Py ML, Rivas E, Rivero R, Rodriguez E, Saleh M, Sánchez P, Sater N, Shah J, Shrestha R, Siika A, Singh C, Singh VB, Tamrakar D, Tavares Da-Silva F, Otieno Tina L, Velasquez H, Wabwire D, Wajja A, Zaworski E, Zhang N.
Author information:
(1)Sanofi, Swiftwater, PA, USA. Electronic address: gustavo.dayan@sanofi.com.
(2)Emory University Hope Clinic, Atlanta, GA, USA.
(3)Harvard Medical School, Boston, MA, USA.
(4)Sanofi, Swiftwater, PA, USA.
(5)Fred Hutchinson Cancer Center, Seattle, WA, USA.
(6)National Institute of Allergy and Infectious Diseases / National Institutes of Health, Bethesda, MD, USA.
(7)Sanofi, Frankfurt, Germany.
(8)Research and Development Division, Ghana Health Service, Kintampo North Municipality, Ghana.
(9)Jeevan Rekha Hospital, Belgavi, India.
(10)Sanofi, Marcy l'Etoile, France.
(11)GlaxoSmithKline, Wavre, Belgium.
(12)Medical Centre, Medical Clinic Blagomed, Kyiv, Ukraine.
(13)Sanofi, Lyon, France.
(14)National Institutes of Health, Rockville, MD, USA; John Hopkins University School of Medicine, Baltimore, MD, USA.
(15)Makerere University Walter Reed Project, Kampala, Uganda.
(16)Walter Reed Army Institute of Research, Silver Spring, MD, USA.
(17)University of Maryland School of Medicine, Baltimore, MD, USA.
(18)Centro de Attencion e Investigation Medica S.A.S. - Caimed Chía, Chía, Colombia.
(19)The Henry M Jackson Foundation for the Advancement of Military Medicine, Bethesda, MA, USA.
(20)Hospital de Temixco en acuerdo con el Instituto Nacional de Pediatria, Temixco, México.
(21)Kenya Medical Research Institute - US Army Medical Research, Kericho, Kenya.
(22)Tunnell Government Services in support of Biomedical Advanced Research and Development Authority, Administration for Strategic Preparedness and Response, Department of Health and Human Services, Washington, DC, USA.
(23)International Vaccine Institute, Seoul, South Korea.
(24)Sanofi, Cambridge, MA, USA.
(25)Sanofi, Reading, UK.
BACKGROUND: COVID-19 vaccines with alternative strain compositions are needed to provide broad protection against newly emergent SARS-CoV-2 variants of concern. This study aimed to describe the clinical efficacy and safety of a bivalent SARS-CoV-2 recombinant protein vaccine as a two-injection primary series during a period of circulation of the omicron (B.1.1.529) variant.
METHODS: We conducted a phase 3, parallel, randomised, modified double-blind, placebo-controlled trial in adults aged 18 years or older at 54 clinical

research centres in eight countries (Colombia, Ghana, India, Kenya, Mexico, Nepal, Uganda, and Ukraine). Participants were recruited from the community and randomly assigned (1:1) by use of an interactive response technology system to receive two intramuscular 0·5 mL injections, 21 days apart, of the bivalent vaccine (5 µg of ancestral [D614] and 5 µg of beta [B.1.351] variant spike protein, with AS03 adjuvant) or placebo (0·9% normal saline). All participants, outcome assessors, and laboratory staff performing assays were masked to group assignments; those involved in the preparation and administration of the vaccines were unmasked. Participants were stratified by age (18-59 years and ≥60 years) and baseline SARS-CoV-2 rapid serodiagnostic test positivity. Symptomatic COVID-19 was defined as laboratory-confirmed (via nucleic acid amplification test or PCR test) COVID-19 with COVID-19-like illness symptoms. The primary efficacy endpoint was the clinical efficacy of the bivalent vaccine for prevention of symptomatic COVID-19 at least 14 days after the second injection (dose 2). Safety was assessed in all participants receiving at least one injection of the study vaccine or placebo. This trial is registered with ClinicalTrials.gov (NCT04904549) and is closed to recruitment.
FINDINGS: Between Oct 19, 2021, and Feb 15, 2022, 13 002 participants were enrolled and randomly assigned to receive the first dose of the study vaccine (n=6512) or placebo (n=6490). 12 924 participants (6472 in the vaccine group and 6452 in the placebo group) received at least one study injection, of whom 7542 (58·4%) were male and 9693 (75·0%) were SARS-CoV-2 non-naive. Of these 12 924 participants, 11 543 (89·3%) received both study injections (5788 in the vaccine group and 5755 in the placebo group). The efficacy-evaluable population after dose 2 comprised 11 416 participants (5736 in the vaccine group and 5680 in the placebo group). The median duration of follow-up was 85 days (IQR 50-95) after dose 1 and 58 days (29-70) after dose 2. 121 symptomatic COVID-19 cases were reported at least 14 days after dose 2 (32 in the vaccine group and 89 in the placebo group), with an overall vaccine efficacy of 64·7% (95% CI 46·6 to 77·2). Vaccine efficacy against symptomatic COVID-19 was 75·1% (95% CI 56·3 to 86·6) in SARS-CoV-2 non-naive participants and 30·9% (-39·3 to 66·7) in SARS-CoV-2-naive participants. Viral genome sequencing identified the infecting strain in 68 (56·2%) of 121 cases (omicron [BA.1 and BA.2] in 63; delta in four; and both omicron and delta in one). Immediate unsolicited adverse events were reported by four (<0·1%) participants in the vaccine group and seven (0·1%) participants in the placebo group. Immediate unsolicited adverse reactions within 30 min after any injection were reported by four (<0·1%) participants in the vaccine group and six (<0·1%) participants in the placebo group. In the reactogenicity subset with available data, solicited reactions (solicited injection-site reactions and solicited systemic reactions) within 7 days after any injection occurred in 1398 (57·8%) of 2420 vaccine recipients and 983 (40·9%) of 2403 placebo recipients. Grade 3 solicited reactions were reported by 196 (8·1%; 95% CI 7·0 to 9·3) of 2420 vaccine recipients and 118 (4·9%; 4·1 to 5·9) of 2403 placebo recipients within 7 days after any injection, with comparable frequencies after dose 1 and dose 2 in the vaccine group. At least one serious adverse event occurred in 30 (0·5%) participants in the vaccine group and 26 (0·4%) in the placebo group. The proportion of adverse events of special interest and deaths was less than 0·1%

in both study groups. No adverse event of special interest, serious adverse event, or death was deemed to be treatment related. There were no reported cases of thrombosis with thrombocytopenia syndrome, myocarditis, pericarditis, Bell's Palsy, or Guillain-Barré syndrome, or other immune-mediated diseases.
INTERPRETATION: The bivalent variant vaccine conferred heterologous protection against symptomatic SARS-CoV-2 infection in the epidemiological context of the circulating contemporary omicron variant. These findings suggest that vaccines developed with an antigen from a non-predominant strain could confer cross-protection against newly emergent SARS-CoV-2 variants, although further investigation is warranted.

64. Int J Infect Dis. 2023 Nov;136:49-56. doi: 10.1016/j.ijid.2023.09.001.
Spike protein is a key target for stronger and more persistent T-cell responses-a study of mild and asymptomatic SARS-CoV-2 infection.
Ssali I(1), Mugaba S(1), Watelo AK(2), Bemanzi J(2), Katende JS(3), Oluka GK(3), Ankunda V(1), Baine C(2), Kato L(1); COVID-19 Immunoprofiling Team(3); Onyachi N(4), Muwanga M(5), Jjuuko M(4), Kayiwa J(2), Nsereko C(5), Auma BO(1), Weiskopf D(6), Sette A(7), Lutalo T(2), Musenero M(8), Kaleebu P(3), Serwanga J(9).
Author information:
(1)Pathogen Genomics, Phenotype, and Immunity Program, Medical Research Council, Uganda Virus Research Institute and London School of Hygiene and Tropical Medicine, Uganda Research Unit, Entebbe, Uganda.
(2)Department of Immunology, Uganda Virus Research Institute, Entebbe, Uganda.
(3)Pathogen Genomics, Phenotype, and Immunity Program, Medical Research Council, Uganda Virus Research Institute and London School of Hygiene and Tropical Medicine, Uganda Research Unit, Entebbe, Uganda; Department of Immunology, Uganda Virus Research Institute, Entebbe, Uganda.
(4)Department of Internal Medicine, Masaka Regional Referral Hospital, Masaka, Uganda.
(5)Department of Internal Medicine, Entebbe Regional Referral Hospital, Entebbe, Uganda.
(6)Center for Infectious Disease and Vaccine Research, La Jolla Institute for Immunology, La Jolla, USA.
(7)Center for Infectious Disease and Vaccine Research, La Jolla Institute for Immunology, La Jolla, USA; Department of Medicine, Division of Infectious Diseases and Global Public Health, University of California, San Diego (UCSD), La Jolla, USA.
(8)Science, Technology, and Innovation Secretariat, Office of the President, Government of Uganda, Kampala, Uganda.
(9)Pathogen Genomics, Phenotype, and Immunity Program, Medical Research Council, Uganda Virus Research Institute and London School of Hygiene and Tropical Medicine, Uganda Research Unit, Entebbe, Uganda; Department of Immunology, Uganda Virus Research Institute, Entebbe, Uganda. Electronic address: Jennifer.Serwanga@mrcuganda.org.
OBJECTIVES: Understanding the immune response in very mild and asymptomatic COVID-19 is crucial for developing effective vaccines and immunotherapies, yet remains poorly characterized. This longitudinal study examined the evolution of

interferon (IFN)-γ responses to SARS-CoV-2 peptides in 109 asymptomatic or mildly symptomatic Ugandan COVID-19 patients across 365 days and explored their association with antibody generation.
METHODS: T-cell responses to spike-containing clusters of differentiation (CD4)-S and CD8 nCoV-A (CD8-A) megapools, and the non-spike CD4-R and CD8 nCoV-B (CD8-B) megapools, were assessed and correlated with demographic and temporal variables.
RESULTS: SARS-CoV-2-specific IFN-γ responses were consistently detected in all peptide pools and time points, with the spike-targeted response exhibiting higher potency and durability than the non-spike responses. Throughout the entire 365-day infection timeline, a robust positive correlation was observed between CD4 T-cell responses to the spike-derived peptides and anti-spike immunoglobulin G antibody levels, underscoring their interdependent dynamics in the immune response against SARS-CoV-2; in contrast, CD8 T-cell responses exhibited no such correlation, highlighting their distinctive, autonomous role in defense. No meaningful variations in complete blood count parameters were observed between individuals with COVID-19 infection and those without, indicating clinical insignificance.
CONCLUSIONS: This study highlights the dominant role of spike-directed T-cell responses in mild and asymptomatic disease and provides crucial longitudinal data from Sub-Saharan African settings. The findings provide valuable insights into the dynamics of T-cell responses and their potential significance in developing effective strategies for combating COVID-19.

65. J Health Commun. 2023 Oct 31;28(sup2):49-60. doi: 10.1080/10810730.2023.2231377.
Co-Designing a Mobile-Based Game to Improve Misinformation Resistance and Vaccine Knowledge in Uganda, Kenya, and Rwanda.
Hopkins KL(1), Lepage C(2), Cook W(3), Thomson A(2)(4), Abeyesekera S(5), Knobler S(1), Boehman N(1), Thompson B(1), Waiswa P(6), Ssanyu JN(6), Kabwijamu L(6), Wamalwa B(7), Aura C(7), Rukundo JC(8), Cook J(9).
Author information:
(1)Vaccine Acceptance & Demand Initiative, Global Immunization, Sabin Vaccine Institute, Washington DC, USA.
(2)Irimi Company, Lyon, France.
(3)Wendy Cook Design, Melbourne, Australia.
(4)School of Liberal Arts, Indiana University-Purdue University, Indianapolis, Indiana, USA.
(5)Immunization Demand Team, UNICEF, New York, USA.
(6)School of Public Health, Makerere University, Kampala, Uganda.
(7)Department of Chemistry, University of Nairobi, Nairobi, Kenya.
(8)Social and Behavior Change Program, UNICEF Country Office, Kigali, Rwanda.
(9)Melbourne Centre for Behaviour Change, University of Melbourne, Melbourne, VIC, Australia.
Misinformation can decrease public confidence in vaccines, and reduce vaccination intent and uptake. One strategy for countering these negative impacts comes from inoculation theory. Similar to biological vaccination, inoculation theory posits that exposure to a weakened form of misinformation can

develop cognitive immunity, reducing the likelihood of being misled. Online games offer an interactive, technology-driven, and scalable solution using an active form of inoculation that engages and incentivizes players to build resilience against misinformation. We document the development of the critical thinking game Cranky Uncle Vaccine. The game applies research findings from inoculation theory, critical thinking, humor in science communication, and serious games. The game content was iterated through a series of co-design workshops in Kampala (Uganda), Kitale (Kenya), and Kigali (Rwanda). Workshop participants offered feedback on cartoon character design, gameplay experience, and the game's content, helping to make the game more culturally relevant and avoid unintended consequences in East African countries. Our co-design methodology offers an approach for further adaptation of the Cranky Uncle Vaccine game to other regions, as well as a template for developing locally relevant interventions to counter future infodemics.

66. Immunother Adv. 2023 Oct 31;3(1):ltad024. doi: 10.1093/immadv/ltad024.
Human infection challenge in the pandemic era and beyond, HIC-Vac annual meeting report, 2022.
Barnes MVC(1), Mandla A(1), Smith E(2), Maskuniitty M(1), Openshaw PJM(1); HIC-Vac meeting contributors.
Collaborators: Abo YN(3), Ascough S(4), Ashwin H(5), Avirutnan P(6), Catchpole AP(7), Chi PC(8), Chiu C(4), Darton TC(9), Driciru E(10), Dula D(11), Ferreira DM(12), Fraser A(13), Garrett P(13), Gbesemete D(14), Gordon S(11), Heymann DL(15), Houlder E(16), Jamrozik E(17), Kapulu M(8), Kessy E(18), Overgaard Kildemoes AM(16), Koopman JP(16), McShane H(17), Navanukroh O(6), Osier FH(4), Osowicki J(19), Pham V(20), Pollard AJ(17), Ratta-Apha W(6), Silk SE(17), Sridhar S(21), Swanson KA(22), Talaat KR(23), Thwaites RS(4), Welch O(13).
Author information:
(1)National Heart and Lung Institute, Imperial College London, London, UK.
(2)Department of Infectious Disease, Imperial College London, London, UK.
(3)Murdoch Children's Research Institute, the Royal Children's Hospital Melbourne, Australia.
(4)Imperial College London, UK.
(5)University of York, UK.
(6)Mahidol University, Thailand.
(7)hVIVO, UK.
(8)KEMRI Wellcome Research Programme, Kenya.
(9)University of Sheffield, UK.
(10)CHI-S-Uganda, Leiden University Medical Center, the Netherlands.
(11)Malawi-Liverpool-Wellcome Trust Clinical Research Programme, Malawi.
(12)University of Oxford, UK, and Liverpool School of Tropical Medicine, UK.
(13)1Day Sooner, UK.
(14)University of Southampton, UK.
(15)London School of Hygiene and Tropical Medicine, UK.
(16)Leiden University Medical Center, the Netherlands.
(17)University of Oxford, UK.
(18)Ifakara Health Institute, Tanzania.

(19)Murdoch Children's Research Institute, the Royal Children's Hospital Melbourne, and the University of Melbourne, Australia.
(20)Oxford University Clinical Research Unit, Vietnam.
(21)Sanofi Pasteur and Inno4Vac, UK.
(22)Pfizer, USA.
(23)Johns Hopkins University, USA.
HIC-Vac is an international network of researchers dedicated to developing human infection challenge studies to accelerate vaccine development against pathogens of high global impact. The HIC-Vac Annual Meeting (3rd and 4th November 2022) brought together stakeholders including researchers, ethicists, volunteers, policymakers, industry partners, and funders with a strong representation from low- and middle-income countries. The network enables sharing of research findings, especially in endemic regions. Discussions included pandemic preparedness and the role of human challenge to accelerate vaccine development during outbreak, with industry speakers emphasising the great utility of human challenge in vaccine development. Public consent, engagement, and participation in human challenge studies were addressed, along with the role of embedded social science and empirical studies to uncover social, ethical, and regulatory issues around human infection challenge studies. Study volunteers shared their experiences and motivations for participating in studies. This report summarises completed and ongoing human challenge studies across a variety of pathogens and demographics, and addresses other key issues discussed at the meeting.

67. Cell Rep. 2023 Oct 31;42(10):113254. doi: 10.1016/j.celrep.2023.113254.
Blocking of ebolavirus spread through intercellular connections by an MPER-specific antibody depends on BST2/tetherin.
Santos RI(1), Ilinykh PA(1), Pietzsch CA(1), Ronk AJ(1), Huang K(1), Kuzmina NA(1), Zhou F(1), Crowe JE(2), Bukreyev A(3).
Author information:
(1)Department of Pathology, University of Texas Medical Branch, Galveston, TX, USA; Galveston National Laboratory, Galveston, TX, USA.
(2)Vanderbilt Vaccine Center, Vanderbilt University Medical Center, Nashville, TN, USA; Department of Pathology, Microbiology and Immunology, Vanderbilt University Medical Center, Nashville, TN, USA; Department of Pediatrics, Vanderbilt University Medical Center, Nashville, TN, USA. Electronic address: james.crowe@vumc.org.
(3)Department of Pathology, University of Texas Medical Branch, Galveston, TX, USA; Galveston National Laboratory, Galveston, TX, USA; Department of Microbiology and Immunology, University of Texas Medical Branch, Galveston, TX, USA. Electronic address: alexander.bukreyev@utmb.edu.
Ebola virus (EBOV) and Bundibugyo virus (BDBV) belong to the family Filoviridae and cause a severe disease in humans. We previously isolated a large panel of monoclonal antibodies from B cells of human survivors from the 2007 Uganda BDBV outbreak, 16 survivors from the 2014 EBOV outbreak in the Democratic Republic of the Congo, and one survivor from the West African 2013-2016 EBOV epidemic. Here, we demonstrate that EBOV and BDBV are capable of spreading to neighboring cells through intercellular connections in a process that depends upon actin and

T cell immunoglobulin and mucin 1 protein. We quantify spread through intercellular connections by immunofluorescence microscopy and flow cytometry. One of the antibodies, BDBV223, specific to the membrane-proximal external region, induces virus accumulation at the plasma membrane. The inhibiting activity of BDBV223 depends on BST2/tetherin.

68. Sci Rep. 2023 Oct 30;13(1):18619. doi: 10.1038/s41598-023-44518-4.
Identifying target areas for risk-based surveillance and control of transboundary animal diseases: a seasonal analysis of slaughter and live-trade cattle movements in Uganda.
González-Gordon L(1)(2), Porphyre T(3), Muwonge A(4)(5), Nantima N(6), Ademun R(6), Ochwo S(7), Mwiine NF(8), Boden L(9), Muhanguzi D(8), Bronsvoort BMC(4).
Author information:
(1)The Epidemiology, Economics and Risk Assessment (EERA) Group, The Roslin Institute at The Royal (Dick) School of Veterinary Studies, University of Edinburgh, Easter Bush, Midlothian, EH25 9RG, UK. lina.gonzalez@ed.ac.uk.
(2)Global Academy of Agriculture and Food Systems, Royal (Dick) School of Veterinary Studies and The Roslin Institute, University of Edinburgh, Easter Bush, Midlothian, EH25 9RG, UK. lina.gonzalez@ed.ac.uk.
(3)Laboratoire de Biométrie et Biologie Évolutive, UMR 5558, Universite Claude Bernard Lyon 1, CNRS, VetAgro Sup, Marcy l'Étoile, France.
(4)The Epidemiology, Economics and Risk Assessment (EERA) Group, The Roslin Institute at The Royal (Dick) School of Veterinary Studies, University of Edinburgh, Easter Bush, Midlothian, EH25 9RG, UK.
(5)The Digital One Health Laboratory, The Roslin Institute at The Royal (Dick) School of Veterinary Studies, University of Edinburgh, Easter Bush, Midlothian, EH25 9RG, UK.
(6)Department of Animal Health, Ministry of Agriculture Animal Industry and Fisheries, Entebbe, Uganda.
(7)Center for Animal Health and Food Safety, College of Veterinary Medicine, University of Minnesota, Saint Paul, MN, 55108, USA.
(8)Department of Biomolecular Resources and Biolaboratory Sciences (BBS), College of Veterinary Medicine, Makerere University, Kampala, Uganda.
(9)Global Academy of Agriculture and Food Systems, Royal (Dick) School of Veterinary Studies and The Roslin Institute, University of Edinburgh, Easter Bush, Midlothian, EH25 9RG, UK.
Animal movements are a major driver for the spread of Transboundary Animal Diseases (TADs). These movements link populations that would otherwise be isolated and hence create opportunities for susceptible and infected individuals to meet. We used social network analysis to describe the seasonal network structure of cattle movements in Uganda and unravel critical network features that identify districts or sub-regions for targeted risk-based surveillance and intervention. We constructed weighted, directed networks based on 2019 between-district cattle movements using official livestock mobility data; the purpose of the movement ('slaughter' vs. 'live trade') was used to subset the network and capture the risks more reliably. Our results show that cattle trade can result in local and long-distance disease spread in Uganda. Seasonal

variability appears to impact the structure of the network, with high heterogeneity of node and edge activity identified throughout the seasons. These observations mean that the structure of the live trade network can be exploited to target influential district hubs within the cattle corridor and peripheral areas in the south and west, which would result in rapid network fragmentation, reducing the contact structure-related trade risks. Similar exploitable features were observed for the slaughter network, where cattle traffic serves mainly slaughter hubs close to urban centres along the cattle corridor. Critically, analyses that target the complex livestock supply value chain offer a unique framework for understanding and quantifying risks for TADs such as Foot-and-Mouth disease in a land-locked country like Uganda. These findings can be used to inform the development of risk-based surveillance strategies and decision making on resource allocation. For instance, vaccine deployment, biosecurity enforcement and capacity building for stakeholders at the local community and across animal health services with the potential to limit the socio-economic impact of outbreaks, or indeed reduce their frequency.

69. Glob Health Sci Pract. 2023 Oct 30;11(5):e2300121. doi: 10.9745/GHSP-D-23-00121.
Supporting the Manufacturing of Medical Supplies in Africa: Collaboration Between Africa CDC, Partners, and Member States.
Mohammed A(1), Idris-Dantata H(2), Okwor T(3), Tanui P(4), Paintsil E(5), Kabwe PC(1), Alimi Y(1), Tajudeen R(1), Mankoula W(1), Ilesanmi OS(6), Balogun MS(7), Ihekweazu C(3), Hornsey E(8), Ogbuagu O(5).
Author information:
(1)Africa Centres for Disease Control and Prevention, Addis Ababa, Ethiopia.
(2)Health Maxima, LLC, Gaithersburg, MD, USA.
(3)Nigeria Centres for Disease Control and Prevention, Abuja, Nigeria.
(4)African Union Development Agency-NEPAD, Midrand, South Africa.
(5)Yale School of Medicine, Yale University, New Haven, CT, USA.
(6)Africa Centres for Disease Control and Prevention, Addis Ababa, Ethiopia. ileolasteve@yahoo.co.uk.
(7)African Field Epidemiology Network, Kampala, Uganda.
(8)UK Health Security Agency, London School of Hygiene and Tropical Medicine, London, United Kingdom.
Inadequate supply of PPE, vaccines, and diagnostics during the COVID-19 pandemic in Africa created an opportunity to promote local manufacturing. Authors describe Africa CDC's contributions and highlight strategies for strengthening the pandemic response.
Inadequate supply of personal protective equipment, vaccines, diagnostics, and therapeutics during the COVID-19 pandemic in Africa created an opportunity to promote local manufacturing. South Africa, Morocco, Tunisia, Egypt, and Senegal are currently involved in local vaccine production that meets approximately 1% of the total continental vaccine demand and should rank topmost in capacity-building for increased production. Four concepts—cooperation, collaboration, coordination, and communication—are underlined in the Africa Centres for Disease Control and Prevention's strategy to promote the procurement of large supplies of diagnostics, therapeutics, and other essential supplies. By

investing in existing manufacturers, challenges in market access can be overcome. Local manufacturers in South Africa, Morocco, Tunisia, Egypt, and Senegal are able to sell to Gavi, the Vaccine Alliance-supported countries and can achieve volume certainty at scale. Beyond manufacturing facilities, specialized financing is needed to improve larger enablers such as expertise for manufacturing plants and research institutions, legal frameworks for vaccine approval, and research and development, particularly for novel vaccines.

70. BMC Vet Res. 2023 Oct 28;19(1):224. doi: 10.1186/s12917-023-03788-w.
Spatio-temporal analysis of sheep and goat pox outbreaks in Uganda during 2011-2022.
Nizeyimana G(1), Vudriko P(1), Erume J(2), Mubiru F(3), Eneku W(1), Biryomumaisho S(1), Mwebe R(4), Arinaitwe E(4), Ademun R(4), Atim S(4), Ayebazibwe C(3), Muhanguzi D(2), Tweyongyere R(5).
Author information:
(1)Department of Pharmacy Clinical and Comparative Medicine, School of Veterinary Medicine and Animal Resources, College of Veterinary Medicine Animal Resources and Biosecurity, Makerere University, Kampala, Uganda.
(2)Department of Bimolecular and Bio Lab Sciences, School of Biosecurity, Biotechnology and Laboratory Sciences, College of Veterinary Medicine Animal Resources and Biosecurity, Makerere University, Kampala, Uganda.
(3)Emergency Centre for Transboundary Animal Diseases, Food and Agriculture Organisation of the United Nations, Kampala, Uganda.
(4)National Animal Disease Diagnostics and Epidemiology Centre, Directorate of Animal Resources, Ministry of Agriculture, Animal Industry and Fisheries, Entebbe, Uganda.
(5)Department of Pharmacy Clinical and Comparative Medicine, School of Veterinary Medicine and Animal Resources, College of Veterinary Medicine Animal Resources and Biosecurity, Makerere University, Kampala, Uganda. robert.tweyongyere@mak.ac.ug.
BACKGROUND: Sheep and goat pox (SGP) caused by sheep poxvirus (SPV) and goat poxvirus (GPV) respectively; are transboundary and World Organisation for Animal Health (WOAH)-notifiable viral diseases. There is barely any coherent information about the distribution and prevalence of SGP for Uganda. We therefore conducted this study to describe the temporal and spatial distribution of SGP suspected outbreaks in Uganda for the period 2011-2020 as well as serologically confirm presence of SGP antibodies in suspected SGP outbreaks reported in 2021-2022.
RESULTS: Thirty-seven [37] SGP outbreaks were reported across the country during the study period. North-eastern region [that comprises of Karamoja region] had the highest number of outbreaks [n = 17, 45%]; followed by Central [n = 9, 2.4%], Northern [n = 8, 2.2%] and Western region [n = 3, 0.08%]. Reports from district veterinary personnel indicate that the prevalence of; and mortality rate and case fatality rate associated with SGP were 0.06%, 0.02% and 32% respectively. There was a steady increase in the number of reported SGP outbreaks [$\bar{x}$ = 4] over the study period. Seropositivity of SGPV antibodies in outbreak sheep and goats that were investigated during the study period

[2021-2022] was [n = 41, 27%, 95 CI;] CONCLUSION: Our analyses of SGPV passive and active reports indicate that SGP is present in Uganda with a decade long average of four outbreaks per annum. During this period, about a third of all SGPV-clinically infected animals died. SPG is therefore a major constraint to small ruminant health and productivity in Uganda. Introduction of animals from infected herds and breach in farm biosecurity were the most important predictors of SGP outbreaks. In addition to the already existing SGP commercial vaccines, small ruminant screening for SGPV before introducing them to naïve herds and ensuring on farm biosecurity should be part of the SGP control tool pack for Ugandan small ruminant farmers.

71. J Public Health Afr. 2023 Oct 24;14(9):2735. doi: 10.4081/jphia.2023.2735.
Continental concerted efforts to control the seventh outbreak of Ebola Virus disease in Uganda: The first 90 days of the response.
Aceng JR(1), Bosa HK(1), Kamara N(2), Atwine D(1), Mwebesa H(1), Nyika H(2), Maureen K(1), Olaro C(1), Kagirita A(1), Larmode M(3), Riek LP(4), Temfack E(2), Salyer S(2), Aliddeki D(4), Machingaidze S(2), Mazuguni F(2), Kirenga B(1), Muttamba W(1), Wayengera M(1), Bbuye M(1), Kasambula A(1), Eurien D(1), Grace A(1), Ampaire I(1), Herbert I(1), Tut M(2), Bangure D(2), Mankoula W(2), Sonko I(2), Kokou AN(2), Magodi S(2), Mhiraf A(4), Bulwadda D(3), Kyabayinze D(1), Kabami Z(1), Muruta A(1), Bahatungire R(1), George U(1), Nabadda S(1), Birungi G(1), Richard K(1), Aragaw M(2), Ouma AO(2).
Author information:
(1)Ministry of Health, Kampala, Uganda.
(2)Africa Centres for Disease Control and Prevention, Addis Ababa, Ethiopia.
(3)Makerere University Institute of Infectious Diseases, Kampala, Uganda.
(4)Africa Centres for Disease Control and Prevention, Nairobi, Kenya.
On 20th September 2022, Uganda declared the 7th outbreak of Ebola virus disease (EVD) caused by the Sudan Ebola strain following the confirmation of a case admitted at Mubende Regional Referral Hospital. Upon confirmation, the Government of Uganda immediately activated the national incident management system to initiate response activities. Additionally, a multi-country emergency stakeholder meeting was held in Kampala; convening Ministers of Health from neighbouring Member States to undertake cross-border preparedness and response actions. The outbreak spanned 69 days and recorded 164 cases (142 confirmed, 22 probable), 87 recoveries and 77 deaths (case fatality ratio of 47%). Nine out of 136 districts were affected with transmission taking place in 5 districts but spilling over in 4 districts without secondary transmission. As part of the response, the Government galvanised robust community mobilisation and initiated assessment of medical counter measures including therapeutics, new diagnostics and vaccines. This paper highlights the response actions that contributed to the containment of this outbreak in addition to the challenges faced with a special focus on key recommendations for better control of future outbreaks.

72. Pathogens. 2023 Oct 19;12(10):1258. doi: 10.3390/pathogens12101258.
Perception of Ticks and Tick-Borne Diseases Worldwide.
de la Fuente J(1)(2), Estrada-Peña A(3)(4), Rafael M(1), Almazán C(5), Bermúdez

S(6), Abdelbaset AE(7), Kasaija PD(8), Kabi F(8), Akande FA(9), Ajagbe DO(10), Bamgbose T(11), Ghosh S(12)(13), Palavesam A(14), Hamid PH(15), Oskam CL(16)(17), Egan SL(16)(17), Duarte-Barbosa A(17)(18), Hekimoğlu O(19), Szabó MPJ(20), Labruna MB(21), Dahal A(22).
Author information:
(1)SaBio, Instituto de Investigación en Recursos Cinegéticos, IREC-CSIC-UCLM-JCCM, Ronda de Toledo 12, 13005 Ciudad Real, Spain.
(2)Department of Veterinary Pathobiology, Center for Veterinary Health Sciences, Oklahoma State University, Stillwater, OK 74078, USA.
(3)Department of Animal Health, Faculty of Veterinary Medicine, University of Zaragoza, 50013 Zaragoza, Spain.
(4)Research Group in Emerging Zoonoses, Instituto Agroalimentario de Aragón-IA2, Universidad de Zaragoza-CITA, 50013 Zaragoza, Spain.
(5)Facultad de Ciencias Naturales, Universidad Autonóma de Querétaro, Avenida de las Ciencias S/N Juriquilla, Querétaro 76230, Mexico.
(6)Medical Entomology Research Department, Gorgas Memorial Institute for Health Research, Panama City 0816-02593, Panama.
(7)Laboratory of Parasitology, Graduate School of Infectious Diseases, Faculty of Veterinary Medicine, Hokkaido University, Kita-18, Nishi-9, Sapporo 060-0818, Hokkaido, Japan.
(8)National Livestock Resources Research Institute (NaLIRRI/NARO), Kampala P.O. Box 5704, Uganda.
(9)Department of Veterinary Parasitology and Entomology, College of Veterinary Medicine, Federal University of Agriculture, Abeokuta 111101, Ogun State, Nigeria.
(10)Department of Pure and Applied Zoology, College of Biological Sciences, Federal University of Agriculture, Abeokuta 111101, Ogun State, Nigeria.
(11)Department of Biological Sciences, Microbiology Unit, Faculty of Science, Kings University, Ode-Omu City 221102, Osun State, Nigeria.
(12)Entomology Laboratory, Indian Veterinary Research Institute, Izatnagar, Bareilly 243122, Uttar Pradesh, India.
(13)IVRI-Eastern Regional Station, 37, Belgachia Road, Kolkata 700037, West Bengal, India.
(14)Translational Research Platform for Veterinary Biologicals, Centre for Animal Health Studies, Tamil Nadu Veterinary and Animal Sciences University, Chennai 600051, Tamil Nadu, India.
(15)Department of Animal Science, Universitas Sebelas Maret, Surakarta 57126, Indonesia.
(16)School of Medical, Molecular and Forensic Sciences, Murdoch University, Perth, WA 6150, Australia.
(17)Centre for One Health and Biosecurity, Harry Butler Institute, Murdoch University, Perth, WA 6150, Australia.
(18)School of Veterinary Medicine, Murdoch University, Perth, WA 6150, Australia.
(19)Division of Ecology, Faculty of Science, Hacettepe University, Beytepe, Ankara 06800, Turkey.
(20)Laboratório de Ixodologia, Faculdade de Medicina Veterinária, Universidade

Federal de Uberlândia, Av. Pará, 1720/Campus Umuarama-Bloco 2T, Uberlândia 38400-902, Brazil.
(21)Faculty of Veterinary Medicine and Animal Science, University of São Paulo, Sao Paulo 05508-220, Brazil.
(22)Department of Microbiology and Parasitology, Faculty of Animal Science, Veterinary Science and Fisheries, Agriculture and Forestry University, Chitwan 44200, Nepal.
In this comprehensive review study, we addressed the challenge posed by ticks and tick-borne diseases (TBDs) with growing incidence affecting human and animal health worldwide. Data and perspectives were collected from different countries and regions worldwide, including America, Europe, Africa, Asia, and Oceania. The results updated the current situation with ticks and TBD and how it is perceived by society with information bias and gaps. The study reinforces the importance of multidisciplinary and international collaborations to advance in the surveillance, communication and proposed future directions to address these challenges.

73. PLoS One. 2023 Oct 19;18(10):e0292053. doi: 10.1371/journal.pone.0292053.
Engagement of community health workers to improve immunization coverage through addressing inequities and enhancing data quality and use is a feasible and effective approach: An implementation study in Uganda.
Bakkabulindi P(1)(2), Ampeire I(3), Ayebale L(2)(4), Mubiri P(1), Feletto M(5), Muhumuza S(1).
Author information:
(1)School of Public Health, Makerere University College of Health Sciences, Kampala, Uganda.
(2)Health Support Initiatives, Kampala, Uganda.
(3)Ministry of Health, Kampala, Uganda.
(4)School of Statistics and Planning, Department of Population Studies Makerere University, Kampala, Uganda.
(5)Global Health Consultant, Geneva, Switzerland.
BACKGROUND: Uganda, like many other developing countries, faces the challenges of unreliable estimates for its immunization target population. Strengthening immunization data quality and its use for improving immunization program performance are critical steps toward improving coverage and equity of immunization programs. The goal of this study was to determine the effectiveness of using community health workers (CHWs) to obtain quality and reliable data that can be used for planning and evidence-based response actions.
METHODS: An implementation study in which 5 health facilities were stratified and randomized in two groups to (i) receive a package of interventions including monthly health unit immunization data audit meetings, and defaulter tracking and linkage and (ii) to serve as a control group was conducted between July and September 2020. Immunization coverage of infants in both arms was determined by a review of records three months before and after the study interventions. In addition, key informant and in-depth interviews were conducted among facility-based health workers and CHWs respectively, at the endline to explore the feasibility of the interventions.

RESULTS: Overall, a total of 2,048 children under one year eligible for immunization were registered in Bukabooli sub-county by CHWs as compared to the estimated district population of 1,889 children representing a moderate variance of 8.4%. The study further showed that it is feasible to use CHWs to track and link defaulters to points of immunization services as more than two-thirds (68%) of the children defaulting returned for catch-up immunization services. At the endline, immunization coverage for the Oral Polio Vaccine third dose; Rotavirus vaccine second dose; Pneumococcal Conjugate Vaccine third dose increased in both the intervention and control health facilities. There was a decrease in coverage for the Measles-Rubella vaccine decreased in the intervention health facilities and a decrease in Bacillus Calmette-Guérin vaccine coverage in the control facilities. Difference in difference analysis demonstrated that the intervention caused a significant 35.1% increase in coverage of Bacillus Calmette-Guérin vaccine (CI 9.00-61.19; p<0.05)). The intervention facilities had a 17.9% increase in DTP3 coverage compared to the control facilities (CI: 1.69-34.1) while for MR, OPV3, and Rota2 antigens, there was no significant effect of the intervention.
CONCLUSION: The use of CHWs to obtain reliable population estimates is feasible and can be useful in areas with consistently poor immunization coverage to estimate the target population. Facilitating monthly health unit immunization data audit meetings to identify, track, and link defaulters to immunization services is effective in increasing immunization coverage and equity.

74. Res Sq. 2023 Oct 9:rs.3.rs-3348260. doi: 10.21203/rs.3.rs-3348260/v1. Preprint.
Awareness, perceptions and challenges among public transport operators during the implementation of COVID-19 preventive measures in eastern Uganda: a qualitative study.
Napyo A(1), Hopp L(2), Mukunya D(3), Soita D(3), Matovu JK(3).
Author information:
(1)Kabale University.
(2)The Committee on Foreign Missions of the Orthodox Presbyterian Church, Akisyon a Yesu Presbyterian Clinic.
(3)Busitema University.
BACKGROUND: Public transportation plays a vital role in increasing transmission of COVID-19 due to the high-risk confined spaces in vehicles. It is therefore very crucial to employ the use of COVID-19 prevention measures during the use of public transportation to reduce risk of COVID-19 transmission. The success of the implementation and use of these measures depends largely on the public transportation users. We aimed at exploring the awareness, perceptions and challenges among public transport operators during the implementation of COVID-19 preventive measures in Eastern Uganda.
METHODS: This qualitative study was done in Eastern Uganda between January and February 2021. We conducted four focus group discussions, six in-depth interviews and three key informant interviews to document the awareness, perceptions and challenges faced by public transport operators including 10 boda boda riders, 19 taxi operators and 11 truck (cargo) transporters. All interviews were audio-recorded, transcribed verbatim, and analyzed using NVIVO software

Version 12 plus using a thematic framework approach.
RESULTS: Generally public transport operators were aware of that COVID-19 exists, its symptoms, how it's transmitted and ways in which it can be prevented.. However, they were not aware of what causes it and had misconceptions that it's spread through food and mosquitoes. Meanwhile some participants perceived COVID-19 as non-existent and that it was manufactured as a biological weapon. Some COVID-19 measures were perceived as having worked well during the pandemic like putting sanctions at the country borders, vaccination, observing hand hygiene, wearing a face mask, avoiding to touch the 'soft parts', quarantining in a hospital setting and social distancing. The COVID-19 preventive measures perceived as having not worked well were: home isolation, covid vaccination, using alcohol-based hand sanitizer, setting up curfew time, wearing a face mask, and reducing the number of passengers in the taxis and other public transportation vehicles. Challenges faced were mainly: financial loss resulting from reduction of passengers that used public transportation and setting up of curfew time, passengers not being able to use alcohol base hand sanitizer due to religious beliefs, loss of trust in public transportation by the public, hostility and defiance from passengers, competition for passengers among public transport operators and being mistreated by implementers of COVID-19 preventive measures like police. Various key players in the implementation of COVID-19 preventive measures included: the government, health workers, media, leaders in public transport and the police.
CONCLUSION AND RECOMMENDATION: Our study brings to light insights on the likely challenges that impede the use of preventive measures in public transportation use during an epidemic / pandemic like COVID-19 which could potentially escalate transmission. Focus should be put to the demystification of myths on COVID-19. Public transport passengers should be sensitized on risk of COVID-19 transmission during public transportation use and on the importance of complying with COVID-19 preventive measures. We recommend further exploration on the challenges faced by the public transportation passengers in implementing preventive measures in the event of an epidemic like COVID-19.

75. Children (Basel). 2023 Oct 7;10(10):1662. doi: 10.3390/children10101662.
Seroprevalence of Dengue and Chikungunya Virus Infections in Children Living in Sub-Saharan Africa: Systematic Review and Meta-Analysis.
Kayange N(1), Hau DK(2), Pain K(3), Mshana SE(4), Peck R(1)(2)(5), Gehring S(6), Groendahl B(6), Koliopoulos P(6), Revocatus B(7), Msaki EB(8), Malande O(9)(10)(11)(12).
Author information:
(1)Department of Pediatrics, Bugando Medical Centre, Weill Bugando School of Medicine, Catholic University of Health and Allied Sciences, Mwanza P.O. Box 1464, Tanzania.
(2)Department of Pediatrics, Weill Cornell Medical College, New York, NY 10065, USA.
(3)Samuel J. Wood Library and C.V. Starr Biomedical Information Center, Weill Cornell Medical College, 1300 York Avenue, New York, NY 10065, USA.
(4)Department of Microbiology and Immunology, Weill Bugando School of Medicine,

Catholic University of Health and Allied Sciences, Mwanza P.O. Box 1464, Tanzania.
(5)Center for Global Health, Department of Medicine, Weill Cornell Medical College, New York, NY 10065, USA.
(6)Department of Pediatrics, University Medical Center of the Johannes Gutenberg University, 55131 Mainz, Germany.
(7)Department of Data and Statistics, Bugando Medical Centre, Mwanza P.O. Box 1370, Tanzania.
(8)Department of Epidemiology and Biostatistics, Bugando Medical Centre, Mwanza P.O. Box 1370, Tanzania.
(9)East Africa Centre for Vaccines and Immunization (ECAVI), Kampala P.O. Box 3040, Uganda.
(10)Department of Public Health Phamarmacy, Sefako Makgatho Health Sciences University, Pretoria P.O. Box 60, South Africa.
(11)Department of Paediatrics & Child Health, Makerere University, Kampala P.O. Box 7072, Uganda.
(12)Department of Public Health, UNICAF University, Lusaka P.O. Box 20842, Zambia.
Dengue and chikungunya viruses are frequent causes of malarial-like febrile illness in children. The rapid increase in virus transmission by mosquitoes is a global health concern. This is the first systematic review and meta-analysis of the childhood prevalence of dengue and chikungunya in Sub-Saharan Africa (SSA). A comprehensive search of the MEDLINE (Ovid), Embase (Ovid), and Cochrane Library (Wiley) databases was conducted on 28 June 2019, and updated on 12 February 2022. The search strategy was designed to retrieve all articles pertaining to arboviruses in SSA children using both controlled vocabulary and keywords. The pooled (weighted) proportion of dengue and chikungunya was estimated using a random effect model. The overall pooled prevalence of dengue and chikungunya in SSA children was estimated to be 16% and 7%, respectively. Prevalence was slightly lower during the period 2010-2020 compared to 2000-2009. The study design varied depending on the healthcare facility reporting the disease outbreak. Importantly, laboratory methods used to detect arbovirus infections differed. The present review documents the prevalence of dengue and chikungunya in pediatric patients throughout SSA. The results provide unprecedented insight into the transmission of dengue and chikungunya viruses among these children and highlight the need for enhanced surveillance and controlled methodology.

76. BMC Proc. 2023 Oct 5;17(Suppl 7):26. doi: 10.1186/s12919-023-00280-z.
Shaping global vaccine acceptance with localized knowledge: a report from the inaugural VARN2022 conference.
Underwood T(1), Hopkins KL(2), Sommers T(3), Howell C(2), Boehman N(2), Dockery M(2), Dubé È(4), Dhaliwal BK(5), Kazi AM(6), Limaye R(5), Qasim R(7), Seale H(8), Kitutu FE(9), Kanwagi R(10), Knobler S(2).
Author information:
(1)Consultant to Sabin Vaccine Institute, Northwich, UK.
(2)Sabin Vaccine Institute, 2175 K Street NW, Suite 400, Washington DC, 20037,

USA.
(3)Sabin Vaccine Institute, 2175 K Street NW, Suite 400, Washington DC, 20037, USA. Theresa.sommers@sabin.org.
(4)Quebec National Institute of Public Health, Quebec City, Canada.
(5)Bloomberg School of Public Health, Johns Hopkins University, Baltimore, MD, USA.
(6)Department of Paediatrics & Child Health, Aga Khan University, Karachi, Pakistan.
(7)Institute of Nursing & Midwifery, Dow University of Health Sciences, Karachi, Pakistan.
(8)School of Population Health, University of New South Wales, Sydney, Australia.
(9)Department of Pharmacy, Makerere Unniversity School of Health Sciences, Kampala, Uganda.
(10)The Vaccine Confidence Project, Nairobi, Kenya.
The first conference of the Vaccination Acceptance Research Network, VARN2022: Shaping Global Vaccine Acceptance with Localized Knowledge, was held virtually, from March 1st to 3rd 2022. This inaugural event brought together a global representation of experts to discuss key priorities and opportunities emerging across the ecosystem of vaccine acceptance and demand, from policies to programs and practice. Convened by the Sabin Vaccine Institute, VARN aims to support dialogue among multidisciplinary stakeholders to enhance the uptake of social and behavioral science-based solutions for vaccination decision-makers and implementers. The conference centered around four key themes: 1) Understanding vaccine acceptance and its drivers; 2) One size does not fit all: community- and context-specific approaches to increase vaccine acceptance and demand; 3) Fighting the infodemic and harnessing social media for good; and 4) Frameworks, data integrity and evaluation of best practices. Across the conference, presenters and participants considered the drivers of and strategies to increase vaccine acceptance and demand relating to COVID-19 vaccination and other vaccines across the life-course and across low-, middle- and high-income settings. VARN2022 provided a wealth of evidence from around the world, highlighting the need for human-centered, multi-sectoral and transdisciplinary approaches to improve vaccine acceptance and demand. This report summarizes insights from the diverse presentations and discussions held at VARN2022, which will form a roadmap for future research, policy making, and interventions to improve vaccine acceptance and demand globally.

77. Trans R Soc Trop Med Hyg. 2023 Oct 3;117(10):697-704. doi: 10.1093/trstmh/trad028.
COVID-19 disease and vaccination in pregnancy: understanding knowledge, perceptions and experiences among pregnant women and community leaders in Uganda.
Nalubega P(1), Namugumya R(1), Zalwango F(1), Ssali A(1), Mboizi R(2), Hookham L(3), Seeley J(1)(4), Le Doare K(1)(3).
Author information:
(1)Medical Research Council/Uganda Virus Research Institute, and London School

of Hygiene and Tropical Medicine Uganda Research Unit, P.O. Box 49, Entebbe, Uganda.
(2)Makerere University-Johns Hopkins University Research Collaboration, P.O. Box 23491, Kampala, Uganda.
(3)St. George's, University of London, Cranmer Terrace, London SW17 0RE, UK.
(4)London School of Hygiene and Tropical Medicine, 15-17 Tavistock Place, London, UK.
BACKGROUND: We investigated pregnant women and community leaders' knowledge, perceptions and experiences of the coronavirus disease 2019 (COVID-19) vaccination program during pregnancy in Uganda and how this changed over the course of the pandemic.
METHODS: We conducted 20 in-depth interviews (IDIs) and two group discussions (GDs) with pregnant women and four GDs with community leaders in Kawempe division of Kampala, Uganda. The first round of IDIs/GDs were carried out in March 2021. In July 2021, telephone IDIs were conducted with 7 pregnant women and 10 community leaders randomly selected from first-round interview participants. Themes were analysed deductively drawing codes from the topic guides.
RESULTS: In the first round, the majority of participants thought COVID-19 was not real because of misconceptions around government messaging/motivation and beliefs that Africans would not be affected. In the second round, participants recognised COVID-19 disease, because of rising case numbers and fatalities. There was increased awareness of the benefits of the vaccine. However, pregnant women remained unsure of vaccine safety and quality, citing side effects like fevers and general body weakness. Role models and coherent public health messaging and healthcare workers were key enablers of vaccine uptake.
CONCLUSIONS: Targeted and sustained COVID-19 communication and engagement strategies are needed, especially for pregnant women and others in their communities, to improve vaccine confidence during outbreaks.

78. J Med Internet Res. 2023 Oct 2;25:e45132. doi: 10.2196/45132.
Implementing Smartphone-Based Telemedicine for Cervical Cancer Screening in Uganda: Qualitative Study of Stakeholders' Perceptions.
Kabukye JK(#)(1)(2), Namugga J(2)(3), Mpamani CJ(2), Katumba A(4), Nakatumba-Nabende J(4), Nabuuma H(4), Musoke SS(5), Nankya E(2), Soomre E(1), Nakisige C(2), Orem J(2).
Author information:
(1)SPIDER - The Swedish Program for ICT in Developing Regions, Department of Computer and Systems Sciences, Stockholm University, Stockholm, Sweden.
(2)Uganda Cancer Institute, Kampala, Uganda.
(3)Mulago Specialised Women and Neonatal Hospital, Kampala, Uganda.
(4)Department of Electrical Engineering, Makerere University, Kampala, Uganda.
(5)Global Programs for Research and Training, University of California San Francisco, Kampala, Uganda.
(#)Contributed equally
BACKGROUND: In Uganda, cervical cancer (CaCx) is the commonest cancer, accounting for 35.7% of all cancer cases in women. The rates of human

papillomavirus vaccination and CaCx screening remain low. Digital health tools and interventions have the potential to improve different aspects of CaCx screening and control in Uganda.
OBJECTIVE: This study aimed to describe stakeholders' perceptions of the telemedicine system we developed to improve CaCx screening in Uganda.
METHODS: We developed and implemented a smartphone-based telemedicine system for capturing and sharing cervical images and other clinical data, as well as an artificial intelligence model for automatic analysis of images. We conducted focus group discussions with health workers at the screening clinics (n=27) and women undergoing screening (n=15) to explore their perceptions of the system. The focus group discussions were supplemented with field observations and an evaluation survey of the health workers on system usability and the overall project.
RESULTS: In general, both patients and health workers had positive opinions about the system. Highlighted benefits included better cervical visualization, the ability to obtain a second opinion, improved communication between nurses and patients (to explain screening findings), improved clinical data management, performance monitoring and feedback, and modernization of screening service. However, there were also some negative perceptions. For example, some health workers felt the system is time-consuming, especially when it had just been introduced, while some patients were apprehensive about cervical image capture and sharing. Finally, commonplace challenges in digital health (eg, lack of interoperability and problems with sustainability) and challenges in cancer screening in general (eg, arduous referrals, inadequate monitoring and quality control) also resurfaced.
CONCLUSIONS: This study demonstrates the feasibility and value of digital health tools in CaCx screening in Uganda, particularly with regard to improving patient experience and the quality of screening services. It also provides examples of potential limitations that must be addressed for successful implementation.

79. BMJ Glob Health. 2023 Oct;8(Suppl 9):e012356. doi: 10.1136/bmjgh-2023-012356.
Research opportunities for the primary prevention and management of acute rheumatic fever and rheumatic heart disease: a National Heart, Lung, and Blood Institute workshop report.
Rwebembera J(1), Cannon JW(2), Sanyahumbi A(3), Sotoodehnia N(4), Taubert K(5)(6), Yilgwan CS(7), Bukhman G(8)(9), Masterson M(10), Bruno FP(10), Bowen A(2)(11), Dale JB(12), Engel ME(13)(14), Beaton A(15)(16), Van Beneden C(17).
Author information:
(1)Division of Adult Cardiology, Uganda Heart Institute Ltd, Kampala, Uganda.
(2)Wesfarmers Centre for Vaccines and Infectious Diseases, Telethon Kids Institute, Nedlands, Western Australia, Australia.
(3)Division of Cardiology, Department of Pediatrics, Baylor College of Medicine, Houston, Texas, USA.
(4)Department of Medicine, University of Washington, Seattle, Washington, USA.
(5)American Heart Association International, Basel, Switzerland.
(6)Department of Physiology, The University of Texas Southwestern Medical Center, Dallas, Texas, USA.

(7)Departments of Paediatrics and West African Center for Emerging Infectious Diseases, University of Jos/Jos University Teaching Hospital, Jos, Nigeria.
(8)Center for Integration Science in Global Health Equity, Brigham and Women's Hospital, Boston, Massachusetts, USA.
(9)Program in Global Noncommunicable Diseases and Social Change, Harvard Medical School, Boston, Massachusetts, USA.
(10)National Heart, Lung, and Blood Institute, National Institutes of Health, Bethesda, Maryland, USA.
(11)Department of Infectious Diseases, Perth Children's Hospital, Nedlands, Western Australia, Australia.
(12)Department of Medicine, The University of Tennessee Health Science Center, Memphis, Tennessee, USA.
(13)AFROStrep Research Initiative, Cape Heart Institute, Department of Medicine, University of Cape Town, Rondebosch, South Africa.
(14)South African Medical Research Council, Cape Town, South Africa.
(15)Cincinnati Children's Hospital Medical Center, Cincinnati, Ohio, USA.
(16)Department of Pediatrics, University of Cincinnati, Cincinnati, Ohio, USA.
(17)CDC Foundation, Atlanta, Georgia, USA cav7@cdc.gov.
Primary prevention of acute rheumatic fever (ARF) and rheumatic heart disease (RHD) encompasses the timely diagnosis and adequate treatment of the superficial group A Streptococcus (GAS) infections pharyngitis and impetigo. GAS is the only known inciting agent in the pathophysiology of the disease. However, sufficient evidence indicates that the uptake and delivery of primary prevention approaches in RHD-endemic regions are significantly suboptimal. This report presents expert deliberations on priority research and implementation opportunities for primary prevention of ARF/RHD that were developed as part of a workshop convened by the US National Heart, Lung, and Blood Institute in November 2021. The opportunities identified by the Primary Prevention Working Group encompass epidemiological, laboratory, clinical, implementation and dissemination research domains and are anchored on five pillars including: (A) to gain a better understanding of superficial GAS infection epidemiology to guide programmes and policies; (B) to improve diagnosis of superficial GAS infections in RHD endemic settings; (C) to develop scalable and sustainable models for delivery of primary prevention; (D) to understand potential downstream effects of the scale-up of primary prevention and (E) to develop and conduct economic evaluations of primary prevention strategies in RHD endemic settings. In view of the multisectoral stakeholders in primary prevention strategies, we emphasise the need for community co-design and government engagement, especially in the implementation and dissemination research arena. We present these opportunities as a reference point for research organisations and sponsors who aim to contribute to the increasing momentum towards the global control and prevention of RHD.

80. Ann Med Surg (Lond). 2023 Aug 17;85(10):5294-5296. doi: 10.1097/MS9.0000000000001183. eCollection 2023 Oct.
Zika virus disease: an alarming situation resurfacing on the radar - a short communication.
Shariff S(1)(2), Kantawala B(1)(2), Hamiidah N(1)(3), Yadav T(1)(4), Nazir

A(1)(5), Uwishema O(1)(6)(7).
Author information:
(1)Oli Health Magazine Organization, Research and Education, Kigali, Rwanda.
(2)Yerevan State Medical University, Yerevan, Armenia.
(3)Faculty of Nursing and Midwifery, Lira University, Lira, Uganda.
(4)Department of Medicine, Jinnah Postgraduate Medical Centre (JPMC), Karachi.
(5)King Edward Medical University, Lahore, Pakistan.
(6)Clinton Global Initiative University, New York, New York, USA.
(7)Faculty of Medicine, Karadeniz Technical University, Trabzon, Turkey.
BACKGROUND/INTRODUCTION: On the 13th of December 2022, a 5-year-old girl from Karnataka, India, tested positive for Zika virus. The first Zika virus was isolated from the serum of a rhesus monkey in the Zika Forest of Uganda in 1947. Zika virus was largely dormant for about 70 years before suddenly resurfacing across all of America, from Brazil to the Pacific Islands and is connected to a grouping of microcephaly phenotypes based on a complete virus genome analysis. All of the aforementioned research provides an overview of the migration of this virus from the Americas to continental Africa via mosquitoes. The current study, therefore, aims to evaluate the virologic characteristics, prophylaxis, transmitting mechanisms, diagnosis, clinical manifestations, and treatment of ZIKV infection in light of the virus's widespread dissemination and deadly nature.
AIM: The investigation's findings aim to demonstrate that in order to prevent further outbreaks, there is a national requirement for active epidemiological and entomological observation of Zika.
MATERIALS AND METHODS: Data were extracted from academic journals of medicine published in MEDLINE, PubMed, ScienceDirect, Ovid, and Embase inventory databases with a predetermined search strategy. Articles considering the Zika virus and its clinical manifestations, especially neurological, were included.
RESULTS: The Zika virus has been declared a public health emergency of global significance by the World Health Organization (WHO). It is of alarming concern that it is now one of the most prevalent infectious diseases associated with birth abnormalities discovered in the past five decades. The onset and accelerated spread of disease to other parts of the world is attributed to the migration of infected hosts and climate change. Rapid laboratory diagnosis, evaluation of serological techniques, and virus isolation are urgently needed, along with newer modalities such as mathematical modeling as prediction devices to curb this issue. Due to its grave neurological manifestations, it is mandated to engineer peptide therapies and a virus-specific vaccination to treat this neurotropic virus.
CONCLUSION: There is currently no vaccination against Zika virus infection. If societies are not adequately prepared, the epidemiological wave will have an impact on the workforce and could pose a serious threat. To alleviate the significant cost on health systems and manage its promotion globally, improved investigation and response activities are needed.

81. Lancet Public Health. 2023 Oct;8(10):e788-e799. doi:
10.1016/S2468-2667(23)00180-9.

Potential population-level effectiveness of one-dose HPV vaccination in low-income and middle-income countries: a mathematical modelling analysis.

Bénard É(1), Drolet M(2), Laprise JF(2), Gingras G(2), Jit M(3), Boily MC(4), Bloem P(5), Brisson M(6).
Author information:
(1)Département de médecine sociale et préventive, Université Laval, Québec City, QC, Canada; Centre de recherche du CHU de Québec-Université Laval, Québec City, QC, Canada.
(2)Centre de recherche du CHU de Québec-Université Laval, Québec City, QC, Canada.
(3)Centre for Mathematical Modelling of Infectious Disease, London School of Hygiene & Tropical Medicine, London, UK; School of Public Health, University of Hong Kong, Hong Kong Special Administrative Region, China.
(4)Département de médecine sociale et préventive, Université Laval, Québec City, QC, Canada; Centre de recherche du CHU de Québec-Université Laval, Québec City, QC, Canada; MRC Centre for Global Infectious Disease Analysis, School of Public Health, Imperial College London, London, UK.
(5)Life Course and Integration, Essential Programme on Immunization, Department of Immunization, Vaccines and Biologicals, World Health Organization, Geneva, Switzerland.
(6)Département de médecine sociale et préventive, Université Laval, Québec City, QC, Canada; Centre de recherche du CHU de Québec-Université Laval, Québec City, QC, Canada; MRC Centre for Global Infectious Disease Analysis, School of Public Health, Imperial College London, London, UK. Electronic address: marc.brisson@crchudequebec.ulaval.ca.
Comment in
Lancet Public Health. 2023 Oct;8(10):e748-e749.
BACKGROUND: Given the accumulating evidence that one-dose vaccination could provide high and sustained protection against human papillomavirus (HPV) infection and related diseases, we examined the population-level effectiveness and efficiency of one-dose HPV vaccination of girls compared with two-dose vaccination, using mathematical modelling.
METHODS: In this mathematical modelling study, we used HPV-ADVISE LMIC, an individual-based transmission-dynamic model independently calibrated to four epidemiologically diverse low-income and middle-income countries (LMICs; India, Nigeria, Uganda, and Viet Nam). We parameterised and calibrated the model using sexual behaviour and epidemiological data identified from international population-based datasets and the literature. All base-case vaccination scenarios start in 2023 with the nonavalent vaccine and assumed 80% vaccination coverage with one or two doses. We assumed that two doses of vaccine provide 100% efficacy against vaccine-type infections and a lifelong duration of protection. We examined a non-inferior vaccination scenario for one dose compared with two doses, pessimistic scenarios of lower one-dose vaccine efficacy (85%) or a shorter duration of protection (ie, 20 or 30 years), and the effectiveness of a mitigation scenario in which schedules would switch from one dose to two doses. We also did sensitivity analyses by varying vaccination coverage. We used three outcomes: the relative reduction in cervical cancer

incidence, the number of cervical cancers averted, and the number of vaccine doses needed to prevent one cervical cancer.
FINDINGS: Assuming non-inferior vaccine characteristics for one dose compared with two doses, the model projections show that two-dose or one-dose routine vaccination of girls aged 9 years (with a multi-age cohort vaccination of girls aged 10-14 years) would avert 12·0 million (80% UI 9·5-14·5) cervical cancers in India, 4·7 million (3·4-5·8) in Nigeria, 2·3 million (1·9-2·6) in Uganda, and 0·4 million (0·2-0·5) in Viet Nam over 100 years. Under pessimistic assumptions of lower one-dose efficacy (85%) or a shorter duration of protection (ie, 30 years), one-dose routine vaccination would avert 69% (61-80) to 94% (92-96) of the cervical cancers averted with two-dose routine vaccination. However, when assuming a duration of protection of 20 years, one-dose routine vaccination would avert substantially fewer cervical cancers (ie, 35% [26-44] to 69% [65-71] of the cervical cancers averted with two-dose routine vaccination). A switch from one-dose to two-dose routine vaccination of girls aged 9 years, with a one-dose catch-up of girls aged 10-14 years, 5 years after the start of the vaccination programme, could mitigate potential losses in cervical cancer prevention from a short one-dose duration of protection (averting 92% [83-98] to 99% [97-100]) of the cervical cancers averted with two-dose routine vaccination). One-dose routine vaccination would result in fewer doses needed to prevent one cervical cancer than two-dose routine vaccination, even if the duration of protection is as low as 20 years. Finally, for countries with two-dose routine vaccination, adding one-dose multi-age cohort vaccination in the first year would provide similar benefits as a two-dose multi-age cohort vaccination, and would be more efficient even under the pessimistic assumptions of lower one-dose vaccine efficacy or duration of protection.
INTERPRETATION: One-dose routine vaccination could avert most of the cervical cancers averted with two-dose vaccination while being more efficient, provided the duration of one-dose protection is greater than 20-30 years (depending on the LMIC). The doses saved by introducing one-dose routine vaccination could offer the opportunity to vaccinate girls before they age out of the vaccination window of 9-14 years and, potentially, to vaccinate boys or older age groups.
FUNDING: Fonds de recherche du Québec-Santé, Digital Research Alliance of Canada, Bill & Melinda Gates Foundation.

82. Lancet Child Adolesc Health. 2023 Oct;7(10):708-717. doi: 10.1016/S2352-4642(23)00174-8. Epub 2023 Aug 24.
Timing of maternal isoniazid preventive therapy on tuberculosis infection among infants exposed to HIV in low-income and middle-income settings: a secondary analysis of the TB APPRISE trial.
Gupta A(1), Singh P(2), Aaron L(3), Montepiedra G(3), Chipato T(4), Stranix-Chibanda L(4), Chanaiwa V(4), Vhembo T(4), Mutambanengwe M(4), Masheto G(5), Raesi M(5), Bradford S(6), Golner A(7), Costello D(8), Kulkarni V(9), Shayo A(10), Kabugho E(11), Jean-Phillippe P(12), Chakhtoura N(13), Sterling TR(14), Theron G(15), Weinberg A(16); IMPAACT P1078 TB APPRISE Study Team.
Author information:
(1)Johns Hopkins University School of Medicine, Baltimore, MD, USA. Electronic

address: agupta25@jhmi.edu.
(2)Johns Hopkins University School of Medicine, Baltimore, MD, USA.
(3)Harvard T H Chan School of Public Health, Boston, MA, USA.
(4)Faculty of Medicine and Health Sciences, Clinical Trials Research Centre, University of Zimbabwe, Harare, Zimbabwe.
(5)Botswana-Harvard AIDS Institute Partnership, Gaborone, Botswana.
(6)FHI 360, Durham, NC, USA.
(7)Frontier Science Foundation, Amherst, NY, USA.
(8)University of California, Los Angeles, CA, USA.
(9)Byramjee Jeejeebhoy Government College-Johns Hopkins Clinical Research Site, Pune, India.
(10)Kilimanjaro Christian Medical Center, Kilimanjaro Christian Medical University College, Moshi, Tanzania.
(11)Makerere University-Johns Hopkins University Research Collaboration, Kampala, Uganda.
(12)Division of AIDS, National Institute of Allergy and Infectious Diseases, National Institutes of Health, Bethesda, MD, USA.
(13)Eunice Kennedy Shriver National Institute of Child Health and Human Development, National Institutes of Health, Bethesda, MD, USA.
(14)Vanderbilt University Medical Center, Nashville, TN, USA.
(15)Department of Obstetrics and Gynecology, Family Centre for Research with Ubuntu, Stellenbosch University, Cape Town, South Africa.
(16)University of Colorado Denver Anschutz Medical Campus, Aurora, CO, USA.
BACKGROUND: Infants born to women with HIV in settings with a high tuberculosis burden are at risk of tuberculosis infection and rapid progression to active disease. Maternal isoniazid preventive therapy might mitigate this risk, but optimal timing of therapy remains unclear. The TB APPRISE trial showed that initiation of isoniazid during pregnancy resulted in more frequent adverse pregnancy outcomes than when initiated postpartum. We aimed to determine the proportion of infants testing positive for tuberculosis infection born to mothers who initiated isoniazid therapy antepartum compared with postpartum using two commonly used tests, the test agreement, and predictors of test positivity.
METHODS: TB APPRISE was a randomised, double-blind, placebo-controlled, non-inferiority trial done at 13 study sites across eight countries (Botswana, Haiti, India, South Africa, Tanzania, Thailand, Uganda, and Zimbabwe). Pregnant women with HIV on antiretroviral therapy were randomly assigned to receive immediate isoniazid preventive therapy (28 weeks isoniazid [300 mg daily], then placebo until week 40 after delivery) or deferred treatment (placebo until week 12 after delivery, then isoniazid [300 mg daily] for 28 weeks). Mother-infant pairs were followed up until 48 weeks after delivery. We included all liveborn infants with a tuberculin skin test or interferon-γ release assay (IGRA) at 44 weeks. The outcomes assessed in this secondary analysis were tuberculosis test positivity by study group, test agreement, and predictors of test positivity. This study was registered with ClinicalTrials.gov, NCT01494038.
FINDINGS: Between Aug 19, 2014, and April 4, 2016, 956 mothers were randomly assigned, and 749 mother-child pairs were included in this secondary analysis.

Of 749 infants, 694 (93%) received Bacille Calmette-Guérin (BCG) vaccination, 675 (90%) were born to mothers who had completed isoniazid treatment, 20 (3%) were exposed to tuberculosis, seven (1%) became HIV positive, and one (<1%) developed probable tuberculosis. 43 (6%; 95% CI 4-8]) of 732 infants had a positive IGRA test result and 55 (8%; 6-10) of 727 infants had a positive tuberculin skin test result. Test positivity did not differ by study group (p=0·88 for IGRA; p=0·44 for tuberculin skin test). Test agreement was poor (κ=0·107 [95% CI 0·002-0·212]). Infant tuberculin skin test positivity was associated with breastfeeding (adjusted odds ratio 6·63 [95% CI 1·57-27·9]), BCG vaccination (4·97 [1·50-16·43]), and maternal tuberculin skin test positivity at delivery (3·28 [1·70-6·33]); IGRA positivity was associated with female sex (2·09 [1·06-4·14]).
INTERPRETATION: Deferral of maternal isoniazid preventive therapy to early postpartum had no effect on infant tuberculosis acquisition in our trial population, regardless of the diagnostic test used; however, tuberculosis test agreement is poor during infancy.
FUNDING: US National Institutes of Health.

83. Lancet Child Adolesc Health. 2023 Oct;7(10):718-727. doi: 10.1016/S2352-4642(23)00164-5. Epub 2023 Aug 8.
Neuropsychiatric manifestations and sleep disturbances with dolutegravir-based antiretroviral therapy versus standard of care in children and adolescents: a secondary analysis of the ODYSSEY trial.
Turkova A(1), White E(2), Kekitiinwa AR(3), Mumbiro V(4), Kaudha E(5), Liberty A(6), Ahimbisibwe GM(7), Moloantoa T(6), Srirompotong U(8), Mosia NR(9), Puthanakit T(10), Kobbe R(11), Fortuny C(12), Kataike H(7), Bbuye D(3), Na-Rajsima S(13), Coelho A(14), Lugemwa A(15), Bwakura-Dangarembizi MF(4), Klein N(16), Mujuru HA(4), Kityo C(5), Cotton MF(17), Ferrand RA(18), Giaquinto C(19), Rojo P(20), Violari A(6), Gibb DM(2), Ford D(2); ODYSSEY trial team.
Collaborators: Mehar Nee Abdulla AF, Abraham P, Abrams E, Acero J, Agaba GM, Ahimbisibwe G, Ainebyoona B, Akobye W, Akhalwaya Y, Akoojee N, Ali SS, Amuge P, Andrea C, Muñoz Fernandez MA, Ankunda R, Rutebarika DA, Anugulruengkitt S, Apollo T, Archary M, Arendze R, Ategeka J, Atim E, Atwine L, Babiker A, Babirye S, Babu E, Bagirigomwa E, Baita A, Balamusani D, Baliram P, Baliruno D, Ball C, Balwa H, Bamford A, Bandi S, Barker D, Barlow-Mosha L, Bbuye D, Begum S, Behuhuma O, Bernays S, Besigye R, Bester M, Bhiri J, Bilardi D, Bird K, Bollen P, Borg C, Borges Da Silva AM, Brown J, Bruno E, Bunupuradah T, Burger D, Buthelezi N, Bwakura-Dangarembizi M, Byaruhanga A, Calvert J, Casey P, Cassim H, Cebekhulu S, Chailert S, Chalermpantmetagul S, Chamjamrat W, Chan M, Chandiwana P, Chankun T, Chanthaburanun S, Chanto N, Chidziva E, Chikowore M, Chimanzi J, Chinwong D, Chitongo S, Chitsamatanga M, Choga J, Chutima D, Clayden P, Coelho A, Colbers A, Compagnucci A, Constança Mendes A, Conway M, Cotton MF, Crawley J, Cressey TR, Crisp J, Matos AC, Dadan S, Daglish J, Danaviah S, Daniel T, De Rossi A, Denjanta S, Dobbels E, Dowie M, Dube P, Dube B, Dudakia N, Elwana A, Epalza C, Eram D, Erasmus J, Erim P, Escosa Garcia L, Essack Z, Estepa C, Etima M, Fernandes A, Fernandez M, Fitzgerald F, Flynn J, Ford D, Fortuny Guasch C, Foster C, Fourie G, Fourie Y, Foxall S, Frank D, Gandhi K, Garcia I, Gartner K,

Gasa J, Gasa G, Giaquinto C, Gibb DM, Gomez Rico C, Gomez-Pena D, Gondo S, Goodman A, Gorreti Nakalema M, Gozhora W, Greetanukroh P, Gregorio Maranon B, Grossele T, Gwande S, Gwaze T, Gwenzi T, Hakim J, Hakiza E, Kaka AH, Harley A, Isaacs M, Isabirye R, Ishemunyoro W, Jacobs T, Jafta L, Jamil N, Janse van Rensburg AJ, Jeaven V, Mellado Peña MJ, Jourdain G, Juliet K, Jumpimai T, Junkaew R, Jupimai T, Kaahwa W, Kabasonga M, Kaboggoza O, Kadhuba RJ, Kaewbundit A, Kaewmamueng K, Kafufu B, Kakayi B, Kamboua P, Kanjanavanit S, Kasangaki G, Kasipong N, Kasozi M, Kataike H, Katemba C, Kaudha E, Kekane N, Kekitiinwa AR, Keminyeto E, Khamduang W, Khamjakkaew W, Khamkon J, Khannak S, Khatngam O, Khayanchoomnoom T, Khumalo B, Khunene M, Khusuwan S, Kibalama P, Kibenge R, Kirabira A, Kityo CM, Kiyimba L, Klein N, Klinprung S, Kobbe R, Kobusingye O, Kobusungye J, Kongponoi A, Königs C, Koole O, Kouakam C, Krueduangkam N, Kruenual N, Kunjaroenrut N, Kyambadde R, Kyobutungi P, Kyomuhendo F, Kyomukama E, Lakha R, Langa C, Laomanit L, Lebotsa E, Leenasirimakul P, Lekku L, Lensen S, Leroy V, Li J, Liberty A, Limplertjareanwanich J, Little E, Lugemwa A, Lutalo E, Jimenez JL, Lyall H, MacDonald C, Machache G, Madlala P, Madonsela T, Maduna N, Maena J, Mahanontharit A, Makanga C, Makola C, Makumbi S, Malgraaf L, Mamiane A, Mantkowski F, Mapfumo W, Marques L, Mugagga AM, Maseko L, Masienyane T, Mathiba R, Matimba F, Mawlana S, Mayanja E, Mayat F, Mbabazi R, Mbadaliga N, Mbasani F, McClaughlin K, McIlleron H, Meethaisong W, Mendez Garcia P, Miwanda A, Miranda C, Mkhize S, Mmolawa K, Mngqibisa R, Mohamed F, Moloantoa T, Monametsi M, Montero S, Moore CL, Mosia R, Moyo C, Mthethwa M, Mudzingwa S, Mudzviti T, Mujuru H, Mujyambere E, Mukanganiki T, Mukisa Williams C, Mulder M, Mulima D, Mulindwa A, Mumbiro V, Mupambireyi Z, Murciano Cabeza A, Murungi H, Murungu D, Musarurwa S, Musiime V, Musiime AV, Musisi M, Musoke P, Musoke Nakirya B, Musoro G, Musumba S, Mustafa S, Mutsai S, Mwesigwa Rubondo P, Naabalamba M, Nagawa I, Naidoo A, Nakabuye S, Nakabuye S, Nakalanzi S, Nalubwama J, Nalugo A, Nalusiba S, Namajja C, Namanda S, Namayanja P, Nambi E, Namuddu RK, Namukwaya S, Namuli F, Namusanje J, Namwanje R, Nanan-Kanjee A, Nanduudu A, Nankunda C, Baddokwaya JN, Nannungi M, Nansamba W, Nanthapisal K, Nanyonjo J, Na-Rajsima S, Nasaazi C, Nascimento H, Nastouli E, Songtaweesin WN, Nathoo K, Natuhurira I, Nazzinda R, Ncgaba T, Ndigendawani M, Ndlovu M, Nentsa G, Ngampiyaskul C, Ngcobo N, Ngo Giang Huong N, Ngwaru P, Nhema R, Ninsiima E, Ninsiima G, Nkalo Phiri M, Noguera Julian A, Nolan M, Noppakaorattanamanee T, Nsibuka Kisekka M, Nsirim E, Nundlal R, Nunes R, Nyantsa L, Nyati M, O'Riordan S, Ocitti Labeja P, Odoch D, Oguntimehin R, Ojok M, Onen G, Orange W, Ounchanum P, Ouma B, Padrao A, Pako D, Parker A, Pasko-Szcech M, Patel R, Peongjakta R, Petpranee T, Phillips T, Philps J, Picault L, Pieterse S, Pinheiro H, Pongprapass S, Pozniak A, Prendergast A, Prieto Tato L, Puangmalai P, Puthanakit T, Rakgokong M, Ramos H, Ramsagar N, Rau C, Riault Y, Rojo Conejo P, Clark BR, Rubanga E, Rubinga B, Ruklao C, Runarassamee P, Rutebarika DA, Saenjum C, Saewtrakool C, Saidi Y, Sainz Costa T, Saisaengjan C, Sakwa R, Sarfati T, Sbisi N, Scheppers D, Schultze-Strasser S, Schulze-Sturm U, Scott K, Seeley J, Serunjogi R, Sewnarain L, Shakeshaft C, Sidhoo S, Shibemba M, Shingadia D, Singh S, Sirirungsi W, Sithebe S, Smit T, Smith K, Smuts M, Spyer M, Sripaoraya W, Srirompotong U, Srisuk W, Ssenyonga M, Sudsaard P, Sukrakanchana P, Tearsansern P, Teixeira C, Than-In-At K, Thapwai T, Thaweesombat Y, Thewsoongnoen J, Thiébaut R, Thomason M, Thrasyvoulou L,

Thungkham K, Tikabibamu J, Tinago G, Trairat K, Tudor-Williams G, Tukamushaba M, Tukwasibwe D, Tumusiime J, Tuna J, Turkova A, Turner R, Udomvised A, Vadee A, Van Huyssteen H, Van Looy N, Variava E, Vaughan-Gordon Y, Vecchia G, Violari A, Vowden R, Waalewijn H, Wampamba R, Welch S, Weller I, Weza S, White E, White I, Widuch K, Wilkes H, Wimonklang S, Wynne B, Yingyong P, Nakawungu ZZ, Zuidewind P.
Author information:
(1)Medical Research Council Clinical Trials Unit at University College London, London, UK. Electronic address: a.turkova@ucl.ac.uk.
(2)Medical Research Council Clinical Trials Unit at University College London, London, UK.
(3)Baylor College of Medicine, Kampala, Uganda.
(4)University of Zimbabwe Clinical Research Centre, Harare, Zimbabwe.
(5)Joint Clinical Research Centre, Kampala, Uganda.
(6)Perinatal HIV Research Unit, University of the Witwarsrand, Johannesburg, South Africa.
(7)Makerere University-Johns Hopkins University Research Collaboration, Kampala, Uganda.
(8)Pediatric Department, Khon Kaen Hospital, Thailand.
(9)Department of Paediatrics and Children Health, King Edward VIII Hospital, Enhancing Care Foundation, University of KwaZulu-Natal, Durban, South Africa.
(10)HIVNAT, Thai Red Cross AIDS Research Center, Bangkok, Thailand; Department of Pediatrics, Faculty of Medicine, Chulalongkorn University, Thailand.
(11)Institute for Infection Research and Vaccine Development, University Medical Centre Hamburg-Eppendorf, Hamburg, Germany; Department of Infectious Disease Epidemiology, Bernhard Nocht Institute for Tropical Medicine, Hamburg, Germany.
(12)Infectious Diseases Department, Institut de Recerca Sant Joan de Déu, Sant Joan de Déu Children's Hospital, Barcelona, Spain; Department of Surgery and Medico-Surgical Specialties, Faculty of Medicine and Health Sciences, Universitat de Barcelona, Barcelona, Spain.
(13)Pediatric Department, Mahasarakam Hospital, Thailand.
(14)INSERM/ANRS SC10-US19, Essais Thérapeutiques et Maladies Infectieuses, Villejuif, France.
(15)Joint Clinical Research Centre, Mbarara, Uganda.
(16)Infection, Immunity & Inflammation Department, UCL Great Ormond Street Institute of Child Health, London, UK; Africa Health Research Institute, Kwazulu-Natal, South Africa.
(17)Children's Infectious Diseases Clinical Research Unit, Family Center for Research with Ubuntu, Department of Paediatrics and Child Health, University of Stellenbosch, Cape Town, South Africa.
(18)Department of Clinical Research, Faculty of Infectious and Tropical Diseases, London School of Hygiene and Tropical Medicine, London, UK.
(19)Department of Women and Child Health, Padova, University of Padova, Italy.
(20)Pediatric Infectious Diseases Unit, Hospital 12 de Octubre, Madrid, Spain.
BACKGROUND: Cohort studies in adults with HIV showed that dolutegravir was associated with neuropsychiatric adverse events and sleep problems, yet data are scarce in children and adolescents. We aimed to evaluate neuropsychiatric

manifestations in children and adolescents treated with dolutegravir-based treatment versus alternative antiretroviral therapy.
METHODS: This is a secondary analysis of ODYSSEY, an open-label, multicentre, randomised, non-inferiority trial, in which adolescents and children initiating first-line or second-line antiretroviral therapy were randomly assigned 1:1 to dolutegravir-based treatment or standard-of-care treatment. We assessed neuropsychiatric adverse events (reported by clinicians) and responses to the mood and sleep questionnaires (reported by the participant or their carer) in both groups. We compared the proportions of patients with neuropsychiatric adverse events (neurological, psychiatric, and total), time to first neuropsychiatric adverse event, and participant-reported responses to questionnaires capturing issues with mood, suicidal thoughts, and sleep problems.
FINDINGS: Between Sept 20, 2016, and June 22, 2018, 707 participants were enrolled, of whom 345 (49%) were female and 362 (51%) were male, and 623 (88%) were Black-African. Of 707 participants, 350 (50%) were randomly assigned to dolutegravir-based antiretroviral therapy and 357 (50%) to non-dolutegravir-based standard-of-care. 311 (44%) of 707 participants started first-line antiretroviral therapy (ODYSSEY-A; 145 [92%] of 157 participants had efavirenz-based therapy in the standard-of-care group), and 396 (56%) of 707 started second-line therapy (ODYSSEY-B; 195 [98%] of 200 had protease inhibitor-based therapy in the standard-of-care group). During follow-up (median 142 weeks, IQR 124-159), 23 participants had 31 neuropsychiatric adverse events (15 in the dolutegravir group and eight in the standard-of-care group; difference in proportion of participants with ≥1 event p=0·13). 11 participants had one or more neurological events (six and five; p=0·74) and 14 participants had one or more psychiatric events (ten and four; p=0·097). Among 14 participants with psychiatric events, eight participants in the dolutegravir group and four in standard-of-care group had suicidal ideation or behaviour. More participants in the dolutegravir group than the standard-of-care group reported symptoms of self-harm (eight vs one; p=0·025), life not worth living (17 vs five; p=0·0091), or suicidal thoughts (13 vs none; p=0·0006) at one or more follow-up visits. Most reports were transient. There were no differences by treatment group in low mood or feeling sad, problems concentrating, feeling worried or feeling angry or aggressive, sleep problems, or sleep quality.
INTERPRETATION: The numbers of neuropsychiatric adverse events and reported neuropsychiatric symptoms were low. However, numerically more participants had psychiatric events and reported suicidality ideation in the dolutegravir group than the standard-of-care group. These differences should be interpreted with caution in an open-label trial. Clinicians and policy makers should consider including suicidality screening of children or adolescents receiving dolutegravir.

84. Nat Rev Immunol. 2023 Sep 28. doi: 10.1038/s41577-023-00941-2.
Immunological factors linked to geographical variation in vaccine responses.
van Dorst MMAR(#)(1), Pyuza JJ(#)(1)(2), Nkurunungi G(3)(4), Kullaya VI(5), Smits HH(1), Hogendoorn PCW(6), Wammes LJ(7), Everts B(1), Elliott AM(3)(4),

Jochems SP(1), Yazdanbakhsh M(8).
Author information:
(1)Department of Parasitology, Leiden University Center for Infectious Diseases, Leiden University Medical Center, Leiden, Netherlands.
(2)Department of Pathology, Kilimanjaro Christian Medical Centre, Moshi, Tanzania.
(3)Immunomodulation and Vaccines Programme, Medical Research Council/Uganda Virus Research Institute and London School of Hygiene and Tropical Medicine Uganda Research Unit, Entebbe, Uganda.
(4)Department of Infection Biology, London School of Hygiene and Tropical Medicine, London, UK.
(5)Kilimanjaro Clinical Research Institute, Kilimanjaro Christian Medical Centre, Moshi, Tanzania.
(6)Department of Pathology, Leiden University Medical Center, Leiden, Netherlands.
(7)Department of Medical Microbiology, Leiden University Center for Infectious Diseases, Leiden University Medical Center, Leiden, Netherlands.
(8)Department of Parasitology, Leiden University Center for Infectious Diseases, Leiden University Medical Center, Leiden, Netherlands. m.yazdanbakhsh@lumc.nl.
(#)Contributed equally
Vaccination is one of medicine's greatest achievements; however, its full potential is hampered by considerable variation in efficacy across populations and geographical regions. For example, attenuated malaria vaccines in high-income countries confer almost 100% protection, whereas in low-income regions these same vaccines achieve only 20-50% protection. This trend is also observed for other vaccines, such as bacillus Calmette-Guérin (BCG), rotavirus and yellow fever vaccines, in terms of either immunogenicity or efficacy. Multiple environmental factors affect vaccine responses, including pathogen exposure, microbiota composition and dietary nutrients. However, there has been variable success with interventions that target these individual factors, highlighting the need for a better understanding of their downstream immunological mechanisms to develop new ways of modulating vaccine responses. Here, we review the immunological factors that underlie geographical variation in vaccine responses. Through the identification of causal pathways that link environmental influences to vaccine responsiveness, it might become possible to devise modulatory compounds that can complement vaccines for better outcomes in regions where they are needed most.

85. PLOS Glob Public Health. 2023 Sep 22;3(9):e0000478. doi: 10.1371/journal.pgph.0000478. eCollection 2023.
Distribution of Hepatitis B prevention services in Wakiso District, Central Uganda.
Ssekamatte T(1), Isunju JB(1), Nalugya A(1), Wafula ST(1), Nuwematsiko R(1), Nakalembe D(1), Kansiime WK(1), Muyanga N(1), Nakiggala J(1), Bukenya JN(2), Mugambe RK(1).
Author information:
(1)Department of Disease Control and Environmental Health, School of Public

Health, College of Health Science, Makerere University, Kampala, Uganda.
(2)Department of Community Health and Behavioural Sciences, School of Public Health, College of Health Science, Makerere University, Kampala, Uganda.
Hepatitis B Virus (HBV) infection remains a significant global public health challenge especially in low-and-middle income countries. Although there are significant global and national efforts to control Hepatitis B, equitable distribution and access to prevention services such as testing and vaccination remains a challenge. Efforts to increase access are hindered by inadequate evidence on the availability and distribution of HBV services. This cross-sectional study aimed at generating evidence of the distribution of HBV prevention services in Wakiso District, Uganda. A total of 55 healthcare facilities (HCFs) including 4 hospitals, and 51 primary care facilities were surveyed. Data were collected using an electronic structured questionnaire and analysed using STATA 14.0. A chi-square test was performed to establish the relationship between HCF characteristics and the availability of hepatitis B services. ArcGIS (version 10.1) was used to generate maps to illustrate the distribution of hepatitis B prevention services. We found out that the hepatitis B vaccine was available in only 27.3% (15) of the HCF, and 60% (33) had testing services. Receipt of the hepatitis B vaccine doses in the last 12 months was associated with the level ($p = \leq 0.001$) and location ($p = 0.030$) of HCF. Availability of the hepatitis B vaccine at the time of the survey was associated with the level ($p = 0.002$) and location ($p = 0.010$) of HCF. The availability of hepatitis B testing services was associated with the level ($p = 0.031$), ownership ($p \leq 0.001$) and location ($p = 0.010$) of HCF. HCFs offering vaccination and testing services were mostly in urban areas, and close to Kampala, Uganda's capital. Based on this study, hepatitis B prevention services were sub-optimal across all HCF levels, locations, and ownership. There is a need to extend hepatitis B prevention services to rural, public and private-not-for-profit HCFs.

86. BMC Res Notes. 2023 Sep 14;16(1):218. doi: 10.1186/s13104-023-06499-5.
Rubella virus genotype 2B endemicity and related utility of serum-based molecular characterization in Uganda.
Tushabe P(1), Bakamutumaho B(2), Eliku JP(2), Birungi M(2), Aine F(2), Namuwulya P(2), Bukenya H(2), Ampeire I(2)(3), Kisakye A(2)(4), Byabamazima CR(2)(5), Bwogi J(2).
Author information:
(1)EPI-Laboratory, Uganda Virus Research Institute, P.O. Box 49, Entebbe, Uganda. ptushabe@uvri.go.ug.
(2)EPI-Laboratory, Uganda Virus Research Institute, P.O. Box 49, Entebbe, Uganda.
(3)Ministry of Health Uganda, P.O. Box 7272, Kampala, Uganda.
(4)World Health Organization, Uganda Country Office, P.O. Box 24578, Kampala, Uganda.
(5)WHO Inter-Country Support Team Office for Eastern and Southern Africa (IST/ESA), Harare, Zimbabwe.
There are 13 globally recognized rubella virus genotypes of which only 2 (1E and

2B) have been detected recently. The largest percentage of all reported rubella virus sequences come from China and Japan with Africa reporting limited data. In a bid to address the lack of rubella genotype data in Uganda and the World Health Organization Africa region, we sought to characterize rubella viruses retrospectively using sera collected from suspected measles patients that turned out rubella IgM positive.Seven sequences belonging to genotype 2B sub-lineage 2B-L2c were obtained. These sequences clustered with other genotype 2B sequences previously reported from Uganda. None of the other genotypes (1E and 1G) reported from Uganda in the earlier years were detected. In addition, none of the sequences were obtained after the introduction of the measles-rubella containing vaccine. The above highlight the need for continuous rubella virological surveillance to confirm interruption of endemic rubella genotype circulation.

87. Global Health. 2023 Sep 6;19(1):68. doi: 10.1186/s12992-023-00968-z.
Socio-demographic factors associated with COVID-19 vaccine uptake and refusal among Ugandan women.
Backhaus A(1).
Author information:
(1)Federal Institute for Population Research (BiB), Wiesbaden, Germany.
andreas.backhaus@bib.bund.de.
BACKGROUND: This paper analyzes associations of socio-demographic factors with the uptake of COVID-19 vaccines, the refusal to get vaccinated against COVID-19, and various reasons stated for refusing vaccination against COVID-19 among a representative sample of Ugandan women.
METHODS: This paper utilizes a representative cross-sectional survey collected among women aged 15-49 years in Uganda between September and November 2021. Regression analyses are used to study the associations of a broad range of socio-demographic characteristics with COVID-19 vaccine uptake, refusal of vaccination, and reasons for refusal among the respondents.
RESULTS: 4211 women were included in the analysis. 11% of them were vaccinated against COVID-19, 76% were willing to get vaccinated, 13% were unwilling to get vaccinated. Fear of side effects was the most commonly stated reason for refusing vaccination (69%). Factors significantly and positively associated with being vaccinated against COVID-19 were age, higher education, urban residency, having savings, partial instead of complete income loss during the pandemic, and usage of modern contraceptives. Factors significantly and positively associated with refusing vaccination against COVID-19 were urban residency and current pregnancy, while age, having savings, and using modern contraceptives were factors associated with a lower likelihood of refusing vaccination, albeit with varying statistical significance. Few factors were strongly related to the stated reasons for refusing the vaccines; the fear of side effects significantly increased with age, while having received negative information on the vaccines was significantly less common among women with higher education.
CONCLUSIONS: This study documents a low COVID-19 vaccination rate and a high willingness to get vaccinated among Ugandan women. Positive age and education gradients in vaccine uptake point to inequity in access to vaccination,

potentially resulting from prioritizations of groups at particularly high risk. Refusal to be vaccinated was relatively low and systematic factors behind vaccine refusal were hardly to be found, even less so for particular reasons given for refusal.

88. Open Forum Infect Dis. 2023 Aug 25;10(9):ofad452. doi: 10.1093/ofid/ofad452.
Implementation of Hepatitis B Screening Into Routine Antenatal Care to Prevent Mother-to-Child Transmission in Rural Western Uganda.
Thahir S(1), Muhindo E(2), Turigye B(3), Kabagambe K(4), Thompson P(5), Mulogo EM(3), Boyce RM(6)(7)(8).
Author information:
(1)Department of Pediatrics, University of North Carolina at Chapel Hill, Chapel Hill, North Carolina, USA.
(2)Peoples Health and Economic Development Organization, Kasese, Uganda.
(3)Department of Community Health, Mbarara University of Science and Technology, Mbarara, Uganda.
(4)The National Organisation for People Living with Hepatitis B, Kampala, Uganda.
(5)Department of Pediatrics, Division of Infectious Diseases, University of North Carolina at Chapel Hill, Chapel Hill, North Carolina, USA.
(6)Department of Medicine, Division of Infectious Diseases, University of North Carolina at Chapel Hill, Chapel Hill, North Carolina, USA.
(7)Department of Epidemiology, Gillings School of Global Public Health, University of North Carolina at Chapel Hill, Chapel Hill, North Carolina, USA.
(8)Carolina Population Center, University of North Carolina at Chapel Hill, Chapel Hill, North Carolina, USA.
In rural Uganda where birth dose vaccination for hepatitis B is not routine, we implemented a pilot program for preventing mother-to-child transmission that effectively identified women with high-risk hepatitis B virus (HBV) infection and started antiviral treatment during pregnancy. Further work is required to enhance antiviral adherence through delivery to ensure effective prevention of vertical HBV transmission.

89. EBioMedicine. 2023 Sep;95:104772. doi: 10.1016/j.ebiom.2023.104772.
Malaria-specific Type 1 regulatory T cells are more abundant in first pregnancies and associated with placental malaria.
Kirosingh AS(1), Delmastro A(1), Kakuru A(2), van der Ploeg K(1), Bhattacharya S(3), Press KD(1), Ty M(1), Parte L(1), Kizza J(4), Muhindo M(4), Devachanne S(5), Gamain B(5), Nankya F(2), Musinguzi K(2), Rosenthal PJ(3), Feeney ME(3), Kamya M(6), Dorsey G(3), Jagannathan P(7).
Author information:
(1)Stanford University School of Medicine, Stanford, USA.
(2)Infectious Diseases Research Collaboration, Kampala, Uganda.
(3)University of California, San Francisco, USA.
(4)Makerere University, Kampala, Uganda.
(5)Université Paris Cité, INSERM, BIGR, F-75014 Paris, France.
(6)Infectious Diseases Research Collaboration, Kampala, Uganda; Makerere

University, Kampala, Uganda.
(7)Stanford University School of Medicine, Stanford, USA. Electronic address: prasj@stanford.edu.
BACKGROUND: Malaria in pregnancy (MIP) causes higher morbidity in primigravid compared to multigravid women; however, the correlates and mechanisms underlying this gravidity-dependent protection remain incompletely understood. We aimed to compare the cellular immune response between primigravid and multigravid women living in a malaria-endemic region and assess for correlates of protection against MIP.
METHODS: We characterised the second trimester cellular immune response among 203 primigravid and multigravid pregnant women enrolled in two clinical trials of chemoprevention in eastern Uganda, utilizing RNA sequencing, flow cytometry, and functional assays. We compared responses across gravidity and determined associations with parasitaemia during pregnancy and placental malaria.
FINDINGS: Using whole blood RNA sequencing, no significant differentially expressed genes were identified between primigravid (n = 12) and multigravid (n = 11) women overall (log 2(FC) > 2, FDR < 0.1). However, primigravid (n = 49) women had higher percentages of malaria-specific, non-naïve CD4+ T cells that co-expressed IL-10 and IFNγ compared with multigravid (n = 85) women (p = 0.000023), and higher percentages of these CD4+ T cells were associated with greater risks of parasitaemia in pregnancy (Rs = 0.49, p = 0.001) and placental malaria (p = 0.0073). These IL-10 and IFNγ co-producing CD4+ T cells had a genomic signature of Tr1 cells, including expression of transcription factors cMAF and BATF and cell surface makers CTLA4 and LAG-3.
INTERPRETATION: Malaria-specific Tr1 cells were highly prevalent in primigravid Ugandan women, and their presence correlated with a higher risk of malaria in pregnancy. Understanding whether suppression of Tr1 cells plays a role in naturally acquired gravidity-dependent immunity may aid the development of new vaccines or treatments for MIP.

90. Soc Sci Med. 2023 Sep;333:116146. doi: 10.1016/j.socscimed.2023.116146.
Addressing vaccine concerns through the spectrum of vaccine acceptance.
Maureen Ayikoru(1), Cole J(2), Dodds K(2), Atcero M(3), Bada JK(3), Petrikova I(2), Worodria W(4).
Author information:
(1)Royal Holloway, University of London (RHUL), Egham Hill, Egham, Surrey, TW20 0EX, UK. Electronic address: Maureen.ayikoru@rhul.ac.uk.
(2)Royal Holloway, University of London (RHUL), Egham Hill, Egham, Surrey, TW20 0EX, UK.
(3)Makerere University Business School (MUBS), Plot 21A, Portbell Road, P.O. Box, 1337, Kampala, Uganda.
(4)Makerere Lung Institute, Makerere University College of Health Sciences, New Mulago Complex, P.O. Box 7749, Kampala, Uganda.
RATIONALE/OBJECTIVE: Several studies have examined attitudes towards COVID-19 vaccines, giving prominence to hesitancy and conceptual models that seek to explain its prevalence, mostly in high-income contexts. An alternative conceptual approach that prioritises an understanding of vaccine concerns, the

rationality of the questions people have, the political and media ecologies that raise them, will help recommend ways in which such concerns can be addressed. This current study employs the Spectrum of Vaccine Acceptance as a conceptual framework to explain vaccine concerns, in a low-income context.
METHODS: A cross-sectional survey was designed by drawing on the extant literature on indirect impacts of COVID-19 pandemic. The survey was administered face to face to a stratified random sample of 459 healthcare professionals in Uganda, from 1st to July 31, 2021. Key findings from the survey were explored using focus group interviews. Descriptive analysis was performed to quantify key responses on socio-demographic characteristics, feelings and views about COVID-19 and vaccines. Qualitative themes from the survey and focus groups were explained through the framework of the Spectrum of Vaccine Acceptance.
RESULTS: Vaccine acceptance was the most dominant attitude among healthcare professionals, with 74.9% of all respondents (N = 224) having been vaccinated. The findings highlight a relationship between vaccine acceptance, vaccine questioning and vaccine hesitancy, in that nearly 60% (N = 127) of those that were already vaccinated had several concerns about the vaccines they had received, suggesting that questions do not necessarily equate to refusal. This led to a partial reframing of the Spectrum of Vaccine Acceptance.
CONCLUSION: Factors which determine and differentiate vaccine concerns among healthcare professionals in a low-income context show that rational concerns far outweigh non-sensical and conspiratorial views. The findings will act as a useful input into the importance of understanding and addressing vaccine concerns, and the role of managing information voids in pandemic management.

91. Trop Med Int Health. 2023 Sep;28(9):720-730. doi: 10.1111/tmi.13916.
Developing HIV risk prediction tools in four African settings.
Kansiime S(1)(2), Hansen CH(1)(2), Hayes R(2), Ruzagira E(1); PrEPVacc Study Team.
Author information:
(1)Medical Research Council/Uganda Virus Research Council and London School of Hygiene and Tropical Medicine, Uganda Research Unit, Entebbe, Uganda.
(2)Medical Research Council International Statistics and Epidemiology Group, London School of Hygiene & Tropical Medicine, London, UK.
OBJECTIVE: HIV risk prediction tools are a critical component of efforts to end the HIV pandemic. We aimed to create and validate tools for identifying individuals at highest risk of prevalent and incident HIV in an African setting.
METHODS: We used Logistic regression and Poisson regression to determine risk factors for HIV prevalence and incidence in a multi-country HIV vaccine trial preparedness cohort study among individuals at high risk of HIV, and used the identified factors to create and validate tools that predict HIV risk. We also assessed the performance of the VOICE risk score in predicting HIV incidence among women in the cohort.
RESULTS: The prevalent HIV prediction tool created had good predictive ability [area under the curve (AUC) = 0.70, 95% CI 0.66-0.74]. It included the following participant variables: age, sex, recreational drug use, unprotected male-to-male anal sex, a sexual partner who had other partners, transactional sex and having

a partner who was a long-distance truck driver/miner. It was not possible to create a valid HIV incidence prediction tool. Participants with high VOICE risk scores (≥7) had slightly higher HIV incidence but this tool performed poorly within our study (AUC = 0.58, 95% CI 0.51-0.64: Harrell's concordance index = 0.59).
CONCLUSION: We created a prevalent HIV prediction tool that could be used to increase efficiency in diagnosis of HIV and linkage to care in sub-Saharan Africa. Existing incident HIV prediction tools may need modification to include context-specific predictors such as calendar period, participant occupation, study site, before adoption in settings different from those in which they were developed.

92. IJID Reg. 2023 May 23;8:31-35. doi: 10.1016/j.ijregi.2023.05.003.
Clinical characteristics and outcomes of patients hospitalized with measles during an outbreak in Somalia.

Hassan MY(1), Mohamud RYH(2), Kassim MM(1), Hussein AI(3), Adam MK(4), Akbulut UE(5), Olum R(6), Okot J(7), Bongomin F(7), Ahmed MAM(8)(9).
Author information:
(1)Department of Paediatrics, Mogadishu Somalia Turkey Recep Tayyip Erdogan Training and Research Hospital, Mogadishu, Somalia.
(2)Department of Education, Mogadishu Somalia Turkey Recep Tayyip Erdogan Training and Research Hospital, Mogadishu, Somalia.
(3)Department of Obstetrics and Gynaecology, Mogadishu Somalia Turkey Recep Tayyip Erdogan Training and Research Hospital, Mogadishu, Somalia.
(4)Department of Surgery, Mogadishu Somalia Turkey Recep Tayyip Erdogan Training and Research Hospital, Mogadishu, Somalia.
(5)University of Health Sciences, Antalya Training and Research Hospital, Antalya, Turkey.
(6)School of Medicine, College of Health Sciences, Makerere University, Kampala, Uganda.
(7)Department of Medical Microbiology and Immunology, Faculty of Medicine, Gul University, Gulu, Uganda.
(8)Department of Paediatrics, Faculty of Medicine and Surgery, Mogadishu University, Mogadishu, Somalia.
(9)Department of Paediatric Cardiology, Uganda Heart Institute, Kampala, Uganda.

OBJECTIVES: Measles is a contagious disease associated with significant morbidity and mortality. This paper describes the clinical characteristics and outcomes of patients hospitalized with measles during an outbreak in Somalia from January 2018 to December 2021.
METHODS: This retrospective study was conducted at Mogadishu Somalia Turkey Recep Tayyip Erdogan Training and Research Hospital. Children aged 6 months to 17 years who were hospitalized with clinical signs and symptoms of measles and its complications were recruited.
RESULTS: In total, 110 participants were enrolled. The median age was 16 [interquartile range (IQR) 12-36] years, and 87 (79.1%) were male. All participants presented with fever, typical measles rash, cough and conjunctivitis, and 43 (39.1%) had received the measles vaccine. Overall, 104 (94.6%) participants were admitted with severe respiratory symptoms, and six

(5.4%) were admitted due to poor feeding and/or significant dehydration.
Overall, all-cause mortality was 1.8% (n=2). The median duration of hospitalization was longer among participants who died compared with those who survived [11 (IQR 8-14) vs 4 (IQR 2-6) days; P=0.046]. Unvaccinated participants were significantly younger than vaccinated participants [36 (IQR 24-72) vs 12 (IQR 9-16) months; P<0.001]. There was a trend towards higher mortality [0/43 (0%) vs 2/67 (3%); P=0.519] and longer length of hospitalization [3 (IQR 2-6) vs 4 (IQR 3-7) days; P=0.056] among unvaccinated participants compared with vaccinated participants. Both the median total leukocyte count [5.7 (IQR 3.9-8.5) vs 11.6 (IQR 5.9-46.3) x 109/L; P<0.001] and platelet count [239 (IQR 202-358) vs 308 (IQR 239-404) x 109/L; P=0.032] were significantly higher among unvaccinated participants compared with vaccinated participants. However, the median haemoglobin concentration was significantly higher among vaccinated participants compared with unvaccinated participants [11.1 (IQR 9.9-12.3) vs 10.1 (IQR 9.1-11.2) g/dL; P=0.006].
CONCLUSIONS: Patients with measles in Somalia have a short hospital stay, low mortality rate and low vaccination rate. Timely vaccination and the need for improved care of patients with measles, particularly vulnerable groups including children and those with undernutrition, are encouraged.

93. PLOS Glob Public Health. 2023 Aug 30;3(8):e0002259. doi: 10.1371/journal.pgph.0002259. eCollection 2023.
Clinic presentation delay and tuberculosis treatment outcomes in the Lake Victoria region of East Africa: A multi-site prospective cohort study.
Mulholland GE(1), Herce ME(2), Okech BA(3), Jeremiah K(4), Bahemuka UM(5), Kwena ZA(6), Nanyonjo G(3), Seeley J(5)(7), Pettifor A(1), Emch M(1)(8), Weir SS(1), Edwards JK(1).
Author information:
(1)Department of Epidemiology, University of North Carolina at Chapel Hill, Chapel Hill, North Carolina, United States of America.
(2)Institute for Global Health and Infectious Diseases, School of Medicine, University of North Carolina at Chapel Hill, Chapel Hill, North Carolina, United States of America.
(3)UVRI-IAVI HIV Vaccine Program Limited, Entebbe, Uganda.
(4)Mwanza Research Centre, National Institute for Medical Research, Mwanza, Tanzania.
(5)Medical Research Council/Uganda Virus Research Institute & London School of Hygiene and Tropical Medicine, Uganda Research Unit, Entebbe, Uganda.
(6)Kenya Medical Research Institute, Nairobi, Kenya.
(7)Global Health and Development Department, London School of Hygiene and Tropical Medicine, London, United Kingdom.
(8)Department of Geography, University of North Carolina at Chapel Hill, Chapel Hill, North Carolina, United States of America.
In the Lake Victoria region of East Africa, little is known about delays between tuberculosis (TB) symptom onset and presentation at a clinic. Associations between clinic presentation delay and TB treatment outcomes are also poorly understood. In 2019, we abstracted data from routine TB treatment records for

all adults (n = 776) initiating TB treatment in a 6-month period across 12 health facilities near Lake Victoria. We interviewed 301 cohort members and assessed whether they experienced a clinic presentation delay longer than 6 weeks. We investigated potential clinical and demographic correlates of clinic presentation delay and examined the association between clinic presentation delay and an unfavorable TB treatment outcome (death, loss to follow-up, or treatment failure). Clinic presentation delay was common, occurring among an estimated 54.7% (95% CI: 48.9%, 61.2%) of cohort members, though no specific correlates were identified. Clinic presentation delay was slightly associated with unfavorable TB treatment outcomes. The 180-day risk of an unfavorable outcome was 14.2% (95% CI: 8.0%, 20.4%) among those with clinic presentation delay, compared to 12.7% (95% CI: 5.1%, 20.3%) among those presenting earlier. Multi-level community-based interventions may be necessary to reduce clinic presentation delays in communities near Lake Victoria.

94. J Community Health. 2023 Aug 30. doi: 10.1007/s10900-023-01261-1.
Compulsory Vaccination Coverage in 12 Sub-Saharan African Countries Two Years Following the COVID-19 Pandemic.
Ghazy RM(1), Gebreal A(2), Saleeb MRA(3), Sallam M(4)(5), El-Deen AEN(6)(7), Sheriff SD(8), Tessema EA(9), Ahurwendeire S(10), Tsoeu N(11), Chamambala PC(12), Cibangu PB(13), Okeh DU(14), Traoré AS(15), Eshun G(16), Kengo NE(17), Kubuka AE(18), Awuah LB(19), Salah A(20), Aljohani M(21), Fadl N(22).
Author information:
(1)Tropical Health Department, High Institute of Public Health, Alexandria University, Alexandria, Egypt.
(2)Faculty of Medicine, Alexandria University, Alexandria, Egypt.
(3)Public Health Institute, Faculty of Health, Liverpool John Moores University, Liverpool, UK.
(4)Department of Pathology, Microbiology and Forensic Medicine, School of Medicine, The University of Jordan, Amman, Jordan.
(5)Department of Clinical Laboratories and Forensic Medicine, Jordan University Hospital, Amman, Jordan.
(6)Department of Physiology, Faculty of Medicine, Al-Azhar University, Assiut, Egypt.
(7)Department of Basic Medical and Dental Sciences, Faculty of Dentistry, Zarqa University, PO Box 2000, Zarqa, 13110, Jordan.
(8)Sierra Leone Ministry of Health and Sanitation, Freetown, Sierra Leone.
(9)College of Health Science, School of Medicine, Addis Ababa University, Addis Ababa, Ethiopia.
(10)Department of Epidemiology and Biostatistics, Makerere University School of Public Health, Kampala, Uganda.
(11)Motebang Hospital, Hlotse, Lesotho.
(12)Zomba Central Hospital, Zomba, Malawi.
(13)Health Officer, Les Ailes du Coeur NGO, Congo, Democratic Republic of Congo.
(14)Department of Community Medicine, Federal Medical Centre Umuahia, Abia, Nigeria.
(15)Association Faso Civic, Ouagadougou, Burkina Faso.

(16)Seventh-Day Adventist Hospital, Agona-Asamang, Ghana.
(17)Faculty of Medicine and Biomedical Sciences, University of Garoua, Garoua, Cameroun.
(18)Saint Francis University College of health and allied Sciences, Ifakara, Morogoro, Tanzania.
(19)Department of Health Promotion and Disability Studies, School of Public Health, Kwame Nkrumah University of Science and Technology, Kumasi, Ghana.
(20)Faculty of Medicine, Algiers University, Algiers, Algeria.
(21)Department of Family and Community Medicine, Unaizah College of Medicine and Medical Sciences, Qassim University, Unaizah, Saudi Arabia.
(22)Family Health Department, High Institute of Public Health, Alexandria University, Alexandria, Egypt. nohaosama@alexu.edu.eg.
The coronavirus disease 2019 (COVID-19) pandemic is a global threat, challenging health services' provision and utilization. This study aimed to assess compulsory vaccination coverage in 12 Sub-Saharan African countries two years following the COVID-19 pandemic using the Health Belief Model. A cross-sectional survey was conducted from November 1 to December 15, 2022. Multivariate logistic regression was conducted to identify the determinants of vaccination coverage. Among the 5032 respondents, 73.1% reported that their children received compulsory vaccination. The lowest coverage was observed in Ghana (36.5%), while the highest was in Burkina Faso and Congo (92.0%). Factors associated with non-vaccination included older mothers (adjusted odds ratio (AOR) = 1.04, 95%CI: 1.03-1.05), lower mothers' education, older children (AOR = 0.76, 95%CI: 0.60-0.96), children with chronic illnesses (AOR = 0.55, 95%CI: 0.45-0.66), and difficult accessibility to healthcare facilities (AOR = 11.27, 95%CI: 9.48-13.44). Low perceived risk, in which non-vaccinated children were believed to be at no higher risk for infectious diseases and the disease severity would not worsen among non-vaccinated children, increased the likelihood of non-vaccination (AOR = 2.29, 95%CI: 1.75-2.99 and AOR = 2.12, 95%CI: 1.64-2.73, respectively). Perceiving vaccines as unnecessary, and needless for breastfed babies increased the probability of non-vaccination (AOR = 1.38, 95%CI: 1.10-1.73 and AOR = 1.69, 95%CI: 1.31-2.19, respectively). Higher odds of non-vaccination were found when the provision of vaccine information did not motivate parents to vaccinate their children (AOR = 4.29, 95%CI: 3.15-5.85). Conversely, believing that vaccines were safe for children decreased the odds of non-vaccination (AOR = 0.72, 95%CI: 0.58-0.88). Parental perceptions and concerns should be considered in interventions aiming to increase compulsory vaccine acceptance and coverage.

95. Res Sq. 2023 Aug 24:rs.3.rs-3210670. doi: 10.21203/rs.3.rs-3210670/v1. Preprint.
Alcohol Consumption Modifies Susceptibility to HIV-1 Entry in Cervical Mucosa-Derived CD4+ T cells of Women Resident in a Fishing Community of Lake Victoria, Uganda.
Bernard Ssentalo B(1), Driciru E(1), Fahad M(1), Nantongo M(1), Namuleme M(2), Kitandwe PK(2), Ssekayita EM(2), Galiwango R(3), Mirembe V(2), Muwenda BK(2), Muwanga M(4), Kayongo A(1), Lutwama F(1).
Author information:

(1)Makerere University.
(2)UVRI-IAVI-HIV Vaccine Program.
(3)Rakai Health Sciences Program.
(4)Entebbe General Referral Hospital.
BACKGROUND: A significant overlap exists in the burden of Alcohol Use Disorders (AUDs) and the HIV epidemic in Sub-Saharan Africa. Over 60% of HIV infections occur in women, mostly through the cervical mucosa. Absorption and systemic circulation of alcohol induces global physiological and immune effects, including at the genital mucosa. Alcohol alters expression of cell surface receptors, mucosal barrier permeability, inflammatory responses, and lymphocyte trafficking and homing. However, a substantial knowledge gap exists on whether these cellular and or immunological effects of alcohol modify the consumers' CD4+ T cell susceptibility to HIV-1 entry at the cervical mucosa. HIV seronegative women, aged 18-49 years were recruited from Kasenyi and Kigungu fish landing sites of Lake Victoria. They were categorized as Alcohol Consumers (n=27) or non-Alcohol Consumers (n=26) based on the World Health Organization Alcohol-Use-Disorder-Test (WHO-AUDIT) at a cut-off score of >=8/40 and <8/40, respectively. Cytobrush-collected Cervical Mononuclear Cells [CMCs] and Peripheral Blood Mononuclear Cells [PBMCs] from heparinized whole-blood were surface stained for CD4+ T cell immunophenotyping. To measure susceptibility to HIV entry, CMCs and PBMCs were co-cultured overnight with equal amount of GFP-tagged HIV-1 pseudo-virus particles. Both immunophenotyping and HIV entry were measured on a BD LSR II flow cytometer.
RESULTS: There was no significant difference in the frequency of CD4+ T cells in blood (p=0.451) or mucosa (p=0.838) compartments across study groups. However, we observed a combined four-fold higher HIV entry (p=0.0001) into cervical versus blood-derived CD4+ T cells regardless of alcohol consumption status. More so, cervical-derived CD4+ T cells of alcohol-consumers showed a two-fold increase in susceptibility to HIV entry (P=0.0185) compared to the non-alcohol consumer group. Double positive α4β7+CD4+T cells of alcohol consumers exhibited a higher HIV entry compared to those from alcohol non-consumers(p=0.0069).
CONCLUSION: This study demonstrates that cervical CD4+ T cells are more susceptible to HIV entry than those from blood. Also, cervical CD4+ T cells of alcohol consumers are more susceptible than those of non-consumers. Differences in frequencies of α4β7+ CD4+ T between alcohol consumers and non-consumers' cells may account for the increased susceptibility to HIV entry.

96. Biomolecules. 2023 Aug 18;13(8):1267. doi: 10.3390/biom13081267.
High-Throughput Antibody Profiling Identifies Targets of Protective Immunity against P. falciparum Malaria in Thailand.
Hassan I(1), Kanoi BN(2), Nagaoka H(1), Sattabongkot J(3), Udomsangpetch R(4), Tsuboi T(5), Takashima E(1).
Author information:
(1)Division of Malaria Research, Proteo-Science Center, Ehime University, Matsuyama 790-8577, Japan.
(2)Centre for Malaria Elimination, Institute of Tropical Medicine, Mount Kenya University, Thika 01000, Kenya.

(3)Mahidol Vivax Research Unit, Faculty of Tropical Medicine, Mahidol University, Bangkok 10400, Thailand.
(4)Center for Research and Innovation, Faculty of Medical Technology, Mahidol University, Salaya, Nakhon Pathom 73170, Thailand.
(5)Division of Cell-Free Sciences, Proteo-Science Center, Ehime University, Matsuyama 790-8577, Japan.
Malaria poses a significant global health challenge, resulting in approximately 600,000 deaths each year. Individuals living in regions with endemic malaria have the potential to develop partial immunity, thanks in part to the presence of anti-plasmodium antibodies. As efforts are made to optimize and implement strategies to reduce malaria transmission and ultimately eliminate the disease, it is crucial to understand how these interventions impact naturally acquired protective immunity. To shed light on this, our study focused on assessing antibody responses to a carefully curated library of P. falciparum recombinant proteins (n = 691) using samples collected from individuals residing in a low-malaria-transmission region of Thailand. We conducted the antibody assays using the AlphaScreen system, a high-throughput homogeneous proximity-based bead assay that detects protein interactions. We observed that out of the 691 variable surface and merozoite stage proteins included in the library, antibodies to 268 antigens significantly correlated with the absence of symptomatic malaria in an univariate analysis. Notably, the most prominent antigens identified were P. falciparum erythrocyte membrane protein 1 (PfEMP1) domains. These results align with our previous research conducted in Uganda, suggesting that similar antigens like PfEMP1s might play a pivotal role in determining infection outcomes in diverse populations. To further our understanding, it remains critical to conduct functional characterization of these identified proteins, exploring their potential as correlates of protection or as targets for vaccine development.

97. PLOS Glob Public Health. 2023 Aug 16;3(8):e0001566. doi: 10.1371/journal.pgph.0001566. eCollection 2023.
Proportion of Ugandans with pre-pandemic SARS-CoV-2 cross-reactive CD4+ and CD8+ T-cell responses: A pilot study.
Namuniina A(1), Muyanja ES(2), Biribawa VM(1), Okech BA(1), Ssemaganda A(3), Price MA(4)(5), Hills N(5)(6), Nanteza A(7), Bagaya BS(8)(9), Weiskopf D(10), Riou C(11)(12), Reynolds SJ(13)(14)(15), Galiwango RM(15), Redd AD(12)(13)(14).
Author information:
(1)UVRI-IAVI HIV Vaccine Program, Entebbe, Uganda.
(2)PATRU, School of Medicine, Emory University, Atlanta, Georgia, United States of America.
(3)Rady Faculty of Health Sciences, Department of Medical Microbiology and Infectious Diseases, Max Rady College of Medicine, University of Manitoba, Winnipeg, Manitoba, Canada.
(4)IAVI, New York, New York, United States of America.
(5)Department of Epidemiology and Biostatistics, University of California San Francisco, San Francisco, California, United States of America.
(6)Department of Neurology, University of California San Francisco, San

Francisco, California, United States of America.
(7)College of Veterinary Medicine, Animal Resources & Bio-security, Makerere University, Kampala, Uganda.
(8)Department of Immunology and Molecular Biology, College of Health Sciences, School of Biomedical Sciences, Makerere University, Kampala, Uganda.
(9)Integrated Biorepository of H3-Africa-Uganda (IBRH3AU), COVID-19 Biobank (COV-BANK), College of Health Sciences, Makerere University, Kampala, Uganda.
(10)Center for Infectious Disease and Vaccine Research, La Jolla Institute for Immunology, La Jolla, California, United States of America.
(11)Department of Pathology, Division of Medical Virology, University of Cape Town, Cape Town, South Africa.
(12)Institute of Infectious Disease and Molecular Medicine, University of Cape Town, Cape Town, South Africa.
(13)Division of Intramural Research, NIAID, NIH, Baltimore Maryland, United States of America.
(14)Division of Infectious Diseases, Johns Hopkins School of Medicine, Baltimore, Maryland, United States of America.
(15)Rakai Health Sciences Program, Kalisizo, Uganda.
The estimated mortality rate of the SARS-CoV-2 pandemic varied greatly around the world. In particular, multiple countries in East, Central, and West Africa had significantly lower rates of COVID-19 related fatalities than many resource-rich nations with significantly earlier wide-spread access to life-saving vaccines. One possible reason for this lower mortality could be the presence of pre-existing cross-reactive immunological responses in these areas of the world. To explore this hypothesis, an exploratory study of stored peripheral blood mononuclear cells (PBMC) from Ugandans collected from 2015-2017 prior to the COVID-19 pandemic (n = 29) and from hospitalized Ugandan COVID-19 patients (n = 3) were examined using flow-cytometry for the presence of pre-existing SARS-CoV-2 cross-reactive CD4+ and CD8+ T-cell populations using four T-cell epitope mega pools. Of pre-pandemic participants, 89.7% (26/29) had either CD4+ or CD8+, or both, SARS-CoV-2 specific T-cell responses. Specifically, CD4+ T-cell reactivity (72.4%) and CD8+ T-cell reactivity (65.5%) were relatively similar, and 13 participants (44.8%) had both types of cross-reactive types of T-cells present. There were no significant differences in response by sex in the population, however this may be in part due to the limited sample size examined. The rates of cross-reactive T-cell populations in this exploratory Ugandan population appears higher than previous estimates from resource-rich countries like the United States (20-50% reactivity). It is unclear what role, if any, this cross-reactivity played in decreasing COVID-19 related mortality in Uganda and other African countries, but does suggest that a better understanding of global pre-existing immunological cross-reactivity could be an informative data of epidemiological intelligence moving forward.

98. medRxiv. 2023 Aug 15:2023.08.09.23293903. doi: 10.1101/2023.08.09.23293903.
Divergent Neuroimmune Signatures in the Cerebrospinal Fluid Predict Differential Gender-Specific Survival Among Patients With HIV-Associated Cryptococcal Meningitis.

Okurut S(1)(2), Boulware DR(3), Okafor E(3), Rhein J(3), Kajumbula H(2), Bagaya B(4), Bwanga F(2), Olobo JO(4), Manabe YC(1)(5), Meya DB(1)(3)(6), Janoff EN(7)(8); ASTRO trial team.
Author information:
(1)Translation Sciences Laboratory, Research Department, Infectious Diseases Institute, Makerere University, Box 22418, Kampala, Uganda.
(2)Department of Medical Microbiology, School of Biomedical Sciences, College of Health Sciences, Makerere University, 7072, Kampala, Uganda.
(3)Division of Infectious Diseases and International Medicine, Department of Medicine, University of Minnesota, Minneapolis, MN, 55455, USA.
(4)Department of Immunology and Molecular Biology, School of Biomedical Sciences, College of Health Sciences, Makerere University, Box 7072, Kampala, Uganda.
(5)Division of Infectious Diseases, Department of Medicine, John Hopkins University School of Medicine, Baltimore, Maryland, MD, 21205, USA.
(6)Department of Medicine, School of Medicine, College of Health Sciences, Makerere University, 7072, Kampala, Uganda.
(7)Mucosal and Vaccine Research Program Colorado, Department of Medicine, Division of Infectious Diseases, University of Colorado Denver, Aurora, Colorado, 80045, USA.
(8)Denver Veterans Affairs Medical Center, Denver CO, 80045, USA.
Update in
Front Immunol. 2023 Dec 13;14:1275443.
Survival among people with HIV-associated cryptococcal meningitis (CM) remains low, exceptionally among women with the increased threat of death on current optimal use of antifungal drugs. Cryptococcus dissemination into the central nervous system (CNS) prompts a neuroimmune reaction to activate pathogen concomitant factors. However, no consistent diagnostic or prognostic immune-mediated signature is reported to underpin the risk of death or mechanism to improve treatment or survival. We theorized that the distinct neuroimmune cytokine or chemokine signatures in the cerebrospinal fluid (CSF), distinguish survivors from people who died on antifungal treatment, who may benefit from tailored therapy. We considered the baseline clinical disease features, cryptococcal microbiologic factors, and CSF neuroimmune modulated signatures among 419 consenting adults by gender (biological sex assigned at birth) (168 females and 251 males) by 18 weeks of survival on antifungal management. Survival at 18 weeks was inferior among females than males (47% vs. 59%; hazard ratio HR=1.4, 95% CI: 1.0 to 1.9, and p=0.023). Unsupervised principal component analysis (PCA) demonstrated the divergent neuroimmune signatures by gender, survival, and intragender-specific survival. Overall, females displayed lower levels of PD-L1, IL-1RA, and IL-15 than males (all p≤0.028). Female survivors compared with those who died, expressed significant fold elevations in levels of CSF (CCL11 - myeloid and CXCL10 - lymphoid chemokine (in both p=0.001), and CSF Th1, Th2, and Th17 cytokines. In contrast, male survivors expressed distinctly lower levels of CSF IL-15 and IL-8 compared with those who died. Survivors of either gender demonstrated a significant increase in the levels of immune regulatory element, IL-10. In the finale, we classified divergent neuroimmune

key signatures in CSF by gender, survival, and intragender-specific survival among people with HIV-associated cryptococcal meningitis. These intragender-specific survival associated-neuroimmune signatures, suggests the discrete role of gender immune regulating mechanisms as the possible targets for interventions to advance therapy to improve survival among people with HIV-associated cryptococcal meningitis.

99. Am J Respir Crit Care Med. 2023 Aug 15;208(4):442-450. doi: 10.1164/rccm.202302-0289OC.
Unmet Diagnostic and Therapeutic Opportunities for Chronic Obstructive Pulmonary Disease in Low- and Middle-Income Countries.
Florman KEH(1), Siddharthan T(2), Pollard SL(3)(4), Alupo P(5), Barber JA(6), Chandyo RK(7), Flores-Flores O(8)(9), Kirenga B(5), Mendes RG(10), Miranda JJ(11)(12), Mohan S(13), Ricciardi F(6), Rykiel NA(3)(4), Sharma AK(14), Wosu AC(3)(4), Checkley W(3)(4), Hurst JR(15); Additional GECo Study Investigators.
Collaborators: Cárdenas MK, Kumar Das S, Hossen S, Kalyesubula R, Mawanda D, Nassali F, Quaderi SA, Robertson N, Shrestha L, Yogi KN.
Author information:
(1)Department of Respiratory Medicine, Royal Free London NHS Foundation Trust, London, United Kingdom.
(2)Division of Pulmonary and Critical Care, Miller School of Medicine, University of Miami, Miami, Florida.
(3)Center for Global Non-Communicable Disease Research and Training and.
(4)Division of Pulmonary and Critical Care, School of Medicine, Johns Hopkins University, Baltimore, Maryland.
(5)Makerere Lung Institute, Makerere University, Kampala, Uganda.
(6)Department of Statistical Science, University College London, London, United Kingdom.
(7)Department of Community Medicine, Kathmandu Medical College, Kathmandu, Nepal.
(8)Facultad de Medicina Humana, Centro de Investigación del Envejecimiento, Universidad de San Martin de Porres, Lima, Peru.
(9)Facultad de Ciencias de la Salud, Universidad Científica del Sur, Lima, Peru.
(10)Cardiopulmonary Physiotherapy Laboratory, Federal University of Sao Carlos, Sao Carlos, Brazil.
(11)CRONICAS Centre of Excellence in Chronic Diseases and.
(12)Department of Medicine, School of Medicine, Universidad Peruana Cayetano Heredia, Lima, Peru.
(13)Centre for Health Economics, University of York, York, United Kingdom.
(14)Child Health Research Project, Institute of Medicine, Tribhuvan University, Kathmandu, Nepal; and.
(15)UCL Respiratory, University College London, London, United Kingdom.
Comment in
Am J Respir Crit Care Med. 2023 Aug 15;208(4):352-354.
Am J Respir Crit Care Med. 2023 Nov 15;208(10):1138-1139.
Rationale: Chronic obstructive pulmonary disease (COPD) is a prevalent and

burdensome condition in low- and middle-income countries (LMICs). Challenges to better care include more effective diagnosis and access to affordable interventions. There are no previous reports describing therapeutic needs of populations with COPD in LMICs who were identified through screening. Objectives: To describe unmet therapeutic need in screening-detected COPD in LMIC settings. Methods: We compared interventions recommended by the international Global Initiative for Chronic Obstructive Lung Disease COPD strategy document, with that received in 1,000 people with COPD identified by population screening at three LMIC sites in Nepal, Peru, and Uganda. We calculated costs using data on the availability and affordability of medicines. Measurement and Main Results: The greatest unmet need for nonpharmacological interventions was for education and vaccinations (applicable to all), pulmonary rehabilitation (49%), smoking cessation (30%), and advice on biomass smoke exposure (26%). Ninety-five percent of the cases were previously undiagnosed, and few were receiving therapy (4.5% had short-acting β-agonists). Only three of 47 people (6%) with a previous COPD diagnosis had access to drugs consistent with recommendations. None of those with more severe COPD were accessing appropriate maintenance inhalers. Even when available, maintenance treatments were unaffordable, with 30 days of treatment costing more than a low-skilled worker's daily average wage. Conclusions: We found a significant missed opportunity to reduce the burden of COPD in LMIC settings, with most cases undiagnosed. Although there is unmet need in developing novel therapies, in LMICs where the burden is greatest, better diagnosis combined with access to affordable interventions could translate to immediate benefit.

100. BMC Health Serv Res. 2023 Aug 14;23(1):856. doi: 10.1186/s12913-023-09875-w.
Comparing full immunisation status of children (0-23 months) between slums of Kampala City and the rural setting of Iganga District in Uganda: a cross-sectional study.
Jammeh A(1)(2), Muhoozi M(3), Kulane A(4), Kajungu D(3)(5).
Author information:
(1)Department of Global Public Health, Karolinska Institutet, Stockholm, Sweden. awajammeh56@gmail.com.
(2)Edward Francis Small Teaching Hospital, Banjul, The Gambia. awajammeh56@gmail.com.
(3)Center for Health and Population Research, Makerere University, Kampala, Uganda.
(4)Department of Global Public Health, Karolinska Institutet, Stockholm, Sweden.
(5)Division of Epidemiology and Biostatistics, Department of Global Health, Faculty of Medicine and Health Sciences, Stellenbosch University, Stellenbosch, South Africa.
BACKGROUND: Immunisation remains the most cost-effective public health intervention in preventing morbidity and mortality due to Vaccine-Preventable Diseases (VPDs). The study aims to compare the differences in immunisation coverage amongst children aged 0 to 23 months living in slums of Kampala city and Iganga as rural districts in Uganda.
METHODS: This study utilises data from a cross-sectional survey done in 2019 in

the slums of Kampala City and the rural district of Iganga within the Health and Demographic Surveillance Site (HDSS). It included 1016 children aged 0-23 months and their parents. A logistic regression model was used to analyse the relationship between multiple independent variables and the binary dependent variables (fully immunised) using Stata statistical software. The measures of association were odds ratios reported with a corresponding 95% confidence interval.
RESULTS: Out of the 1016 participants, 544 participants live in the rural area and 472 participants in the slums. Slums had 48.9% (n = 231) of fully immunised children whilst rural areas had 43.20% (n = 235). The multivariate analysis showed that children living in slums are more likely to be fully immunised as compared to their counterparts in rural areas (Odds ratio:1.456; p = 0.033; CI:1.030-2.058). Immunisation coverage for BCG (98.9%), Polio 0 (88.2%), Penta1 (92.7%), and Pneumo1 (89.8%) were high in both settlements. However, the dropout rate for subsequent vaccines was high 17%, 20% and 41% for Penta, pneumococcal and rota vaccines respectively. There was poor uptake of the new vaccines with slums having 73.4% and 47.9% coverage for pneumococcal and rota vaccines respectively and rural areas had 72.1% and 7.5% for pneumococcal and rota vaccines respectively.
CONCLUSION: The low full immunisation status in this study was attributed to the child's residence and the occupation of the parents. Lack of education and poor access to messages on immunisation (inadequate access to mass media) are other contributing factors. Educational messages on the importance of immunisation targeting these underserved populations will improve full immunisation coverage.

101. PLoS Negl Trop Dis. 2023 Aug 10;17(8):e0011496. doi: 10.1371/journal.pntd.0011496. eCollection 2023 Aug.
Soil-transmitted helminths: A critical review of the impact of co-infections and implications for control and elimination.
Lebu S(1), Kibone W(2), Muoghalu CC(1), Ochaya S(3)(4)(5), Salzberg A(1), Bongomin F(3), Manga M(1).
Author information:
(1)The Water Institute at UNC, Department of Environmental Sciences and Engineering, Gillings School of Public Health, University of North Carolina at Chapel Hill, Chapel Hill, North Carolina, United States of America.
(2)School of Medicine, College of Health Sciences, Makerere University, Kampala, Uganda.
(3)Department of Microbiology and Immunology, Faculty of Medicine, Gulu University, Gulu, Uganda.
(4)Department of Biology, Faculty of Science, Gulu University, Gulu, Uganda.
(5)Department of Clinical Pathology, Uppsala Academic Hospital, Uppsala, Sweden.
Researchers have raised the possibility that soil-transmitted helminth (STH) infections might modify the host's immune response against other systemic infections. STH infections can alter the immune response towards type 2 immunity that could then affect the likelihood and severity of other illnesses. However, the importance of co-infections is not completely understood, and the impact and direction of their effects vary considerably by infection. This review

synthesizes evidence regarding the relevance of STH co-infections, the potential mechanisms that explain their effects, and how they might affect control and elimination efforts. According to the literature reviewed, there are both positive and negative effects associated with STH infections on other diseases such as malaria, human immunodeficiency virus (HIV), tuberculosis, gestational anemia, pediatric anemia, neglected tropical diseases (NTDs) like lymphatic filariasis, onchocerciasis, schistosomiasis, and trachoma, as well as Coronavirus Disease 2019 (COVID-19) and human papillomavirus (HPV). Studies typically describe how STHs can affect the immune system and promote increased susceptibility, survival, and persistence of the infection in the host by causing a TH2-dominated immune response. The co-infection of STH with other diseases has important implications for the development of treatment and control strategies. Eliminating parasites from a human host can be more challenging because the TH2-dominated immune response induced by STH infection can suppress the TH1 immune response required to control other infections, resulting in an increased pathogen load and more severe disease. Preventive chemotherapy and treatment are currently the most common approaches used for the control of STH infections, but these approaches alone may not be adequate to achieve elimination goals. Based on the conclusions drawn from this review, integrated approaches that combine drug administration with water, sanitation and hygiene (WASH) interventions, hygiene education, community engagement, and vaccines are most likely to succeed in interrupting the transmission of STH co-infections. Gaining a better understanding of the behavior and relevance of STH co-infections in the context of elimination efforts is an important intermediate step toward reducing the associated burden of disease.

102. PLoS One. 2023 Aug 4;18(8):e0287516. doi: 10.1371/journal.pone.0287516.
Increased frequency and quantity of mucosal and plasma cytomegalovirus replication among Ugandan Adults Living with HIV.
McClymont E(1)(2)(3), Bone J(3)(4), Orem J(5), Okuku F(5), Kalinaki M(5), Saracino M(6), Huang ML(6)(7), Selke S(6), Wald A(6)(7)(8)(9), Corey L(6)(7)(8), Casper C(6)(7)(8)(9), Boucoiran I(10), Johnston C(7)(8), Gantt S(11).
Author information:
(1)Department of Pediatrics, University of British Columbia, Vancouver, Canada.
(2)CIHR Canadian HIV Trials Network, Vancouver, Canada.
(3)Department of Obstetrics and Gynecology, University of British Columbia, Vancouver, Canada.
(4)British Columbia Children's Hospital Research Institute, Vancouver, Canada.
(5)Uganda Cancer Institute, Mulago Hospital, Makerere University, Kampala, Uganda.
(6)Department of Laboratory Medicine, University of Washington, Seattle, Washington, United States of America.
(7)Vaccine and Infectious Disease Division, Fred Hutchinson Cancer Research Center, Seattle, Washington, United States of America.
(8)Department of Medicine, University of Washington, Seattle, Washington, United States of America.
(9)Department of Epidemiology, University of Washington, Seattle, Washington,

United States of America.
(10)Département d'Obstétrique-Gynécologie, Université de Montréal, Montréal, Canada.
(11)Département de Microbiologie, Infectiologie et Immunologie, Université de Montréal, Montréal, Canada.
BACKGROUND: Co-infection with HIV can result in impaired control of cytomegalovirus (CMV) replication, increasing the likelihood of disease and onward transmission. The objective of this analysis was to measure the impact of HIV on CMV replication in an intensively-sampled cohort in Kampala, Uganda.
METHODS: CMV seropositive men and women aged 18-65, with or without HIV co-infection, were followed for one month. Daily oral swabs and weekly anogenital swabs and plasma were collected. Quantitative CMV PCR was performed on all samples.
RESULTS: Eighty-five participants were enrolled and provided ≥1 oral swab; 43 (51%) were HIV-seropositive. People living with HIV (PLWH; median CD4 count 439 cells/mm3; none on antiretrovirals) had 2-4 times greater risk of CMV detection at each anatomical site assessed. At the oral site, 773 of 1272 (61%) of samples from PLWH had CMV detected, compared to 214 of 1349 (16%) among people without HIV. Similarly, the mean CMV quantity was higher among PLWH at all anatomical sites, with the largest difference seen for oral swabs (mean difference 1.63 log/mL; 95% CI 1.13-2.13). Among PLWH, absolute quantity of CD4+ T-cells was not associated with risk of CMV detection. HIV plasma RNA quantity was positively correlated with oral CMV shedding frequency, but not detection at other sites.
CONCLUSIONS: Mucosal and systemic CMV replication occurs at higher levels in PLWH than people without HIV, particularly oral shedding, which is a major mode of CMV transmission. Increased CMV replication despite relatively preserved CD4+ T-cell counts suggests that additional interventions are required to improve CMV control in PLWH.

103. Virol J. 2023 Aug 2;20(1):172. doi: 10.1186/s12985-023-02143-7.
Sabin polio virus protein 1 (VP1) evolution in patients with acute flaccid paralysis from 2010 to 2016 in Uganda.
Nanteza MB(1), Bakamutumaho B(2), Tushabe P(2), Namuwulya P(2), Birungi M(2), Dhatemwa R(2), Eliku JP(2), Tibanagwa M(2), Kakooza P(3), Bukenya H(2), Bwogi J(2), Byabamazima CR(4).
Author information:
(1)Uganda Virus Research Institute, Plot 51-59 Nakiwogo Road, P. O. Box 49, Entebbe, Uganda. marybridgetnanteza@yahoo.com.
(2)Uganda Virus Research Institute, Plot 51-59 Nakiwogo Road, P. O. Box 49, Entebbe, Uganda.
(3)WHO Country Office, P. O. Box 24578, Kampala, Uganda.
(4)World Health Organization AFRO, East and Southern Africa (ESA), 82-86 Enterprise Road, Highlands, Belvedere, P. O. Box BE 773, Harare, Zimbabwe.
Acute flaccid paralysis (AFP) is a rare side effect of the oral polio vaccine but can be associated with outbreaks and permanent disability in patients harboring circulating vaccine-derived polioviruses (cVDPVs). With the advancement of polio abolition in a glimpse, cVDPVs are causing outbreaks and

slowing the polio eradication process. The polio virus protein 1 (VP1) contains the binding site that is key for virus transmission. Understanding the evolution of VP1 among AFP patients could yield more insight into the early events of cVDPVs. Polioviruses were identified from stool specimens of AFP patients using cell culture; and confirmed by the real time RT PCR intra-typic differentiation and vaccine-derived poliovirus assays. Seventy-nine (79) Sabin-like poliovirus 1 (SL1) and 86 Sabin-like poliovirus 3 (SL3) were sequenced. The VP1 amino acid substitutions T106A in Sabin poliovirus 1 and A54V in Sabin poliovirus 3 were common among the AFP patients as has been found in previous studies. Other substitutions that were associated with AFP were: T290A and A54T in SL1 and SL3 respectively. Nucleotide mutations that were common among the AFP patients included T402C, C670A, and T816C in SL1, and G22A, C375Y, A472R, and A694T in SL3 polioviruses. Characterizing mutations that are associated with AFP could contribute to efforts pursued to mitigate the risk of vaccine-derived polioviruses and promote development of safer vaccines.

104. JHEP Rep. 2023 Apr 24;5(8):100777. doi: 10.1016/j.jhepr.2023.100777.
Enhancing interventions for prevention of mother-to-child- transmission of hepatitis B virus.
Matthews PC(1)(2)(3), Ocama P(4), Wang S(5)(6), El-Sayed M(7), Turkova A(8), Ford D(8), Torimiro J(9)(10), Garcia Ferreira AC(11), Espinosa Miranda A(11), De La Hoz Restrepo FP(12), Seremba E(4), Mbu R(10), Pan CQ(13), Razavi H(14), Dusheiko G(15), Spearman CW(16), Hamid S(17).
Author information:
(1)The Francis Crick Institute, 1 Midland Road, London, NW1 1AT, UK.
(2)Division of Infection and Immunity, University College London, Gower St, London WC1E 6BT, UK.
(3)Department of Infection, University College London Hospitals, 235 Euston Rd, London NW1 2BU, UK.
(4)Department of Medicine, Makerere University College of Health Sciences, Kampala, Uganda.
(5)Cooperman Barnabas Medical Center, Florham Park, NJ, USA.
(6)Hepatitis B Foundation, Doylestown, PA, USA.
(7)Department of Paediatrics, Faculty of Medicine, Ain Shams University, Cairo, Egypt.
(8)Medical Research Council Clinical Trials Unit, University College London, 90 High Holborn, London WC1V 6LJ, UK.
(9)Chantal Biya International Reference Centre for Research on Prevention and Management of HIV/AIDS (CIRCB), Yaounde, Cameroon.
(10)Faculty of Medicine and Biomedical Sciences, University of Yaounde, Yaounde, Cameroon.
(11)Ministry of Health, Health Surveillance Department, Department of Chronic Diseases and Sexually Transmitted Infections, SRTVN Quadra 701, Lote D, PO700 Building, CEP: 70719-040, Brasília/DF, Brazil.
(12)Universidad Nacional de Colombia, Bogotá, Colombia.
(13)Division of Gastroenterology and Hepatology, NYU Langone Health, NYU Grossman School of Medicine, NY, USA.

(14)Center for Disease Analysis Foundation, 1120 W South Boulder Rd Suite 102, Lafayette, CO 80026, USA.
(15)Liver Unit, King's College Hospital, Denmark Hill, London SE5 9RS, UK.
(16)Division of Hepatology, Department of Medicine, Faculty of Health Sciences, University of Cape Town, South Africa.
(17)Department of Medicine, Aga Khan University, Karachi, Pakistan.
Prevention of mother-to-child transmission of hepatitis B virus (HBV) infection is a cornerstone of efforts to support progress towards elimination of viral hepatitis. Current guidelines recommend maternal screening, antiviral therapy during the third trimester of high-risk pregnancies, universal and timely HBV birth dose vaccination, and post-exposure prophylaxis with hepatitis B immunoglobulin for selected neonates. However, serological and molecular diagnostic testing, treatment and HBV vaccination are not consistently deployed, particularly in many high endemicity settings, and models predict that global targets for reduction in paediatric incidence will not be met by 2030. In this article, we briefly summarise the evidence for current practice and use this as a basis to discuss areas in which prevention of mother-to-child transmission can potentially be enhanced. By reducing health inequities, enhancing pragmatic use of resources, filling data gaps, developing advocacy and education, and seeking consistent investment from multilateral agencies, significant advances can be made to further reduce vertical transmission events, with wide health, societal and economic benefits.

105. Hum Vaccin Immunother. 2023 Aug 1;19(2):2240690. doi: 10.1080/21645515.2023.2240690.
A phase I COVID-19 vaccine trial among SARS-CoV-2 seronegative and seropositive individuals in Uganda utilizing a self-amplifying RNA vaccine platform: Screening and enrollment experiences.
Kitonsa J(1), Kamacooko O(1), Ruzagira E(1), Nambaziira F(1), Abaasa A(1)(2), Serwanga J(1), Gombe B(1), Lunkuse J(1), Naluyinda H(1), Tukamwesiga N(1), Namata T(1), Kigozi A(1), Kafeero P(1), Basajja V(1), Joseph S(3), Pierce BF(3), Shattock R(3), Kaleebu P(1).
Author information:
(1)Medical Research Council/Uganda Virus Research Institute, London School of Hygiene and Tropical Medicine Uganda Research Unit, Entebbe, Uganda.
(2)Department of Infectious Disease Epidemiology, London School of Hygiene & Tropical Medicine, London, UK.
(3)Department of Infectious Disease, Imperial College London, London, UK.
We report the screening and enrollment process for a phase I vaccine trial in Masaka, Uganda that investigated the safety and immunogenicity of a self-amplifying SARS-CoV-2 RNA vaccine amongst individuals with and without antibodies to SARS-CoV-2. Participant screening and enrollment were conducted between December 2021 and April 2022. Individuals were eligible if they were aged between 18 and 45 years, healthy, and never vaccinated against COVID-19. SARS-CoV-2 antibody status was determined using two point-of-care rapid tests, i.e. Multi G (MGFT3) and Standard Q (Standard Q COVID-19 IgM/IgG Plus). Data were entered and managed in OpenClinica. Analyses were performed and presented

descriptively. A total of 212 individuals were screened and 43(20.3%) enrolled. The most common reasons for exclusion were ≥ grade 1 laboratory abnormalities (39, 18.4%), followed by discordant SARS-CoV-2 antibody results (23, 10.9%). While the first 38 participants were quickly enrolled over a period of 9 weeks, it took another 9 weeks to enroll the remaining five, as antibody negative participants became scarce during the surge of the Omicron variant. The SARS-CoV-2 antibody positivity rate was determined to be 60.8% and 84.4% in each half of the 18 months of screening respectively. The mean age (±Standard Deviation, SD) of screened and enrolled participants was 27.7 (±8.1) and 30.2 (±8.3) years respectively. We demonstrated that it is feasible to successfully screen and enroll participants for COVID-19 vaccine trials in Uganda in the time of a pandemic. Our experiences may be useful for investigators planning to undertake similar work in Africa.

106. Nature. 2023 Aug;620(7976):1025-1030. doi: 10.1038/s41586-023-06370-4.
Africa-specific human genetic variation near CHD1L associates with HIV-1 load.
McLaren PJ(#)(1)(2), Porreca I(#)(3), Iaconis G(#)(4), Mok HP(#)(4), Mukhopadhyay S(#)(5), Karakoc E(#)(3), Cristinelli S(6), Pomilla C(3), Bartha I(7)(8), Thorball CW(7)(8)(9), Tough RH(10)(11), Angelino P(8), Kiar CS(5), Carstensen T(3)(4), Fatumo S(12)(13), Porter T(3), Jarvis I(4), Skarnes WC(14), Bassett A(3), DeGorter MK(15), Sathya Moorthy MP(15)(16), Tuff JF(10), Kim EY(17), Walter M(17), Simons LM(17), Bashirova A(18)(19), Buchbinder S(20), Carrington M(18)(19)(21), Cossarizza A(22), De Luca A(23)(24), Goedert JJ(25), Goldstein DB(26), Haas DW(27), Herbeck JT(28), Johnson EO(29), Kaleebu P(30)(31), Kilembe W(32), Kirk GD(33), Kootstra NA(34), Kral AH(35), Lambotte O(36)(37), Luo M(11)(38), Mallal S(27)(39), Martinez-Picado J(40)(41)(42)(43), Meyer L(44)(45), Miro JM(43)(46), Moodley P(47), Motala AA(48), Mullins JI(49), Nam K(17), Obel N(50), Pirie F(48), Plummer FA(11), Poli G(51)(52), Price MA(53)(54), Rauch A(55), Theodorou I(56), Trkola A(57), Walker BD(21)(58), Winkler CA(59), Zagury JF(60), Montgomery SB(15)(16), Ciuffi A(6), Hultquist JF(17), Wolinsky SM(17), Dougan G(3)(4), Lever AML(4)(61), Gurdasani D(62)(63), Groom H(4), Sandhu MS(64)(65)(66), Fellay J(67)(68)(69).
Author information:
(1)Sexually Transmitted and Blood-Borne Infections Division at JC Wilt Infectious Diseases Research Centre, National Microbiology Laboratory Branch, Public Health Agency of Canada, Winnipeg, Manitoba, Canada. paul.mclaren@phac-aspc.gc.ca.
(2)Department of Medical Microbiology and Infectious Diseases, University of Manitoba, Winnipeg, Manitoba, Canada. paul.mclaren@phac-aspc.gc.ca.
(3)Wellcome Trust Sanger Institute, Hinxton, UK.
(4)Department of Medicine, University of Cambridge, Cambridge, UK.
(5)Peter Gorer Department of Immunobiology, School of Immunology and Microbial Sciences, King's College London, London, UK.
(6)Institute of Microbiology, Lausanne University Hospital and University of Lausanne, Lausanne, Switzerland.
(7)Global Health Institute, School of Life Sciences, École Polytechnique Fédérale de Lausanne, Lausanne, Switzerland.

(8)Swiss Institute of Bioinformatics, Lausanne, Switzerland.
(9)Precision Medicine Unit, Biomedical Data Science Center, Lausanne University Hospital (CHUV) and University of Lausanne, Lausanne, Switzerland.
(10)Sexually Transmitted and Blood-Borne Infections Division at JC Wilt Infectious Diseases Research Centre, National Microbiology Laboratory Branch, Public Health Agency of Canada, Winnipeg, Manitoba, Canada.
(11)Department of Medical Microbiology and Infectious Diseases, University of Manitoba, Winnipeg, Manitoba, Canada.
(12)The African Computational Genomics (TACG) Research Group, MRC/UVRI and LSHTM Uganda Research Unit, Entebbe, Uganda.
(13)Department of Non-Communicable Disease Epidemiology, Faculty of Epidemiology and Population Health, London School of Hygiene and Tropical Medicine, London, UK.
(14)The Jackson Laboratory for Genomic Medicine, Farmington, CT, USA.
(15)Department of Pathology, Stanford University School of Medicine, Stanford, CA, USA.
(16)Department of Genetics, Stanford University School of Medicine, Stanford, CA, USA.
(17)Division of Infectious Diseases, Feinberg School of Medicine, Northwestern University, Chicago, IL, USA.
(18)Basic Science Program, Frederick National Laboratory for Cancer Research, National Cancer Institute, Frederick, MD, USA.
(19)Laboratory of Integrative Cancer Immunology, Center for Cancer Research, National Cancer Institute, Bethesda, MD, USA.
(20)Bridge HIV, San Francisco Department of Public Health, San Francisco, CA, USA.
(21)Ragon Institute of MGH, MIT and Harvard, Boston, MA, USA.
(22)Department of Medical and Surgical Sciences for Children and Adults, University of Modena and Reggio Emilia, Modena, Italy.
(23)University Division of Infectious Diseases, Siena University Hospital, Siena, Italy.
(24)Department of Medical Biotechnologies, University of Siena, Siena, Italy.
(25)Epidemiology and Biostatistics Program, Division of Cancer Epidemiology and Genetics, National Cancer Institute, National Institutes of Health, Bethesda, MD, USA.
(26)Institute for Genomic Medicine, Columbia University, New York, NY, USA.
(27)Department of Medicine, Vanderbilt University School of Medicine, Nashville, TN, USA.
(28)Department of Global Health, University of Washington, Seattle, WA, USA.
(29)GenOmics and Translational Research Center and Fellow Program, RTI International, Research Triangle Park, NC, USA.
(30)Medical Research Council/Uganda Virus Research Institute & London School of Hygiene and Tropical Medicine, Uganda Research Unit, Entebbe, Uganda.
(31)London School of Hygiene and Tropical Medicine, London, UK.
(32)Center for Family Health Research-Zambia, Lusaka, Zambia.
(33)Department of Epidemiology, Johns Hopkins University, Baltimore, MD, USA.
(34)Department of Experimental Immunology, Amsterdam UMC, University of

Amsterdam, Amsterdam, The Netherlands.
(35)Community Health Research Division, RTI International, Berkeley, CA, USA.
(36)Université Paris Saclay, Inserm UMR1184, CEA, Le Kremlin-Bicêtre, France.
(37)APHP, Department of Clinical Immunology, Bicêtre Hospital, Le Kremlin-Bicêtre, France.
(38)Vaccine and Therapeutics Laboratory, Medical and Scientific Affairs, National Microbiology Laboratory Branch, Public Health Agency of Canada, Winnipeg, Manitoba, Canada.
(39)Institute for Immunology & Infectious Diseases, Murdoch University, Perth, Western Australia, Australia.
(40)University of Vic-Central University of Catalonia, Vic, Spain.
(41)IrsiCaixa AIDS Research Institute, Badalona, Spain.
(42)Catalan Institution for Research and Advanced Studies, Barcelona, Spain.
(43)CIBERINFEC, Instituto de Salud Carlos III, Madrid, Spain.
(44)INSERM U1018, Université Paris-Saclay, Le Kremlin Bicêtre, France.
(45)AP-HP, Hôpital de Bicêtre, Département d'Épidémiologie, Le Kremlin Bicêtre, France.
(46)Infectious Diseases Service, Hospital Clinic-Institut d'Investigacions Biomèdiques August Pi I Sunyer (IDIBAPS), University of Barcelona, Barcelona, Spain.
(47)National Health Laboratory Service, South Africa and University of KwaZulu-Natal, Durban, South Africa.
(48)Department of Diabetes and Endocrinology, School of Clinical Medicine, University of KwaZulu-Natal, Durban, South Africa.
(49)Department of Microbiology, University of Washington, Seattle, WA, USA.
(50)Department of Infectious Diseases, Copenhagen University Hospital, Rigshospitalet, Copenhagen, Denmark.
(51)Division of Immunology, Transplantation and Infectious Diseases, San Raffaele Scientific Institute, Milan, Italy.
(52)School of Medicine, Vita-Salute San Raffaele University, Milan, Italy.
(53)International AIDS Vaccine Initiative, New York, NY, USA.
(54)Department of Epidemiology and Biostatistics, University of California, San Francisco, CA, USA.
(55)Department of Infectious Diseases, Inselspital, Bern University Hospital, University of Bern, Bern, Switzerland.
(56)Laboratoire d'Immunologie, Hôpital Robert Debré Paris, Paris, France.
(57)Institute of Medical Virology, University of Zurich, Zurich, Switzerland.
(58)Howard Hughes Medical Institute, Chevy Chase, MD, USA.
(59)Basic Research Laboratory, Molecular Genetic Epidemiology Section, Frederick National Laboratory for Cancer Research and Cancer Innovative Laboratory, Center for Cancer Research, National Cancer Institute, Frederick, MD, USA.
(60)Laboratoire Génomique, Bioinformatique et Chimie Moléculaire, EA7528, Conservatoire National des Arts et Métiers, HESAM Université, Paris, France.
(61)Department of Medicine, National University of Singapore, Singapore, Singapore.
(62)Queen Mary University of London, London, UK.
(63)Kirby Institute, University of New South Wales, Sydney, New South Wales,

Australia.
(64)Department of Epidemiology & Biostatistics, School of Public Health, Imperial College London, London, UK. m.sandhu@imperial.ac.uk.
(65)MRC Centre for Environment and Health, School of Public Health, Imperial College London, London, UK. m.sandhu@imperial.ac.uk.
(66)Omnigen Biodata, Cambridge, UK. m.sandhu@imperial.ac.uk.
(67)Global Health Institute, School of Life Sciences, École Polytechnique Fédérale de Lausanne, Lausanne, Switzerland. jacques.fellay@epfl.ch.
(68)Swiss Institute of Bioinformatics, Lausanne, Switzerland. jacques.fellay@epfl.ch.
(69)Precision Medicine Unit, Biomedical Data Science Center, Lausanne University Hospital (CHUV) and University of Lausanne, Lausanne, Switzerland. jacques.fellay@epfl.ch.
(#)Contributed equally
Erratum in
Nature. 2023 Sep;621(7979):E42.
HIV-1 remains a global health crisis1, highlighting the need to identify new targets for therapies. Here, given the disproportionate HIV-1 burden and marked human genome diversity in Africa2, we assessed the genetic determinants of control of set-point viral load in 3,879 people of African ancestries living with HIV-1 participating in the international collaboration for the genomics of HIV3. We identify a previously undescribed association signal on chromosome 1 where the peak variant associates with an approximately 0.3 log10-transformed copies per ml lower set-point viral load per minor allele copy and is specific to populations of African descent. The top associated variant is intergenic and lies between a long intergenic non-coding RNA (LINC00624) and the coding gene CHD1L, which encodes a helicase that is involved in DNA repair4. Infection assays in iPS cell-derived macrophages and other immortalized cell lines showed increased HIV-1 replication in CHD1L-knockdown and CHD1L-knockout cells. We provide evidence from population genetic studies that Africa-specific genetic variation near CHD1L associates with HIV replication in vivo. Although experimental studies suggest that CHD1L is able to limit HIV infection in some cell types in vitro, further investigation is required to understand the mechanisms underlying our observations, including any potential indirect effects of CHD1L on HIV spread in vivo that our cell-based assays cannot recapitulate.

107. BMJ Paediatr Open. 2023 Aug;7(1):e001334. doi: 10.1136/bmjpo-2021-001334.
Antitetanus toxoid antibodies in mothers and neonates: a single-centre study from Uganda.
Mugagga N(1), Bagaya BS(2)(3), Nantongo M(4), Muwanda F(4), Mukunya D(5)(6), Musaba MW(7), Nakimuli AO(1), Musooko M(1), Sekikubo M(8).
Author information:
(1)Obstetrics and Gynaecology, Makerere University College of Health Sciences, Kampala, Uganda.
(2)Immunology and Molecular Biology, Makerere University College of Health Sciences, Kampala, Uganda bernard.bagaya@mak.ac.ug.
(3)Department of Research and Innovation, BMK Medical Laboratory Services,

Mityana, Uganda.
(4)Immunology and Molecular Biology, Makerere University College of Health Sciences, Kampala, Uganda.
(5)Community and Public Health, Busitema University, Mbale, Uganda.
(6)Department of Research, Nikao Medical Center, Kampala, Uganda.
(7)Department of Obstetrics and Gynaecology, Busitema University, Mbale, Uganda.
(8)Obstetrics and Gynaecology, College of Health Sciences, Makerere University, Kampala, Uganda.
BACKGROUND: Neonatal mortality due to tetanus persists in Uganda despite the mandatory vaccination of pregnant mothers. Maternal antibodies wane within a year. Uganda's maternal vaccination guidelines do not specify the timing or frequency of tetanus shots, contributing to suboptimal transfer of tetanus antibodies to neonates. We aimed to determine the prevalence and factors associated with protective tetanus antibodies among newborns at Kawempe National Referral Hospital.
METHODS: We conducted a cross-sectional study among 293 mother-newborn pairs. At delivery, neonatal cord and maternal venous blood were collected and titred for antitetanus antibodies using a quantitative ELISA kit. The primary outcome of the study was the proportion of newborn babies with tetanus antibodies ≥0.1 IU/mL. Associated factors were determined using generalised linear models for the Poisson family with a log link and robust variance estimation.
RESULTS: A total of 258/293 (88.1%) newborns had protective antibody titres. Factors associated with adequate protective antibodies in the newborn included: high (≥0.1 IU/mL) maternal antibody titres, first antenatal visit ≥12 weeks of gestation and receiving a tetanus toxoid (TT) shot ≥28 weeks of gestation. However, number of doses received before current pregnancy was not associated with adequate protective antibody titres.
CONCLUSION: There is a high prevalence of adequate protective levels of antibodies among TT-vaccinated mothers. Maternal titres and a third trimester TT dose correlate with adequate levels of protective anti-TT antibodies among newborns. A third trimester TT dose is recommended.

108. Curr Opin Virol. 2023 Aug;61:101339. doi: 10.1016/j.coviro.2023.101339. Epub
A review of broadly protective monoclonal antibodies to treat Ebola virus disease.
Rijal P(1), Donnellan FR(2).
Author information:
(1)Center for Translational Immunology, Chinese Academy of Medical Science Oxford Institute, Nuffield Department of Medicine, University of Oxford, UK; MRC Human Immunology Unit, MRC Weatherall Institute of Molecular Medicine, John Radcliffe Hospital, Headington, Oxford, OX3 9DS, United Kingdom. Electronic address: pramila.rijal@rdm.ox.ac.uk.
(2)Department of Biochemistry, Dorothy Crowfoot Hodgkin Building, South Parks Road, Oxford, OX1 3QU, United Kingdom; Kavli Institute for Nanoscience Discovery, University of Oxford, UK. Electronic address: francesca.donnellan@bioch.ox.ac.uk.
The filovirus vaccine and the therapeutic monoclonal antibody (mAb) research

have made substantial progress. However, existing vaccines and mAbs approved for
use in humans are specific to Zaire ebolavirus (EBOV). Since other
Ebolavirus species are a continuing threat to public health, the search for
broadly protective mAbs has drawn attention. Here, we review viral
glycoprotein-targeting mAbs that have proved their broader protective efficacy
in animal models. MBP134AF, the most advanced of these new-generation mAb
therapies, has recently been deployed in Uganda during the Sudan
ebolavirus outbreak. Furthermore, we discuss the measures associated with
enhancing antibody therapies and the risks associated with them, including the
rise of escape mutations following the mAb treatment and naturally occurring
EBOV variants.
109. Lancet Infect Dis. 2023 Aug;23(8):e288-e300. doi: 10.1016/S1473-3099(22)00878-7.
Opportunities and challenges of leveraging COVID-19 vaccine innovation and
technologies for developing sustainable vaccine manufacturing capabilities in
Africa.
Kana BD(1), Arbuthnot P(2), Botwe BK(3), Choonara YE(4), Hassan F(5), Louzir
H(6), Matsoso P(7), Moore PL(8), Muhairwe A(9), Naidoo K(10), Ndomondo-Sigonda
M(11), Madhi SA(12).
Author information:
(1)Department of Science and Innovation/National Research Foundation Centre of
Excellence for Biomedical Tuberculosis Research, Faculty of Health Sciences,
University of the Witwatersrand, Johannesburg, South Africa.
(2)South African Medical Research Council Antiviral Gene Therapy Research Unit,
Faculty of Health Sciences, University of the Witwatersrand, Johannesburg, South
Africa.
(3)BB Associates, Medicines Regulatory Consultants, Accra, Ghana.
(4)Wits Advanced Drug Delivery Platform Research Unit, Department of Pharmacy
and Pharmacology, Faculty of Health Sciences, University of the Witwatersrand,
Johannesburg, South Africa; African Network for Drugs and Diagnostics Innovation
Centre of Excellence in Advanced Drug Delivery, Faculty of Health Sciences,
University of the Witwatersrand, Johannesburg, South Africa.
(5)Health Justice Initiative, University of Cape Town School of Public Health
and Family Medicine, Cape Town, South Africa.
(6)Laboratory of Transmission, Control and Immunobiology of Infections
(LR11IPT02), Institut Pasteur de Tunis, University of Tunis El Manar, Tunis,
Tunisia.
(7)Health Regulatory Science Platform, Wits Health Consortium, Faculty of Health
Sciences, University of the Witwatersrand, Johannesburg, South Africa.
(8)South African Medical Research Council Antibody Immunity Research Unit,
Faculty of Health Sciences, University of the Witwatersrand, Johannesburg, South
Africa; National Institute for Communicable Diseases, Johannesburg, South
Africa.
(9)National Drug Authority, Kampala, Uganda.
(10)South African Medical Research Council Antiviral Gene Therapy Research Unit,
Faculty of Health Sciences, University of the Witwatersrand, Johannesburg, South
Africa; National Health Laboratory Service, Johannesburg, South Africa.
(11)Wits Advanced Drug Delivery Platform Research Unit, Department of Pharmacy

and Pharmacology, Faculty of Health Sciences, University of the Witwatersrand, Johannesburg, South Africa; African Union Development Agency-New Partnership for Africa's Development, Midrand, South Africa.
(12)South African Medical Research Council Vaccines and Infectious Diseases Analytics Research Unit, Faculty of Health Sciences, University of the Witwatersrand, Johannesburg, South Africa; African Leadership in Vaccinology Expertise, Faculty of Health Sciences, University of the Witwatersrand, Johannesburg, South Africa. Electronic address: shabir.madhi@wits.ac.za.
Comment in
Lancet Infect Dis. 2023 Aug;23(8):890-892.
The COVID-19 pandemic heralded unprecedented resource mobilisation and global scientific collaboration to rapidly develop effective vaccines. Regrettably, vaccine distribution has been inequitable, particularly in Africa where manufacturing capacity remains nominal. To address this, several initiatives are underway to develop and manufacture COVID-19 vaccines in Africa. Nevertheless, diminishing demand for COVID-19 vaccines, the cost competitiveness of producing goods locally, intellectual property rights issues, and complex regulatory environments among other challenges can undermine these ventures. We outline how extending COVID-19 vaccine manufacturing in Africa to include diverse products, multiple vaccine platforms, and advanced delivery systems will ensure sustainability. Possible models, including leveraging public-academic-private partnerships to enhance success of vaccine manufacturing capacity in Africa are also discussed. Intensifying research in vaccine discovery on the continent could yield vaccines that further bolster sustainability of local production, ensuring greater pandemic preparedness in resource-constrained environments, and long-term health systems security.

110. Lancet Infect Dis. 2023 Aug;23(8):965-973. doi: 10.1016/S1473-3099(23)00131-7.
Immunogenicity and safety of fractional doses of 17D-213 yellow fever vaccine in children (YEFE): a randomised, double-blind, non-inferiority substudy of a phase 4 trial.
Juan-Giner A(1), Namulwana ML(2), Kimathi D(3), Grantz KH(4), Fall G(5), Dia M(5), Bob NS(5), Sall AA(5), Nerima C(2), Sahani MK(2), Mulogo EM(6), Ampeire I(7), Hombach J(8), Nanjebe D(2), Mwanga-Amumpaire J(2), Cummings DAT(9), Bejon P(3), Warimwe GM(3), Grais RF(10).
Author information:
(1)Epicentre, Paris, France.
(2)Epicentre Mbarara Research Centre, Mbarara, Uganda.
(3)Kenya Medical Research Institute-Wellcome Trust Research Programme, Kilifi, Kenya; Centre for Tropical Medicine & Global Health, University of Oxford, Oxford, UK.
(4)Department of Biology and Emerging Pathogens Institute, University of Florida, Gainesville, FL, USA; Department of Epidemiology, Johns Hopkins Bloomberg School of Public Health, Baltimore, MD, USA.
(5)Institut Pasteur Dakar, Dakar, Senegal.
(6)Department of Community Health, Mbarara University of Science & Technology, Mbarara, Uganda.

(7)Expanded Program on Immunization, Ministry of Health, Kampala, Uganda.
(8)Immunization, Vaccines, and Biologicals, World Health Organization, Geneva, Switzerland.
(9)Department of Biology and Emerging Pathogens Institute, University of Florida, Gainesville, FL, USA.
(10)Epicentre, Paris, France. Electronic address: rebecca.grais@epicentre.msf.org.
Comment in
Lancet Infect Dis. 2023 Aug;23(8):889-890.
BACKGROUND: Current supply shortages constrain yellow fever vaccination activities, particularly outbreak response. Although fractional doses of all WHO-prequalified yellow fever vaccines have been shown to be safe and immunogenic in a randomised controlled trial in adults, they have not been evaluated in a randomised controlled trial in young children (9-59 months old). We aimed to assess the immunogenicity and safety of fractional doses compared with standard doses of the WHO-prequalified 17D-213 vaccine in young children.
METHODS: This substudy of the YEFE phase 4 study was conducted at the Epicentre Mbarara Research Centre (Mbarara, Uganda). Eligible children were aged 9-59 months without contraindications for vaccination, without history of previous yellow fever vaccination or infection and not requiring yellow fever vaccination for travelling. Participants were randomly assigned, using block randomisation, 1:1 to standard or fractional (one-fifth) dose of yellow fever vaccine. Investigators, participants, and laboratory personnel were blinded to group allocation. Participants were followed for immunogenicity and safety at 10 days, 28 days, and 1 year after vaccination. The primary outcome was non-inferiority in seroconversion (-10 percentage point margin) 28 days after vaccination measured by 50% plaque reduction neutralisation test (PRNT50) in the per-protocol population. Safety and seroconversion at 10 days and 12-16 months after vaccination (given COVID-19 resctrictions) were secondary outcomes. This study is registered with ClinicalTrials.gov, NCT02991495.
FINDINGS: Between Feb 20, 2019, and Sept 9, 2019, 433 children were assessed, and 420 were randomly assigned to fractional dose (n=210) and to standard dose (n=210) 17D-213 vaccination. 28 days after vaccination, 202 (97%, 95% CI 95-99) of 207 participants in the fractional dose group and 191 (100%, 98-100) of 191 in the standard dose group seroconverted. The absolute difference in seroconversion between the study groups in the per-protocol population was -2 percentage points (95% CI -5 to 1). 154 (73%) of 210 participants in the fractional dose group and 168 (80%) of 210 in the standard dose group reported at least one adverse event 28 days after vaccination. At 10 days follow-up, seroconversion was lower in the fractional dose group than in the standard dose group. The most common adverse events were upper respiratory tract infections (n=221 [53%]), diarrhoea (n=68 [16%]), rhinorrhoea (n=49 [12%]), and conjunctivitis (n=28 [7%]). No difference was observed in incidence of adverse events and serious adverse events between study groups.
CONCLUSIONS: Fractional doses of the 17D-213 vaccine were non-inferior to standard doses in inducing seroconversion 28 days after vaccination in children aged 9-59 months when assessed with PRNT50, but we found fewer children

seroconverted at 10 days. The results support consideration of the use of fractional dose of yellow fever vaccines in WHO recommendations for outbreak response in the event of a yellow fever vaccine shortage to include children.
FUNDING: Médecins Sans Frontières Foundation.

111. Lancet Infect Dis. 2023 Aug;23(8):956-964. doi: 10.1016/S1473-3099(23)00068-3.
Safety and immunogenicity of a ChAdOx1 vaccine against Rift Valley fever in UK adults: an open-label, non-randomised, first-in-human phase 1 clinical trial.
Jenkin D(1), Wright D(2), Folegatti PM(1), Platt A(1), Poulton I(1), Lawrie A(1), Tran N(1), Boyd A(1), Turner C(1), Gitonga JN(3), Karanja HK(3), Mugo D(3), Ewer KJ(1), Bowden TA(4), Gilbert SC(5), Charleston B(6), Kaleebu P(7), Hill AVS(1), Warimwe GM(8).
Author information:
(1)The Jenner Institute, University of Oxford, Oxford, UK.
(2)The Jenner Institute, University of Oxford, Oxford, UK; Department of Paediatrics, University of Oxford, Oxford, UK.
(3)Kenya Medical Research Institute-Wellcome Trust Research Programme, Kilifi, Kenya.
(4)Wellcome Centre for Human Genetics, Division of Structural Biology, University of Oxford, Oxford, UK.
(5)Chinese Academy of Medical Science Oxford Institute, University of Oxford, Oxford, UK; Pandemic Sciences Institute, University of Oxford, Oxford, UK.
(6)The Pirbright Institute, Pirbright, UK.
(7)Medical Research Council-Uganda Virus Research Institute and The London School of Hygiene & Tropical Medicine, Uganda Research Unit, Entebbe, Uganda.
(8)Centre for Tropical Medicine and Global Health, University of Oxford, Oxford, UK; Kenya Medical Research Institute-Wellcome Trust Research Programme, Kilifi, Kenya. Electronic address: gwarimwe@kemri-wellcome.org.
Comment in
Lancet Infect Dis. 2023 Aug;23(8):887-889.
BACKGROUND: Rift Valley fever is a viral epidemic illness prevalent in Africa that can be fatal or result in debilitating sequelae in humans. No vaccines are available for human use. We aimed to evaluate the safety and immunogenicity of a non-replicating simian adenovirus-vectored Rift Valley fever (ChAdOx1 RVF) vaccine in humans.
METHODS: We conducted a phase 1, first-in-human, open-label, dose-escalation trial in healthy adults aged 18-50 years at the Centre for Clinical Vaccinology and Tropical Medicine, Oxford, UK. Participants were required to have no serious comorbidities or previous history of receiving an adenovirus-based vaccine before enrolment. Participants were non-randomly allocated to receive a single ChAdOx1 RVF dose of either 5 × 109 virus particles (vp), 2·5 × 1010 vp, or 5 × 1010 vp administered intramuscularly into the deltoid of their non-dominant arm; enrolment was sequential and administration was staggered to allow for safety to be assessed before progression to the next dose. Primary outcome measures were assessment of adverse events and secondary outcome measures were Rift Valley fever neutralising antibody titres, Rift Valley fever GnGc-binding antibody titres (ELISA), and cellular response (ELISpot), analysed in all

participants who received a vaccine. This trial is registered with
ClinicalTrials.gov (NCT04754776).
FINDINGS: Between June 11, 2021, and Jan 13, 2022, 15 volunteers received a single dose of either 5 × 109 vp (n=3), 2·5 × 1010 vp (n=6), or 5 × 1010 vp (n=6) ChAdOx1 RVF. Nine participants were female and six were male. 14 (93%) of 15 participants reported solicited local adverse reactions; injection-site pain was the most frequent (13 [87%] of 15). Ten (67%) of 15 participants (from the 2·5 × 1010 vp and 5 × 1010 vp groups only) reported systemic symptoms, which were mostly mild in intensity, the most common being headache (nine [60%] of 15) and fatigue (seven [47%]). All unsolicited adverse events reported within 28 days were either mild or moderate in severity; gastrointestinal symptoms were the most common reaction (at least possibly related to vaccination), occurring in four (27%) of 15 participants. Transient decreases in total white cell, lymphocyte, or neutrophil counts occurred at day 2 in some participants in the intermediate-dose and high-dose groups. Lymphopenia graded as severe occurred in two participants in the 5 × 1010 vp group at a single timepoint, but resolved at the subsequent follow-up visit. No serious adverse events occurred. Rift Valley fever neutralising antibodies were detectable across all dose groups, with all participants in the 5 × 1010 vp dose group having high neutralising antibody titres that peaked at day 28 after vaccination and persisted through the 3-month follow-up. High titres of binding IgG targeting Gc glycoprotein were detected whereas those targeting Gn were comparatively low. IFNγ cellular responses against Rift Valley fever Gn and Gc glycoproteins were observed in all participants except one in the 5 × 1010 vp dose group. These IFNγ responses peaked at 2 weeks after vaccination, were highest in the 5 × 1010 vp dose group, and tended to be more frequent against the Gn glycoprotein.
INTERPRETATION: ChAdOx1 RVF was safe, well tolerated, and immunogenic when administered as a single dose in this study population. The data support further clinical development of ChAdOx1 RVF for human use.
FUNDING: UK Department of Health and Social Care through the UK Vaccines Network, Oak Foundation, and the Wellcome Trust.
TRANSLATION: For the Swahili translation of the abstract see Supplementary Materials section.

112. Int J Epidemiol. 2023 Aug;52(4):1150-1162. doi: 10.1093/ije/dyad005. Epub 2023
Field investigation of high reported non-neonatal tetanus burden in Uganda, 2016-2017.
Casey RM(1), Nguna J(2), Opar B(2), Ampaire I(2), Lubwama J(3), Tanifum P(4), Zhu BP(5), Kisakye A(6), Kabwongera E(7), Tohme RA(1), Dahl BA(1), Ridpath AD(1), Scobie HM(1).
Author information:
(1)Global Immunization Division, Centers for Disease Control and Prevention (CDC), Atlanta, GA, USA.
(2)Expanded Programme on Immunization, Ministry of Health, Kampala, Uganda.
(3)Division of Global HIV and Tuberculosis, Centers for Disease Control and Prevention, Kampala, Uganda.
(4)Global Immunization Division, Centers for Disease Control and Prevention,

Kampala, Uganda.
(5)Division of Global Health Protection, Centers for Disease Control and Prevention, Kampala, Uganda.
(6)World Health Organization, Country Office, Kampala, Uganda.
(7)UNICEF, Country Office, Kampala, Uganda.
BACKGROUND: Despite providing tetanus-toxoid-containing vaccine (TTCV) to infants and reproductive-age women, Uganda reports one of the highest incidences of non-neonatal tetanus (non-NT). Prompted by unusual epidemiologic trends among reported non-NT cases, we conducted a retrospective record review to see whether these data reflected true disease burden.
METHODS: We analysed nationally reported non-NT cases during 2012-2017. We visited 26 facilities (14 hospitals, 12 health centres) reporting high numbers of non-NT cases (n = 20) or zero cases (n = 6). We identified non-NT cases in facility registers during 1 January 2016-30 June 2017; the identified case records were abstracted.
RESULTS: During 2012-2017, a total of 24 518 non-NT cases were reported and 74% were ≥5 years old. The average annual incidence was 3.43 per 100 000 population based on inpatient admissions. Among 482 non-NT inpatient cases reported during 1 January 2016-30 June 2017 from hospitals visited, 342 (71%) were identified in facility registers, despite missing register data (21%). Males comprised 283 (83%) of identified cases and 60% were ≥15 years old. Of 145 cases with detailed records, 134 (92%) were clinically confirmed tetanus; among these, the case-fatality ratio (CFR) was 54%. Fourteen cases were identified at two hospitals reporting zero cases. Among >4000 outpatient cases reported from health centres visited, only 3 cases were identified; the remainder were data errors.
CONCLUSIONS: A substantial number of non-NT cases and deaths occur in Uganda. The high CFR and high non-NT burden among men and older children indicate the need for TTCV booster doses across the life course to all individuals as well as improved coverage with the TTCV primary series. The observed data errors indicate the need for data quality improvement activities.

113. PLoS One. 2023 Jul 28;18(7):e0289295. doi: 10.1371/journal.pone.0289295.
Prevalence and predictors of COVID-19 vaccination hesitancy among healthcare workers in Sub-Saharan Africa: A systematic review and meta-analysis.
Kigongo E(1), Kabunga A(2), Tumwesigye R(3), Musinguzi M(4), Izaruku R(5), Acup W(4).
Author information:
(1)Faculty of Public Health, Department of Environmental Health and Disease Control, Lira University, Lira, Uganda.
(2)Faculty of Medicine, Department of Psychiatry, Lira University, Lira, Uganda.
(3)Faculty of Nursing and Midwifery, Department of Nursing, Lira University, Lira, Uganda.
(4)Faculty of Public Health, Department of Community Health, Lira University, Lira, Uganda.
(5)Department of Library and Information Services, Lira University, Lira, Uganda.

BACKGROUND: The COVID-19 vaccination is regarded as an effective intervention for controlling the pandemic. However, COVID-19 vaccine hesitancy is hampering efforts geared towards reducing the burden of the pandemic. Therefore, examining COVID-19 hesitancy and its predictors among healthcare workers is essential to improving COVID-19 uptake. In sub-Saharan Africa, the pooled proportion of COVID-19 vaccine hesitancy is yet to be known.
PURPOSE: The present study was to estimate the pooled proportion of COVID-19 vaccine hesitancy and its predictors among healthcare workers in Sub-Saharan Africa.
METHODS: A systematic search of articles was conducted in PubMed, Science Direct, African Journal Online, and Google Scholar. Data was extracted with the help of Excel. Data analysis was conducted using STATA 17. Heterogeneity in the studies was assessed using Cochrane Q and 12 tests. A random effects model was used to examine the pooled estimates to determine if heterogeneity was exhibited.
RESULTS: A total of 15 studies involving 7498 participants were included in the final analysis. The pooled prevalence of COVID-19 vaccination hesitancy among healthcare workers was 46%, 95% CI (0.38-0.54). The predictors of COVID-19 hesitancy were negative beliefs towards vaccine 14.0% (OR = 1.05, 95% CI: 1.04, 1.06), perceived low risk of COVID-19 infection 24.0% (OR = 1.25, 95% CI: 1.23, 1.28), and vaccine side effects 25.0% (OR = 1.23, 95% CI: 1.21, 1.24).
CONCLUSION: The data revealed generally high hesitancy of COVID-19 vaccine among health workers in Sub-Saharan Africa. Future COVID-19 adoption and uptake should be improved by national and individual level efforts. In Sub-Saharan Africa, it is crucial to address the myths and obstacles preventing healthcare professionals from accepting the COVID-19 vaccination as soon as feasible since their willingness to get the vaccine serves as an important example for the broader public.

114. Epidemiol Infect. 2023 Jul 25;151:e142. doi: 10.1017/S0950268823001164.
COVID-19 second wave and clinical characteristics of cases in Uganda: A retrospective cross-sectional survey of confirmed SARS-CoV-2 cases, March-June 2021.
Walekhwa AW(1)(2), Nakazibwe B(1)(2), Nantongo M(1), Wafula ST(2), Bulafu D(2), Ayugi B(2), Nankabirwa C(3), Nsereko G(4), Nalweyiso MD(3), Tindyebwa T(2)(4), Mayega RW(2), Ekiri AB(5), Bagenda D(6), Musenero M(1), Mugisha L(1)(3)(7).
Author information:
(1)Science, Technology and Innovation Secretariat- Office of the President, Kampala, Uganda.
(2)Makerere University School of Public Health, Kampala, Uganda.
(3)Makerere University College of Veterinary Medicine, Animal Resources and Biosecurity, Kampala, Uganda.
(4)Ministry of Health, Kampala, Uganda.
(5)School of Veterinary Medicine, University of Surrey, UK.
(6)University of Nebraska Medical Center, Omaha, NE, USA.
(7)Ecohealth Research Group, Conservation and Ecosystem Health Alliance (CEHA), Kampala, Uganda.

We conducted a retrospective cross-sectional population-based survey among recovered COVID-19 cases in Uganda to establish the case presentations of the second wave SARS-CoV-2 infections. We interviewed 1,120 recovered COVID-19 cases from 10 selected districts in Uganda. We further conducted 38 key informant interviews with members of the COVID-19 District Taskforce and 19 in-depth interviews among COVID-19 survivors from March to June 2021. Among them, 62% were aged 39 years and below and 51.5% were female with 90.9% under home-based care management. Cases were more prevalent among businesspeople (25.9%), students (16.2%), farmers (16.1%), and health workers (12.4%). Being asymptomatic was found to be associated with not seeking healthcare (APR 2, P < 0.001). The mortality rate was 3.6% mostly among the elderly (6.3%) and 31.3% aged 40 years and above had comorbidities of high blood pressure, diabetes, and asthma. Being asymptomatic, or under home-based care management (HBCM), working/operating/studying at schools, and not being vaccinated were among the major drivers of the second wave of the resurgence of COVID19 in Uganda. Managing future COVID-19 waves calls for proactive efforts for improving homebased care services, ensuring strict observation of SOPs in schools, and increasing the uptake of COVID-19 vaccination.

115. Vaccines (Basel). 2023 Jul 21;11(7):1268. doi: 10.3390/vaccines11071268.
Predictors of Tetanus Vaccine Uptake among Pregnant Women in Khartoum State, Sudan: A Hospital-Based Cross-Sectional Study.
Ibrahim ZA(1), Sabahelzain MM(2), Elhadi YAM(3), Malande OO(4)(5)(6), Babiker S(7).
Author information:
(1)Federal Ministry of Health, Khartoum P.O. Box 303, Sudan.
(2)Nutrition and Health Center for Training and Research, Ahfad University for Women, Omdurman P.O. Box 167, Sudan.
(3)Public Health Department, Sudanese Medical Research Association, Khartoum P.O. Box 303, Sudan.
(4)Department of Child Health and Paediatrics, Egerton University, Nakuru P.O. Box 3366-20100, Kenya.
(5)East Africa Centre for Vaccines and Immunization (ECAVI), Namela House, Kampala P.O. Box 3040, Uganda.
(6)Department of Paediatrics & Child Health, Makerere University, Kampala P.O. Box 3040, Uganda.
(7)School of Medicine, Ahfad University for Women, Omdurman P.O. Box 167, Sudan.
Tetanus toxoid (TT) vaccination during pregnancy has been proven as an effective preventative measure to reduce the incidence of maternal and neonatal morbidity and mortality worldwide. This study aimed to assess the determinants of TT vaccine uptake among pregnant women at two public maternity specialized hospitals in Sudan. A hospital-based cross-sectional study was conducted at two public hospitals, Omdurman Maternity Hospital and Al Saudi Hospital in Omdurman, Khartoum State, in Sudan from February to April 2020. Logistic regression analysis was carried out to identify factors associated with receiving three or more doses of the TT vaccine among pregnant women, presented as odds ratios, with p-values < 0.05 considered significant (at a 95% confidence interval). The

study recruited 350 pregnant women, with 313 participants included in the analysis. This study found that only 40% of the pregnant women had received three doses or more of the TT vaccine. Pregnant women who attended Al Saudi Hospital were less likely to be vaccinated with the recommended dose of the TT vaccine in districts at high risk (received ≥3 doses) compared to those who attended Omdurman Hospital [OR = 0.49 (95% C.I. 0.29-0.82), p-value < 0.05]. Furthermore, the number of children at home was a significant predictor of the mothers' immunization status as those with five children or more were ten times more likely to be vaccinated with three doses or more [OR = 10.54 (95% C.I. 4.30-25.86), p-value < 0.05]. We conclude that this low rate of TT vaccine uptake found in this study among pregnant women increases the number of newborn babies susceptible to contracting neonatal tetanus. The findings of this study should be considered in the development of communication strategies targeting and prioritizing at-risk groups to increase TT vaccine uptake among pregnant women in Sudan.

116. Trials. 2023 Jul 21;24(1):465. doi: 10.1186/s13063-023-07477-9.
Optimising DTwP-containing vaccine infant immunisation schedules (OptImms) - a protocol for two parallel, open-label, randomised controlled trials.
Kelly S(#)(1)(2), Liu X(#)(1)(2), Theiss-Nyland K(#)(1)(2), Voysey M(#)(1)(2), Murphy S(1)(2), Li G(3)(4), Nyantaro M(5), Gurung M(6), Basnet S(7), Pokhrel B(6), Bijukchhe SM(6), Eordogh A(1), Gombe B(5), Kakande A(5), Kerridge S(1), Kimbugwe G(5), Kusemererwa S(5), Lubyayi L(5), Luzze H(8), Mazur O(1)(2), Mujadidi YF(1), Nabukenya S(5), Nagumo WR(1)(2), Nareeba T(5), Noristani R(1)(2), O'Reilly P(1)(2), Roberts A(1)(2), Shah G(6), Shrestha S(1)(2), Shrestha LP(7), Thapa SB(7), Kibengo FM(5), Sharma AK(7), Elliott A(5), Shrestha S(6), Pollard AJ(1)(2).
Author information:
(1)Oxford Vaccine Group, University of Oxford, Oxford, UK.
(2)NIHR Oxford Biomedical Research Centre, Oxford, UK.
(3)Oxford Vaccine Group, University of Oxford, Oxford, UK. grace.li@paediatrics.ox.ac.uk.
(4)NIHR Oxford Biomedical Research Centre, Oxford, UK. grace.li@paediatrics.ox.ac.uk.
(5)MRC/UVRI and LSHTM Uganda Research Unit, Entebbe, Uganda.
(6)Patan Academy of Health Sciences, Lalitpur, Nepal.
(7)Department of Paediatrics, Institute of Medicine, Tribhuvan University, Kathmandu, Nepal.
(8)Ministry of Health, Kampala, Uganda.
(#)Contributed equally
BACKGROUND: Universal immunisation is the cornerstone of preventive medicine for children, The World Health Organisation (WHO) recommends diphtheria-tetanus-pertussis (DTP) vaccine administered at 6, 10 and 14 weeks of age as part of routine immunisation. However, globally, more than 17 unique DTP-containing vaccine schedules are in use. New vaccines for other diseases continue to be introduced into the infant immunisation schedule, resulting in an increasingly crowded schedule. The OptImms trial will assess whether antibody

titres against pertussis and other antigens in childhood can be maintained whilst adjusting the current Expanded Programme on Immunisation (EPI) schedule to provide space for the introduction of new vaccines.
METHODS: The OptImms studies are two randomised, five-arm, non-inferiority clinical trials in Nepal and Uganda. Infants aged 6 weeks will be randomised to one of five primary vaccination schedules based on age at first DTwP-vaccination (6 versus 8 weeks of age), number of doses in the DTwP priming series (two versus three), and spacing of priming series vaccinations (4 versus 8 weeks). Additionally, participants will be randomised to receive their DTwP booster at 9 or 12 months of age. A further sub-study will compare the co-administration of typhoid vaccine with other routine vaccines at one year of age. The primary outcome is anti-pertussis toxin IgG antibodies measured at the time of the booster dose. Secondary outcomes include antibodies against other vaccine antigens in the primary schedule and their safety.
DISCUSSION: These data will provide key data to inform policy decisions on streamlining vaccination schedules in childhood.
TRIAL REGISTRATIONS: ISRCTN12240140 (Nepa1, 7th January 2021) and ISRCTN6036654 (Uganda, 17th February 2021).

117. BMC Infect Dis. 2023 Jul 21;23(1):487. doi: 10.1186/s12879-023-08462-y.
Cholera and COVID-19 pandemic prevention in multiple hotspot districts of Uganda: vaccine coverage, adverse events following immunization and WASH conditions survey.
Bwire G(1)(2), Kisakye A(3), Amulen E(4), Bwanika JB(4), Badebye J(5), Aanyu C(4), Nakirya BD(6), Okello A(7), Okello SA(8), Bukenya JN(4), Orach CG(4).
Author information:
(1)School of Public Health, Makerere University, Kampala, Uganda. gbwire1@yahoo.com.
(2)Division of Public Health Emergency Preparedness and Response, Ministry of Health, Kampala, Uganda. gbwire1@yahoo.com.
(3)World Health Organization, Kampala, Uganda.
(4)School of Public Health, Makerere University, Kampala, Uganda.
(5)School of Forestry, Environmental and Geographical Sciences, Makerere University, Kampala, Uganda.
(6)Community Concerns Uganda Initiative, Jinja, Uganda.
(7)Department of Public Health, St Mary's Hospital Lacor, Gulu, Uganda.
(8)Department of Health Services, Kyambogo University, Kampala, Uganda.
BACKGROUND: Between March, 2020 and December, 2021 due to cholera and coronavirus disease 2019 (COVID-19) pandemics, there were 1,534 cholera cases with 14 deaths and 136,065 COVID-19 cases with 3,285 deaths reported respectively in Uganda. This study investigated mass vaccination campaigns for the prevention of the two pandemics namely: oral cholera vaccine (OCV) and COVID-19 vaccine coverage; adverse events following immunization (AEFI); barriers and enablers for the vaccine uptake and assessed water, sanitation and hygiene (WASH) conditions in the six cholera and COVID-19 hotspot districts of Uganda.
METHODS: A household survey was conducted between January and February, 2022 in

the six cholera hotspot districts of Uganda which had recently conducted OCV mass vaccination campaigns and had ongoing COVID-19 mass vaccination campaigns. The survey randomly enrolled 900 households with 4,315 persons of whom 2,085 were above 18 years. Data were collected using a data entry application designed in KoBoToolbox and analysed using STATA version 14. Frequencies, percentages, odds ratios, means, confidence intervals and maps were generated and interpreted.
RESULTS: The OCV coverage for dose one and two were 85% (95% CI: 84.2-86.4) and 67% (95% CI: 65.6-68.4) respectively. Among the 4,315 OCV recipients, 2% reported mild AEFI, 0.16% reported moderate AEFI and none reported severe AEFI. The COVID-19 vaccination coverage for dose one and two were 69.8% (95% CI: 67.8-71.8) and 18.8% (95% CI: 17.1-20.5) respectively. Approximately, 23% (478/2,085) of COVID-19 vaccine recipient reported AEFI; most 94% were mild, 0.6% were moderate and 2 cases were severe. The commonest reason for missing COVID-19 vaccine was fear of the side effects. For most districts (5/6), sanitation (latrine/toilet) coverage were low at 7.4%-37.4%.
CONCLUSION: There is high OCV coverage but low COVID-19 vaccine and sanitation coverage with high number of moderate cases of AEFI recorded due to COVID-19 vaccines. The low COVID-19 vaccine coverage could indicate vaccine hesitancy for COVID-19 vaccines. Furthermore, incorporation of WASH conditions assessment in the OCV coverage surveys is recommended for similar settings to generate data for better planning. However, more studies are required on COVID-19 vaccine hesitancy.

118. PLOS Glob Public Health. 2023 Jul 21;3(7):e0001523. doi: 10.1371/journal.pgph.0001523. eCollection 2023.

The immediate treatment outcomes and cost estimate for managing clinical measles in children admitted at Mulago Hospital: A retrospective cohort study.

Namugga B(1), Malande O(1), Kitonsa J(2), Manirakiza L(3), Banura C(4), Mupere E(1).
Author information:
(1)Kampala Uganda Department of Paediatrics, Makerere University College of Health Sciences, Kampala, Uganda.
(2)Medical Research Council/Uganda Virus Research Institute and London School of Hygiene and Tropical Medicine, London, England.
(3)Uganda National Bureau of Standards, Kampala, Uganda.
(4)Kampala Uganda Department of Medical Epidemiology and Biostatistics, Makerere University College of Health Sciences, Kampala, Uganda.

Over the recent years, the Ministry of Health in Uganda has reported multiple measles outbreaks in various districts despite the availability of a safe cost effective vaccine. Measles, especially among the unvaccinated can lead to serious complications including death while its management heavily burdens the family and health care system. This study aims to determine the immediate treatment outcomes and estimate the cost of treating a measles case. A retrospective cohort study using records review was conducted among children 0-12 years admitted at Mulago hospital throughout 2018. Demographics, complications, vaccination status, discharge status, duration of hospital stay,

type of treatment, supplies and investigations done were abstracted from the patient charts. Treatment costs were obtained from the hospital pharmacy price list while the unit cost of utilities, human resource, food and security were obtained from the hospital accounts department. Patients' characteristics were summarized descriptively. Cost information, was reported as mean with standard deviation (SD) and range, and was stratified and presented as direct health care (blood test, radiology and treatment) and direct non health care costs. Among 267 reviewed patient charts, the median age was 1.0 ((IQR 0.75-2) years. 63patients (24%) were immunised, 79 (29%) were not immunized, Median length of hospital stay was 4.0 days (IQR 3.0-7.0) with majority (n = 207, 77%) staying < 7 days. 30 patients (11%) died with mortality highest among the unimmunised (n = 13, 44%) and severe pneumonia (39.5%) was the commonest complication. 114.5 USD was estimated to treat a child with measles. Human resource (79.33USD, SD 4.63) and treatment costs (21.98USD, SD 22.77) were the largest expenses. Complications are common in majority of fatal measles cases and these carry a high cost to the healthcare system.

119. Immunother Adv. 2023 Jul 20;3(1):ltad010. doi: 10.1093/immadv/ltad010.
Establishing a single-sex controlled human Schistosoma mansoni infection model for Uganda: protocol for safety and dose-finding trial.
Abaasa A(1)(2), Egesa M(1)(2), Driciru E(1), Koopman JPR(3), Kiyemba R(1), Sanya RE(1)(4), Nassuuna J(1), Ssali A(1)(2), Kimbugwe G(1), Wajja A(1), van Dam GJ(3), Corstjens PLAM(3), Cose S(1)(2), Seeley J(1)(2), Kamuya D(5), Webb EL(2), Yazdanbakhsh M(3), Kaleebu P(1)(2), Siddiqui AA(6), Kabatereine N(7), Tukahebwa E(7), Roestenberg M(3), Elliott AM(1)(2).
Author information:
(1)MRC/UVRI & LSHTM Uganda Research Unit, Entebbe, Uganda.
(2)London School of Hygiene & Tropical Medicine, London, UK.
(3)Leiden University Medical Center, Leiden, The Netherlands.
(4)African Population and Health Research Center, Nairobi, Kenya.
(5)Kenya Medical Research Institute (KEMRI), Kilifi, Kenya.
(6)Texas Tech University Health Sciences Center, Lubbock, TX, USA.
(7)Ministry of Health of Uganda, Kampala, Uganda.
Control of schistosomiasis depends on a single drug, praziquantel, with variable cure rates, high reinfection rates, and risk of drug resistance. A vaccine could transform schistosomiasis control. Preclinical data show that vaccine development is possible, but conventional vaccine efficacy trials require high incidence, long-term follow-up, and large sample size. Controlled human infection studies (CHI) can provide early efficacy data, allowing the selection of optimal candidates for further trials. A Schistosoma CHI has been established in the Netherlands but responses to infection and vaccines differ in target populations in endemic countries. We aim to develop a CHI for Schistosoma mansoni in Uganda to test candidate vaccines in an endemic setting. This is an open-label, dose-escalation trial in two populations: minimal, or intense, prior Schistosoma exposure. In each population, participants will be enrolled in sequential dose-escalating groups. Initially, three volunteers will be exposed to 10 cercariae. If all show infection, seven more will be exposed to the same

dose. If not, three volunteers in subsequent groups will be exposed to higher doses (20 or 30 cercariae) following the same algorithm, until all 10 volunteers receiving a particular dose become infected, at which point the study will be stopped for that population. Volunteers will be followed weekly after infection until CAA positivity or to 12 weeks. Once positive, they will be treated with praziquantel and followed for one year. The trial registry number is ISRCTN14033813 and all approvals have been obtained. The trial will be subjected to monitoring, inspection, and/or audits.

120. BMC Public Health. 2023 Jul 20;23(1):1401. doi: 10.1186/s12889-023-16320-4.
Understanding the barriers and facilitators of COVID-19 risk mitigation strategy adoption and COVID-19 vaccination in refugee settlements in Uganda: a qualitative study.
Klabbers RE(1)(2), Muwonge TR(3), Ajidiru S(3), Borthakur S(4), Mujugira A(5)(3), Sharma M(5), Vinck P(6), Pham P(6), Celum C(5)(7)(8), Parkes-Ratanshi R(3)(9), O'Laughlin KN(10)(5).
Author information:
(1)Department of Emergency Medicine, University of Washington, Seattle, WA, USA. reklabb@uw.edu.
(2)Department of Global Health, University of Washington, Seattle, WA, USA. reklabb@uw.edu.
(3)Infectious Diseases Institute, Makerere University, Kampala, Uganda.
(4)Medical Teams International, Kampala, Uganda.
(5)Department of Global Health, University of Washington, Seattle, WA, USA.
(6)Harvard Humanitarian Initiative, Harvard University, Cambridge, MA, USA.
(7)Department of Epidemiology, University of Washington, Seattle, WA, USA.
(8)Department of Medicine, University of Washington, Seattle, WA, USA.
(9)Department of Psychiatry, University of Cambridge, Cambridge, UK.
(10)Department of Emergency Medicine, University of Washington, Seattle, WA, USA.
BACKGROUND: Perspectives on COVID-19 risk and the willingness and ability of persons living in refugee settlements to adopt COVID-19 prevention strategies have not been rigorously evaluated. The realities of living conditions in Ugandan refugee settlements may limit the extent to which refugees can uptake strategies to mitigate COVID-19 risk.
METHODS: In-depth qualitative interviews were conducted between April 2021 and April 2022 to assess COVID-19 knowledge, risk perception, prevention strategy adoption including COVID-19 vaccination, and COVID-19 impact on living conditions in refugee settlements in Uganda. Interview participants included 28 purposively selected refugees who called into "Dial-COVID", a free telephone COVID-19 information collection and dissemination platform that was advertised in refugee settlements by community health workers. Interviews were analyzed using a combination of deductive and inductive content analysis. Emerging themes were mapped onto the Theoretical Domains Framework to identify domains influencing prevention behavior. Results were synthesized to provide intervention and policy recommendations for risk mitigation in refugee settlements for COVID-19 and future infectious disease outbreaks.

RESULTS: The COVID-19 pandemic detrimentally impacted economic and food security as well as social interactions in refugee settlements. Youth were considered especially impacted, and participants reported incidents of child marriage and teenage pregnancy following school closures. Participants displayed general knowledge of COVID-19 and expressed willingness to protect themselves and others from contracting COVID-19. Risk mitigation strategy uptake including COVID-19 vaccination was influenced by COVID-19 knowledge, emotions surrounding COVID-19, the environmental context and resources, personal goals, beliefs about the consequences of (non)adoption, social influences, and behavior reinforcement. Resource constraints, housing conditions, and competing survival needs challenged the adoption of prevention strategies and compliance decreased over time.
CONCLUSIONS: Contextual challenges impact the feasibility of COVID-19 risk mitigation strategy uptake in refugee settlements. Pre-existing hardships in this setting were amplified by the COVID-19 pandemic and related lockdowns. Targeted dispelling of myths, alignment of information across communication mediums, supporting survival needs and leveraging of respected role models are strategies that may hold potential to mitigate risk of infectious diseases in this setting.
REGISTRATION DETAILS: World Pandemic Research Network - 490,652.

121. BMC Pediatr. 2023 Jul 17;23(1):368. doi: 10.1186/s12887-023-04174-z.
Knowledge, perceptions and uptake of human papilloma virus vaccine among adolescent girls in Kampala, Uganda; a mixed-methods school-based study.
Bitariho GK(1), Tuhebwe D(1), Tigaiza A(2)(3), Nalugya A(1)(4), Ssekamatte T(1), Kiwanuka SN(5).
Author information:
(1)Department of Disease control and Environmental Health, School of Public Health, College of Health Sciences, Makerere University, P.O BOX 7072, Kampala, Uganda.
(2)Department of Disease control and Environmental Health, School of Public Health, College of Health Sciences, Makerere University, P.O BOX 7072, Kampala, Uganda. arnoldtigaiza32@gmail.com.
(3)Elevate Research and Health Services Limited, P.O BOX 3712, Kampala, Uganda. arnoldtigaiza32@gmail.com.
(4)Elevate Research and Health Services Limited, P.O BOX 3712, Kampala, Uganda.
(5)Department of Health Policy Planning and Management, School of Public Health, College of Health Sciences, Makerere University, P.O BOX 7072, Kampala, Uganda.
BACKGROUND: Cervical cancer is a major public health challenge, accounting for substantial morbidity and mortality. Human Papilloma Virus (HPV) vaccination is the recommended primary public health intervention for HPV infection prevention. However, there's limited evidence on the level of knowledge, attitude, and practices of adolescent girls regarding HPV vaccination in Kampala city, Uganda. This study assessed the knowledge, perceptions, and practices of adolescent girls aged 10-14 years towards HPV vaccination program in Kampala, Uganda to generate evidence to guide programs targeted at improving uptake of the vaccine.
METHODS: A convergent parallel mixed methods study was conducted in Kampala,

Uganda. A structured questionnaire was used to elicit data from 524 adolescent girls. In addition, 6 Focus group discussions, and 24 key informant interviews (teacher and parents) were conducted. Multistage and purposive sampling techniques were used to select quantitative and qualitative participants respectively. Quantitative data were entered using epidata, cleaned and analyzed using Stata v14 while qualitative data were analyzed using thematic content analysis in atlas ti version 8.
RESULTS: Overall, only 8.6% (45/524) of the girls had completed the HPV vaccine schedule of two dozes, 49.2% (258/524) of the girls had low knowledge about the HPV vaccine and teachers and parents affirmed this lack of knowledge among adolescent girls especially concerning the target age group, dosage, and vaccine interval. About 51.9% (272/524) of girls had negative perceptions towards HPV vaccination. Parents expressed negative perceptions, beliefs, superstitions, and safety concerns of the vaccine.Girls residing in rural areas (adjusted prevalence ratio, aPR = 0.35, C. I = 0.14-0.85) had lower knowledge levels compared to those in urban areas. Girls whose mothers were healthcare providers (aPR = 1.94, C. I = 1.10-3.41), girls with high knowledge levels (aPR = 1.79, C. I = 1.21-2.63) and positive perceptions (aPR = 2.87, C. I = 1.93-4.27) had a higher prevalence of being fully vaccinated.
CONCLUSION: Girls generally had low levels of knowledge, negative perceptions, and poor uptake of HPV vaccination. We recommend sensitization campaigns in schools and communities to improve awareness, perceptions, and practices of stakeholders towards HPV vaccination.

122. PLoS One. 2023 Jul 17;18(7):e0288604. doi: 10.1371/journal.pone.0288604.
Hepatitis B status and associated factors among participants screened for simulated HIV vaccine efficacy trials in Kenya and Uganda.
Mayanja Y(1), Rida W(2), Kimani J(3), Ssetala A(4), Mpendo J(4), Nanvubya A(4), Mutua G(5), Anzala O(6), Price MA(7)(8).
Author information:
(1)Medical Research Council/ Uganda Virus Research Institute and London School of Hygiene and Tropical Medicine (MRC/UVRI & LSHTM) Uganda Research Unit, Entebbe, Uganda.
(2)Biostatistics Consultant, Arlington, Virginia, United States of America.
(3)SWOP-PHDA, University of Nairobi/University of Manitoba, Nairobi, Kenya.
(4)Uganda Virus Research Institute/ International AIDS Vaccine Initiative (UVRI-IAVI) HIV Vaccine Programme, Entebbe, Uganda.
(5)IAVI, Nairobi, Kenya.
(6)KAVI- Institute for Clinical Research, Nairobi, Kenya.
(7)Department of Epidemiology and Biostatistics, University of California San Francisco, San Francisco, CA, United States of America.
(8)IAVI, New York, New York, United States of America.
INTRODUCTION: Hepatitis B (HBV) prevalence remains high in Sub Saharan Africa and among some key populations such as those with continued exposure through sexual contact. We assessed the HBV status among potential participants who were screened for simulated HIV vaccine efficacy trials in Kenya and Uganda.
METHODS: We conducted a cross sectional analysis of data collected from

individuals who were screened in Kenya (Nairobi) and Uganda (Entebbe and Kampala). The studies followed hypothetical procedures of an HIV vaccine efficacy trial and aimed to enroll HIV negative key and vulnerable populations at elevated risk of HIV acquisition. HBV status was the main outcome categorized using Hepatitis B surface antigen (HBsAg) and total Hepatitis B core antibody (HBcAb). Baseline characteristics potentially associated with never being infected were analyzed using logistic regression.
RESULTS: We screened 1,366 participants with mean age (SD) 28.7 (7.3) years. Overall, 46.6% were from Entebbe, 50.7% had secondary or higher level of education, 76.4% had informal high-risk jobs and 56.3% were male. Kampala had only female participants contributing 60.6% of females screened. Of the screened participants, 94.7% and 3.4% were negative and positive for HBsAg respectively. The prevalence on HBV infection was 3.9% among males and 2.8% among females while prevalence by site was: Entebbe (4.9%); Kampala (4.1%) and Nairobi (0.3%). The highest HBV prevalence was found among participants aged 25-29-years (5.2%), those with primary level education (4.5%), and those in informal low risk jobs (6.5%). Considering 1265 participants with complete data on HBsAg and HBcAb-Total, HBV status was never infected (67.9%), past infection (28.5%), chronic infection (3.2%) and acute infection (0.5%). Of 859 who were never infected, 685 (79.7%) were tested for anti-HBs titers of whom 60 (8.8%) had titers >10IU/L (immune due to vaccination). The odds of never being HBV infected were lower among older individuals 25-29 years (AOR 0.51; 95%CI 0.36-0.71) and ≥30 years (AOR 0.35; 95% CI 0.25-0.49). The odds were higher among participants with informal high-risk jobs from Kampala (AOR 2.21; 95% CI 1.41-3.47) and Nairobi (AOR 2.61; 95% CI 1.72-4.00) compared to those from Entebbe.
CONCLUSION: HBV prevalence and immunity due to vaccination were low among HIV negative individuals who are eligible for HIV vaccine trials and prevalence varies by age, education level and main occupation. Younger individuals and those recruited from existing cohorts/ clinics have a higher likelihood of having no prior HBV infection. HIV prevention intervention trials are a platform to identify individuals that need HBV vaccination.

123. NPJ Vaccines. 2023 Jul 11;8(1):99. doi: 10.1038/s41541-023-00699-7.
YF17D-vectored Ebola vaccine candidate protects mice against lethal surrogate Ebola and yellow fever virus challenge.
Lemmens V(1), Kelchtermans L(1), Debaveye S(1), Chiu W(1), Vercruysse T(2)(3), Ma J(1), Thibaut HJ(2), Neyts J(1)(4), Sanchez-Felipe L(5), Dallmeier K(6).
Author information:
(1)KU Leuven Department of Microbiology, Immunology and Transplantation, Rega Institute, Virology and Chemotherapy, Molecular Vaccinology & Vaccine Discovery, BE-3000, Leuven, Belgium.
(2)KU Leuven Department of Microbiology, Immunology and Transplantation, Rega Institute, Translational Platform Virology and Chemotherapy (TPVC), BE-3000, Leuven, Belgium.
(3)AstriVax, BE-3001, Heverlee, Belgium.
(4)GVN, Global Virus Network, Baltimore, MD, USA.
(5)KU Leuven Department of Microbiology, Immunology and Transplantation, Rega

Institute, Virology and Chemotherapy, Molecular Vaccinology & Vaccine Discovery, BE-3000, Leuven, Belgium. lorena.sanchezfelipe@kuleuven.be.
(6)KU Leuven Department of Microbiology, Immunology and Transplantation, Rega Institute, Virology and Chemotherapy, Molecular Vaccinology & Vaccine Discovery, BE-3000, Leuven, Belgium. kai.dallmeier@kuleuven.be.
Ebola virus (EBOV) and related filoviruses such as Sudan virus (SUDV) threaten global public health. Effective filovirus vaccines are available only for EBOV, yet restricted to emergency use considering a high reactogenicity and demanding logistics. Here we present YF-EBO, a live YF17D-vectored dual-target vaccine candidate expressing EBOV glycoprotein (GP) as protective antigen. Safety of YF-EBO in mice was further improved over that of parental YF17D vaccine. A single dose of YF-EBO was sufficient to induce high levels of EBOV GP-specific antibodies and cellular immune responses, that protected against lethal infection using EBOV GP-pseudotyped recombinant vesicular stomatitis virus (rVSV-EBOV) in interferon-deficient (Ifnar-/-) mice as surrogate challenge model. Concomitantly induced yellow fever virus (YFV)-specific immunity protected Ifnar-/- mice against intracranial YFV challenge. YF-EBO could thus help to simultaneously combat both EBOV and YFV epidemics. Finally, we demonstrate how to target other highly pathogenic filoviruses such as SUDV at the root of the 2022 outbreak in Uganda.

124. Global Health. 2023 Jul 10;19(1):48. doi: 10.1186/s12992-023-00945-6.
The impact of donor transition on continuity of maternal and newborn health service delivery in Rwenzori sub-region of Uganda: a qualitative country case study analysis.
Ssegujja E(1), Namakula J(2), Kabagenyi A(3), Kyozira C(4), Musila T(4), Zakumumpa H(2), Ssengooba F(2).
Author information:
(1)Department of Health Policy Planning and Management, School of Public Health, College of Health Sciences, Makerere University- Kampala, Kampala, Uganda. ssega2001@yahoo.com.
(2)Department of Health Policy Planning and Management, School of Public Health, College of Health Sciences, Makerere University- Kampala, Kampala, Uganda.
(3)Department of Statistics and Population Studies, College of Business and Management Studies, Makerere University- Kampala, Kampala, Uganda.
(4)Ministry of Health- Kampala, Kampala, Uganda.
BACKGROUND: The transition of donor-supported health programmes to country ownership is gaining increasing attention due to reduced development assistance for health globally. It is further accelerated by the ineligibility of previously Low-Income Countries' elevation into Middle-income status. Despite the increased attention, little is known about the long-term impact of this transition on the continuity of maternal and child health service provision. Hence, we conducted this study to explore the impact of donor transition on the continuity of maternal and newborn health service provision at the sub-national level in Uganda between 2012 and 2021.
METHODS: We conducted a qualitative case study of the Rwenzori sub-region in mid-western Uganda which benefited from a USAID project to reduce maternal and

newborn deaths between 2012 and 2016. We purposively sampled three districts. Data were collected between January and May 2022 among subnational key informants (n = 26), national level key informants at the Ministry of Health [3], national level donor representatives [3] and subnational level donor representatives [4] giving a total of 36 respondents. Thematic analysis was deductively conducted with findings structured along the WHO's health systems building blocks (Governance, Human resources for health, Health financing, Health information systems, medical products, Vaccines and Technologies and service delivery) framework.
RESULTS: Overall, continuity of maternal and newborn health service provision was to a greater extent maintained post-donor support. The process was characterised by a phased implementation approach. The embedded learning offered the opportunity to plough back lessons into intervention modification which reflected contextual adaptation. The availability of successor grants from other donors (such as Belgian ENABEL), counterpart funding from the government to bridge the gaps left behind, absorption of USAID-project salaried workforce (such as midwives) onto the public sector payroll, harmonisation of salary structures, the continued use of infrastructure (such as newborn intensive care units), and support for MCH services under PEPFAR support post-transition contributed to the maintenance of coverage. The demand creation for MCH services pre-transition ensured patient demand post-transition. Challenges to the maintenance of coverage were drug stockouts and sustainability of the private sector component among others.
CONCLUSION: A general perception of the continuity of maternal and newborn health service provision post-donor transition was observed with internal (government counterpart funding) and external enablers (successor donor funding) contributing to this performance. Opportunities for the continuity of maternal and newborn service delivery performance post-transition exist when harnessed well within the prevailing context. The ability to learn and adapt, the presence of government counterpart funding and commitment to carry on with implementation were major ingredients signalling a crucial role of government in the continuity of service provision post-transition.

125. BMC Womens Health. 2023 Jul 8;23(1):362. doi: 10.1186/s12905-023-02511-z.
Factors associated with uptake of human papilloma virus vaccine among school girls aged 9-14 years in Lira City northern Uganda: a cross-sectional study.
Nakayita RM(1), Benyumiza D(2), Nekesa C(1), Misuk I(1), Kyeswa J(1), Nalubuuka A(1), Murungi T(1), Udho S(1), Kumakech E(3).
Author information:
(1)Department of Midwifery, Faculty of Nursing and Midwifery, Lira University, P.O. BOX 1035, Lira City, Uganda.
(2)Department of Midwifery, Faculty of Nursing and Midwifery, Lira University, P.O. BOX 1035, Lira City, Uganda. benyumizadeo@gmail.com.
(3)Department of Nursing, Faculty of Nursing and Midwifery, Lira University, P.O. Box 1035, Lira City, Uganda.
BACKGROUND: Cervical cancer is the most common Human Papilloma Virus (HPV)-related disease among women. Since 2008, HPV vaccination has been

routinely recommended for pre-adolescent and adolescent girls in Uganda as the primary preventive measure for cervical cancer. However, in Uganda, most especially in Lira district, there is limited literature on HPV vaccination uptake and associated factors among girls aged 9-14years. This study assessed the uptake of HPV vaccine and associated factors among in-school girls aged 9-14 years in Lira City, northern Uganda.
METHODS: A cross-sectional study was conducted among 245 primary school girls aged 9-14 years in Lira City, northern Uganda. Multistage sampling technique was used to sample eligible participants and data was collected using interviewer administered questionnaire. Data was analysed using SPSS version 23.0. Descriptive statistics and multivariate logistic regression at 95% level of significance were used to identify the level of HPV vaccine uptake and predictors respectively.
RESULTS: HPV vaccination uptake was at 19.6% (95% CI,14.8-25.1) among the school girls aged 9-14 years in Lira City, northern Uganda. The mean age of the girls was 12.11 (± 1.651) years. Predictors that were independently associated with HPV vaccine uptake included; recommendation from health worker [aOR 9.09, 95% CI (3.19-25.88), P ≤ 0.001], taught about cervical cancer at school [aOR,12.56, 95% CI (4.60-34.28), P ≤ 0.001], and exposure to outreach clinics [aOR, 4.41, 95% CI (1.37-14.19), P = 0.013].
CONCLUSION: The study found that one in five of the school girls in Lira City, northern Uganda. received HPV vaccine. Girls who were taught about cervical cancer at school, exposure to outreach clinics and received health worker recommendation had more odds of receiving HPV vaccine than their counter parts. The Ministry of Health should strengthen school based cervical cancer education, awareness raising about HPV vaccination and health worker recommendations to improve HPV vaccine uptake among school girls in Uganda.

126. Pathogens. 2023 Jul 6;12(7):912. doi: 10.3390/pathogens12070912.
Whole Genome Sequencing Shows that African Swine Fever Virus Genotype IX Is Still Circulating in Domestic Pigs in All Regions of Uganda.
Okwasiimire R(1), Flint JF(2), Kayaga EB(1), Lakin S(3), Pierce J(3), Barrette RW(3), Faburay B(3), Ndoboli D(1), Ekakoro JE(2), Wampande EM(1), Havas KA(2).
Author information:
(1)Central Diagnostic Laboratory, College of Veterinary Medicine, Animal Resources and Biosecurity, Makerere University, Kampala P.O. Box 7062, Uganda.
(2)Department of Public and Ecosystem Health, College of Veterinary Medicine, Cornell University, Ithaca, NY 14853, USA.
(3)Foreign Animal Disease Diagnostic Laboratory, National Veterinary Services Laboratories, Veterinary Services, Animal and Plant Health Inspection Services, United States Department of Agriculture, Greenport, NY 11957, USA.
Blood samples were collected from pigs at six abattoirs in the Kampala, Uganda metropolitan area from May 2021 through June 2022, and tested for African swine fever virus. Thirty-one samples with cycle threshold values < 26 from pigs with different geographic origins, clinical and pathologic signs, and Ornithodoros moubata exposure underwent whole genome sequencing. The p72 gene was used to genotype the isolates, and all were found to be genotype IX; whole genome

sequences to previous genotype IX isolates confirmed their similarity. Six of the isolates had enough coverage to evaluate single nucleotide polymorphisms (SNPs). Five of the isolates differed from historic regional isolates, but had similar SNPs to one another, and the sixth isolate also differed from historic regional isolates, but also differed from the other five isolates, even though they are all genotype IX. Whole genome sequencing data provide additional detail on viral evolution that can be useful for molecular epidemiology, and understanding the impact of changes in genes to disease phenotypes, and may be needed for vaccine targeting should a commercial vaccine become available. More sequencing of African swine fever virus isolates is needed in Uganda to understand how and when the virus is changing.

127. BMC Proc. 2023 Jul 6;17(Suppl 7):8. doi: 10.1186/s12919-023-00266-x.

The evolution of COVID-19 vaccine hesitancy in Sub-Saharan Africa: evidence from panel survey data.

Wollburg P(1), Markhof Y(2)(3), Kanyanda S(4), Zezza A(2).
Author information:
(1)World Bank, Development Economics Data Group, Washington, DC, USA. pwollburg@worldbank.org.
(2)World Bank, Development Economics Data Group, Washington, DC, USA.
(3)UNU-MERIT, United Nations University, Maastricht, Netherlands.
(4)National Statistical Office, Zomba, Malawi.

BACKGROUND: COVID-19 vaccination efforts are lagging in Sub-Saharan Africa, as just over 20 percent of the population has been fully vaccinated. COVID-19 vaccine hesitancy is considered important as a prerequisite for widespread vaccine take-up. Here, we study the dynamics of vaccine acceptance, its correlates, and reasons for hesitancy over time, drawing on two years of panel survey data.

METHODS: In this observational study, we use multiple rounds of data from national High Frequency Phone Surveys (HFPS) in five countries in East and West Africa (Burkina Faso, Ethiopia, Malawi, Nigeria, and Uganda), covering a period between 2020 and 2022. The surveys are cross-country comparable and draw their samples from nationally representative sampling frames. Based on this data source, the study presents population-weighted means and performs multivariate regression analysis.

RESULTS: COVID-19 vaccine acceptance was high throughout the study period (68% to 98%). However, acceptance levels were lower in 2022 than in 2020 in three countries (Burkina Faso, Malawi, Nigeria), and higher in one country (Uganda). Moreover, individuals are observed to change their stated vaccine attitudes between survey rounds, to a limited extent in some countries (Ethiopia) and more frequently in others (Burkina Faso, Malawi, Nigeria, Uganda). Vaccine hesitancy is higher in richer households, and those residing in urban areas; among women and those better educated. Hesitancy is lower in larger households and among heads of the household. The main reasons for hesitancy are concerns about side effects of the vaccine, its safety and efficacy, as well as assessments of COVID-19 risk, though these reasons fluctuate over time.

CONCLUSIONS: Reported COVID-19 vaccine acceptance levels remain far above vaccination rates in the study countries, suggesting that vaccine hesitancy is

not the primary obstacle to reaching greater vaccine coverage, which may instead be related to access and delivery barriers as well as supply shortages. Nevertheless, vaccine attitudes appear malleable so that continued efforts are needed to retain high levels of vaccine acceptance.

128. PLoS Negl Trop Dis. 2023 Jul 5;17(7):e0011089. doi: 10.1371/journal.pntd.0011089. eCollection 2023 Jul.

Schistosoma mansoni infection alters the host pre-vaccination environment resulting in blunted Hepatitis B vaccination immune responses.

Muir R(1), Metcalf T(1), Fourati S(2), Bartsch Y(3), Kyosiimire-Lugemwa J(4), Canderan G(5), Alter G(3), Muyanja E(2)(6), Okech B(6), Namatovu T(6), Namara I(6), Namuniina A(6), Ssetaala A(6), Mpendo J(6), Nanvubya A(6), Kitandwe PK(4), Bagaya BS(7), Kiwanuka N(8), Nassuna J(8), Biribawa VM(6), Elliott AM(4)(9), de Dood CJ(10), Senyonga W(4), Balungi P(4), Kaleebu P(4), Mayanja Y(4), Odongo M(4), Connors J(1), Fast P(11)(12), Price MA(11)(13), Corstjens PLAM(10), van Dam GJ(14), Kamali A(6)(11)(15), Sekaly RP(2), Haddad EK(1).

Author information:
(1)Division of Infectious Diseases and HIV Medicine, Department of Medicine, Drexel University College of Medicine, Philadelphia, Pennsylvania, United States of America.
(2)PATRU, School of Medicine, Emory University, Atlanta, Georgia, United States of America.
(3)Ragon Institute of MGH, MIT, and Harvard, Cambridge, Massachusetts, United States of America.
(4)MRC/UVRI and LSHTM Uganda Research Unit, Entebbe, Uganda.
(5)Department of Medicine, Allergy and Immunology, University of Virginia, Charlottesville, Virginia, United States of America.
(6)UVRI-IAVI HIV Vaccine Program, Entebbe, Uganda.
(7)Department of Immunology and Molecular Biology, School of Biomedical Sciences, Makerere University, College of Health Sciences, Kampala, Uganda.
(8)Department of Epidemiology and Biostatistics, School of Public Health, Makerere University, College of Health Sciences, Kampala, Uganda.
(9)Department of Clinical Research, London School of Hygiene and Tropical Medicine, London, United Kingdom.
(10)Department of Cell and Chemical Biology, Leiden University Medical Center, Leiden, Netherlands.
(11)International AIDS Vaccine Initiative, New York, New York, United States of America.
(12)Pediatric Infectious Diseases, Stanford University School of Medicine, Palo Alto, California, United States of America.
(13)Department of Epidemiology and Biostatistics, University of California at San Francisco, San Francisco, California, United States of America.
(14)Department of Parasitology, Leiden University Medical Center, Leiden, the Netherlands.
(15)IAVI, New York, New York, United States of America, and Nairobi, Kenya.

Update of
medRxiv. 2023 Feb 26;:

Schistosomiasis is a disease caused by parasitic flatworms of the Schistosoma spp., and is increasingly recognized to alter the immune system, and the potential to respond to vaccines. The impact of endemic infections on protective immunity is critical to inform vaccination strategies globally. We assessed the influence of Schistosoma mansoni worm burden on multiple host vaccine-related immune parameters in a Ugandan fishing cohort (n = 75) given three doses of a Hepatitis B (HepB) vaccine at baseline and multiple timepoints post-vaccination. We observed distinct differences in immune responses in instances of higher worm burden, compared to low worm burden or non-infected. Concentrations of pre-vaccination serum schistosome-specific circulating anodic antigen (CAA), linked to worm burden, showed a significant bimodal distribution associated with HepB titers, which was lower in individuals with higher CAA values at month 7 post-vaccination (M7). Comparative chemokine/cytokine responses revealed significant upregulation of CCL19, CXCL9 and CCL17 known to be involved in T cell activation and recruitment, in higher CAA individuals, and CCL17 correlated negatively with HepB titers at month 12 post-vaccination. We show that HepB-specific CD4+ T cell memory responses correlated positively with HepB titers at M7. We further established that those participants with high CAA had significantly lower frequencies of circulating T follicular helper (cTfh) subpopulations pre- and post-vaccination, but higher regulatory T cells (Tregs) post-vaccination, suggesting changes in the immune microenvironment in high CAA could favor Treg recruitment and activation. Additionally, we found that changes in the levels of innate-related cytokines/chemokines CXCL10, IL-1β, and CCL26, involved in driving T helper responses, were associated with increasing CAA concentration. This study provides further insight on pre-vaccination host responses to Schistosoma worm burden which will support our understanding of vaccine responses altered by pathogenic host immune mechanisms and memory function and explain abrogated vaccine responses in communities with endemic infections.

129. Clin Infect Dis. 2023 Jul 5;77(1):1-8. doi: 10.1093/cid/ciad117.
Risk Factors, Temporal Dependence, and Seasonality of Human Extended-Spectrum β-Lactamases-Producing Escherichia coli and Klebsiella pneumoniae Colonization in Malawi: A Longitudinal Model-Based Approach.
Sammarro M(1)(2), Rowlingson B(2), Cocker D(1)(3), Chidziwisano K(4)(5), Jacob ST(1)(6), Kajumbula H(7), Mugisha L(8)(9), Musoke D(10), Lester R(1)(3), Morse T(4)(5), Feasey N(1)(3), Jewell C(2).
Author information:
(1)Department of Clinical Sciences, Liverpool School of Tropical Medicine, Liverpool, United Kingdom.
(2)Centre for Health Informatics, Computing, and Statistics, Lancaster University, Lancaster, United Kingdom.
(3)Malawi-Liverpool-Wellcome Research Programme, Kamuzu University of Health Sciences, Blantyre, Malawi.
(4)Centre for Water, Sanitation, Health and Appropriate Technology Development (WASHTED), Malawi University of Business and Applied Sciences (MUBAS), Blantyre, Malawi.

(5)Department of Civil and Environmental Engineering, University of Strathclyde, Glasgow, United Kingdom.
(6)Global Health Security Department, Infectious Disease Institute, Makerere University, Kampala, Uganda.
(7)Department of Medical Microbiology, College of Health Sciences, Makerere University, Kampala, Uganda.
(8)College of Veterinary Medicine, Animal Resources and Biosecurity (COVAB), Makerere University, Kampala, Uganda.
(9)Conservation & Ecosystem Health Alliance, Kampala, Uganda.
(10)Department of Disease Control and Environmental Health, College of Health Sciences, Makerere University, Kampala, Uganda.

BACKGROUND: Sub-Saharan Africa has the highest estimated death rate attributable to antimicrobial resistance, especially from extended-spectrum β-lactamase-producing Enterobacterales (ESBL-E). However, the dynamics of human colonization in the community with ESBL-E are not well described. Inadequate water, sanitation, and hygiene infrastructure and associated behaviors are believed to play an important role in transmission of ESBL-E, and an improved understanding of the temporal dynamics of within-household transmission could help inform the design of future policies.
METHODS: In this 18-month study, using microbiological data and household surveys, we built a multivariable hierarchical harmonic logistic regression model to identify risk factors for colonization with ESBL-producing Escherichia coli and Klebsiella pneumoniae, reflecting household structure and temporal correlation of colonization status.
RESULTS: Being male was associated with a lower risk of colonization with ESBL-producing E. coli (odds ratio [OR], 0.786; credible interval [CrI], .678-.910), whereas the use of a tube well or a borehole was associated with an increased risk (OR, 1.550; CrI, 1.003-2.394). For ESBL-producing K. pneumoniae, recent antibiotic exposure increased risk of colonization (OR, 1.281; CrI, 1.049-1.565), whereas sharing plates decreased that risk (OR, 0.672; CrI, .460-.980). Finally, the temporal correlation range of 8 to 11 weeks provided evidence that within-household transmission occurs within this time frame.
CONCLUSIONS: We describe different risks for colonization with different enteric bacterial species. Our findings suggest interventions to reduce transmission targeted at the household level need to focus on improving water, sanitation, and hygiene infrastructure and associated behaviors, whereas at the community level, they should focus on both environmental hygiene and antibiotic stewardship.

130. Front Vet Sci. 2023 Jul 3;10:1159331. doi: 10.3389/fvets.2023.1159331.
Poultry health constraints in smallholder village poultry systems in Northern Ghana and Central Tanzania.
Ouma EA(1)(2), Kankya C(3), Dione M(1), Kelly T(2)(4), Enahoro D(1)(2), Chiwanga G(2)(5), Abukari Y(6), Msoffe P(2)(7), Kayang BB(2)(8), Zhou H(2)(9).
Author information:
(1)International Livestock Research Institute, Nairobi, Kenya.

(2)Feed the Future Innovation Lab for Genomics to Improve Poultry, University of California, Davis, Davis, CA, United States.
(3)Department of Biosecurity, Ecosystems and Veterinary Public Health, College of Veterinary Medicine, Animal Resources and Biosecurity, Makerere University, Kampala, Uganda.
(4)One Health Institute, University of California, Davis, Davis, CA, United States.
(5)Tanzania Veterinary Laboratory Agency, South Zone, Mtwara, Tanzania.
(6)Regional Department of Agriculture, Northern Regional Coordinating Council, Tamale, Ghana.
(7)Department of Veterinary Medicine and Public Health, Sokoine University of Agriculture, Morogoro, Tanzania.
(8)Department of Animal Science, School of Agriculture, College of Basic and Applied Sciences, University of Ghana, Accra, Ghana.
(9)Department of Animal Science, University of California, Davis, Davis, CA, United States.
INTRODUCTION: Smallholder poultry production is a major contributor to food security and rural livelihoods in low-and middle-income countries. However, infectious diseases limit improvements to smallholder poultry production and performance of the sector in general. Infectious diseases of poultry, especially viral diseases, have major impacts on the health and productivity of flocks and account for significant morbidities and mortalities of birds each year.
METHODS: This study utilized participatory epidemiology approaches to better understand the poultry health constraints and challenges faced by smallholder poultry producers in village poultry systems in Northern Ghana and Central Tanzania.
RESULTS: The results show dominance of small-scale semi-intensive and extensive scavenging poultry production systems in the study areas. Newcastle disease ranked as the highest cause of morbidity and mortality in chickens in the two countries. The disease occurred mainly during the months coinciding with the dry season in both countries. Other health challenges among poultry flocks included worm infestation, fowl pox, coryza, and coccidiosis. Producers, especially in rural locations, had poor access to veterinary services and critical inputs necessary for poultry production. In the Northern region of Ghana, producers lacked definitive diagnoses for sick poultry due to a shortage of veterinary personnel and diagnostic laboratories.
DISCUSSION: These challenges point to the need for increased investment in poultry disease control and prevention programs, particularly in rural areas. Interventions focused on expansion of veterinary and agricultural extension services and diagnostic laboratory capacity in rural areas and increased gender-sensitive training to enhance smallholder knowledge in poultry husbandry and disease prevention measures will support the development of the smallholder village poultry systems. Tapping into the diverse genetic reservoir of local chicken ecotypes with enhanced resistance to Newcastle disease through genomic selection, coupled with models for enhancing ND vaccination supply and use in the rural areas are potential future avenues for addressing ND constraints to production.

131. Expert Rev Anti Infect Ther. 2023 Jul-Dec;21(10):1025-1055. doi: 10.1080/14787210.2023.2259106. Epub 2023 Oct 27.
Current rates of purchasing of antibiotics without a prescription across sub-Saharan Africa; rationale and potential programmes to reduce inappropriate dispensing and resistance.
Sono TM(1)(2), Yeika E(3), Cook A(4)(5), Kalungia A(6), Opanga SA(7), Acolatse JEE(8), Sefah IA(9), Jelić AG(10), Campbell S(1)(11), Lorenzetti G(4), Ul Mustafa Z(12)(13), Marković-Peković V(10), Kurdi A(1)(14)(15)(16), Anand Paramadhas BD(17), Rwegerera GM(18)(19), Amu AA(20), Alabi ME(21), Wesangula E(22), Oluka M(7), Khuluza F(23), Chikowe I(23), Fadare JO(24)(25), Ogunleye OO(26)(27), Kibuule D(28), Hango E(29), Schellack N(30), Ramdas N(1), Massele A(31), Mudenda S(6)(32), Hoxha I(33), Moore CE(4), Godman B(1)(14), Meyer JC(1)(34).
Author information:
(1)Department of Public Health Pharmacy and Management, Schoolof Pharmacy, Sefako Makgatho Health Sciences University, Garankuwa, Pretoria, South Africa.
(2)Saselamani Pharmacy, Saselamani, South Africa.
(3)Programs coordinator/Technical supervisor for HIV/Malaria, Delegation of Public Health, Cameroon.
(4)Centre for Neonatal and Paediatric Infection, Institute of Infection and Immunity, St. George's University of London, London, UK.
(5)Nuffield Department of Population Health, Health Economics Research Centre, University of Oxford, Oxford, UK.
(6)Department of Pharmacy, School of Health Sciences, University of Zambia, Lusaka, Zambia.
(7)Department of Pharmacology, Clinical Pharmacy and Pharmacy Practice, School of Pharmacy, University of Nairobi, Nairobi, Kenya.
(8)Pharmacy Directorate, Cape Coast Teaching Hospital (CCTH), Cape Coast, Ghana.
(9)Pharmacy Practice Department, School of Pharmacy, University of Health and Allied Sciences, Volta Region, Ho, Ghana.
(10)Department of Pharmacy, Faculty of Medicine, University of Banja Luka, Banja Luka, Bosnia & Herzegovina.
(11)Centre for Epidemiology and Public Health, School of Health Sciences, University of Manchester, Manchester, UK.
(12)Discipline of Clinical Pharmacy, School of Pharmaceutical Sciences, Universiti Sains Malaysia, Gelugor, Penang, Malaysia.
(13)Department of Pharmacy Services, District Headquarter (DHQ) Hospital, Pakpattan, Pakistan.
(14)Department of Pharmacoepidemiology, Strathclyde Institute of Pharmacy and Biomedical Sciences, University of Strathclyde, Glasgow, UK.
(15)Department of Pharmacology, College of Pharmacy, Hawler Medical University, Erbil, Iraq.
(16)College of Pharmacy, Al-Kitab University, Kirkuk, Iraq.
(17)Department of Health Services Management, Central Medical Stores, Ministry of Health, Gaborone, Botswana.
(18)Faculty of Medicine, Department of Internal Medicine, University of

Botswana, Gaborone, Botswana.
(19)DestinyMedical and Research Solutions Proprietary Limited, Gaborone, Botswana.
(20)Pharmacy Department, Eswatini Medical Christian University, Mbabane, Kingdom of Eswatini.
(21)School of Pharmaceutical Sciences, College of Health Sciences,University of Kwazulu-natal (UKZN), Durban, South Africa.
(22)East Central and Southern Africa Health Community, Arusha, Tanzania.
(23)Pharmacy Department, Kamuzu University of Health Sciences (KUHeS) (formerly College of Medicine), Blantyre, Malawi.
(24)Department of Pharmacology and Therapeutics, Ekiti State University, Ado-Ekiti, Nigeria.
(25)Department of Medicine, Ekiti State University Teaching Hospital, Ado-Ekiti, Nigeria.
(26)Department of Pharmacology, Therapeutics and Toxicology, Lagos State University College of Medicine, Ikeja, Lagos, Nigeria.
(27)Department of Medicine, Lagos State University Teaching Hospital, Ikeja, Uganda.
(28)Department of Pharmacology & Therapeutics, Busitema University, Mbale, Uganda.
(29)Department of Pharmacy Practice and Policy, School of Pharmacy, Faculty of Health Sciences, University of Namibia, Windhoek, Namibia.
(30)Department of Pharmacology, Faculty of Health Sciences, University of Pretoria, Pretoria, SouthAfrica.
(31)Department of Clinical Pharmacology and Therapeutics, Hurbert Kairuki Memorial University, Dar Es Salaam, Tanzania.
(32)Africa Center of Excellence for Infectious Diseases of Humans and Animals, School of Veterinary Meicine, University of Zambia, Lusaka, Zambia.
(33)Department of Pharmacy, Faculty of Medicine, University of Medicine Tirana, Tirana, Albania.
(34)South African Vaccination and Immunisation Centre, Sefako Makgatho HealthSciences University, Garankuwa, Pretoria, South Africa.
INTRODUCTION: Antimicrobial resistance (AMR) is a global concern. Currently, the greatest mortality due to AMR is in Africa. A key driver continues to be high levels of dispensing of antibiotics without a prescription.
AREAS COVERED: A need to document current rates of dispensing, their rationale and potential ways forward including antimicrobial stewardship programmes (ASPs). A narrative review was undertaken. The highest rates of antibiotic purchasing were in Eritrea (up to 89.2% of antibiotics dispensed), Ethiopia (up to 87.9%), Nigeria (up to 86.5%), Tanzania (up to 92.3%) and Zambia (up to 100% of pharmacies dispensing antibiotics without a prescription). However, considerable variation was seen with no dispensing in a minority of countries and situations. Key drivers of self-purchasing included high co-payment levels for physician consultations and antibiotic costs, travel costs, convenience of pharmacies, patient requests, limited knowledge of antibiotics and AMR and weak enforcement. ASPs have been introduced in some African countries along with quality targets to reduce inappropriate dispensing, centering on educating

pharmacists and patients.
EXPERT OPINION: ASP activities need accelerating among community pharmacies alongside quality targets, with greater monitoring of pharmacists' activities to reduce inappropriate dispensing. Such activities, alongside educating patients and healthcare professionals, should enhance appropriate dispensing of antibiotics and reduce AMR.

132. Transfusion. 2023 Jul;63(7):1354-1365. doi: 10.1111/trf.17449. Epub 2023 Jun 16.
SARS-CoV-2 seroprevalence among blood donors in Uganda: 2019-2022.
Bloch EM(1), Kyeyune D(2), White JL(1), Ddungu H(3), Ashokkumar S(4), Habtehyimer F(1), Baker O(5), Kasirye R(6), Patel EU(1), Grabowski MK(1), Musisi E(2), Moses K(2), Hume HA(7), Lubega I(6), Shrestha R(1), Motevalli M(1), Fernandez RE(5), Reynolds SJ(4), Redd AD(4), Wambongo Musana H(6), Dhabangi A(8), Ouma J(6), Eroju P(6), de Lange T(9), Fowler MG(1), Musoke P(6), Stramer SL(10), Whitby D(11), Zimmerman PA(12), McCullough J(13), Sachithanandham J(14), Pekosz A(14), Goodrich R(15), Quinn TC(4), Ness PM(1), Laeyendecker O(4), Tobian AAR(1).
Author information:
(1)Department of Pathology, School of Medicine, Johns Hopkins University, Baltimore, Maryland, USA.
(2)Uganda Blood Transfusion Services, Kampala, Uganda.
(3)Uganda Cancer Institute, Kampala, Uganda.
(4)Division of Intramural Research, National Institute of Allergy and Infectious Diseases, National Institutes of Health, Bethesda, Maryland, USA.
(5)Department of Medicine, School of Medicine, Johns Hopkins University, Baltimore, Maryland, USA.
(6)MUJHU Research Collaboration, Kampala, Uganda.
(7)Department of Pediatrics, University of Montreal, Montréal, Quebec, Canada.
(8)Department of Paediatrics and Child Health, Makerere University College of Health Sciences, Kampala, Uganda.
(9)National Institute of Allergy and Infectious Diseases Office of Cyber Infrastructure and Computational Biology, Bethesda, Maryland, USA.
(10)Scientific Affairs, American Red Cross, Gaithersburg, Maryland, USA.
(11)Viral Oncology Section, AIDS and Cancer Virus Program, Frederick National Laboratory for Cancer Research, Frederick, Maryland, USA.
(12)The Center for Global Health & Diseases, Pathology Department, Case Western Reserve University, Cleveland, Ohio, USA.
(13)College of Health Solutions, Arizona State University, Phoenix, Arizona, USA.
(14)Department of Molecular Microbiology and Immunology, Johns Hopkins Bloomberg School of Public Health, Baltimore, Maryland, USA.
(15)Department of Microbiology, Immunology and Pathology, Colorado State University, Fort Collins, Colorado, USA.
BACKGROUND: The true burden of COVID-19 in low- and middle-income countries remains poorly characterized, especially in Africa. Even prior to the availability of SARS-CoV-2 vaccines, countries in Africa had lower numbers of reported COVID-19 related hospitalizations and deaths than other regions

globally.
METHODS: Ugandan blood donors were evaluated between October 2019 and April 2022 for IgG antibodies to SARS-CoV-2 nucleocapsid (N), spike (S), and five variants of the S protein using multiplexed electrochemiluminescence immunoassays (MesoScale Diagnostics, Rockville, MD). Seropositivity for N and S was assigned using manufacturer-provided cutoffs and trends in seroprevalence were estimated by quarter. Statistically significant associations between N and S antibody seropositivity and donor characteristics in November-December 2021 were assessed by chi-square tests.
RESULTS: A total of 5393 blood unit samples from donors were evaluated. N and S seropositivity increased throughout the pandemic to 82.6% in January-April 2022. Among seropositive individuals, N and S antibody levels increased ≥9-fold over the study period. In November-December 2021, seropositivity to N and S antibody was higher among repeat donors (61.3%) compared with new donors (55.1%; p = .043) and among donors from Kampala (capital city of Uganda) compared with rural regions (p = .007). Seropositivity to S antibody was significantly lower among HIV-seropositive individuals (58.8% vs. 84.9%; p = .009).
CONCLUSIONS: Despite previously reported low numbers of COVID-19 cases and related deaths in Uganda, high SARS-CoV-2 seroprevalence and increasing antibody levels among blood donors indicated that the country experienced high levels of infection over the course of the pandemic.

133. Int J Gynaecol Obstet. 2023 Jul;162(1):29-38. doi: 10.1002/ijgo.14843. Epub 2023
Standardizing case definitions for monitoring the safety of maternal vaccines globally: GAIA definitions, a review of progress to date.
Davies HG(1)(2), Bowman C(3), Watson G(4), Dodd C(5), Jones CE(4)(6), Munoz FM(7), Heath PT(1), Cutland CL(8), Le Doare K(1)(2).
Author information:
(1)Centre for Paediatric and Neonatal Infection, Institute of Infection & Immunity, St George's, University of London, London, UK.
(2)Makerere University Johns Hopkins University Research Collaboration, Kampala, Uganda.
(3)Department of Microbiology, University College London Hospital, London, UK.
(4)Department of Paediatric Infectious Diseases and Immunology, University Hospital Southampton, Southampton, UK.
(5)Julius Global Health, Universitair Medisch Centrum, Utrecht, the Netherlands.
(6)Clinical and Experimental Sciences, University of Southampton and NIHR Southampton Clinical Research Facility and Biomedical Research Centre, University Hospital Southampton NHS Foundation Trust, Southampton, UK.
(7)Departments of Pediatrics and Molecular Virology and Microbiology, Baylor College of Medicine, Houston, Texas, USA.
(8)African Leadership in Vaccinology Expertise (Alive), Faculty of Health Sciences, University of Witwatersrand, Johannesburg, South Africa.
In 2014, the Global Alignment on Immunization safety Assessment in pregnancy consortium (GAIA) was formed, with the goal of developing a harmonized, globally-concerted approach to actively monitor the safety of vaccines in pregnancy. A total of 26 standardized definitions for the classification of

adverse events have been developed. The aim of this review was to identify and describe studies undertaken to assess the performance of these definitions. A literature search was undertaken to identify published studies assessing the performance of the definitions, and reference lists were snowballed. Data were abstracted by two investigators and a narrative review of the results is presented. Four studies that have evaluated 13 GAIA case definitions (50%) were identified. Five case definitions have been assessed in high-income settings only. Recommendations have been made by the investigators to improve the performance of the definitions. These include ensuring consistency across definitions, removal of the potential for ambiguity or variations in interpretation and ensuring that higher-level criteria are acceptable at lower levels of confidence. Future research should prioritize the key case definitions that have not been assessed in low- and middle-income settings, as well as the 13 that have not undergone any validation.

134. medRxiv. 2023 Jun 29:2023.06.26.23291885. doi: 10.1101/2023.06.26.23291885. Preprint.
Targeting hepatitis B vaccine escape using immunogenetics in Bangladeshi infants.
Butler-Laporte G(1)(2)(3), Auckland K(1), Noor Z(4), Kabir M(4), Alam M(4), Carstensen T(5)(6), Wojcik GL(7), Chong AY(1), Pomilla C(5), Noble JA(8)(9), McDevitt SL(10), Smits G(11), Wareing S(12), van der Klis FR(11), Jeffery K(12), Kirkpatrick BD(13), Sirima S(14), Madhi S(15), Elliott A(16)(17), Richards JB(2)(18)(19)(20)(21), Hill AV(22), Duggal P(7); PROVIDE authors, Cryptosporidiosis Birth Cohort authors; Sandhu MS(23), Haque R(4), Petri WA Jr(24), Mentzer AJ(1).
Author information:
(1)Wellcome Centre for Human Genetics, University of Oxford, Oxford, United Kingdom.
(2)Lady Davis Institute, Jewish General Hospital, McGill University, Montréal, Québec, Canada.
(3)Division of Infectious Diseases, McGill University Health Centre, Montréal, Québec, Canada.
(4)International Centre for Diarrhoeal Disease Research, Bangladesh, Dhaka, Bangladesh.
(5)Wellcome Trust Sanger Institute, University of Cambridge, Hinxton, United Kingdom.
(6)Queen Mary University of London, London, United Kingdom.
(7)Department of Epidemiology, Johns Hopkins Bloomberg School of Public Health, Baltimore, Maryland, USA.
(8)Children's Hospital Oakland Research Institute, Oakland, California, USA.
(9)Department of Pediatrics, University of California, San Francisco, California, USA.
(10)Media Labs, Inc. Alameda, CA, USA.
(11)National Institute for Public Health and the Environment, Bilthoven, The Netherlands.
(12)Microbiology Department, John Radcliffe Hospital, Oxford University NHS

Foundation Trust, Oxford, UK.
(13)Department of Microbiology and Molecular Genetics, Vaccine Testing Center, University of Vermont College of Medicine, Vermont, USA.
(14)Groupe de Recherche Action en Santé (GRAS) 06 BP 10248 Ouagadougou, Burkina Faso.
(15)South African Medical Research Council Vaccines and Infectious Diseases Analytics Research Unit, University of the Witwatersrand, Johannesburg, South Africa.
(16)Department of Clinical Research, London School of Hygiene & Tropical Medicine, London, UK.
(17)Medical Research Council/Uganda Virus Research Institute and London School of Hygiene & Tropical Medicine Uganda Research Unit, Entebbe, Uganda.
(18)Department of Human Genetics, McGill University, Montréal, Québec, Canada.
(19)5 Prime Sciences Inc, Montreal, Quebec, Canada.
(20)Department of Epidemiology, Biostatistics and Occupational Health, McGill University, Montréal, Québec, Canada.
(21)Department of Twin Research, King's College London, London, United Kingdom.
(22)The Jenner Institute, University of Oxford, Oxford, United Kingdom.
(23)Department of Epidemiology and Biostatistics, School of Public Health, Imperial College London, United Kingdom.
(24)Department of Medicine, Infectious Diseases and International Health, University of Virginia School of Medicine, Charlottesville, Virginia, USA.
Hepatitis B virus (HBV) vaccine escape mutants (VEM) are increasingly described, threatening progress in control of this virus worldwide. Here we studied the relationship between host genetic variation, vaccine immunogenicity and viral sequences implicating VEM emergence. In a cohort of 1,096 Bangladeshi children, we identified human leukocyte antigen (HLA) variants associated with response vaccine antigens. Using an HLA imputation panel with 9,448 south Asian individuals DPB1*04:01 was associated with higher HBV antibody responses (p=4.5×10-30). The underlying mechanism is a result of higher affinity binding of HBV surface antigen epitopes to DPB1*04:01 dimers. This is likely a result of evolutionary pressure at the HBV surface antigen 'a-determinant' segment incurring VEM specific to HBV. Prioritizing pre-S isoform HBV vaccines may tackle the rise of HBV vaccine evasion.

135. BMC Infect Dis. 2023 Jun 27;23(1):435. doi: 10.1186/s12879-023-08403-9.
Molecular characterisation of human adenoviruses associated with respiratory infections in Uganda.
Ukuli QA(1), Erima B(1), Mubiru A(1), Atim G(1), Tugume T(1), Kibuuka H(1), Mworozi E(2), Ducatez MF(3), Wabwire-Mangen F(1)(4), Byarugaba DK(5)(6).
Author information:
(1)Makerere University Walter Reed Project, Kampala, Uganda.
(2)College of Health Sciences, Makerere University, P.O. Box 7062, Kampala, Uganda.
(3)IHAP, UMR1225, Université de Toulouse, INRAE, ENVT, Toulouse, France.
(4)School of Public Health, Makerere University, P.O. Box 7062, Kampala, Uganda.
(5)Makerere University Walter Reed Project, Kampala, Uganda.

denis.byarugaba@mak.ac.ug.
(6)College of Veterinary Medicine, Makerere University, P.O. Box 7062, Kampala, Uganda. denis.byarugaba@mak.ac.ug.
Human adenoviruses (HAdV) are a diverse group of viruses causing a broad range of infections of the respiratory, urogenital and gastrointestinal tracts and keratoconjunctivitis. There are seven species of human adenoviruses with 113 genotypes which may contain multiple genetic variants. This study characterised respiratory human adenoviruses and associated factors in samples collected from selected hospitals in Uganda. A total of 2,298 nasopharyngeal samples were collected between the period of 2008 to 2016 from patients seeking health care at tertiary hospitals for influenza-like illness. They were screened by polymerase chain reaction (PCR) to determine the prevalence of HAdV. HAdV was cultured in A549 cell lines and the hexon gene was sequenced for genotyping. Of the 2,298 samples tested, 225 (9.8%) were adenovirus-positive by PCR. Age was found to be significantly associated with HAdV infections ($p = 0.028$) with 98% (220/225) of the positives in children aged 5 years and below and none in adults above 25 years of age. The sequenced isolates belonged to species HAdV-B and HAdV-C with most isolates identified as genotype B3. The results showed a high prevalence and genetic diversity in respiratory HAdV circulating in Ugandan population. Deeper genomic characterization based on whole genome sequencing may be necessary to further elucidate possible transmission and impact of current adenovirus-vectored vaccines in Africa.

136. Glob Health Sci Pract. 2023 Jun 21;11(3):e2200496. doi: 10.9745/GHSP-D-22-00496.
Community Barriers, Enablers, and Normative Embedding of Second Year of Life Vaccination in Ghana: A Qualitative Study.
Wolff B(1), Aborigo RA(2), Dalaba M(2)(3), Opare JKL(4), Conklin L(5), Bonsu G(6), Amponsa-Achiano K(6).
Author information:
(1)Global Immunization Division, U.S. Centers for Disease Control and Prevention, Atlanta, GA, USA. brentwolff@cdc.gov.
(2)Navrongo Health Research Centre, Navrongo, Ghana.
(3)Institute of Health Research, University of Health and Allied Sciences, Ho, Ghana.
(4)African Field Epidemiology Network, Kampala, Uganda.
(5)Global Immunization Division, U.S. Centers for Disease Control and Prevention, Atlanta, GA, USA.
(6)Ghana Health Service, Accra, Ghana.
INTRODUCTION: Coverage rates for second year of life (2YL) vaccination still lag behind infant vaccination in most settings. We conducted a qualitative baseline study of community barriers and enablers to acceptance of 2YL vaccines in Ghana 4 years after introducing the second dose of the measles-containing vaccine.
METHODS: We conducted 26 focus group discussions in 2016 with men and women caregivers from mixed urban, peri-urban, and rural areas, as well as pastoralists, using semistructured topic guides based on the Health Belief Model theory. We conducted a thematic analysis of the discussion using NVivo software. We use Normalization Process Theory to contextualize results as a snapshot of a

dynamic process of community adaptation to change to a well-established routine immunization schedule following 2YL introduction.
RESULTS: Routine immunization for infants enjoys resilient demand, grounded in strong community norms despite surprisingly low levels of vaccine literacy. Despite best practices like integration with the established 18-month "weighing visit," demand for 2YL vaccination is still conditional on individual awareness and competition for limited maternal time, household resources, and other health concerns. An embedded norm that children should be fully vaccinated by 12 months originally sustained Expanded Programme for Immunization goals but now discouraged some caregivers from seeking vaccines for children perceived to be "too old" to vaccinate. Caregivers cited greater costs and inconvenience of taking older, heavier children in for vaccination and anticipated criticism from both community members and health care providers for coming "too late."
CONCLUSION: Closing the 2YL vaccination coverage gap will ultimately require modifying embedded norms among caregivers and health care providers alike. Time is necessary but not sufficient to reach this goal. Progress can be accelerated by increasing the level of community and institutional engagement and adapting services where possible to minimize added costs to caregivers of vaccinating older children.

137. Vaccines (Basel). 2023 Jun 15;11(6):1103. doi: 10.3390/vaccines11061103.
COVID-19 Impact on DTP Vaccination Trends in Africa: A Joinpoint Regression Analysis.
Aguinaga-Ontoso I(1)(2), Guillen-Aguinaga S(1), Guillen-Aguinaga L(1)(3), Alas-Brun R(1), Onambele L(4), Aguinaga-Ontoso E(5), Guillen-Grima F(1)(2)(6).
Author information:
(1)Department of Health Sciences, Public University of Navarra, 31008 Pamplona, Spain.
(2)Healthcare Research Institute of Navarra (IdiSNA), 31008 Pamplona, Spain.
(3)Sykepleieavdelingen, Suldal Sykehjem, 4230 Sand, Norway.
(4)School of Health Sciences, Catholic University of Central Africa, Yaounde 1110, Cameroon.
(5)Department of Sociosanitary Sciences, University of Murcia, 30120 Murcia, Spain.
(6)Department of Preventive Medicine, Clínica Universidad de Navarra, 31008 Pamplona, Spain.
BACKGROUND: Deaths due to vaccine-preventable diseases are one of the leading causes of death among African children. Vaccine coverage is an essential measure to decrease infant mortality. The COVID-19 pandemic has affected the healthcare system and may have disrupted vaccine coverage.
METHODS: DTP third doses (DTP3) Vaccine Coverage was extracted from UNICEF databases from 2012 to 2021 (the last available date). Joinpoint regression was performed to detect the point where the trend changed. The annual percentage change (APC) with 95% confidence intervals (95% CI) was calculated for Africa and the regions. We compared DTP3 vaccination coverage in 2019-2021 in each country using the Chi-square test.
RESULT: During the whole period, the vaccine coverage in Africa increased with

an Annual Percent change of 1.2% (IC 95% 0.9-1.5): We detected one joinpoint in 2019. In 2019-2021, there was a decrease in DTP3 coverage with an APC of -3.5 (95% -6.0; -0,9). (p < 0.001). Vaccination rates decreased in many regions of Sub-Saharan Africa, especially in Eastern and Southern Africa. There were 26 countries (Angola, Cabo Verde, Comoros, Congo, Côte d'Ivoire, Democratic Republic of the Congo, Djibouti, Ethiopia, Eswatini, The Gambia, Guinea-Bissau, Liberia, Madagascar, Malawi, Mauritania, Mauritius, Mozambique, Rwanda, Senegal, Seychelles, Sierra Leone, Sudan, Tanzania, Togo, Tunisia, Uganda, and Zimbabwe) where the vaccine coverage during the two years decreased. There were 10 countries (Angola, Cabo Verde, Comoros, Democratic Republic of the Congo, Eswatini, The Gambia, Mozambique, Rwanda, Senegal, and Sudan) where the joinpoint regression detected a change in the trend.
CONCLUSIONS: COVID-19 has disrupted vaccine coverage, decreasing it all over Africa.

138. Trop Med Health. 2023 Jun 12;51(1):34. doi: 10.1186/s41182-023-00526-8.
Medicinal plants used for treatment of malaria by indigenous communities of Tororo District, Eastern Uganda.
Tabuti JRS(1), Obakiro SB(2), Nabatanzi A(3), Anywar G(3), Nambejja C(4), Mutyaba MR(5), Omara T(6), Waako P(7).
Author information:
(1)Department of Environmental Management, Makerere University, P.O. Box 7062, Kampala, Uganda.
(2)Department of Pharmacology and Therapeutics, Faculty of Health Sciences, Busitema University, P.O. Box 1460, Mbale, Uganda. sobakiro@gmail.com.
(3)Department of Plant Sciences, Microbiology & Biotechnology, College of Natural Sciences, Makerere University, P.O. Box 7062, Kampala, Uganda.
(4)Natural Chemotherapeutics Research Institute (NCRI), Ministry of Health, P.O. Box 4864, Kampala, Uganda.
(5)National Drug Authority, Ministry of Health, P.O. Box 23096, Kampala, Uganda.
(6)Institute of Chemistry of Renewable Resources, Department of Chemistry, University of Natural Resources and Life Sciences, Vienna (BOKU), The Tulln University and Research Center (UFT), Konrad-Lorenz-Straße 24, 3430, Tulln an der Donau, Austria.
(7)Department of Pharmacology and Therapeutics, Faculty of Health Sciences, Busitema University, P.O. Box 1460, Mbale, Uganda.
BACKGROUND: Malaria remains the leading cause of death in sub-Saharan Africa. Although recent developments such as malaria vaccine trials inspire optimism, the search for novel antimalarial drugs is urgently needed to control the mounting resistance of Plasmodium species to the available therapies. The present study was conducted to document ethnobotanical knowledge on the plants used to treat symptoms of malaria in Tororo district, a malaria-endemic region of Eastern Uganda.
METHODS: An ethnobotanical study was carried out between February 2020 and September 2020 in 12 randomly selected villages of Tororo district. In total, 151 respondents (21 herbalists and 130 non-herbalists) were selected using multistage random sampling method. Their awareness of malaria, treatment-seeking

behaviour and herbal treatment practices were obtained using semi-structured questionnaires and focus group discussions. Data were analysed using descriptive statistics, paired comparison, preference ranking and informant consensus factor.
RESULTS: A total of 45 plant species belonging to 26 families and 44 genera were used in the preparation of herbal medicines for management of malaria and its symptoms. The most frequently mentioned plant species were Vernonia amygdalina, Chamaecrista nigricans, Aloe nobilis, Warburgia ugandensis, Abrus precatorius, Kedrostis foetidissima, Senna occidentalis, Azadirachta indica and Mangifera indica. Leaves (67.3%) were the most used plant part while maceration (56%) was the major method of herbal remedy preparation. Oral route was the predominant mode of administration with inconsistencies in the posology prescribed.
CONCLUSION: This study showed that the identified medicinal plants in Tororo district, Uganda, are potential sources of new antimalarial drugs. This provides a basis for investigating the antimalarial efficacy, phytochemistry and toxicity of the unstudied species with high percentage use values to validate their use in the management of malaria.

139. NPJ Vaccines. 2023 Jun 10;8(1):91. doi: 10.1038/s41541-023-00685-z.
The rVSV-EBOV vaccine provides limited cross-protection against Sudan virus in guinea pigs.
Cao W(#)(1), He S(#)(1), Liu G(#)(1), Schulz H(1), Emeterio K(1), Chan M(1), Tierney K(1), Azaransky K(1), Soule G(1), Tailor N(1), Salawudeen A(2), Nichols R(3), Fusco J(3), Safronetz D(1)(2), Banadyga L(4)(5).
Author information:
(1)Special Pathogens Program, National Microbiology Laboratory, Public Health Agency of Canada, Winnipeg, MB, R3E 3R2, Canada.
(2)Department of Medical Microbiology and Infectious Diseases, University of Manitoba, Winnipeg, MB, R3E 0J9, Canada.
(3)Public Health Vaccines, Cambridge, MA, 02142, USA.
(4)Special Pathogens Program, National Microbiology Laboratory, Public Health Agency of Canada, Winnipeg, MB, R3E 3R2, Canada. logan.banadyga@phac-aspc.gc.ca.
(5)Department of Medical Microbiology and Infectious Diseases, University of Manitoba, Winnipeg, MB, R3E 0J9, Canada. logan.banadyga@phac-aspc.gc.ca.
(#)Contributed equally
Recombinant vesicular stomatitis viruses (rVSVs) engineered to express heterologous viral glycoproteins have proven to be remarkably effective vaccines. Indeed, rVSV-EBOV, which expresses the Ebola virus (EBOV) glycoprotein, recently received clinical approval in the United States and Europe for its ability to prevent EBOV disease. Analogous rVSV vaccines expressing glycoproteins of different human-pathogenic filoviruses have also demonstrated efficacy in pre-clinical evaluations, yet these vaccines have not progressed far beyond research laboratories. In the wake of the most recent outbreak of Sudan virus (SUDV) in Uganda, the need for proven countermeasures was made even more acute. Here we demonstrate that an rVSV-based vaccine expressing the SUDV glycoprotein (rVSV-SUDV) generates a potent humoral immune response that protects guinea pigs from SUDV disease and death. Although the

cross-protection generated by rVSV vaccines for different filoviruses is thought to be limited, we wondered whether rVSV-EBOV might also provide protection against SUDV, which is closely related to EBOV. Surprisingly, nearly 60% of guinea pigs that were vaccinated with rVSV-EBOV and challenged with SUDV survived, suggesting that rVSV-EBOV offers limited protection against SUDV, at least in the guinea pig model. These results were confirmed by a back-challenge experiment in which animals that had been vaccinated with rVSV-EBOV and survived EBOV challenge were inoculated with SUDV and survived. Whether these data are applicable to efficacy in humans is unknown, and they should therefore be interpreted cautiously. Nevertheless, this study confirms the potency of the rVSV-SUDV vaccine and highlights the potential for rVSV-EBOV to elicit a cross-protective immune response.

140. PLoS One. 2023 Jun 9;18(6):e0287041. doi: 10.1371/journal.pone.0287041.
Limitations and opportunities of smallholders' practical knowledge when dealing with pig health issues in northern Uganda.
Arvidsson A(1), Fischer K(1), Chenais E(2), Kiguli J(3), Sternberg-Lewerin S(4), Ståhl K(2).
Author information:
(1)Department of Urban and Rural Development, Swedish University of Agricultural Sciences, Uppsala, Sweden.
(2)Department of Epidemiology and Disease Control, National Veterinary Institute (SVA), Uppsala, Sweden.
(3)Department of Community Health and Behavioural Sciences, Makerere University, Kampala, Uganda.
(4)Department of Biomedical Science and Veterinary Public Health, Swedish University of Agricultural Sciences, Uppsala, Sweden.
Pig production has a short history in Uganda. The majority of pigs are kept by smallholder farmers in rural areas where access to veterinary services is limited, and pig keeping has been suggested as a potential pathway out of poverty for smallholders. Previous research has identified the disease of African swine fever (ASF) as a major threat, causing high mortalities in pigs. With no available cure or vaccine, the only option is to implement biosecurity measures, i.e. strategies that prevent the spread of ASF. This paper draws on data from four months of ethnographic fieldwork in rural northern Uganda. Combining methods of participant observation, semi-structured interviews, focus group discussions and a survey, the aim was to improve understanding of smallholders' perceptions and responses to pig health issues such as ASF. Applying the concept of practical knowledge, this paper analyses the potential and limitations of smallholders' practice-based knowledge as a means of dealing with pig health issues. The results show that while pigs were appreciated locally for providing an income, many informants found it difficult to deal with pig diseases effectively. Consequently, informants commonly expressed a need for other kinds of knowledge in their pig production, indicating that veterinary advice can play an important role in reducing the negative impact of pig health issues. For animal health provision to have relevance in this context, however, veterinary practitioners must pay close attention to smallholders' priorities

and ways of knowing in their livestock keeping. Results further show that pig
health issues made some informants abandon pig production altogether. To enhance
the potential of pig production as a poverty mitigation strategy in Uganda,
research and policy need to focus on ways of bettering general conditions for
smallholder pig keeping, including improving the quality of and access to
veterinary services in rural areas.

141. Clin Infect Dis. 2023 Jun 8;76(11):2018-2024. doi: 10.1093/cid/ciad066.
Guidance on the Use of Convalescent Plasma to Treat Immunocompromised Patients
With Coronavirus Disease 2019.
Bloch EM(1), Focosi D(2), Shoham S(3), Senefeld J(4), Tobian AAR(1), Baden
LR(5), Tiberghien P(6), Sullivan DJ(7), Cohn C(8), Dioverti V(3), Henderson
JP(9), So-Osman C(10)(11), Juskewitch JE(12), Razonable RR(13), Franchini M(14),
Goel R(15), Grossman BJ(16), Casadevall A(7), Joyner MJ(4), Avery RK(3),
Pirofski LA(17), Gebo KA(3).
Author information:
(1)Department of Pathology, Johns Hopkins University School of Medicine,
Baltimore, Maryland, USA.
(2)North-Western Tuscany Blood Bank, Pisa University Hospital, Pisa, Italy.
(3)Department of Medicine, Division of Infectious Diseases, Johns Hopkins
University School of Medicine, Baltimore, Maryland, USA.
(4)Department of Anesthesiology and Perioperative Medicine, Mayo Clinic,
Rochester, Minnesota, USA.
(5)Department of Infectious Diseases, Brigham and Women's Hospital, Boston,
Massachusetts, USA.
(6)Etablissement Français du Sang, La Plaine-St-Denis and Université de
Franche-Comté, Besançon, France.
(7)Department of Molecular Microbiology and Immunology, Johns Hopkins Bloomberg
School of Public Health, Baltimore, MD, USA.
(8)Department of Laboratory Medicine and Pathology, University of Minnesota,
Minneapolis, Minnesota, USA.
(9)Departments of Internal Medicine (Division of Infectious Diseases) and
Molecular Microbiology, Washington University School of Medicine, St. Louis, MO,
USA.
(10)Department Transfusion Medicine, Division Blood Bank, Sanquin Blood Supply
Foundation, Amsterdam, The Netherlands.
(11)Department Haematology, Erasmus Medical Centre, Rotterdam, The Netherlands.
(12)Department of Laboratory Medicine and Pathology, Mayo Clinic, Rochester
campus, Minnesota, USA.
(13)Department of Internal Medicine, Division of Infectious Diseases, Mayo
Clinic, Rochester, Minnesota, USA.
(14)Department of Hematology and Transfusion Medicine, Carlo Poma Hospital,
Mantua, Italy.
(15)Division of Hematology/Oncology, Simmons Cancer Institute at SIU School of
Medicine and Mississippi Valley Regional Blood Center, Springfield, Illinois,
USA.
(16)Department of Pathology & Immunology, Washington University School of

Medicine, St. Louis, Missouri, USA.
(17)Department of Medicine, Infectious Diseases, Albert Einstein College of Medicine, Bronx, New York, USA.
Coronavirus disease 2019 (COVID-19) convalescent plasma (CCP) is a safe and effective treatment for COVID-19 in immunocompromised (IC) patients. IC patients have a higher risk of persistent infection, severe disease, and death from COVID-19. Despite the continued clinical use of CCP to treat IC patients, the optimal dose, frequency/schedule, and duration of CCP treatment has yet to be determined, and related best practices guidelines are lacking. A group of individuals with expertise spanning infectious diseases, virology and transfusion medicine was assembled to render an expert opinion statement pertaining to the use of CCP for IC patients. For optimal effect, CCP should be recently and locally collected to match circulating variant. CCP should be considered for the treatment of IC patients with acute and protracted COVID-19; dosage depends on clinical setting (acute vs protracted COVID-19). CCP containing high-titer severe acute respiratory syndrome coronavirus 2 (SARS-CoV-2) antibodies, retains activity against circulating SARS-CoV-2 variants, which have otherwise rendered monoclonal antibodies ineffective.

142. Digit Health. 2023 Jun 7;9:20552076231180733. doi: 10.1177/20552076231180733.
Social media use and COVID-19 vaccine status among a nationally representative population sample in Uganda.
Greenleaf AR(1)(2), Croker-Benn A(2), Aibo D(3), Biraro S(3), Mugisha V(4), Kwizera MH(5), Kabanda R(6)(7), Justman J(1), El-Sadr WM(1).
Author information:
(1)ICAP at Columbia University, Mailman School of Public Health, New York, NY, USA.
(2)Heilbrunn Department of Population and Family Health, Columbia University, New York, NY, USA.
(3)ICAP Uganda, Kampala, Uganda.
(4)ICAP Rwanda, Kigali, Rwanda.
(5)Department of BioStatistics, Columbia University, New York, NY, USA.
(6)Department of Health Promotion, Education & Communication, Ministry of Health, Kampala, Uganda.
(7)Faculty of Health Sciences, Uganda Martyrs University, Nkozi, Uganda.
OBJECTIVES: The effect of social media on COVID-19 vaccination behavior is sub-Saharan Africa is unclear. We conducted a study to determine social media use among a random nationally representative sample of adults in Uganda and assessed the association between recent social media use and COVID-19 vaccination uptake.
METHODS: We used data from the 2020 general population survey in Uganda, the Population-based HIV Impact Assessment Survey, to identify a probability sample for a mobile phone survey and included nonphone owners in the phone survey by asking phone owners to pass the phone.
RESULTS: In March 2022, of the 1022 survey participants, 213 (20%) did not own a mobile phone, 842 (80%) owned a mobile phone, of whom 199 (24%) indicated social media use, and 643 (76%) of whom did not use social media. Among all

participants, the most frequent source of COVID-19 vaccine information was radio. Overall, 62% reported receiving the COVID-19 vaccination. The multivariable logistic regression model found that social media use was not associated with vaccination status.
CONCLUSION: Social media users in this population sample from Uganda-who were mainly young, urban residents with higher educational attainment-continue to utilize TV, radio and health care workers for public health messages, thus the Government of Uganda should continue to conduct public health communication through these mediums.

143. PLOS Glob Public Health. 2023 Jun 5;3(6):e0001992. doi: 10.1371/journal.pgph.0001992. eCollection 2023.
Geographic mobility and treatment outcomes among people in care for tuberculosis in the Lake Victoria region of East Africa: A multi-site prospective cohort study.
Mulholland GE(1), Herce ME(2), Bahemuka UM(3), Kwena ZA(4), Jeremiah K(5), Okech BA(6), Bukusi E(4), Okello ES(5), Nanyonjo G(6), Ssetaala A(6), Seeley J(3)(7), Emch M(1)(8), Pettifor A(1), Weir SS(1), Edwards JK(1).
Author information:
(1)Department of Epidemiology, University of North Carolina at Chapel Hill, Chapel Hill, North Carolina, United States of America.
(2)Institute for Global Health and Infectious Diseases, School of Medicine, University of North Carolina at Chapel Hill, Chapel Hill, North Carolina, United States of America.
(3)Medical Research Council/Uganda Virus Research Institute & London School of Hygiene and Tropical Medicine, Uganda Research Unit, Entebbe, Uganda.
(4)Kenya Medical Research Institute, Nairobi, Kenya.
(5)Mwanza Intervention Trials Unit, Mwanza Research Centre, National Institute for Medical Research, Mwanza, Tanzania.
(6)UVRI-IAVI HIV Vaccine Program Limited, Entebbe, Uganda.
(7)Global Health and Development Department, London School of Hygiene and Tropical Medicine, London, United Kingdom.
(8)Department of Geography, University of North Carolina at Chapel Hill, Chapel Hill, North Carolina, United States of America.
Geographic mobility may disrupt continuity of care and contribute to poor clinical outcomes among people receiving treatment for tuberculosis (TB). This may occur especially where health services are not well coordinated across international borders, particularly in lower and middle income country settings. In this work, we describe mobility and the relationship between mobility and unfavorable TB treatment outcomes (i.e., death, loss to follow-up, or treatment failure) among a cohort of adults who initiated TB treatment at one of 12 health facilities near Lake Victoria. We abstracted data from health facility records for all 776 adults initiating TB treatment during a 6-month period at the selected facilities in Kenya, Tanzania, and Uganda. We interviewed 301 cohort members to assess overnight travel outside one's residential district/sub-county. In our analyses, we estimated the proportion of cohort members traveling in 2 and 6 months following initiation of TB treatment,

explored correlates of mobility, and examined the association between mobility and an unfavorable TB treatment outcome. We estimated that 40.7% (95% CI: 33.3%, 49.6%) of people on treatment for TB traveled overnight at least once in the 6 months following treatment initiation. Mobility was more common among people who worked in the fishing industry and among those with extra-pulmonary TB. Mobility was not strongly associated with other characteristics examined, however, suggesting that efforts to improve TB care for mobile populations should be broad ranging. We found that in this cohort, people who were mobile were not at increased risk of an unfavorable TB treatment outcome. Findings from this study can help inform development and implementation of mobility-competent health services for people with TB in East Africa.

144. Immunogenetics. 2023 Jun;75(3):207-214. doi: 10.1007/s00251-023-01306-8. Epub
Impact of high human genetic diversity in Africa on immunogenicity and efficacy of RTS,S/AS01 vaccine.
Tukwasibwe S(1)(2)(3), Mboowa G(4), Sserwadda I(4), Nankabirwa JI(5), Arinaitwe E(5), Ssewanyana I(5), Taremwa Y(5), Tumusiime G(6), Kamya MR(5)(7), Jagannathan P(8), Nakimuli A(7).
Author information:
(1)Infectious Diseases Research Collaboration, Kampala, Uganda. stephentukwasibwe@yahoo.com.
(2)Infectious Diseases Institute, College of Health Sciences, Makerere University, Kampala, Uganda. stephentukwasibwe@yahoo.com.
(3)School of Medicine, Uganda Christian University, Kampala, Uganda. stephentukwasibwe@yahoo.com.
(4)Infectious Diseases Institute, College of Health Sciences, Makerere University, Kampala, Uganda.
(5)Infectious Diseases Research Collaboration, Kampala, Uganda.
(6)School of Medicine, Uganda Christian University, Kampala, Uganda.
(7)School of Medicine, College of Health Sciences, Makerere University, Kampala, Uganda.
(8)Stanford University, Stanford, USA.
In modern medicine, vaccination is one of the most effective public health strategies to prevent infectious diseases. Indisputably, vaccines have saved millions of lives by reducing the burden of many serious infections such as polio, tuberculosis, measles, pneumonia, and tetanus. Despite the recent recommendation by the World Health Organization (WHO) to roll out RTS,S/AS01, this malaria vaccine still faces major challenges of variability in its efficacy partly due to high genetic variation in humans and malaria parasites. Immune responses to malaria vary between individuals and populations. Human genetic variation in immune system genes is the probable cause for this heterogeneity. In this review, we will focus on human genetic factors that determine variable responses to vaccination and how variation in immune system genes affect the immunogenicity and efficacy of the RTS,S/AS01 vaccine.

145. Int J Infect Dis. 2023 Jun;131:183-192. doi: 10.1016/j.ijid.2023.03.049. Epub
Prevalence of immunoglobulin G and M to SARS-CoV-2 and other human coronaviruses

in The Democratic Republic of Congo, Sierra Leone, and Uganda: A longitudinal study.
Lawal BJ(1), Gallagher KE(2), Kitonsa J(3), Tindanbil D(1), Kasonia K(4), Drammeh A(1), Lowe B(5), Mukadi-Bamuleka D(6), Patterson C(5), Greenwood B(5), Samai M(7), Leigh B(7), Tetteh KKA(5), Ruzagira E(3), Watson-Jones D(8), Kavunga-Membo H(6).
Author information:
(1)LSHTM-COMAHS Research Partnership, Kambia District, Sierra Leone.
(2)London School of Hygiene & Tropical Medicine (LSHTM), London, United Kingdom; KEMRI-Wellcome Trust Research Programme, Kilifi, Kenya. Electronic address: Katherine.gallagher@lshtm.ac.uk.
(3)MRC/UVRI and LSHTM Uganda Research Unit, Entebbe, Uganda.
(4)LSHTM-INRB Research Partnership, Goma, Democratic Republic of the Congo.
(5)London School of Hygiene & Tropical Medicine (LSHTM), London, United Kingdom.
(6)Laboratoire Rodolphe Merieux-Institute National Research biomedical (INRB), Goma, Democratic Republic of the Congo.
(7)University of Sierra Leone College of Medicine and Allied Health Sciences (COMAHS), Freetown, Sierra Leone.
(8)London School of Hygiene & Tropical Medicine (LSHTM), London, United Kingdom; Mwanza Intervention Trials Unit, National Institute for Medical Research, Mwanza, Tanzania.
OBJECTIVES: We assessed the prevalence of immunoglobulin G (IgG) and IgM against four endemic human coronaviruses and two SARS-CoV-2 antigens among vaccinated and unvaccinated staff at health care centers in Uganda, Sierra Leone, and the Democratic Republic of Congo.
METHODS: The government health facility staff who had patient contact in Goma (Democratic Republic of Congo), Kambia District (Sierra Leone), and Masaka District (Uganda) were enrolled. Questionnaires and blood samples were collected at three time points over 4 months. Blood samples were analyzed with the Luminex MAGPIX®.
RESULTS: Among unvaccinated participants, the prevalence of IgG/IgM antibodies against SARS-CoV-2 receptor-binding domain or nucleocapsid protein at enrollment was 70% in Goma (138 of 196), 89% in Kambia (112 of 126), and 89% in Masaka (190 of 213). The IgG responses against endemic human coronaviruses at baseline were not associated with SARS-CoV-2 sero-acquisition during follow-up. Among the vaccinated participants, those who had evidence of SARS-CoV-2 IgG/IgM at baseline tended to have higher IgG responses to vaccination than those who were SARS-CoV-2 seronegative at baseline, controlling for the time of sample collection since vaccination.
CONCLUSION: The high levels of natural immunity and hybrid immunity should be incorporated into both vaccination policies and prediction models of the impact of subsequent waves of infection in these settings.

146. Environ Sci Policy. 2023 Jun;144:110-123. doi: 10.1016/j.envsci.2023.03.008.
Policy responses to COVID-19 in Sri Lanka and the consideration of Indigenous Peoples.
Galappaththi EK(1), Perera CD(1)(2), Dharmasiri IP(1), Ford JD(3), Kodithuwakku

SS(4), Chicmana-Zapata V(5), Zavaleta-Cortijo C(5), Pickering K(6), van Bavel
B(3), Hyams K(7), Arotoma-Rojas I(3), Akugre FA(8), Nkalubo J(9), Namanya
DB(10), Mensah A(8), Hangula MM(11).
Author information:
(1)Department of Geography, Virginia Polytechnic Institute and State University,
Blacksburg, United States.
(2)University of Colombo, Colombo, Sri Lanka.
(3)Priestley International Centre for Climate, University of Leeds, Leeds,
United Kingdom.
(4)Department of Agricultural Economics & Business Management, University of
Peradeniya, Sri Lanka.
(5)Unidad de Ciudadanía Intercultural y Salud Indígena (UCISI), Facultad de
Salud Pública y Administración, Universidad Peruana Cayetano Heredia, Lima,
Peru.
(6)University of the Sunshine Coast, Queensland, Australia.
(7)Department of Politics and International Studies, University of Warwick,
Coventry, UK.
(8)Institute for Environment and Sanitation Studies, University of Ghana, Accra,
Ghana.
(9)Uganda National Health Research Organization & Mulago National Referral
Hospital, Uganda.
(10)Ministry of Health-Uganda National Health Research Organisation, & Uganda
Martyrs, University, Uganda.
(11)Department of Animal Production, Agribusiness and Economics, University of
Namibia, Namibia.
COVID-19 has had uneven impacts on health and well-being, with Indigenous communities in the Global South facing some of the highest risks. Focusing on the experience of Sri Lanka, this study identifies key policy responses to COVID-19, documents how they evolved over two years of the pandemic, and examines if and how government responses have addressed issues pertaining to Indigenous Peoples. Drawing upon an analysis of policy documents (n = 110) and interviews with policymakers (n = 20), we characterize seven key policy responses implemented by the Sri Lankan government: i) testing for and identifying COVID-19; ii) quarantine procedures; iii) provisional clinical treatments; iv) handling other diseases during COVID-19; v) movement; vi) guidelines to be adhered to by the general public; and vii) health and vaccination. The nature of these responses changed as the pandemic progressed. There is no evidence that policy development or implementation incorporated the voices and needs of Indigenous Peoples.

147. Acta Trop. 2023 Jun;242:106902. doi: 10.1016/j.actatropica.2023.106902. Epub
Genetic diversity and genomic analysis of G3P[6] and equine-like G3P[8] in children under-five from Southern Highlands and Eastern Tanzania.
Malakalinga JJ(1), Misinzo G(2), Msalya GM(3), Shayo MJ(4), Kazwala RR(5).
Author information:
(1)Department of Veterinary Medicine and Public Health, College of Veterinary
Medicine and Biomedical Sciences, Sokoine University of Agriculture, P.O. Box

3021, Morogoro, Tanzania; SACIDS Africa Centre of Excellence for Infectious Diseases, SACIDS Foundation for One Health, Sokoine University of Agriculture, P.O. Box 3297, Morogoro, Tanzania; Food and Microbiology Laboratory, Tanzania Bureau of Standards, Ubungo Area, Morogoro Road/Sam Nujoma Road, P.O. Box 9524, Dar es Salaam, Tanzania. Electronic address: joseph.malakalinga@sacids.org.
(2)SACIDS Africa Centre of Excellence for Infectious Diseases, SACIDS Foundation for One Health, Sokoine University of Agriculture, P.O. Box 3297, Morogoro, Tanzania; Department of Veterinary Microbiology, Parasitology and Biotechnology, College of Veterinary Medicine and Biomedical Sciences, Sokoine University of Agriculture, P.O. Box 3019, Morogoro, Tanzania.
(3)Department of Animal, Aquaculture and Range Sciences, College of Agriculture, Sokoine University of Agriculture, P.O. Box 3004, Morogoro, Tanzania.
(4)Department of Biological and Pre-clinical Studies, Muhimbili University of Health and Allied sciences, PO Box 65001, Dar es Salaam, Tanzania.
(5)Department of Veterinary Medicine and Public Health, College of Veterinary Medicine and Biomedical Sciences, Sokoine University of Agriculture, P.O. Box 3021, Morogoro, Tanzania.
Rotavirus group A genomic characterization is critical for understanding the mechanisms of rotavirus diversity, such as reassortment events and possible interspecies transmission. However, little is known about the genetic diversity and genomic relationship of the rotavirus group A strains circulating in Tanzania. The genetic and genomic relationship of RVA genotypes was investigated in children under the age of five. A total of 169 Fecal samples were collected from under-five with diarrhea in Mbeya, Iringa and Morogoro regions of Tanzania. The RVA were screened in children under five with diarrhea using reverse transcription PCR for VP7 and VP4, and the G and P genotypes were determined using Sanger dideoxynucleotide cycle sequencing. Whole-genome sequencing was performed on selected genotypes. The overall RVA rate was 4.7% (8/169). The G genotypes were G3 (7/8) and G6 (1/8) among the 8 RVA positives, while the P genotypes were P[6] (4/8) and P[8] (2), and the other two were untypeable. G3P[6] and G3P[8] were the identified genotype combinations. The genomic analysis reveals that the circulating G3P[8] and G3P[6] isolates from children under the age of five with diarrhea had a DS-1-like genome configuration (I2-R2-C2-M2-axe-N2-T2-E2-H2). The phylogenic analysis revealed that all 11 segments of G3P[6] were closely related to human G3P[6] identified in neighboring countries such as Uganda, Kenya, and other African countries, implying that G3P[6] strains descended from a common ancestor. Whereas, G3P[8] were closely related to previously identified equine-like G3P[P8] from Kenya, Japan, Thailand, Brazil, and Taiwan, implying that this strain was introduced rather than reassortment events. We discovered amino acid differences at antigenic epitopes and N-linked glycosylation sites between the wild type G3 and P[8] compared to vaccine strains, implying that further research into the impact of these differences on vaccine effectiveness is warranted. The phylogenic analysis of VP7 also identified a bovine-like G6. For the first time in Tanzania, we report the emergence of novel equine-like G3 and bovine-like G6 RVA strains, highlighting the importance of rotavirus genotype monitoring and genomic analysis of representative genotypes.

148. One Health. 2023 Jan 13;16:100488. doi: 10.1016/j.onehlt.2023.100488.
Migrant health during public health emergencies: The Ebola crisis in Uganda.
Bellizzi S(1), Pichierri G(2), Popescu C(3).
Author information:
(1)University of Sassari, Sassari, Italy.
(2)Microbiology, Torbay and South Devon NHS Foundation Trust, Torquay, United Kingdom.
(3)University Medical Center Goettingen, Goettingen, Germany.
The Ebola virus diseases (EVD) declared in Uganda in September 22, 2022, has spread to seven districts by early November, with a total of 131 confirmed cases and 48 deaths. Public health emergency response in Uganda deserves a specific and tailored approach due to the current population composition, which accounts to around 1.4 million refugees and asylum seekers. Indeed, Uganda is a potential example of how increased international connectivity has resulted in forced migration with profound impacts on global health. In consideration of the vulnerability of refugees and migrants due to poor living, housing, and working conditions, inclusive policies are even more critical during public health emergencies. Inclusivity lessons learned from COVID-19 in several settings, such as access to treatment and vaccination for all individuals regardless of nationality, residence, and legal status, would be critical to ensure wellbeing of migrants, refugees and host communities.

149. One Health. 2022 Dec 6;16:100470. doi: 10.1016/j.onehlt.2022.100470.
Determining the acceptability of a novel One Health vaccine for Rift Valley Fever prior to phase II/III clinical trials in Uganda.

Bowmer A(1), Ssembatya J(1), Okot M(1), Bagyenyi R(1), Rubanga SV(1), Kalema-Zikusoka G(1).

Author information:
(1)London School of Hygiene and Tropical Medicine | Conservation Through Public Health | Uganda Virus Research Institute, Uganda.

Several vaccine candidates for Rift Valley Fever (RVF) are in development for use in humans. A promising candidate, ChAdOx1 RVF vaccine, has been developed for use in both humans and animals, and has undergone field trials in livestock in Kenya. We conducted a qualitative study to explore the acceptability of this novel One Health vaccine for Rift Valley Fever prior to phase II/III trials, in two rural Ugandan cohorts between January to June 2020. Data was obtained from 96 semi-structured interviews at Bwindi Impenetrable National Park (BINP) and Kyamulibwa, Kalungu District, in Southern Uganda. The study found that 42% of those interviewed were willing to receive a vaccine that was the same for both humans and animals. 45% of those interviewed said that they would not be willing to receive a One Health vaccine and a further 13% were unsure whether or not they would be happy to receive such a vaccine. Semi-structured interviews were conducted to explore their reasons for and against the acceptability of a novel

One Health vaccine to highlight potential barriers to deployment once a vaccine candidate for RVF becomes available.

© 2022 The Authors.

DOI: 10.1016/j.onehlt.2022.100470
PMCID: PMC9747655
PMID: 36531661

Conflict of interest statement: The authors declare that there is no conflict of interest.

150. Malar J. 2023 May 26;22(1):167. doi: 10.1186/s12936-023-04588-1.

Ex vivo RSA and pfkelch13 targeted-amplicon deep sequencing reveal parasites susceptibility to artemisinin in Senegal, 2017.

Yade MS(1)(2), Dièye B(3)(4), Coppée R(5), Mbaye A(6), Diallo MA(3)(4), Diongue K(3)(4)(7), Bailly J(8), Mama A(9), Fall A(3)(4), Thiaw AB(10), Ndiaye IM(3)(4), Ndiaye T(3)(4), Gaye A(3)(4), Tine A(3)(4), Diédhiou Y(3)(4), Mbaye AM(3)(4), Doderer-Lang C(11), Garba MN(3)(4), Bei AK(3)(12)(13), Ménard D(#)(11)(14)(15)(16), Ndiaye D(#)(3)(4)(7).

Author information:
(1)Laboratory of Parasitology-Mycology, Aristide le Dantec Hospital, Université Cheikh Anta Diop, Dakar, Senegal. borombakh66@gmail.com.
(2)International Research Training Center on Genomics and Health Surveillance (CIGASS), Dakar, Senegal. borombakh66@gmail.com.
(3)Laboratory of Parasitology-Mycology, Aristide le Dantec Hospital, Université Cheikh Anta Diop, Dakar, Senegal.
(4)International Research Training Center on Genomics and Health Surveillance (CIGASS), Dakar, Senegal.
(5)Université Paris Cité and Sorbonne Paris Nord, Inserm, IAME, 75018, Paris, France.
(6)Université Gamal Abdel Nasser de Conakry/Centre for Research and Training in Infectiology of Guinea (CERFIG), Conakry, Guinea.
(7)Service of Parasitology-Mycology, Faculty of Medecine, Pharmacy, and Odontostomatology, Cheikh Anta Diop University of Dakar, Dakar, 10700, Senegal.
(8)Université Paris Cité, IRD, MERIT, 75006, Paris, France.
(9)Université de Paris, Institut Cochin, Inserm U1016, Service de Parasitologie Hôpital Cochin, 75014, Paris, France.
(10)Department of Biochemistry and Functional Genomics, Faculty of Medicine and Health Sciences, Université de Sherbrooke, Sherbrooke, Canada.
(11)Université de Strasbourg, Institute of Parasitology and Tropical Diseases, UR7292 Dynamics of Host-Pathogen Interactions, 67000, Strasbourg, France.
(12)Department of Epidemiology of Microbial Diseases, Yale School of Public

Health, New Haven, CT, USA.
(13)Department of Immunology and Infectious Diseases, Harvard TH Chan School of Public Health, Boston, MA, 02115, USA.
(14)CHU Strasbourg, Laboratory of Parasitology and Medical Mycology, 67000, Strasbourg, France.
(15)Institut Pasteur, Université Paris Cité, Malaria Genetics and Resistance Unit, INSERM U1201, 75015, Paris, France.
(16)Institut Pasteur, Université de Paris, Malaria Parasite Biology and Vaccines Unit, Paris, France.
(#)Contributed equally

Update of
Res Sq. 2023 Feb 06;:

BACKGROUND: Malaria control is highly dependent on the effectiveness of artemisinin-based combination therapy (ACT), the current frontline malaria curative treatment. Unfortunately, the emergence and spread of parasites resistant to artemisinin (ART) derivatives in Southeast Asia and South America, and more recently in Rwanda and Uganda (East Africa), compromise their long-term use in sub-Saharan Africa, where most malaria deaths occur.
METHODS: Here, ex vivo susceptibility to dihydroartemisinin (DHA) was evaluated from 38 Plasmodium falciparum isolates collected in 2017 in Thiès (Senegal) expressed in the Ring-stage Survival Assay (RSA). Both major and minor variants were explored in the three conserved-encoding domains of the pfkelch13 gene, the main determinant of ART resistance using a targeted-amplicon deep sequencing (TADS) approach.
RESULTS: All samples tested in the ex vivo RSA were found to be susceptible to DHA (parasite survival rate < 1%). The non-synonymous mutations K189T and K248R in pfkelch13 were observed each in one isolate, as major (99%) or minor (5%) variants, respectively.
CONCLUSION: The results suggest that ART is still fully effective in the Thiès region of Senegal in 2017. Investigations combining ex vivo RSA and TADS are a useful approach for monitoring ART resistance in Africa.

© 2023. The Author(s).

DOI: 10.1186/s12936-023-04588-1
PMCID: PMC10223908
PMID: 37237307 [Indexed for MEDLINE]

Conflict of interest statement: The authors declare no competing interests.

151. BMC Public Health. 2023 May 26;23(1):969. doi: 10.1186/s12889-023-15903-5.

Community dialogue meetings among district leaders improved their willingness to receive COVID-19 vaccines in Western Uganda, May 2021.

Nsubuga EJ(1), Fitzmaurice AG(2), Komakech A(3), Odoi TD(4), Kadobera D(3)(5), Bulage L(3)(5), Kwesiga B(3)(5), Elyanu PJ(4), Ario AR(3)(5), Harris JR(2).

Author information:
(1)Uganda Public Health Fellowship Program, Kampala, Uganda.
nsubugaeddiej@musph.ac.ug.
(2)United States Centers for Disease Control and Prevention, Kampala, Uganda.
(3)Uganda Public Health Fellowship Program, Kampala, Uganda.
(4)Baylor College of Medicine Children's Foundation, Kampala, Uganda.
(5)Uganda National Institute of Public Health, Kampala, Uganda.

BACKGROUND: Widespread COVID-19 vaccine uptake can facilitate epidemic control. A February 2021 study in Uganda suggested that public vaccine uptake would follow uptake among leaders. In May 2021, Baylor Uganda led community dialogue meetings with district leaders from Western Uganda to promote vaccine uptake. We assessed the effect of these meetings on the leaders' COVID-19 risk perception, vaccine concerns, perception of vaccine benefits and access, and willingness to receive COVID-19 vaccine.
METHODS: All departmental district leaders in the 17 districts in Western Uganda, were invited to the meetings, which lasted approximately four hours. Printed reference materials about COVID-19 and COVID-19 vaccines were provided to attendees at the start of the meetings. The same topics were discussed in all meetings. Before and after the meetings, leaders completed self-administered questionnaires with questions on a five-point Likert Scale about risk perception, vaccine concerns, perceived vaccine benefits, vaccine access, and willingness to receive the vaccine. We analyzed the findings using Wilcoxon's signed-rank test.
RESULTS: Among 268 attendees, 164 (61%) completed the pre- and post-meeting questionnaires, 56 (21%) declined to complete the questionnaires due to time constraints and 48 (18%) were already vaccinated. Among the 164, the median COVID-19 risk perception scores changed from 3 (neutral) pre-meeting to 5 (strong agreement with being at high risk) post-meeting ($p < 0.001$). Vaccine concern scores reduced, with medians changing from 4 (worried about vaccine side effects) pre-meeting to 2 (not worried) post-meeting ($p < 0.001$). Median scores regarding perceived COVID-19 vaccine benefits changed from 3 (neutral) pre-meeting to 5 (very beneficial) post-meeting ($p < 0.001$). The median scores for perceived vaccine access increased from 3 (neutral) pre-meeting to 5 (very accessible) post-meeting ($p < 0.001$). The median scores for willingness to receive the vaccine changed from 3 (neutral) pre-meeting to 5 (strong willingness) post-meeting ($p < 0.001$).
CONCLUSION: COVID-19 dialogue meetings led to district leaders' increased risk perception, reduced concerns, and improvement in perceived vaccine benefits, vaccine access, and willingness to receive the COVID-19 vaccine. These could potentially influence public vaccine uptake if leaders are vaccinated publicly as a result. Broader use of such meetings with leaders could increase vaccine uptake among themselves and the community.

© 2023. The Author(s).

DOI: 10.1186/s12889-023-15903-5
PMCID: PMC10213578
PMID: 37237258 [Indexed for MEDLINE]

Conflict of interest statement: The authors declare no competing interests.

152. Virus Evol. 2023 May 22;9(1):vead034. doi: 10.1093/ve/vead034. eCollection 2023.

Variation within major internal repeats of KSHV in vivo.

Santiago JC(1), Westfall DH(1), Adams SV(2), Okuku F(3), Phipps W(2)(4), Mullins JI(1)(4)(5).

Author information:
(1)Department of Microbiology, University of Washington, 960 Republican St, Seattle, WA 98109-4325, USA.
(2)Global Oncology and Vaccine and Infectious Diseases Division,Fred Hutchinson Cancer Center, 1100 Eastlake Ave, Seattle, 98109-4487 WA, USA.
(3)Uganda Cancer Institute, Upper Mulago Hill Road, Kampala, Uganda.
(4)Department of Medicine, University of Washington, 1959 NE Pacific St., Seattle, WA 98195-8070, USA.
(5)Department of Global Health, University of Washington, 3980 15th Ave NE, Seattle, WA 98195, USA.

Kaposi's sarcoma-associated herpesvirus (KSHV) is the etiologic agent of Kaposi's sarcoma (KS), yet the viral genetic factors that lead to the development of KS in KSHV-infected individuals have not been fully elucidated. Nearly, all previous analyses of KSHV genomic evolution and diversity have excluded the three major internal repeat regions: the two origins of lytic replication, internal repeats 1 and 2 (IR1 and IR2), and the latency-associated nuclear antigen (LANA) repeat domain (LANAr). These regions encode protein domains that are essential to the KSHV infection cycle but have been rarely sequenced due to their extended repetitive nature and high guanine and cytosine (GC) content. The limited data available suggest that their sequences and repeat lengths are more heterogeneous across individuals than in the remainder of the KSHV genome. To assess their diversity, the full-length IR1, IR2, and LANAr sequences, tagged with unique molecular identifiers (UMIs), were obtained by Pacific Biosciences' single-molecule real-time sequencing (SMRT-UMI) from twenty-four tumors and six matching oral swabs from sixteen adults in Uganda with advanced KS. Intra-host single-nucleotide variation involved an average of 0.16 per cent of base positions in the repeat regions compared to a nearly identical average of 0.17 per cent of base positions in the remainder of the genome. Tandem repeat unit (TRU) counts varied by only one from the intra-host

consensus in a majority of individuals. Including the TRU indels, the average intra-host pairwise identity was 98.3 per cent for IR1, 99.6 per cent for IR2 and 98.9 per cent for LANAr. More individuals had mismatches and variable TRU counts in IR1 (twelve/sixteen) than in IR2 (two/sixteen). There were no open reading frames in the Kaposin coding sequence inside IR2 in at least fifty-five of ninety-six sequences. In summary, the KSHV major internal repeats, like the rest of the genome in individuals with KS, have low diversity. IR1 was the most variable among the repeats, and no intact Kaposin reading frames were present in IR2 of the majority of genomes sampled.

© The Author(s) 2023. Published by Oxford University Press.

DOI: 10.1093/ve/vead034
PMCID: PMC10266750
PMID: 37325087

Conflict of interest statement: None declared.

153. PLoS Med. 2023 May 22;20(5):e1004239. doi: 10.1371/journal.pmed.1004239. eCollection 2023 May.

Global, regional, and national estimates of the impact of a maternal Klebsiella pneumoniae vaccine: A Bayesian modeling analysis.

Kumar CK(1), Sands K(2), Walsh TR(2), O'Brien S(3), Sharland M(4), Lewnard JA(5), Hu H(6), Srikantiah P(6), Laxminarayan R(1)(7).

Author information:
(1)Princeton University, Princeton, New Jersey, United States of America.
(2)Ineos Oxford Institute for Antimicrobial Resistance, Department of Zoology, Oxford, United Kingdom.
(3)Global Antibiotic Research and Development Partnership, Geneva, Switzerland.
(4)Center for Neonatal and Paediatric Infection (CNPI), Institute of Infection and Immunity, St George's University of London, London, United Kingdom.
(5)Division of Epidemiology, School of Public Health, University of California at Berkeley, Berkeley, California, United States of America.
(6)Bill & Melinda Gates Foundation, Seattle, Washington, United States of America.
(7)One Health Trust, Bengaluru, India.

BACKGROUND: Despite significant global progress in reducing neonatal mortality, bacterial sepsis remains a major cause of neonatal deaths. Klebsiella pneumoniae (K. pneumoniae) is the leading pathogen globally underlying cases of neonatal sepsis and is frequently resistant to antibiotic treatment regimens recommended by the World Health Organization (WHO), including first-line therapy with ampicillin and gentamicin, second-line therapy with amikacin and ceftazidime,

and meropenem. Maternal vaccination to prevent neonatal infection could reduce the burden of K. pneumoniae neonatal sepsis in low- and middle-income countries (LMICs), but the potential impact of vaccination remains poorly quantified. We estimated the potential impact of such vaccination on cases and deaths of K. pneumoniae neonatal sepsis and project the global effects of routine immunization of pregnant women with the K. pneumoniae vaccine as antimicrobial resistance (AMR) increases.
METHODS AND FINDINGS: We developed a Bayesian mixture-modeling framework to estimate the effects of a hypothetical K. pneumoniae maternal vaccine with 70% efficacy administered with coverage equivalent to that of the maternal tetanus vaccine on neonatal sepsis infections and mortality. To parameterize our model, we used data from 3 global studies of neonatal sepsis and/or mortality-with 2,330 neonates who died with sepsis surveilled from 2016 to 2020 undertaken in 18 mainly LMICs across all WHO regions (Ethiopia, Kenya, Mali, Mozambique, Nigeria, Rwanda, Sierra Leone, South Africa, Uganda, Brazil, Italy, Greece, Pakistan, Bangladesh, India, Thailand, China, and Vietnam). Within these studies, 26.95% of fatal neonatal sepsis cases were culture-positive for K. pneumoniae. We analyzed 9,070 K. pneumoniae genomes from human isolates gathered globally from 2001 to 2020 to quantify the temporal rate of acquisition of AMR genes in K. pneumoniae isolates to predict the future number of drug-resistant cases and deaths that could be averted by vaccination. Resistance rates to carbapenems are increasing most rapidly and 22.43% [95th percentile Bayesian credible interval (CrI): 5.24 to 41.42] of neonatal sepsis deaths are caused by meropenem-resistant K. pneumoniae. Globally, we estimate that maternal vaccination could avert 80,258 [CrI: 18,084 to 189,040] neonatal deaths and 399,015 [CrI: 334,523 to 485,442] neonatal sepsis cases yearly worldwide, accounting for more than 3.40% [CrI: 0.75 to 8.01] of all neonatal deaths. The largest relative benefits are in Africa (Sierra Leone, Mali, Niger) and South-East Asia (Bangladesh) where vaccination could avert over 6% of all neonatal deaths. Nevertheless, our modeling only considers country-level trends in K. pneumoniae neonatal sepsis deaths and is unable to consider within-country variability in bacterial prevalence that may impact the projected burden of sepsis.
CONCLUSIONS: A K. pneumoniae maternal vaccine could have widespread, sustained global benefits as AMR in K. pneumoniae continues to increase.

Copyright: © 2023 Kumar et al. This is an open access article distributed under the terms of the Creative Commons Attribution License, which permits unrestricted use, distribution, and reproduction in any medium, provided the original author and source are credited.

DOI: 10.1371/journal.pmed.1004239
PMCID: PMC10270628
PMID: 37216371 [Indexed for MEDLINE]

Conflict of interest statement: We have read the journal's policy and the authors of this manuscript have the following competing interests: two study

authors (PS and HH) are employed by an organization that funded the study. They provided input in the writing of the report, acting in a purely scientific capacity. In particular, PS provided critical expertise on K. pneumoniae epidemiology and vaccine development. HH provided valuable insights into modeling bacterial pathogens.

154. Vacunas. 2023 May 18. doi: 10.1016/j.vacun.2023.05.003. Online ahead of print.

Adverse events following COVID-19 vaccination among pregnant women attending primary health centers: An active-surveillance study.

Goruntla N(1), Karisetty B(2), Nandini N(2), Bhupasamudram B(2), Gangireddy HR(2), Veerabhadrappa KV(3), Ezeonwumelu JOC(1), Bandaru V(4).

Author information:
(1)Department of Clinical Pharmacy and Pharmacy Practice, School of Pharmacy, Kampala International University, Western Campus, Uganda.
(2)Department of Pharmacy Practice, Raghavendra Institute of Pharmaceutical Education and Research (RIPER) - Autonomous, Anantapur, Andhra Pradesh, India.
(3)Department of Pharmacognosy and Pharmaceutical Chemistry, School of Pharmacy, Kampala International University, Western Campus, Uganda.
(4)Department of Pharmaceutical Analysis, Balaji College of Pharmacy, Anantapur, Andhra Pradesh, India.

INTRODUCTION: Vaccine safety is a major barrier to the uptake of the COVID-19 vaccine by pregnant women. To bring confidence among pregnant women towards vaccine intake, there is a need to synthesize evidence on safety profile of vaccination.
OBJECTIVE: To assess adverse events (AEs) following COVID-19 vaccination among pregnant women.
MATERIALS AND METHODS: A vaccine safety surveillance was conducted at 2 rural primary health centers (PHC) located in Anantapur District, India. A total of 420 pregnant women were monitored for AEs following COVID-19 vaccination for a period of 30 min and followed for 1 month for late reactions through telephonic interviews. All AEs were subjected to causality and severity assessment. Descriptive statistics were used to represent adverse events.
RESULTS: The COVID-19 vaccine acceptance rate among pregnant women was 64.4%. A total of 420 pregnant women received 670 vaccine doses (Covishield = 372, Covaxin = 298) against COVID-19. Majority of vaccine intake was observed during the second trimester. The incidence rate of AEs following the COVID-19 vaccine among pregnant women was 93.8%, and the majority include injection site pain (28.4%, 29.6%), fever (25.5%, 19.0%), myalgia (8.21%, 12.3%), and malaise (13.6%, 8.4%). Most AEs notified are probable and mild in nature.
CONCLUSION: The COVID-19 vaccine acceptance rate among pregnant women was 64.4%. A 30 days incidence rate of AEs following COVID-19 vaccination among pregnant women was 93.8%, with the most common mild events like injection site pain, and

fever. A further follow-up cohort study by taking an adequate sample size was recommended to capture fetal-maternal outcomes.

Publisher: INTRODUCCIÓN: La seguridad de la vacuna es una barrera importante para la adopción de la vacuna COVID-19 por parte de las mujeres embarazadas. Para llevar confianza entre las mujeres embarazadas hacia la ingesta de vacunas, es necesario sintetizar la evidencia sobre el perfil de seguridad de la vacunación.
OBJETIVO: Evaluar los eventos adversos (EA) después de la vacunación contra la COVID-19 en mujeres embarazadas.
MATERIALES Y MÉTODOS: Se llevó a cabo una vigilancia de la seguridad de las vacunas en dos centros rurales de atención primaria de salud (PHC) ubicados en el distrito de Anantapur, India. Un total de 420 mujeres embarazadas fueron monitoreadas para detectar EA después de la vacunación COVID-19 durante un período de 30 minutos y seguidas durante un mes para detectar reacciones tardías a través de entrevistas telefónicas. Todos los EA se sometieron a una evaluación de causalidad y gravedad. Se utilizaron estadísticas descriptivas para representar los eventos adversos.
RESULTADOS: La tasa de aceptación de la vacuna COVID-19 entre las mujeres embarazadas fue del 64,4%. Un total de 420 mujeres embarazadas recibieron 670 dosis de vacunas (Covishield = 372, Covaxin = 298) contra COVID-19. La mayoría de la ingesta de vacunas se observó durante el segundo trimestre. La tasa de incidencia de EA después de la vacuna COVID-19 entre las mujeres embarazadas fue del 93,8%, y la mayoría incluye dolor en el lugar de la inyección (28,4%, 29,6%), fiebre (25,5%, 19,0%), mialgia (8,21%, 12,3%) y malestar general (13,6%, 8,4%). La mayoría de los EA notificados son de naturaleza probable y leve.
CONCLUSIÓN: La tasa de aceptación de la vacuna COVID-19 entre las mujeres embarazadas fue del 64,4%. Una tasa de incidencia de EA a 30 días después de la vacunación contra COVID-19 entre las mujeres embarazadas fue del 93,8%, con los eventos leves más comunes como dolor en el lugar de la inyección y fiebre. Se recomendó un estudio de cohorte de seguimiento adicional mediante la toma de un tamaño de muestra adecuado para capturar los resultados maternos fetales.

© 2023 Elsevier España, S.L.U. All rights reserved.

DOI: 10.1016/j.vacun.2023.05.003
PMCID: PMC10192595
PMID: 37362835

Conflict of interest statement: The authors declare that they have no conflicts of interest.

155. Antibiotics (Basel). 2023 May 17;12(5):923. doi: 10.3390/antibiotics12050923.

Challenges in the Implementation of the NeoOBS Study, a Global Pragmatic Observational Cohort Study, to Investigate the Aetiology and Management of

Neonatal Sepsis in the Hospital Setting.

Riddell A(1), Cook A(1), Khavessian N(2), Ellis S(2), Bilardi D(3), Correia E(2), Kostyanev T(4), Nardone A(3), Russell N(1), Vilken T(4), Stohr W(5), Adhisivam B(6), Moraes IRA(7), Ahmed NU(8), Bekker A(9), Berezin EN(7), Boonkasidecha S(10), Carvalheiro CG(11), Chauhan P(12), Chiurchiù S(13), Chorafa E(14), Dramowski A(9), Ds M(15), Feng J(16), Jia S(17), Kong Y(18), Kyohere M(19), Kontou A(20), Lochindarat S(10), De Luca M(13), Mphaphuli A(21), Mussi-Pinhata MM(11), Murunga S(22), Nakwa FL(23), Nangia S(12), Nassolo E(19), Hoang NTB(24), Obiero CW(22)(25), Olson L(26), Ping W(27)(28), Plakkal N(6), Prasad P(15), Preedisripipat K(29), Rahman SW(8), Seef T(21), Sukrakanchana PO(30), Thomas R(23), Yu Z(27)(28), Zhang Q(17), Walker AS(5), Bielicki J(1), Heath PT(1), Sharland M(1), Munera-Huertas T(1).

Author information:
(1)Centre for Neonatal and Paediatric Infection, St. George's University of London, London SW17 0RE, UK.
(2)GARDP, 1202 Geneva, Switzerland.
(3)Fondazione Penta ETS, 35127 Padova, Italy.
(4)Laboratory of Medical Microbiology, Vaccine & Infectious Disease Institute, University of Antwerp, 2610 Antwerpen, Belgium.
(5)MRC Clinical Trials Unit, University College London, London WC1V 6LJ, UK.
(6)Department of Neonatology, Jawaharlal Institute of Postgraduate Medical Education & Research (JIPMER), Pondicherry 605006, India.
(7)Santa Casa de São Paulo, Sao Paulo 01221-010, Brazil.
(8)Child Health Research Foundation, Dhaka 1207, Bangladesh.
(9)Department of Paediatrics and Child Health, Faculty of Medicine and Health Sciences, Stellenbosch University, Cape Town 8000, South Africa.
(10)Queen Sirikit National Institute of Child Health, Bangkok 10400, Thailand.
(11)Department of Pediatrics, Ribeirão Preto Medical School, University of São Paulo, Ribeirão Preto 14049-900, Brazil.
(12)Department of Neonatology, Lady Hardinge Medical College and Kalawati Saran Children's Hospital, New Delhi 110001, India.
(13)Academic Department of Pediatrics (DPUO), Infectious Disease Unit, Bambino Gesù Children's Hospital, IRCCS, 00165 Rome, Italy.
(14)Infectious Diseases Unit, 3rd Dept Pediatrics, School of Medicine, Faculty of Health Sciences, Aristotle University and Hippokration General Hospital, 546 43 Thessaloniki, Greece.
(15)Seth G. S. Medical College & KEM Hospital, Mumbai 400012, India.
(16)Department of Neonatology, Shenzhen Children's Hospital, Shenzhen 518048, China.
(17)Department of Neonatology, Beijing Children's Hospital, Capital Medical University, National Center for Children's Health, Beijing 100051, China.
(18)Clinical Laboratory, Shenzhen Children's Hospital, Shenzhen 518048, China.
(19)MUJHU Research Collaboration, Kampala P.O. Box 23491, Uganda.
(20)1st Neonatology Department and Neonatal Intensive Care Unit, School of Medicine, Faculty of Health Sciences, Aristotle University of Thessaloniki,

Hippokration General Hospital, 546 42 Thessaloniki, Greece.
(21)School of Clinical Medicine, Faculty of Health Sciences, University of Witwatersrand, Johannesburg, South Africa AND Department of Paediatrics and Child Health, Charlotte Maxeke Johannesburg Academic Hospital, Johannesburg 2193, South Africa.
(22)Clinical Research Department, KEMRI-Wellcome Trust Research Programme, Kilifi P.O. Box 230, Kenya.
(23)Department of Paediatrics and Child Health, School of Clinical Medicine, Faculty of Health Sciences, University of the Witwatersrand, Johannesburg 1864, South Africa.
(24)Department of Microbiology, Vietnam National Children's Hospital, Hanoi 100000, Vietnam.
(25)Amsterdam UMC, University of Amsterdam, Emma Children's Hospital, Department of Global Health, 1081 Amsterdam, The Netherlands.
(26)Department of Women's and Children's Health, Karolinska institutet, Stockholm, Sweden and Department of Global Public Health, Karolinska Institutet, 171 77 Stockholm, Sweden.
(27)Beijing Obstetrics and Gynecology Hospital, Capital Medical University, Beijing 100026, China.
(28)Beijing Maternal and Child Health Care Hospital, Beijing 100026, China.
(29)Chiangrai Prachanukroh Hospital, Chiang Rai 57000, Thailand.
(30)AMS-PHPT Research Collaboration, Chiang Mai University, Chiang Mai 50200, Thailand.

Neonatal sepsis is a significant cause of mortality and morbidity in low- and middle-income countries. To deliver high-quality data studies and inform future trials, it is crucial to understand the challenges encountered when managing global multi-centre research studies and to identify solutions that can feasibly be implemented in these settings. This paper provides an overview of the complexities faced by diverse research teams in different countries and regions, together with actions implemented to achieve pragmatic study management of a large multi-centre observational study of neonatal sepsis. We discuss specific considerations for enrolling sites with different approval processes and varied research experience, structures, and training. Implementing a flexible recruitment strategy and providing ongoing training were necessary to overcome these challenges. We emphasize the attention that must be given to designing the database and monitoring plans. Extensive data collection tools, complex databases, tight timelines, and stringent monitoring arrangements can be problematic and might put the study at risk. Finally, we discuss the complexities added when collecting and shipping isolates and the importance of having a robust central management team and interdisciplinary collaborators able to adapt easily and make swift decisions to deliver the study on time and to target. With pragmatic approaches, appropriate training, and good communication, these challenges can be overcome to deliver high-quality data from a complex study in challenging settings through a collaborative research network.

DOI: 10.3390/antibiotics12050923

PMCID: PMC10215156
PMID: 37237826

Conflict of interest statement: The authors declare no conflict of interest.
GARDP as funder and sponsor of the study was involved in the design of the study and study monitoring.

156. Health Serv Res Manag Epidemiol. 2023 May 16;10:23333928231175798. doi: 10.1177/23333928231175798. eCollection 2023 Jan-Dec.

Predictors of Parents' Willingness to Vaccinate Their Children Against COVID-19 in India: A Web-Based Cross-Sectional Survey.

Goruntla N(1), Ayisha MU(2), Sreeram M(2).

Author information:
(1)Department of Clinical Pharmacy and Pharmacy Practice, School of Pharmacy, Kampala International University, Western Campus, Ishaka, Bushenyi, Uganda.
(2)Department of Pharmacy Practice, Raghavendra Institute of Pharmaceutical Education and Research (RIPER) (Autonomous), Anantapur, India.

BACKGROUND: Parents are more concerned about the available evidence of the safety, efficacy, and tolerability of the pediatric COVID-19 vaccine.
AIM: To assess the parents' willingness to vaccinate their children against COVID-19 and associate it with health belief model constructs.
MATERIALS AND METHODS: A countrywide, online, self-administered, cross-sectional survey was conducted from December 15, 2021 to March 8, 2022. The HBM approach was used as a theoretical context to assess the predictors of parents' willingness to vaccinate their children against COVID-19.
RESULTS: The majority (1,563; 95.4%) of parents are intended to immunize their children against COVID-19. Parent education, financial status, job, number of children, age-related vaccination status of the child, and household suffering from chronic disorders were significantly associated with a parent's willingness to recommend the COVID-19 vaccine for their children. The findings of HBM constructs indicated that perceived benefits (OR 14.222; 95% CI 7.192-28.124) of the COVID-19 vaccine in children, susceptibility (OR 7.758; 95% CI 3.508-17.155) of children toward COVID-19, and severity (OR 3.820; 95% CI 2.092-6.977) of COVID-19 infection in children were significantly associated with parent acceptance to vaccinate their children. Parents' higher perception of barriers (OR 0.609; 95% CI 0.372-0.999) to vaccination reduces the intention to vaccinate children against COVID-19.
CONCLUSION: The findings of our study reveal that the value of HBM constructs in the identification of predictors associated with the parents' willingness to encourage COVID-19 vaccine for their children. It is important to improve health and reduce the barriers to COVID-19 vaccination among Indian parents having children less than 18 years.

© The Author(s) 2023.

DOI: 10.1177/23333928231175798
PMCID: PMC10192664
PMID: 37213221

Conflict of interest statement: The author(s) declared no potential conflicts of interest with respect to the research, authorship, and/or publication of this article.

157. Am J Respir Crit Care Med. 2023 May 15;207(10):1376-1382. doi: 10.1164/rccm.202206-1118OC.

A Standardized Approach for Collection of Objective Data to Support Outcome Determination for Late-Phase Tuberculosis Clinical Trials.

Kurbatova EV(1), Phillips PPJ(2), Dorman SE(3), Sizemore EE(1), Bryant KE(1), Purfield AE(1)(4), Ricaldi J(1), Brown NE(1), Johnson JL(5)(6), Wallis CL(7), Akol JP(6), Ocheretina O(8), Van Hung N(9), Mayanja-Kizza H(6), Lourens M(10), Dawson R(11), Nhung NV(9), Pierre S(8), Musodza Y(12), Shenje J(13), Badal-Faesen S(14), Vilbrun SC(8), Waja Z(15), Peddareddy L(1), Scott NA(1), Yuan Y(1), Goldberg SV(1), Swindells S(16), Chaisson RE(17), Nahid P(2).

Author information:
(1)U.S. Centers for Disease Control and Prevention, Atlanta, Georgia.
(2)UCSF Center for Tuberculosis, University of California San Francisco, San Francisco, California.
(3)Medical University of South Carolina, Charleston, South Carolina.
(4)United States Public Health Service Commissioned Corps, Rockville, Maryland.
(5)Case Western Reserve University, University Hospitals Cleveland Medical Center, Cleveland, Ohio.
(6)Uganda-Case Western Reserve University Research Collaboration, Kampala, Uganda.
(7)Lancet Laboratories and Bio Analytical Research Corporation South Africa (BARC SA), Johannesburg, South Africa.
(8)GHESKIO, Port-au-Prince, Haiti.
(9)Vietnam National Tuberculosis Program/National Lung Hospital, Hanoi, Vietnam.
(10)TASK, Cape Town, South Africa.
(11)Division of Pulmonology, Department of Medicine, University of Cape Town and University of Cape Town Lung Institute, Cape Town, South Africa.
(12)University of Zimbabwe College of Health Sciences, Harare, Zimbabwe.
(13)South African Tuberculosis Vaccine Initiative, Cape Town, South Africa.
(14)Clinical HIV Research Unit, University of the Witwatersrand, Johannesburg, South Africa.
(15)Perinatal HIV Research Unit, University of the Witwatersrand, Johannesburg,

South Africa.
(16)University of Nebraska Medical Center, Omaha, Nebraska; and.
(17)Johns Hopkins University School of Medicine, Baltimore, Maryland.

Comment in
   Am J Respir Crit Care Med. 2023 May 15;207(10):1269-1270.

Rationale: We developed a standardized method, possible poor treatment response (PPTR), to help ascertain efficacy endpoints in Study S31/A5349 (NCT02410772), an open-label trial comparing two 4-month rifapentine-based regimens with a standard 6-month regimen for the treatment of pulmonary tuberculosis (TB). Objectives: We describe the use of the PPTR process and evaluate whether the goals of minimizing bias in efficacy endpoint assessment and attainment of relevant data to determine outcomes for all participants were achieved. Methods: A PPTR event was defined as the occurrence of one or more prespecified triggers. Each PPTR required initiation of a standardized evaluation process that included obtaining multiple sputum samples for microbiology. Measurements and Main Results: Among 2,343 participants with culture-confirmed drug-susceptible TB, 454 individuals (19.4%) had a total of 534 individual PPTR events, of which 76.6% were microbiological (positive smear or culture at or after 17 wk). At least one PPTR event was experienced by 92.4% (133 of 144) of participants with TB-related unfavorable outcome and between 13.8% and 14.7% of participants with favorable and not-assessable outcomes. A total of 75% of participants with TB-related unfavorable outcomes had microbiological confirmation of failure to achieve a disease-free cure. Conclusions: Standardized methodologies, such as our PPTR approach, could facilitate unbiased efficacy outcome determinations, improve discrimination between outcomes that are related and unrelated to regimen efficacy, and enhance the ability to conduct pooled analyses of contemporary trials.

DOI: 10.1164/rccm.202206-1118OC
PMCID: PMC10595436
PMID: 36790881 [Indexed for MEDLINE]

158. Am J Trop Med Hyg. 2023 Apr 10;108(5):954-962. doi: 10.4269/ajtmh.23-0007. Print 2023 May 3.

Development of a Scorecard to Monitor Progress toward National Cholera Elimination: Its Application in Uganda.

Bwire G(1), Sack DA(2), Lunkuse SM(3), Ongole F(4), Ngwa MC(2), Namanya DB(5), Nsungwa J(6), Aceng Ocero JR(7), Mwebesa HG(8), Muruta A(9), Nakinsige A(10), Kisakye A(11), Kalyebi P(5), Kemirembe J(12), Makumbi I(13), Kagirita A(3), Ampeire I(14), Mutegeki D(13), Matseketse D(15), Debes AK(2), Orach CG(16).

Author information:

(1)Department of Community Health, Ministry of Health Uganda, Kampala, Uganda.
(2)Department of International Health, Johns Hopkins Bloomberg School of Public Health, Baltimore, Maryland.
(3)Division of Surveillance, Knowledge and Information Management, Ministry of Health, Kampala, Uganda.
(4)Department of National Health Laboratory and Diagnostic Services, Ministry of Health, Kampala, Uganda.
(5)Department of Environmental Health, Ministry of Health, Kampala, Uganda.
(6)Department of Maternal and Child Health, Ministry of Health, Kampala, Uganda.
(7)Office of the Minister, Ministry of Health, Kampala, Uganda.
(8)Office of the Director General Health Service, Ministry of Health, Kampala, Uganda.
(9)Department of Integrated Epidemiology and Public Health Emergencies, Ministry of Health, Kampala, Uganda.
(10)Division of Public Health Emergency Preparedness and Response, Ministry of Health, Kampala, Uganda.
(11)World Health Organization, Kampala, Uganda.
(12)Masaka Regional Referral Hospital, Masaka City, Uganda.
(13)Public Health Emergency Operation Centre, Ministry of Health, Kampala, Uganda.
(14)Uganda National Immunization Programme, Ministry of Health, Kampala, Uganda.
(15)UNICEF, Kampala, Uganda.
(16)College of Health Sciences, Makerere University School of Public Health, Kampala, Uganda.

In 2017, the Global Task Force for Cholera Control (GTFCC) set a goal to eliminate cholera from ≥ 20 countries and to reduce cholera deaths by 90% by 2030. Many countries have included oral cholera vaccine (OCV) in their cholera control plans. We felt that a simple, user-friendly monitoring tool would be useful to guide national progress toward cholera elimination. We reviewed cholera surveillance data of Uganda from 2015 to 2021 by date and district. We defined a district as having eliminated cholera if cholera was not reported in that district for at least 4 years. We prepared maps to show districts with cholera, districts that had eliminated it, and districts that had eliminated it but then "relapsed." These maps were compared with districts where OCV was used and the hotspot map recommended by the GTFCC. Between 2018 and 2021, OCV was administered in 16 districts previously identified as hotspots. In 2018, cholera was reported during at least one of the four previous years from 36 of the 146 districts of Uganda. This number decreased to 18 districts by 2021. Cholera was deemed "eliminated" from four of these 18 districts but then "relapsed." The cholera elimination scorecard effectively demonstrated national progress toward cholera elimination and identified districts where additional resources are needed to achieve elimination by 2030. Identification of the districts that have eliminated cholera and those that have relapsed will assist the national programs to focus on addressing the factors that result in elimination or relapse of cholera.

DOI: 10.4269/ajtmh.23-0007
PMCID: PMC10160876
PMID: 37037429 [Indexed for MEDLINE]

159. Am J Trop Med Hyg. 2023 Mar 13;108(5):995-1002. doi: 10.4269/ajtmh.22-0410. Print 2023 May 3.

Detection of Sporadic Outbreaks of Rift Valley Fever in Uganda through the National Viral Hemorrhagic Fever Surveillance System, 2017-2020.

Nyakarahuka L(1)(2), Whitmer S(3), Klena J(3), Balinandi S(1), Talundzic E(3), Tumusiime A(1), Kyondo J(1), Mulei S(1), Patel K(3), Baluku J(1), Akurut G(4), Namanya D(4), Kamugisha K(4), Cossaboom C(3), Whitesell A(3), Telford C(3), Graziano J(3), Montgomery J(3), Nichol S(3), Lutwama J(1), Shoemaker T(3).

Author information:
(1)Department of Arbovirology, Emerging and Reemerging Infectious Diseases, Uganda Virus Research Institute, Entebbe, Uganda.
(2)Department of Biosecurity, Ecosystems and Veterinary Public Health, Makerere University, Kampala, Uganda.
(3)Viral Special Pathogens Branch, Division of High-Consequence Pathogens and Pathology, U.S. Centers for Disease Control and Prevention, Atlanta, Georgia.
(4)Uganda Wildlife Authority, Kampala, Uganda.

Rift Valley fever (RVF) is a zoonotic disease of public health and economic importance. Uganda has reported sporadic outbreaks of RVF in both humans and animals across the country, especially in the southwestern part of the "cattle corridor" through an established viral hemorrhagic fever surveillance system. We report 52 human cases of laboratory-confirmed RVF from 2017 to 2020. The case fatality rate was 42%. Among those infected, 92% were males and 90% were adults (≥ 18 years). Clinical symptoms were characterized by fever (69%), unexplained bleeding (69%), headache (51%), abdominal pain (49%), and nausea and vomiting (46%). Most of the cases (95%) originated from central and western districts that are part of the cattle corridor of Uganda, where the main risk factor was direct contact with livestock ($P = 0.009$). Other predictors of RVF positivity were determined to be male gender ($P = 0.001$) and being a butcher ($P = 0.04$). Next-generation sequencing identified the predominant Ugandan clade as Kenya-2, observed previously across East Africa. There is need for further investigation and research into the effect and spread of this neglected tropical disease in Uganda and the rest of Africa. Control measures such as promoting vaccination and limiting animal-human transmission could be explored to reduce the impact of RVF in Uganda and globally.

DOI: 10.4269/ajtmh.22-0410
PMCID: PMC10160879
PMID: 36913925 [Indexed for MEDLINE]

160. Clin Infect Dis. 2023 May 3;76(9):1585-1593. doi: 10.1093/cid/ciac944.

The Impact of Sequestration on Artemisinin-Induced Parasite Clearance in Plasmodium falciparum Malaria in Africa.

Fukuda N(1), Balikagala B(1), Ueno T(2), Anywar DA(3), Kimura E(4), Palacpac NMQ(5), Odongo-Aginya EI(3), Ogwang M(6), Horii T(5), Miida T(2), Mita T(1).

Author information:
(1)Department of Tropical Medicine and Parasitology, Faculty of Medicine, Juntendo University, Tokyo, Japan.
(2)Department of Clinical Laboratory Medicine, Faculty of Medicine, Juntendo University, Tokyo, Japan.
(3)Faculty of Medicine, Gulu University, Gulu, Uganda.
(4)School of Tropical Medicine and Global Health, Nagasaki University, Nagasaki, Japan.
(5)Department of Malaria Vaccine Development, Research Institute for Microbial Diseases, Osaka University, Osaka, Japan.
(6)St. Mary's Hospital Lacor, Gulu, Uganda.

BACKGROUND: Artemisinin-resistant Plasmodium falciparum is spreading in Southeast Asia and Africa. In vivo susceptibility to artemisinin is studied by looking at the rate of decline of peripheral parasitemia (parasite clearance half-life). However, parasites that are adhered/sequestered to the endothelium and undetectable in the peripheral blood are not considered in the estimation of parasite clearance. Here, we evaluated the influence of sequestration on in vivo artemisinin efficacy in Uganda, where artemisinin resistance is spreading.
METHODS: We analyzed 133 patients with P. falciparum malaria included in an in vivo study on artemisinin efficacy in northern Uganda in 2018 and 2019. The parasite clearance half-life was estimated from peripheral parasitemia after artemisinin monotherapy. P. falciparum histidine-rich protein 2 (PfHRP2) was measured in pretreatment plasma. The number of sequestered parasites was estimated from PfHRP2 concentration and peripheral parasitemia.
RESULTS: The estimated number of sequestered parasites per plasma volume ranged from 0 to 2 564 000/µL. Inflammation, thrombocytopenia, and dyslipidemia were significantly associated with sequestration independent of peripheral parasitemia. The median parasite clearance half-lives were 1.65 hours in patients infected with Pfkelch13 wild-type parasites (n = 104) and 3.95 hours in those with A675V artemisinin-resistant mutant (n = 18). In the multivariable model for the wild-type population, 1 000 000/µL of sequestered parasites were estimated to delay parasite clearance by 16.8% (95% confidence interval, 5.1%-28.5%), although it was not clear in the A675V population.
CONCLUSIONS: In patients with P. falciparum malaria without artemisinin-resistant mutations, intensive sequestration delays parasite clearance after treatment, which may contribute to reduced artemisinin efficacy.

© The Author(s) 2022. Published by Oxford University Press on behalf of
Infectious Diseases Society of America. All rights reserved. For permissions,
please e-mail: journals.permissions@oup.com.

DOI: 10.1093/cid/ciac944
PMID: 36519341 [Indexed for MEDLINE]

Conflict of interest statement: Potential conflicts of interest. The authors: No
reported conflicts of interest. All authors have submitted the ICMJE Form for
Disclosure of Potential Conflicts of Interest.

161. Front Immunol. 2023 May 2;14:1183983. doi: 10.3389/fimmu.2023.1183983.
eCollection 2023.

Seroprevalence and durability of antibody responses to AstraZeneca vaccination
in Ugandans with prior mild or asymptomatic COVID-19: implications for vaccine
policy.

Serwanga J(1)(2), Baine C(2), Mugaba S(1), Ankunda V(2), Auma BO(1), Oluka
GK(1)(2), Kato L(1), Kitabye I(1), Sembera J(2), Odoch G(1), Ejou P(1),
Nalumansi A(2), Gombe B(1), Musenero M(3), Kaleebu P(1)(2).

Author information:
(1)Pathogen Genomics, Phenotype, and Immunity Program, Medical Research Council,
Uganda Virus Research Institute and London School of Hygiene and Tropical
Medicine, Uganda Research Unit, Entebbe, Uganda.
(2)Department of Immunology, Uganda Virus Research Institute, Entebbe, Uganda.
(3)Science, Technology, and Innovation Secretariat, Office of the President,
Government of Uganda, Kampala, Uganda.

INTRODUCTION: The duration and timing of immunity conferred by COVID-19
vaccination in sub-Saharan Africa are crucial for guiding pandemic policy
interventions, but systematic data for this region is scarce. This study
investigated the antibody response after AstraZeneca vaccination in COVID-19
convalescent Ugandans.
METHODS: We recruited 86 participants with a previous rt-PCR-confirmed mild or
asymptomatic COVID-19 infection and measured the prevalence and levels of
spike-directed IgG, IgM, and IgA antibodies at baseline, 14 and 28 days after
the first dose (priming), 14 days after the second dose (boosting), and at six-
and nine-months post-priming. We also measured the prevalence and levels of
nucleoprotein-directed antibodies to assess breakthrough infections.
RESULTS: Within two weeks of priming, vaccination substantially increased the
prevalence and concentrations of spike-directed antibodies ($p < 0.0001$, Wilcoxon
signed rank test), with 97.0% and 66% of vaccinated individuals possessing S-IgG
and S-IgA antibodies before administering the booster dose. S-IgM prevalence

changed marginally after the initial vaccination and barely after the booster, consistent with an already primed immune system. However, we also observed a rise in nucleoprotein seroprevalence, indicative of breakthroughs six months after the initial vaccination.
DISCUSSION: Our results suggest that vaccination of COVID-19 convalescent individuals with the AstraZeneca vaccine induces a robust and differential spike-directed antibody response. The data highlights the value of vaccination as an effective method for inducing immunity in previously infected individuals and the importance of administering two doses to maintain protective immunity. Monitoring anti-spike IgG and IgA when assessing vaccine-induced antibody responses is suggested for this population; assessing S-IgM will underestimate the response. The AstraZeneca vaccine is a valuable tool in the fight against COVID-19. Further research is needed to determine the durability of vaccine-induced immunity and the potential need for booster doses.

Copyright © 2023 Serwanga, Baine, Mugaba, Ankunda, Auma, Oluka, Kato, Kitabye, Sembera, Odoch, Ejou, Nalumansi, Gombe, Musenero, Kaleebu and the COVID-19 Immunoprofiling Team.

DOI: 10.3389/fimmu.2023.1183983
PMCID: PMC10187141
PMID: 37205095 [Indexed for MEDLINE]

Conflict of interest statement: The authors declare that the research was conducted in the absence of any commercial or financial relationships that could be construed as a potential conflict of interest.

162. Lancet Public Health. 2023 May;8(5):e383-e390. doi: 10.1016/S2468-2667(23)00060-9.

The road to achieving epidemic-ready primary health care.

Frieden TR(1), Lee CT(2), Lamorde M(3), Nielsen M(2), McClelland A(2), Tangcharoensathien V(4).

Author information:
(1)Resolve to Save Lives, New York, NY, USA. Electronic address: trfrieden@resolvetosavelives.org.
(2)Resolve to Save Lives, New York, NY, USA.
(3)Infectious Diseases Institute, Makerere University, Kampala, Uganda.
(4)International Health Policy Program, Amphur Muang, Nonthaburi, Thailand.

Millions of avoidable deaths arising from the COVID-19 pandemic emphasise the need for epidemic-ready primary health care aligned with public health to identify and stop outbreaks, maintain essential services during disruptions, strengthen population resilience, and ensure health worker and patient safety.

The improvement in health security from epidemic-ready primary health care is a strong argument for increased political support and can expand primary health-care capacities to improve detection, vaccination, treatment, and coordination with public health-needs that became more apparent during the pandemic. Progress towards epidemic-ready primary health care is likely to be stepwise and incremental, advancing when opportunity arises based on explicit agreement on a core set of services, improved use of external and national funds, and payment based in large part on empanelment and capitation to improve outcomes and accountability, supplemented with funding for core staffing and infrastructure and well designed incentives for health improvement. Health-care worker and broader civil society advocacy, political consensus, and bolstering government legitimacy could promote strong primary health care. Epidemic-ready primary health-care infrastructure that is able to help prevent and withstand the next pandemic will require substantial financial and structural reforms and sustained political and financial commitment. Governments, advocates, and bilateral and multilateral agencies should seize this window of opportunity before it closes.

Copyright © 2023 The Author(s). Published by Elsevier Ltd. This is an Open Access article under the CC BY 4.0 license. Published by Elsevier Ltd.. All rights reserved.

DOI: 10.1016/S2468-2667(23)00060-9
PMCID: PMC10139016
PMID: 37120262 [Indexed for MEDLINE]

Conflict of interest statement: Declaration of interests We declare no competing interests.

163. EBioMedicine. 2023 May;91:104562. doi: 10.1016/j.ebiom.2023.104562. Epub 2023 Apr 24.

Immune response of a two-dose heterologous Ebola vaccine regimen: summary of three African clinical trials using a single validated Filovirus Animal Nonclinical Group enzyme-linked immunosorbent assay in a single accredited laboratory.

McLean C(1), Barry H(2), Kieh M(3), Anywaine Z(4), Tapima Rogers B(5), Doumbia S(6), Sirima SB(7), Serry-Bangura A(5), Habib Beavogui A(8), Gaddah A(9), Katwere M(10), Hendriks J(10), Keshinro B(10), Eholie S(11), Kibuuka H(12), Kennedy SB(3), Anzala O(13), Samai M(5), D'Ortenzio E(14), Leigh B(5), Sow S(15), Thiébaut R(16), Greenwood B(17), Watson-Jones D(18), Douoguih M(10), Luhn K(10), Robinson C(10).

Author information:
(1)Janssen Vaccines and Prevention BV, Leiden, the Netherlands. Electronic

address: cmclean@its.jnj.com.
(2)Centre MURAZ, Bobo-Dioulasso, Burkina Faso.
(3)Partnership for Research on Ebola Virus in Liberia (PREVAIL), Monrovia, Liberia.
(4)Medical Research Council/Uganda Virus Research Institute and London School of Hygiene and Tropical Medicine Uganda Research Unit, Entebbe, Uganda.
(5)University of Sierra Leone, Freetown, Sierra Leone.
(6)University Clinical Research Center, University of Sciences, Technique and Technology of Bamako, Bamako, Mali.
(7)Groupe de Recherche Action en Santé (GRAS), Ouagadougou, Burkina Faso.
(8)Centre National de Formation et de Recherche en Santé Rurale de Mafèrinyah, Mafèrinyah, Guinea.
(9)Janssen Research and Development, Beerse, Belgium.
(10)Janssen Vaccines and Prevention BV, Leiden, the Netherlands.
(11)Medical School, University Felix Houphouet Boigny, Abidjan, Cote d'Ivoire.
(12)Makerere University Walter Reed Project, Kampala, Uganda.
(13)Kenya AIDS Vaccine Initiative (KAVI), University of Nairobi, Nairobi, Kenya.
(14)ANRS Emerging Infectious Diseases, Institut national de la santé et de la recherche médicale (Inserm), Paris, France.
(15)Centre pour le Développement des Vaccins, Bamako, Mali.
(16)University of Bordeaux, Inserm, Bordeaux Population Health Research Center, Bordeaux, France.
(17)London School of Hygiene and Tropical Medicine, London, UK.
(18)London School of Hygiene and Tropical Medicine, London, UK; Mwanza Intervention Trials Unit, National Institute for Medical Research, Mwanza, Tanzania.

BACKGROUND: This analysis evaluated the immune response to the two-dose, heterologous Ad26.ZEBOV, MVA-BN-Filo Ebola virus vaccine regimen, administered 56-days apart, from multiple African sites based on results from one analytic laboratory.
METHODS: Immunogenicity across three trials (EBL2002, EBL2004/PREVAC, EBL3001) conducted in East and West Africa is summarised. Vaccine-induced Ebola glycoprotein-binding antibody concentrations were analysed by Q2 Solutions laboratory at baseline, 21 days (EBL2002 and EBL3001) or 28 days (EBL2004) post-dose 2 (regimen completion), and 12 months post-dose 1 using the validated Filovirus Animal Nonclinical Group Ebola glycoprotein enzyme-linked immunosorbent assay (ELISA). Responders were defined as those with a >2.5-fold increase from baseline or the lower limit of quantification (LLOQ) if <LLOQ at baseline.
FINDINGS: At 21 or 28 (21/28) days post-dose 2, the geometric mean concentration (GMC) range was 3810-7518 ELISA units (EU)/mL (percent responders: ≥98%) in adults, 9929-13532 EU/mL (≥98%) in adolescents aged 12-17 years, 10,212-17388 EU/mL (≥99%) in older children, and 22,568-25111 EU/mL (≥98%) in younger children. When stratified by country, GMCs at 21/28 days post-dose 2 were generally similar among adults and within paediatric cohorts (percent responders: 95%-100%). At month 12, GMC range was 259-437 EU/mL (percent

responders: 49%-88%) in adults and 386-1139 EU/mL (70%-100%) in paediatric participants.
INTERPRETATION: Based on data from a single laboratory using a single validated assay, Ad26.ZEBOV, MVA-BN-Filo induced a strong humoral immune response, with ≥95% of participants across countries classified as responders at 21/28 days post-dose 2 (regimen completion), regardless of age.
FUNDING: Janssen Vaccines & Prevention BV; Innovative Medicines Initiative.

Copyright © 2023 The Author(s). Published by Elsevier B.V. All rights reserved.

DOI: 10.1016/j.ebiom.2023.104562
PMCID: PMC10149382
PMID: 37099841 [Indexed for MEDLINE]

Conflict of interest statement: Declaration of interests CM, AG, JH, BK, MD, KL, and CR were full-time employees of Janssen Pharmaceuticals at the time of the study and report stock or stock options in Janssen Pharmaceuticals. HB's institution has received funding from the Innovative Medicines Initiative to conduct the EBL2002 study. M Katwere was a full-time employee of Janssen Pharmaceuticals at the time of the study. SE's institution has received funding from Inserm France to conduct the EBL2002 study. BL's institution has received funding from the Innovative Medicines Initiative to conduct the EBL3005 study. DW-J is a consortia partner with Janssen Pharmaceuticals on EBOVAC1 and EBOVAC3 projects, has received Ad26.MVA Ebola vaccines donated by Janssen Pharmaceuticals for clinical trials under EBOVAC1 and EBOVAC3 projects, and her institution has received funding from the Innovative Medicines Initiative to conduct the EBL3005 study. M Kieh, ZA, BTR, SD, SBS, AS-B, AHB, HK, SBK, OA, MS, EDO, SS, RT, and BG declare no competing interests.

164. Lancet Microbe. 2023 May;4(5):e300-e308. doi: 10.1016/S2666-5247(22)00332-9. Epub 2023 Apr 17.

Optimising the timing of whooping cough immunisation in mums (OpTIMUM) through investigating pertussis vaccination in pregnancy: an open-label, equivalence, randomised controlled trial.

Calvert A(1), Amirthalingam G(2), Andrews N(2), Basude S(3), Coleman M(4), Cuthbertson H(5), England A(5), Greening V(6), Hallis B(5), Johnstone E(7), Jones CE(8), Karampatsas K(6), Khalil A(6), Le Doare K(9), Matheson M(5), Peregrine E(10), Snape MD(11), Vatish M(12), Heath PT(6); OpTIMUM Study Group.

Collaborators: Burtt A, Byrne W, Capp A, Carty L, Chawla K, Collins S, Cornish E, Daniel O, Fretwell J, Gorringe A, Gubbins T, Hall T, Johnston S, Khan U, Lim S, Martin N, Morey E, Mossop J, O'Brien K, Owino N, Powell D, Ramkhelawon L, Ratcliffe H, Roberts H, Roseman F, Sparks L, Stapley L, Taylor S, Walbridge F, Watts R, Wellstead SJ, Wishlade T.

Author information:
(1)Centre for Neonatal and Paediatric Infection and Vaccine Institute St George's, University of London, London, UK; St George's University Hospitals NHS Foundation Trust, London, UK. Electronic address: acalvert@sgul.ac.uk.
(2)Immunisation and Vaccine Preventable Diseases Division, UK Health Security Agency, London, UK.
(3)University Hospital Bristol and Weston NHS Foundation Trust, Bristol, UK.
(4)NIHR Southampton Clinical Research Facility and Biomedical Research Centre, University Hospital Southampton NHS Foundation Trust, Southampton, UK; Reproductive Health Research Team, University Hospital Southampton NHS Foundation Trust, Southampton, UK.
(5)Research and Evaluation, UK Health Security Agency, Salisbury, UK.
(6)Centre for Neonatal and Paediatric Infection and Vaccine Institute St George's, University of London, London, UK; St George's University Hospitals NHS Foundation Trust, London, UK.
(7)Manchester Academic Health Science Centre, St Mary's Hospital, Manchester University NHS Foundation Trust, Manchester, UK; Maternal and Fetal Health Research Centre, School of Medical Sciences, University of Manchester, Manchester, UK.
(8)NIHR Southampton Clinical Research Facility and Biomedical Research Centre, University Hospital Southampton NHS Foundation Trust, Southampton, UK; Clinical and Experimental Sciences, University of Southampton, Southampton, UK.
(9)Centre for Neonatal and Paediatric Infection and Vaccine Institute St George's, University of London, London, UK; St George's University Hospitals NHS Foundation Trust, London, UK; Research and Evaluation, UK Health Security Agency, Salisbury, UK; Medical Research Council and Ugandan Virus Research Institute and the London School of Hygiene and Tropical Medicine, Kampala, Uganda; Makerere University Johns Hopkins University, Kampala, Uganda.
(10)Kingston Hospital NHS Foundation Trust, Surrey, UK.
(11)Department of Paediatrics, University of Oxford, Oxford, UK; Oxford NIHR Biomedical Research Centre, University of Oxford, Oxford, UK.
(12)Nuffield Department of Women's and Reproductive Health, University of Oxford, Oxford, UK; Clinical Research Network, Thames Valley and South Midlands.

BACKGROUND: Pertussis vaccination in pregnancy is recommended in many countries to provide protection to young infants. The best timing for this vaccination is uncertain. In the UK, vaccination is recommended between 16 weeks and 32 weeks of gestation. In this trial we aimed to investigate the equivalence of three time periods for pertussis vaccination in pregnancy.
METHODS: In this open-label, equivalence, randomised controlled trial to investigate equivalence of different time windows for pertussis vaccination in pregnancy, participants were randomly assigned (1:1:1 ratio) to receive a pertussis-containing vaccine (Boostrix-inactivated poliovirus vaccine) in one of three gestational age groups, comprising group 1 (≤23 weeks + 6 days), group 2 (24-27 weeks + 6 days), and group 3 (28-31 weeks + 6 days) using a computer-generated randomisation list. The primary outcome was concentration of

pertussis-specific antibodies in the infant born at term at birth. Maternal blood sampling was done before and 2 weeks after vaccination and at delivery, together with a cord sample, and an infant sample was collected at least 4 weeks after primary vaccination. Reactogenicity was assessed for 7 days after vaccination. This trial was registered with ClinicalTrials.gov (NCT03908164).
FINDINGS: Between May 7, 2019, and Feb 13, 2020, of 1010 women assessed for eligibility, 364 women were recruited and 351 received the intervention (120 in group 1, 119 in group 2, and 112 in group 3). Equivalence of time periods was demonstrated for anti-pertussis toxin and anti-pertactin IgG concentrations. The cord blood geometric mean concentrations of anti-filamentous haemagglutinin IgG were higher with increasing gestational age at vaccination, such that for infants in group 1 (≤23 weeks + 6 days), equivalence to group 3 (28-31 weeks + 6 days) was not shown. Reported rates of fever were similar between study groups.
INTERPRETATION: Pertussis vaccination at three different time intervals in pregnancy resulted in equivalent concentrations of IgG antibodies in infants against two of the three pertussis antigens assessed. Overall, these findings support recommendations to vaccinate any time between 16 weeks and 32 weeks of gestation.
FUNDING: The Thrasher Research Fund and the National Immunisation Schedule Evaluation Consortium through the National Institute for Health and Care Research policy research programme.

Copyright © 2023 The Author(s). Published by Elsevier Ltd. This is an Open Access article under the CC BY 4.0 license. Published by Elsevier Ltd.. All rights reserved.

DOI: 10.1016/S2666-5247(22)00332-9
PMID: 37080224 [Indexed for MEDLINE]

Conflict of interest statement: Declaration of interests GA and NA report that the Immunisation and Vaccine Preventable Diseases Division has provided vaccine manufacturers with postmarketing surveillance reports on pneumococcal and meningococcal infection, which the companies are required to submit to the UK Licensing Authority in compliance with their Risk Management Strategy. A cost recovery charge is made for these reports. CEJ has done studies on behalf of the University of Southampton and University Hospital Southampton NHS Foundation Trust funded by vaccine manufacturers, including Novavax, Moderna, Medicago, and Pfizer, but receives no personal funding for these activities. CEJ has served on advisory boards, data safety monitoring boards, or as a consultant for Moderna, MSD, Sanofi, Minervax, and Pfizer. MDS has acted as an investigator on behalf of the University of Oxford for studies funded or supported by vaccine manufacturers including GlaxoSmithKline, Pfizer, MCM vaccines, Novavax, AstraZeneca, and Janssen. MDS received no direct financial benefit for this work. From September, 2022 (after completion of this work), MDS became an employee of Medimmune and Moderna. PTH has conducted studies on behalf of St George's, University of London funded by vaccine manufacturers, including AstraZeneca, Novavax, Moderna, Valneva, Janssen, Minervax, and Pfizer, but

receives no personal funding for these activities. All other authors declare no competing interests.

165. Lancet Glob Health. 2023 May;11(5):e729-e739. doi: 10.1016/S2214-109X(23)00109-2.

Timing of seasonal influenza epidemics for 25 countries in Africa during 2010-19: a retrospective analysis.

Igboh LS(1), Roguski K(2), Marcenac P(3), Emukule GO(4), Charles MD(3), Tempia S(5), Herring B(6), Vandemaele K(7), Moen A(3), Olsen SJ(3), Wentworth DE(3), Kondor R(3), Mott JA(7), Hirve S(7), Bresee JS(8), Mangtani P(9), Nguipdop-Djomo P(9), Azziz-Baumgartner E(3).

Author information:
(1)Influenza Division, National Center for Immunization and Respiratory Diseases, Centers for Disease Control and Prevention, Atlanta, GA, USA; Faculty of Epidemiology and Population Health, London School of Hygiene & Tropical Medicine, London, UK; Immunization Systems Branch, Global Immunization Division, Center for Global Health, Centers for Disease Control and Prevention, Atlanta, GA, USA. Electronic address: ymq4@cdc.gov.
(2)National Center for Emerging and Zoonotic Infectious Diseases, Centers for Disease Control and Prevention, Atlanta, GA, USA.
(3)Influenza Division, National Center for Immunization and Respiratory Diseases, Centers for Disease Control and Prevention, Atlanta, GA, USA.
(4)Centers for Disease Control and Prevention, Nairobi, Kenya.
(5)School of Public Health, Faculty of Health Sciences, University of the Witwatersrand, Johannesburg, South Africa; Department of Infectious Hazard Management, World Health Organization, Geneva, Switzerland.
(6)World Health Organization-Regional Office for Africa, Brazzaville, Congo.
(7)Department of Infectious Hazard Management, World Health Organization, Geneva, Switzerland.
(8)The Task Force for Global Health, Decatur, GA, USA.
(9)Faculty of Epidemiology and Population Health, London School of Hygiene & Tropical Medicine, London, UK.

Comment in
    Lancet Glob Health. 2023 May;11(5):e640-e641.

BACKGROUND: Using country-specific surveillance data to describe influenza epidemic activity could inform decisions on the timing of influenza vaccination. We analysed surveillance data from African countries to characterise the timing of seasonal influenza epidemics to inform national vaccination strategies.
METHODS: We used publicly available sentinel data from African countries reporting to the WHO Global Influenza Surveillance and Response FluNet platform that had 3-10 years of data collected during 2010-19. We calculated a 3-week

moving proportion of samples positive for influenza virus and assessed epidemic timing using an aggregate average method. The start and end of each epidemic were defined as the first week when the proportion of positive samples exceeded or went below the annual mean, respectively, for at least 3 consecutive weeks. We categorised countries into five epidemic patterns: northern hemisphere-dominant, with epidemics occurring in October-March; southern hemisphere-dominant, with epidemics occurring in April-September; primarily northern hemisphere with some epidemic activity in southern hemisphere months; primarily southern hemisphere with some epidemic activity in northern hemisphere months; and year-round influenza transmission without a discernible northern hemisphere or southern hemisphere predominance (no clear pattern).
FINDINGS: Of the 34 countries reporting data to FluNet, 25 had at least 3 years of data, representing 46% of the countries in Africa and 89% of Africa's population. Study countries reported RT-PCR respiratory virus results for a total of 503 609 specimens (median 12 971 [IQR 9607-20 960] per country-year), of which 74 001 (15%; median 2078 [IQR 1087-3008] per country-year) were positive for influenza viruses. 248 epidemics occurred across 236 country-years of data (median 10 [range 7-10] per country). Six (24%) countries had a northern hemisphere pattern (Algeria, Burkina Faso, Egypt, Morocco, Niger, and Tunisia). Eight (32%) had a primarily northern hemisphere pattern with some southern hemisphere epidemics (Cameroon, Ethiopia, Mali, Mozambique, Nigeria, Senegal, Tanzania, and Togo). Three (12%) had a primarily southern hemisphere pattern with some northern hemisphere epidemics (Ghana, Kenya, and Uganda). Three (12%) had a southern hemisphere pattern (Central African Republic, South Africa, and Zambia). Five (20%) had no clear pattern (Côte d'Ivoire, DR Congo, Madagascar, Mauritius, and Rwanda).
INTERPRETATION: Most countries had identifiable influenza epidemic periods that could be used to inform authorities of non-seasonal and seasonal influenza activity, guide vaccine timing, and promote timely interventions.
FUNDING: None.
TRANSLATIONS: For the Berber, Luganda, Xhosa, Chewa, Yoruba, Igbo, Hausa and Afan Oromo translations of the abstract see Supplementary Materials section.

Copyright © 2023 The Author(s). Published by Elsevier Ltd. This is an Open Access article under the CC BY-NC-ND 4.0 license. Published by Elsevier Ltd.. All rights reserved.

DOI: 10.1016/S2214-109X(23)00109-2
PMCID: PMC10126228
PMID: 37061311 [Indexed for MEDLINE]

Conflict of interest statement: Declaration of interests We declare no competing interests.

166. Lancet Child Adolesc Health. 2023 May;7(5):336-346. doi: 10.1016/S2352-4642(23)00004-4. Epub 2023 Mar 13.

Development of treatment-decision algorithms for children evaluated for pulmonary tuberculosis: an individual participant data meta-analysis.

Gunasekera KS(1), Marcy O(2), Muñoz J(3), Lopez-Varela E(4), Sekadde MP(5), Franke MF(6), Bonnet M(7), Ahmed S(8), Amanullah F(9), Anwar A(10), Augusto O(11), Aurilio RB(12), Banu S(13), Batool I(10), Brands A(14), Cain KP(15), Carratalá-Castro L(16), Caws M(17), Click ES(15), Cranmer LM(18), García-Basteiro AL(19), Hesseling AC(20), Huynh J(21), Kabir S(13), Lecca L(22), Mandalakas A(23), Mavhunga F(14), Myint AA(24), Myo K(25), Nampijja D(26), Nicol MP(27), Orikiriza P(28), Palmer M(20), Sant'Anna CC(29), Siddiqui SA(30), Smith JP(31), Song R(32), Thuong Thuong NT(21), Ung V(33), van der Zalm MM(20), Verkuijl S(14), Viney K(34), Walters EG(35), Warren JL(36), Zar HJ(37), Marais BJ(38), Graham SM(39), Debray TPA(3), Cohen T(40), Seddon JA(41).

Author information:
(1)Department of Epidemiology of Microbial Diseases, Yale School of Public Health, New Haven, CT, USA. Electronic address: kenneth.gunasekera@yale.edu.
(2)Inserm UMR1219, Institut de Recherche pour le Développement EMR 271, GHiGS, University of Bordeaux, Bordeaux, France.
(3)Julius Center for Health Sciences and Primary Care, University Medical Center Utrecht, Utrecht University, Utrecht, Netherlands.
(4)ISGlobal, Hospital Clínic - Universitat de Barcelona, Barcelona, Spain; Centro de Investigação em Saúde de Manhiça, Maputo, Mozambique; Desmond Tutu TB Centre, Department of Paediatrics and Child Health, Faculty of Medicine and Health Sciences, Stellenbosch University, Tygerberg, South Africa.
(5)National Tuberculosis and Leprosy Program, Kampala, Uganda.
(6)Department of Global Health and Social Medicine, Harvard Medical School, Boston, MA, USA.
(7)University of Montpellier, TransVIHMI, Institut de Recherche pour le Développement, Inserm, Montpellier, France; Epicentre, Mbarara, Uganda.
(8)Department of Paediatrics, Dhaka Medical College Hospital, Dhaka, Bangladesh.
(9)Indus Hospital & Health Network, Karachi, Pakistan; The Aga Khan University Hospital, Karachi, Pakistan.
(10)Indus Hospital & Health Network, Karachi, Pakistan.
(11)Centro de Investigação em Saúde de Manhiça, Maputo, Mozambique.
(12)Instituto de Puericultura e Pediatria Martagao Gesteira, Universidade Federal do Rio de Janeiro, Rio de Janeiro, Brazil.
(13)Programme on Emerging Infections, Infectious Disease Division, icddr,b, Dhaka, Bangladesh.
(14)Global Tuberculosis Programme, WHO, Geneva, Switzerland.
(15)US Centers for Disease Control and Prevention, Atlanta, GA, USA.
(16)ISGlobal, Hospital Clínic - Universitat de Barcelona, Barcelona, Spain; Centro de Investigação em Saúde de Manhiça, Maputo, Mozambique.
(17)Department of Clinical Sciences, Liverpool School of Tropical Medicine, Liverpool, UK; Birat Nepal Medical Trust, Lazmipat, Kathmandu, Nepal.
(18)Department of Pediatrics, Emory School of Medicine, Atlanta, GA, USA;

Department of Epidemiology, Emory Rollins School of Public Health, Atlanta, GA, USA; Children's Healthcare of Atlanta, Atlanta, GA, USA.
(19)ISGlobal, Hospital Clínic - Universitat de Barcelona, Barcelona, Spain; Centro de Investigação em Saúde de Manhiça, Maputo, Mozambique; Centro de Investigación Biomédica en Red de Enfermedades Infecciosas, Barcelona, Spain.
(20)Desmond Tutu TB Centre, Department of Paediatrics and Child Health, Faculty of Medicine and Health Sciences, Stellenbosch University, Tygerberg, South Africa.
(21)Oxford University Clinical Research Unit, Centre for Tropical Diseases, Ho Chi Minh City, Viet Nam; Nuffield Department of Medicine, Centre for Tropical Medicine and Global Health, University of Oxford, Oxford, UK.
(22)Department of Global Health and Social Medicine, Harvard Medical School, Boston, MA, USA; Socios En Salud Surcursal Perú, Lima, Perú.
(23)Global TB Program, Baylor College of Medicine and Texas Children's Hospital, Houston, TX, USA; Clinical Infectious Disease Group, German Center for Infectious Research, Clinical TB Unit, Research Center Borstel, Borstel, Germany.
(24)Department of Paediatrics, University of Medicine, Mandalay, Myanmar.
(25)Department of Paediatrics, University of Medicine, Magway, Myanmar.
(26)Department of Paediatrics, Mbarara University of Science and Technology, Mbarara, Uganda.
(27)Division of Infection and Immunity, Department of Biomedical Sciences, University of Western Australia, Perth, WA, Australia.
(28)Epicentre, Mbarara, Uganda; Department of Microbiology, Division of Basic Medical Sciences, School of Medicine, University of Global Health Equity, Kigali, Rwanda.
(29)Faculty of Medicine, Universidade Federal do Rio de Janeiro, Rio de Janeiro, Brazil.
(30)Department of Global Health and Social Medicine, Harvard Medical School, Boston, MA, USA; Indus Hospital & Health Network, Karachi, Pakistan.
(31)Department of Health Policy and Management, Yale School of Public Health, New Haven, CT, USA; US Centers for Disease Control and Prevention, Atlanta, GA, USA.
(32)Department of Pediatrics, Harvard Medical School, Boston, MA, USA; Oxford Vaccine Group, Department of Paediatrics, University of Oxford, Oxford, UK.
(33)University of Health Sciences, Phnom Penh, Cambodia; National Pediatric Hospital, Phnom Penh, Cambodia.
(34)Global Tuberculosis Programme, WHO, Geneva, Switzerland; School of Public Health, University of Sydney, Sydney, NSW, Australia.
(35)Desmond Tutu TB Centre, Department of Paediatrics and Child Health, Faculty of Medicine and Health Sciences, Stellenbosch University, Tygerberg, South Africa; Directorate of Integrated Laboratory Medicine, Institute of Human Genetics, Newcastle upon Tyne NHS Foundation Trust, Newcastle upon Tyne, UK.
(36)Department of Biostatistics, Yale School of Public Health, New Haven, CT, USA.
(37)Department of Paediatrics and Child Health, Red Cross Children's Hospital, and SA-MRC Unit on Child and Adolescent Health, University of Cape Town, Cape

Town, South Africa.
(38)The Children's Hospital at Westmead Clinical School, Faculty of Medicine and Health, University of Sydney, Sydney, NSW, Australia.
(39)Department of Paediatrics and Murdoch Children's Research Institute, Royal Children's Hospital, University of Melbourne, Melbourne, VIC, Australia; Burnet Institute, Melbourne, VIC, Australia.
(40)Department of Epidemiology of Microbial Diseases, Yale School of Public Health, New Haven, CT, USA.
(41)Desmond Tutu TB Centre, Department of Paediatrics and Child Health, Faculty of Medicine and Health Sciences, Stellenbosch University, Tygerberg, South Africa; Department of Infectious Diseases, Imperial College London, London, UK.

BACKGROUND: Many children with pulmonary tuberculosis remain undiagnosed and untreated with related high morbidity and mortality. Recent advances in childhood tuberculosis algorithm development have incorporated prediction modelling, but studies so far have been small and localised, with limited generalisability. We aimed to evaluate the performance of currently used diagnostic algorithms and to use prediction modelling to develop evidence-based algorithms to assist in tuberculosis treatment decision making for children presenting to primary health-care centres.
METHODS: For this meta-analysis, we identified individual participant data from a WHO public call for data on the management of tuberculosis in children and adolescents and referral from childhood tuberculosis experts. We included studies that prospectively recruited consecutive participants younger than 10 years attending health-care centres in countries with a high tuberculosis incidence for clinical evaluation of pulmonary tuberculosis. We collated individual participant data including clinical, bacteriological, and radiological information and a standardised reference classification of pulmonary tuberculosis. Using this dataset, we first retrospectively evaluated the performance of several existing treatment-decision algorithms. We then used the data to develop two multivariable prediction models that included features used in clinical evaluation of pulmonary tuberculosis-one with chest x-ray features and one without-and we investigated each model's generalisability using internal-external cross-validation. The parameter coefficient estimates of the two models were scaled into two scoring systems to classify tuberculosis with a prespecified sensitivity target. The two scoring systems were used to develop two pragmatic, treatment-decision algorithms for use in primary health-care settings.
FINDINGS: Of 4718 children from 13 studies from 12 countries, 1811 (38·4%) were classified as having pulmonary tuberculosis: 541 (29·9%) bacteriologically confirmed and 1270 (70·1%) unconfirmed. Existing treatment-decision algorithms had highly variable diagnostic performance. The scoring system derived from the prediction model that included clinical features and features from chest x-ray had a combined sensitivity of 0·86 [95% CI 0·68-0·94] and specificity of 0·37 [0·15-0·66] against a composite reference standard. The scoring system derived from the model that included only clinical features had a combined sensitivity of 0·84 [95% CI 0·66-0·93] and specificity of 0·30 [0·13-0·56] against a

composite reference standard. The scoring system from each model was placed after triage steps, including assessment of illness acuity and risk of poor tuberculosis-related outcomes, to develop treatment-decision algorithms.
INTERPRETATION: We adopted an evidence-based approach to develop pragmatic algorithms to guide tuberculosis treatment decisions in children, irrespective of the resources locally available. This approach will empower health workers in primary health-care settings with high tuberculosis incidence and limited resources to initiate tuberculosis treatment in children to improve access to care and reduce tuberculosis-related mortality. These algorithms have been included in the operational handbook accompanying the latest WHO guidelines on the management of tuberculosis in children and adolescents. Future prospective evaluation of algorithms, including those developed in this work, is necessary to investigate clinical performance.
FUNDING: WHO, US National Institutes of Health.

© 2023 World Health Organization. Published by Elsevier Ltd. All rights reserved. This is an Open Access article published under the CC BY 3.0 IGO license which permits unrestricted use, distribution, and reproduction in any medium, provided the original work is properly cited. In any use of this article, there should be no suggestion that WHO endorses any specific organisation, products or services. The use of the WHO logo is not permitted. This notice should be preserved along with the article's original URL.

DOI: 10.1016/S2352-4642(23)00004-4
PMCID: PMC10127218
PMID: 36924781 [Indexed for MEDLINE]

Conflict of interest statement: Declaration of interests We declare no competing interests.

167. Lancet Infect Dis. 2023 May;23(5):e185-e189. doi: 10.1016/S1473-3099(22)00723-X. Epub 2022 Dec 20.

Advancing detection and response capacities for emerging and re-emerging pathogens in Africa.

Nachega JB(1), Nsanzimana S(2), Rawat A(3), Wilson LA(4), Rosenthal PJ(5), Siedner MJ(6), Varma JK(7), Kilmarx PH(8), Mutesa L(9), Tanner M(10), Binagwaho A(11), Forrest J(4), Mbala-Kingebeni P(12), Muyembe-Tamfum JJ(12), Ntoumi F(13), Zumla A(14), de Oliveira T(15), Mills EJ(16).

Author information:
(1)Department of Epidemiology, University of Pittsburgh School of Public Health, Pittsburgh, PA, USA; Department of Infectious Diseases and Microbiology, University of Pittsburgh School of Public Health, Pittsburgh, PA, USA; Department of Medicine, Division of Infectious Diseases, Faculty of Science and

Faculty of Medicine and Health Sciences, Stellenbosch University, Cape Town, South Africa; Department of Epidemiology, Johns Hopkins Bloomberg School of Public Health, Baltimore, MD, USA; Department of International Health, Johns Hopkins Bloomberg School of Public Health, Baltimore, MD, USA. Electronic address: jbn16@pitt.edu.
(2)University Teaching Hospital, Butare, Rwanda; University of Global Health Equity, Kigali, Rwanda.
(3)Department of Health Research Methods, Evidence and Impact, Faculty of Health Sciences, McMaster University, Hamilton, ON, Canada; School of Population and Public Health, University of British Columbia, Vancouver, BC, Canada.
(4)Platform Life Sciences, Vancouver, BC, Canada; Department of Real World and Advanced Analytics, Vancouver, Cytel, Vancouver, BC, Canada.
(5)Department of Medicine, Division of Infectious Diseases, University of California San Francisco, San Francisco, CA, USA.
(6)Department of Medicine, Harvard Medical School and Massachusetts General Hospital, Boston, MA, USA; Faculty of Medicine, Mbarara University of Science and Technology, Mbarara, Uganda.
(7)Department of Population Health Sciences, Weill Cornell Medicine, NY, USA.
(8)Fogarty International Center, National Institutes of Health, Bethesda, MD, USA.
(9)Center for Human Genetics, University of Rwanda, Kigali, Rwanda.
(10)Swiss Tropical and Public Health Institute, University of Basel, Basel, Switzerland.
(11)University of Global Health Equity, Kigali, Rwanda.
(12)National Institute for Bio-Medical Research, Kinshasa, Democratic Republic of the Congo; University of Kinshasa School of Medicine, Kinshasa, Democratic Republic of the Congo.
(13)Fondation Congolaise pour la Recherche Médicale, Brazzaville, Republic of the Congo; Institute for Tropical Medicine, University of Tübingen, Tübingen, Germany.
(14)Division of Infection and Immunity, Centre for Clinical Microbiology, University College London, London, UK; NIHR Biomedical Research Centre, University College London Hospitals NHS Foundation Trust, London, UK.
(15)School for Data Science and Computational Thinking, Faculty of Science and Faculty of Medicine and Health Sciences, Stellenbosch University, Cape Town, South Africa; Centre for Epidemic Response and Innovation, Faculty of Science and Faculty of Medicine and Health Sciences, Stellenbosch University, Cape Town, South Africa.
(16)Department of Health Research Methods, Evidence and Impact, Faculty of Health Sciences, McMaster University, Hamilton, ON, Canada; Platform Life Sciences, Vancouver, BC, Canada.

Recurrent disease outbreaks caused by a range of emerging and resurging pathogens over the past decade reveal major gaps in public health preparedness, detection, and response systems in Africa. Underlying causes of recurrent disease outbreaks include inadequacies in the detection of new infectious disease outbreaks in the community, in rapid pathogen identification, and in

proactive surveillance systems. In sub-Saharan Africa, where 70% of zoonotic
outbreaks occur, there remains the perennial risk of outbreaks of new or
re-emerging pathogens for which no vaccines or treatments are available. As the
Ebola virus disease, COVID-19, and mpox (formerly known as monkeypox) outbreaks
highlight, a major paradigm shift is required to establish an effective
infrastructure and common frameworks for preparedness and to prompt national and
regional public health responses to mitigate the effects of future pandemics in
Africa.

Copyright © 2023 Elsevier Ltd. All rights reserved.

DOI: 10.1016/S1473-3099(22)00723-X
PMCID: PMC10023168
PMID: 36563700 [Indexed for MEDLINE]

Conflict of interest statement: Declaration of interests JBN is supported by the
US National Institutes of Health (NIH; grant numbers NIH/FIC 1R25TW011217-01,
NIH/FIC 1D43TW010937-01A1, NIH/FIC D43TW011827-01A1, NIH/FIC 1R21TW011706-0,
and
NIH/NIAID 5U01AI096299-13). FN and AZ are Co-directors of the Pan-African
Network on Emerging and Re-Emerging Infections funded by the European and
Developing Countries Clinical Trials Partnership (EDCTP) within the EU Horizon
2020 Framework Programme. FN and AZ also acknowledge support from the EDCTP
Central Africa Clinical Research Network. AZ is a UK National Institute for
Health Research senior investigator, and a Mahathir Science Award and EU-EDCTP
Pascoal Mocumbi Prize laureate. J-JM-T is supported by Africa Centre for Disease
Control and Prevention and the Bill and Melinda Gates Foundation, and holds NIH
grants (NIH/NIAID grant number 75N91019D00024-P00001-759102000025-5). J-JM-T was
part of the group that discovered the Ebola virus in 1976, and was awarded Le
Prix Christophe Merieux from Institut de France in 2015 and was the recipient of
the third Hideyo Noguchi Africa Prize for Medical Research in 2019 from the
Japan Government, for his research to confront Ebola and other emerging and
re-emerging pathogens, and for his efforts to train legions of disease fighters
in Africa. All other authors declare no competing interests. TdO led the team of
South African researchers who discovered the omicron variant of SARS-CoV-2 in
November, 2021.

168. Pathogens. 2023 Apr 28;12(5):658. doi: 10.3390/pathogens12050658.

The Evolution of Post-Vaccine G8P[4] Group a Rotavirus Strains in Rwanda;
Notable Variance at the Neutralization Epitope Sites.

Mwangi PN(1), Potgieter RL(1), Uwimana J(2), Mutesa L(2)(3), Muganga N(2),
Murenzi D(2), Tusiyenge L(2), Mwenda JM(4), Mogotsi MT(1), Rakau K(5), Esona
MD(5), Steele AD(5), Seheri ML(5), Nyaga MM(1).

Author information:
(1)Next Generation Sequencing Unit, Division of Virology, Faculty of Health Sciences, University of the Free State, Bloemfontein 9300, South Africa.
(2)Kigali University Teaching Hospital, College of Medicine and Health Sciences, University of Rwanda, Kigali P.O. Box 4285, Rwanda.
(3)Centre for Human Genetics, College of Medicine and Health Sciences, University of Rwanda, Kigali P.O. Box 4285, Rwanda.
(4)World Health Organization, Regional Office for Africa, Brazzaville P.O. Box 06, Congo.
(5)Diarrhoeal Pathogens Research Unit, Sefako Makgatho Health Sciences University (MEDUNSA), Pretoria 0204, South Africa.

Africa has a high level of genetic diversity of rotavirus strains, which is suggested to be a possible reason contributing to the suboptimal effectiveness of rotavirus vaccines in this region. One strain that contributes to this rotavirus diversity in Africa is the G8P[4]. This study aimed to elucidate the entire genome and evolution of Rwandan G8P[4] strains. Illumina sequencing was performed for twenty-one Rwandan G8P[4] rotavirus strains. Twenty of the Rwandan G8P[4] strains had a pure DS-1-like genotype constellation, and one strain had a reassortant genotype constellation. Notable radical amino acid differences were observed at the neutralization sites when compared with cognate regions in vaccine strains potentially playing a role in neutralization escape. Phylogenetic analysis revealed that the closest relationship was with East African human group A rotavirus (RVA) strains for five of the genome segments. Two genome sequences of the NSP4 genome segment were closely related to bovine members of the DS-1-like family. Fourteen VP1 and eleven VP3 sequences had the closest relationships with the RotaTeq™ vaccine WC3 bovine genes. These findings suggest that the evolution of VP1 and VP3 might have resulted from reassortment events with RotaTeq™ vaccine WC3 bovine genes. The close phylogenetic relationship with East African G8P[4] strains from Kenya and Uganda suggests co-circulation in these countries. These findings highlight the need for continued whole-genomic surveillance to elucidate the evolution of G8P[4] strains, especially after the introduction of rotavirus vaccination.

DOI: 10.3390/pathogens12050658
PMCID: PMC10223037
PMID: 37242329

Conflict of interest statement: The authors declare no conflict of interest.

169. Sci Rep. 2023 Apr 25;13(1):6746. doi: 10.1038/s41598-023-33740-9.

Genotyping of Mycobacterium tuberculosis complex isolated from humans and animals in northeastern Iran.

Ghazvini K(#)(1)(2), Khoshbakht R(#)(3)(4), Tadayon K(5), Mosavari N(6),

BahramiTaghanaki HR(7), Mohammadi GR(8), Rashti Baf M(9), Nourian K(10), Samiei A(11), Ghavidel M(12).

Author information:
(1)Antimicrobial Resistance Research Center, Mashhad University of Medical Sciences, Mashhad, Iran.
(2)Department of Microbiology and Virology, School of Medicine, Mashhad University of Medical Sciences, Mashhad, Iran.
(3)Department of Laboratory Sciences, School of Paramedical Sciences, Mashhad University of Medical Sciences, Mashhad, Iran.
(4)Student Research Committee, Mashhad University of Medical Sciences, Mashhad, Iran.
(5)Department of Microbiology, Razi Vaccine and Serum Research Institute (RVSRI), Agricultural Research, Education and Extension Organization (AREEO), Karaj, Iran.
(6)PPD Tuberculin Department, Razi Vaccine and Serum Research Institute, (RVSRI), Agricultural Research, Education and Extension Organization (AREEO), Karaj, Iran.
(7)School of Persian Medicine, Mashhad University of Medical Sciences, Mashhad, Iran.
(8)Department of Clinical Sciences, School of Veterinary Medicine, Ferdowsi University of Mashhad, Mashhad, Iran.
(9)Deputy of Veterinary Administration of Khorasan Razavi Province, Mashhad, Iran.
(10)Department of Veterinary Medicine, Ferdowsi University of Mashhad, Mashhad, Iran.
(11)Tuberculosis and Leprosy Coordinator at Health Chancellor, Health Center of Khorasan State, Mashhad University of Medical Sciences, Mashhad, Iran.
(12)Shahid Hasheminejad Hospital, Mashhad University of Medical Sciences, Mashhad, Iran. Ghavidelmd@mums.ac.ir.
(#)Contributed equally

The objective of this study was to genotype Mycobacterium tuberculosis complex isolated from humans and cattle in northern Iran. Over the course of one year, a total of 120 human and 21 cattle isolates were tested using region of difference (RD)-based polymerase chain reaction (PCR) and mycobacterial interspersed repetitive unites-variable number tandem repeats (MIRU-VNTR). In M. tuberculosis, out of 120 isolates investigated, the most common genotype detected was NEW-1 (53.3%), followed by CAS/ Delhi (24.1%), Haarlem (5%), Beijing (4.16%), Uganda I (4.16%), S (3.3%), Ural (0.83%), TUR (0.83%), Uganda II (0.83%), Lam (0.83%) and Cameroon (0.83%). The HGDI rate was 0.9981 and the clustering rate was 10.83. Of the isolates, QUB26 had the highest allele diversity (h: 0.76), while the loci Mtub29 and MIRU24 had the lowest (h: 0). In M. Bovis, out of 123 collected tissue samples, 21 (17%) grew on culture media. The HGDI rate was 0.71 and clustering rate was 85.7%. The locus ETRC had the highest allele diversity (h: 0.45). The findings of this study suggest that there is high genetic diversity among M. tuberculosis isolates in Khorasan

Razavi Province, which is consistent with similar results from other studies in other provinces in Iran and neighboring countries. This indicates that the prevalent genotypes in this study are spreading in the Middle East region. Furthermore, considering that M. Bovis isolates were identified in two clusters, it seems that all of them have a common origin and are circulating among the livestock farms in the province.

© 2023. The Author(s).

DOI: 10.1038/s41598-023-33740-9
PMCID: PMC10127167
PMID: 37185604 [Indexed for MEDLINE]

Conflict of interest statement: The authors declare no competing interests.

170. BMC Public Health. 2023 Apr 25;23(1):761. doi: 10.1186/s12889-023-15555-5.

COVID-19 and the law in Uganda: a case study on development and application of the public health act from 2020 to 2021.

Achan MI(1), Nabukenya I(2), Mitanda S(3), Nakacwa J(3), Bakiika H(1), Nabatanzi M(1), Bukirwa J(1), Nakanwagi A(1), Nakiire L(1), Aperce C(4), Schwid A(5), Okware S(6), Obuku EA(1), Lamorde M(1), Luswata B(7), Makumbi I(7), Muruta A(7), Mwebesa HG(7), Aceng Ocero JR(7).

Author information:
(1)Infectious Diseases Institute, Makerere University, P.O. Box 22418, Kampala, Uganda.
(2)Infectious Diseases Institute, Makerere University, P.O. Box 22418, Kampala, Uganda. inabukenya@idi.co.ug.
(3)Ministry of Justice and Constitutional Affairs, P.O Box 7183, Kampala, Uganda.
(4)Resolve to Save Lives, New York 100 Broadway, 4th Floor, New York, NY, 10005, USA.
(5)Vital Strategies, New York 100 Broadway, 4th Floor, New York, NY, 10005, USA.
(6)World Health Organization, Uganda Country Office, P. O. Box 24578, Kampala, Uganda.
(7)Ministry of Health, P.O. Box 7272, Kampala, Uganda.

BACKGROUND: Despite the discovery of vaccines, the control, and prevention of Coronavirus disease 2019 (COVID-19) relied on non-pharmaceutical interventions (NPIs). This article describes the development and application of the Public Health Act to implement NPIs for COVID-19 pandemic control in Uganda.
METHODS: This is a case study of Uganda's experience with enacting COVID-19 Rules under the Public Health Act Cap. 281. The study assessed how and what Rules were developed, their influence on the outbreak progress, and litigation.

The data sources reviewed were applicable laws and policies, Presidential speeches, Cabinet resolutions, statutory instruments, COVID-19 situation reports, and the registry of court cases that contributed to a triangulated analysis.
RESULTS: Uganda applied four COVID-19 broad Rules for the period March 2020 to October 2021. The Minister of Health enacted the Rules, which response teams, enforcement agencies, and the general population followed. The Presidential speeches, their expiry period and progress of the pandemic curve led to amendment of the Rules twenty one (21) times. The Uganda Peoples Defense Forces Act No. 7 of 2005, the Public Finance Management Act No. 3 of 2015, and the National Policy for Disaster Preparedness and Management supplemented the enacted COVID-19 Rules. However, these Rules attracted specific litigation due to perceived infringement on certain human rights provisions.
CONCLUSIONS: Countries can enact supportive legislation within the course of an outbreak. The balance of enforcing public health interventions and human rights infringements is an important consideration in future. We recommend public sensitization about legislative provisions and reforms to guide public health responses in future outbreaks or pandemics.

© 2023. The Author(s).

DOI: 10.1186/s12889-023-15555-5
PMCID: PMC10126532
PMID: 37098568 [Indexed for MEDLINE]

Conflict of interest statement: The authors declare no competing interests as defined by BMC, or other interests that might be perceived to influence the results and/or discussion reported in this paper.

171. iScience. 2023 Mar 21;26(4):106454. doi: 10.1016/j.isci.2023.106454. eCollection 2023 Apr 21.

Upregulation of IFN-stimulated genes persists beyond the transitory broad immunologic changes of acute HIV-1 infection.

Mackelprang RD(1)(2), Filali-Mouhim A(3), Richardson B(4), Lefebvre F(5), Katabira E(6), Ronald A(7), Gray G(8), Cohen KW(9), Klatt NR(10), Pecor T(11), Celum C(2)(12)(13), McElrath MJ(9), Hughes SM(1), Hladik F(1)(9)(13), Cameron MJ(4), Lingappa JR(2)(13)(14); Partners in Prevention HSV/HIV Transmission Study; Partners PrEP Study Teams.

Author information:
(1)Department of Obstetrics and Gynecology, University of Washington, Seattle, WA, USA.
(2)Department of Global Health, University of Washington, Seattle, WA, USA.
(3)University of Montreal Hospital Research Center, Laval, QC, Canada.

(4)Department of Population and Quantitative Health Sciences, School of Medicine, Case Western Reserve University, Cleveland, OH, USA.
(5)Canadian Centre for Computational Genomics, McGill University, Montreal, QC, Canada.
(6)Infectious Disease Institute, Makerere University, Kampala, Uganda.
(7)Canada Departments of Medical Microbiology and Internal Medicine, University of Manitoba, Winnipeg, MB, USA.
(8)Perinatal HIV Research Unit, University of the Witwatersrand, Johannesburg, South Africa.
(9)Vaccine and Infectious Disease Division, Fred Hutchinson Cancer Research Center, Seattle, WA, USA.
(10)Department of Surgery, University of Minnesota Medical School, Minneapolis, MN, USA.
(11)Washington National Primate Research Center, Seattle, WA, USA.
(12)Department of Epidemiology, University of Washington, Seattle, WA, USA.
(13)Department of Medicine, University of Washington School of Medicine, Seattle, WA, USA.
(14)Department of Pediatrics, University of Washington, Seattle, WA, USA.

Chronic immune activation during HIV-1 infection contributes to morbidity and mortality in people living with HIV. To elucidate the underlying biological pathways, we evaluated whole blood gene expression trajectories from before, through acute, and into chronic HIV-1 infection. Interferon-stimulated genes, including MX1, IFI27 and ISG15, were upregulated during acute infection, remained elevated into chronic infection, and were strongly correlated with plasma HIV-1 RNA as well as TNF-α and CXCL10 cytokine levels. In contrast, genes involved in cellular immune responses, such as CD8A, were upregulated during acute infection before reaching a peak and returning to near pre-infection levels in chronic infection. Our results indicate that chronic immune activation during HIV-1 infection is characterized by persistent elevation of a narrow set of interferon-stimulated genes and innate cytokines. These findings raise the prospect of devising a targeted intervention to restore healthy immune homeostasis in people living with HIV-1.

© 2023 The Authors.

DOI: 10.1016/j.isci.2023.106454
PMCID: PMC10067744
PMID: 37020953

Conflict of interest statement: The authors declare no competing interests.

172. Pediatric Health Med Ther. 2023 Apr 20;14:131-139. doi: 10.2147/PHMT.S404620. eCollection 2023.

Parents' Readiness to Vaccinate Their Children Aged 5 to 17 Years Against

Covid-19 and Its Associated Factors in Lira District, Uganda.

Puleh SS(1), Kigongo E(2), Opio IO(1), Akech SI(1), Opollo MS(3), Achan E(3), Acen J(4), Anyolitho MK(3), Acup W(5), Kabunga A(6).

Author information:
(1)Department of Epidemiology and Biostatistics, Lira University, Lira City, Uganda.
(2)Department of Environmental Health and Disease Control, Lira University, Lira City, Uganda.
(3)Department of Community Health, Lira University, Lira City, Uganda.
(4)Department of Midwifery, Lira University, Lira City, Uganda.
(5)Department of Public Health, Gulu University, Gulu City, Uganda.
(6)Department of Psychiatry, Lira University, Lira City, Uganda.

BACKGROUND: Data on parents' readiness to vaccinate their children aged 5 to 17 years against COVID-19 is still scarce. This study assessed parents' readiness to vaccinate their children aged 5 to 17 years against COVID-19 and factors associated in Lira district, Uganda.
METHODS AND MATERIALS: A cross-sectional survey employing quantitative methods was conducted between October and November 2022 among 578 parents of children aged 5-17 years in 3 sub-counties in Lira district. An interviewer-administered questionnaire was used to collect data. Data was analyzed using descriptive statistics including means, percentages, frequencies, and odds ratios. Logistic regression was used to determine associations between the factors and the readiness of parents at a 95% level of significance.
RESULTS: Out of 634 participants, 578 responded to the questionnaire, giving a response rate of 91.2%. The majority of the parents (327, 56.8%) were female, had children aged between 12 and 15 years (266, 46.4%), and had completed primary education (351, 60.9%). Most of the parents were Christian (565, 98.4%), married (499, 86.6%), and had been vaccinated against COVID-19 (535, 92.6%). Results also indicated that 75.6% (ranging from 71.9% to 78.9%) of the parents were unwilling to vaccinate their children for the COVID-19 virus. The predictors of readiness were the age of the child (AOR: 2.02; 95% CI: 0.97-4.20; p=0.05) and lack of trust in the vaccine (AOR: 3.33; 95% CI: 1.95-5.71; p0.001).
CONCLUSION: Our study shows that parents' readiness to vaccinate their children aged 5 to 17 years was only 24.6%, which is suboptimal. The predictors of hesitancy were the age of the child and a lack of trust in the vaccine. Based on our results, the Ugandan authorities should provide health education interventions targeting parents to combat mistrust with respect to COVID-19 and the COVID-19 vaccine and highlight the benefits of the vaccines.

© 2023 Puleh et al.

DOI: 10.2147/PHMT.S404620
PMCID: PMC10124741
PMID: 37101835

Conflict of interest statement: The authors declare no conflict of interest.

173. Virus Evol. 2023 Apr 15;9(1):vead025. doi: 10.1093/ve/vead025. eCollection 2023.

Genomic epidemiology of the rotavirus G2P[4] strains in coastal Kenya pre- and post-rotavirus vaccine introduction, 2012-8.

Makori TO(1)(2), Bargul JL(2)(3), Lambisia AW(1), Mwanga MJ(1), Murunga N(1), de Laurent ZR(1), Lewa CS(1), Mutunga M(1), Kellam P(4)(5), Cotten M(6)(7), Nokes DJ(1)(8), Phan M(6)(7), Agoti CN(1)(9).

Author information:
(1)Epidemiology and Demography Department Kenya Medical Research Institute (KEMRI)-Wellcome Trust Research Programme, Off Hospital Road, P.O BOX 230-80108, Kilifi, Kenya.
(2)Department of Biochemistry, Jomo Kenyatta University of Agriculture and Technology, Kalimoni, PO Box 62000-00200, Juja, Kenya.
(3)International Centre of Insect Physiology and Ecology, Animal Health Theme, ICIPE Road Kasarani, P.O BOX 30772-00100, Nairobi, Kenya.
(4)Department of Infectious Diseases, Faculty of Medicine, Imperial College London, Exhibition Road, London SW7 2AZ, UK.
(5)Kymab Ltd, The Bennet Building (B930), Babraham Research Campus, Cambridge CB22 3AT, UK.
(6)Medical Research Centre (MRC)/Uganda Virus Research Institute, Plot No: 51-59 Nakiwogo Road, P.O.Box 49, Entebbe, Uganda.
(7)MRC-University of Glasgow, Centre for Virus Research Glasgow, 464 Bearsden Road, Glasgow G61 1QH UK.
(8)School of Life Sciences and Zeeman Institute (SBIDER), The University of Warwick, Gibbet Hill Campus, Coventry CV4 7AL, UK.
(9)School of Health and Human Sciences, Pwani University, Kilifi-Malindi Road, P.O BOX 195-80108, Kilifi, Kenya.

The introduction of rotavirus vaccines into the national immunization programme in many countries has led to a decline in childhood diarrhoea disease burden. Coincidentally, the incidence of some rotavirus group A (RVA) genotypes has increased, which may result from non-vaccine-type replacement. Here, we investigate the evolutionary genomics of rotavirus G2P[4] which has shown an increase in countries that introduced the monovalent Rotarix® vaccine. We examined sixty-three RVA G2P[4] strains sampled from children (aged below 13 years) admitted to Kilifi County Hospital, coastal Kenya, pre- (2012 to June 2014) and post-(July 2014 to 2018) rotavirus vaccine introduction. All the sixty-three genome sequences showed a typical DS-1-like genome constellation (G2-P[4]-I2-R2-C2-M2-A2-N2-T2-E2-H2). Pre-vaccine G2 sequences predominantly classified as sub-lineage IVa-3 and co-circulated with low numbers of sub-lineage IVa-1 strains, whereas post-vaccine G2 sequences mainly classified

into sub-lineage IVa-3. In addition, in the pre-vaccine period, P[4] sub-lineage IVa strains co-circulated with low numbers of P[4] lineage II strains, but P[4] sub-lineage IVa strains predominated in the post-vaccine period. On the global phylogeny, the Kenyan pre- and post-vaccine G2P[4] strains clustered separately, suggesting that different virus populations circulated in the two periods. However, the strains from both periods exhibited conserved amino acid changes in the known antigenic epitopes, suggesting that replacement of the predominant G2P[4] cluster was unlikely a result of immune escape. Our findings demonstrate that the pre- and post-vaccine G2P[4] strains circulating in Kilifi, coastal Kenya, differed genetically but likely were antigenically similar. This information informs the discussion on the consequences of rotavirus vaccination on rotavirus diversity.

© The Author(s) 2023. Published by Oxford University Press.

DOI: 10.1093/ve/vead025
PMCID: PMC10190042
PMID: 37207000

Conflict of interest statement: None declared.

174. Arch Virol. 2023 Apr 15;168(5):140. doi: 10.1007/s00705-023-05759-w.

Environmental surveillance detects circulating vaccine-derived poliovirus type 2 that was undetected by acute flaccid paralysis surveillance in 2021 in Uganda.

Tushabe P(1), Bwogi J(2), Eliku JP(2), Aine F(2), Birungi M(2), Gaizi J(2), Nakabazzi L(2), Kabaliisa T(2), Turyahabwe I(2), Namuwulya P(2), Nanteza MB(2), Bukenya H(2), Kanyesigye C(3), Katushabe E(4), Ampeire I(5), Kisakye A(4), Bakamutumaho B(2), Byabamazima CR(6).

Author information:
(1)Expanded Programme on Immunization Laboratory, Uganda Virus Research Institute, Entebbe, Uganda. ptushabe@uvri.go.ug.
(2)Expanded Programme on Immunization Laboratory, Uganda Virus Research Institute, Entebbe, Uganda.
(3)National Water and Sewerage Corporation, Kampala, Uganda.
(4)World Health Organization, Uganda Country Office, Kampala, Uganda.
(5)Ministry of Health Uganda, Kampala, Uganda.
(6)WHO Inter-Country Support Team Office for Eastern and Southern Africa (IST/ESA), Harare, Zimbabwe.

The success of the global polio eradication initiative is threatened by the genetic instability of the oral polio vaccine, which can result in the emergence of pathogenic vaccine-derived polioviruses following prolonged replication in the guts of individuals with primary immune deficiencies or in communities with

low vaccination coverage. Through environmental surveillance, circulating vaccine-derived poliovirus type 2 was detected in Uganda in the absence of detection by acute flaccid paralysis (AFP) surveillance. This underscores the sensitivity of environmental surveillance and emphasizes its usefulness in supplementing AFP surveillance for poliovirus infections in the race towards global polio eradication.

© 2023. The Author(s).

DOI: 10.1007/s00705-023-05759-w
PMCID: PMC10104764
PMID: 37059887 [Indexed for MEDLINE]

Conflict of interest statement: The authors have no relevant financial or non-financial interests to declare.

175. Int J Womens Health. 2023 Apr 14;15:611-620. doi: 10.2147/IJWH.S404845. eCollection 2023.

Knowledge, Attitude, and Practice Toward Cervical Cancer Screening Among Female University Students in Ishaka Western Uganda.

Usman IM(1), Chama N(2), Aigbogun EO Jr(3), Kabanyoro A(4), Kasozi KI(5), Usman CO(6), Fernandez Diaz ME(1), Ndyamuhakyi E(1), Archibong VB(7), Onongha C(4), Ochieng JJ(1), Kanee RB(8), Ssebuufu R(9).

Author information:
(1)Department of Human Anatomy, Kampala International University Western Campus, Bushenyi, Uganda.
(2)Department of Surgery, Kampala International University Teaching Hospital, Ishaka, Uganda.
(3)Department of Human Anatomy, Enugu State University of Science and Technology, Enugu, Nigeria.
(4)Department of Nursing, Aga Khan University, Kampala, Uganda.
(5)Physiology Department, Kabale University, Kabale, Uganda.
(6)Nursing Department, Ahmadu Bello University Teaching Hospital, Tudun Wada, Nigeria.
(7)Department of Human Anatomy, University of Rwanda, Kigali, Rwanda.
(8)Institute of Geo-Science and Space Technology, Rivers State University, Port Harcourt, Nigeria.
(9)Department of Surgery, Mengo Hospital, Kampala, Uganda.

PURPOSE: Cervical cancer (CC) is the leading cause of cancer-related death among women living in third-world countries. CC is preventable, with the possibility of complete treatment if detected early. The objective of the study was to assess the level of knowledge, attitude, and practice on CC screening (CCS)

among female university students (FUS) in Ishaka, western Uganda.
METHODS: The study was a descriptive cross-sectional study among FUS (n = 407) in western Uganda conducted after the first nationwide lockdown in Uganda. Information was collected using a questionnaire and descriptively presented as frequency and percentages.
RESULTS: The majority of our respondents were medical students 283 (69.5%), below 25 years 339 (83.3%), with Anglican Christian religious background 150 (36.9%). Respondents above the age of 25 years ($p = 0.0052$) and those in the medical profession ($p < 0.001$) had more knowledge. More medical students had a better attitude ($p = 0.0043$) and favorable practices (0.0134) compared to their non-medical counterparts. There is a weak correlation between attitude ($r = 0.206$, $p < 0.001$) and practice ($r = 0.181$, $p = 0.0003$) with knowledge on CCS.
CONCLUSION: Observation from the present study suggests the need for more efforts in the fight against cervical cancer and encouragement of positive attitude and practice towards cervical cancer screening and uptake of vaccination.

© 2023 Usman et al.

DOI: 10.2147/IJWH.S404845
PMCID: PMC10112480
PMID: 37082233

Conflict of interest statement: The authors report no conflicts of interest in this work.

176. PLoS One. 2023 Apr 12;18(4):e0284362. doi: 10.1371/journal.pone.0284362. eCollection 2023.

Perceptions about COVID-19 preventive measures among Ghanaian women.

Kyei-Arthur F(1), Agyekum MW(2), Afrifa-Anane GF(1), Larbi RT(3), Kisaakye P(4).

Author information:
(1)Department of Environment and Public Health, University of Environment and Sustainable Development, Somanya, Ghana.
(2)Institute for Educational Research and Innovation Studies, University of Education Winneba, Winneba, Ghana.
(3)Regional Institute for Population Studies, University of Ghana, Legon, Ghana.
(4)Department of Population Studies, Makerere University, Kampala, Uganda.

Though the advent of COVID-19 vaccines has significantly reduced severe morbidity and mortality, infection rates continue to rise. Therefore, adhering to COVID-19 preventive measures remains essential in the fight against the pandemic, particularly in Africa, where vaccination rates remain low. However, the perceived risk associated with COVID-19 and public education and awareness

campaigns has waned over time. COVID-19 vaccine hesitancy is consistently high among women globally. This study, therefore, assessed the facilitators, and barriers to adherence to COVID-19 preventive measures. A qualitative descriptive study was conducted among Ghanaian women. Twenty-seven in-depth interviews were conducted with women in the Greater Accra and Ashanti regions. All interviews were audio-recorded and transcribed verbatim into English. The data were analysed using NVivo 10 software. While some participants found the use of face masks as the easiest, others found it as the most difficult. In addition, institutional and policy decisions such as access to water and the use of public transport impacted individual level adherence to preventive measures. In conclusion, the fight against COVID-19 is not over; hence public education and the provision of facilities that would enhance compliance with preventive measures should continue to be prioritised.

Copyright: © 2023 Kyei-Arthur et al. This is an open access article distributed under the terms of the Creative Commons Attribution License, which permits unrestricted use, distribution, and reproduction in any medium, provided the original author and source are credited.

DOI: 10.1371/journal.pone.0284362
PMCID: PMC10096443
PMID: 37043460 [Indexed for MEDLINE]

Conflict of interest statement: The authors have declared that no competing interests exist.

177. Vaccines (Basel). 2023 Apr 7;11(4):814. doi: 10.3390/vaccines11040814.

Evaluation of Container Clinics as an Urban Immunization Strategy: Findings from the First Year of Implementation in Ghana, 2017-2018.

Shaum A(1), Wardle MT(1), Amponsa-Achiano K(2), Aborigo R(3), Opare J(4), Wallace AS(1), Bandoh D(5), Quaye P(2), Osei-Sarpong F(2), Abotsi F(2), Bonsu G(2), Conklin L(1).

Author information:
(1)Center for Global Health, Global Immunization Division, Centers for Disease Control and Prevention, Atlanta, GA 30329, USA.
(2)Department of Disease Control and Prevention, Public Health Division, Ghana Health Service, Accra 00233, Ghana.
(3)Navrongo Health Research Centre, Health Research Division, Ghana Health Service, Navrongo 03821, Ghana.
(4)African Field Epidemiology Network, Kampala 10102, Uganda.
(5)Ghana Field Epidemiology and Laboratory Training Programme, School of Public Health, University of Ghana, Accra 00233, Ghana.

BACKGROUND: In 2017, the Expanded Programme on Immunization in Ghana opened two container clinics in Accra, which were cargo containers outfitted to deliver immunizations. At each clinic, we assessed performance and clinic acceptance during the first 12 months of implementation.
METHODS: We employed a descriptive mixed-method design using monthly administrative immunization data, exit interviews with caregivers of children of <5 years (N = 107), focus group discussions (FGDs) with caregivers (n = 6 FGDs) and nurses (n = 2 FGDs), and in-depth interviews (IDIs) with community leaders (n = 3) and health authorities (n = 3).
RESULTS: Monthly administrative data showed that administered vaccine doses increased from 94 during the opening month to 376 in the 12th month across both clinics. Each clinic exceeded its target doses for the 12-23 month population (second dose of measles). Almost all (98%) exit interview participants stated that the clinics made it easier to receive child health services compared to previous health service interactions. The accessibility and acceptability of the container clinics were also supported from health worker and community perspectives.
CONCLUSIONS: Our initial data support container clinics as an acceptable strategy for delivering immunization services in urban populations, at least in the short term. They can be rapidly deployed and designed to serve working mothers in strategic areas.

DOI: 10.3390/vaccines11040814
PMCID: PMC10143135
PMID: 37112727

Conflict of interest statement: The authors declare no conflict of interest. The findings and conclusions in this report are those of the authors and do not necessarily represent the views of the Centers for Disease Control and Prevention.

178. Adolesc Health Med Ther. 2023 Apr 5;14:87-96. doi: 10.2147/AHMT.S397734. eCollection 2023.

Factors Associated with the Human Papillomavirus Vaccine Coverage in Gulu District, Uganda.

Odongo Ojok I(1), Ogwal JB(1), Wwesige B(1), Bongomin F(2), Akello F(3).

Author information:
(1)Faculty of Medicine, Gulu University, Gulu, Uganda.
(2)Department of Medical Microbiology and Immunology, Faculty of Medicine, Gulu University, Gulu, Uganda.
(3)Department of Pediatrics and Child Health, Faculty of Medicine, Gulu University, Gulu, Uganda.

BACKGROUND: Human papillomavirus (HPV) vaccination is an effective public health measure for the prevention of cancer of the cervix. We aimed to assess HPV vaccine coverage and associated factors in Gulu, Uganda.
METHODS: In October 2021, we conducted a cross-sectional study among girls aged 9 to 13 years residing in Pece-Laroo Division, Gulu City, Uganda. HPV vaccine coverage was defined as receipt of at least a dose of the HPV vaccine.
RESULTS: A total of 197 girls, with a mean age of 11±1.4 years, were enrolled. Most participants were of the Acholi tribe (89.3%, n=176), catholic (58.4%, n=115), and in primary 5 level of education (36%, n=71). Overall, 68(35%) participants had received the HPV vaccine. Factors associated with HPV vaccine utilization were, good knowledge of the HPV vaccine (adjusted odds ratio (aOR) =0.233, 95% confidence interval (95CI): 0.037-0.640, p=0.101), methods of HPV prevention (OR=0.320, 95CI: 0.112-0.914, p = 0.033), good knowledge of the importance of HPV vaccination (OR=0.458, 95% CI: 0.334-0.960, p=0.021), knowledge on frequency of HPV vaccination (OR=0.423, 95CI:0.173-0.733, p=0.059), and good mobilization (OR=0.443, 95% CI: 0.023-0.923, p=0.012).
CONCLUSION: In this community-based study, only one third of eligible girls received the HPV vaccine. Public health interventions are recommended exponentially to enhance the utilization of the HPV vaccine in this community.

© 2023 Odongo Ojok et al.

DOI: 10.2147/AHMT.S397734
PMCID: PMC10083017
PMID: 37041756

Conflict of interest statement: Felix Bongomin and Franceska Akello are co-senior authors for this study. The authors report no conflicts of interest in this work.

179. Trials. 2023 Apr 5;24(1):257. doi: 10.1186/s13063-023-07274-4.

Dihydroartemisinin-piperaquine or sulphadoxine-pyrimethamine for the chemoprevention of malaria in children with sickle cell anaemia in eastern and southern Africa (CHEMCHA): a protocol for a multi-centre, two-arm, double-blind, randomised, placebo-controlled superiority trial.

Nkosi-Gondwe T(1), Robberstad B(2), Opoka R(3), Kalibbala D(4), Rujumba J(3), Galileya LT(1), Akun P(3), Nambatya W(3), Ssenkusu J(3), TerKuile F(5), Phiri K(1), Idro R(6).

Author information:
(1)Training and Research Unit of Excellence, Blantyre, Malawi.
(2)University of Bergen, Bergen, Norway.
(3)Makerere University College of Health Sciences, Kampala, Uganda.
(4)Global Health Uganda, Kampala, Uganda.

(5)Liverpool School of Tropical Medicine, Liverpool, UK.
(6)Makerere University College of Health Sciences, Kampala, Uganda.
ridro1@gmail.com.

BACKGROUND: An estimated 300,000 babies are born with sickle cell anaemia (SCA) annually. Affected children have chronic ill health and suffer premature death. Febrile illnesses such as malaria commonly precipitate acute crises in children with SCA. Thus, chemoprophylaxis for malaria is an important preventive strategy, but current regimes are either sub-optimally effective (e.g. monthly sulphadoxine-pyrimethamine, SP) or difficult to adhere to (e.g. daily proguanil). We propose dihydroartemisinin-piperaquine (DP) as the agent with the most potential to be used across Africa.
METHODS: This will be a randomised, double-blind, parallel-group superiority trial of weekly single-day courses of DP compared to monthly single-day courses of SP in children with SCA. The study will be conducted in eastern (Uganda) and southern (Malawi) Africa using randomisation stratified by body weight and study centre. Participants will be randomised using an allocation of 1:1 to DP or SP. We will investigate the efficacy, safety, acceptability and uptake and cost-effectiveness of malaria chemoprevention with weekly courses of DP vs monthly SP in 548 to 824 children with SCA followed up for 12-18 months. We will also assess toxicity from cumulative DP dosing and the development of resistance. Participant recruitment commenced on 30 April 2021; follow-up is ongoing.
DISCUSSION: At the end of this study, findings will be used to inform regional health policy. This manuscript is prepared from protocol version 2.1 dated 1 January 2022.
TRIAL REGISTRATION: The trial was registered at ClinicalTrials.gov, NCT04844099 . Registered on 08 April 2021.

© 2023. The Author(s).

DOI: 10.1186/s13063-023-07274-4
PMCID: PMC10074896
PMID: 37016392 [Indexed for MEDLINE]

Conflict of interest statement: The authors declare that they have no competing interests.

180. Sci Rep. 2023 Apr 4;13(1):5516. doi: 10.1038/s41598-023-30667-z.

Phylogenomic analysis uncovers a 9-year variation of Uganda influenza type-A strains from the WHO-recommended vaccines and other Africa strains.

Nabakooza G(1)(2)(3)(4), Owuor DC(5), de Laurent ZR(5), Galiwango R(6)(7)(8), Owor N(9), Kayiwa JT(9), Jjingo D(8)(10), Agoti CN(5), Nokes DJ(5)(11), Kateete DP(12)(6), Kitayimbwa JM(6)(7), Frost SDW(#)(13)(14), Lutwama JJ(#)(9).

Author information:
(1)Department of Immunology and Molecular Biology, Makerere University, Kampala, Uganda. Grace.Nabakooza@mrcuganda.org.
(2)Makerere University/UVRI Centre of Excellence in Infection and Immunity Research and Training (MUII-Plus), Uganda Virus Research Institute (UVRI), Entebbe, Uganda. Grace.Nabakooza@mrcuganda.org.
(3)Centre for Computational Biology, Uganda Christian University, Mukono, Uganda. Grace.Nabakooza@mrcuganda.org.
(4)Oak Ridge Institute for Science and Education, Bioinformatics Research Fellow to the Division of Viral Diseases, Centers for Disease Control and Prevention, Atlanta, Georgia, United States. Grace.Nabakooza@mrcuganda.org.
(5)Epidemiology and Demography Department, KEMRI-Wellcome Trust Research Programme, Kilifi, Kenya.
(6)Makerere University/UVRI Centre of Excellence in Infection and Immunity Research and Training (MUII-Plus), Uganda Virus Research Institute (UVRI), Entebbe, Uganda.
(7)Centre for Computational Biology, Uganda Christian University, Mukono, Uganda.
(8)The African Center of Excellence in Bioinformatics and Data Intensive Sciences (ACE), Infectious Diseases Institute, Makerere University, Kampala, Uganda.
(9)Department of Arbovirology Emerging and Re-Emerging Infectious Diseases, Uganda Virus Research Institute (UVRI), Entebbe, Uganda.
(10)Department of Computer Science, College of Computing, Makerere University, Kampala, Uganda.
(11)School of Life Sciences and Zeeman Institute for Systems Biology and Infectious Disease Epidemiology Research (SBIDER), University of Warwick, Coventry, United Kingdom.
(12)Department of Immunology and Molecular Biology, Makerere University, Kampala, Uganda.
(13)Microsoft Research, Redmond, Washington, 98052, United States.
(14)London School of Hygiene and Tropical Medicine (LSHTM), Keppel St, Bloomsbury, London, United Kingdom.
(#)Contributed equally

Genetic characterisation of circulating influenza viruses directs annual vaccine strain selection and mitigation of infection spread. We used next-generation sequencing to locally generate whole genomes from 116 A(H1N1)pdm09 and 118 A(H3N2) positive patient swabs collected across Uganda between 2010 and 2018. We recovered sequences from 92% (215/234) of the swabs, 90% (193/215) of which were whole genomes. The newly-generated sequences were genetically and phylogenetically compared to the WHO-recommended vaccines and other Africa strains sampled since 1994. Uganda strain hemagglutinin (n = 206), neuraminidase (n = 207), and matrix protein (MP, n = 213) sequences had 95.23-99.65%, 95.31-99.79%, and 95.46-100% amino acid similarity to the 2010-2020 season vaccines, respectively, with several mutated hemagglutinin antigenic, receptor

binding, and N-linked glycosylation sites. Uganda influenza type-A virus strains sequenced before 2016 clustered uniquely while later strains mixed with other Africa and global strains. We are the first to report novel A(H1N1)pdm09 subclades 6B.1A.3, 6B.1A.5(a,b), and 6B.1A.6 (± T120A) that circulated in Eastern, Western, and Southern Africa in 2017-2019. Africa forms part of the global influenza ecology with high viral genetic diversity, progressive antigenic drift, and local transmissions. For a continent with inadequate health resources and where social distancing is unsustainable, vaccination is the best option. Hence, African stakeholders should prioritise routine genome sequencing and analysis to direct vaccine selection and virus control.

© 2023. The Author(s).

DOI: 10.1038/s41598-023-30667-z
PMCID: PMC10072032
PMID: 37015946 [Indexed for MEDLINE]

Conflict of interest statement: The authors declare no competing interests.

181. Nat Commun. 2023 Apr 3;14(1):1863. doi: 10.1038/s41467-023-37502-z.

Pulmonary inflammation promoted by type-2 dendritic cells is a feature of human and murine schistosomiasis.

Houlder EL(1)(2), Costain AH(1)(2), Nambuya I(1)(3), Brown SL(1), Koopman JPR(2), Langenberg MCC(2), Janse JJ(2), Hoogerwerf MA(2), Ridley AJL(1), Forde-Thomas JE(4), Colombo SAP(1), Winkel BMF(2), Galdon AA(1), Hoffmann KF(4), Cook PC(1)(5), Roestenberg M(2), Mpairwe H(3), MacDonald AS(6).

Author information:
(1)Lydia Becker Institute of Immunology and Inflammation, University of Manchester, Manchester, UK.
(2)Leiden University Center for Infectious Diseases (LU-CID), Leiden University Medical Centre, Leiden, Netherlands.
(3)MRC/UVRI and LSHTM Uganda Research Unit, Entebbe, Uganda.
(4)Department of Life Sciences, Aberystwyth University, Aberystwyth, SY23 3DA, UK.
(5)MRC Centre for Medical Mycology, University of Exeter, Exeter, UK.
(6)Lydia Becker Institute of Immunology and Inflammation, University of Manchester, Manchester, UK. Andrew.MacDonald@manchester.ac.uk.

Schistosomiasis is a parasitic disease affecting over 200 million people in multiple organs, including the lungs. Despite this, there is little understanding of pulmonary immune responses during schistosomiasis. Here, we show type-2 dominated lung immune responses in both patent (egg producing) and pre-patent (larval lung migration) murine Schistosoma mansoni (S. mansoni)

infection. Human pre-patent S. mansoni infection pulmonary (sputum) samples
revealed a mixed type-1/type-2 inflammatory cytokine profile, whilst a
case-control study showed no significant pulmonary cytokine changes in endemic
patent infection. However, schistosomiasis induced expansion of pulmonary type-2
conventional dendritic cells (cDC2s) in human and murine hosts, at both
infection stages. Further, cDC2s were required for type-2 pulmonary inflammation
in murine pre-patent or patent infection. These data elevate our fundamental
understanding of pulmonary immune responses during schistosomiasis, which may be
important for future vaccine design, as well as for understanding links between
schistosomiasis and other lung diseases.

© 2023. The Author(s).

DOI: 10.1038/s41467-023-37502-z
PMCID: PMC10070318
PMID: 37012228 [Indexed for MEDLINE]

Conflict of interest statement: The Manchester Collaborative Centre for
Inflammation Research is a joint venture between the University of Manchester
and GSK. The authors working at the MCCIR (A.S.M., E.L.H., A.H.C., S.L.B.,
A.J.L.R., P.C.C., A.A.G., S.A.C.) declare that the research was conducted in the
absence of any commercial or financial relationships that could be construed as
a potential conflict of interest. The remaining authors declare no competing
interests.

182. Lancet Reg Health Eur. 2023 Jan 13;27:100581. doi: 10.1016/j.lanepe.2022.100581.
eCollection 2023 Apr.

Malaria parasite prevalence in Sub-Saharan African migrants screened in Sweden:
a cross-sectional study.

Wångdahl A(1)(2), Bogale RT(1), Eliasson I(1), Broumou I(1), Faroogh F(1), Lind
F(1), Vashchuk G(1), Hildell A(1), Franson S(1), Hallberg E(1), Grip I(1),
Nordling I(3), Gervin A(4), Kaitoly S(4), Tekleab B(4), Wyss K(1)(3),
Requena-Méndez A(1)(3)(5), Hertting O(6)(7), Färnert A(1)(3).

Author information:
(1)Division of Infectious Diseases, Department of Medicine Solna, Karolinska
Institutet, Stockholm, Sweden.
(2)Department of Infectious Diseases, Västerås Hospital, Västerås, Sweden.
(3)Department of Infectious Diseases, Karolinska University Hospital, Stockholm,
Sweden.
(4)Rissne Vårdcentral, Migration Health Centre, Region Stockholm, Stockholm,
Sweden.
(5)Barcelona Institute for Global Health (ISGlobal, University of Barcelona),
Spain.

(6)Paediatric Infectious Diseases, Astrid Lindgren Children's Hospital, Karolinska University Hospital, Stockholm, Sweden.
(7)Department of Women's and Children's Health, Karolinska Institutet, Stockholm, Sweden.

BACKGROUND: Asymptomatic infections with malaria parasites are common in populations in endemic areas. These infections may persist in migrants after arrival in a non-endemic area. Screening to find and clear these infections is generally not implemented in non-endemic countries, despite a potential negative health impact. We performed a study to evaluate the Plasmodium parasite prevalence in migrants living in Sweden.
METHODS: Adults and children born in Sub-Saharan Africa (SSA) were invited in the study between April 2019 and June 2022 at 10 different sites, mainly as part of the national Migrant Health Assessment Program in Stockholm and Västerås, Sweden. Rapid diagnostic tests (RDT) and real-time PCR were used to detect malaria parasites. Prevalence and test sensitivity were calculated with 95% confidence intervals (CI). Univariate and multivariable logistic regression were used to evaluate associations with PCR positivity.
FINDINGS: In total, 789 individuals were screened for Plasmodium spp. of which 71 (9.0%) were positive by PCR and 18 (2.3%) also by RDT. When performed during the national screening program, 10.4% was PCR positive. A high prevalence was detected in migrants with Uganda as the country of last residence, 53/187 (28.3%), and in this group the prevalence was highest in children, 29/81 (35.8%). Among the PCR positive, 47/71 (66.2%) belonged to families with at least one other member testing positive (odds ratio [OR] 43.4 (95% CI 19.0-98.9), and the time lived in Sweden ranged between 6 and 386 days.
INTERPRETATION: A high malaria parasite prevalence was found in migrants from SSA, particularly in children offered screening in Stockholm, Sweden during the study period. Awareness of asymptomatic malaria infection is needed and screening for malaria in migrants arriving from high endemic countries should be considered.
FUNDING: The Swedish Research Council, Stockholm County Council and Centre for Clinical Research, Västmanland, Sweden.

© 2022 The Authors.

DOI: 10.1016/j.lanepe.2022.100581
PMCID: PMC10105256
PMID: 37069854

Conflict of interest statement: Isabelle Grip was working part time at Svea Vaccine Travel Clinic in Stockholm, Sweden, unrelated to the study. We declare no other competing interests.

183. Nat Hum Behav. 2023 Apr;7(4):529-544. doi: 10.1038/s41562-023-01522-y. Epub 2023 Feb 27.

Changes in preterm birth and stillbirth during COVID-19 lockdowns in 26 countries.

Calvert C(#)(1), Brockway MM(#)(2), Zoega H(#)(3)(4), Miller JE(#)(5)(6), Been JV(7), Amegah AK(8), Racine-Poon A(9), Oskoui SE(1), Abok II(10), Aghaeepour N(11), Akwaowo CD(12)(13), Alshaikh BN(14), Ayede AI(15), Bacchini F(16), Barekatain B(17), Barnes R(18), Bebak K(19), Berard A(20)(21)(22), Bhutta ZA(23)(24), Brook JR(25), Bryan LR(26), Cajachagua-Torres KN(27)(28)(29), Campbell-Yeo M(30), Chu DT(31), Connor KL(32), Cornette L(33), Cortés S(34), Daly M(35), Debauche C(36)(37), Dedeke IOF(38), Einarsdóttir K(4)(39), Engjom H(40), Estrada-Gutierrez G(41), Fantasia I(42), Fiorentino NM(2), Franklin M(43), Fraser A(44), Gachuno OW(45), Gallo LA(46), Gissler M(47)(48)(49), Håberg SE(50), Habibelahi A(51), Häggström J(52), Hookham L(53), Hui L(54), Huicho L(55), Hunter KJ(1), Huq S(56), Kc A(57), Kadambari S(58), Kelishadi R(59), Khalili N(60), Kippen J(19), Le Doare K(61)(62), Llorca J(63)(64), Magee LA(65), Magnus MC(50), Man KKC(66)(67)(68), Mburugu PM(69), Mediratta RP(70), Morris AD(71), Muhajarine N(72), Mulholland RH(1), Bonnard LN(73), Nakibuuka V(74), Nassar N(75), Nyadanu SD(39)(76), Oakley L(50)(77), Oladokun A(78), Olayemi OO(78), Olutekunbi OA(79), Oluwafemi RO(80), Ogunkunle TO(81), Orton C(82), Örtqvist AK(83)(84), Ouma J(85), Oyapero O(86), Palmer KR(87), Pedersen LH(88), Pereira G(50)(89), Pereyra I(90), Philip RK(91), Pruski D(19), Przybylski M(19), Quezada-Pinedo HG(27)(28)(29), Regan AK(92), Rhoda NR(93)(94), Rihs TA(95), Riley T(96), Rocha TAH(97), Rolnik DL(87), Saner C(5)(98)(99), Schneuer FJ(100), Souter VL(101), Stephansson O(83), Sun S(102), Swift EM(103), Szabó M(104), Temmerman M(105), Tooke L(106), Urquia ML(107), von Dadelszen P(65), Wellenius GA(102), Whitehead C(108), Wong ICK(66)(67)(68), Wood R(1)(109), Wróblewska-Seniuk K(110), Yeboah-Antwi K(111), Yilgwan CS(112), Zawiejska A(113), Sheikh A(1), Rodriguez N(2), Burgner D(114)(115), Stock SJ(116), Azad MB(117)(118).

Author information:
(1)Usher Institute, University of Edinburgh, Edinburgh, UK.
(2)Children's Hospital Research Institute of Manitoba, Department of Pediatrics and Child Health, University of Manitoba, Winnipeg, Manitoba, Canada.
(3)School of Population Health, Faculty of Medicine & Health, University of New South Wales Sydney, Sydney, New South Wales, Australia.
(4)Centre of Public Health Sciences, Faculty of Medicine, University of Iceland, Reykjavik, Iceland.
(5)Murdoch Children's Research Institute, Royal Children's Hospital, Parkville, Victoria, Australia.
(6)Department of Paediatrics, University of Melbourne, Parkville, Victoria, Australia.
(7)Division of Neonatology, Department of Paediatrics; Department of Obstetrics and Gynaecology; Department of Public Health; Erasmus MC - Sophia Children's Hospital, University Medical Centre Rotterdam, Rotterdam, the Netherlands.
(8)Public Health Research Group, Department of Biomedical Sciences, University

of Cape Coast, Cape Coast, Ghana.
(9)Novartis, Basel, Switzerland.
(10)Department of Pediatrics, University of Jos/Jos University Teaching Hospital, Jos, Nigeria.
(11)Department of Anesthesiology, Pain, and Perioperative Medicine, Stanford University School of Medicine, Stanford, CA, USA.
(12)Institute of Health Research and Development, University of Uyo Teaching Hospital, Uyo, Nigeria.
(13)College of Health Sciences, University of Uyo, Uyo, Nigeria.
(14)Department of Pediatrics, University of Calgary, Calgary, Alberta, Canada.
(15)Department of Pediatrics, College of Medicine, University of Ibadan and University College Hospital, Ibadan, Nigeria.
(16)Canadian Premature Babies Foundation, Toronto, Ontario, Canada.
(17)Department of Pediatrics, Division of Neonatology, Child Growth and Development Research Center, Isfahan University of Medical Sciences, Isfahan, Iran.
(18)Aridhia Informatics, Glasgow, UK.
(19)Obstetrics and Gynaecology Ward, District Public Hospital in Poznań, Poznań, Poland.
(20)Faculty of Pharmacy, University of Montreal, Montreal, Quebec, Canada.
(21)CHU Ste-Justine, Montreal, Quebec, Canada.
(22)Faculty of Medicine, Université Claude Bernard Lyon 1, Lyon, France.
(23)Center of Excellence in Women Child Health, The Aga Khan University, Karachi, Pakistan.
(24)Centre for Global Child Health, Hospital for Sick Children, Toronto, Ontario, Canada.
(25)Dalla Lana School of Public Health, University of Toronto, Toronto, Ontario, Canada.
(26)Department of Obstetrics & Gynaecology and Child Health, University of The West MonaIndies, Mona, Jamaica.
(27)The Generation R Study Group, Erasmus MC, University Medical Center Rotterdam, Rotterdam, The Netherlands.
(28)The Department of Paediatrics, Erasmus MC, University Medical Center Rotterdam, Rotterdam, The Netherlands.
(29)Centro de Investigación en Salud Materna e Infantil and Centro de Investigación para el Desarrollo Integral y Sostenible, Universidad Peruana Cayetano Heredia, Lima, Peru.
(30)School of Nursing, Dalhousie University and IWK Health, Halifax, Nova Scotia, Canada.
(31)Center for Biomedicine and Community Health, International School, Vietnam National University, Hanoi, Vietnam.
(32)Department of Health Sciences, Carleton University, Ottawa, Ontario, Canada.
(33)AZ St-Jan Bruges-Ostend AV Hospital, Bruges, Belgium.
(34)Department of Public Health, School of Medicine, Advanced Center for Chronic Diseases Diagonal (ACCDIS), Santiago, Chile.
(35)Irish Neonatal Health Alliance, Wicklow, Ireland.
(36)Department of Neonatology, Cliniques Universitaires Saint-Luc, IREC,

UCLouvain, Brussels, Belgium.
(37)CEpiP (Centre d'Epidémiologie Périnatale), Brussels, Belgium.
(38)Department of Paediatrics, Federal Medical Centre, Abeokuta, Nigeria.
(39)Curtin School of Population Health, Curtin University, Perth, Western Australia, Australia.
(40)Department of Health Registry Research and Development, Norwegian Institute of Public Health, Oslo, Norway.
(41)National Institute of Perinatology, Mexico City, Mexico.
(42)Institute for Maternal and Child Health, IRCCS Burlo Garofolo Children's Hospital, Trieste, Italy.
(43)Department of Statistical Sciences and School of the Environment, University of Toronto, Toronto, Ontario, Canada.
(44)Population Health Sciences, Bristol Medical School, University of Bristol, Bristol, UK.
(45)Obstetrics and Gynecology, Medicine, University of Nairobi, Nairobi, Kenya.
(46)School of Biomedical Sciences, The University of Queensland, St. Lucia, Queensland, Australia.
(47)Department of Knowledge Brokers, THL Finnish Institute for Health and Welfare, Helsinki, Finland.
(48)Academic Primary Health Care Centre, Region Stockholm, Stockholm, Sweden.
(49)Department of Molecular Medicine and Surgery, Karolinska Institutet, Stockholm, Sweden.
(50)Centre for Fertility and Health, Norwegian Institute of Public Health, Oslo, Norway.
(51)Neonatology, Neonatal Health Office, Ministry of Health and Medical Education, Tehran, Iran.
(52)Cytel, Waltham, MA, USA.
(53)St. George's University, Makerere University - Johns Hopkins University Research Collaboration, London, UK.
(54)Department of Obstetrics and Gynaecology, University of Melbourne, Melbourne, Victoria, Australia.
(55)Centro de Investigación en Salud Materna e Infantil, Centro de Investigación para el Desarrollo Integral y Sostenible and School of Medicine, Universidad Peruana Cayetano Heredia, Lima, Peru.
(56)Nutrition and Clinical Services Division, ICDDR,B (International Centre for Diarrhoeal Disease Research, Bangladesh), Dhaka, Bangladesh.
(57)Uppsala University, Uppsala, Sweden.
(58)Oxford Vaccine Group, Department of Paediatrics, University of Oxford and the NIHR Oxford Biomedical Research Centre, Oxford, UK.
(59)Child Growth and Development Research Center, Research Institute for Primordial Prevention of Non-Communicable Disease, Isfahan University of Medical Sciences, Isfahan, Iran.
(60)Preventive Medicine and Public Health Research Center, Psychosocial Health Research Institute, Department of Community and Family Medicine, School of Medicine, Iran University of Medical Sciences, Tehran, Iran.
(61)International Centre for Neonatal and Paediatric Infection, St. George's, University of London, London, UK.

(62)Medical Research Council/Uganda Virus Research Institute and London School of Medical Hygiene & Tropical Medicine Uganda Research Unit, Entebbe, Uganda.
(63)Universidad de Cantabria, Santander, Spain.
(64)CIBERESP (Consortium for Biomedical Research in Epidemiology & Public Health, en Epidemiología y Salud Pública), Madrid, Spain.
(65)Institute of Women and Children's Health, School of Life Course and Population Sciences, King's College London, London, UK.
(66)Research Department of Practice and Policy, University College London School of Pharmacy, London, UK.
(67)Department of Pharmacology and Pharmacy, Li Ka Shing Faculty of Medicine, The University of Hong Kong, Hong Kong, Hong Kong.
(68)Laboratory of Data Discovery for Health, Hong Kong Science Park, Hong Kong, Hong Kong.
(69)Department of Child Health and Paediatrics, School of Medicine, Jomo Kenyatta University of Agriculture and Technology, Nairobi, Kenya.
(70)Division of Pediatric Hospital Medicine, Department of Pediatrics, Stanford University School of Medicine, Stanford, CA, USA.
(71)Health Data Research UK, London, UK.
(72)Community Health and Epidemiology, College of Medicine, University of Saskatchewan, Saskatoon, Saskatchewan, Canada.
(73)Melletted a helyem Egyesület, Right(s) Beside You Association, Budapest, Hungary.
(74)Department of Paediatrics, St. Francis Nsambya Hospital, Kampala, Uganda.
(75)Child Population and Translational Health Research, Children's Hospital at Westmead Clinical School, Faculty of Medicine and Health, University of Sydney, Sydney, New South Wales, Australia.
(76)Education, Culture, and Health Opportunities (ECHO) Research Group International, Aflao, Ghana.
(77)Faculty of Epidemiology and Population Health, London School of Hygiene and Tropical Medicine, London, UK.
(78)Department of Obstetrics and Gynaecology, College of Medicine, University of Ibadan and University College Hospital, Ibadan, Nigeria.
(79)Lagos Island Maternity Hospital, Lagos, Nigeria.
(80)Department of Paediatrics and Child Health, Mother and Child Hospital, Akure, Nigeria.
(81)Department of Paediatrics, Dalhatu Araf Specialist Hospital, Lafia, Nigeria.
(82)Swansea University, Swansea, UK.
(83)Clinical Epidemiology Division, Department of Medicine, Solna, Karolinska Institutet, Stockholm, Sweden.
(84)Department of Obstetrics and Gynecology, Visby County Hospital, Visby, Sweden.
(85)Makerere University - Johns Hopkins University Research Collaboration, Kampala, Uganda.
(86)Paediatrics Department, Ikorodu General Hospital, Ikorodu, Nigeria.
(87)Department of Obstetrics and Gynaecology, Monash University, Clayton, Victoria, Australia.
(88)Department of Obstetrics and Gynecology, Aarhus University and Aarhus

University Hospital, Aarhus, Denmark.
(89)Curtin School of Population Health and enAble Institute, Curtin University, Perth, Western Australia, Australia.
(90)School of Nutrition, Catholic University del Maule, Region del Maule, Chile.
(91)Division of Neonatology, Department of Paediatrics, University Maternity Hospital Limerick and University of Limerick School of Medicine, Limerick, Ireland.
(92)School of Nursing and Health Professions, University of San Francisco, San Francisco, CA, USA.
(93)Paediatric Department, School of Adolescent and Child Health, University of Cape Town, Cape Town, South Africa.
(94)Mowbray Maternity Hospital, Western Cape Department of Health, Cape Town, South Africa.
(95)Federal Statistical Office (FSO), Neuchâtel, Switzerland.
(96)Department of Epidemiology, University of Washington, Seattle, WA, USA.
(97)Evidence and Intelligence for Action in Health Department, Pan-American Health Organization - World Health Organization, Washington, DC, USA.
(98)Division of Pediatric Endocrinology, Diabetology and Metabolism, Department of Pediatrics, Inselspital, Bern University Hospital, University of Bern, Bern, Switzerland.
(99)Department of Biomedical Research, University of Bern, Bern, Switzerland.
(100)The Children's Hospital at Westmead Clinical School, Faculty of Medicine and Health, The University of Sydney, Sydney, New South Wales, Australia.
(101)Foundation for Health Care Quality, Seattle, WA, USA.
(102)Department of Environmental Health, Boston University School of Public Health, Boston, MA, USA.
(103)Faculty of Nursing, Department of Midwifery, University of Iceland, Reykjavík, Iceland.
(104)Division of Neonatology, 1st Department of Pediatrics, Semmelweis University, Budapest, Hungary.
(105)Centre of Excellence in Women and Child Health, Aga Khan University, Nairobi, Kenya.
(106)Department of Paediatrics, University of Cape Town, Cape Town, South Africa.
(107)Manitoba Centre for Health Policy, Department of Community Health Sciences, Rady Faculty of Health Sciences, University of Manitoba, Winnipeg, Manitoba, Canada.
(108)The Royal Women's Hospital, University of Melbourne, Melbourne, Victoria, Australia.
(109)Public Health Scotland, Edinburgh, UK.
(110)II Department of Neonatology, Poznań University of Medical Sciences, Poznań, Poland.
(111)Public Health Unit, Father Thomas Alan Rooney Memorial Hospital, Asankrangwa, Western Region, Ghana.
(112)Department of Paediatrics, University of Jos, Jos, Nigeria.
(113)Department of Medical Simulation, Chair of Medical Education, Poznań University of Medical Sciences, Poznań, Poland.

(114)Murdoch Children's Research Institute, Royal Children's Hospital, Parkville, Victoria, Australia. david.burgner@mcri.edu.au.
(115)Department of Paediatrics, University of Melbourne, Parkville, Victoria, Australia. david.burgner@mcri.edu.au.
(116)Usher Institute, University of Edinburgh, Edinburgh, UK. sarah.stock@ed.ac.uk.
(117)Children's Hospital Research Institute of Manitoba, Department of Pediatrics and Child Health, University of Manitoba, Winnipeg, Manitoba, Canada. meghan.azad@umanitoba.ca.
(118)Departments of Pediatrics and Child Health, Community Health Sciences, and Immunology, University of Manitoba, Winnipeg, Manitoba, Canada, Winnipeg, Manitoba, Canada. meghan.azad@umanitoba.ca.
(#)Contributed equally

Preterm birth (PTB) is the leading cause of infant mortality worldwide. Changes in PTB rates, ranging from -90% to +30%, were reported in many countries following early COVID-19 pandemic response measures ('lockdowns'). It is unclear whether this variation reflects real differences in lockdown impacts, or perhaps differences in stillbirth rates and/or study designs. Here we present interrupted time series and meta-analyses using harmonized data from 52 million births in 26 countries, 18 of which had representative population-based data, with overall PTB rates ranging from 6% to 12% and stillbirth ranging from 2.5 to 10.5 per 1,000 births. We show small reductions in PTB in the first (odds ratio 0.96, 95% confidence interval 0.95-0.98, P value <0.0001), second (0.96, 0.92-0.99, 0.03) and third (0.97, 0.94-1.00, 0.09) months of lockdown, but not in the fourth month of lockdown (0.99, 0.96-1.01, 0.34), although there were some between-country differences after the first month. For high-income countries in this study, we did not observe an association between lockdown and stillbirths in the second (1.00, 0.88-1.14, 0.98), third (0.99, 0.88-1.12, 0.89) and fourth (1.01, 0.87-1.18, 0.86) months of lockdown, although we have imprecise estimates due to stillbirths being a relatively rare event. We did, however, find evidence of increased risk of stillbirth in the first month of lockdown in high-income countries (1.14, 1.02-1.29, 0.02) and, in Brazil, we found evidence for an association between lockdown and stillbirth in the second (1.09, 1.03-1.15, 0.002), third (1.10, 1.03-1.17, 0.003) and fourth (1.12, 1.05-1.19, <0.001) months of lockdown. With an estimated 14.8 million PTB annually worldwide, the modest reductions observed during early pandemic lockdowns translate into large numbers of PTB averted globally and warrant further research into causal pathways.

© 2023. The Author(s).

DOI: 10.1038/s41562-023-01522-y
PMCID: PMC10129868
PMID: 36849590 [Indexed for MEDLINE]

Conflict of interest statement: M.B.A. holds a Tier 2 Canada Research Chair in

the Developmental Origins of Chronic Disease at the University of Manitoba and is a Fellow in the Canadian Institutes for Advanced Research (CIFAR) Humans and the Microbiome Program. Her effort on this project was partly supported by HDR UK and ICODA. K.K.C.M. declares support from The Innovation and Technology Commission of the Hong Kong Special Administrative Region Government, and Hong Kong Research Grants Council Collaborative Research Fund Coronavirus Disease (COVID-19) and Novel Infectious Disease Research Exercise (Ref: C7154-20G) and grants from C W Maplethorpe Fellowship, National Institute of Health Research UK, European Commission Framework Horizon 2020 and has consulted for IQVIA Ltd. A.S. is supported by ICODA and HDR UK, and has received a research grant from HDR UK to the BREATHE Hub. He participates on the Scottish and UK Government COVID-19 Advisory Committees, unremunerated. S.J.S. is supported by a Wellcome Trust Clinical Career Development Fellowship (209560/Z/17/Z) and HDR UK, and has received personal fees from Hologic and Natera outside the submitted work. D.B. is supported by a National Health and Medical Research Council (Australia) Investigator Grant (GTN1175744). I.C.K.W. declares support from The Innovation and Technology Commission of the Hong Kong Special Administrative Region Government, and Hong Kong Research Grants Council Collaborative Research Fund Coronavirus Disease (COVID-19) and Novel Infectious Disease Research Exercise (Ref: C7154-20G), and grants from Hong Kong Research Grant Council, National Institute of Health Research UK, and European Commission Framework Horizon 2020. H.Z. is supported by a UNSW Scientia Program Award and reports grants from European Commission Framework Horizon 2020, Icelandic Centre for Research, and Australia's National Health and Medical Research Council. H.Z. was an employee of the UNSW Centre for Big Data Research in Health, which received funding from AbbVie Australia to conduct research, unrelated to the current study. I.I.A.A., C.D.A., K.A., A.I.A., L.C., S.S., G.E.-G., O.W.G., L. Huicho, S.H., A.K., K.L., V.N., I.P., N.R.R., T.R., T.A.H.R., V.L.S., E.M.S., L.T., R.W. and H.Z. received funding from HDRUK (grant #2020.106) to support data collection for the iPOP study. K.H., R.B., S.O.E., A.R.-P. and J.H. receive salary from ICODA. M.B. received trainee funding from HDRUK (grant #2020.106). J.E.M. received trainee funding from HDRUK (grant #2020.109). Other relevant funding awarded to authors to conduct research for iPOP include: M.G. received funding from THL, Finnish Institute for Health and Welfare to support data collection. K.D. received funding from EDCTP RIA2019 and HDRUK (grant #2020.106) to support data collection. R.B. received funding from Alzheimer's Disease Data Initiative and ICODA for the development of federated analysis. A.D.M. received funding from HDR UK who receives its funding from the UK Medical Research Council, Engineering and Physical Sciences Research Council, Economic and Social Research Council, Department of Health and Social Care (England), Chief Scientist Office of the Scottish Government Health and Social Care Directorates, Health and Social Care Research and Development Division (Welsh Government), Public Health Agency (Northern Ireland), British Heart Foundation (BHF) and the Wellcome Trust; and Administrative Data Research UK, which is funded by the Economic and Social Research Council (grant ES/S007393/1). N.A. received funding from the National Institutes of Health (R35GM138353). O.S received funding from NordForsk (grant #105545). The remaining authors declare no competing interests.

184. Int J Med Inform. 2023 Apr;172:105018. doi: 10.1016/j.ijmedinf.2023.105018. Epub 2023 Feb 10.

Acceptability and applicability of biometric iris scanning for the identification and follow up of highly mobile research participants living in fishing communities along the shores of Lake Victoria in Kenya, Tanzania, and Uganda.

Okello E(1), Ayieko P(2), Kwena Z(3), Nanyonjo G(4), Bahemuka U(5), Price M(6), Bukusi E(3), Hashim R(7), Nakamanya S(5), Okech B(4), Kuteesa M(8), Oketch B(3), Ssetaala A(4), Ruzagira E(9), Kidega W(8), Fast P(10), Kibengo F(5), Grosskurth H(2), Seeley J(9), Kapiga S(2).

Author information:
(1)Mwanza Intervention Trials Unit, National Institute for Medical Research, P.O Box 11936, Mwanza, Tanzania. Electronic address: elialilia.okello@mitu.or.tz.
(2)Mwanza Intervention Trials Unit, National Institute for Medical Research, P.O Box 11936, Mwanza, Tanzania; London School of Hygiene and Tropical Medicine London, United Kingdom.
(3)Kenya Medical Research Institute, KEMRI, Kisumu, Kenya.
(4)UVRI-IAVI HIV Vaccine Program, Entebbe, Uganda.
(5)Medical Research Council/Uganda Virus Research Institute Uganda Research Unit & London School of Hygiene and Tropical Medicine (MRC/UVRI & LSHTM) Uganda Research Unit, Entebbe, Uganda.
(6)International AIDS Vaccine Initiative, NY, USA; Department of Epidemiology and Biostatistics, University of California at San Francisco, San Francisco, CA, USA.
(7)Mwanza Intervention Trials Unit, National Institute for Medical Research, P.O Box 11936, Mwanza, Tanzania.
(8)International AIDS Vaccine Initiative, NY, USA.
(9)London School of Hygiene and Tropical Medicine London, United Kingdom; Medical Research Council/Uganda Virus Research Institute Uganda Research Unit & London School of Hygiene and Tropical Medicine (MRC/UVRI & LSHTM) Uganda Research Unit, Entebbe, Uganda.
(10)International AIDS Vaccine Initiative, NY, USA; Pediatric Infectious Diseases, School of Medicine, Stanford University, Palo Alto CA, USA.

BACKGROUND: Recruitment and retention of participants in research studies conducted in fishing communities remain a challenge because of population mobility. Reliable and acceptable methods for identifying and tracking participants taking part in HIV prevention and treatment research are needed. The study aims to assess the acceptability, and technical feasibility of iris scans as a biometric identification method for research participants in fishing communities.
METHODS: This was a cross-sectional study conducted in eight fishing communities

in Kenya, Tanzania, and Uganda, with follow-up after one month in a randomly selected subset of participants. All consenting participants had their iris scanned and then responded to the survey.
RESULTS: 1,199 participants were recruited. The median age was 33 [Interquartile range (IQR) 24-42] years; 56% were women. The overall acceptability of iris scanning was 99%, and the success rate was 98%. Eighty one percent (n = 949) had a successful scan on first attempt, 116 (10%) on second and 113 (9%) after more than two attempts. A month later, 30% (n = 341) of participants were followed up. The acceptability of repeat iris scanning was 99% (n = 340). All participants who accepted repeat iris scanning had successful scans, with 307 (90%) scans succeeding on first attempt; 25 (7%) on second attempt, and 8 (2%) after several attempts. The main reason for refusing iris scanning was fear of possible side effects of the scan on the eyes or body.
CONCLUSION: The acceptability and applicability of biometric iris scan as a technique for unique identification of research participants is high in fishing communities. However, successful use of the iris scanning technology in research will require education regarding the safety of the procedure.

Copyright © 2023 The Author(s). Published by Elsevier B.V. All rights reserved.

DOI: 10.1016/j.ijmedinf.2023.105018
PMID: 36774907 [Indexed for MEDLINE]

Conflict of interest statement: Declaration of Competing Interest The authors declare that they have no known competing financial interests or personal relationships that could have appeared to influence the work reported in this paper.

185. Curr Opin Pediatr. 2023 Apr 1;35(2):223-230. doi: 10.1097/MOP.0000000000001223. Epub 2023 Feb 16.

20 million pregnant women with group B streptococcus carriage: consequences, challenges, and opportunities for prevention.

Paul P(1), Gonçalves BP(1), Le Doare K(2)(3), Lawn JE(1).

Author information:
(1)Maternal, Adolescent, Reproductive & Child Health (MARCH) Centre, London School of Hygiene & Tropical Medicine.
(2)Paediatric Infectious Diseases Research Group, Institute of Infection and Immunity, St. George's, University of London, London, UK.
(3)Makerere University Johns Hopkins University, Kampala, Uganda.

PURPOSE OF REVIEW: Intrapartum antibiotic prophylaxis (IAP) is currently the only recommended preventive approach against clinical consequences of maternal Group B Streptococcus (GBS) colonization. In this review, we discuss new

findings of total perinatal GBS burden and relative effectiveness of differing targeting of IAP, notably microbiology-based and risk factor-based screening, including potential limitations. Finally, we provide updates on maternal GBS vaccines and their potential cost-effectiveness in disease reduction.
RECENT FINDINGS: Updated estimates of the burden of GBS related to pregnancy outcomes show (1) early-onset GBS disease incidence and deaths are high in some low- and middle-income countries where IAP has not been implemented and (2) late-onset GBS disease, preterm birth, and stillbirth, which are not preventable by IAP, remain a public health problem in both high and low-middle income settings. Observational evidence indicates that microbiology-based screening may be more effective than risk factor-based screening, but even in high-income countries, compliance is imperfect. To address the need for alternative prevention strategies, several maternal vaccine candidates are in clinical development, and modelling suggests these could be cost-effective in most scenarios.
SUMMARY: Recent progress in GBS vaccine research holds promise of reducing the large and preventable burden of mortality and disability caused by GBS disease, especially in higher-burden settings where clinical and laboratory services may be limited. Importantly vaccines also hold potential to prevent GBS stillbirths and GBS-associated preterm births.

Copyright © 2023 The Author(s). Published by Wolters Kluwer Health, Inc.

DOI: 10.1097/MOP.0000000000001223
PMCID: PMC9994794
PMID: 36749143 [Indexed for MEDLINE]

Conflict of interest statement: There are no conflicts of interest.

186. Vaccine X. 2023 Apr;13:100260. doi: 10.1016/j.jvacx.2023.100260. Epub 2023 Jan 7.

COVID-19 vaccine hesitancy among health workers in rural Uganda: A mixed methods study.

Ouni PD(1), Namulondo R(1), Wanume B(1), Okia D(1), Olupot PO(1)(2), Nantale R(3), Matovu JKB(1)(4), Napyo A(1), Moses Lubaale YA(1), Nshakira N(5), Mukunya D(1)(6).

Author information:
(1)Department of Community and Public Health, Busitema University, Mbale, Uganda.
(2)Department of Research, Mbale Clinical Research Institute, Mbale, Uganda.
(3)Department of Nursing, Busitema University, Mbale City, Uganda.
(4)Department of Disease Control and Environmental Health, Makerere University School of Public Health, Kampala, Uganda.

(5)Department of Research, Nikao Medical Center, Kampala, Uganda.
(6)Department of Public Health, Kabale University, Kabale, Uganda.

BACKGROUND: COVID-19 vaccination is the latest preventive intervention strategy in an attempt to control the global pandemic. Its efficacy has come under scrutiny because of break through infections among the vaccinated and need for booster doses. Besides, although health workers were prioritized for COVID-19 vaccine in most countries, anecdotal evidence points to high levels of reluctance to take the vaccine among health workers. We assessed COVID-19 vaccine hesitancy among health workers in Dokolo district, northern Uganda.
METHODS: This was a mixed-method, cross-sectional descriptive study. A customised self-administered data collection tool was used to collect quantitative data on characteristics, vaccination status and factors for or rejection of vaccine uptake. We conducted multivariable logistic regression to assess the association between selected exposures and vaccine hesitancy using Stata version 15. Conversely, qualitative data were collected using key informant interviews (KIIs) among 15 participants that were purposively selected. Data were analysed using thematic content analysis with the help of NVivo 12.0.
RESULTS: Of the 346 health workers enrolled, (13.3% [46/346]) were vaccine hesitant. Factors associated with vaccine hesitancy included fear of side effects (Adjusted Odds Ratio [AOR]: 2.55; 95% Confidence Interval [95%CI]: 1.00, 6.49) and health workers' lack of trust in the information provided by health authorities (AOR: 6.74; 95% CI: 2.43, 18.72). Similar factors were associated with vaccine hesitancy when we used the vaccine hesitancy score. Fear of side effects, distrust in vaccine stakeholders, and lack of trust in the vaccine were barriers to COVID-19 vaccination among health workers.
CONCLUSION: A small proportion of health workers were found to be hesitant to take the COVID-19 vaccine in this study. The paucity of COVID-19 vaccine safety information, which eroded the health workers' trust in the information they received on the vaccine, was responsible for health workers hesitancy to take up the vaccine in Uganda.

© 2023 The Authors.

DOI: 10.1016/j.jvacx.2023.100260
PMCID: PMC9824947
PMID: 36643854

Conflict of interest statement: The authors declare that they have no known competing financial interests or personal relationships that could have appeared to influence the work reported in this paper.

187. Lab Anim. 2023 Apr;57(2):136-148. doi: 10.1177/00236772221129976. Epub 2022 Nov 3.

Sustainable education and training in laboratory animal science and ethics in low- and middle-income countries in Africa - challenges, successes, and the way forward.

Mohr BJ(1)(2)(3), Souilem O(4), Abdussamad AM(5), Benmouloud A(6)(7)(8), Bugnon P(9), Chipangura JK(1), Fahmy SR(10), Fourie T(11)(12), Gaafar K(10), Jillani NE(13), Kantyok JT(14), Martin LF(9)(15), Mokopasetso M(16), Mugisha L(17), Nyachieo A(13), Lewis DI(18)(19).

Author information:
(1)Centre for Animal Research, Faculty of Health Sciences, University of Cape Town, South Africa.
(2)Department of Medicine, Faculty of Health Sciences, University of Cape Town, South Africa.
(3)Scientific Veterinary Consulting Inc., South Africa.
(4)Laboratory of Physiology and Pharmacology, National School of Veterinary Medicine, University of Manouba, Tunisia.
(5)Department of Veterinary Physiology and Biochemistry, Faculty of Veterinary Medicine, Bayero University, Nigeria.
(6)Department of Biology and Physiology of Organism, Houari Boumedienne University of Sciences and Technology, Algeria.
(7)Department of Biology, University of M'hamed Bougara Boumerdes, Algeria.
(8)Department of Nature and Life Sciences, University of Algiers 1, Algeria.
(9)Institute of Laboratory Animal Science, University of Zürich, Switzerland.
(10)Zoology Department, Faculty of Science, Cairo University, Egypt.
(11)LAS Veterinary Consultancy, South Africa.
(12)South African Association for Laboratory Animal Science (SAALAS), South Africa.
(13)Institute of Primate Research, Kenya.
(14)Johan Vet Network, Nigeria.
(15)Clinic for Zoo Animals, Exotic Pets and Wildlife, University of Zurich, Switzerland.
(16)Botswana Vaccine Institute, Botswana.
(17)College of Veterinary Medicine, Animal Resources and Biosecurity (COVAB), Makerere University, Uganda.
(18)School of Biomedical Sciences, Faculty of Biological Sciences, University of Leeds, UK.
(19)International Union of Basic and Clinical Pharmacology-Integrative and Organ Systems Pharmacology Initiative (IUPHAR IOSP).

Despite the recognised need for education and training in laboratory animal science (LAS) and ethics in Africa, access to such opportunities has historically been limited. To address this, the Pan-African Network for Laboratory Animal Science and Ethics (PAN-LASE) was established to pioneer a support network for the development of education and training in LAS and ethics across the African continent.In the 4.5 years since the establishment of PAN-LASE, 3635 individuals from 28 African countries have participated in our

educational activities. Returning to their home institutions, they have both established and strengthened institutional and regional hubs of knowledge and competence across the continent. Additionally, PAN-LASE supported the development of guidelines for establishment of institutional Animal Ethics Committees, a critical step in the implementation of ethical review processes across the continent, and in enhancing animal welfare and scientific research standards.Key challenges and opportunities for PAN-LASE going forward include the formalisation of the network; the sustainability of education and training programmes; implementation of effective hub-and-spoke models of educational provision; strengthening governance frameworks at institutional, national and regional levels; and the availability of Africa-centric open access educational resources.Our activities are enhancing animal welfare and the quality of animal research undertaken across Africa, enabling African researchers to undertake world-leading research to offer solutions to the challenges facing the continent. The challenges, successes and the lessons learnt from PAN-LASE's journey are applicable to other low- and middle-income countries across the world seeking to enhance animal welfare, research ethics and ethical review in their own country or region.

DOI: 10.1177/00236772221129976
PMID: 36329615 [Indexed for MEDLINE]

188. PLoS Negl Trop Dis. 2023 Mar 30;17(3):e0011236. doi: 10.1371/journal.pntd.0011236. eCollection 2023 Mar.

A randomized, controlled Phase 1b trial of the Sm-TSP-2 Vaccine for intestinal schistosomiasis in healthy Brazilian adults living in an endemic area.

Diemert DJ(1)(2), Correa-Oliveira R(3), Fraga CG(3), Talles F(3), Silva MR(3), Patel SM(4), Galbiati S(5), Kennedy JK(5), Lundeen JS(5), Gazzinelli MF(3), Li G(2), Hoeweler L(2), Deye GA(6), Bottazzi ME(7), Hotez PJ(7), El Sahly HM(4), Keitel WA(4), Bethony J(2), Atmar RL(4).

Author information:
(1)Department of Medicine, School of Medicine and Health Sciences, The George Washington University, Washington DC, United States of America.
(2)Department of Microbiology, Immunology and Tropical Medicine, School of Medicine and Health Sciences, The George Washington University, Washington DC, United States of America.
(3)Instituto René Rachou, Fundação Oswaldo Cruz em Minas Gerais, Belo Horizonte, Minas Gerais, Brazil.
(4)Departments of Molecular Virology & Microbiology and Medicine, Baylor College of Medicine, Houston, Texas, United States of America.
(5)The Emmes Company, LLC, Frederick, Maryland, United States of America.
(6)Division of Microbiology and Infectious Diseases (DMID), National Institutes of Allergy and Infectious, Diseases (NIAID), National Institutes of Health

(NIH), United States of America.
(7)Texas Children's Hospital Center for Vaccine Development, Department of Pediatrics, Baylor College of Medicine, Houston, Texas, United States of America.

BACKGROUND: Recombinant Schistosoma mansoni Tetraspanin-2 formulated on Alhydrogel (Sm-TSP-2/Alhydrogel) is being developed to prevent intestinal and hepatic disease caused by S. mansoni. The tegumentary Sm-TSP-2 antigen was selected based on its unique recognition by cytophilic antibodies in putatively immune individuals living in areas of ongoing S. mansoni transmission in Brazil, and preclinical studies in which vaccination with Sm-TSP-2 protected mice following infection challenge.
METHODS: A randomized, observer-blind, controlled, Phase 1b clinical trial was conducted in 60 healthy adults living in a region of Brazil with ongoing S. mansoni transmission. In each cohort of 20 participants, 16 were randomized to receive one of two formulations of Sm-TSP-2 vaccine (adjuvanted with Alhydrogel only, or with Alhydrogel plus the Toll-like receptor-4 agonist, AP 10-701), and 4 to receive Euvax B hepatitis B vaccine. Successively higher doses of antigen (10 µg, 30 µg, and 100 µg) were administered in a dose-escalation fashion, with progression to the next dose cohort being dependent upon evaluation of 7-day safety data after all participants in the preceding cohort had received their first dose of vaccine. Each participant received 3 intramuscular injections of study product at intervals of 2 months and was followed for 12 months after the third vaccination. IgG and IgG subclass antibody responses to Sm-TSP-2 were measured by qualified indirect ELISAs at pre- and post-vaccination time points through the final study visit.
RESULTS: Sm-TSP-2/Alhydrogel administered with or without AP 10-701 was well-tolerated in this population. The most common solicited adverse events were mild injection site tenderness and pain, and mild headache. No vaccine-related serious adverse events or adverse events of special interest were observed. Groups administered Sm-TSP-2/Alhydrogel with AP 10-701 had higher post-vaccination levels of antigen-specific IgG antibody. A significant dose-response relationship was seen in those administered Sm-TSP-2/Alhydrogel with AP 10-701. Peak anti-Sm-TSP-2 IgG levels were observed approximately 2 weeks following the third dose, regardless of Sm-TSP-2 formulation. IgG levels fell to low levels by Day 478 in all groups except the 100 µg with AP 10-701 group, in which 57% of subjects (4 of 7) still had IgG levels that were ≥4-fold higher than baseline. IgG subclass levels mirrored those of total IgG, with IgG1 being the predominant subclass response.
CONCLUSIONS: Vaccination of adults with Sm-TSP-2/Alhydrogel in an area of ongoing S. mansoni transmission was safe, minimally reactogenic, and elicited significant IgG and IgG subclass responses against the vaccine antigen. These promising results have led to initiation of a Phase 2 clinical trial of this vaccine in an endemic region of Uganda.
TRIAL REGISTRATION: NCT03110757.

Copyright: This is an open access article, free of all copyright, and may be

freely reproduced, distributed, transmitted, modified, built upon, or otherwise used by anyone for any lawful purpose. The work is made available under the Creative Commons CC0 public domain dedication.

DOI: 10.1371/journal.pntd.0011236
PMCID: PMC10089325
PMID: 36996185 [Indexed for MEDLINE]

Conflict of interest statement: The authors declare the following financial interests/personal relationships which may be considered as potential competing interests: PH, MEB, DJD and JB are patent holders for a multivalent anthelminthic vaccine including schistosomiasis. GAD is an employee of the Sponsor, NIAID. RLA, RC-O, CGF, FT, MR, SG, JKK, JSL, MFG, GL, LH, WAK, SP, HMES: None.

189. PLOS Glob Public Health. 2023 Mar 29;3(3):e0001289. doi: 10.1371/journal.pgph.0001289. eCollection 2023.

A structural equation modelling approach to understanding the determinants of childhood vaccination in Nigeria, Uganda and Guinea.

Bell J(1), Lartey B(1), Fernandez M(1), Darrell N(1), Exton-Smith H(1), Gardner C(1), Richards E(1), Akilo A(2), Odongo E(3), Ssenkungu J(3), Kotchi Kouadio R(4), Cissé M(5), Rérambyah ABAI(6), Adou M(6), West R(1)(7), Sharma S(1).

Author information:
(1)Ipsos Healthcare, London, United Kingdom.
(2)Ipsos Nigeria, Lagos, Nigeria.
(3)Ipsos Uganda, Kampala, Uganda.
(4)Ciblage, Dakar, Senegal.
(5)Ciblage, Conakry, Guinea.
(6)Ciblage, Abidjan, Ivory Coast.
(7)Boston University School of Public Health, Boston, Massachusetts, United States of America.

Vaccines have contributed to reductions in morbidity and mortality from preventable diseases globally, but low demand for vaccination threatens to reverse these gains. Explorations of the determinants of vaccination uptake may rely on proxy variables to describe complex phenomena and construct models without reference to underlying theories of vaccine demand. This study aimed to use the results of a formative qualitative study (described elsewhere) to construct and test a model to explain the determinants of vaccination uptake. Using the results of a survey among more than 3,000 primary caregivers of young children in Nigeria, Uganda and Guinea, factor analysis produced six explanatory factors. We then estimated the effects of each of these factors on uptake of immunization using a structural equation model. The results showed that the

probability that a child is fully vaccinated increases if a caregiver has support from others to vaccinate them (B = 0.33, β = 0.21, p<0.001) and if caregivers had poor experiences with the healthcare system (B = 0.09, β = 0.09, p = 0.007). Conversely, the probability of full vaccination decreases if the caregiver's husband exerts control over her decision-making ability (B = -0.29, β = -0.20, p<0.001), or if the caregiver perceives vaccines to be of low importance (B = -0.37, β = -0.27, p<0.001). Belief in religious protection (B = -0.07, β = -0.05, p = 0.118) and a belief that vaccines are harmful (B = -0.12, β = -0.04, p = 0.320) did not have an observed effect on vaccination status. This research suggests that interventions may benefit from that including entire families and communities in their design.

Copyright: © 2023 Bell et al. This is an open access article distributed under the terms of the Creative Commons Attribution License, which permits unrestricted use, distribution, and reproduction in any medium, provided the original author and source are credited.

DOI: 10.1371/journal.pgph.0001289
PMCID: PMC10058155
PMID: 36989212

Conflict of interest statement: I have read the journal's policy and the authors of this manuscript have the following competing interests: all authors work for market and social research organisations that carry out research on behalf of commercial companies, including in the field of vaccines

190. PLoS One. 2023 Mar 27;18(3):e0281559. doi: 10.1371/journal.pone.0281559. eCollection 2023.

Determinants of QuantiFERON Plus-diagnosed tuberculosis infection in adult Ugandan TB contacts: A cross-sectional study.

Mayito J(1)(2), Martineau AR(3), Tiwari D(3), Nakiyingi L(2)(4), Kateete DP(1), Reece ST(5), Biraro IA(4)(6).

Author information:
(1)Department of Immunology and Molecular Biology, School of Biomedical Sciences, Makerere University College of Health Sciences, Kampala, Uganda.
(2)Infectious Diseases Institute, Makerere University College of Health Sciences, Kampala, Uganda.
(3)Centre for Immunobiology, Blizard Institute, Barts and The London School of Medicine and Dentistry, Queen Mary University of London, London, United Kingdom.
(4)Department of Internal Medicine, Makerere University College of Health Sciences, Kampala, Uganda.
(5)School of Clinical Medicine, University of Cambridge, Cambridge, United Kingdom.

(6)MRC/UVRI & LSHTM Uganda Research Unit, Entebbe, Uganda.

BACKGROUND: The tuberculin skin test is commonly used to diagnose latent tuberculosis infection (LTBI) in resource-limited settings, but its specificity is limited by factors including cross-reactivity with BCG vaccine and environmental mycobacteria. Interferon-gamma release assays (IGRA) overcome this problem by detecting M. tuberculosis complex-specific responses, but studies to determine risk factors for IGRA-positivity in high TB burden settings are lacking.
METHODS: We conducted a cross-sectional study to determine factors associated with a positive IGRA by employing the QuantiFERON-TB® Gold-plus (QFT Plus) assay in a cohort of asymptomatic adult TB contacts in Kampala, Uganda. Multivariate logistic regression analysis with forward stepwise logit function was employed to identify independent correlates of QFT Plus-positivity.
RESULTS: Of the 202 participants enrolled, 129/202 (64%) were female, 173/202 (86%) had a BCG scar, and 67/202 (33%) were HIV-infected. Overall, 105/192 (54%, 95% CI 0.48-0.62) participants had a positive QFT Plus result. Increased risk of QFT-Plus positivity was independently associated with casual employment/unemployment vs. non-casual employment (adjusted odds ratio (aOR) 2.18, 95% CI 1.01-4.72), a family vs. non-family relation to the index patient (aOR 2.87, 95% CI 1.33-6.18), living in the same vs. a different house as the index (aOR 3.05, 95% CI 1.28-7.29), a higher body mass index (BMI) (aOR per additional kg/m2 1.09, 95% CI 1.00-1.18) and tobacco smoking vs. not (aOR 2.94, 95% CI 1.00-8.60). HIV infection was not associated with QFT-Plus positivity (aOR 0.91, 95% CI 0.42-1.96).
CONCLUSION: Interferon Gamma Release Assay positivity in this study population was lower than previously estimated. Tobacco smoking and BMI were determinants of IGRA positivity that were previously unappreciated.

Copyright: © 2023 Mayito et al. This is an open access article distributed under the terms of the Creative Commons Attribution License, which permits unrestricted use, distribution, and reproduction in any medium, provided the original author and source are credited.

DOI: 10.1371/journal.pone.0281559
PMCID: PMC10042355
PMID: 36972254 [Indexed for MEDLINE]

Conflict of interest statement: The authors have declared that no competing interests exist.

191. BMC Infect Dis. 2023 Mar 22;23(1):173. doi: 10.1186/s12879-023-08113-2.

Modelling the impact of combining HIV prevention interventions on HIV dynamics in fishing communities in Uganda.

Kremer C(1), Kamali A(2), Kuteesa M(3), Seeley J(3)(4), Hens N(5)(6), Nsubuga RN(4).

Author information:
(1)Interuniversity Institute for Biostatistics and Bioinformatics (I-BioStat), Data Science Institute, Hasselt University, Hasselt, Belgium. cecile.kremer@uhasselt.be.
(2)International AIDS Vaccine Initiative, Nairobi, Kenya.
(3)London School of Hygiene and Tropical Medicine, London, UK.
(4)Medical Research Council, Virus Research Unit & LSHTM Uganda Research Unit (MRC/UVRI & LSHTM), Entebbe, Uganda.
(5)Interuniversity Institute for Biostatistics and Bioinformatics (I-BioStat), Data Science Institute, Hasselt University, Hasselt, Belgium.
(6)Centre for Health Economics Research and Modelling Infectious Diseases (CHERMID), Vaccine and Infectious Disease Institute, University of Antwerp, Antwerp, Belgium.

BACKGROUND: In countries with mature generalized HIV epidemics such as Uganda, there are still groups of individuals that are disproportionately affected.
Among the key populations in Uganda are fishing communities, which make up about 10% of the population. Compared to the general population, HIV prevalence and incidence among individuals living in these communities is high. This high HIV burden has been attributed to several factors including limited access to prevention and treatment services as well as ongoing high-risk sexual behaviour.
METHODS: We investigated the impact of combined HIV prevention interventions on HIV transmission dynamics in high-risk fishing communities in Uganda using a deterministic compartmental model. The model was calibrated to seroprevalence data from a census performed in 2014. To account for remaining uncertainty in the calibrated model parameters, 50 000 simulated scenarios were modelled to investigate the impact of combined prevention interventions.
RESULTS: The projected HIV incidence decreased from 1.87 per 100 PY without intervention scale-up to 0.25 per 100 PY after 15 years (2014-2029) of intervention scale-up. A potential combination achieving this 87% reduction in incidence over 15 years in Ugandan FCs included condom use in about 60% of sexual acts, 23% of susceptible men circumcised, 87% of people living with HIV aware of their status, 75% of those on ART, and about 3% of susceptible individuals on oral PrEP. Uncertainty analysis revealed relative reductions in incidence ranging from 30.9 to 86.8%. Sensitivity analyses suggested that condom use and early ART were the most important interventions.
CONCLUSION: Reducing HIV incidence, as well as prevalence and AIDS-related mortality, in these high-risk fishing communities in Uganda is attainable over 15 years with a combination prevention package. Our projected intervention coverage levels are well within the national targets set by the Uganda government and enable coming close to reaching the UNAIDS 95-95-95 targets to end AIDS as a public health threat by 2030.

© 2023. The Author(s).

DOI: 10.1186/s12879-023-08113-2
PMCID: PMC10031877
PMID: 36949387 [Indexed for MEDLINE]

Conflict of interest statement: The authors declare that they have no competing interests.

192. BMJ Open. 2023 Mar 17;13(3):e067377. doi: 10.1136/bmjopen-2022-067377.

Uptake of COVID-19 vaccines and associated factors among adults in Uganda: a cross-sectional survey.

Ndejjo R(1), Chen N(2), Kabwama SN(3), Namale A(4), Wafula ST(4), Wanyana I(5), Kizito S(4), Kiwanuka SN(6), Sambisa W(7), Tsai LL(2), Wanyenze RK(4).

Author information:
(1)Department of Disease Control and Environmental Health, School of Public Health, College of Health Sciences, Makerere University, Kampala, Uganda rndejjo@musph.ac.ug.
(2)Department of Political Science, Massachusetts Institute of Technology, Cambridge, Massachusetts, USA.
(3)Department of Community Health and Behavioral Sciences, School of Public Health, College of Health Sciences, Makerere University, Kampala, Uganda.
(4)Department of Disease Control and Environmental Health, School of Public Health, College of Health Sciences, Makerere University, Kampala, Uganda.
(5)Department of Epidemiology and Biostatistics, School of Public Health, College of Health Sciences, Makerere University, Kampala, Uganda.
(6)Department of Health Policy, Planning and Management, School of Public Health, College of Health Sciences, Makerere University, Kampala, Uganda.
(7)Bill & Melinda Gates Foundation, Seattle, Washington, USA.

OBJECTIVE: COVID-19 pandemic remains one of the most significant public health challenges ever faced globally. Vaccines are key to ending the pandemic as well as minimise its consequences. This study determined the uptake of COVID-19 vaccines and associated factors among adults in Uganda.
DESIGN, SETTING AND PARTICIPANTS: We conducted a cross-sectional mobile phone survey among adults in Uganda.
MAIN OUTCOME VARIABLE: Participants reported their uptake of COVID-19 vaccines.
RESULTS: Of the participants contacted, 94% (1173) completed the survey. Overall, 49.7% had received COVID-19 vaccines with 19.2% having obtained a full dose and 30.5% an incomplete dose. Among the unvaccinated, 91.0% indicated intention to vaccinate. Major reasons for vaccine uptake were protection of self from COVID-19 (86.8%) and a high perceived risk of getting the virus (19.6%). On the other hand, non-uptake was related to vaccine unavailability (42.4%), lack of time (24.1%) and perceived safety (12.5%) and effectiveness concerns (6.9%).

The factors associated with receiving COVID-19 vaccines were older age (≥65 years) (Adjusted Prevalence Ratio (APR)=1.32 (95% CI: 1.08 to 1.61)), secondary (APR=1.36 (95% CI: 1.12 to 1.65)) or tertiary education (APR=1.62 (95% CI: 1.31 to 2.00)) and health workers as a source of information on COVID-19 (APR=1.26 (95% CI: 1.10 to 1.45)). Also, reporting a medium-income (APR=1.24 (95% CI: 1.02 to 1.52)) and residence in Northern (APR=1.55, 95% CI: 1.18 to 2.02) and Central regions (APR=1.48, 95% CI: 1.16 to 1.89) were associated with vaccine uptake.
CONCLUSIONS: Uptake of COVID-19 vaccines was moderate in this sample and was associated with older age, secondary and tertiary education, medium-income, region of residence and health workers as a source of COVID-19 information. Efforts are needed to increase access to vaccines and should use health workers as champions to enhance uptake.

© Author(s) (or their employer(s)) 2023. Re-use permitted under CC BY. Published by BMJ.

DOI: 10.1136/bmjopen-2022-067377
PMCID: PMC10030279
PMID: 36931667 [Indexed for MEDLINE]

Conflict of interest statement: Competing interests: None declared.

193. iScience. 2023 Mar 17;26(3):106230. doi: 10.1016/j.isci.2023.106230. Epub 2023 Feb 18.

Evolution of increased positive charge on the SARS-CoV-2 spike protein may be adaptation to human transmission.

Cotten M(1)(2), Phan MVT(2).

Author information:
(1)Medical Research Council-University of Glasgow Centre for Virus Research, 464 Bearsden Road, Glasgow G61 1QH, Scotland, UK.
(2)UK Medical Research Council-Uganda Virus Research Institute and London School of Hygiene and Tropical Medicine Uganda Research Unit, Plot 51- 59 Nakiwogo Road, P.O Box 49, Entebbe, Uganda, UK.

The severe acute respiratory syndrome coronavirus 2 (SARS-CoV-2) continues to evolve and infect individuals. The exterior surface of the SARS-CoV-2 virion is dominated by the spike protein, and the current work examined spike protein biochemical features that have changed during the 3 years in which SARS-CoV-2 has infected humans. Our analysis identified a striking change in spike protein charge, from -8.3 in the original Lineage A and B viruses to -1.26 in most of the current Omicron viruses. We conclude that in addition to immune selection pressure, the evolution of SARS-CoV-2 has also altered viral spike protein biochemical properties, which may influence virion survival and promote

transmission. Future vaccine and therapeutic development should also exploit and target these biochemical properties.

© 2023 The Author(s).

DOI: 10.1016/j.isci.2023.106230
PMCID: PMC9937996
PMID: 36845032

Conflict of interest statement: The authors declare no competing interests.

194. Front Immunol. 2023 Mar 16;14:1152522. doi: 10.3389/fimmu.2023.1152522. eCollection 2023.

Rapid, early, and potent Spike-directed IgG, IgM, and IgA distinguish asymptomatic from mildly symptomatic COVID-19 in Uganda, with IgG persisting for 28 months.

Serwanga J(1)(2), Ankunda V(2), Sembera J(2), Kato L(1), Oluka GK(1)(2), Baine C(2), Odoch G(1), Kayiwa J(3), Auma BO(1), Jjuuko M(4), Nsereko C(5), Cotten M(1)(6), Onyachi N(4), Muwanga M(5), Lutalo T(7), Fox J(8), Musenero M(9), Kaleebu P(1)(2); COVID-19 Immunoprofiling Team.

Collaborators: Namubiru P, Akoli HC, Mugaba S, Nalumansi A, Odoch G, Freddie K, Mwesigwa D, Katende JS.

Author information:
(1)Pathogen Genomics, Phenotype, and Immunity Program, Medical Research Council, Uganda Virus Research Institute and London School of Hygiene and Tropical Medicine, Uganda Research Unit, Entebbe, Uganda.
(2)Department of Immunology, Uganda Virus Research Institute, Entebbe, Uganda.
(3)Department of Virology, Uganda Virus Research Institute, Entebbe, Uganda.
(4)Department of Internal Medicine, Masaka Regional Referral Hospital, Masaka, Uganda.
(5)Department of Internal Medicine, Entebbe Regional Referral Hospital, Entebbe, Uganda.
(6)Medical Research Council, University of Glasgow Centre for Virus Research, Glasgow, United Kingdom.
(7)Department of Epidemiology and Data Management, Uganda Virus Research Institute, Entebbe, Uganda.
(8)Guy's and St Thomas' National Health Services Foundation Trust, King's College London, London, United Kingdom.
(9)Science, Technology, and Innovation Secretariat, Office of the President, Government of Uganda, Kampala, Uganda.

INTRODUCTION: Understanding how spike (S)-, nucleoprotein (N)-, and RBD-directed

antibody responses evolved in mild and asymptomatic COVID-19 in Africa and their interactions with SARS-CoV-2 might inform development of targeted treatments and vaccines.
METHODS: Here, we used a validated indirect in-house ELISA to characterise development and persistence of S- and N-directed IgG, IgM, and IgA antibody responses for 2430 SARS-CoV-2 rt-PCR-diagnosed Ugandan specimens from 320 mild and asymptomatic COVID-19 cases, 50 uninfected contacts, and 54 uninfected non-contacts collected weekly for one month, then monthly for 28 months.
RESULTS: During acute infection, asymptomatic patients mounted a faster and more robust spike-directed IgG, IgM, and IgA response than those with mild symptoms (Wilcoxon rank test, p-values 0.046, 0.053, and 0.057); this was more pronounced in males than females. Spike IgG antibodies peaked between 25 and 37 days (86.46; IQR 29.47-242.56 BAU/ml), were significantly higher and more durable than N- and RBD IgG antibodies and lasted for 28 months. Anti-spike seroconversion rates consistently exceeded RBD and nucleoprotein rates. Spike- and RBD-directed IgG antibodies were positively correlated until 14 months (Spearman's rank correlation test, p-values 0.0001 to 0.05), although RBD diminished faster. Significant anti-spike immunity persisted without RBD. 64% and 59% of PCR-negative, non-infected non-contacts and suspects, exhibited baseline SARS-CoV-2 N-IgM serological cross-reactivity, suggesting undetected exposure or abortive infection. N-IgG levels waned after 787 days, while N-IgM levels remained undetectable throughout.
DISCUSSION: Lower N-IgG seroconversion rates and the absence of N-IgM indicate that these markers substantially underestimate the prior exposure rates. Our findings provide insights into the development of S-directed antibody responses in mild and asymptomatic infections, with varying degrees of symptoms eliciting distinct immune responses, suggesting distinct pathogenic pathways. These longer-lasting data inform vaccine design, boosting strategies, and surveillance efforts in this and comparable settings.

Copyright © 2023 Serwanga, Ankunda, Sembera, Kato, Oluka, Baine, Odoch, Kayiwa, Auma, Jjuuko, Nsereko, Cotten, Onyachi, Muwanga, Lutalo, Fox, Musenero, Kaleebu and The COVID-19 Immunoprofiling Team.

DOI: 10.3389/fimmu.2023.1152522
PMCID: PMC10060567
PMID: 37006272 [Indexed for MEDLINE]

Conflict of interest statement: The authors declare that the research was conducted in the absence of any commercial or financial relationships that could be construed as a potential conflict of interest.

195. AIDS. 2023 Mar 15;37(4):625-635. doi: 10.1097/QAD.0000000000003437. Epub 2022 Dec 1.

High-risk human papillomavirus genotype distribution among women living with and

at risk for HIV in Africa.

Chachage M(1)(2)(3), Parikh AP(4)(5), Mahenge A(1), Bahemana E(4)(6), Mnkai J(1), Mbuya W(1), Mcharo R(1), Maganga L(1), Mwamwaja J(1), Gervas R(4)(6), Kibuuka H(7), Maswai J(4)(8), Singoei V(9)(10), Iroezindu M(4)(11), Fasina A(4)(11), Esber A(4)(5), Dear N(4)(5), Imbach M(4)(5), Crowell TA(4)(5), Hern J(4)(5), Song X(4)(5), Hoelscher M(3)(12), Polyak CS(4)(5), Ake JA(4), Geldmacher C(3)(12); AFRICOS Study Group.

Author information:
(1)National Institute for Medical Research-Mbeya Medical Research Centre.
(2)University of Dar es Salaam - Mbeya College of Health and Allied Sciences (UDSM-MCHAS), Mbeya, Tanzania.
(3)Division of Infectious Diseases and Tropical Medicine, Medical Center of the University of Munich (LMU), Munich, Germany.
(4)U.S. Military HIV Research Program, Walter Reed Army Institute of Research, Silver Spring.
(5)Henry M. Jackson Foundation for the Advancement of Military Medicine Inc., Bethesda, Maryland.
(6)HJF Medical Research International, Mbeya, Tanzania.
(7)Makerere University Walter Reed Project, Kampala, Uganda.
(8)U.S. Army Medical Research Directorate - Africa, Kericho.
(9)U.S. Army Medical Research Directorate - Africa, Kisumu.
(10)HJF Medical Research International, Kisumu, Kenya.
(11)HJF Medical Research International, Abuja, Nigeria.
(12)German Center for Infection Research (DZIF), partner site Munich, Munich, Germany.

OBJECTIVE: Cervical cancer is a common preventable cancer among African women living with HIV (WLWH). Molecular diagnostics for high-risk human papillomavirus (HR-HPV) genotypes are standard components of cervical cancer screening in resource-rich countries but not in resource-limited settings. We evaluated HR-HPV genotypes among women with and without HIV in four African countries to inform cervical cancer preventive strategies.
METHODS: The African Cohort Study (AFRICOS) enrolled participants with and without HIV at 12 clinics in Tanzania, Kenya, Uganda, and Nigeria. Cervical cytobrush specimens from women were genotyped for 14 HR-HPV types using the multiplex Seegene Anyplex real-time PCR assay. Robust Poisson regression was used to estimate relative risks (RRs) and 95% confidence intervals (CIs) for factors associated with HR-HPV in WLWH.
RESULTS: From January 2015 to March 2020, 868 WLWH and 134 women living without HIV (WLWoH) were tested for HR-HPV with prevalence of 50.9 and 38.1%, respectively (P =0.007). Among WLWH, 844 (97.4%) were antiretroviral therapy (ART)-experienced and 772 (89.7%) virally suppressed 1000 copies/ml or less. The most frequent HR-HPV types among WLWH were HPV-16 (13.5%), HPV-52 (9.5%), and HPV-35 (9.3%). HR-HPV infection was more common among Tanzanian WLWH (adjusted RR: 1.23, 95% CI 1.05-1.44, P =0.012). Also, WLWH with CD4 + T cells of less

than 200 cell/µl had 1.51-fold increased risk of having HR-HPV (95% CI 1.23-1.86, P <0.001).
CONCLUSION: HR-HPV was common in WLWH in four African countries, particularly among women with low CD4 + cell count. Scale up of HPV vaccines and development of vaccines with broader activity against less common HR-HPV types may improve cervical cancer prevention in Africa.

Copyright © 2022 The Author(s). Published by Wolters Kluwer Health, Inc.

DOI: 10.1097/QAD.0000000000003437
PMCID: PMC9994804
PMID: 36398743 [Indexed for MEDLINE]

Conflict of interest statement: There are no conflicts of interest.

196. Front Immunol. 2023 Mar 14;14:1113194. doi: 10.3389/fimmu.2023.1113194. eCollection 2023.

Optimisation and Validation of a conventional ELISA and cut-offs for detecting and quantifying anti-SARS-CoV-2 Spike, RBD, and Nucleoprotein IgG, IgM, and IgA antibodies in Uganda.

Oluka GK(1)(2), Namubiru P(1), Kato L(1), Ankunda V(2), Gombe B(1), Cotten M(1)(3); COVID-19 Immunoprofiling Team; Musenero M(4), Kaleebu P(1)(2), Fox J(5), Serwanga J(1)(2).

Collaborators: Baine C, Sembera J, Mugaba S, Akoli CH, Odoch G, Mwesigwa D, Ejou P, Katende JS.

Author information:
(1)Pathogen Genomics, Phenotype, and Immunity Program, Medical Research Council, Uganda Virus Research Institute and London School of Hygiene and Tropical Medicine, Uganda Research Unit, Entebbe, Uganda.
(2)Department of Immunology, Uganda Virus Research Institute, Entebbe, Uganda.
(3)Medical Research Council, University of Glasgow Centre for Virus Research, Glasgow, United Kingdom.
(4)Science, Technology, and Innovation Secretariat, Office of the President, Government of Uganda, Kampala, Uganda.
(5)Guy's and St Thomas' National Health Services Foundation Trust, King's College London, London, United Kingdom.

There is an urgent need for better immunoassays to measure antibody responses as part of immune-surveillance activities and to profile immunological responses to emerging SARS-CoV-2 variants. We optimised and validated an in-house conventional ELISA to identify and quantify SARS-CoV-2 spike- (S-), receptor binding domain- (RBD-), and nucleoprotein- (N-) directed IgG, IgM, and IgA

binding antibodies in the Ugandan population and similar settings. Pre- and post-pandemic specimens were used to compare the utility of mean ± 2SD, mean ± 3SD, 4-fold above blanks, bootstrapping, and receiver operating characteristic (ROC) analyses in determining optimal cut-off optical densities at 450 nm (OD) for discriminating between antibody positives and negatives. "Limits of detection" (LOD) and "limits of quantitation" (LOQ) were validated alongside the assay's uniformity, accuracy, inter-assay and inter-operator precision, and parallelism. With spike-directed sensitivity and specificity of 95.33 and 94.15%, respectively, and nucleoprotein sensitivity and specificity of 82.69 and 79.71%, ROC was chosen as the best method for determining cutoffs. Accuracy measurements were within the expected CV range of 25%. Serum and plasma OD values were highly correlated (r = 0.93, p=0.0001). ROC-derived cut-offs for S-, RBD-, and N-directed IgG, IgM, and IgA were 0.432, 0.356, 0.201 (S), 0.214, 0.350, 0.303 (RBD), and 0.395, 0.229, 0.188 (N). The sensitivity and specificity of the S-IgG cut-off were equivalent to the WHO 20/B770-02 S-IgG reference standard at 100% level. Spike negative IgG, IgM, and IgA ODs corresponded to median antibody concentrations of 1.49, 3.16, and 0 BAU/mL, respectively, consistent with WHO low titre estimates. Anti-spike IgG, IgM, and IgA cut-offs were equivalent to 18.94, 20.06, and 55.08 BAU/mL. For the first time, we provide validated parameters and cut-off criteria for the in-house detection of subclinical SARS-CoV-2 infection and vaccine-elicited binding antibodies in the context of Sub-Saharan Africa and populations with comparable risk factors.

Copyright © 2023 Oluka, Namubiru, Kato, Ankunda, Gombe, Cotten, The COVID-19 Immunoprofiling Team, Musenero, Kaleebu, Fox and Serwanga.

DOI: 10.3389/fimmu.2023.1113194
PMCID: PMC10045470
PMID: 36999017 [Indexed for MEDLINE]

Conflict of interest statement: The authors declare that the research was conducted in the absence of any commercial or financial relationships that could be construed as a potential conflict of interest.

197. Vaccines (Basel). 2023 Mar 14;11(3):647. doi: 10.3390/vaccines11030647.

Assessing Potential Exemplars in Reducing Zero-Dose Children: A Novel Approach for Identifying Positive Outliers in Decreasing National Levels and Geographic Inequalities in Unvaccinated Children.

Fullman N(1), Correa GC(2), Ikilezi G(1), Phillips DE(1), Reynolds HW(2).

Author information:
(1)Exemplars in Global Health, Gates Ventures, 2401 Elliott Ave, Seattle, WA 98121, USA.
(2)Gavi, the Vaccine Alliance, Chemin du Pommier 40, Le Grand-Saconnex, 1218

Geneva, Switzerland.

BACKGROUND: Understanding past successes in reaching unvaccinated or "zero-dose" children can help inform strategies for improving childhood immunization in other settings. Drawing from positive outlier methods, we developed a novel approach for identifying potential exemplars in reducing zero-dose children.
METHODS: Focusing on 2000-2019, we assessed changes in the percentage of under-one children with no doses of the diphtheria-tetanus-pertussis vaccine (no-DTP) across two geographic dimensions in 56 low- or lower-middle-income countries: (1) national levels; (2) subnational gaps, as defined as the difference between the 5th and 95th percentiles of no-DTP prevalence across second administrative units. Countries with the largest reductions for both metrics were considered positive outliers or potential 'exemplars', demonstrating exception progress in reducing national no-DTP prevalence and subnational inequalities. Last, so-called "neighborhood analyses" were conducted for the Gavi Learning Hub countries (Nigeria, Mali, Uganda, and Bangladesh), comparing them with countries that had similar no-DTP measures in 2000 but different trajectories through 2019.
RESULTS: From 2000 to 2019, the Democratic Republic of the Congo, Ethiopia, and India had the largest absolute decreases for the two no-DTP dimensions-national prevalence and subnational gaps-while Bangladesh and Burundi registered the largest relative reductions for each no-DTP metric. Neighborhood analyses highlighted possible opportunities for cross-country learning among Gavi Learning Hub countries and potential exemplars in reducing zero-dose children.
CONCLUSIONS: Identifying where exceptional progress has occurred is the first step toward better understanding how such gains could be achieved elsewhere. Further examination of how countries have successfully reduced levels of zero-dose children-especially across variable contexts and different drivers of inequality-could support faster, sustainable advances toward greater vaccination equity worldwide.

DOI: 10.3390/vaccines11030647
PMCID: PMC10056845
PMID: 36992231

Conflict of interest statement: N.F., G.I. and D.E.P. are paid employees of Gates Ventures, which coordinates the Exemplars in Global Health (EGH) program. N.F., G.I., and D.E.P. previously worked at the Institute for Metrics and Evaluation (IHME): September 2008–2011 and February 2013–June 2022 for N.F.; August 2014–July 2020 for G.I.; and June 2009–February 2019 for D.E.P., G.C.C. and H.W.R. are employed by Gavi, the Vaccine Alliance. They had total freedom to express their views, which do not necessarily reflect those of Gavi.

198. BMC Pregnancy Childbirth. 2023 Mar 13;23(1):172. doi:
10.1186/s12884-023-05417-8.

Data collection systems for active safety surveillance of vaccines during pregnancy in low- and middle-income countries: developing and piloting an assessment tool (VPASS).

Belizán M(#)(1), Rodriguez Cairoli F(#)(2), Mazzoni A(2), Goucher E(3), Zaraa S(4), Matthews S(3), Pingray V(2), Stergachis A(4)(5), Xiong X(3), Berrueta M(2), Buekens P(3).

Author information:
(1)Instituto de Efectividad Clínica y Sanitaria (IECS), Dr. Emilio Ravignani 2024 (C1014CPV), Buenos Aires, Argentina. mbelizan@iecs.org.ar.
(2)Instituto de Efectividad Clínica y Sanitaria (IECS), Dr. Emilio Ravignani 2024 (C1014CPV), Buenos Aires, Argentina.
(3)Tulane University School of Public Health and Tropical Medicine, New Orleans, LA, 70112, USA.
(4)School of Pharmacy, University of Washington, Seattle, WA, 98195, USA.
(5)School of Public Health, University of Washington, Seattle, WA, 98195, USA.
(#)Contributed equally

BACKGROUND: There is an urgent need for active safety surveillance to monitor vaccine exposure during pregnancy in low- and middle-income countries (LMICs). Existing maternal, newborn, and child health (MNCH) data collection systems could serve as platforms for post-marketing active surveillance of maternal immunization safety. To identify sites using existing systems, a thorough assessment should be conducted. Therefore, this study had the objectives to first develop an assessment tool and then to pilot this tool in sites using MNCH data collection systems through virtual informant interviews.
METHODS: We conducted a rapid review of the literature to identify frameworks on population health or post-marketing drug surveillance. Four frameworks that met the eligibility criteria were identified and served to develop an assessment tool capable of evaluating sites that could support active monitoring of vaccine safety during pregnancy. We conducted semi-structured interviews in six geographical sites using MNCH data collection systems (DHIS2, INDEPTH, and GNMNHR) to pilot domains included in the assessment tool.
RESULTS: We developed and piloted the "VPASS (Vaccines during Pregnancy - sites supporting Active Safety Surveillance) assessment tool" through interviews with nine stakeholders, including central-level systems key informants and site-level managers from DHIS2 and GNMNHR; DHIS2 in Kampala (Uganda) and Kigali (Rwanda); GNMNHR from Belagavi (India) and Lusaka (Zambia); and INDEPTH from Nanoro (Burkina Faso) and Manhica (Mozambique). The tool includes different domains such as the system's purpose, the scale of implementation, data capture and confidentiality, type of data collected, the capability of integration with other platforms, data management policies and data quality monitoring. Similarities among sites were found regarding some domains, such as data confidentiality, data management policies, and data quality monitoring. Four of the six sites met some domains to be eligible as potential sites for active surveillance of vaccinations during pregnancy, such as a routine collection of

MNCH individual data and the capability of electronically integrating individual MNCH outcomes with information related to vaccine exposure during pregnancy. Those sites were: Rwanda (DHIS2), Manhica (IN-DEPTH), Lusaka (GNMNHR), and Belagavi (GNMNHR).
CONCLUSION: This study's findings should inform the successful implementation of active safety surveillance of vaccines during pregnancy by identifying and using active individual MNCH data collection systems in LMICs.

© 2023. The Author(s).

DOI: 10.1186/s12884-023-05417-8
PMCID: PMC10010225
PMID: 36915061 [Indexed for MEDLINE]

Conflict of interest statement: The authors declare no competing interests.

199. Int J Appl Posit Psychol. 2023 Mar 10:1-24. doi: 10.1007/s41042-023-00091-9. Online ahead of print.

Psychological Capital and Quality of life of Refugees in Uganda During COVID-19 Pandemic: A Serial Mediation Model.

Baluku MM(1).

Author information:
(1)School of Psychology, Makerere University, P.O Box 7062, Kampala, Uganda.

Background: The COVID-19 pandemic has in the past two years caused and continues to cause enormous effects on lifestyle, mental health, and quality of life. With no known treatment and vaccination, behavioral control measures became central in controlling the pandemic. However, the intensity of the pandemic and the stringent control measures were immensely stressful. The control measures became an added psychological burden to people living in precarious situations such as refugees in low-income countries. Purpose: Given the benefits of psychological capital, the present study aimed at investigating the role of psychological capital in enhancing the quality of life among refugees in Uganda during the COVID-19 pandemic. It was hypothesized that the effects of psychological capital on quality of life are serially mediated through coping strategies, adherence to COVID-19 control measures, and mental health. Methods: Data was collected using a self-administered questionnaire in July and August 2020 after the first lockdown. Participants were 353 South Sudanese and Somali refugees living in Kampala city suburbs and Bidibidi refugee settlement. Findings: Psychological capital was positively associated with approach coping, mental health, and quality of life. However, psychological capital was negatively associated with adherence to COVID-19 control measures. Significant indirect effects of psychological capital on quality of life through approach coping, mental health,

and adherence were found. However, serial mediation effects were only substantial via approach coping and mental health. Conclusion: Psychological capital is an important resource in coping with the challenges posed by COVID-19 and maintaining a good level of psychological functioning and quality of life. Preserving and boosting psychological capital is essential in responding to COVID-19 and other related disasters and crises, which are common in vulnerable populations such as refugee communities in low-income countries.

© The Author(s), under exclusive licence to Springer Nature Switzerland AG 2023, Springer Nature or its licensor (e.g. a society or other partner) holds exclusive rights to this article under a publishing agreement with the author(s) or other rightsholder(s); author self-archiving of the accepted manuscript version of this article is solely governed by the terms of such publishing agreement and applicable law.

DOI: 10.1007/s41042-023-00091-9
PMCID: PMC10000344
PMID: 37361624

Conflict of interest statement: Conflict of InterestThe authors declare no conflict of interest.

200. JMIR Res Protoc. 2023 Mar 8;12:e40753. doi: 10.2196/40753.

The Good Talk! A Serious Game to Boost People's Competence to Have Open Conversations About COVID-19: Protocol for a Randomized Controlled Trial.

Elkin JA(#)(1), McDowell M(#)(2)(3), Yau B(4), Machiri SV(5), Pal S(6), Briand S(4), Muneene D(1), Nguyen T(4), Purnat TD(4).

Author information:
(1)Digital Health and Innovation Department, World Health Organization, Geneva, Switzerland.
(2)Harding Center for Risk Literacy, Faculty of Health Sciences Brandenburg, University of Potsdam, Potsdam, Germany.
(3)Max Planck Institute for Human Development, Berlin, Germany.
(4)Department of Pandemic and Epidemic Preparedness and Prevention, World Health Organization Health Emergencies Programme, World Health Organization, Geneva, Switzerland.
(5)Vaccine Safety Project, African Field Epidemiology Network, Kampala, Uganda.
(6)Department of Regulation and Prequalification, World Health Organization, Geneva, Switzerland.
(#)Contributed equally

BACKGROUND: Vaccine hesitancy is one of the many factors impeding efforts to control the COVID-19 pandemic. Exacerbated by the COVID-19 infodemic,

misinformation has undermined public trust in vaccination, led to greater polarization, and resulted in a high social cost where close social relationships have experienced conflict or disagreements about the public health response.
OBJECTIVE: The purpose of this paper is to describe the theory behind the development of a digital behavioral science intervention-The Good Talk!-designed to target vaccine-hesitant individuals through their close contacts (eg, family, friends, and colleagues) and to describe the methodology of a research study to evaluate its efficacy.
METHODS: The Good Talk! uses an educational serious game approach to boost the skills and competences of vaccine advocates to have open conversations about COVID-19 with their close contacts who are vaccine hesitant. The game teaches vaccine advocates evidence-based open conversation skills to help them speak with individuals who have opposing points of view or who may ascribe to nonscientifically supported beliefs while retaining trust, identifying common ground, and fostering acceptance and respect of divergent views. The game is currently under development and will be available on the web, free to access for participants worldwide, and accompanied by a promotional campaign to recruit participants through social media channels. This protocol describes the methodology for a randomized controlled trial that will compare participants who play The Good Talk! game with a control group that plays the widely known noneducational game Tetris. The study will evaluate a participant's open conversation skills, self-efficacy, and behavioral intentions to have an open conversation with a vaccine-hesitant individual both before and after game play.
RESULTS: Recruitment will commence in early 2023 and will cease once 450 participants complete the study (225 per group). The primary outcome is improvement in open conversation skills. Secondary outcomes are self-efficacy and behavioral intentions to have an open conversation with a vaccine-hesitant individual. Exploratory analyses will examine the effect of the game on implementation intentions as well as potential covariates or subgroup differences based on sociodemographic information or previous experiences with COVID-19 vaccination conversations.
CONCLUSIONS: The outcome of the project is to promote more open conversations regarding COVID-19 vaccination. We hope that our approach will encourage more governments and public health experts to engage in their mission to reach their citizens directly with digital health solutions and to consider such interventions as an important tool in infodemic management.
INTERNATIONAL REGISTERED REPORT IDENTIFIER (IRRID): PRR1-10.2196/40753.

©Javier A Elkin, Michelle McDowell, Brian Yau, Sandra Varaidzo Machiri, Shanthi Pal, Sylvie Briand, Derrick Muneene, Tim Nguyen, Tina D Purnat. Originally published in JMIR Research Protocols (https://www.researchprotocols.org), 08.03.2023.

DOI: 10.2196/40753
PMCID: PMC9997707
PMID: 36884269

Conflict of interest statement: Conflicts of Interest: None declared.

201. Mol Psychiatry. 2023 Mar 3:1-8. doi: 10.1038/s41380-023-02004-3. Online ahead of print.

Association of fluvoxamine with mortality and symptom resolution among inpatients with COVID-19 in Uganda: a prospective interventional open-label cohort study.

Kirenga BJ(1)(2), Mugenyi L(3)(4), Sánchez-Rico M(5), Kyobe H(6), Muttamba W(3)(7), Mugume R(3), Mwesigwa E(3)(8), Kalimo E(8), Nyombi V(8), Segawa I(3), Namakula LO(3)(8), Sekibira R(3), Kabweru W(8), Byanyima R(8), Aanyu H(8), Byakika-Kibwika P(9), Mwebesa HG(6), Hoertel N(5)(10)(11), Bazeyo W(12).

Author information:
(1)Department of Internal Medicine, Makerere University, Kampala, Uganda. brucekirenga@yahoo.co.uk.
(2)Makerere University Lung Institute, Kampala, Uganda. brucekirenga@yahoo.co.uk.
(3)Makerere University Lung Institute, Kampala, Uganda.
(4)Medical Research Council, Uganda Virus Research Institute and London School of Hygiene and Tropical Medicine, Entebbe Unit, Entebbe, Uganda.
(5)Assistance Publique-Hôpitaux de Paris (AP-HP), DMU Psychiatrie et Addictologie, Hôpital Corentin-Celton, F-92130, Issy-les-Moulineaux, France.
(6)Ministry of Health Uganda, Kampala, Uganda.
(7)Division of Infection and Global Health, School of Medicine, University of St Andrews, St Andrews, UK.
(8)Mulago National Referral Hospital, Kampala, Uganda.
(9)Department of Internal Medicine, Makerere University, Kampala, Uganda.
(10)Université Paris Cité, Paris, France.
(11)INSERM U1266, Institut de Psychiatrie et Neuroscience de Paris, Paris, France.
(12)Makerere University, Kampala, Uganda.

Prior research suggests that fluvoxamine, a selective serotonin reuptake inhibitor (SSRI) used for the treatment of obsessive-compulsive disorder and major depressive disorder, could be repurposed against COVID-19. We undertook a prospective interventional open-label cohort study to evaluate the efficacy and tolerability of fluvoxamine among inpatients with laboratory-confirmed COVID-19 in Uganda. The main outcome was all-cause mortality. Secondary outcomes were hospital discharge and complete symptom resolution. We included 316 patients, of whom 94 received fluvoxamine in addition to standard care [median age, 60 years (IQR = 37.0); women, 52.2%]. Fluvoxamine use was significantly associated with reduced mortality [AHR = 0.32; 95% CI = 0.19-0.53; p < 0.001, NNT = 4.46] and with increased complete symptom resolution [AOR = 2.56; 95% CI = 1.53-5.51;

p < 0.001, NNT = 4.44]. Sensitivity analyses yielded similar results. These effects did not significantly differ by clinical characteristic, including vaccination status. Among the 161 survivors, fluvoxamine was not significantly associated with time to hospital discharge [AHR 0.81, 95% CI (0.54-1.23), p = 0.32]. There was a trend toward greater side effects with fluvoxamine (7.45% versus 3.15%; SMD = 0.21; $\chi 2 = 3.46$, p = 0.06), most of which were light or mild in severity and none of which were serious. One hundred mg of fluvoxamine prescribed twice daily for 10 days was well tolerated and significantly associated with reduced mortality and with increased complete symptom resolution, without a significant increase in time to hospital discharge, among inpatients with COVID-19. Large-scale randomized trials are urgently needed to confirm these findings, especially for low- and middle-income countries, where access to vaccines and approved treatments against COVID-19 is limited.

© 2023. The Author(s).

DOI: 10.1038/s41380-023-02004-3
PMCID: PMC9982784
PMID: 36869228

Conflict of interest statement: NH is an inventor on a patent application related to methods of treating COVID-19 (FIASMA antidepressants), filled by Assistance Publique-Hopitaux de Paris in France. The other authors have no conflicts of interest related to this work to declare.

202. Afr Health Sci. 2023 Mar;23(1):1-15. doi: 10.4314/ahs.v23i1.2.

Staging of COVID-19 disease; using selected laboratory profiles for prediction, prevention and management of severe SARS-CoV-2 infection in Africa-review.

Sendagire H(1)(2)(3), Kiwuwa S(1), Dhamani A(1), Akugizibwe R(1), Lwasa Y(1), Bukenya A(1), Mukasa HK(2), Kakeeto P(4), Nankinga Z(2), Bbosa G(1), Babirye J(1), Nankabirwa H(5), Nabadda S(3).

Author information:
(1)College of Health Sciences, Makerere University, Kampala, Uganda.
(2)Faculty of Health Sciences, Islamic University in Uganda.
(3)National Health Laboratory and Diagnostics Services, Ministry of Health, Uganda.
(4)Mengo Hospital, Kampala, Uganda.
(5)Mulago National Specialized Referral Hospital, Kampala, Uganda.

There are many uncertainties on the future management of the coronavirus disease 19 (COVID-19) in Africa. By July 2021, Africa had lagged behind the rest of the world in Covid-19 vaccines uptake, accounting for just 1.6% of doses administered globally. During that time COVID 19 was causing an average death

rate of 2.6% in Africa, surpassing the then global average of 2.2%. There were no clear therapeutic guidelines, yet inappropriate and unnecessary treatments may have led to unwanted adverse events such as worsening of hyperglycemia and precipitating of ketoacidosis in administration of steroid therapy. in order to provide evidence-based policy guidelines, we examined peer-reviewed published articles in PubMed on COVID 19, or up-to date data, we focused our search on publications from 1st May 2020 to 15th July, 2021. For each of the studies, we extracted data on pathophysiology, selected clinical chemistry and immunological tests, clinical staging and treatment. Our review reports a gross unmet need for vaccination, inadequate laboratory capacity for immunological tests and the assessment of individual immune status, clinical staging and prediction of disease severity. We recommend selected laboratory tools in the assessment of individual immune status, prediction of disease severity and determination of the exact timing for suitable therapy, especially in individuals with co-morbidities.

© 2023 Sendagire H et al.

DOI: 10.4314/ahs.v23i1.2
PMCID: PMC10398495
PMID: 37545952 [Indexed for MEDLINE]

203. IJID Reg. 2023 Feb 3;6:171-176. doi: 10.1016/j.ijregi.2023.01.013. eCollection 2023 Mar.

Arbovirus circulation, epidemiology and spatiotemporal distribution in Uganda.

Byaruhanga T(1)(2), Kayiwa JT(2), Nankya AM(2), Ataliba IJ(2), McClure CP(1), Ball JK(1), Lutwama JJ(2).

Author information:
(1)University of Nottingham School of Life Sciences, Wolfson Centre for Global Virus Research, Nottingham, UK.
(2)Uganda Virus Research Institute, Department of Arbovirology, Emerging and Re-emerging infectious diseases.

BACKGROUND: Arboviruses are endemic in Uganda; however, little is known about their epidemiology, seasonality and spatiotemporal distribution. Our study sought to provide information on arbovirus outbreaks from acute clinical presentations.
METHODS: Immunoglobulin M (IgM) and confirmatory Plaque Reduction Neutralisation Test (PRNT) results for arbovirus diagnosis of samples collected from patients attending sentinel sites from 2016-19 were analysed retrospectively. Demographic data were analysed with SaTScan and SPSS software to determine the epidemiology and spatiotemporal distribution of arboviruses.
RESULTS: Arbovirus activity peaked consistently during March-May rainy seasons.

Overall, arbovirus seroprevalence was 9.5%. Of 137 IgM positives, 52.6% were confirmed by PRNT, of which 73.6% cases were observed in central Uganda with Yellow Fever Virus had the highest prevalence (27.8%). The 5-14 age group were four times more likely to be infected with an arbovirus p=0.003, 4.1 (95% CI 1.3-12.3). Significant arboviral activity was observed among outdoor workers(p=0.05) . Spatiotemporal analysis indicated arboviral activity in 23 of the 85 districts analysed..
INTERPRETATION: Our study shows that arbovirus activity peaks during the March-May rainy season and highlights the need for YFV mass vaccination to reduce the clinical burden of arboviruses transmitted within the region.

© 2023 The Authors.

DOI: 10.1016/j.ijregi.2023.01.013
PMCID: PMC10006739
PMID: 36915800

Conflict of interest statement: All authors declare no conflict of interest.

204. Lancet Microbe. 2023 Mar;4(3):e171-e178. doi: 10.1016/S2666-5247(23)00001-0. Epub 2023 Feb 2.

Species-specific immunogenicity and protective efficacy of a vesicular stomatitis virus-based Sudan virus vaccine: a challenge study in macaques.

Marzi A(1), Fletcher P(2), Feldmann F(3), Saturday G(3), Hanley PW(3), Feldmann H(4).

Author information:
(1)Laboratory of Virology, Division of Intramural Research, National Institute of Allergy and Infectious Diseases, National Institutes of Health, Rocky Mountain Laboratories, Hamilton, MT, USA. Electronic address: marzia@niaid.nih.gov.
(2)Laboratory of Virology, Division of Intramural Research, National Institute of Allergy and Infectious Diseases, National Institutes of Health, Rocky Mountain Laboratories, Hamilton, MT, USA.
(3)Rocky Mountain Veterinary Branch, Division of Intramural Research, National Institute of Allergy and Infectious Diseases, National Institutes of Health, Rocky Mountain Laboratories, Hamilton, MT, USA.
(4)Laboratory of Virology, Division of Intramural Research, National Institute of Allergy and Infectious Diseases, National Institutes of Health, Rocky Mountain Laboratories, Hamilton, MT, USA. Electronic address: feldmannh@niaid.nih.gov.

BACKGROUND: The recent Sudan virus (SUDV) outbreak in Uganda highlights the need for rapid response capabilities, including development of vaccines against

emerging viruses with high public health impact. We aimed to develop a Sudan virus-specific vaccine suitable for emergency use during outbreaks.
METHODS: We generated and characterised a vesicular stomatitis virus (VSV)-based vaccine, VSV- SUDV, and evaluated the protective efficacy following a single-dose vaccination against lethal SUDV infection in non-human primates (NHPs). We used male and female cynomolgus macaques (n=11) aged 6-11 years and weighing 3·8-9·0 kg. Animals received a 1 mL intramuscular injection for vaccination containing either 1 × 107 plaque forming units (PFU) VSV-SUDV or 1 × 107 PFU of a VSV-based vaccine against Marburg virus (control; five NHPs). NHPs were challenged intramuscularly 28 days after vaccination with 1 × 104 TCID50 SUDV-Gulu. We assessed anaesthetised NHPs on days 28, 21, 14, and 7 before challenge; days 0, 3, 6, 9, 14, 21, 28, and 35 after challenge; and at euthanasia (day 40 for survivors). As we repurposed NHPs from a successful VSV-Ebola virus (EBOV) vaccine efficacy study, we also investigated VSV-EBOV's cross-protective potential against SUDV challenge.
FINDINGS: Of the six NHPs given VSV-SUDV, none showed any signs of disease in response to the challenge. Four of the five NHPs in the control group developed characteristic clinical signs of Sudan virus diseases. SUDV glycoprotein-specific IgG concentrations peaked 14 days after vaccination (titre of >1:10 000) and reached their highest concentrations at 6 days after challenge (1:25 600-1:102 400). Although the NHPs developed cross-reactive humoral responses to SUDV after VSV-EBOV vaccination and EBOV challenge, there was little cross-protection.
INTERPRETATION: These data emphasise the need for species-specific vaccines for each human-pathogenic Ebolavirus. Furthermore, although previous VSV-EBOV immunity is boosted through VSV-SUDV vaccination, it only has a small effect on the immunogenicity and protective efficacy of VSV-SUDV vaccination against SUDV challenge.
FUNDING: Intramural Research Program, US National Institute of Allergy and Infectious Diseases, National Institutes of Health.

Copyright © 2023 The Author(s). Published by Elsevier Ltd. This is an Open Access article under the CC BY-NC-ND 4.0 license. Published by Elsevier Ltd.. All rights reserved.

DOI: 10.1016/S2666-5247(23)00001-0
PMCID: PMC10010116
PMID: 36739878 [Indexed for MEDLINE]

Conflict of interest statement: Declaration of interests HF claims intellectual property of VSV-based filovirus vaccines. All other authors declare no competing interests.

205. Trends Immunol. 2023 Mar;44(3):172-187. doi: 10.1016/j.it.2023.01.005. Epub 2023 Jan 11.

COVID-19 vaccines and a perspective on Africa.

Mantovani A(1), Rescigno M(2), Forni G(3), Tognon F(4), Putoto G(5), Ictho J(6), Lochoro P(7).

Author information:
(1)IRCCS Humanitas Research Hospital, via Manzoni 56, 20089 Rozzano, Milan, Italy; Department of Biomedical Sciences, Humanitas University, Via Rita Levi Montalcini 4, 20072 Pieve Emanuele, Milan, Italy; William Harvey Research Institute, Queen Mary University, London EC1M 6BQ, UK. Electronic address: alberto.mantovani@humanitasresearch.it.
(2)IRCCS Humanitas Research Hospital, via Manzoni 56, 20089 Rozzano, Milan, Italy; Department of Biomedical Sciences, Humanitas University, Via Rita Levi Montalcini 4, 20072 Pieve Emanuele, Milan, Italy.
(3)Accademia Nazionale dei Lincei, Rome, Italy.
(4)Operational Research Unit, Doctors with Africa CUAMM, Italy.
(5)Head of Planning and Operational Research, Doctors with Africa CUAMM, Italy.
(6)Clinical Epidemiology, Doctors with Africa CUAMM, Uganda.
(7)Health Service Management, Doctors with Africa CUAMM, Uganda. Electronic address: p.lochoro@cuamm.org.

Vaccines have dramatically changed the COVID-19 pandemic. Over 30 vaccines that were developed on four main platforms are currently being used globally, but a deep dissection of the immunological mechanisms by which they operate is limited to only a few of them. Here, we review the evidence describing specific aspects of the modes of action of COVID-19 vaccines; these include innate immunity, trained innate immunity, and mucosal responses. We also discuss the use of COVID-19 vaccines in the African continent which is ridden with inequality in its access to vaccines and vaccine-related immunological research. We argue that strengthening immunology research in Africa should inform on fundamental aspects of vaccination, including the relevance of genetics, trained innate immunity, and microbiome diversity.

Copyright © 2023 The Authors. Published by Elsevier Ltd.. All rights reserved.

DOI: 10.1016/j.it.2023.01.005
PMCID: PMC9832054
PMID: 36709083 [Indexed for MEDLINE]

Conflict of interest statement: Declaration of interests No interests are declared.

206. IJID Reg. 2023 Mar;6:99-107. doi: 10.1016/j.ijregi.2023.01.003. Epub 2023 Jan 9.

Compartmental mathematical modelling of dynamic transmission of COVID-19 in Rwanda.

Mpinganzima L(1), Ntaganda JM(1), Banzi W(1), Muhirwa JP(1), Nannyonga BK(2), Niyobuhungiro J(3), Rutaganda E(4), Ngaruye I(1), Ndanguza D(1), Nzabanita J(1), Masabo E(1)(5), Gahamanyi M(1), Dushimirimana J(1), Nyandwi B(1), Kurujyibwami C(1), Ruganzu LFU(1), Nyagahakwa V(1), Mukeshimana S(1), Ngendahayo JP(1), Nsabimana JP(1), Niyigena JD(1), Uwonkunda J(6), Mbalawata IS(7).

Author information:
(1)Department of Mathematics, School of Science, College of Science and Technology, University of Rwanda, Kigali, Rwanda.
(2)Department of Mathematics, School of Physical Sciences, College of Natural Sciences, Makerere University, Kampala, Uganda.
(3)National Council for Science and Technology, Kigali, Rwanda.
(4)Department of Internal Medicine, Kigali University Teaching Hospital, Kigali, Rwanda.
(5)African Centre of Excellence in Data Science, Kigali, Rwanda.
(6)Great Mind for Career Development Group Ltd, Kigali, Rwanda.
(7)African Institute of Mathematical Sciences, NEI Global Secretariat, Kigali, Rwanda.

OBJECTIVES: Mathematical modelling is of interest to study the dynamics of coronavirus disease 2019 (COVID-19), and models such as SEIR (Susceptible-Exposed-Infected-Recovered) have been considered. This article describes the development of a compartmental transmission network model - Susceptible-Exposed-Quarantine-Infectious-Infectious, undetected-Infectious, home-based care-Hospitalized-Vaccinated-Recovered-Dead - to simulate the dynamics of COVID-19 in order to account for specific measures put into place by the Government of Rwanda to prevent further spread of the disease.
METHODS: The compartments of this model are connected by parameters, some of which are known from the literature, and others are estimated from available data using the least squares method. For the stability of the model, equilibrium points were determined and the basic reproduction number R0 was studied; R 0 is an indicator for contagiousness.
RESULTS: The model showed that secondary infections are generated from the exposed group, the asymptomatic group, the infected (symptomatic) group, the infected (undetected) group, the infected (home-based care) group and the hospitalized group. The formulated model was reliable and fit the data. Furthermore, the estimated R0 of 2.16 shows that COVID-19 will persist without the application of control measures.
CONCLUSIONS: This article presents results regarding predicted spread of COVID-19 in Rwanda.

© 2023 The Author(s).

DOI: 10.1016/j.ijregi.2023.01.003
PMCID: PMC9827742
PMID: 36644499

Conflict of interest statement: The authors declare that they have no known competing financial interests or personal relationships that could have appeared to influence the work reported in this paper.

207. AIDS Behav. 2023 Mar;27(3):880-890. doi: 10.1007/s10461-022-03824-0. Epub 2022 Sep 10.

Factors Associated with Short and Long Term Mobility and HIV Risk of Women Living in Fishing Communities Around Lake Victoria in Kenya, Tanzania, and Uganda: A Cross Sectional Survey.

Bahemuka UM(1), Okimat P(2), Webb EL(3), Seeley J(2)(3), Ssetaala A(4), Okech B(4), Oketch B(5), Kibengo FM(2), Okello E(6), Kwena Z(5), Kuteesa MO(7), Price MA(7)(8), Kaleebu P(2)(3), Grosskurth H(3)(6), Fast P(7); LVCHR.

Author information:
(1)Medical Research Council/Uganda Virus Research Institute Uganda Research Unit & London School of Hygiene and Tropical Medicine (MRC/UVRI & LSHTM) Uganda Research Unit, P.O. Box 49, Entebbe, Uganda. ubaldo.bahemuka@mrcuganda.org.
(2)Medical Research Council/Uganda Virus Research Institute Uganda Research Unit & London School of Hygiene and Tropical Medicine (MRC/UVRI & LSHTM) Uganda Research Unit, P.O. Box 49, Entebbe, Uganda.
(3)London School of Hygiene and Tropical Medicine, London, UK.
(4)UVRI-IAVI HIV Vaccine Program, Entebbe, Uganda.
(5)Kenya Medical Research Institute, KEMRI, Kisumu, Kenya.
(6)Mwanza Intervention Trials Unit, Mwanza, Tanzania.
(7)International AIDS Vaccine Initiative, New York, USA.
(8)University of California, San Francisco, San Francisco, USA.

Mobility is linked to negative HIV care continuum outcomes. We sought to understand factors associated with short and long term mobility among women in fishing communities in Kenya, Tanzania, and Uganda. From 2018 through 2019 we conducted a cross-sectional survey of women aged 15 years and above, randomly selected from a census of six fishing villages, around Lake Victoria. Data collected included: demographics, risky sexual behaviour on the most recent trip, and travel behaviour in the previous 4 months. Mobility was recorded as any overnight trip outside the participant's village. A two-level multinomial logistic regression model was used to determine the associated factors. A total of 901 participants were enrolled, of whom 645 (71.6%) reported travelling (53.4%; short and 18.2% long term trips). Five factors were associated with long term travel: age, travel purpose, frequency of travel, sexual behaviour while travelling, and destination. Trips made by women aged 46-75 years were less likely to be long term. Long term trips were more common if the trip was to visit, rather than to trade, and more common for women who reported one or two trips rather than three or more trips. Women who made long term trips were more

likely to engage in unprotected sex while on a trip. Women who travelled to a regional town/district or another town/district were more likely to take long term trips. The factors associated with travel duration among women living in fishing communities could inform planning of future health care interventions in these communities.

© 2022. The Author(s).

DOI: 10.1007/s10461-022-03824-0
PMCID: PMC9944640
PMID: 36088399 [Indexed for MEDLINE]

Conflict of interest statement: The authors declare no conflict of interest.

208. AIDS Behav. 2023 Mar;27(3):1030-1043. doi: 10.1007/s10461-022-03841-z. Epub 2022 Sep 6.

Correlates of Dapivirine Vaginal Ring Acceptance among Women Participating in an Open Label Extension Trial.

Mirembe BG(1)(2)(3), Cabrera MV(4), van der Straten A(5)(6), Nakalega R(7), Cobbing M(8), Mgodi NM(9), Palanee-Phillips T(10), Mayo AJ(11), Dadabhai S(12), Mansoor LE(13), Siva S(8), Nair G(14), Chinula L(15), Akello CA(7), Nakabiito C(7), Soto-Torres LE(16), Baeten JM(17)(18), Brown ER(4)(19).

Author information:
(1)Makerere University-Johns Hopkins University Research Collaboration, Kampala, Uganda. bgati@mujhu.org.
(2)Vaccine and Infectious Disease and Public Health Sciences Divisions, Fred Hutchinson Cancer Research Center, Seattle, WA, USA. bgati@mujhu.org.
(3)MU-JHU research Collaboration/MU-JHU CARE LTD, P.o. Box 23491, Kampala, Uganda. bgati@mujhu.org.
(4)Department of Biostatistics, University of Washington, Seattle, WA, USA.
(5)Center for AIDS Prevention Studies, University of California San Francisco, San Francisco, CA, USA.
(6)ASTRA Consulting, Kensington, CA, USA.
(7)Makerere University-Johns Hopkins University Research Collaboration, Kampala, Uganda.
(8)HIV Prevention Research Unit, South African Medical Research Council, Durban, South Africa.
(9)University of Zimbabwe Clinical Trials Research Centre, Harare, Zimbabwe.
(10)Wits Reproductive Health and HIV Institute (Wits RHI), University of the Witwatersrand, 8 FHI 360, Johannesburg, South Africa.
(11)FHI 360, Durham, USA.
(12)College of Medicine-Johns Hopkins Research Project, Blantyre, Malawi.
(13)Centre for AIDS Programme of Research in South Africa, University of

KwaZulu-Natal, Durban, South Africa.
(14)The Desmond Tutu HIV Centre, University of Cape Town, Cape Town, South Africa.
(15)University of North Carolina (UNC) Project Malawi Clinical Research Site, Lilongwe, Malawi.
(16)Division of AIDS, National Institute of Allergy and Infectious Diseases, National Institutes of Health, Bethesda, MD, USA.
(17)Departments of Global Health, Medicine, and Epidemiology, University of Washington, Seattle, USA.
(18)Gilead Sciences, Foster City, CA, USA.
(19)Vaccine and Infectious Disease and Public Health Sciences Divisions, Fred Hutchinson Cancer Research Center, Seattle, WA, USA.

MTN-025/HOPE was an open-label trial of the dapivirine vaginal ring conducted in four African countries between 2016 and 2018. Women were first offered one ring monthly (at baseline, months 1 and 2), thereafter, transitioned to a more applicable real-world dispensation schedule, - 3 rings quarterly (at months 3, 6 and 9). Logistic regression analysis was used to assess correlates of ring acceptance at baseline and through follow-up. A total of 1456 women (median age 31 years) enrolled, 1342 (92.2%) accepted the ring at baseline and 1163 (79.9%) accepted the ring(s) at all visits. Changing ring dispensation from a monthly to a quarterly schedule had no negative effect on acceptance. Having a primary partner and him knowing about the ring being offered in HOPE, use of long-acting contraception (implants, injections, IUDs) or sterilization were associated with ring acceptance, along with prior strong intention to use the ring in the future. Efforts should consider these factors when rolling out the ring for HIV prevention.

© 2022. The Author(s), under exclusive licence to Springer Science+Business Media, LLC, part of Springer Nature.

DOI: 10.1007/s10461-022-03841-z
PMCID: PMC10102709
PMID: 36066762 [Indexed for MEDLINE]

Conflict of interest statement: Declarations Conflicts of interest/Competing interests The authors have no relevant financial or non-financial interests to disclose.

209. medRxiv. 2023 Feb 26:2023.02.24.23284435. doi: 10.1101/2023.02.24.23284435. Preprint.

Schistosoma mansoni infection alters the host pre-vaccination environment resulting in blunted Hepatitis B vaccination immune responses.

Muir R, Metcalf T, Fourati S, Bartsch Y, Lugemwa JK, Canderan G, Alter G,

Muyanja E, Okech B, Namatovu T, Namara I, Namuniina A, Ssetaala A, Mpendo J, Nanvubya A, Kitandwe PK, Bagaya BS, Kiwanuka N, Nassuna J, Biribawa VM, Elliott AM, de Dood CJ, Senyonga W, Balungi P, Kaleebu P, Mayanja Y, Odongo M, Fast P, Price MA, Corstjens PLAM, van Dam GJ, Kamali A, Sekaly RP, Haddad EK.

Update in
PLoS Negl Trop Dis. 2023 Jul 5;17(7):e0011089.

The impact of endemic infections on protective immunity is critical to inform vaccination strategies. In this study, we assessed the influence of Schistosoma mansoni infection on host responses in a Ugandan fishing cohort given a Hepatitis B (HepB) vaccine. Concentrations of schistosome-specific circulating anodic antigen (CAA) pre-vaccination showed a significant bimodal distribution associated with HepB titers, which were lower in individuals with high CAA. We established that participants with high CAA had significantly lower frequencies of circulating T follicular helper (cTfh) subpopulations pre- and post-vaccination and higher regulatory T cells (Tregs) post-vaccination. Polarization towards higher frequencies of Tregs: cTfh cells can be mediated by changes in the cytokine environment favoring Treg differentiation. In fact, we observed higher levels of CCL17 and soluble IL-2R pre-vaccination (important for Treg recruitment and development), in individuals with high CAA that negatively associated with HepB titers. Additionally, alterations in pre-vaccination monocyte function correlated with HepB titers, and changes in innate-related cytokines/chemokine production were associated with increasing CAA concentration. We report, that by influencing the immune landscape, schistosomiasis has the potential to modulate immune responses to HepB vaccination. These findings highlight multiple Schistosoma -related immune associations that could explain abrogated vaccine responses in communities with endemic infections.
AUTHOR SUMMARY: Schistosomiasis drives host immune responses for optimal pathogen survival, potentially altering host responses to vaccine-related antigen. Chronic schistosomiasis and co-infection with hepatotropic viruses are common in countries where schistosomiasis is endemic. We explored the impact of Schistosoma mansoni (S. mansoni) infection on Hepatitis B (HepB) vaccination of individuals from a fishing community in Uganda. We demonstrate that high schistosome-specific antigen (circulating anodic antigen, CAA) concentration pre-vaccination, is associated with lower HepB antibody titers post-vaccination. We show higher pre-vaccination levels of cellular and soluble factors in instances of high CAA that are negatively associated with HepB antibody titers post-vaccination, which coincided with lower frequencies of circulating T follicular helper cell populations (cTfh), proliferating antibody secreting cells (ASCs), and higher frequencies of regulatory T cells (Tregs). We also show that monocyte function is important in HepB vaccine responses, and that high CAA is associated with alterations in the early innate cytokine/chemokine microenvironment. Our findings suggest that in individuals with high CAA and likely high worm burden, schistosomiasis creates and sustains an environment that is polarized against optimal host immune responses to the vaccine, which

puts many endemic communities at risk for infection against HepB and other diseases that are preventable by vaccines.

DOI: 10.1101/2023.02.24.23284435
PMCID: PMC9980246
PMID: 36865336

210. PLOS Glob Public Health. 2023 Feb 15;3(2):e0000785. doi: 10.1371/journal.pgph.0000785. eCollection 2023.

Health facility assessments of cervical cancer prevention, early diagnosis, and treatment services in Gulu, Uganda.

Chongsuwat T(1), Ibrahim AO(2), Evensen AE(1), Conway JH(3), Zwick M(1), Oloya W(2).

Author information:
(1)Department of Family Medicine and Community Health, University of Wisconsin-Madison School of Medicine & Public Health, Madison, Wisconsin, United States of America.
(2)Gulu Women's Economic Development & Globalization (GWED-G), Gulu, Uganda.
(3)Department of Pediatrics, University of Wisconsin-Madison School of Medicine & Public Health, Madison, Wisconsin, United States of America.

BACKGROUND: Cervical cancer is ranked globally in the top three cancers for women younger than 45 years, with the average age of death at 59 years of age. The highest burden of disease is in low-to-middle income countries (LMICs), responsible for 90% of the 311,000 cervical cancer deaths in 2018. This growing health disparity is due to the lack of quality screening and treatment programs, low human papillomavirus (HPV) vaccination rates, and high human immunodeficiency virus (HIV) co-infection rates. To address these gaps in care, we need to develop a clear understanding of the resources and capabilities of LMICs' health care facilities to provide prevention, early diagnosis through screening, and treatment for cervical cancer.
OBJECTIVES: This project aimed to assess baseline available cervical cancer prevention, early diagnosis, and treatment resources, at facilities designated as Health Center III or above, in Gulu, Uganda.
METHODS: We adapted the World Health Organization's Harmonized Health Facility Assessment for our own HFA and grading scale, deploying it in October 2021 for a cross-sectional analysis of 21 health facilities in Gulu.
RESULTS: Grading of Health Center IIIs (n = 16) concluded that 37% had "excellent" or "good" resources available, and 63% of facilities had "poor" or "fair" resources available. Grading of Health Center IVs and above (n = 5) concluded that 60% of facilities had "excellent" or "good" resources, and 40% had "fair" resources available.
DISCUSSION: The analysis of health facilities in Gulu demonstrated subpar

resources available for cervical cancer prevention, early diagnosis, and treatment. Focused efforts are needed to expand health centers' resources and capability to address rising cervical cancer rates and related health disparities in LMICs. The development process for this project's HFA can be applied to global cervical cancer programming to determine gaps in resources and indicate areas to target improved health equity.

Copyright: © 2023 Chongsuwat et al. This is an open access article distributed under the terms of the Creative Commons Attribution License, which permits unrestricted use, distribution, and reproduction in any medium, provided the original author and source are credited.

DOI: 10.1371/journal.pgph.0000785
PMCID: PMC10021907
PMID: 36962762

Conflict of interest statement: The authors have declared that no competing interests exist.

211. Front Public Health. 2023 Feb 15;11:942703. doi: 10.3389/fpubh.2023.942703. eCollection 2023.

Digital health in the era of COVID-19: Reshaping the next generation of healthcare.

Getachew E(1)(2), Adebeta T(3), Muzazu SGY(1)(4), Charlie L(1)(5), Said B(1)(6), Tesfahunei HA(1)(7), Wanjiru CL(1), Acam J(1)(8), Kajogoo VD(1), Solomon S(9), Atim MG(1)(10), Manyazewal T(1).

Author information:
(1)Center for Innovative Drug Development and Therapeutic Trials for Africa (CDT-Africa), College of Health Sciences, Addis Ababa University, Addis Ababa, Ethiopia.
(2)Department of Public Health, College of Health Science, Arsi University, Asella, Ethiopia.
(3)Outpatient Department, Ethiopian Airlines Medical Unit, Addis Ababa, Ethiopia.
(4)Enteric Diseases and Vaccines Research Unit, Centre for Infectious Disease Research in Zambia (CIDRZ), Lusaka, Zambia.
(5)KNCV TB Foundation, Challenge TB Project, Blantyre, Malawi.
(6)Outpatient Department, Kibong'oto National Tuberculosis Hospital, Moshi, Kilimanjaro, Tanzania.
(7)Department of Public Health, Hager Biomedical Research Institute, Asmara, Eritrea.
(8)Outpatient Department, Pope John's Hospital Aber, Atapara, Uganda.
(9)School of Public Health, Saint Paul's Hospital Millennium Medical College,

Addis Ababa, Ethiopia.
(10)Soroti Regional Referral Hospital, Soroti, Uganda.

COVID-19 is one of the most deadly diseases to have stricken us in recent decades. In the fight against this disease, governments and stakeholders require all the assistance they can get from various systems, including digital health interventions. Digital health technologies are supporting the tracking of the COVID-19 outbreak, diagnosing patients, expediting the process of finding potential medicines and vaccines, and disinfecting the environment, The establishment of electronic medical and health records, computerized clinical decision support systems, telemedicine, and mobile health have shown the potential to strengthen the healthcare system. Recently, these technologies have aided the health sector in a variety of ways, including prevention, early diagnosis, treatment adherence, medication safety, care coordination, documentation, data management, outbreak tracking, and pandemic surveillance. On the other hand, implementation of such technologies has questions of cost, compatibility with existing systems, disruption in patient-provider interactions, and sustainability, calling for more evidence on clinical utility and economic evaluations to help shape the next generation of healthcare. This paper argues how digital health interventions assist in the fight against COVID-19 and their opportunities, implications, and limitations.

Copyright © 2023 Getachew, Adebeta, Muzazu, Charlie, Said, Tesfahunei, Wanjiru, Acam, Kajogoo, Solomon, Atim and Manyazewal.

DOI: 10.3389/fpubh.2023.942703
PMCID: PMC9976934
PMID: 36875401 [Indexed for MEDLINE]

Conflict of interest statement: The authors declare that the research was conducted in the absence of any commercial or financial relationships that could be construed as a potential conflict of interest.

212. Elife. 2023 Feb 15;12:e81401. doi: 10.7554/eLife.81401.

Antibodies to repeat-containing antigens in Plasmodium falciparum are exposure-dependent and short-lived in children in natural malaria infections.

Raghavan M(1), Kalantar KL(2), Duarte E(3), Teyssier N(1), Takahashi S(1), Kung AF(1), Rajan JV(1), Rek J(4), Tetteh KKA(5), Drakeley C(5), Ssewanyana I(4)(5), Rodriguez-Barraquer I(1)(6), Greenhouse B(1)(6), DeRisi JL(1)(6).

Author information:
(1)University of California, San Francisco, San Francisco, United States.
(2)Chan Zuckerberg Initiative, Redwood City, United States.
(3)University of California, Berkeley, Berkeley, United States.

(4)Infectious Diseases Research Collaboration, Kampala, Uganda.
(5)London School of Hygiene and Tropical Medicine, London, United Kingdom.
(6)Chan Zuckerberg Biohub, San Francisco, United States.

Update of
doi: 10.1101/2022.06.24.497532.

Protection against Plasmodium falciparum, which is primarily antibody-mediated, requires recurrent exposure to develop. The study of both naturally acquired limited immunity and vaccine induced protection against malaria remains critical for ongoing eradication efforts. Towards this goal, we deployed a customized P. falciparum PhIP-seq T7 phage display library containing 238,068 tiled 62-amino acid peptides, covering all known coding regions, including antigenic variants, to systematically profile antibody targets in 198 Ugandan children and adults from high and moderate transmission settings. Repeat elements - short amino acid sequences repeated within a protein - were significantly enriched in antibody targets. While breadth of responses to repeat-containing peptides was twofold higher in children living in the high versus moderate exposure setting, no such differences were observed for peptides without repeats, suggesting that antibody responses to repeat-containing regions may be more exposure dependent and/or less durable in children than responses to regions without repeats.
Additionally, short motifs associated with seroreactivity were extensively shared among hundreds of antigens, potentially representing cross-reactive epitopes. PfEMP1 shared motifs with the greatest number of other antigens, partly driven by the diversity of PfEMP1 sequences. These data suggest that the large number of repeat elements and potential cross-reactive epitopes found within antigenic regions of P. falciparum could contribute to the inefficient nature of malaria immunity.

© 2023, Raghavan et al.

DOI: 10.7554/eLife.81401
PMCID: PMC10005774
PMID: 36790168 [Indexed for MEDLINE]

Conflict of interest statement: MR, KK, ED, NT, ST, AK, JR, JR, KT, CD, IS, BG No competing interests declared, IR Reviewing editor, eLife, JD Paid scientific advisor for Allen & Co. Paid scientific advisor for the Public Health Company, Inc and holds stock options. Founder and holding stock options in VeriPhi Health, Inc

213. Front Neurol. 2023 Feb 14;14:822575. doi: 10.3389/fneur.2023.822575. eCollection 2023.

Bacterial meningitis in Africa.

Barichello T(1)(2), Rocha Catalão CH(2)(3), Rohlwink UK(4)(5)(6), van der Kuip M(7), Zaharie D(8)(9), Solomons RS(10), van Toorn R(10), Tutu van Furth M(7), Hasbun R(11), Iovino F(12), Namale VS(13)(14).

Author information:
(1)Graduate Program in Health Sciences, University of Southern Santa Catarina (UNESC), Criciúma, SC, Brazil.
(2)Faillace Department of Psychiatry and Behavioral Sciences, McGovern Medical School, The University of Texas Health Science Center at Houston, Houston, TX, United States.
(3)Department of Neuroscience and Behavioral Science, Ribeirao Preto Medical School, University of São Paulo (USP), Ribeirao Preto, SP, Brazil.
(4)Pediatric Neurosurgery Unit, Red Cross War Memorial Children's Hospital, Cape Town, South Africa.
(5)Division of Neurosurgery, University of Cape Town, Cape Town, South Africa.
(6)Neuroscience Institute, University of Cape Town, Cape Town, South Africa.
(7)Department of Pediatric Infectious Diseases and Immunology, Amsterdam Infection and Immunity Institute, Amsterdam University Medical Centers, Vrije Universiteit, Amsterdam, Netherlands.
(8)Department of Anatomical Pathology, Faculty of Medicine and Health Sciences, Stellenbosch University, Cape Town, South Africa.
(9)National Health Laboratory Services, Tygerberg Hospital, Cape Town, South Africa.
(10)Department of Pediatric and Child Health, Faculty of Medicine and Health Sciences, Stellenbosch University, Cape Town, South Africa.
(11)Division of Infectious Diseases, Department of Internal Medicine, UT Health, McGovern Medical School, Houston, TX, United States.
(12)Department of Neuroscience, Karolinska Institutet, Stockholm, Sweden.
(13)Columbia University Irving Medical Center and New York Presbyterian Hospital, New York, NY, United States.
(14)Department of Paediatrics and Child Health, Makerere University College of Health Sciences, Kampala, Uganda.

Bacterial meningitis differs globally, and the incidence and case fatality rates vary by region, country, pathogen, and age group; being a life-threatening disease with a high case fatality rate and long-term complications in low-income countries. Africa has the most significant prevalence of bacterial meningitis illness, and the outbreaks typically vary with the season and the geographic location, with a high incidence in the meningitis belt of the sub-Saharan area from Senegal to Ethiopia. Streptococcus pneumoniae (pneumococcus) and Neisseria meningitidis (meningococcus) are the main etiological agents of bacterial meningitis in adults and children above the age of one. Streptococcus agalactiae (group B Streptococcus), Escherichia coli, and Staphylococcus aureus are neonatal meningitis's most common causal agents. Despite efforts to vaccinate against the most common causes of bacterial neuro-infections, bacterial meningitis remains a significant cause of mortality and morbidity in Africa, with children below 5 years bearing the heaviest disease burden. The factors

attributed to this continued high disease burden include poor infrastructure, continued war, instability, and difficulty in diagnosis of bacterial neuro-infections leading to delay in treatment and hence high morbidity. Despite having the highest disease burden, there is a paucity of African data on bacterial meningitis. In this article, we discuss the common etiologies of bacterial neuroinfectious diseases, diagnosis and the interplay between microorganisms and the immune system, and the value of neuroimmune changes in diagnostics and therapeutics.

Copyright © 2023 Barichello, Rocha Catalão, Rohlwink, Kuip, Zaharie, Solomons, van Toorn, Tutu van Furth, Hasbun, Iovino and Namale.

DOI: 10.3389/fneur.2023.822575
PMCID: PMC9972001
PMID: 36864913

Conflict of interest statement: The authors declare that the research was conducted in the absence of any commercial or financial relationships that could be construed as a potential conflict of interest.

214. Vaccines (Basel). 2023 Feb 14;11(2):440. doi: 10.3390/vaccines11020440.

Differential COVID-19 Vaccination Uptake and Associated Factors among the Slum and Estate Communities in Uganda: A Cross-Sectional Population-Based Survey.

Kawuki J(1), Nambooze J(2), Chan PS(1), Chen S(1), Liang X(1), Mo PKH(1), Wang Z(1).

Author information:
(1)Centre for Health Behaviours Research, Jockey Club School of Public Health and Primary Care, The Chinese University of Hong Kong, Hong Kong, China.
(2)Department of Nutritional Sciences and Dietetics, Kyambogo University, Kyambogo, Kampala P.O. Box 1, Uganda.

Vaccination against COVID-19 remains one of the ultimate solutions to the ongoing pandemic. This study examined and compared the completion of primary COVID-19 vaccination series and associated factors in the slum and estate communities of Uganda. This was a cross-sectional survey conducted among 1025 slum and estate residents. Logistic regression models were fitted. Of the 1025 participants, 511 were slum residents and 514 were estate residents. Completion of COVID-19 vaccination was 43.8% in the slum community and 39.9% in the estate community (p = 0.03). Having more knowledge about COVID-19 was positively associated with completing COVID-19 vaccination in both communities. Perceived benefits and cues to action also had a positive association, but only among the slum residents. However, perceiving people infected with COVID-19 as having a high death rate, perceived barriers such as serious side effects and long

distances, and depressive symptoms had negative associations with vaccine uptake among the slum community, but not in the estate community. Addressing barriers to vaccination, strengthening and utilizing the various cues to action, engagement of religious and cultural leaders, and continued community education and sensitization tailored to the needs of each community are potentially vital strategies in raising vaccination rates. Consideration of socioeconomic impact-alleviation strategies, especially among the urban poor, would also be beneficial.

DOI: 10.3390/vaccines11020440
PMCID: PMC9965522
PMID: 36851317

Conflict of interest statement: The authors declare no conflict of interest.

215. Immunity. 2023 Feb 14;56(2):420-432.e7. doi: 10.1016/j.immuni.2023.01.013.

Potent transmission-blocking monoclonal antibodies from naturally exposed individuals target a conserved epitope on Plasmodium falciparum Pfs230.

Ivanochko D(1), Fabra-García A(2), Teelen K(2), van de Vegte-Bolmer M(2), van Gemert GJ(2), Newton J(1), Semesi A(1), de Bruijni M(3), Bolscher J(3), Ramjith J(4), Szabat M(5), Vogt S(5), Kraft L(5), Duncan S(5), Lee SM(6), Kamya MR(7), Feeney ME(8), Jagannathan P(9), Greenhouse B(10), Sauerwein RW(3), Richter King C(6), MacGill RS(6), Bousema T(11), Jore MM(12), Julien JP(13).

Author information:
(1)Program in Molecular Medicine, the Hospital for Sick Children Research Institute, Toronto, ON, Canada.
(2)Department of Medical Microbiology, Radboudumc, Nijmegen, the Netherlands.
(3)TropIQ Health Sciences, Nijmegen, the Netherlands.
(4)Radboud Institute for Health Sciences, Department for Health Evidence, Biostatistics Section, Radboudumc, Nijmegen, the Netherlands.
(5)AbCellera Biologics Inc., Vancouver, BC, Canada.
(6)PATH's Malaria Vaccine Initiative, Washington, DC 20001, USA.
(7)Infectious Disease Research Collaboration, Kampala, Uganda.
(8)Department of Medicine, University of California, San Francisco, San Francisco, CA, USA; Department of Pediatrics, University of California, San Francisco, San Francisco, CA, USA.
(9)Department of Microbiology and Immunology, Stanford University, Stanford, CA, USA.
(10)Department of Medicine, University of California, San Francisco, San Francisco, CA, USA.
(11)Department of Medical Microbiology, Radboudumc, Nijmegen, the Netherlands. Electronic address: teun.bousema@radboudumc.nl.
(12)Department of Medical Microbiology, Radboudumc, Nijmegen, the Netherlands.

Electronic address: matthijs.jore@radboudumc.nl.
(13)Program in Molecular Medicine, the Hospital for Sick Children Research Institute, Toronto, ON, Canada; Departments of Biochemistry and Immunology, University of Toronto, Toronto, ON, Canada. Electronic address: jean-philippe.julien@sickkids.ca.

Comment in
Immunity. 2023 Feb 14;56(2):234-236.

Pfs230 is essential for Plasmodium falciparum transmission to mosquitoes and is the protein targeted by the most advanced malaria-transmission-blocking vaccine candidate. Prior understanding of functional epitopes on Pfs230 is based on two monoclonal antibodies (mAbs) with moderate transmission-reducing activity (TRA), elicited from subunit immunization. Here, we screened the B cell repertoire of two naturally exposed individuals possessing serum TRA and identified five potent mAbs from sixteen Pfs230 domain-1-specific mAbs. Structures of three potent and three low-activity antibodies bound to Pfs230 domain 1 revealed four distinct epitopes. Highly potent mAbs from natural infection recognized a common conformational epitope that is highly conserved across P. falciparum field isolates, while antibodies with negligible TRA derived from natural infection or immunization recognized three distinct sites. Our study provides molecular blueprints describing P. falciparum TRA, informed by contrasting potent and non-functional epitopes elicited by natural exposure and vaccination.

Copyright © 2023 The Authors. Published by Elsevier Inc. All rights reserved.

DOI: 10.1016/j.immuni.2023.01.013
PMCID: PMC9942874
PMID: 36792575 [Indexed for MEDLINE]

Conflict of interest statement: Declaration of interests The authors declare no competing interests.

216. Immunity. 2023 Feb 14;56(2):406-419.e7. doi: 10.1016/j.immuni.2023.01.009.

Highly potent, naturally acquired human monoclonal antibodies against Pfs48/45 block Plasmodium falciparum transmission to mosquitoes.

Fabra-García A(1), Hailemariam S(2), de Jong RM(1), Janssen K(1), Teelen K(1), van de Vegte-Bolmer M(1), van Gemert GJ(1), Ivanochko D(3), Semesi A(3), McLeod B(2), Vos MW(4), de Bruijni MHC(4), Bolscher JM(4), Szabat M(5), Vogt S(5), Kraft L(5), Duncan S(5), Kamya MR(6), Feeney ME(7), Jagannathan P(8), Greenhouse B(9), Dechering KJ(4), Sauerwein RW(4), King CR(10), MacGill RS(10), Bousema T(11), Julien JP(12), Jore MM(13).

Author information:

(1)Department of Medical Microbiology, Radboudumc, Nijmegen, the Netherlands.
(2)Program in Molecular Medicine, The Hospital for Sick Children Research Institute, Toronto, ON, Canada; Department of Biochemistry, University of Toronto, Toronto, ON, Canada.
(3)Program in Molecular Medicine, The Hospital for Sick Children Research Institute, Toronto, ON, Canada.
(4)TropIQ Health Sciences, Nijmegen, the Netherlands.
(5)AbCellera Biologics Inc., Vancouver, BC, Canada.
(6)Infectious Disease Research Collaboration, Kampala, Uganda.
(7)Department of Medicine, University of California, San Francisco, San Francisco, CA, USA; Department of Pediatrics, University of California, San Francisco, San Francisco, CA, USA.
(8)Department of Microbiology and Immunology, Stanford University, Stanford, CA, USA.
(9)Department of Medicine, University of California, San Francisco, San Francisco, CA, USA.
(10)PATH's Malaria Vaccine Initiative, Washington, DC 20001, USA.
(11)Department of Medical Microbiology, Radboudumc, Nijmegen, the Netherlands. Electronic address: teun.bousema@radboudumc.nl.
(12)Program in Molecular Medicine, The Hospital for Sick Children Research Institute, Toronto, ON, Canada; Department of Biochemistry, University of Toronto, Toronto, ON, Canada; Department of Immunology, University of Toronto, Toronto, ON, Canada. Electronic address: jean-philippe.julien@sickkids.ca.
(13)Department of Medical Microbiology, Radboudumc, Nijmegen, the Netherlands. Electronic address: matthijs.jore@radboudumc.nl.

Comment in
Immunity. 2023 Feb 14;56(2):234-236.

Malaria transmission-blocking vaccines (TBVs) aim to induce antibodies that interrupt malaria parasite development in the mosquito, thereby blocking onward transmission, and provide a much-needed tool for malaria control and elimination. The parasite surface protein Pfs48/45 is a leading TBV candidate. Here, we isolated and characterized a panel of 81 human Pfs48/45-specific monoclonal antibodies (mAbs) from donors naturally exposed to Plasmodium parasites. Genetically diverse mAbs against each of the three domains (D1-D3) of Pfs48/45 were identified. The most potent mAbs targeted D1 and D3 and achieved >80% transmission-reducing activity in standard membrane-feeding assays, at 10 and 2 µg/mL, respectively. Co-crystal structures of D3 in complex with four different mAbs delineated two conserved protective epitopes. Altogether, these Pfs48/45-specific human mAbs provide important insight into protective and non-protective epitopes that can further our understanding of transmission and inform the design of refined malaria transmission-blocking vaccine candidates.

Copyright © 2023 The Author(s). Published by Elsevier Inc. All rights reserved.

DOI: 10.1016/j.immuni.2023.01.009

PMCID: PMC9942873
PMID: 36792574 [Indexed for MEDLINE]

Conflict of interest statement: Declaration of interests The authors declare no competing interests.

217. Pathogens. 2023 Feb 10;12(2):295. doi: 10.3390/pathogens12020295.

East Coast Fever Carrier Status and Theileria parva Breakthrough Strains in Recently ITM Vaccinated and Non-Vaccinated Cattle in Iganga District, Eastern Uganda.

Oligo S(1), Nanteza A(1), Nsubuga J(1), Musoba A(1), Kazibwe A(1), Lubega GW(1).

Author information:
(1)College of Veterinary Medicine, Animal Resources and Biosecurity (COVAB), Makerere University, Kampala P.O. Box 7062, Uganda.

East Coast fever (ECF) is a tick-borne disease of cattle that hinders the development of the livestock industry in eastern, central and southern Africa. The 'Muguga cocktail' live vaccine, delivered by an infection and treatment method (ITM), remains the only immunisation strategy of controlling ECF. However, there are challenges of the live vaccine inducing ECF carrier status in immunised animals and the possibility of lack of protection from parasite strains that are antigenically different from the vaccine strains. In Uganda, there are insufficient data regarding the ECF carrier status and T. parva genetic diversity in vaccinated and associated non-vaccinated cattle to assess the effectiveness of ITM vaccination. Blood was collected from recently ECF vaccinated (98) and non-vaccinated (73) cattle from Iganga district in Eastern Uganda at 120 days post-vaccination. The p104 gene nested PCR was used to screen for T. parva DNA, 11 minisatellite and 3 microsatellite markers (SSR) were used for genotyping. Two minisatellite markers (MS7 and MS19) were used to determine whether ECF carrier status was due to the T. parva vaccine or local strains. The prevalence of T. parva based on p104 nPCR was 61.2% (60/98) (RR 2.234, 95% CI 1.49-3.35, p-value < 0.001) among recently vaccinated cattle and 27.4% (20/73) (RR 1.00) among associated non-vaccinated cattle. The Muguga cocktail vaccine strains were responsible for carrier status in 10 (58.8%) by MS7 and 11 (64.7%) by MS19 in vaccinated cattle. Genotypes of T. parva with different-sized alleles to the vaccine strains that could be potential 'breakthroughs' were detected in 2 (11.8%)) and 4 (23.5%) isolates from vaccinated cattle based on MS7 and MS19 minisatellite markers, respectively. Using 14 SSR markers, T. parva diversity was higher in vaccinated (Na = 2.214, Ne = 1.978, He = 0.465) than associated non-vaccinated (Na = 1.071, Ne = 1.048, He = 0.259) cattle. The principal component analysis (PCA) showed isolates from vaccinated cattle were closely related to those from non-vaccinated cattle. The analysis of molecular variance (AMOVA) revealed high genetic variation (96%) within T. parva isolates from

vaccinated and non-vaccinated cattle but low variation (4%) between vaccinated and non-vaccinated cattle. This study reveals the role of ITM in inducing the carrier status and higher T. parva genetic diversity in vaccinated cattle. The low genetic variation between T. parva isolates in both vaccinated and non-vaccinated cattle may be suggestive of the protective role of vaccine strains against genetically related local strains in the study area.

DOI: 10.3390/pathogens12020295
PMCID: PMC9965312
PMID: 36839567

Conflict of interest statement: The authors declare no conflict of interest.

218. Clin Infect Dis. 2023 Feb 8;76(3):e902-e909. doi: 10.1093/cid/ciac669.

The Association Between Social Network Characteristics and Tuberculosis Infection Among Adults in 9 Rural Ugandan Communities.

Marquez C(1), Chen Y(2), Atukunda M(3), Chamie G(1), Balzer LB(4), Kironde J(3), Ssemmondo E(3), Mwangwa F(3), Kabami J(3), Owaraganise A(3), Kakande E(3), Abbott R(1), Ssekyanzi B(3), Koss C(1), Kamya MR(3)(5), Charlebois ED(6), Havlir DV(1), Petersen ML(4).

Author information:
(1)Division of HIV, Infectious Diseases, and Global Medicine, Department of Medicine, University of California, San Francisco, California, USA.
(2)Department of Biostatistics, University of Washington, Seattle, Washington, USA.
(3)Infectious Diseases Research Collaboration, Kampala, Uganda.
(4)Division of Biostatistics, School of Public Health, University of California, Berkeley, Berkeley, California, USA.
(5)School of Medicine, Makerere University College of Health Sciences, Kampala, Uganda.
(6)Center for AIDS Prevention Studies, School of Medicine, University of California, San Francisco, San Francisco, California, USA.

BACKGROUND: Social network analysis can elucidate tuberculosis transmission dynamics outside the home and may inform novel network-based case-finding strategies.
METHODS: We assessed the association between social network characteristics and prevalent tuberculosis infection among residents (aged ≥15 years) of 9 rural communities in Eastern Uganda. Social contacts named during a census were used to create community-specific nonhousehold social networks. We evaluated whether social network structure and characteristics of first-degree contacts (sex, human immunodeficiency virus [HIV] status, tuberculosis infection) were associated with revalent tuberculosis infection (positive tuberculin skin test

[TST] result) after adjusting for individual-level risk factors (age, sex, HIV status, tuberculosis contact, wealth, occupation, and Bacillus Calmette-Guérin [BCG] vaccination) with targeted maximum likelihood estimation.
RESULTS: Among 3 335 residents sampled for TST, 32% had a positive TST results and 4% reported a tuberculosis contact. The social network contained 15 328 first-degree contacts. Persons with the most network centrality (top 10%) (adjusted risk ratio, 1.3 [95% confidence interval, 1.1-1.1]) and the most (top 10%) male contacts (1.5 [1.3-1.9]) had a higher risk of prevalent tuberculosis, than those in the remaining 90%. People with ≥1 contact with HIV (adjusted risk ratio, 1.3 [95% confidence interval, 1.1-1.6]) and ≥2 contacts with tuberculosis infection were more likely to have tuberculosis themselves (2.6 [95% confidence interval: 2.2-2.9]).
CONCLUSIONS: Social networks with higher centrality, more men, contacts with HIV, and tuberculosis infection were positively associated with tuberculosis infection. Tuberculosis transmission within measurable social networks may explain prevalent tuberculosis not associated with a household contact. Further study on network-informed tuberculosis case finding interventions is warranted.

© The Author(s) 2022. Published by Oxford University Press on behalf of Infectious Diseases Society of America. All rights reserved. For permissions, please e-mail: journals.permissions@oup.com.

DOI: 10.1093/cid/ciac669
PMCID: PMC10169405
PMID: 35982635 [Indexed for MEDLINE]

Conflict of interest statement: Potential conflicts of interest. G. C. reports participation on a data safety monitoring board or advisory board as a member of a data monitoring committee for an National Institutes of Health (NIH)–funded clinical trial (TB Screening Improves Preventive Therapy Uptake [TB SCRIPT] trial). E. D. C. reports support from the NIH (grant 1R01AI151209-01 [paid to institution]) and consulting fees from the Infectious Diseases Research Collaboration, Kampala, Uganda (paid to the author for Integrated HIV & Hypertension Study consulting). D. V. H. reports nonfinancial support from Gilead (drug donation for an NIH study) and Abbott (diagnostic test donation). All other authors report no potential conflicts. All authors have submitted the ICMJE Form for Disclosure of Potential Conflicts of Interest. Conflicts that the editors consider relevant to the content of the manuscript have been disclosed.

219. Vaccines (Basel). 2023 Feb 7;11(2):375. doi: 10.3390/vaccines11020375.

Recovering from the Unprecedented Backsliding in Immunization Coverage: Learnings from Country Programming in Five Countries through the Past Two Years of COVID-19 Pandemic Disruptions.

Athiyaman A(1), Ajayi T(1), Mutuku F(1), Luwaga F(1), Bryer S(1), Giwa O(1),

Mngemane S(1), Edwige NN(1), Berman L(1).

Author information:
(1)Clinton Health Access Initiative (CHAI), Global Vaccines Delivery Team and Country Offices, Boston, MA 02127, USA.

Between 2020 and 2021, the COVID-19 pandemic severely strained health systems across countries, leaving millions without access to essential healthcare services. Immunization programs experienced a 'double burden' of challenges: initial pandemic-related lockdowns disrupted access to routine immunization services, while subsequent COVID-19 vaccination efforts shifted often limited resources away from routine services. The latest World Health Organization (WHO) and United Nations Children's Fund (UNICEF) estimates suggest that 25 million children did not receive routine vaccinations in 2021, six million more than in 2019 and the highest number witnessed in nearly two decades. Recovering from this sobering setback requires a united push on several fronts. Intensifying the catch-up of routine immunization services is critical to reach children left behind during the pandemic and bridge large immunity gaps in countries. At the same time, we must strengthen the resilience of immunization systems to withstand future pandemics if we hope to achieve the goals of Immunization Agenda 2030 to ensure vaccinations are available for everyone, everywhere by 2030. In this article, leveraging the key actions for sustainable global immunization progress as a framework, we spotlight examples of strategies used by five countries-Cambodia, Cameroon, Kenya, Nigeria, and Uganda-who have exhibited exemplar performance in strengthening routine immunization programs and restored lost coverage levels in the last two years of the COVID-19 pandemic. The contents of this article will be helpful for countries seeking to maintain, restore, and strengthen their immunization services and catch up missed children in the context of pandemic recovery and to direct their focus toward building back a better resilience of their immunization systems to respond more rapidly and effectively, despite new and emerging challenges.

DOI: 10.3390/vaccines11020375
PMCID: PMC9960337
PMID: 36851253

Conflict of interest statement: The authors declare no conflict of interest.

220. Vaccines (Basel). 2023 Feb 4;11(2):357. doi: 10.3390/vaccines11020357.

Using Dried Blood Spots for a Sero-Surveillance Study of Maternally Derived Antibody against Group B Streptococcus.

Auma E(1)(2), Hall T(2), Chopra S(3), Bilton S(4), Ramkhelawon L(2), Amini F(2), Calvert A(2), Amirthalingam G(5), Jones CE(2)(6)(7), Andrews N(5), Heath PT(2), Le Doare K(2)(8)(9).

Author information:
(1)Department of Biology, Université Claude Bernard Lyon, ENS de Lyon, CNRS, UMR, 69100 Villeurbanne, France.
(2)Centre for Neonatal and Paediatric Infection, Institute for Infection and Immunity, St George's, University of London, London SW17 0RE, UK.
(3)Immunity and Infection, Faculty of Medicine, Imperial College London, London SW7 2BX, UK.
(4)Neonatal Unit, John Radcliffe Hospital, Oxford University Hospitals NHS Foundation Trust, Oxford OX3 9DU, UK.
(5)Immunisation and Vaccine Preventable Diseases Division, UK Health Security Agency, London NW9 5EQ, UK.
(6)Faculty of Medicine and Institute for Life Sciences, University of Southampton, Southampton, SO16 6YD, UK.
(7)NIHR Southampton Clinical Research Facility and Biomedical Research Centre, University Hospital Southampton NHS Foundation Trust, Southampton SO16 6YD, UK.
(8)Makerere University-Johns Hopkins University Research Collaboration, Kampala P.O. Box 23491, Uganda.
(9)Pathogen Immunology Group, UK Health Security Agency, Porton Down, Salisbury SP4 0JG, UK.

Vaccination during pregnancy could protect women and their infants from invasive Group B Streptococcus (GBS) disease. To understand if neonatal dried blood spots (DBS) can be used to determine the amount of maternally derived antibody that protects infants against invasive GBS disease, a retrospective case-control study was conducted in England between 1 April 2014 and 30 April 2015. The DBS of cases with invasive GBS disease (n = 61) were matched with healthy controls (n = 125). The haematocrit, DBS storage temperature, freeze-thaw cycle, and paired serum/DBS studies were set up to optimise the antibody assessment. The samples were analysed using a multiplex immunoassay, and the results were assessed using parametric and nonparametric tests. Antibody concentrations were stable at haematocrits of up to 50% but declined at 75%. DBS storage at room temperature was stable for three months compared with storage from collection at -20 °C and rapidly degraded thereafter. Total IgG levels measured in DBS and paired serum showed a good correlation (r2 = 0.99). However, due to suboptimal storage conditions, no difference was found in the GBS IgG levels between DBS samples from cases and controls. We have demonstrated a proof of concept that assays utilising DBS for assessing GBS serotype-specific antibodies in infants is viable. This method could be used to facilitate future large sero-correlate studies, but DBS samples must be stored at -20 °C for long term preservation of antibody.

DOI: 10.3390/vaccines11020357
PMCID: PMC9966576
PMID: 36851236

Conflict of interest statement: The authors declare no conflict of interest.

221. EClinicalMedicine. 2023 Jan 5;56:101797. doi: 10.1016/j.eclinm.2022.101797. eCollection 2023 Feb.

Meningococcal A conjugate vaccine coverage in the meningitis belt of Africa from 2010 to 2021: a modelling study.

Bender RG(1)(2), Shen J(3), Aravkin A(1)(4), Bita Fouda AA(5), Bwaka AM(6), Galles NC(1), Haeuser E(1), Hay SI(1)(2), Latt A(7), Mwenda JM(5), Rogowski ELB(1), Sbarra AN(1)(8), Sorensen RJD(1), Vongpradith A(1), Wright C(9), Zheng P(1)(2), Mosser JF(1)(2), Kyu HH(1)(2).

Author information:
(1)Institute for Health Metrics and Evaluation, University of Washington, Seattle, WA, USA.
(2)Department of Health Metrics Sciences, University of Washington, Seattle, WA, USA.
(3)School of Medicine, University of Washington, Seattle, WA, USA.
(4)Department of Applied Mathematics, University of Washington, Seattle, WA, USA.
(5)World Health Organization Regional Office for Africa, Brazzaville, Republic of Congo.
(6)World Health Organization Regional Office for Africa, Inter-Country Support Team, Ouagadougou, Burkina Faso.
(7)World Health Organization Regional Office for Africa, Emergency Preparedness and Response Cluster, Dakar Emergency Hub, Dakar, Senegal.
(8)Department of Infectious Disease Epidemiology, London School of Hygiene and Tropical Medicine, London, UK.
(9)Meningitis Research Foundation, Bristol, UK.

BACKGROUND: As of the end of 2021, twenty-four countries in the African meningitis belt have rolled out mass campaigns of MenAfriVac®, a meningococcal A conjugate vaccine (MACV) first introduced in 2010. Twelve have completed introduction of MACV into routine immunisation (RI) schedules. Although select post-campaign coverage data are published, no study currently comprehensively estimates MACV coverage from both routine and campaign sources in the meningitis belt across age, country, and time.
METHODS: In this modelling study, we assembled campaign data from the twenty-four countries that had introduced any immunisation activity during or before the year 2021 (Benin, Burkina Faso, Burundi, Cameroon, Central African Republic, Chad, Côte d'Ivoire, Democratic Republic of the Congo, Ethiopia, Eritrea, the Gambia, Ghana, Guinea, Guinea Bissau, Kenya, Mali, Mauritania, Niger, Nigeria, Senegal, South Sudan, Sudan, Togo and Uganda) via WHO reports and RI data via systematic review. Next, we modelled RI coverage using Spatiotemporal Gaussian Process Regression. Then, we synthesized these estimates with campaign data into a cohort model, tracking coverage for each age cohort

from age 1 to 29 years over time for each country.
FINDINGS: Coverage in high-risk locations amongst children aged 1-4 in 2021 was estimated to be highest in Togo with 96.0% (95% uncertainty interval [UI] 92.0-99.0), followed by Niger with 87.2% (95% UI 85.3-89.0) and Burkina Faso, with 86.4% (95% UI 85.1-87.6). These countries had high coverage values driven by an initial successful mass immunisation campaign, followed by a catch-up campaign, followed by introduction of RI. Due to the influence of older mass vaccination campaigns, coverage proportions skewed higher in the 1-29 age group than the 1-4 group, with a median coverage of 82.9% in 2021 in the broader age group compared to 45.6% in the narrower age group.
INTERPRETATION: These estimates highlight where gaps in immunisation remain and emphasise the need for broader efforts to strengthen RI systems. This methodological framework can be applied to estimate coverage for any vaccine that has been delivered in both routine and supplemental immunisation activities.
FUNDING: Bill and Melinda Gates Foundation.

© 2023 The Authors.

DOI: 10.1016/j.eclinm.2022.101797
PMCID: PMC9985031
PMID: 36880052

Conflict of interest statement: IHME is primarily funded by the Bill and Melinda Gates Foundation. JM & AS additionally report receiving funding from Gavi. CW reports that the Meningitis Research Foundation has received grants in support of their charitable objectives from GSK, Pfizer, Sanofi Pasteur, Serum Institute, and Tableau foundation. CW reports that the Meningitis Research foundation has received consulting fees from GSK within the last 4 years.

222. JAMA Netw Open. 2023 Feb 1;6(2):e2255978. doi: 10.1001/jamanetworkopen.2022.55978.

Seroprevalence of Antibodies to SARS-CoV-2 in Rural Households in Eastern Uganda, 2020-2022.

Briggs J(1), Takahashi S(1), Nayebare P(2), Cuu G(2), Rek J(2), Zedi M(2), Kizza T(2), Arinaitwe E(2), Nankabirwa JI(2)(3), Kamya M(2)(3), Jagannathan P(4)(5), Jacobson K(4), Rosenthal PJ(1), Dorsey G(1), Greenhouse B(1), Ssewanyana I(2)(6), Rodríguez-Barraquer I(1).

Author information:
(1)Division of HIV, ID, and Global Medicine, Department of Medicine, University of California San Francisco, San Francisco.
(2)Infectious Diseases Research Collaboration, Kampala, Uganda.
(3)Department of Medicine, Makerere University, Kampala, Uganda.

(4)Department of Medicine, Stanford University, Stanford, California.
(5)Department of Microbiology and Immunology, Stanford University, Stanford, California.
(6)Central Public Health Laboratories, Butabika, Uganda.

IMPORTANCE: Estimating the true burden of SARS-CoV-2 infection has been difficult in sub-Saharan Africa owing to asymptomatic infections and inadequate testing capacity. Antibody responses from serologic surveys can provide an estimate of SARS-CoV-2 exposure at the population level.
OBJECTIVE: To estimate SARS-CoV-2 seroprevalence, attack rates, and reinfection in eastern Uganda using serologic surveillance from 2020 to early 2022.
DESIGN, SETTING, AND PARTICIPANTS: This cohort study was conducted in the Tororo and Busia districts of eastern Uganda. Plasma samples from participants in the Program for Resistance, Immunology, Surveillance, and Modeling of Malaria in Uganda Border Cohort were obtained at 4 sampling intervals: October to November 2020, March to April 2021, August to September 2021, and February to March 2022. Each participant contributed up to 4 time points for SARS-CoV-2 serology, with almost half of all participants contributing at all 4 time points, and almost 90% contributing at 3 or 4 time points. Information on SARS-CoV-2 vaccination status was collected from participants, with the earliest reported vaccinations in the cohort occurring in May 2021.
MAIN OUTCOMES AND MEASURES: The main outcomes of this study were antibody responses to the SARS-CoV-2 spike protein as measured with a bead-based serologic assay. Individual-level outcomes were aggregated to population-level SARS-CoV-2 seroprevalence, attack rates, and boosting rates. Estimates were weighted by the local age distribution according to census data.
RESULTS: A total of 1483 samples from 441 participants living in 76 households were tested. Of the 441 participants, 245 (55.6%) were female, and their mean (SD) age was 16.04 (16.04) years. By the end of the Delta wave and before widespread vaccination, adjusted SARS-CoV-2 seroprevalence was 67.7% (95% credible interval [CrI], 62.5%-72.6%) in the study population. During the subsequent Omicron wave, 84.8% (95% CrI, 67.9%-93.7%) of unvaccinated, previously seronegative individuals were infected for the first time, and 50.8% (95% CrI, 40.6%-59.7%) of unvaccinated, already seropositive individuals were likely reinfected, leading to an overall seropositivity of 96.0% (95% CrI, 93.4%-97.9%) in this population. These results suggest a lower probability of reinfection in individuals with higher preexisting antibody levels. There was evidence of household clustering of SARS-CoV-2 seroconversion. No significant associations were found between SARS-CoV-2 seroconversion and gender, household size, or recent Plasmodium falciparum malaria exposure.
CONCLUSIONS AND RELEVANCE: In this cohort study in a rural population in eastern Uganda, there was evidence of very high SARS-CoV-2 infection rates throughout the pandemic inconsistent with national level case data and high reinfection rates during the Omicron wave.

DOI: 10.1001/jamanetworkopen.2022.55978
PMCID: PMC9932849

PMID: 36790811 [Indexed for MEDLINE]

Conflict of interest statement: Conflict of Interest Disclosures: None reported.

223. J Acquir Immune Defic Syndr. 2023 Feb 1;92(2):122-126. doi:
10.1097/QAI.0000000000003110.

Brief Report: Dapivirine Ring HIV-1 Prevention Effectiveness for Women Engaged
in Vaginal and Anal Intercourse: Insights From Mathematical Modeling.

Peebles K(1), Brown ER(2)(3), Hendrix CW(4), Palanee-Phillips T(5), van der
Straten A(6), Harkoo I(7), Reddy K(6), Mirembe BG(8), Jeenarain N(9), Hillier
SL(10), Baeten JM(1)(11)(12), Barnabas RV(1)(11)(12); MTN-020/ASPIRE study team.

Author information:
(1)Department of Global Health, University of Washington, Seattle, WA.
(2)Vaccine and Infectious Disease and Public Health Sciences Divisions, Fred
Hutchinson Cancer Research Center, Seattle, WA.
(3)Department of Biostatistics, University of Washington, Seattle, WA.
(4)Department of Medicine (Clinical Pharmacology), Johns Hopkins University,
Baltimore, MD.
(5)Wits Reproductive Health and HIV Institute, University of the Witwatersrand,
Johannesburg, South Africa.
(6)RTI International, Women's Global Health Imperative (WGHI), San Francisco,
CA.
(7)Centre for the AIDS Program of Research in South Africa, Durban, South
Africa.
(8)Makerere University-Johns Hopkins University Research Collaboration, Kampala,
Uganda.
(9)South African Medical Research Council, Durban, South Africa.
(10)Department of Obstetrics, Gynecology and Reproductive Sciences, University
of Pittsburgh, Pittsburgh, PA.
(11)Department of Epidemiology, University of Washington, Seattle, WA; and.
(12)Department of Medicine, University of Washington, Seattle, WA.

BACKGROUND: The dapivirine vaginal ring reduces the risk of HIV-1 acquisition in
acts of vaginal intercourse (VI), and although it does not offer HIV-1
protection in acts of anal intercourse (AI), it may provide some overall risk
reduction for women for whom most sex acts are vaginal. We estimated the
protective effect of the ring among women with high ring adherence engaged in
both VI and AI.
METHODS: We developed a microsimulation model using data from the MTN-020/ASPIRE
trial. Among women who reported any AI, we estimated the proportion of all sex
acts that were AI. Model scenarios varied this proportion among women engaged in
both VI and AI from 5% to 30%, including the trial-observed median proportion of
6.3% of all acts being AI. In primary analyses, dapivirine ring efficacy was

model-calibrated at 70% for vaginal exposures and assumed to be 0% for anal exposures.
RESULTS: Among highly adherent women for whom 6.3% of sex acts were AI, the ring reduced HIV-1 risk by 53% (interquartile range: 44, 60), with a decline to 26% (interquartile range: 16, 36) among women for whom 30% of acts were AI. Ring effectiveness was less than 40% among women for whom AI accounted for greater than 16% of all sex acts, although this represented less than 5% of all women in the ASPIRE trial.
CONCLUSIONS: For most women, including those who engage in AI, because most HIV-1 risk occurs in acts of vaginal sex, the dapivirine vaginal ring can provide important HIV-1 protection.

Copyright © 2022 Wolters Kluwer Health, Inc. All rights reserved.

DOI: 10.1097/QAI.0000000000003110
PMCID: PMC9839478
PMID: 36184755 [Indexed for MEDLINE]

Conflict of interest statement: The authors have no conflicts of interest to disclose.

224. Am J Obstet Gynecol. 2023 Feb;228(2):161-177. doi: 10.1016/j.ajog.2022.08.038. Epub 2022 Aug 24.

Clinical risk factors of adverse outcomes among women with COVID-19 in the pregnancy and postpartum period: a sequential, prospective meta-analysis.

Smith ER(1), Oakley E(2), Grandner GW(2), Rukundo G(3), Farooq F(2), Ferguson K(2), Baumann S(2), Adams Waldorf KM(4), Afshar Y(5), Ahlberg M(6), Ahmadzia H(7), Akelo V(8), Aldrovandi G(9), Bevilacqua E(10), Bracero N(11), Brandt JS(12), Broutet N(13), Carrillo J(14), Conry J(15), Cosmi E(16), Crispi F(17), Crovetto F(17), Del Mar Gil M(18), Delgado-López C(19), Divakar H(20), Driscoll AJ(21), Favre G(22), Fernandez Buhigas I(18), Flaherman V(23), Gale C(24), Godwin CL(13), Gottlieb S(13), Gratacós E(17), He S(2), Hernandez O(25), Jones S(26), Joshi S(20), Kalafat E(27), Khagayi S(28), Knight M(29), Kotloff KL(21), Lanzone A(30), Laurita Longo V(30), Le Doare K(31), Lees C(32), Litman E(7), Lokken EM(4), Madhi SA(26), Magee LA(33), Martinez-Portilla RJ(34), Metz TD(35), Miller ES(36), Money D(37), Moungmaithong S(38), Mullins E(39), Nachega JB(40), Nunes MC(26), Onyango D(41), Panchaud A(42), Poon LC(38), Raiten D(43), Regan L(44), Sahota D(38), Sakowicz A(36), Sanin-Blair J(45), Stephansson O(6), Temmerman M(46), Thorson A(13), Thwin SS(13), Tippett Barr BA(47), Tolosa JE(48), Tug N(49), Valencia-Prado M(50), Visentin S(16), von Dadelszen P(51), Whitehead C(52), Wood M(53), Yang H(54), Zavala R(2), Tielsch JM(2).

Author information:
(1)Department of Global Health, Milken Institute School of Public Health, George

Washington University, Washington, DC. Electronic address: EmilySmith@gwu.edu.
(2)Department of Global Health, Milken Institute School of Public Health, George Washington University, Washington, DC.
(3)PeriCOVID (PREPARE)-Uganda Team, Makerere University-Johns Hopkins University Research Collaboration, Kampala, Uganda.
(4)Department of Obstetrics and Gynecology, University of Washington School of Medicine, Seattle, WA; Department of Global Health, University of Washington, Seattle, WA.
(5)Division of Maternal-Fetal Medicine, University of California, Los Angeles, Los Angeles, CA.
(6)Division of Division of Clinical Epidemiology, Department of Medicine, Solna, Karolinska Institutet, Stockholm, Sweden.
(7)Division of Maternal-Fetal Medicine, The George Washington University School of Medicine and Health Sciences, Washington, DC.
(8)Centers for Disease Control and Prevention, Kisumu, Kenya.
(9)Department of Pediatrics, University of California, Los Angeles, Los Angeles, CA.
(10)Department of Women and Child Health, Women Health Area, Fondazione Policlinico Universitario Agostino Gemelli, Istituto di Ricovero e Cura a Carattere Scientifico, Rome, Italy.
(11)Department of Obstetrics and Gynecology, University of Puerto Rico School of Medicine, San Juan, PR; Puerto Rico Obstetrics and Gynecology (PROGyn).
(12)Division of Maternal-Fetal Medicine, Department of Obstetrics, Gynecology and Reproductive Sciences, Rutgers Robert Wood Johnson Medical School, New Brunswick, NJ.
(13)Department of Sexual and Reproductive Health and Research, World Health Organization, Geneva, Switzerland.
(14)Departamento de Obstetricia y Ginecologia, Clinica Alemana de Santiago, Facultad de Medicina Clinica Alemana-Universidad del Desarrollo, Santiago, Chile.
(15)International Federation of Gynecology and Obstetrics, London, United Kingdom.
(16)Department of Women's and Children's Health, Obstetrics and Gynecology Clinic, University of Padua, Padua, Italy.
(17)BCNatal, Barcelona Center for Maternal-Fetal and Neonatal Medicine, Hospital Sant Joan de Déu Barcelona and Hospital Clínic de Barcelona, Universitat de Barcelona, and Center for Biomedical Research on Rare Diseases, Barcelona, Spain.
(18)Department of Obstetrics and Gynecology, Hospital Universitario de Torrejón, Madrid, Spain; School of Medicine, Universidad Francisco de Vitoria, Madrid, Spain.
(19)Surveillance for Emerging Threats to Mothers and Babies, Puerto Rico Department of Health, San Juan, PR.
(20)Asian Research & Training Institute for Skill Transfer, Bengaluru, India.
(21)Center for Vaccine Development and Global Health, University of Maryland School of Medicine, Baltimore, MD.
(22)Materno-fetal and Obstetrics Research Unit, Département Femme-Mère-Enfant,

Lausanne University Hospital, Lausanne, Switzerland.
(23)Department of Pediatrics, University of California, San Francisco, San Francisco, CA.
(24)Neonatal Medicine, School of Public Health, Faculty of Medicine, Imperial College London, London, United Kingdom.
(25)Gynecology and Obstetrics, Félix Bulnes Hospital and RedSalud Clinic, Santiago, Chile.
(26)South African Medical Research Council, Vaccines and Infectious Diseases Analytics Research Unit and Department of Science and Technology/National Research Foundation, South African Research Chair Initiative in Vaccine Preventable Diseases, Faculty of Health Sciences, University of the Witwatersrand, Johannesburg, South Africa.
(27)Department of Obstetrics and Gynecology, School of Medicine, Koç University, Istanbul, Turkey.
(28)Kenya Medical Research Institute-Centre for Global Health Research, Kisumu, Kenya.
(29)National Perinatal Epidemiology Unit, Nuffield Department of Population Health, University of Oxford, Oxford, United Kingdom.
(30)Department of Women and Child Health, Women Health Area, Fondazione Policlinico Universitario Agostino Gemelli, Istituto di Ricovero e Cura a Carattere Scientifico, Rome, Italy; Catholic University of Sacred Heart, Rome, Italy.
(31)PeriCOVID (PREPARE)-Uganda Team, Makerere University-Johns Hopkins University Research Collaboration, Kampala, Uganda; Medical Research Council /Uganda Virus Research Institute and London School of Hygiene & Tropical Medicine Uganda Research Unit, Entebbe, Uganda; Paediatric Infectious Disease Research Group, St George's University of London, London, United Kingdom.
(32)Department of Metabolism, Digestion and Reproduction, Imperial College London, London, United Kingdom.
(33)Department of Women and Children's Health, School of Life Course Sciences, King's College London, London, United Kingdom; Institute of Women and Children's Health, King's College Hospital, London, United Kingdom.
(34)Clinical Research Branch, National Institute of Perinatology, Mexico City, Mexico.
(35)Division of Maternal-Fetal Medicine, The University of Utah Health, Salt Lake City, UT.
(36)Division of Maternal-Fetal Medicine, Department of Obstetrics and Gynecology, Northwestern University Feinberg School of Medicine, Chicago, IL.
(37)Department of Obstetrics and Gynecology, The University of British Columbia, Vancouver, Canada.
(38)Department of Obstetrics and Gynecology, The Chinese University of Hong Kong, Hong Kong.
(39)Department of Metabolism, Digestion and Reproduction, Imperial College London, London, United Kingdom; George Institute for Global Health, London, United Kingdom.
(40)Department of Epidemiology and Center for Global Health, University of Pittsburgh Graduate School of Public Health, Pittsburgh, PA; Department of

Medicine, Faculty of Medicine and Health Sciences, Stellenbosch University, Cape Town, South Africa; Departments of Epidemiology and International Health, Johns Hopkins Bloomberg School of Public Health, Baltimore, MD.
(41)Kisumu County Department of Health, Kisumu, Kenya.
(42)Institute of Primary Health Care (BIHAM), University of Bern, Bern, Switzerland; Service of Pharmacy, Lausanne University Hospital, University of Lausanne, Lausanne, Switzerland.
(43)Pediatric Growth and Nutrition Branch, Eunice Kennedy Shriver National Institute of Child Health and Human Development, National Institutes of Health, Bethesda, MD.
(44)International Federation of Gynecology and Obstetrics, Imperial College London, London, United Kingdom.
(45)Maternal-Fetal Unit, Universidad Pontificia Bolivariana, Medellín, Colombia.
(46)Centre of Excellence in Women and Child Health, Aga Khan University, Nairobi, Kenya.
(47)Centers for Disease Control and Prevention, Kisumu, Kenya; Nyanja Health Research Institute, Salima, Malawi.
(48)Maternal-Fetal Unit, Universidad Pontificia Bolivariana, Medellín, Colombia; Department of Obstetrics and Gynecology, Maternal Fetal Medicine, Oregon Health & Science University, Portland, OR; Department of Obstetrics and Gynecology, Maternal Fetal Medicine, St. Luke's University Health Network, Bethlehem, PA.
(49)Department of Obstetrics and Gynecology, Sancaktepe Sehit Prof. Dr. Ilhan Varank Training and Research Hospital, Istanbul, Turkey.
(50)Division of Children with Special Medical Needs, Puerto Rico Department of Health, San Juan, PR.
(51)Department of Women and Children's Health, School of Life Course Sciences, King's College London, London, United Kingdom; Global Health Institute, King's College London, London, United Kingdom.
(52)Department of Maternal Fetal Medicine, University of Melbourne, Royal Women's Hospital, Melbourne, Australia.
(53)Department of Epidemiology, Gillings School of Global Public Health, The University of North Carolina at Chapel Hill, Chapel Hill, NC.
(54)Department of Obstetrics and Gynecology, Peking University First Hospital, Beijing, China.

OBJECTIVE: This sequential, prospective meta-analysis sought to identify risk factors among pregnant and postpartum women with COVID-19 for adverse outcomes related to disease severity, maternal morbidities, neonatal mortality and morbidity, and adverse birth outcomes.
DATA SOURCES: We prospectively invited study investigators to join the sequential, prospective meta-analysis via professional research networks beginning in March 2020.
STUDY ELIGIBILITY CRITERIA: Eligible studies included those recruiting at least 25 consecutive cases of COVID-19 in pregnancy within a defined catchment area.
METHODS: We included individual patient data from 21 participating studies. Data quality was assessed, and harmonized variables for risk factors and outcomes were constructed. Duplicate cases were removed. Pooled estimates for the

absolute and relative risk of adverse outcomes comparing those with and without each risk factor were generated using a 2-stage meta-analysis.
RESULTS: We collected data from 33 countries and territories, including 21,977 cases of SARS-CoV-2 infection in pregnancy or postpartum. We found that women with comorbidities (preexisting diabetes mellitus, hypertension, cardiovascular disease) vs those without were at higher risk for COVID-19 severity and adverse pregnancy outcomes (fetal death, preterm birth, low birthweight). Participants with COVID-19 and HIV were 1.74 times (95% confidence interval, 1.12-2.71) more likely to be admitted to the intensive care unit. Pregnant women who were underweight before pregnancy were at higher risk of intensive care unit admission (relative risk, 5.53; 95% confidence interval, 2.27-13.44), ventilation (relative risk, 9.36; 95% confidence interval, 3.87-22.63), and pregnancy-related death (relative risk, 14.10; 95% confidence interval, 2.83-70.36). Prepregnancy obesity was also a risk factor for severe COVID-19 outcomes including intensive care unit admission (relative risk, 1.81; 95% confidence interval, 1.26-2.60), ventilation (relative risk, 2.05; 95% confidence interval, 1.20-3.51), any critical care (relative risk, 1.89; 95% confidence interval, 1.28-2.77), and pneumonia (relative risk, 1.66; 95% confidence interval, 1.18-2.33). Anemic pregnant women with COVID-19 also had increased risk of intensive care unit admission (relative risk, 1.63; 95% confidence interval, 1.25-2.11) and death (relative risk, 2.36; 95% confidence interval, 1.15-4.81).
CONCLUSION: We found that pregnant women with comorbidities including diabetes mellitus, hypertension, and cardiovascular disease were at increased risk for severe COVID-19-related outcomes, maternal morbidities, and adverse birth outcomes. We also identified several less commonly known risk factors, including HIV infection, prepregnancy underweight, and anemia. Although pregnant women are already considered a high-risk population, special priority for prevention and treatment should be given to pregnant women with these additional risk factors.

Copyright © 2022. Published by Elsevier Inc.

DOI: 10.1016/j.ajog.2022.08.038
PMCID: PMC9398561
PMID: 36027953 [Indexed for MEDLINE]

225. Br J Clin Pharmacol. 2023 Feb;89(2):491-509. doi: 10.1111/bcp.15193. Epub 2022 Feb 9.

Pharmacovigilance in low- and middle-income countries: A review with particular focus on Africa.

Kiguba R(1), Olsson S(2), Waitt C(3)(4).

Author information:
(1)Department of Pharmacology and Therapeutics, Makerere University, Kampala,

Uganda.
(2)Pharmacovigilance Consulting, Uppsala, Sweden.
(3)Department of Pharmacology and Therapeutics, University of Liverpool, UK.
(4)Infectious Diseases Institute, Makerere University College of Health
Sciences, Uganda.

Low- and middle-income countries (LMIC) face unique challenges with regard to the establishment of robust pharmacovigilance systems capable of generating data to inform healthcare policy and practice. These include the limited integration and reliability of pharmacovigilance systems across LMIC despite recent efforts to harmonize pharmacovigilance rules and regulations in several regional economic communities. There are particular challenges relating to the need to translate reporting tools into numerous local languages and the low numbers of healthcare providers relative to number of patients, with very short consultation times. Additional factors frequent in LMIC include high uptake of herbal and traditional medication, mostly by self-medication; disruptive political conflicts jeopardizing fragile systems; and little or no access to drug utilization data, which makes it difficult to reliably estimate the true risks of medicines use. Pharmacovigilance activities are hindered by the scarcity of well-trained personnel with little or no budgetary support from national governments; high turnover of pharmacovigilance staff whose training involves a substantial amount of resources; and little awareness of pharmacovigilance among healthcare workers, decision makers and consumers. Furthermore, little collaboration between public health programmes and national medicines regulatory authorities coupled with limited investment in pharmacovigilance activities, especially during mass drug administration for neglected tropical diseases and mass vaccinations, produces major challenges in establishing a culture where pharmacovigilance is systematically embedded. Very low spontaneous reporting rates with poor quality reports hinders robust signal detection analyses. This review summarises the specific challenges and areas of progress in pharmacovigilance in LMIC with special focus on the situation in Africa.

© 2021 The Authors. British Journal of Clinical Pharmacology published by John Wiley & Sons Ltd on behalf of British Pharmacological Society.

DOI: 10.1111/bcp.15193
PMID: 34937122 [Indexed for MEDLINE]

226. J Educ Health Promot. 2023 Jan 31;12:34. doi: 10.4103/jehp.jehp_687_22. eCollection 2023.

Prevalence and predictors of vaccine hesitancy among mothers of under-five children: A hospital-based cross-sectional study.

Goruntla N(1), Akanksha K(2), Lalithaasudhaa K(2), Pinnu V(2), Jinka D(3),

Bhupalam P(4), Doniparthi J(5).

Author information:
(1)Department of Clinical Pharmacy and Pharmacy Practice, School of Pharmacy, Kampala International University (KIU), Western Campus, Uganda.
(2)Department of Pharmacy Practice, Raghavendra Institute of Pharmaceutical Education and Research (RIPER) - Autonomous, Anantapur, Andhra Pradesh, India.
(3)Department of Pediatrics, Rural Development Trust Hospital, Bathalapalli, Andhra Pradesh, India.
(4)Department of Pharmacology, Raghavendra Institute of Pharmaceutical Education and Research (RIPER) - Autonomous, Anantapur, Andhra Pradesh, India.
(5)Department of Pharmaceutics, Sri Krishnadevaraya University College of Pharmaceutical Sciences, S.K. University, Anantapuramu, Andhra Pradesh, India.

BACKGROUND: The World Health Organization (WHO) states that vaccine hesitancy is one of the top 10 threats to global public health. Evidence shows that vaccine hesitancy studies in India are limited and targeted toward individual vaccines. The study aimed to fill this gap by exploring the relationship between demographics and SAGE factors toward vaccine hesitancy.
MATERIALS AND METHODS: A hospital-based, cross-sectional, analytical study was conducted in a non-governmental organization (NGO) hospital with 330 beds, located in Bathalapalli, Andhra Pradesh, India. Mothers of under-five children who attended outpatient departments of pediatrics or obstetrics and gynecology were included. A total of 574 mothers were enrolled and vaccine hesitancy was determined by reviewing the mother-child protection card for the presence of delay or refusal of the recommended vaccine. A face-to-face interview was conducted to obtain demographics and WHO-SAGE variables from the participants. Binary logistic regression analysis was performed to associate independent variables (demographics and SAGE variables) with the dependent variable (vaccine hesitancy).
RESULTS: Out of 574 respondents, 161 mother's children were noted as vaccine-hesitant (refusal = 7; delay = 154); and the prevalence of vaccine hesitancy was 28.05%. The delay was observed in all recommended vaccines, but the refusal or reluctance was seen in only four vaccines (hepatitis B birth dose = 1; IPV 1 and 2 = 2; Measles 1 and 2 = 3; and Rota 1, 2, and 3 = 1). The respondents' demographics like no or lower parent education (OR = 3.17; 95%CI = 1.50-6.72) and fewer antenatal visits (OR = 2.30; 95%CI = 1.45-3.36) showed higher odds, whereas the upper socioeconomic status showed lower odds (OR = 0.09; 95%CI = 0.02-0.36) toward vaccine hesitancy. The WHO-SAGE dimensions like awareness (OR = 0.14; 95%CI = 0.03-0.53), poor access (OR = 7.76; 95%CI = 3.65-16.51), and low acceptability of the individual (OR = 07.15; 95%CI = 1.87-27.29), community (OR = 6.21; 95%CI = 1.58-24.33) were significantly associated with vaccine hesitancy.
CONCLUSION: The study concludes that the prevalence of vaccine hesitancy was high. Vaccine safety and children's health are primary concerns for parents' refusal/reluctance. To achieve 100% immunization coverage, policymakers need to reduce vaccine hesitancy by developing strategies based on demographic and

WHO-SAGE working group predictors.

Copyright: © 2023 Journal of Education and Health Promotion.

DOI: 10.4103/jehp.jehp_687_22
PMCID: PMC10079200
PMID: 37034856

Conflict of interest statement: There are no conflicts of interest.

227. PLoS One. 2023 Jan 27;18(1):e0280052. doi: 10.1371/journal.pone.0280052. eCollection 2023.

Involving men in cervical cancer prevention; a qualitative enquiry into male perspectives on screening and HPV vaccination in Mid-Western Uganda.

de Fouw M(1), Stroeken Y(1), Niwagaba B(2), Musheshe M(2), Tusiime J(2), Sadayo I(2), Reis R(3)(4)(5), Peters AAW(1), Beltman JJ(1).

Author information:
(1)Department of Gynaecology, Leiden University Medical Centre, Leiden, The Netherlands.
(2)Department of Technologies for Rural Transformation, African Rural University, Kagadi, Uganda.
(3)Department of Public Health and Primary Care, Leiden University Medical Center, Leiden, The Netherlands.
(4)Department of Anthropology, University of Amsterdam, Amsterdam, The Netherlands.
(5)Children's Institute, University of Cape Town, Cape Town, South Africa.

INTRODUCTION: Evidence-based preventive strategies for cervical cancer in low-resource setting have been developed, but implementation is challenged, and uptake remains low. Women and girls experience social and economic barriers to attend screening and human papillomavirus (HPV) vaccination programs. Male support has been proven successful in uptake of other reproductive healthcare services. This qualitative study with focus groups aimed to understand the perspectives of males on cervical cancer screening and HPV vaccination in Western-Uganda This knowledge could be integrated into awareness activities to increase the attendance of cervical cancer screening and HPV vaccination programs.
MATERIALS AND METHODS: Focus group discussions were conducted with men aged 25 to 60 years, who were married and/or had daughters, in Kagadi district, Mid-Western Uganda. All interviews were transcribed verbatim and thematically analyzed using an inductive approach.
RESULTS: Eleven focus group discussions were conducted with 67 men. Men were willing to support their wives for screening and their daughters for HPV

vaccination. Misperceptions such as family planning and poor personal hygiene leading to cervical cancer, and misperception of the preventative aspect of screening and vaccination were common. Women with cervical cancer suffer from stigmatization and family problems due to loss of fertility, less marital sexual activity, domestic violence and decreased economic productivity.
CONCLUSIONS: Ugandan men were willing to support cervical cancer prevention for their wives and daughters after being informed about cervical cancer. Limited knowledge among men about the risk factors and causes of cervical cancer, and about the preventative aspect of HPV vaccination and screening and their respective target groups, can limit uptake of both services. Screening and vaccination programs should actively involve men in creating awareness to increase uptake and acceptance of prevention.

Copyright: © 2023 de Fouw et al. This is an open access article distributed under the terms of the Creative Commons Attribution License, which permits unrestricted use, distribution, and reproduction in any medium, provided the original author and source are credited.

DOI: 10.1371/journal.pone.0280052
PMCID: PMC9882699
PMID: 36706114 [Indexed for MEDLINE]

Conflict of interest statement: The authors have declared that no competing interests exist.

228. Vaccines (Basel). 2023 Jan 26;11(2):268. doi: 10.3390/vaccines11020268.

Ebola Virus Disease Vaccines: Development, Current Perspectives & Challenges.

Malik S(1), Kishore S(1), Nag S(2), Dhasmana A(3), Preetam S(4), Mitra O(2), León-Figueroa DA(5), Mohanty A(6), Chattu VK(7)(8)(9), Assefi M(10), Padhi BK(11), Sah R(12)(13).

Author information:
(1)Amity Institute of Biotechnology, Amity University Jharkhand, Ranchi 834001, Jharkhand, India.
(2)Department of Biotechnology, School of Biosciences & Technology, Vellore Institute of Technology (VIT), Tiruvalam Road, Vellore 632014, Tamil Nadu, India.
(3)Himalayan School of Biosciences, Swami Rama Himalayan University, Jolly Grant, Dehradun 248140, Uttarakhand, India.
(4)Institute of Advanced Materials, IAAM, Gammalkilsvägen 18, 59053 Ulrika, Sweden.
(5)Facultad de Medicina Humana, Universidad de San Martín de Porres, Chiclayo 15011, Peru.
(6)Department of Microbiology, All India Institute of Medical Sciences,

Gorakhpur 273008, Uttar Pradesh, India.
(7)Department of Occupational Science & Occupational Therapy, Temerty Faculty of Medicine, University of Toronto, Toronto, ON M5G 1V7, Canada.
(8)Center for Transdisciplinary Research, Saveetha Dental College, Saveetha Institute of Medical and Technical Sciences, Saveetha University, Chennai 600077, Tamil Nadu, India.
(9)Department of Community Medicine, Faculty of Medicine, Datta Meghe Institute of Medical Sciences, Wardha 442107, Maharashtra, India.
(10)Joint School of NanoScience and Nano Engineering, University of North Carolina, Greensboro, NC 27402-6170, USA.
(11)Department of Community Medicine and School of Public Health, Postgraduate Institute of Medical Education and Research, Chandigarh 160012, Punjab, India.
(12)Tribhuvan University Teaching Hospital, Institute of Medicine, Kathmandu 44600, Nepal.
(13)Dr. D.Y Patil Medical College, Hospital and Research Centre, Dr. D.Y.Patil Vidyapeeth, Pune 411018, Maharashtra, India.

The global outgoing outbreaks of Ebola virus disease (EVD) in different regions of Sudan, Uganda, and Western Africa have brought into focus the inadequacies and restrictions of pre-designed vaccines for use in the battle against EVD, which has affirmed the urgent need for the development of a systematic protocol to produce Ebola vaccines prior to an outbreak. There are several vaccines available being developed by preclinical trials and human-based clinical trials. The group of vaccines includes virus-like particle-based vaccines, DNA-based vaccines, whole virus recombinant vaccines, incompetent replication originated vaccines, and competent replication vaccines. The limitations and challenges faced in the development of Ebola vaccines are the selection of immunogenic, rapid-responsive, cross-protective immunity-based vaccinations with assurances of prolonged protection. Another issue for the manufacturing and distribution of vaccines involves post authorization, licensing, and surveillance to ensure a vaccine's efficacy towards combating the Ebola outbreak. The current review focuses on the development process, the current perspective on the development of an Ebola vaccine, and future challenges for combatting future emerging Ebola infectious disease.

DOI: 10.3390/vaccines11020268
PMCID: PMC9963029
PMID: 36851146

Conflict of interest statement: The authors declare no conflict of interest.

229. medRxiv. 2023 Jan 20:2023.01.16.23284626. doi: 10.1101/2023.01.16.23284626. Preprint.

High proportion of Ugandans with pre-pandemic SARS-CoV-2 cross-reactive CD4+ and CD8+ T-cell responses.

Namuniina A(1), Muyanja ES(2), Biribawa VM(1), Okech BA(1), Ssemaganda A(3), Price MA(4)(5), Hills N(6), Nanteza A(7), Bagaya BS(8)(9), Weiskopf D(10), Riou C(11)(12), Reynolds SJ(13)(14)(15), Galwango RM(15), Redd AD(12)(13)(14).

Author information:
(1)UVRI-IAVI HIV Vaccine Program, Entebbe, Uganda.
(2)Emory University, Department of Pathology and Laboratory Medicine.
(3)Department of Medical Microbiology and Infectious Diseases, University of Manitoba, Winnipeg, MB, Canada.
(4)IAVI, Epidemiology & Biostatistics, University of California San Francisco, San Francisco, USA.
(5)Department of Epidemiology and Biostatistics, University of California San Francisco, San Francisco, USA.
(6)University of California, San Francisco, Department of Neurology, Department of Epidemiology and Biostatistics.
(7)Makerere University, College of Veterinary Medicine, Animal Resources & Bio-security, Kampala Uganda.
(8)Makerere University, College of Health Sciences, School of Biomedical Sciences, Department of Immunology and Molecular Biology, Kampala Uganda.
(9)Makerere University, College of Health Sciences, Integrated Biorepository of H3-Africa-Uganda (IBRH3AU), COVID-19 Biobank (COV-BANK).
(10)Center for Infectious Disease and Vaccine Research, La Jolla Institute for Immunology, La Jolla, CA, USA.
(11)University of Cape Town, Cape Town, South Africa.
(12)Institute of Infectious Disease and Molecular Medicine, University of Cape Town, Cape Town, South Africa.
(13)Division of Intramural Research, NIAID, NIH, Baltimore MD, USA.
(14)Division of Infectious Diseases, Johns Hopkins School of Medicine, Baltimore, MD, USA.
(15)Rakai Health Sciences Program, Kalisizo, Uganda.

Update in
PLOS Glob Public Health. 2023 Aug 16;3(8):e0001566.

The estimated mortality rate of the SARS-CoV-2 pandemic varied greatly around the world with multiple countries in East, Central, and West Africa having significantly lower rates of COVID-19 related fatalities than many resource-rich nations with significantly earlier wide-spread access to life-saving vaccines. One possible reason for this lower mortality could be the presence of pre-existing cross-reactive immunological responses in these areas of the world. To explore this hypothesis, stored peripheral blood mononuclear cells (PBMC) from Ugandans collected from 2015-2017 prior to the COVID-19 pandemic (n=29) and from hospitalized Ugandan COVID-19 patients (n=3) were examined using flow-cytometry for the presence of pre-existing SARS-CoV-2 cross-reactive CD4+ and CD8+ T-cell populations using four T-cell epitope mega pools. Of pre-pandemic participants, 89.7% (26/29) had either CD4+ or CD8+, or both,

SARS-CoV-2 specific T-cell responses. Specifically, CD4+ T-cell reactivity (72.4%) and CD8+ T-cell reactivity (65.5%) were relatively similar, and 13 participants (44.8%) had both types of cross-reactive types of T-cells present. There were no significant differences in response by sex in the population. The rates of cross-reactive T-cell populations in these Ugandans is higher than previous estimates from resource-rich countries like the United States (20-50% reactivity). It is unclear what role, if any, this cross-reactivity played in decreasing COVID-19 related mortality in Uganda and other African countries, but does suggest that a better understanding of global pre-existing immunological cross-reactivity could be an informative data of epidemiological intelligence moving forward.

DOI: 10.1101/2023.01.16.23284626
PMCID: PMC9882430
PMID: 36711579

Conflict of interest statement: Competing interests: LJI has filed for patent protection for various aspects of T cell epitope and vaccine design work.

230. J Pharm Policy Pract. 2023 Jan 18;16(1):8. doi: 10.1186/s40545-023-00516-5.

Cold chain management practices of non-vaccine commodities in public health facilities of Rwenzori Region, Western Uganda.

Tumwine Y(1)(2), Rajab K(1)(3), Kutyabami P(1)(3), Nyandwi JB(1), Asingizwe D(4).

Author information:
(1)EAC Regional Centre of Excellence for Vaccines, Immunization, and Health Supply Chain Management, College of Medicine and Health Sciences, University of Rwanda, Kigali, Rwanda.
(2)National Medical Stores, Kampala, Uganda.
(3)College of Health Sciences, Makerere University, Kampala, Uganda.
(4)EAC Regional Centre of Excellence for Vaccines, Immunization, and Health Supply Chain Management, College of Medicine and Health Sciences, University of Rwanda, Kigali, Rwanda. adomina23@gmail.com.

BACKGROUND: Despite the evidence that cold chain management practices affect the potency and effectiveness of both vaccines and non-vaccine commodities, most of the researches in Uganda focus on vaccines. This study assessed the cold chain management practices of non-vaccine cold chain commodities in public health facilities of the Rwenzori Region, Uganda.
METHODS: A cross-sectional study was conducted in a random sample of 69 level III and IV health facilities. The respondents were store managers at health facilities. Data on stock and storage management practices and level of knowledge on cold chain management were assessed. Data were collected and

entered into Microsoft excel 2017, cleaned, and later exported into IBM SPSS
version 26 for analysis. The management practices were graded as poor (< 50%
score), fair (50-75% score), or good (> 75% score).
RESULTS: Results from the 69 facilities indicated that the stock management
practices were graded as fair for lesser than half of the facilities 28 (40.6%).
Few facilities were correctly filling stock cards 20 (29%) and conducting
physical inventories 19 (27.5%). The refrigerator storage management practices
were fair for nearly half of the facilities 32 (46.4%). Among the facilities
that had a refrigerator 53 (76.8%), 39 (70.9%) utilized it for storing both
vaccines and non-vaccine commodities. The cold chain management practices at
service delivery points were fair for 32 (46.4%) health centers. A larger
proportion of the participants 69 (65%) had knowledge of cold chain storage.
Most of the participants 47 (67.8%) were knowledgeable about the heat
sensitivity of the cold chain commodities, however, almost half (48.1%) of them
lacked knowledge on refrigerator use.
CONCLUSION: The management practices for non-vaccine cold chain commodities in
health centers were fair. More than a third of the facility store managers
lacked knowledge on cold chain management practices for non-vaccine commodities.
There is a need to strengthen the capacity of the facilities' store managers and
provide equipment specific for non-vaccine cold chain commodities.

© 2023. The Author(s).

DOI: 10.1186/s40545-023-00516-5
PMCID: PMC9850693
PMID: 36653823

Conflict of interest statement: The authors declare that they do not have any
competing interests.

231. J Pharm Policy Pract. 2023 Jan 17;16(1):6. doi: 10.1186/s40545-023-00517-4.

Leveraging electronic logistics management information systems to enhance and
optimize supply chain response during public health emergencies: lessons from
COVID-19 response in Uganda.

Wasswa JH(1), Oundo H(2), Oteba MO(2), Komakech H(2), Ochola I(2), Mwebaze S(2),
Okidi D(2), Kirunda A(2), Nakadde S(2), Oteba NO(3), Lugada E(2).

Author information:
(1)Management Sciences for Health, Uganda, Plot 15, Princess Anne Drive,
Bugolobi, P. O. Box 71419, Kampala, Uganda. jhwasswa@ug-sscs.org.
(2)Management Sciences for Health, Uganda, Plot 15, Princess Anne Drive,
Bugolobi, P. O. Box 71419, Kampala, Uganda.
(3)Ministry of Health, Uganda, Plot 6, Lourdel Road, Wandegeya, P. O. Box 7272,
Kampala, Uganda.

BACKGROUND: Health supply chain is crucial for proper functioning of a health system and advancing national and international health security goals. The Coronavirus 2019 pandemic caused major challenges for health supply chain systems in Uganda and globally.
OBJECTIVES: This study involved literature review to examine how the electronic logistics management information system and related digital systems were harnessed be best support public health emergencies.
METHODS: We describe how the health supply chain system leveraged the emergency Electronic Logistic Management Information System developed during the Ebola epidemic in 2019 to support the COVID-19 response in Uganda. The findings are based on the analysis of reports, guidelines, and discussions with stakeholders involved in implementing the electronic Management Information System during the COVID-19 response. Lessons and experiences are shared on how the system supported data visibility, use and health commodity management.
RESULTS: A web-based emergency Electronic Management Information System was developed to support the supply chain system during preparedness and response to the Ebola Virus. The system facilitated coordination, information management and provided real-time data for planning, decision making, and distribution of commodities during the COVID-19 response. To address any human resource challenges, 863 staff were trained and mentored in the use of the system. The system enabled the Ministry of Health to track the distribution of Medical Counter Measures through the warehouses, eight regional pre-positioning centers, and over 2000 user units in 136 district vaccine stores. In addition, the system provided quality data for the quantification and monitoring of commodities at all levels of care. Over 1800 bulk orders were processed through the system. Real time stock status reports were transmitted from all national, regional, district and health facility levels. Procurement tracking reports, stock gap analysis and partner contribution were all accessible and visible in the system. This supported the Ministry of Health's resource mobilization and coordination efforts.
CONCLUSIONS: Availability of reliable, quality real-time data are essential for effective decision making during public health emergencies. The emergency Electronic Logistic Management Information Systems supported health authorities to mount coordinated and effective responses to ensure timely availability of commodities and supplies to support the COVID-19 pandemic response. Lessons learnt from the Ebola epidemic response were translated into actions that enabled effective preparedness and response to the COVID-19 pandemic.

© 2023. The Author(s).

DOI: 10.1186/s40545-023-00517-4
PMCID: PMC9844934
PMID: 36650571

Conflict of interest statement: The authors declare that they have no competing interests.

232. PLoS One. 2023 Jan 17;18(1):e0276351. doi: 10.1371/journal.pone.0276351. eCollection 2023.

Predicting the combined effects of case isolation, safe funeral practices, and contact tracing during Ebola virus disease outbreaks.

Bouba A(1)(2), Helle KB(3), Schneider KA(1).

Author information:
(1)Hochschule Mittweida, University of Applied Sciences Mittweida, Mittweida, Germany.
(2)African Institute for Mathematical Sciences (AIMS), Limbe, Cameroon.
(3)Hochschule Merseburg, University of Applied Sciences Merseburg, Mittweida, Germany.

BACKGROUND: The recent outbreaks of Ebola virus disease (EVD) in Uganda and the Marburg virus disease (MVD) in Ghana reflect a persisting threat of Filoviridae to the global health community. Characteristic of Filoviridae are not just their high case fatality rates, but also that corpses are highly contagious and prone to cause infections in the absence of appropriate precautions. Vaccines against the most virulent Ebolavirus species, the Zaire ebolavirus (ZEBOV) are approved. However, there exists no approved vaccine or treatment against the Sudan ebolavirus (SUDV) which causes the current outbreak of EVD. Hence, the control of the outbreak relies on case isolation, safe funeral practices, and contact tracing. So far, the effectiveness of these control measures was studied only separately by epidemiological models, while the impact of their interaction is unclear.
METHODS AND FINDINGS: To sustain decision making in public health-emergency management, we introduce a predictive model to study the interaction of case isolation, safe funeral practices, and contact tracing. The model is a complex extension of an SEIR-type model, and serves as an epidemic preparedness tool. The model considers different phases of the EVD infections, the possibility of infections being treated in isolation (if appropriately diagnosed), in hospital (if not properly diagnosed), or at home (if the infected do not present to hospital for whatever reason). It is assumed that the corpses of those who died in isolation are buried with proper safety measures, while those who die outside isolation might be buried unsafely, such that transmission can occur during the funeral. Furthermore, the contacts of individuals in isolation will be traced. Based on parameter estimates from the scientific literature, the model suggests that proper diagnosis and hence isolation of cases has the highest impact in reducing the size of the outbreak. However, the combination of case isolation and safe funeral practices alone are insufficient to fully contain the epidemic under plausible parameters. This changes if these measures are combined with contact tracing. In addition, shortening the time to successfully trace back contacts contribute substantially to contain the outbreak.

CONCLUSIONS: In the absence of an approved vaccine and treatment, EVD management by proper and fast diagnostics in combination with epidemic awareness are fundamental. Awareness will particularly facilitate contact tracing and safe funeral practices. Moreover, proper and fast diagnostics are a major determinant of case isolation. The model introduced here is not just applicable to EVD, but also to other viral hemorrhagic fevers such as the MVD or the Lassa fever.

Copyright: © 2023 Bouba et al. This is an open access article distributed under the terms of the Creative Commons Attribution License, which permits unrestricted use, distribution, and reproduction in any medium, provided the original author and source are credited.

DOI: 10.1371/journal.pone.0276351
PMCID: PMC9844901
PMID: 36649296 [Indexed for MEDLINE]

Conflict of interest statement: The authors have declared that no competing interests exist.

233. Wellcome Open Res. 2023 Jan 16;8:22. doi: 10.12688/wellcomeopenres.18681.1. eCollection 2023.

Pf7: an open dataset of Plasmodium falciparum genome variation in 20,000 worldwide samples.

MalariaGEN; Abdel Hamid MM(1), Abdelraheem MH(1)(2), Acheampong DO(3), Ahouidi A(4), Ali M(5), Almagro-Garcia J(5), Amambua-Ngwa A(5)(6), Amaratunga C(7), Amenga-Etego L(8)(9), Andagalu B(10), Anderson T(11), Andrianaranjaka V(12), Aniebo I(13), Aninagyei E(14), Ansah F(8), Ansah PO(9), Apinjoh T(15), Arnaldo P(16), Ashley E(17)(18), Auburn S(19)(20), Awandare GA(8), Ba H(21), Baraka V(22)(23), Barry A(24)(25)(26), Bejon P(27), Bertin GI(28), Boni MF(20)(29), Borrmann S(30), Bousema T(31)(32), Bouyou-Akotet M(33), Branch O(34), Bull PC(27)(35), Cheah H(36), Chindavongsa K(37), Chookajorn T(38), Chotivanich K(38), Claessens A(6)(39), Conway DJ(31), Corredor V(40), Courtier E(5), Craig A(41)(42), D'Alessandro U(6), Dama S(43), Day N(17)(18), Denis B(42), Dhorda M(17)(44), Diakite M(43)(45), Djimde A(43), Dolecek C(20), Dondorp A(17)(18), Doumbia S(43)(45), Drakeley C(31), Drury E(5), Duffy P(7), Echeverry DF(46)(47), Egwang TG(48), Enosse SMM(16), Erko B(49), Fairhurst RM(50), Faiz A(51), Fanello CA(17), Fleharty M(52), Forbes M(5), Fukuda M(53), Gamboa D(54), Ghansah A(55), Golassa L(49), Goncalves S(5), Harrison GLA(24), Healy SA(7), Hendry JA(56), Hernandez-Koutoucheva A(5), Hien TT(18)(29), Hill CA(57), Hombhanje F(58), Hott A(59), Htut Y(60), Hussein M(1), Imwong M(38), Ishengoma D(22)(61), Jackson SA(62), Jacob CG(5), Jeans J(5), Johnson KJ(5), Kamaliddin C(28)(63), Kamau E(64), Keatley J(5), Kochakarn T(38), Konate DS(43), Konaté A(65), Kone A(43), Kwiatkowski DP(5), Kyaw MP(66)(67), Kyle D(59)(68), Lawniczak M(5), Lee SK(52), Lemnge M(22), Lim P(7)(69), Lon C(70), Loua KM(71)(72), Mandara CI(22), Marfurt

J(19), Marsh K(20)(27), Maude RJ(17)(18)(73), Mayxay M(18)(74)(75), Maïga-Ascofaré O(43)(76)(77), Miotto O(5)(17)(78), Mita T(79), Mobegi V(80), Mohamed AO(81), Mokuolu OA(82), Montgomery J(42)(83), Morang'a CM(8), Mueller I(24)(84), Murie K(5), Newton PN(18)(74), Ngo Duc T(85), Nguyen T(5), Nguyen TN(18)(29), Nguyen Thi Kim T(29), Nguyen Van H(85), Noedl H(86)(87), Nosten F(18)(88), Noviyanti R(89), Ntui VN(15), Nzila A(90), Ochola-Oyier LI(27), Ocholla H(91)(92), Oduro A(9), Omedo I(5)(27), Onyamboko MA(93), Ouedraogo JB(94), Oyebola K(95)(96), Oyibo WA(97), Pearson R(5), Peshu N(27), Phyo AP(17)(98), Plowe CV(99), Price RN(17)(18)(19), Pukrittayakamee S(38), Quang HH(100), Randrianarivelojosia M(101)(102), Rayner JC(103), Ringwald P(104), Rosanas-Urgell A(105), Rovira-Vallbona E(105), Ruano-Rubio V(52), Ruiz L(106), Saunders D(107), Shayo A(108), Siba P(109), Simpson VJ(5), Sissoko MS(43), Smith C(5), Su XZ(7), Sutherland C(31), Takala-Harrison S(110), Talman A(111), Tavul L(109), Thanh NV(29), Thathy V(27)(112), Thu AM(88), Toure M(43), Tshefu A(113), Verra F(114), Vinetz J(54)(115), Wellems TE(7), Wendler J(7)(116), White NJ(17)(18), Whitton G(5), Yavo W(65)(117), van der Pluijm RW(17).

Author information:
(1)Institute of Endemic Diseases, University of Khartoum, Khartoum, Sudan.
(2)Nuclear Applications In Biological Sciences, Sudan Atomic Energy Commission, Khartoum, Sudan.
(3)Department of Biomedical Sciences, School of Allied Health Sciences, University of Cape Coast, Cape Coast, Ghana.
(4)Health Research Epidemiological Surveillance and Training Institute (IRESSEF), Université Cheikh Anta Diop, Dakar, Senegal.
(5)Wellcome Sanger Institute, Hinxton, UK.
(6)Medical Research Council Unit The Gambia at the London School of Hygiene and Tropical Medicine, Banjul, The Gambia.
(7)National Institute of Allergy and Infectious Diseases (NIAID), NIH, Maryland, USA.
(8)West African Centre for Cell Biology of Infectious Pathogens (WACCBIP), University of Ghana, Legon, Ghana.
(9)Navrongo Health Research Centre, Ghana Health Service, Navrongo, Ghana.
(10)United States Army Medical Research Directorate-Africa, Kenya Medical Research Institute/Walter Reed Project, Kisumu, Kenya.
(11)Texas Biomedical Research Institute, San Antonio, USA.
(12)Université d'Antananarivo, Antananarivo, Madagascar.
(13)Health Strategy and Delivery Foundation, Lagos, Nigeria.
(14)Department of Biomedical Sciences, School of Basic and Biomedical Sciences, University of Health & Allied Sciences, Ho, Ghana.
(15)University of Buea, Buea, Cameroon.
(16)Instituto Nacional de Saúde (INS), Maputo, Mozambique.
(17)Mahidol-Oxford Tropical Medicine Research Unit (MORU), Bangkok, Thailand.
(18)Centre for Tropical Medicine and Global Health, University of Oxford, Oxford, UK.
(19)Menzies School of Health Research, Charles Darwin University, Darwin, Northern Territory, Australia.

(20)Nuffield Department of Medicine, University of Oxford, UK.
(21)Institut National de Recherche en Santé Publique, Nouakchott, Mauritania.
(22)National Institute for Medical Research (NIMR), Dar es Salaam, Tanzania.
(23)Department of Epidemiology, International Health Unit, Universiteit Antwerpen, Antwerp, Belgium.
(24)Walter and Eliza Hall Institute, Melbourne, Australia.
(25)Deakin University, Geelong, Australia.
(26)Burnet Institute, Melbourne, Australia.
(27)KEMRI Wellcome Trust Research Programme, Kilifi, Kenya.
(28)Institute of Research for Development (IRD), Paris, France.
(29)Oxford University Clinical Research Unit (OUCRU), Ho Chi Minh City, Vietnam.
(30)Institute for Tropical Medicine, University of Tübingen, Tübingen, Germany.
(31)London School of Hygiene and Tropical Medicine, London, UK.
(32)Radboud University Medical Center, Nijmegen, The Netherlands.
(33)Department of Parasitology-Mycology, Université des Sciences de la Santé, Libreville, Gabon.
(34)NYU School of Medicine Langone Medical Center, New York, USA.
(35)Department of Pathology, University of Cambridge, Cambridge, UK.
(36)National Center for Parasitology, Entomology and Malaria Control, Phnom Penh, Cambodia.
(37)Center of Malariology, Parasitology and Entomology (CMPE), Vientiane, Lao People's Democratic Republic.
(38)Mahidol University, Bangkok, Thailand.
(39)LPHI, MIVEGEC, INSERM, CNRS, IRD, University of Montpellier, Montpellier, France.
(40)National University of Colombia, Bogota, Colombia.
(41)Liverpool School of Tropical Medicine, Liverpool, UK.
(42)Malawi-Liverpool-Wellcome Trust Clinical Research Program, Blantyre, Malawi.
(43)Malaria Research and Training Centre, University of Science, Techniques and Technologies of Bamako, Bamako, Mali.
(44)WorldWide Antimalarial Resistance Network - Asia Regional Centre, Bangkok, Thailand.
(45)University Clinical Research Center (UCRC), Bamako, Mali.
(46)Departamento de Microbiología, Universidad del Valle, Cali, Colombia.
(47)Centro Internacional de Entrenamiento e Investigaciones Médicas - CIDEIM, Cali, Colombia.
(48)Biotech Laboratories, Kampala, Uganda.
(49)Aklilu Lemma Institute of Pathobiology, Addis Ababa University, Addis Ababa, Ethiopia.
(50)National Institutes of Health (NIH), Maryland, USA.
(51)Dev Care Foundation, Dhaka, Bangladesh.
(52)Broad Institute of Harvard and MIT and Harvard, Cambridge, MA, USA.
(53)Department of Immunology and Medicine, US Army Medical Component, Armed Forces Research Institute of Medical Sciences (USAMC-AFRIMS), Bangkok, Thailand.
(54)Laboratorio ICEMR-Amazonia, Laboratorios de Investigacion y Desarrollo, Facultad de Ciencias y Filosofia, Universidad Peruana Cayetano Heredia, Lima, Peru.

(55)Nogouchi Memorial Institute for Medical Research, Legon-Accra, Ghana.
(56)Wellcome Centre for Human Genetics, University of Oxford, Oxford, UK.
(57)Department of Entomology, Purdue University, West Lafayette, USA.
(58)Centre for Health Research & Diagnostics, Divine Word University, Madang, Papua New Guinea.
(59)University of South Florida, Tampa, USA.
(60)Department of Medical Research, Yangon, Myanmar.
(61)East African Consortium for Clinical Research (EACCR), Dar es Salaam, Tanzania.
(62)Center for Applied Genetic Technologies, University of Georgia, Athens, GA, USA.
(63)The University of Calgary, Calgary, Canada.
(64)U.S. Military HIV Research Program, Walter Reed Army Institute of Research, Silver Spring, MD, USA.
(65)University Félix Houphouët-Boigny, Abidjan, Cote d'Ivoire.
(66)Myanmar Oxford Clinical Research Unit, University of Oxford, Yangon, Myanmar.
(67)University of Public Health, Yangon, Myanmar.
(68)University of Georgia, Athens, USA.
(69)Medical Care Development International, Maryland, USA.
(70)National Institute of Allergy and Infectious Diseases, Phnom Penh, Cambodia.
(71)University Gamal Abdel Nasser of Conakry, Conakry, Guinea.
(72)Institut National de Santé Publique, Conakry, Guinea.
(73)Harvard TH Chan School of Public Health, Harvard University, Boston, USA.
(74)Lao-Oxford-Mahosot Hospital-Wellcome Trust Research Unit, Microbiology Laboratory, Mahosot Hospital, Vientiane, Lao People's Democratic Republic.
(75)Institute of Research and Education Development (IRED), University of Health Sciences, Ministry of Health, Vientiane, Lao People's Democratic Republic.
(76)Bernhard Nocht Institute for Tropical Medicine, Hamburg, Germany.
(77)Research in Tropical Medicine, Kwame Nkrumah University of Sciences and Technology, Kumasi, Ghana.
(78)MRC Centre for Genomics and Global Health, Big Data Institute, Oxford University, Oxford, UK.
(79)Juntendo University, Tokyo, Japan.
(80)Department of Biochemistry and Centre for Biotechnology and Bioinformatics, University of Nairobi, Nairobi, Kenya.
(81)Faculty of Medicine, University of Khartoum, Khartoum, Sudan.
(82)Department of Paediatrics and Child Health, University of Ilorin, Ilorin, Nigeria.
(83)World Mosquito Program, Monash University, Melbourne, Australia.
(84)University of Melbourne, Melbourne, Australia.
(85)National Institute of Malariology, Parasitology and Entomology (NIMPE), Hanoi, Vietnam.
(86)MARIB - Malaria Research Initiative Bandarban, Bandarban, Bangladesh.
(87)Medical University of Vienna, Vienna, Austria.
(88)Shoklo Malaria Research Unit, Mahidol-Oxford Tropical Medicine Research Unit, Faculty of Tropical Medicine, Mahidol University, Mae Sot, Thailand.

(89)Eijkman Institute for Molecular Biology, Jakarta, Indonesia.
(90)King Fahid University of Petroleum and Minerals (KFUMP), Dhahran, Saudi Arabia.
(91)KEMRI Centres for Disease Control and Prevention (CDC) Research Program, Kisumu, Kenya.
(92)Centre for Bioinformatics and Biotechnology, University of Nairobi, Nairobi, Kenya.
(93)Kinshasa School of Public Health, University of Kinshasa, Kinshasa, Congo, Democratic Republic.
(94)Institut de Recherche en Sciences de la Santé, Ouagadougou, Burkina Faso.
(95)Nigerian Institute of Medical Research, Lagos, Nigeria.
(96)Parasitology and Bioinformatics Unit, Faculty of Science, University of Lagos, Lagos, Nigeria.
(97)College of Medicine, University of Lagos, Lagos, Nigeria.
(98)Shoklo Malaria Research Unit, Bangkok, Thailand.
(99)University of Maryland School of Medicine, Maryland, USA.
(100)Institute of Malariology, Parasitology, and Entomology (IMPE) Quy Nhon, Ministry of Health, Quy Nhon, Vietnam.
(101)Institut Pasteur de Madagascar, Antananarivo, Madagascar.
(102)Universités d'Antananarivo et de Mahajanga, Antananarivo, Madagascar.
(103)Cambridge Institute for Medical Research, University of Cambridge, Cambridge, UK.
(104)World Health Organization (WHO), Geneva, Switzerland.
(105)Institute of Tropical Medicine Antwerp, Antwerp, Belgium.
(106)Universidad Nacional de la Amazonia Peruana, Iquitos, Peru.
(107)Department of Medicine, Uniformed Services University, Bethesda, MD, USA.
(108)Nelson Mandela Institute of Science and Technology, Arusha, Tanzania.
(109)Papua New Guinea Institute of Medical Research, Goroka, Papua New Guinea.
(110)Center for Vaccine Development and Global Health, University of Maryland, School of Medicine, Baltimore, MD, USA.
(111)MIVEGEC, Université de Montpellier, IRD, CNRS, Montpellier, France.
(112)Department of Microbiology and Immunology, Columbia University Irving Medical Center, New York, NY, USA.
(113)University of Kinshasa, Kinsasha, Congo, Democratic Republic.
(114)Sapienza University of Rome, Rome, Italy.
(115)Yale School of Medicine, New Haven, CT, USA.
(116)Seattle Children's Hospital, Seattle, USA.
(117)Malaria Research and Control Center of the National Institute of Public Health, Abidjan, Cote d'Ivoire.

We describe the MalariaGEN Pf7 data resource, the seventh release of Plasmodium falciparum genome variation data from the MalariaGEN network. It comprises over 20,000 samples from 82 partner studies in 33 countries, including several malaria endemic regions that were previously underrepresented. For the first time we include dried blood spot samples that were sequenced after selective whole genome amplification, necessitating new methods to genotype copy number variations. We identify a large number of newly emerging crt mutations in parts

of Southeast Asia, and show examples of heterogeneities in patterns of drug resistance within Africa and within the Indian subcontinent. We describe the profile of variations in the C-terminal of the csp gene and relate this to the sequence used in the RTS,S and R21 malaria vaccines. Pf7 provides high-quality data on genotype calls for 6 million SNPs and short indels, analysis of large deletions that cause failure of rapid diagnostic tests, and systematic characterisation of six major drug resistance loci, all of which can be freely downloaded from the MalariaGEN website.

Copyright: © 2023 MalariaGEN et al.

DOI: 10.12688/wellcomeopenres.18681.1
PMCID: PMC9971654
PMID: 36864926

Conflict of interest statement: No competing interests were disclosed.

234. medRxiv. 2023 Jan 13:2022.12.05.22282933. doi: 10.1101/2022.12.05.22282933. Preprint.

Efficacy of a bivalent (D614 + B.1.351) SARS-CoV-2 Protein Vaccine.

Dayan GH(1), Rouphael N(2), Walsh SR(3), Chen A(1), Grunenberg N(4), Allen M(5), Antony J(6), Asante KP(7), Bhate AS(8), Beresnev T(5), Bonaparte MI(1), Ceregido MA(9), Dobrianskyi D(10), Fu B(1), Grillet MH(11), Keshtkar-Jahromi M(12)(13), Juraska M(4), Kee JJ(4), Kibuuka H(14), Koutsoukos M(9), Masotti R(1), Michael NL(15), Reynales H(16), Robb ML(17), Villagómez Martínez SM(18), Sawe F(19), Schuerman L(9), Tong T(5), Treanor J(20), Wartel TA(21), Diazgranados CA(1), Chicz RM(22), Gurunathan S(1), Savarino S(1), Sridhar S(23); VAT00008 study team.

Author information:
(1)Sanofi, Swiftwater, PA, USA.
(2)Emory University Hop eClininc, Atlanta, GA, USA.
(3)Harvard Medical School, Boston, MA, USA.
(4)Fred Hutchinson Cancer Center, Seattle, WA, USA.
(5)National Institute of Allergy and Infectious Diseases / National Institutes of Health, Bethedsa, MA, USA.
(6)Sanofi, Frankfurt, Germany.
(7)Research and Development Division, Ghana Health Service, Kintampo North Municipality, Ghana.
(8)Jeevan Rekha Hospital, Belgavi, India.
(9)GSK, Wavre, Belgium.
(10)Medical Centre, Medical Clinic Blagomed LLC, Kyiv, Ukraine.
(11)Sanofi, Lyon, France.
(12)National Institute of Health, Rockville, Maryland.

(13)John Hopkins University School of Medicine, Baltimore, Maryland.
(14)Makerere University Walter Reed Project, Kampala, Uganda.
(15)Walter Reed Army Institute of Research, MD, USA.
(16)Centro de Attencion e Investigation Medica S.A.S. - Caimed Chía, Chía, Colombia.
(17)The Henry M Jackson Foundation for the Advancement of Military Medicine, Bethesda, MA, USA.
(18)Hospital de Temixco en acuerdo con el Instituto Nacional de Pediatria, México.
(19)Kenya Medical Research Institute - US Army Medical Research, Kisumu, Kenya.
(20)Tunnell Government Services in support of Biomedical Advanced Research and Development Authority (BARDA), Administration for Strategic Preparedness and Response (ASPR), Department of Health and Human Services (HHS), Washington, DC, USA.
(21)International Vaccine Institute, Seoul, South Korea.
(22)Sanofi, Cambridge, MA, USA.
(23)Sanofi, Reading, UK.

BACKGROUND: COVID-19 vaccines with alternative strain compositions are needed to provide broad protection against newly emergent SARS-CoV-2 variants of concern.
METHODS: We conducted a global Phase 3, multi-stage efficacy study (NCT04904549) among adults aged ≥18 years. Participants were randomized 1:1 to receive two intramuscular injections 21 days apart of a bivalent SARS-CoV-2 recombinant protein vaccine with AS03-adjuvant (5 µg of ancestral (D614) and 5 µg of B.1.351 [beta] variant spike protein) or placebo. Symptomatic COVID-19 was defined as laboratory-confirmed COVID-19 with COVID-19-like illness (CLI) symptoms. The primary efficacy endpoint was the prevention of symptomatic COVID-19 ≥14 days after the second injection (post-dose 2 [PD2]).
RESULTS: Between 19 Oct 2021 and 15 Feb 2022, 12,924 participants received ≥1 study injection. 75% of participants were SARS-CoV-2 non-naïve. 11,416 participants received both study injections (efficacy-evaluable population [vaccine, n=5,736; placebo, n=5,680]). Up to 15 March 2022, 121 symptomatic COVID-19 cases were reported (32 in the vaccine group and 89 in the placebo group) ≥14 days PD2 with a vaccine efficacy (VE) of 64.7% (95% confidence interval [CI] 46.6; 77.2%). VE was 75.1% (95% CI 56.3; 86.6%) in non-naïve and 30.9% (95% CI -39.3; 66.7%) in naïve participants. Viral genome sequencing identified the infecting strain in 68 cases (Omicron [BA.1 and BA.2 subvariants]: 63; Delta: 4; Omicron and Delta: 1). The vaccine was well-tolerated and had an acceptable safety profile.
CONCLUSIONS: A bivalent vaccine conferred heterologous protection against symptomatic infection with newly emergent Omicron (BA.1 and BA.2) in non-naïve adults 18-59 years of age.

DOI: 10.1101/2022.12.05.22282933
PMCID: PMC9753788
PMID: 36523415

Conflict of interest statement: Declaration of interests GHD, MIB, BF, M-HG, CAG, RMC, SS are Sanofi employees. MIB, BF, M-HG, CAG, RMC, SS hold stock or stock options in Sanofi. SS hold patents pending on COVID-19 vaccine. RMC has Received institutional funding from BARDA for the present study; has received support for attending meetings and/or travel from Sanofi; and holds patents planned, issued or pending from Sanofi. M-HG has received payment or honoraria for lectures, presentations, speakers' bureaus, manuscript writing or educational events from Sanofi. NR has received institutional funding from the National Institutes of Health; and institutional grants or contracts from Merck, Sanofi, Quidel, Pfizer and Lilly. SRW has received institutional funding from Sanofi and the National Institute of Allergy and Immunology/National Institutes of Health; and institutional grants or contracts from Janssen Vaccines/Johnson & Johnson, Moderna Tx, Vir Biotechnology and Worcester HIV vaccine; has participated on data safety monitoring or advisory boards for Janssen Vaccines/Johnson & Johnson; and his spouse holds stock/stock options in Regeneron Pharmaceuticals. NG has received institutional funding from Sanofi, GSK and the National Institute of Allergy and Immunology/National Institutes of Health; and is in receipt of grants or contracts from the NIH/NIAID/DAIDS. MA is an employee of the NIAID, which funded aspects of the current study. MAC and MK are employees of GSK and owns shares in the GSK group of companies. LS is an employee of the GSK group of companies. MJ and JJK have received institutional support from Sanofi and the NIAID/NIH with respect to this study. MLR has received institutional support/contracts for the present manuscript from WRAIR IPA and the US Medical Research and Development Command. MA is an employee of the NIAID, which funded aspects of the current study; The NIAID provides grant funding to the HIV Vaccine Trials Network (HVTN) Leadership and Operations Center (UM1 AI 68614HVTN), the Statistics and Data Management Center (UM1 AI 68635), the HVTN Laboratory Center (UM1 AI 68618), the HIV Prevention Trials Network Leadership and Operations Center (UM1 AI 68619), the AIDS Clinical Trials Group Leadership and Operations Center (UM1 AI 68636), and the Infectious Diseases Clinical Research Consortium leadership group 5 (UM1 AI 148684-03). SSa was a Sanofi employee at the time of study conduct; and holds patents planned, issued of pending on COVID-19 vaccines. AC, JA KPA, ASB, TB, DD, MKJ, HK, RM, NM, HR, SMVM, FS, JT, TAW, SG have no interests to declare.

235. Front Immunol. 2023 Jan 9;13:1075606. doi: 10.3389/fimmu.2022.1075606. eCollection 2022.

Systematic comparison of HIV-1 Envelope-specific IgG responses induced by different vaccination regimens: Can we steer IgG recognition towards regions of viral vulnerability?

Horvath A(1)(2), Rogers L(1)(2), Pollakis G(3), Baranov O(1)(2), Pieroth N(1)(2), Joseph S(4), Chachage M(5), Heitzer A(1), Maganga L(5), Msafiri F(6), Joachim A(6), Viegas E(7), Eller LA(8)(9)(10), Kibuuka H(9), Rerks-Ngarm S(11), Pitisuttithum P(12), Nitayapan S(13), Dhitavat J(12), Premsri N(11), Fidler

S(14), Shattock RJ(14), Robb ML(8)(10), Weber J(14), McCormack S(4), Munseri PJ(6), Lyamuya E(6), Nilsson C(15)(16), Kroidl A(1)(2), Hoelscher M(1)(2), Wagner R(17)(18), Geldmacher C(1)(2), Held K(1)(2).

Author information:
(1)Division of Infectious Diseases and Tropical Medicine, University Hospital, LMU Munich, Munich, Germany.
(2)German Center for Infection Research (DZIF), Partner Site Munich, Munich, Germany.
(3)Institute of Infection Veterinary and Ecological Sciences (IVES/CIMI), University of Liverpool, Liverpool, United Kingdom.
(4)MRC Clinical Trials Unit at UCL, Institute of Clinical Trials and Methodology, University College London, London, United Kingdom.
(5)National Institute for Medical Research-Mbeya Medical Research Centre (NIMR-MMRC), Mbeya, Tanzania.
(6)Muhimbili University of Health and Allied Sciences, Dar es Salaam, Tanzania.
(7)Instituto Nacional de Saúde, Maputo, Mozambique.
(8)United States Military HIV Research Program, Silver Spring, MD, United States.
(9)Makerere University Walter Reed Project, Kampala, Uganda.
(10)Henry M. Jackson Foundation for the Advancement of Military Medicine, Bethesda, MD, United States.
(11)Department of Disease Control, Ministry of Public Health, Mueang Nonthaburi, Thailand.
(12)Faculty of Tropical Medicine, Mahidol University, Bangkok, Thailand.
(13)Armed Forces Research Institute of Medical Science, Bangkok, Thailand.
(14)Department of Medicine, Imperial College London, London, United Kingdom.
(15)Department of Laboratory Medicine, Karolinska Institute, Huddinge, Sweden.
(16)The Public Health Agency of Sweden, Solna, Sweden.
(17)Institute of Medical Microbiology and Hygiene, University Regensburg, Regensburg, Germany.
(18)Institute of Clinical Microbiology and Hygiene; University Hospital Regensburg, Regensburg, Germany.

Immunogens and vaccination regimens can influence patterns of immune-epitope recognition, steering them towards or away from epitopes of potential viral vulnerability. HIV-1 envelope (Env)-specific antibodies targeting variable region 2 (V2) or 3 (V3) correlated with protection during the RV144 trial, however, it was suggested that the immunodominant V3 region might divert antibody responses away from other relevant sites. We mapped IgG responses against linear Env epitopes in five clinical HIV vaccine trials, revealing a specific pattern of Env targeting for each regimen. Notable V2 responses were only induced in trials administering CRF01_AE based immunogens, but targeting of V3 was seen in all trials, with the soluble, trimeric CN54gp140 protein eliciting robust V3 recognition. Strong V3 targeting was linked to greater overall response, increased number of total recognised antigenic regions, and where present, stronger V2 recognition. Hence, strong induction of V3-specific

antibodies did not negatively impact the targeting of other linear epitopes in this study, suggesting that the induction of antibodies against V3 and other regions of potential viral vulnerability need not be necessarily mutually exclusive.

Copyright © 2023 Horvath, Rogers, Pollakis, Baranov, Pieroth, Joseph, Chachage, Heitzer, Maganga, Msafiri, Joachim, Viegas, Eller, Kibuuka, Rerks-Ngarm, Pitisuttithum, Nitayapan, Dhitavat, Premsri, Fidler, Shattock, Robb, Weber, McCormack, Munseri, Lyamuya, Nilsson, Kroidl, Hoelscher, Wagner, Geldmacher and Held.

DOI: 10.3389/fimmu.2022.1075606
PMCID: PMC9891136
PMID: 36741409 [Indexed for MEDLINE]

Conflict of interest statement: The authors declare that the research was conducted in the absence of any commercial or financial relationships that could be construed as a potential conflict of interest.

236. Healthcare (Basel). 2023 Jan 6;11(2):177. doi: 10.3390/healthcare11020177.

Assessment of Knowledge, Stigmatizing Attitudes and Health-Seeking Behaviors Regarding Hepatitis B Virus Infection in a Pharmacy and Community Setting in Sierra Leone: A Cross-Sectional Study.

Ghazzawi M(1), Yendewa SA(2), James PB(3), Massaquoi SP(2), Babawo LS(4), Sahr F(2), Deen GF(2)(5), Kabba M(2), Ocama P(6), Lakoh S(2)(5), Salata RA(7)(8), Yendewa GA(7)(8)(9).

Author information:
(1)KnowHep Foundation, Freetown, Sierra Leone.
(2)Ministry of Health and Sanitation, Freetown, Sierra Leone.
(3)Faculty of Health, Southern Cross University, Lismore, NSW 2480, Australia.
(4)Department of Nursing, School of Community Health Sciences, Njala University, Bo Campus, Freetown, Sierra Leone.
(5)Department of Medicine, College of Medicine and Allied Health Sciences, University of Sierra Leone, Freetown, Sierra Leone.
(6)Department of Medicine, College of Health Sciences, Makerere University, Kampala P.O. Box 7062, Uganda.
(7)Department of Medicine, Case Western Reserve University School of Medicine, Cleveland, OH 44106, USA.
(8)Division of Infectious Diseases and HIV Medicine, University Hospitals Cleveland Medical Center, Cleveland, OH 44106, USA.
(9)Johns Hopkins Bloomberg School of Public Health, Baltimore, MD 21205, USA.

Hepatitis B virus (HBV) is a major global health challenge. Emerging evidence

suggests that poor knowledge and stigma are impacting HBV control efforts in sub-Saharan Africa (SSA), but their role is not well understood. We conducted a cross-sectional study of adults aged ≥18 years in a community and pharmacy setting in Freetown, Sierra Leone. A structured questionnaire was used to assess knowledge, stigmatizing attitudes and health-seeking behaviors regarding HBV. Logistic regression was used to identify predictors of HBV knowledge and related stigma. A total of 306 adult participants were enrolled (50.7% male, 7.5% HBV positive and 11.7% vaccinated). Overall, 52.2% had good HBV knowledge and 49.3% expressed a stigmatizing attitude towards people with HBV. Notwithstanding, 72.2% stated they would receive the HBV vaccine if offered, 80.4% would take anti-HBV medication and 78.8% would be willing to attend clinic regularly. Good HBV knowledge was associated with HBV positive status (aOR 4.41; p = 0.029) and being vaccinated against HBV (aOR 3.30; p = 0.034). HBV-related stigma was associated with secondary or higher level of education (aOR 2.36; p < 0.001), good HBV knowledge (aOR 2.05; p = 0.006) and pharmacy setting (aOR 1.74, p = 0.037). These findings suggest that education and stigma reduction may benefit HBV elimination efforts in SSA.

DOI: 10.3390/healthcare11020177
PMCID: PMC9859485
PMID: 36673546

Conflict of interest statement: The authors declare no conflict of interest.

237. Viruses. 2023 Jan 3;15(1):146. doi: 10.3390/v15010146.

Combating Lassa Fever in West African Sub-Region: Progress, Challenges, and Future Perspectives.

Aloke C(1)(2), Obasi NA(2), Aja PM(3)(4)(5), Emelike CU(6), Egwu CO(2), Jeje O(1), Edeogu CO(7), Onisuru OO(1), Orji OU(3), Achilonu I(1).

Author information:
(1)Protein Structure-Function and Research Unit, School of Molecular and Cell Biology, Faculty of Science, University of the Witwatersrand, Braamfontein, Johannesburg 2050, South Africa.
(2)Department of Medical Biochemistry, Alex Ekwueme Federal University Ndufu-Alike, Abakaliki PMB 1010, Ebonyi State, Nigeria.
(3)Department of Biochemistry, Faculty of Biological Sciences, Ebonyi State University, Abakaliki PMB 053, Ebonyi State, Nigeria.
(4)Department of Biochemistry, Faculty of Medicine, Mbarara University of Science and Technology (MUST), Mbarara P.O. Box 1410, Uganda.
(5)Department of Medical Biochemistry, Kampala International University, Bushenyi, Ishaka P.O. Box 71, Uganda.
(6)Department of Physiology, Alex Ekwueme Federal University Ndufu-Alike, Abakaliki PMB 1010, Ebonyi State, Nigeria.

(7)Department of Medical Biochemistry, Faculty of Basic Medical Sciences, Ebonyi State University, Abakaliki PMB 053, Ebonyi State, Nigeria.

Lassa fever (LF) is a rodent-borne disease that threatens human health in the sub-region of West Africa where the zoonotic host of Lassa virus (LASV) is predominant. Currently, treatment options for LF are limited and since no preventive vaccine is approved for its infectivity, there is a high mortality rate in endemic areas. This narrative review explores the transmission, pathogenicity of LASV, advances, and challenges of different treatment options. Our findings indicate that genetic diversity among the different strains of LASV and their ability to circumvent the immune system poses a critical challenge to the development of LASV vaccines/therapeutics. Thus, understanding the biochemistry, physiology and genetic polymorphism of LASV, mechanism of evading host immunity are essential for development of effective LASV vaccines/therapeutics to combat this lethal viral disease. The LASV nucleoprotein (NP) is a novel target for therapeutics as it functions significantly in several aspects of the viral life cycle. Consequently, LASV NP inhibitors could be employed as effective therapeutics as they will potentially inhibit LASV replication. Effective preventive control measures, vaccine development, target validation, and repurposing of existing drugs, such as ribavirin, using activity or in silico-based and computational bioinformatics, would aid in the development of novel drugs for LF management.

DOI: 10.3390/v15010146
PMCID: PMC9864412
PMID: 36680186 [Indexed for MEDLINE]

Conflict of interest statement: The authors declare no conflict of interest.

238. Syst Rev. 2023 Jan 3;12(1):1. doi: 10.1186/s13643-022-02163-4.

Unsupervised title and abstract screening for systematic review: a retrospective case-study using topic modelling methodology.

Natukunda A(1)(2), Muchene LK(3).

Author information:
(1)Immunomodulation and Vaccines Programme, MRC/UVRI and LSHTM Uganda Research Unit, Entebbe, Uganda. natukundagnes2@gmail.com.
(2)MRC International Statistics and Epidemiology Group, Department of Infectious Disease Epidemiology, LSHTM, London, UK. natukundagnes2@gmail.com.
(3)StatsDecide Analytics and Consulting Limited, Nairobi, Kenya.

BACKGROUND: The importance of systematic reviews in collating and summarising available research output on a particular topic cannot be over-emphasized. However, initial screening of retrieved literature is significantly time and

labour intensive. Attempts at automating parts of the systematic review process have been made with varying degree of success partly due to being domain-specific, requiring vendor-specific software or manually labelled training data. Our primary objective was to develop statistical methodology for performing automated title and abstract screening for systematic reviews. Secondary objectives included (1) to retrospectively apply the automated screening methodology to previously manually screened systematic reviews and (2) to characterize the performance of the automated screening methodology scoring algorithm in a simulation study.
METHODS: We implemented a Latent Dirichlet Allocation-based topic model to derive representative topics from the retrieved documents' title and abstract. The second step involves defining a score threshold for classifying the documents as relevant for full-text review or not. The score is derived based on a set of search keywords (often the database retrieval search terms). Two systematic review studies were retrospectively used to illustrate the methodology.
RESULTS: In one case study (helminth dataset), [Formula: see text] sensitivity compared to manual title and abstract screening was achieved. This is against a false positive rate of [Formula: see text]. For the second case study (Wilson disease dataset), a sensitivity of [Formula: see text] and specificity of [Formula: see text] were achieved.
CONCLUSIONS: Unsupervised title and abstract screening has the potential to reduce the workload involved in conducting systematic review. While sensitivity of the methodology on the tested data is low, approximately [Formula: see text] specificity was achieved. Users ought to keep in mind that potentially low sensitivity might occur. One approach to mitigate this might be to incorporate additional targeted search keywords such as the indexing databases terms into the search term copora. Moreover, automated screening can be used as an additional screener to the manual screeners.

© 2022. The Author(s).

DOI: 10.1186/s13643-022-02163-4
PMCID: PMC9811792
PMID: 36597132 [Indexed for MEDLINE]

Conflict of interest statement: The authors declare that they have no competing interests.

239. Glob Public Health. 2023 Jan;18(1):2185800. doi: 10.1080/17441692.2023.2185800.

Multi-method findings on COVID-19 vaccine acceptability among urban refugee adolescents and youth in Kampala, Uganda.

Logie CH(1)(2)(3)(4), Okumu M(5), Berry I(6), McAlpine A(1), Musoke DK(7), Hakiza R(8), Perez-Brumer A(6), Baral S(9), Kyambadde P(10)(11).

Author information:
(1)Factor-Inwentash Faculty of Social Work, University of Toronto, Toronto, Canada.
(2)Women's College Research Institute, Women's College Hospital, Toronto, Canada.
(3)United Nations University Institute for Water, Environment & Health, Hamilton, Canada.
(4)Centre for Gender & Sexual Health Equity, Vancouver, Canada.
(5)School of Social Work, University of Illinois Urbana-Champaign, Urbana, IL, USA.
(6)Dalla Lana School of Public Health, University of Toronto, Toronto, Canada.
(7)International Research Consortium, Kampala, Uganda.
(8)Young African Refugees for Integral Development (YARID), Kampala, Uganda.
(9)Johns Hopkins Bloomberg School of Public Health, Johns Hopkins University, Baltimore, MD, USA.
(10)National AIDS Control Program, Ministry of Health, Kampala, Uganda.
(11)Most At Risk Population Initiative Clinic, Mulago Hospital, Kampala, Uganda.

ABSTRACTScant studies have explored COVID-19 vaccine acceptability among refugees. However, contexts of forced migration may elevate COVID-19 vulnerabilities, and suboptimal refugee immunisation rates are reported for other vaccine-preventable diseases. We conducted a multi-methods study to describe COVID-19 vaccine acceptability among urban refugee youth in Kampala, Uganda. This study uses cross-sectional survey data from a cohort study with refugees aged 16-24 in Kampala to examine socio-demographic factors associated with vaccine acceptability. A purposively sampled cohort subset (n = 24) participated in semi-structured in-depth individual interviews, as did key informants (n = 6), to explore COVID-19 vaccine acceptance. Among 326 survey participants (mean age: 19.9; standard deviation 2.4; 50.0% cisgender women), vaccine acceptance was low (18.1% reported they were very likely to accept an effective COVID-19 vaccine). In multivariable models, vaccine acceptance likelihood was significantly associated with age and country of origin. Qualitative findings highlighted COVID-19 vaccine acceptability barriers and facilitators spanning social-ecological levels, including fear of side effects and mistrust (individual level), misinformed healthcare, community and family attitudes (community level), tailored COVID-19 services for refugees (organisational and practice setting), and political support for vaccines (policy environment). These data signal the urgent need to address social-ecological factors shaping COVID-19 vaccine acceptability among Kampala's young urban refugees.Trial registration: ClinicalTrials.gov identifier: NCT04631367.

DOI: 10.1080/17441692.2023.2185800
PMID: 36883681 [Indexed for MEDLINE]

240. BMJ Glob Health. 2023 Jan;8(1):e009495. doi: 10.1136/bmjgh-2022-009495.

Adverse maternal, fetal, and newborn outcomes among pregnant women with SARS-CoV-2 infection: an individual participant data meta-analysis.

Smith ER(1), Oakley E(2), Grandner GW(2), Ferguson K(2), Farooq F(2), Afshar Y(3), Ahlberg M(4), Ahmadzia H(5), Akelo V(6), Aldrovandi G(7), Tippett Barr BA(6), Bevilacqua E(8), Brandt JS(9), Broutet N(10), Fernández Buhigas I(11), Carrillo J(12), Clifton R(13), Conry J(14), Cosmi E(15), Crispi F(16), Crovetto F(16), Delgado-López C(17), Divakar H(18), Driscoll AJ(19), Favre G(20), Flaherman VJ(21), Gale C(22), Gil MM(11), Gottlieb SL(10), Gratacós E(16), Hernandez O(23), Jones S(24), Kalafat E(25), Khagayi S(26), Knight M(27), Kotloff K(28), Lanzone A(8), Le Doare K(29)(30), Lees C(31), Litman E(5), Lokken EM(32), Laurita Longo V(33), Madhi SA(24), Magee LA(34), Martinez-Portilla RJ(35), McClure EM(36), Metz TD(37), Miller ES(38), Money D(39), Moungmaithong S(40), Mullins E(31), Nachega JB(41), Nunes MC(24), Onyango D(42), Panchaud A(43), Poon LC(40), Raiten D(44), Regan L(14), Rukundo G(29), Sahota D(40), Sakowicz A(38), Sanin-Blair J(45), Söderling J(4), Stephansson O(4), Temmerman M(46), Thorson A(10), Tolosa JE(47), Townson J(48), Valencia-Prado M(49), Visentin S(15), von Dadelszen P(50), Adams Waldorf K(32), Whitehead C(51), Yassa M(52), Tielsch JM(2); Perinatal COVID PMA Study Collaborators; Perinatal COVID PMA Study Collaborators.

Collaborators: Langenegger E, Sam-Agudu NA, Gachuno OW, Sekikubo M, Mukwege DM, Omore R, Ouma G, Onyango C, Otieno K, Were ZA, Were J, İlter PB, Mboizi R, Hookham L, Meli F, Bonanni G, Romanzi F, Torcia E, Ilio CD, Ananth CV, Hill J, Reddy A, Patrick HS, Baba V, Adam M, Mlandu P, Adam Y, Strehlau R.

Author information:
(1)Department of Global Health, The George Washington University Milken Institute School of Public Health, Washington, DC, USA emilysmith@gwu.edu.
(2)Department of Global Health, The George Washington University Milken Institute School of Public Health, Washington, DC, USA.
(3)Division of Maternal Fetal Medicine, University of California Los Angeles, Los Angeles, California, USA.
(4)Department of Medicine, Solna, Clinical Epidemiology Division, Karolinska Institute, Stockholm, Sweden.
(5)Division of Maternal-Fetal Medicine, The George Washington University School of Medicine and Health Sciences, Washington, DC, USA.
(6)Office of the Director, US Centers for Disease Control and Prevention, Kisumu, Kenya.
(7)Department of Pediatrics, University of California Los Angeles, Los Angeles, California, USA.
(8)Department of Women and Child Health, Women Health Area, Fondazione Policlinico Universitario Agostino Gemelli, IRCCS, Roma, Italy.
(9)Division of Maternal-Fetal Medicine, Department of Obstetrics, Gynecology, and Reproductive Sciences, Rutgers Robert Wood Johnson Medical School, New

Brunswick, New Jersey, USA.
(10)Department of Sexual and Reproductive Health and Research, World Health Organization, Geneve, Switzerland.
(11)Department of Obstetrics and Gynecology, Hospital Universitario de Torrejón, Madrid, Spain.
(12)Departamento de Obstetricia y Ginecologia, Universidad del Desarrollo Facultad de Medicina Clinica Alemana, Santiago, Chile.
(13)The Biostatistics Center, The George Washington University Milken Institute School of Public Health, Rockville, Maryland, USA.
(14)International Federation of Gynecology and Obstetrics, London, UK.
(15)Department of Women's and Children's Health, University of Padua, Padova, Italy.
(16)Department of Maternal-Fetal Medicine, BCNatal, Barcelona Center for Maternal-Fetal and Neonatal Medicine, Hospital Sant Joan de Déu and Hospital Clínic, Universitat de Barcelona, Barcelona, Spain.
(17)Surveillance for Emerging Threats to Mothers and Babies, Puerto Rico Department of Health, San Juan, Puerto Rico.
(18)Asian Research and Training Institute for Skill Transfer (ARTIST), Bengaluru, India.
(19)Center for Vaccine Development, University of Maryland School of Medicine, Baltimore, Maryland, USA.
(20)Materno-Fetal and Obstetrics Research Unit, Department 'Femme-Mère-Enfant', Lausanne University Hospital, Lausanne, Switzerland.
(21)Department of Pediatrics, University of California San Francisco, San Francisco, California, USA.
(22)Neonatal Medicine, School of Public Health, Imperial College London Faculty of Medicine, London, UK.
(23)Gynecology and Obstetrics, Felix Bulnes Hospital and RedSalud Clinic, Santiago, Chile.
(24)South African Medical Research Council, Vaccines and Infectious Diseases Analytics Research Unit, University of the Witwatersrand Faculty of Health Sciences, Johannesburg, South Africa.
(25)Department of Obstetrics and Gynecology, Koç University School of Medicine, Istanbul, Turkey.
(26)Center for Global Health Research, Kenya Medical Research Institute, Kisumu, Kenya.
(27)Nuffield Department of Population Health, University of Oxford, Oxford, UK.
(28)Department of Pediatrics, University of Maryland School of Medicine, Baltimore, Maryland, USA.
(29)Uganda Virus Institute and the London School of Hygiene & Tropical Medicine, Entebbe, Uganda.
(30)Pediatric Infectious Diseases Research Group, St George's University of London, London, UK.
(31)Department of Metabolism, Digestion and Reproduction, Imperial College London, London, UK.
(32)Department of Obstetrics and Gynecology, University of Washington School of Medicine, Seattle, Washington, USA.

(33)Institute of Obstetrics and Gynecology Clinic, Catholic University of Sacred Heart, Rome, Italy.
(34)Department of Women and Children's Health, School of Life Course and Population Sciences, King's College London, London, UK.
(35)Clinical Research Division, National Institute of Perinatology, Mexico City, Mexico.
(36)RTI International, Research Triangle Park, North Carolina, USA.
(37)Departments of Obstetrics and Gynecology, University of Utah Health Sciences Center, Salt Lake, Utah, USA.
(38)Division of Maternal-Fetal Medicine, Department of Obstetrics and Gynecology, Northwestern University Feinberg School of Medicine, Chicago, Illinois, USA.
(39)Department of Obstetrics and Gynecology, The University of British Columbia, Vancouver, British Columbia, Canada.
(40)Department of Obstetrics and Gynecology, The Chinese University of Hong Kong, Hong Kong, Hong Kong.
(41)Department of Epidemiology, University of Pittsburgh Graduate School of Public Health, Pittsburgh, Pennsylvania, USA.
(42)Kisumu County Department of Health, Kisumu, Kenya.
(43)Institute of Primary Health Care (BIHAM), University of Bern, Bern, Switzerland.
(44)Pediatric Growth and Nutrition Branch, National Institute of Health, Bethesda, Maryland, USA.
(45)Universidad Pontificia Bolivariana, Medellin, Antioquia, Colombia.
(46)Centre of Excellence in Women and Child Health, Aga Khan University, Nairobi, Kenya.
(47)Department of Obstetrics and Gynecology, St Luke's University Health Network, Bethlehem, Pennsylvania, USA.
(48)Centre for Trials Research, Cardiff University, Cardiff, UK.
(49)Children with Special Medical Needs Division, Puerto Rico Department of Health, San Juan, Puerto Rico.
(50)Department of Women and Children's Health, King's College London Faculty of Life Sciences and Medicine, London, UK.
(51)Department of Maternal-Fetal Medicine, The Royal Women's Hospital, University of Melbourne, Parkville, Victoria, Australia.
(52)Department of Obstetrics and Gynecology, Sancaktepe Sehit Prof Dr Ilhan Varank Training and Research Hospital, Istanbul, Turkey.

INTRODUCTION: Despite a growing body of research on the risks of SARS-CoV-2 infection during pregnancy, there is continued controversy given heterogeneity in the quality and design of published studies.
METHODS: We screened ongoing studies in our sequential, prospective meta-analysis. We pooled individual participant data to estimate the absolute and relative risk (RR) of adverse outcomes among pregnant women with SARS-CoV-2 infection, compared with confirmed negative pregnancies. We evaluated the risk of bias using a modified Newcastle-Ottawa Scale.
RESULTS: We screened 137 studies and included 12 studies in 12 countries

involving 13 136 pregnant women.Pregnant women with SARS-CoV-2 infection-as compared with uninfected pregnant women-were at significantly increased risk of maternal mortality (10 studies; n=1490; RR 7.68, 95% CI 1.70 to 34.61); admission to intensive care unit (8 studies; n=6660; RR 3.81, 95% CI 2.03 to 7.17); receiving mechanical ventilation (7 studies; n=4887; RR 15.23, 95% CI 4.32 to 53.71); receiving any critical care (7 studies; n=4735; RR 5.48, 95% CI 2.57 to 11.72); and being diagnosed with pneumonia (6 studies; n=4573; RR 23.46, 95% CI 3.03 to 181.39) and thromboembolic disease (8 studies; n=5146; RR 5.50, 95% CI 1.12 to 27.12).Neonates born to women with SARS-CoV-2 infection were more likely to be admitted to a neonatal care unit after birth (7 studies; n=7637; RR 1.86, 95% CI 1.12 to 3.08); be born preterm (7 studies; n=6233; RR 1.71, 95% CI 1.28 to 2.29) or moderately preterm (7 studies; n=6071; RR 2.92, 95% CI 1.88 to 4.54); and to be born low birth weight (12 studies; n=11 930; RR 1.19, 95% CI 1.02 to 1.40). Infection was not linked to stillbirth. Studies were generally at low or moderate risk of bias.
CONCLUSIONS: This analysis indicates that SARS-CoV-2 infection at any time during pregnancy increases the risk of maternal death, severe maternal morbidities and neonatal morbidity, but not stillbirth or intrauterine growth restriction. As more data become available, we will update these findings per the published protocol.

© Author(s) (or their employer(s)) 2023. Re-use permitted under CC BY. Published by BMJ.

DOI: 10.1136/bmjgh-2022-009495
PMCID: PMC9895919
PMID: 36646475 [Indexed for MEDLINE]

Conflict of interest statement: Competing interests: CW declares a relationship with Ferring Pharmaceuticals COVID-19 Investigational Grant and NHMRC Fellowship (salary support). AP declares the following research grants to her institution: ‘H2020-Grant—Consortium member of Innovative medicine initiative call 13 topic 9 «ConcePTION», Efficacy and safety studies on Medicines EMA/2017/09/PE/11, Lot 4, WP 2 lead, Safety monitoring of COVID-19 vaccines in the EU—Reopening of competition no. 20 under a framework contract following procurement procedure EMA/2017/09/PE (Lot 3) (Euro 110,000), Federal Office of Public Health (207,000 CHF)’. EM declares a relationship with the National Institute for Health Research (project grant for PAN COVID study). DM declares a relationship with the Canadian Institutes of Health Research (payments to institution only), Public Health Agency of Canada (payments to institution only), BC Women’s Foundation (payments to institution only) and is a member of the COVID-19 Immunity Task Force sponsored by the Canadian government. TDM declares a relationship with Pfizer (site principal investigator for SARS-CoV-2 vaccination in pregnancy study, money paid to institution and member of Medical Advisory Board for SARS-CoV-2 vaccination in pregnancy study, money paid to TDM), NICHD (subcommittee chair for the NICHD Maternal-Fetal Medicine Units Network Gestational Research Assessments of COVID-19 (GRAVID) study) and Society for

Maternal-Fetal Medicine (board member). EL declares a relationship with the US NIH (paid institution) and is an employee of AbbVie, but was employed at the University of Washington at the time of the study. KK declares a relationship with the Bill & Melinda Gates Foundation. VJF declares a relationship with the Bill & Melinda Gates Foundation (payments to institution), Yellow Chair Foundation (payments to institution), Robert Woods Johnson Foundation (payments to institution), CDC Foundation, California Health Care Foundation (payments to institution), Tara Health Foundation (payments to institution), UCSF Women's Health Center of Excellence (payments to institution) and California Department of Health Care Services (payments made to institution). JS-B declares a relationship with the Ferring Pharmaceuticals, which gave a grant ($10 000) for the expenses of RECOGEST trial and is a part of the Columbian Federation of Perinatology. YA declares a relationship with the Bill & Melinda Gates Foundation (payments made to institution), CDC Foundation (payments made to institution), Robert Woods Johnson Foundation (payments made to institution) and UCLA Dean's Office COVID-19 research (payments made to institution). RC declares a relationship with the NIH HD36801 (MFMU Network DCC). MCN declares a relationship with the BMGF (project grant made to institution), EDCTP, Sanofi, AstraZeneca, Pfizer (research grants made to institution), Sanofi Pasteur (payment or honoraria for lectures, presentations, speakers bureaus, manuscript writing or educational events) and Sanofi Pasteur and Pfizer (payment for expert testimony). ESM declares a relationship with Pfizer (site principal investigator for phase 2/3 RCT of COVID vaccine during pregnancy). OS declares a relationship with the NordForsk Funding (Nordic research funding grant number: 105545), the Swedish Medical Products Agency (funding for reports on COVID-19 vaccines and pregnancy) and Karolinska Institutet (funding for COVID research and pregnancy: 2020-01567). EG declares a relationship with the Stavros Niarchos Foundation, Santander Foundation and 'La Caixa' Foundation (payments made to institution). SAM declares a relationship with BMGF (funded study in South Africa).

241. Sci Prog. 2023 Jan-Mar;106(1):368504221148933. doi: 10.1177/00368504221148933.

Factors associated with mortality in patients with tetanus in Cameroon.

Sangwe Clovis N(1), Palle JN(1), Linwa EMM(1), Ndung Ako F(1), Tabe Benem-Orock V(1), Chichom Mefire A(1).

Author information:
(1)The Faculty of Health Sciences, 107749University of Buea, Buea, Cameroon.

BACKGROUND: Tetanus constitutes a significant cause of death in sub-Saharan Africa, with case fatality rates as high as 64%, 47%, and 43.1% reported in Nigeria, Uganda, and Tanzania, respectively. However, the knowledge gap on factors that influence tetanus mortality still exists in Cameroon.
OBJECTIVE: To identify the factors that influence the mortality of tetanus patients in three regional hospitals in the North West and South West Regions of

Cameroon.
METHODOLOGY: This was a 7-year retrospective case-series study. Socio-demographic and clinical data of tetanus cases admitted in Bamenda, Buea, and Limbe Regional Hospitals from January 2010 to March 2017 were collected and analysed.
RESULTS: This study recorded 38 cases, but excluded five due to missing inpatient files. Of the 33 cases considered in this study, 27 (81.8%) were males. The mean age of the patients was 32 years. A tetanus mortality rate of 48.5% was recorded - thus, 16 tetanus-related deaths. Mortality was higher amongst patients with incubation periods less than 7 days; the onset of trismus and generalized spasms less than 8 days after exposure; the presence of autonomic dysfunction; the presence of respiratory dysfunction; application or introduction of local regimens, soil or animal waste at the portal of entry; delayed debridement greater than 2 days after onset of tetanus; and a severe clinical disease state.
CONCLUSION: This study, therefore, highlights the need to ensure universal vaccination coverage for high-risk populations; males, active population (20-40 years), and farmers. More still, this study emphasizes the need to improve access and utilisation of mechanical ventilation in severe tetanus cases.

DOI: 10.1177/00368504221148933
PMCID: PMC10370253
PMID: 36617884 [Indexed for MEDLINE]

Conflict of interest statement: The author(s) declared no potential conflicts of interest with respect to the research, authorship, and/or publication of this article.

242. Emerg Infect Dis. 2023 Jan;29(1):224-226. doi: 10.3201/eid2901.220981. Epub 2022 Dec 18.

SARS-CoV-2 Omicron BA.5 Infections in Vaccinated Persons, Rural Uganda.

Mugisha J, Mpairwe B, Newton R, Cotten M, Phan MVT.

We describe a cluster of COVID-19 breakthrough infections after vaccination in Kyamulibwa, Kalungu District, Uganda. All but 1 infection were from SARS-CoV-2 Omicron strain BA.5.2.1. We identified 6 distinct genotypes by genome sequencing. Infections were mild, suggesting vaccination is not protective against infection but may limit disease severity.

DOI: 10.3201/eid2901.220981
PMCID: PMC9796194
PMID: 36529453 [Indexed for MEDLINE]

243. Lancet Glob Health. 2023 Jan;11(1):e48-e58. doi: 10.1016/S2214-109X(22)00475-2.

Potential benefit of extended dose schedules of human papillomavirus vaccination in the context of scarce resources and COVID-19 disruptions in low-income and middle-income countries: a mathematical modelling analysis.

Bénard É(1), Drolet M(2), Laprise JF(2), Jit M(3), Prem K(4), Boily MC(5), Brisson M(6).

Author information:
(1)Département de médecine sociale et préventive, Université Laval, QC, Canada; Centre de recherche du CHU de Québec, Université Laval, QC, Canada.
(2)Centre de recherche du CHU de Québec, Université Laval, QC, Canada.
(3)Centre for Mathematical Modelling of Infectious Disease, London School of Hygiene and Tropical Medicine, London, UK; School of Public Health, University of Hong Kong, Hong Kong Special Administrative Region, China.
(4)Centre for Mathematical Modelling of Infectious Disease, London School of Hygiene and Tropical Medicine, London, UK; Saw Swee Hock School of Public Health, National University of Singapore, Singapore.
(5)Département de médecine sociale et préventive, Université Laval, QC, Canada; Centre de recherche du CHU de Québec, Université Laval, QC, Canada; MRC Centre for Global Infectious Disease Analysis, School of Public Health, Imperial College London, London, UK.
(6)Département de médecine sociale et préventive, Université Laval, QC, Canada; Centre de recherche du CHU de Québec, Université Laval, QC, Canada; MRC Centre for Global Infectious Disease Analysis, School of Public Health, Imperial College London, London, UK. Electronic address: marc.brisson@crchudequebec.ulaval.ca.

Comment in
Lancet Glob Health. 2023 Jan;11(1):e4-e5.

BACKGROUND: The WHO Strategic Advisory Group of Experts recommended that an extended interval of 3-5 years between the two doses of the human papillomavirus (HPV) vaccine could be considered to alleviate vaccine supply shortages. However, three concerns have limited the introduction of extended schedules: girls could be infected between the two doses, the vaccination coverage for the second dose could be lower at ages 13-14 years than at ages 9-10 years, and identifying girls vaccinated with a first dose to give them the second dose could be difficult. Using mathematical modelling, we examined the potential effect of these concerns on the population-level impact and efficiency of extended dose HPV vaccination schedules.
METHODS: We used HPV-ADVISE, an individual-based, transmission-dynamic model of multitype HPV infection and disease, calibrated to country-specific data for four low-income and middle-income countries (India, Viet Nam, Uganda, and Nigeria). For the extended dose scenarios, we varied the vaccination coverage of the second dose among girls previously vaccinated, the one-dose vaccine

efficacy, and the one-dose vaccine duration of protection. We also examined a strategy in which girls aged 14 years were vaccinated irrespective of their previous vaccination status. We used a scenario of girls-only two-dose vaccination at age 9 years (vaccine=9 valent, vaccine-type efficacy=100%, duration of protection=lifetime, and coverage=80%) as our comparator. We estimated two outcomes: the relative reduction in the age-standardised cervical cancer incidence (population-level impact) and the number of cervical cancers averted per 100 000 doses (efficiency).
FINDINGS: Our model projected substantial reductions in cervical cancer incidence over 100 years with the two-dose schedule (79-86% depending on the country), compared with no vaccination. Projections for the 5-year extended schedule, in which the second dose is given only to girls previously vaccinated at age 9 years, were similar to the current two-dose schedule, unless vaccination coverage of the second dose is very low (reductions in cervical cancer incidence of 71-78% assuming 30% coverage at age 14 years among girls vaccinated at age 9 years). However, when the dose at age 14 years is given to girls irrespective of vaccination status and assuming high vaccination coverage, the model projected a substantially greater reduction in cervical cancer incidence compared with the current two-dose schedule (reductions in cervical cancer incidence of 86-93% assuming 70% coverage at age 14 years, irrespective of vaccination status). Efficiency of the extended schedule was greater than the two-dose schedule, even with a drop in vaccination coverage.
INTERPRETATION: The three concerns are unlikely to have a substantial effect on the population-level impact of extended dose schedules. Hence, extended dose schedules will likely provide similar cervical cancer reductions as two-dose schedules, while reducing the number of doses required in the short-term, providing a more efficient use of scarce resources, and offering a 5-year time window to reassess the necessity of the second dose.
FUNDING: WHO, Canadian Institute of Health Research Foundation, Fonds de recherche du Québec-Santé, Digital Research Alliance of Canada, and Bill & Melinda Gates Foundation.

Copyright © 2023 The Author(s). Published by Elsevier Ltd. This is an Open Access article under the CC BY 4.0 license. Published by Elsevier Ltd.. All rights reserved.

DOI: 10.1016/S2214-109X(22)00475-2
PMCID: PMC9764452
PMID: 36521952 [Indexed for MEDLINE]

Conflict of interest statement: Declaration of interests MB, MD, and MJ are members of the Single-Dose HPV Vaccine Evaluation Consortium. EB, J-FL, KP, and M-CB declare no competing interests.

244. Lancet Glob Health. 2023 Jan;11(1):e155-e164. doi: 10.1016/S2214-109X(22)00465-X. Epub 2022 Dec 1.

The use of imagery in global health: an analysis of infectious disease documents and a framework to guide practice.

Charani E(1), Shariq S(2), Cardoso Pinto AM(2), Farooqi R(2), Nambatya W(3), Mbamalu O(4), Abimbola S(5), Mendelson M(4).

Author information:
(1)Division of Infectious Diseases and HIV Medicine, Department of Medicine, Groote Schuur Hospital, University of Cape Town, Cape Town, South Africa; National Institute for Health Research Health Protection Research Unit in Healthcare Associated Infection and Antimicrobial Resistance, London, UK; Department of Infection Control and Epidemiology, Amrita Institute of Medical Science, Amrita Vishwa Vidyapeetham, Kochi, India; Centre for Excellence in Infectious Diseases Research, University of Liverpool, Liverpool, UK. Electronic address: esmita.charani@uct.ac.za.
(2)Imperial College London, St Mary's Hospital Medical School, London, UK.
(3)Pharmacy Department, College of Health Sciences Makerere University, Kampala, Uganda.
(4)Division of Infectious Diseases and HIV Medicine, Department of Medicine, Groote Schuur Hospital, University of Cape Town, Cape Town, South Africa.
(5)School of Public Health, University of Sydney, Sydney, NSW, Australia.

Erratum in
Lancet Glob Health. 2023 Jan 12;:

Comment in
Lancet Glob Health. 2023 Apr;11(4):e503.
Lancet Glob Health. 2023 Oct;11(10):e1496-e1498.

We report an empirical analysis of the use of imagery by the key actors in global health who set policy and strategy, and we provide a comprehensive overview, particularly related to images used in reports on vaccination and antimicrobial resistance. The narrative currently depicted in imagery is one of power imbalances, depicting women and children from low-income and middle-income countries (LMICs) with less dignity, respect, and power than those from high-income countries. The absence of any evidence of consent for using intrusive and out-of-context images, particularly of children in LMICs, is concerning. The framework we have developed provides a platform for global health actors to redefine their intentions and recommission appropriate images that are relevant to the topic, respect the integrity of all individuals depicted, are accompanied by evidence of consent, and are equitable in representation. Adhering to these standards will help to avoid inherent biases that lead to insensitive content and misrepresentation, stigmatisation, and racial stereotyping.

Copyright © 2023 The Author(s). Published by Elsevier Ltd. This is an Open

Access article under the CC BY 4.0 license. Published by Elsevier Ltd.. All rights reserved.

DOI: 10.1016/S2214-109X(22)00465-X
PMID: 36463917 [Indexed for MEDLINE]

Conflict of interest statement: Declaration of interests We declare no competing interests.

245. Int J Health Policy Manag. 2023;12:7519. doi: 10.34172/ijhpm.2022.7519. Epub 2022 Sep 14.

COVID-19 Aftermath: Direction Towards Universal Health Coverage in Low-Income Countries Comment on "Health Coverage and Financial Protection in Uganda: A Political Economy Perspective".

Tangcharoensathien V(1), Panichkriangkrai W(1), Witthyapipopsakul W(1), Patcharanarumol W(1).

Author information:
(1)International Health Policy Program, Ministry of Public Health, Nonthaburi, Thailand.

Comment on
Int J Health Policy Manag. 2022 Sep 1;11(9):1894-1904.

Progressive realization of universal health coverage (UHC) requires health systems capacity to provide quality service and financial risk protection which supports access to services without financial hardship. Government health spending in low-income countries (LICs) has been low and heavily relied on external donor resources and out-of-pocket payment. This has resulted in high prevalence of catastrophic health spending or foregone care by those who cannot afford. Under fiscal constraints posed by pandemic, reforms in LICs should focus on efficiency through health resource waste reduction. Targeting the poor even with low level of health spending can make a significant health gain. Investment in primary healthcare and health workforce is the foundation for realizing UHC which cannot be postponed. Innovative tax on health hazardous products, conditional debt relief can increase fiscal space for health; while international collaboration to accelerate coronavirus disease 2019 (COVID-19) vaccine coverage can bring LICs out of acute phase of pandemic.

© 2023 The Author(s); Published by Kerman University of Medical Sciences This is an open-access article distributed under the terms of the Creative Commons Attribution License (http://creativecommons.org/licenses/by/4.0), which permits unrestricted use, distribution, and reproduction in any medium, provided the original work is properly cited.

DOI: 10.34172/ijhpm.2022.7519
PMCID: PMC10125230
PMID: 36243945 [Indexed for MEDLINE]

Conflict of interest statement: Authors declare that they have no competing interests.

246. Vaccines (Basel). 2022 Dec 31;11(1):99. doi: 10.3390/vaccines11010099.

Inspiring Anti-Tick Vaccine Research, Development and Deployment in Tropical Africa for the Control of Cattle Ticks: Review and Insights.

Kasaija PD(1)(2), Contreras M(1), Kirunda H(3), Nanteza A(4), Kabi F(2), Mugerwa S(2), de la Fuente J(1)(5).

Author information:
(1)SaBio, Instituto de Investigación en Recursos Cinegéticos (IREC), Consejo Superior de Investigaciones Científicas (CSIC), Universidad de Castilla-La Mancha (UCLM)-Junta de Comunidades de Castilla-La Mancha (JCCM), Ronda de Toledo s/n, 13005 Ciudad Real, Spain.
(2)National Livestock Resources Research Institute (NaLIRRI), Kampala P.O. Box 5704, Uganda.
(3)National Agricultural Research Organisation (NARO), Mbarara ZARDI, Mbarara P.O. Box 389, Uganda.
(4)College of Veterinary Medicine, Animal Resources and Biosecurity, Makerere University, Kampala P.O. Box 7062, Uganda.
(5)Department of Veterinary Pathobiology, Center for Veterinary Health Sciences, Oklahoma State University, Stillwater, OK 74078, USA.

Ticks are worldwide ectoparasites to humans and animals, and are associated with numerous health and economic effects. Threatening over 80% of the global cattle population, tick and tick-borne diseases (TTBDs) particularly constrain livestock production in the East, Central and Southern Africa. This, therefore, makes their control critical to the sustainability of the animal industry in the region. Since ticks are developing resistance against acaricides, anti-tick vaccines (ATVs) have been proposed as an environmentally friendly control alternative. Whereas they have been used in Latin America and Australia to reduce tick populations, pathogenic infections and number of acaricide treatments, commercially registered ATVs have not been adopted in tropical Africa for tick control. This is majorly due to their limited protection against economically important tick species of Africa and lack of research. Recent advances in various omics technologies and reverse vaccinology have enabled the identification of many candidate anti-tick antigens (ATAs), and are likely to usher in the next generation of vaccines, for which Africa should prepare to embrace. Herein, we highlight some scientific principles and approaches that

have been used to identify ATAs, outline characteristics of a desirable ATA for vaccine design and propose the need for African governments to investment in ATV research to develop vaccines relevant to local tick species (personalized vaccines). We have also discussed the prospect of incorporating anti-tick vaccines into the integrated TTBDs control strategies in the sub-Saharan Africa, citing the case of Uganda.

DOI: 10.3390/vaccines11010099
PMCID: PMC9866923
PMID: 36679944

Conflict of interest statement: The authors declare no conflict of interest.

247. BMC Womens Health. 2022 Dec 28;22(1):555. doi: 10.1186/s12905-022-02144-8.

Social networks, mobility, and HIV risk among women in the fishing communities of Lake Victoria.

Nakamanya S(1), Okello ES(2), Kwena ZA(3), Nanyonjo G(4), Bahemuka UM(5), Kibengo FM(5), Ssetaala A(4), Bukusi EA(3), Kapiga S(2), Fast PE(6)(7), Seeley J(5)(8).

Author information:
(1)Medical Research Council/Uganda Virus Research Institute and London School of Hygiene and Tropical Medicine (MRC/UVRI and LSHTM), Uganda Research Unit, P.O. Box 49, Entebbe, Uganda. Sarah.nakamanya@mrcuganda.org.
(2)Mwanza Intervention Trials Unit (MITU), National Institute for Medical Research, Mwanza, Tanzania.
(3)Research Care and Training Program (RCTP), Centre for Microbiology Research, Kenya Medical Research Institute (KEMRI), Nairobi, Kenya.
(4)UVRI-IAVI HIV Vaccine Program, Entebbe, Uganda.
(5)Medical Research Council/Uganda Virus Research Institute and London School of Hygiene and Tropical Medicine (MRC/UVRI and LSHTM), Uganda Research Unit, P.O. Box 49, Entebbe, Uganda.
(6)International AIDS Vaccine Initiative (IAVI), New York, USA.
(7)Pediatric Infectious Diseases, School of Medicine, Stanford University, Palo Alto, CA, USA.
(8)Department of Global Health and Development, London School of Hygiene and Tropical Medicine, London, UK.

BACKGROUND: Population mobility is a demonstrated barrier to reducing HIV incidence. A clear understanding of social networks and their influence on mobility among women in the fishing communities of Lake Victoria may contribute to tailoring effective interventions that suit the needs of these mobile women.
METHODS: A cross-sectional qualitative methods study was conducted to understand mobility patterns among women resident and or working in fishing communities of

Lake Victoria in Kenya, Tanzania, and Uganda. The study was conducted in six fishing communities from March 2018 to June 2019. The communities were purposively selected, based on population size (1000 people or more) and HIV prevalence of > 15% among women aged 18 years or older who had lived in the fishing community for at least six months. In-depth interviews were conducted with 24 key informants and 72 women from the sites in the three countries. Questions focused on women's social networks and other factors that fuelled or facilitated women's mobility as well as challenges they faced due to mobility. Data analysis followed a thematic framework approach.
RESULTS: Different social groupings/networks existed among women in the fishing communities of Lake Victoria. These included female sex workers, women fish processors/traders, women bar workers/owners, restaurant workers, and family networks. Networks encouraged mobility, supporting finding work opportunities, but also increased sexual risks through partner changes. The benefits of networks included information sharing, financial support, and group protection, especially against violence.
CONCLUSION: Social networks and groupings among women in the fishing communities of Lake Victoria could be useful in tailoring HIV prevention and HIV care interventions to suit the needs of these highly mobile populations.

© 2022. The Author(s).

DOI: 10.1186/s12905-022-02144-8
PMCID: PMC9798550
PMID: 36578062 [Indexed for MEDLINE]

Conflict of interest statement: The authors declare that they have no competing interests.

248. Blood Adv. 2022 Dec 27;6(24):6187-6197. doi: 10.1182/bloodadvances.2022007698.

The Consortium on Newborn Screening in Africa for sickle cell disease: study rationale and methodology.

Green NS(1), Zapfel A(2), Nnodu OE(3), Franklin P(4), Tubman VN(5), Chirande L(6), Kiyaga C(7), Chunda-Liyoka C(8), Awuonda B(9), Ohene-Frempong K(10), Inusa BPD(11), Ware RE(12), Odame I(13), Ambrose EE(14), Dogara LG(15), Oron AP(16), Willett C(2)(17), Thompson AA(18), Berliner N(19), Coetzer TL(20), Novelli EM(21).

Author information:
(1)Department of Pediatrics, Columbia University Irving Medical Center, New York, NY.
(2)American Society of Hematology, Washington, DC.
(3)Department of Hematology and Blood Transfusion, Centre of Excellence for Sickle Cell Disease Research and Training, University of Abuja, Abuja, Nigeria.

(4)Department of Paediatrics, John F. Kennedy Medical Center, Monrovia, Liberia.
(5)Department of Pediatrics, Baylor College of Medicine, Houston, TX.
(6)Department of Pediatrics, Muhimbili National Hospital and Muhimbili University of Health and Allied Sciences, Dar es Salaam, Tanzania.
(7)Central Public Health Laboratories, Ministry of Health, Kampala, Uganda.
(8)Department of Paediatrics, University Teaching Hospitals - Children's Hospital, Lusaka, Zambia.
(9)Department of Pediatrics, Jaramogi Oginga Odinga Teaching and Referral Hospital, Kisumu, Kenya.
(10)Sickle Cell Foundation of Ghana, Accra, Ghana.
(11)Guy's and St. Thomas's Private Healthcare, London, United Kingdom.
(12)Division of Hematology, Cancer and Blood Diseases Institute, Global Health Center, Cincinnati Children's Hospital, Cincinnati, OH.
(13)Division of Haematology and Oncology, SickKids, Toronto, ON, Canada.
(14)Department of Paediatrics and Child Health, Catholic University of Health & Allied Science and Bugando Medical Centre, Mwanza, Tanzania.
(15)Department of Haematology, Kaduna State University, Kaduna, Nigeria.
(16)Department of Statistics, Institute for Health Metrics and Evaluation, University of Washington, Seattle, WA.
(17)Willows Consulting, Seattle, WA.
(18)Division of Hematology, Children's Hospital of Philadelphia, Philadelphia, PA.
(19)Division of Hematology, Brigham and Women's Hospital, Harvard Medical School, Harvard University, Boston, MA.
(20)Department of Molecular Medicine and Haematology, Wits Research Institute for Malaria, University of the Witwatersrand, Johannesburg, South Africa.
(21)Department of Medicine, University of Pittsburgh Medical Center, Pittsburgh, PA.

Sickle cell disease (SCD) is a common condition within sub-Saharan Africa and associated with high under-5 mortality (U5M). The American Society of Hematology instituted the Consortium on Newborn Screening in Africa (CONSA) for SCD, a 7-country network of sites to implement standardized newborn hemoglobinopathy screening and early intervention for children with SCD in sub-Saharan Africa. CONSA's overall hypothesis is that early infant SCD screening and entry into standardized, continuous care will reduce U5M compared with historical estimates in the region. Primary trial objectives are to determine the population-based birth incidence of SCD and effectiveness of early standardized care for preventing early mortality consortium-wide at each country's site(s). Secondary objectives are to establish universal screening and early interventions for SCD within clinical networks of CONSA partners and assess trial implementation. Outcomes will be evaluated from data collected using a shared patient registry. Standardized trial procedures will be implemented among designated birth populations in 7 African countries whose programs met eligibility criteria. Treatment protocol includes administering antibacterial and antimalarial prophylaxis and standard childhood vaccinations against infections commonly affecting children with SCD. Infants with a positive screen and confirmation of

SCD within the catchment areas defined by each consortium partner will be enrolled in the clinical intervention protocol and followed regularly until age of 5 years. Effectiveness of these early interventions, along with culturally appropriate family education and counseling, will be evaluated by comparing U5M in the enrolled cohort to estimated preprogram data. Here, we describe the methodology planned for this trial.

© 2022 by The American Society of Hematology. Licensed under Creative Commons Attribution-NonCommercial-NoDerivatives 4.0 International (CC BY-NC-ND 4.0), permitting only noncommercial, nonderivative use with attribution. All other rights reserved.

DOI: 10.1182/bloodadvances.2022007698
PMCID: PMC9791313
PMID: 36264096 [Indexed for MEDLINE]

Conflict of interest statement: Conflict-of-interest disclosure: V.N.T. has served as a consultant to Novartis and has received honorarium from PerkinElmer. The remaining authors declare no competing financial interests.

249. PLoS One. 2022 Dec 22;17(12):e0279428. doi: 10.1371/journal.pone.0279428. eCollection 2022.

Retrospective in silico mutation profiling of SARS-CoV-2 structural proteins circulating in Uganda by July 2021: Towards refinement of COVID-19 disease vaccines, diagnostics, and therapeutics.

Odongo S(1)(2)(3), Okella H(2)(4), Ndekezi C(5), Okee M(6)(7), Namayanja M(2), Mujuni B(7), Sterckx YGJ(8), Kizito D(5), Radwanska M(3)(9), Magez S(3)(10), Ikwap K(11), Mwiine FN(11), Lutwama JJ(5), Ibingira C(12).

Author information:
(1)Department of Biotechnical and Diagnostic Sciences, College of Veterinary Medicine, Animal Resources and Biosecurity (COVAB), Makerere University, Kampala, Uganda.
(2)Center for Biosecurity and Global Health, College of Veterinary Medicine, Animal Resources and Biosecurity (COVAB), Makerere University, Kampala, Uganda.
(3)Laboratory for Biomedical Research, Ghent University Global Campus, Incheon, South Korea.
(4)Pharm-Biotechnology and Traditional Medicine Center, Mbarara University of Science and Technology, Mbarara, Uganda.
(5)Uganda Virus Research Institute, Entebbe, Uganda.
(6)Department of Medical Microbiology, College of Health Sciences, Makerere University, Kampala, Uganda.
(7)Africa Center of Excellence in Materials, Product Development and Nanotechnology, College of Engineering, Makerere University, Kampala, Uganda.

(8)Laboratory of Medical Biochemistry and the Infla-Med Centre of Excellence, University of Antwerp, Campus Drie Eiken, Universiteitsplein, Wilrijk, Belgium.
(9)Department of Biomedical Molecular Biology, Ghent University, Ghent, Belgium.
(10)Laboratory of Cellular and Molecular Immunology, Vrije Universiteit Brussel, Brussels, Belgium.
(11)Department of Biomolecular Resources, College of Veterinary Medicine, Animal Resources and Biosecurity, Makerere University, Kampala, Uganda.
(12)Department of Anatomy, School of Biomedical Sciences, College of Health Sciences, Makerere University, Kampala, Uganda.

The SARS-CoV-2 virus, the agent of COVID-19, caused unprecedented loss of lives and economic decline worldwide. Although the introduction of public health measures, vaccines, diagnostics, and therapeutics disrupted the spread of the SARS-CoV-2, the emergence of variants poses substantial threat. This study traced SARS-CoV-2 variants circulating in Uganda by July 2021 to inform the necessity for refinement of the intervention medical products. A comprehensive in silico analysis of the SARS-CoV-2 genomes detected in clinical samples collected from COVID-19 patients in Uganda revealed occurrence of structural protein variants with potential of escaping detection, resisting antibody therapy, or increased infectivity. The genome sequence dataset was retrieved from the GISAID database and the open reading frame encoding the spike, envelope, membrane, or nucleocapsid proteins was translated. The obtained protein sequences were aligned and inspected for existence of variants. The variant positions on each of the four alignment sets were mapped on predicted epitopes as well as the 3D structures. Additionally, sequences within each of the sets were clustered by family. A phylogenetic tree was constructed to assess relationship between the encountered spike protein sequences and Wuhan-Hu-1 wild-type, or the Alpha, Beta, Delta and Gamma variants of concern. Strikingly, the frequency of each of the spike protein point mutations F157L/Del, D614G and P681H/R was over 50%. The furin and the transmembrane serine protease 2 cleavage sites were unaffected by mutation. Whereas the Delta dominated the spike sequences (16.5%, 91/550), Gamma was not detected. The envelope protein was the most conserved with 96.3% (525/545) sequences being wild-type followed by membrane at 68.4% (397/580). Although the nucleocapsid protein sequences varied, the variant residue positions were less concentrated at the RNA binding domains. The dominant nucleocapsid sequence variant was S202N (34.5%, 205/595). These findings offer baseline information required for refining the existing COVID-19 vaccines, diagnostics, and therapeutics.

Copyright: © 2022 Odongo et al. This is an open access article distributed under the terms of the Creative Commons Attribution License, which permits unrestricted use, distribution, and reproduction in any medium, provided the original author and source are credited.

DOI: 10.1371/journal.pone.0279428
PMCID: PMC9778641
PMID: 36548384 [Indexed for MEDLINE]

Conflict of interest statement: The authors have declared that no competing interests exist.

250. BMC Public Health. 2022 Dec 20;22(1):2385. doi: 10.1186/s12889-022-14875-2.

Feasibility of conducting HIV prevention trials among key populations in Nairobi, Kenya.

Mutisya EM(1), Muturi-Kioi V(2), Abaasa A(3), Nyasani D(4), Kabuti RW(5), Lunani L(4), Kotikot T(4), Mundia M(4), Mutua G(2), Ombati G(4), Nduta H(4), Price MA(6)(7), Kimani J(5), Anzala AO(4)(8).

Author information:
(1)KAVI-Institute of Clinical Research, University of Nairobi, Nairobi, Kenya. Emutisya@kaviuon.org.
(2)IAVI, Nairobi, Kenya.
(3)MRC/UVRI and LSHTM Uganda Research Unit, Entebbe, Uganda.
(4)KAVI-Institute of Clinical Research, University of Nairobi, Nairobi, Kenya.
(5)SWOP-PHDA, University of Nairobi/University of Manitoba, Nairobi, Kenya.
(6)IAVI, New York, USA.
(7)Department of Epidemiology and Biostatistics, University of California, San Francisco, California, USA.
(8)School of Medicine, College of Health Sciences, University of Nairobi, Nairobi, Kenya.

OBJECTIVE: To assess the feasibility of conducting HIV prevention trials among key populations in Nairobi, Kenya.
BACKGROUND: HIV prevention trials require the inclusion of those at high risk of HIV infection and their informed decision to take part and remain in the clinical trial to the end is crucial. In Kenya key populations including men who have sex with men (MSM) and female sex workers (FSW) are, disproportionately, at high risk of HIV infection when compared to the general population. Few trials testing biomedical prevention products against HIV have enrolled Kenyan FSW and MSM.
METHODS: We performed simulated vaccine efficacy trial (SiVET) using licensed hepatitis B vaccines as substitutes for a HIV vaccine candidate and included randomization for those immune to hep B. The SiVET was an observational study designed to mimic the rigors of a clinical trial; we assessed HIV risk, provided risk counselling and prevention tools and performed HIV testing at baseline and periodically until the end of the trial. MSM and FSW were enrolled at a ratio of 4:1. Volunteers were assigned to either hepatitis B vaccine or placebo.
RESULTS: Recruitment took approximately 24 months between Sep 2015 and Sep 2017. Of the 368 volunteers screened, 250 (200 MSM and 50 FSW) were enrolled. Reasons for exclusion at screening included: being positive for HIV (n = 7), hepatitis (n = 14), other pre-existing medical conditions (n = 41), eligible but chose not

to enrol (n = 47). Most of the volunteers adhered to study procedures and attended their study visits within the study window. These include volunteers who received the second vaccination 244 (98%), the third vaccination 228 (91%) and, the final study visit 217 (87%). The reasons volunteers discontinued from the study early included: relocation and loss to follow up (n = 14). A total of 8 cases of HIV infection were observed in 174.5 Person Years at Risk (PYAR), all among MSM, including 5 seroconversions identified at the last study visit, for a HIV incidence of 4.58 cases/ 100 PYAR, among MSM enrolled in the study.
CONCLUSION: Our findings suggest that it is possible to conduct HIV prevention trials among key populations in Nairobi with a good adherence to a vaccine efficacy trial schedule. Despite HIV prevention efforts, we also noted a high incidence of HIV infection. This demonstrates the need for effective HIV prevention products in these populations.

© 2022. The Author(s).

DOI: 10.1186/s12889-022-14875-2
PMCID: PMC9762985
PMID: 36536335 [Indexed for MEDLINE]

Conflict of interest statement: The Authors declare that they have no competing interests.

251. BMC Med. 2022 Dec 19;20(1):488. doi: 10.1186/s12916-022-02639-z.

Mapping age- and sex-specific HIV prevalence in adults in sub-Saharan Africa, 2000-2018.

Haeuser E(1), Serfes AL(2), Cork MA(2), Yang M(2), Abbastabar H(3), Abhilash ES(4), Adabi M(5), Adebayo OM(6), Adekanmbi V(7), Adeyinka DA(8)(9), Afzal S(10)(11), Ahinkorah BO(12), Ahmadi K(13), Ahmed MB(14)(15), Akalu Y(16), Akinyemi RO(17)(18), Akunna CJ(19)(20), Alahdab F(21), Alanezi FM(22), Alanzi TM(23), Alene KA(24)(25), Alhassan RK(26), Alipour V(27)(28), Almasi-Hashiani A(29), Alvis-Guzman N(30)(31), Ameyaw EK(12), Amini S(32), Amugsi DA(33), Ancuceanu R(34), Anvari D(35)(36), Appiah SCY(37)(38), Arabloo J(27), Aremu O(39), Asemahagn MA(40), Jafarabadi MA(41)(42), Awedew AF(43), Quintanilla BPA(44)(45), Ayanore MA(46)(47), Aynalem YA(48), Azari S(49), Azene ZN(50), Darshan BB(51), Babalola TK(52)(53), Baig AA(54), Banach M(55)(56), Bärnighausen TW(57)(58), Bell AW(59)(60), Bhagavathula AS(61)(62), Bhardwaj N(63), Bhardwaj P(64)(65), Bhattacharyya K(66)(67), Bijani A(68), Bitew ZW(69)(70), Bohlouli S(71), Bolarinwa OA(52), Boloor A(72), Bozicevic I(73)(74), Butt ZA(75)(76), Cárdenas R(77), Carvalho F(78), Charan J(79), Chattu VK(80)(81), Chowdhury MAK(82)(83), Chu DT(84), Cowden RG(85), Dahlawi SMA(86), Damiani G(87)(88), Darteh EKM(89), Darwesh AM(90), das Neves J(91)(92), Weaver ND(2), De Leo D(93), De Neve JW(57), Deribe K(94)(95), Deuba K(96)(97), Dharmaratne S(2)(98)(99), Dianatinasab M(100)(101), Diaz D(102)(103), Didarloo A(104), Djalalinia S(105),

Dorostkar F(106), Dubljanin E(107), Duko B(108)(109), El Tantawi M(110), El-Jaafary SI(111), Eshrati B(112), Eskandarieh S(113), Eyawo O(114), Ezeonwumelu IJ(115)(116), Ezzikouri S(117), Farzadfar F(118), Fattahi N(119), Fauk NK(120)(121), Fernandes E(122), Filip I(123)(124), Fischer F(125), Foigt NA(126), Foroutan M(127)(128), Fukumoto T(129), Gad MM(130)(131), Gaidhane AM(132), Gebregiorgis BG(48), Gebremedhin KB(133), Getacher L(134), Ghadiri K(135)(136), Ghashghaee A(137), Golechha M(138), Gubari MIM(139), Gugnani HC(140)(141), Guimarães RA(142), Haider MR(143), Haj-Mirzaian A(144)(145), Hamidi S(146), Hashi A(147), Hassanipour S(148)(149), Hassankhani H(150)(151), Hayat K(152)(153), Herteliu C(154)(155), Ho HC(156), Holla R(51), Hosseini M(157)(158), Hosseinzadeh M(159)(160), Hwang BF(161), Ibitoye SE(162), Ilesanmi OS(163)(164), Ilic IM(165), Ilic MD(166), Islam RM(167), Iwu CCD(168), Jakovljevic M(169)(170), Jha RP(171)(172), Ji JS(173), Johnson KB(2), Joseph N(174), Joshua V(175), Joukar F(148)(149), Jozwiak JJ(176), Kalankesh LR(177), Kalhor R(178)(179), Kamyari N(180), Kanchan T(181), Matin BK(119), Karimi SE(182), Kayode GA(183)(184), Karyani AK(119), Keramati M(185), Khan EA(186), Khan G(187), Khan MN(188), Khatab K(189)(190), Khubchandani J(191), Kim YJ(192), Kisa A(193)(194), Kisa S(195), Kopec JA(196)(197), Kosen S(198), Laxminarayana SLK(199), Koyanagi A(200)(201), Krishan K(202), Defo BK(203)(204), Kugbey N(205), Kulkarni V(174), Kumar M(206)(207), Kumar N(174), Kusuma D(208)(209), La Vecchia C(210), Lal DK(211), Landires I(212)(213), Larson HJ(2)(214), Lasrado S(215), Lee PH(216), Li S(217), Liu X(218)(219), Maleki A(220)(221), Malik P(222)(223), Mansournia MA(157), Martins-Melo FR(224), Mendoza W(225), Menezes RG(226), Mengesha EW(227), Meretoja TJ(228)(229), Mestrovic T(230)(231), Mirica A(154), Moazen B(57)(232), Mohamad O(233), Mohammad Y(234), Mohammadian-Hafshejani A(235), Mohammadpourhodki R(236), Mohammed S(237)(238), Mohammed S(239)(240), Mokdad AH(2)(99), Moradi M(119), Moraga P(241), Mubarik S(242), Mulu GBB(243), Mwanri L(244), Nagarajan AJ(245)(246), Naimzada MD(247)(248), Naveed M(249), Nazari J(250), Ndejjo R(251), Negoi I(252)(253), Ngalesoni FN(254), Nguefack-Tsague G(255), Ngunjiri JW(256), Nguyen CT(257), Nguyen HLT(257), Nnaji CA(258)(259), Noubiap JJ(260), Nuñez-Samudio V(261)(262), Nwatah VE(263)(264), Oancea B(265), Odukoya OO(266)(267), Olagunju AT(268)(269), Olakunde BO(270), Olusanya BO(271), Olusanya JO(271), Bali AO(272), Onwujekwe OE(273), Orisakwe OE(274), Otstavnov N(247), Otstavnov SS(247)(275), Owolabi MO(276)(277), Mahesh PA(278), Padubidri JR(279), Pana A(154)(280), Pandey A(281)(282), Pandi-Perumal SR(283), Kan FP(284), Patton GC(285)(286), Pawar S(287), Peprah EK(288), Postma MJ(289)(290), Preotescu L(291)(292), Syed ZQ(132), Rabiee N(293)(294), Radfar A(295), Rafiei A(296)(297), Rahim F(298), Rahimi-Movaghar V(299), Rahmani AM(300), Ramezanzadeh K(144), Rana J(301)(302), Ranabhat CL(303)(304), Rao SJ(305), Rawaf DL(306)(307), Rawaf S(308)(309), Rawassizadeh R(310), Regassa LD(311), Rezaei N(312)(313), Rezapour A(27), Riaz MA(314), Ribeiro AI(315), Ross JM(2)(316)(317), Rubagotti E(318)(319), Rumisha SF(320)(321), Rwegerera GM(322), Moghaddam SS(118), Sagar R(323), Sahiledengle B(324), Sahu M(2), Salem MR(325), Kafil HS(326), Samy AM(327), Sartorius B(99)(328)(329), Sathian B(330)(331), Seidu AA(89)(332), Shaheen AA(333), Shaikh MA(334), Shamsizadeh M(335), Shiferaw WS(48), Shin JI(336), Shrestha R(337), Singh JA(338)(339), Skryabin VY(340), Skryabina AA(341), Soltani S(119), Sufiyan

MB(342), Tabuchi T(343), Tadesse EG(344), Taveira N(345)(346), Tesfay
FH(347)(348), Thapar R(174), Tovani-Palone MR(349)(350), Tsegaye GW(351),
Umeokonkwo CD(352), Unnikrishnan B(353), Villafañe JH(354), Violante
FS(355)(356), Vo B(357), Vu GT(358), Wado YD(359), Waheed Y(360), Wamai
RG(361)(362), Wang Y(363), Ward P(364), Wickramasinghe ND(365), Wilson K(366),
Yaya S(367)(368), Yip P(369)(370), Yonemoto N(371)(372), Yu C(242), Zastrozhin
MS(373)(374), Zhang Y(375)(376), Zhang ZJ(377), Hay SI(2)(99), Dwyer-Lindgren
L(2)(99); Local Burden of Disease sub-Saharan Africa HIV Prevalence
Collaborators.

Author information:
(1)Institute for Health Metrics and Evaluation, University of Washington,
Seattle, WA, USA. ehaeuser@uw.edu.
(2)Institute for Health Metrics and Evaluation, University of Washington,
Seattle, WA, USA.
(3)Advanced Diagnostic and Interventional Radiology Research Center, Tehran
University of Medical Sciences, Tehran, Iran.
(4)Department of Botany, Sree Narayana Guru College Chelannur, Kozhikode, India.
(5)Hamadan University of Medical Sciences, Hamadan, Iran.
(6)College of Medicine, University College Hospital, Ibadan, Ibadan, Nigeria.
(7)Department of Population Medicine, Cardiff University, Cardiff, UK.
(8)Department of Community Health and Epidemiology, University of Saskatchewan,
Saskatoon, SK, Canada.
(9)Department of Public Health, Federal Ministry of Health, Abuja, Nigeria.
(10)Department of Community Medicine, King Edward Memorial Hospital, Lahore,
Pakistan.
(11)Department of Public Health, Public Health Institute, Lahore, Pakistan.
(12)The Australian Centre for Public and Population Health Research (ACPPHR),
University of Technology Sydney, Sydney, NSW, Australia.
(13)School of Public Health, Imperial College London, London, UK.
(14)Department of Epidemiology, Jimma University, Jimma, Ethiopia.
(15)Australian Center for Precision Health, University of South Australia,
Adelaide, SA, Australia.
(16)Department of Medical Physiology, University of Gondar, Gondar, Ethiopia.
(17)Institute for Advanced Medical Research and Training, University of Ibadan,
Ibadan, Nigeria.
(18)Institute of Neuroscience, Newcastle University, Newcastle upon Tyne, UK.
(19)Department of Public Health, The Intercountry Centre for Oral Health (ICOH)
for Africa, Jos, Nigeria.
(20)Department of Public Health, Federal Ministry of Health, Garki, Nigeria.
(21)Mayo Evidence-based Practice Center, Mayo Clinic Foundation for Medical
Education and Research, Rochester, MN, USA.
(22)Imam Abdulrahman Bin Faisal University, Dammam, Saudi Arabia.
(23)Health Information Management and Technology Department, Imam Abdulrahman
Bin Faisal University, Dammam, Saudi Arabia.
(24)Faculty of Health Sciences, Curtin University, Perth, WA, Australia.
(25)Wesfarmers Centre of Vaccines and Infectious Diseases, Telethon Kids

Institute, Perth, WA, Australia.
(26)Institute of Health Research, University of Health and Allied Sciences, Ho, Ghana.
(27)Health Management and Economics Research Center, Iran University of Medical Sciences, Tehran, Iran.
(28)Department of Health Economics, Iran University of Medical Sciences, Tehran, Iran.
(29)Department of Epidemiology, Arak University of Medical Sciences, Arak, Iran.
(30)Research Group in Hospital Management and Health Policies, Universidad de la Costa (University of the Coast), Barranquilla, Colombia.
(31)Research Group in Health Economics, University of Cartagena, Cartagena, Colombia.
(32)Department of Health Services Management, Khomein University of Medical Sciences, Khomein, Iran.
(33)Department of Maternal and Child Wellbeing, African Population and Health Research Center, Nairobi, Kenya.
(34)Pharmacy Department, Carol Davila University of Medicine and Pharmacy, Bucharest, Romania.
(35)Department of Parasitology, Mazandaran University of Medical Sciences, Sari, Iran.
(36)Department of Parasitology, Iranshahr University of Medical Sciences, Iranshahr, Iran.
(37)Department of Sociology and Social Work, Kwame Nkrumah University of Science and Technology, Kumasi, Ghana.
(38)Center for International Health, Ludwig Maximilians University, Munich, Germany.
(39)Department of Public Health, Birmingham City University, Birmingham, UK.
(40)School of Public Health, Bahir Dar University, Bahir Dar, Ethiopia.
(41)Department of Biostatistics and Epidemiology, Tabriz University of Medical Sciences, Tabriz, Iran.
(42)Department of Biostatistics and Epidemiology, Zanjan University of Medical Sciences, Zanjan, Iran.
(43)Department of Surgery, Addis Ababa University, Addis Ababa, Ethiopia.
(44)The Judith Lumley Centre, La Trobe University, Melbourne, VIC, Australia.
(45)San Martin de Porres University, Lima, Peru.
(46)Department of Health Policy Planning and Management, University of Health and Allied Sciences, Ho, Ghana.
(47)Department of Health Economics, Centre for Health Policy Advocacy Innovation & Research in Africa (CHPAIR-Africa), Accra, Ghana.
(48)Department of Nursing, Debre Berhan University, Debre Berhan, Ethiopia.
(49)Hospital Management Research Center, Iran University of Medical Sciences, Tehran, Iran.
(50)Department of Reproductive Health, University of Gondar, Gondar, Ethiopia.
(51)Kasturba Medical College, Mangalore, Manipal Academy of Higher Education, Manipal, India.
(52)Discipline of Public Health Medicine, University of KwaZulu-Natal, Durban, South Africa.

(53)Department of Community Health and Primary Care, University of Lagos, Lagos, Nigeria.
(54)Unit of Biochemistry, Universiti Sultan Zainal Abidin (Sultan Zainal Abidin University), Kuala Terengganu, Malaysia.
(55)Department of Hypertension, Medical University of Lodz, Lodz, Poland.
(56)Polish Mothers' Memorial Hospital Research Institute, Lodz, Poland.
(57)Heidelberg Institute of Global Health (HIGH), Heidelberg University, Heidelberg, Germany.
(58)T.H. Chan School of Public Health, Harvard University, Boston, MA, USA.
(59)Department of Global Health and Social Medicine, Harvard University, Boston, MA, USA.
(60)Department of Social Services, Tufts Medical Center, Boston, MA, USA.
(61)Department of Social and Clinical Pharmacy, Charles University, Hradec Kralova, Czech Republic.
(62)Institute of Public Health, United Arab Emirates University, Al Ain, United Arab Emirates.
(63)Department of Anatomy, All India Institute of Medical Sciences, Jodhpur, India.
(64)Department of Community Medicine and Family Medicine, All India Institute of Medical Sciences, Jodhpur, India.
(65)School of Public Health, All India Institute of Medical Sciences, Jodhpur, India.
(66)Department of Statistical and Computational Genomics, National Institute of Biomedical Genomics, Kalyani, India.
(67)Department of Statistics, University of Calcutta, Kolkata, India.
(68)Social Determinants of Health Research Center, Babol University of Medical Sciences, Babol, Iran.
(69)Nutrition Department, St. Paul's Hospital Millennium Medical College, Addis Ababa, Ethiopia.
(70)St. Paul's Hospital Millennium Medical College, Addis Ababa, Ethiopia.
(71)Department of Veterinary Medicine, Islamic Azad University, Kermanshah, Iran.
(72)Department of Internal Medicine, Manipal Academy of Higher Education, Mangalore, India.
(73)WHO Collaborating Centre for HIV Strategic Information, University of Zagreb, Zagreb, Croatia.
(74)Faculty of Public Health and Policy, London School of Hygiene & Tropical Medicine, London, UK.
(75)School of Public Health and Health Systems, University of Waterloo, Waterloo, ON, Canada.
(76)Al Shifa School of Public Health, Al Shifa Trust Eye Hospital, Rawalpindi, Pakistan.
(77)Department of Health Care, Metropolitan Autonomous University, Mexico City, Mexico.
(78)Research Unit on Applied Molecular Biosciences (UCIBIO), University of Porto, Porto, Portugal.
(79)Department of Pharmacology, All India Institute of Medical Sciences,

Jodhpur, India.
(80)Department of Community Medicine, Datta Meghe Institute of Medical Sciences, Sawangi, India.
(81)Saveetha Medical College, Saveetha University, Chennai, India.
(82)James P Grant School of Public Health, BRAC University, Dhaka, Bangladesh.
(83)Department of Epidemiology and Biostatistics, University of South Carolina, Columbia, SC, USA.
(84)Center for Biomedicine and Community Health, VNU-International School, Hanoi, Vietnam.
(85)Department of Psychology, University of the Free State, Park West, South Africa.
(86)Environmental Health Department, Imam Abdulrahman Bin Faisal University, Dammam, Saudi Arabia.
(87)IRCCS Istituto Ortopedico Galeazzi (Galeazzi Orthopedic Institute IRCCS), University of Milan, Milan, Italy.
(88)Department of Dermatology, Case Western Reserve University, Cleveland, OH, USA.
(89)Department of Population and Health, University of Cape Coast, Cape Coast, Ghana.
(90)Department of Information Technology, University of Human Development, Sulaymaniyah, Iraq.
(91)Institute for Research and Innovation in Health, University of Porto, Porto, Portugal.
(92)Institute of Biomedical Engineering (INEB), University of Porto, Porto, Portugal.
(93)Australian Institute for Suicide Research and Prevention, Griffith University, Mount Gravatt, QLD, Australia.
(94)Wellcome Trust Brighton and Sussex Centre for Global Health Research, Brighton and Sussex Medical School, Brighton, UK.
(95)School of Public Health, Addis Ababa University, Addis Ababa, Ethiopia.
(96)National Centre for AIDS and STD Control, Save the Children, Kathmandu, Nepal.
(97)Department of Global Public Health, Karolinska Institute, Stockholm, Sweden.
(98)Department of Community Medicine, University of Peradeniya, Peradeniya, Sri Lanka.
(99)Department of Health Metrics Sciences, School of Medicine, University of Washington, Seattle, WA, USA.
(100)Department of Epidemiology, Maastricht University, Maastricht, Netherlands.
(101)Department of Epidemiology, Shiraz University of Medical Sciences, Shiraz, Iran.
(102)Center of Complexity Sciences, National Autonomous University of Mexico, Mexico City, Mexico.
(103)Faculty of Veterinary Medicine and Zootechnics, Autonomous University of Sinaloa, Rosales, Culiacán, Mexico.
(104)Department of Community Medicine and Public Health, Urmia University of Medical Science, Urmia, Iran.
(105)Development of Research and Technology Center, Ministry of Health and

Medical Education, Tehran, Iran.
(106)Department of Medical Laboratory Sciences, Iran University of Medical Sciences, Tehran, Iran.
(107)Institute of Microbiology and Immunology, University of Belgrade, Belgrade, Serbia.
(108)School of Public Health, Hawassa University, Hawassa, Ethiopia.
(109)School of Public Health, Curtin University, Perth, WA, Australia.
(110)Pediatric Dentistry and Dental Public Health Department, Alexandria University, Alexandria, Egypt.
(111)Department of Neurology, Cairo University, Cairo, Egypt.
(112)Preventive Medicine and Public Health Research Center, Iran University of Medical Sciences, Tehran, Iran.
(113)Multiple Sclerosis Research Center, Tehran University of Medical Sciences, Tehran, Iran.
(114)Faculty of Health, York University, Toronto, BC, Canada.
(115)Institute for Health Science Research Germans Trias i Pujol, Autonomous University of Barcelona, Badalona, Spain.
(116)IrsiCaixa AIDS Research Institute, Badalona, Spain.
(117)Department of Virology, Pasteur Institute of Morocco, Casablanca, Morocco.
(118)Non-communicable Diseases Research Center, Tehran University of Medical Sciences, Tehran, Iran.
(119)Research Center for Environmental Determinants of Health, Kermanshah University of Medical Sciences, Kermanshah, Iran.
(120)Torrens University Australia, Adelaide, SA, Australia.
(121)Institute of Resource Governance and Social Change, Kupang, Indonesia.
(122)Associated Laboratory for Green Chemistry (LAQV), University of Porto, Porto, Portugal.
(123)Psychiatry Department, Kaiser Permanente, Fontana, CA, USA.
(124)School of Health Sciences, A.T. Still University, Mesa, AZ, USA.
(125)Institute of Public Health, Charité Universitätsmedizin Berlin (Charité Medical University Berlin), Berlin, Germany.
(126)Institute of Gerontology, National Academy of Medical Sciences of Ukraine, Kyiv, Ukraine.
(127)Department of Medical Parasitology, Abadan University of Medical Sciences, Abadan, Iran.
(128)Faculty of Medicine, Abadan University of Medical Sciences, Abadan, Iran.
(129)Department of Dermatology, Kobe University, Kobe, Japan.
(130)Department of Cardiovascular Medicine, Cleveland Clinic, Cleveland, OH, USA.
(131)Gillings School of Global Public Health, University of North Carolina Chapel Hill, Chapel Hill, NC, USA.
(132)Department of Community Medicine, Datta Meghe Institute of Medical Sciences, Wardha, India.
(133)Department of Nursing and Midwifery, Addis Ababa University, Addis Ababa, Ethiopia.
(134)Department of Public Health, Debre Berhan University, Debre Berhan, Ethiopia.

(135)Infectious Disease Research Center, Kermanshah University of Medical Sciences, Kermanshah, Iran.
(136)Pediatric Department, Kermanshah University of Medical Sciences, Kermanshah, Iran.
(137)School of Public Health, Qazvin University of Medical Sciences, Qazvin, Iran.
(138)Health Systems and Policy Research, Indian Institute of Public Health, Gandhinagar, India.
(139)Department of Family and Community Medicine, University Of Sulaimani, Sulaimani, Iraq.
(140)Department of Microbiology, Saint James School of Medicine, The Valley, Anguilla.
(141)Department of Epidemiology, Saint James School of Medicine, The Valley, Anguilla.
(142)Faculty of Nursing, Federal University of Goiás, Goiânia, Brazil.
(143)Department of Health Policy and Management, University of Georgia, Athens, GA, USA.
(144)Department of Pharmacology, Shahid Beheshti University of Medical Sciences, Tehran, Iran.
(145)Obesity Research Center, Shahid Beheshti University of Medical Sciences, Tehran, Iran.
(146)School of Health and Environmental Studies, Hamdan Bin Mohammed Smart University, Dubai, United Arab Emirates.
(147)Department of Public Health, Jigjiga University, Jijiga, Ethiopia.
(148)Gastrointestinal and Liver Diseases Research Center, Guilan University of Medical Sciences, Rasht, Iran.
(149)Caspian Digestive Disease Research Center, Guilan University of Medical Sciences, Rasht, Iran.
(150)School of Nursing and Midwifery, Tabriz University of Medical Sciences, Tabriz, Iran.
(151)Independent Consultant, Tabriz, Iran.
(152)Institute of Pharmaceutical Sciences, University of Veterinary and Animal Sciences, Lahore, Pakistan.
(153)Department of Pharmacy Administration and Clinical Pharmacy, Xian Jiaotong University, Xian, China.
(154)Department of Statistics and Econometrics, Bucharest University of Economic Studies, Bucharest, Romania.
(155)School of Business, London South Bank University, London, UK.
(156)Department of Urban Planning and Design, University of Hong Kong, Hong Kong, China.
(157)Department of Epidemiology and Biostatistics, Tehran University of Medical Sciences, Tehran, Iran.
(158)Pediatric Chronic Kidney Disease Research Center, Tehran University of Medical Sciences, Tehran, Iran.
(159)Institute of Research and Development, Duy Tan University, Da Nang, Vietnam.
(160)Department of Computer Science, University of Human Development,

Sulaymaniyah, Iraq.
(161)Department of Occupational Safety and Health, China Medical University, Taichung, Taiwan.
(162)Department of Health Promotion and Education, University of Ibadan, Ibadan, Nigeria.
(163)Department of Community Medicine, University of Ibadan, Ibadan, Nigeria.
(164)Department of Community Medicine, University College Hospital, Ibadan, Ibadan, Nigeria.
(165)Faculty of Medicine, University of Belgrade, Belgrade, Serbia.
(166)Department of Epidemiology, University of Kragujevac, Kragujevac, Serbia.
(167)Department of Epidemiology and Preventive Medicine, Monash University, Melbourne, VIC, Australia.
(168)School of Health Systems and Public Health, University of Pretoria, Pretoria, South Africa.
(169)Institute of Advanced Manufacturing Technologies, Peter the Great St. Petersburg Polytechnic University, St. Petersburg, Russia.
(170)Institute of Comparative Economic Studies, Hosei University, Tokyo, Japan.
(171)Department of Community Medicine, Dr. Baba Saheb Ambedkar Medical College & Hospital, Delhi, India.
(172)Department of Community Medicine, Banaras Hindu University, Varanasi, India.
(173)Vanke School of Public Health, Tsinghua University, Beijing, China.
(174)Department of Community Medicine, Manipal Academy of Higher Education, Mangalore, India.
(175)National Institute of Epidemiology, Indian Council of Medical Research, Chennai, India.
(176)Department of Family Medicine and Public Health, University of Opole, Opole, Poland.
(177)School of Management and Medical Informatics, Tabriz University of Medical Sciences, Tabriz, Iran.
(178)Institute for Prevention of Non-communicable Diseases, Qazvin University of Medical Sciences, Qazvin, Iran.
(179)Health Services Management Department, Qazvin University of Medical Sciences, Qazvin, Iran.
(180)Department of Biostatistics, Abadan University of Medical Sciences, Abadan, Iran.
(181)Department of Forensic Medicine and Toxicology, All India Institute of Medical Sciences, Jodhpur, India.
(182)Social Determinants of Health Research Center, Tabriz University of Medical Sciences, Tabriz, Iran.
(183)International Research Center of Excellence, Institute of Human Virology Nigeria, Abuja, Nigeria.
(184)Julius Centre for Health Sciences and Primary Care, Utrecht University, Utrecht, Netherlands.
(185)Mashhad University of Medical Sciences, Mashhad, Iran.
(186)Department of Epidemiology and Biostatistics, Health Services Academy, Islamabad, Pakistan.

(187)Department of Medical Microbiology & Immunology, United Arab Emirates University, Al Ain, United Arab Emirates.
(188)Department of Population Science, Jatiya Kabi Kazi Nazrul Islam University, Mymensingh, Bangladesh.
(189)Faculty of Health and Wellbeing, Sheffield Hallam University, Sheffield, UK.
(190)College of Arts and Sciences, Ohio University, Zanesville, OH, USA.
(191)Department of Public Health, New Mexico State University, Las Cruces, NM, USA.
(192)School of Traditional Chinese Medicine, Xiamen University Malaysia, Sepang, Malaysia.
(193)School of Health Sciences, Kristiania University College, Oslo, Norway.
(194)Department of Global Community Health and Behavioral Sciences, Tulane University, New Orleans, LA, USA.
(195)Department of Nursing and Health Promotion, Oslo Metropolitan University, Oslo, Norway.
(196)School of Population and Public Health, University of British Columbia, Vancouver, BC, Canada.
(197)Arthritis Research Canada, Richmond, Canada.
(198)Independent Consultant, Jakarta, Indonesia.
(199)Kasturba Medical College, Udupi, India.
(200)Biomedical Research Networking Center for Mental Health Network (CIBERSAM), San Juan de Dios Sanitary Park, Sant Boi de Llobregat, Spain.
(201)Catalan Institution for Research and Advanced Studies (ICREA), Barcelona, Spain.
(202)Department of Anthropology, Panjab University, Chandigarh, India.
(203)Department of Demography, University of Montreal, Montreal, QC, Canada.
(204)Department of Social and Preventive Medicine, University of Montreal, Montreal, Canada.
(205)University of Environment and Sustainable Development, Somanya, Ghana.
(206)Department of Psychiatry, University of Nairobi, Nairobi, Kenya.
(207)Division of Psychology and Language Sciences, University College London, London, UK.
(208)Imperial College Business School, Imperial College London, London, UK.
(209)Faculty of Public Health, University of Indonesia, Depok, Indonesia.
(210)Department of Clinical Sciences and Community Health, University of Milan, Milan, Italy.
(211)Public Health Foundation of India, Gurugram, India.
(212)Unit of Genetics and Public Health, Institute of Medical Sciences, Las Tablas, Panama.
(213)Ministry of Health, Herrera, Panama.
(214)Department of Infectious Disease Epidemiology, London School of Hygiene & Tropical Medicine, London, UK.
(215)Department of Otorhinolaryngology, Father Muller Medical College, Mangalore, India.
(216)Department of Health Sciences, University of Leicester, Leicester, UK.
(217)School of Public Health and Preventive Medicine, Monash University,

Melbourne, VIC, Australia.
(218)Lerner Research Institute, Cleveland Clinic, Cleveland, OH, USA.
(219)Department of Quantitative Health Science, Case Western Reserve University, Cleveland, OH, USA.
(220)Department of Environmental Health Engineering, Tehran University of Medical Sciences, Tehran, Iran.
(221)Environmental Health Research Center, Kurdistan University of Medical Sciences, Sanandaj, Iran.
(222)Department of Pediatrics, Montefiore Medical Center, New York, NY, USA.
(223)Department of Environmental Medicine and Public Health, Icahn School of Medicine at Mount Sinai, New York, NY, USA.
(224)Campus Caucaia, Federal Institute of Education, Science and Technology of Ceará, Caucaia, Brazil.
(225)Peru Country Office, United Nations Population Fund (UNFPA), Lima, Peru.
(226)Forensic Medicine Division, Imam Abdulrahman Bin Faisal University, Dammam, Saudi Arabia.
(227)Department of Reproductive Health and Population Studies, Bahir Dar University, Bahir Dar, Ethiopia.
(228)Breast Surgery Unit, Helsinki University Hospital, Helsinki, Finland.
(229)University of Helsinki, Helsinki, Finland.
(230)Clinical Microbiology and Parasitology Unit, Dr. Zora Profozic Polyclinic, Zagreb, Croatia.
(231)University Centre Varazdin, University North, Varazdin, Croatia.
(232)Institute of Addiction Research (ISFF), Frankfurt University of Applied Sciences, Frankfurt, Germany.
(233)Department of Radiation Oncology, University of California San Francisco, San Francisco, CA, USA.
(234)Internal Medicine Department, King Saud University, Riyadh, Saudi Arabia.
(235)Department of Epidemiology and Biostatistics, Shahrekord University of Medical Sciences, Shahrekord, Iran.
(236)Department of Nursing, Mashhad University of Medical Sciences, Mashhad, Iran.
(237)Department of Biomolecular Sciences, University of Mississippi, Oxford, MS, USA.
(238)Department of Pharmacy, Mizan-Tepi University, Mizan, Ethiopia.
(239)Health Systems and Policy Research Unit, Ahmadu Bello University, Zaria, Nigeria.
(240)Department of Health Care Management, Technical University of Berlin, Berlin, Germany.
(241)Computer, Electrical, and Mathematical Sciences and Engineering Division, King Abdullah University of Science and Technology, Thuwal, Saudi Arabia.
(242)Department of Epidemiology and Biostatistics, Wuhan University, Wuhan, China.
(243)Department of Pediatrics and Child Health, Debre Berhan University, Debre Berhan, Ethiopia.
(244)College of Medicine and Public Health, Flinders University, Adeaide, SA, Australia.

(245)Research and Analytics Department, Initiative for Financing Health and Human Development, Chennai, India.
(246)Department of Research and Analytics, Bioinsilico Technologies, Chennai, India.
(247)Laboratory of Public Health Indicators Analysis and Health Digitalization, Moscow Institute of Physics and Technology, Dolgoprudny, Russia.
(248)Experimental Surgery and Oncology Laboratory, Kursk State Medical University, Kursk, Russia.
(249)Department of Biotechnology, University of Central Punjab, Lahore, Pakistan.
(250)Department of Pediatrics, Arak University of Medical Sciences, Arak, Iran.
(251)Department of Disease Control and Environmental Health, Makerere University, Kampala, Uganda.
(252)Department of General Surgery, Carol Davila University of Medicine and Pharmacy, Bucharest, Romania.
(253)Department of General Surgery, Emergency Hospital of Bucharest, Bucharest, Romania.
(254)Ministry of Health, Community Development, Gender, Elderly and Children, Dar es Salaam, Tanzania.
(255)Department of Public Health, University of Yaoundé I, Yaoundé, Cameroon.
(256)Department of Biological Sciences, University of Embu, Embu, Kenya.
(257)Institute for Global Health Innovations, Duy Tan University, Hanoi, Vietnam.
(258)South African Medical Research Council, Cape Town, South Africa.
(259)School of Public Health and Family Medicine, University of Cape Town, Cape Town, South Africa.
(260)Centre for Heart Rhythm Disorders, University of Adelaide, Adelaide, SA, Australia.
(261)Unit of Microbiology and Public Health, Institute of Medical Sciences, Las Tablas, Panama.
(262)Department of Public Health, Ministry of Health, Herrera, Panama.
(263)Department of Pediatrics, National Hospital, Abuja, Nigeria.
(264)Department of International Public Health, University of Liverpool, Liverpool, UK.
(265)Administrative and Economic Sciences Department, University of Bucharest, Bucharest, Romania.
(266)Department of Community Health and Primary Care, University of Lagos, Idi Araba, Nigeria.
(267)Department of Family and Preventive Medicine, University of Utah, Salt Lake City, UT, USA.
(268)Department of Psychiatry and Behavioural Neurosciences, McMaster University, Hamilton, ON, Canada.
(269)Department of Psychiatry, University of Lagos, Lagos, Nigeria.
(270)Community Prevention and Care Services, National AIDS Control Committee, Abuja, Nigeria.
(271)Centre for Healthy Start Initiative, Lagos, Nigeria.
(272)Diplomacy and Public Relations Department, University of Human Development,

Sulaymaniyah, Iraq.
(273)Department of Pharmacology and Therapeutics, University of Nigeria Nsukka, Enugu, Nigeria.
(274)University of Port Harcourt, Port Harcourt, Nigeria.
(275)Department of Project Management, National Research University Higher School of Economics, Moscow, Russia.
(276)Department of Medicine, University of Ibadan, Ibadan, Nigeria.
(277)Department of Medicine, University College Hospital, Ibadan, Ibadan, Nigeria.
(278)Department of Respiratory Medicine, Jagadguru Sri Shivarathreeswara Academy of Health Education and Research, Mysore, India.
(279)Department of Forensic Medicine and Toxicology, Manipal Academy of Higher Education, Manipal, India.
(280)Department of Health Metrics, Center for Health Outcomes & Evaluation, Bucharest, Romania.
(281)Research Department, Nepal Health Research Council, Kathmandu, Nepal.
(282)Research Department, Public Health Research Society Nepal, Kathmandu, Nepal.
(283)Saveetha Medical College and Hospitals, Saveetha University, Chennai, India.
(284)Iran University of Medical Sciences, Tehran, Iran.
(285)Department of Pediatrics, University of Melbourne, Melbourne, VIC, Australia.
(286)Population Health Theme, Murdoch Children's Research Institute, Melbourne, VIC, Australia.
(287)Department of Genetics, Yale University, New Haven, CT, USA.
(288)School of Global Public Health, New York University, New York, NY, USA.
(289)University Medical Center Groningen, University of Groningen, Groningen, Netherlands.
(290)School of Economics and Business, University of Groningen, Groningen, Netherlands.
(291)National Institute of Infectious Diseases, Bucuresti, Romania.
(292)Department of Infectious Diseases, Carol Davila University of Medicine and Pharmacy, Bucharest, Romania.
(293)School of Engineering, Macquarie University, Sydney, NSW, Australia.
(294)Pohang University of Science and Technology, Pohang, South Korea.
(295)College of Medicine, University of Central Florida, Orlando, FL, USA.
(296)Department of Immunology, Mazandaran University of Medical Sciences, Sari, Iran.
(297)Molecular and Cell Biology Research Center, Mazandaran University of Medical Sciences, Sari, Iran.
(298)Metabolomics and Genomics Research Center, Tehran University of Medical Sciences, Tehran, Iran.
(299)Sina Trauma and Surgery Research Center, Tehran University of Medical Sciences, Tehran, Iran.
(300)Future Technology Research Center, National Yunlin University of Science and Technology, Yunlin, Taiwan.

(301)Department of Epidemiology, Biostatistics and Occupational Health, McGill University, Montreal, QC, Canada.
(302)Research and Innovation Division, South Asian Institute for Social Transformation (SAIST), Dhaka, Bangladesh.
(303)Research Department, Policy Research Institute, Kathmandu, Nepal.
(304)Health and Public Policy Department, Global Center for Research and Development, Kathmandu, Nepal.
(305)Department of Oral Pathology, Sharavathi Dental College and Hospital, Shimogga, India.
(306)WHO Collaborating Centre for Public Health Education and Training, Imperial College London, London, UK.
(307)University College London Hospitals, London, UK.
(308)Department of Primary Care and Public Health, Imperial College London, London, UK.
(309)Academic Public Health England, Public Health England, London, UK.
(310)Department of Computer Science, Boston University, Boston, MA, USA.
(311)Department of Epidemiology and Biostatistics, Haramaya University, Harar, Ethiopia.
(312)Research Center for Immunodeficiencies, Tehran University of Medical Sciences, Tehran, Iran.
(313)Network of Immunity in Infection, Malignancy and Autoimmunity (NIIMA), Universal Scientific Education and Research Network (USERN), Tehran, Iran.
(314)Faculty of Business and Management, Universiti Sultan Zainal Abidin (Sultan Zainal Abidin University), Kuala Terengganu, Malaysia.
(315)Epidemiology Research Unit (EPIUnit), University of Porto, Porto, Portugal.
(316)Department of Global Health, University of Washington, Seattle, WA, USA.
(317)Department of Medicine, University of Washington, Seattle, WA, USA.
(318)African Genome Center, Mohammed VI Polytechnic University (UM6P), Ben Guerir, Morocco.
(319)Centro de Investigaciones en Anomalías Congénitas y Enfermedades Raras (Center for Research in Congenital Anomalies and Rare Diseases), Universidad ICESI (ICESI University), Cali, Colombia.
(320)Malaria Atlas Project, Telethon Kids Institute, Perth, Australia.
(321)Department of Health Statistics, National Institute for Medical Research, Dar es Salaam, Tanzania.
(322)Department of Internal Medicine, University of Botswana, Gaborone, Botswana.
(323)Department of Psychiatry, All India Institute of Medical Sciences, New Delhi, India.
(324)Department of Public Health, Madda Walabu University, Bale Robe, Ethiopia.
(325)Public Health and Community Medicine Department, Cairo University, Giza, Egypt.
(326)Drug Applied Research Center, Tabriz University of Medical Sciences, Tabriz, Iran.
(327)Department of Entomology, Ain Shams University, Cairo, Egypt.
(328)Centre for Tropical Medicine and Global Health, University of Oxford, Oxford, UK.

(329)Nuffield Department of Medicine, University of Oxford, Oxford, UK.
(330)Geriatric and Long Term Care Department, Hamad Medical Corporation, Doha, Qatar.
(331)Faculty of Health & Social Sciences, Bournemouth University, Bournemouth, UK.
(332)College of Public Health, Medical and Veterinary Sciences, James Cook University, QLD, Townsville, Australia.
(333)Public Health Division, An-Najah National University, Nablus, Palestine.
(334)Independent Consultant, Karachi, Pakistan.
(335)Faculty of Caring Science, Work Life, and Social Welfare, University of Borås, Borås, Sweden.
(336)College of Medicine, Yonsei University, Seoul, South Korea.
(337)Department of Internal Medicine, Yale University, New Haven, CT, USA.
(338)School of Medicine, University of Alabama at Birmingham, Birmingham, AL, USA.
(339)Medicine Service, US Department of Veterans Affairs (VA), Birmingham, AL, USA.
(340)Department No.16, Moscow Research and Practical Centre on Addictions, Moscow, Russia.
(341)Department of Infectious Diseases and Epidemiology, Pirogov Russian National Research Medical University, Moscow, Russia.
(342)Department of Community Medicine, Ahmadu Bello University, Zaria, Nigeria.
(343)Cancer Control Center, Osaka International Cancer Institute, Osaka, Japan.
(344)Department of Biomedical Sciences, Arba Minch University, Arba Minch, Ethiopia.
(345)University Institute "Egas Moniz", Monte da Caparica, Portugal.
(346)Research Institute for Medicines, University of Lisbon, Lisbon, Portugal.
(347)School of Public Health, Mekelle University, Mekelle, Ethiopia.
(348)Southgate Institute for Health and Society, Flinders University, Adelaide, SA, Australia.
(349)Department of Pathology and Legal Medicine, University of São Paulo, Ribeirão Preto, Brazil.
(350)Modestum LTD, London, UK.
(351)College of Medicine and Health Sciences, Bahir Dar University, Bahir Dar, Ethiopia.
(352)Department of Community Medicine, Alex Ekwueme Federal University Teaching Hospital Abakaliki, Abakaliki, Nigeria.
(353)Kasturba Medical College, Manipal Academy of Higher Education, Mangalore, India.
(354)Clinical Research Department, IRCCS Fondazione Don Carlo Gnocchi, Milan, Italy.
(355)Department of Medical and Surgical Sciences, University of Bologna, Bologna, Italy.
(356)Occupational Health Unit, Sant'Orsola Malpighi Hospital, Bologna, Italy.
(357)Faculty of Information Technology, HUTECH University, Ho Chi Minh City, Vietnam.
(358)Center of Excellence in Behavioral Medicine, Nguyen Tat Thanh University,

Ho Chi Minh City, Vietnam.
(359)Population Dynamics and Sexual and Reproductive Health, African Population and Health Research Center, Nairobi, Kenya.
(360)Foundation University Medical College, Foundation University Islamabad, Islamabad, Pakistan.
(361)Department of Cultures, Societies and Global Studies, Northeastern University, Boston, MA, USA.
(362)School of Public Health, University of Nairobi, Nairobi, Kenya.
(363)School of Population Health and Environmental Sciences, King's College London, London, UK.
(364)Centre for Health Policy Research, Torrens University Australia, Adelaide, SA, Australia.
(365)Department of community Medicine, Rajarata University of Sri Lanka, Anuradhapura, Sri Lanka.
(366)Department of Biostatistics, University of Washington, Seattle, WA, USA.
(367)School of International Development and Global Studies, University of Ottawa, Ottawa, ON, Canada.
(368)The George Institute for Global Health, University of Oxford, Oxford, UK.
(369)Centre for Suicide Research and Prevention, University of Hong Kong, Hong Kong, China.
(370)Department of Social Work and Social Administration, University of Hong Kong, Hong Kong, China.
(371)Department of Neuropsychopharmacology, National Center of Neurology and Psychiatry, Kodaira, Japan.
(372)Department of Public Health, Juntendo University, Tokyo, Japan.
(373)Department of Bioengineering and Therapeutic Sciences, University of California San Francisco, San Francisco, CA, USA.
(374)Addictology Department, Russian Medical Academy of Continuous Professional Education, Moscow, Russia.
(375)School of Public Health, Wuhan University of Science and Technology, Wuhan, China.
(376)Hubei Province Key Laboratory of Occupational Hazard Identification and Control, Wuhan University of Science and Technology, Wuhan, China.
(377)School of Medicine, Wuhan University, Wuhan, China.

BACKGROUND: Human immunodeficiency virus and acquired immune deficiency syndrome (HIV/AIDS) is still among the leading causes of disease burden and mortality in sub-Saharan Africa (SSA), and the world is not on track to meet targets set for ending the epidemic by the Joint United Nations Programme on HIV/AIDS (UNAIDS) and the United Nations Sustainable Development Goals (SDGs). Precise HIV burden information is critical for effective geographic and epidemiological targeting of prevention and treatment interventions. Age- and sex-specific HIV prevalence estimates are widely available at the national level, and region-wide local estimates were recently published for adults overall. We add further dimensionality to previous analyses by estimating HIV prevalence at local scales, stratified into sex-specific 5-year age groups for adults ages 15-59 years across SSA.

METHODS: We analyzed data from 91 seroprevalence surveys and sentinel surveillance among antenatal care clinic (ANC) attendees using model-based geostatistical methods to produce estimates of HIV prevalence across 43 countries in SSA, from years 2000 to 2018, at a 5 × 5-km resolution and presented among second administrative level (typically districts or counties) units.
RESULTS: We found substantial variation in HIV prevalence across localities, ages, and sexes that have been masked in earlier analyses. Within-country variation in prevalence in 2018 was a median 3.5 times greater across ages and sexes, compared to for all adults combined. We note large within-district prevalence differences between age groups: for men, 50% of districts displayed at least a 14-fold difference between age groups with the highest and lowest prevalence, and at least a 9-fold difference for women. Prevalence trends also varied over time; between 2000 and 2018, 70% of all districts saw a reduction in prevalence greater than five percentage points in at least one sex and age group. Meanwhile, over 30% of all districts saw at least a five percentage point prevalence increase in one or more sex and age group.
CONCLUSIONS: As the HIV epidemic persists and evolves in SSA, geographic and demographic shifts in prevention and treatment efforts are necessary. These estimates offer epidemiologically informative detail to better guide more targeted interventions, vital for combating HIV in SSA.

© 2022. The Author(s).

DOI: 10.1186/s12916-022-02639-z
PMCID: PMC9760541
PMID: 36529768 [Indexed for MEDLINE]

Conflict of interest statement: S Afzal reports leadership or fiduciary role in other board, society, committee or advocacy group, unpaid, with the Pakistan society of Community Medicine & Public Health, the Pakistan Association of Medical Editors, and the Pakistan Society of Medical Infectious Diseases, all outside the submitted work. R Ancuceanu reports 5 payment or honoraria for lectures, presentations, speakers bureaus, manuscript writing, or educational events from Avvie, Sandoz, and B Braun, all outside the submitted work. T W Bärnighausen reports research grants from the European Union (Horizon 2020 and EIT Health), German Research Foundation (DFG), US National Institutes of Health, German Ministry of Education and Research, Alexander von Humboldt Foundation, Else-Kröner-Fresenius-Foundation, Wellcome Trust, Bill & Melinda Gates Foundation, KfW, UNAIDS, and WHO; consulting fees from KfW on the OSCAR initiative in Vietnam; participation on a Data Safety Monitoring Board or Advisory Board with the NIH-funded study “Healthy Options” (PIs: Smith Fawzi, Kaaya), Chair, Data Safety and Monitoring Board (DSMB), German National Committee on the “Future of Public Health Research and Education,” Chair of the scientific advisory board to the EDCTP Evaluation, Member of the UNAIDS Evaluation Expert Advisory Committee, National Institutes of Health Study Section Member on Population and Public Health Approaches to HIV/AIDS (PPAH), US

National Academies of Sciences, Engineering, and Medicine's Committee for the "Evaluation of Human Resources for Health in the Republic of Rwanda under the President's Emergency Plan for AIDS Relief (PEPFAR)," University of Pennsylvania (UPenn) Population Aging Research Center (PARC) External Advisory Board Member; leadership or fiduciary role in other board, society, committee or advocacy group, paid or unpaid, as co-chair of the Global Health Hub Germany (which was initiated by the German Ministry of Health); all outside the submitted work. J das Neves reports grants or contracts from Ref. 13605 – Programa GÉNESE, Gilead Portugal (PGG/002/2016 – Programa GÉNESE, Gilead Portugal) outside the submitted work. L Dwyer-Lindgren reports support for the present manuscript from the Bill & Melinda Gates Foundation through grant OPP1132415. I Filip reports other financial or non-financial interests from Avicenna Medical and Clinical Research Institute, outside the submitted work. E Haeuser reports support for the present manuscript from the Bill & Melinda Gates Foundation through grant OPP1132415. C Herteliu reports grants from Romanian Ministry of Research Innovation and Digitalization, MCID, for project number ID-585-CTR-42-PFE-2021 (Jan 2022-Jun 2023) "Enhancing institutional performance through development of infrastructure and transdisciplinary research ecosystem within socio-economic domain – PERFECTIS," from Romanian National Authority for Scientific Research and Innovation, CNDS-UEFISCDI, for project number PN-III-P4-ID-PCCF-2016-0084 (Oct 2018-Sep 2022) "Understanding and modelling time-space patterns of psychology-related inequalities and polarization," and project number PN-III-P2-2.1-SOL-2020-2-0351 (Jun 2020-Oct 2020) "Approaches within public health management in the context of COVID-19 pandemic," and from the Ministry of Labour and Social Justice, Romania for project number "Agenda for skills Romania 2020-2025"; all outside the submitted work. J J Jozwiak reports payment or honoraria for lectures, presentations, speakers bureaus, manuscript writing, or educational events from Teva, Amgen, Synexus, Boehringer Ingelheim, Zentiva, and Sanofi as personal fees, all outside the submitted work. J Khubchandani reports other financial interests from Teva Pharmaceuticals, all outside the submitted work. K Krishnan reports other non-financial support from UGC Centre of Advanced Study, CAS II, Department of Anthropology, Panjab University, Chandigarh, India, outside the submitted work. H J Larson reports grants or contracts from the MacArthur Foundation and Merck to London School of Hygeine and Tropical Medicine, and from the Vaccine Confidence Fund to the University of Washington; payment or honoraria for lectures, presentations, speakers bureaus, manuscript writing, or educational events from Center for Strategic and International Studies as payment to LSHTM for co-chairing HighLevel Panel and from GSK as personal payment for developing training sessions and lectures; leadership or fiduciary role in other board, society, committee or advocacy group, pair, with the ApiJect Advisory Board; all outside the submitted work. O O Odukoya reports support for the present manuscript from the Fogarty International Center of the National Institutes of Health under the Award Number K43TW010704. The content is solely the responsibility of the authors and does not necessarily represent the official views of the National Institutes of Health. A Pans reports grants from Romanian National Authority for Scientific Research and Innovation, CNDS-UEFISCDI, for project number PN-III-P4-ID-PCCF-2016-0084 (Oct 2018-Sep

2022) "Understanding and modelling time-space patterns of psychology-related inequalities and polarization," and project number PN-III-P2-2.1-SOL-2020-2-0351 (Jun 2020-Oct 2020) "Approaches within public health management in the context of COVID-19 pandemic," outside the submitted work. S R Pandi-Perumal reports royalties from Springer for editing services; stock or stock options in Somnogen Canada Inc as the President and Chief Executive Officer; all outside the submitted work. A Radfar reports other financial or non-financial interests from Avicenna Medical and Clinical Research Institute, outside the submitted work. A I Ribeiro reports grants or contracts from National Funds through FCT, under the ʻStimulus of Scientific Employment – Individual Support' program within the contract CEECIND/02386/2018, outside the submitted work. J M Ross reports support for the present manuscript from the Bill & Melinda Gates Foundation through grant OPP1132415; grants or contracts from National Institutes of Health and Firland Foundation as payments to their institution; consulting fees from United States Agency for International Development as personal payments, and from KNCV Tuberculosis Foundation as payments to their institution; all outside the submitted work. E Rubagotti reports payment or honoraria for lectures, presentations, speakers bureaus, manuscript writing, or educational events from the Greenwich China Office and Unviersity Prince Mohammad VI, Morocco, all outside the submitted work. B Sartorius reports grants or contracts from DHSC – GRAM Project; Leadership or fiduciary role in other board, society, committee or advocacy group, paid or unpaid, as a member of the GBD Scientific Council and a Member of WHO RGHS; all outside the submitted work. J A Singh reports consulting fees from Crealta/Horizon, Medisys, Fidia, PK Med, Two labs Inc, Adept Field Solutions, Clinical Care options, Clearview healthcare partners, Putnam associates, Focus forward, Navigant consulting, Spherix, MedIQ, Jupiter Life Science LLC, UBM LLC, Trio Health, Medscape, WebMD, and Practice Point communications, and the National Institutes of Health and the American College of Rheumatology; payment or honoraria for participating in the speakers bureau for Simply Speaking; support for attending meetings and/or travel from the steering committee of OMERACT, to attend their meeting every 2 years; participation on a Data Safety Monitoring Board or Advisory Board as an unpaid member of the FDA Arthritis Advisory Committee; leadership or fiduciary role in other board, society, committee or advocacy group, paid or unpaid, as a member of the steering committee of OMERACT, an international organization that develops measures for clinical trials and receives arm's length funding from 12 pharmaceutical companies, with the Veterans Affairs Rheumatology Field Advisory Committee as Chair, and with the UAB Cochrane Musculoskeletal Group Satellite Center on Network Meta-analysis as a director and editor; stock or stock options in TPT Global Tech, Vaxart pharmaceuticals, Atyu Biopharma, Adaptimmune Therapeutics, GeoVax Labs, Pieris Pharmaceuticals, Enzolytics Inc, Series Therapeutics, Tonix Pharmaceuticals, and Charlotte's Web Holdings Inc. and previously owned stock options in Amarin, Viking, and Moderna pharmaceuticals; all outside the submitted work. N Taveira reports grants or contracts from FCT and Aga Khan Development Network (AKDN) – Portugal Collaborative Research Network in Portuguese speaking countries in Africa (Project reference: 332821690) and from European & Developing Countries Clinical Trials Partnership

(EDCTP), UE (Project reference: RIA2016MC-1615), as payments made to their institution, all outside the submitted work.

252. Front Cell Infect Microbiol. 2022 Dec 16;12:1058081. doi: 10.3389/fcimb.2022.1058081. eCollection 2022.

African-specific polymorphisms in Plasmodium falciparum serine repeat antigen 5 in Uganda and Burkina Faso clinical samples do not interfere with antibody response to BK-SE36 vaccination.

Arisue N(1)(2), Palacpac NMQ(3), Ntege EH(4), Yeka A(5), Balikagala B(6), Kanoi BN(7), Bougouma EC(8)(9), Tiono AB(8)(9), Nebie I(8)(9), Diarra A(8)(9), Houard S(10), D'Alessio F(10), Leroy O(10)(11), Sirima SB(8)(9), Egwang TG(12), Horii T(3).

Author information:
(1)Research Center for Infectious Disease Control, Research Institute for Microbial Diseases, Osaka University, Suita, Osaka, Japan.
(2)Section of Global Health, Division of Public Health, Department of Hygiene and Public Health, Tokyo Women's Medical University, Tokyo, Japan.
(3)Department of Malaria Vaccine Development, Research Institute for Microbial Diseases, Osaka University, Suita, Osaka, Japan.
(4)Department of Plastic and Reconstructive Surgery, University of the Ryukyus, Graduate School of Medicine and Hospital, Okinawa, Japan.
(5)Makerere University School of Public Health, Kampala, Uganda.
(6)Department of Tropical Medicine and Parasitology, School of Medicine, Juntendo University, Tokyo, Japan.
(7)Centre for Malaria Elimination (CME) and Centre for Research in Infectious Diseases (CRID), Directorate of Research and Innovation, Mount Kenya University, Thika, Kenya.
(8)Public Health Department, Institut National de Santé Publique/Centre National de Recherche et de Formation sur le Paludisme (INSP/CNRFP), Ouagadougou, Burkina Faso.
(9)Groupe de Recherche Action en Santé (GRAS), Ouagadougou, Burkina Faso.
(10)European Vaccine Initiative (EVI), Universitäts Klinikum Heidelberg, Heidelberg, Germany.
(11)Sorekara-x consultant, Paris, France.
(12)Med Biotech Laboratories, Kampala, Uganda.

BK-SE36, based on Plasmodium falciparum serine repeat antigen 5 (SERA5), is a blood-stage malaria vaccine candidate currently being evaluated in clinical trials. Phase 1 trials in Uganda and Burkina Faso have demonstrated promising safety and immunogenicity profiles. However, the genetic diversity of sera5 in Africa and the role of allele/variant-specific immunity remain a major concern. Here, sequence analyses were done on 226 strains collected from the two clinical trial/follow-up studies and 88 strains from two cross-sectional studies in

Africa. Compared to other highly polymorphic vaccine candidate antigens, polymorphisms in sera5 were largely confined to the repeat regions of the gene. Results also confirmed a SERA5 consensus sequence with African-specific polymorphisms. Mismatches with the vaccine-type SE36 (BK-SE36) in the octamer repeat, serine repeat, and flanking regions, and single-nucleotide polymorphisms in non-repeat regions could compromise vaccine response and efficacy. However, the haplotype diversity of SERA5 was similar between vaccinated and control participants. There was no marked bias or difference in the patterns of distribution of the SE36 haplotype and no statistically significant genetic differentiation among parasites infecting BK-SE36 vaccinees and controls. Results indicate that BK-SE36 does not elicit an allele-specific immune response.

Copyright © 2022 Arisue, Palacpac, Ntege, Yeka, Balikagala, Kanoi, Bougouma, Tiono, Nebie, Diarra, Houard, D'Alessio, Leroy, Sirima, Egwang and Horii.

DOI: 10.3389/fcimb.2022.1058081
PMCID: PMC9802637
PMID: 36590593 [Indexed for MEDLINE]

Conflict of interest statement: TH is the inventor of BK-SE36 and all rights have now been turned over to NPC. NP, EN, AY, BB, BK, and TE received remuneration from BIKEN for the Ugandan trial and follow-up. For the Burkina Faso clinical trial/follow-up study, the following received some support from NPC: EB, AD, SH, FD, OL, and NP. TH and NP are currently supported by a research fund from NPC. The remaining authors declare that the research was conducted in the absence of any commercial or financial relationships that could be construed as a potential conflict of interest.

253. Antibiotics (Basel). 2022 Dec 15;11(12):1824. doi: 10.3390/antibiotics11121824.

Ongoing Efforts to Improve Antimicrobial Utilization in Hospitals among African Countries and Implications for the Future.

Saleem Z(1), Godman B(2)(3)(4), Cook A(5)(6), Khan MA(7), Campbell SM(4)(8)(9), Seaton RA(10)(11), Siachalinga L(12), Haseeb A(13), Amir A(14), Kurdi A(2)(4)(15)(16), Mwita JC(17), Sefah IA(18), Opanga SA(19), Fadare JO(20)(21), Ogunleye OO(22)(23), Meyer JC(4)(24), Massele A(25), Kibuule D(26), Kalungia AC(27), Shahwan M(3)(28), Nabayiga H(29), Pichierri G(30), Moore CE(5).

Author information:
(1)Department of Pharmacy Practice, Faculty of Pharmacy, Bahauddin Zakariya University, Multan 60800, Pakistan.
(2)Department of Pharmacoepidemiology, Strathclyde Institute of Pharmacy and Biomedical Sciences, University of Strathclyde, Glasgow G4 0RE, UK.
(3)Centre of Medical and Bio-Allied Health Sciences Research, Ajman University,

Ajman 346, United Arab Emirates.
(4)Department of Public Health Pharmacy and Management, School of Pharmacy, Sefako Makgatho Health Sciences University, Molotlegi Street, Garankuwa, Pretoria 0208, South Africa.
(5)Centre for Neonatal and Paediatric Infection, St. George's University of London, London SW17 0RE, UK.
(6)Health Economics Research Centre, Nuffield Department of Population Health, University of Oxford, Oxford OX1 2JD, UK.
(7)The Indus Hospital, Bedian Road, Lahore 54000, Pakistan.
(8)Centre for Epidemiology and Public Health, School of Health Sciences, University of Manchester, Manchester M13 9PL, UK.
(9)NIHR Greater Manchester Patient Safety Translational Research Centre, School of Health Sciences, University of Manchester, Manchester M13 9PL, UK.
(10)Queen Elizabeth University Hospital, Govan Road, Glasgow G51 4TF, UK.
(11)Scottish Antimicrobial Prescribing Group, Healthcare Improvement Scotland, Delta House, 50 West Nile Street, Glasgow G1 2NP, UK.
(12)College of Pharmacy, Yeungnam University, Daehak-Ro, Gyeongsan, Gyeongbuk 38541, Republic of Korea.
(13)Department of Clinical Pharmacy, College of Pharmacy, Umm Al-Qura University, Makkah 24382, Saudi Arabia.
(14)Department of Microbiology, Armed Forces Institute of Pathology, National University of Medical Sciences, Rawalpindi 46000, Pakistan.
(15)Department of Pharmacology, College of Pharmacy, Hawler Medical University, Erbil 44001, Iraq.
(16)Center of Research and Strategic Studies, Lebanese French University, Erbil 44001, Iraq.
(17)Department of Internal Medicine, Faculty of Medicine, University of Botswana, Private Bag 0713 UB, Gaborone 00704, Botswana.
(18)Pharmacy Practice Department, School of Pharmacy, University of Health and Allied Sciences, Volta Region, Hohoe PMB 31, Ghana.
(19)Department of Pharmaceutics and Pharmacy Practice, School of Pharmacy, University of Nairobi, Nairobi P.O. Box 19676-00202, Kenya.
(20)Department of Pharmacology and Therapeutics, Ekiti State University, Ado Ekiti 362103, Nigeria.
(21)Department of Medicine, Ekiti State University Teaching Hospital, Ado Ekiti 360211, Nigeria.
(22)Department of Pharmacology, Therapeutics and Toxicology, Lagos State University College of Medicine, Ikeja, Lagos 100271, Nigeria.
(23)Department of Medicine, Lagos State University Teaching Hospital, Ikeja 100271, Nigeria.
(24)South African Vaccination and Immunisation Centre, Sefako Makgatho Health Sciences University, Molotlegi Street, Garankuwa, Pretoria 0208, South Africa.
(25)Department of Clinical Pharmacology and Therapeutics, Hurbert Kairuki Memorial University, 70 Chwaku Road Mikocheni, Dar Es Salaam P.O. Box 65300, Tanzania.
(26)Department of Pharmacology & Therapeutics, Busitema University, Mbale P.O. Box 236, Uganda.

(27)Department of Pharmacy, School of Health Sciences, University of Zambia, Lusaka P.O. Box 50110, Zambia.
(28)Department of Clinical Sciences, College of Pharmacy and Health Sciences, Ajman University, Ajman 346, United Arab Emirates.
(29)Management Science Department, Strathclyde Business School, University of Strathclyde, 199 Cathedral Street, Glasgow G4 0QU, UK.
(30)Microbiology Department, Torbay and South Devon Foundation Trust, Lowes Bridge Torbay Hospital, Torquay TQ2 7AA, UK.

There are serious concerns with rising antimicrobial resistance (AMR) across countries increasing morbidity, mortality and costs. These concerns have resulted in a plethora of initiatives globally and nationally including national action plans (NAPs) to reduce AMR. Africa is no exception, especially with the highest rates of AMR globally. Key activities in NAPs include gaining a greater understanding of current antimicrobial utilization patterns through point prevalence surveys (PPS) and subsequently instigating antimicrobial stewardship programs (ASPs). Consequently, there is a need to comprehensively document current utilization patterns among hospitals across Africa coupled with ASP studies. In total, 33 PPS studies ranging from single up to 18 hospitals were documented from a narrative review with typically over 50% of in-patients prescribed antimicrobials, up to 97.6% in Nigeria. The penicillins, ceftriaxone and metronidazole, were the most prescribed antibiotics. Appreciable extended prescribing of antibiotics up to 6 days or more post-operatively was seen across Africa to prevent surgical site infections. At least 19 ASPs have been instigated across Africa in recent years to improve future prescribing utilizing a range of prescribing indicators. The various findings resulted in a range of suggested activities that key stakeholders, including governments and healthcare professionals, should undertake in the short, medium and long term to improve future antimicrobial prescribing and reduce AMR across Africa.

DOI: 10.3390/antibiotics11121824
PMCID: PMC9774141
PMID: 36551481

Conflict of interest statement: The authors declare no relevant conflict of interest.

254. Malar J. 2022 Dec 10;21(1):380. doi: 10.1186/s12936-022-04386-1.

Modelling longitudinal binary outcomes with outcome dependent observation times: an application to a malaria cohort study.

Martins A(1)(2), Herzog SA(3)(4), Mugenyi L(5), Faes C(1), Hens N(1)(4)(6), Abrams S(7)(8).

Author information:

(1)Interuniversity Institute for Biostatistics and statistical Bioinformatics, Data Science Institute, UHasselt, Diepenbeek, Belgium.
(2)Department of Mathematics and Informatics, Eduardo Mondlane University, Maputo, Mozambique.
(3)Institute for Medical Informatics, Statistics and Documentation (IMI), Medical University of Graz, Graz, Austria.
(4)Centre for Health Economics Research and Modelling Infectious Diseases, Vaccine and Infectious Disease Institute, University of Antwerp, Antwerp, Belgium.
(5)Infectious Diseases Research Collaboration, Plot 2C Nakasero Hill road, Kampala, Uganda.
(6)Family Medicine and Population Health, University of Antwerp, Antwerp, Belgium.
(7)Interuniversity Institute for Biostatistics and statistical Bioinformatics, Data Science Institute, UHasselt, Diepenbeek, Belgium. steven.abrams@uhasselt.be.
(8)Family Medicine and Population Health, University of Antwerp, Antwerp, Belgium. steven.abrams@uhasselt.be.

BACKGROUND : In spite of the global reduction of 21% in malaria incidence between 2010 and 2015, the disease still threatens many lives of children and pregnant mothers in African countries. A correct assessment and evaluation of the impact of malaria control strategies still remains quintessential in order to eliminate the disease and its burden. Malaria follow-up studies typically involve routine visits at pre-scheduled time points and/or clinical visits whenever individuals experience malaria-like symptoms. In the latter case, infection triggers outcome assessment, thereby leading to outcome-dependent sampling (ODS). Commonly used methods to analyze such longitudinal data ignore ODS and potentially lead to biased estimates of malaria-specific transmission parameters, hence, inducing an incorrect assessment and evaluation of malaria control strategies. METHODS : In this paper, a new method is proposed to handle ODS by use of a joint model for the longitudinal binary outcome measured at routine visits and the clinical event times. The methodology is applied to malaria parasitaemia data from a cohort of [Formula: see text] Ugandan children aged 0.5-10 years from 3 regions (Walukuba-300 children, Kihihi-355 children and Nagongera-333 children) with varying transmission intensities (entomological inoculation rate equal to 2.8, 32 and 310 infectious bites per unit year, respectively) collected between 2011-2014. RESULTS : The results indicate that malaria parasite prevalence and force of infection (FOI) increase with age in the region of high malaria intensity with highest FOI in age group 5-10 years. For the region of medium intensity, the prevalence slightly increases with age and the FOI for the routine process is highest in age group 5-10 years, yet for the clinical infections, the FOI gradually decreases with increasing age. For the region with low intensity, both the prevalence and FOI peak at the age of 1 year after which the former remains constant with age yet the latter suddenly decreases with age for the clinically observed infections. CONCLUSION : Malaria parasite prevalence and FOI increase with age in the region of high malaria

intensity. In all study sites, both the prevalence and FOI are highest among previously asymptomatic children and lowest among their symptomatic counterparts. Using a simulation study inspired by the malaria data at hand, the proposed methodology shows to have the smallest bias, especially when consecutive positive malaria parasitaemia presence results within a time period of 35 days were considered to be due to the same infection.

© 2022. The Author(s).

DOI: 10.1186/s12936-022-04386-1
PMCID: PMC9741489
PMID: 36496382 [Indexed for MEDLINE]

Conflict of interest statement: The authors declare that they have no competing interests.

255. Nutrients. 2022 Dec 7;14(24):5208. doi: 10.3390/nu14245208.

Identifying Predictors for Minimum Dietary Diversity and Minimum Meal Frequency in Children Aged 6-23 Months in Uganda.

Scarpa G(1)(2), Berrang-Ford L(1), Galazoula M(2)(3), Kakwangire P(4), Namanya DB(5), Tushemerirwe F(6), Ahumuza L(5), Cade JE(2).

Author information:
(1)School of Environment, University of Leeds, Leeds LS2 9JT, UK.
(2)School of Food Science and Nutrition, University of Leeds, Leeds LS2 9JT, UK.
(3)Leeds Institute for Data Analytics, University of Leeds, Leeds LS2 9JT, UK.
(4)Department of Nutrition, Lira Regional Referral Hospital, Lira P.O. Box 2, Uganda.
(5)Ministry of Health, Kampala P.O. Box 7272, Uganda.
(6)Department of Community Health and Behavioural Sciences, Makerere University, Kampala P.O. Box 7062, Uganda.

Adequate complementary foods contribute to good health and growth in young children. However, many countries are still off-track in achieving critical complementary feeding indicators, such as minimum meal frequency (MMF), minimum dietary diversity (MDD) and minimum acceptable diet (MAD). In this study, we used the 2016 Ugandan Demographic Health Survey (UDHS) data to assess child feeding practices in young children aged 6-23 months. We assess and describe complementary feeding indicators (MMF, MDD and MAD) for Uganda, considering geographic variation. We construct multivariable logistic regression models-stratified by age-to evaluate four theorized predictors of MMF and MDD: health status, vaccination status, household wealth and female empowerment. Our findings show an improvement of complementary feeding practice indicators in Uganda compared to the past, although the MAD threshold was reached by only 22%

of children. Children who did not achieve 1 or more complementary feeding indicators are primarily based in the northern regions of Uganda. Cereals and roots were the foods most consumed daily by young children (80%), while eggs were rarely eaten. Consistent with our hypotheses, we found that health status, vaccination status and wealth were significantly positively associated with MDD and MMF, while female empowerment was not. Improving nutrition in infant and young children is a priority. Urgent nutritional policies and acceptable interventions are needed to guarantee nutritious and age-appropriate complementary foods to each Ugandan child in the first years of life.

DOI: 10.3390/nu14245208
PMCID: PMC9786234
PMID: 36558366 [Indexed for MEDLINE]

Conflict of interest statement: The funders had no role in the design of the study; in the collection, analyses, or interpretation of data; in the writing of the manuscript, or in the decision to publish the results. J.E.C. is Director of Dietary assessment Ltd. No other conflicts of interests to declare.

256. Glob Pediatr Health. 2022 Dec 2;9:2333794X221140518. doi: 10.1177/2333794X221140518. eCollection 2022.

Predictors of Adherence to Routine Immunization Schedule Among Caretakers of Children Aged 10 to 18 Months in Lira City, Uganda.

Abor J(1), Kabunga A(1), Nabasirye CK(1).

Author information:
(1)Lira University, Lira, Uganda.

BACKGROUND: Although the majority of nations have routine immunization programs in place as a public health strategy, more than 1.5 million children under the age of 5 die yearly worldwide due to inadequate vaccination coverage. This study investigated the predictors of adherence to routine immunization schedules in Lira city.
METHODS: This was a cross-sectional study among 420 caretakers of children aged 10 to 18 months. Bivariate and multiple regression analyses were conducted to assess the predictors of adherence to the full immunization schedule. A P-value > .05 was considered statistically significant at 95% CI.
RESULTS: The study result indicated that the majority, 237 (56.4%) of caretakers were aged 25 to 34 years, 205 (48.8%) had attained primary level education, and 284 (67.6%) were married. The results showed that 365 (87.0%) had their children fully immunized. The predictors of adherence to full immunization schedule were knowledge on when to start vaccination (AOR:5.65; 95% CI:1.82-17.55; P = .003), maternal outcome expectations (AOR:3.45; 95% CI:1.16-10.29; P = .03) and maternal knowledge (AOR:2.15; 95% CI:1.18-3.90; P = .01).

CONCLUSION: The study findings show that 9 in 10 of the caregivers adhered to the immunization schedule. The significant predictors of adherence to full immunization were flexible clinical hours, maternal outcome expectations and maternal knowledge. Based on the conclusions we recommend that government and service providers be flexible in clinic hours and continue health education to women of childbearing age at an early stage, especially during antenatal care visits, delivery and the postnatal period on childhood vaccination to maintain adherence to the routine immunization schedule.

© The Author(s) 2022.

DOI: 10.1177/2333794X221140518
PMCID: PMC9720796
PMID: 36479309

Conflict of interest statement: The author(s) declared no potential conflicts of interest with respect to the research, authorship, and/or publication of this article.

257. PLoS One. 2022 Dec 2;17(12):e0278692. doi: 10.1371/journal.pone.0278692. eCollection 2022.

COVID-19 vaccine acceptability, and uptake among people living with HIV in Uganda.

Muhindo R(1), Okoboi S(2), Kiragga A(2), King R(3), Arinaitwe WJ(2), Castelnuovo B(2).

Author information:
(1)Department of Nursing, College of Health Sciences, Makerere University, Kampala, Uganda.
(2)Infectious Diseases Institute, College of Health Sciences, Makerere University, Kampala, Uganda.
(3)University of California, San Francisco, California, United states of America.

BACKGROUND: Despite being a priority population for COVID-19 vaccination, limited data are available regarding acceptability of COVID-19 vaccines among people living with HIV (PLWH) in Sub-Saharan Africa. We described COVID-19 vaccine acceptability and factors associated with vaccine acceptability among PLWH in Uganda.
METHODS: This was a cross-sectional study conducted among PLWH, aged ≥18 years, enrolled participants who were seeking HIV care from six purposely selected accredited ART clinics in Kampala. We obtained data on vaccine acceptability defined as willingness to accept any of the available COVID-19 vaccines using interviewer-administered questionnaires. In addition, we assessed vaccination

status, complacency regarding COVID-19 disease, vaccine confidence, and vaccine convenience. Factors associated with COVID-19 vaccine acceptability were evaluated using modified Poisson regression with robust standard errors.
RESULTS: We enrolled 767 participants of whom 485 (63%) were women. The median age was 33 years [interquartile range (IQR) 28-40] for women and 40 years [IQR], (34-47) for men. Of the respondents 534 (69.6%,95% confidence interval [CI]: 66.3%-72.8%) reported receiving at least one vaccine dose, with women significantly more likely than men to have been vaccinated (73% vs. 63%; p = 0.003). Among the unvaccinated 169 (72.7%, 95% CI: 66.6%-78.0%) were willing to accept vaccination, had greater vaccine confidence (85.9% had strong belief that the vaccines were effective; 81.9% that they were beneficial and 71% safe for PLWH; 90.5% had trust in health care professionals or 77.4% top government officials), and believed that it would be easy to obtain a vaccine if one decided to be vaccinated (83.6%). Vaccine acceptability was positively associated with greater vaccine confidence (adjusted prevalence ratio [aPR] 1.44; 95% CI: 1.08-1.90), and positive perception that it would be easy to obtain a vaccine (aPR 1.57; 95% CI: 1.26-1.96).
CONCLUSION: vaccine acceptance was high among this cohort of PLWH, and was positively associated with greater vaccine confidence, and perceived easiness (convince) to obtained the vaccine. Building vaccine confidence and making vaccines easily accessible should be a priority for vaccination programs targeting PLWH.

Copyright: © 2022 Muhindo et al. This is an open access article distributed under the terms of the Creative Commons Attribution License, which permits unrestricted use, distribution, and reproduction in any medium, provided the original author and source are credited.

DOI: 10.1371/journal.pone.0278692
PMCID: PMC9718389
PMID: 36459514 [Indexed for MEDLINE]

Conflict of interest statement: The author(s) declare that they have no competing interests.

258. J R Stat Soc Ser A Stat Soc. 2022 Dec;185(Suppl 2):S668-S691. doi: 10.1111/rssa.12946. Epub 2022 Nov 24.

A Semiparametric Approach to Model-Based Sensitivity Analysis in Observational Studies.

Zhang B(1), Tchetgen Tchetgen EJ(2).

Author information:
(1)Vaccine and Infectious Disease Division, Fred Hutchinson Cancer Center, Seattle, WA, U.S.A.

(2)Department of Statistics and Data Science, The Wharton School, University of Pennsylvania, Philadelphia, PA, U.S.A.

When drawing causal inference from observational data, there is almost always concern about unmeasured confounding. One way to tackle this is to conduct a sensitivity analysis. One widely-used sensitivity analysis framework hypothesizes the existence of a scalar unmeasured confounder U and asks how the causal conclusion would change were U measured and included in the primary analysis. Work along this line often makes various parametric assumptions on U, for the sake of mathematical and computational convenience. In this article, we further this line of research by developing a valid sensitivity analysis that leaves the distribution of U unrestricted. Compared to many existing methods in the literature, our method allows for a larger and more flexible family of models, mitigates observable implications (Franks et al., 2019), and works seamlessly with any primary analysis that models the outcome regression parametrically. We construct both pointwise confidence intervals and confidence bands that are uniformly valid over a given sensitivity parameter space, thus formally accounting for unknown sensitivity parameters. We apply our proposed method on an influential yet controversial study of the causal relationship between war experiences and political activeness using observational data from Uganda.

DOI: 10.1111/rssa.12946
PMCID: PMC9916707
PMID: 36777968

259. BMJ Glob Health. 2022 Dec;7(12):e010982. doi: 10.1136/bmjgh-2022-010982.

Sudan virus disease outbreak in Uganda: urgent research gaps.

Ibrahim SK(1), Ndwandwe DE(2), Thomas K(3), Sigfrid L(4)(5), Norton A(1).

Author information:
(1)GloPID-R Research and Policy Team, Pandemic Sciences Institute, University of Oxford, Oxford, UK.
(2)Cochrane South Africa, South African Medical Research Council, Cape Town, South Africa.
(3)Walimu, NGO, Kampala, Uganda.
(4)GloPID-R Research and Policy Team, Pandemic Sciences Institute, University of Oxford, Oxford, UK louise.sigfrid@gmail.com.
(5)ISARIC Global Support Centre, Pandemic Sciences Institute, University of Oxford, Oxford, UK.

The Sudan ebolavirus (SUDV) outbreak highlights our ongoing vulnerability to re-emerging high-consequence infectious diseases. Although the Minister of health in Uganda has initiated public health measures in collaboration with

neighbouring countries and with support of the WHO, cases have continued to spread to several regions including the capital. The ongoing transmission, uncertain case numbers and no licensed vaccine or therapeutics available are a cause for concern. We searched four databases for SUDV research using the search terms "SUDV", "Sudan Virus" and "Ebola Sudan". Our analysis identified only 20 SUDV research studies. Most were implemented in the USA and only one in Uganda. Nine studies were on therapeutics, eight on vaccines, one on diagnostics, one in one health and one in social science. Our data highlight a lack of SUDV research and an urgent need for investment to identify an effective vaccine, and optimal supportive care and therapeutic strategies for all at risk groups as a key research priority. Research investments should be prioritised into vaccines and treatment strategies that will be accessible to high-risk populations in affected regions during the outbreak, to protect populations, improve individual outcomes and facilitate outbreak control.

© Author(s) (or their employer(s)) 2022. Re-use permitted under CC BY. Published by BMJ.

DOI: 10.1136/bmjgh-2022-010982
PMCID: PMC9809242
PMID: 36585031 [Indexed for MEDLINE]

Conflict of interest statement: Competing interests: None declared.

260. Front Immunol. 2022 Dec 1;13:1029029. doi: 10.3389/fimmu.2022.1029029. eCollection 2022.

Assessment of a diverse panel of transmitted/founder HIV-1 infectious molecular clones in a luciferase based CD8 T-cell mediated viral inhibition assay.

Fernandez N(1), Hayes P(1), Makinde J(1), Hare J(1)(2), King D(1), Xu R(3), Rehawi O(4), Mezzell AT(4), Kato L(5)(6), Mugaba S(5)(6), Serwanga J(5)(6), Chemweno J(7), Nduati E(7), Price MA(2)(8), Osier F(1), Ochsenbauer C(4), Yue L(3), Hunter E(3), Gilmour J(9); IAVI protocol C investigators.

Author information:
(1)IAVI Human Immunology Laboratory, Imperial College, London, United Kingdom.
(2)IAVI, New York, NY, United States.
(3)Emory Vaccine Center at Yerkes National Primate Research Center, Emory University, Atlanta, GA, United States.
(4)University of Alabama at Birmingham, Birmingham, AL, United States.
(5)Uganda Virus Research Institute, Entebbe, Uganda.
(6)Medical Research Council, Uganda Virus Research Institute and London School of Hygiene and Tropical Medicine Uganda Research Unit, Entebbe, Uganda.
(7)Kenya Medical Research Institute (KEMRI) Wellcome Trust Research Programme, Kilifi, Kenya.

(8)Department of Epidemiology and Biostatistics, University of California at San Francisco, San Francisco, CA, United States.
(9)Department of Infectious Diseases, Imperial College, London, United Kingdom.

INTRODUCTION: Immunological protection against human immunodeficiency virus-1 (HIV-1) infection is likely to require both humoral and cell-mediated immune responses, the latter involving cytotoxic CD8 T-cells. Characterisation of CD8 T-cell mediated direct anti-viral activity would provide understanding of potential correlates of immune protection and identification of critical epitopes associated with HIV-1 control.
METHODS: The present report describes a functional viral inhibition assay (VIA) to assess CD8 T-cell-mediated inhibition of replication of a large and diverse panel of 45 HIV-1 infectious molecular clones (IMC) engineered with a Renilla reniformis luciferase reporter gene (LucR), referred to as IMC-LucR. HIV-1 IMC replication in CD4 T-cells and CD8 T-cell mediated inhibition was characterised in both ART naive subjects living with HIV-1 covering a broad human leukocyte antigen (HLA) distribution and compared with uninfected subjects.
RESULTS & DISCUSSION: CD4 and CD8 T-cell lines were established from subjects vaccinated with a candidate HIV-1 vaccine and provided standard positive controls for both assay quality control and facilitating training and technology transfer. The assay was successfully established across 3 clinical research centres in Kenya, Uganda and the United Kingdom and shown to be reproducible. This IMC-LucR VIA enables characterisation of functional CD8 T-cell responses providing a tool for rational T-cell immunogen design of HIV-1 vaccine candidates and evaluation of vaccine-induced T-cell responses in HIV-1 clinical trials.

Copyright © 2022 Fernandez, Hayes, Makinde, Hare, King, Xu, Rehawi, Mezzell, Kato, Mugaba, Serwanga, Chemweno, Nduati, Price, Osier, Ochsenbauer, Yue, Hunter, Gilmour and The IAVI protocol C investigators.

DOI: 10.3389/fimmu.2022.1029029
PMCID: PMC9751811
PMID: 36532063 [Indexed for MEDLINE]

Conflict of interest statement: The authors declare that the research was conducted in the absence of any commercial or financial relationships that could be construed as a potential conflict of interest.

261. SSM Qual Res Health. 2022 Dec;2:None. doi: 10.1016/j.ssmqr.2022.100180.

Applying a social-ecological model to understand factors impacting demand for childhood vaccinations in Nigeria, Uganda, and Guinea.

Bell J(1), Lartey B(1), Spickernell G(1), Darrell N(1), Salt F(1), Gardner C(1), Richards E(1), Fasakin L(2), Egbeniyi S(2), Odongo E(3), Ssenkungu J(3), Kouadio

RK(4), Cissé M(5), Rérambyah ABAI(6), Adou M(6), West R(1)(7), Sharma S(1).

Author information:
(1)Ipsos Healthcare, 3 Thomas More Square, London, E1W 1YW, UK.
(2)CMRG, 24A, Eric Moore Street, Wemabod Estate, Off Ajao Road, Ikeja, Lagos, Nigeria.
(3)Ipsos Uganda, 3rd Floor Padre Pio House, Plot 32, Lumumba Avenue P.O. Box 21571, Kampala, Uganda.
(4)Ciblage, Immeuble 5658, 2ème Etage, Rue 13, Liberté 5, Sicap Liberte, Dakar, Senegal.
(5)Ciblage, En Face de l'Ecole les Ecureuils, Lambanyi, Ratoma, Conakry, Guinea.
(6)Ciblage, Face SODECI, Imm. Hué, Porte B10, Riviera Attoban, Cocody, 09 BP 799, Abidjan 09, Cote d'Ivoire.
(7)Boston University School of Public Health, 715 Albany St, Boston, MA, 02118, United States.

Vaccines have reduced child mortality across the world, but low levels of demand for vaccination threatens to undermine progress. Existing frameworks to understand demand tend to prioritise primary caregivers' decision-making processes. We aimed to build a wider understanding of vaccine demand by applying an adapted socio-ecological model to analyse 158 interviews with primary caregivers and fathers of young children, and community influencers in Nigeria, Uganda, and Guinea. We found that several factors come together to inform a primary caregiver's demand for vaccination, including their familial and social relationships, their interactions with government and healthcare institutions, and the wider social and cultural norms in their communities. The study suggests that interventions targeted at families and communities instead of individuals could be effective. The results could be used to ensure that vaccine demand frameworks used by researchers and intervention designers are comprehensive and consider a wider range of influences on the primary caregiver.

© 2022 The Authors.

DOI: 10.1016/j.ssmqr.2022.100180
PMCID: PMC9748306
PMID: 36531294

Conflict of interest statement: The authors declare the following financial interests/personal relationships which may be considered as potential competing interests: All authors work for market and social research organisations that carry out research on behalf of commercial companies, including in the field of vaccines.

262. J Int AIDS Soc. 2022 Dec;25(12):e26036. doi: 10.1002/jia2.26036.

Service delivery challenges in HIV care during the first year of the COVID-19

pandemic: results from a site assessment survey across the global IeDEA consortium.

Brazier E(1)(2), Ajeh R(3), Maruri F(4), Musick B(5), Freeman A(6), Wester CW(4), Lee MP(7), Shamu T(8)(9), Crabtree Ramírez B(10), d'Almeida M(11), Wools-Kaloustian K(5), Kumarasamy N(12), Althoff KN(6), Twizere C(13), Grinsztejn B(14), Tanser F(15), Messou E(16), Byakwaga H(17), Duda SN(18)(19), Nash D(1)(2); International epidemiology Databases to Evaluate AIDS.

Collaborators: Chansilpa C, Dougherty T, Karminia A, Law M, Ross J, Sohn A, Aguirre I, Baker D, Bloch M, Cabot S, Carr A, Couldwell D, Edwards S, Eu B, Farlow H, Finlayson R, Gunathilake M, Hazlewood C, Hoy J, Langton-Lockton J, Le J, Leprince E, Minc A, Moore R, O'Sullivan M, Roth N, Rowling D, Russell D, Ryder N, Saunders C, Silvers J, Smith DJ, Sowden D, Sweeney G, Tan L, Teague R, Templeton D, Thng C, Woolley I, Khol V, Ly PS, Li TH, Po LM, Kinikar A, Kumarasamy N, Mundhe S, Pujari S, Sangle S, Nimkar S, Jassin M, Kurniati N, Merati TP, Muktiarti D, Amalia R, Sukmawati NMDD, Wati KDK, Yunihastuti E, Tanuma J, Choi JY, Azwa RISR, Cheng CK, Gani YM, Mohamed TJ, Moy FS, Nallusamy R, Nor MZM, Rudi N, Shyan WP, Yusoff NKN, Ditangco R, Chan YJ, Wu PC, Wu PF, Avihingsanon A, Chaiwarith R, Chokephaibulkit K, Khusuwan S, Kiertiburanakul S, Kosalaraksa P, Lumbiganon P, Ounchanam P, Puthanakit T, Rungmaitree S, Solai N, Sudjaritruk T, An VT, Cuong DD, Do CV, Huy BV, Quy T, Van Nguyen K, Nguyen L, Nguyen VL, Nguyen YT, Nong VM, Truong HK, Tuyen NTT, McGowan CC, Duda S, Maruri F, Wester CW, Cahn F, Cahn P, Cesar C, Fink V, Sued O, Coelho L, Machado DM, Pinto J, Wolff M, Rouzier V, Padgett D, Ramírez BC, Gotuzzo E, Brazier E, Nash D, Biziragusenyuka J, Gateretse P, Nimbona P, Niyonkuru O, Twizere C, Ajeh R, Anicetus S, Djenabou A, Enow P, Mbu E, Manga M, Ndobe M, Nasah J, Ekossono ENS, Bouseko MT, Kitetele F, Lelo P, Diafouka MIJ, Mafoua A, Nsonde DM, Bihira UAM, Dusabe MC, Feza R, Habanabashaka JC, Habumuremyi V, Igizeneza E, Kamigisha AM, Kubwimana G, Maniriho G, Mbaraga G, Muhoza B, Mukakarangwa J, Mukamana J, Mukanyirigira P, Mukeshimana YC, Munyaneza A, Murenzi G, Musaninyange J, Nyiraneza JN, Ntarambirwa F, Nyiraneza ML, Tuyishime J, Tuyishimire Y, Ubandutira A, Umugiraneza F, Umugwaneza R, Uwamahoro O, Uwamahoro P, Uwambaje MV, Uwimpuhwe C, Uwiragiye S, Kuhn YY, Musick B, Wools-Kaloustian K, Adera F, Adhiambo B, Aggrey K, Akadikor D, Ambulla F, Apiyo D, Ariya P, Atemba N, Ayodi F, Benard C, Bett M, Birgen S, Bwalei R, Chebon N, Chebor VJ, Chebuiywo P, Chemutai J, Chepkorir E, Chepseba C, Chirchir J, Chirchir J, Diero L, Dukwa B, Elphas A, Etyang T, Idiama A, Jebichuko A, Jepchumba D, Juma C, Juma M, Juma S, Kadima J, Karani R, Keitany C, Keter P, Kiavoga L, Kibet H, Kimutai R, Kiplagat M, Kiprono W, Kipruto NK, Kirimi A, Koech Z, Kosgei C, Kutto K, Kweyu M, Liech EK, Limo M, Maina R, Marumbu P, Masese A, Mochotto P, Molly O, Momanyi T, Murutu JW, Mwanda P, Ndakalu L, Nderitu RN, Obatsa S, Obiga F, Oboya M, Odhiambo J, Olaya G, Omanyala O, Oray C, Otieno M, Otwane MT, Ouma P, Owuor C, Pepela DT, Pessah C, Rotich E, Rotich EK, Rutto TC, Shikuku M, Sibweche RN, Simiyu RW, Siria H, Some M, Songok WC, Tanui I, Wafula G, Wambura R, Wanjala E, Wanyama C, Wanyonyi H, Woyakapel E, Zelbabel W, Judy, Kiprop, Beatrice, Leah, Dominic, Tallam, Gwimo D, Kinyota E, Lwali J, Lyamuya R, Machemba R, Mathias J,

Mkombachepa L, Mokiwa A, Mushi O, Ndunguru C, Ngonyani K, Nyaga C, Ruta H, Urassa M, Akanyihayo J, Arinaitwe A, Batuuka J, Birungi W, Bugembe JN, Ddungu A, Francis K, Imran B, Kafuuma GW, Kalulue JB, Kanaabi G, Kanyesigye M, Karuhanga G, Kasozi C, Kasule G, Katusime A, Kibalama D, Kibalama D, Kimera SP, Kulusumu N, Lule Y, Lwanga I, Mluindwa M, Moses J, Mubarak S, Muggaga D, Mukalazi E, Muleebwa J, Mulema D, Musisi I, Muwawu J, Muyindike W, Mwaka D, Naava M, Nabiyki I, Nabusulwa A, Nakabugo D, Nakamya E, Nakanwagi D, Nakato O, Nakayi L, Nakigozi P, Nakku J, Nakuya J, Nakyomu J, Namayanja J, Namirembe S, Namugumya J, Namukasa E, Namulindwa V, Nankya I, Nannyondo GM, Nansamba H, Nansera D, Nanyanzi B, Nanyonjo EC, Nayiga I, Opira I, Owarwo NC, Resty S, Semuwemba H, Senoga J, Sseguya G, Ssekyewa JP, Ssemakadde M, Tebajjwa J, Tugumisirize D, Tushemerirwe R, Waliyi K, Fenehance, Medard, Moore R, Althoff K, Freeman A, Bishop J, Gill MJ, Loutfy M, Smith G, Bamford L, Black A, Brice A, Brown S, Colasanti J, Duarte P, Firnhaber C, Goetz M, Grasso C, Gripshover B, Horberg M, Kelly R, Levine K, Luu M, Marconi V, Maroney K, Mayer K, Mayor A, McGowan C, Moore R, Multani A, Napravnik S, Nijhawan A, Novak R, Palella F, Rodriguez MC, Scott M, Tedaldi E, Willig J, Cornell M, Davies MA, Egger M, Haas A, Bereng M, Kalake M, Lenela K, Seretse R, Chintenga M, Chiwoko J, Gumulira J, Huwa J, Maluwa R, Matanje B, Mbewe R, Mfungwe S, Mphande Z, Tweya H, Rafael I, Apolles P, Beneke E, Dlamini S, Edson C, Eley B, Euvrard J, Fatti G, Goeieman B, Grimwood A, Huang D, Hugo S, Ismail Z, Jennings L, Mathenjwa T, Monteith L, Mshweshwe Z, Ntuli M, Ndlovu EN, Ndlozi H, Noyakaza S, Prozesky H, Rabie H, Sipambo N, Technau KG, Tembe T, Xaba N, Njobvu T, Munthaly M, Mwetwa E, Kabeba G, Mwendafilumba D, Maanguka E, Manyika N, Mwansa C, Banda F, Mwenda D, Bwalya A, Shapi L, Syame K, Sashi R, Mulenga C, Nanyangwe R, Chimbetete C, Chinofunga A, Mhike J, Mubvigwi E, Nyika F, Quarter KP, Arikawa SC, Becquet R, Bernard C, Dabis F, Desmonde S, Dahourou D, Ekouevi DK, Jaquet A, Jesson J, Leroy V, Malateste K, Rabourdin E, Tiendrebeogo T, Assogba M, d'Almeida M, Zannou DM, Hounhoui G, Bere D, Poda A, Pooda G, Traore R, Abauble Y, Abby O, Acquah P, Andoble V, N'Dzama Aude Y, Azani JC, Berete O, Beugre JD, Bohoussou CY, Brou SBE, Chenal H, Cissé A, Coulibaly N, Dainguy ME, Daligou M, Thomas d'Aquin T, Dasse CD, Folquet MA, Gnepa G, Gobe O, Guira S, Hawerlander D, Horo A, Kanga G, Messou ZKE, Minga KA, Moh R, N'Gbeche M, Ogbo P, Oulai M, Stéphanie SE, Eboua T, Valère IM, Afrane AKA, Akrofi E, Andoh JC, Renner L, Bagayoko A, Bagayoko K, Bah AS, Berthe A, Coulibaly B, Coulibaly F, Coulibaly YA, Diakité A, Bocoum F, Boré F, Dicko F, Koné O, Sylla M, Tangara A, Traoré M, Seydi M, Amegatse E, Djossou J, Takassi E, Palanga S.

Author information:
(1)Institute for Implementation Science in Population Health, City University of New York, New York, New York, USA.
(2)Graduate School of Public Health and Health Policy, City University of New York, New York, New York, USA.
(3)Clinical Research Education Networking and Consultancy, Yaoundé, Cameroon.
(4)Department of Medicine, Division of Infectious Diseases, Vanderbilt University Medical Center, Nashville, Tennessee, USA.
(5)Department of Biostatistics and Health Data Science, Indiana University School of Medicine, Indianapolis, Indiana, USA.

(6)Department of Epidemiology, Bloomberg School of Public Health, Johns Hopkins University, Baltimore, Maryland, USA.
(7)Queen Elizabeth Hospital, Hong Kong, China.
(8)Newlands Clinic, Harare, Zimbabwe.
(9)Institute of Social and Preventive Medicine (ISPM), University of Bern, Bern, Switzerland.
(10)Departamento de Infectología, Instituto Nacional de Ciencias Médicas y Nutrición, Mexico City, Mexico.
(11)Centre National Hospitalier, Universitaire Hubert K. Maga, Cotonou, Benin.
(12)VHS Infectious Diseases Medical Centre, Voluntary Health Services, Chennai, India.
(13)Centre National de Reference en Matière de VIH/SIDA, Bujumbura, Burundi.
(14)Laboratory of Clinical Research in STD/AIDS (LAPCLIN-AIDS), Evandro Chagas National Institute of Infectious Diseases-Oswaldo Cruz Foundation (INI/FIOCRUZ), Rio de Janeiro, Brazil.
(15)Africa Health Research Institute, University of KwaZulu-Natal, Durban, South Africa.
(16)ACONDA - Centre de Prise en Charge, de Recherche et de Formation (CePReF), Abidjan, Côte d'Ivoire.
(17)Mbarara University of Science and Technology, Mbarara, Uganda.
(18)Department of Biomedical Informatics, Vanderbilt University Medical Center (VUMC), Nashville, Tennessee, USA.
(19)Vanderbilt Institute for Clinical and Translational Research, Vanderbilt University Medical Center (VUMC), Nashville, Tennessee, USA.

INTRODUCTION: Interruptions in treatment pose risks for people with HIV (PWH) and threaten progress in ending the HIV epidemic; however, the COVID-19 pandemic's impact on HIV service delivery across diverse settings is not broadly documented.
METHODS: From September 2020 to March 2021, the International epidemiology Databases to Evaluate AIDS (IeDEA) research consortium surveyed 238 HIV care sites across seven geographic regions to document constraints in HIV service delivery during the first year of the pandemic and strategies for ensuring care continuity for PWH. Descriptive statistics were stratified by national HIV prevalence (<1%, 1-4.9% and ≥5%) and country income levels.
RESULTS: Questions about pandemic-related consequences for HIV care were completed by 225 (95%) sites in 42 countries with low (n = 82), medium (n = 86) and high (n = 57) HIV prevalence, including low- (n = 57), lower-middle (n = 79), upper-middle (n = 39) and high- (n = 50) income countries. Most sites reported being subject to pandemic-related restrictions on travel, service provision or other operations (75%), and experiencing negative impacts (76%) on clinic operations, including decreased hours/days, reduced provider availability, clinic reconfiguration for COVID-19 services, record-keeping interruptions and suspension of partner support. Almost all sites in low-prevalence and high-income countries reported increased use of telemedicine (85% and 100%, respectively), compared with less than half of sites in high-prevalence and lower-income settings. Few sites in high-prevalence settings

(2%) reported suspending antiretroviral therapy (ART) clinic services, and many reported adopting mitigation strategies to support adherence, including multi-month dispensing of ART (95%) and designating community ART pick-up points (44%). While few sites (5%) reported stockouts of first-line ART regimens, 10-11% reported stockouts of second- and third-line regimens, respectively, primarily in high-prevalence and lower-income settings. Interruptions in HIV viral load (VL) testing included suspension of testing (22%), longer turnaround times (41%) and supply/reagent stockouts (22%), but did not differ across settings.
CONCLUSIONS: While many sites in high HIV prevalence settings and lower-income countries reported introducing or expanding measures to support treatment adherence and continuity of care, the COVID-19 pandemic resulted in disruptions to VL testing and ART supply chains that may negatively affect the quality of HIV care in these settings.

© 2022 The Authors. Journal of the International AIDS Society published by John Wiley & Sons Ltd on behalf of the International AIDS Society.

DOI: 10.1002/jia2.26036
PMCID: PMC9742047
PMID: 36504431 [Indexed for MEDLINE]

Conflict of interest statement: KN Althoff is a consultant to the All of Us Research Program and TrioHealth, Inc. D Nash reports a research grant from Pfizer to assess the effect of COVID-19 vaccination on long-haul COVID. All authors have no competing interests.

263. Bull World Health Organ. 2022 Dec 1;100(12):808-814. doi: 10.2471/BLT.22.288526. Epub 2022 Oct 3.

Saliva-based methods for SARS-CoV-2 testing in low- and middle-income countries.

Tan SH(1), Allicock OM(1), Katamba A(2), Carrington CVF(3), Wyllie AL(1), Armstrong-Hough M(4).

Author information:
(1)Department of Epidemiology of Microbial Diseases, Yale School of Public Health, 60 College St, New Haven, CT06510, United States of America (USA).
(2)Uganda TB Implementation Research Consortium, Makerere University, Kampala, Uganda.
(3)Department of Preclinical Sciences, University of the West Indies, St. Augustine, Trinidad and Tobago.
(4)Departments of Social & Behavioral Science and Epidemiology, New York University School of Global Public Health, New York, USA.

As the coronavirus disease 2019 (COVID-19) continues to disproportionately

affect low- and middle-income countries, the need for simple, accessible and frequent diagnostic testing grows. In lower-resource settings, case detection is often limited by a lack of available testing for severe acute respiratory syndrome coronavirus 2 (SARS-CoV-2). To address global inequities in testing, alternative sample types could be used to increase access to testing by reducing the associated costs. Saliva is a sensitive, minimally invasive and inexpensive diagnostic sample for SARS-CoV-2 detection that is appropriate for asymptomatic surveillance, symptomatic testing and at-home collection. Saliva testing can lessen two major challenges faced by lower- and middle-income countries: constrained resources and overburdened health workers. Saliva sampling enables convenient self-collection and requires fewer resources than swab-based methods. However, saliva testing for SARS-CoV-2 diagnostics has not been implemented on a large scale in low- and middle-income countries. While numerous studies based in these settings have demonstrated the usefulness of saliva sampling, there has been insufficient attention on optimizing its implementation in practice. We argue that implementation science research is needed to bridge this gap between evidence and practice. Low- and middle-income countries face many barriers as they continue their efforts to provide mass COVID-19 testing in the face of substantial inequities in global access to vaccines. Laboratories should look to replicate successful approaches for sensitive detection of SARS-CoV-2 in saliva, while governments should act to facilitate mass testing by lifting restrictions that limit implementation of saliva-based methods.

264. BMC Psychol. 2022 Dec 1;10(1):284. doi: 10.1186/s40359-022-00998-z.

The psychological impact, risk factors and coping strategies to COVID-19 pandemic on healthcare workers in the sub-Saharan Africa: a narrative review of existing literature.

Oyat FWD(1), Oloya JN(1)(2), Atim P(1)(3), Ikoona EN(4), Aloyo J(1)(5), Kitara DL(6)(7)(8).

Author information:
(1)Uganda Medical Association (UMA), UMA-Acholi Branch, Gulu City, Uganda.
(2)Moroto Regional Referral Hospital, Moroto, Uganda.
(3)St. Joseph's Hospital, Kitgum District, Uganda.
(4)ICAP at Columbia University, Freetown, Sierra Leone.
(5)Rhites-N, Acholi, Gulu City, Uganda.
(6)Uganda Medical Association (UMA), UMA-Acholi Branch, Gulu City, Uganda. klagoro2@gmail.com.
(7)Faculty of Medicine, Department of Surgery, Gulu University, P.O. Box 166, Gulu City, Uganda. klagoro2@gmail.com.
(8)Harvard University, Cambridge, USA. klagoro2@gmail.com.

BACKGROUND: The ongoing COVID-19 pandemic has significantly impacted the physical and mental health of the general population worldwide, with healthcare workers at particular risk. The pandemic's effect on healthcare workers' mental

well-being has been characterized by depression, anxiety, work-related stress, sleep disturbances, and post-traumatic stress disorder. Hence, protecting the mental well-being of healthcare workers (HCWs) is a considerable priority. This review aimed to determine risk factors for adverse mental health outcomes and protective or coping measures to mitigate the harmful effects of the COVID-19 crisis among HCWs in sub-Saharan Africa.
METHODS: We performed a literature search using PubMed, Google Scholar, Cochrane Library, and Embase for relevant materials. We obtained all articles published between March 2020 and April 2022 relevant to the subject of review and met pre-defined eligibility criteria. We selected 23 articles for initial screening and included 12 in the final review.
RESULT: A total of 5,323 participants in twelve studies, predominantly from Ethiopia (eight studies), one from Uganda, Cameroon, Mali, and Togo, fulfilled the eligibility criteria. Investigators found 16.3-71.9% of HCWs with depressive symptoms, 21.9-73.5% with anxiety symptoms, 15.5-63.7% experienced work-related stress symptoms, 12.4-77% experienced sleep disturbances, and 51.6-56.8% reported PTSD symptoms. Healthcare workers, working in emergency, intensive care units, pharmacies, and laboratories were at higher risk of adverse mental health impacts. HCWs had deep fear, anxious and stressed with the high transmission rate of the virus, high death rates, and lived in fear of infecting themselves and families. Other sources of fear and work-related stress were the lack of PPEs, availability of treatment and vaccines to protect themselves against the virus. HCWs faced stigma, abuse, financial problems, and lack of support from employers and communities.
CONCLUSION: The prevalence of depression, anxiety, insomnia, and PTSD in HCWs in sub-Saharan Africa during the COVID-19 pandemic has been high. Several organizational, community, and work-related challenges and interventions were identified, including improvement of workplace infrastructures, adoption of correct and shared infection control measures, provision of PPEs, social support, and implementation of resilience training programs. Setting up permanent multidisciplinary mental health teams at regional and national levels to deal with mental health and providing psychological support to HCWs, supported with long-term surveillance, are recommended.

© 2022. The Author(s).

DOI: 10.1186/s40359-022-00998-z
PMCID: PMC9714392
PMID: 36457038 [Indexed for MEDLINE]

Conflict of interest statement: All authors declare no conflict of interest.

265. BMC Public Health. 2022 Dec 1;22(1):2243. doi: 10.1186/s12889-022-14723-3.

Dynamics of Hepatitis B infection prevention practices among pregnant women attending antenatal care at Lubaga Hospital Kampala, Uganda using the constructs

of information-motivation-behavioural skills model.

Afolabi IB(1), Aremu AB(2), Maidoki LA(3), Atulomah NO(3).

Author information:
(1)Faculty of Science and Technology, Department of Public Health, Cavendish University, Kampala, Uganda. harforly@gmail.com.
(2)Faculty of Health Sciences, Department of Human Anatomy, Islamic University in Uganda, Kampala Campus, Kampala, Uganda.
(3)Faculty of Science and Technology, Department of Public Health, Cavendish University, Kampala, Uganda.

BACKGROUND: Hepatitis B virus (HBV) infection is considered a significant global public health challenge with infectivity as well as estimated potential for transmission more than 50 to 100 times that of HIV. Over time, numerous empirical studies have shown that majority of HBV-related yearly global deaths are secondary to carcinoma of the liver. It is also known that HBV infected Women have the potential to transmit the infection vertically to their infants during pregnancy. This accounts for the WHO reported 3.16% prevalence among children less than 5 years of age in Uganda. This study assessed the predictors of HBV infection prevention practices among eligible consenting pregnant women using Lubaga health facility for antenatal care (ANC).
METHODS: A cross-sectional descriptive study employing quantitative data collection based on the constructs of IMB model was used to capture data on the study variables among 385 randomly selected eligible pregnant women attending antenatal care at Lubaga hospital between September 2020 and October 2020. Data derived from the quantitative instrument was analysed by data reduction and transformation to summaries of descriptive statistics using (SPSS version 26) and regression analysis was performed to establish characteristics of the association between the variables with significance level set as ($p < 0.05$).
Chi-square goodness-of-fit test was employed for significant differences in the proportion of dichotomous responses.
RESULTS: The findings showed that more than half of the respondents (59%) were between the ages of 18 and 28 and majority of them (42.3%) had secondary education. Furthermore, an average but inadequate knowledge ([Formula: see text] 5.97 ± 6.61; B = 0.57; $p < .001$), positive perception ([Formula: see text] 17.10 ± 18.31; B = 0.97; $p = .014$) and good behavioural skills ([Formula: see text] 12.39 ± 13.37; B = 0.56; $p < .001$) for adopting prevention practices all statistically predicted the averagely acceptable level of prevention practices ([Formula: see text] 15.03 ± 16.20) among the study respondents as measured on rating scales of 12, 33, 21 and 30 respectively.
CONCLUSION AND RECOMMENDATION: There were observed gaps in their knowledge about some basic features of the infection like transmission and risk factors as well as some misperceptions about vaccination despite the relatively average score level for both, which is likely to influence their prevention behaviours and predispose them to the risk of the infection if actions are not taken.
Therefore, personalized health education is needed during antenatal visits and

subsequent health campaign in order to inform better prevention practices among this vulnerable population group.

© 2022. The Author(s).

DOI: 10.1186/s12889-022-14723-3
PMCID: PMC9714095
PMID: 36457005 [Indexed for MEDLINE]

Conflict of interest statement: The Authors declared no competing interests for the study.

266. Lancet Glob Health. 2022 Dec;10(12):e1835-e1844. doi: 10.1016/S2214-109X(22)00432-6.

Influenza vaccine to reduce adverse vascular events in patients with heart failure: a multinational randomised, double-blind, placebo-controlled trial.

Loeb M(1), Roy A(2), Dokainish H(3), Dans A(4), Palileo-Villanueva LM(4), Karaye K(5), Zhu J(6), Liang Y(6), Goma F(7), Damasceno A(8), Alhabib KF(9), Yonga G(10), Mondo C(11), Almahmeed W(12), Al Mulla A(13), Thanabalan V(14), Rao-Melacini P(3), Grinvalds A(3), McCready T(3), Bangdiwala SI(15), Yusuf S(16); Influenza Vaccine to Prevent Adverse Vascular Events investigators.

Author information:
(1)Department of Pathology and Molecular Medicine, McMaster University, Hamilton, ON, Canada; Department of Health Research Methods, Evidence, and Impact, McMaster University, Hamilton, ON, Canada. Electronic address: loebm@mcmaster.ca.
(2)All India Institute of Medical Sciences, Delhi, India.
(3)Population Health Research Institute, Hamilton, ON, Canada.
(4)Department of Medicine, College of Medicine, University of the Philippines Manila, Manila, Philippines.
(5)Department of Medicine, Bayero University and Aminu Kano Teaching Hospital, Kano, Nigeria.
(6)Department of Cardiovascular Medicine, Cardiovascular Institute and Fuwai Hospital, Chinese Academy of Medical Sciences and Peking Union Medical College, Beijing, China.
(7)Department of Physiological Sciences, School of Medicine, University of Zambia, Lusaka, Zambia.
(8)Department of Medicine, Faculty of Medicine, Eduardo Mondlane University, Maputo, Mozambique.
(9)Department of Cardiac Sciences, King Fahad Cardiac Center, College of Medicine, King Saud University, Riyadh, Saudi Arabia.
(10)Department of Clinical Medicine and Therapeutics, University of Nairobi, Nairobi, Kenya.

(11)Kirudda Hospital, Kampala, Uganda.
(12)Department of Cardiovascular Medicine, Heart and Vascular Institute, Cleveland Clinic Abu Dhabi, Abu Dhabi, United Arab Emirates.
(13)Division of Cardiology, Cardiac Sciences Institute, Sheikh Khalifa Medical City, Abu Dhabi, United Arab Emirates.
(14)Department of Pathology and Molecular Medicine, McMaster University, Hamilton, ON, Canada.
(15)Department of Health Research Methods, Evidence, and Impact, McMaster University, Hamilton, ON, Canada; Population Health Research Institute, Hamilton, ON, Canada.
(16)Department of Medicine, McMaster University, Hamilton, ON, Canada; Population Health Research Institute, Hamilton, ON, Canada.

Erratum in
Lancet Glob Health. 2022 Nov 30;:

Comment in
Lancet Glob Health. 2022 Dec;10(12):e1703-e1704.

BACKGROUND: Influenza increases the risk of cardiovascular events and deaths. We aimed to see whether influenza vaccination reduces death and vascular events in patients with heart failure.
METHODS: We did a pragmatic, randomised, double-blind, placebo-controlled trial in 30 centres (mostly hospitals affliated with universities or a research institute) in ten countries in Asia, the Middle East, and Africa (7 in India, 4 in Philippines, 4 in Nigeria, 6 in China, 1 in Zambia, 2 in Mozambique, 3 in Saudi Arabia, 1 in Kenya, 1 in Uganda, and 1 in Zambia). Participants (aged ≥18 years; 52·1% female; not disaggregated by race or ethnicity) with heart failure (New York Heart Association class II, III, or IV) were randomly assigned (1:1) by a centralised web-based system with block randomisation stratified by site, to receive 0·5 ml intramuscularly once a year for up to 3 years of either inactivated standard dose influenza vaccine or placebo (saline). We excluded people who had received influenza vaccine in 2 of the previous 3 years, and those likely to require valve repair or replacement. Those who administered assigned treatments were not masked and had no further role in the study. Investigators, study coordinators, outcome adjudicators, and participants were masked to group assignment. The first of two co-primary outcomes was a first-event composite for cardiovascular death, non-fatal myocardial infarction, and non-fatal stroke, and the second was a recurrent-events composite for cardiovascular death, non-fatal myocardial infarction, non-fatal stroke, and hospitalisation for heart failure. Outcomes were assessed every 6 months in the intention-to-treat population. Secondary outcomes were all-cause death, cardiovascular death, non-fatal myocardial infarction, non-fatal stroke, all-cause hospitalisation, hospitalisation for heart failure, and pneumonia, both overall and during periods of peak influenza exposure. This study is registered with ClinicalTrials.gov, NCT02762851.
FINDINGS: Between June 2, 2015, and Nov 21, 2021, we enrolled 5129 participants

and randomly assigned (1:1) 2560 (50·0%) to influenza vaccine and 2569 (50·0%) to placebo. The first co-primary outcome occurred in 380 (14·8%) of 2560 participants in the vaccine group and 410 (16·0%) of 2569 participants in the placebo group (hazard ratio [HR] 0·93 [95% CI 0·81-1·07]; p=0·30). The second co-primary outcome occurred in 754 (29·5%) of 2560 participants in the vaccine group and 819 (31·9%) of 2569 participants in the placebo group; HR 0·92 [95% CI 0·84-1·02]; p=0·12). The secondary outcomes of all-cause hospitalisations (HR 0·84 [95% CI 0·74-0·97]; p=0·013) and pneumonia (HR 0·58 [0·42-0·80]; p=0·0006) were significantly reduced in the vaccine group compared with in the placebo group but there was no significant difference between groups for all-cause death, cardiovascular death, non-fatal myocardial infarction, non-fatal stroke, and hospitalisation for heart failure. In a prespecified analysis, in which events were limited to periods of peak influenza circulation, the first co-primary outcome, and the secondary outcomes of all-cause death, cardiovasular death, and pneumonia were significantly lower in the vaccinated group than in the placebo group, whereas the second co-primary outcome and the secondary outcomes of non-fatal myocardial infarction, non-fatal stroke, all-cause hospitalisation, and hospitalisation for heart failure were not significantly lower.
INTERPRETATION: Although the prespecified co-primary outcomes during the entire period of observation were not statistically significant, the reduction during the peak influenza circulating period suggests that there is likely to be a clinical benefit of giving influenza vaccine, given the clear reduction in pneumonia, a moderate reduction in hospitalisations, and a reduction in cardiovascular events and deaths during periods of peak circulation of influenza. Taken in conjunction with previous trials and the observational studies, the collective data suggest benefit.
FUNDING: UK Joint Global Health Trials Scheme and Canadian Institutes for Health Research Foundation.

Copyright © 2022 The Author(s). Published by Elsevier Ltd. This is an Open Access article under the CC BY 4.0 license. Published by Elsevier Ltd.. All rights reserved.

DOI: 10.1016/S2214-109X(22)00432-6
PMID: 36400089 [Indexed for MEDLINE]

Conflict of interest statement: Declaration of interests ML reports grants from the Joint Global Health Trials Scheme of the UK Dept for International Development, UK Medical Research Council (MRC), UK National Institute for Health Research, and the Wellcome Trust) and the Canadian Institutes of Health Research (CIHR); in-kind support from Sanofi Pasteur during the conduct of the study; paid participation on advisory boards for Sanofi Pasteur, Medicago, GlaxoSmithKline, Pfizer, Merck, and Seqirus and on the data safety monitoring board for CanSino Biologics. LMP-V reports grant support from the Wellcome Trust. JZ and YL report grant payments from the Population Health Research Institute to their institution, payment from Bayer, Boehringer Ingelheim, and

Novartis for presentations, and travel support from Bayer to attend the European Society of Cardiology 2019 Congress. TM reports grants from CIHR and the Joint Global Health Scheme paid to McMaster University and Hamilton Health Sciences for the conduct of the study. AR, HD, AnD, KK, FG, AlD, KFA, GY, CM, WA, AAM, VT, PR-M, AG, SIB, and SY declare no competing interests.

267. IJID Reg. 2022 Sep 5;5:44-50. doi: 10.1016/j.ijregi.2022.08.017. eCollection 2022 Dec.

Measles outbreak in Semuto Subcounty, Nakaseke District, Uganda, June-August 2021.

Nsubuga EJ(1), Morukileng J(1), Namayanja J(1), Kadobera D(1)(2), Nsubuga F(3), Kyamwine IB(1)(2), Bulage L(1)(2), Kwesiga B(1)(2), Ario AR(1)(2), Harris JR(4)(5).

Author information:
(1)Uganda Public Health Fellowship Programme, Kampala, Uganda.
(2)Uganda National Institute of Public Health, Kampala, Uganda.
(3)Uganda National Expanded Programme on Immunization, Ministry of Health, Kampala, Uganda.
(4)United States Centers for Disease Control and Prevention, Kampala, Uganda.
(5)Workforce and Institute Development Branch, Division of Global Health Protection, Center for Global Health, Centers for Disease Control and Prevention, Atlanta, GA, USA.

BACKGROUND: Semuto Subcounty reported rubella/measles outbreaks in January 2020 and June-August 2021. This study investigated the outbreak in 2021 to determine the scope, and the factors associated with transmission.
METHODS: A probable case was defined as a resident of Semuto Subcounty with acute onset of fever and a generalized maculopapular rash with either cough/cold or red eyes from 1 June to 31 August 2021. A confirmed case was defined as a probable case with a blood sample positive for measles-specific IgM. A village-matched case-control study was conducted with 30 cases and 122 controls (1:4 ratio). A control was defined as an individual aged 6 months-9 years, sampled at random, with no signs or symptoms of measles from 1 June to 31 August 2021, residing in the same village as the matched case. Adjusted Mantel-Haenszel odds ratios (ORMH) and confidence intervals (CIs) were calculated.
RESULTS: Of the 30 cases (27 probable and three confirmed), 16 (53%) were male. The subcounty attack rate (AR) was 3.2/1000. Children aged 5-9 years were the most affected (AR 5.0/1000). Twenty-two (79%) cases and 116 (97%) controls had ever received measles vaccine (ORMH 0.13, 95% CI 0.03-0.52). Interaction with symptomatic persons at water collection points (ORMH 4.4, 95% CI 1.6-12) and playing at community playgrounds (ORMH 4.2, 95% CI 1.7-11) increased the odds of infection.
CONCLUSIONS: Socializing/congregating at water collection points and community

playgrounds facilitated the transmission of measles in this outbreak.

© 2022 The Author(s).

DOI: 10.1016/j.ijregi.2022.08.017
PMCID: PMC9515593
PMID: 36188443

268. AIDS Behav. 2022 Dec;26(12):3834-3847. doi: 10.1007/s10461-022-03712-7. Epub 2022 Jun 15.

Real-time Feedback to Improve HIV Treatment Adherence in Pregnant and Postpartum Women in Uganda: A Randomized Controlled Trial.

Sabin LL(1), Simmons E(2)(3), Halim N(2), Hamer DH(2)(4), Gifford AL(4)(5)(6), West RL(2), Larson A(2), Bonawitz R(2), Aroda P(7), Banigbe B(2), Holderman AJ(8), Murray L(9), DeSilva MB(10), Gasuza J(7), Mukasa B(7), Messersmith LJ(2).

Author information:
(1)Department of Global Health, Boston University School of Public Health, 801 Massachusetts Avenue, Crosstown Center, 3rd Floor, 02118, Boston, MA, USA. lsabin@bu.edu.
(2)Department of Global Health, Boston University School of Public Health, 801 Massachusetts Avenue, Crosstown Center, 3rd Floor, 02118, Boston, MA, USA.
(3)Carolina Population Center, University of North Carolina at Chapel Hill, 27516, Chapel Hill, NC, USA.
(4)Department of Medicine, Boston University School of Medicine, 02118, Boston, MA, USA.
(5)Department of Health Law, Policy, and Management, Boston University School of Public Health, 02118, Boston, MA, USA.
(6)Center for Healthcare Organization and Implementation Research, VA Boston Healthcare System, 02130, Boston, MA, United States.
(7)Mildmay Uganda, Kampala Road, Kampala, Uganda.
(8)Department of Human Genetics, University of Pittsburgh School of Public Health, 15261, Pittsburgh, PA, USA.
(9)Department of Epidemiology & Biostatistics, Boston University School of Public Health, 02118, Boston, MA, USA.
(10)Center for Excellence in Public Health, University of New England, 04103, Portland, ME, USA.

We assessed an intervention aimed at improving adherence to antiretroviral therapy (ART) among pregnant and postpartum women living with HIV (PPWLH). We randomized 133 pregnant women initiating ART in Uganda to receive text reminders generated by real time-enabled electronic monitors and data-informed counseling through 3 months postpartum (PPM3) or standard care. Intention-to-treat analyses found low adherence levels and no intervention impact. Proportions achieving

≥95% adherence in PPM3 were 16.4% vs. 9.1% (t = -1.14, p = 0.26) in intervention
vs. comparison groups, respectively; 30.9% vs. 29.1% achieved ≥80% adherence.
Additional analyses found significant adherence declines after delivery, and no
effect on disease progression (CD4-cell count, viral load), though treatment
interruptions were significantly fewer in intervention participants.
Per-protocol analyses encompassing participants who used adherence monitors as
designed experienced better outcomes, suggesting potential benefit for some
PPWLH. The study was registered on ClinicalTrials.Gov (NCT02396394).

© 2022. The Author(s).

DOI: 10.1007/s10461-022-03712-7
PMCID: PMC9640413
PMID: 35704124 [Indexed for MEDLINE]

Conflict of interest statement: Anna Larson is currently employed by Merck and
has received past funding from them for vaccine policy work unrelated to this
study. All other authors have no relevant financial or non-financial interests
to disclose. All authors declare that they have no conflicts of interest.

269. Emerg Microbes Infect. 2022 Dec;11(1):1635-1646. doi:
10.1080/22221751.2022.2086072.

Natural history of Sudan ebolavirus infection in rhesus and cynomolgus macaques.

Woolsey C(1)(2), Fears AC(1)(2), Borisevich V(1)(2), Agans KN(1)(2), Dobias
NS(1)(2), Prasad AN(1)(2), Deer DJ(1)(2), Geisbert JB(1)(2), Fenton KA(1)(2),
Geisbert TW(1)(2), Cross RW(1)(2).

Author information:
(1)Department of Microbiology and Immunology, University of Texas Medical
Branch, Galveston, TX, USA.
(2)Galveston National Laboratory, University of Texas Medical Branch, Galveston,
TX, USA.

Due to its high mortality rate and continued re-emergence, Ebolavirus disease
(EVD) continues to pose a serious threat to global health. A group of viruses
within the genus Ebolavirus causes this severe hemorrhagic disease in humans:
Ebola virus (EBOV; species Zaire ebolavirus), Sudan virus (SUDV; species Sudan
ebolavirus), Bundibugyo virus, and Taï Forest virus. EBOV and SUDV are
associated with the highest case fatality rates. While the host response to EBOV
has been comprehensively examined, limited data exists for SUDV infection. For
medical countermeasure testing, well-characterized SUDV nonhuman primate (NHP)
models are thus needed. Here, we describe a natural history study in which
rhesus (N = 11) and cynomolgus macaques (N = 14) were intramuscularly exposed to
a 1000 plaque-forming unit dose of SUDV (Gulu variant). Time-course analyses of

various hematological, pathological, serological, coagulation, and transcriptomic findings are reported. SUDV infection was uniformly lethal in cynomolgus macaques (100% mortality), whereas a single rhesus macaque subject (91% mortality) survived to the study endpoint (median time-to-death of ~8.0 and ~8.5 days in cynomolgus and rhesus macaques, respectively). Infected macaques exhibited hallmark features of human EVD. The early stage was typified by viremia, granulocytosis, lymphopenia, albuminemia, thrombocytopenia, and decreased expression of HLA-class transcripts. At mid-to-late disease, animals developed fever and petechial rashes, and expressed high levels of pro-inflammatory mediators, pro-thrombotic factors, and markers indicative of liver and kidney injury. End-stage disease was characterized by shock and multi-organ failure. In summary, macaques recapitulate human SUDV disease, supporting these models for use in the development of vaccines and therapeutics.

DOI: 10.1080/22221751.2022.2086072
PMCID: PMC9225728
PMID: 35657325 [Indexed for MEDLINE]

Conflict of interest statement: No potential conflict of interest was reported by the author(s).

270. Clin Infect Dis. 2022 Nov 30;75(11):1950-1961. doi: 10.1093/cid/ciac294.

Severe Acute Respiratory Syndrome Coronavirus 2 Infection and Pregnancy in Sub-Saharan Africa: A 6-Country Retrospective Cohort Analysis.

Nachega JB(1)(2)(3)(4), Sam-Agudu NA(5)(6)(7), Machekano RN(8), Rosenthal PJ(9), Schell S(10), de Waard L(10), Bekker A(11), Gachuno OW(12), Kinuthia J(12)(13), Mwongeli N(13), Budhram S(14), Vannevel V(15), Somapillay P(16), Prozesky HW(1), Taljaard J(1), Parker A(1), Agyare E(17), Opoku AB(18), Makarfi AU(19), Abdullahi AM(20), Adirieje C(5), Ishoso DK(21), Pipo MT(22), Tshilanda MB(22), Bongo-Pasi Nswe C(23)(24), Ditekemena J(21), Sigwadhi LN(8), Nyasulu PS(8), Hermans MP(25), Sekikubo M(26), Musoke P(27), Nsereko C(28), Agbeno EK(29), Yeboah MY(19), Umar LW(30), Ntakwinja M(31), Mukwege DM(31), Birindwa EK(32), Mushamuka SZ(32), Smith ER(33), Mills EJ(34), Otshudiema JO(35), Mbala-Kingebeni P(36), Tamfum JM(36), Zumla A(37)(38), Tsegaye A(39), Mteta A(40), Sewankambo NK(41), Suleman F(42), Adejumo P(43), Anderson JR(44), Noormahomed EV(45), Deckelbaum RJ(46), Stringer JSA(47), Mukalay A(48), Taha TE(3), Fowler MG(49), Wasserheit JN(50), Masekela R(51), Mellors JW(52), Siedner MJ(53)(54), Myer L(55), Kengne AP(56), Yotebieng M(57), Mofenson LM(58), Langenegger E(10); AFREhealth Research Collaboration on COVID-19 and Pregnancy.

Author information:
(1)Division of Infectious Diseases, Department of Medicine, Stellenbosch University Faculty of Medicine and Health Sciences, Cape Town, South Africa.
(2)Department of Epidemiology, Infectious Diseases, and Microbiology, and Center

for Global Health, University of Pittsburgh, Pittsburgh, Pennsylvania, USA.
(3)Department of Epidemiology, Johns Hopkins Bloomberg School of Public Health, Baltimore, Maryland, USA.
(4)Department of International Health, Johns Hopkins University, Bloomberg School of Public Health, Baltimore, Maryland, USA.
(5)International Research Center of Excellence, Institute of Human Virology Nigeria, Abuja, Nigeria.
(6)Department of Paediatrics and Child Health, University of Cape Coast School of Medical Sciences, Cape Coast, Ghana.
(7)Institute of Human Virology and Department of Pediatrics, University of Maryland School of Medicine, Baltimore, Maryland, USA.
(8)Division of Epidemiology and Biostatistics, Department of Global Health, Faculty of Medicine and Health Sciences, Stellenbosch University, Cape Town, South Africa.
(9)Department of Medicine, Division of Infectious Diseases, University of California, San Francisco, California, USA.
(10)Department of Obstetrics and Gynecology, Tygerberg Teaching Hospital and Faculty of Medicine and Health Sciences, Stellenbosch University, Cape Town, South Africa.
(11)Department of Paediatrics and Child Health; Faculty of Medicine and Health Sciences, Stellenbosch University, Cape Town, South Africa.
(12)Department of Obstetrics and Gynaecology, University of Nairobi, Nairobi, Kenya.
(13)Department of Research, Department of Reproductive Health, Kenyatta National Hospital, Nairobi, Kenya.
(14)Department of Obstetrics and Gynecology, University of KwaZulu Natal, Durban, South Africa.
(15)Department of Obstetrics and Gynecology, Kalafong Hospital, University of Pretoria, Pretoria, South Africa.
(16)Maternal Foetal Medicine; Steve Biko Hospital, University of Pretoria, Pretoria, South Africa.
(17)Department of Microbiology, School of Medical Sciences, University of Cape Coast and Cape Coast Teaching Hospital, Cape Coast, Ghana.
(18)Department of Obstetrics and Gynaecology, Komfo Anokye Teaching Hospital, Kumasi, Ghana.
(19)Department of Obstetrics and Gynaecology, College of Health Sciences, Ahmadu Bello University and Ahmadu Bello University Teaching Hospital, Zaria, Nigeria.
(20)Department of Medicine, College of Health Sciences, Ahmadu Bello University and Ahmadu Bello University Teaching Hospital, Zaria, Nigeria.
(21)University of Kinshasa School of Medicine, Kinshasa, Democratic Republic of the Congo.
(22)Monkole Hospital Center, Kinshasa, Democratic Republic of the Congo.
(23)Department of Public Health, Centre Interdisciplinaire de Recherche en Ethnopharmacologie, Faculty of Medicine, Université Notre-Dame du Kasayi, Kananga, Democratic Republic of the Congo.
(24)Faculty of Public Health, Université Moderne de Kinkole, Kinshasa, Democratic Republic of the Congo.

(25)Department of Endocrinology and Nutrition, Cliniques Universitaires St-Luc, Brussels, Belgium.
(26)Department of Obstetrics and Gynaecology, School of Medicine, College of Health Sciences, Makerere University, Kampala, Uganda.
(27)Department of Paediatrics and Child Health, School of Medicine, Makerere University, Kampala, Uganda.
(28)Department of Medicine, Entebbe Regional Reference Hospital, Entebbe, Uganda.
(29)Department of Obstetrics and Gynecology, School of Medical Sciences, University of Cape Coast and Cape Coast Teaching Hospital, Cape Coast, Ghana.
(30)Department of Pediatrics, College of Health Sciences, Ahmadu Bello University and Ahmadu Bello Teaching Hospital, Zaria, Nigeria.
(31)Gynaecology and General Surgery, Panzi General Referral Hospital, Bukavu, and Université Evangelique en Afrique (UEA), Bukavu, Democratic Republic of the Congo.
(32)Hôpital Provincial Général de Référence de Bukavu and Faculty of Medicine, Université Catholique de Bukavu (UCB), Bukavu, Democratic Republic of the Congo.
(33)Department of Global Health, Milken Institute School of Public Health, The George Washington University, Washington, DC, USA.
(34)Department of Health Research Evidence and Impact, Faculty of Health Sciences, McMaster University, Hamilton, Canada.
(35)Epidemiological Surveillance Team, COVID-19 Response, Health Emergencies Program, World Health Organization, Kinshasa, Democratic Republic of the Congo.
(36)Department of Medical Microbiology and Virology, Faculty of Medicine, University of Kinshasa, National Institute of Biomedical Research, Kinshasa, Democratic Republic of the Congo.
(37)Division of Infection and Immunity, Department of Infection, Centre for Clinical Microbiology, University College London, London, United Kingdom.
(38)National Institute for Health Research Biomedical Research Centre, University College London Hospitals, London, United Kingdom.
(39)Department of Medical Laboratory Sciences, College of Health Sciences, Addis Ababa University, Addis Ababa, Ethiopia.
(40)Kilimanjaro Christian Medical University College, Moshi, United Republic of Tanzania.
(41)School of Medicine, College of Health Sciences, Makerere University, Kampala, Uganda.
(42)Discipline of Pharmaceutical Sciences, University of KwaZulu-Natal, Durban, South Africa.
(43)Department of Nursing, University of Ibadan, Ibadan, Nigeria.
(44)Department of Obstetrics and Gynecology, Johns Hopkins School of Medicine, Baltimore, Maryland, USA.
(45)Faculty of Medicine, Eduardo Mondlane University, Maputo, Mozambique.
(46)Department of Pediatrics, Columbia University Irving Medical Center, New York, New York, USA.
(47)Department of Obstetrics and Gynecology, University of North Carolina, School of Medicine, Chapel Hill, North Carolina, USA.
(48)Faculty of Medicine, University of Lubumbashi, Lubumbashi, Democratic

Republic of the Congo.
(49)Department of Pathology, Johns Hopkins University School of Medicine, Baltimore, Maryland, USA.
(50)Departments of Global Health and Medicine, Schools of Medicine and Public Health, University of Washington, Seattle, Washington, USA.
(51)Department of Pediatrics and Child Health, School of Clinical Medicine, College of Health Sciences, University of KwaZulu Natal, Durban, South Africa.
(52)Department of Medicine, Division of Infectious Diseases, University of Pittsburgh School of Medicine, Pittsburgh, Pennsylvania, USA.
(53)Harvard Medical School and Massachusetts General Hospital, Boston, Massachusetts, USA.
(54)Mbarara University of Science and Technology, Mbarara, Uganda.
(55)Division of Epidemiology and Biostatistics, School of Public Health and Family Medicine, University of Cape Town, Cape Town, South Africa.
(56)Non-Communicable Diseases Research Unit, South African Medical Research Council, Cape Town, South Africa.
(57)Department of Medicine, Albert Einstein College of Medicine, New York, New York, USA.
(58)Elizabeth Glaser Pediatric AIDS Foundation, Washington, DC, USA.

BACKGROUND: Few data are available on COVID-19 outcomes among pregnant women in sub-Saharan Africa (SSA), where high-risk comorbidities are prevalent. We investigated the impact of pregnancy on SARS-CoV-2 infection and of SARS-CoV-2 infection on pregnancy to generate evidence for health policy and clinical practice.
METHODS: We conducted a 6-country retrospective cohort study among hospitalized women of childbearing age between 1 March 2020 and 31 March 2021. Exposures were (1) pregnancy and (2) a positive SARS-CoV-2 RT-PCR test. The primary outcome for both analyses was intensive care unit (ICU) admission. Secondary outcomes included supplemental oxygen requirement, mechanical ventilation, adverse birth outcomes, and in-hospital mortality. We used log-binomial regression to estimate the effect between pregnancy and SARS-CoV-2 infection. Factors associated with mortality were evaluated using competing-risk proportional subdistribution hazards models.
RESULTS: Our analyses included 1315 hospitalized women: 510 pregnant women with SARS-CoV-2, 403 nonpregnant women with SARS-CoV-2, and 402 pregnant women without SARS-CoV-2 infection. Among women with SARS-CoV-2 infection, pregnancy was associated with increased risk for ICU admission (adjusted risk ratio [aRR]: 2.38; 95% CI: 1.42-4.01), oxygen supplementation (aRR: 1.86; 95% CI: 1.44-2.42), and hazard of in-hospital death (adjusted sub-hazard ratio [aSHR]: 2.00; 95% CI: 1.08-3.70). Among pregnant women, SARS-CoV-2 infection increased the risk of ICU admission (aRR: 2.0; 95% CI: 1.20-3.35), oxygen supplementation (aRR: 1.57; 95% CI: 1.17-2.11), and hazard of in-hospital death (aSHR: 5.03; 95% CI: 1.79-14.13).
CONCLUSIONS: Among hospitalized women in SSA, both SARS-CoV-2 infection and pregnancy independently increased risks of ICU admission, oxygen supplementation, and death. These data support international recommendations to

prioritize COVID-19 vaccination among pregnant women.

© The Author(s) 2022. Published by Oxford University Press on behalf of the Infectious Diseases Society of America.

DOI: 10.1093/cid/ciac294
PMCID: PMC9214158
PMID: 36130257 [Indexed for MEDLINE]

Conflict of interest statement: Potential conflicts of interest. J. B. N. is an infectious disease internist and epidemiologist and Principal Investigator (PI) of NIH/FIC grant numbers 1R25TW011217-01, 1R21TW011706-01, and 1D43TW010937-01A1; and payment to University of Pittsburgh. N. A. S.-A. is a clinician-scientist in Pediatric Infectious Diseases and implementation research; she is supported by the NIH National Institute of Child Health and Human Development (NICHD) grant number R01HD089866; by an NIH/FIC award through the Adolescent HIV Prevention and Treatment Implementation Science Alliance (AHISA) for the Central and West Africa Implementation Science Alliance (CAWISA) (payment to institution); and by NIH/FIC grant number 1D43TW012280-01. F. S., N. K. S., and A. P. are supported as PIs by NIH/FIC grant number 1R25TW011217-01. A. Z. is PIs of the Pan-African Network on Emerging and Re-Emerging Infections (PANDORA-ID-NET; https://www.pandora-id.net) funded by the European Developing Countries Clinical Trial Partnership (EDCTP) and the European Union Horizon 2020 Framework Program for Research and Innovation, paid to institution (University College London, London, UK). R. N. M. reports grants or contracts unrelated to this work, and payment to their institution: NIH/FIC1D43TW010547-01—The African Center for Biostatistical Excellence (ACBE). P. J. R. reports the following grants or contracts unrelated to this work and paid to their institution: 5R01AI075045-12, 5R01AI139179-04, and 5R01AI117001-07. E. R. S. reports a Bill and Melinda Gates Foundation grant for a prospective meta-analysis of COVID-19 in pregnancy, unrelated to this work, and payment to George Washington University. A. B. reports grants or contracts unrelated to this work—NIH: IMPAACT Vice-chair Funding for P1106; and UNITAID: Benefit KIDS funding for PETITE Study. P. A. reports grants or contracts unrelated to this work and paid to the University of Ibadan, Nigeria: NIH/FIC R25 grant number 1R25TW011217-01 to AFREhealth. J. W. N. reports grants paid to the University of Pittsburgh from the NIH to the Pitt-Ohio State Clinical Trials Unit (UM1 AI068636), the University of Pittsburgh Virology Support Laboratory (UM1 AI106701), the I4C Martin Delaney Collaboratory for an HIV Cure (UM1 AI126603), the REACH Martin Delaney Collaboratory for HIV Cure (UM1 AI164565), and from the National Cancer Institute through Leidos contract numbers HHSN261200800001E and 75N91019D00024 USAID; Gilead Sciences, Inc; and Janssen Pharmaceuticals. J. W. N. also reports consulting fees from Gilead Sciences, Inc (Scientific Advisory Board), Accelevir Diagnostics (Consulting Agreement), and Merck (Consulting Agreement); shares from Abound Bio, Inc, share options from Co-Crystal Pharma, Inc, and share options from Infectious Diseases Connect; a consulting agreement with Xi'an Yufan Biotechnologies; and employment with the University of Pittsburgh. M. S.

reports grants or contracts unrelated to this work and paid to their institution (MGH-Boston, MA, USA): NIH/NIA R01AG059504-03 and NIH/NHLBI R01 HL141053-04. A.-P. K. reports research support unrelate to this work paid to their institution (South African Medical Research Council): NIH/FIC 1R21TW011706-01-Dolutegravir, Weight Gain and Metabolic Outcomes in South Africa. L. M. reports consulting fees from the World Health Organization on COVID in pregnancy and mother to child SARS-CoV-2 transmission (this contract is now completed); and payment from Virology Education for a talk on SARS-CoV-2 in pregnancy and possibility of mother-to-child SARS-CoV-2 transmission for continuing education sponsored by Virology Education. M. Y. is PI of the NIH/National Institute of Allergy and Infectious Diseases (NIH/NIAID) grant number 5U01AI096299-13 of the Central Africa-International epidemiology to Evaluate AIDS (CA-IeDEA). M. Y. also reports grants or contracts unrelated to this work and paid to their institution: NIH grant numbers 5U01AI096299 (NIAID), R01HD087993 (NICHD), U54CA254568 (National Cancer Institute), and R01HD105526 (NICHD). All other authors report no potential conflicts. All authors have submitted the ICMJE Form for Disclosure of Potential Conflicts of Interest. Conflicts that the editors consider relevant to the content of the manuscript have been disclosed.

271. Int J Environ Res Public Health. 2022 Nov 19;19(22):15303. doi: 10.3390/ijerph192215303.

Side-Effects following Oxford/AstraZeneca COVID-19 Vaccine in Tororo District, Eastern Uganda: A Cross-Sectional Study.

Onyango J(1), Mukunya D(1)(2), Napyo A(1), Nantale R(3), Makoko BT(1), Matovu JKB(1)(4), Wanume B(1), Okia D(1), Okello F(1), Okware S(5), Olupot-Olupot P(1)(6), Lubaale Y(1).

Author information:
(1)Department of Community and Public Health, Busitema University, Mbale P.O. Box 236, Uganda.
(2)Department of Research, Nikao Medical Center, Kampala P.O. Box 10005, Uganda.
(3)Department of Nursing, Busitema University, Mbale P.O. Box 236, Uganda.
(4)Department of Disease Control and Environmental Health, Makerere University School of Public Health, Kampala P.O. Box 7072, Uganda.
(5)Department of Research, Uganda National Health Research Organizations, Kampala P.O. Box 465, Uganda.
(6)Department of Research, Mbale Clinical Research Institute, Mbale P.O. Box 1966, Uganda.

Effective, safe and proven vaccines would be the most effective strategy against the COVID-19 pandemic but have faced rollout challenges partly due to fear of potential side-effects. We assessed the prevalence, profiles, and predictors of Oxford/AstraZeneca vaccine side-effects in Tororo district of Eastern Uganda. We

conducted telephone interviews with 2204 participants between October 2021 and January 2022. Multivariable logistic regression was conducted to assess factors associated with Oxford/AstraZeneca vaccine side-effects using Stata version 15.0. A total of 603/2204 (27.4%) of the participants experienced one or more side-effects (local, systemic, allergic, and other side-effects). Of these, 253/603 (42.0%) experienced local side-effects, 449/603 (74.5%) experienced systemic side-effects, 11/603 (1.8%) experienced allergic reactions, and 166/603 (27.5%) experienced other side-effects. Ten participants declined to receive the second dose because of side-effects they had experienced after the first dose. Previous infection with COVID-19 (adjusted odds ratio (AOR): 4.3, 95% confidence interval (95% CI): 2.7-7.0), being female (AOR: 1.3, 95% CI: 1.1-1.6) and being a security officer (AOR: 0.4, 95% CI: 0.2-0.6) were associated with side-effects to the Oxford/AstraZeneca vaccine. We recommend campaigns to disseminate correct information about potential side-effects of the Oxford/AstraZeneca vaccine and strengthen surveillance for adverse events following vaccination.

DOI: 10.3390/ijerph192215303
PMCID: PMC9690167
PMID: 36430019 [Indexed for MEDLINE]

Conflict of interest statement: The authors declare no conflict of interest.

272. Ther Adv Infect Dis. 2022 Nov 14;9:20499361221136415. doi: 10.1177/20499361221136415. eCollection 2022 Jan-Dec.

Coronavirus disease-2019 morbidity and mortality among health care workers in Uganda.

Ssetaba LJ(1), Mirembe J(1), Omega J(2), Okot J(3), Kiguli S(1), Nakwagala FN(2), Bongomin F(4).

Author information:
(1)School of Medicine, College of Health Sciences, Makerere University, Kampala, Uganda.
(2)Department of Medicine, Mulago National Referral Hospital, Kampala, Uganda.
(3)Faculty of Medicine, Gulu University, Gulu, Uganda.
(4)Faculty of Medicine, Gulu University, P. O. Box, 166, Gulu, Uganda.

BACKGROUND: Health care workers (HCWs) are at increased risk of acquiring coronavirus disease 2019 (COVID-19). This study aimed to determine and compare the morbidity and mortality rates due to COVID-19 among the HCWs and the general population (non-HCWs).
METHODS: We conducted a retrospective chart review. We accessed electronic database of participants admitted at Mulago National Referral Hospital COVID-19 Treatment Unit (CTU) between March 2020 and September 2021. Participants with missing occupations were excluded.

RESULTS: Of 594 eligible participants, 6.4% (n = 38) were HCWs. Compared with non-HCWs, HCWs were much younger (48 versus 55 years, p = 0.020). The proportion of participants with severe disease (73.7% versus 77.6%, p = 0.442), who had not received COVID-19 vaccine (91.2% versus 94.7%, p = 0.423), mortality rate (44.7% versus 54.8%, p = 0.243) and the median length of hospitalization (6 versus 7 days, p = 0.913) were similar among HCWs and non-HCWs, respectively. A higher proportion of HCWs required oxygen therapy (24.3% versus 9.7%, p < 0.01). At admission, the presence of cough (p = 0.723), breathlessness (p = 0.722), fever (p = 0.19), sore throat (p = 0.133), comorbidities (p = 0.403) and headache (p = 0.162) were similar across groups. Rhinorrhoea was more common among HCWs (34.4% versus 16.6%, p = 0.017). Among HCWs, nurses had the highest morbidity (52.6%) and mortality (58.8%).
CONCLUSION: The morbidity and mortality among HCWs in Uganda were substantial, with a low COVID-19 vaccination rate and a higher requirement for oxygen therapy despite a younger age.

© The Author(s), 2022.

DOI: 10.1177/20499361221136415
PMCID: PMC9666413
PMID: 36406814

Conflict of interest statement: The authors declared no potential conflicts of interest with respect to the research, authorship and/or publication of this article.

273. BMC Womens Health. 2022 Nov 10;22(1):440. doi: 10.1186/s12905-022-02018-z.

Uptake of and adherence to oral pre-exposure prophylaxis among adolescent girls and young women at high risk of HIV-infection in Kampala, Uganda: A qualitative study of experiences, facilitators and barriers.

Kayesu I(1), Mayanja Y(2), Nakirijja C(2), Machira YW(3), Price M(3)(4), Seeley J(2)(5), Siu G(2)(6).

Author information:
(1)Medical Research Council, Uganda Virus Research Institute and London School of Hygiene and Tropical Medicine Uganda Research Unit, P.O. Box 49, Entebbe, Uganda. Ivy.Kayesu@mrcuganda.org.
(2)Medical Research Council, Uganda Virus Research Institute and London School of Hygiene and Tropical Medicine Uganda Research Unit, P.O. Box 49, Entebbe, Uganda.
(3)International AIDS Vaccine Initiative (IAVI), 125 Broad Street, 9th Floor, 10004, New York, NY, USA.
(4)Department of Epidemiology and Biostatistics, University of California, San Francisco (UCSF), 550 16th St, 94158, San Francisco, CA, USA.

(5)Global Health and Development Department, London School of Hygiene and Tropical Medicine, 15-17 Tavistock Place, WC1H 9SH, London, UK.
(6)Child Health and Development Centre, Makerere University, Mulago Hill Road, Kampala, Uganda.

BACKGROUND: There is limited information on factors that influence oral pre-exposure prophylaxis (PrEP) uptake and adherence among adolescent girls and young women (AGYW). We conducted a qualitative methods study to explore experiences, facilitators and barriers of PrEP uptake and adherence to PrEP among AGYW at risk of Human Immunodeficiency Virus (HIV) infection in Kampala, Uganda.
METHODS: This study was nested in a prospective cohort study that offered daily oral PrEP to AGYW. Between April 2019 and October 2020 we conducted in-depth interviews with 26 AGYW aged 14-24 years who had been offered or had been using PrEP for at least 6 months, including PrEP adherers (8), non-adherers (8) and those who had declined PrEP (10). After 12 months, follow-up interviews were conducted with 12 AGYW who had adhered to PrEP and those who had dropped it. Thematic analysis was conducted and data were further examined and categorized into the 5 constructs of the Socio-Ecological Model (SEM).
RESULTS: PrEP uptake and adherence were facilitated by factors including: perceptions that one's own or partner's sexual behaviour was high risk, a negative attitude towards condoms, social support and wanting to maintain a negative HIV status after receiving a negative HIV test result. Good adherence to PrEP was enabled by effective counselling, support tools such as alarms and phone reminders and incentives like free treatment for STIs and other illnesses during study visits. Barriers to uptake included: anxiety about the pill burden, perceptions of being too young for PrEP and fear of being labelled `prostitute' or `HIV positive'. Poor adherence was attributed to doubt over the efficacy of PrEP as a result of beliefs that because HIV was incurable, no medicine could prevent it. Alcohol use, side effects experienced, and mobility all had a negative impact on adherence. The majority of PrEP users reported feeling safe as a result of using PrEP which had both good and negative implications on their sexual behaviour, specifically the number of sexual partners and condom use.
CONCLUSION: Addressing community misconceptions to maximize uptake of PrEP among AGYW is important. Targeted education messages, and counselling to address misconceptions in ways that capture the attention of AGYW in communities are required.

© 2022. The Author(s).

DOI: 10.1186/s12905-022-02018-z
PMCID: PMC9648457
PMID: 36357920 [Indexed for MEDLINE]

Conflict of interest statement: The authors declare that they have no competing interests.

274. BMC Health Serv Res. 2022 Nov 10;22(1):1336. doi: 10.1186/s12913-022-08730-8.

Examining oral pre-exposure prophylaxis (PrEP) literacy among participants in an HIV vaccine trial preparedness cohort study.

Chimukuche RS(1), Kawuma R(2), Mahapa N(3), Mkhwanazi S(4), Singh N(5), Siva S(5), Ruzagira E(6)(7), Seeley J(3)(2)(8); PrEPVacc Study Group.

Collaborators: Gray G, Singh N, Gaffoor Z, Morar N, Sithole T, Woeber K, Siva S, Hwengwere E, Chidawanyika RS, Mahapa N, Khanyile P, Jani I, Viegas E, Remane I, Bule O, Nhacule E, Ramgi P, Chissumba R, Namalango E, Manganhe Y, Massingue C, Capitine I, Ribeiro J, Maganga L, William W, Kapesa E, Danstan E, Pamba D, Kway MMA, Kisinda A, Njovu L, Sudi L, Kunambi R, Aboud S, Munseri P, Lyamuya E, Msafiri F, Joachim A, Tarimo E, Nagu DFT, Buma D, Bakari M, Kaleebu P, Kibengo FM, Kakande A, Serwanga J, Kawuma R, Holmes CH, Kansiime S, Ruzagira E, Seeley J, Kusemererwa S, Masawi S, Basajja V, Vudriko T, Hughes P, Nabukenya S, Mutonyi G, Nakiboneka R, Mugaba S, Weber J, Kingsley C, Miller T, McCormack S, Crook A, Dunn D, Bern H, Sy A, Brodnicki L, Joseph S, Wenden C, Chinyenze K, Musau J, Matsoso M, Amondi M, Chetty P, Gumbe A, Pantaleo G, Ding S, Nilsson C, Kroidl A, Fox J, Doncel G, Matthews A, Rooney J, Lee C, Robb M.

Author information:
(1)Africa Health Research Institute, KwaZulu-Natal, South Africa. Rujeko.Chidawanyika@ahri.org.
(2)Social Aspects of Health Programme, MRC/UVRI and LSHTM Uganda Research Unit, Entebbe, Uganda.
(3)Africa Health Research Institute, KwaZulu-Natal, South Africa.
(4)Gender and Health Research Unit, South African Medical Research Council, Durban, South Africa.
(5)HIV and Other Infectious Diseases Research Unit, South African Medical Research Council, Durban, South Africa.
(6)HIV Epidemiology and Interventions Programme, MRC/UVRI and LSHTM Uganda Research Unit, Entebbe, Uganda.
(7)Faculty of Epidemiology and Population Health, London School of Hygiene and Tropical Medicine, London, UK.
(8)Department of Global Health and Development, London School of Hygiene and Tropical Medicine, London, UK.

BACKGROUND: PrEP literacy is influenced by many factors including the types of information available and how it is interpreted. The level of PrEP literacy may influence acceptability and uptake.
METHODS: We conducted 25 in-depth interviews in a HIV vaccine trial preparedness cohort study. We explored what participants knew about PrEP, sources of PrEP knowledge and how much they know about PrEP. We used the framework approach to generate themes for analysis guided by the Social Ecological Model and examined levels of PrEP literacy using the individual and interpersonal constructs of the

SEM.
RESULTS: We found that PrEP awareness is strongly influenced by external factors such as social media and how much participants know about HIV treatment and prevention in the local community. However, while participants highlighted the importance of the internet/social media as a source of information about PrEP they talked of low PrEP literacy in their communities. Participants indicated that their own knowledge came as a result of joining the HIV vaccine trial preparedness study. However, some expressed doubts about the effectiveness of the drug and worried about side effects. Participants commented that at the community level PrEP was associated with being sexually active, because it was used to prevent the sexual transmission of HIV. As a result, some participants commented that one could feel judged by the health workers for asking for PrEP at health facilities in the community.
CONCLUSION: The information collected in this study provided an understanding of the different layers of influence around individuals that are important to address to improve PrEP acceptability and uptake. Our findings can inform strategies to address the barriers to PrEP uptake, particularly at structural and community levels.
TRIAL REGISTRATION: https://clinicaltrials.gov/ct2/show/NCT04066881.

© 2022. The Author(s).

DOI: 10.1186/s12913-022-08730-8
PMCID: PMC9649006
PMID: 36357877 [Indexed for MEDLINE]

Conflict of interest statement: N/A

275. BMJ Open. 2022 Nov 8;12(11):e060786. doi: 10.1136/bmjopen-2022-060786.

Indicators of optimal diabetes care and burden of diabetes complications in Africa: a systematic review and meta-analysis.

Kibirige D(1), Chamba N(2)(3), Andia-Biraro I(4)(5), Kilonzo K(2)(3), Laizer SN(3), Sekitoleko I(6), Kyazze AP(4), Ninsiima S(7), Ssekamatte P(7), Bongomin F(8), Mrema LE(9), Olomi W(10), Mbunda TD(9), Ntinginya NE(9), Sabi I(11), Sharples K(12), Hill P(12), Te Brake L(13), VandeMaat J(14), vanCrevel R(15)(16), Critchley JA(17); PROTID consortium.

Author information:
(1)Department of Medicine, Lubaga Hospital, Kampala, Uganda kibirigedavis@gmail.com.
(2)Department of Internal Medicine, Kilimanjaro Christian Medical Centre, Moshi, Kilimanjaro, Tanzania.
(3)Department of Medicine, Kilimanjaro Christian Medical University College, Moshi, Kilimanjaro, Tanzania.

(4)Department of Internal Medicine, Makerere University College of Health Sciences, Kampala, Uganda.
(5)Department of Immunomudation and Vaccines, MRC/UVRI and LSHTM Uganda Research Unit, Entebbe, Uganda.
(6)Non-Communicable Diseases Program, Medical Research Council/Uganda Virus Research Institute & London School of Hygiene and Tropical Medicine Uganda Research Unit, Entebbe, Uganda.
(7)Department of Immunology, Makerere University College of Health Sciences, Kampala, Uganda.
(8)Department of Medical Microbiology and Immunology, Faculty of Medicine, Gulu University, Gulu, Uganda.
(9)Department of Medicine, NIMR-Mbeya Medical Research Programme, Mbeya, Mbeya, Tanzania.
(10)Department of Medical Statistics, NIMR-Mbeya Medical Research Programme, Mbeya, Mbeya, Tanzania.
(11)Department of Paediatrics and Child Health, NIMR-Mbeya Medical Research Programme, Mbeya, Tanzania.
(12)Centre for International Health, University of Otago, Dunedin, New Zealand.
(13)Department of Pharmacology, Radboud University Nijmegen, Nijmegen, Gelderland, The Netherlands.
(14)Department of Medicine, Radboud University Nijmegen, Nijmegen, Gelderland, The Netherlands.
(15)Department of Internal Medicine, Radboud University Nijmegen, Nijmegen, Gelderland, The Netherlands.
(16)University of Oxford Centre for Tropical Medicine and Global Health, Oxford, Oxfordshire, UK.
(17)Population Health Research Institute, St George's University of London, London, UK.

OBJECTIVE: Contemporary data on the attainment of optimal diabetes treatment goals and the burden of diabetes complications in adult populations with type 2 diabetes in Africa are lacking. We aimed to document the current status of attainment of three key indicators of optimal diabetes care and the prevalence of five diabetes complications in adult African populations with type 2 diabetes.
METHODS: We systematically searched Embase, PubMed and the Cochrane library for published studies from January 2000 to December 2020. Included studies reported any information on the proportion of attainment of optimal glycated haemoglobin (HbA1c), blood pressure (BP) and low-density lipoprotein cholesterol (LDLC) goals and/or prevalence of five diabetes complications (diabetic peripheral neuropathy, retinopathy, nephropathy, foot ulcers and peripheral arterial disease). Random effect model meta-analysis was performed to determine the pooled proportion of attainment of the three treatment goals and the prevalence of five diabetes complications.
RESULTS: In total, 109 studies with a total of 63 890 participants (53.3% being females) were included in the meta-analysis. Most of the studies were conducted in Eastern African countries (n=44, 40.4%). The pooled proportion of attainment

of an optimal HbA1c, BP and LDLC goal was 27% (95% CI 24 to 30, I2=94.7%), 38% (95% CI 30 to 46, I2=98.7%) and 42% (95% CI 32 to 52, I2=97.4%), respectively. The pooled prevalence of diabetic peripheral neuropathy, retinopathy, diabetic nephropathy, peripheral arterial disease and foot ulcers was 38% (95% CI 31 to 45, I2=98.2%), 32% (95% CI 28 to 36, I2=98%), 31% (95% CI 22 to 41, I2=99.3%), 19% (95% CI 12 to 25, I2=98.1%) and 11% (95% CI 9 to 14, I2=97.4%), respectively.
CONCLUSION: Attainment of optimal diabetes treatment goals, especially HbA1c, in adult patients with type 2 diabetes in Africa remains a challenge. Diabetes complications, especially diabetic peripheral neuropathy and retinopathy, are highly prevalent in adult populations with type 2 diabetes in Africa.

© Author(s) (or their employer(s)) 2022. Re-use permitted under CC BY-NC. No commercial re-use. See rights and permissions. Published by BMJ.

DOI: 10.1136/bmjopen-2022-060786
PMCID: PMC9644326
PMID: 36351737 [Indexed for MEDLINE]

Conflict of interest statement: Competing interests: None declared.

276. Nat Rev Dis Primers. 2022 Nov 3;8(1):71. doi: 10.1038/s41572-022-00399-x.

Asplenia and spleen hypofunction.

Lenti MV(1)(2), Luu S(3)(4), Carsetti R(5), Osier F(6)(7), Ogwang R(6)(8), Nnodu OE(9), Wiedermann U(10), Spencer J(11), Locatelli F(12), Corazza GR(1)(2), Di Sabatino A(13)(14).

Author information:
(1)Department of Internal Medicine and Medical Therapeutics, University of Pavia, Pavia, Italy.
(2)First Department of Internal Medicine, IRCCS San Matteo Hospital Foundation, Pavia, Italy.
(3)Monash Infectious Diseases, Monash Health, Clayton, Victoria, Australia.
(4)Australian Centre for Blood Diseases, Monash University, Clayton, Victoria, Australia.
(5)B cell Unit, Immunology Research Area, IRCCS Bambino Gesù Children's Hospital, Rome, Italy.
(6)KEMRI-Wellcome Trust Research Programme, Centre for Geographic Medicine Research, Coast, Kilifi, Kenya.
(7)Department of Life Sciences, Imperial College London, London, UK.
(8)Centre of Tropical Neuroscience, Kitgum, Uganda.
(9)Department of Haematology and Blood Transfusion, College of Health Sciences, University of Abuja, Abuja, Nigeria.
(10)Institute of Specific Prophylaxis and Tropical Medicine, Center of

Pathophysiology, Infectiology and Immunology, Medical University of Vienna, Vienna, Austria.
(11)Peter Gorer Department of Immunology, School of Immunology and Microbial Sciences, King's College London, London, UK.
(12)Department of Paediatric Haematology/Oncology and Cell and Gene Therapy, IRCCS Bambino Gesù Children's Hospital, Catholic University of the Sacred Heart, Rome, Italy.
(13)Department of Internal Medicine and Medical Therapeutics, University of Pavia, Pavia, Italy. a.disabatino@smatteo.pv.it.
(14)First Department of Internal Medicine, IRCCS San Matteo Hospital Foundation, Pavia, Italy. a.disabatino@smatteo.pv.it.

Asplenia (the congenital or acquired absence of the spleen) and hyposplenism (defective spleen function) are common causes of morbidity and mortality. The spleen is a secondary lymphoid organ that is responsible for the regulation of immune responses and blood filtration. Hence, asplenia or hyposplenism increases susceptibility to severe and invasive infections, especially those sustained by encapsulated bacteria (namely, Neisseria meningitidis, Streptococcus pneumoniae, Haemophilus influenzae type b). Asplenia is predominantly due to splenectomy for either traumatic events or oncohaematological conditions. Hyposplenism can be caused by several conditions, including haematological, infectious, autoimmune and gastrointestinal disorders. Anatomical disruption of the spleen and depletion of immune cells, especially IgM memory B cells, seem to be predominantly responsible for the clinical manifestations. Early recognition of hyposplenism and proper management of asplenia are warranted to prevent overwhelming post-splenectomy infections through vaccination and antibiotic prophylaxis. Although recommendations are available, the implementation of vaccination strategies, including more effective and immunogenic vaccines, is needed. Additionally, screening programmes for early detection of hyposplenism in high-risk patients and improvement of patient education are warranted.

© 2022. Springer Nature Limited.

DOI: 10.1038/s41572-022-00399-x
PMID: 36329079 [Indexed for MEDLINE]

277. PLoS One. 2022 Nov 3;17(11):e0275927. doi: 10.1371/journal.pone.0275927. eCollection 2022.

Optimization and validation of an ELISA assay for the determination of antibody responses to CN54gp140 and AIDSVAX BE for use in the Phase IIb PrEPVacc vaccine trial.

Gombe B(1)(2), Streatfield C(3), Leal L(3)(4), Opio S(1)(2), Joseph S(3), Weber J(5), Hare J(3)(6), Kaleebu P(1)(2)(7), Serwanga J(1)(2).

Author information:
(1)MRC/UVRI and LSHTM Uganda Research Unit, Entebbe, Uganda.
(2)Uganda Virus Research Institute, Entebbe, Uganda.
(3)IAVI Human Immunology Laboratory, Imperial College, London, United Kingdom.
(4)AIDS and HIV Infection Research Group, IDIBAPS, Barcelona, Spain.
(5)Faculty of Medicine, Imperial College, London, United Kingdom.
(6)IAVI, New York, New York, United States of America.
(7)London School of Hygiene and Tropical Medicine (LSHTM), London, United Kingdom.

PrEPVacc is an international, multi-centre, double-blind vaccine study comparing experimental combination vaccine regimens including DNA/AIDSVAX BE and DNA/CN54gp140 with placebo control. Simultaneously, daily oral PrEP is compared for efficacy against daily Truvada in the context of the current PrEP availability situation at the study sites. An important clinical trial outcome is the accurate measurement of in vivo antibody titer induced through vaccination. Here we report the validation of two ELISAs for CN54gp140 and AIDSVAX BE at Uganda Virus Research Institute that demonstrates precision, specificity, and robustness for assessing the reciprocal antibody end point titer in human serum. This is a critical endpoint for determining whether vaccination can provide any protection against HIV in populations at risk of acquiring HIV.

DOI: 10.1371/journal.pone.0275927
PMCID: PMC9632806
PMID: 36327320 [Indexed for MEDLINE]

Conflict of interest statement: The authors have declared that no competing interests exist.

278. Emerg Infect Dis. 2022 Nov;28(11):2214-2225. doi: 10.3201/eid2811.221041. Epub 2022 Oct 11.

Coronavirus Antibody Responses before COVID-19 Pandemic, Africa and Thailand.

Li Y, Merbah M, Wollen-Roberts S, Beckman B, Mdluli T, Swafford I, Mayer SV, King J, Corbitt C, Currier JR, Liu H, Esber A, Pinyakorn S, Parikh A, Francisco LV, Phanuphak N, Maswai J, Owuoth J, Kibuuka H, Iroezindu M, Bahemana E, Vasan S, Ake JA, Modjarrad K, Gromowski G, Paquin-Proulx D, Rolland M.

Prior immune responses to coronaviruses might affect human SARS-CoV-2 response. We screened 2,565 serum and plasma samples collected from 2013 through early 2020, before the COVID-19 pandemic began, from 2,250 persons in 4 countries in Africa (Kenya, Nigeria, Tanzania, and Uganda) and in Thailand, including persons living with HIV-1. We detected IgG responses to SARS-CoV-2 spike (S) subunit 2 protein in 1.8% of participants. Profiling against 23 coronavirus antigens

revealed that responses to S, subunit 2, or subunit 1 proteins were significantly more frequent than responses to the receptor-binding domain, S-Trimer, or nucleocapsid proteins (p<0.0001). We observed similar responses in persons with or without HIV-1. Among all coronavirus antigens tested, SARS-CoV-2, SARS-CoV-1, and Middle East respiratory syndrome coronavirus antibody responses were much higher in participants from Africa than in participants from Thailand (p<0.01). We noted less pronounced differences for endemic coronaviruses. Serosurveys could affect vaccine and monoclonal antibody distribution across global populations.

DOI: 10.3201/eid2811.221041
PMCID: PMC9622245
PMID: 36220131 [Indexed for MEDLINE]

279. Lancet Gastroenterol Hepatol. 2022 Nov;7(11):1036-1048. doi: 10.1016/S2468-1253(22)00041-3. Epub 2022 Jul 8.

Hepatocellular carcinoma: measures to improve the outlook in sub-Saharan Africa.

Spearman CW(1), Dusheiko G(2), Jonas E(3), Abdo A(4), Afihene M(5), Cunha L(6), Desalegn H(7), Kassianides C(8), Katsidzira L(9), Kramvis A(10), Lam P(11), Lesi OA(12), Micah EA(13), Musabeyezu E(14), Ndow G(15), Nnabuchi CV(16), Ocama P(17), Okeke E(18), Rwegasha J(19), Shewaye AB(20), Some FF(21), Tzeuton C(22), Sonderup MW(23); Gastroenterology and Hepatology Association of sub-Saharan Africa (GHASSA).

Author information:
(1)Division of Hepatology, Faculty of Health Sciences, University of Cape Town, Cape Town, South Africa; Department of Medicine, Faculty of Health Sciences, University of Cape Town, Cape Town, South Africa. Electronic address: wendy.spearman@uct.ac.za.
(2)University College London Medical School, London, UK; Kings College Hospital, London, UK.
(3)Surgical Gastroenterology Unit, Division of General Surgery, Faculty of Health Sciences, University of Cape Town, Cape Town, South Africa.
(4)National Centre for Gastrointestinal and Liver Disease, Ibn Sina Hospital, Alamarat, Khartoum, Sudan.
(5)Department of Medicine, College of Health Sciences, Kwame Nkrumah University of Science and Technology, Kumasi, Ghana.
(6)Gastroenterology Unit, Maputo Private Hospital, Maputo, Mozambique.
(7)Department of Internal Medicine, St Paul's Hospital Millennium Medical College, Addis Ababa, Ethiopia.
(8)Department of Medicine, Faculty of Health Sciences, University of Cape Town, Cape Town, South Africa.
(9)Internal Medicine Unit, Faculty of Medicine and Health Sciences, University of Zimbabwe, Harare, Zimbabwe.

(10)Hepatitis Virus Diversity Research Unit, Department of Internal Medicine, School of Clinical Medicine, University of the Witwatersrand, Johannesburg, South Africa.
(11)Clinique Darné, Curepipe, Mauritius.
(12)Gastroenterology and Hepatology Unit, College of Medicine, University of Lagos, Lagos, Nigeria.
(13)Department of Medicine, Komfo Anokye Teaching Hospital, Kumasi, Ghana.
(14)Department of Medicine, King Faisal Hospital, Kigali, Rwanda.
(15)Disease Control and Elimination Theme, MRC Unit The Gambia at the London School of Tropical Medicine, London, UK; Department of Metabolism, Digestion and Reproduction, Imperial College London, London, UK.
(16)Asokoro District Hospital, Nile University Teaching Hospital, Abuja, Nigeria.
(17)School of Medicine, Makerere University College of Health Sciences, Kampala, Uganda.
(18)Gastroenterology and Hepatology Unit, Department of Internal Medicine, College of Health Sciences, University of Jos, Jos University Teaching Hospital, Jos, Nigeria.
(19)Gastroenterology Training Centre, Department of Internal Medicine, Muhimbili National Hospital, Dar Es Salaam, Tanzania.
(20)Division of Gastroenterology and Hepatology, Department of Internal Medicine, College of Health Sciences, Addis Ababa University, Addis Ababa, Ethiopia.
(21)Department of Medicine, School of Medicine, College of Health Sciences, Moi University, Eldoret, Kenya.
(22)Faculty of Medicine and Pharmaceutical Sciences of Douala, University of Douala, Douala, Cameroon.
(23)Division of Hepatology, Faculty of Health Sciences, University of Cape Town, Cape Town, South Africa; Department of Medicine, Faculty of Health Sciences, University of Cape Town, Cape Town, South Africa.

Comment in
Lancet Gastroenterol Hepatol. 2023 Jan;8(1):13-14.

Hepatocellular carcinoma is a leading public health concern in sub-Saharan Africa, and it is most prevalent in young adults (median 45 years [IQR 35-57]). Overall, outcomes are poor, with a median survival of 2·5 months after presentation. Major risk factors for hepatocellular carcinoma are hepatitis B virus (HBV), hepatitis C virus, aflatoxin B1 exposure, and alcohol consumption, with metabolic dysfunction-associated fatty liver disease slowly emerging as a risk factor over the past few years. Crucially, these risk factors are preventable and manageable with effective implementation of the HBV birth-dose vaccination, treatment of chronic viral hepatitis, provision of harm reduction services, and by decreasing aflatoxin B1 exposure and harmful alcohol consumption. Primary prevention is central to the management of hepatocellular carcinoma, especially in poorly resourced environments. Effective screening and surveillance programmes with recall policies need to be implemented, because

detection and curative management of hepatocellular carcinoma is possible if it is detected at an early stage, even in countries with minimal resources, with appropriate upskilling of medical personnel. The establishment of centres of excellence with advanced diagnostic and therapeutic capabilities within countries should improve hepatocellular carcinoma outcomes and assist in driving the implementation of much needed systematic data systems focused on hepatocellular carcinoma to establish the accurate burden in sub-Saharan Africa. Such data would support the public health importance of hepatocellular carcinoma and provide a strong basis for advocacy, programme development, resource allocation, and monitoring of progress in reducing mortality.

Copyright © 2022 Elsevier Ltd. All rights reserved.

DOI: 10.1016/S2468-1253(22)00041-3
PMID: 35810766 [Indexed for MEDLINE]

Conflict of interest statement: Declaration of interests GD reports grants from Abbott Diagnostics for hepatitis B biomarker research; speaker honoraria from Cepheid and Clinical Care Options; participation on data safety monitoring boards for Aligos, Arbutus, Enanta, Gilead, GlaxoSmithKline, and Janssen; participation on advisory boards for Aligos, Antios, Arbutus, and Gilead; previously being medical director for Skipton Fund and research appraiser for Singapore National Medical Research Council. CK is chairman of the Gastroenterology Foundation of sub-Saharan Africa and the Gastroenterology and Hepatology Association of sub-Saharan Africa. MWS received a speaker honorarium from Roche for a cancer meeting in Accra, Ghana, in October, 2021. PO reports US National Institutes of Health (NIH) grants for HIV and hepatocellular carcinoma in Africa: The H2A consortium (NIH fund U54), and Clinical and Immunological Impact of Schistosoma mansoni infection and treatment on the course of chronic HBV in Uganda (grant number 1R01AI150523-01). No NIH funding and no other funding was received for the writing of this manuscript. All other authors declare no competing interests.

280. Vaccines (Basel). 2022 Oct 28;10(11):1816. doi: 10.3390/vaccines10111816.

Current Advances in Zika Vaccine Development.

Wang Y(1), Ling L(1), Zhang Z(1), Marin-Lopez A(2).

Author information:
(1)Department of Inspection and Quarantine Technology Communication, Shanghai Customs College, Shanghai 201204, China.
(2)Section of Infectious Diseases, Department of Internal Medicine, Yale University School of Medicine, New Haven, CT 06420, USA.

Zika virus (ZIKV), an emerging arthropod-borne flavivirus, was first isolated in

Uganda in 1947 from monkeys and first detected in humans in Nigeria in 1952; it has been associated with a dramatic burden worldwide. Since then, interventions to reduce the burden of ZIKV infection have been mainly restricted to mosquito control, which in the end proved to be insufficient by itself. Hence, the situation prompted scientists to increase research on antivirals and vaccines against the virus. These efforts are still ongoing as the pathogenesis and immune evasion mechanisms of ZIKV have not yet been fully elucidated. Understanding the viral disease mechanism will provide a better landscape to develop prophylactic and therapeutic strategies against ZIKV. Currently, no specific vaccines or drugs have been approved for ZIKV. However, some are undergoing clinical trials. Notably, different platforms have been evaluated for the design of vaccines, including DNA, mRNA, viral vectors, virus-like particles (VLPs), inactivated virus, live attenuated virus, peptide and protein-based vaccines, passive immunizations by using monoclonal antibodies (MAbs), and vaccines that target vector-derived antigens. These vaccines have been shown to induce specific humoral and cellular immune responses and reduce viremia and viral RNA titers, both in vitro and in vivo. This review provides a comprehensive summary of current advancements in the development of vaccines against Zika virus.

DOI: 10.3390/vaccines10111816
PMCID: PMC9694033
PMID: 36366325

Conflict of interest statement: The authors declare no conflict of interest.

281. Science. 2022 Oct 28;378(6618):340-341. doi: 10.1126/science.adf4959. Epub 2022 Oct 27.

Forgotten Ebola vaccine could help in outbreak.

Cohen J.

Merck unearths a frozen batch of an experimental vaccine it made years ago.

DOI: 10.1126/science.adf4959
PMID: 36302017 [Indexed for MEDLINE]

282. Vaccines (Basel). 2022 Oct 25;10(11):1794. doi: 10.3390/vaccines10111794.

An Overview of Rift Valley Fever Vaccine Development Strategies.

Kitandwe PK(1), McKay PF(2), Kaleebu P(1), Shattock RJ(2).

Author information:

(1)MRC/UVRI & LSHTM Uganda Research Unit, Entebbe 31410, Uganda.
(2)Department of Infectious Diseases, Division of Medicine, Imperial College London, Norfolk Place, London W2 1PG, UK.

Rift Valley fever (RVF) is a mosquito-borne viral zoonosis that causes high fetal and neonatal mortality in ruminants and a mild to fatal hemorrhagic fever in humans. There are no licensed RVF vaccines for human use while for livestock, commercially available vaccines are all either live attenuated or inactivated and have undesirable characteristics. The live attenuated RVF vaccines are associated with teratogenicity and residual virulence in ruminants while the inactivated ones require multiple immunisations to induce and maintain protective immunity. Additionally, nearly all licensed RVF vaccines lack the differentiating infected from vaccinated animals (DIVA) property making them inappropriate for use in RVF nonendemic countries. To address these limitations, novel DIVA-compatible RVF vaccines with better safety and efficacy than the licensed ones are being developed, aided fundamentally by a better understanding of the molecular biology of the RVF virus and advancements in recombinant DNA technology. For some of these candidate RVF vaccines, sterilizing immunity has been demonstrated in the discovery/feasibility phase with minimal adverse effects. This review highlights the progress made to date in RVF vaccine research and development and discusses the outstanding research gaps.

DOI: 10.3390/vaccines10111794
PMCID: PMC9697312
PMID: 36366303

Conflict of interest statement: The authors declare no conflict of interest.

283. Hepat Med. 2022 Oct 19;14:163-172. doi: 10.2147/HMER.S381809. eCollection 2022.

Prevalence and Factors Associated with Hepatitis B Infection Among Outpatient Adults in South-Western Uganda.

Rugaatwa Ndibarema E(1)(2)(3), Olum R(4), Ayebare D(1), Kabakyenga J(1).

Author information:
(1)Mbarara University of Science and Technology, Mbarara, Uganda.
(2)Mother Kevin Postgraduate Medical School / St. Francis Hospital Nsambya, Uganda Martyrs University, Kampala, Uganda.
(3)Mbarara Regional Referral Hospital, Mbarara, Uganda.
(4)St Francis Hospital Nsambya, Kampala, Uganda.

BACKGROUND: About 2 billion people in the world are exposed to hepatitis B virus. Africa contributes 25% of the global Hepatitis B burden and prevalence in Uganda is 4.3%. Routine testing to establish the burden, improve prevention and control through early diagnosis and management are rare in hospital settings. We

aimed at establishing the prevalence and factors associated with hepatitis B infection among adults attending outpatient clinic at Mbarara Regional Referral Hospital (MRRH) in Uganda.
METHODS: A hospital-based cross-sectional study was conducted among outpatients attending MRRH. Consecutive sampling method was used to recruit participants. Interviewer-administered questionnaires were used to collect data. Blood samples were collected to test HBsAg. Summary statistics were used to describe the socio-demographic characteristics of study participants and the proportion of Serostatus for hepatitis B infection. Bivariate followed by multivariate logistic regression analysis was conducted to assess the factors associated with hepatitis B infections. All independent variables with p-values <0.2 were entered into a multivariate model to adjust for confounding. A p value <0.05 was considered statistically significant.
RESULTS: A total of 400 participants were recruited. 64.5% were females (n=258) and aged 18-29 years (48.5%, n=193). Overall, 22(5.5%) participants were found to be seropositive for hepatitis B infection. Residing in Kiruhura district (AOR = 11.9, 95% CI: 2.40-59.25, p<0.003) was significantly associated with hepatitis B infection while being female (AOR = 0.3, 95% CI: 0.11-0.88, p<0.018) was protective.
CONCLUSION: Prevalence of hepatitis B infection among adult patients attending outpatient clinic at MRRH was 5.5% with male gender and residing in Kiruhura district as factors associated with high prevalence of hepatitis B infection. Ministry of Health should scale up hepatitis B routine testing and treatment at MRRH and its catchment area, with special control programs such as screening, vaccination, and sensitization involving men.

© 2022 Rugaatwa Ndibarema et al.

DOI: 10.2147/HMER.S381809
PMCID: PMC9592731
PMID: 36304917

Conflict of interest statement: The authors declare no conflicts of interest in this work.

284. PLoS One. 2022 Oct 13;17(10):e0270518. doi: 10.1371/journal.pone.0270518. eCollection 2022.

Breaking institutional barriers to enhance women's participation in and benefit from the Peste des Petits Ruminants and Newcastle Disease vaccine value chains for Sembabule district of Uganda.

Bikaako W(1), Kabahango P(2), Mugabi K(3), Yawe A(1), Stallon K(4), Kyewalabye E(2), Tukahirwa L(2), Kusiimakwe D(2), Stanley M(5), Miller B(6), Mugisha A(2), Rosenbaum MH(5), Amuguni H(5).

Author information:
(1)Africa One Health University Network (AFROHUN), Kampala, Uganda.
(2)College of Veterinary Medicine, Animal Resources and Bio-security, Makerere University, Kampala, Uganda.
(3)Department of Animal Health, Ministry of Agriculture, Animal Industry and Fisheries, Kampala, Uganda.
(4)Uganda Martyrs University, Kampala, Uganda.
(5)Cummings School of Veterinary Medicine, Tufts University, North Grafton, Massachusetts, United States of America.
(6)Miller Consulting Inc, Little Rock, Arkansas, United States of America.

This paper describes the institutional context that shapes the visibility and positioning of women along the Peste des Petits Ruminants (PPR) and Newcastle Disease (ND) vaccine value chains for Sembabule district of Uganda. It examines the institutional barriers and opportunities that affect women's empowerment derived from inclusion of women in the decision-making processes along the livestock vaccine value chain (LVVC) and that can support viable women-centered and owned enterprises, at the vaccine development, delivery, distribution and use level. Qualitative data analysis tools such as focus group discussions, focus meals, jar voices and key informant interviews were used. Using outcome mapping, a stakeholder analysis of the critical partners in the PPR and ND value chain was done involving the regulators, vaccine manufacturers, importers, distributors, agrovets, public and private veterinary service deliverers, local leaders, women groups, and farmers. The study concluded that training related to gender equality and livestock vaccines, infrastructural and technical support to the poultry and goat women and men farmers and other chain actors are inadequate in themselves to increase vaccine adoption and improve livestock productivity in Sembabule district. Strategies that promote gender-transformative collaborative efforts among the LVVC actors and build viable gender-transformed women groups and networks are critical to increase women's participation in and benefit from the livestock vaccine value chain.

DOI: 10.1371/journal.pone.0270518
PMCID: PMC9560069
PMID: 36227838 [Indexed for MEDLINE]

Conflict of interest statement: The authors have declared that no competing interests exist.

285. BMC Med. 2022 Oct 12;20(1):344. doi: 10.1186/s12916-022-02543-6.

Characterising social contacts under COVID-19 control measures in Africa.

Dobreva Z(1), Gimma A(2), Rohan H(3), Djoudalbaye B(4), Tshangela A(4), Jarvis CI(2), van Zandvoort K(2), Quaife M(5).

Author information:
(1)Faculty of Public Health and Policy, London School of Hygiene and Tropical Medicine, London, UK. zdobreva@who.int.
(2)Faculty of Epidemiology and Population Health, London School of Hygiene and Tropical Medicine, London, UK.
(3)UK Public Health Rapid Support Team, London School of Hygiene and Tropical Medicine, London, UK.
(4)Africa Centres for Disease Control and Prevention, Addis Ababa, Ethiopia.
(5)Faculty of Public Health and Policy, London School of Hygiene and Tropical Medicine, London, UK.

BACKGROUND: Early in the COVID-19 pandemic, countries adopted non-pharmaceutical interventions (NPIs) such as lockdowns to limit SARS-CoV-2 transmission. Social contact studies help measure the effectiveness of NPIs and estimate parameters for modelling SARS-CoV-2 transmission. However, few contact studies have been conducted in Africa.
METHODS: We analysed nationally representative cross-sectional survey data from 19 African Union Member States, collected by the Partnership for Evidence-based Responses to COVID-19 (PERC) via telephone interviews at two time points (August 2020 and February 2021). Adult respondents reported contacts made in the previous day by age group, demographic characteristics, and their attitudes towards COVID-19. We described mean and median contacts across these characteristics and related contacts to Google Mobility reports and the Oxford Government Response Stringency Index for each country at the two time points.
RESULTS: Mean reported contacts varied across countries with the lowest reported in Ethiopia (9, SD=16, median = 4, IQR = 8) in August 2020 and the highest in Sudan (50, SD=53, median = 33, IQR = 40) in February 2021. Contacts of people aged 18-55 represented 50% of total contacts, with most contacts in household and work or study settings for both surveys. Mean contacts increased for Ethiopia, Ghana, Liberia, Nigeria, Sudan, and Uganda and decreased for Cameroon, the Democratic Republic of Congo (DRC), and Tunisia between the two time points. Men had more contacts than women and contacts were consistent across urban or rural settings (except in Cameroon and Kenya, where urban respondents had more contacts than rural ones, and in Senegal and Zambia, where the opposite was the case). There were no strong and consistent variations in the number of mean or median contacts by education level, self-reported health, perceived self-reported risk of infection, vaccine acceptance, mask ownership, and perceived risk of COVID-19 to health. Mean contacts were correlated with Google mobility (coefficient 0.57, p=0.051 and coefficient 0.28, p=0.291 in August 2020 and February 2021, respectively) and Stringency Index (coefficient -0.12, p = 0.304 and coefficient -0.33, p=0.005 in August 2020 and February 2021, respectively).
CONCLUSIONS: These are the first COVID-19 social contact data collected for 16 of the 19 countries surveyed. We find a high reported number of daily contacts in all countries and substantial variations in mean contacts across countries and by gender. Increased stringency and decreased mobility were associated with a reduction in the number of contacts. These data may be useful to understand

transmission patterns, model infection transmission, and for pandemic planning.

© 2022. The Author(s).

DOI: 10.1186/s12916-022-02543-6
PMCID: PMC9553295
PMID: 36221094 [Indexed for MEDLINE]

Conflict of interest statement: The authors declare that they have no competing interests.

286. Clin Infect Dis. 2022 Oct 12;75(8):1462-1466. doi: 10.1093/cid/ciac362.

Prioritizing Pregnant Women for Coronavirus Disease 2019 Vaccination in African Countries.

Nachega JB(1)(2)(3), Sam-Agudu NA(4)(5)(6), Siedner MJ(7)(8), Rosenthal PJ(9), Mellors JW(10), Zumla A(11)(12), Hermans MP(13), Ntakwinja MB(14), Mukwege DM(14), Langenegger E(15), Mofenson LM(16); African Forum for Research and Education in Health (AFREhealth) Research Collaboration on COVID-19 and Pregnancy.

Author information:
(1)Department of Epidemiology, Infectious Diseases and Microbiology, and Center for Global Health, University of Pittsburgh, Pittsburgh, Pennsylvania, USA.
(2)Departments of Epidemiology and International Health, Johns Hopkins Bloomberg School of Public Health, Baltimore, Maryland, USA.
(3)Department of Medicine, Division of Infectious Diseases, Stellenbosch University Faculty of Medicine and Health Sciences, Cape Town, South Africa.
(4)International Research Center of Excellence, Institute of Human Virology Nigeria, Abuja, Nigeria.
(5)Institute of Human Virology, University of Maryland School of Medicine, Baltimore, Maryland, USA.
(6)Department of Paediatrics and Child Health, University of Cape Coast School of Medical Sciences, Cape Coast, Ghana.
(7)Harvard Medical School and Massachusetts General Hospital, Boston, Massachusetts, USA.
(8)Mbarara University of Science and Technology, Mbarara, Uganda.
(9)Division of Infectious Diseases, Department of Medicine, University of California, San Francisco, San Francisco, California, USA.
(10)Division of Infectious Diseases, Department of Medicine, University of Pittsburgh School of Medicine, Pittsburgh, Pennsylvania, USA.
(11)Center for Clinical Microbiology, Division of Infection and Immunity, University College London, London, United Kingdom.
(12)National Institutes of Health and Research Biomedical Research Centre, University College London Hospitals NHS Foundation Trust, London, United

Kingdom.
(13)Department of Endocrinology and Nutrition, Cliniques Universitaires St-Luc, Brussels, Belgium.
(14)Department of Obstetrics and Gynecology, Panzi Hospital and Université Evangelique en Afrique, Bukavu, Democratic Republic of the Congo.
(15)Department of Obstetrics and Gynecology, Stellenbosch University Faculty of Medicine and Health Sciences, Cape Town, South Africa.
(16)Elizabeth Glaser Pediatric AIDS Foundation, Washington, District of Columbia, USA.

Coronavirus disease 2019 (COVID-19) in pregnancy is associated with excess maternal and infant morbidity and mortality in both African and higher-resource settings. Furthermore, mounting evidence demonstrates the safety and efficacy of COVID-19 vaccination for pregnant women and infants. However, national guidelines in many African countries are equivocal or lack recommendations on COVID-19 vaccine in pregnancy. We summarize key data on COVID-19 epidemiology and vaccination among pregnant African women to highlight major barriers to vaccination and recommend 4 interventions. First, policymakers should prioritize pregnant women for COVID-19 vaccination, with a target of 100% coverage. Second, empirically supported public health campaigns should be sustainably implemented to inform and support pregnant women and their healthcare providers in overcoming vaccine hesitancy. Third, COVID-19 vaccination for pregnant women should be expanded to include antenatal care, obstetrics/gynecology, and targeted mass vaccination campaigns. Fourth, national monitoring and evaluation of COVID-19 vaccine uptake, safety, surveillance, and prospective outcomes assessment should be conducted.

© The Author(s) 2022. Published by Oxford University Press on behalf of the Infectious Diseases Society of America. All rights reserved. For permissions, please e-mail: journals.permissions@oup.com.

DOI: 10.1093/cid/ciac362
PMCID: PMC9214132
PMID: 36130226 [Indexed for MEDLINE]

Conflict of interest statement: Potential conflicts of interest. J. B. N. reports grants from NIH/FIC paid to her institution and unrelated to this work (grant numbers 1R25TW011217-01, 1R21TW011706-01, and 1D43TW010937-01A1). N. S. A. reports grants from the Eunice Kennedy Shriver National Institute of Child Health and Human Development, NIH, unrelated to this work and paid to institution (grant number R01HD089866) and an NIH/FIC award through the Adolescent HIV Prevention and Treatment Implementation Science Alliance for the Central and West Africa Implementation Science Alliance. P. J. R. reports grants or contracts unrelated to this work and paid to institution (grant numbers 5R01AI075045-12, 5R01AI139179-04, and 5R01AI117001-07). A. Z. reports grants or contracts unrelated to this work and paid to institution: Pan-African Network on Emerging and Re-Emerging Infections (PANDORA-ID-NET, https://www.pandoraid.net/)

funded by the European and Developing Countries Clinical Trials Partnership and the European Union Horizon 2020 Framework Program for Research and Innovation. J. W. M. reports research grants unrelated to this work and paid to the University of Pittsburgh from the NIH to the Pitt-Ohio State Clinical Trials Unit (grant number UM1 AI068636), the University of Pittsburgh Virology Support Laboratory (grant number UM1 AI106701), the I4C Martin Delaney Collaboratory for an HIV Cure (grant number UM1 AI126603), the REACH Martin Delaney Collaboratory for HIV Cure (grant number UM1 AI164565), and the National Cancer Institute through Leidos (contract numbers HHSN261200800001E and 75N91019D00024), US Agency for International Development, Gilead Sciences, and Janssen Pharmaceuticals; consulting fees from Gilead Sciences (scientific advisory board), Accelevir Diagnostics (consulting agreement), and Merck (consulting agreement); shares from Abound Bio, and share options with Co-Crystal Pharma and Infectious Diseases Connect; a consulting agreement with Xi-an Yufan Biotechnologies; and employment with the University of Pittsburgh. M. J. S. reports grants unrelated to this work and paid to institution (Massachusetts General Hospital): NIH/National Institute on Aging (grant number R01AG059504-03) and NIH/National Heart, Lung, and Blood Institute (R01 HL141053-04). L. M. M. reports consulting fees from the World Health Organization as a consultant on COVID-19 in pregnancy and mother-to-child SARS-CoV-2 transmission (this contract is now completed) and payment from Virology Education for a continuing education talk on SARS-CoV-2 in pregnancy and possibility of mother-to-child SARS-CoV-2 transmission. All other authors report no potential conflicts. All authors have submitted the ICMJE Form for Disclosure of Potential Conflicts of Interest. Conflicts that the editors consider relevant to the content of the manuscript have been disclosed.

287. Clin Infect Dis. 2022 Oct 12;75(8):1359-1369. doi: 10.1093/cid/ciac156.

Altered Maternal Antibody Profiles in Women With Human Immunodeficiency Virus Drive Changes in Transplacental Antibody Transfer.

Dolatshahi S(1), Butler AL(2), Siedner MJ(3)(4)(5), Ngonzi J(6), Edlow AG(7), Adong J(6), Jennewein MF(8), Atyeo C(9), Bassett IV(3)(4), Roberts DJ(10), Lauffenburger DA(11), Alter G(3)(9), Bebell LM(3)(4)(5).

Author information:
(1)Department of Biomedical Engineering, University of Virginia, Charlottesville, Virginia, USA.
(2)State University of New York Upstate Medical University, Syracuse, New York, USA.
(3)Division of Infectious Diseases, Massachusetts General Hospital, Boston, Massachusetts, USA.
(4)Medical Practice Evaluation Center, Massachusetts General Hospital, Boston, Massachusetts, USA.
(5)Center for Global Health, Massachusetts General Hospital, Boston,

Massachusetts, USA.
(6)Mbarara University of Science and Technology, Mbarara, Uganda.
(7)Division of Maternal-Fetal Medicine, Department of Obstetrics and Gynecology, Massachusetts General Hospital, Boston, Massachusetts, USA.
(8)Fred Hutchinson Cancer Center, Seattle, Washington, USA.
(9)Ragon Institute of Massachusetts General Hospital, Massachusetts Institute of Technology and Harvard, Cambridge, Massachusetts, USA.
(10)Department of Pathology, Massachusetts General Hospital, Boston, Massachusetts, USA.
(11)Massachusetts Institute of Technology, Cambridge, Massachusetts, USA.

BACKGROUND: Human immunodeficiency virus (HIV)-exposed, uninfected (HEU) children have a higher risk of severe infection, but the causes are poorly understood. Emerging data point to altered antibody transfer in women with HIV (WHIV); however, specific perturbations and the influence of antiretroviral therapy (ART) and HIV viremia remain unclear.
METHODS: We evaluated antigen-specific transplacental antibody transfer across 14 antigens in paired maternal and umbilical cord plasma from 352 Ugandan women; 176 were WHIV taking ART. We measured antigen-specific immunoglobulin G (IgG) sub-class (IgG1, 2, 3, 4) levels and antibody Fcγ receptor (FcγRn, 2a, 2b, 3a, 3b) binding profiles. We used partial least squares discrimi-nant analysis to define antigen-specific transplacental antibody transfer features.
RESULTS: Global antibody transfer patterns were similar by maternal HIV serostatus, pointing to effective placental function in WHIV. However, HEU umbilical cord antibody profiles were altered, driven by perturbed WHIV seroprofiles, with higher levels of herpesvirus antibodies ($P < .01$ for Epstein-Barr virus, herpes simplex virus) and lower levels of classic vaccine-induced antibodies ($P < .01$ for tetanus, polio, Haemophilus influenzae type b), suggesting that umbilical cord antibody profile differences arise from imbalanced WHIV immunity. Abnormal WHIV antibody profiles were associated with HIV viremia, lower CD4 count, and postconception ART initiation ($P = .01$).
CONCLUSIONS: Perturbed immune-dominance profiles in WHIV shift the balance of immunity delivered to neonates. Perturbed HIV-associated maternal antibody profiles are a key determinant of com-promised neonatal immunity. Maternal vaccination interventions may promote transfer of relevant, effective antibodies to protect HEU children against early-life infections.

© The Author(s) 2022. Published by Oxford University Press for the Infectious Diseases Society of America. All rights reserved. For permissions, e-mail: journals.permissions@oup.com.

DOI: 10.1093/cid/ciac156
PMCID: PMC9555842
PMID: 35245365 [Indexed for MEDLINE]

Conflict of interest statement: Potential conflicts of interest. G. A. reports grants or contracts from the Ragon Institute and the MGH Scholars Program; has

received consulting fees from Sanofi Pasteur and payment or honoraria for lectures from Princeton University, George Washington University, Johns Hopkins University, Washington University, University of Washington, Yale University, Walter Reed Medical Institute, State University of New York (Upstate), and Mount Sinai; holds patents on direct expression of antibodies (Novartis) (PAT056060-EP-EPA), antigen-specific antibody glycosylation as a diagnostic marker of disease state (MGH21944), system and method for the multiplexed affinity purification of proteins and cells (MIT 16927), and a rapid Fc-enhancing screening approach (MGH in preparation 2021; pending); participates on a data and safety monitoring board for a CAPRISA monoclonal therapeutic trial for the University of KwaZulu-Natal, South Africa; and has stock options with Systems Seromyx and Leyden Labs. D. J. R. reports author royalties from UpToDate and Cambridge University Press; honoraria for lectures from various academic organizations; academic support for traveling from her institution; and a pending patent for an ex vivo dual perfusion placental chamber magnetic resonance imaging compatible. All other authors report no potential conflicts. All authors have submitted the ICMJE Form for Disclosure of Potential Conflicts of Interest. Conflicts that the editors consider relevant to the content of the manuscript have been disclosed.

288. Ecancermedicalscience. 2022 Oct 7;16:1454. doi: 10.3332/ecancer.2022.1454. eCollection 2022.

Targeting elimination of cervical cancer by 2030: a baseline assessment in six African countries-part II.

Stefan DC(1), Dangou JM(2), Barango P(2), Mahamadou ID(2), Kapambwe S(2).

Author information:
(1)Institute of Global Health Equity Research Rwanda, University of Global Health Equity, 0000 Kigali, Rwanda.
(2)World Health Organization Regional Office for Africa, P.O. Box: 06, Brazzaville, Republic of Congo.

BACKGROUND: Africa is home to many countries with the highest incidence of cervical cancer in the world. It is encouraging to see that the efforts to prevent and manage this disease are intensifying across the continent. The World Health Organization (WHO) has, in the last years, led a world-wide initiative to eliminate cervical cancer as a public health problem, starting by setting clear targets for 2030.
AIM: To assist those African countries with the largest burden of cervical cancer, to reach the set targets, as a first step, WHO initiated a baseline capacity assessment in African high burden countries. We present and discuss the results thereof in this paper.
METHOD: The countries selected for the baseline evaluation were Eswatini, Guinea, Malawi, Rwanda, Uganda and Zambia. The data were collected by a mailed

questionnaire in English, with 129 questions, most of them with preset answer options. It was answered by national coordinators of non-communicable diseases, cancer control or reproductive health programmes in the ministries of health and by the WHO country representatives. Based on the answers, suggestions were made towards optimising the efforts for cancer control.
RESULTS: Except for Eswatini and Guinea, vaccination against the human papilloma virus (HPV) has reached between 74% and 98% of girls in the age bracket. The main method for cervical screening is still inspection with acid acetic. HPV testing, recommended by WHO, is being introduced slowly. The numbers of women screened are not yet nearing the required levels. Between 30% and 70% of the cervical cancers diagnosed are being treated with palliative intent. A deficit of personnel was reported for all professions involved in cervical cancer care.
CONCLUSIONS: Guinea will need assistance to implement HPV immunisations and expand screening. In all six countries surveyed, steps should be taken to introduce or scale up the more precise HPV screening instead of acid acetic inspection of the cervix, to replace the current cryoablation of the preinvasive lesions of the cervix with thermal ablation and to increase the capacity for screening. Solutions need to be found for covering the dearth in gynaecological oncologists and radiotherapy installations and personnel.

© the authors; licensee ecancermedicalscience.

DOI: 10.3332/ecancer.2022.1454
PMCID: PMC9666272
PMID: 36405933

Conflict of interest statement: None declared.

289. Science. 2022 Oct 7;378(6615):14-15. doi: 10.1126/science.adf1847. Epub 2022 Oct 6.

Uganda's Ebola outbreak will put novel vaccines to the test.

Cohen J.

As a less-studied ebolavirus kills dozens, researchers again scramble to launch trials during a crisis.

DOI: 10.1126/science.adf1847
PMID: 36201577 [Indexed for MEDLINE]

290. Science. 2022 Oct 7;378(6615):eabq5358. doi: 10.1126/science.abq5358. Epub 2022 Oct 7.

The evolving SARS-CoV-2 epidemic in Africa: Insights from rapidly expanding

genomic surveillance.

Tegally H(#)(1)(2), San JE(1)(2), Cotten M(3)(4), Moir M(1), Tegomoh B(5)(6), Mboowa G(7), Martin DP(8)(9), Baxter C(1)(10), Lambisia AW(11), Diallo A(12), Amoako DG(13)(14), Diagne MM(12), Sisay A(15)(16), Zekri AN(17), Gueye AS(18), Sangare AK(19), Ouedraogo AS(20), Sow A(21), Musa AO(22)(23)(24), Sesay AK(25), Abias AG(26), Elzagheid AI(27), Lagare A(28), Kemi AS(29), Abar AE(30), Johnson AA(31)(32), Fowotade A(33)(34), Oluwapelumi AO(35)(36), Amuri AA(37)(38), Juru A(39), Kandeil A(40), Mostafa A(40), Rebai A(41), Sayed A(42), Kazeem A(43)(44), Balde A(45)(46), Christoffels A(7)(47), Trotter AJ(48), Campbell A(49), Keita AK(50)(51), Kone A(52), Bouzid A(41)(53), Souissi A(41), Agweyu A(11), Naguib A(54), Gutierrez AV(48), Nkeshimana A(55), Page AJ(48), Yadouleton A(56), Vinze A(57), Happi AN(43), Chouikha A(58)(59), Iranzadeh A(8)(9), Maharaj A(1), Batchi-Bouyou AL(60)(61), Ismail A(13), Sylverken AA(62)(63), Goba A(64)(65), Femi A(43)(44), Sijuwola AE(43), Marycelin B(66)(67), Salako BL(29)(32), Oderinde BS(66), Bolajoko B(43), Diarra B(52), Herring BL(18), Tsofa B(11), Lekana-Douki B(68)(69), Mvula B(70), Njanpop-Lafourcade BM(18), Marondera BT(71), Khaireh BA(72)(73), Kouriba B(19), Adu B(74), Pool B(75), McInnis B(17), Brook C(76)(77), Williamson C(9)(10)(78), Nduwimana C(55), Anscombe C(79)(80), Pratt CB(81), Scheepers C(13)(82), Akoua-Koffi CG(83)(84), Agoti CN(11)(85), Mapanguy CM(60)(86), Loucoubar C(12), Onwuamah CK(87), Ihekweazu C(88), Malaka CN(89), Peyrefitte C(12), Grace C(43)(44), Omoruyi CE(33)(34), Rafaï CD(90), Morang'a CM(91), Erameh C(92), Lule DB(3), Bridges DJ(93), Mukadi-Bamuleka D(37), Park D(57), Rasmussen DA(94)(95), Baker D(48), Nokes DJ(11)(96), Ssemwanga D(3)(97), Tshiabuila D(2), Amuzu DSY(91), Goedhals D(98), Grant DS(64)(65)(99), Omuoyo DO(11), Maruapula D(100), Wanjohi DW(7), Foster-Nyarko E(48), Lusamaki EK(37)(38)(51), Simulundu E(101), Ong'era EM(11), Ngabana EN(37)(38), Abworo EO(102), Otieno E(11), Shumba E(71), Barasa E(11), Ahmed EB(103)(104), Ahmed EA(23), Lokilo E(37), Mukantwari E(105), Philomena E(43), Belarbi E(106), Simon-Loriere E(107), Anoh EA(83), Manuel E(108), Leendertz F(106), Taweh FM(109), Wasfi F(58), Abdelmoula F(41)(110), Takawira FT(39), Derrar F(111), Ajogbasile FV(43), Treurnicht F(112)(113), Onikepe F(43)(44), Ntoumi F(60)(114), Muyembe FM(37)(38), Ragomzingba FEZ(115), Dratibi FA(116)(18), Iyanu FA(43), Mbunsu GK(38), Thilliez G(48), Kay GL(48), Akpede GO(92), van Zyl GU(117)(118), Awandare GA(91), Kpeli GS(119)(120), Schubert G(106), Maphalala GP(121), Ranaivoson HC(77), Omunakwe HE(122), Onywera H(7), Abe H(123), Karray H(124), Nansumba H(125), Triki H(58), Kadjo HAA(126), Elgahzaly H(127), Gumbo H(39), Mathieu H(128), Kavunga-Membo H(37), Smeti I(41), Olawoye IB(43), Adetifa IMO(88)(129), Odia I(92), Ben Boubaker IB(130)(131), Muhammad IA(43), Ssewanyana I(125), Wurie I(132), Konstantinus IS(133), Halatoko JWA(134), Ayei J(26), Sonoo J(135), Makangara JC(37)(38), Tamfum JM(37)(38), Heraud JM(12)(77), Shaffer JG(136), Giandhari J(2), Musyoki J(11), Nkurunziza J(137), Uwanibe JN(43), Bhiman JN(13)(113), Yasuda J(123), Morais J(138)(139), Kiconco J(97), Sandi JD(64)(65), Huddleston J(140), Odoom JK(74), Morobe JM(11), Gyapong JO(119), Kayiwa JT(3), Okolie JC(43), Xavier JS(1)(141)(142), Gyamfi J(119), Wamala JF(143), Bonney JHK(74), Nyandwi J(55)(144), Everatt J(13), Nakaseegu J(97), Ngoi JM(91), Namulondo J(97), Oguzie JU(43)(44), Andeko JC(68),

Lutwama JJ(3), Mogga JJH(143), O'Grady J(48), Siddle KJ(57), Victoir K(145), Adeyemi KT(43)(44), Tumedi KA(146), Carvalho KS(147), Mohammed KS(11), Dellagi K(145), Musonda KG(148), Duedu KO(119)(120), Fki-Berrajah L(124), Singh L(2), Kepler LM(94)(95), Biscornet L(75), de Oliveira Martins L(48), Chabuka L(149), Olubayo L(8), Ojok LD(26), Deng LL(26), Ochola-Oyier LI(11), Tyers L(9), Mine M(150), Ramuth M(135), Mastouri M(151)(152), ElHefnawi M(153), Mbanne M(12), Matsheka MI(146), Kebabonye M(154), Diop M(12), Momoh M(64)(65)(155), Lima Mendonça MDL(147), Venter M(156), Paye MF(57), Faye M(12), Nyaga MM(157), Mareka M(158), Damaris MM(159), Mburu MW(11), Mpina MG(160)(161)(162), Owusu M(163), Wiley MR(81)(164), Tatfeng MY(165), Ayekaba MO(161), Abouelhoda M(166)(167), Beloufa MA(111), Seadawy MG(168)(169), Khalifa MK(170), Matobo MM(158), Kane M(12), Salou M(171), Mbulawa MB(154), Mwenda M(93), Allam M(172), Phan MVT(3), Abid N(151)(173), Rujeni N(174)(175), Abuzaid N(176), Ismael N(177), Elguindy N(54), Top NM(12), Dia N(12), Mabunda N(177), Hsiao NY(9)(78), Silochi NB(161), Francisco NM(138), Saasa N(178), Bbosa N(3), Murunga N(11), Gumede N(18), Wolter N(13)(113), Sitharam N(1), Ndodo N(88), Ajayi NA(179), Tordo N(180), Mbhele N(9), Razanajatovo NH(77), Iguosadolo N(43), Mba N(88), Kingsley OC(181), Sylvanus O(92), Femi O(182), Adewumi OM(31)(32), Testimony O(43)(44), Ogunsanya OA(43), Fakayode O(183), Ogah OE(184), Oludayo OE(43), Faye O(12), Smith-Lawrence P(154), Ondoa P(71), Combe P(185), Nabisubi P(186)(187), Semanda P(125), Oluniyi PE(43), Arnaldo P(177), Quashie PK(91), Okokhere PO(92)(188), Bejon P(11), Dussart P(77), Bester PA(189), Mbala PK(37)(38), Kaleebu P(3)(97), Abechi P(43)(44), El-Shesheny R(40)(190), Joseph R(9), Aziz RK(191)(192), Essomba RG(193)(194), Ayivor-Djanie R(91)(119)(120), Njouom R(195), Phillips RO(63), Gorman R(63), Kingsley RA(48), Neto Rodrigues RMDESA(196)(197), Audu RA(29), Carr RAA(119)(120), Gargouri S(124), Masmoudi S(41), Bootsma S(143), Sankhe S(12), Mohamed SI(198), Femi S(43), Mhalla S(131)(199), Hosch S(160)(200), Kassim SK(127), Metha S(57), Trabelsi S(201), Agwa SH(127), Mwangi SW(7), Doumbia S(52), Makiala-Mandanda S(37)(38), Aryeetey S(63), Ahmed SS(54), Ahmed SM(103), Elhamoumi S(57), Moyo S(100)(202), Lutucuta S(138), Gaseitsiwe S(100)(202), Jalloh S(64)(65), Andriamandimby SF(77), Oguntope S(43), Grayo S(180), Lekana-Douki S(68), Prosolek S(48), Ouangraoua S(203)(204), van Wyk S(1), Schaffner SF(57), Kanyerezi S(186)(187), Ahuka-Mundeke S(37)(38), Rudder S(48), Pillay S(2), Nabadda S(125), Behillil S(205), Budiaki SL(158), van der Werf S(205), Mashe T(39)(206), Mohale T(13), Le-Viet T(48), Velavan TP(114)(207), Schindler T(160)(161)(200), Maponga TG(117), Bedford T(140)(208), Anyaneji UJ(2), Chinedu U(43)(44), Ramphal U(2)(10)(209), George UE(43), Enouf V(205), Nene V(102), Gorova V(210)(211), Roshdy WH(54), Karim WA(1), Ampofo WK(212), Preiser W(117)(118), Choga WT(100)(213), Ahmed YA(18), Ramphal Y(1), Bediako Y(91)(214), Naidoo Y(2), Butera Y(174)(215)(216), de Laurent ZR(11); Africa Pathogen Genomics Initiative (Africa PGI)‡; Ouma AEO(7), von Gottberg A(13)(113), Githinji G(11)(217), Moeti M(18), Tomori O(43), Sabeti PC(57), Sall AA(12), Oyola SO(102), Tebeje YK(7), Tessema SK(7), de Oliveira T(1)(2)(10)(218), Happi C(43)(44), Lessells R(2), Nkengasong J(7), Wilkinson E(#)(1)(2).

Author information:

(1)Centre for Epidemic Response and Innovation (CERI), School of Data Science and Computational Thinking, Stellenbosch University, Stellenbosch, South Africa.
(2)KwaZulu-Natal Research Innovation and Sequencing Platform (KRISP), Nelson R Mandela School of Medicine, University of KwaZulu-Natal, Durban, South Africa.
(3)MRC/UVRI and LSHTM Uganda Research Unit, Entebbe, Uganda.
(4)MRC-University of Glasgow Centre for Virus Research, Glasgow, UK.
(5)The Biotechnology Centre of the University of Yaoundé I, Yaoundé, Cameroon.
(6)CDC Foundation, Atlanta, Georgia, Nebraska Department of Health and Human Services, Lincoln, NE, USA.
(7)Institute of Pathogen Genomics, Africa Centres for Disease Control and Prevention (Africa CDC), Addis Ababa, Ethiopia.
(8)Institute of Infectious Diseases and Molecular Medicine, Department of Integrative Biomedical Sciences, Computational Biology Division, University of Cape Town, Cape Town, South Africa.
(9)Division of Medical Virology, Wellcome Centre for Infectious Diseases in Africa, Institute of Infectious Disease and Molecular Medicine, University of Cape Town, Cape Town, South Africa.
(10)Centre for the AIDS Programme of Research in South Africa (CAPRISA), Durban, South Africa.
(11)KEMRI-Wellcome Trust Research Programme, Kilifi, Kenya.
(12)Institut Pasteur de Dakar, Dakar, Senegal.
(13)National Institute for Communicable Diseases (NICD) of the National Health Laboratory Service (NHLS), Johannesburg, South Africa.
(14)School of Health Sciences, College of Health Sciences, University of KwaZulu-Natal, Durban, KwaZulu-Natal, South Africa.
(15)Department of Medical Laboratory Sciences, College of Health Sciences, Addis Ababa University, Addis Ababa, Ethiopia.
(16)Department of Microbial, Cellular and Molecular Biology, College of Natural and Computational Sciences, Addis Ababa University, Addis Ababa, Ethiopia.
(17)Cancer Biology Department, Virology and Immunology Unit, National Cancer Institute, Cairo University, Cairo, Egypt.
(18)World Health Organization, Africa Region, Brazzaville, Republic of the Congo.
(19)Centre d'Infectiologie Charles Mérieux-Mali (CICM-Mali), Bamako, Mali.
(20)Bacteriology and Virology Department Souro Sanou University Hospital, Bobo-Dioulasso, Burkina Faso.
(21)West African Health Organisation, Bobo-Dioulasso, Burkina Faso.
(22)Faculty of Medicine and Health Sciences, Kassala University, Kassala City, Sudan.
(23)Department of Microbiology, Faculty of Medical Laboratory Sciences, University of Gezira, Gezira, Sudan.
(24)General Administration of Laboratories and Blood Banks, Ministry of Health, Kassala State, Sudan.
(25)MRC Unit The Gambia at LSHTM, Fajara, Gambia.
(26)National Public Health Laboratory, Ministry of Health, Juba, Republic of South Sudan.
(27)Libyan Biotechnology Research Center, Tripoli, Libya.

(28)Center for Medical and Sanitary Research (CERMES), Niamey, Niger.
(29)The Nigerian Institute of Medical Research, Yaba, Lagos, Nigeria.
(30)Laboratoire de la Caisse Nationale de Sécurité Sociale, Djibouti, Republic of Djibouti.
(31)Department of Virology, College of Medicine, University of Ibadan, Ibadan, Nigeria.
(32)Infectious Disease Institute, College of Medicine, University of Ibadan, Ibadan, Nigeria.
(33)Medical Microbiology and Parasitology Department, College of Medicine, University of Ibadan, Ibadan, Nigeria.
(34)Biorepository Clinical Virology Laboratory, College of Medicine, University of Ibadan, Ibadan, Nigeria.
(35)Department of Medical Microbiology and Parasitology, Faculty of Basic Clinical Sciences, College of Health Sciences, University of Ilorin, Ilorin, Kwara State, Nigeria.
(36)The Pirbright Institute, Woking, UK.
(37)Pathogen Sequencing Lab, Institut National de Recherche Biomédicale (INRB), Kinshasa, the Democratic Republic of the Congo.
(38)Université de Kinshasa (UNIKIN), Kinshasa, the Democratic Republic of the Congo.
(39)National Microbiology Reference Laboratory, Harare, Zimbabwe.
(40)Center of Scientific Excellence for Influenza Viruses, National Research Centre (NRC), Cairo, Egypt.
(41)Laboratory of Molecular and Cellular Screening Processes, Centre of Biotechnology of Sfax, University of Sfax, Sfax, Tunisia.
(42)Genomics and Epigenomics Program, Research Department CCHE57357, Cairo, Egypt.
(43)African Centre of Excellence for Genomics of Infectious Diseases (ACEGID), Redeemer's University, Ede, Osun State, Nigeria.
(44)Department of Biological Sciences, Faculty of Natural Sciences, Redeemer's University, Ede, Osun State, Nigeria.
(45)Laboratório de Biologia Molecular Jean Piaget, Bissau, Guinea-Bissau.
(46)University Jean Piaget in Guinea-Bissau, Bissau, Guinea-Bissau.
(47)SAMRC Bioinformatics Unit, SA Bioinformatics Institute, University of the Western Cape, Cape Town, South Africa.
(48)Quadram Institute Bioscience, Norwich, UK.
(49)Central Public Health Reference Laboratories, Freetown, Sierra Leone.
(50)Centre de Recherche et de Formation en Infectiologie de Guinée (CERFIG), Université de Conakry, Conakry, Guinea.
(51)TransVIHMI, Institut de Recherche pour le Développement, Institut National de la Santé et de la Recherche Médicale (INSERM), Montpellier University, 34090, Montpellier, France.
(52)University Clinical Research Center (UCRC), University of Sciences, Techniques and Technology of Bamako, Bamako, Mali.
(53)Sharjah Institute for Medical Research, College of Medicine, University of Sharjah, Sharjah, United Arab Emirates.
(54)Central Public Health Laboratories (CPHL), Cairo, Egypt.

(55)National Institute of Public Health, Bujumbura, Burundi.
(56)Laboratoire des Fièvres Hémorragiques Virales du Benin, Cotonou, Benin.
(57)Infectious Disease and Microbiome Program, Broad Institute of Harvard and MIT, Cambridge, MA, USA.
(58)Laboratory of Clinical Virology, WHO Reference Laboratory for Poliomyelitis and Measles in the Eastern Mediterranean Region, Pasteur Institute of Tunis, University Tunis El Manar (UTM), Tunis 1002, Tunisia.
(59)Research Laboratory "Virus, Vectors and Hosts: One Health Apporach and Technological Innovation for a Better Health", LR20IPT02, Pasteur Institute, Tunis 1002, Tunisia.
(60)Fondation Congolaise pour la Recherche Médicale, Brazzaville, Republic of the Congo.
(61)Marien Ngouabi, Brazzaville, Republic of the Congo.
(62)Kwame Nkrumah University of Science and Technology, Department of Theoretical and Applied Biology, Kumasi, Ghana.
(63)Kumasi Centre for Collaborative Research in Tropical Medicine, Kwame Nkrumah University of Science and Technology, Kumasi, Ghana.
(64)Viral Haemorrhagic Fever Laboratory, Kenema Government Hospital, Kenema, Sierra Leone.
(65)Ministry of Health and Sanitation, Freetown, Sierra Leone.
(66)Department of Immunology, University of Maiduguri Teaching Hospital, P.M.B. 1414, Maiduguri, Nigeria.
(67)Department of Medical Laboratory Science, College of Medical Sciences, University of Maiduguri, P.M.B. 1069, Maiduguri, Borno State, Nigeria.
(68)Centre Interdisciplinaires de Recherches Medicales de Franceville (CIRMF), Franceville, Gabon.
(69)Département de Parasitologie-Mycologie Université des Sciences de la Santé (USS), Libreville, Gabon.
(70)National HIV Reference Laboratory, Community Health Sciences Unit, Ministry of Health, Lilongwe, Malawi.
(71)African Society for Laboratory Medicine, Addis Ababa, Ethiopia.
(72)National Medical and Molecular Biology Laboratory, Ministry of Health, Djibouti, Republic of Djibouti.
(73)Africa CDC, Rapid Responder, Team Djibouti, Djibouti, Djibouti.
(74)Noguchi Memorial Institute for Medical Research, University of Ghana, Legon, Ghana.
(75)Seychelles Public Health Laboratory, Public Health Authority, Ministry of Health Seychelles, Victoria, Seychelles.
(76)Department of Ecology and Evolution, University of Chicago, Chicago, IL, USA.
(77)Virology Unit, Institut Pasteur de Madagascar, Antananarivo, Madagascar.
(78)National Health Laboratory Service (NHLS), Cape Town, South Africa.
(79)Malawi-Liverpool-Wellcome Trust Clinical Research Programme, Blantyre, Malawi.
(80)Liverpool School of Tropical Medicine, Liverpool, UK.
(81)University of Nebraska Medical Center (UNMC), Omaha, NE, USA.
(82)SAMRC Antibody Immunity Research Unit, School of Pathology, University of

the Witwatersrand, Johannesburg, South Africa.
(83)CHU de Bouaké, Laboratoire/Unité de Diagnostic des Virus des Fièvres Hémorragiques et Virus Émergents, Bouaké, Côte d'Ivoire.
(84)UFR Sciences Médicales, Universite Alassane Ouattara, Bouaké, Côte d'Ivoire.
(85)School of Public Health, Pwani University, Kilifi, Kenya.
(86)Faculty of Science and Techniques, University Marien Ngouabi, Brazzaville, Republic of the Congo.
(87)Centre for Human Virology and Genomics, Nigerian Institute of Medical Research, Yaba, Lagos, Nigeria.
(88)Nigeria Centre for Disease Control and Prevention, Abuja, Nigeria.
(89)Laboratoire des Arbovirus, Fièvres Hémorragiques virales, Virus Emergents et Zoonoses, Institut Pasteur de Bangui, Bangui, Central African Republic.
(90)Le Laboratoire National de Biologie Clinique et de Santé Publique (LNBCSP), Bangui, Central African Republic.
(91)West African Centre for Cell Biology of Infectious Pathogens (WACCBIP), College of Basic and Applied Sciences, University of Ghana, Accra, Ghana.
(92)Institute of Lassa Fever Research and Control, Irrua Specialist Teaching Hospital, Irrua, Nigeria.
(93)PATH, Lusaka, Zambia.
(94)Department of Entomology and Plant Pathology, North Carolina State University, Raleigh, NC, USA.
(95)Bioinformatics Research Center, North Carolina State University, Raleigh, NC, USA.
(96)School of Life Sciences and Zeeman Institute for Systems Biology and Infectious Disease Epidemiology Research (SBIDER), University of Warwick, Coventry, UK.
(97)Uganda Virus Research Institute, Entebbe, Uganda.
(98)PathCare Vermaak, Pretoria, South Africa and Division of Virology, University of the Free State, Bloemfontein, South Africa.
(99)College of Medicine and Allied Health Sciences, University of Sierra Leone, Freetown, Sierra Leone.
(100)Botswana Harvard AIDS Institute Partnership and Botswana Harvard HIV Reference Laboratory, Gaborone, Botswana.
(101)Macha Research Trust, Choma, Zambia.
(102)International Livestock Research Institute (ILRI), Nairobi, Kenya.
(103)INRSP, Nouakchott, Mauritania.
(104)Faculté de Médecine de Nouakchott, Nouakchott, Mauritani.
(105)Rwanda National Reference Laboratory, Kigali, Rwanda.
(106)Robert Koch-Institute, Berlin, Germany.
(107)G5 Evolutionary Genomics of RNA Viruses, Institut Pasteur, Paris, France.
(108)Direcção Nacional da Saúde Pública, Ministério da Saúde, Luanda, Angola.
(109)National Public Health Reference Laboratory-National Public Health Institute of Liberia, Monrovia, Liberia.
(110)Faculty of Pharmacy of Monastir, Monastir, Tunisia.
(111)National Influenza Centre, Institut Pasteur d'Algérie, Algiers, Algeria.
(112)Department of Virology, National Health Laboratory Service (NHLS), Charlotte Maxeke Johannesburg Academic Hospital, Johannesburg, South Africa.

(113)School of Pathology, Faculty of Health Science, University of the Witwatersrand, Johannesburg, South Africa.
(114)Institute of Tropical Medicine, Universitätsklinikum Tübingen, Tübingen, Germany.
(115)Ministère de Santé Publique et de la Solidarité Nationale, Ndjamena, Chad.
(116)WHO Int Comoros, Moroni, Union of Comoros.
(117)Division of Medical Virology, Faculty of Medicine and Health Sciences, Stellenbosch University, Tygerberg, Cape Town, South Africa.
(118)National Health Laboratory Service (NHLS), Tygerberg, Cape Town, South Africa.
(119)UHAS COVID-19 Testing and Research Centre, University of Health and Allied Sciences, Ho, Ghana.
(120)Department of Biomedical Sciences, University of Health and Allied Sciences, PMB 31, Ho, Ghana.
(121)Ministry of Health, COVID-19 Testing Laboratory, Mbabane, Kingdom of Eswatini.
(122)Satellite Molecular Laboratory, Rivers State University Teaching Hospital, Port Harcourt, Nigeria.
(123)Department of Emerging Infectious Diseases, Institute of Tropical Medicine, Nagasaki University, Nagasaki, Japan.
(124)CHU Habib Bourguiba, Laboratory of Microbiology, Faculty of Medicine of Sfax, University of Sfax, Sfax, Tunisia.
(125)Central Public Health Laboratories (CPHL), Kampala, Uganda.
(126)Institut Pasteur de Côte d'Ivoire, Departement des Virus Epidemiques, Abidjan, Côte d'Ivoire.
(127)Faculty of Medicine Ain Shams Research Institute (MASRI), Ain Shams University, Cairo, Egypt.
(128)Doctoral School of Technical and Environmental Sciences, Department of Biology and Human Health, N'Djamena, Chad.
(129)Department of Infectious Diseases Epidemiology, London School of Hygiene and Tropical Medicine, London, UK.
(130)Charles Nicolle Hospital, Laboratory of Microbiology, National Influenza Center, Tunis, Tunisia.
(131)University of Tunis El Manar, Faculty of Medicine of Tunis, Research Laboratory LR99ES09, Tunis, Tunisia.
(132)College of Medicine and Allied Health Science, University of Sierra Leone, Freetown, Sierra Leone.
(133)Namibia Institute of Pathology, Windhoek, Namibia.
(134)National Institute of Hygiene, Lomé, Togo.
(135)Virology/Molecular Biology Department, Central Health Laboratory, Victoria Hospital, Ministry of Health and Wellness, Port Louis, Mauritius.
(136)Department of Biostatistics and Data Science, School of Public Health and Tropical Medicine, Tulane University, New Orleans, LA, USA.
(137)WHO Burundi, Gitega, Burundi.
(138)Grupo de Investigação Microbiana e Imunológica, Instituto Nacional de Investigação em Saúde (National Institute for Health Research), Luanda, Angola.
(139)Departamento de Bioquímica, Faculdade de Medicina, Universidade Agostinho

Neto, Luanda, Angola.
(140)Vaccine and Infectious Disease Division, Fred Hutchinson Cancer Center, Seattle, WA, USA.
(141)Universidade Federal de Minas Gerais, Belo Horizonte, Brazil.
(142)Institute of Agricultural Sciences, Universidade Federal dos Vales do Jequitinhonha e Mucuri, Unaí, Brazil.
(143)WHO South Sudan, Juba, South Sudan.
(144)Faculty of Medicine, University of Burundi, Bujumbura, Burundi.
(145)Pasteur Network, Institut Pasteur, Paris, France.
(146)Botswana Institute for Technology Research and Innovation, Gaborone, Botswana.
(147)Instituto Nacional de Saúde Pública, Praia, Cape Verde.
(148)Zambia National Public Health Institute, Lusaka, Zambia.
(149)Public Health Institute of Malawi, Lilongwe, Malawi.
(150)National Health Laboratory, Gaborone, Botswana.
(151)Laboratory of Transmissible Diseases and Biologically Active Substances (LR99ES27), Faculty of Pharmacy, University of Monastir, Monastir, Tunisia.
(152)Laboratory of Microbiology, University Hospital of Monastir, Monastir, Tunisia.
(153)Biomedical Informatics and Chemoinformatics Group, Informatics and Systems Department, National Research Centre, Cairo, Egypt.
(154)Ministry of Health and Wellness, Gaborone, Botswana.
(155)Eastern Technical University of Sierra Leone, Kenema, Sierra Leone.
(156)Zoonotic Arbo and Respiratory Virus Program, Centre for Viral Zoonoses, Department of Medical Virology, University of Pretoria, Pretoria, South Africa.
(157)Next Generation Sequencing Unit and Division of Virology, Faculty of Health Sciences, University of the Free State, Bloemfontein, South Africa.
(158)National Reference Laboratory Lesotho, Maseru, Lesotho.
(159)Centre for Biotechnology Research and Development, Kenya Medical Research Institute, Nairobi, Kenya.
(160)Swiss Tropical and Public Health Institute, Basel, Switzerland.
(161)Laboratorio de Investigaciones de Baney, Baney, Equatorial Guinea.
(162)Ifakara Health Insitute, Ifakara, Tanzania.
(163)Department of Medical Diagnostics, Kumasi Centre for Collaborative Research in Tropical Medicine, Kwame Nkrumah University of Science and Technology, Kumasi, Ghana.
(164)PraesensBio, Lincoln, NE, USA.
(165)Department of Medical Laboratory Science, Niger Delta University, Bayelsa State, Nigeria.
(166)Systems and Biomedical Engineering Department, Faculty of Engineering, Cairo University, Cairo, Egypt.
(167)King Faisal Specialist Hospital and Research Center, Riyadh, Kingdom of Saudi Arabia.
(168)Biological Prevention Department, Ministry of Defence, Cairo, Egypt.
(169)Faculty of Science, Fayoum University, Fayoum, Egypt.
(170)Molecular Pathology Lab, Children's Cancer Hospital, Cairo, Egypt.
(171)LaboratoireBiolim FSS/Université de Lomé, Lomé, Togo.

(172)Department of Genetics and Genomics, College of Medicine and Health Sciences, United Arab Emirates University, Abu Dhabi, United Arab Emirates.
(173)High Institute of Biotechnology of Monastir, University of Monastir, Rue Taher Haddad 5000, Monastir, Tunisia.
(174)Rwanda National Joint Task Force COVID-19, Rwanda Biomedical Centre, Ministry of Health, Kigali, Rwanda.
(175)School of Health Sciences, College of Medicine and Health Sciences, University of Rwanda, Kigali, Rwanda.
(176)Department of Microbiology, Faculty of Medical Laboratory Sciences, Omdurman Islamic University, Sudan.
(177)Instituto Nacional de Saúde (INS), Marracuene, Mozambique.
(178)Department of Disease Control, School of Veterinary Medicine, University of Zambia, Lusaka, Zambia.
(179)Internal Medicine Department, Alex Ekwueme Federal University Teaching Hospital, Abakaliki, Nigeria.
(180)Institut Pasteur de Guinée, Conarky, Guinea.
(181)Virology Laboratory, Alex Ekwueme Federal University Teaching Hospital, Abakaliki, Nigeria.
(182)Department of Epidemiology and Community Health, Faculty of Clinical Sciences. College of Health Sciences. University of Ilorin, Ilorin, Kwara State, Nigeria.
(183)Department of Public Health, Ministry of Health, Ilorin, Kwara State, Nigeria.
(184)Alex Ekwueme Federal University Teaching Hospital, Abakaliki, Nigeria.
(185)Mayotte Hospital Center, Mayotte, France.
(186)The African Center of Excellence in Bioinformatics and Data-Intensive Sciences, The Infectious Diseases Institute, Kampala, Uganda.
(187)Immunology and Molecular Biology, Makerere University, Kampala, Uganda.
(188)Department of Medicine, Faculty of Clinical Sciences, College of Medicine, Ambrose Alli University, Ekpoma, Edo State, Nigeria.
(189)Division of Virology, National Health Laboratory Service and University of the Free State, Bloemfontein, South Africa.
(190)Infectious Hazards Preparedness, World Health Organization, Eastern Mediterranean Regional Office, Cairo, Egypt.
(191)Department of Microbiology and Immunology, Faculty of Pharmacy, Cairo University, Cairo, Egypt.
(192)Microbiology and Immunology Research Program, Children's Cancer Hospital Egypt, Cairo, Egypt.
(193)National Public Health Laboratory, Ministry of Public Health of Cameroon, Yaoundé, Cameroon.
(194)Faculty of Medicine and Biomedical Sciences, University of Yaoundé, Yaoundé, Cameroon.
(195)Virology Service, Centre Pasteur of Cameroun, Yaounde, Cameroon.
(196)Coordenadora da rede do Diagnóstico Tuberculose/HIV/COVID-19 na Instituição - Laboratório Nacional de Referência da Tuberculose em São Tomé e Príncipe, São Tomé, São Tomé and Principe.
(197)Ponto focal para Melhoria da qualidade dos Laboratórios (SLIPTA) ao nível

de São Tomé e Príncipe, São Tomé, São Tomé and Principe.
(198)National Public Health Reference Laboratory (NPHRL), Mogadishu, Somalia.
(199)Faculty of Medicine of Monastir, University of Monastir, Monastir, Tunisia.
(200)University of Basel, Basel, Switzerland.
(201)Clinical and Experimental Pharmacology Lab, LR16SP02, National Center of Pharmacovigilance, University of Tunis El Manar, Tunis, Tunisia.
(202)Harvard T.H. Chan School of Public Health, Boston, MA, USA.
(203)Centre MURAZ, Ouagadougou, Burkina Faso.
(204)National Institute of Public Health of Burkina Faso (INSP/BF), Ouagadougou, Burkina Faso.
(205)National Reference Center for Respiratory Viruses, Molecular Genetics of RNA Viruses, UMR 3569 CNRS, Université Paris Cité, Institut Pasteur, Paris, France.
(206)World Health Organization, Harare, Zimbabwe.
(207)Vietnamese-German Center for Medical Research, Hanoi, Vietnam.
(208)Howard Hughes Medical Institute, Fred Hutchinson Cancer Center, Seattle, WA, USA.
(209)Sub-Saharan African Network For TB/HIV Research Excellence (SANTHE), Durban, South Africa.
(210)World Health Organization, WHO Lesotho, Maseru, Lesotho.
(211)Med24 Medical Centre, Ruwa, Zimbabwe.
(212)Department of Virology, Noguchi Memorial Institute for Medical Research, University of Ghana, Legon, Ghana.
(213)Division of Human Genetics, Department of Pathology, University of Cape Town, Cape Town, South Africa.
(214)Yemaachi Biotech, Accra, Ghana.
(215)Center for Human Genetics, College of Medicine and Health Sciences, University of Rwanda, Kigali, Rwanda.
(216)Laboratory of Human Genetics, GIGA Research Institute, Liège, Belgium.
(217)Department of Biochemistry and Biotechnology, Pwani University, Kilifi, Kenya.
(218)Department of Global Health, University of Washington, Seattle, WA, USA.
(#)Contributed equally

Investment in severe acute respiratory syndrome coronavirus 2 (SARS-CoV-2) sequencing in Africa over the past year has led to a major increase in the number of sequences that have been generated and used to track the pandemic on the continent, a number that now exceeds 100,000 genomes. Our results show an increase in the number of African countries that are able to sequence domestically and highlight that local sequencing enables faster turnaround times and more-regular routine surveillance. Despite limitations of low testing proportions, findings from this genomic surveillance study underscore the heterogeneous nature of the pandemic and illuminate the distinct dispersal dynamics of variants of concern-particularly Alpha, Beta, Delta, and Omicron-on the continent. Sustained investment for diagnostics and genomic surveillance in Africa is needed as the virus continues to evolve while the continent faces many emerging and reemerging infectious disease threats. These investments are

crucial for pandemic preparedness and response and will serve the health of the continent well into the 21st century.

DOI: 10.1126/science.abq5358
PMCID: PMC9529057
PMID: 36108049 [Indexed for MEDLINE]

291. EClinicalMedicine. 2022 Aug 11;52:101611. doi: 10.1016/j.eclinm.2022.101611. eCollection 2022 Oct.

PrEP uptake and HIV viral suppression when PrEP is integrated into Ugandan ART clinics for HIV-negative members of HIV-serodifferent couples: A stepped wedge cluster randomized trial.

Heffron R(1)(2), Muwonge TR(3), Thomas KK(1), Nambi F(3), Nakabugo L(3), Kibuuka J(3), Thomas D(1), Feutz E(1), Meisner A(1)(4), Ware NC(5)(6), Wyatt MA(5)(7), Simoni JM(1), Katz IT(6)(6), Kadama H(8), Baeten JM(1)(9), Mujugira A(3); Partners PrEP Program Team.

Collaborators: Heffron R, Baeten JM, Simoni J, Donnell D, Barnabas R, Thomas KK, Thomas D, Feutz E, Grabow C, Meisner A, Ciccarelli K, Scoville C, Ortblad K, Mujugira A, Muwonge TR, Kibuuka J, Nakabugo L, Nambi F, Nakitende M, Izizinga D, Kasita V, Kamusiime B, Nalumansi A, Twesige C, Kakoola G, Brown C, Namanda S, Kadama H, Ware NC, Wyatt MA, Pisarski E, Katz IT.

Author information:
(1)University of Washington, 325 Ninth Avenue, Box 359927 Seattle, Washington, USA.
(2)University of Alabama Birmingham, Birmingham, Alabama, USA.
(3)Infectious Diseases Institute, Makerere University, Kampala, Uganda.
(4)Fred Hutch, Vaccine and Infectious Disease Division, 1100 Fairview Ave N, Seattle, Washington, USA.
(5)Harvard Medical School, 25 Shattuck St., Boston, Massachusetts, USA.
(6)Brigham and Women's Hospital, Harvard Medical School, 75 Francis St, Boston, Massachusetts, USA.
(7)Harvard Global, Cambridge, Massachusetts, USA.
(8)Ministry of Health, Box 7272, Kampala, Uganda.
(9)Gilead Sciences LLC, Foster City, California, USA.

BACKGROUND: Global scale-up of HIV pre-exposure prophylaxis (PrEP) includes services to HIV-negative people in partnerships with people living with HIV (serodifferent couples). Data are needed on HIV outcomes, including uptake and adherence to PrEP and antiretroviral treatment (ART), to describe the impact of integrating PrEP into an existing HIV program.
METHODS: Using a stepped-wedge cluster randomized trial design, we launched PrEP delivery for HIV-negative members of serodifferent couples in Uganda by

integrating PrEP into existing ART programs for people living with HIV. The program provided PrEP training for ART providers, ongoing technical assistance, and a provisional supply chain mechanism for PrEP medication. Primary data on PrEP initiation, PrEP refills, ART initiation, and HIV viremia at 6 months (measured at 42-270 days) were collected through data abstraction of medical records from HIV-serodifferent couples sequentially enrolling at the ART clinics. Modified Poisson regression models, controlling for time and cluster, compared viral suppression (<1000 copies/ml) before and after launch of the PrEP program. This trial was registered at ClinicalTrials.gov, NCT03586128.
FINDINGS: From June 1, 2018-December 15, 2020, 1,381 HIV-serodifferent couples were enrolled across 12 ART clinics in Kampala and Wakiso, Uganda, including 730 enrolled before and 651 after the launch of PrEP delivery. During the baseline period, 99.4% of partners living with HIV initiated ART and 85.0% were virally suppressed at 6 months. Among HIV-negative partners enrolled after PrEP launched, 81.0% (527/651) initiated PrEP within 90 days of enrolling; among these 527, 11.2% sought a refill 6 months later. In our powered intent-to-treat analysis, 82.1% and 76.7% of partners living with HIV were virally suppressed, respectively, which was not a statistically significant difference (RR=0.94, 95% CI: 0.82-1.07) and was stable across sensitivity analyses.
INTERPRETATION: Integration of PrEP into ART clinics reached a high proportion of people in HIV-serodifferent relationships and did not improve the already high frequency of HIV viral suppression among partners living with HIV.
FUNDING: National Institute of Mental Health (R01MH110296).

© 2022 The Authors.

DOI: 10.1016/j.eclinm.2022.101611
PMCID: PMC9386395
PMID: 35990584

Conflict of interest statement: JMB reports being an employee of Gilead Sciences, with salary and stock/options, outside the submitted work. All other authors declare no competing interests.

292. Clin Infect Dis. 2022 Sep 30;75(7):1255-1264. doi: 10.1093/cid/ciac206.

Clinical Risk Factors Associated With Late-Onset Invasive Group B Streptococcal Disease: Systematic Review and Meta-Analyses.

Karampatsas K(1), Davies H(1), Mynarek M(2), Andrews N(3), Heath PT(1), Le Doare K(1)(4)(5).

Author information:
(1)Paediatric Infectious Diseases Research Group, Institute of Infection and Immunity, St. George's, University of London, London, United Kingdom.
(2)Center for Early Brain Development, Department of Clinical and Molecular

Medicine, Norwegian University of Science and Technology (NTNU), Trondheim, Norway.
(3)UK Health Security Agency, London, United Kingdom.
(4)MRC/UVRI @LHSTM Uganda Research Unit, Entebbe, Uganda.
(5)Pathogen Immunity Group, Public Health England, Porton Down, United Kingdom.

BACKGROUND: Group B streptococcal (GBS) infection remains one of the most significant causes of late-onset sepsis and meningitis (LOGBS) among young infants. However, transmission routes and risk factors for LOGBS are not yet fully understood.
METHODS: We conducted systematic reviews on clinical risk factors previously reported in the literature (prematurity, low birth weight [<2500 g], antenatal colonization, multiple-gestation pregnancy, maternal age <20 years, male infant sex, intrapartum fever, prolonged rupture of membranes) and meta-analyses to determine pooled estimates of risk.
RESULTS: We included 27 articles, reporting 5315 cases. Prematurity (odds ratio [OR] 5.66; 95% confidence interval [CI]: 4.43-7.22), low birth weight (OR 6.73; 95% CI: 4.68-9.67), maternal colonization (2.67; [2.07-3.45]), and multiple-gestation pregnancies (OR 8.01; 95% CI: 5.19-12.38) were associated with an increased risk of LOGBS.
CONCLUSIONS: Prematurity/low birth weight and maternal colonization are major risk factors for LOGBS. Future GBS vaccine studies should try to establish the optimal time for vaccination during pregnancy to protect preterm infants.

© The Author(s) 2022. Published by Oxford University Press for the Infectious Diseases Society of America.

DOI: 10.1093/cid/ciac206
PMCID: PMC9525091
PMID: 35275986 [Indexed for MEDLINE]

Conflict of interest statement: Potential conflicts of interest. K. L. D. is supported by Future Leaders Fellowships by UK Research and Innovation (UKRI) Future Leaders Fellowship (MR/S016570/1). P. T. H. reports research grants to the institution from Pfizer and Minervax outside of the submitted work. All other authors report no potential conflicts. All authors have submitted the ICMJE Form for Disclosure of Potential Conflicts of Interest. Conflicts that the editors consider relevant to the content of the manuscript have been disclosed.

293. Nutr Metab Insights. 2022 Sep 27;15:11786388221125107. doi: 10.1177/11786388221125107. eCollection 2022.

Predictors of Stunting and Underweight Among Children Aged 6 to 59 months in Bussi Islands, Wakiso District, Uganda: A Cross-Sectional Study.

Nsubuga EJ(1), Arinda Kato I(2), Lee S(3), Ssenyondo M(4), Isunju JB(5).

Author information:
(1)Ministry of Health, Kampala, Uganda.
(2)Public Health Nutrition Consultant, Kampala, Uganda.
(3)Department of Neuroscience, University of Pennsylvania, Philadelphia, PA, USA.
(4)United Nations Children's Fund (UNICEF), Kampala, Uganda.
(5)Department of Disease Control and Environmental Health, Makerere University School of Public Health, Kampala, Uganda.

BACKGROUND: Child undernutrition is a major public health concern in Uganda that can lead to increased risks of death with its prevalence higher in rural hard-to-reach areas than in urban areas. While it is assumed that the prevalence will be more concerning in islands with restricted accessibility to healthcare resources, limited research has been conducted on the prevalence and associated factors of stunting and underweight among children in Bussi Islands of Uganda. This study aimed to assess the prevalence and predictors of stunting and underweight among children aged 6 to 59 months in Bussi Islands of Wakiso District in Uganda.
METHODS: A cross-sectional study was conducted in Bussi Islands of Wakiso District. Sociodemographic and anthropometric measurements were obtained for randomly sampled 409 caretaker-child pairs from 409 households. Data was collected using pre-tested structured electronic questionnaires validated by the Uganda Ministry of Health. Anthropometric indices were calculated using ENA-SMART version 2011 and data analysis was conducted using STATA version 14. Modified Poisson regression was used to generate Unadjusted and Adjusted Prevalence Ratios (APRs) with 95% confidence intervals.
RESULTS: Prevalence of stunting and underweight among children in Bussi Islands were 29.8% and 16.1%, respectively. Independent predictors of stunting included: suffering from diarrhea (APR: 1.8; 95% CI: 1.3, 2.5); household food insecurity (APR: 1.7; 95% CI: 1.2, 2.4); and child age of 12 to 23 months and 24 to 35 months (APR: 2.3; 95% CI: 1.3, 4.0 and APR: 2.0; 95% CI: 1.1, 3.6 respectively). Protective factors against stunting were not suffering from measles (APR: 0.62; 95% CI: 0.42, 0.92); receiving deworming tablets every 6 months (APR: 0.58; 95% CI: 0.42, 0.81); and daily household utilization of more than 80 L of water (APR: 0.48; 95% CI: 0.24, 0.95). Predictors of underweight were suffering from diarrhea (APR: 2.2; 95% CI: 1.4, 3.4) and having more than 9 household members (APR: 2.8; 95% CI: 1.1, 7.5).
CONCLUSIONS: Child stunting and underweight are prevalent public health problems in Bussi Islands of Wakiso District. Therefore, the study suggests that nutrition interventions in the Islands should focus on childhood vaccination, family planning, sufficient safe water coverage, household food security, and health education of child caretakers on optimal infant and young child feeding and development.

© The Author(s) 2022.

DOI: 10.1177/11786388221125107
PMCID: PMC9520166
PMID: 36187343

Conflict of interest statement: Declaration of Conflicting Interests: The author(s) declared no potential conflicts of interest with respect to the research, authorship, and/or publication of this article.

294. Adolesc Health Med Ther. 2022 Sep 25;13:77-91. doi: 10.2147/AHMT.S383872. eCollection 2022.

Human Papillomavirus Vaccination Uptake and Its Predictors Among Female Adolescents in Gulu Municipality, Northern Uganda.

Aruho C(1), Mugambe S(2), Baluku JB(3), Taremwa IM(2).

Author information:
[truncated: 515,874 more chars]
